# Synthesis, Cytotoxicity, and Mechanistic Evaluation of Thiazolium-Based Gold(I) Bis‑*N*‑Heterocyclic Carbene Complexes

**DOI:** 10.1021/acs.inorgchem.6c02992

**Published:** 2026-10-10

**Authors:** Melanie E. Hoffmann, Lisa C. Haas, Fernanda Marques, Sarah Sarwary, Charlotte Theile, Bakr Mustafa, João D. G. Correia, Aram Prokop, Fritz E. Kühn

**Affiliations:** † TUM School of Natural Science, Department of Chemistry and Catalysis Research Centre, Molecular Catalysis, Technical University of Munich, Lichtenbergstr. 4, 85748 Garching bei München, Germany; ‡ Department of Human Medicine, 236368 MSH Medical School Hamburg, am Kaiserkai 1, 20457 Hamburg, Germany; § Centro de Ciências e Tecnologias Nucleares and Departamento de Engenharia e Ciências Nucleares, Instituto Superior Técnico, CTN, Universidade de Lisboa, Estrada Nacional 10 (km 139,7), 2695-066 Bobadela LRS, Portugal; ∥ Department of Pediatric Hematology/Oncology, 62491 HELIOS Clinics Schwerin, Wismarsche Strasse 393-397, 19055 Schwerin, Germany; ⊥ 26583 Universität Potsdam, Karl-Liebknecht-Str. 24-25, 14476 Potsdam, Germany

## Abstract

Imidazole- and benzimidazole-based Au­(I) *N*-heterocyclic carbene (NHC) complexes show great potency as anticancer agents. However, other NHC scaffolds are only sparsely explored. Four gold­(I) NHC complexes featuring thiazolium-based ligands (NHC_thia_) with varying *N*-substituents were synthesized and evaluated for their biological activity. The complexes containing aromatic *N*-substituents demonstrate higher stability, higher TrxR inhibition, and activity against breast (MDA-MB-231 and MCF-7) and ovarian (A2780) cancer cell lines, compared to the tested alkyl *N*-substituted complexes. Specifically, mesityl-substituted complex **1** exhibits high activity with IC_50_ values in the nanomolar range, exceeding the potency of auranofin in MDA-MB-231 cells. Mechanistic studies in leukemia cells (Nalm-6) revealed that complex **1** induces ROS formation, activates BAX and BAK, and causes mitochondrial membrane potential loss, initiating intrinsic apoptosis. Notably, the ligand precursor **L1** shows no significant cytotoxicity, confirming that the gold­(I) moiety drives the activity. Additionally, complex **1** can overcome resistance to etoposide and prednisolone without affecting healthy leukocytes, making Au­(I) bis-NHC_thia_ complexes a compelling class of antiproliferative agents.

## Graphical abstract

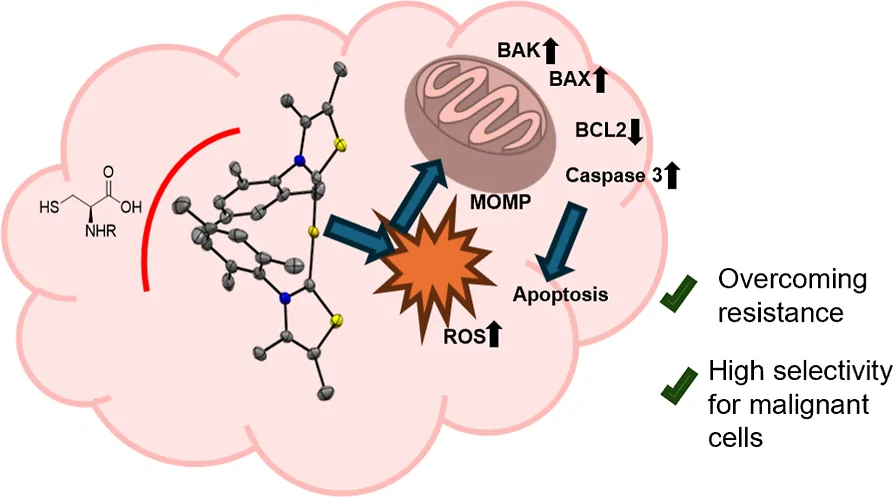

## 1 Introduction

The rise in incidence and mortality rates of cancer in the developed world is making cancer a preeminent global health challenge.^1^ Projections from the European Commission’s Joint Research Center suggest that in the absence of preventive measures, better diagnostic tools, and therapeutic interventions, cancer will be the leading cause of death in the EU by 2035.^2^ While advances in targeted therapies and potent chemotherapeutic agents have led to immense progress in cancer therapy,^3,4^ the long-term clinical utility of chemotherapeutics is frequently compromised by the emergence of drug resistance and, in some cases, even multidrug resistance.^5^ The persistence of these recalcitrant cells underscores the need for next-generation anticancer agents that can bypass or inhibit these resistance mechanisms.

Highly cytotoxic gold complexes have been investigated intensively as potential anticancer agents after the discovery of the antirheumatic auranofin’s anticancer activity.^6^ Replacing the phosphine ligand of auranofin with *N*-heterocyclic carbenes (NHCs) improves the stability of the gold complexes under physiological conditions.^7,8^ Owing to NHCs’ sigma-donating properties and their highly tunable structure, gold NHC complexes have been established in the field of medicinal inorganic chemistry.^8–12^ Au­(I) NHC complexes have shown high potency across a range of solid and hematologic cancer cell lines, in particular toward breast cancer (MCF-7) and leukemia cells.^13–16^ Additionally, Au­(I) NHC complexes can overcome drug resistance to common cytostatics, such as daunorobicin and cisplatin,^12–14,17,18^ as well as auranofin.^19^ Imidazole- and benzimidazole-based NHC have been the primary focus,^8,9^ however, there have been multiple notable accounts of alternative scaffolds, such as triazole and tetrazole.^14,15,20^

The group of Metzler-Nolte was the first to investigate gold NHC complexes with thiazole-based NHC scaffolds (NHC_thia_) toward their anticancer properties.^21^ The mono-NHC_Thia_ complex [Au­(NHC_Thia_)­I], containing a thiazolylalinine derivative, shows high cytotoxic activity in lung carcinoma (A549) cells. The complex reached IC_50_ values in the nanomolar range (400 ± 10 nM).^21^ These results piqued our interest, as only a few mono-NHC Au­(I) complexes have reported IC_50_ values in the nanomolar range.^22^ In addition to Metzler-Nolte’s results,^21^ NHC_thia_ is a compelling class of NHC ligands for anticancer therapy. Thiazole derivatives play a vital role in the human body as vitamin B1.^23^ NHC_thia_ are weak sigma-donating NHCs compared to imidazolium, benzimidazolium, and 1,2,3-triazolium-based NHCs.^24^ In contrast to the imidazolium and benzimidazolium scaffolds, the thiazolium scaffold has only one nitrogen atom and therefore only one “wingtip” residue at the nitrogen position.

Besides these interesting properties, Au­(I) bis-NHC_Thia_ complexes (containing two NHC_thia_ ligands) have, to the best of our knowledge, only been studied once before. Gupta et al. tested a Au­(I) bis-NHC_thia_ complex, against MCF-7 cells, showing only moderate to low activity.^25^ In general, Au­(I) bis-NHC complexes have shown higher activity against cancer cells than their corresponding Au­(I) mono-NHC halide complexes,^8,22^ making Au­(I) bis-NHC_thia_ complexes compelling to investigate further. Accordingly, we synthesized different Au­(I) bis-NHC_thia_ complexes. The complexes were modified at the N-substituent (wingtip), and their general biological activity was assessed to identify a stable, active compound, to be further investigated.

## 2 Results and Discussion

Four complexes with different wingtips, namely mesityl, isopropyl, hexyl, and phenyl have been prepared: bis­(3-mesityl-4,5-dimethyl-3,4-thiazol-2-yl)­gold­(I) hexafluorophosphate (**1**), bis­(3-isopropyl-4,5-dimethyl-3,4-thiazol-2-yl)­gold­(I) hexafluorophosphate (**2**), bis­(3-hexyl-4,5-dimethyl-3,4-thiazol-2-yl)­gold­(I) (**3**) and bis­(3-phenyl-4,5-dimethyl-3,4-thiazol-2-yl)­gold­(I) hexafluorophosphate (**4**) (see Chart 1).

**Chart 1:**
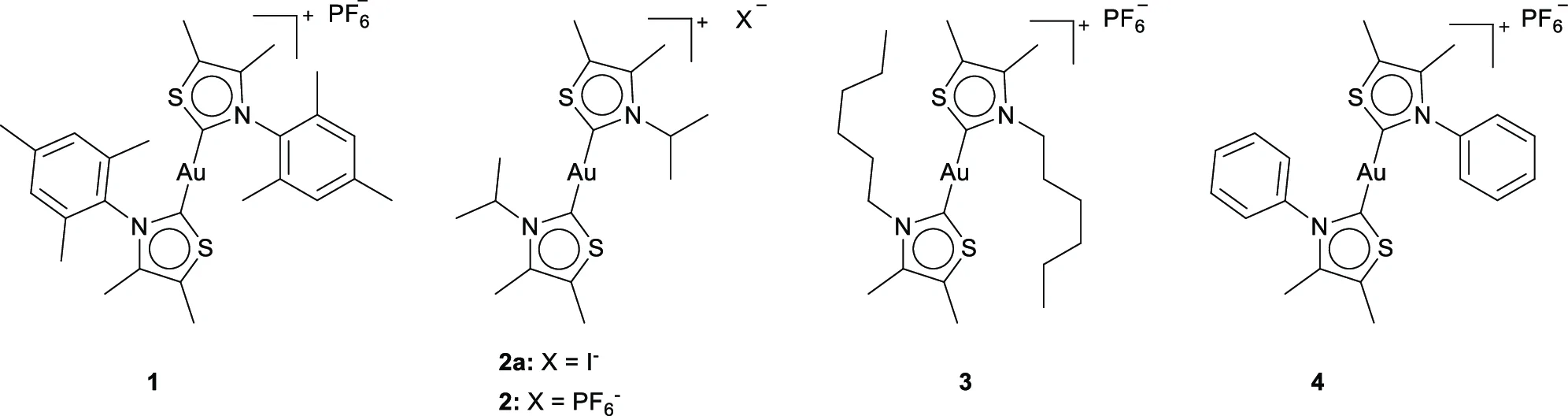
Au­(I) Bis-NHC Complexes **1**–**4** Evaluated in this Study

This selection was inspired by the following considerations: Au­(I) NHC complexes containing aromatic moieties, such as mesityl and 2,6-diisopropylphenyl groups have shown great activity.^8,15,26^ Additionally, lipophilic residues on the NHCs have also been shown to increase activity against cancer cells,^27,28^ while steric wingtip substituents can enhance stability.^29^

### 2.1 Synthesis

All ligand precursors have been previously reported.^30,31^ 3-Phenyl-4,5-dimethylthiazol-3-ium hexafluorophosphate **L4** and 3-mesityl-4,5-dimethylthiazol-3-ium hexafluorophosphate **L1** have been synthesized according to the literature procedure. Due to the nature of the synthesis, the hexafluorophosphate salt was obtained for both ligands (**L1** and **L4**). To enable comparability between the complexes, all following syntheses were optimized to yield the hexafluorophosphate complexes.

Ligand precursor salts 3-hexyl-4,5-dimethylthiazol-3-ium hexafluorophosphate **L3** and 3-isopropyl-4,5-dimethylthiazol-3-ium iodide **L2a** have been synthesized as per literature,^31^ however, the purification method has been modified.

**L2a** and **L3** were synthesized by refluxing 4,5-dimehtly thiazole in acetonitrile in the presence of isopropyl iodide and hexyl bromide, respectively. After solvent evaporation, **L2a** was obtained as a brown oil, which was extracted with water. The water was removed *in vacuo*, and the resulting yellow solid was washed with cold acetone to obtain colorless crystals in 68% yield. **L3** was purified by the addition of water and ammonium hexafluorophosphate. The reaction was stirred overnight and afterward extracted three times with dichloromethane (DCM). The organic phases were combined, washed with water and brine, dried with MgSO_4_, and the solvent removed under vacuum. The resulting colorless crystals were obtained in 72% yield.

All ligands are analyzed via elemental analysis and ^1^H-, ^13^C-nuclear magnetic resonance spectroscopy (NMR). **L1**, **L3**, and **L4** are present as hexafluorophosphate salts, while **L2a** was used as an iodide salt.

For complexation, [Au­(tht)­Cl] (tht = tetrahydrothiophene) was synthesized according to a literature procedure^32^ and mixed with the thiazolium salts in the presence of potassium carbonate in acetone (see Scheme 1). The formation of potassium chloride, which is insoluble in acetone, is suspected to be the key driving force in this reaction. Exchanging potassium carbonate with other bases (HMDS, Ag_2_O, KH) did not lead to the desired complexes. While Ag_2_O could form the corresponding silver complex, no transmetalation with gold occurred. This is in alignment with the work of Metzler-Nolte’s group.^21^ HMDS and KH, as strong bases, lead to undesirable side reactions.

**Scheme 1:**
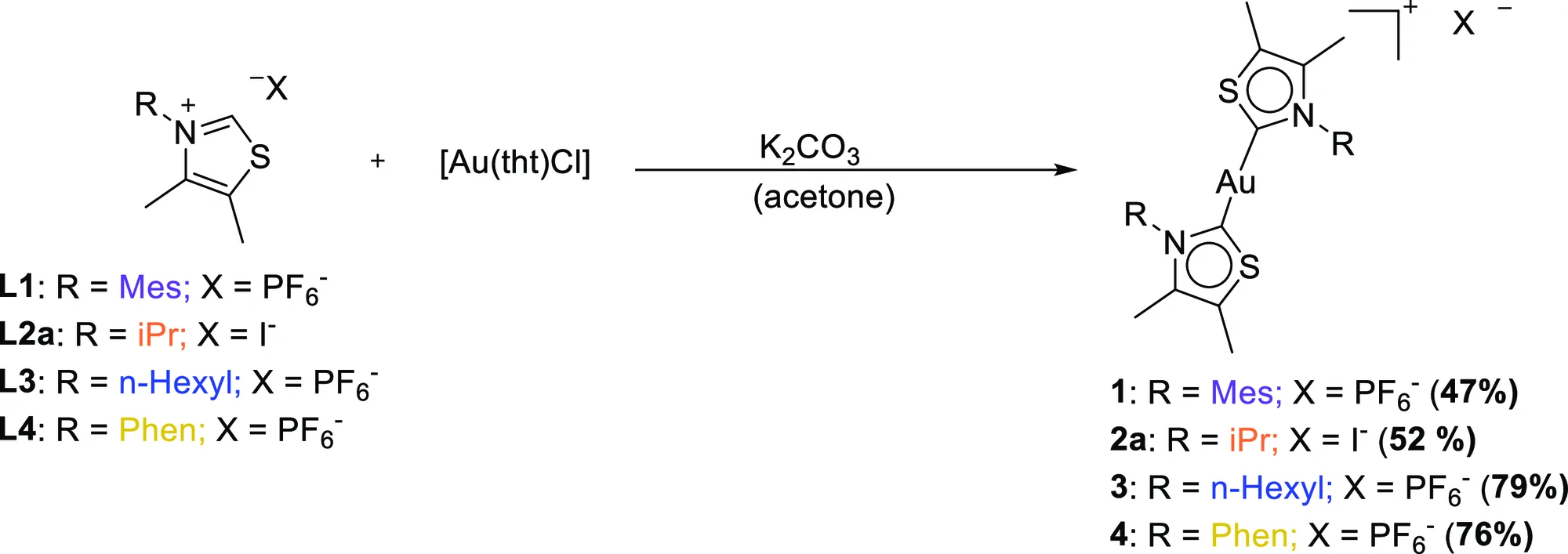
General Synthesis of Complexes **1**, **2a**, **3**, and **4**. For the synthesis of complexes **1**, **3** and **4**, **L1**, **L3** and **L4** were stirred in the presence of [Au­(tht)­Cl] with an excess of base for 16 h at room temperature. For the synthesis of complex **2a**, the reaction mixture was heated to 65 °C, and to achieve full conversion, the reaction time was extended to 3 days. Yields are given in brackets

The reactions were monitored via electrospray ionization mass spectrometry (ESI-MS). The Au­(I) mono-NHC_thia_ halogen complex was observed only sparingly, and it could not be isolated under the reaction conditions. Changing the equivalents to 1:1 thiazolium salt/[AuCl­(tht)], did not change the outcome, still no Au­(I) mono-NHC_thia_ halogen complex could be isolated. A side product can be detected by ESI-MS: a mass peak corresponding to a thiazole-dimer coordinated to an Au­(I) mono-NHC_thia_ species is visible during the formation of all four complexes. Thiazole-based carbenes possess only one wingtip ligand and, therefore, lack the necessary steric bulk to shield the free carbene site. Consequently, due to the Wanzlick equilibrium,^33^ the thiazole dimer occurs. Increasing the reaction temperature can reduce the formation of the thiazole-dimer, however, it simultaneously accelerates the formation of gold nanoparticles, as indicated by a violet coloration. Therefore, the reaction time and temperature were optimized to minimize gold nanoparticle accumulation while avoiding excessive thiazole-dimer formation.

Due to the weak base route, all four complexes **1**–**4** can be obtained. While complexes **1**, **3**, and **4** form at room temperature in comparatively short time (2–16 h), complex **2a** (**[2]­I**) needed to be stirred for 3 d at 65 °C for the reaction to reach completion. This is assumed to be due to the influence of the counterion, iodide.

The purification to remove the thiazole-dimer species depends on the substituent. The alkyl-substituted complexes **2a** and **3** can be purified by slow recrystallization. Therefore, the compounds were dissolved in dichloromethane (DCM) and layered with diethyl ether (1:3). Slow diffusion of diethyl ether into the DCM phase yielded pure colorless crystals overnight. Complexes **1** and **4** can be obtained in pure form through column chromatography (2.5 cm x 10 cm, silica gel, 10% methanol in DCM). Yields between 47 and 79% can be achieved. After the successful synthesis of **2a**, **2a** reacted with ammonium hexafluorophosphate to generate **2** in 89% yield. Complexes **1**–**4** are >95% pure, indicated by ^1^H-, ^13^C NMR spectroscopy, ESI-MS, high resolution (HR) ESI-MS, and elemental analysis. Characterization by HPLC was not possible, as the gold complexes appear to affect the column of the reverse-phase HPLC.

### 2.2 X-ray Structural Characterization

Crystals of complexes **1** and **2** were grown by slow diffusion of diethyl ether into saturated DCM solutions of **1** and **2**. The crystals were analyzed by single-crystal X-ray diffraction (SC-XRD) (see Figure 1). Both complexes deviate slightly from the expected linear geometry for an Au­(I) complex. The facing orientation of the two isopropyl wingtips of **2** pushes the complex to an angle of 174.08(8)°. Complex **1** shows a more linear geometry (176.90(14)°). The steric bulk of the mesityl wingtips hinders coplanarity of the thiazole moiety, resulting in twisted orientations. This difference in angles results in a longer bond length between the carbene moiety and the gold atom (2.0202(19) Å) in **2** than in **1** (2.015(3) Å), which is slightly longer than the bond lengths of Au­(I) bis-NHC complexes containing 1,4-dimesityl-3-methyl-triazolylidene (∼2.011 Å) and 1,3-dimesityl-imidazolylidene (∼2.008 Å).^15,34^

**Figure 1:**
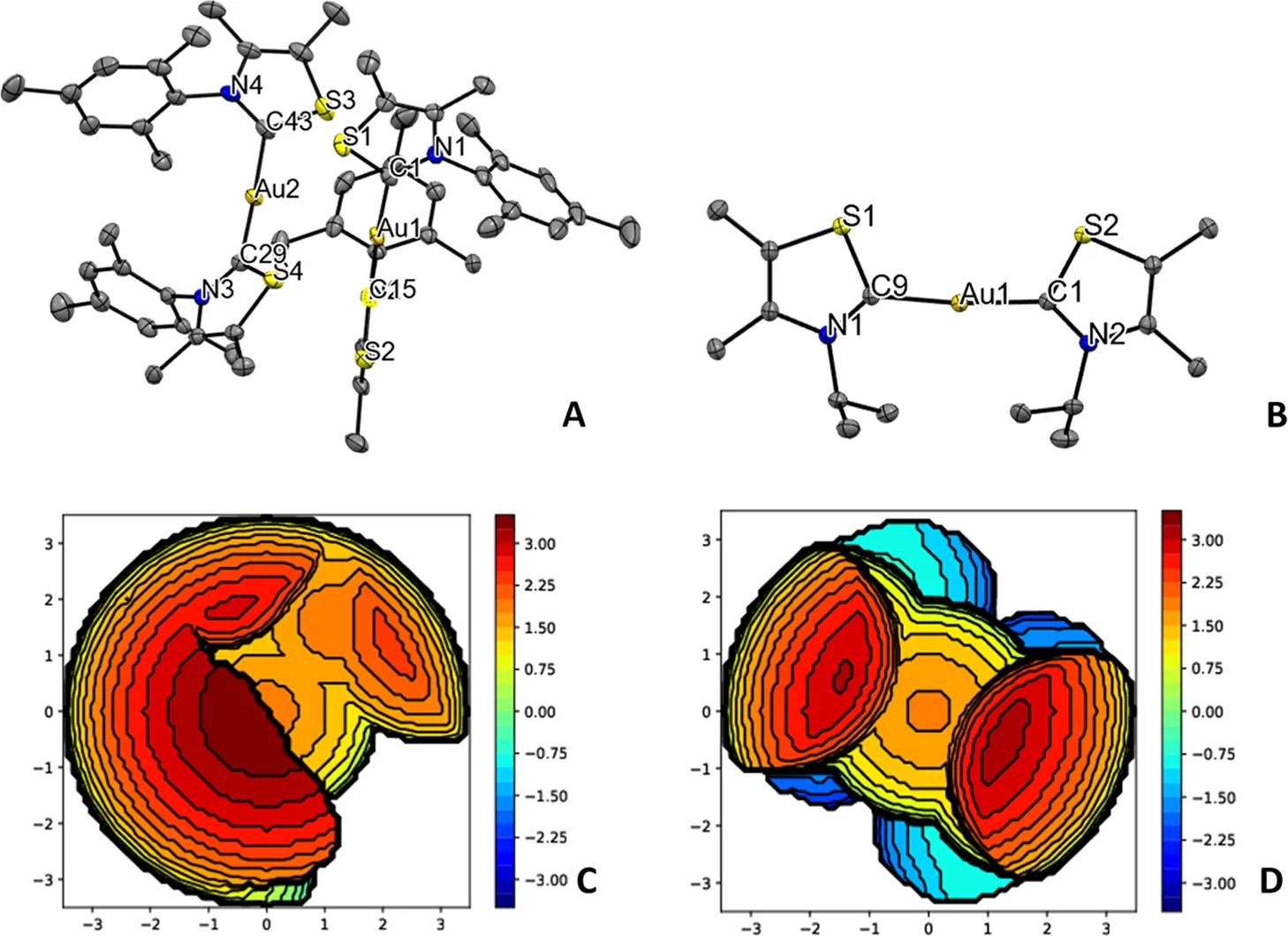
(A,B) Show the crystal structure of complexes **1** and **2** in ORTEP depiction. Ellipsoids are shown at a 50% probability level. Disorders are omitted for clarity. Gray = carbon, white = hydrogen, blue = nitrogen, violet = phosphorus, green = fluorine, yellow = sulfur, gold = gold. (C,D) Show the buried volume calculations based on the crystal structure data of **1** and **2**, respectively. The color scale represents the occupied space along the visual axis. The buried volume calculated around 3.5 Å around the gold center of complex **1** and **2** is 66.9% and 65.1% respectively.

Interestingly, one molecule of complex **1** orientates toward another molecule of **1**: the two gold centers face each other at a distance of 3.3232(2) Å, which can be considered as a minor aurophilic interaction.^35^ The formation of an aurophilic interaction is possible due to the nature of the thiazole scaffold. Because it possesses only a single wingtip ligand, it is able to combine sufficient high steric bulk with a nevertheless accessible axial face. While such structural features may potentially be exploited to design weak interactions for other applications, such as photophysics,^35^ investigations in these directions are outside of the scope of this study.

### 2.3 Stability and Activity Studies

Gold complexes are known for their affinity to bind seleno and thiol groups. This property is assumed to be the key to gold’s cytotoxic activity against cancer cells, as gold binds to vital cellular enzymes, such as thioredoxin reductase (TrxR),^8,15^ glutathione reductase,^36^ and glutathione *S*-transferase.^37^ However, the seleno- and thiol-affinity makes gold complexes prone to react with other small molecules, such as the antioxidant l-glutathione or l-cysteine, which can lead to deactivation.^21^ This portrays the fiddle play between activity and inactivity for gold complexes. The stability and activity of the four complexes were evaluated against water, l-glutathione, *N*-acetylcysteine and TrxR.

As regards to stability, all four complexes are stable in a water/DMSO solution for 72 h, as monitored by ^1^H NMR spectroscopy (see Supporting Information Figures S30–S33). The stability of the complexes in the presence of l-glutathione was also assessed by UV–vis spectroscopy.

Therefore, a saturated, degassed 1 M phosphate-buffered saline (PBS) of each complex was mixed in the presence of l-glutathione. All complexes show a broad band between 250 and 350 nm. No reaction in PBS solution without l-glutathione was observed. After the addition of l-glutathione, the signals of all complexes change over the course of 48 h, indicating a reaction with l-glutathione (see Supporting Information Figure S29). While complexes **2** and **3** transition over time to a new set of distinct signals, complexes **1** and **4** display a two-step spectral evolution: an initial appearance of new signals followed by a second time-dependent transition. However, this interesting formation could not be analyzed further due to the poor solubility of all complexes in water and the poor solubility of l-glutathione in anything other than water; a reaction mixture suitable for ^1^H NMR spectroscopy cannot be obtained to examine the reactivity quantitatively.

Regardless, to better understand Au­(I) bis-NHC_thia_ complexes’ interactions with thiols, *N*-acetyl cysteine (NAC), a DMSO-soluble cysteine analog, was mixed with complexes **1**–**4**, and the reaction was monitored via ^1^H NMR for 72 h (see Figure 2 and Supporting Information Figures S34–S37). For further support of the ^1^H NMR results, ESI-MS spectra were taken after 48 h (see Supporting Information Figures S38–S41).

**Figure 2:**
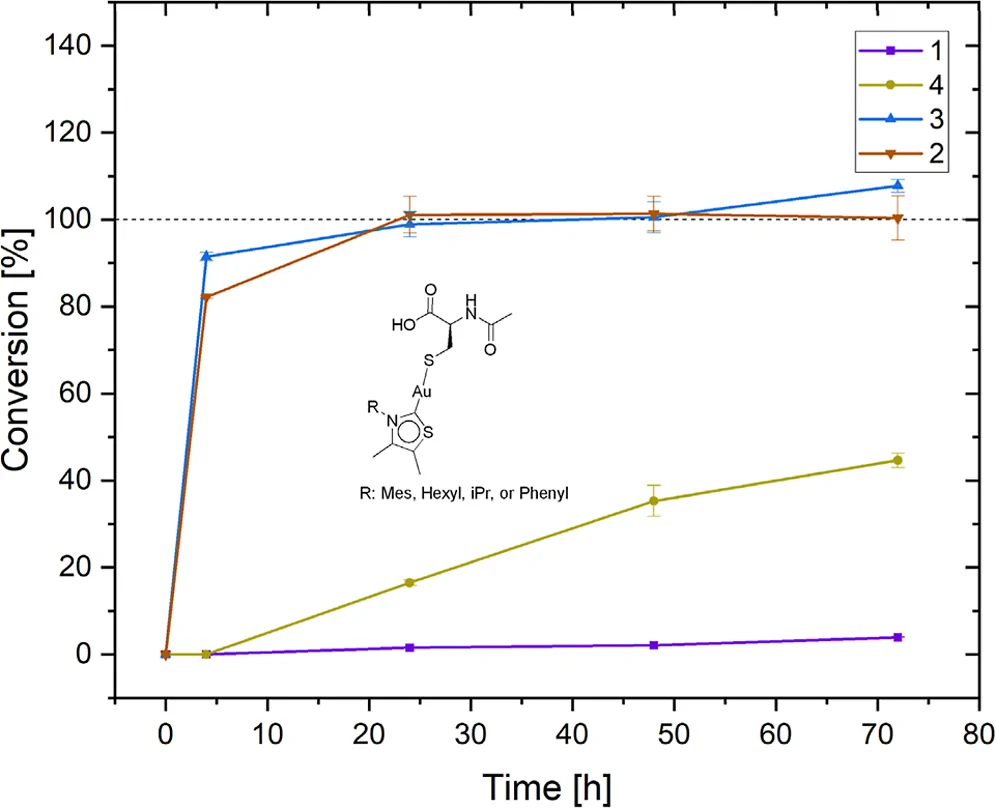
Conversion of complexes **1**, **2**, **3**, and **4** with *N*-acetylcysteine (NAC). One equiv of complex was mixed with 3 equiv of NAC in DMSO-*d*_6_. The release of NHC ligands as free thiazolium salt was monitored via ^1^H NMR. The formation of [Au­(I)­(NAC)­(NHC_thia_)] complex is demonstrated in percentage. A conversion over 100% is an indication of the loss of both NHC ligands.

Complexes **2** and **3**, containing alkyl wingtips, react rapidly. This is indicated by the appearance of free thiazolium salts’ signals in the ^1^H NMR. The acidic proton signal at ca. 10 ppm appears as a singlet. In addition to NMR analysis, the ESI-MS spectra of **2** and **3** show peaks at 155.97 and 198.12 *m*/*z*, corresponding to respective masses of **L2** and **L3**, deducting hexafluorophosphate. In accordance with these signals, masses of 866.07 and 950.15 *m*/*z* can be observed for complexes **2** and **3**, respectively, which can correspond to the mass of a species [Au_2_(NHC)_2_NAC]^+^. These species probably arise from [Au­(I)­(NHC_thia_)­NAC], as the neutral species cannot be observed in an ESI-MS spectrum. Moreover, the ^1^H NMR spectrum around 8 ppm, where two NAC-associated doublets are observed, a new signal appears, indicating a reaction of NAC, likely with the gold complexes. With regard to the internal standard benzene, over 80% of **2** and **3** react with NAC over 4 h. After 48 h, **2** and **3** are converted fully to the [Au­(I)­(NAC)­(NHC_thia_)] complex.

Complexes **1** and **4**, containing aromatic wingtips, do not show the same affinity toward NAC. While complex **1** does not react with NAC over the course of 48 h, complex **4**, which, compared to complex **1**, contains a less sterically demanding phenyl group, does react with NAC. However, complex **4** does not show the same conversion rate as **2** or **3**. After 72 h, only 40% of **4** reacts with NAC to free thiazolium salt and the complex [Au­(I)­(NAC)­(L4)]. Monitoring via ESI-MS confirmed the formation of free thiazolium salt (190.07 *m*/*z*). Additionally, a peak (933.99 *m*/*z*) corresponding to [Au_2_(NAC)­(L4)_2_]^+^ is visible. Overall, the stability trend of the chosen compounds seems to depend more on the electronic than on the steric nature of the ligands. Steric-bulk comparisons of complexes **1** and **2** indicate similar free access to the gold center. Buried volume calculations for complex **2** and **1** are 65.1% vs 66.9%, respectively.

However, **L2** is a stronger electron-donor than **L1**, as determined by the carbene signal in the ^13^C NMR: 199 vs 204 ppm. This enhanced electron density around the gold center leads to a more active species, as evidenced by the rapid reaction of NAC with **2**, but not with **1**.

Overall, the stability of the four Au­(I) bis-NHC_thia_ complexes seem to depend significantly more on the one wingtip ligand of the NHC_thia_, in contrast to what is reported for Au­(I) bis-NHC complexes containing NHCs with two wingtips.^15^

Additionally, the complexes were investigated against TrxR, widely considered the most prominent biological target of gold compounds.^8^ TrxR is inhibited through binding of the gold center to the seleno-group at the active site of the enzyme.^10^ This causes the Trx system to collapse, and the subsequent accumulation of reactive oxygen species (ROS) leads to the initiation of apoptosis.^38^

The effect of complexes **1**–**4** on the activity of TrxR was evaluated by colorimetric DTNB assay as previously described with modifications by using MCF-7 and MDA-MB-231 cell lysates (see Figure 3).^39^ For comparison, the TrxR activity of untreated cells and of cells treated with auranofin is given. With MCF-7 cell lysates, all four complexes reduce TrxR activity in the same range as auranofin. Notably, complexes **1** and **4** seem to decrease the TrxR activity in MDA-MB-231 cell lysates to a greater extent than in MCF-7 cell lysates. With MDA-MB-231 cell lysates, complexes **2** and **3** show minimal decrease in TrxR. In contrast, complex **4** decreases TrxR activity even more than auranofin. Given that complexes **2** and **3** exhibit high activity toward small molecules, they are presumably deactivated before they can reach the TrxR active site.

**Figure 3:**
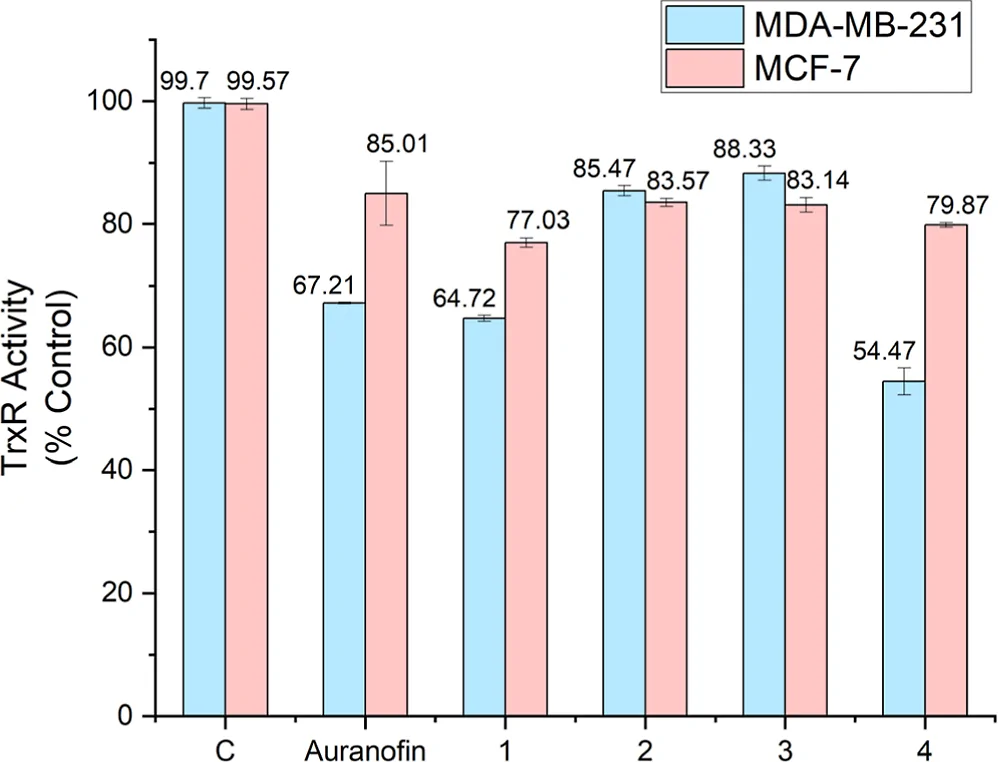
The effect of complexes **1**–**4** on the TrxR activity upon incubation with the complexes at 20 μM for 2 h. The most active TrxR inhibitors were **1** and **4** in the MDA-MB-231 cells. Positive control = auranofin; negative control = untreated cells. Results are mean ± SD of two independent experiments.

### 2.4 Cytotoxicity Against Solid Cancer Cells

The cytotoxicity of the cationic Au­(I) NHC complexes **1**–**4** is tested against solid cancer cell lines A2780 (ovarian), MCF-7, and MDA-MB-231 (breast). The cell growth inhibition (IC_50_) is assessed using the MTT assay (see Table 1). Previous studies with cisplatin and auranofin are included as ref 15. All complexes exhibit higher cytotoxicity than cisplatin in the chosen cell line. In MCF-7, auranofin is more potent than the investigated complexes. Complexes **1** and **4** show similar reactivity in A2780 cells, and even enhanced activity in MDA-MB-231 cells compared to auranofin. Complexes **2** and **3** perform similarly in MDA-MD-231 cells to auranofin, but do not show the same activity as auranofin in A2780 cells.

**Table 1:** IC_50_ Values ±SD (μM) of **1**, **2**, **3**, and **4** Together with the Reference Drugs Cisplatin and Auranofin in Human Cancer Cell Lines (Ovarian and Breast Cancer Cell Lines) After 48 h Incubation Determined by the MTT Assay^a^

	A2780	MDA-MB-231	MCF-7
cisplatin^15^	3.6 ± 1.3	13.8 ± 4.5	21.0 ± 6.3
auranofin^15^	0.43 ± 0.23	1.9 ± 0.7	0.28 ± 0.14
1	0.58 ± 0.11	0.83 ± 0.07	0.79 ± 0.10
2	1.68 ± 0.42	1.54 ± 0.25	2.43 ± 0.26
3	2.21 ± 0.76	1.90 ± 0.42	3.02 ± 0.73
4	0.59 ± 0.16	0.84 ± 0.14	1.04 ± 0.13

a Additionally, complexes **1** and **4** were tested against leukemia cells. *n* = 2.

Interestingly, complexes **2** and **3** show similar cytotoxicity, while complex **3** has a more lipophilic N-substituent than complex **2**. These findings are compelling, as previous reports of imidazolium-based Au­(I) bis-NHC complexes suggested that increased lipophilicity correlates with increased cytotoxicity.^28^ In general, complexes **1** and **4,** containing aromatic substituents, are alike in their activity and perform significantly better in the chosen cell lines than complexes **3** and **2**, containing alkyl residues. This trend of reactivity among the four tested complexes is directly correlated with the ligands’ stability against NAC and their ability to deactivate TrxR.

### 2.5 Cytotoxicity Against Nonsolid Cancer Cells

Additionally, to the evaluation of the complexes’ activity in solid cancer cell lines, we investigated the cytotoxicity of most potent complexes **1** and **4** against hematologic lymphoblastic leukemia cells (ALL, Nalm-6) (see Table 2). Our group has taken a special interest in investigating gold NHC complexes in this cell line as they have shown great activity in acute lymphoblastic leukemia cells,^13–15^ one of the most prevalent childhood cancers.^40^ Complexes **1** and **4** exhibit high cytotoxicity in Nalm-6 cells, with IC_50_ reaching 0.208 ± 0.016 and 0.133 ± 0.004 μM respectively. This excessed the IC_50_ values of the previously published Au­(I) bis-NHC complexes, based on 1,4-dimesityl-3-methyl-1,2,3-triazolium salts.^15^ Due to the high activity in this cell line, and complex **1**’s high stability against NAC, the complex was investigated further in Nalm-6 cells.

**Table 2:** IC_50_ Values ±SD (μM) of **1** and **4** in Human Leukemia Cancer Cell Lines After 48 h Incubation Determined by the MTT Assay^a^

	Nalm-6
1	0.208 ± 0.016
4	0.133 ± 0.004

a *n* = 2.

### 2.6 Mechanisms of Cell Death

Annexin V/propidium iodide (Pl) double staining was performed to determine if cells following treatment with complex **1** for 48 h undergo a regulated cell death or necrosis (see Figure 4). Cells were evaluated by flow cytometry.

**Figure 4:**
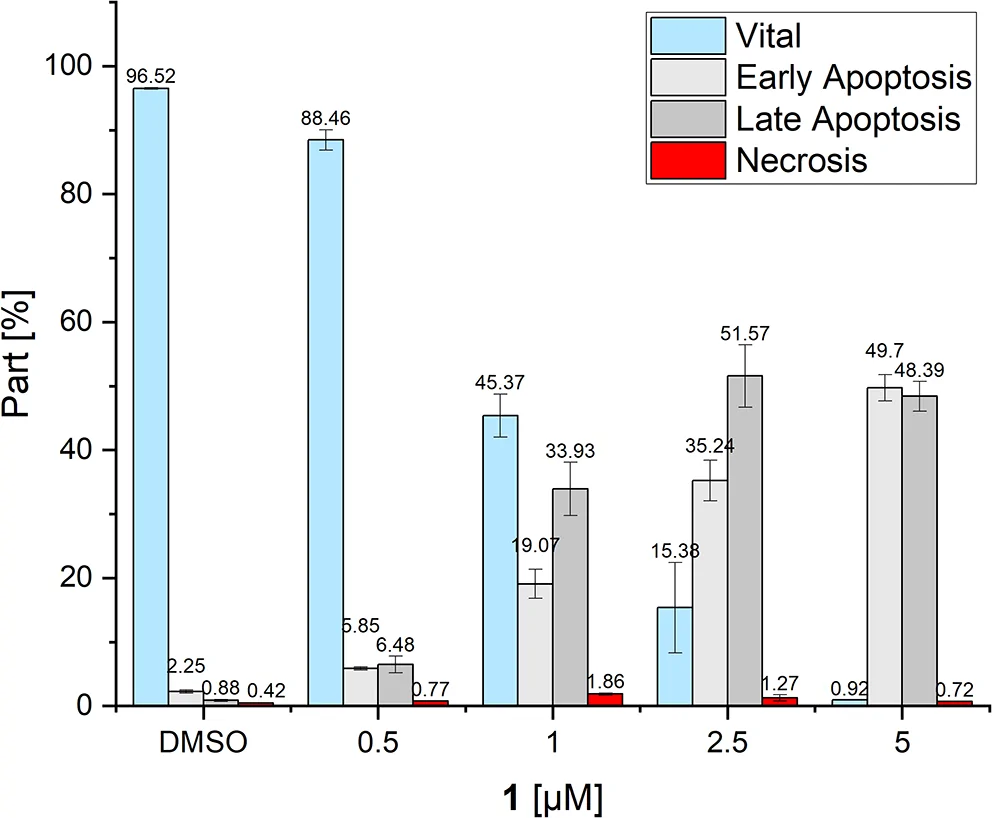
Induction of apoptosis by **1** in Nalm-6 leukemia cells. After 48 h of treatment with increasing concentrations of **1**, Annexin V/PI double staining was performed and measured by flow cytometry. It shows the difference between vital, early apoptotic, late apoptotic, and necrotic cells. Treatment with **1** resulted in a decrease in viable cells and a marked increase in apoptotic populations. Data are presented as mean ± SD of two biological replicates (*n* = 2), each with three technical replicates.

A remarkable shift in cell population, with 19.07% of cells in a stage of early apoptosis (Annexin V+/PI−) and 33.93% in late apoptosis (Annexin V+/PI+) was observed at 1 μM. Notably, the population of cells undergoing primary necrosis (Annexin V–/PI+) is negligible. The high percentage of late apoptotic cells relative to early apoptotic cells, alongside a large viable population, suggests slow initiation followed by a rapid transition through the execution phase of apoptosis.

To determine if complex **1** induces apoptosis via the mitochondrial pathway, like previously reported gold NHC complexes,^8,14^ the influence of complex **1** on the mitochondrial membrane potential and the accumulation of reactive oxygen species (ROS) was investigated. Loss of mitochondrial membrane potential can result from ROS accumulation, which can be due to TrxR inhibition.^38^ Nalm-6 cells were incubated with complex **1** for 48 h, followed by staining with 5,5′,6,6′-tetrachloro-1,1′,3,3′-tetra-ethylbenzimidazoyl-carbocyanide iodide (JC-1). As a positive control, carbonyl cyanide *m*-chlorophenyl hydrazone (CCCP) was used. The shift of JC-1 fluorescence from red to green, indicates a loss of the mitochondrial membrane potential. Cells were analyzed via flow cytometry (see Figure 5). With an increase in **1** concentration, an increase in mitochondrial membrane potential depolarization can be observed. Hence, it was investigated whether **1** induces ROS. Nalm-6 cells were pretreated with NAC for 1.5 h. NAC is a common antioxidant used as an ROS inhibitor.^41^ Afterward, one batch of Nalm-6 cells was incubated with complex **1**, another with DMSO, and a third with 100 μM hydrogen peroxide for 3.5 h (see Figure 6).

**Figure 5:**
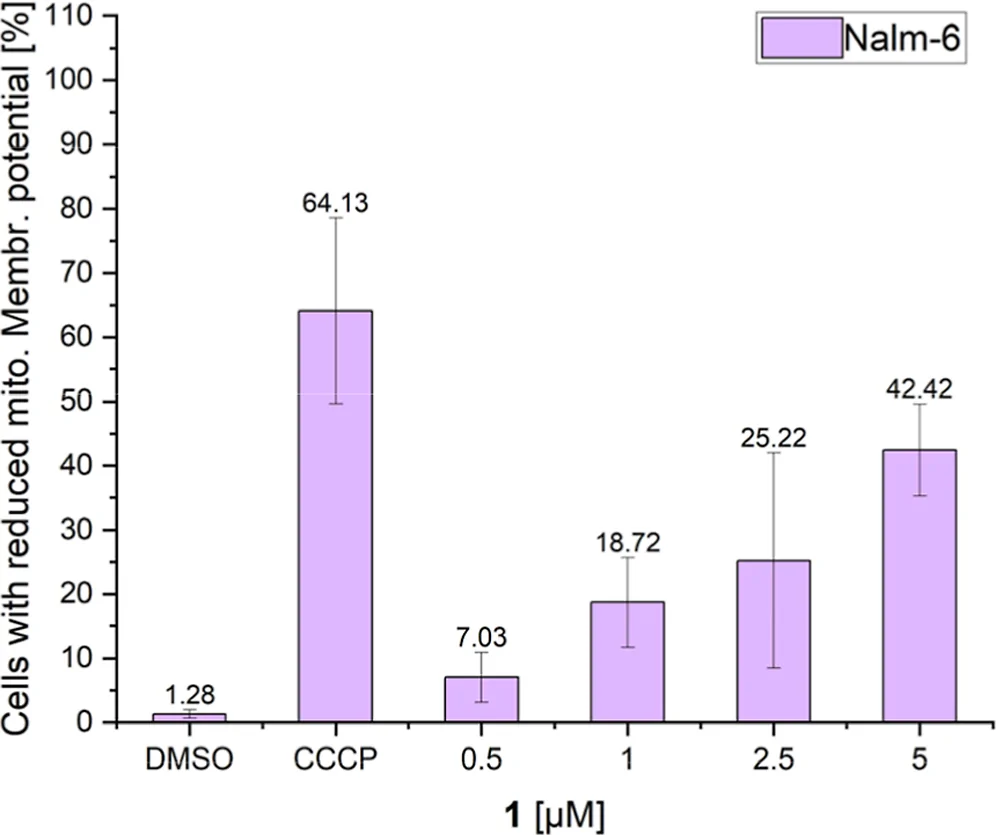
Mitochondrial membrane potential depolarization induced by **1** in Nalm-6 cells. JC1-staining revealed a mitochondrial membrane potential depolarization 48 h post-treatment with different concentrations of **1** on Nalm-6 leukemia cells. DMSO-treated cells served as a negative control, while CCCP-treated cells served as a positive control. Data are presented as mean ± SD of two biological replicates (*n* = 2), each with three technical replicates.

**Figure 6:**
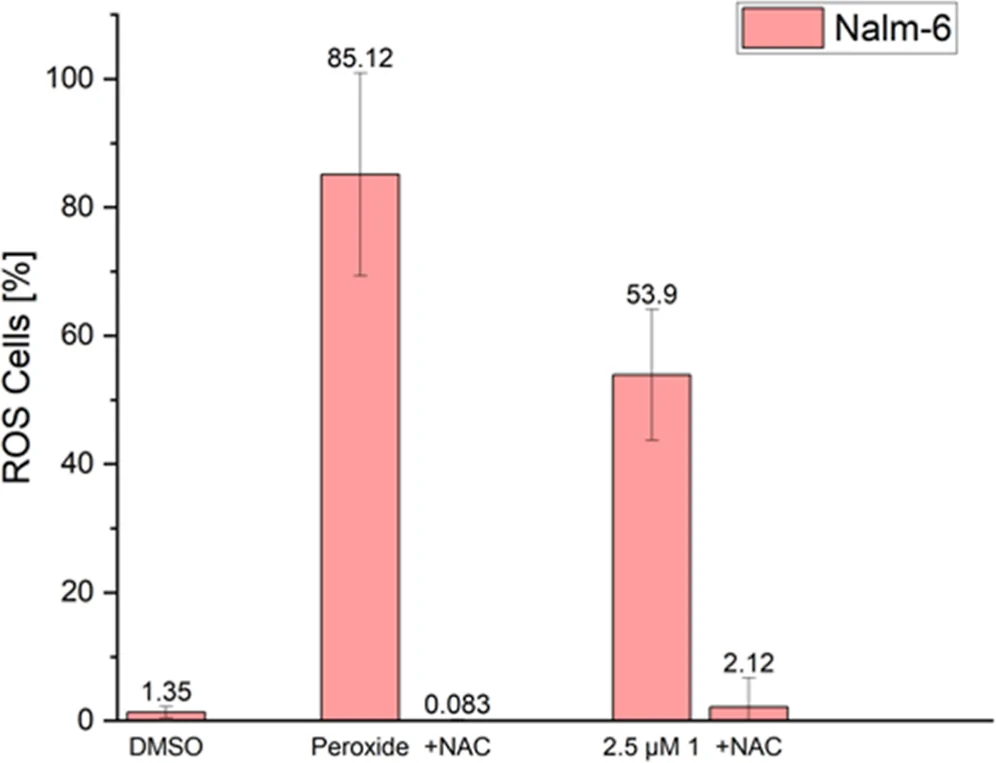
Release of ROS molecules was stained by CellRox dye and analyzed via flow cytometry after incubation with **1** and controls. 100 μM of H_2_O_2_ was used as a positive control for ROS induction. Values are relative to DMSO control. Treatment with **1** and hydrogen peroxide showed a increase in ROS levels. Co-treatment with NAC suppressed ROS levels, indicating ROS-dependent activity. Data are presented as mean ± SD of two biological replicates (*n* = 2), each with three technical replicates.

Additionally, cells were treated with fluorescent CellRox dye for 1 h. ROS levels were measured by flow cytometry. The same procedure was repeated in the absence of NAC for all three experiments (**1**, DMSO, H_2_O_2_). Cells that were only treated with complex **1** showed a high level of ROS formation, while cells pretreated with NAC exhibited less ROS accumulation. This suggests that **1** actively induces ROS formation in the cell.

### 2.7 Western Blot Analysis

To further investigate the effect of **1** on the protein expression level, Western blot analyses were performed to determine if treatment with **1** induces apoptosis via the intrinsic (mitochondrial) pathway.^42,43^ Mitochondrial damage leads to the release of cytochrome *c* and subsequent activation of the initiator caspases, promoting activation of the intrinsic apoptotic pathway.^44^ The pro-apoptotic members BAX and BAK of the BCL-2 protein family that promote mitochondrial outer membrane permeabilization,^42^ were chosen for Western blot analysis. Therefore, Nalm-6 (leukemia) (see Figure 7) and BJAB (lymphoma) (see Supporting Information Figure S44). Cells were treated with complex **1** at different time points (6, 12, and 18 h). Treatment of cells with DMSO served as a vehicle control. β-Actin was used as a loading control.

**Figure 7:**
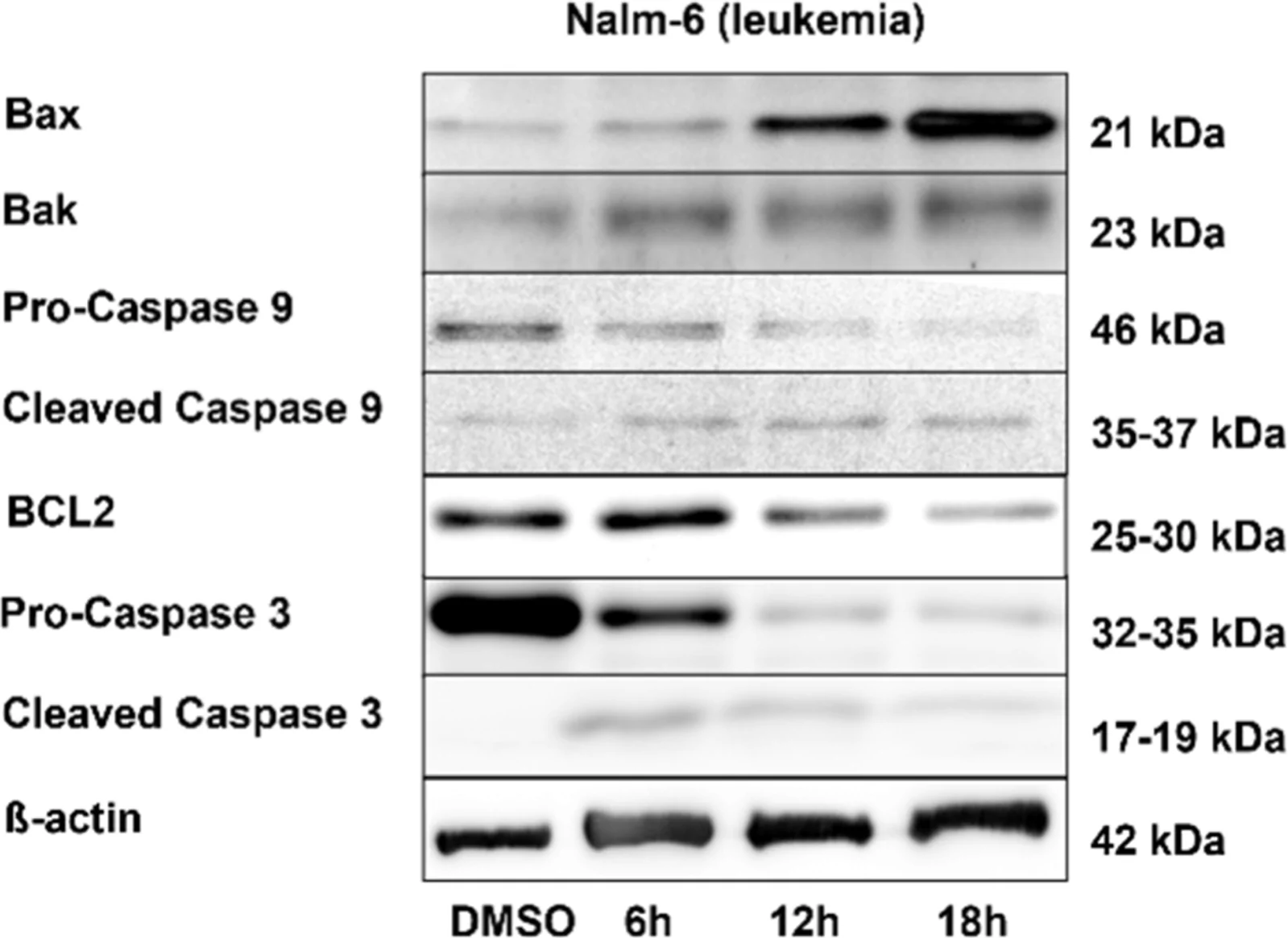
Western blot analysis: Nalm-6 (leukemia) cells were treated with the gold complex 1 at different time points (6 h, 12 h, and 18 h). Cells treated with DMSO served as vehicle control. The analysis was done using antibodies against apoptosis-related proteins. β-Actin was used as a loading control. The appearance of cleaved caspase fragments and increased levels of Bax and Bak indicate activation of the apoptotic signaling pathway in response to treatment with complex **1**.

Treatment with complex **1** resulted in a steady upregulation of BAX in both cell lines, while BAK levels also increased, although to a lesser extent. Consistent with activation of this pathway, a decrease in pro-caspase-9 levels accompanied by the appearance of cleaved caspase-9 fragments was observed following treatment with complex **1**. Proteolytic cleavage of pro-caspase-9 into its active fragments represents a hallmark of intrinsic apoptotic signaling.^44^

In addition to the upregulation of the pro-apoptotic proteins BAX and BAK, a downregulation of the antiapoptotic protein BCL-2 was observed after treatment with complex **1**. BCL-2 is a key regulator of mitochondrial integrity. It opposes apoptosis by inhibiting the activation of BAX and BAK at the outer mitochondrial membrane.^45^ Downregulation of BCL-2 shifts the BCL-2 family toward a pro-apoptotic state. Decreased BCL-2 expression supports the activation of the intrinsic apoptotic pathway in leukemia cells induced by complex **1**.

Downstream caspase signaling was further supported by processing of caspase-3, the central executioner caspase responsible for proteolytic cleavage of numerous cellular substrates during apoptosis.^46^ Western blot analysis revealed reduced levels of pro-caspase-3 accompanied by an increase in cleaved caspase-3, indicating activation of the terminal apoptotic machinery.

Overall, the observed protein expression patterns indicate that treatment with **1** induces apoptotic signaling, characterized by upregulation of the pro-apoptotic Bax and Bak proteins and activation of the initiator and executioner caspases. While activation of extrinsic apoptotic signaling cannot be entirely excluded, the combined changes in ROS levels, mitochondrial membrane potential, and caspase-9 activation observed in this study support involvement of the intrinsic pathway in apoptosis induction by complex **1**.^14,47^

### 2.8 1 Tested Against Leukemia Cells and Healthy Leukocytes

As selectivity is key to reducing side effects in patients and enhancing their quality of life,^4^ complex **1’s** selectivity was investigated. Leukemia cells and isolated healthy leukocytes were incubated with compound **1** at different concentrations. Healthy human leukocytes were isolated from a member of the research group.

Nalm-6 and healthy human leukocytes were incubated with complex **1** for 72 h, stained with propidium iodide and the DNA fragmentation was analyzed by flow cytometry. This represents cell death characterization by DNA loss.^48^ In contrast to Nalm-6 cells and sublines, in healthy human leukocytes, complex **1** shows almost no activity (see Figure 8). With an increase in concentration of **1**, no increase in activity was observed. In comparison, at 0.5 μM of complex **1**, 48.93% of Nalm-6 cells exhibit DNA fragmentation.

**Figure 8:**
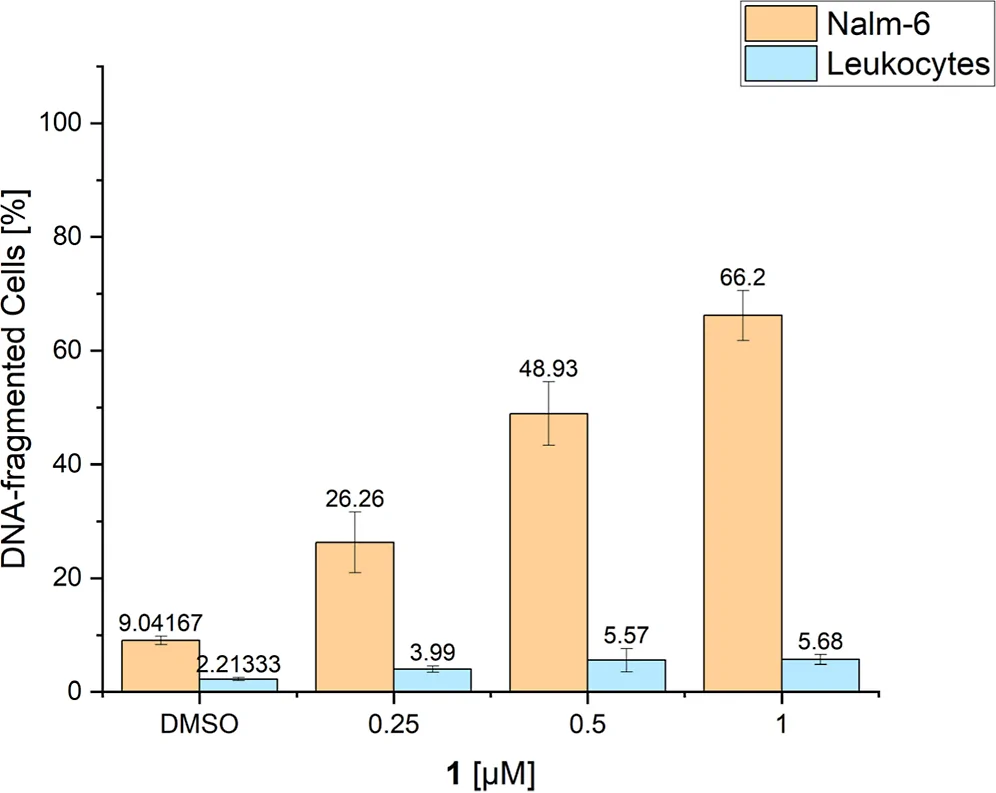
Healthy leukocytes from a healthy donor (ex vivo) and Nalm-6 cells were treated with complex **1**. Each cell line was incubated with **1** for 72 h. **1** barely induces DNA-fragmentation on human leukocytes, while it was observed in Nalm-6 cells. Two biological replicates (*n* = 2), each performed with three technical replicates.

### 2.9 Cytotoxicity of **L1**

A challenge when designing metallodrugs is distinguishing the activity of the complex from the cytotoxicity of its organic ligands. Inherent ligand cytotoxicity can increase the risk of off-target effects.^17,49^ Therefore, establishing the biological ‘innocence’ of the scaffold is essential for developing a well-defined gold­(I) anticancer agent.

To investigate if the ligand might play a role in the activity of complex **1**, the ligand precursor **L1** was tested against Nalm-6 cells. After incubation of Nalm-6 cells with different concentrations of **L1** (0.1, 0.5, 1, 2.5, 5 μM) for 72 h, the cells were stained with propidium iodide, and DNA fragmentation was analyzed by flow cytometry. No activity toward Nalm-6 cells can be detected up to 5 μM. Only at 10 μM of **L1**, a minimal percentage of cells with DNA fragmentation (5.62%) can be detected (see Supporting Information Figure S43).

### 2.10 Overcoming Resistant Leukemia Cell Lines

Especially in patients in relapse, a resistance to chemotherapeutics is the major challenge.^5^ Treatments of ALL cancer with steroids like prednisolone and cytostatics like etoposide, have high effectiveness,^50^ however, relapsed ALL patients often harbor cell resistance against these conventional therapies.^51^

Gold­(I) bis-NHC complexes are known to overcome resistance in cell lines.^8,19^ Therefore, the activity of complexes **1** against resistant Nalm-6 sublines, multidrug-resistant leukemia cells NaKu (prednison-resistant) and JeFri (etoposide-resistant) was investigated.^52^

After incubation of Naku and JeFri cells with different concentrations of complex **1** (0.1, 0.5, 1, 2.5, 5 μM) for 72 h, the cells were stained with propidium iodide, and the DNA fragmentation was analyzed by flow cytometry (see Figure 9). Additionally, the cells were tested against prednisolone and etoposide. The cytostatics had scarcely an effect on Naku and JeFri cells, whereas complex **1** displays a dose-dependent response in both resistant cell lines.

**Figure 9:**
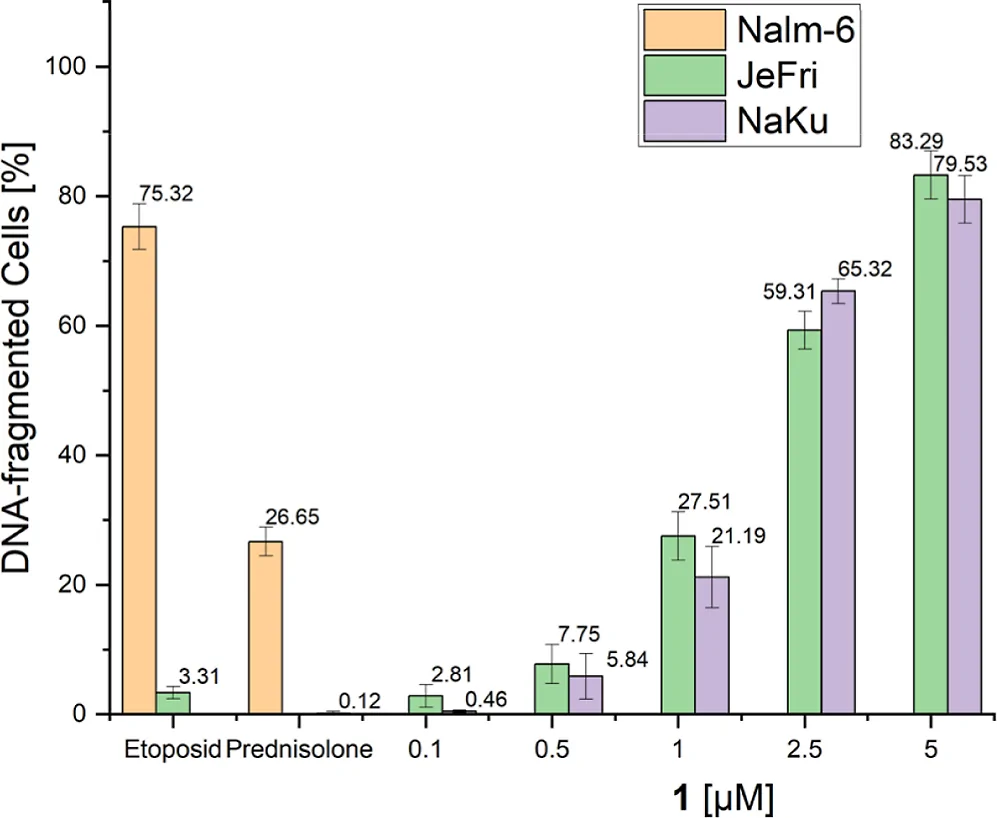
JeFri (etoposide resistant) and NaKu (prednisolone resistant) leukemia cells were treated with increasing concentrations of complex **1** for 72 h. Cells treated with etoposide and prednisolone served as controls. Afterward, the impact of **1** on the drug-resistant cells was measured via flow cytometry. Resistant cell lines showed lower levels of apoptosis upon treatment with their cytostatic controls compared to Nalm-6 cells. In contrast, cells treated with **1** induced an increase in apoptosis in both resistant cell lines, indicating an overcoming of drug resistance. Two biological replicates (*n* = 2), each performed with three technical replicates.

At 2.5 μM of complex **1**, 59.31 ± 2.9% (JeFri) and 65.32 ± 1.89% (Naku) cells exhibit DNA fragmentation, showing good activity against the resistant cell lines.

## 3 Conclusion

Au­(I) bis-NHC_thia_ complexes, containing thiazolium-based ligands, were synthesized and biologically evaluated. The complexes with different N-substituents (isopropyl, hexyl, phenyl, and mesityl) were characterized by ESI-MS, EA, ^1^H-, ^13^C-NMR, and their stability was examined. Even though all four complexes are stable in water, only those containing aromatic substituents show significant stability in the presence of thiols, which correlates with higher activity against different solid cancer cell lines (A2780, MDA-MB-231, MCF-7) and higher TrxR inhibition. In particular, the mesityl-containing complex **1** and the phenyl-containing complex **4** show high stability and high activity with IC_50_ values in the nanomolar range across the three cell lines and exceeding the potency of auranofin in MDA-MB-231 cells.

Due to complexes **1**’s and **4**’s high activity and stability, they were also investigated against leukemia cells (Nalm-6), exhibiting IC_50_ values in the nanomolar range. Western blot analysis, mitochondrial membrane potential analysis, and staining for reactive oxygen species (ROS) revealed that complex **1** induces ROS formation, leading to the activation of the pro-apoptotic proteins BAX and BAK, causing a loss of mitochondrial membrane potential, and initiating the intrinsic apoptosis pathway.

Furthermore, the activity of the ligand precursor **L1** against Nalm-6 cells was evaluated to confirm that the anticancer profile is driven by the gold­(I) moiety. The thiazolium salt **L1** does not show notable activity in Nalm-6 cells. The lack of ligand activity mitigates the risk of ligand-induced off-target effects,^17,49^ making NHC_Thia_ an interesting type of ligand class for metallodrugs.

Lastly, complex **1** has been shown to overcome drug resistance caused by the common cytostatics etoposide (JeFri) and prednisolone (NaKu). This property, along with its lack of activity against healthy human leukocytes, makes Au­(I) bis-NHC_thia_ viable cytotoxic agents and a promising framework for further exploration in broader biomedical research.

## 4 Experimental Section

### 4.1 Materials and Methods

Reagents were purchased from Sigma-Aldrich, TLC, and abcr and used without further purification. All reactions were performed under aerobic conditions unless otherwise stated. Dry solvents were obtained by a Mbraun solvent purification system. Chloro­(tetrahydrothiophene)­gold­(I) ([Au­(tht)­Cl]), 3-mesityl-4,5-dimethylthiazol-3-ium hexafluoro-phosphate **L1** and 3-phenyl-4,5-dimethylthiazol-3-ium hexafluorophosphate **L4** were obtained through synthesis according to the literature procedures.^30,32,53^

The NMR spectra were conducted on a Bruker AVANCE DPX 400 and AV500C. The chemical shifts are given in parts per million and referenced to the residual signal of deuterated solvent (dichloromethan-*d*_2_: 5.20 ppm, dimethylsulfide-*d*_6_: 2.50 ppm, chloroform-*d*: 7.26 ppm, acetonitrile-*d*_3_: 1.94 ppm). Analytical reversed-phase HPLC-HESI-MS was performed on an UltiMate 3000 UHPLC focused chromatographic system (Dionex) connected to an LCG Fleet mass spectrometer (thermo Scientific) equipped with a C18 column (Hypersil GOLD aQ, 150 × 2.1 mm, 3 μm). Elemental analyses (C/H/N/S) were performed by the microanalytical laboratory of the Technical University of Munich using a HEKAtech Euro EA-CHNS combustion analyzer.

### 4.2 Synthesis

#### 4.2.1 3-Isopropyl-4,5-dimethylthiazol-3-ium Iodide **L2a**

The synthesis of **L2a** was conducted according to a modified literature procedure.^54^ 3,4-Dimethyl thiazole (0.47 mL, 4.42 mmol, 1.00 equiv) was added into a predried Schlenk tube filled with dry acetonitrile (5 mL). Isopropyl bromide (3.01 g, 17.68 mmol, 4.0 equiv) was added to the solution. The reaction mixture was refluxed for 4 days at 80 °C. The solution was removed via *vacuo*, affording a black oil. The oil was extracted three times with 5 mL of water. The water was evaporated under reduced pressure, resulting in yellow crystals. The crystals were filtered, washed with cold acetone (3 × 5 mL), and dried *in vacuo*, yielding L2 (851 mg, 3.00 mmol, 68%) as colorless crystals.

^1^H NMR (400 MHz, CD_3_CN, δ [ppm]): 9.69 (1s, 1H, CH_thia_), 4.83 (sp, *J* = 6.6 Hz, 1H, CH), 2.49 (s, 3H, CH_3thia_), 2.45 (s, 3H, CH_3_), 1.60 (d, *J* = 6.6 Hz, 6H, CH_3ipr_).

^13^C­{^1^H}-NMR (101 MHz, CD_3_CN, δ [ppm]): 153.1 (CH_thia_), 143.3 (N–C_thia_), 134.6 (S–C_thia_), 57.6 (N–CH_iPr_), 22.8 (CH_3ipr_), 12.7 (CH_3thia_), 12.1 (CH_3thia_).

**EA**: Calcd for C_8_H_14_NSI (%): C, 33.93; H, 4.98; N, 4.95; S, 11.32. Found: C, 34.28; H, 4.87; N, 4.97; S, 11.29.

#### 4.2.2 3-Hexyl-4,5-dimethylthiazol-3-ium Hexafluorophosphate **L3**

The synthesis of **L3** was conducted according to a modified literature procedure.^54^ 3,4-Dimethyl thiazole (0.56 mL, 5.3 mmol, 1.00 equiv) was added into a predried pressure tube filled with dry acetonitrile (5 mL). Hexyl bromide (2.97 mL, 21.2 mmol, 4.0 equiv) was added to the solution. The reaction mixture was refluxed for 4 d at 80 °C. The solution was removed via *vacuo*, affording a yellow oil. The oil was solved in a mixture of acetonitrile/water (1:2), before the addition of ammonium hexafluorophosphate (1.76 g, 10.78 mmol, 2.00 equiv). The solution was stirred for 16 h. Afterward, the solution was extracted three times with dcm. The organic phases were combined and extracted one time with brine and on time with water. The organic phase was dried over MgSO_4_, filtered and the solvent was removed via *vacuo*, yielding **L3** (0.885 mg, 72%) as colorless crystals.

^1^H NMR (400 MHz, CD_3_CN,δ [ppm]): 9.34 (1H, CH_thia_), 4.30 (t, *J* = 7.2 Hz, 2H, CH_2_), 2.47 (s, 3H, CH_3thia_), 2.40 (s, 3H, CH_3thia_), 1.82 (p, *J* = 7.2 Hz, 2H, CH_2_), 1.38–1.30 (m, 6H, CH_2_), 0.90 (t, *J* = 7.09 Hz, 3H, CH_3_).

^13^C­{^1^H}-NMR (101 MHz, CH_3_CN, δ [ppm]): 154.4 (CH_thia_), 143.4 (N–C_thia_), 134.9 (S–C_Thia_), 54.7 (N–CH_2Hex_), 31.8 (CH_2Hex_), 30.0 (CH_2Hex_), 26.4 (CH_2Hex_), 23.1 (CH_2Hex_), 14.2 (CH_3Hex_), 12.7 (CH_3thia_), 11.8 (CH_3thia_).

EA: Calcd for C_11_H_20_F_6_NPS (%): C, 38.48; H, 5.87; N, 4.08; S, 9.34. Found: C, 38.52; H, 5.80; N, 4.27; S, 9.11.

#### 4.2.3 Bis­(3-mesityl-4,5-dimethyl-3,4-thiazol-2-yl)­gold­(I) Hexafluorophosphate **1**

The reaction was performed under the exclusion of light. **L1** (188 mg, 499 μmol, 2.00 equiv), [Au­(tht)­Cl] (80 mg, 250 μmol, 1.00 equiv) and K_2_CO_3_ (103 mg, 749 μmol, 3.00 equiv) were dissolved in acetone (10 mL). The reaction mixture was stirred at room temperature for 16 h. The reaction was monitored via ESI-MS. After full completion, the precipitants were filtered over Celite, and the excess solvent was removed under reduced pressure. The off-white residue was dissolved in CH_2_Cl_2_ (4.0 mL), filtered and precipitated with Et_2_O (6.0 mL) in the fridge overnight. The excess solvent was removed, and the colorless crystals were washed with diethyl ether (15 mL). The colorless solid was dried *in vacuo*.

**1** was obtained as a colorless solid (95.3 mg, 118 μmol, 47%).

^1^H NMR (400 MHz, CD_2_Cl_2_, δ [ppm]): 6.93 (s, 4H, CH_Mes_), 2.42 (s, 6H, N–CH_3_), 2.41 (s, 6H, S–CH_3_), 1.86 (s, 6H, CH_3Mes_), 1.63 (s, 12H, CH_3Mes_).

^13^C­{^1^H}-NMR (101 MHz, CD_2_Cl_2_, δ [ppm]): 204.6 (Carbene), 141.7 (N–C_NHC_), 140.0 (C_Mes_), 137.6 (C_Mes_), 133.6 (C_Mes_), 132.2 (S–C_NHC_), 129.6 (CH_Mes_), 21.0 (CH_3Mes_), 17.3 (CH_3Mes_), 12.6 (CH_3NHC_), 11.9 (CH_3NHC_).

ESI-MS *m*/*z* [1 – PF6]^+^ calcd 659.18; found, 659.71.

HR ESI-MS *m*/*z* [1 – PF6]^+^ calcd 659.1824; found, 659.1812.

**EA** Calcd for C_28_H_34_AuF_6_N_2_PS_2_ (%): C, 41.80; H, 4.26; N, 3.48; S, 7.97. Found: C, 41.92; H, 4.06; N, 3.63; S, 8.12.

#### 4.2.4 Bis­(3-isopropyl-4,5-dimethyl-3,4-thiazol-2-yl)­gold­(I) Iodide **2a**

The reaction was performed under the exclusion of light. In a pressure tube, **L2a** (200 mg, 706 μmol, 2.00 equiv), [AuCl­(tht)] (113 mg, 353 μmol, 1.00 equiv), and K_2_CO_3_ (146 mg, 1.06 mmol, 3.00 equiv), were dissolved in 16 mL of acetone. The reaction mixture was heated to 65 °C and stirred in a pressure tube for 72 h. After cooling to r.t., the solution was filtered through Celite and removed via vacuo. The crude product was dissolved in 5 mL DCM, the resulting solution was filtered, and 15 mL diethyl ether was added slowly. Slow diffusion of the diethyl ether layer into DCM overnight resulted in colorless crystals, which were collected by filtration, washed with diethyl ether, and dried in *vacuo* (116 mg, 180 μmol, 52%).

^1^H NMR (400 MHz, CD_3_CN, δ [ppm]): 5.07–5.00 (m, 2H, C*H*_ipr_), 2.41 (s, 6H, CH_3NHC_), 2.37 (s, 6H, CH_3NHC_), 1.80 (d, *J* = 6.71 Hz, 12H, CH_3ipr_).

ESI-MS *m*/*z*: [**2a** – I]^+^ calcd 507.12; found, 507.30.

EA: Calcd for C_16_H_26_AuN_2_S_2_I (%): C, 30.29; H, 4.13; N, 4.42; S, 10.11. Found: C, 30.12; H, 3.85; N, 4.40; S, 10.27.

#### 4.2.5 Bis­(3-isopropyl-4,5-dimethyl-3,4-thiazol-2-yl)­gold­(I) Hexafluorophosphate **2**

**2a** (38 mg, 59.9 μM, 1.00 equiv) were solved in a mixture of acetonitrile/water (1:2). Under constant stirring, ammonium hexafluorophosphate (71.69 mg, 0.44 mM, 7.34 equiv) was added to the solution. The resulting reaction mixture is stirred for 16 h at room temperature. Afterward, the solution is extracted with DCM (8 mL × 3). The combined organic phases are extracted once with brine (10 mL) and once with water (10 mL). The organic phase is dried over MgSO_4_, filtered and the solvent is removed under reduced pressure. The resulting white powder was dried *in vacuo*, resulting in (35 mg, 53.6 μM, 89%) of **2**.

^1^H NMR (400 MHz, CD_3_CN, δ [ppm]): 5.04–5.01 (m, 2H, C*H*_ipr_), 2.41 (s, 6H, CH_3NHC_), 2.37 (s, 6H, CH_3NHC_), 1.80 (d, *J* = 6.71 Hz, 12H, CH_3ipr_).

^13^C­{^1^H}-NMR (101 MHz, CD_3_CN, δ [ppm]): 199.6 (Carbene), 143.8 (N–C_NHC_), 131.9 (S–C_NHC_), 56.0 (N–CH_iPr_), 24.3 (CH_3ipr_), 13.2 (CH_3NHC_), 12.4 (CH_3NHC_).

ESI-MS *m*/*z*: [**2** – PF_6_]^+^ calcd 507.12; found, 507.35, HR ESI-MS *m*/*z*: [**2a** – I]^+^ calcd 507.1198; found, 507.1200.

**EA**: Calcd for C_16_H_26_AuF_6_N_2_PS_2_ (%): C, 29.45; H, 4.02; N, 4.29; S, 9.83. Found: C, 29.66; H, 4.03; N, 4.22; S, 9.73.

#### 4.2.6 Bis­(3-hexyl-4,5-dimethyl-3,4-thiazol-2-yl)­gold­(I) Hexafluorophosphate **3**

The reaction was performed under the exclusion of light. **L3** (200 mg, 0.58 mmol, 2.00 equiv), [Au­(tht)­Cl] (93,38 mg, 0.29 mmol, 1.00 equiv) and K_2_CO_3_ (161.0 mg, 1.17 mmol, 4.00 equiv) were dissolved in acetone (10 mL). The reaction mixture was stirred for 16 h at room temperature. Afterward, the solution was filtered over Celite, and the solvent was removed via *vacuo*. The off-white residue was dissolved in CH_2_Cl_2_ (4.0 mL), layered with Et_2_O (6.0 mL) and placed in the fridge overnight. The colorless crystals were filtered, washed with Et_2_O (15 mL) and dried *in vacuo*. The complex **3** was obtained as a colorless solid (168.5 mg, 0.23 mmol, 79%).

^1^H NMR (400 MHz, CH_3_CN, δ [ppm]): \4.44 (t, *J* = 8.05 Hz 4H, N–CH_2Hex_), 2.37 (s, 6H, CH_3NHC_), 2.37 (s, 6H, CH_3NHC_), 1.91–1.87 (m, 4H, N–CH_2_–CH_2Hex_), 1.45–1.31 (m, 12H, CH_2Hex_) 0.87 (t, *J* = 8.05 Hz, 6H, CH_3Hex_).

^13^C­{^1^H}-NMR (101 MHz, CH_3_CN, δ [ppm]): 203.4 (Carbene), 143.1 (N–C_NHC_), 133.2 (S–C_NHC_), 57.0 (N–CH_2Hex_), 32.0 (CH_2Hex_), 31.5 (CH_2Hex_), 26.9 (CH_2Hex_), 23.2 (CH_2Hex_), 14.2 (CH_3Hex_), 12.5 (CH_3NHC_), 12.4 (CH_3NHC_).

ESI-MS: *m*/*z* [**3** – PF_6_]^+^ calcd 591.21; found, 591.59.

HR ESI-MS *m*/*z*: [**2a** – I]^+^ calcd 591.2146; found, 591.2137.

**EA**: Calcd for C_22_H_38_AuF_6_N_2_PS_2_ (%): C, 35.87; H, 5.20; N, 3.80; S, 8.70. Found: C, 35.92; H, 5.07; N, 3.74; S, 8.77.

#### 4.2.7 Bis­(3-phenyl-4,5-dimethyl-3,4-thiazol-2-yl)­gold­(I) Hexafluorophosphate **4**

The reaction was performed under the exclusion of light. **L4** (200 mg, 0.60 mmol, 2.00 equiv), [Au­(tht)­Cl] (95.6 mg, 0.30 mmol, 1.00 equiv) and K_2_CO_3_ (164.9 mg, 1.19 mmol, 4.00 equiv) were dissolved in acetone (10 mL). The reaction mixture was stirred for 2 h at 45 °C. Afterward, the solution was filtered over Celite, and the solvent was removed via *vacuo*. The green oil was purified via column chormatographie (2.5 cm, 10 cm, silica gel, 10% methanol in DCM). The resulting product was dried *in vacuo*. Complex **4** was obtained as a colorless solid (163.5 mg, 0.23 mmol, 76%).

^1^H NMR (400 MHz, CD_3_CN, δ [ppm]): 7.63–7.59 (m, 2H, CH_Phen_), 7.51 (t, *J* = 7.82 Hz, 4H, CH_Phen_), 7.24 (d, *J* = 7.82 Hz, 4H, CH_Phen_) 2.37 (s, 6H, CH_3NHC_), 2.01 (s, 6H, CH_3NHC_).

^13^C­{^1^H}-NMR (101 MHz, CD_3_CN, δ [ppm]): 204.6 (Carbene), 143.9 (N–C_NHC_), 143.0 (S–C_NHC_), 132.7 (C_Phen_), 131.1 (CH_Phen_), 130.8 (CH_Phen_), 127.3 (CH_Phen_), 13.1 (CH_3NHC_), 12.3 (CH_3NHC_).

ESI-MS *m*/*z* [**4** – PF_6_]^+^ calcd 575.09; found, 575.44.

HR ESI-MS *m*/*z* [**4** – PF_6_]^+^ calcd 575.0885; found, 575.0881.

**EA** Calcd for C_22_H_22_AuF_6_N_2_PS_2_ (%): C, 36.68; H, 3.08; N, 3.89; S, 8.90. Found: C, 37.04; H, 2.90; N, 3.93; S, 9.16.

### 4.3 ^1^H NMR-Activity Studies

All ^1^H NMR-activity studies were conducted in degassed DMSO-*d*_6_.

#### 4.3.1 Activity toward Water

Compounds **1**, **2**, **3**, and **4** are tested against their stability in water. Therefore, the compounds (2.50 μM) are dissolved in 400 μL of DMSO-*d*_6_. A ^1^H NMR spectra of the individual compounds were measured before 10 μL of water was added to each solution. Afterward, a ^1^H NMR spectrum was taken after 24, 48, and 72 h.

#### 4.3.2 Activity Toward *N*-Acetylcysteine

The stability toward *N*-acetylcysteine (NAC) was investigated for **1**, **2**, **3**, and **4**. Therefore, the compounds (2.50 μM, 1.00 equiv) are dissolved in 400 μL of DMSO-*d*_6_, and 0.5 μL of benzene was added for **1**, **2**, and **3** and 1 μL of DCM was added for **4**. A ^1^H NMR spectrum of the individual compounds was measured, before a 100 μL DMSO-*d*_6_ solution of *N*-acetylcysteine (1.22 mg, 7.5 μM, 3.00 equiv) was added to each complex solution. An ^1^H NMR spectra was obtained every after 30 min, 4 h, 24 h, 48 h, 72 h. An ESI-MS Spectra was taken after 48 h.

### 4.4 Biological Studies

#### 4.4.1 Cell Lines

Cells from human solid tumors A2780 (ovarian, Sigma-Aldrich), MDA-MB-231 and MCF-7 (breast, ATCC) and nonsolid tumors Nalm-6 (B-lymphoblastic leukemia, Cytion, product number 300297) their corresponding drug-resistant sublines and BJAB cells (B-cell lymphoma, Cytion, product number 302006) were used. The drug-resistant sublines were specifically produced.

#### 4.4.2 Cell Media

Ovarian A2780 cells were grown in RPMI 1604 medium, breast cells MDA-MB-231 and MCF-7 were grown in DMEM + GlutaMAX media (Gibco, Invitrogen, Karlsruhe, Germany), supplemented with 10% FBS (FBS; Merck KGaA, Darmstadt, Germany) and maintained at 37 °C in an incubator with 5% CO_2_.

Leukemia cells were maintained in RPMI 1604 supplemented with 10% FBS and 1% penicillin–streptomycin (ThermoFisher Scientific Inc., Waltham, USA) and maintained at 37 °C in an incubator with 5% CO_2_.

#### 4.4.3 Studies with Cells from Solid Tumors

##### 4.4.3.1 Cytotoxic Activity

The cytotoxic activity of the complexes was evaluated by using the colorimetric MTT assay, following a similar procedure as previously described.^55^ For the assays, cells (∼2 × 10^4^ cells/200 μL medium) were seeded in 96-well plates and left to adhere for 24 h. Complexes were first diluted in DMSO and then in medium to prepare serial dilutions in the range 0.001–50 μM. The reference drugs cisplatin and auranofin were first diluted in water (cisplatin) or DMSO (auranofin) and then in medium to prepare serial dilutions in the range 0.1–100 μM. Each compound’s dilution (200 μL) was added to the cells and incubated for 48 h at 37 °C. After, the medium was discarded and 200 μL of MTT solution in PBS (0.5 mg/mL) were applied to each well, Results are shown as the mean ± SD of at least two independent experiments done with six replicates per condition.

##### 4.4.3.2 Thioredoxin Inhibition Assay

The TrxR inhibition assays were performed using a commercial kit from Sigma-Aldrich, and following the instructions introducing modifications for a 96-well plate format and using lysates from MCF-7 and MDA-MB-231 cells. The assay is based on the reduction of DTNB (5,5′ dithiobis­(2-nitrobenzoic acid)) into TNB (5-thio-2-nitrobenzoic acid) with the concomitant oxidation of NADPH, in a reaction catalyzed by TrxR. Briefly, ∼2 × 10^6^ cells were first treated with the complexes at 20 μM for 2 h, and then homogenized on ice in 200 μL cold TrxR assay buffer for 30 min. Lysates were centrifuged at 10,000*g* for 15 min at 4 °C. After, 50 μL of cell lysates were introduced into each well, the total volume of 200 μL adjusting with the reaction mix (150 μL) containing assay buffer (140 μL) NADPH (2 μL) and DTNB (8 μL). A positive control (no complexes) were included in the assays. The formation of TNB was monitored after 20 min at 412 nm using a plate spectrophotometer (Power Wave Xs, Bio-Tek, Marshall Scientific, USA).

#### 4.4.4 Studies with Cells from Nonsolid Tumors

Cell lines were maintained in RPMI 1640 medium (Gibco, Invitrogen, Karlsruhe, Germany). They were supplemented with 10% (v/v) heat-inactivated fetal bovine serum (FBS; Merck KGaA, Darmstadt, Germany) and 1% (v/v) penicillin–streptomycin (ThermoFisher Scientific Inc., Waltham, USA). Cell lines were cultivated at 37 °C with 5% CO_2_.

Cultures were split twice weekly. Cells were seeded into 75 cm^2^ culture flasks at a density of 0.5 × 10^5^ cells/mL. The suspension cells were adjusted 24 h prior to treatment to 3 × 10^5^ cells/mL to ensure standardized growth conditions. On the day of the experiment, cells were transferred into 12-well plates at a density of 1 × 10^5^ cells/mL in 2 mL RPMI 1640 per well.

Depending on each assay, **1** and reference cytostatic agents were added at the indicated concentrations and incubated for the specified time periods. A 40 mM stock solution of complex **1** in dimethyl sulfoxide (DMSO; Serva Electrophoresis GmbH, Heidelberg, Germany) was prepared. Including vehicle controls, the final DMSO concentrations did not exceed 0.5% (v/v).

#### 4.4.5 Cell Concentration and Cellular Viability

Cell concentration and viability of all suspension cells were measured using the CASYCell Counter and Analyzer System (OMNI Life Science GmbH, Bremen, Germany). The CASYCell Counter had a specific set up for each cell line. 20 μL of the suspension was transferred into 10 mL CASYton (isotonic saline solution) and measured. Result was multiplied by five to define number of cells per mL. Cells treated with 0.5% DMSO in cell culture medium are used as the reference for the maximum cell count and as a negative control.

#### 4.4.6 DNA Fragmentation

Nalm-6 cells were treated with 1 for 72 h. Cells were collected by centrifugation (5900, 5 min, 4 °C). The cell pellet was resuspended in 200 μL 2% (v/v) formaldehyde (Carl Roth GmbH, Karlsruhe, Germany) and incubated on ice for 30 min. Afterward, the cells were centrifuged (370*g*, 5 min, 4 °C), resuspended in 180 μL 2:1 (v/v) ethanol/1× PBS (Merck KGaA, Darmstadt, Germany; ThermoFisher Scientific Inc., Waltham, USA), following another 15 min of incubation on ice. After incubation, cells were centrifuged again and resuspended in 50 μL RNase A solution (10 mg/mL in 1× PBS; VWR International GmbH, Darmstadt, Germany) for 30 min at 37 °C. The RNase solution removed residual RNA. Cells are centrifuged again (370*g*, 5 min, 4 °C) and stained with 200 μL of 1× PBS containing 50 μg/μL propidium iodide (PI). As a DNA-intercalating dye, PI enables detection of hypodiploid nuclei indicative of apoptotic DNA fragmentation. The DNA fragmentation was quantified by flow cytometry using the FACS Lyric system (Becton Dickinson GmbH, Heidelberg, Germany). Data evaluation was performed using CellQuest software.

#### 4.4.7 Reactive Oxygen Species

Detection of ROS was evaluated using the CellROX Green Reagent (Thermo Fisher Scientific Inc., Waltham, USA, product number 3281125) in accordance with the instructions, with minor adjustments. Hydrogen peroxide (30% H_2_O_2_) was used as a positive control in 1:100 dilution. Fluorescence intensity corresponding to ROS levels was quantified by flow cytometry. Nalm-6 cells were incubated with NAC for 1.5 h at 37 °C. NAC was dissolved in 500 μL aqua dest. Nalm-6 cells were pretreated with NAC for 1.5 h at 37 °C. Subsequently, 2 μL of the 1:100 dilution of the 30% H_2_O_2_ stock solution was added per 2 mL cell suspension for the H_2_O_2_ control. Complex 1 was added at the indicated concentrations, and a DMSO vehicle control was included for normalization. Cells were then incubated for 3.5 h at 37 °C. Reagent (1:10 dilution) was added. Cells were incubated for 1 h and measured. Experiments were performed in two biological replicates, each with three technical replicates.

#### 4.4.8 Determination of Mitochondrial Membrane Potential Depolarization

To detect mitochondrial membrane potential depolarization, the fluorescent 5,5′,6,6′-tetrachloro-1,1′,3,3′-tetraethylbenzimidazolylcarbocyanine iodide (JC-1) is applied. Cells are first incubated with the gold complexes for 48 h. After the incubation, cells are collected by centrifugation (800*g*, 5 min, 4 °C). The resulting cell pellet is resuspended in phenol red-free RPMI medium. 6.25 μL of JC-1 (Thermo Fisher Scientific, Waltham, MA, USA, product number 3095169) was added and the suspension was incubated (30 min, 37 °C) to allow dye uptake. After this, the cells were centrifuged again at (1500*g*, 5 min, 4 °C). The cell pellet was resuspended in 1× PBS. The proportion of cells showing mitochondrial membrane potential depolarization was quantified by flow cytometry using CellQuest software.

#### 4.4.9 Apoptosis and Necrosis by Annexin V/Propidium Iodide Staining

Different stages of apoptosis and necrosis are determined using the Annexin-V staining kit (ThermoFisher Scientific Inc., Waltham, USA, product number 468908-000). After 48 h of treatment with **1**, Nalm-6 cells were centrifuged (8000 rpm, 5 min, 4 °C), resuspended in 200 μL 1× PBS and centrifuged again (1500 rpm, 5 min, 4 °C). After centrifugation binding buffer was diluted in 1:4 and cells were washed in 200 μL. 195 μL binding buffer and 5 μL Annexin V was charged in each well except Control and Control/propidium iodide. Following 10 min of incubation at room temperature. Afterward the cells were washed and centrifuged again (1500 rpm, 5 min, 4 °C) and 190 μL binding buffer and 10 μL propidium iodide was charged in each well except Control and Control/Annexin. Samples were analyzed by flow cytometry, and data were evaluated using Cell Quest software.

#### 4.4.10 Protein Isolation and Western Blot Analysis

Protein expression studies were conducted using cells treated for 6, 12, 18, and 24 h with **1**, while DMSO vehicle served as control. Whole-cell lysates were prepared in RIPA buffer (50 mM Tris–HCl, 150 mM NaCl, 1% Triton X-100, 0.1% SDS, 1 mM EDTA, pH 7.4) supplemented with protease inhibitors (Roche, Mannheim, Germany). Following incubation on ice for 60 min, lysates were clarified by centrifugation (12,000*g*, 30 min, 4 °C). Supernatants containing total protein were stored at −80 °C until further analysis.

Protein concentrations were determined using the bicinchoninic acid (BCA) assay (Thermo Scientific, Rockford, USA) based on a bovine serum albumin (BSA) standard curve to ensure equal loading (40 μg per sample). Samples were mixed with 4× Laemmli buffer (12% SDS, 25% glycerol, 150 mM Tris–HCl, 0.03% bromophenol blue, 20% β-mercaptoethanol) and denatured at 95 °C for 5 min. Proteins were separated by SDS-PAGE on 12% polyacrylamide gels using Laemmli running buffer (Serva, Heidelberg, Germany) and subsequently transferred to nitrocellulose membranes with Towbin transfer buffer (Serva, Heidelberg, Germany).

Uniform protein transfer was verified by Ponceau Red staining. Membranes were blocked in 5% (w/v) nonfat milk prepared in PBST (1× PBS, 0.05% Tween-20). Primary antibodies were diluted in 5% BSA in PBST and incubated with the membranes for 2 h at room temperature. The following antibodies were used: anticaspase-9 (R&D Systems, Minneapolis, USA) anticaspse-8, anticaspase-3, anti-Bak and anti-BAX (Proteintech Europe, Manchester, UK); and anti-β-actin (Sigma, St. Louis, USA). After three washing steps in PBST, membranes were incubated with the appropriate HRP-conjugated secondary antibodies (antimouse IgG, antirabbit IgG, antigoat IgG; Promega, Madison, USA) diluted in 5% milk in PBST for 2 h at room temperature.

Following additional washes, protein bands were visualized using ECL Western Blotting Detection Reagents (GE Healthcare, Chicago, USA) and SuperSignal West Pico substrate (Thermo Scientific, Rockford, USA). Chemiluminescent signals were recorded with the Imager Chemi Premium system (VWR, Pennsylvania, USA). Densitometric evaluation was performed using VWR Image Capture Software, and target protein signals were normalized to β-actin as a loading control.

#### 4.4.11 Isolation of Peripheral Blood Leukocytes from Healthy Donors

To investigate the compound selectivity in nonmalignant proliferating human cells, leukocytes were isolated from peripheral blood obtained from a healthy donor. The blood (40 mL) was donated by a member of our working group. It was diluted with 14 mL RPMI 1640 medium supplemented with 20% (v/v) heat-inactivated fetal bovine serum and 1% (v/v) penicillin/streptomycin. Four mL Lymphosep solution (Biowest, Nuaillé, France) was layered beneath each 5 mL portion of diluted blood to generate a buffy coat after centrifugation (2000 rpm, 18 min, 18 °C).

The leukocyte-containing interphase was carefully collected, transferred to a 50 mL tube, diluted 1:1 with RPMI medium, and centrifuged again (2000 rpm, 5 min, 18 °C). The resulting cell pellet was resuspended in 10 mL RPMI medium containing 20% FBS and seeded into 12-well plates at a density of 3 × 10^6^ cells/mL.

#### 4.4.12 Statistical Evaluation

All data were collected in duplicates. Each with three technical replicates. Results are presented as mean values ±standard deviation (SD). SD was indicated as error bars in the figures. Statistics and graphical representations were generated using Microsoft Excel and OriginLab.

## Supplementary Material





## Data Availability

NMR, ESI-MS, and UV–vis spectra, SC-XRD data, and dose-dependent curves supporting this article have been included as part of the Supporting Information.

## Notes

NMR, ESI-MS spectra of the newly synthesized compounds, crystallographic information, dose–response curves, tables of proliferation UV–vis, ^1^H NMR, and corresponding ESI-MS of stability studies against small molecules (PDF)
All stain experiments are also given (PDF)

## Abbreviations

NMR: nuclear magnetic resonance spectroscopy
ESI-MS: electrospray ionization mass spectrometry
DMSO: dimethyl sulfoxide
EA: elemental analysis
JC-1: 5,5′,6 6′-tetrachloro-1,1′,3,3′-tetra-ethylbenzimidazoyl-carbocyanide iodide
CCCP: carbonyl cyanide *m*-chlorophenyl hydrazone
MTT: (3-4,5-dimethylthiazol-2-yl)-2,5-diphenyltetrazolium bromide
NHC_thia_: thiazolium-based *N*-heterocyclic carbenes
