# Supplementary material for: Synthesis, Cytotoxicity, and Mechanistic Evaluation of Thiazolium-Based Gold(I) Bis‑N‑Heterocyclic Carbene Complexes
Source: Inorg Chem. 2026 Oct 10;65(42):24706–19. doi: 10.1021/acs.inorgchem.6c02992 (PMC13715578; doi:10.1021/acs.inorgchem.6c02992)
Supplement: Supplementary file 1 [file ic6c02992_si_001.pdf]

Supporting Information

Synthesis, Cytotoxicity, and Mechanistic Evaluation of Thiazolium-based Gold(I) bis-N-Heterocyclic Carbene Complexes

Melanie E. Hoffmann,[+a] Lisa C. Haas[+b], Fernanda Marques[c], Sarah Sarwary[b], Charlotte Theile[d,e], Bakr Mustafa[b,d], João D. G. Correia[c], Aram Prokop[b,d], Fritz E. Kühn[a]**

[+]Equally contributed

*Corresponding Authors; Email: aram.prokop@helios-gesundheit.de; fritz.kuehn@ch.tum.de

[a]Technical University of Munich, TUM School of Natural Science, Department of Chemistry and Catalysis Research Centre, Molecular Catalysis, Lichtenbergstr. 4, 85748 Garching bei München, Germany

[b]MSH Medical School Hamburg, Department of Human Medicine, am Kaiserkai 1, 20457 Hamburg, Germany

[c] Centro de Ciências e Tecnologias Nucleares and Departamento de Engenharia e Ciências Nucleares, Instituto Superior Técnico, Universidade de Lisboa, CTN, Estrada Nacional 10 (km 139,7) 2695-066 Bobadela LRS, Portugal

[d]HELIOS Clinics Schwerin, Department of Pediatric Hematology/Oncology, Wismarsche Strasse 393-397, 19055 Schwerin, Germany

[5]Universität Potsdam, Karl-Liebknecht-Str. 24-25, 14476, Potsdam, Germany

Table of Contents

NMR Spectra....3
ESI-MS Spectra....12
Stability Studies....15
Single Crystal X-Ray Diffraction....21
Biological Studies....23
  Determination of Anitproliferattive Activity – MTT Assay....23
  Investigations in Nalm6 and BJAB cells....24
References....34

NMR Spectra

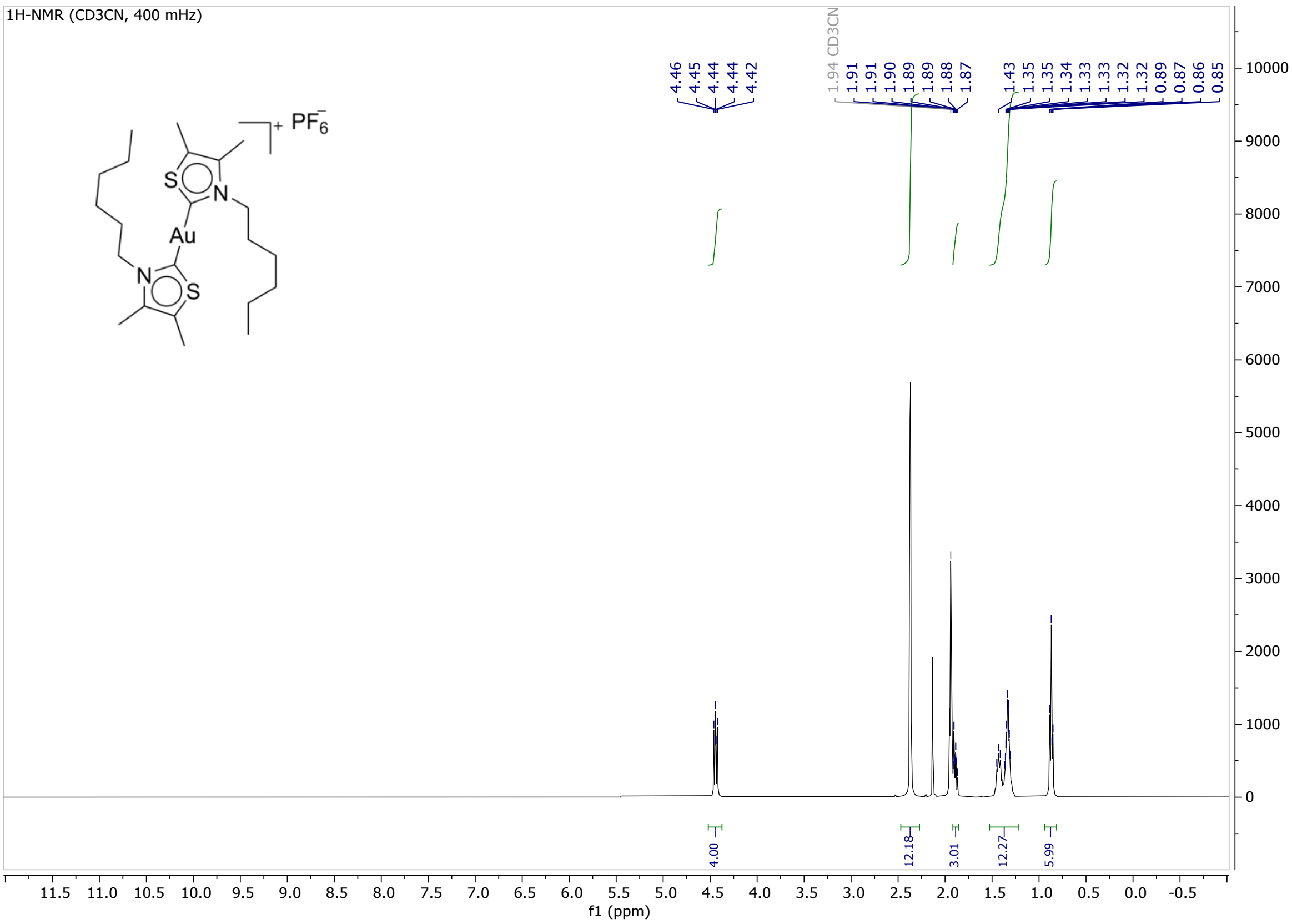

Figure S1: ^{1}H-NMR of complex 3 solved in acetonitrile-*d*$_3$.

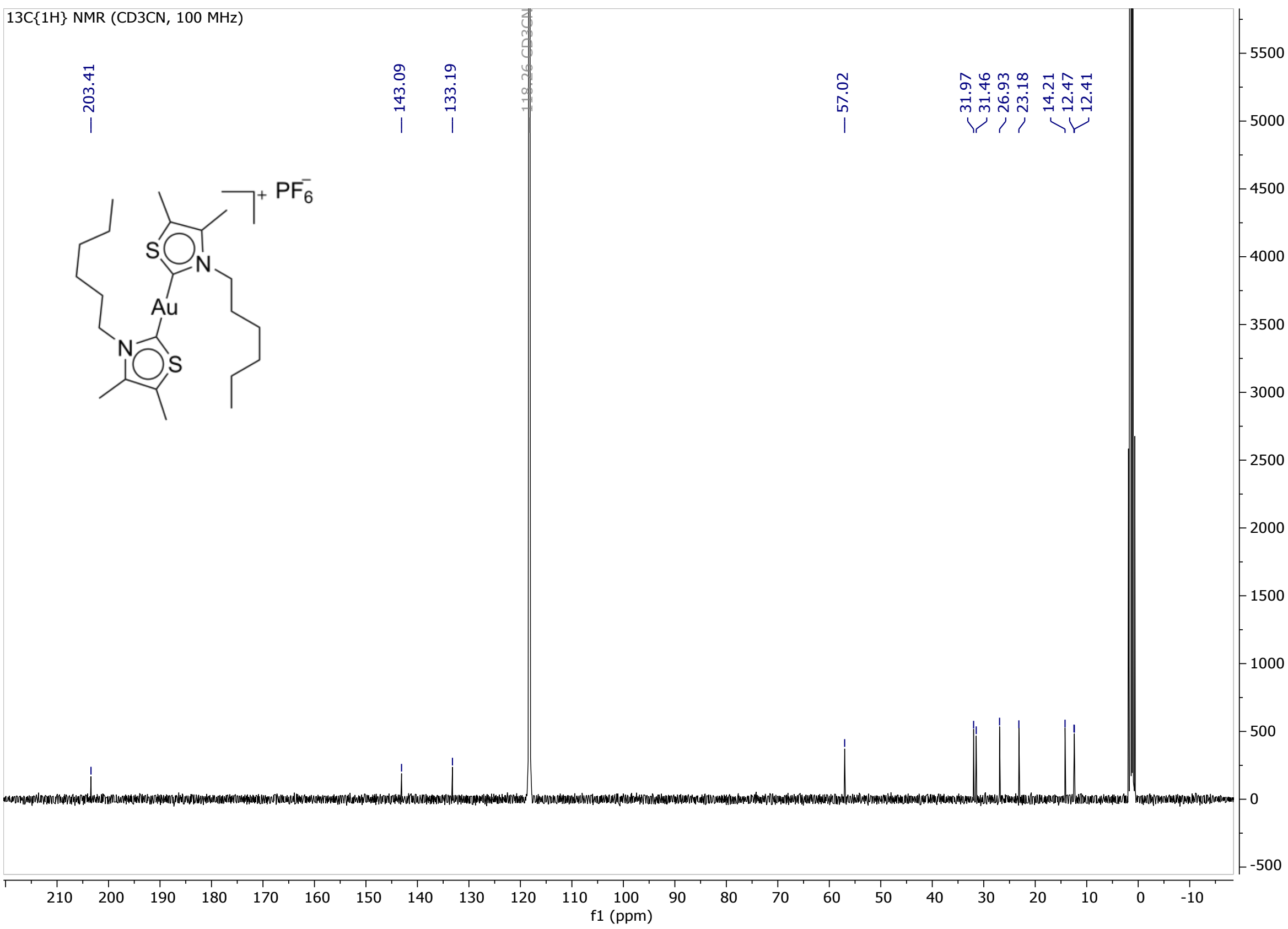

Figure S2: ^{13}C{^{1}H}-NMR of complex 3 solved in acetonitrile-*d*$_3$.

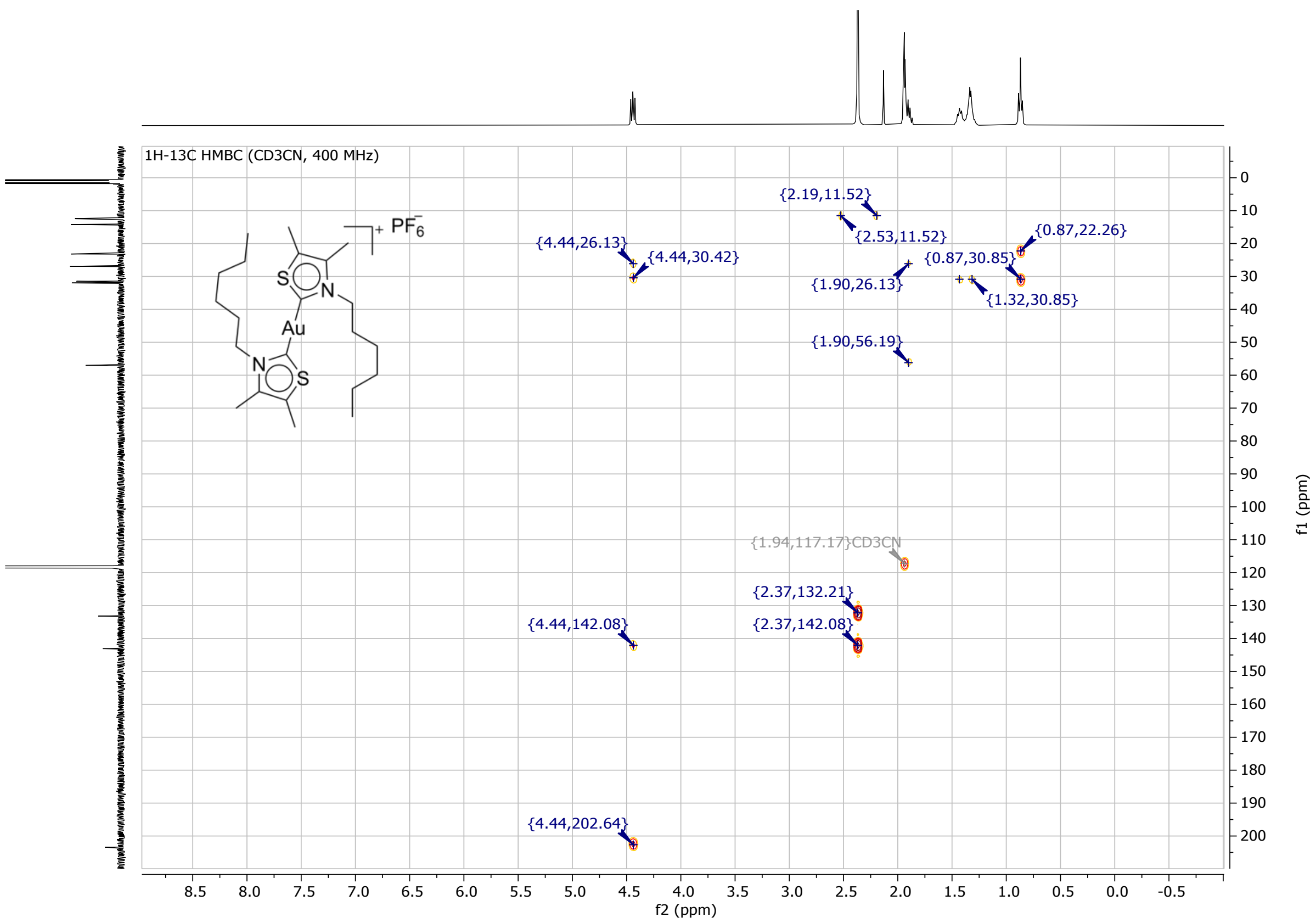

Figure S3: ^{1}H-^{13}C--HMBC-NMR of complex 3 solved in acetonitrile-d_3.

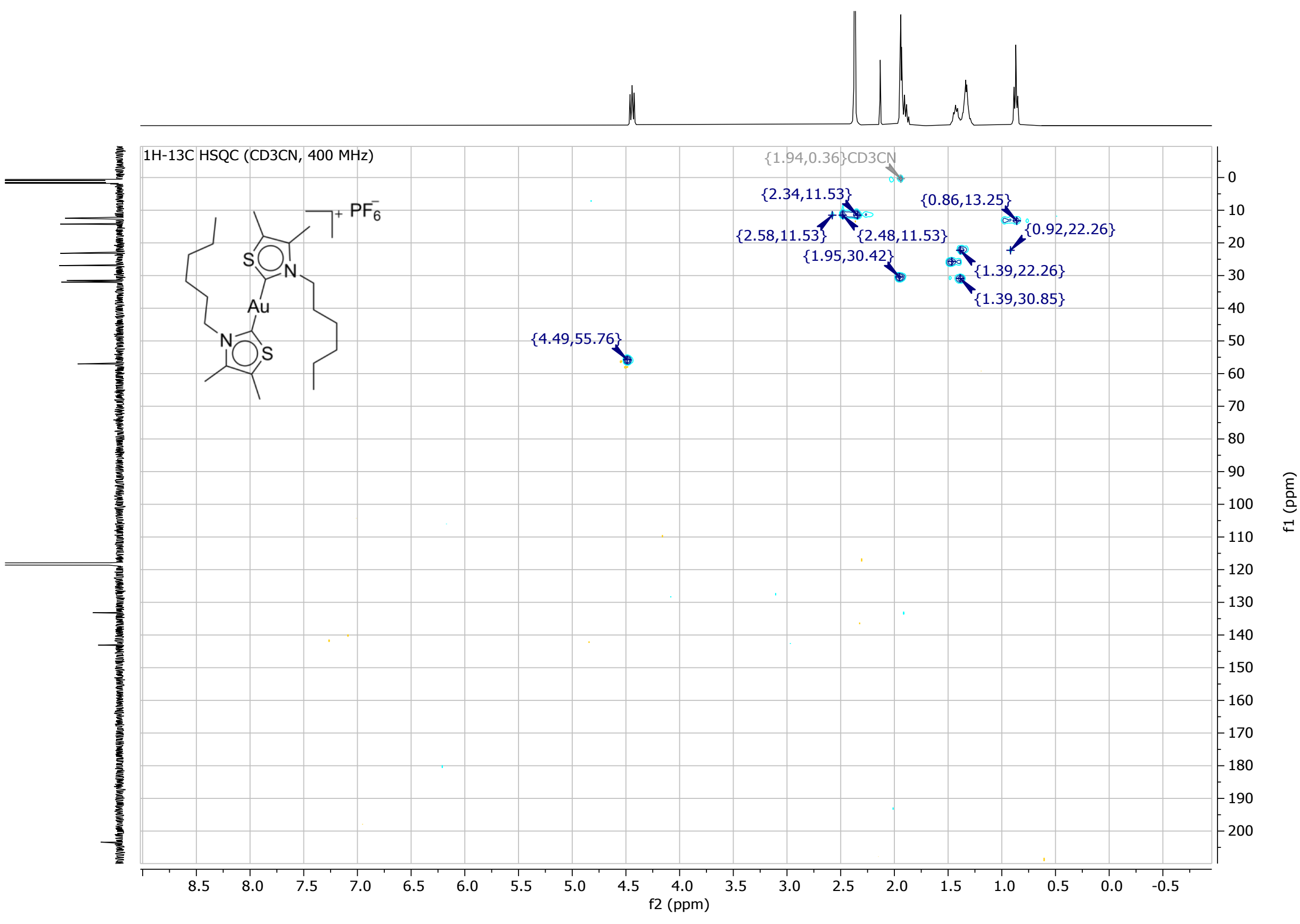

Figure S4: ^{1}H-$^{13}C\{^{1}H\}$--HSQC-NMR of complex 3 solved in acetonitrile-d_3.

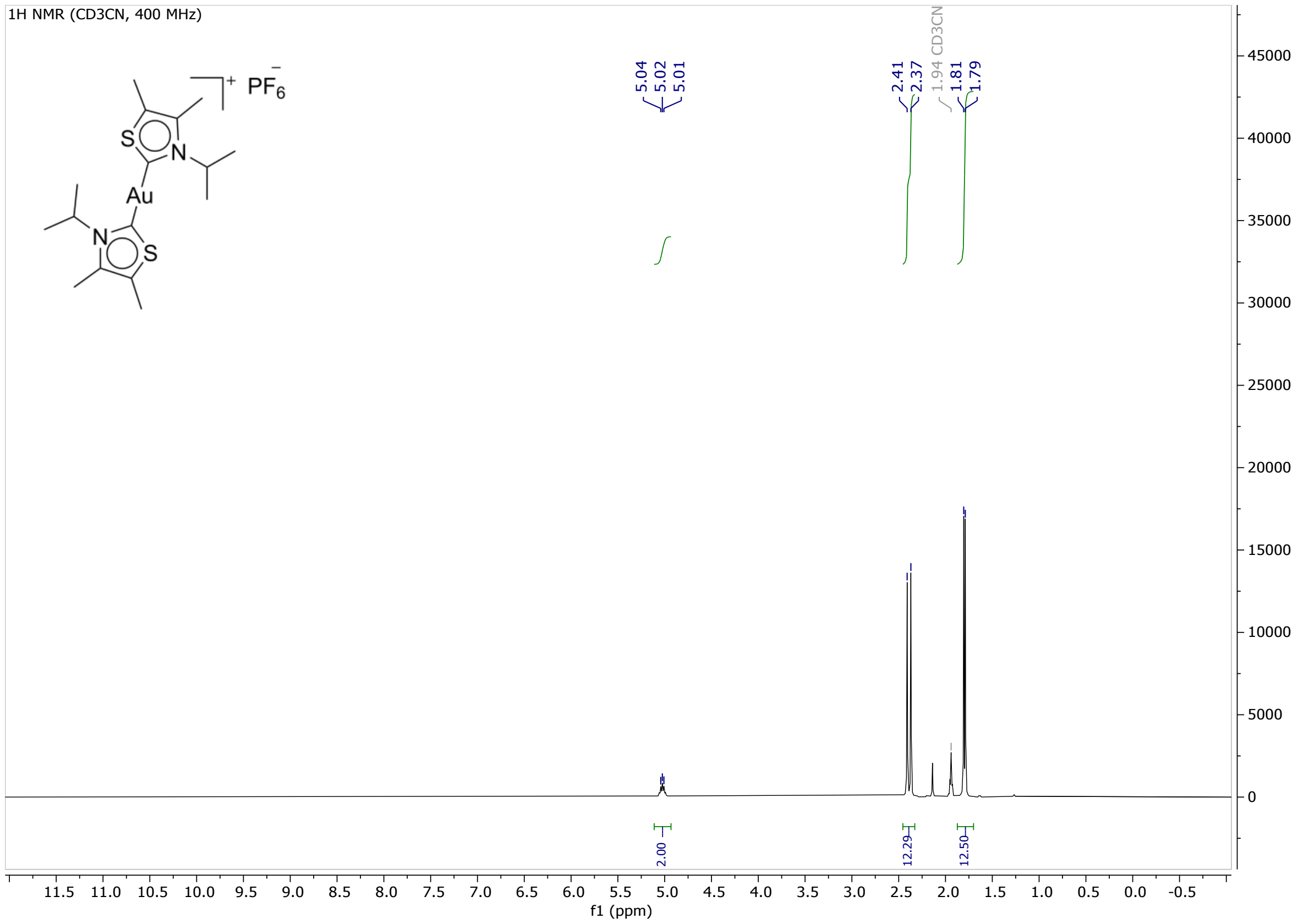

Figure S5: ^{1}H-NMR of complex 2 solved in acetonitrile-d_3.

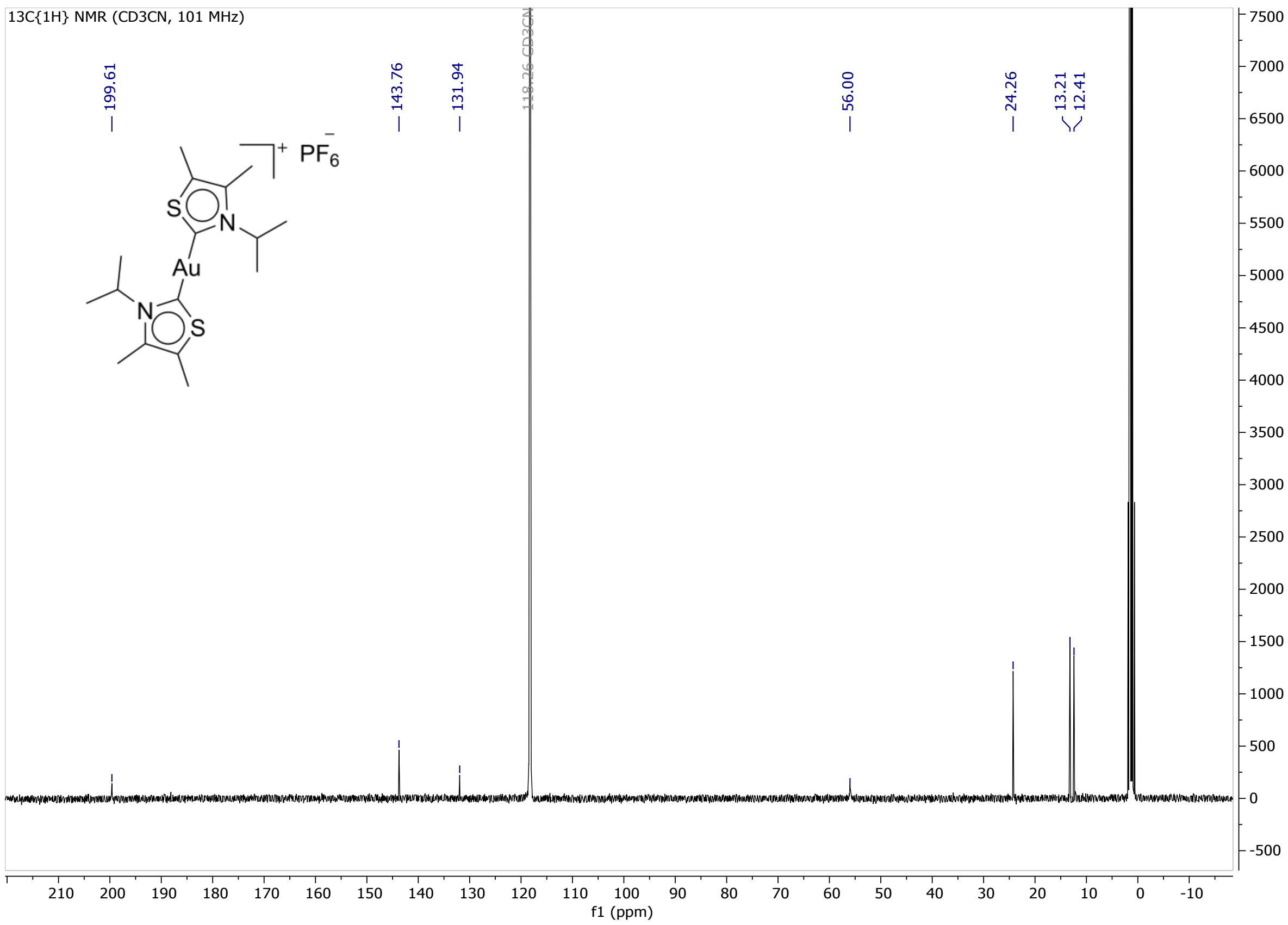

Figure S6: ^{13}C{^{1}H}--NMR of complex 2 solved in acetonitrile-d_3.

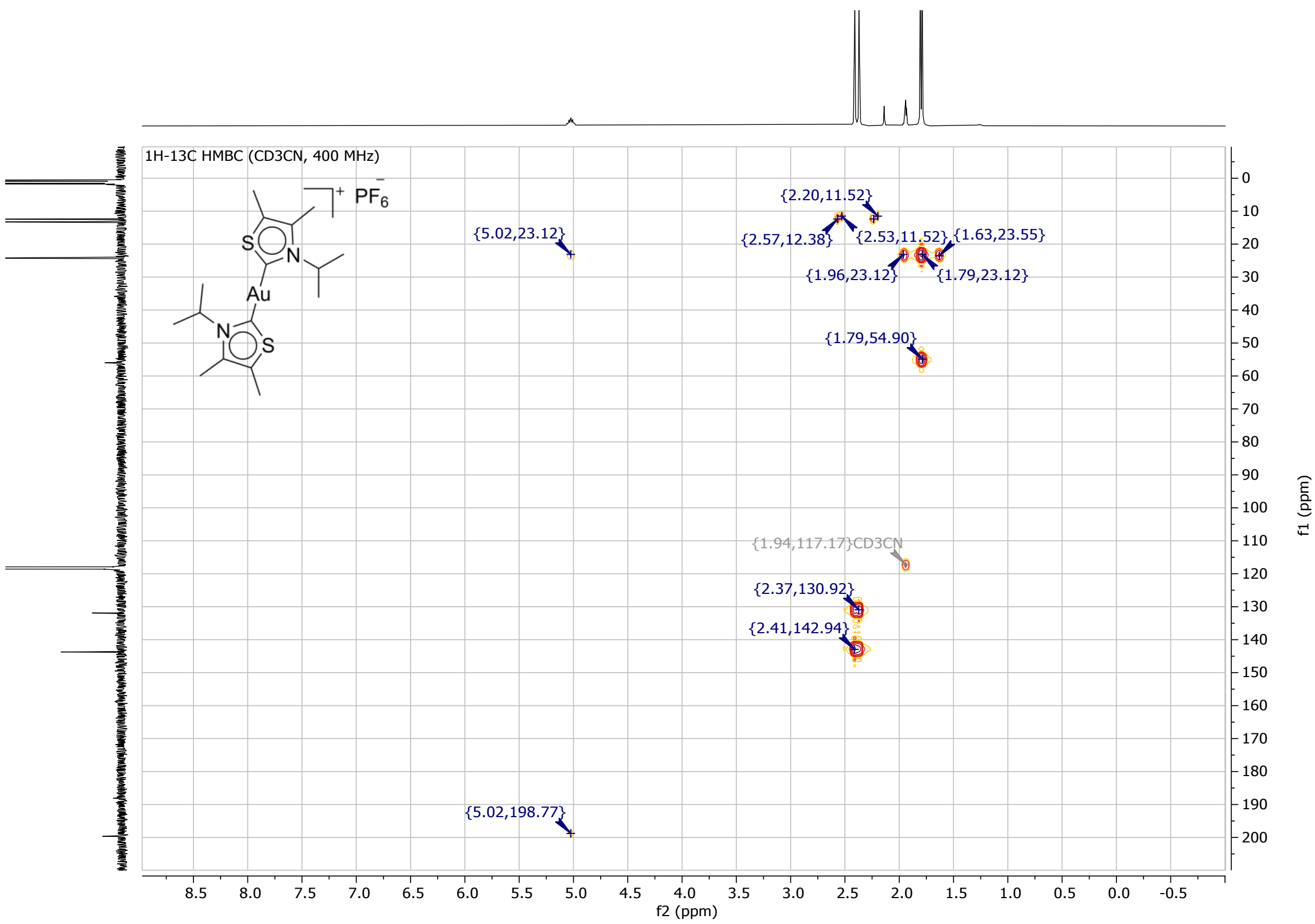

Figure S7: ^{1}H-^{13}C--HMBC-NMR of complex 2 solved in acetonitrile-d_3.

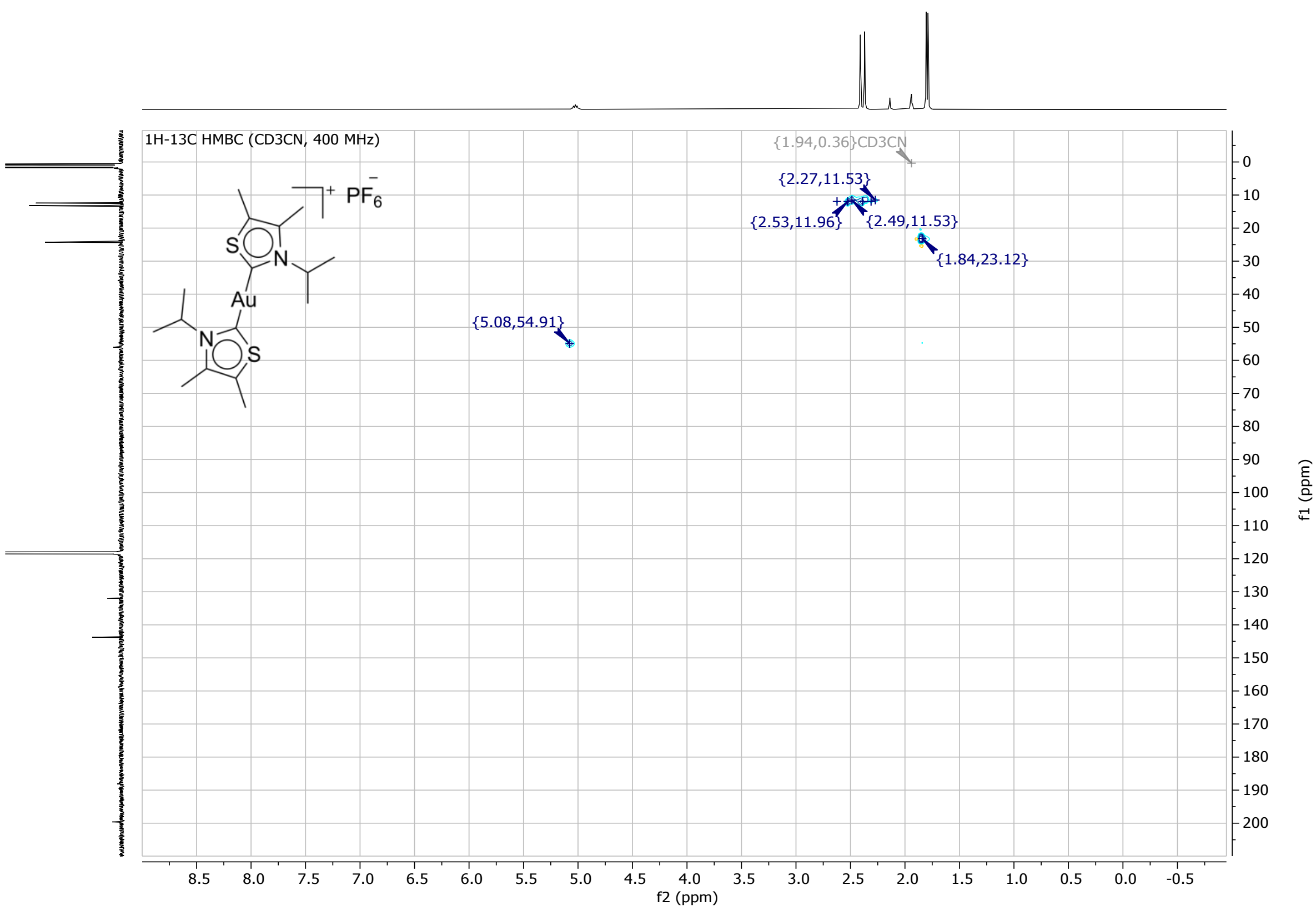

Figure S8: ^{1}H-^{13}C--HSQC-NMR of complex 2 solved in acetonitrile-d_3.

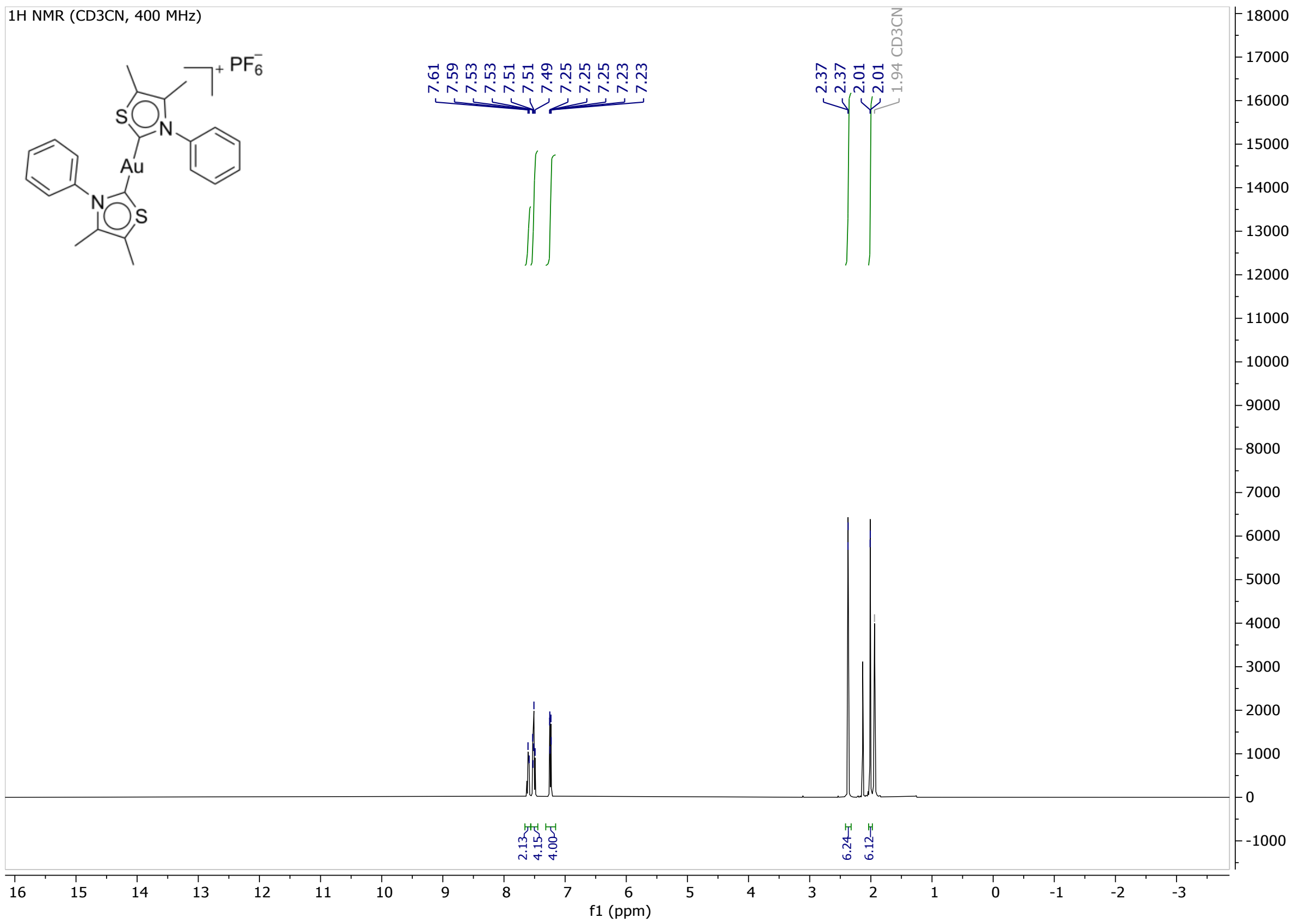

Figure S9: ^{1}H-NMR of complex 4 solved in acetonitrile-d_3.

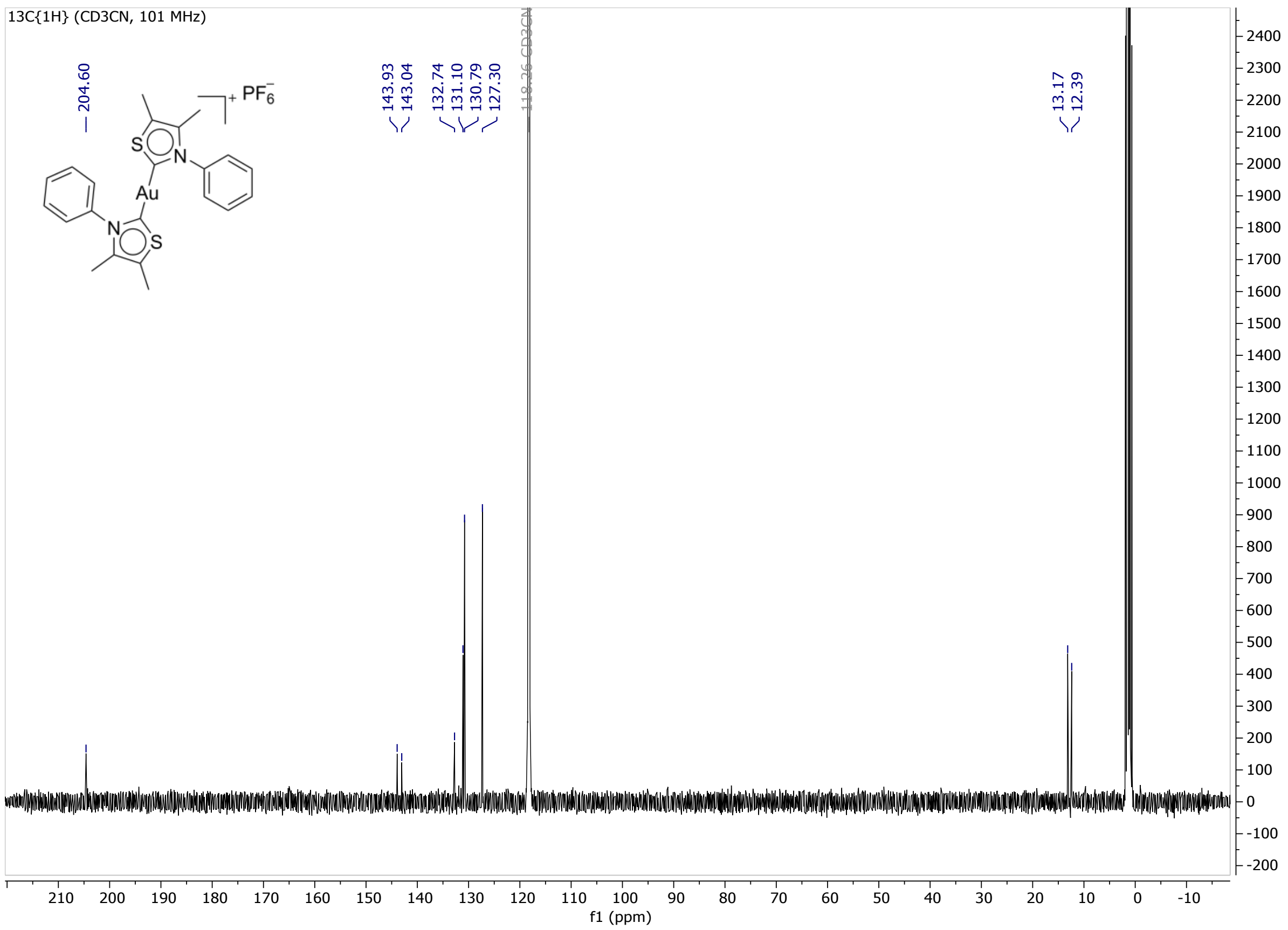

Figure S10: $^{13}C\{^{1}H\}$--NMR of complex 4 solved in acetonitrile-d_3.

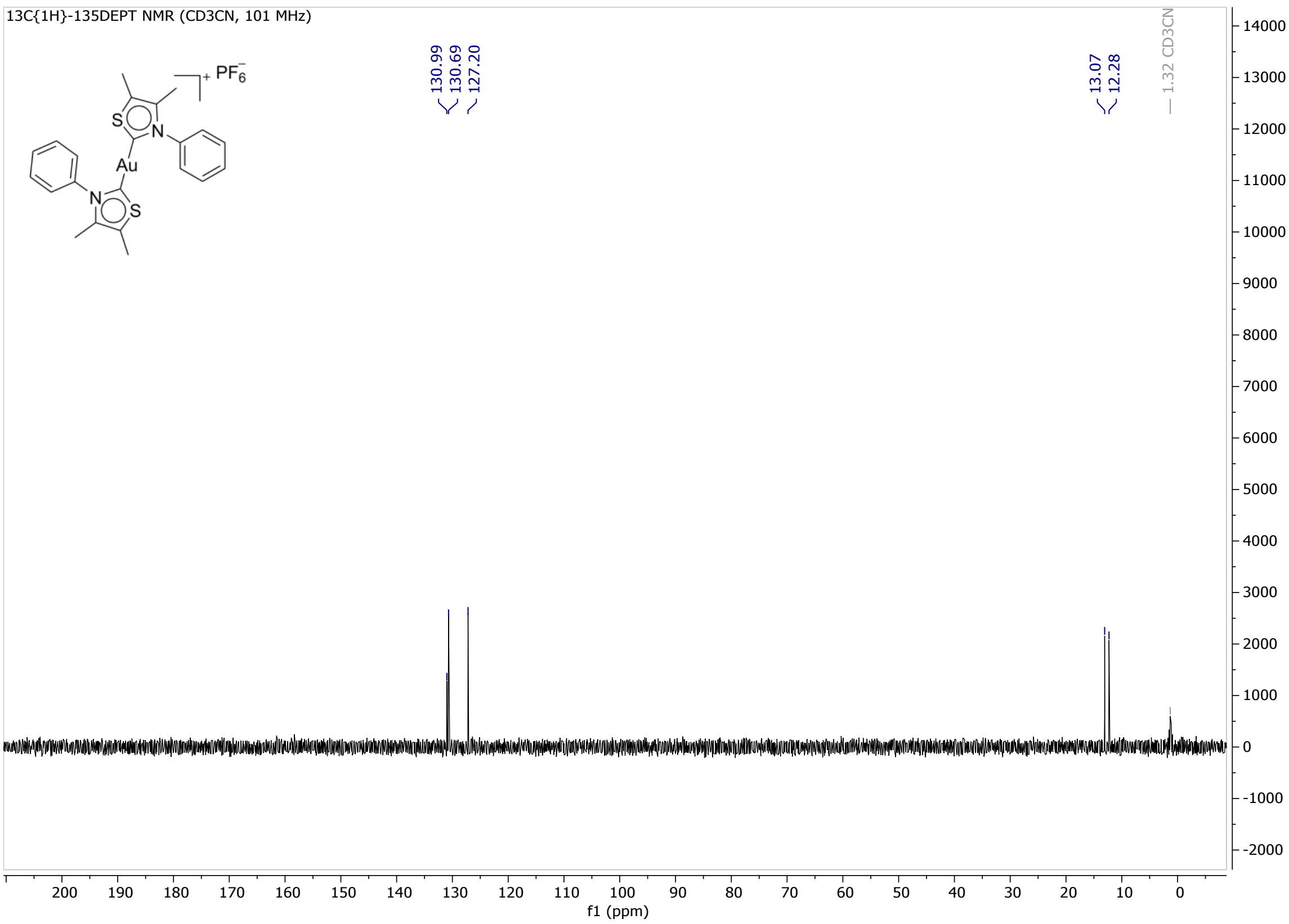

Figure S11: [135]DEPT-NMR of complex 4 solved in acetonitrile-d_3.

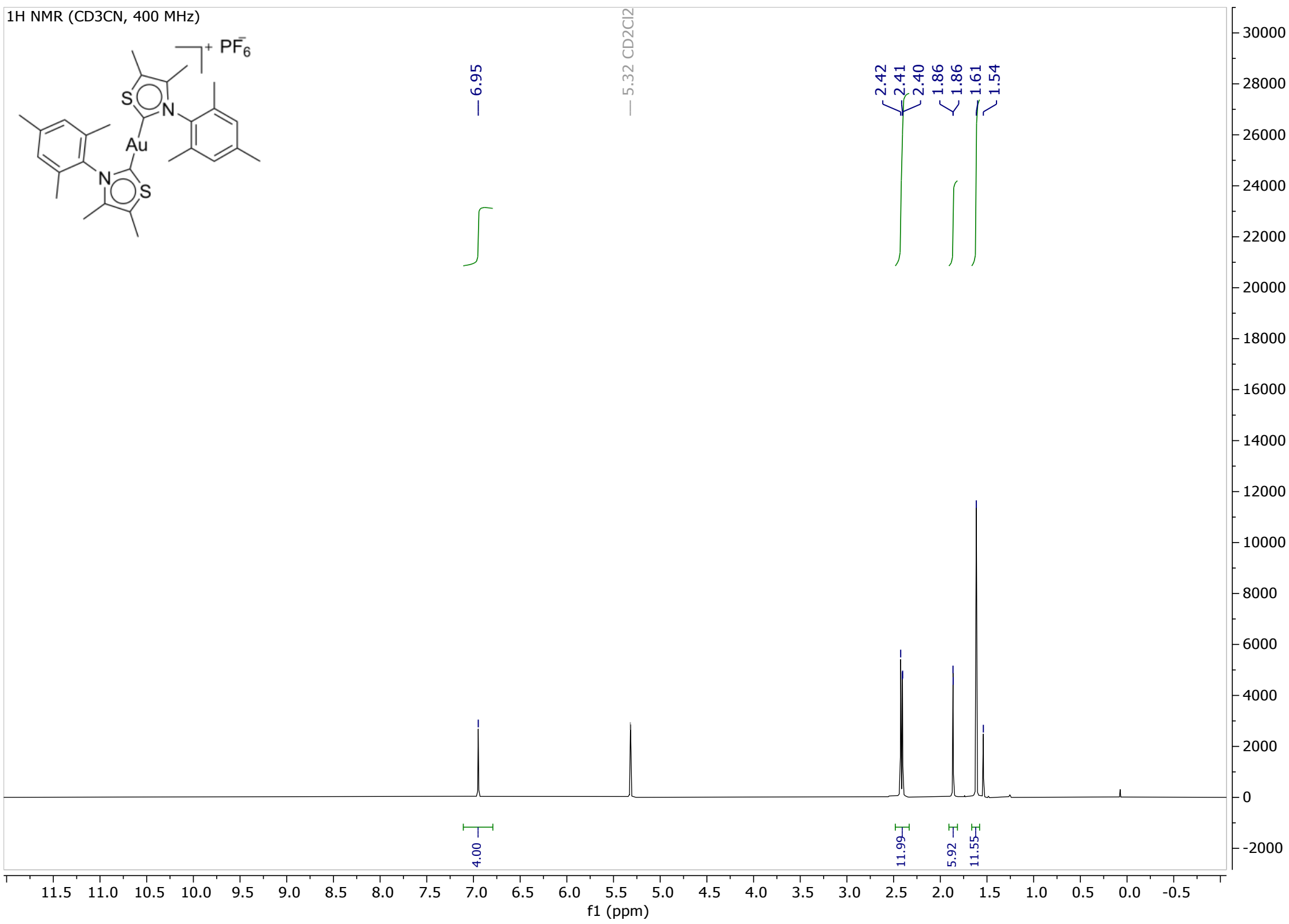

Figure S12: ^{1}H-NMR of complex 1 solved in dichlormethane-d_2.

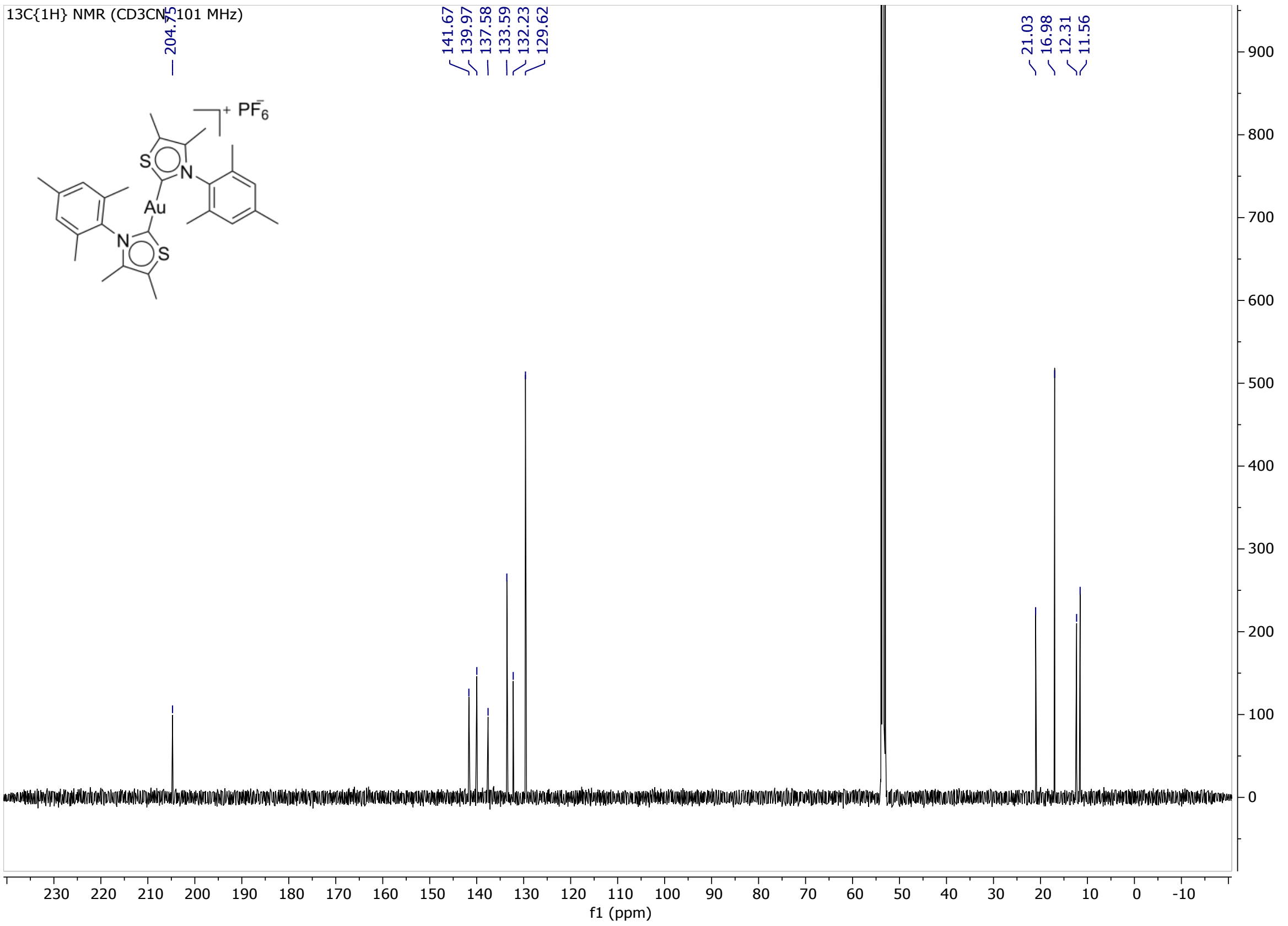

Figure S13: $^{13}C\{^{1}H\}$--NMR of complex 1 solved in dichlormethane-d_2.

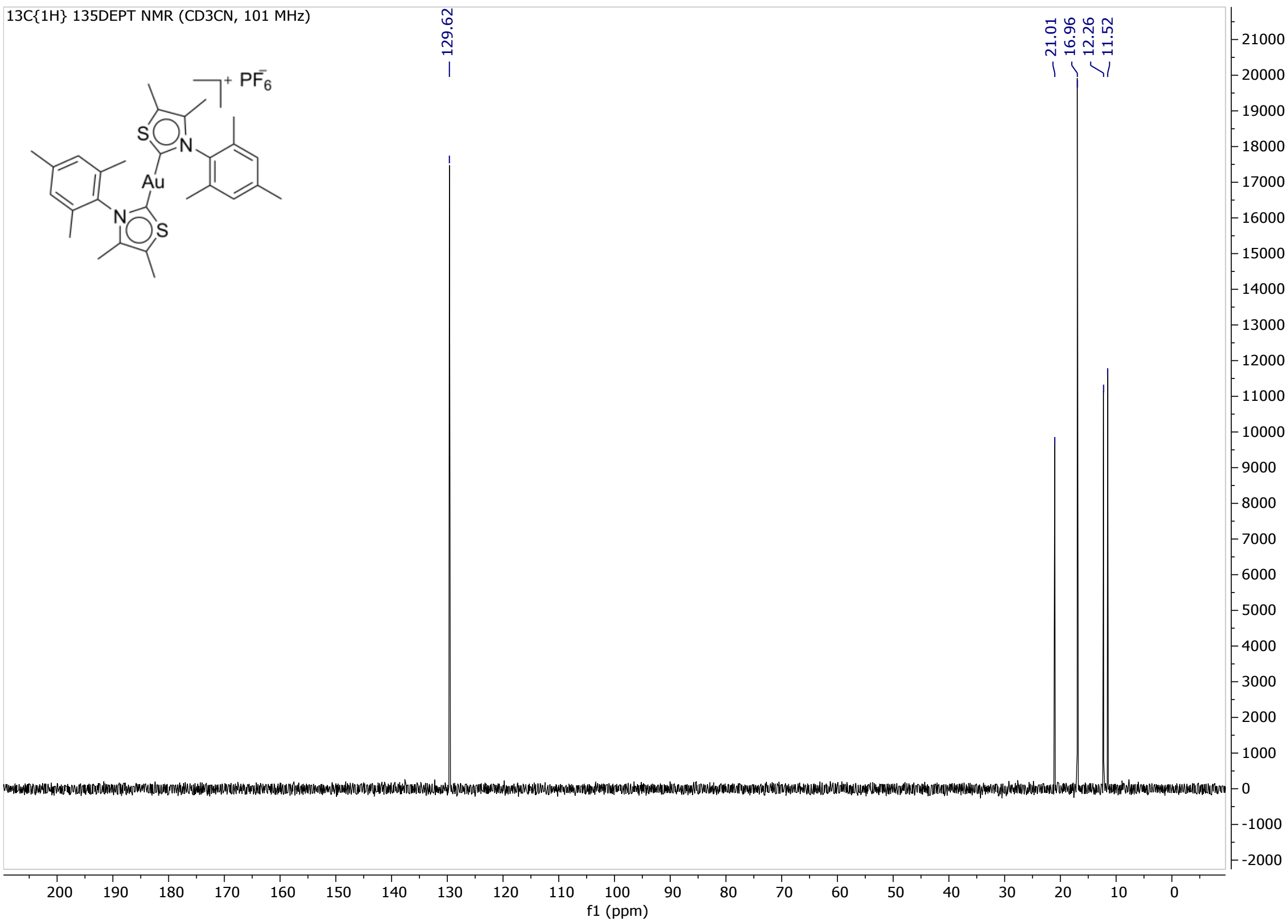

Figure S14: ^{135}DEPT-NMR of complex 1 solved in dichlormethane-d_2.

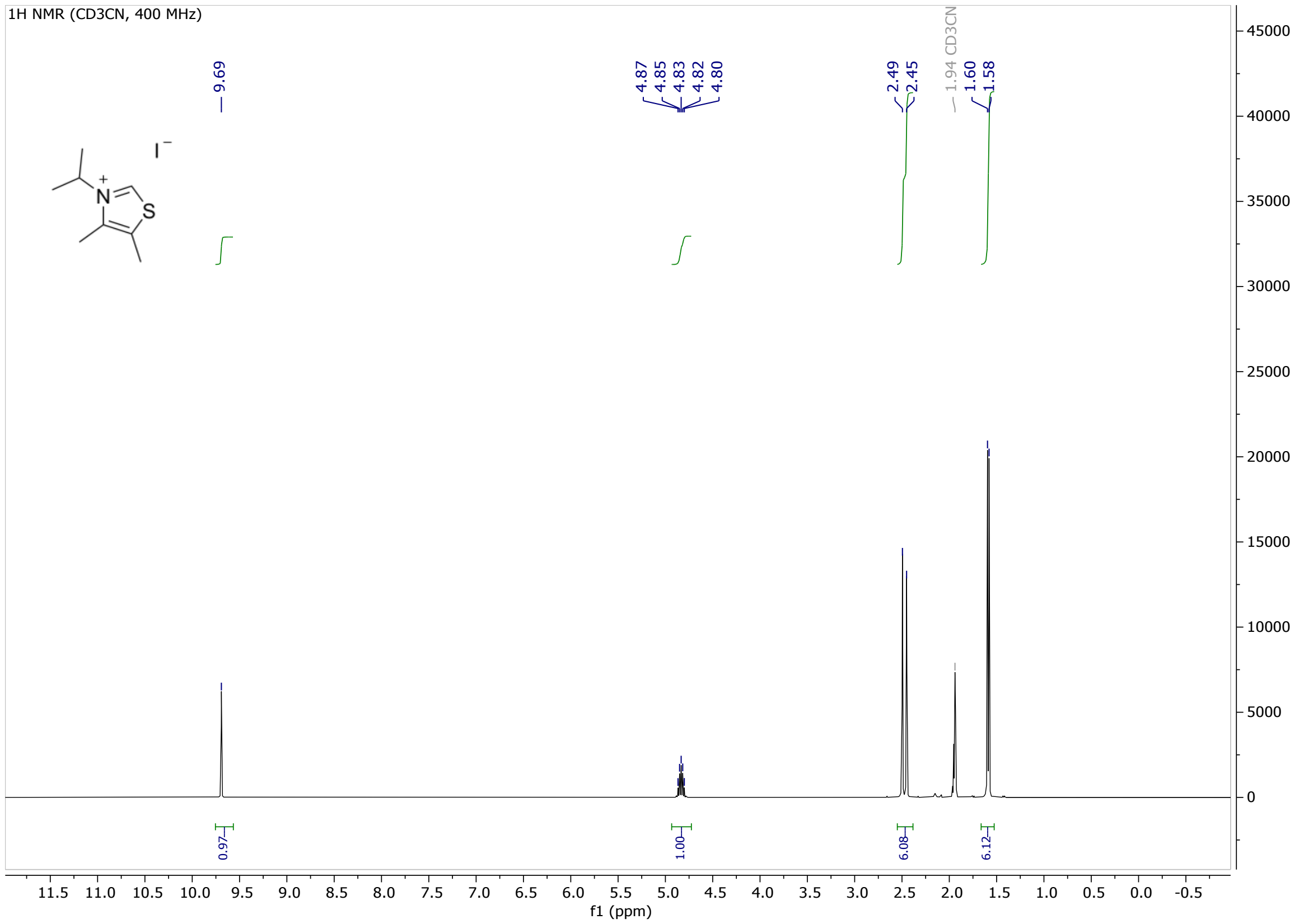

Figure S15: ^{1}H-NMR of ligand L2a solved in acetonitrile-*d*$_3$.

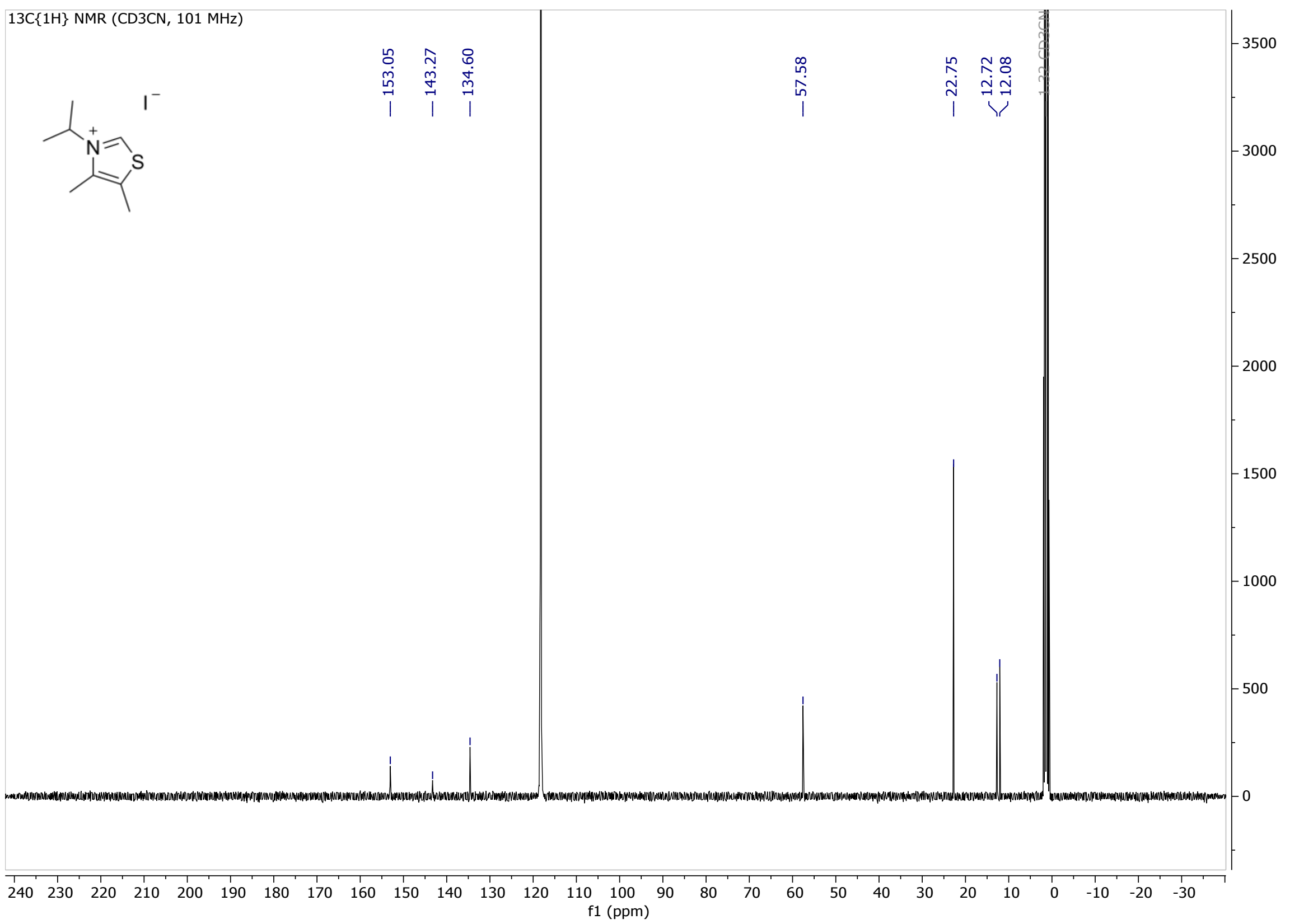

Figure S16: ^{13}C{^{1}H}--NMR of ligand L2a solved in acetonitrile-*d*$_3$.

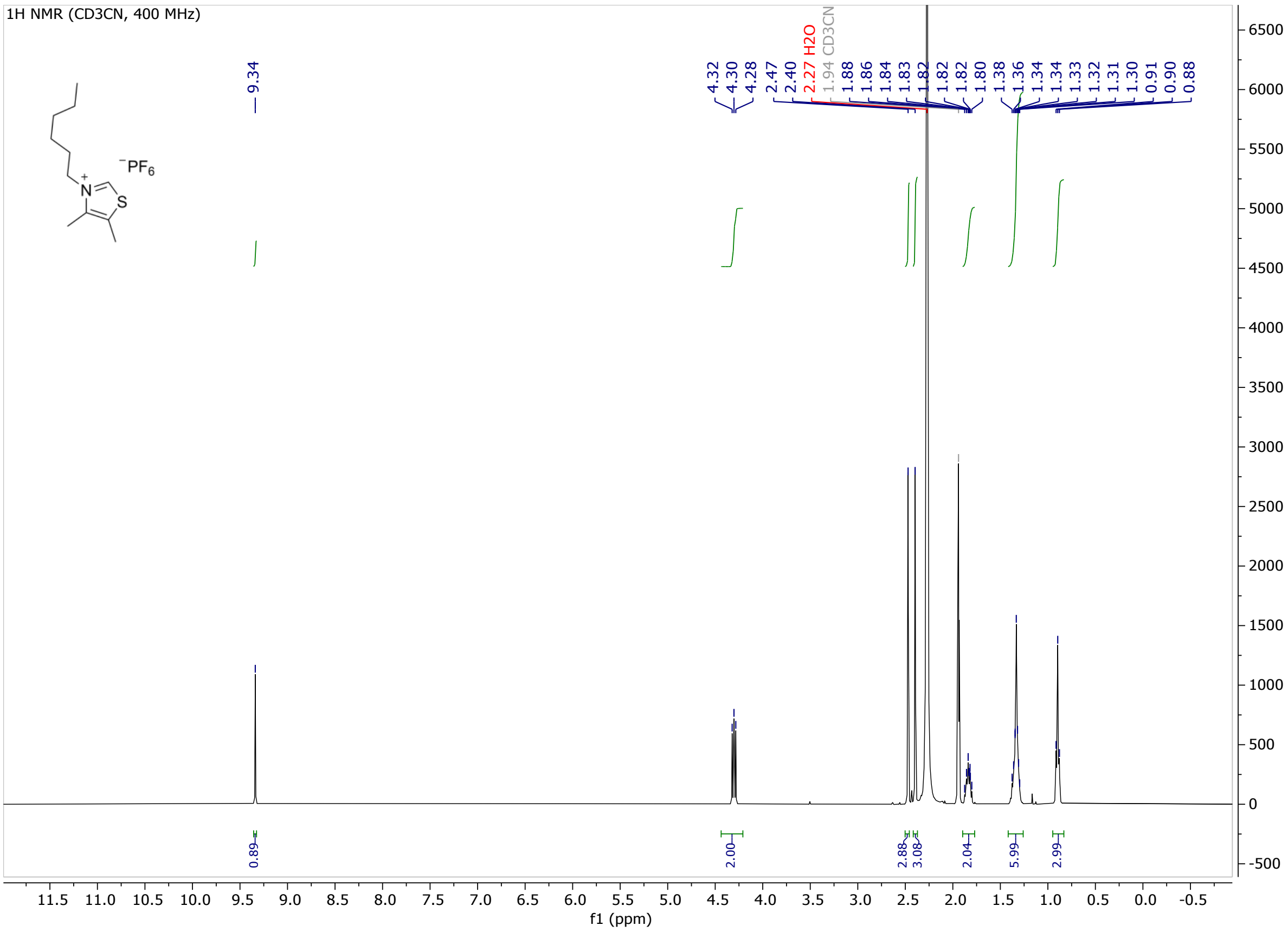

Figure S17: ^{1}H-NMR of ligand L3 solved in acetonitrile-*d*$_3$.

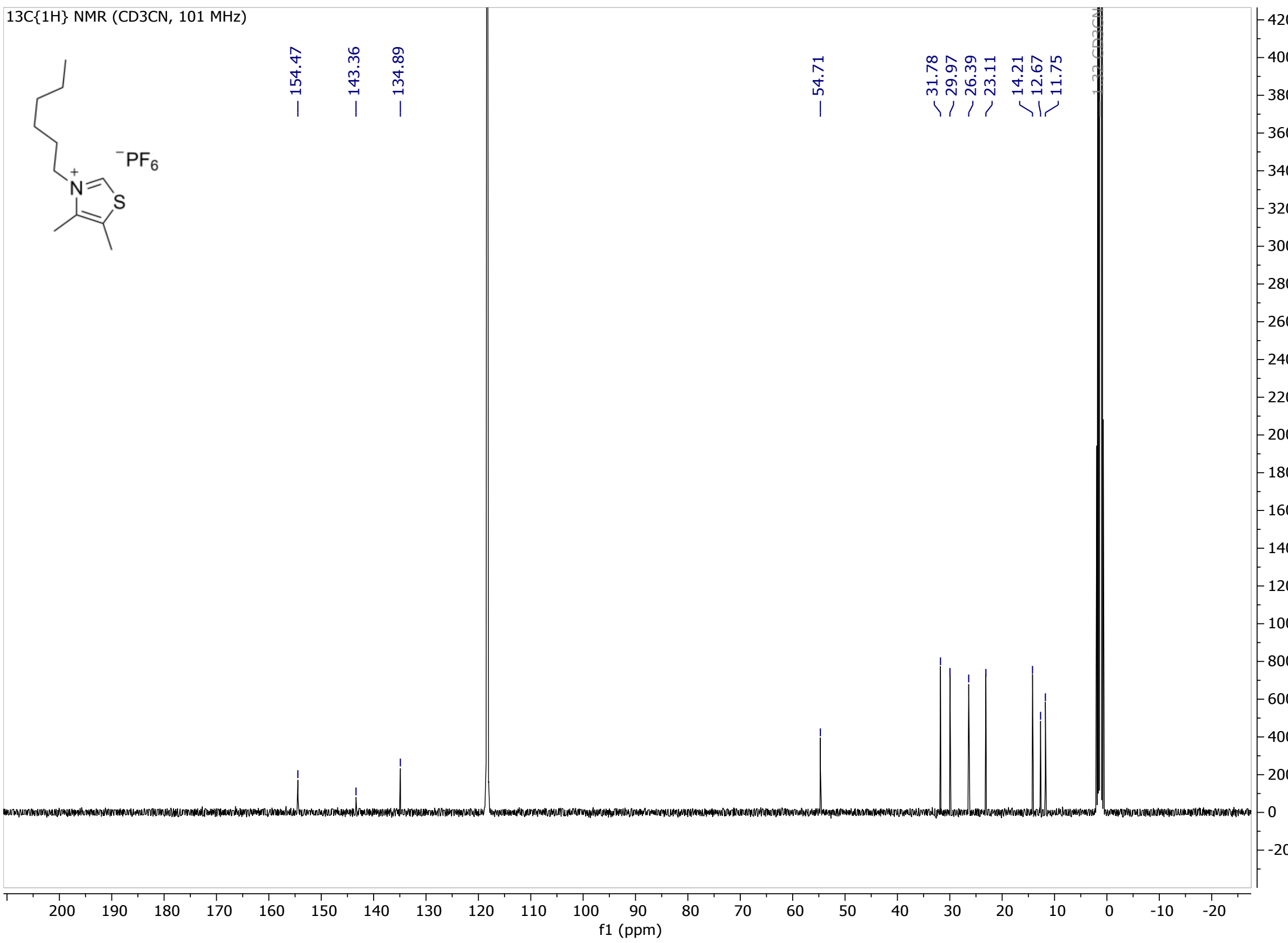

Figure S18: ^{13}C{^{1}H}--NMR of ligand L3 solved in acetonitrile-*d*$_3$.

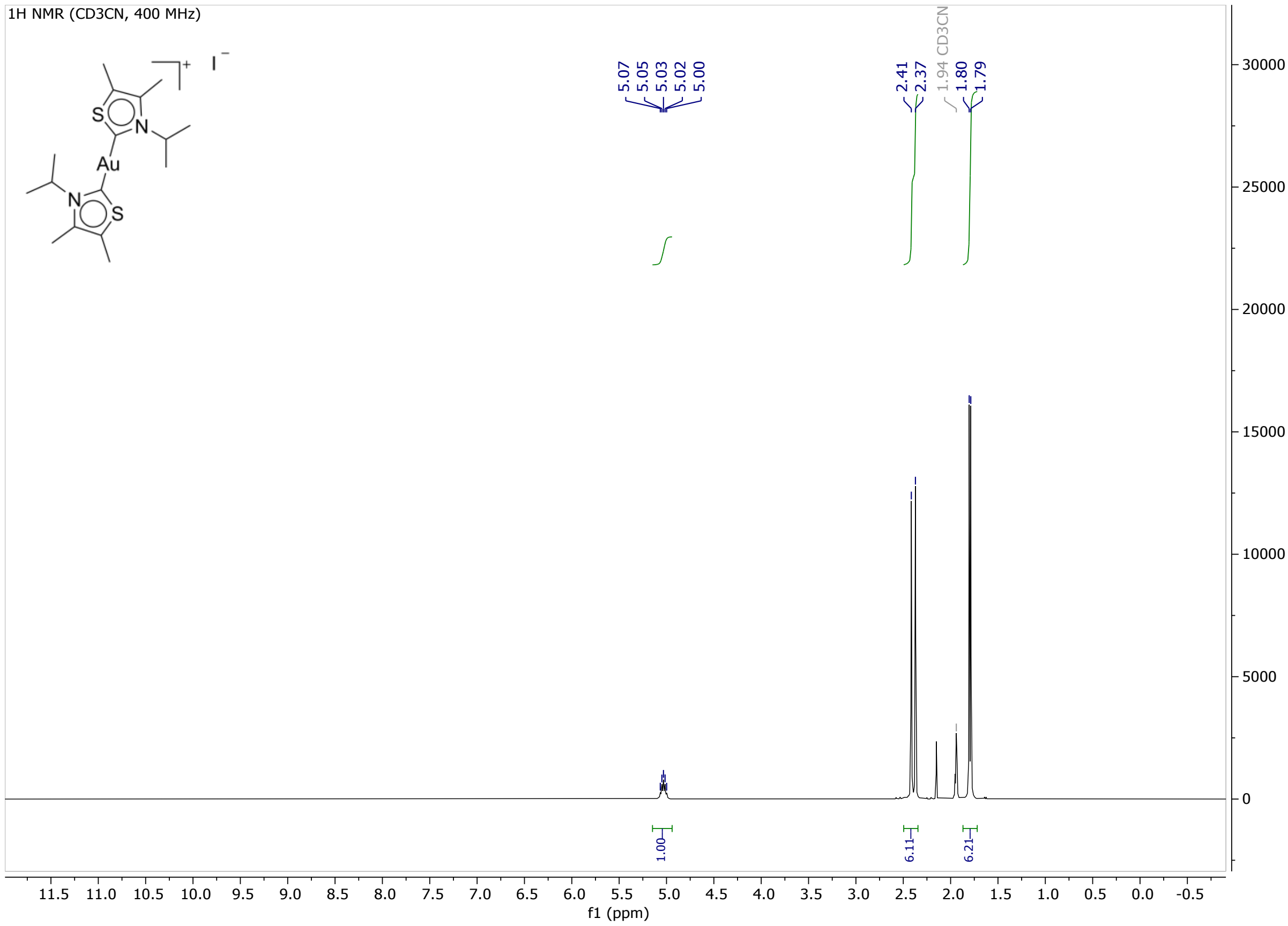

Figure S19: ^{1}H-NMR of complex 2a solved in acetonitrile-d_3.

ESI-MS Spectra

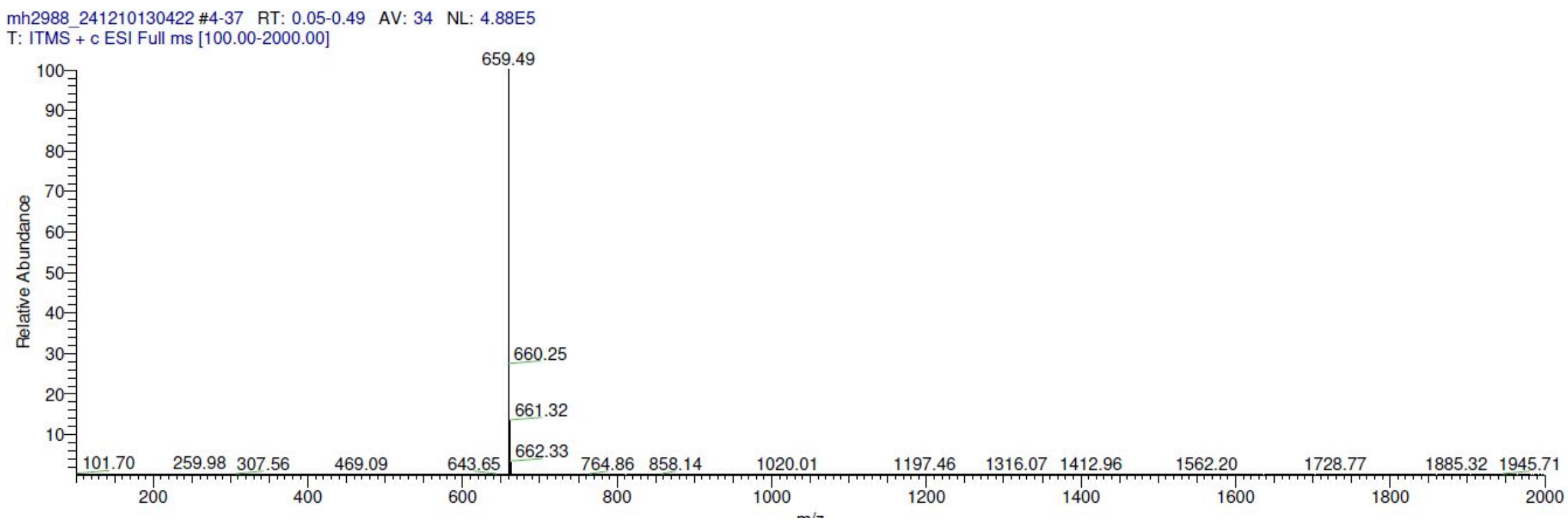

Figure S20: ESI-MS spectrum of complex **1**.

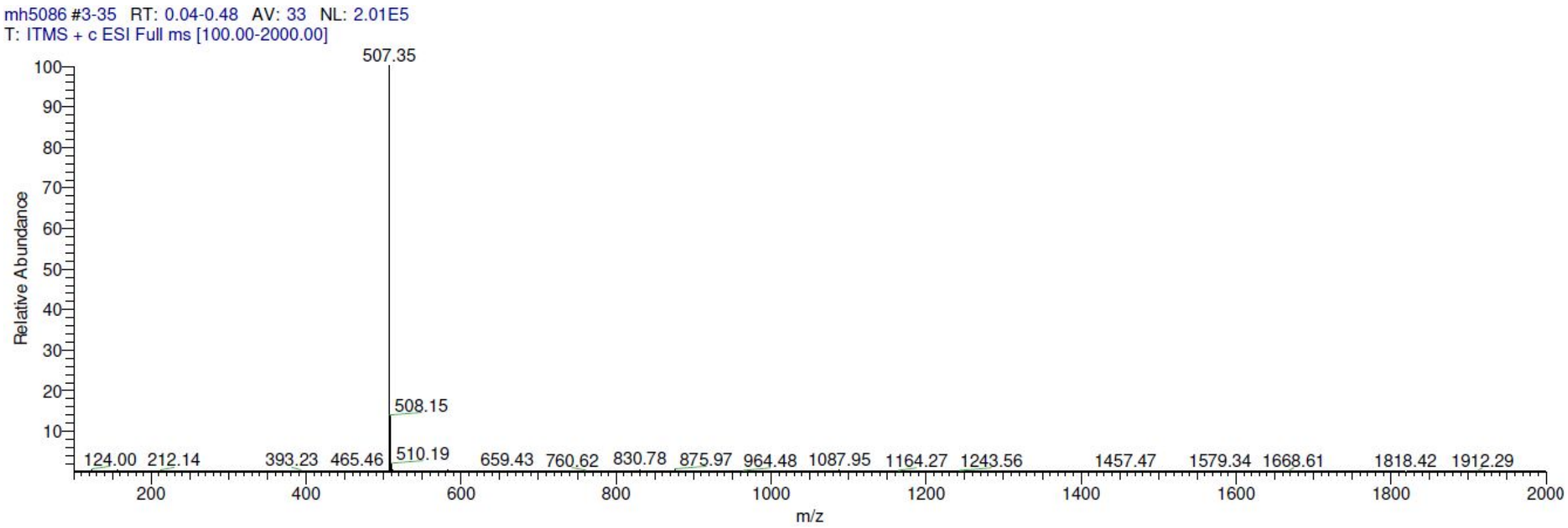

Figure S21: ESI-MS spectrum of complex **2**.

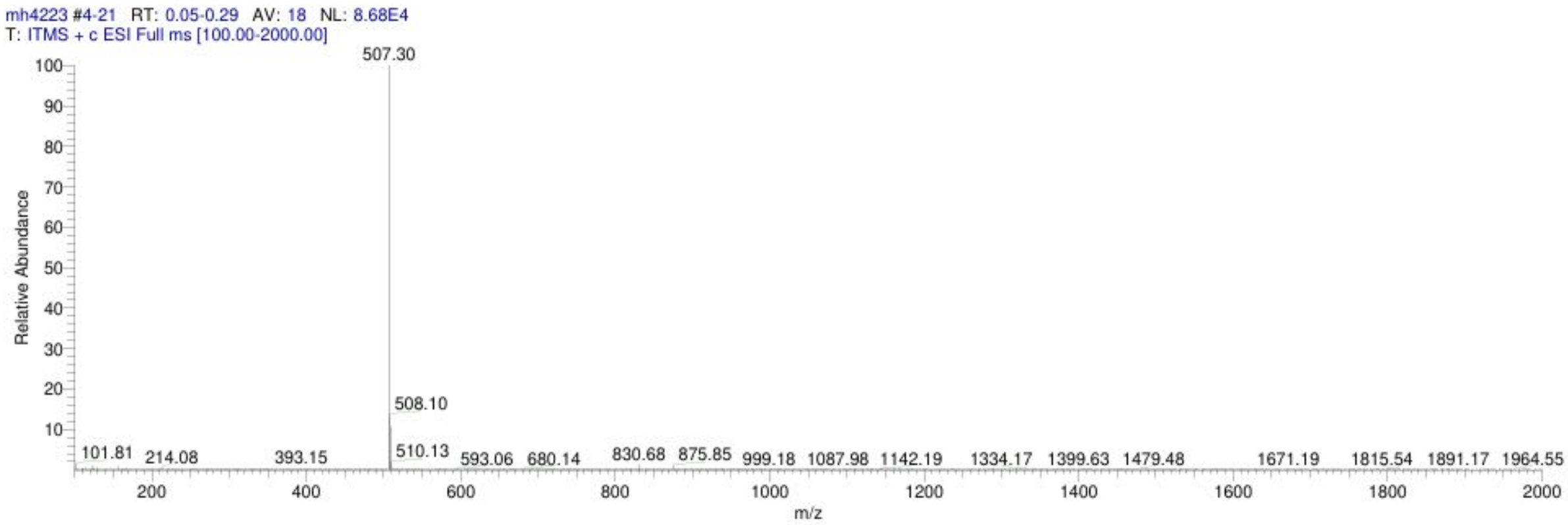

Figure S22: ESI-MS spectrum of complex **2a**.

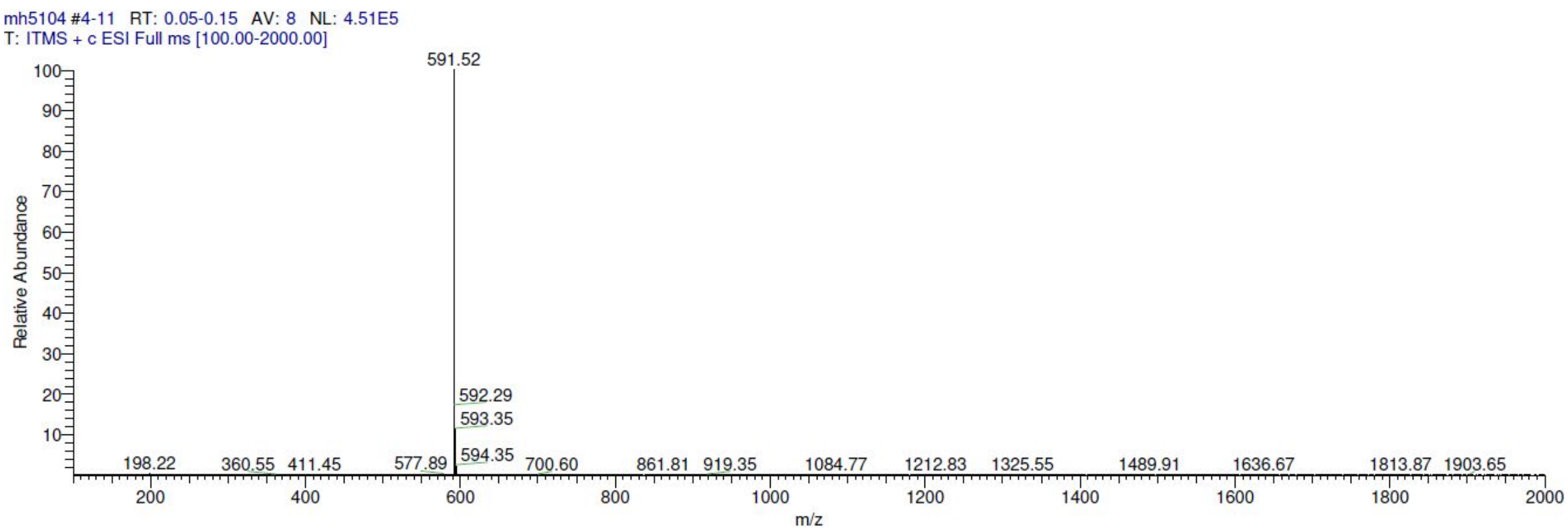

Figure S23: ESI-MS spectrum of complex **3.**

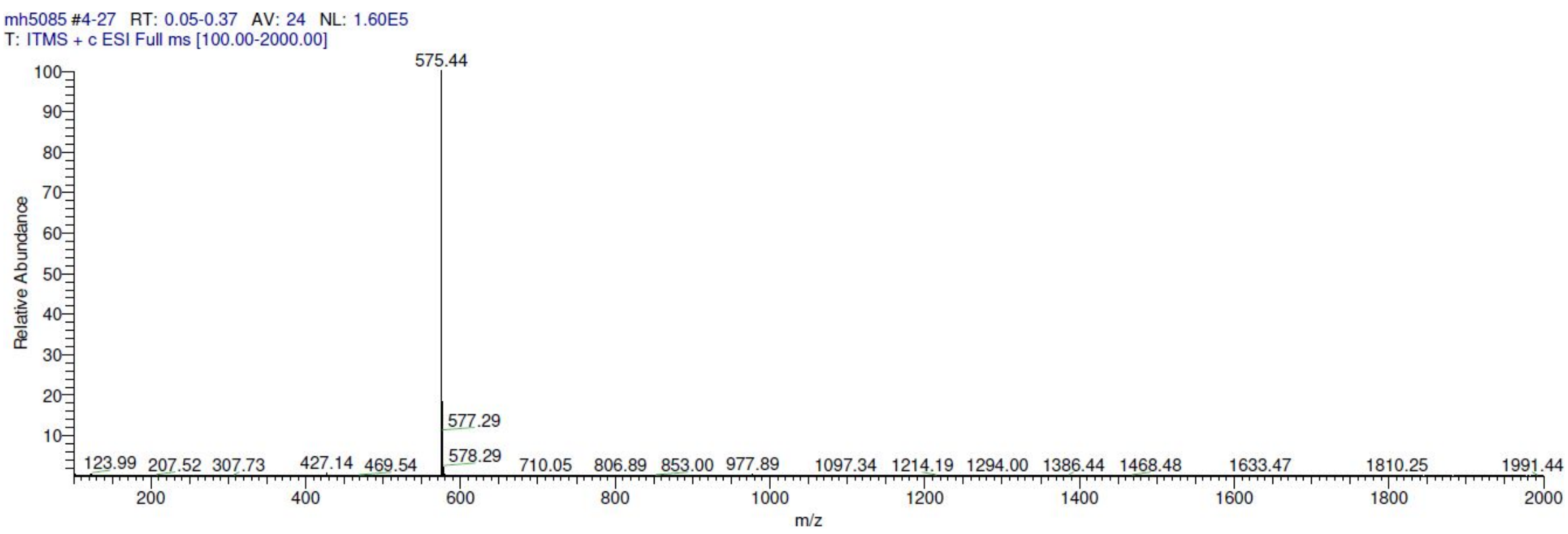

Figure S24: ESI-MS spectrum of complex **4**.

HR ESI MS

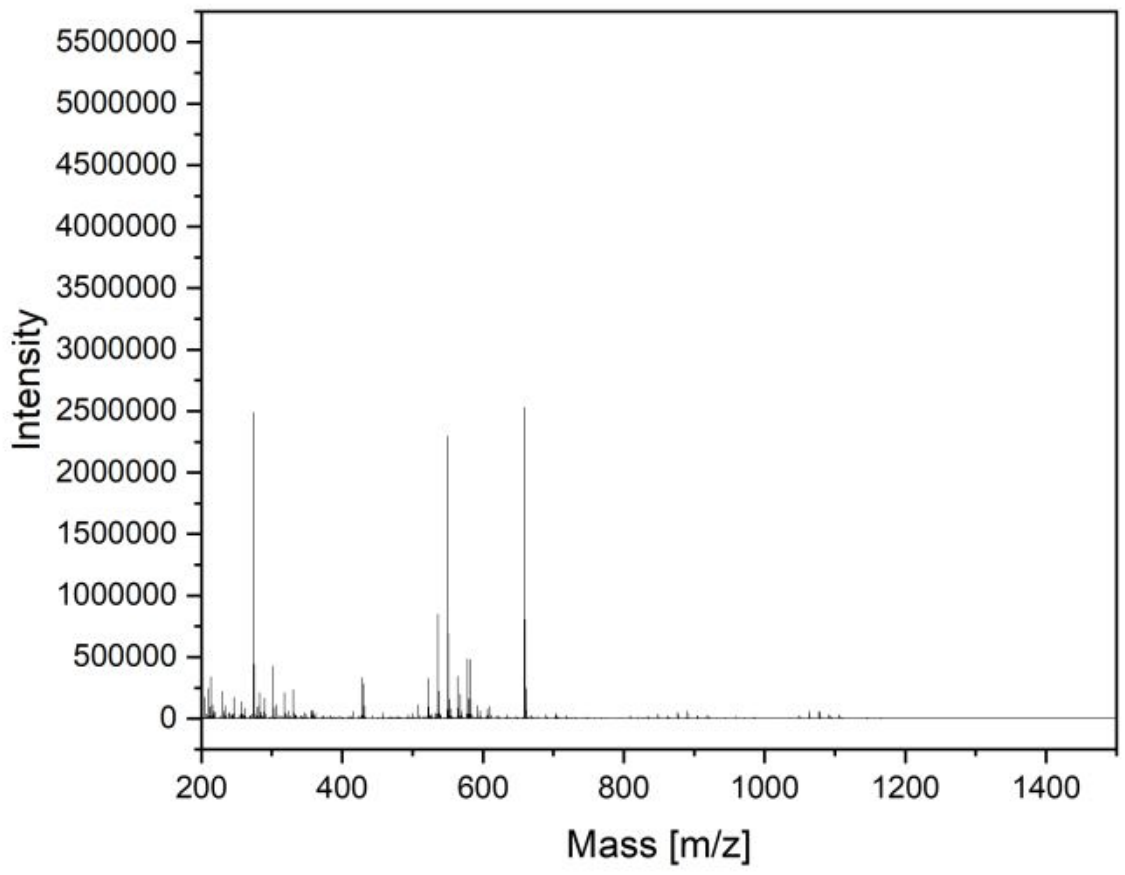

Figure S25: HR ESI-MS spectrum of complex **1**.

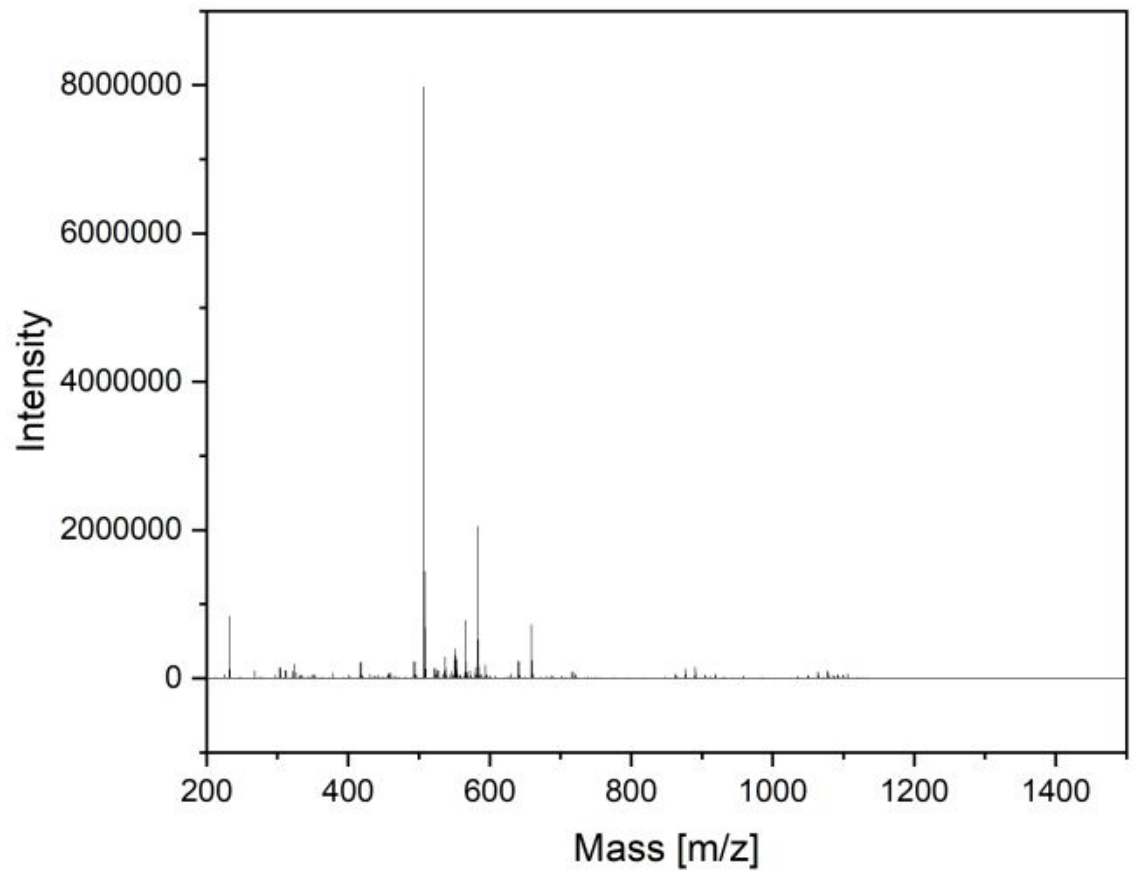

Figure S26: HR ESI-MS spectrum of complex **2**.

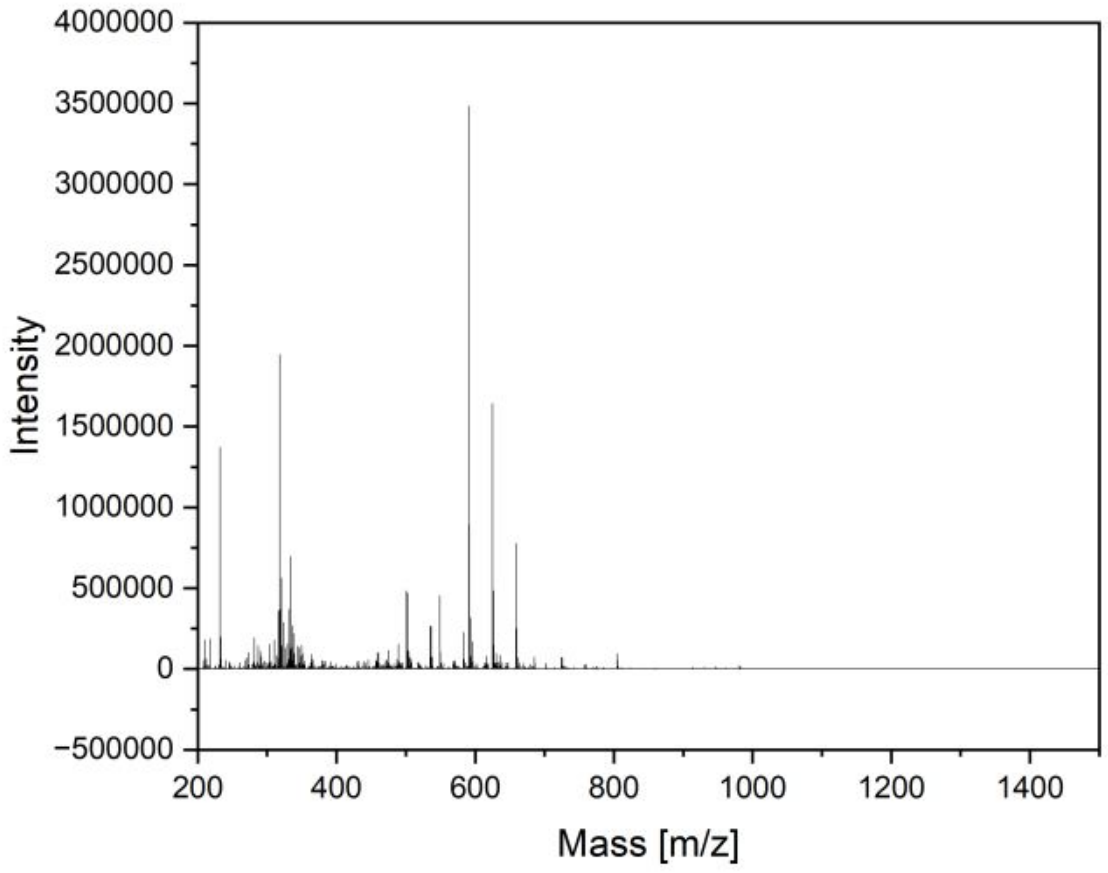

Figure S27: HR ESI-MS spectrum of complex **3**.

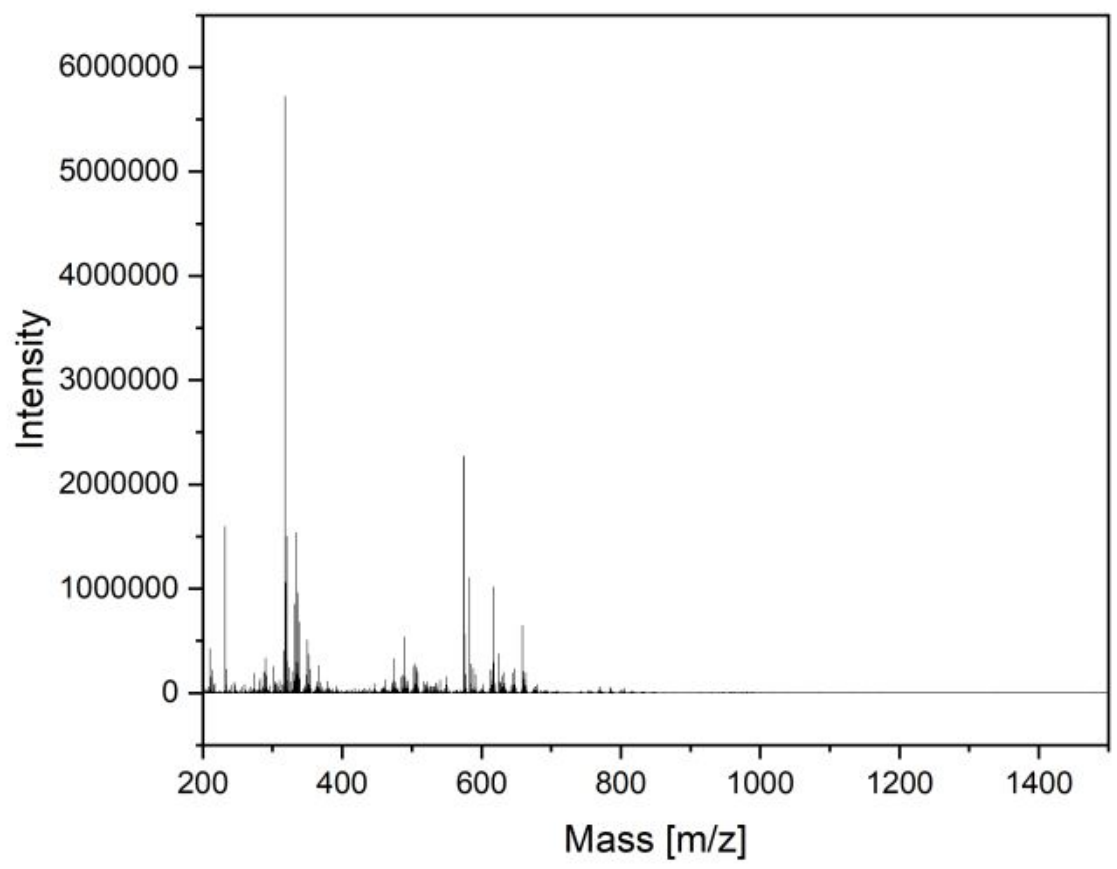

Figure S28: HR ESI-MS spectrum of complex **4**.

Stability Studies

Activity towards L-Glutathione (GSH)

The activity towards L-Glutathione was tested for **1, 2, 3** and **4**. Due to poor solubility in water or PBS. A saturated solution of each compound was prepared by weighing 1 mg of the compound and adding 40 mL of PBS Buffer solution. The PBS Buffer solution was prepared by degassing 1L of H_2O through diffusion of argon through the solution for 1 hour. In the argon-saturated H_2O, a PBS tablet from Merk was added to create a 10 mM phosphate buffer and 3 mM KCl solution. The saturated solution of the complex was stirred for one hour and afterwards filtered. A solution of 1 mg (3.25 µmol) of L-Glutathione in 30 mL PBS buffer solution. 0.5 mL of saturated complex solution and 0.5 ML of L-Glutathione solution were mixed together in a Suprasil cuvette. The mixture was analyzed via UV-VIS spectroscopy. After an initial measurement after direct mixing, the mixture was analyzed every 30 min for 48 hours. The experiment was repeated twice for each complex individually.

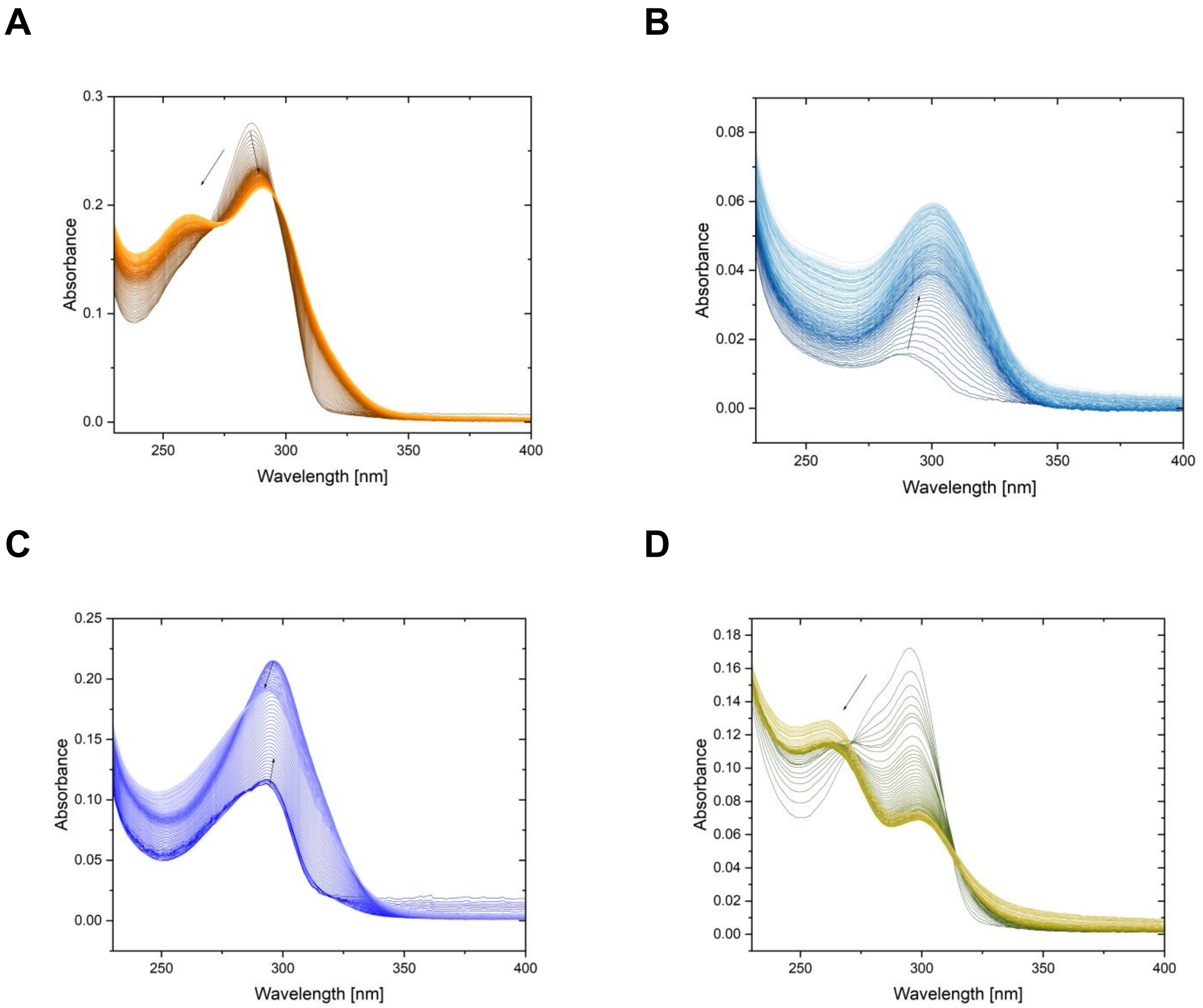

Figure S29: Reaction of complexes 1, 2, 3, and 4 with L-glutathione in a degassed PBS Buffer solution, tracked by UV-VIS spectroscopy. A: 2, B: 3, C: 1, D: 4. The reaction was monitored for 48 h, a spectrum was taken every 30 min. The first spectrum is shown in dark colour, with time the colour changes gradually to a light colour.

Water Stability

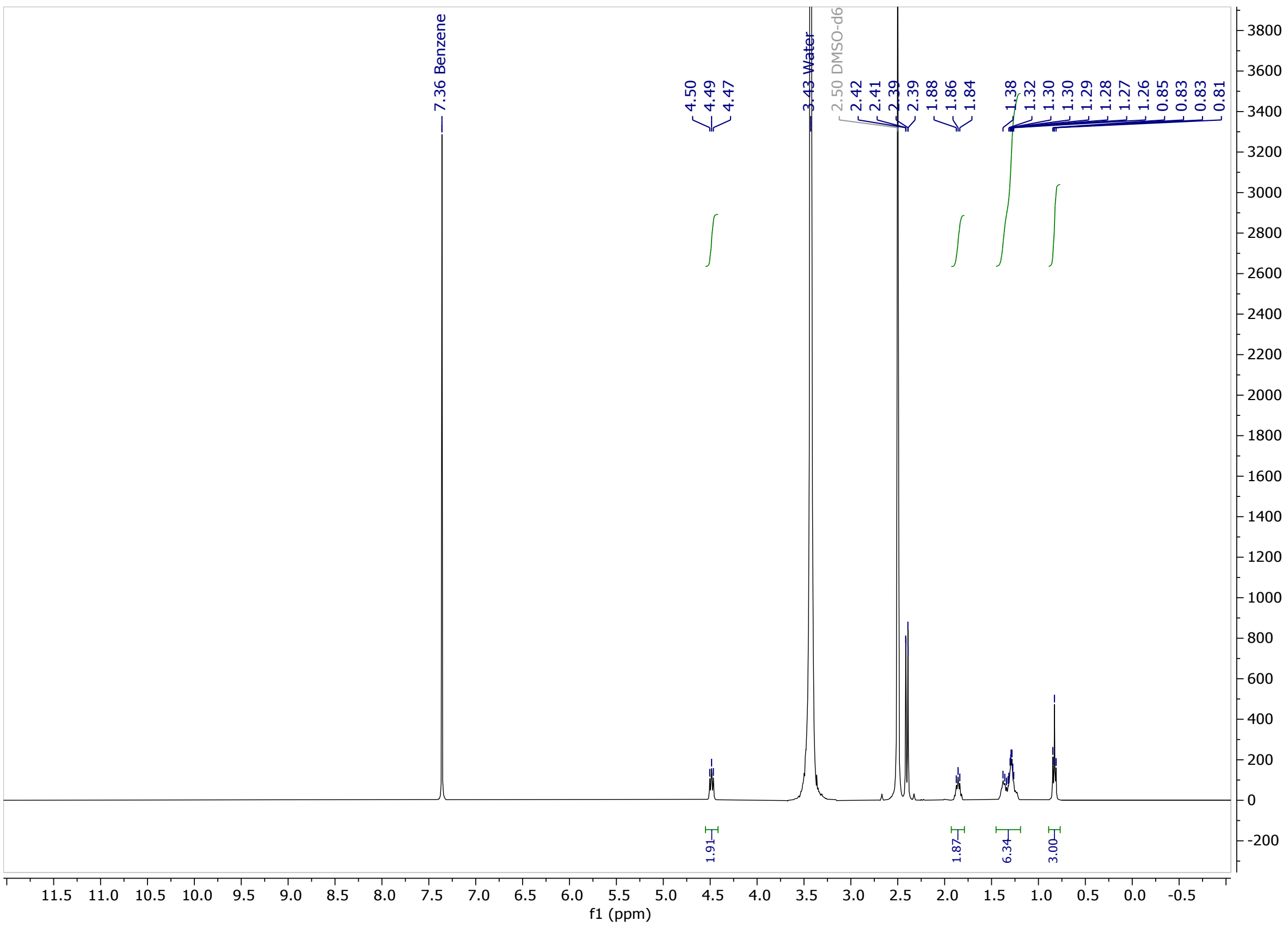

Figure S30: ^{1}H-NMR spectrum of complex **3** after 72 h in water/dmso with benzene as internal standard.

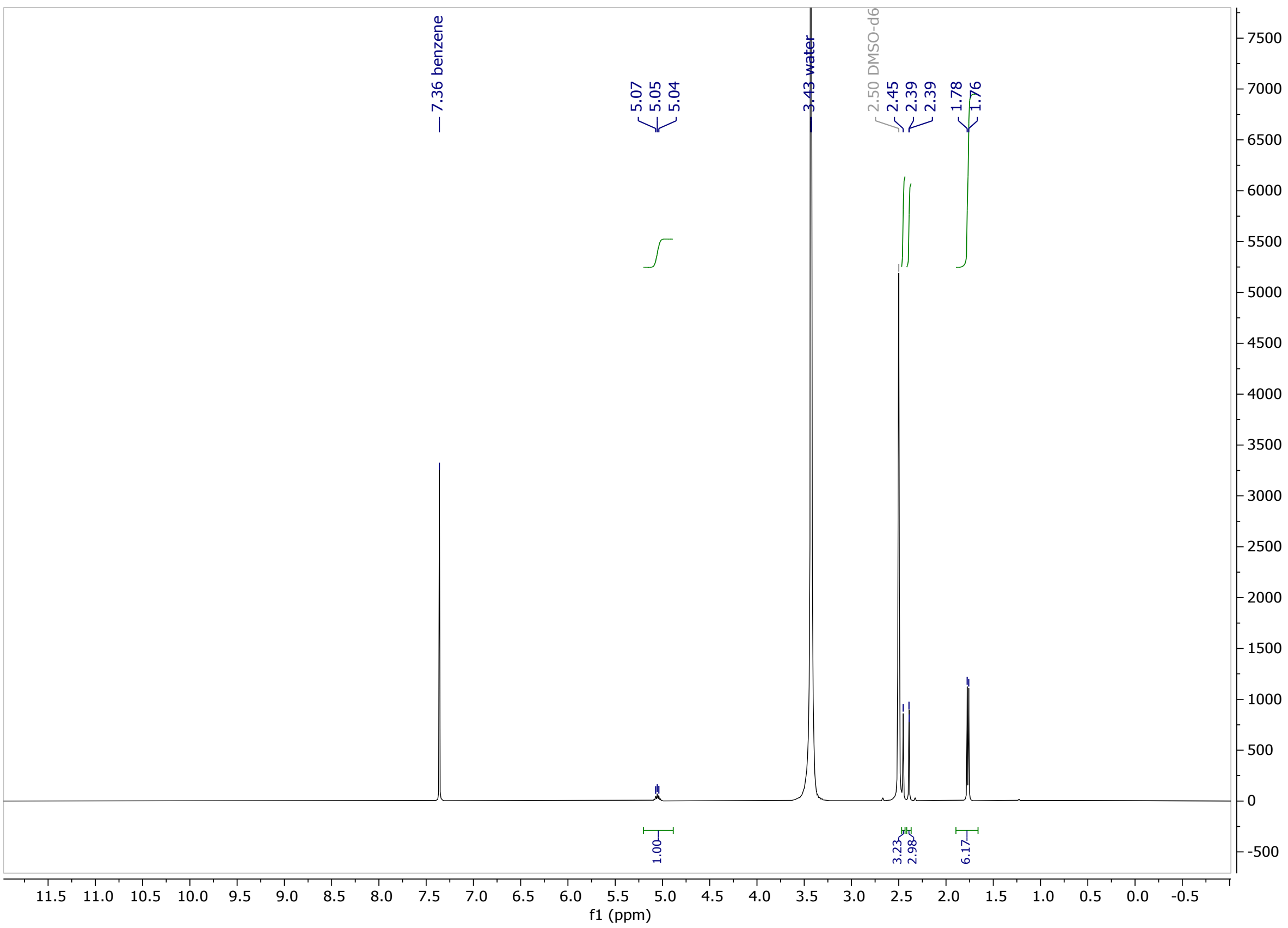

Figure S31: ^{1}H-NMR spectrum of complex **2** after 72 h in water/dmso with benzene as internal standard.

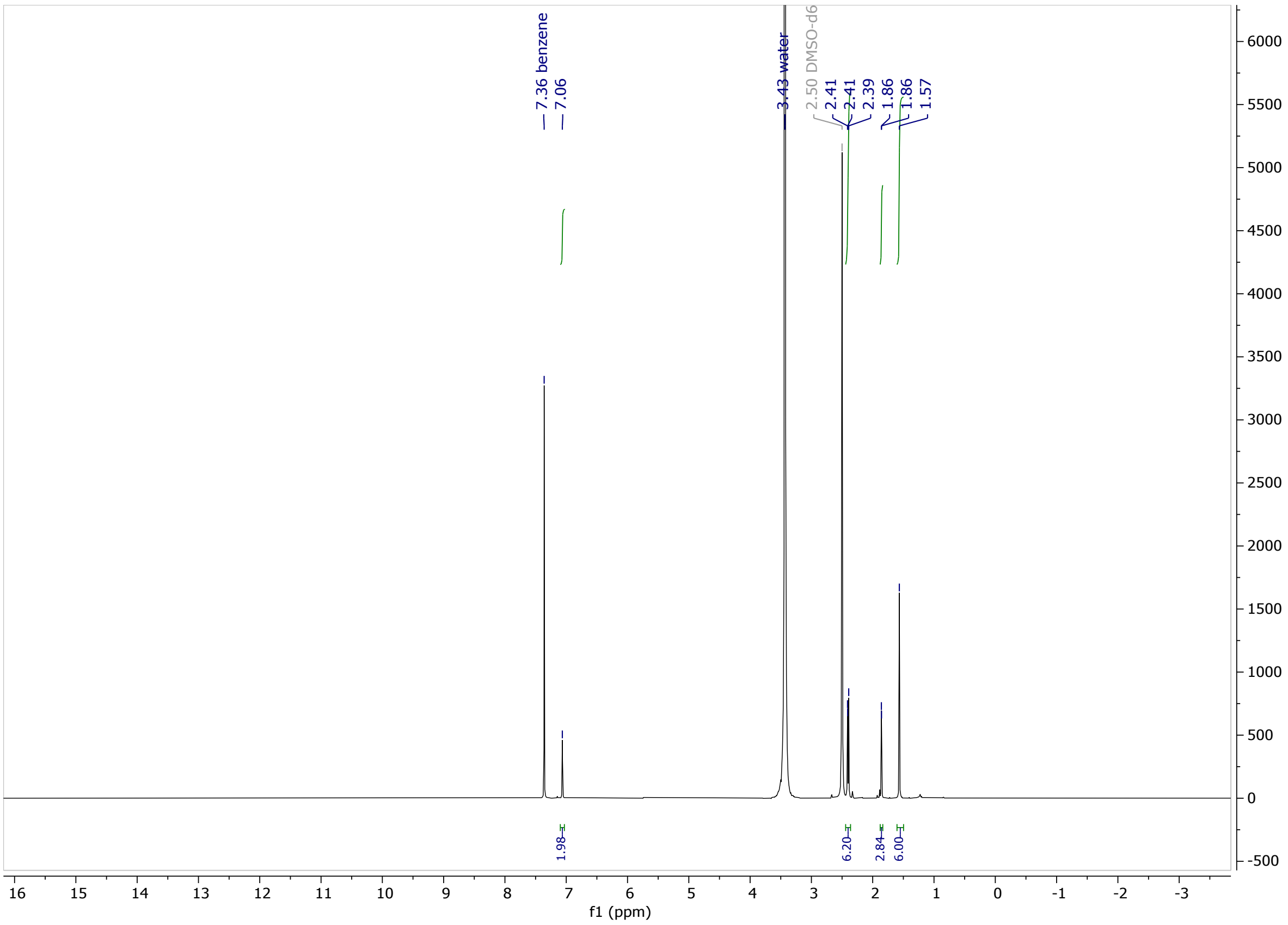

Figure S32: ^{1}H-NMR spectrum of complex **1** after 72 h in water/dmso with benzene as internal standard.

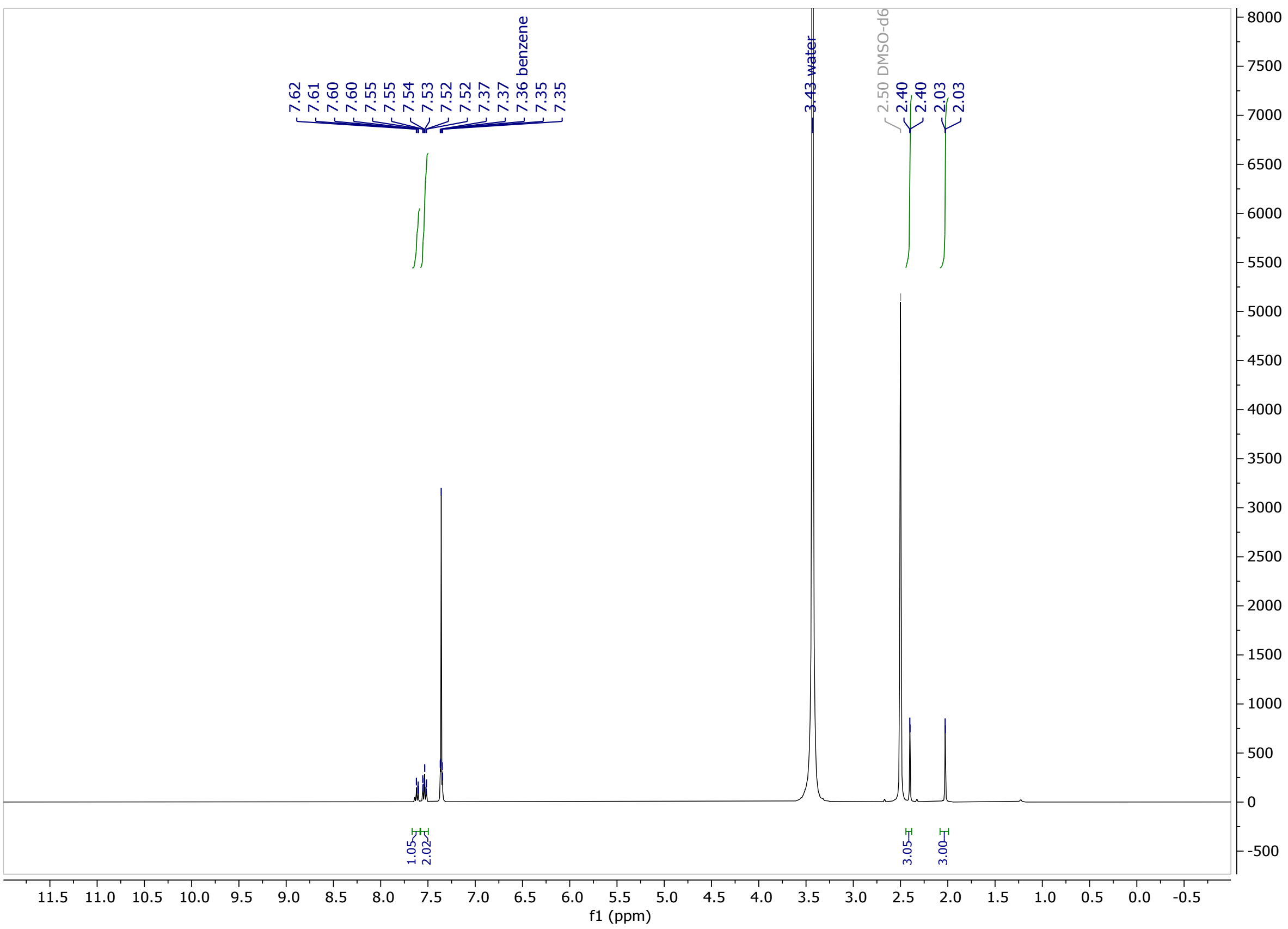

Figure S33: ^{1}H-NMR spectrum of complex **4** after 72 h in water/dmso with benzene as internal standard.

Reaction with N-acetyl-cysteine

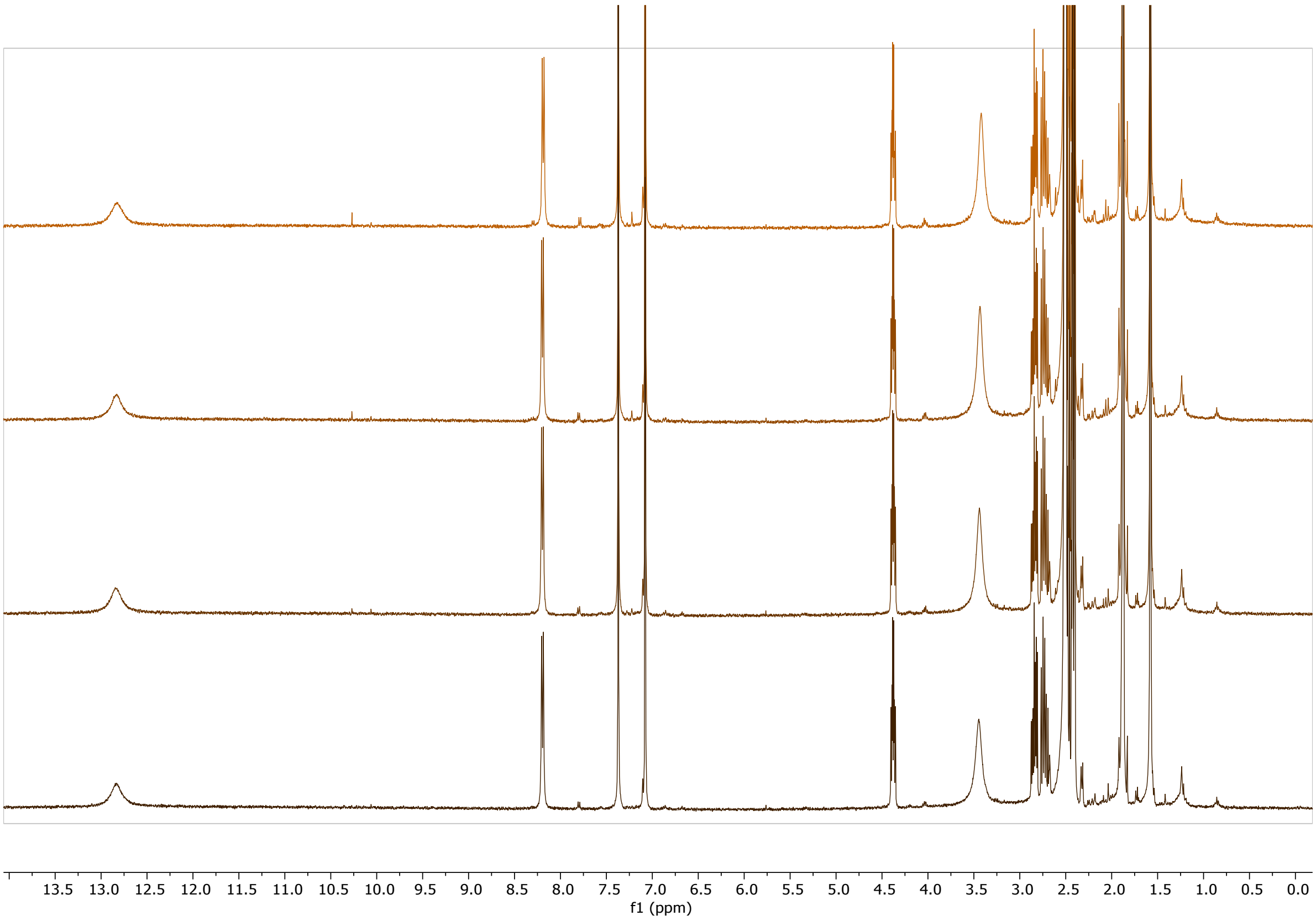

Figure S34: ^{1}H-NMR spectrum of complex **1** after4 h, 24 h, 48 h and 72 h in the presence of NAC with benzene as internal standard.

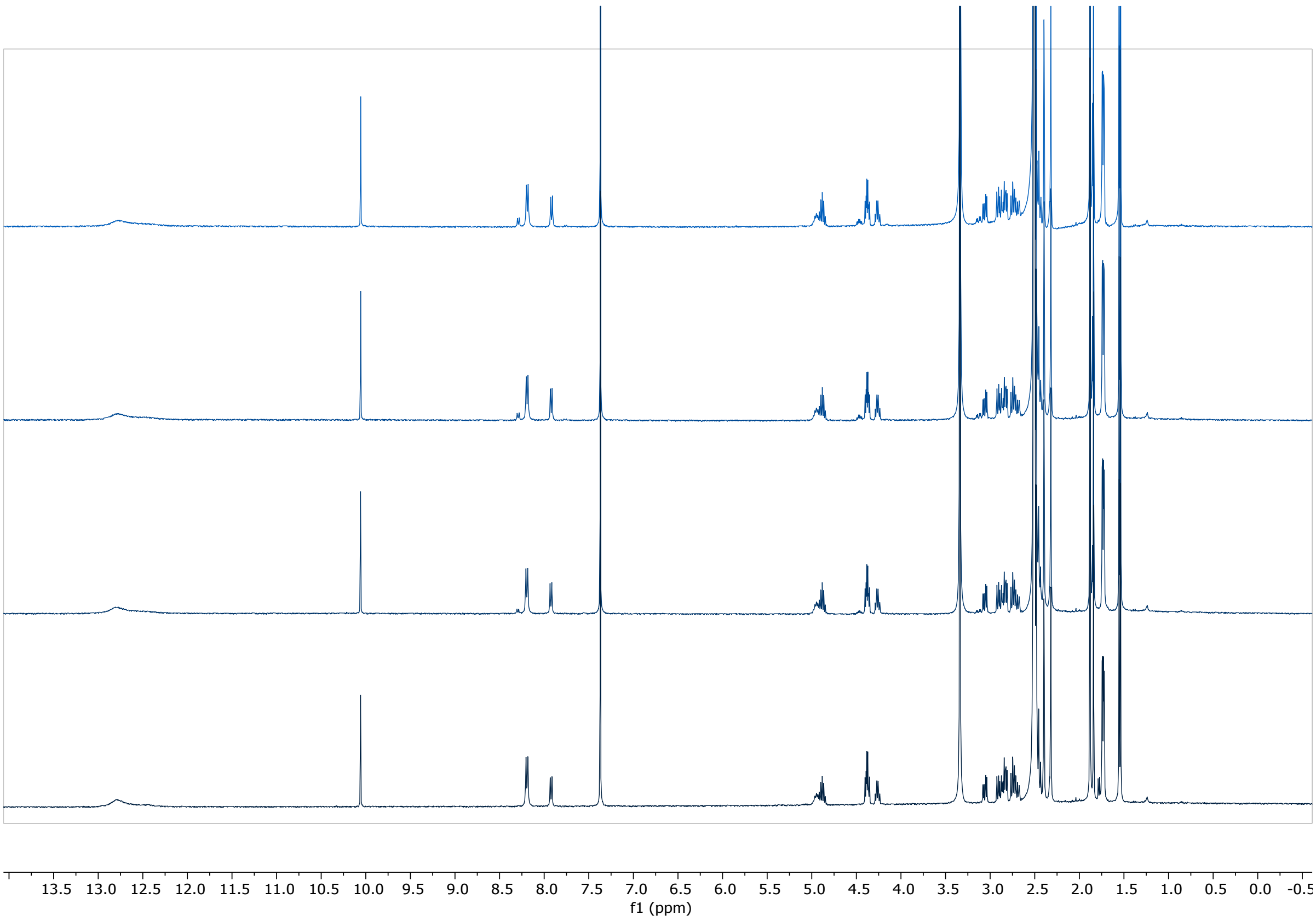

Figure S35: ^{1}H-NMR spectrum of complex **2** after4 h, 24 h, 48 h and 72 h in the presence of NAC with benzene as internal standard.

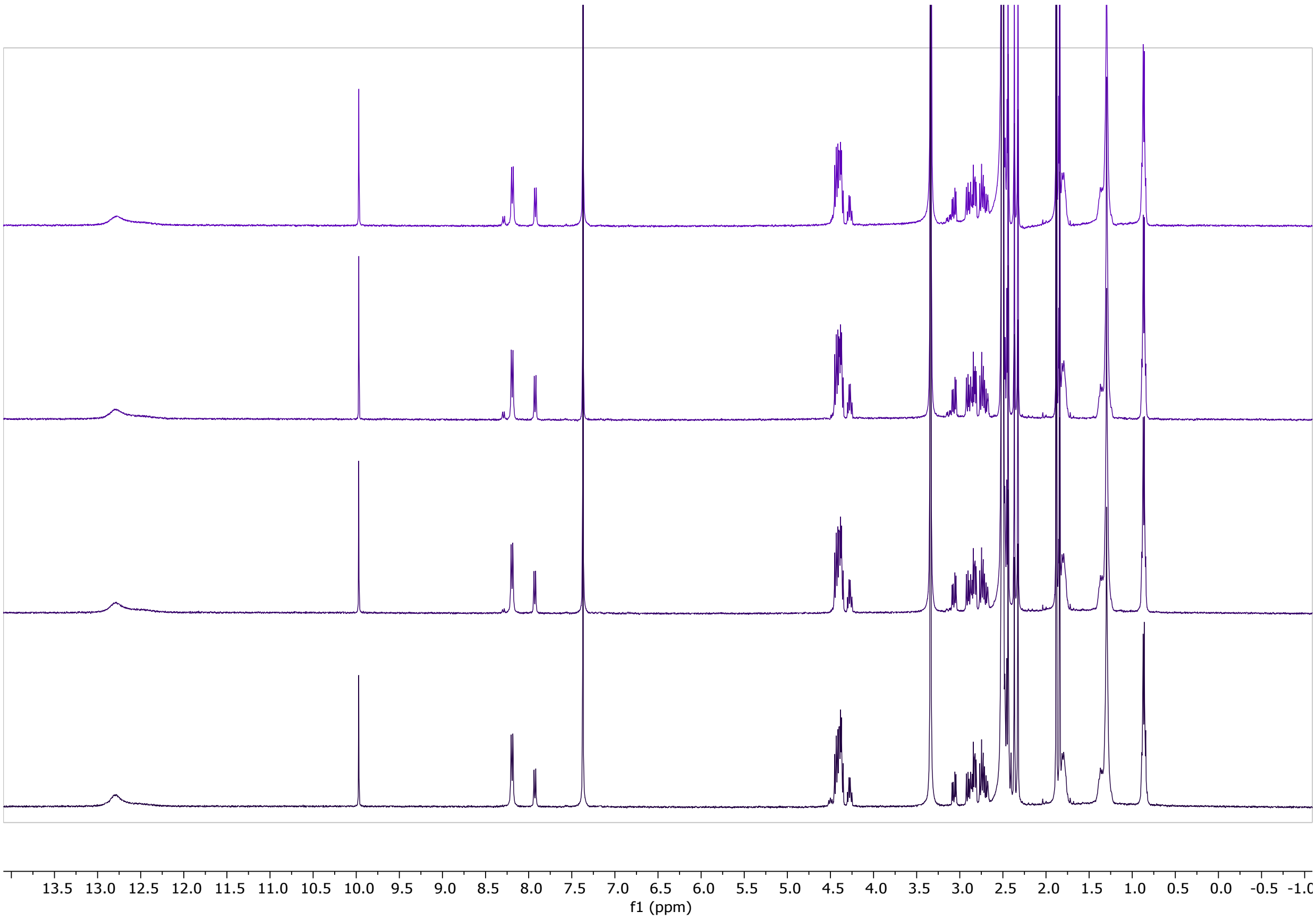

Figure S36: ^{1}H-NMR spectrum of complex **3** after4 h, 24 h, 48 h and 72 h in the presence of NAC with benzene as internal standard.

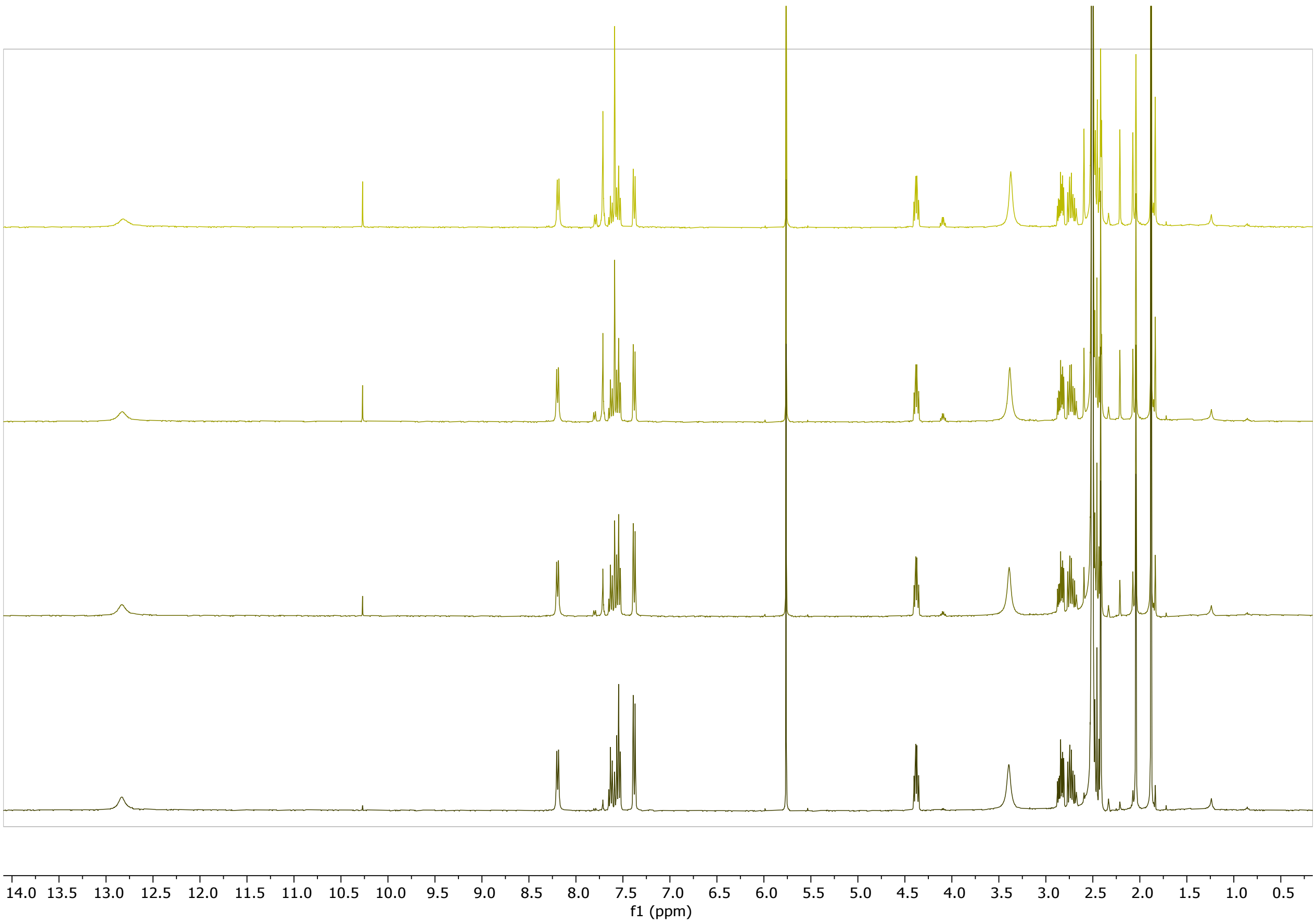

Figure S37: ^{1}H-NMR spectrum of complex **4** after4 h, 24 h, 48 h and 72 h in the presence of NAC with benzene as internal standard.

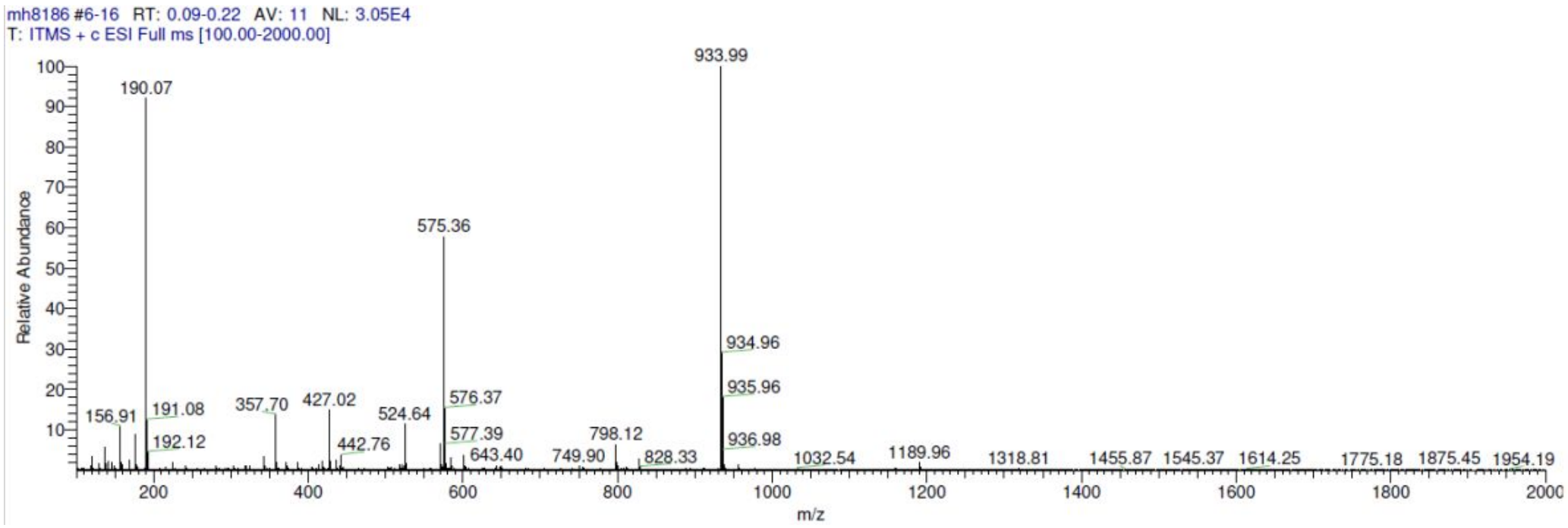

Figure S38: ESI-MS spectrum of complex **4** after 48 h in the presence of NAC in 10% dmso 90% acetonitrile.

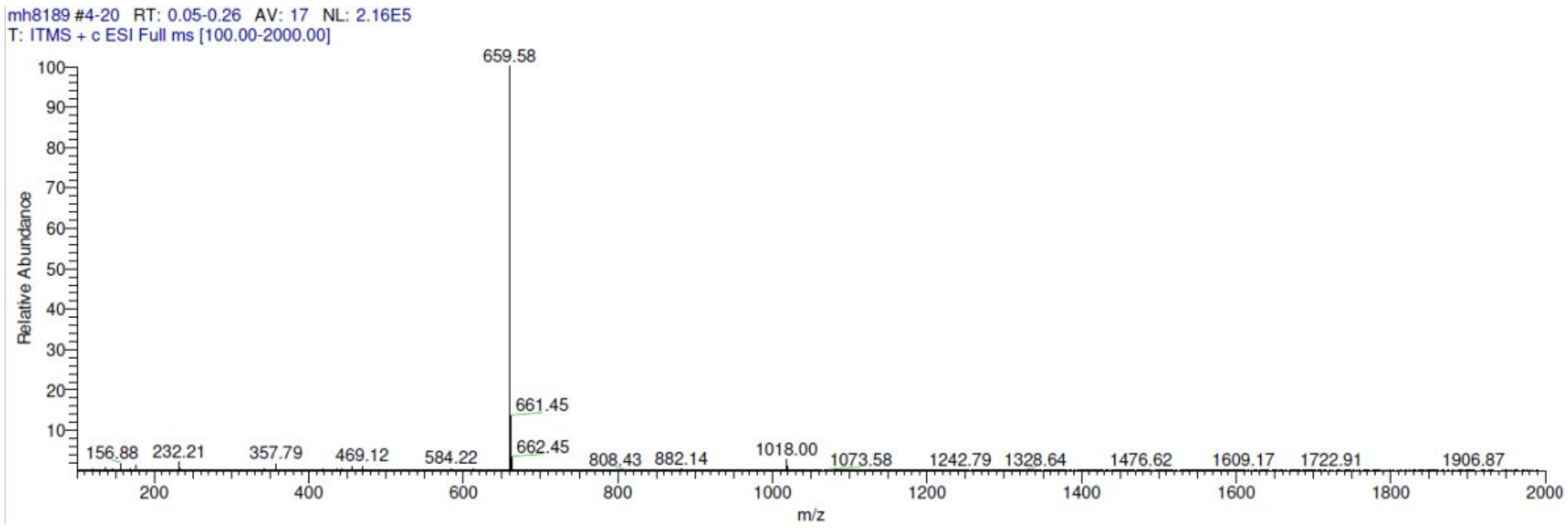

Figure S39: ESI-MS spectrum of complex **1** after 48 h in the presence of NAC in 10% dmso 90% acetonitrile.

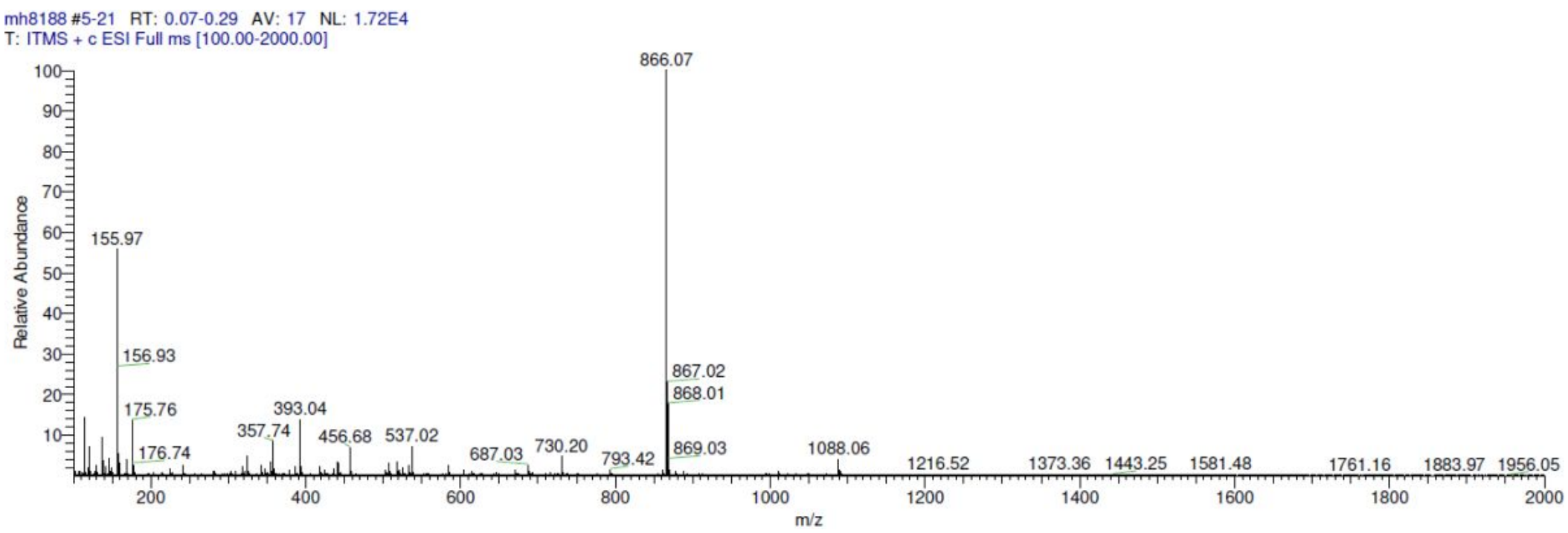

Figure S40: ESI-MS spectrum of complex **2** after 48 h in the presence of NAC in 10% dmso 90% acetonitrile.

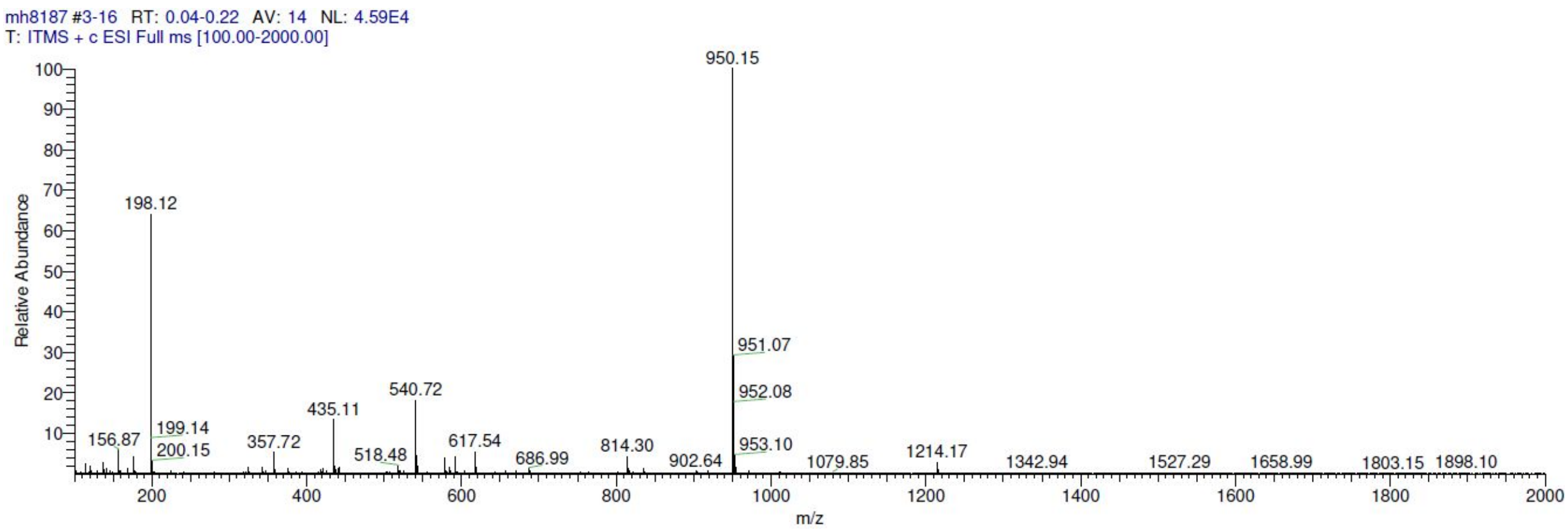

Figure S41: ESI-MS spectrum of complex **3** after 48 h in the presence of NAC in 10% dmso 90% acetonitrile.

Single Crystal X-Ray Diffraction

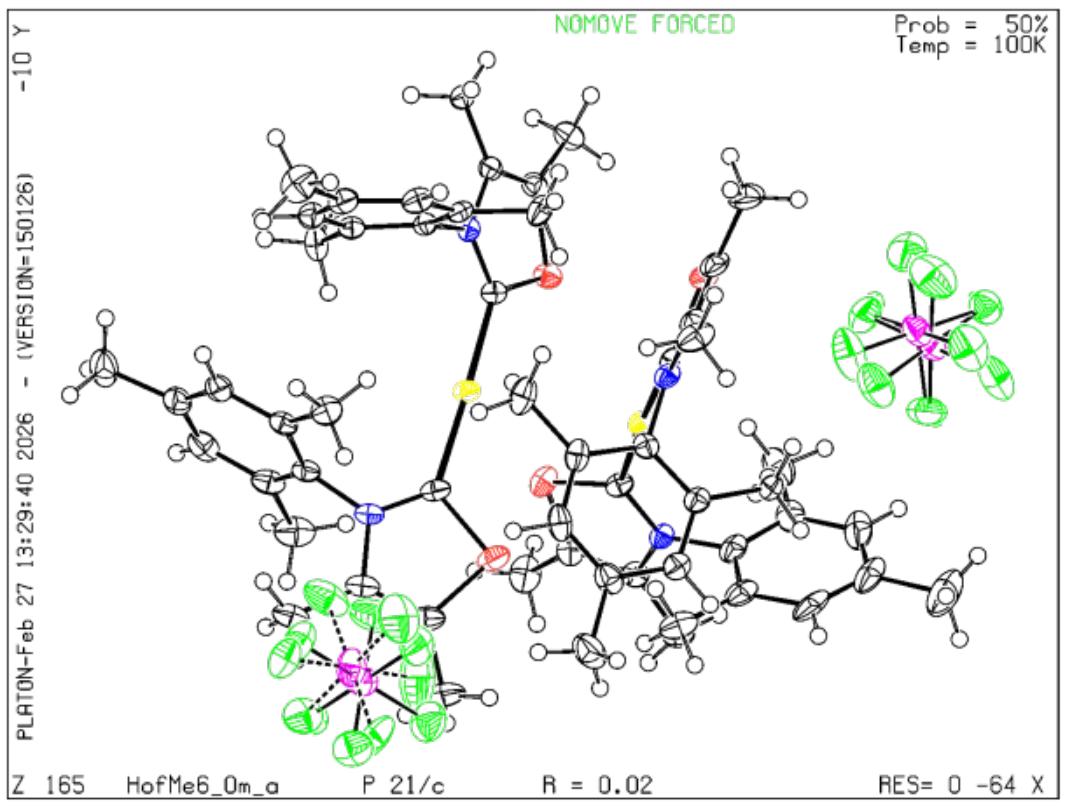

A colourless, needle-shaped crystal was mounted on a MiTeGen micromount with perfluoroether oil. Data for **1** were collected from a shock-cooled single crystal at 100(2) K on a Bruker D8 VENTURE three-angle diffractometer with a TXS rotating anode using a Helios optic as monochromator and a Bruker PHOTON III detector. The diffractometer used MoK_α radiation (λ = 0.71073 Å). All data were integrated with SAINT V8.41 and a multi-scan absorption correction using TWINABS 2012/1 was applied.[1,2] The structure was solved by iterative methods with SHELXT and refined by full-matrix least-squares methods against F^2 using SHELXL.[3,4] All non-hydrogen atoms were refined with anisotropic displacement parameters. All hydrogen atoms were refined isotropic on calculated positions using a riding model with their U_{iso} values constrained to 1.5 times the U_{eq} of their pivot atoms for terminal sp^3 carbon atoms and 1.2 times for all other carbon atoms. Disordered moieties were refined using bond lengths restraints and displacement parameter restraints. Some parts of the disorder model were introduced by the program DSR.[5] Crystallographic data for the structures reported in this paper have been deposited with the Cambridge Crystallographic Data Centre.[6] CCDC 2544149 contain the supplementary crystallographic data for this paper. These data can be obtained free of charge from The Cambridge Crystallographic Data Centre via www.ccdc.cam.ac.uk. This report and the CIF file were generated using FinalCif.[7]

Table S1. Crystal data and structure refinement for complex **1**

CCDC number	2544149
Empirical formula	$C_{28}H_{34}AuF_{6.02}N_2PS_2$
Formula weight	805.06
Temperature [K]	100(2)
Crystal system	monoclinic
Space group (number)	$P2_1/c$ (14)
a [Å]	22.0540(12)
b [Å]	11.7517(5)
c [Å]	23.8574(12)
α [°]	90
β [°]	98.249(2)
γ [°]	90
Volume [Å^3]	6119.2(5)
Z	8
ρ_{calc} [gcm^{-3}]	1.748
μ [mm^{-1}]	5.055
F(000)	3170
Crystal size [mm^3]	0.072×0.074×0.079
Crystal colour	colourless
Crystal shape	needle
Radiation	MoK_α (λ=0.71073 Å)
2θ range [°]	4.71 to 53.54 (0.79 Å)
Index ranges	−27 ≤ h ≤ 27 −14 ≤ k ≤ 14 −30 ≤ l ≤ 30
Reflections collected	295198
Independent reflections	13007 R_{int} = 0.0970 R_{sigma} = 0.0299
Completeness to θ = 25.242°	99.9 %
Data / Restraints / Parameters	13007 / 1516 / 869
Absorption correction T_{min}/T_{max} (method)	0.6516 / 0.7454 (multi-scan)
Goodness-of-fit on F^2	1.035
Final *R* indexes [I≥2σ(I)]	R_1 = 0.0231 wR_2 = 0.0438
Final *R* indexes [all data]	R_1 = 0.0422 wR_2 = 0.0511
Largest peak/hole [eÅ^{-3}]	0.81/−0.40

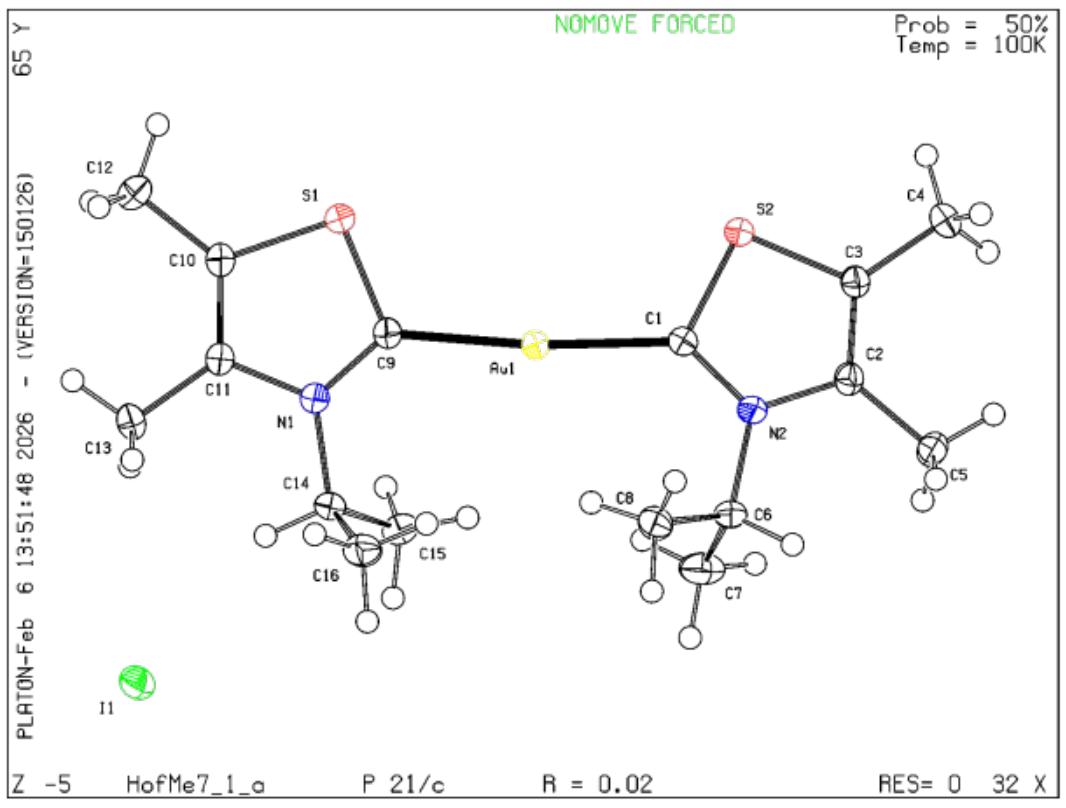

A white, needle-shaped crystal was mounted on a MiTeGen micromount with perfluoroether oil. Data for complex **2** were collected from a shock-cooled single crystal at 100(2) K on a Bruker D8 VENTURE three-angle diffractometer with a TXS rotating anode using a Helios optic as monochromator and a Bruker PHOTON III detector. The diffractometer used MoK_α radiation (λ = 0.71073 Å). All data were integrated with SAINT V8.41 and a multi-scan absorption correction using SADABS 2016/2 was applied.[1,2] The structure was solved by iterative methods with SHELXT and refined by full-matrix least-squares methods against F^2 using SHELXL.[3,4] All non-hydrogen atoms were refined with anisotropic displacement parameters. All hydrogen atoms were refined isotropic on calculated positions using a riding model with their U_{iso} values constrained to 1.5 times the U_{eq} of their pivot atoms for terminal sp^3 carbon atoms and 1.2 times for all other carbon atoms. Crystallographic data for the structures reported in this paper have been deposited with the Cambridge Crystallographic Data Centre.[5] CCDC 2544150 contain the supplementary crystallographic data for this paper. These data can be obtained free of charge from The Cambridge Crystallographic Data Centre via www.ccdc.cam.ac.uk. This report and the CIF file were generated using FinalCif.[6]

Table S2 Crystal data and structure refinement for complex **2**.

CCDC number	2544150
Empirical formula	$C_{16}H_{26}AuIN_2S_2$
Formula weight	634.37
Temperature [K]	100(2)
Crystal system	monoclinic
Space group (number)	$P2_1/c$ (14)
a [Å]	7.8753(5)
b [Å]	18.8387(13)
c [Å]	13.5339(9)
α [°]	90
β [°]	97.606(2)
γ [°]	90
Volume [Å^3]	1990.2(2)
Z	4
ρ_{calc} [gcm^{-3}]	2.117
μ [mm^{-1}]	9.151
F(000)	1200
Crystal size [mm^3]	0.034×0.038×0.059
Crystal colour	white
Crystal shape	needle
Radiation	MoK_α (λ=0.71073 Å)
2θ range [°]	5.22 to 61.01 (0.70 Å)
Index ranges	−11 ≤ h ≤ 11 −26 ≤ k ≤ 26 −19 ≤ l ≤ 19
Reflections collected	299756
Independent reflections	6071 R_{int} = 0.0826 R_{sigma} = 0.0149
Completeness to θ = 25.242°	99.9 %
Data / Restraints / Parameters	6071 / 0 / 207
Absorption correction T_{min}/T_{max} (method)	0.0625 / 0.0998 (multi-scan)
Goodness-of-fit on F^2	1.046
Final *R* indexes [I≥2σ(I)]	R_1 = 0.0156 wR_2 = 0.0359
Final *R* indexes [all data]	R_1 = 0.0178 wR_2 = 0.0365
Largest peak/hole [eÅ^{-3}]	1.32/−0.46

Biological Studies

Determination of Anitproliferattive Activity – MTT Assay

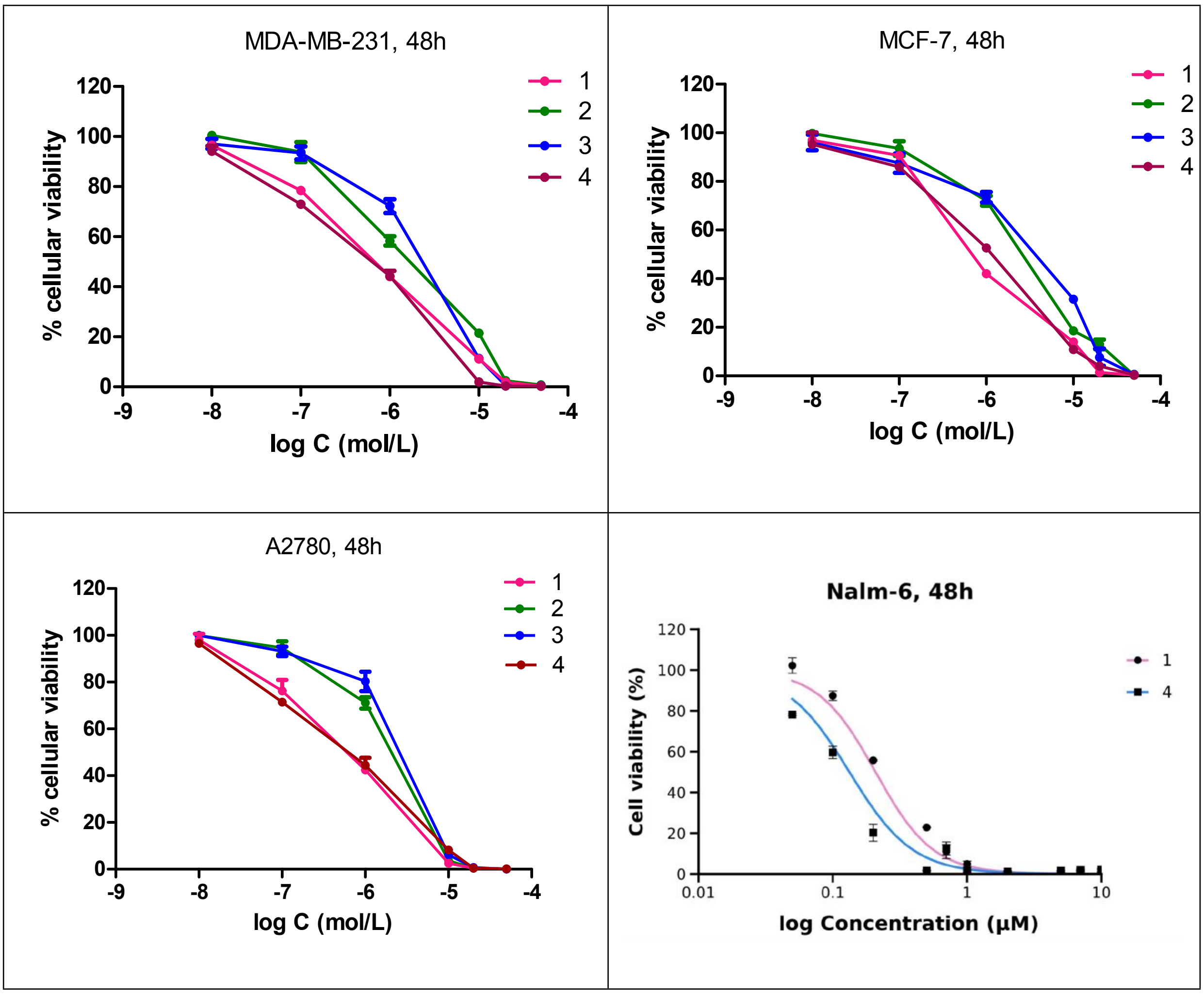

Figure S42: Dose-response curves for calculation of the IC50 values of **1**-**4**, using the GraphPad Prism software (vs. 5.0)

Investigations in Nalm6 and BJAB cells

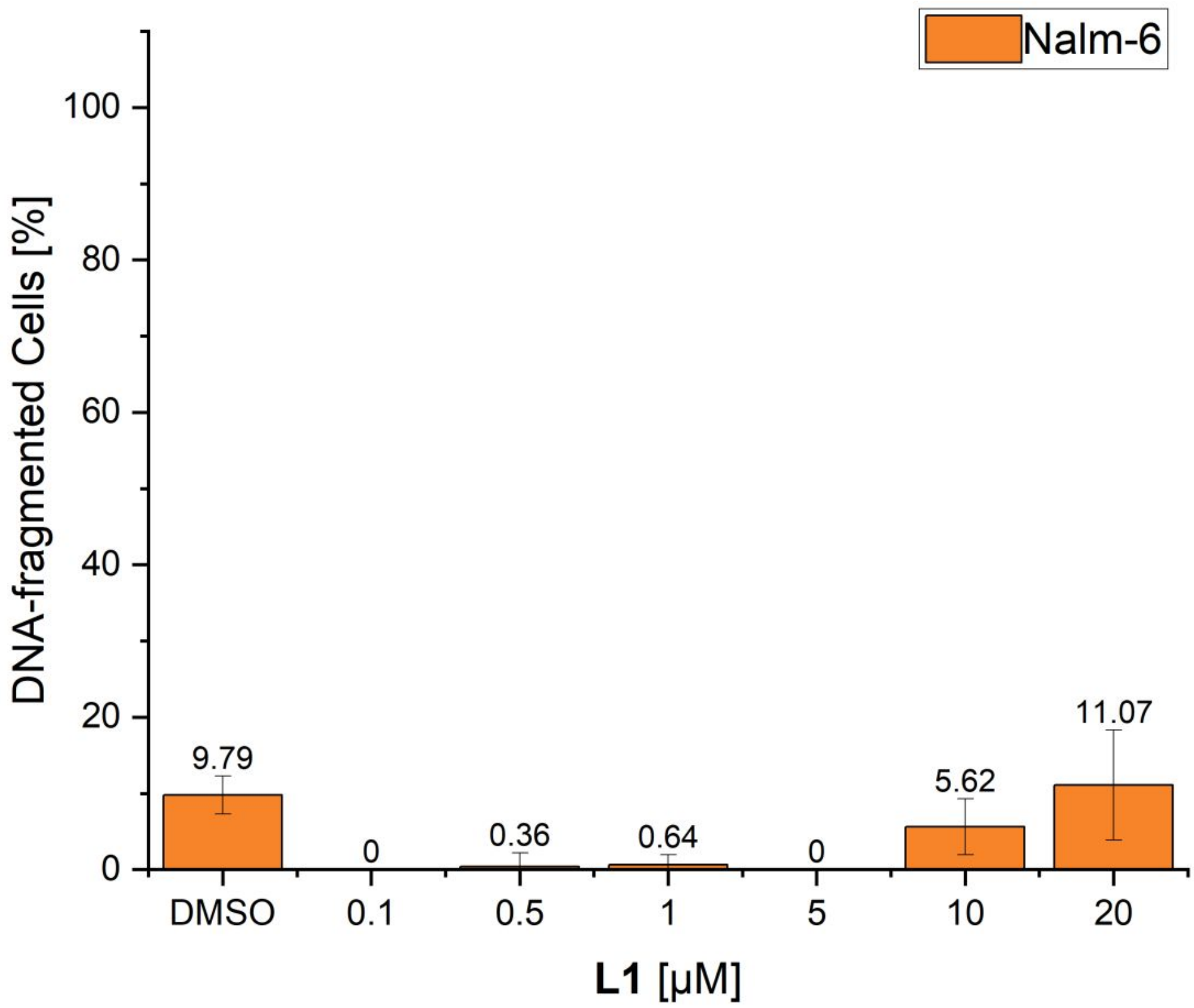

Figure S43: Nalm-6 cells were treated with ligand precursor L**1**. Each cell line was incubated with L**1** for 72 h. **1** barely induces apoptosis on Nalm-6 cells.

Table S3. Proliferation, viability, and inhibition of Nalm-6 cells after 24 h of treatment with complex 1

Cells were adjusted 24 h prior to treatment to 3 × 10^5 cells/mL to ensure standardized growth conditions. On the day of the experiment, cells were transferred into 12-well plates at a density of 1 × 10^5 cells/mL in 2 mL RPMI 1640 per well. Nalm-6 cells were incubated with complex **1** for 24 h 37 °C with 5% CO_2 with the indicated concentrations. Cell number and viability were measured using CASYCell Counter and Analyzer System (OMNI Life Science GmbH, Bremen, Germany). Data are presented as biological duplicates with three technical replicates (a–f) each and mean ± SD. Summarized data are shown in Figure 3.

Proliferation (cell count ×10^5)

Conc. [µM]	a	b	c	d	e	f	Mean ± SD
C	2.278	2.365	2.147	3.45	4.34	2.45	2.84 ± 0.87
DMSO	2.213	1.82	2.265	2.56	1.91	2.65	2.24 ± 0.33
0.5	1.466	1.453	1.51	1.67	1.87	1.65	1.60 ± 0.16
1	1.209	1.257	1.34	1.78	1.43	1.765	1.46 ± 0.25
2.5	0.729	0.781	0.843	0.898	0.99	0.934	0.86 ±

							0.10
5	0.58	0.506	0.664	0.65	0.43	0.6	0.57 ± 0.09

Viability [%]

Conc. [μM]	a	b	c	d	e	f	Mean ± SD
C	95.43	92.49	91.79	92.3	91.7	92.6	92.72 ± 1.38
DMSO	93.89	91.05	94.54	94.65	92.45	94.56	93.52 ± 1.47
0.5	91.55	94.07	92.27	93.12	92.21	93.32	92.76 ± 0.91
1	87.94	83.97	89.24	84.9	87.66	89.11	87.14 ± 2.20
2.5	67.89	71.89	71.75	70.23	72.65	68.11	70.42 ± 2.03
5	56.36	56.86	62.04	57.36	60	61.11	58.96 ± 2.41

Proliferation inhibition [%]

Conc. [μM]	a	b	c	d	e	f	Mean ± SD
DMSO	1.887	33.675	0	0	41.667	0	12.87 ± 19.39
0.5	62.308	63.359	58.749	57.051	44.231	58.333	57.34 ± 6.87
1	83.095	79.213	72.499	50	72.436	50.962	68.03 ± 14.20
2.5	121.92	117.714	112.699	106.538	100.641	104.231	100.00 ± 8.24
5	133.971	139.957	127.177	122.436	136.538	125.641	100.00 ± 6.88

C = untreated control. DMSO = vehicle control.

Table S4. Annexin V/PI staining of Nalm6 cells after 48 h treatment with gold complex 1

Nalm-6 cells were adjusted 24 h prior to treatment to 3 × 10^5 cells/mL to ensure standardized growth conditions. On the day of the experiment, cells were transferred into 12-well plates at a density of 1 × 10^5 cells/mL in 2 mL RPMI 1640 per well. Cells were incubated with increasing concentrations of **1** for 48 h at 37 °C with 5% CO_2. Viability, early apoptosis, late apoptosis, and necrosis were analyzed using flow cytometry. Data are presented as biological duplicates with three technical replicates (a–f) each and mean ± SD. Summarized data are shown in Figure 4.

Treatment	Annexin V+	Early apoptosis	Late apoptosis	Necrosis

DMSO	96.52	2.25	0.88	0.42
Control	100.00	0.16	0.02	0.01
0.5 µM	88.46	5.85	6.48	0.77
1 µM	45.37	19.07	33.93	1.86
2.5 µM	15.38	35.24	51.57	1.27
5 µM	0.92	49.70	48.39	0.72

Table S4A. Annexin V+ raw data

Treatment	a	b	c	d	e	f
DMSO	96.88	95.99	96.44	95.79	96.23	96.70
Control	99.82	99.88	99.87	99.75	99.74	99.81
0.5	87.38	87.85	89.37	84.77	85.26	85.12
1	45.91	40.53	47.32	37.76	49.49	50.70
2.5	20.35	13.59	8.20	8.04	8.68	10.36
5	0.71	1.11	1.96	0.75	0.53	0.73

Table S4B. Early apoptosis raw data

Treatment	a	b	c	d	e	f
DMSO	2.06	2.55	2.24	2.43	1.95	1.99
Control	0.15	0.10	0.11	0.25	0.21	0.15
0.5	5.82	5.76	5.71	6.27	5.67	5.34
1	20.14	22.34	18.47	18.08	16.33	17.40
2.5	33.17	34.94	40.65	32.67	34.75	35.84
5	51.29	51.32	50.02	49.59	46.29	42.95

Table S4C. Late apoptosis raw data

Treatment	a	b	c	d	e	f
DMSO	0.41	0.43	0.47	0.94	0.80	0.62
Control	0.00	0.01	0.00	0.00	0.00	0.02
0.5	0.80	0.74	0.85	1.22	1.12	2.97
1	1.98	1.74	4.62	3.78	1.84	1.90
2.5	1.61	0.93	1.15	1.83	1.61	1.17
5	0.69	0.74	1.29	0.83	0.93	0.94

Table S4D. Necrosis raw data

Treatment	a	b	c	d	e	f
DMSO	0.65	1.03	0.85	0.84	1.02	0.69
Control	0.03	0.01	0.02	0.00	0.05	0.02
0.5	6.00	5.65	5.07	7.74	7.94	6.57
1	31.97	35.39	29.59	40.38	32.34	30.00
2.5	44.87	50.54	50.00	57.46	54.96	52.63
5	47.31	46.83	46.73	48.83	52.25	55.39

Table S5. JC-1 assay of mitochondrial membrane potential in Nalm6 cells after treatment with 1

Suspension cells were adjusted 24 h prior to treatment to 3 × 10^5 cells/mL to ensure standardized growth conditions. On the day of the experiment, cells were transferred into 12-well plates at a density of 1 × 10^5 cells/mL in 2 mL RPMI 1640 per well. Nalm-6 cells were treated with the indicated concentrations of **1** for 48h at 37 °C with 5% CO_2. Changes in mitochondrial membrane potential was assessed using JC-1 staining and measured by flow cytometry. Data are presented as biological duplicates with three technical replicates (a–f) each and mean ± SD and adjusted apoptosis values. Summarized data are shown in Figure 5.

Treatment	a	b	c	d	e	f	Mean ± SD	Adjusted apoptosis [%]
Control + JC-1	1.9	1.5	1.5	1.9	1.5	1.5	1.63 ± 0.21	0.35
Control	0.1	0.1	0.1	0.1	0.1	0.1	0.10 ± 0.00	0.00
DMSO	1	0.9	0.9	1.6	2.5	0.8	1.28 ± 0.66	0.00
CCCP	68.6	47.7	46	78.1	78.5	73.6	65.42 ± 14.83	64.13
0.5 µM	11.4	9.9	10.6	6.3	5.3	6.4	8.32 ± 2.61	7.03
1 µM	25.8	24.8	25.7	18.6	16.6	8.5	20.00 ± 6.85	18.72
2.5 µM	57.4	40	48.3	14.2	17.7	12.6	26.56 ± 16.43	25.28
5 µM	41.6	41.3	43.8	33	56.3	44.4	43.40 ± 7.53	42.12

Adjusted data (background-corrected)

Treatment	a	b	c	d	e	f	Mean ± SD
CCCP	67.6	46.8	45.1	76.5	76	72.8	64.13 ± 14.45

0.5 µM	10.4	9	9.7	4.7	2.8	5.6	7.03 ± 3.09
1 µM	24.8	23.9	24.8	17	14.1	7.7	18.72 ± 7.02
2.5 µM	56.4	39.1	47.4	12.6	15.2	11.8	25.22 ± 16.77
5 µM	40.6	40.4	42.9	31.4	53.8	43.6	42.42 ± 7.20

CCCP = positive control for mitochondrial depolarization.

Table S6. ROS levels in Nalm6 cells treated with 1

Suspension cells were adjusted 24 h prior to treatment to 3 × 10^5 cells/mL to ensure standardized growth conditions. On the day of the experiment, cells were transferred into 12-well plates at a density of 1 × 10^5 cells/mL in 2 mL RPMI 1640 per well. Nalm-6 cells were incubated with NAC, hydrogen peroxide and **1** (indicated concentrations.) for 6 h at 37 °C with 5% CO_2. ROS was measured using flow cytometry. Data are presented as biological duplicates with three technical replicates (a–f) each and mean ± SD, and corresponding adjusted and uncorrected ROS values. Summarized data are shown in Figure 6. Pipetting error = #

Treatment	a	b	c	d	e	f	Mean ± SD	Adjusted	Uncorrected
DMSO	1.11	0.70	0.47	1.65	2.91	1.26	1.35 ± 0.87	0.00	0.00
KO	0.54	0.26	0.25	0.96	0.94	0.74	0.62 ± 0.32	0.00	-0.74
H2O2 (4 µL)	99.94	99.80	100.00	79.34	69.38	70.37	86.47 ± 15.13	85.12	85.12
H2O2 + NAC	0.12	0.28	0.10	0.00	0.00	0.00	0.08 ± 0.11	0.00	-1.27
NAC	0.32	0.05	1.02	0.32	0.05	1.02	0.46 ± 0.45	0.00	-0.89
1 µM	17.41	21.52	15.81	22.03	28.32	24.08	21.53 ± 4.53	20.18	20.18
1 µM + NAC	6.48	4.40	6.68	0.01	0.00	0.00	2.93 ± 3.30	1.58	1.58
2.5 µM	64.97	64.34	60.81	50.21	42.01	49.13	55.25 ± 9.45	53.90	53.90
2.5 µM + NAC	6.27	6.10	8.43	0.01	0.03	0.00	3.47 ± 3.88	2.12	2.12
5 µM	41.07	46.46	46.66	48.64	46.56	59.74	48.19 ± 6.20	46.84	46.84
5 µM + NAC	3.17	13.40#	2.86	0.26	0.03	0.01	3.29 ± 5.16	1.94	1.94

Adjusted data (background-corrected)

Treatment	a	b	c	d	e	f	Mean ± SD

H2O2 (4 µL)	98.83	99.10	99.53	77.69	66.47	69.11	85.12 ± 15.81
H2O2 + NAC	-0.99	-0.42	-0.37	-1.65	-2.91	-1.26	0.00 ± 0.00
NAC	-0.79	-0.65	0.55	-1.33	-2.86	-0.24	0.00 ± 0.00
1 µM	16.30	20.82	15.34	20.38	25.41	22.82	20.18 ± 3.83
1 µM + NAC	5.37	3.70	6.21	-1.64	-2.91	-1.26	1.58 ± 3.97
2.5 µM	63.86	63.64	60.34	48.56	39.10	47.87	53.90 ± 10.19
2.5 µM + NAC	5.16	5.40	7.96	-1.64	-2.88	-1.26	2.12 ± 4.58
5 µM	39.96	45.76	46.19	46.99	43.65	58.48	46.84 ± 6.24
5 µM + NAC	2.06	12.70	2.39	-1.39	-2.88	-1.25	1.94 ± 5.67

NAC = N-acetylcysteine.

Table S7. Resistance studies, activity of 1 in Nalm6 and JeFri cells (etoposide resistant)

Suspension cells were adjusted 24 h prior to treatment to 3 × 10^5 cells/mL to ensure standardized growth conditions. On the day of the experiment, cells were transferred into 12-well plates at a density of 1 × 10^5 cells/mL in 2 mL RPMI 1640 per well. Nalm-6 cells and JeFri cells were incubated with **1** and etoposide for 72 h at 37 °C with 5% CO with the indicated concentrations. Cell number and apoptosis induction were measured using flow cytometry. Data are presented as biological duplicates with three technical replicates (a–f) each and mean ± SD. Summarized data are shown in Figure 8.

Cell type	Treatment	a	b	c	d	e	f	Mean ± SD	Adjusted	Uncorrected
Nalm6	DMSO	6.55	7.84	8.50	2.8	3.4	2.94	5.34 ± 2.60	0.00	0.00
Nalm6	C	4.94	5.20	5.72	11.07	12.27	1.3	7.43 ± 2.23	0.00	-2.07
Nalm6	Etoposide	85.93	85.82	85.92	72.92	74.16	79.22	80.66 ± 6.10	75.32	75.32
Nalm6	0.1	20.33	13.83	21.94	5	5.07	5.75	11.99 ± 7.84	6.65	6.65
Nalm6	0.5	39.55	37.82	44.29	9.79	9.52	12.03	25.50 ± 16.65	20.16	20.16
Nalm6	1	68.97	72.23	71.40	29.83	27.72	23.47	48.94 ± 24.13	43.60	43.60
Nalm6	2.5	85.93	83.66	84.84	50.63	69.8	78	75.48 ± 13.56	70.14	70.14

Nalm6	5	87.12	85.77	86.71	93.28	93.64	93.6	90.02 ± 3.85	84.68	84.68
JeFri	DMSO	0.87	1.05	1.31	3.07	3.53	3.64	2.25 ± 1.30	0.00	0.00
JeFri	C	0.54	0.99	0.73	1.25	1.85	2.08	1.24 ± 0.61	0.00	-1.01
JeFri	Etoposide	3.17	3.53	4.09	6.88	7.38	8.25	5.55 ± 2.20	3.31	3.31
JeFri	0.1	1.66	1.19	1.9	3.18	5.05	4.6	2.81 ± 1.73	0.57	0.57
JeFri	0.5	5.25	7.07	4.62	7.69	8.88	12.98	7.75 ± 3.00	5.50	5.50
JeFri	1	25.17	30.71	28.06	31.68	27.91	21.53	27.51 ± 3.72	25.27	25.27
JeFri	2.5	55.98	61.26	58.74	56.35	63.49	60.05	59.31 ± 2.90	57.07	57.07
JeFri	5	80.65	80.67	80.25	89.77	83.57	84.88	83.30 ± 3.68	81.05	81.05

Table S8. Resistance studies, activity of 1 in Nalm6 and NaKu cells (prednisolone resistant)

Suspension cells were adjusted 24 h prior to treatment to 3 × 10^5 cells/mL to ensure standardized growth conditions. On the day of the experiment, cells were transferred into 12-well plates at a density of 1 × 10^5 cells/mL in 2 mL RPMI 1640 per well. Nalm-6 cells and NaKu cells were incubated with **1** and prednisolone for 72 h at 37 °C with 5% CO with the indicated concentrations. Cell number and apoptosis induction were measured using flow cytometry. Data are presented as biological duplicates with three technical replicates (a–f) each and mean ± SD. Summarized data are shown in Figure 8.

Cell type	Treatment	a	b	c	d	e	f	Mean ± SD	Adjusted	Uncorrected
Nalm6	DMSO	6.55	7.84	8.50	6.55	7.84	8.50	7.63 ± 0.89	0.00	0.00
Nalm6	C	4.94	5.20	5.72	4.94	5.20	5.72	5.29 ± 0.36	0.00	-2.34
Nalm6	Pred	30.50	35.05	37.30	30.50	35.05	37.30	34.28 ± 3.10	26.65	26.65
Nalm6	0.1	20.33	13.83	21.94	20.33	13.83	21.94	18.70 ± 3.84	11.07	11.07
Nalm6	0.5	39.55	37.82	44.29	39.55	37.82	44.29	40.55 ± 3.00	32.92	32.92
Nalm6	1	68.97	72.23	71.40	68.97	72.23	71.40	70.87 ± 1.52	63.24	63.24
Nalm6	2.5	85.93	83.66	84.84	85.93	83.66	84.84	84.81 ± 1.02	77.18	77.18
Nalm6	5	87.12	85.77	86.71	87.12	85.77	86.71	86.53 ± 0.62	78.90	78.90
NaKu	DMSO	0.77	0.80	0.89	1.45	1.52	1.75	1.20 ±	0.00	0.00

								0.43		
NaKu	C	0.43	0.64	0.57	1.43	1.55	2.08	1.12 ± 0.67	0.00	-0.08
NaKu	Pred	0.66	0.72	0.69	1.60	2.10	2.13	1.32 ± 0.71	0.12	0.12
NaKu	0.1	1.34	1.23	1.21	2.21	1.71	2.22	1.65 ± 0.47	0.46	0.46
NaKu	0.5	10.98	8.45	9.90	4.35	4.47	4.04	7.03 ± 3.12	5.84	5.84
NaKu	1	25.58	25.66	23.22	24.94	20.72	14.91	22.51 ± 4.16	21.31	21.31
NaKu	2.5	66.83	64.25	68.48	66.83	64.25	68.48	66.52 ± 1.91	65.32	65.32
NaKu	5	83.40	82.48	82.64	74.24	81.21	80.04	80.72 ± 3.42	79.53	79.53

Table S9. Selectivity of complex 1 in Nalm6 cells and healthy human leukocytes

Nalm-6 cells and leucocytes were incubated with complex **1** for 72h with the indicated concentrations. Apoptosis induction was measured using flow cytometry. Data are presented as biological duplicates with three technical replicates (a–f) each and mean ± SD, and adjusted apoptosis values. Summarized data are shown in Figure 9.

Cell type	Conc. [μM]	a	b	c	d	e	f	Mean ± SD	Adjusted	Uncorrected
Nalm6	DMSO	9.24	8.86	9.61	7.83	8.68	10.03	9.04 ± 0.77	0.00	0.00
Nalm6	0	5.35	6.67	6.00	7.83	8.68	10.03	7.43 ± 1.76	0.00	-1.62
Nalm6	0.25	33.95	39.93	40.71	35.80	34.74	26.69	35.30 ± 5.04	26.26	26.26
Nalm6	0.5	54.12	52.98	58.10	59.53	54.24	68.87	57.97 ± 5.91	48.93	48.93
Nalm6	1	72.50	73.71	71.20	72.05	79.24	82.73	75.24 ± 4.66	66.20	66.20
Nalm6	2.5	86.78	85.84	86.87	97.36	96.58	95.45	91.48 ± 5.50	82.44	82.44
Leukocytes	DMSO	2.13	2.13	2.48	2.63	1.81	2.10	2.21 ± 0.29	0.00	0.00
Leukocytes	0	2.23	1.71	1.98	1.84	1.93	1.84	1.92 ± 0.18	0.00	-0.29
Leukocytes	0.25	6.81	6.64	5.77	6.73	5.82	5.44	6.20 ± 0.59	3.99	3.99
Leukocytes	0.5	9.88	9.53	9.58	5.99	5.93	5.81	7.79 ± 2.06	5.57	5.57
Leukocytes	1	8.47	8.52	8.41	6.87	7.94	7.16	7.90 ± 0.72	5.68	5.68

Leukocytes	2.5	8.99	11.26	11.21	9.58	8.20	8.46	9.62 ± 1.34	7.40	7.40

Nalm-6 = leukemia cell line; leukocytes = healthy human leukocytes ex vivo.

Table S10: Apoptosis induction in Nalm-6 cells after treatment with L1

Nalm-6 cells were adjusted 24 h prior to treatment to 3 × 10^5 cells/mL to ensure standardized growth conditions. On the day of the experiment, cells were treated with **L1** at the indicated concentrations. Apoptosis induction was analyzed. Apoptosis was rarely seen. Data are presented as biological duplicates with three technical replicates (a–f) each and mean ± SD, including adjusted and uncorrected apoptosis values.

Conc. [μM]	a	b	c	d	e	f	Mean ± SD	Adjusted	Uncorrected
DMSO	12.57	12.22	11.36	7.55	7.68	7.36	12.05 ± 2.51	0.00	0.00
KO	8.90	8.75	8.75	4.65	4.53	5.09	6.78 ± 0.09	0.00	-5.27
0.1	11.08	11.66	11.69	7.54	5.65	5.98	8.93 ± 2.87	0.00	-3.12
0.5	12.80	14.56	13.84	6.09	5.72	7.80	10.14 ± 4.04	0.00	-1.92
1	14.06	14.02	13.42	6.67	7.09	7.29	10.43 ± 0.36	0.00	-1.63
5	9.12	7.55	6.96	7.48	9.57	7.69	8.06 ± 1.03	0.00	-3.99
10	20.94	20.82	20.97	10.37	10.91	8.42	15.41 ± 6.09	3.36	3.36
20	28.83	28.11	31.58	13.00	13.34	10.32	20.86 ± 9.60	8.81	8.81

Adjusted data (background-corrected)

Conc. [μM]	a	b	c	d	e	f	Mean ± SD
0.1	-1.49	-0.56	0.33	-0.01	-2.03	-1.38	0.00 ± 0.00
0.5	0.23	2.34	2.48	-1.46	-1.96	0.44	0.35 ± 1.85
1	1.49	1.80	2.06	-0.88	-0.59	-0.07	0.64 ± 1.30
5	-3.45	-4.67	-4.40	-0.07	1.89	0.33	0.00 ± 0.00
10	8.37	8.60	9.61	2.82	3.23	1.06	5.62 ± 3.65
20	16.26	15.89	20.22	5.45	5.66	2.96	11.07 ± 7.22

Western Blot Analysis of apoptosis-related proteins in BJAB cells.

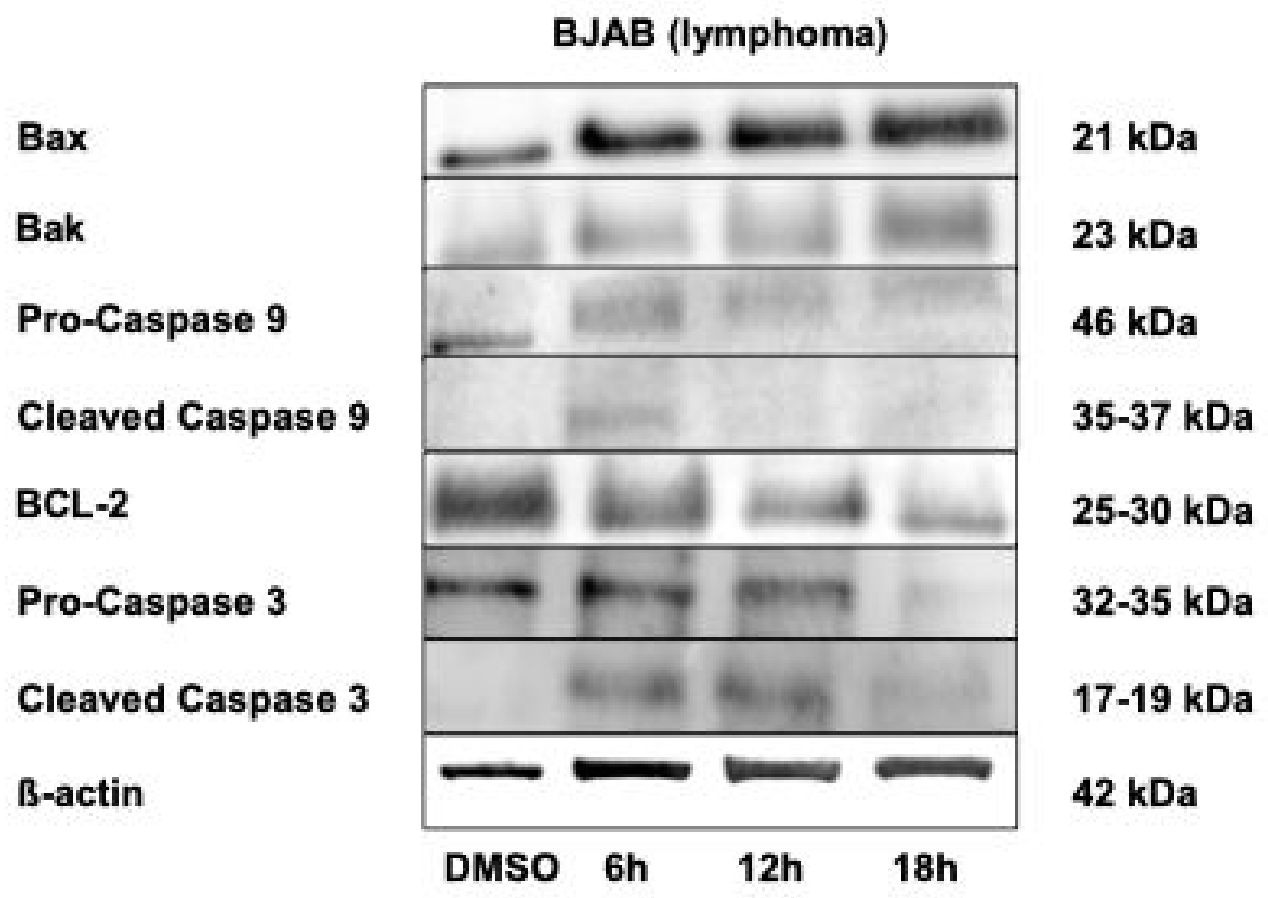

Figure S44: BJAB (b-cell lymphoma) cells were treated with **1** (10 µM) for the indicated time points (0, vehicle control (DMSO), 6, 12, 18, and 24 h). Whole-cell lysates were analyzed by Western blot for the expression of BAX, BAK, BCL-2, pro-caspase-9, cleaved caspase-9, pro-caspase-3, and cleaved caspase-3. β-Actin was used as a loading control. Results in Nalm-6 cells are shown in Figure 7 of the main text.

Proliferation inhibition

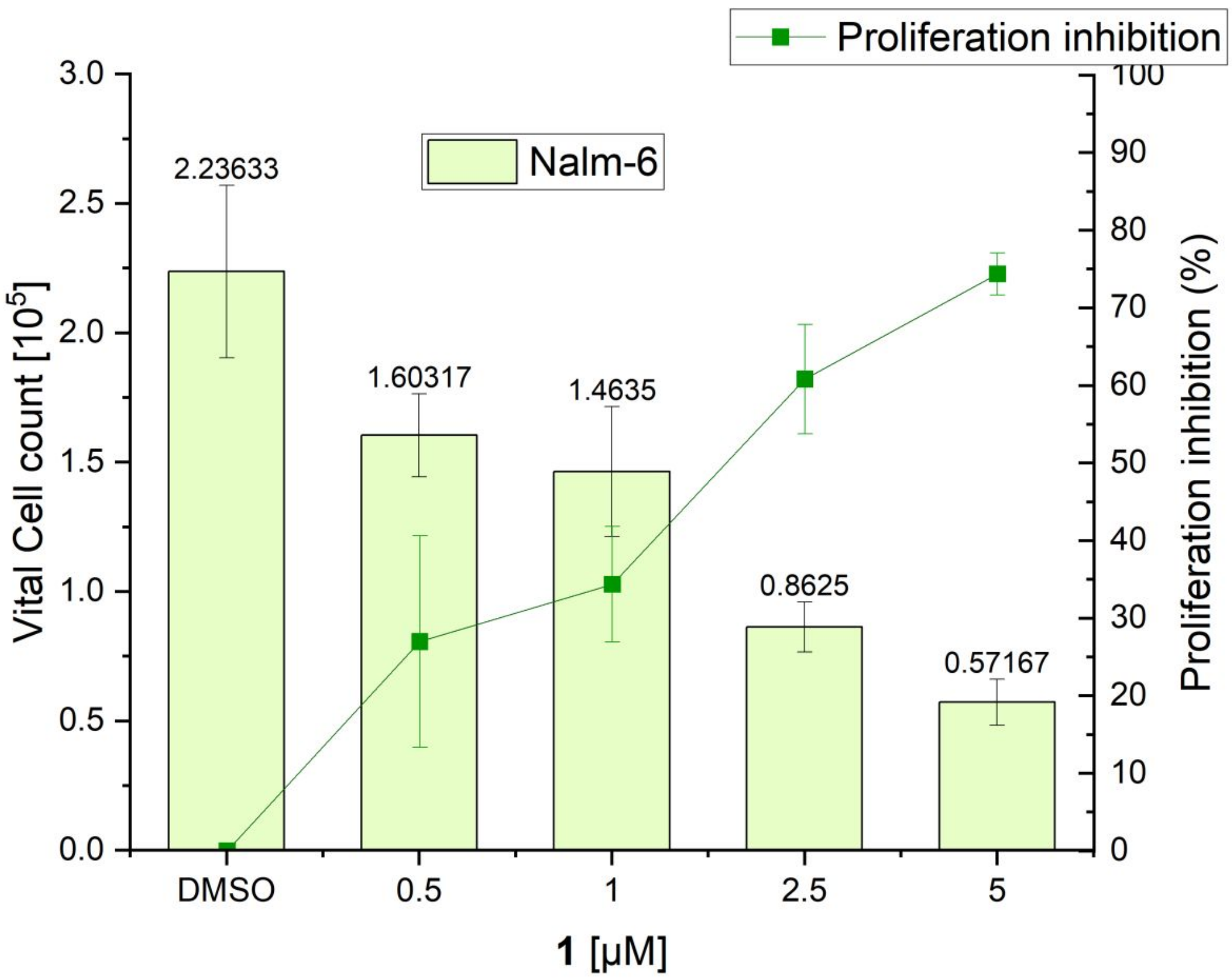

Figure S45: Inhibition of proliferation in leukemia cells (Nalm-6 cells): The cells were treated with increasing concentrations of complex **1** for 24 h. Afterwards the cell count was measured. Compared to DMSO control, the cells treated with **1** resulted in a concentration-dependent decrease in cell viability. At higher concentrations a complete inhibition of proliferation is reached.

References

[1] Bruker, *SAINT*, *V8.41*, Bruker AXS SE, Karlsruhe, Germany.
[2] L. Krause, R. Herbst-Irmer, G. M. Sheldrick, D. Stalke, *J. Appl. Cryst.* **2015**, *48*, 3–10, doi:10.1107/S1600576714022985.
[3] G. M. Sheldrick, *Acta Cryst.* **2015**, *A71*, 3–8, doi:10.1107/S2053273314026370.
[4] G. M. Sheldrick, *Acta Cryst.* **2015**, *C71*, 3–8, doi:10.1107/S2053229614024218.
[5] D. Kratzert, I. Krossing, *J. Appl. Cryst.* **2018**, *51*, 928–934, doi:10.1107/S1600576718004508.
[6] C. R. Groom, I. J. Bruno, M. P. Lightfoot, S. C. Ward, *Acta Cryst.* **2016**, *B72*, 171–179, doi:10.1107/S2052520616003954.
[7] D. Kratzert, *FinalCif*, *V156*, https://dkratzert.de/finalcif.html.
