# Supplementary material for: Synthesis, Cytotoxicity, and Mechanistic Evaluation of Thiazolium-Based Gold(I) Bis‑N‑Heterocyclic Carbene Complexes
Source: Inorg Chem. 2026 Oct 10;65(42):24706–19. doi: 10.1021/acs.inorgchem.6c02992 (PMC13715578; doi:10.1021/acs.inorgchem.6c02992)
Supplement: Supplementary file 2 [file ic6c02992_si_002.pdf]

CellROX Assay

Sample ID: N6-ATM_0,5 NAC

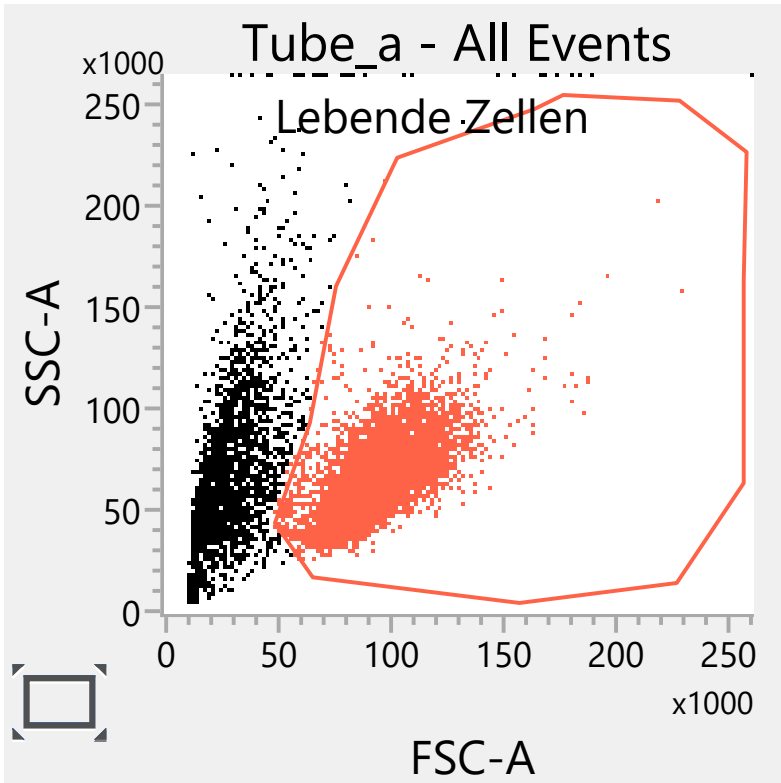

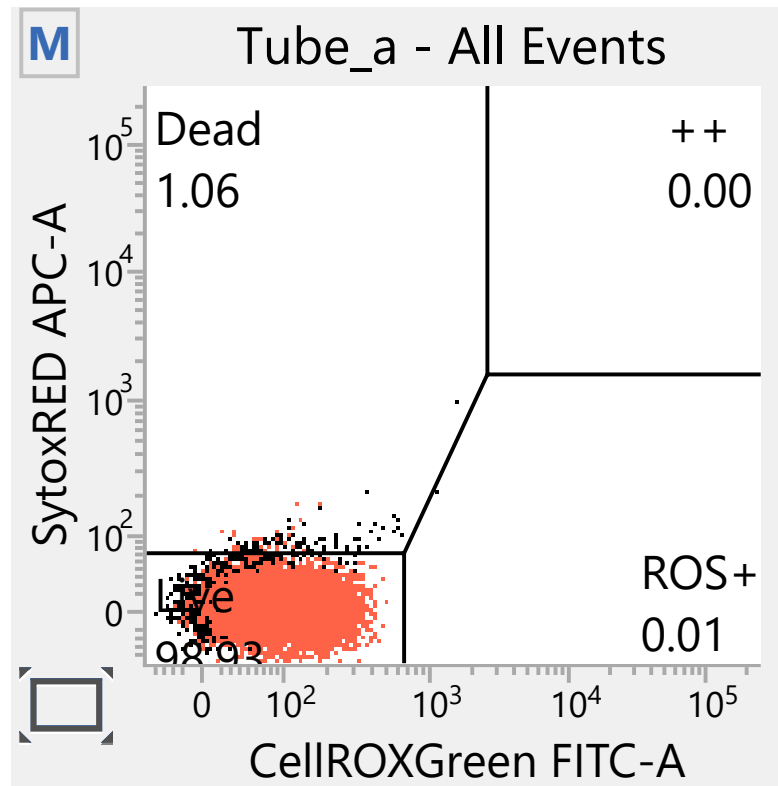

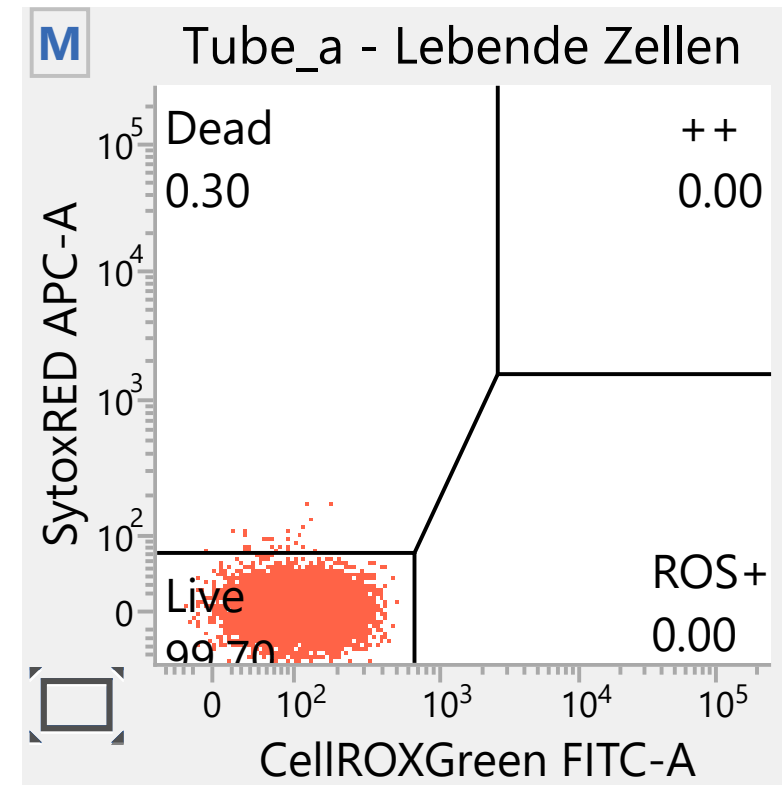

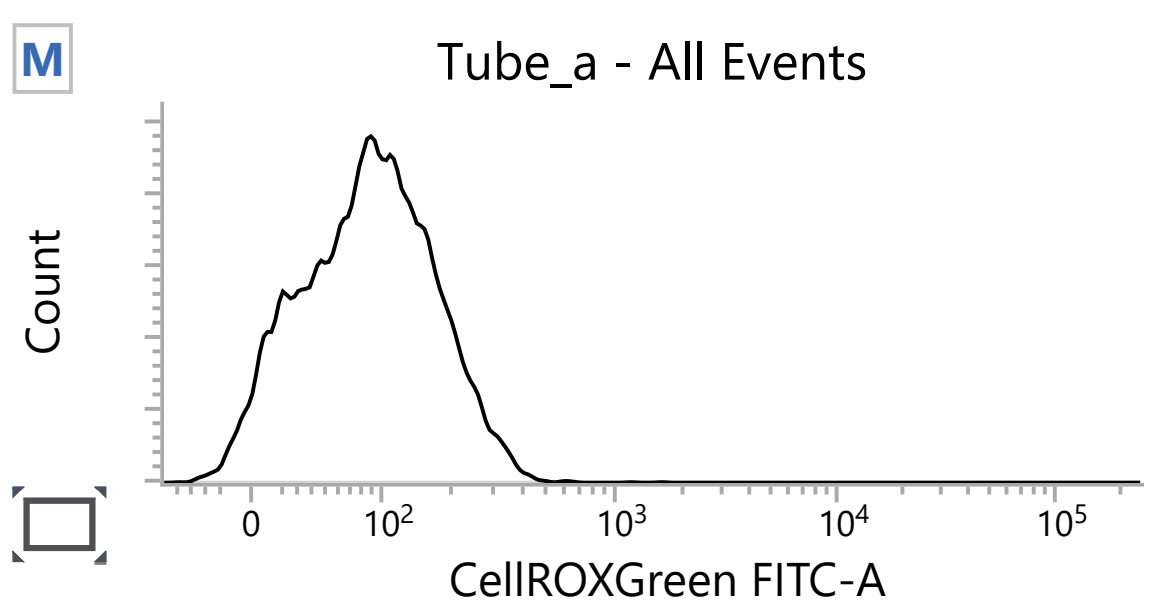

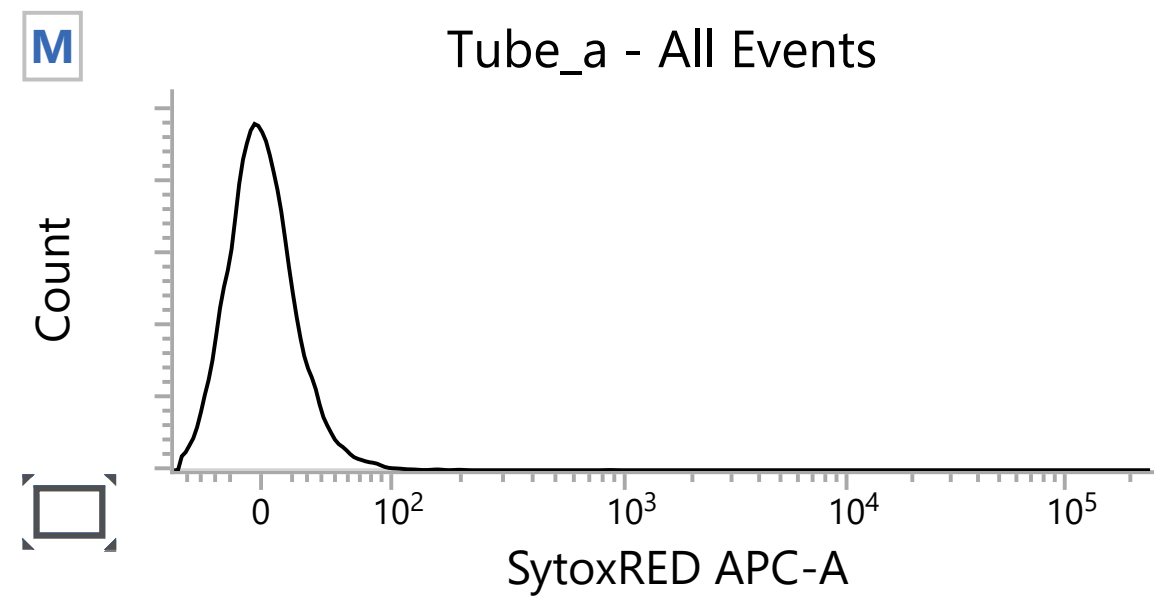

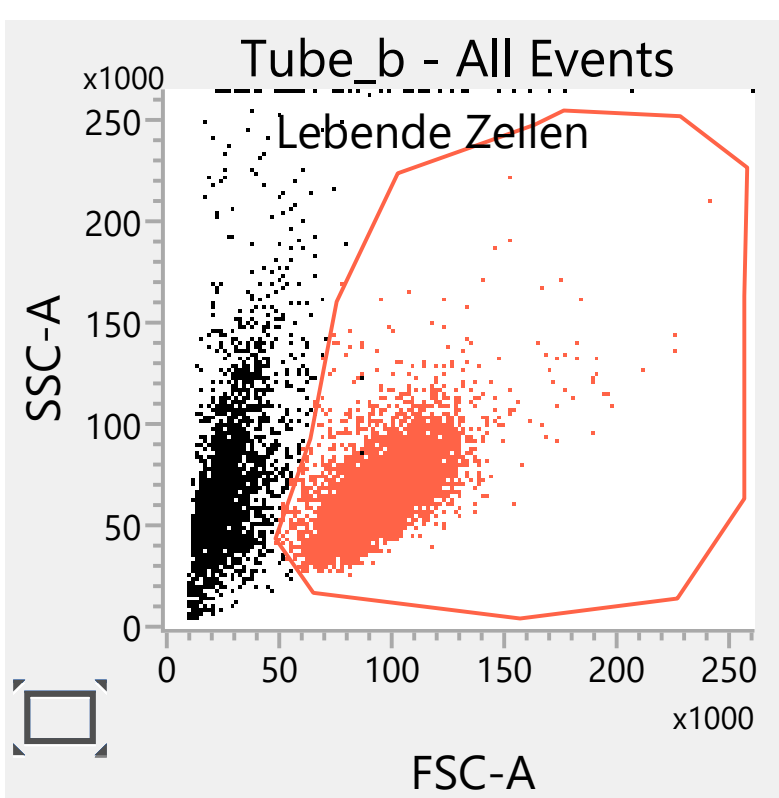

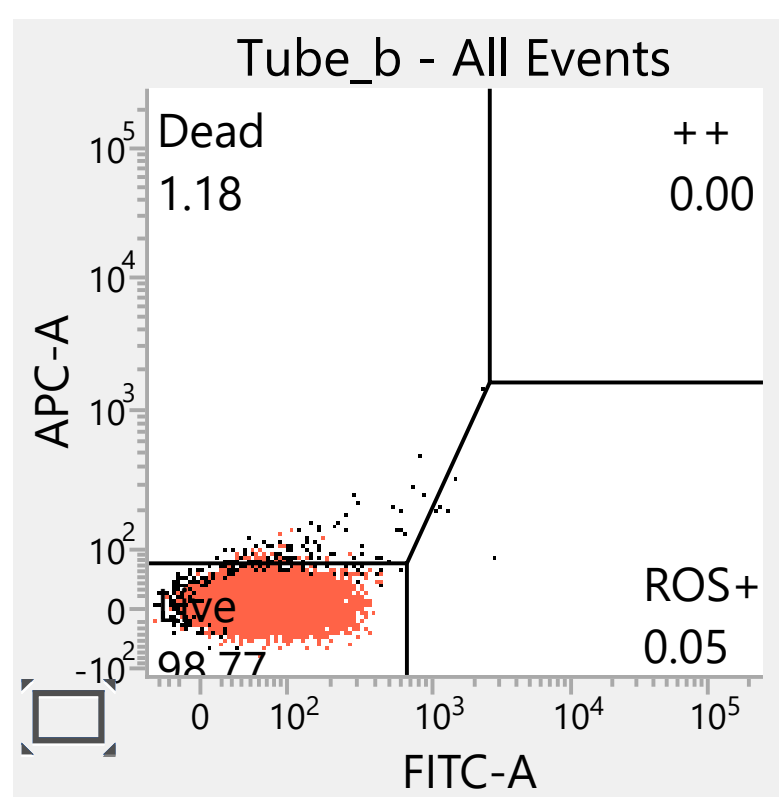

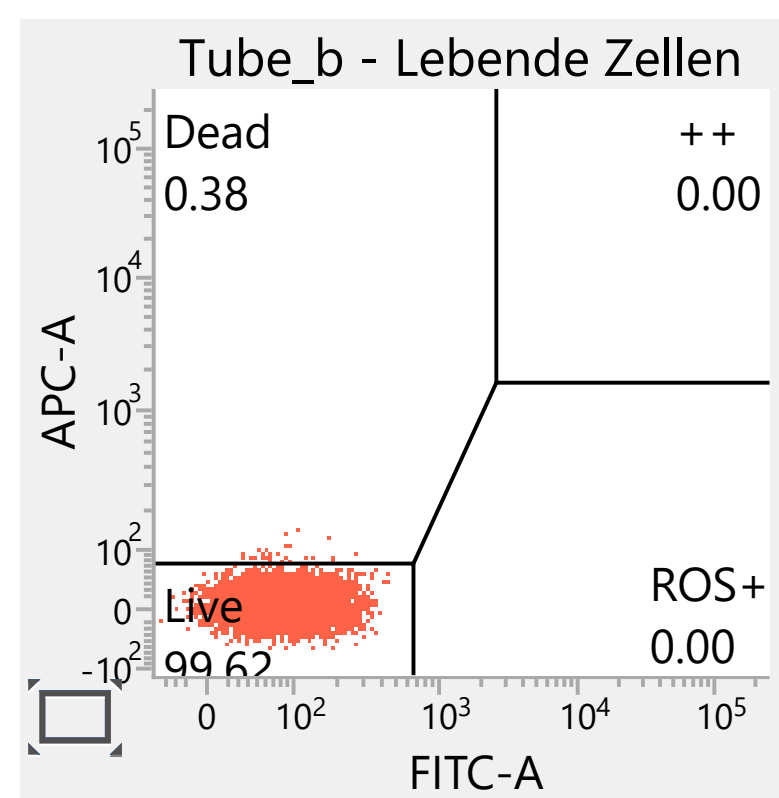

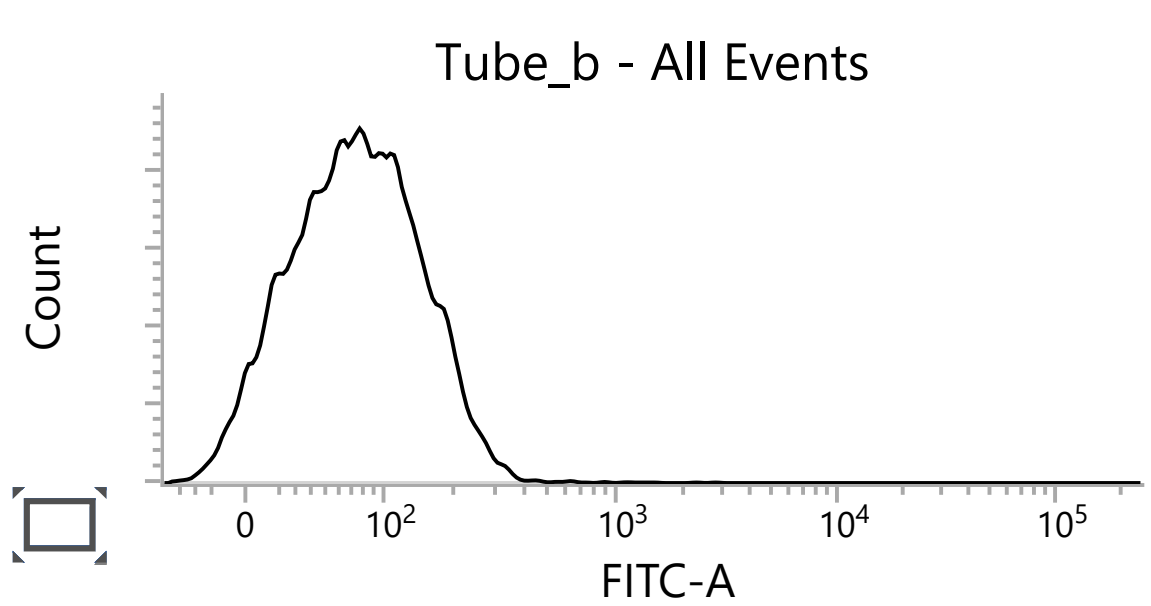

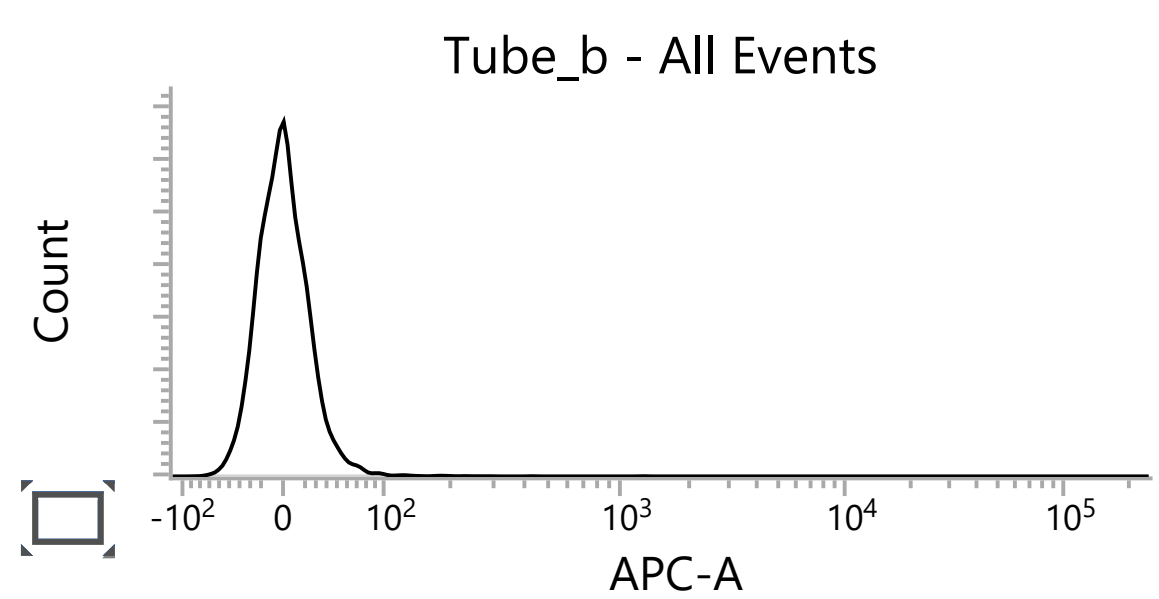

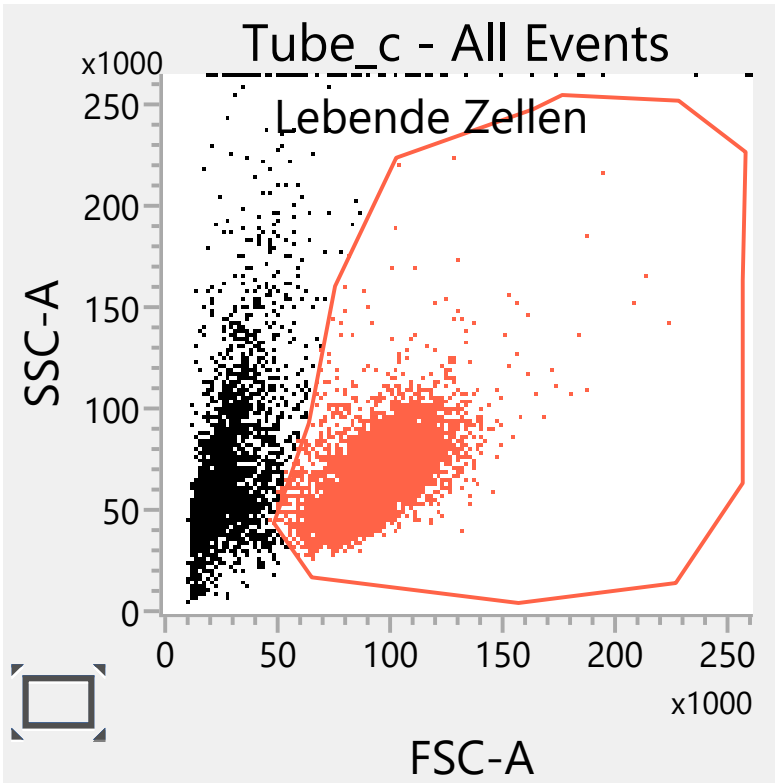

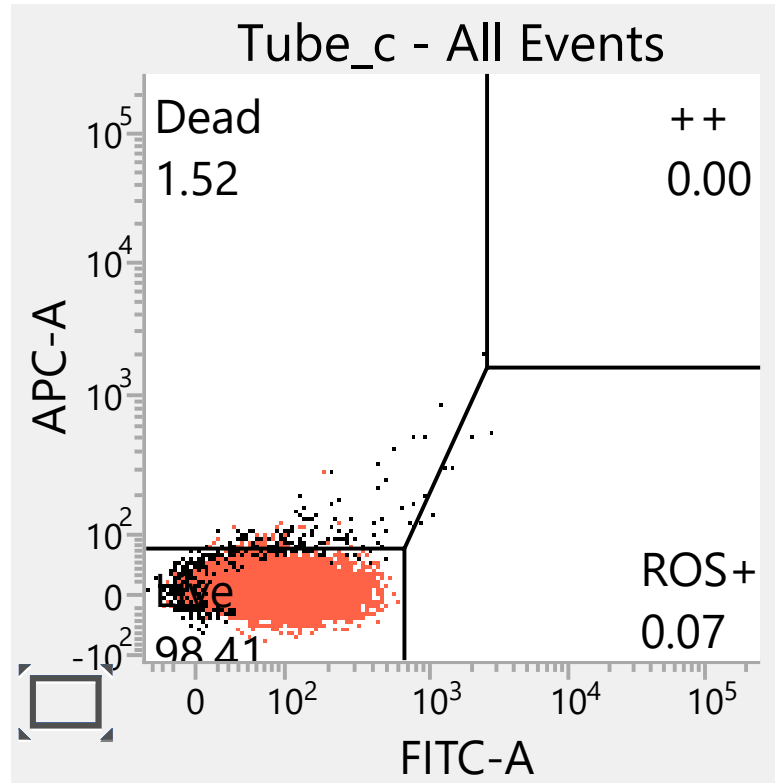

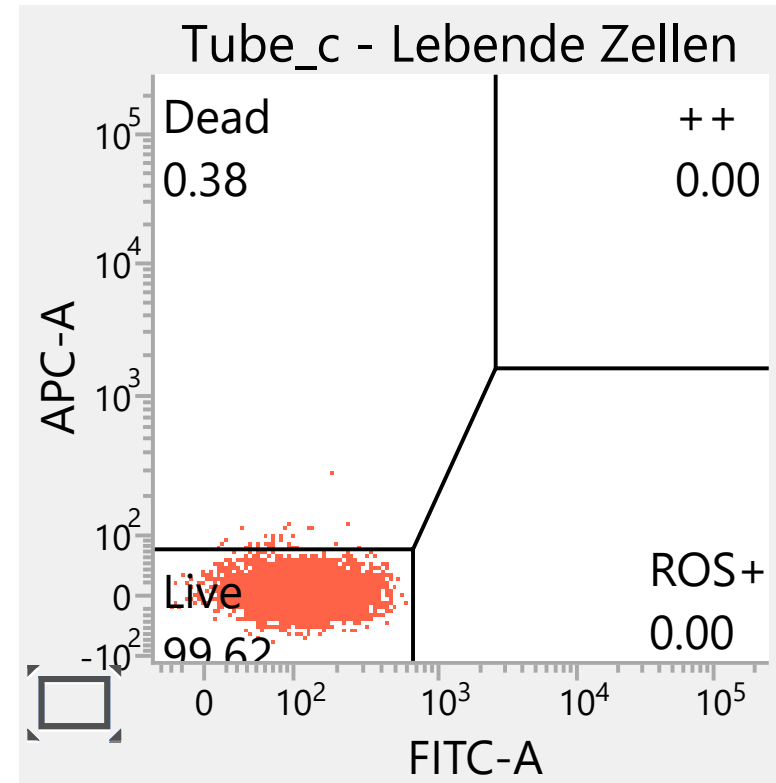

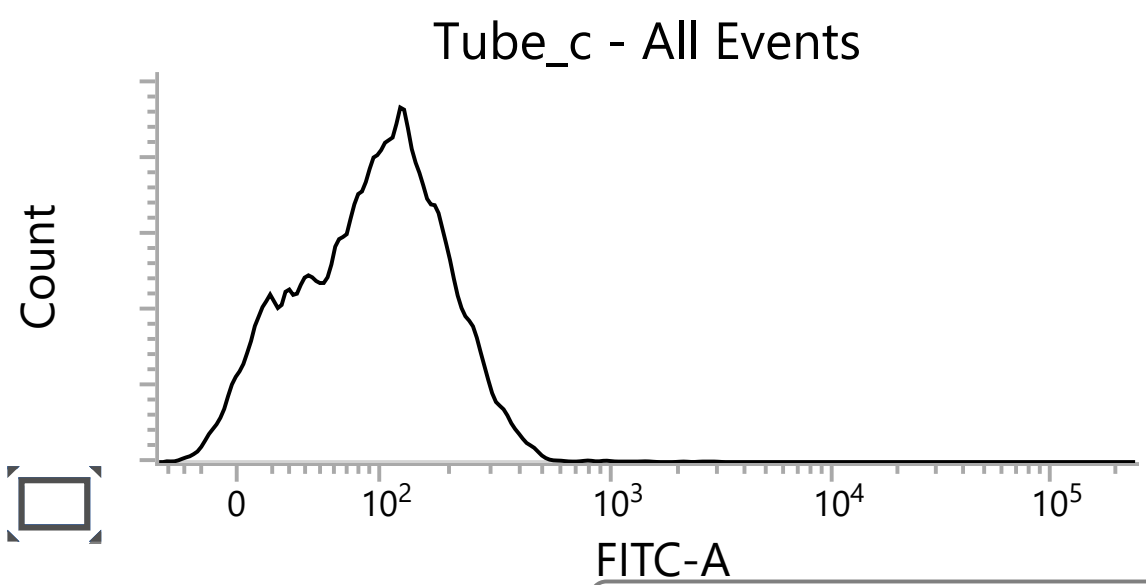

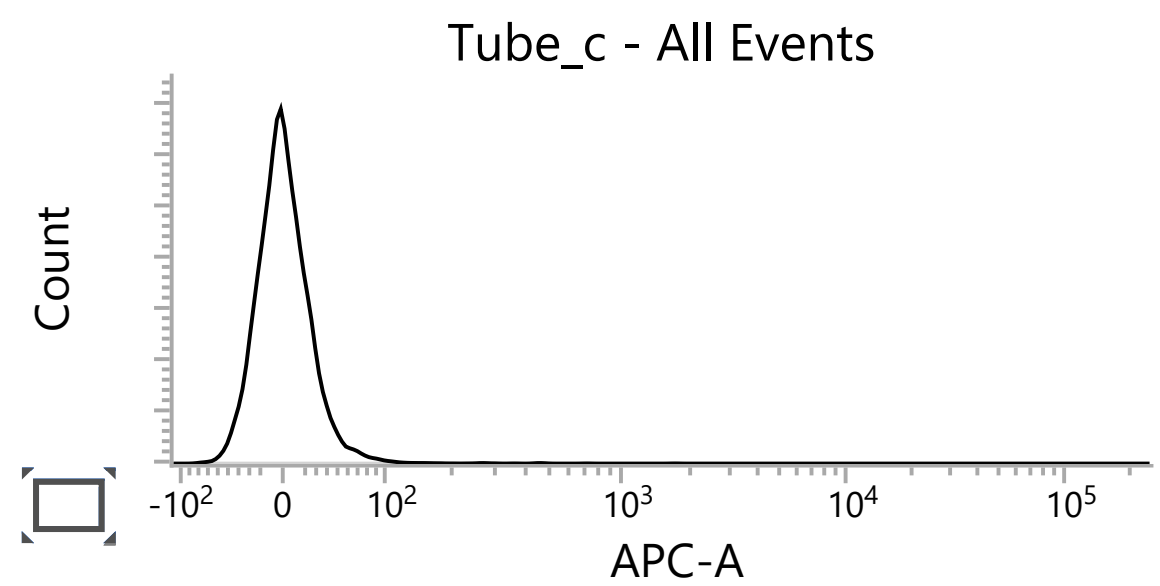

Statistics

Name	Events	% Parent	% Grandparent	% Total
Tube_a:All Events	10,000	***	***	100.00
Tube_b:All Events	10,000	***	***	100.00
Tube_c:All Events	10,000	***	***	100.00
Tube_a:Lebende Zellen	7,716	77.16	***	77.16
Tube_b:Lebende Zellen	7,618	76.18	***	76.18
Tube_c:Lebende Zellen	7,462	74.62	***	74.62

CellROX Assay

Sample ID: N6-ATM_0,5

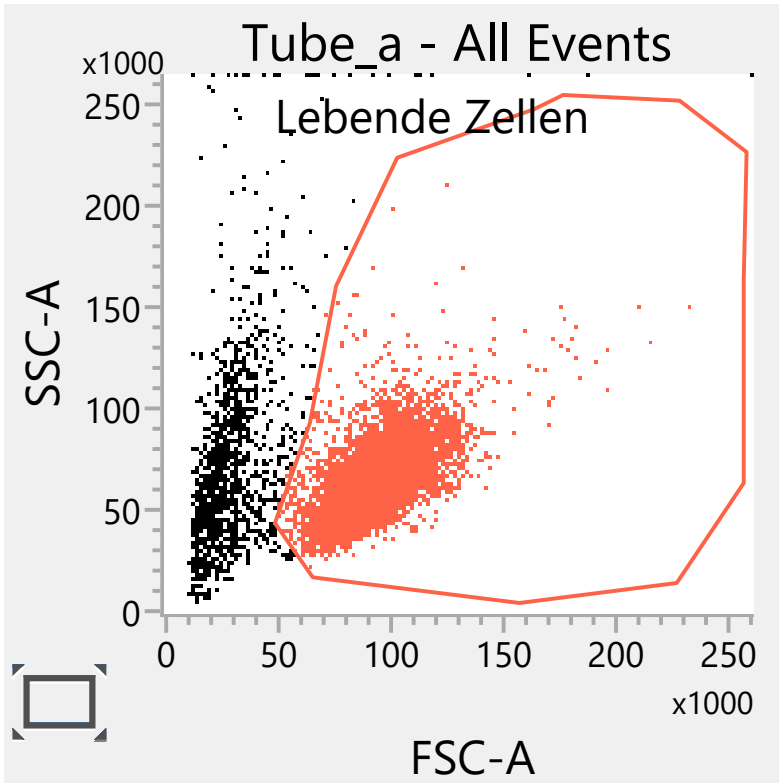

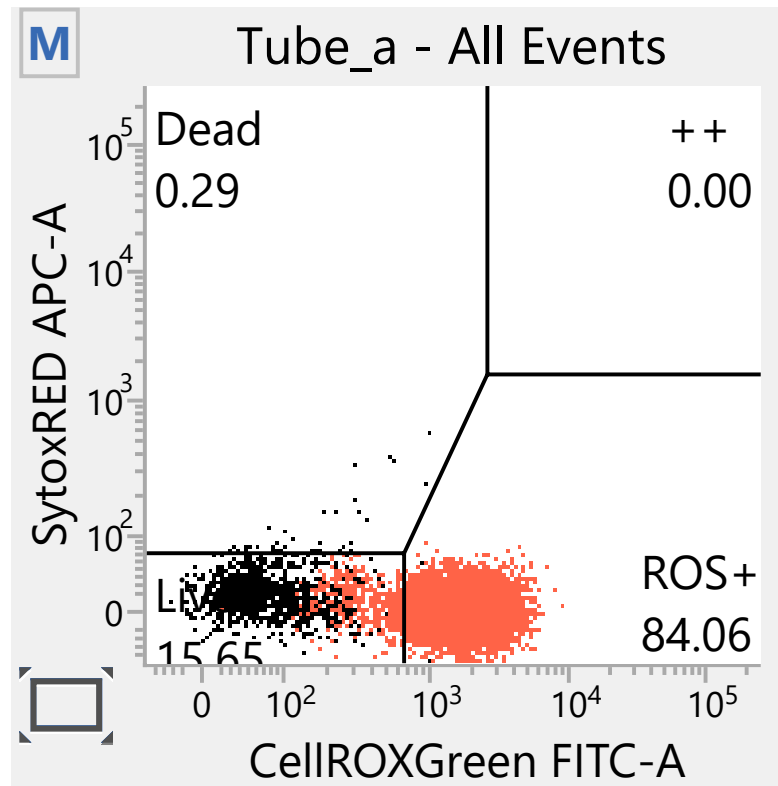

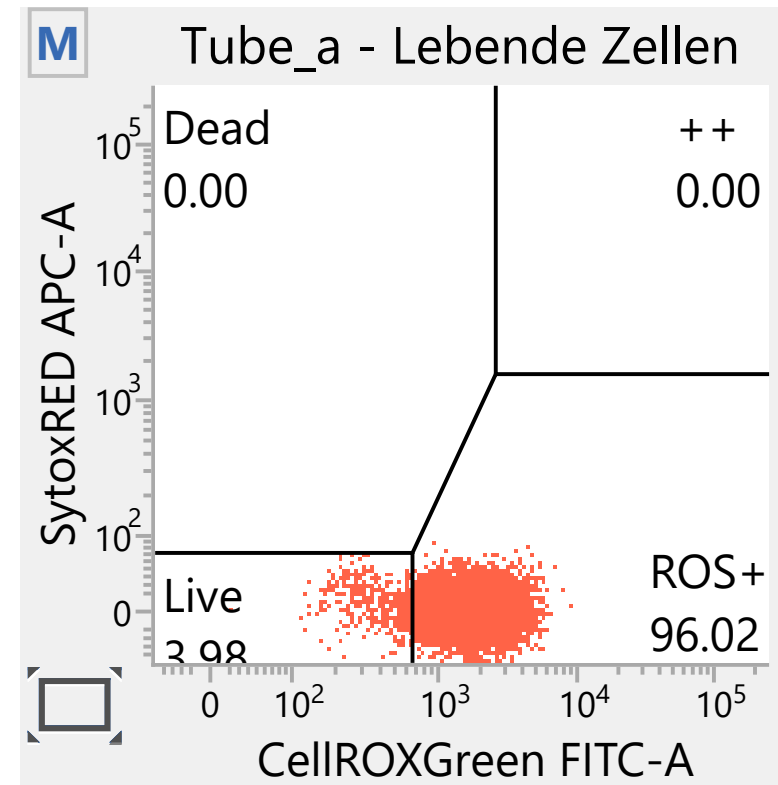

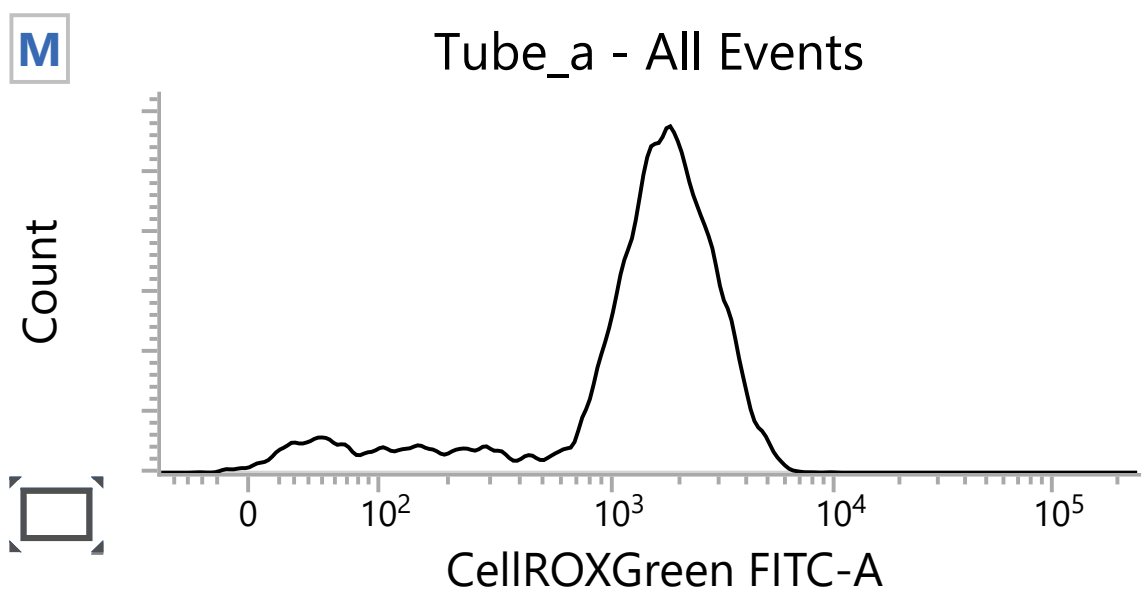

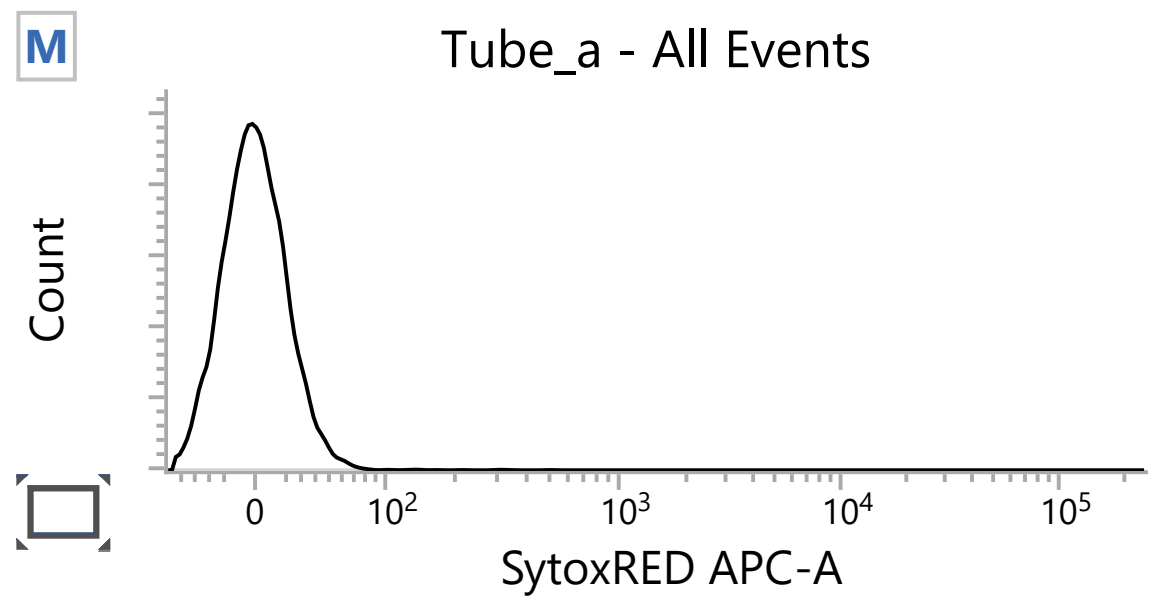

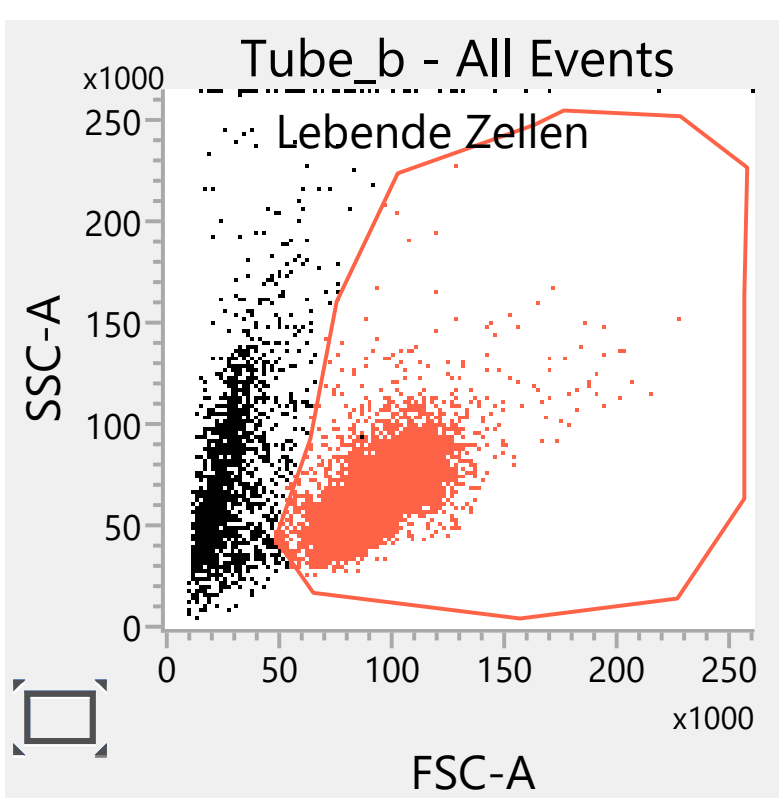

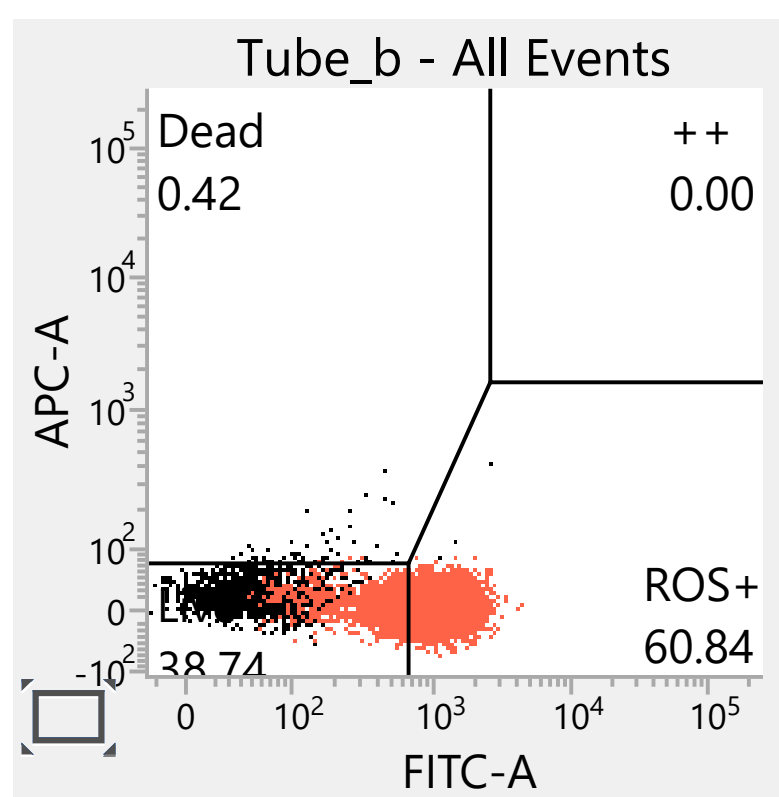

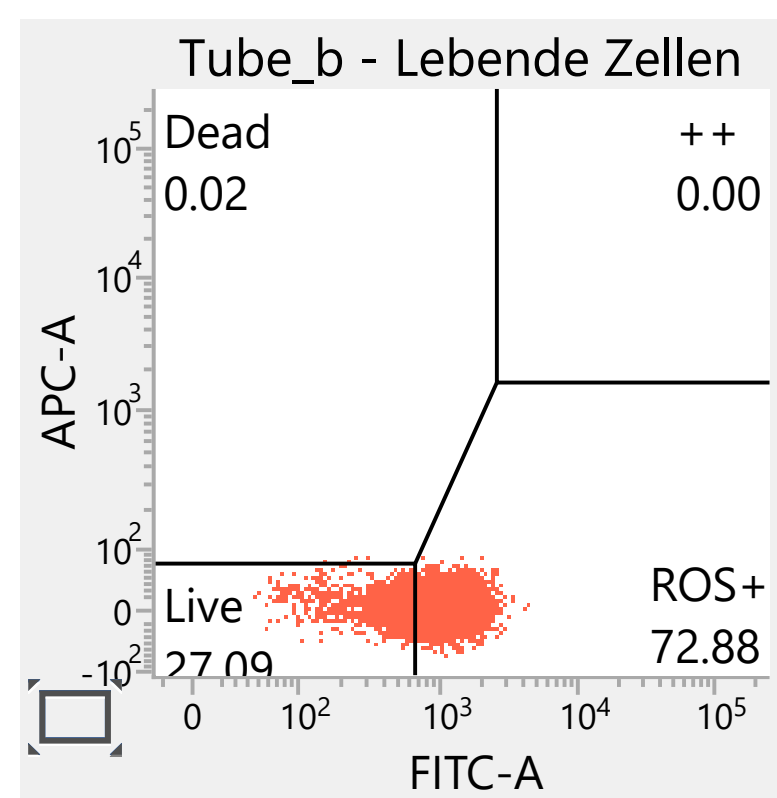

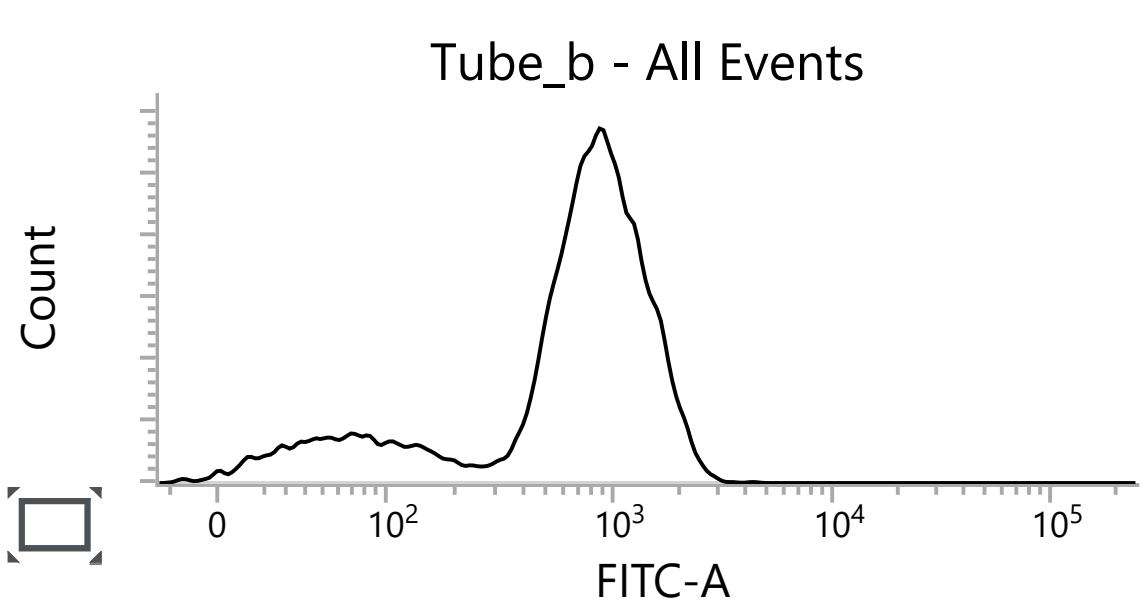

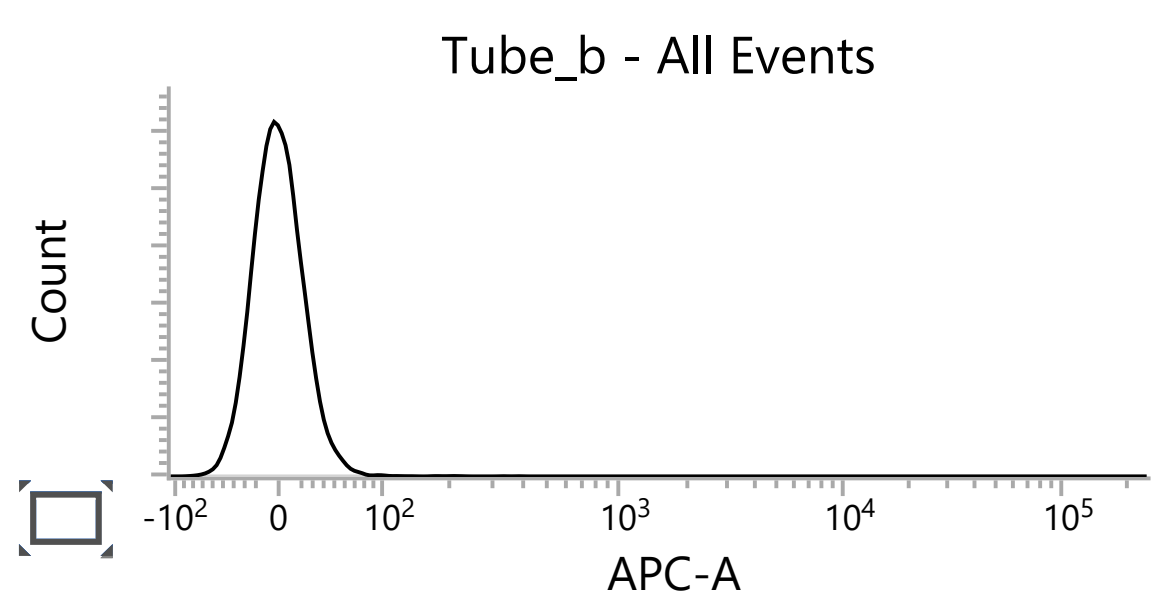

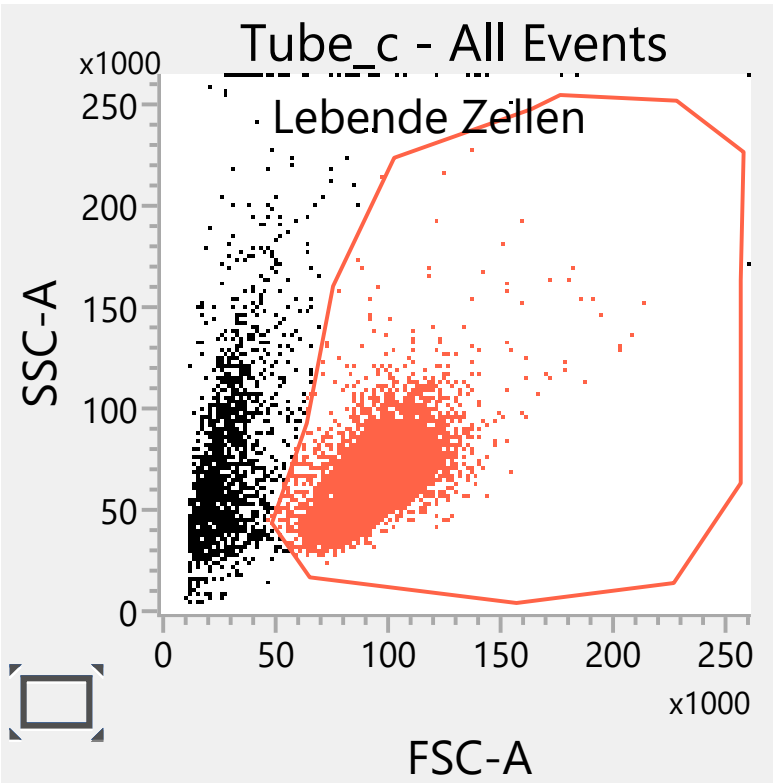

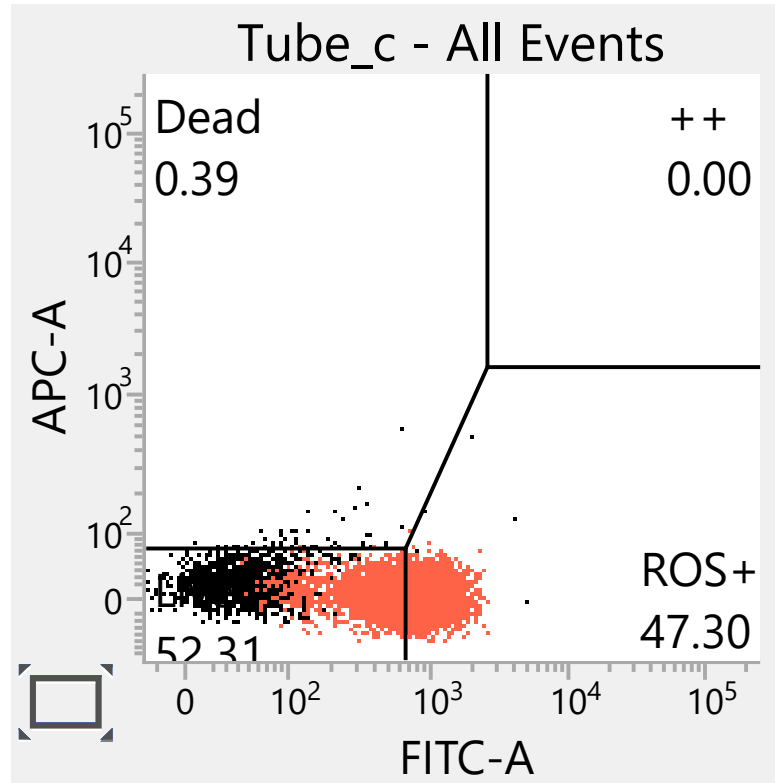

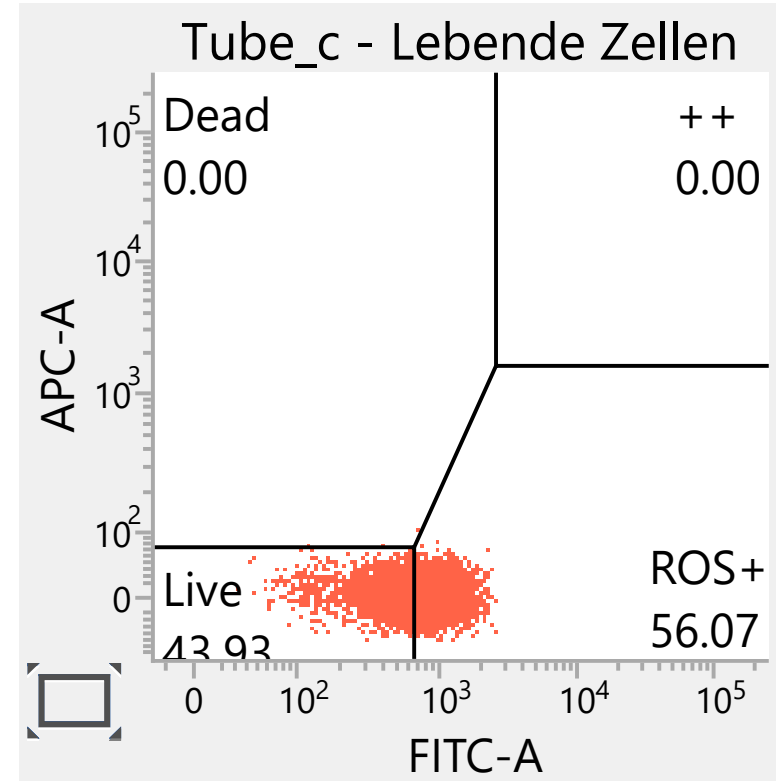

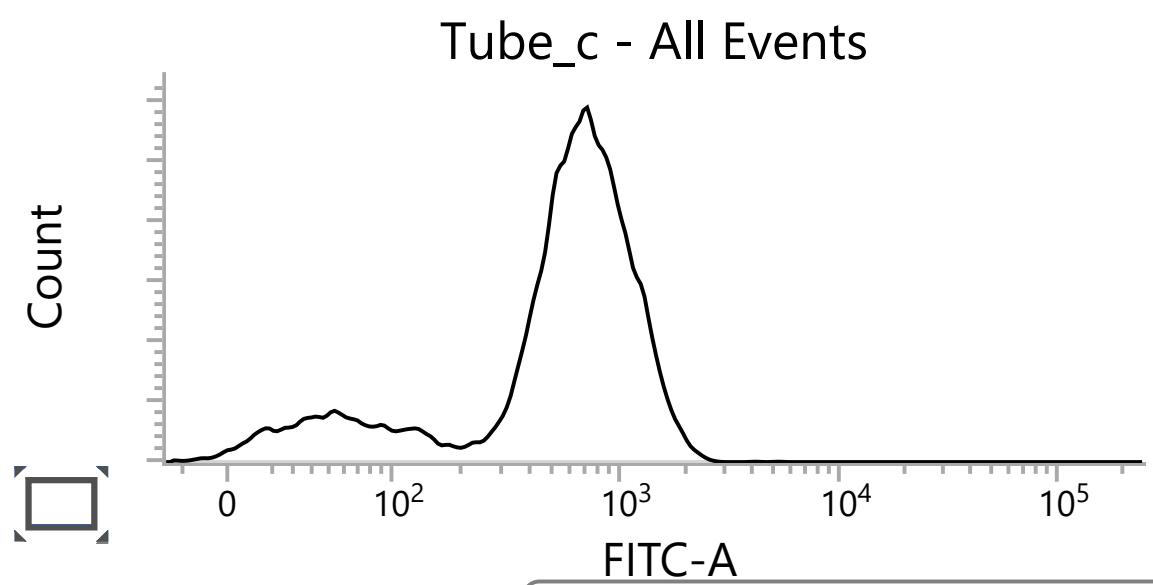

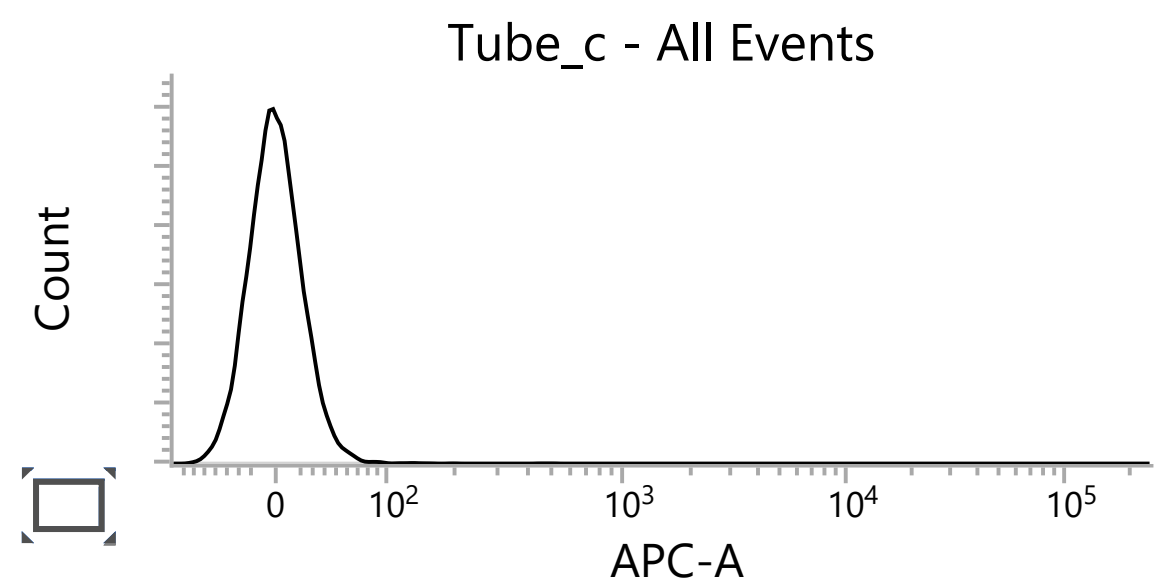

Statistics

Name	Events	% Parent	% Grandparent	% Total
Tube_a:All Events	10,000	***	***	100.00
Tube_b:All Events	10,000	***	***	100.00
Tube_c:All Events	10,000	***	***	100.00
Tube_a:Lebende Zellen	8,733	87.33	***	87.33
Tube_b:Lebende Zellen	8,330	83.30	***	83.30
Tube_c:Lebende Zellen	8,418	84.18	***	84.18

CellROX Assay

Sample ID: N6-ATM_1 NAC

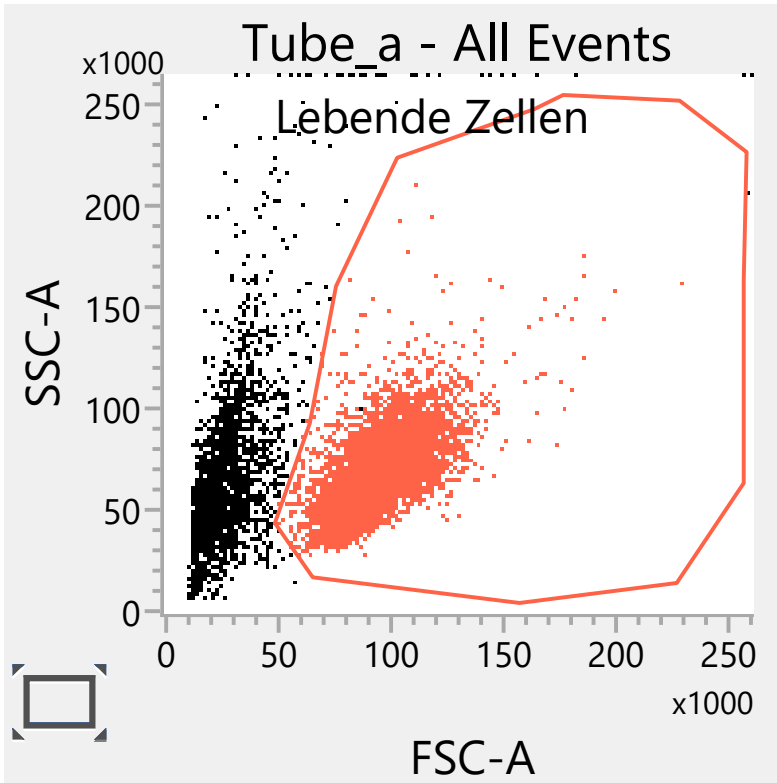

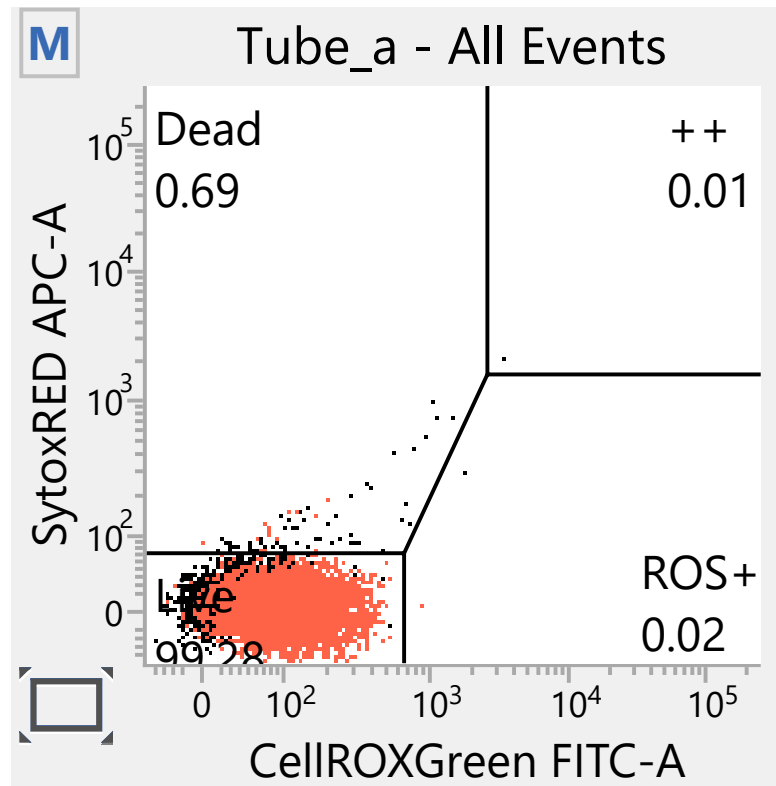

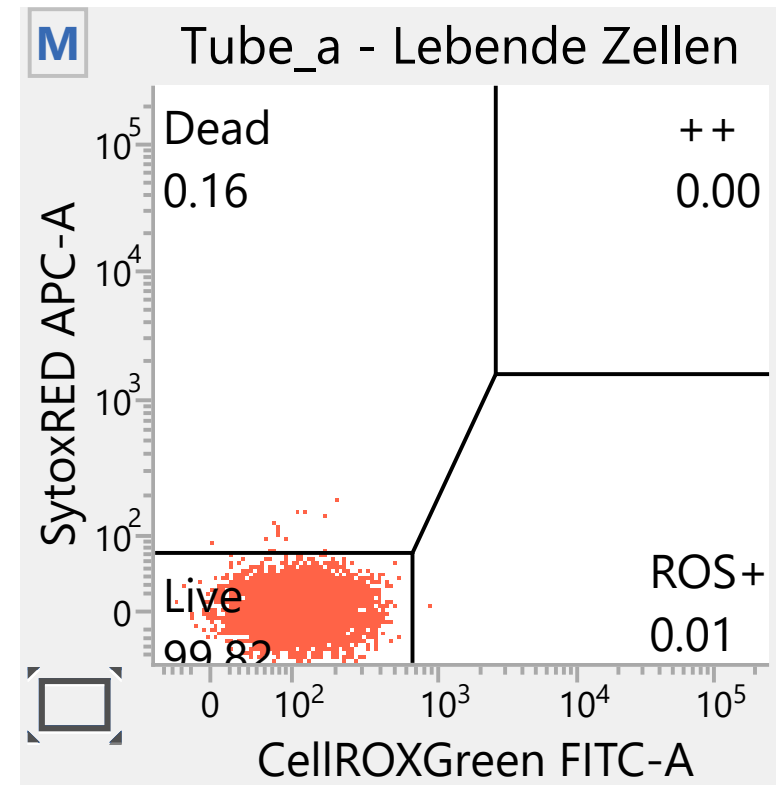

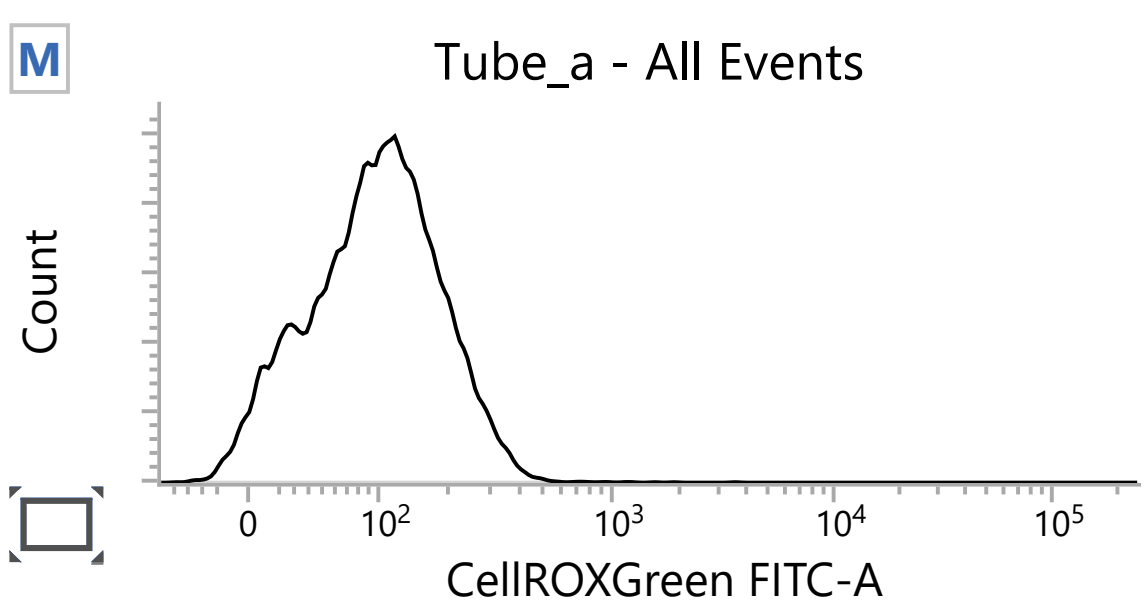

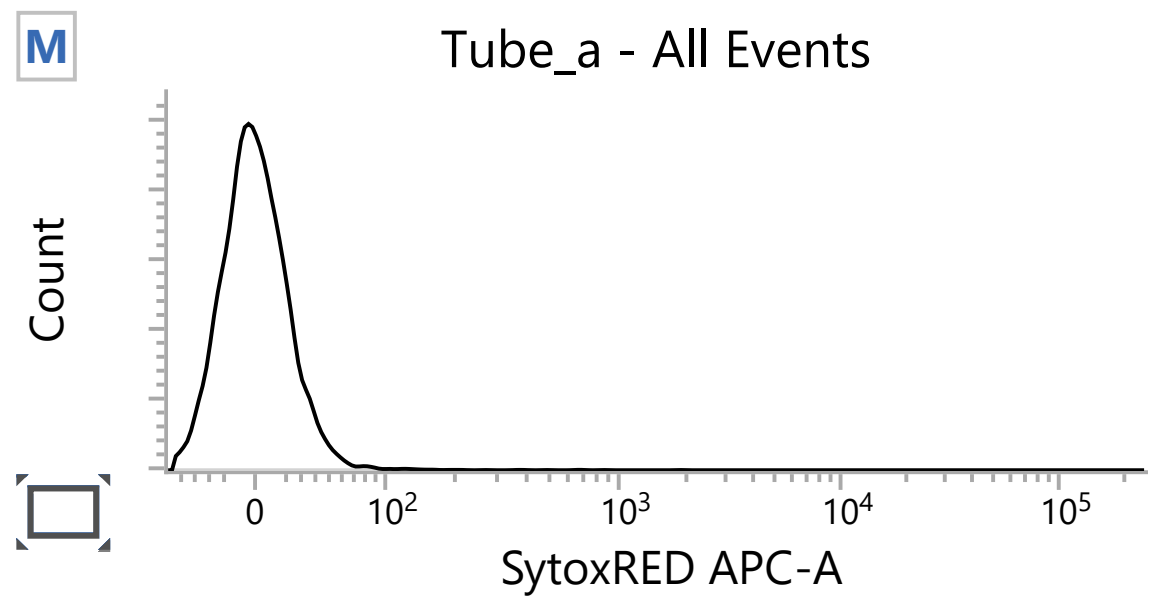

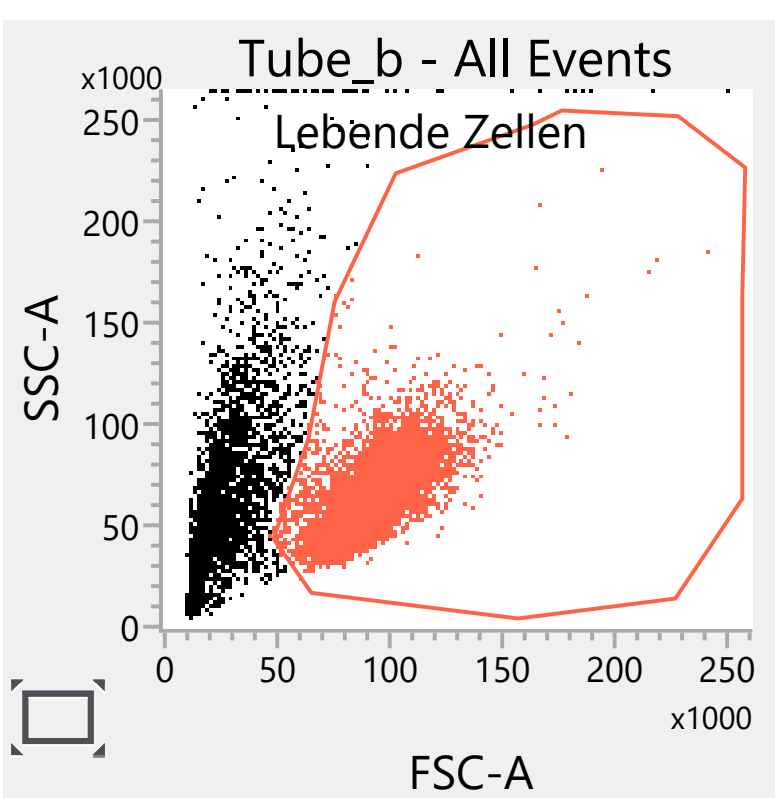

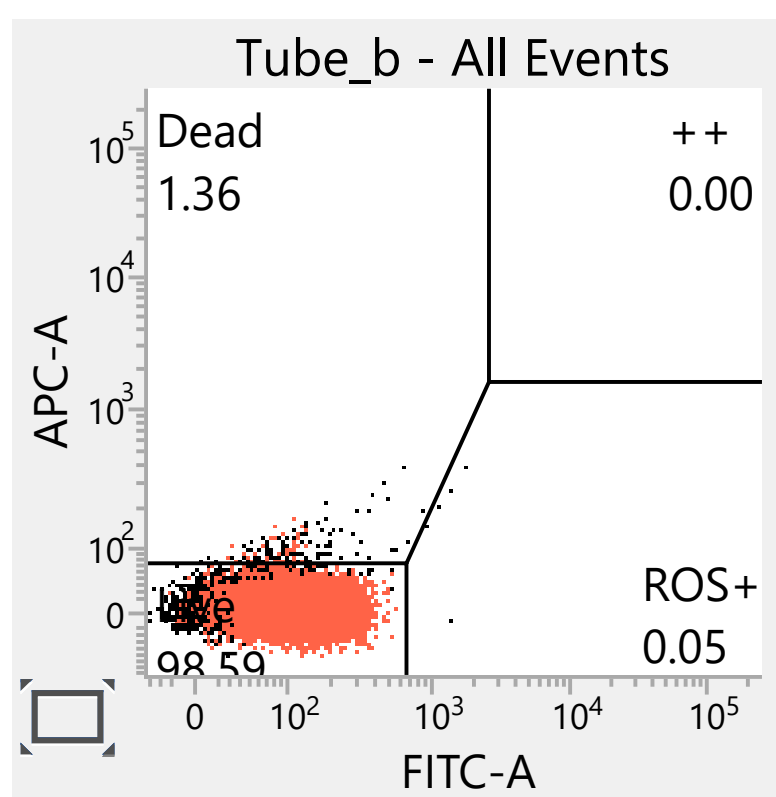

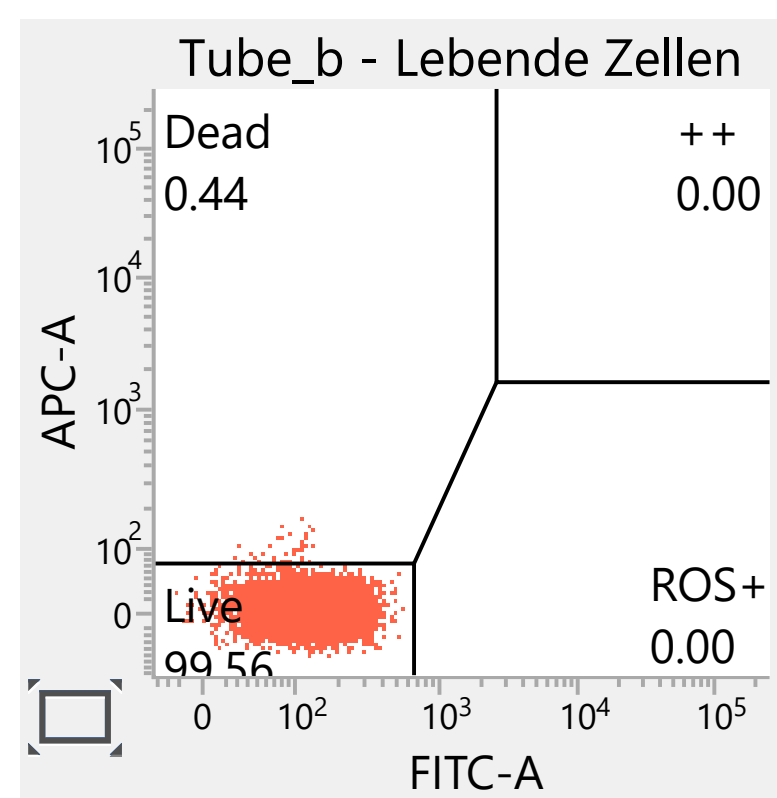

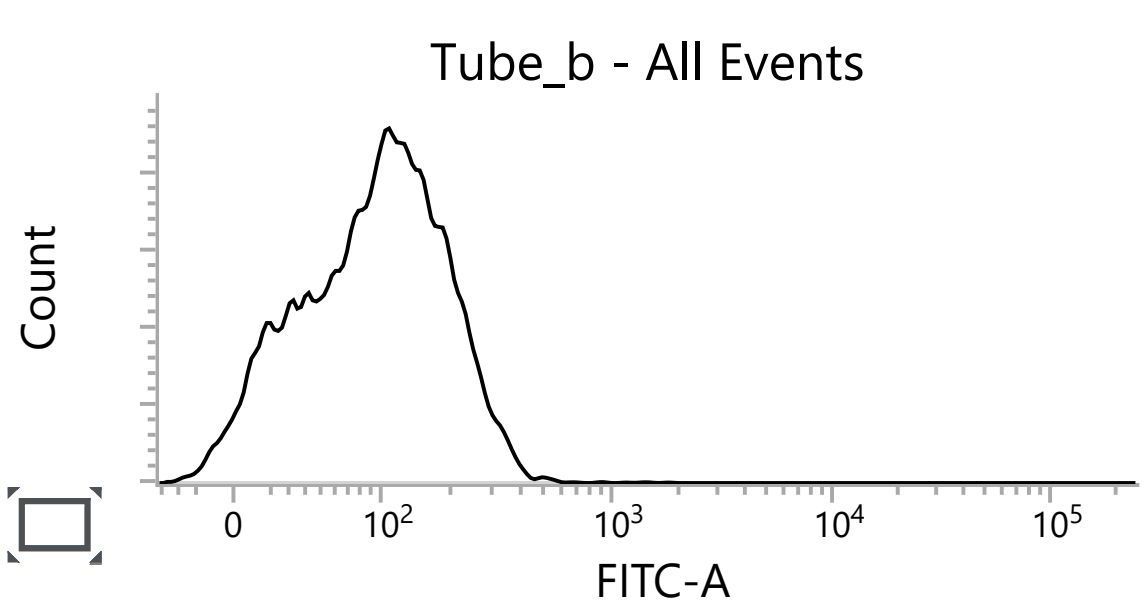

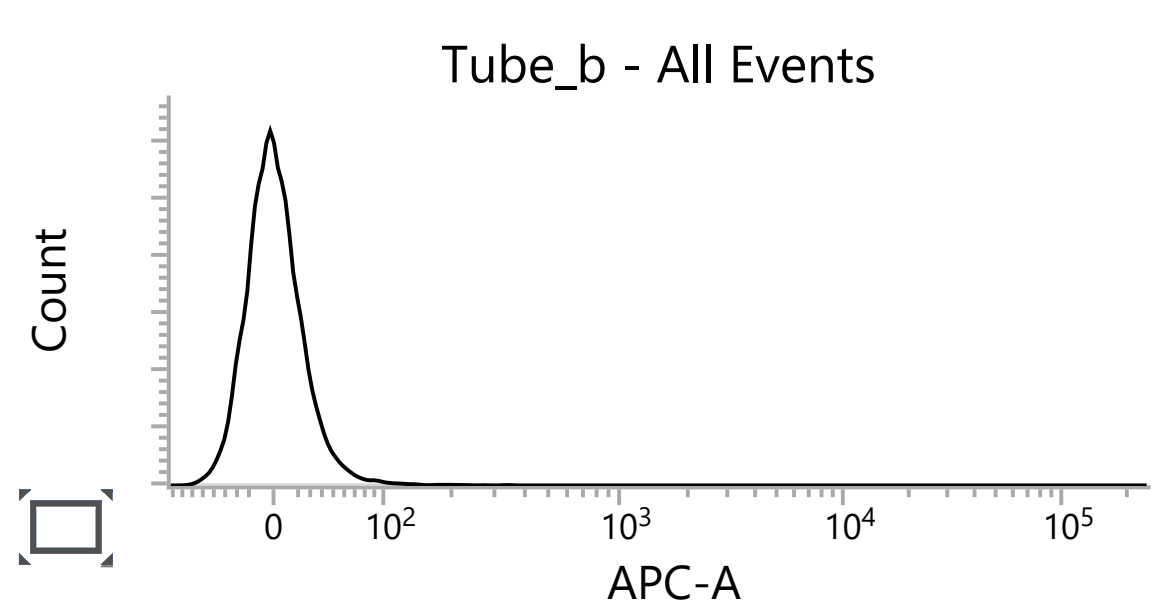

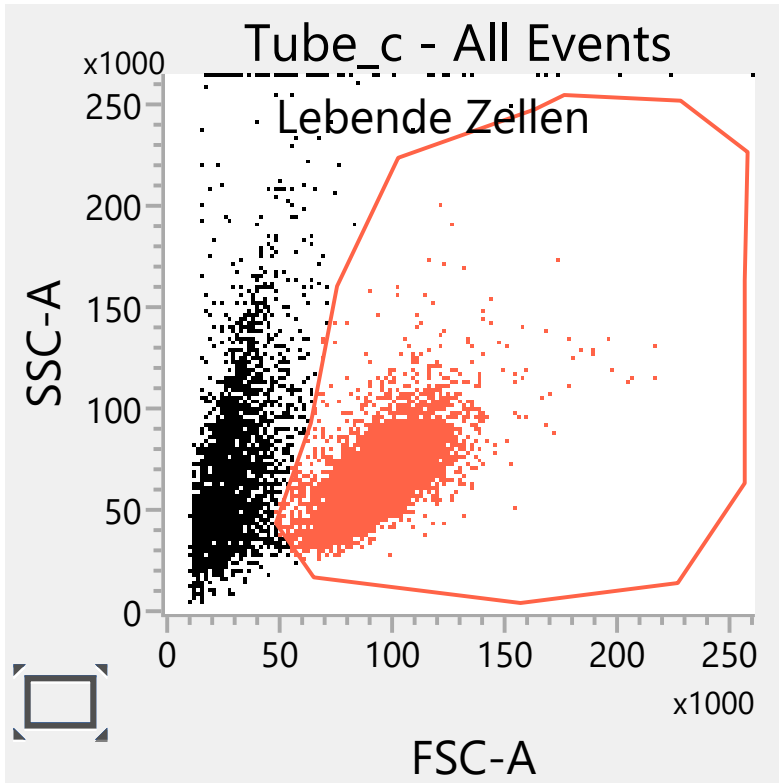

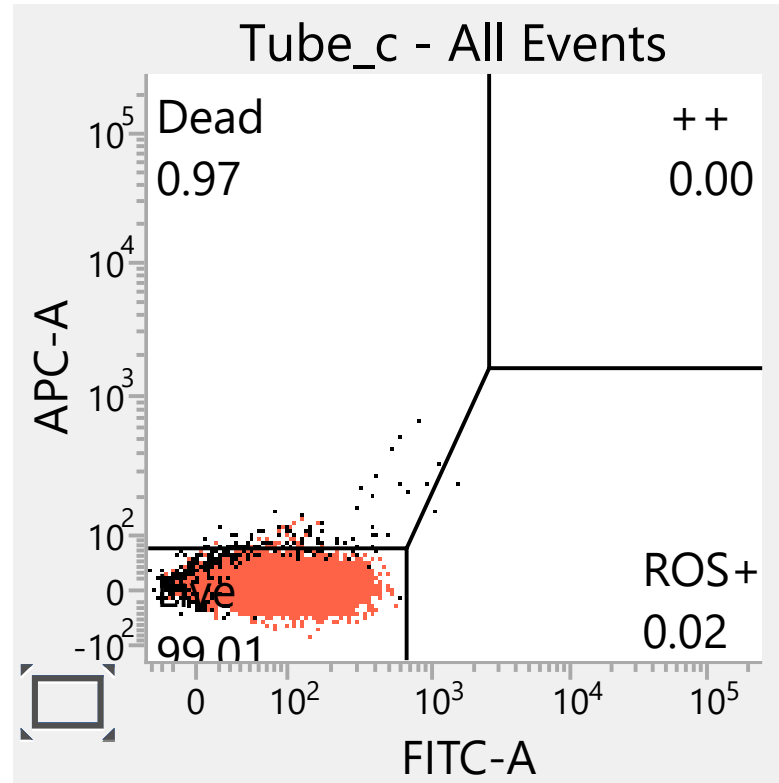

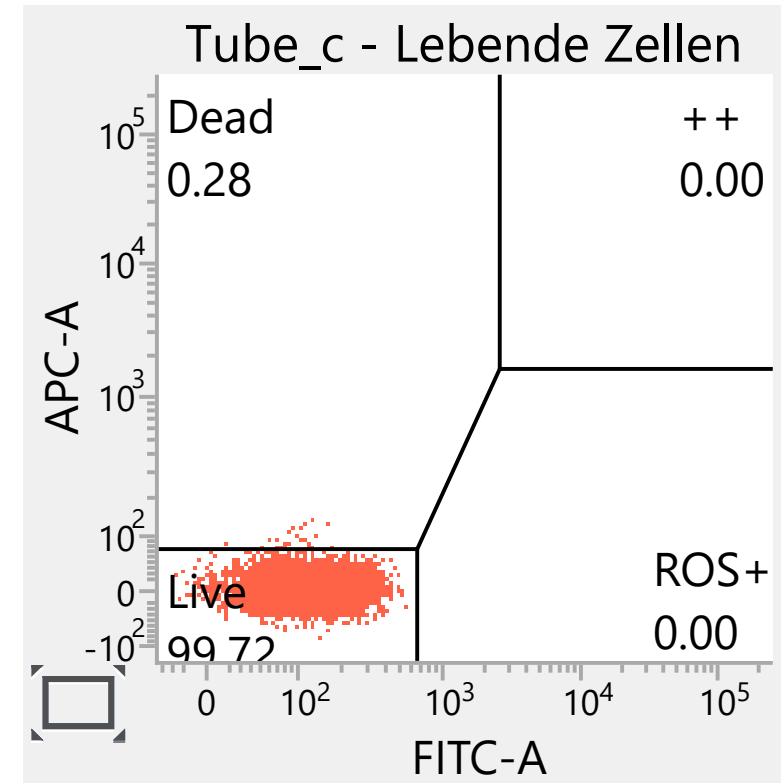

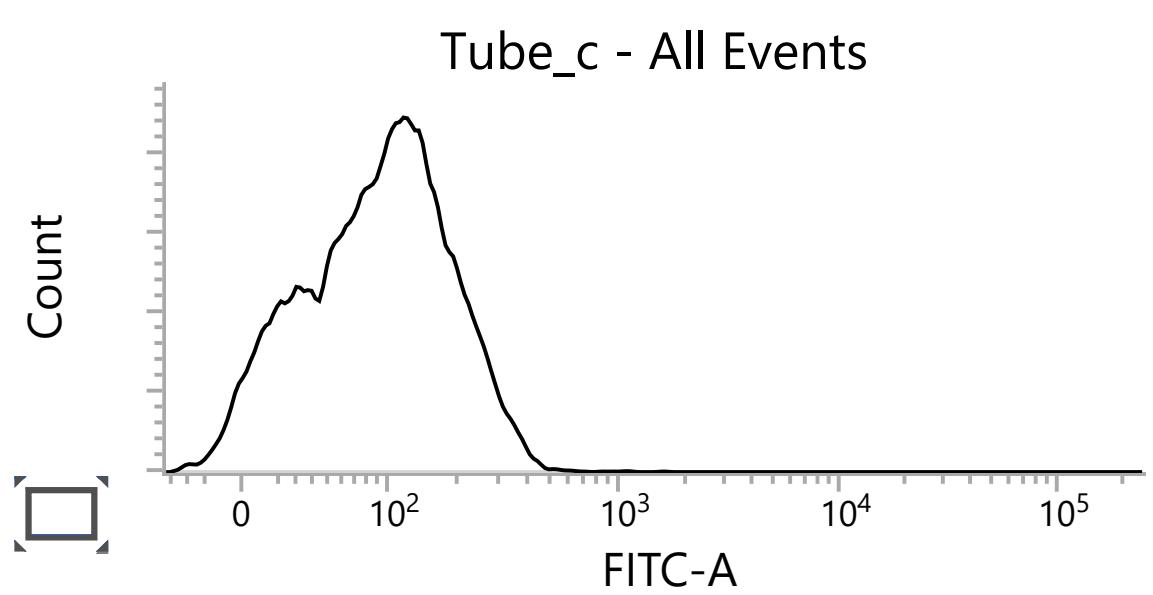

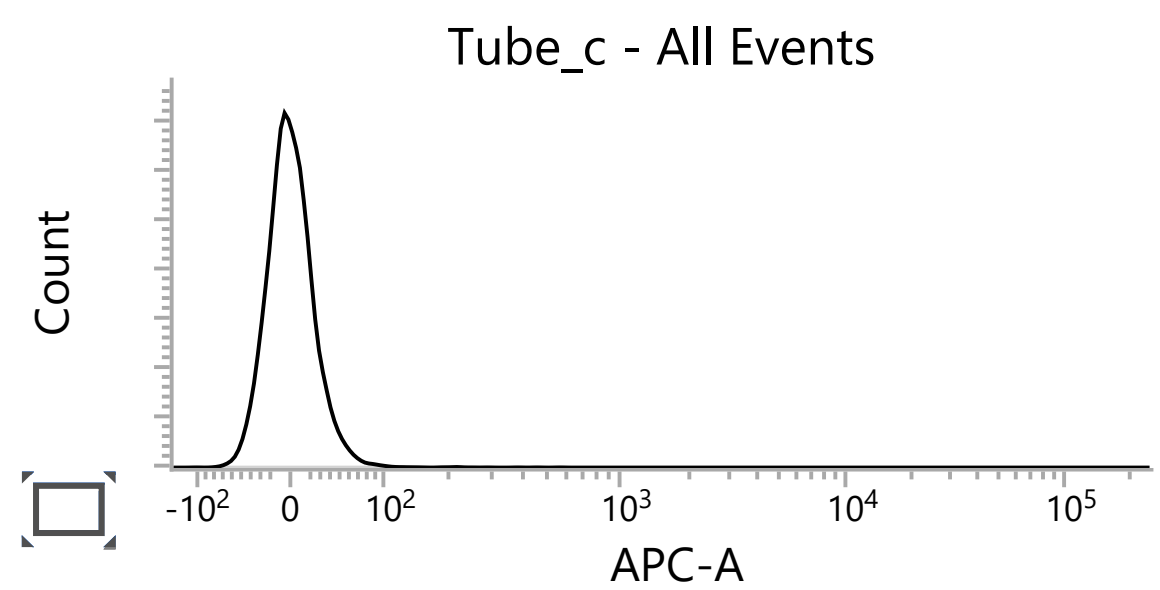

Statistics

Name	Events	% Parent	% Grandparent	% Total
Tube_a:All Events	10,000	***	***	100.00
Tube_b:All Events	10,000	***	***	100.00
Tube_c:All Events	10,000	***	***	100.00
Tube_a:Lebende Zellen	7,940	79.40	***	79.40
Tube_b:Lebende Zellen	7,352	73.52	***	73.52
Tube_c:Lebende Zellen	7,208	72.08	***	72.08
Tube_a:Dead	69	0.69	***	0.69
Tube_a:++	1	0.01	***	0.01
Tube_a:Live	9,928	99.28	***	99.28
Tube_a:ROS+	2	0.02	***	0.02
Tube_a:Dead	13	0.16	0.13	0.13
Tube_a:++	0	0.00	0.00	0.00
Tube_a:Live	7,926	99.82	79.26	79.26
Tube_a:ROS+	1	0.01	0.01	0.01
Tube_b:Dead	136	1.36	***	1.36
Tube_b:++	0	0.00	***	0.00
Tube_b:Live	9,859	98.59	***	98.59
Tube_b:ROS+	5	0.05	***	0.05
Tube_b:Dead	32	0.44	0.32	0.32
Tube_b:++	0	0.00	0.00	0.00
Tube_b:Live	7,320	99.56	73.20	73.20
Tube_b:ROS+	0	0.00	0.00	0.00
Tube_c:Dead	97	0.97	***	0.97
Tube_c:++	0	0.00	***	0.00
Tube_c:Live	9,901	99.01	***	99.01
Tube_c:ROS+	2	0.02	***	0.02
Tube_c:Dead	20	0.28	0.20	0.20
Tube_c:++	0	0.00	0.00	0.00
Tube_c:Live	7,188	99.72	71.88	71.88
Tube_c:ROS+	0	0.00	0.00	0.00

LDT

CellROX Assay

Sample ID: N6-ATM_1

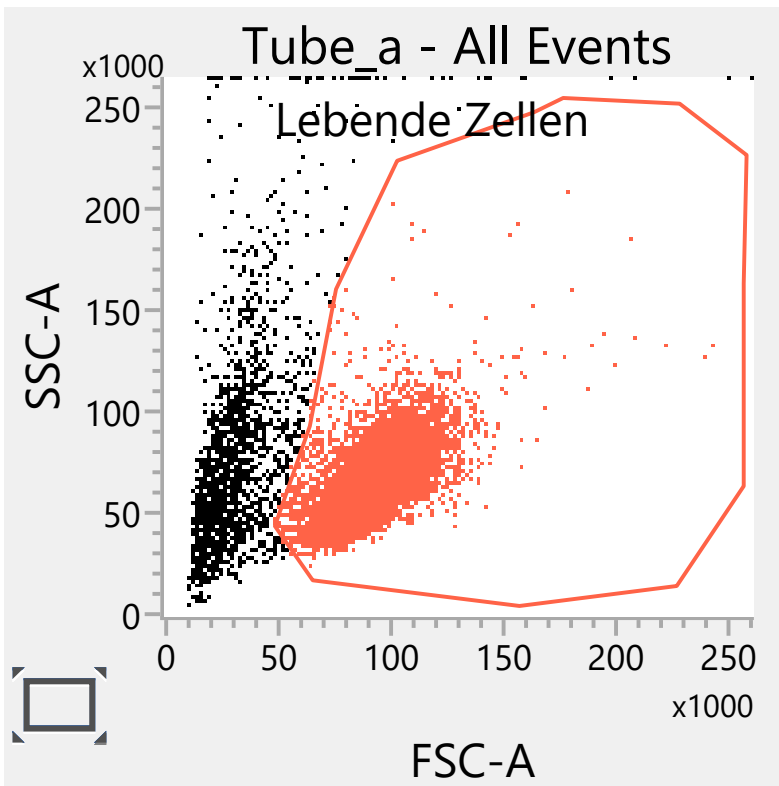

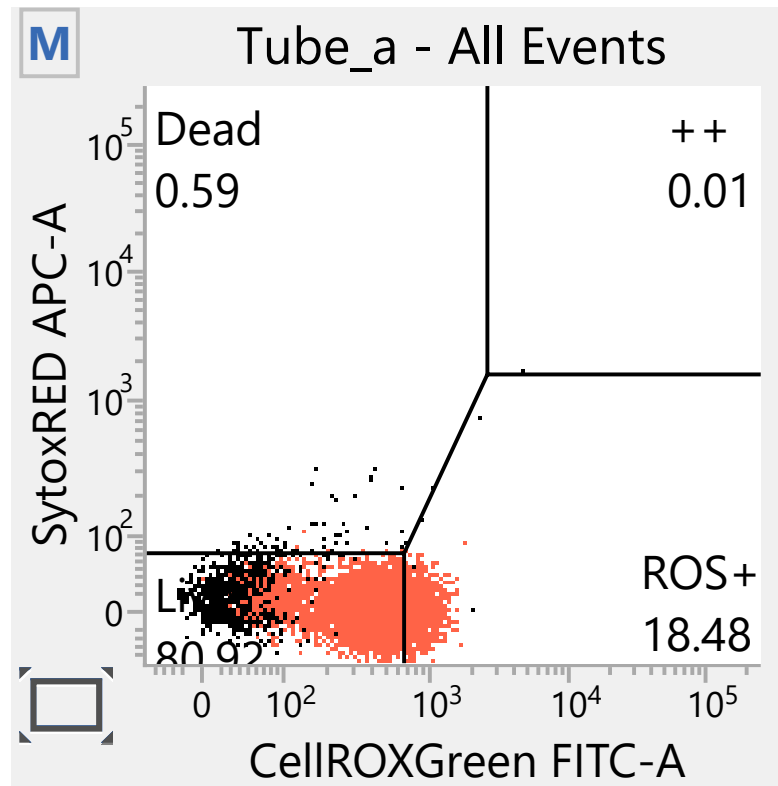

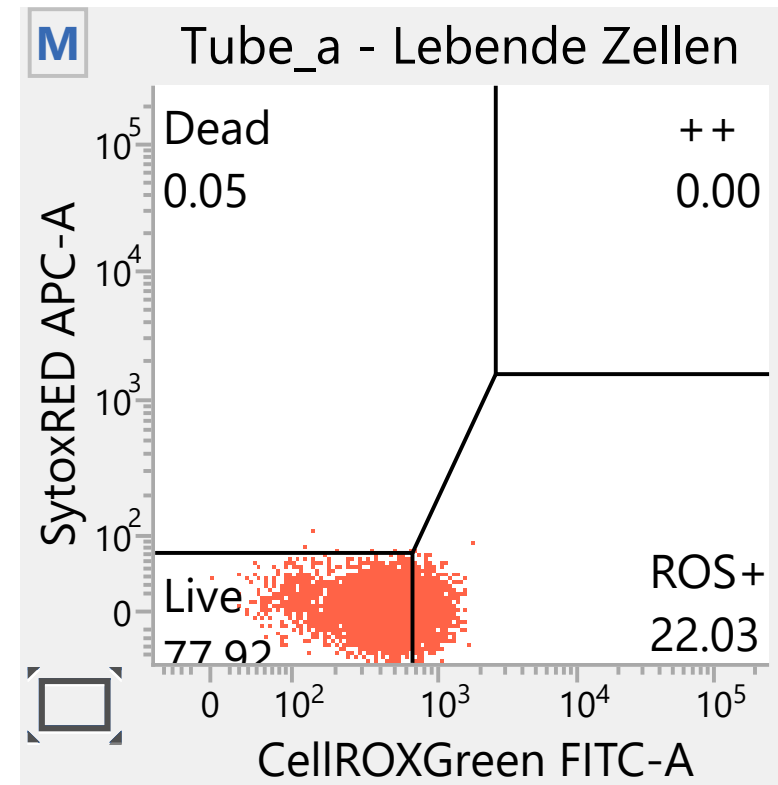

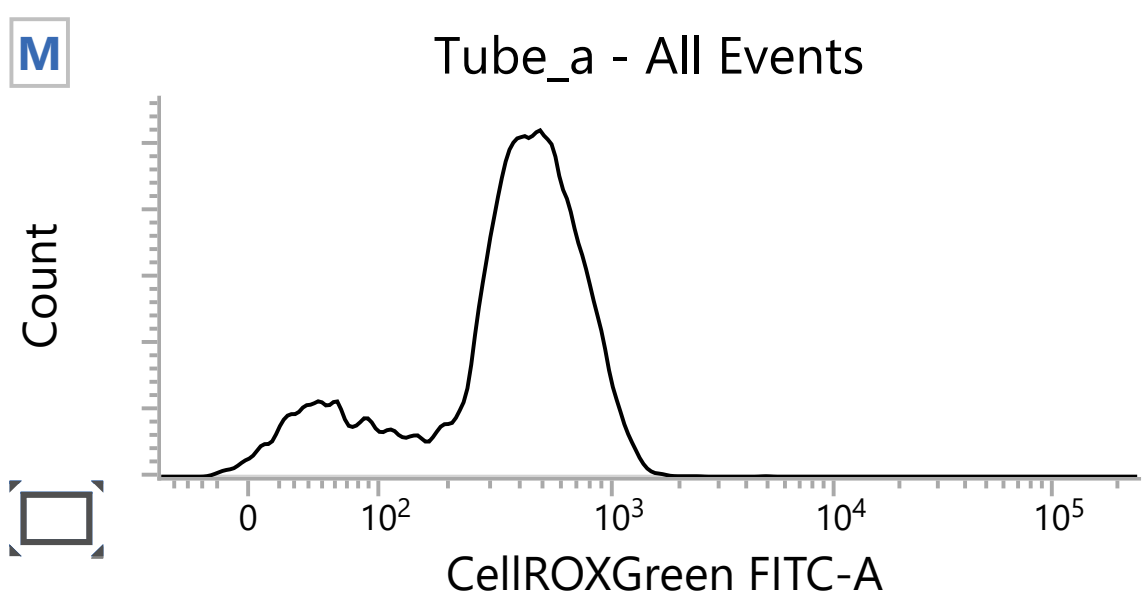

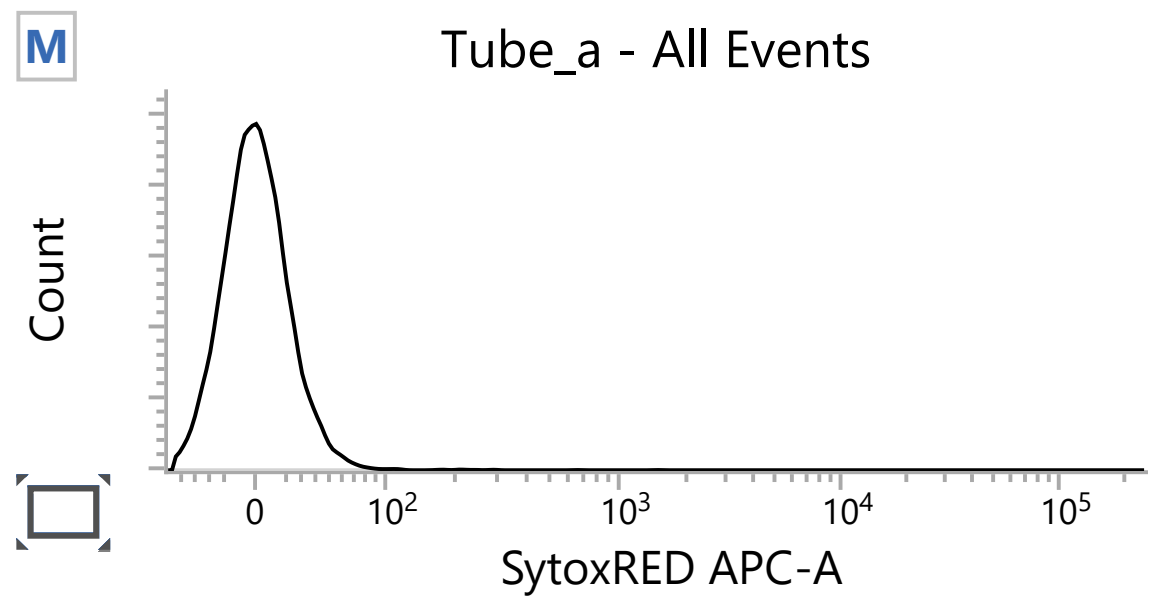

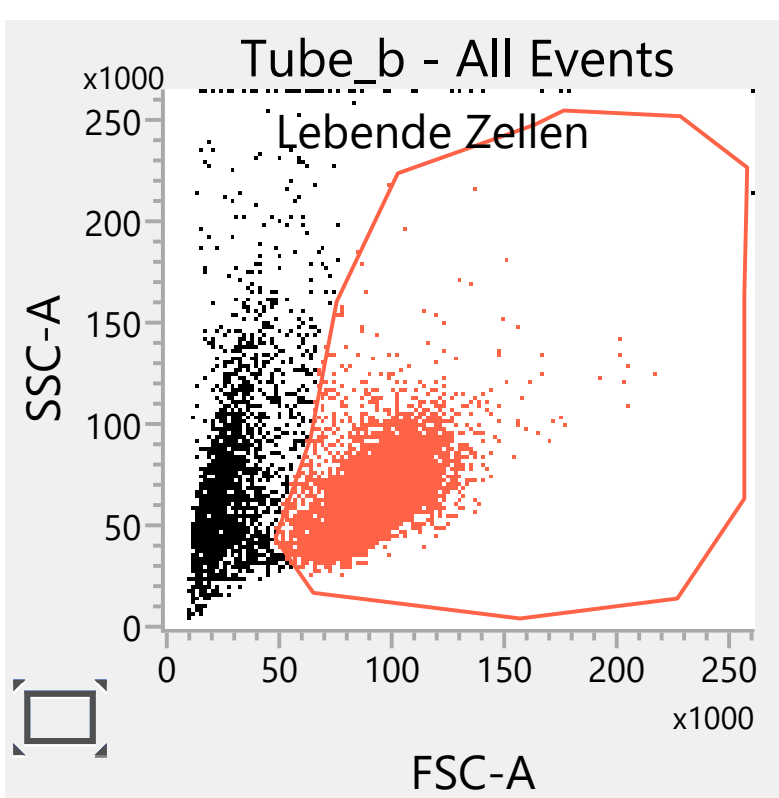

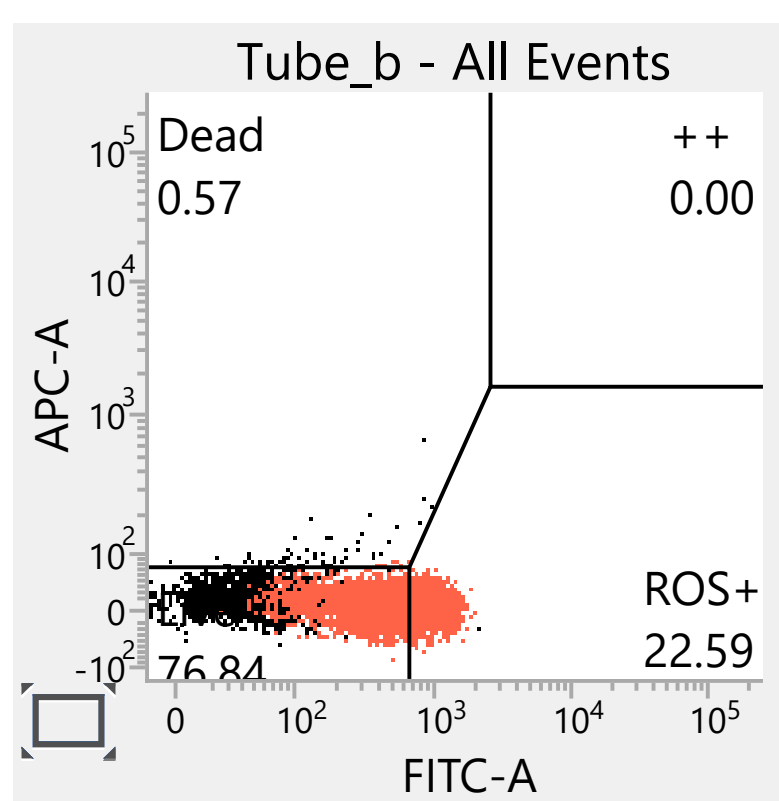

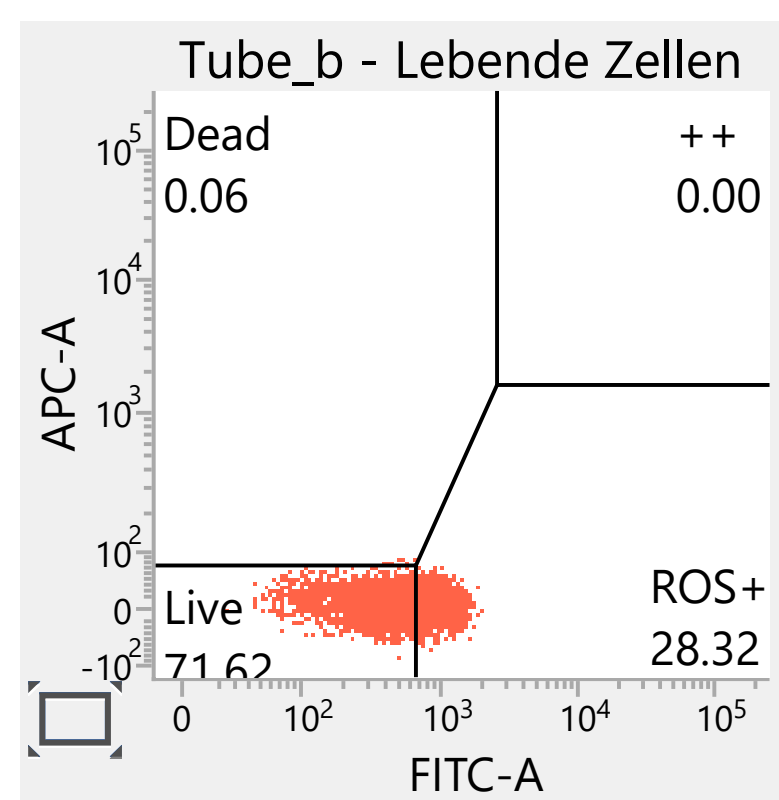

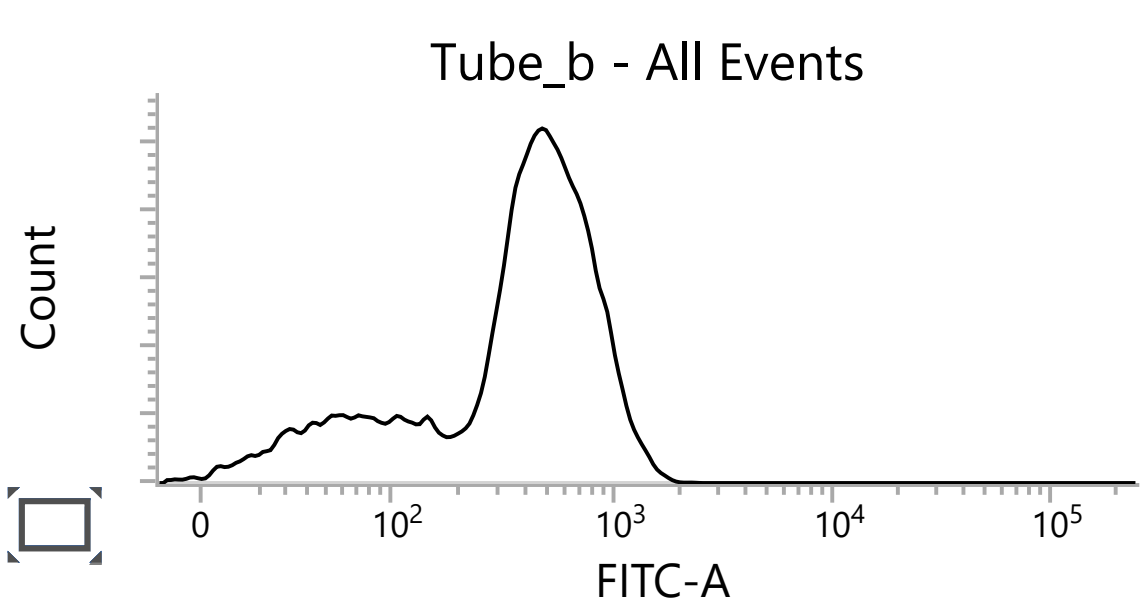

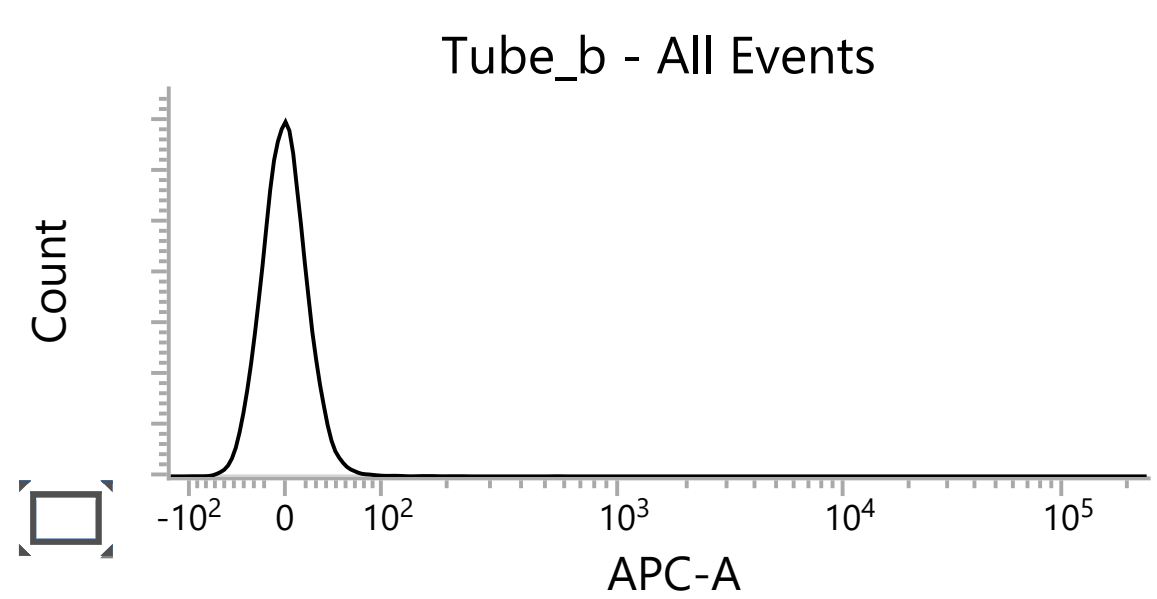

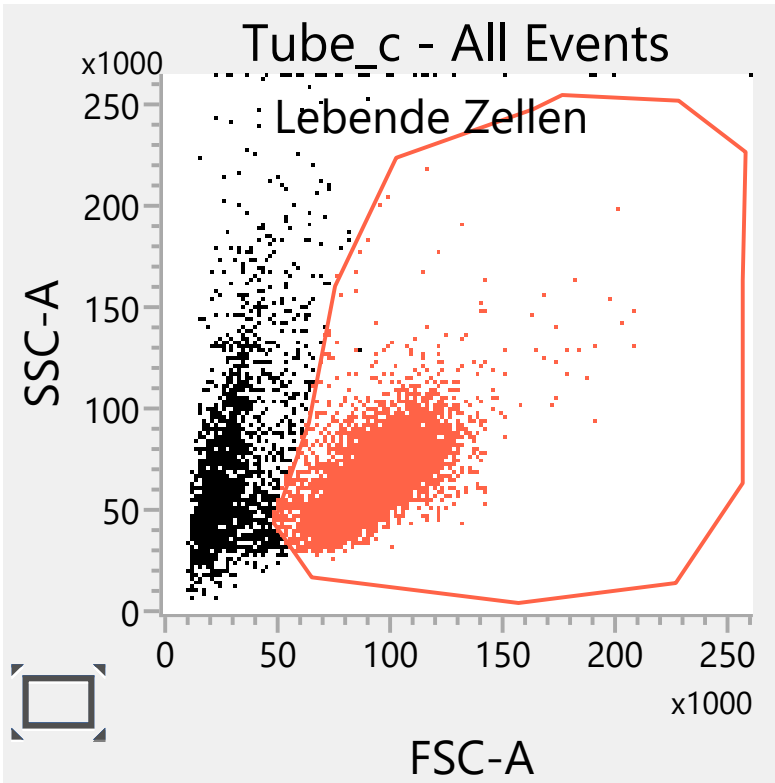

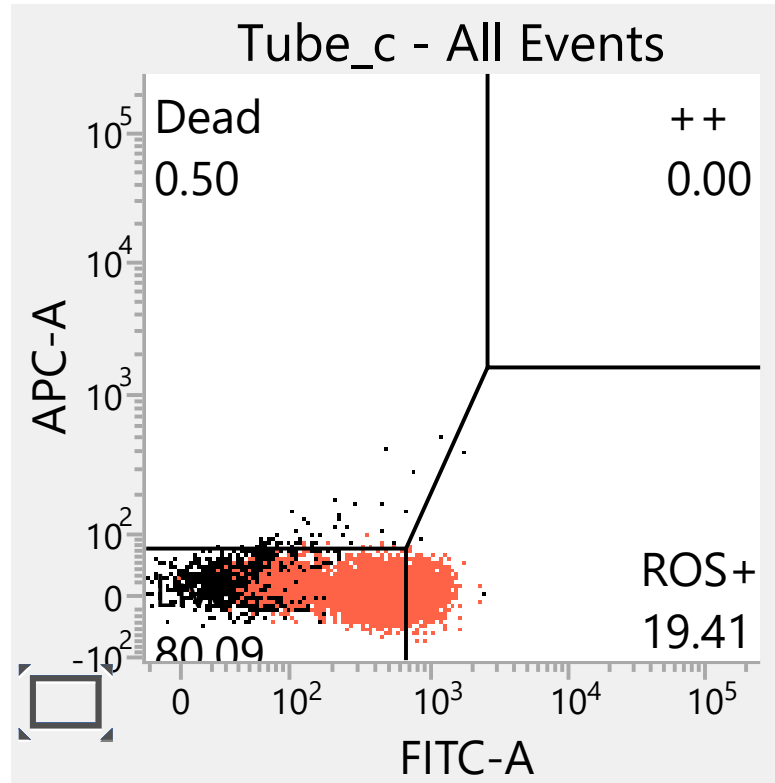

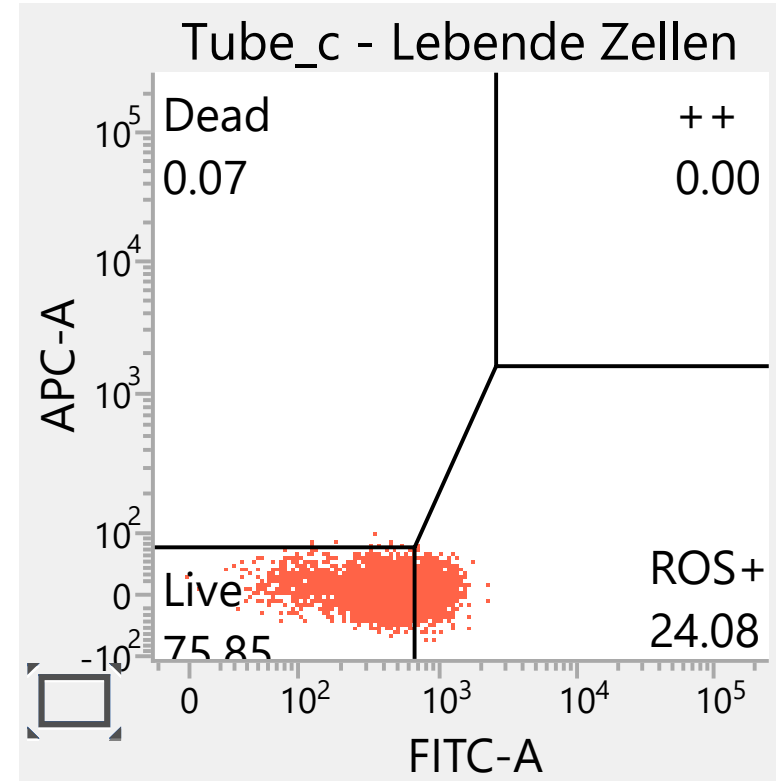

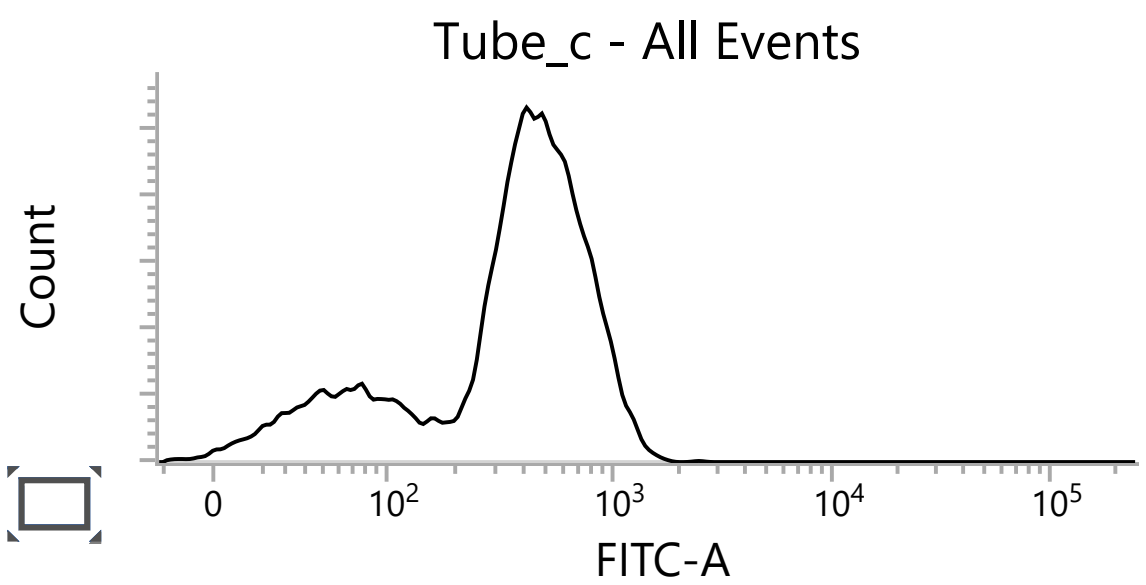

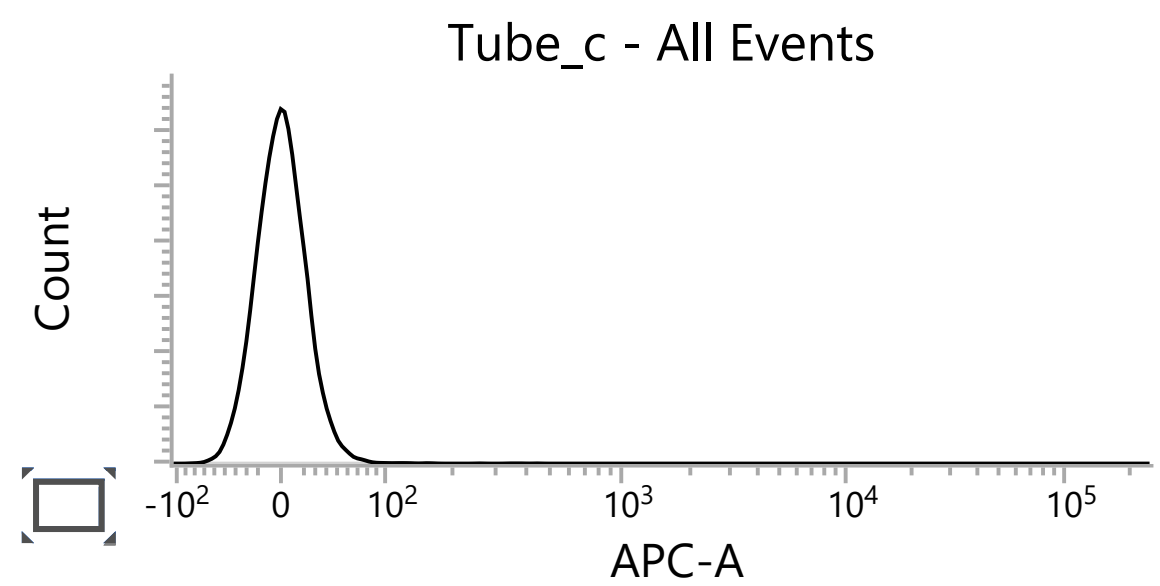

Statistics

Name	Events	% Parent	% Grandparent	% Total
Tube_a:All Events	10,000	***	***	100.00
Tube_b:All Events	10,000	***	***	100.00
Tube_c:All Events	10,000	***	***	100.00
Tube_a:Lebende Zellen	8,355	83.55	***	83.55
Tube_b:Lebende Zellen	7,971	79.71	***	79.71
Tube_c:Lebende Zellen	8,045	80.45	***	80.45

CellROX Assay

Sample ID: N6-ATM_2,5 NAC

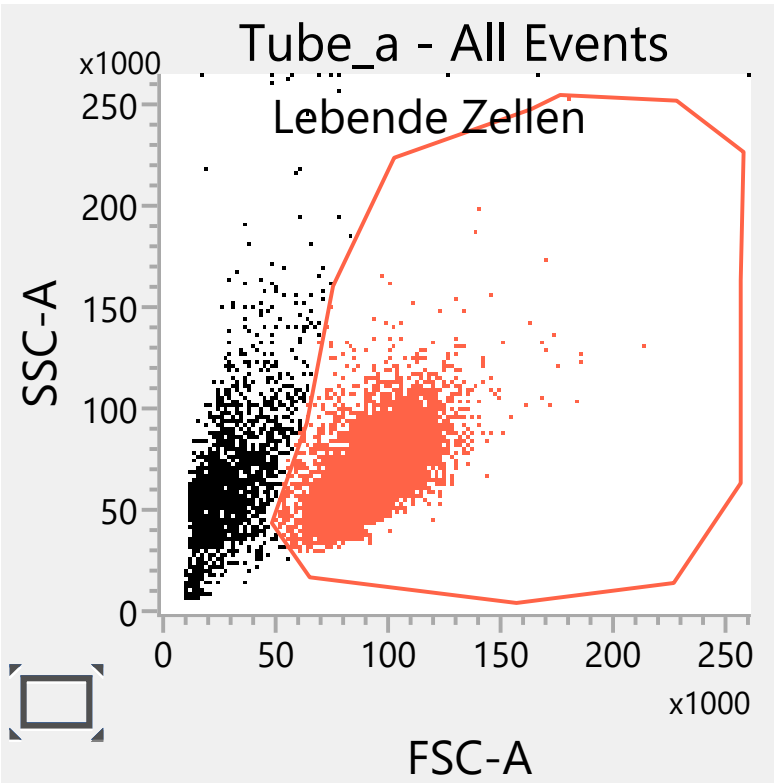

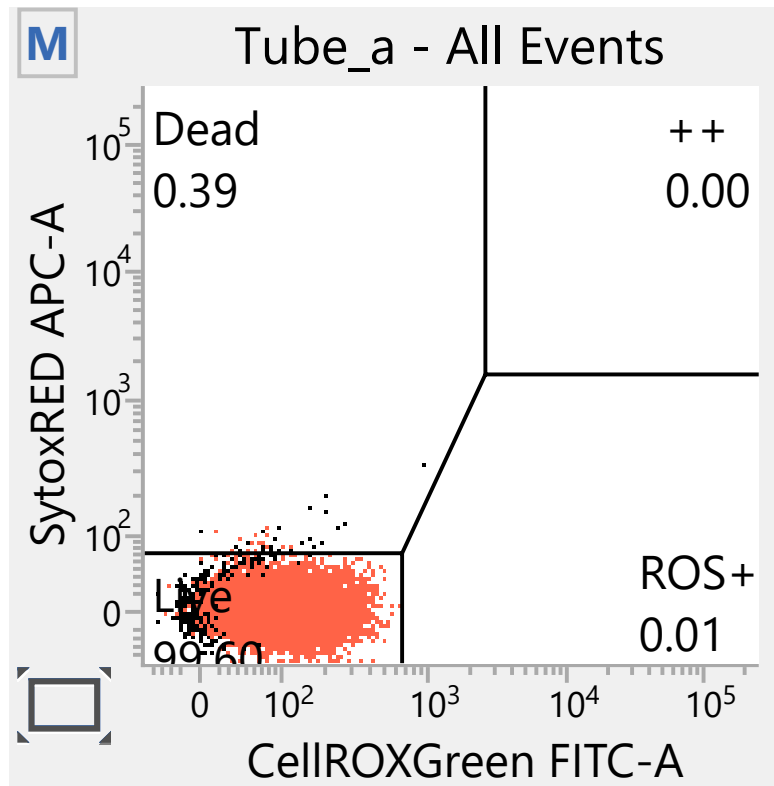

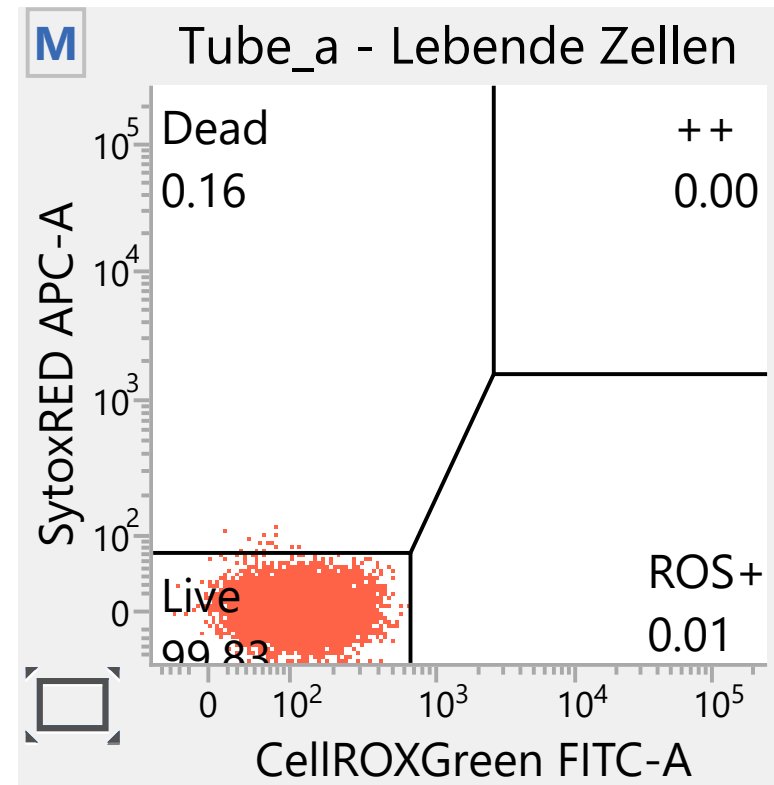

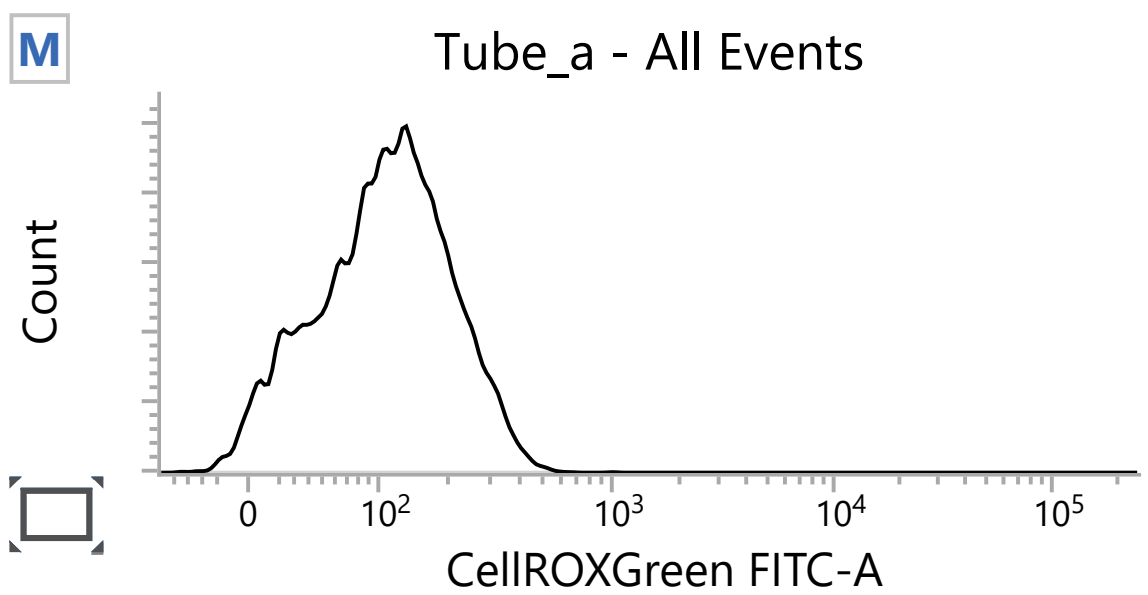

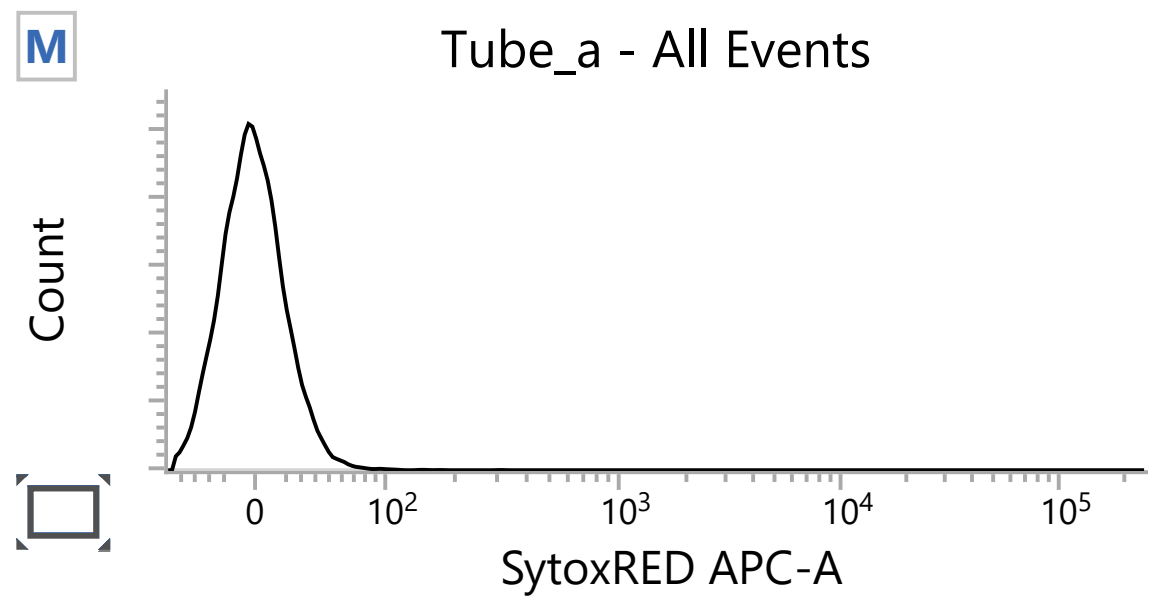

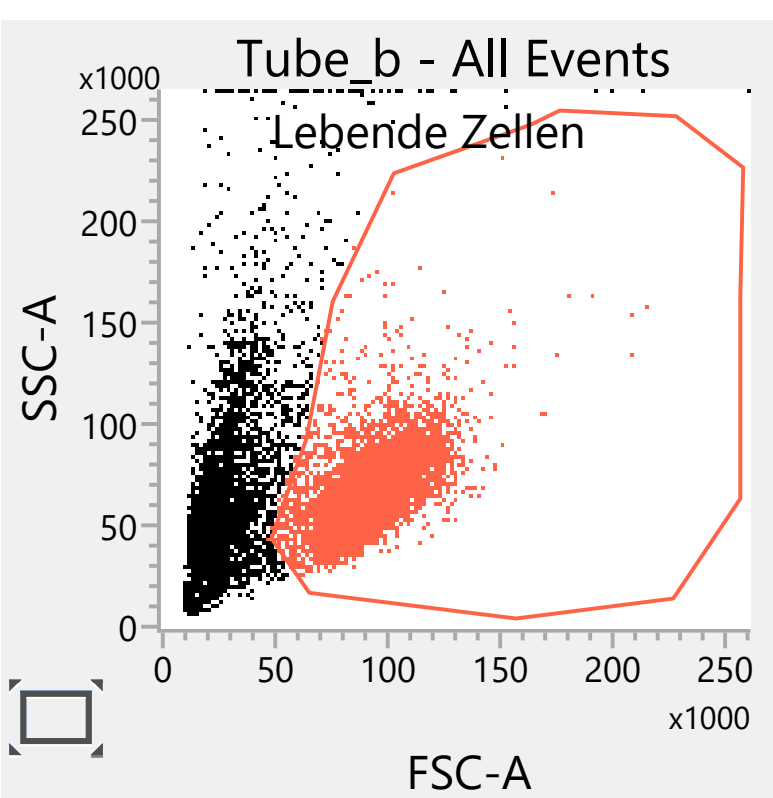

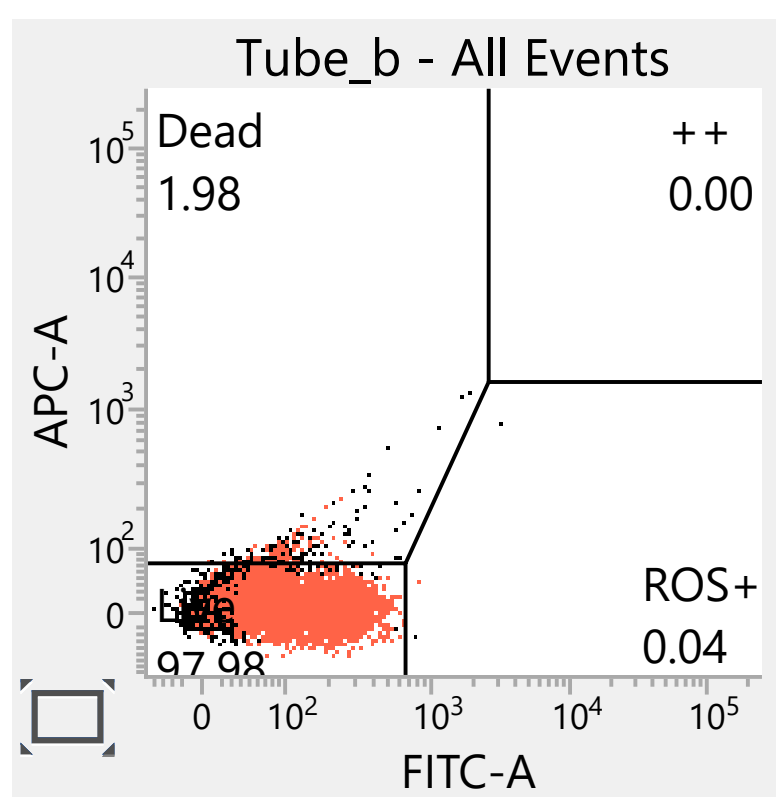

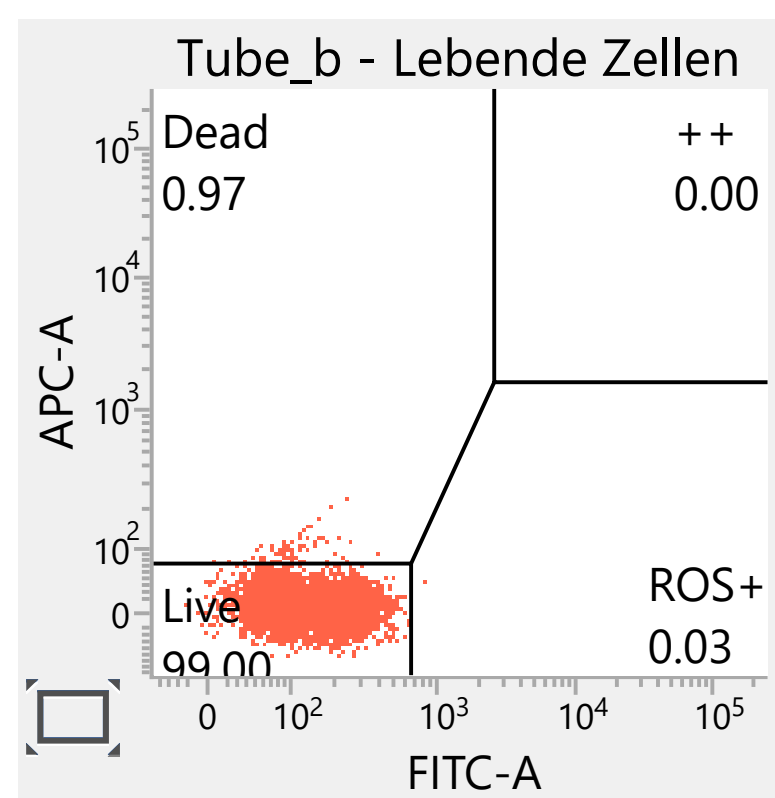

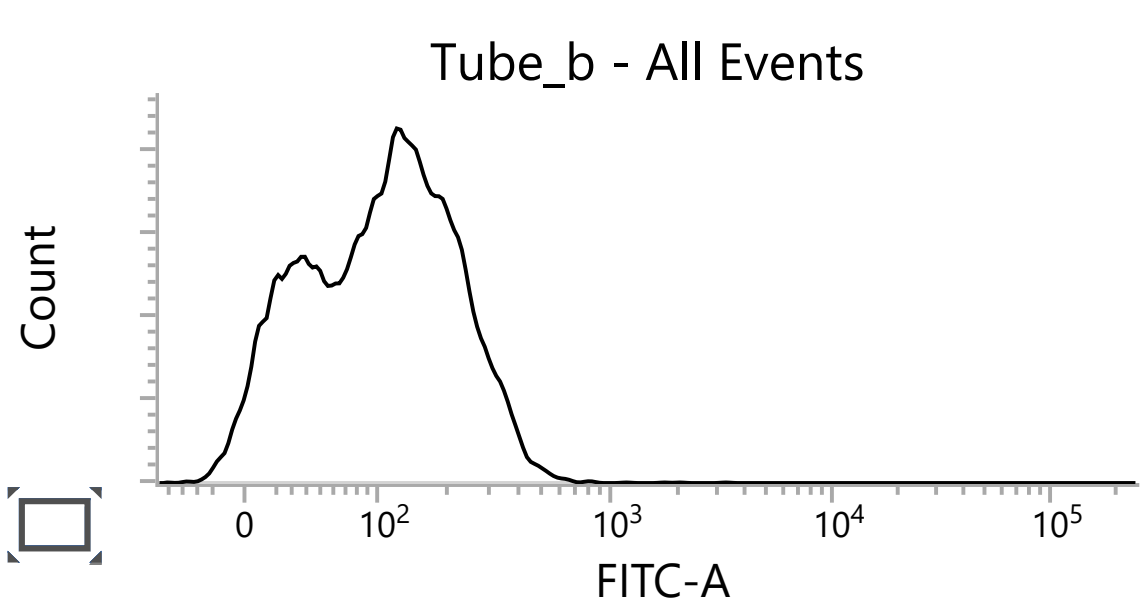

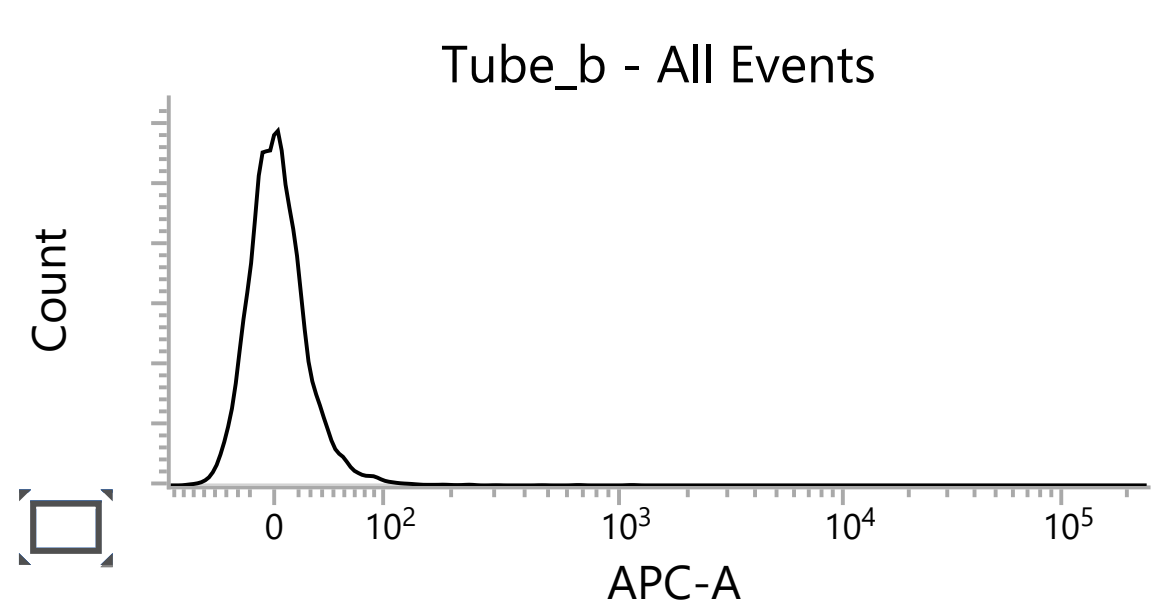

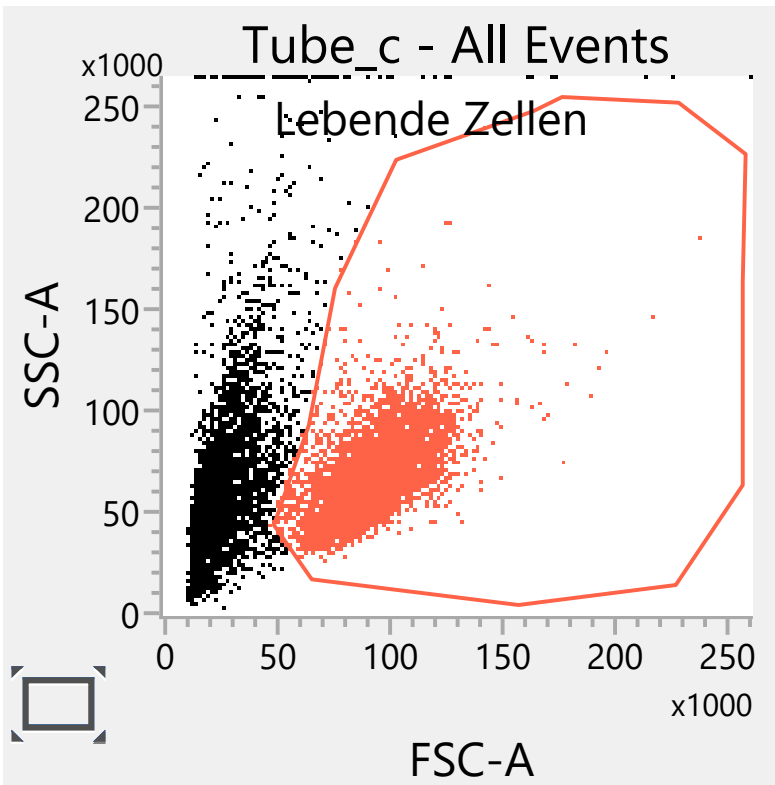

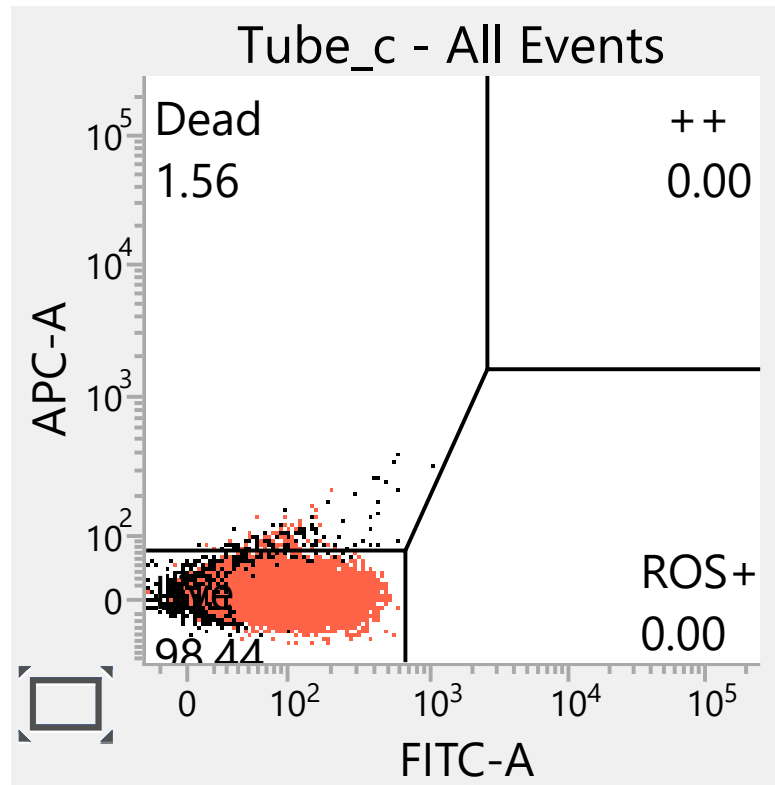

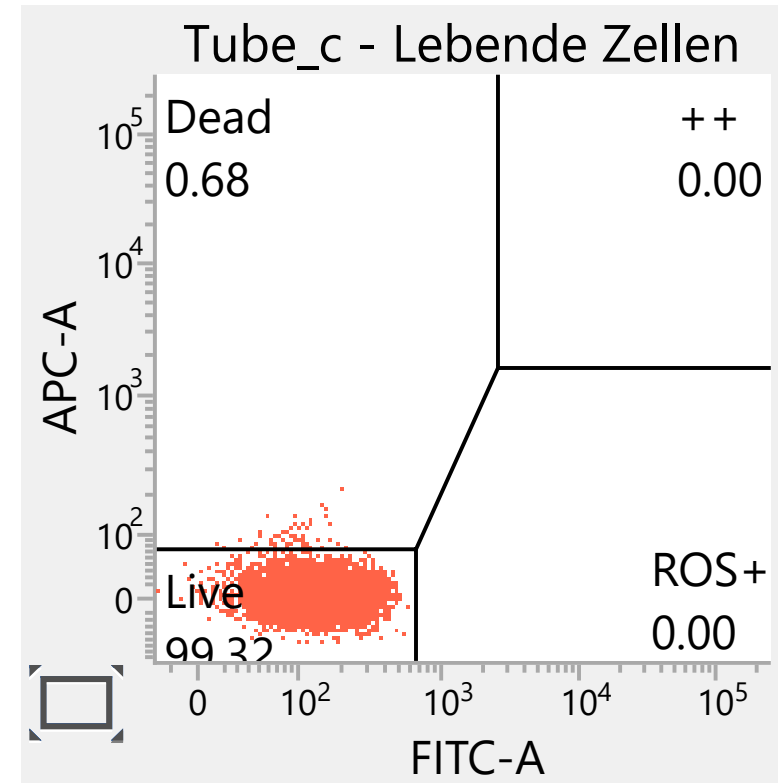

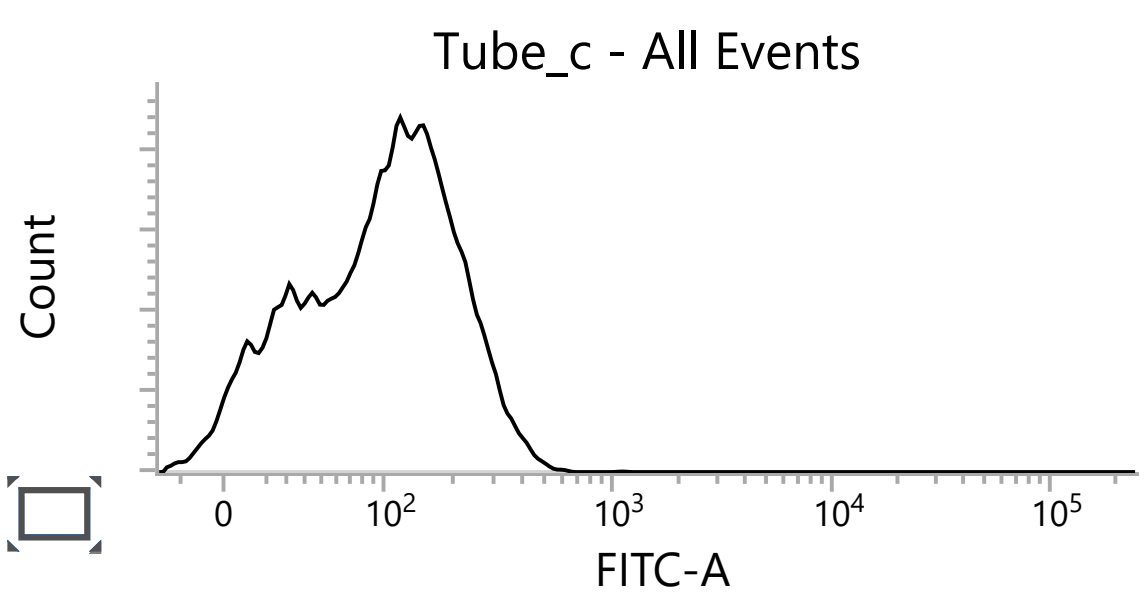

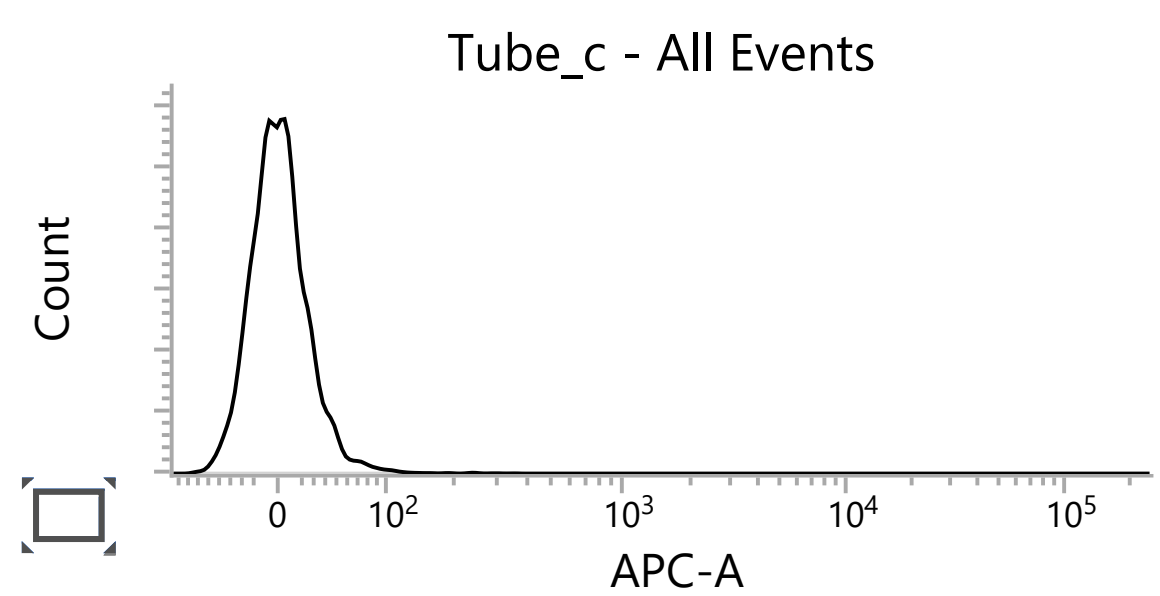

Statistics

Name	Events	% Parent	% Grandparent	% Total
Tube_a:All Events	10,000	***	***	100.00
Tube_b:All Events	10,000	***	***	100.00
Tube_c:All Events	10,000	***	***	100.00
Tube_a:Lebende Zellen	8,266	82.66	***	82.66
Tube_b:Lebende Zellen	6,784	67.84	***	67.84
Tube_c:Lebende Zellen	6,891	68.91	***	68.91
Tube_a:Dead	39	0.39	***	0.39
Tube_a:++	0	0.00	***	0.00
Tube_a:Live	9,960	99.60	***	99.60
Tube_a:ROS+	1	0.01	***	0.01
Tube_a:Dead	13	0.16	0.13	0.13
Tube_a:++	0	0.00	0.00	0.00
Tube_a:Live	8,252	99.83	82.52	82.52
Tube_a:ROS+	1	0.01	0.01	0.01
Tube_b:Dead	198	1.98	***	1.98
Tube_b:++	0	0.00	***	0.00
Tube_b:Live	9,798	97.98	***	97.98
Tube_b:ROS+	4	0.04	***	0.04
Tube_b:Dead	66	0.97	0.66	0.66
Tube_b:++	0	0.00	0.00	0.00
Tube_b:Live	6,716	99.00	67.16	67.16
Tube_b:ROS+	2	0.03	0.02	0.02
Tube_c:Dead	156	1.56	***	1.56
Tube_c:++	0	0.00	***	0.00
Tube_c:Live	9,844	98.44	***	98.44
Tube_c:ROS+	0	0.00	***	0.00
Tube_c:Dead	47	0.68	0.47	0.47
Tube_c:++	0	0.00	0.00	0.00
Tube_c:Live	6,844	99.32	68.44	68.44
Tube_c:ROS+	0	0.00	0.00	0.00

LDT

CellROX Assay

Sample ID: N6-ATM_2,5

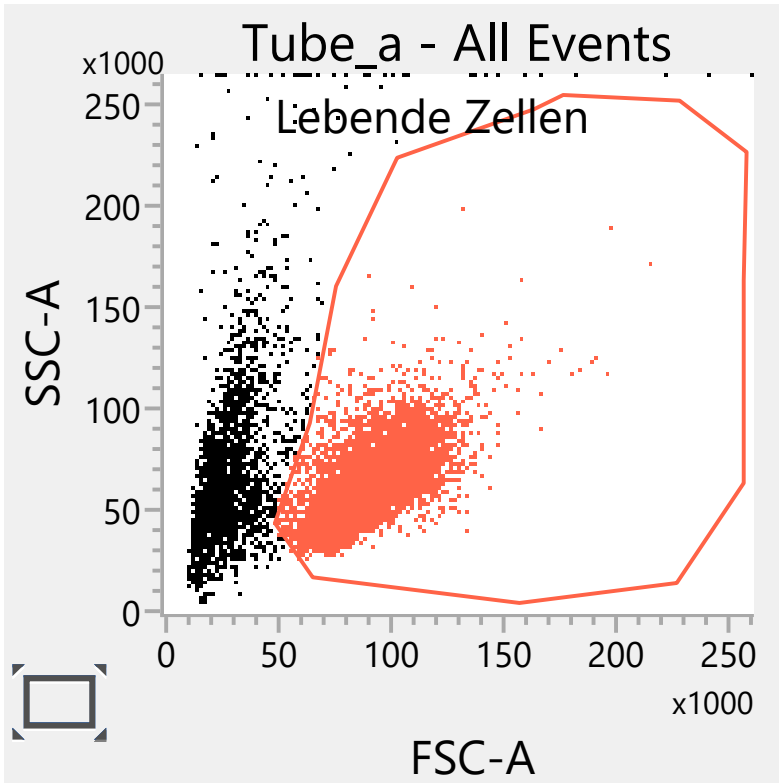

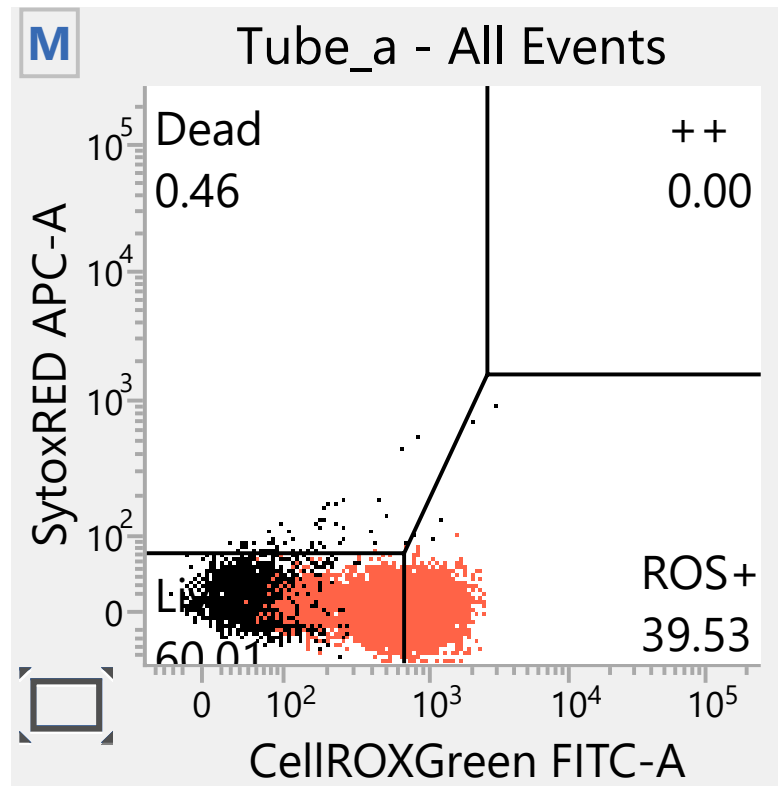

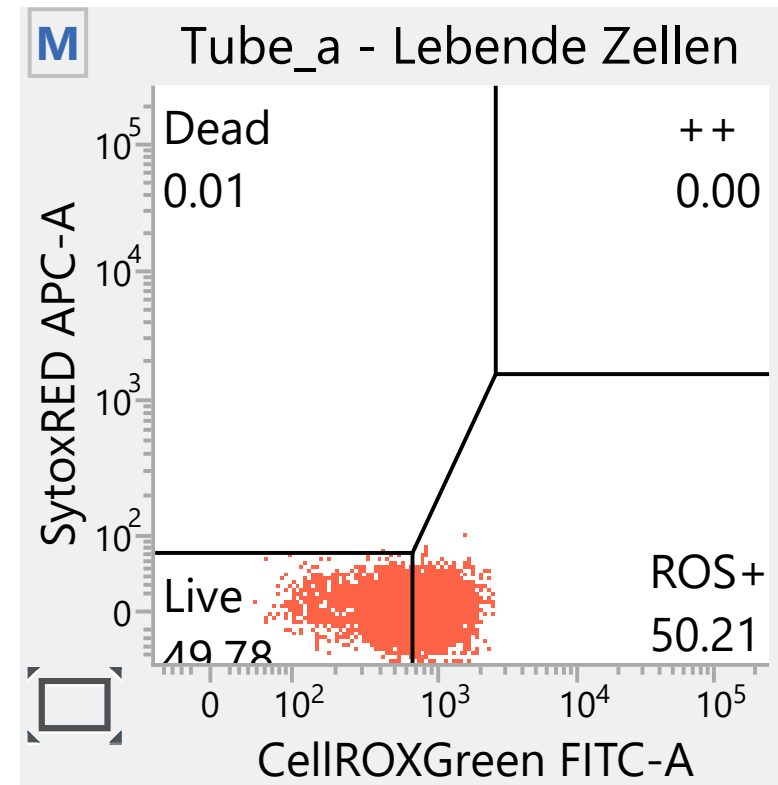

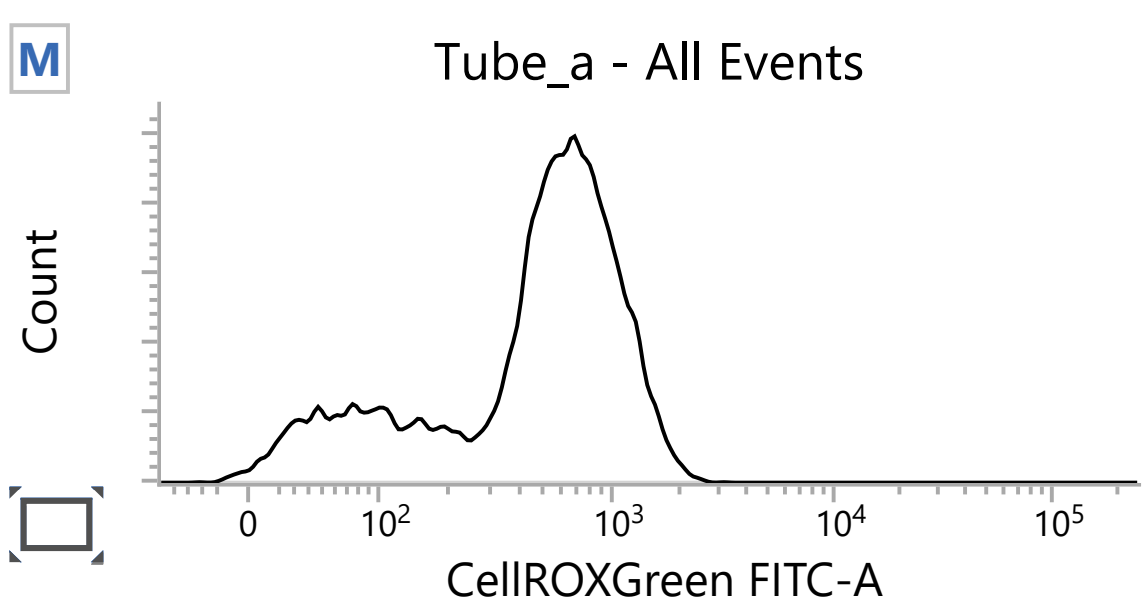

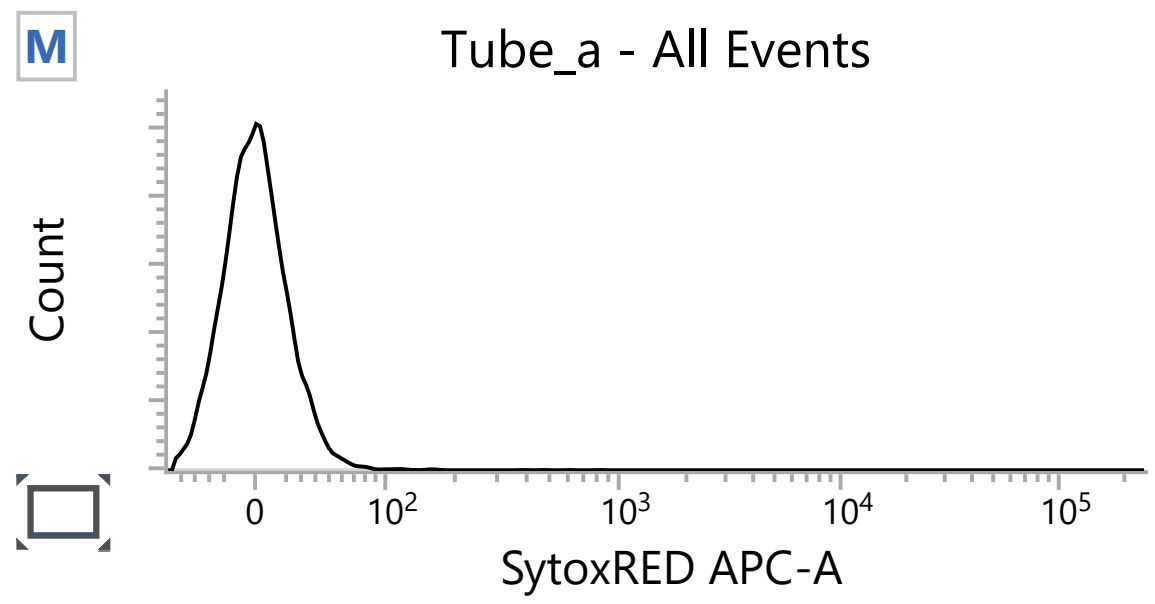

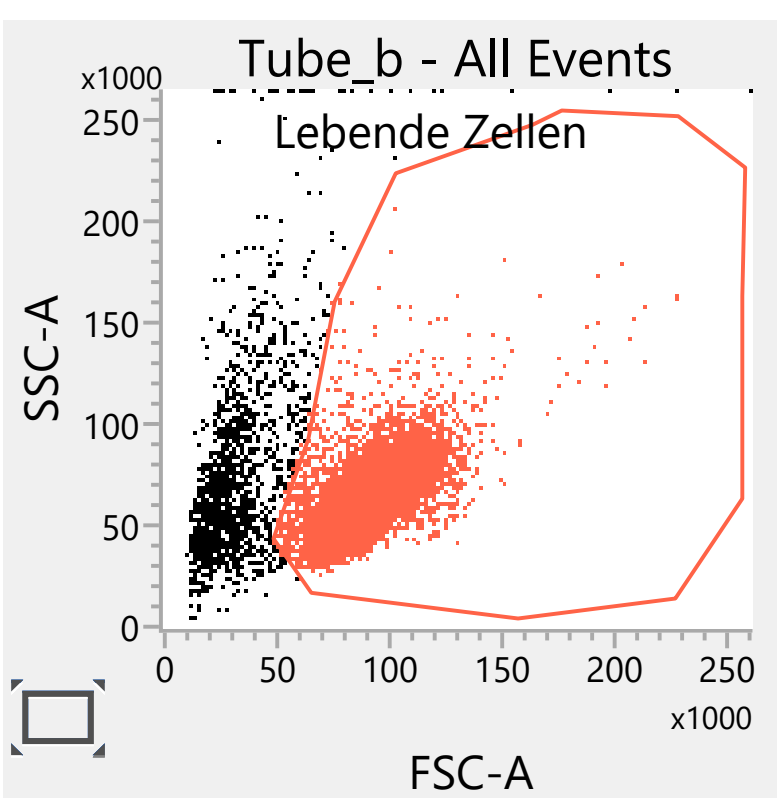

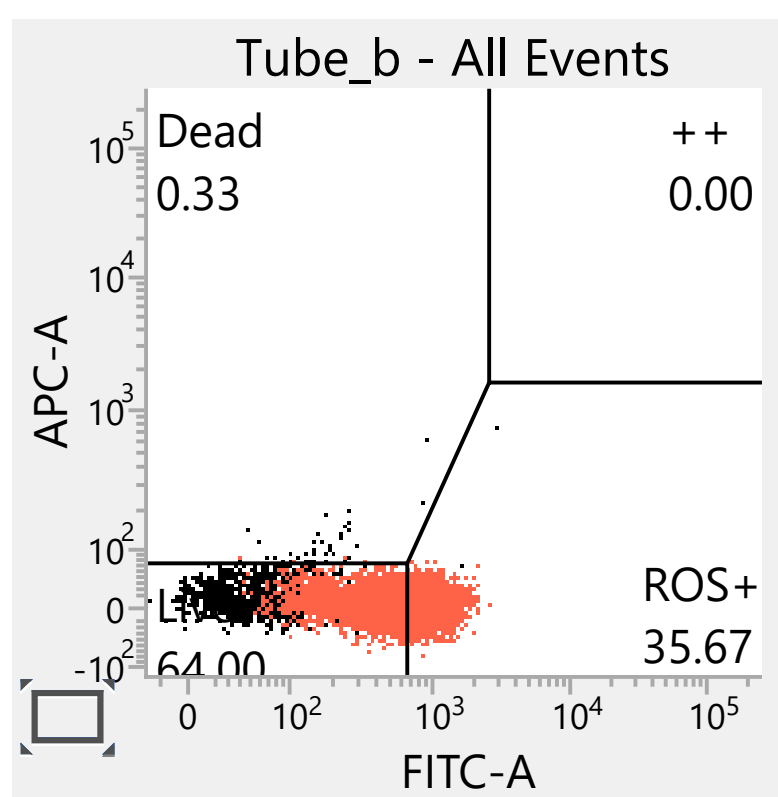

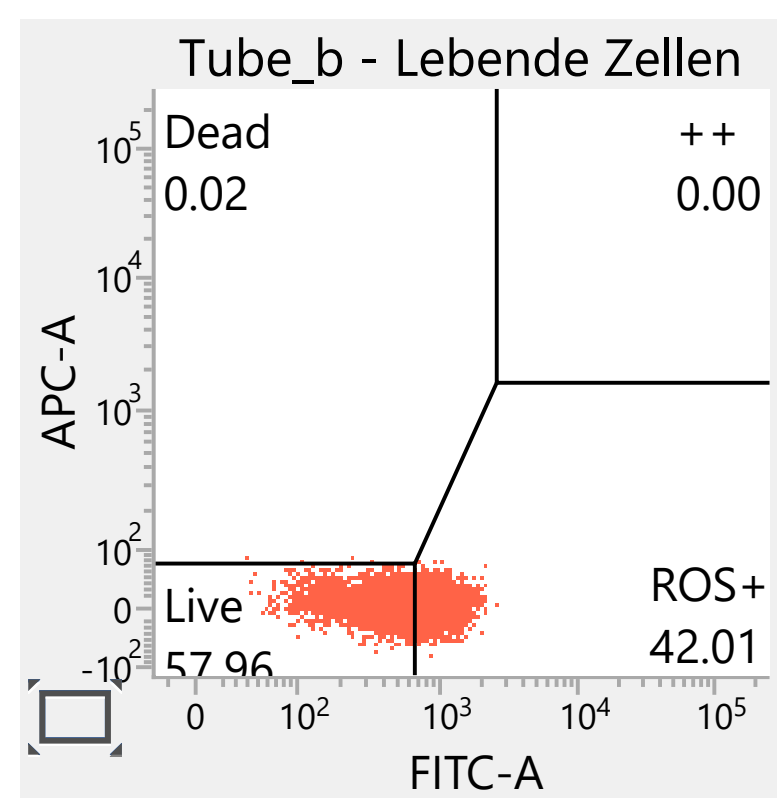

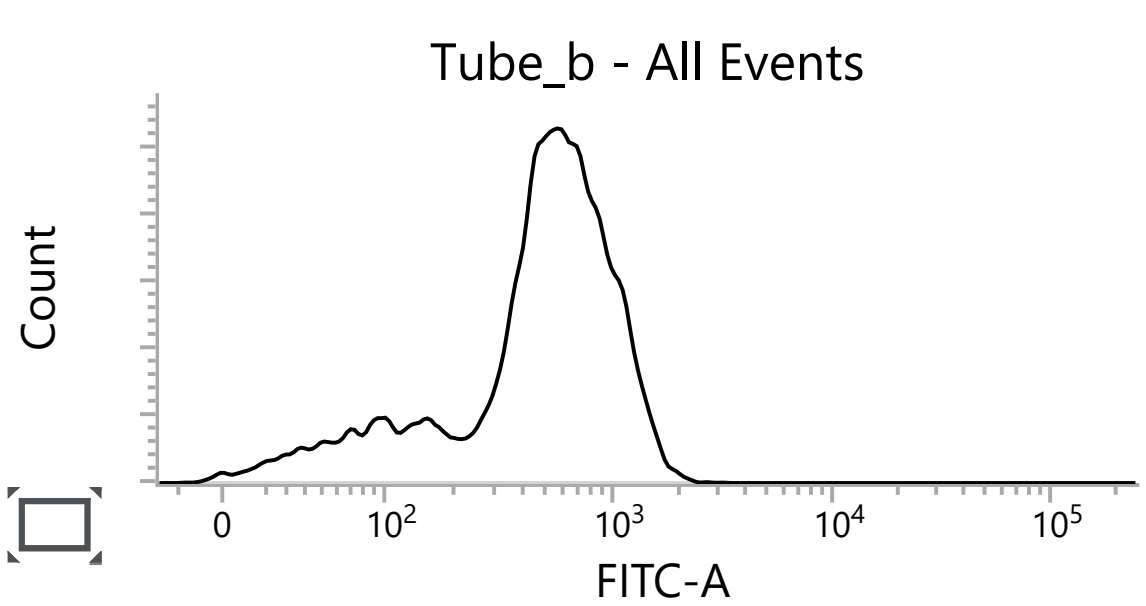

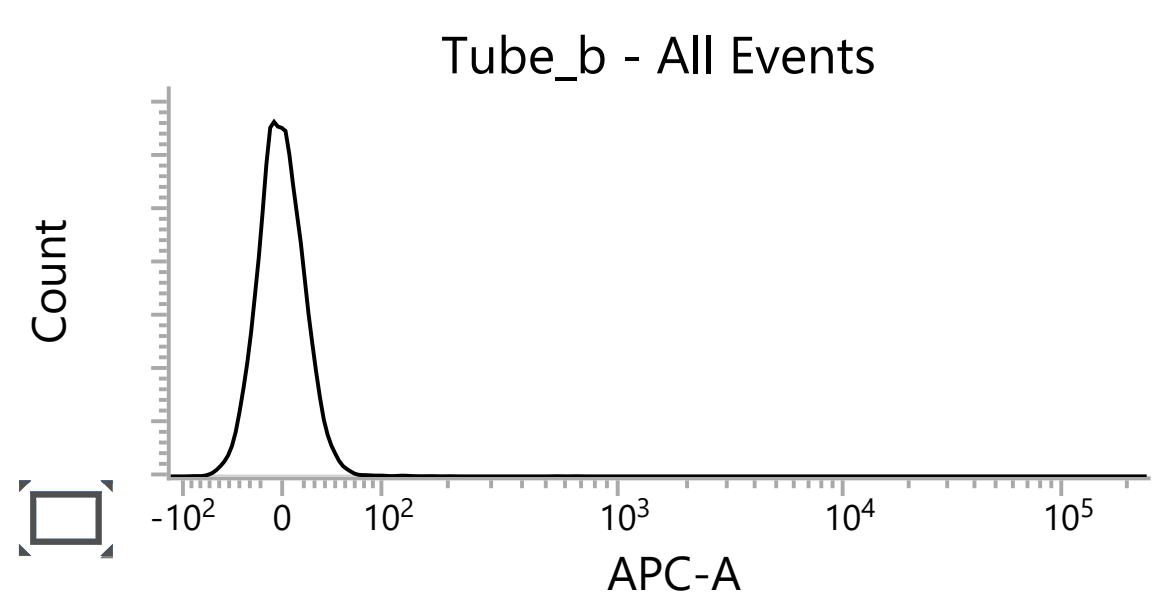

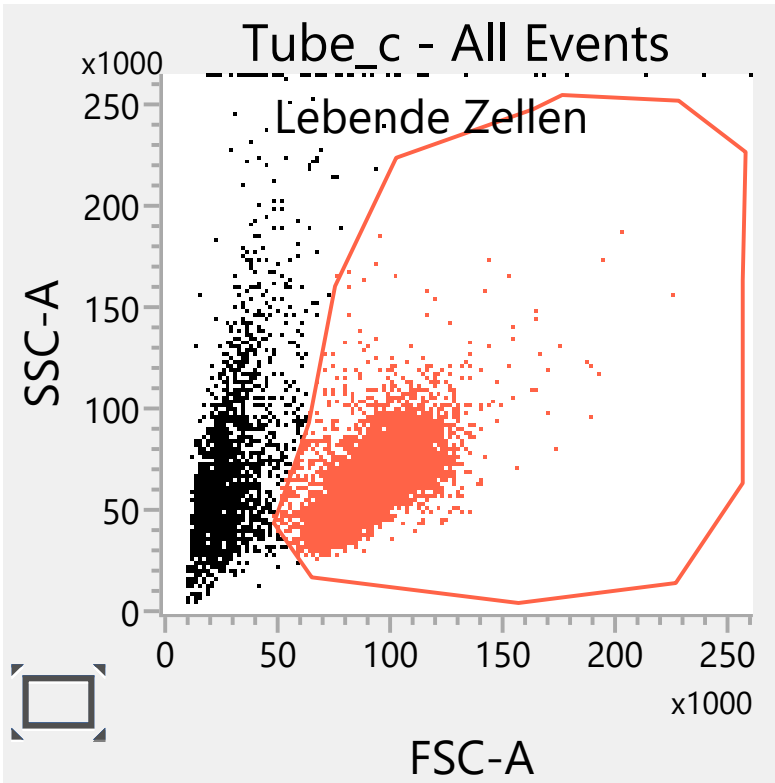

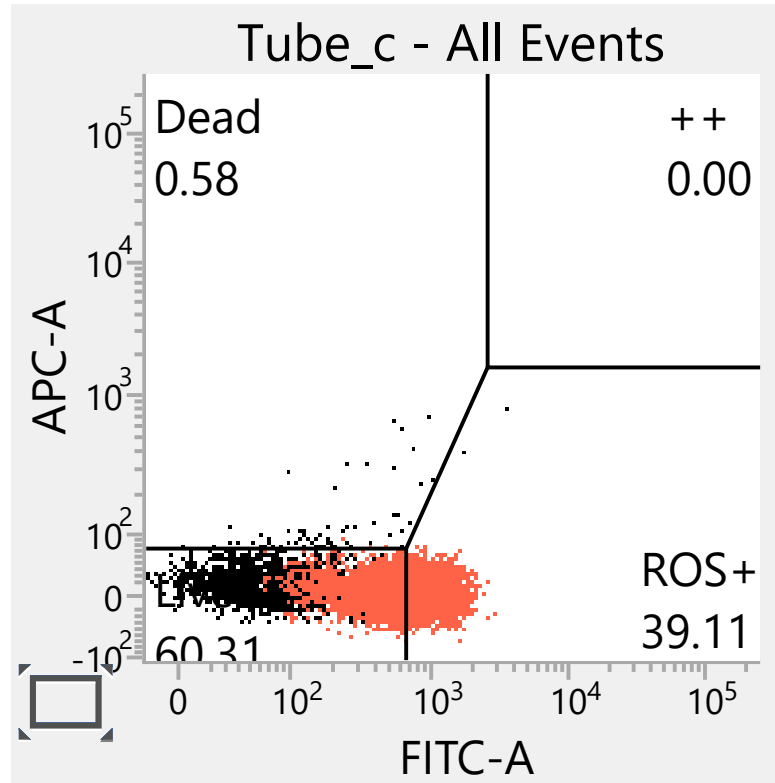

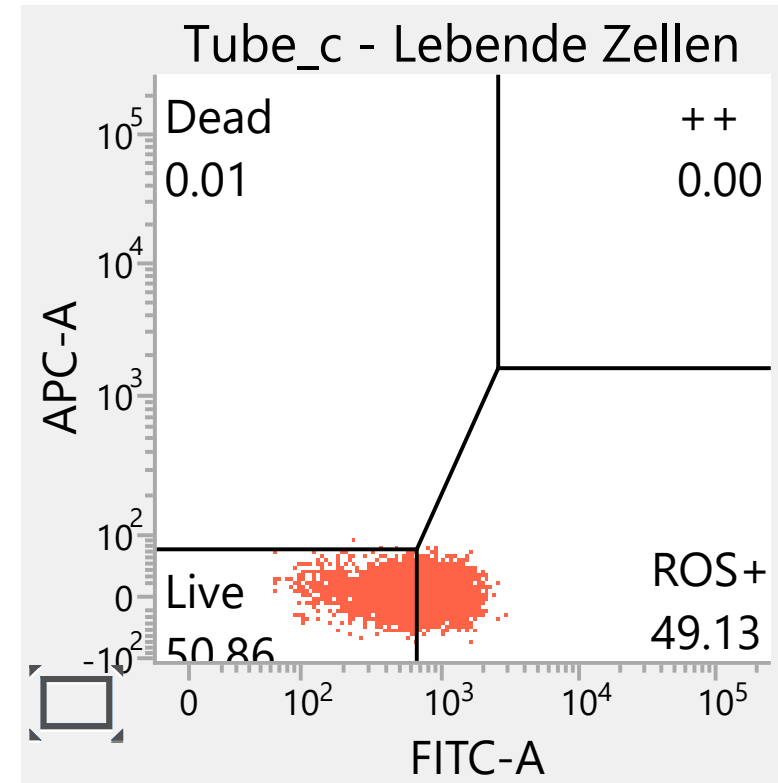

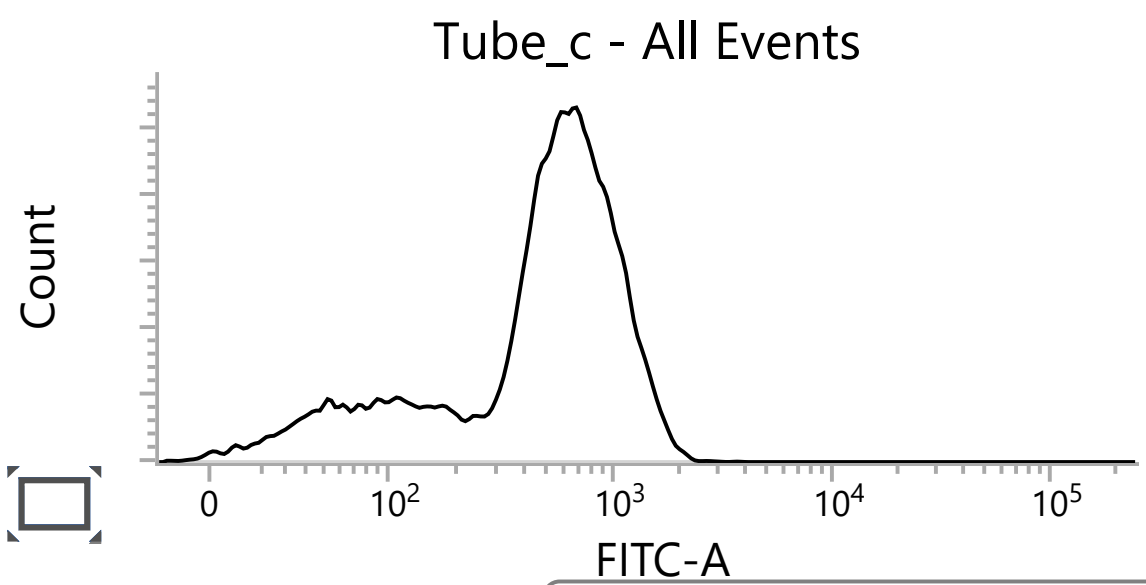

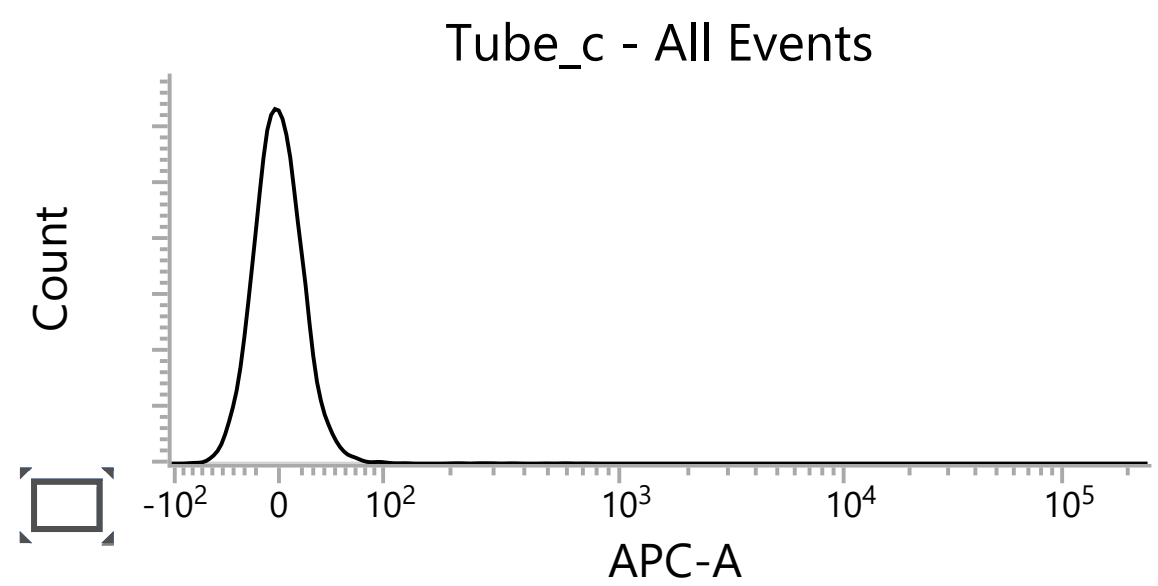

Statistics

Name	Events	% Parent	% Grandparent	% Total
Tube_a:All Events	10,000	***	***	100.00
Tube_b:All Events	10,000	***	***	100.00
Tube_c:All Events	10,000	***	***	100.00
Tube_a:Lebende Zellen	7,853	78.53	***	78.53
Tube_b:Lebende Zellen	8,481	84.81	***	84.81
Tube_c:Lebende Zellen	7,952	79.52	***	79.52
Tube_a:Dead	46	0.46	***	0.46
Tube_a:++	0	0.00	***	0.00
Tube_a:Live	6,001	60.01	***	60.01
Tube_a:ROS+	3,953	39.53	***	39.53
Tube_a:Dead	1	0.01	0.01	0.01
Tube_a:++	0	0.00	0.00	0.00
Tube_a:Live	3,909	49.78	39.09	39.09
Tube_a:ROS+	3,943	50.21	39.43	39.43
Tube_b:Dead	33	0.33	***	0.33
Tube_b:++	0	0.00	***	0.00
Tube_b:Live	6,400	64.00	***	64.00
Tube_b:ROS+	3,567	35.67	***	35.67
Tube_b:Dead	2	0.02	0.02	0.02
Tube_b:++	0	0.00	0.00	0.00
Tube_b:Live	4,916	57.96	49.16	49.16
Tube_b:ROS+	3,563	42.01	35.63	35.63
Tube_c:Dead	58	0.58	***	0.58
Tube_c:++	0	0.00	***	0.00
Tube_c:Live	6,031	60.31	***	60.31
Tube_c:ROS+	3,911	39.11	***	39.11
Tube_c:Dead	1	0.01	0.01	0.01
Tube_c:++	0	0.00	0.00	0.00
Tube_c:Live	4,044	50.86	40.44	40.44
Tube_c:ROS+	3,907	49.13	39.07	39.07

LDT

CellROX Assay

Sample ID: N6-ATM_5 NAC

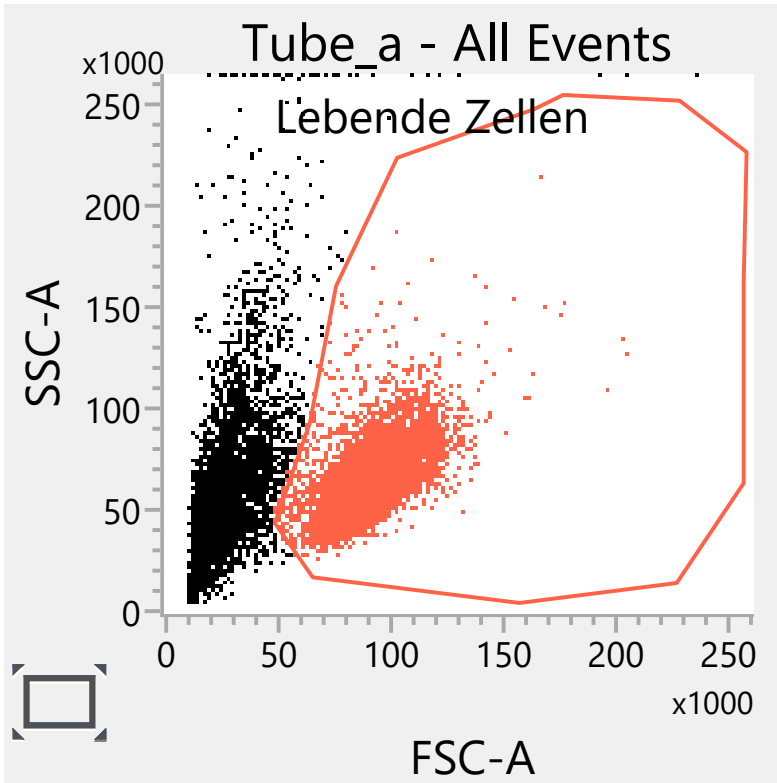

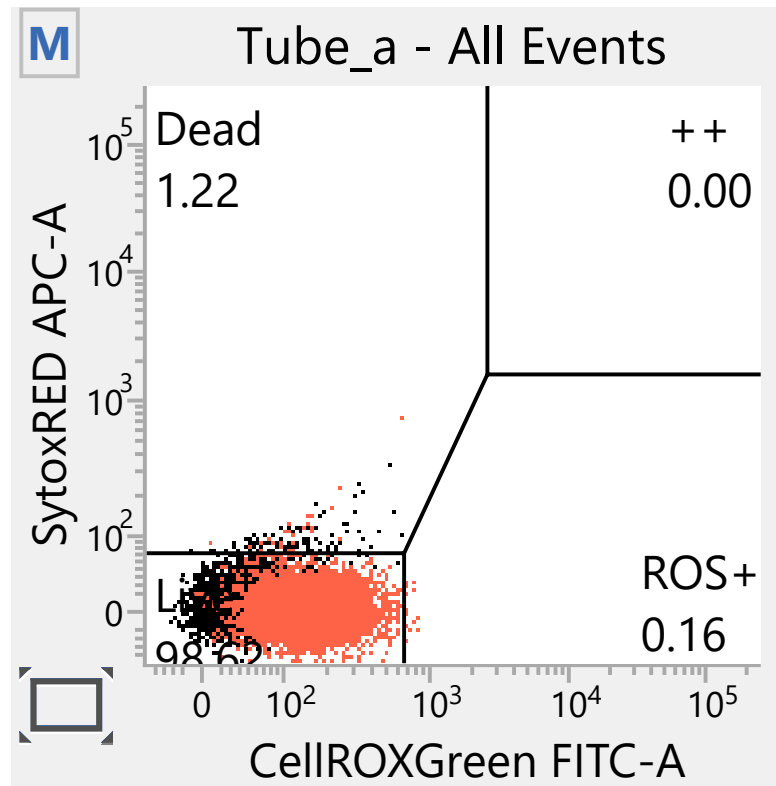

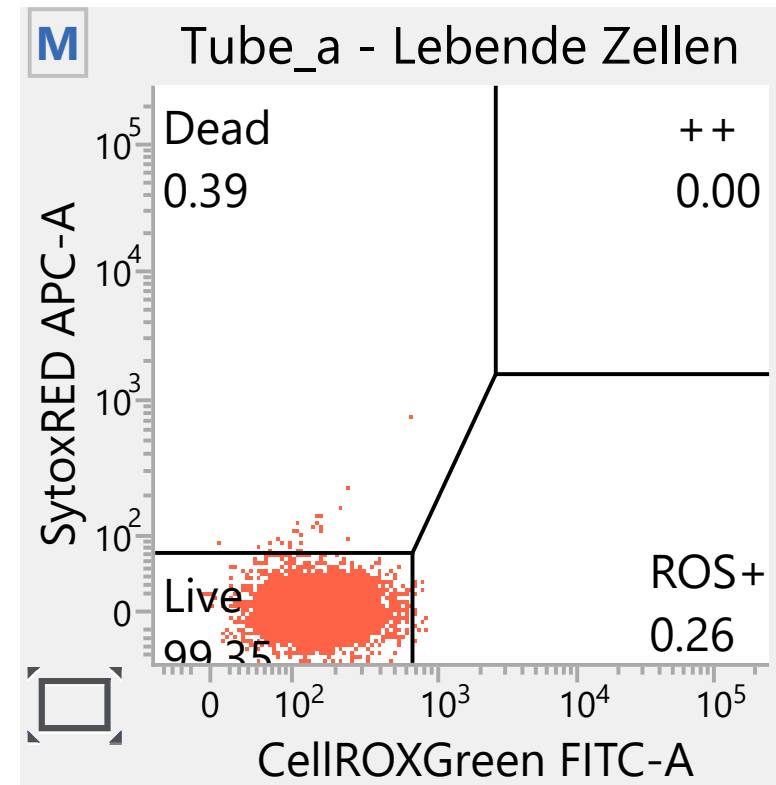

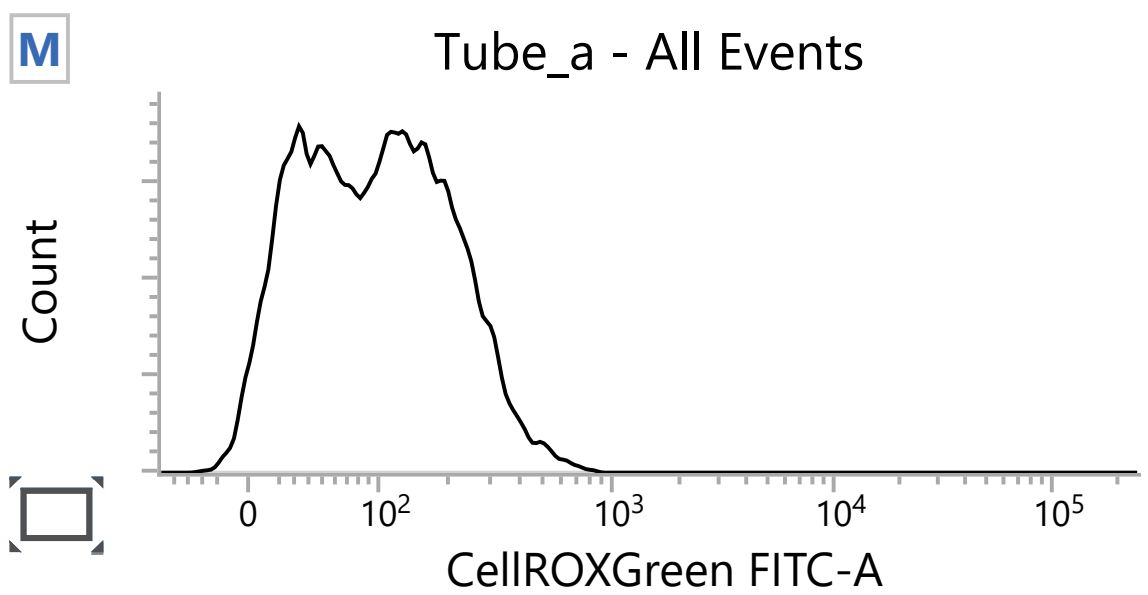

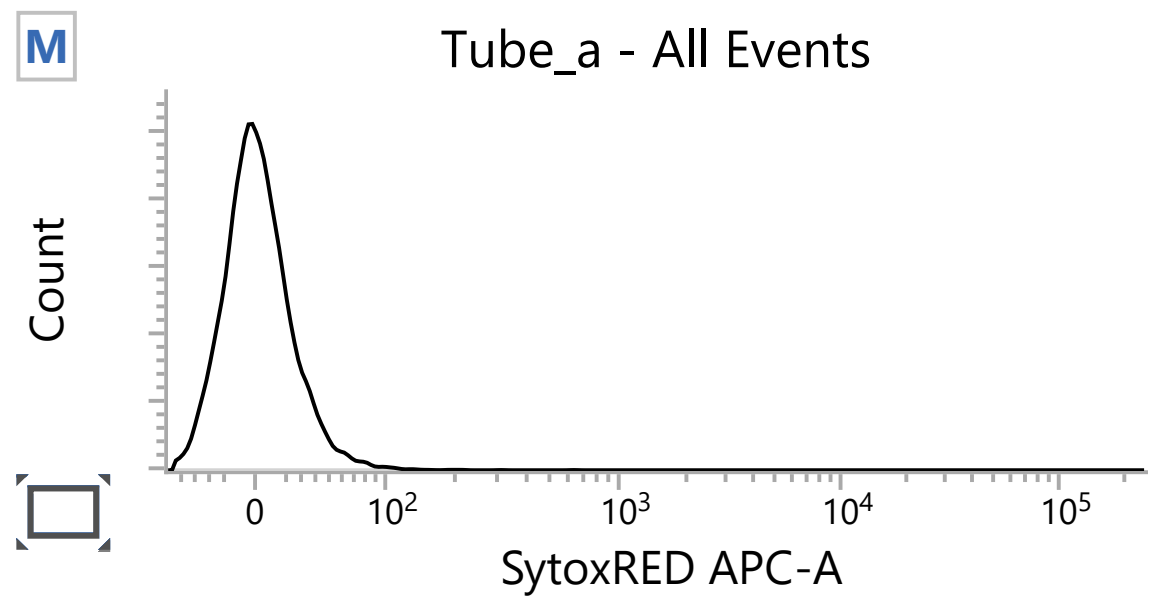

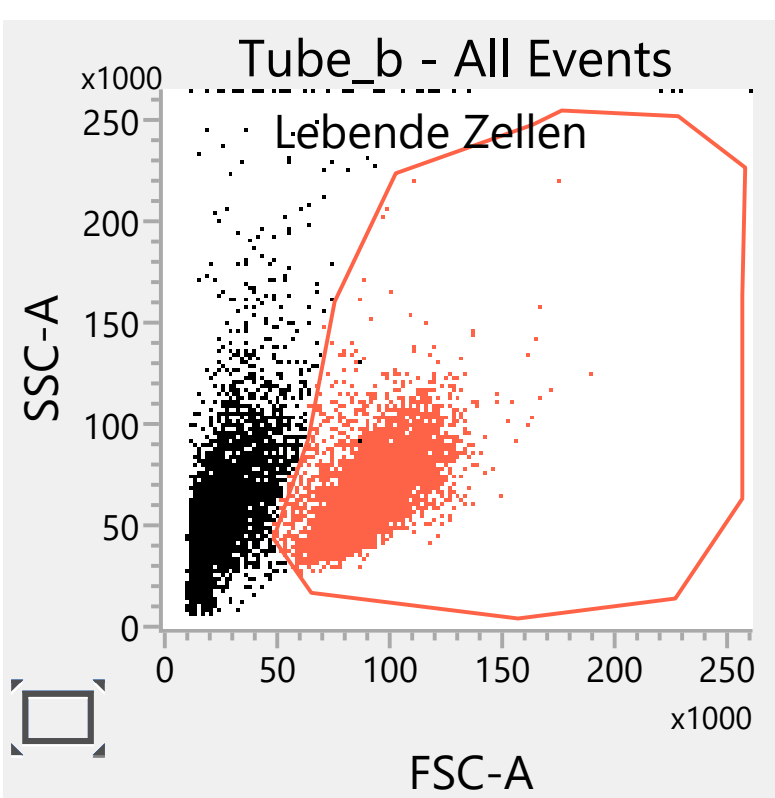

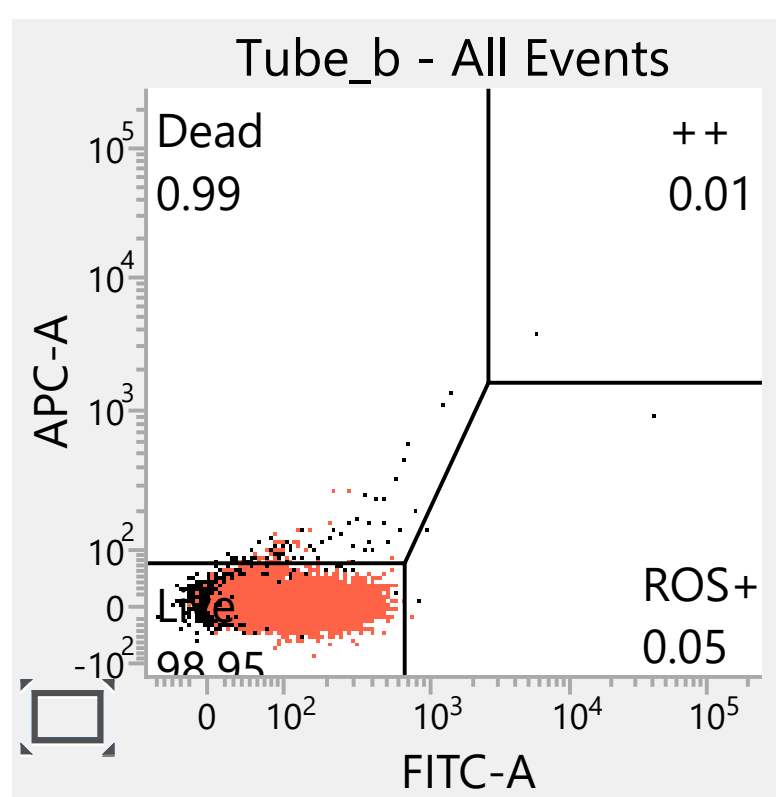

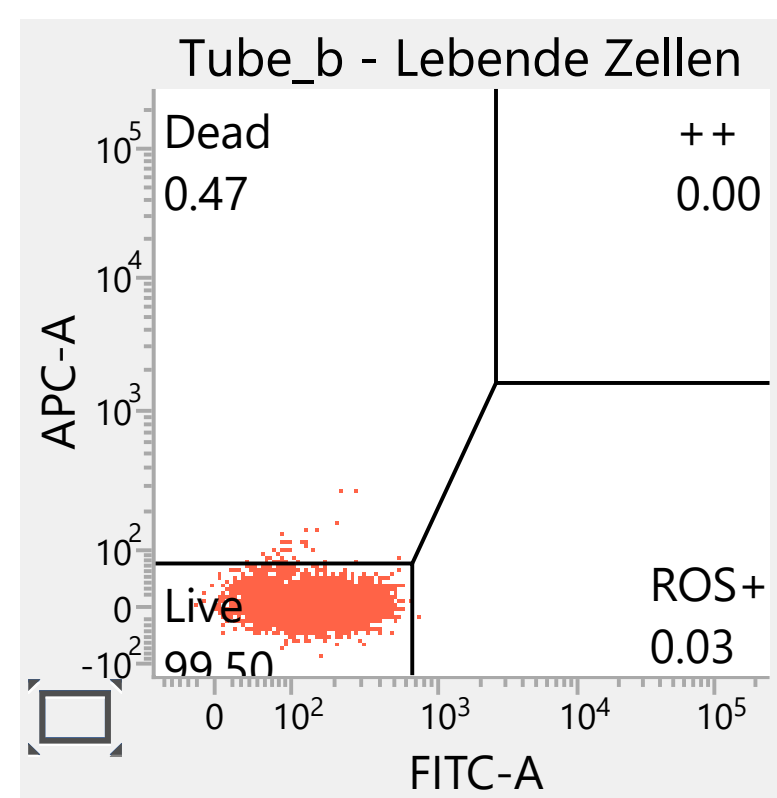

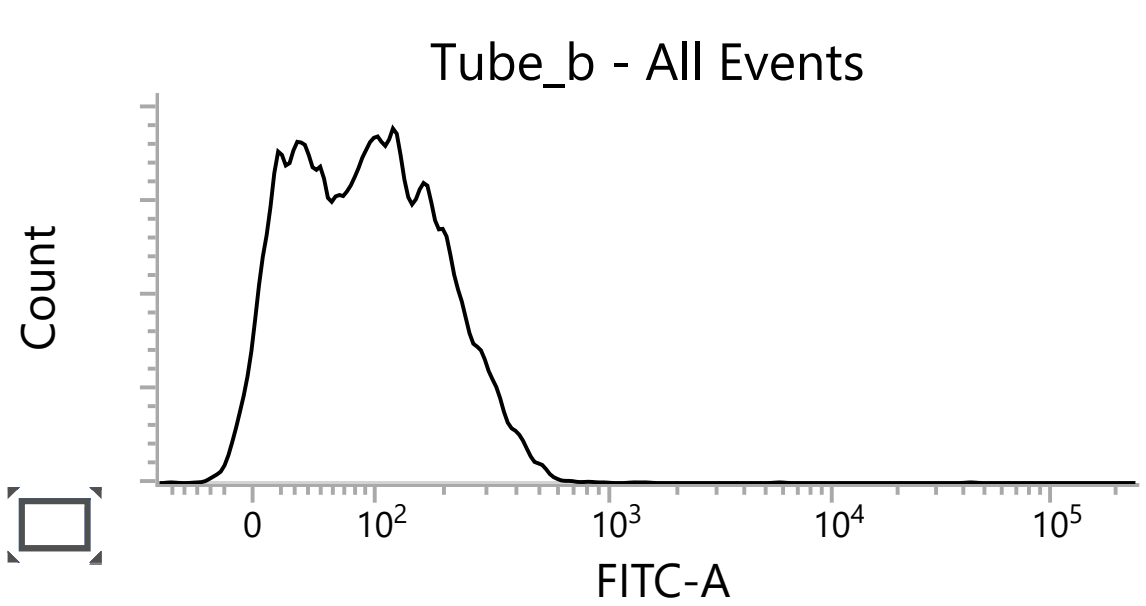

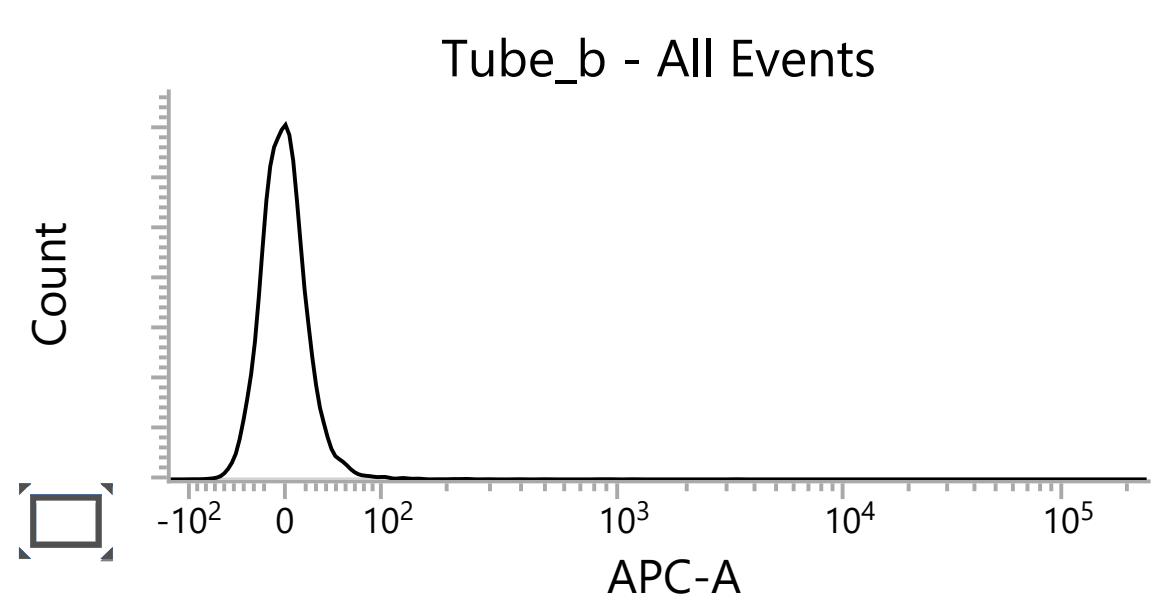

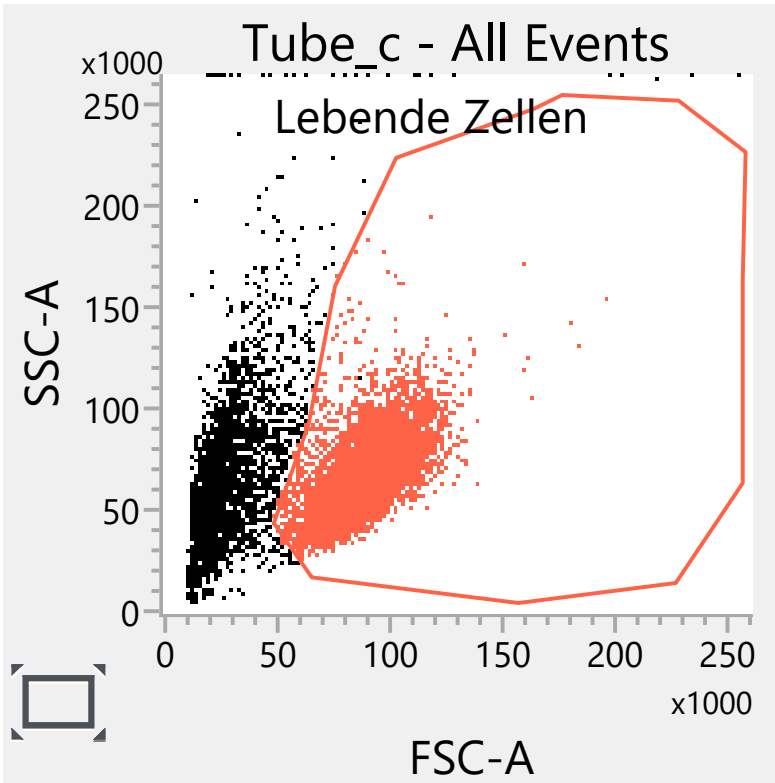

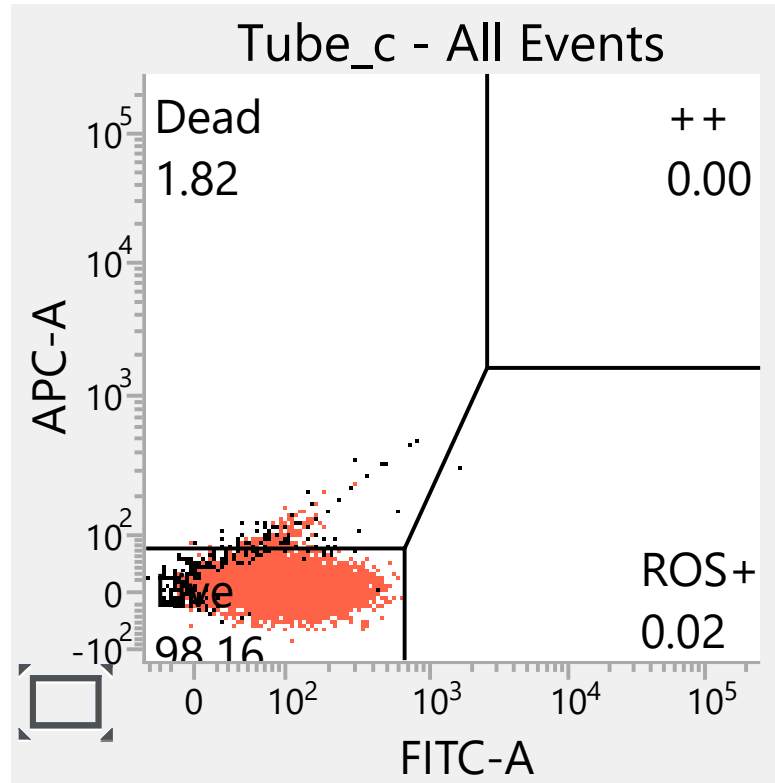

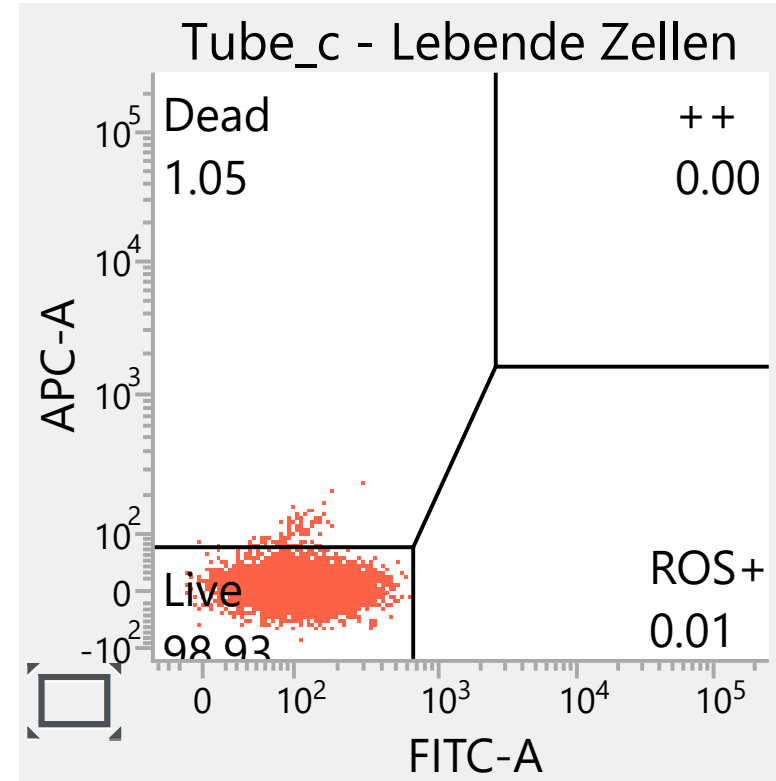

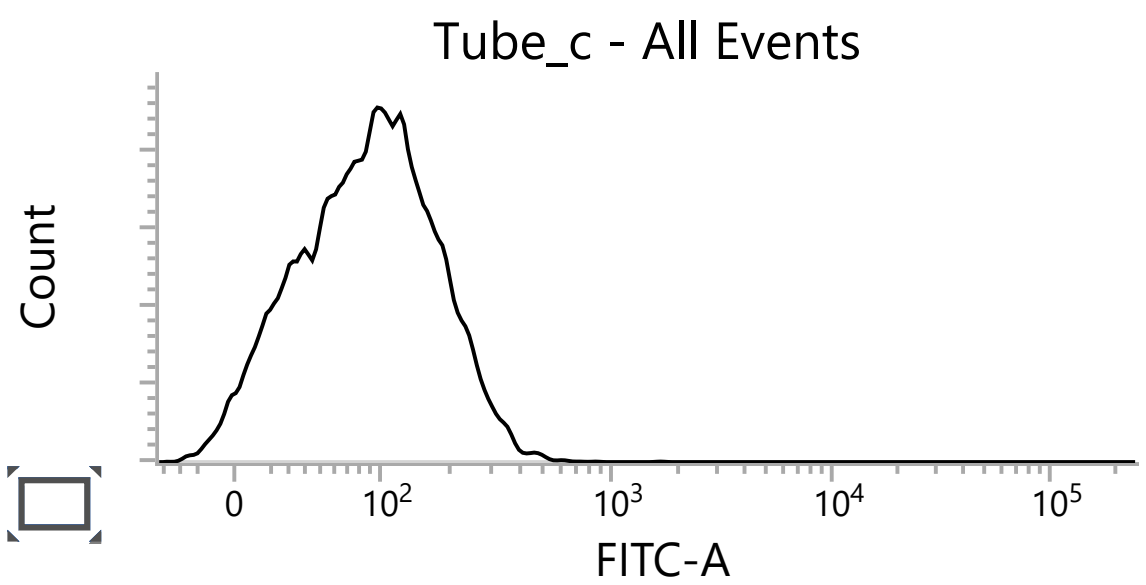

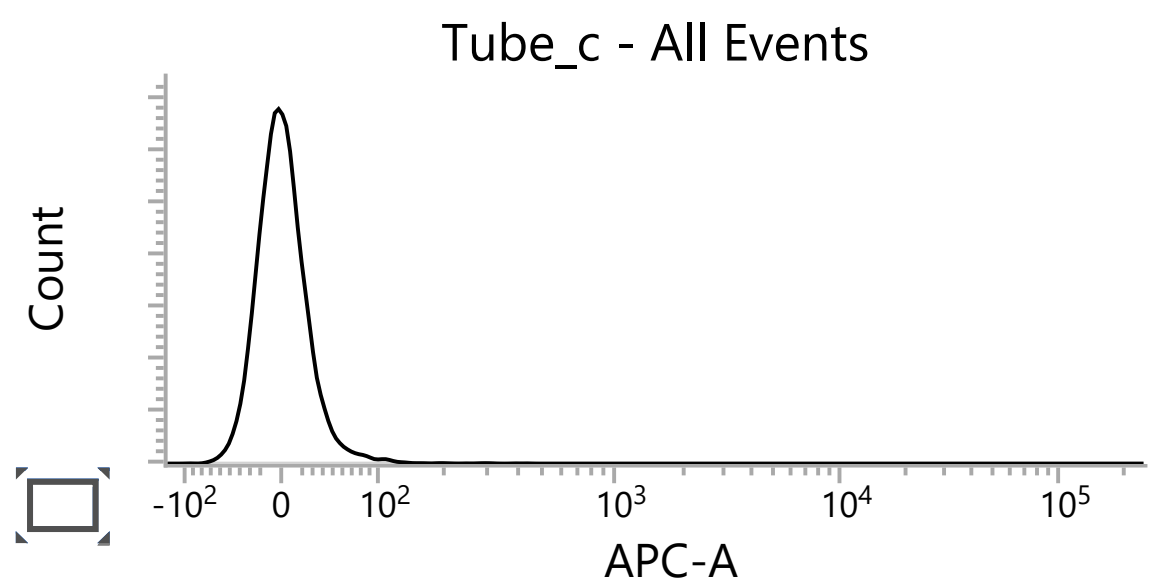

Statistics

Name	Events	% Parent	% Grandparent	% Total
Tube_a:All Events	10,000	***	***	100.00
Tube_b:All Events	10,000	***	***	100.00
Tube_c:All Events	10,000	***	***	100.00
Tube_a:Lebende Zellen	5,699	56.99	***	56.99
Tube_b:Lebende Zellen	6,430	64.30	***	64.30
Tube_c:Lebende Zellen	7,398	73.98	***	73.98
Tube_a:Dead	122	1.22	***	1.22
Tube_a:++	0	0.00	***	0.00
Tube_a:Live	9,862	98.62	***	98.62
Tube_a:ROS+	16	0.16	***	0.16
Tube_a:Dead	22	0.39	0.22	0.22
Tube_a:++	0	0.00	0.00	0.00
Tube_a:Live	5,662	99.35	56.62	56.62
Tube_a:ROS+	15	0.26	0.15	0.15
Tube_b:Dead	99	0.99	***	0.99
Tube_b:++	1	0.01	***	0.01
Tube_b:Live	9,895	98.95	***	98.95
Tube_b:ROS+	5	0.05	***	0.05
Tube_b:Dead	30	0.47	0.30	0.30
Tube_b:++	0	0.00	0.00	0.00
Tube_b:Live	6,398	99.50	63.98	63.98
Tube_b:ROS+	2	0.03	0.02	0.02
Tube_c:Dead	182	1.82	***	1.82
Tube_c:++	0	0.00	***	0.00
Tube_c:Live	9,816	98.16	***	98.16
Tube_c:ROS+	2	0.02	***	0.02
Tube_c:Dead	78	1.05	0.78	0.78
Tube_c:++	0	0.00	0.00	0.00
Tube_c:Live	7,319	98.93	73.19	73.19
Tube_c:ROS+	1	0.01	0.01	0.01

LDT

CellROX Assay

Sample ID: N6-ATM_5

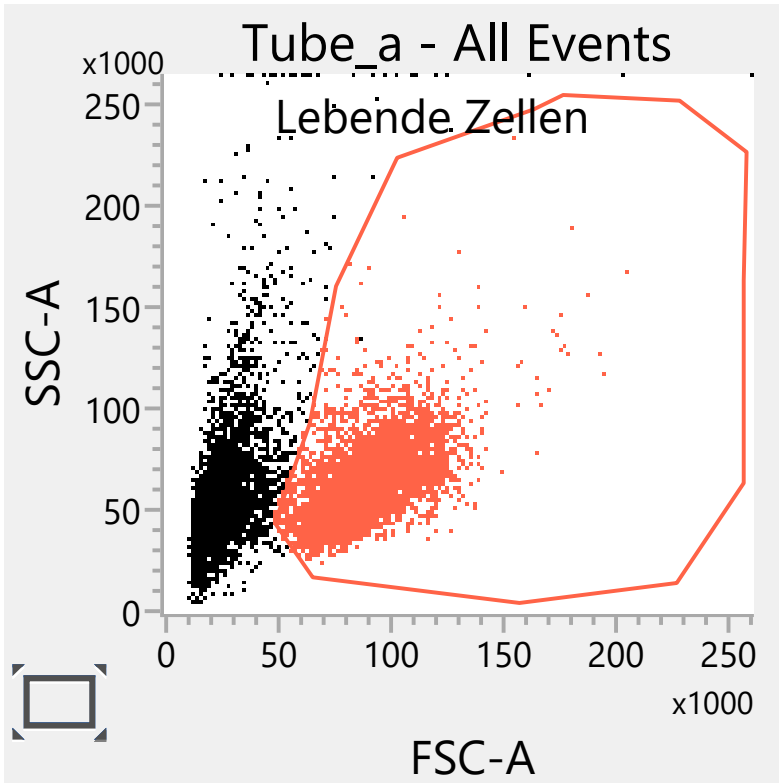

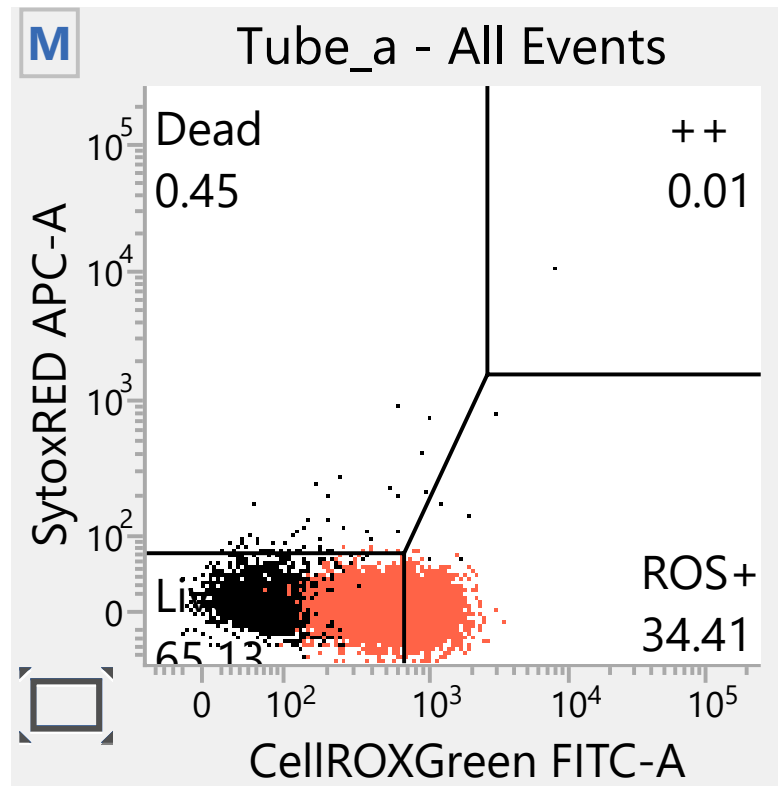

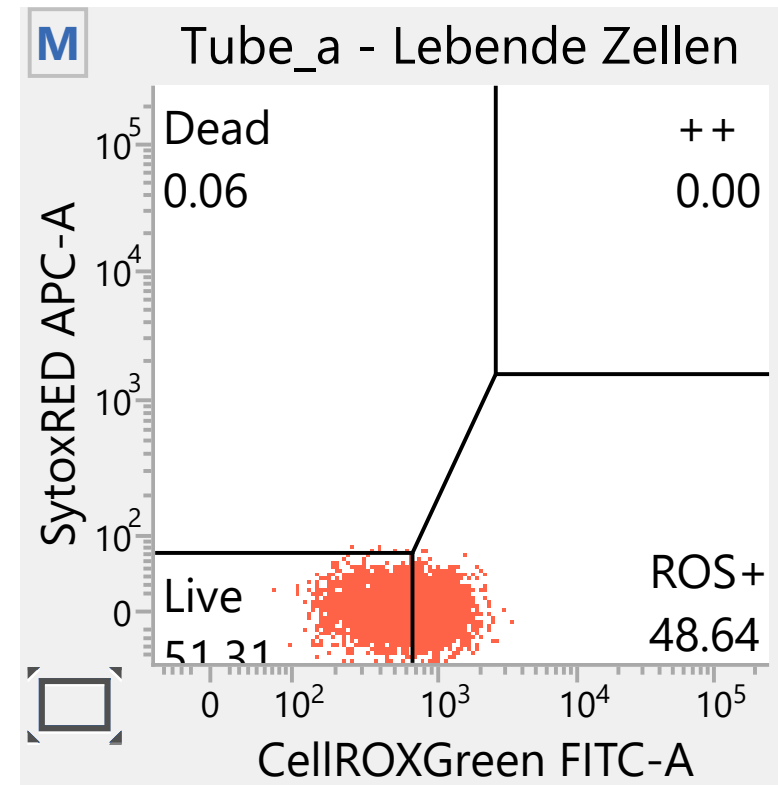

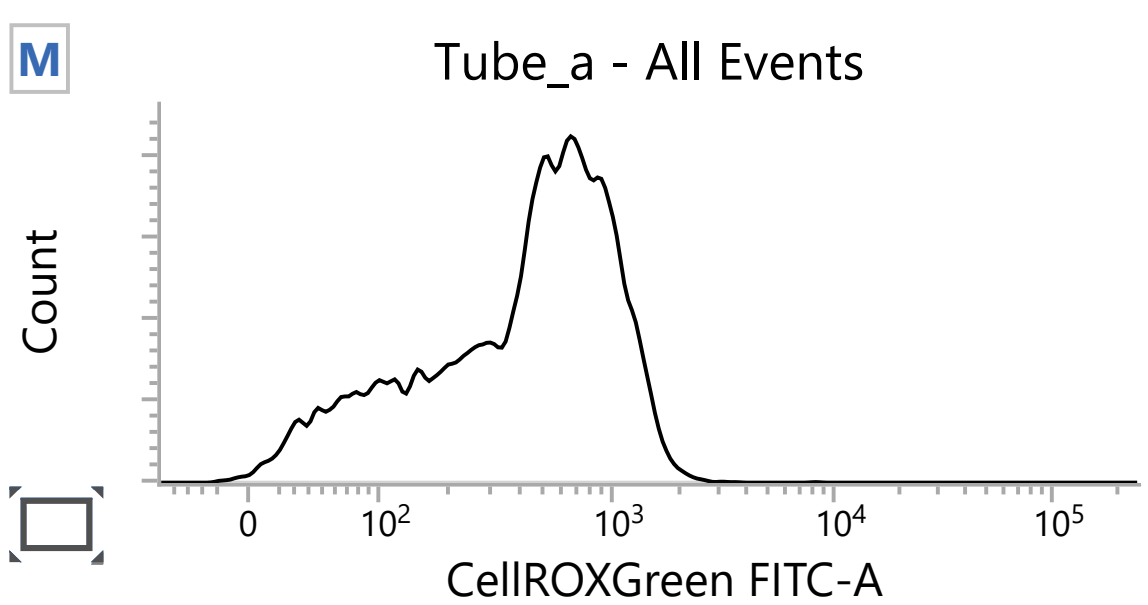

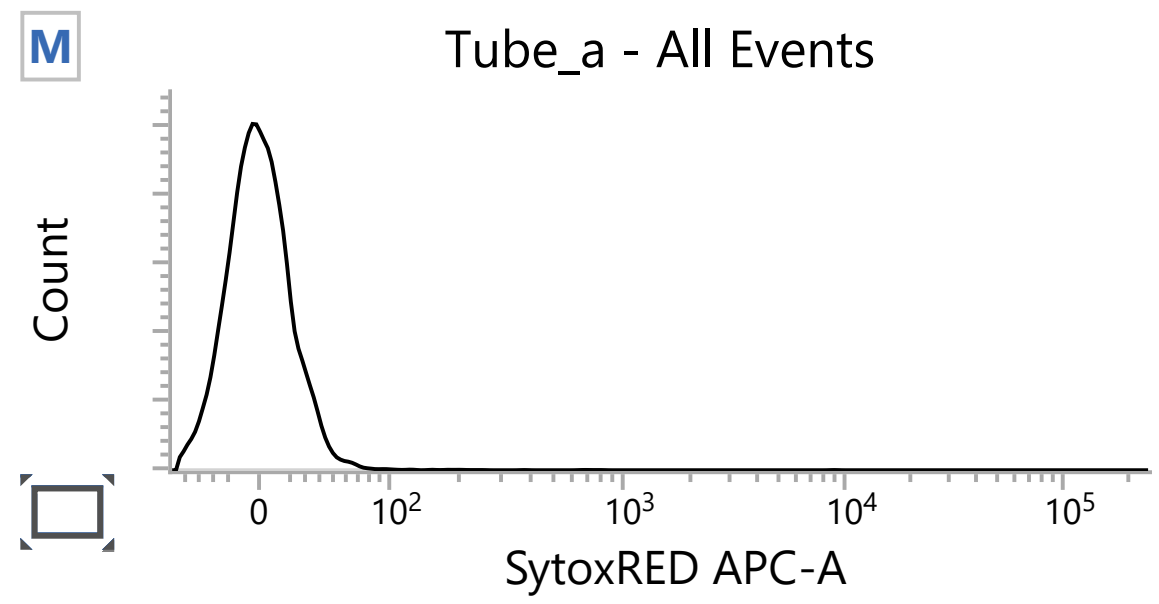

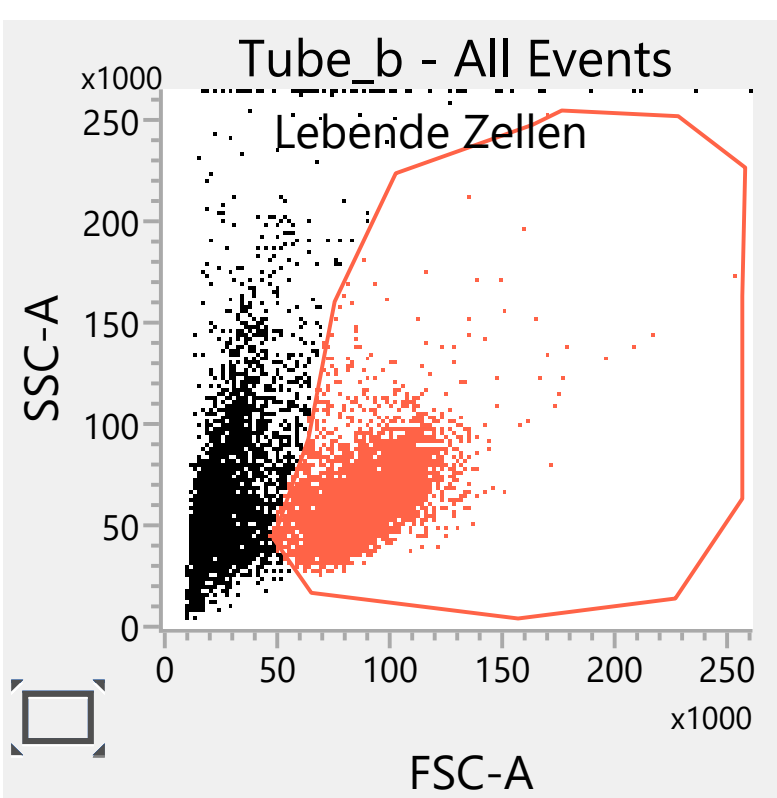

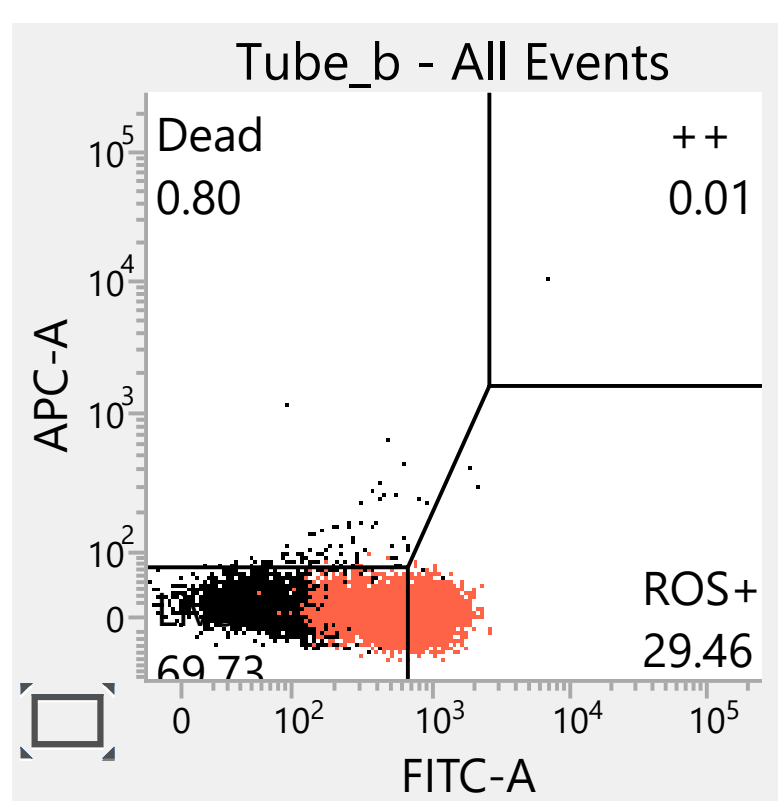

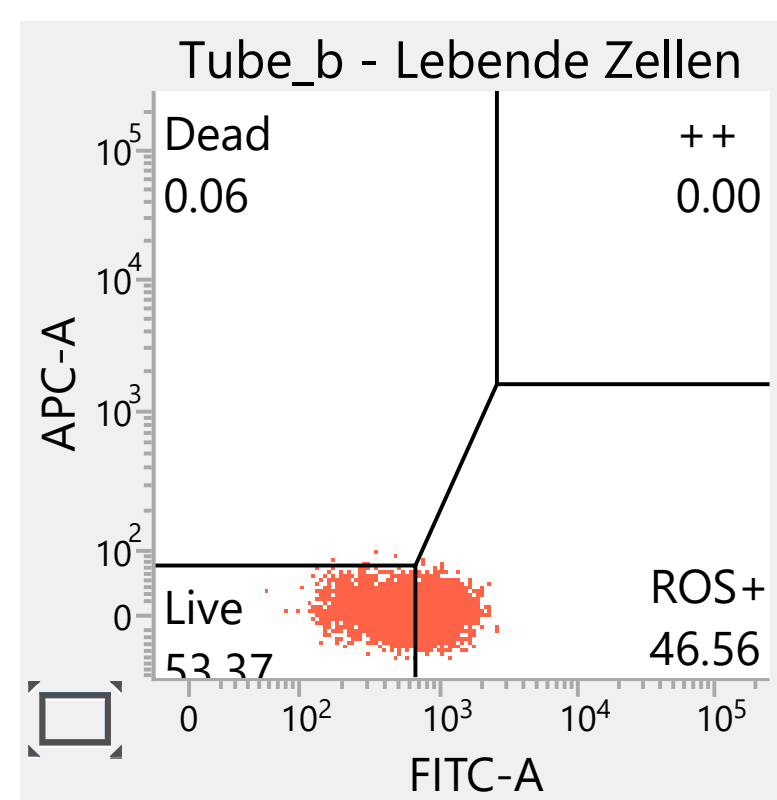

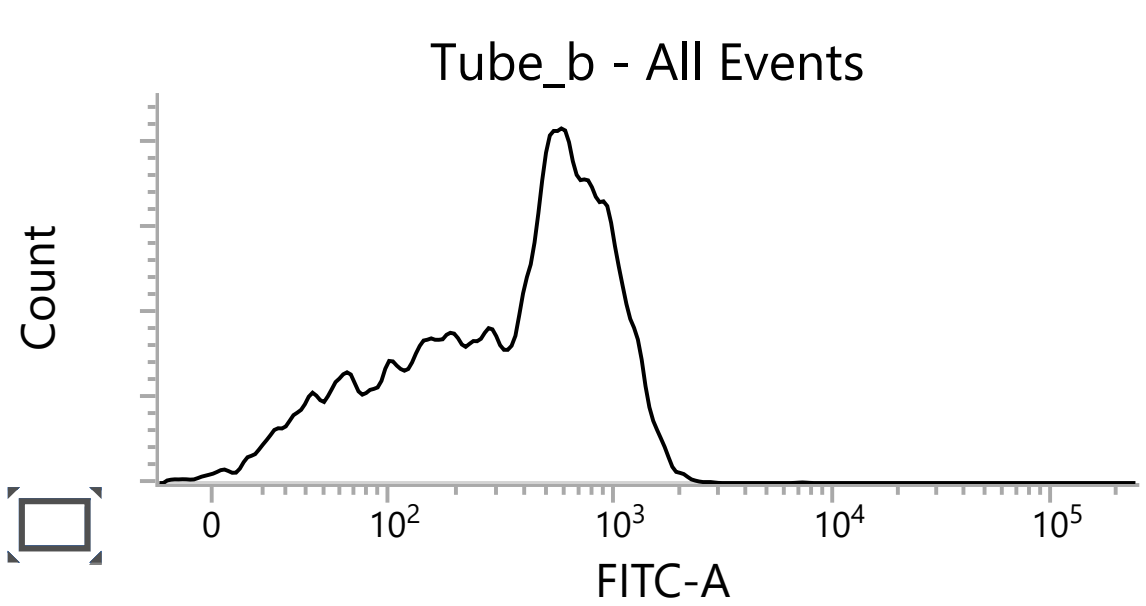

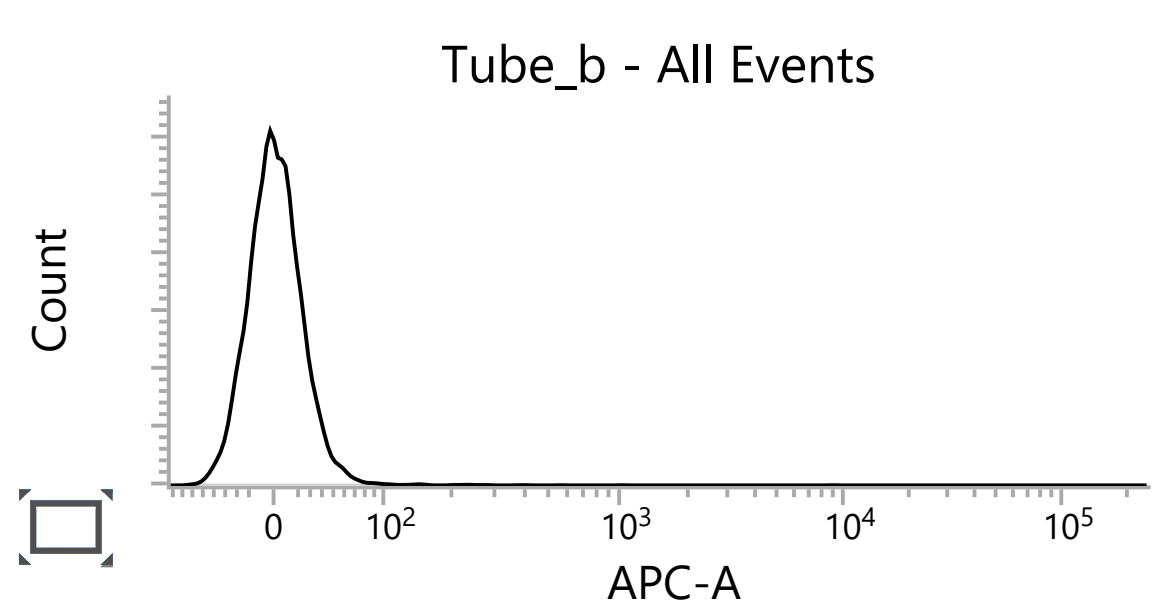

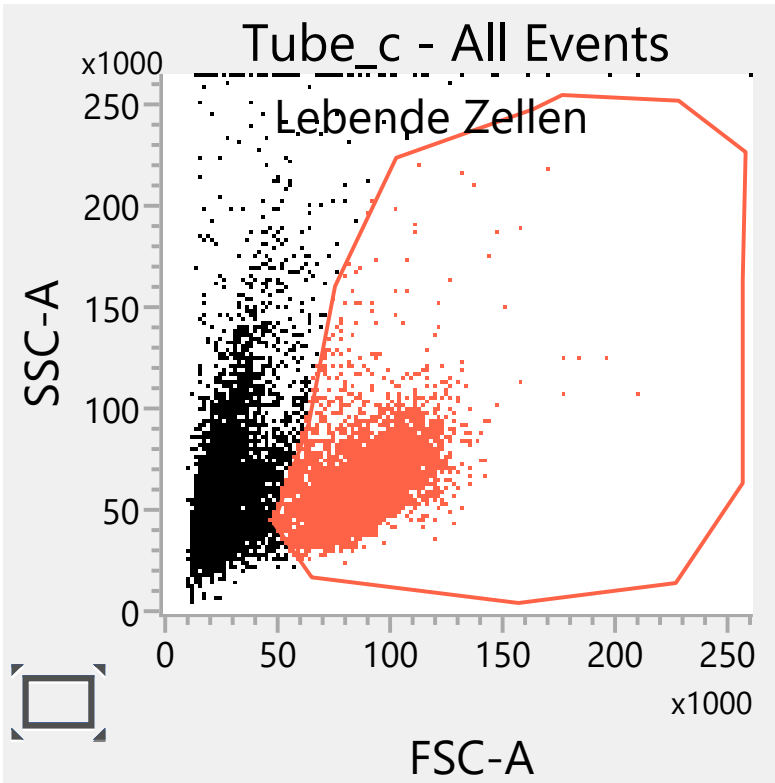

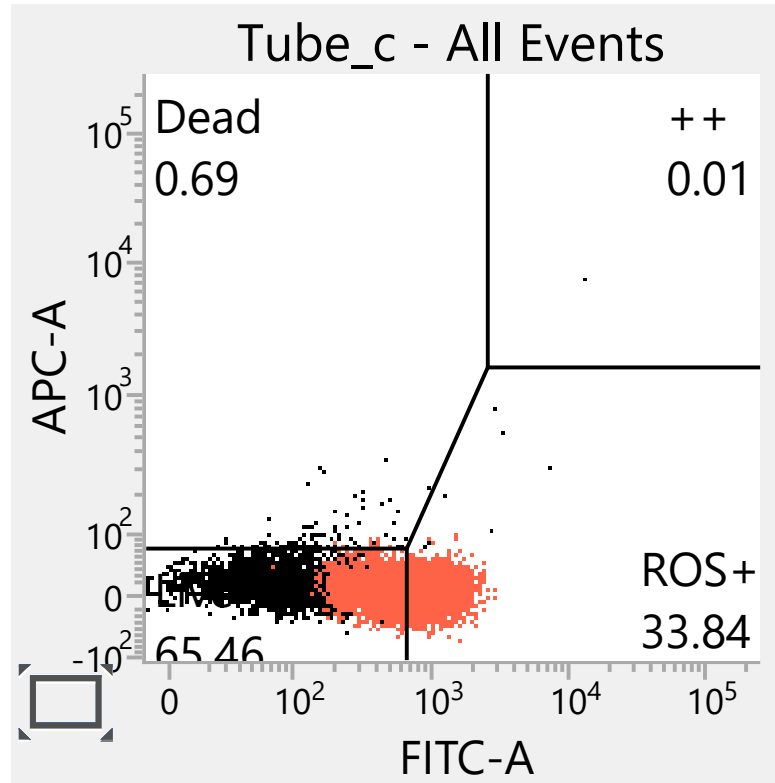

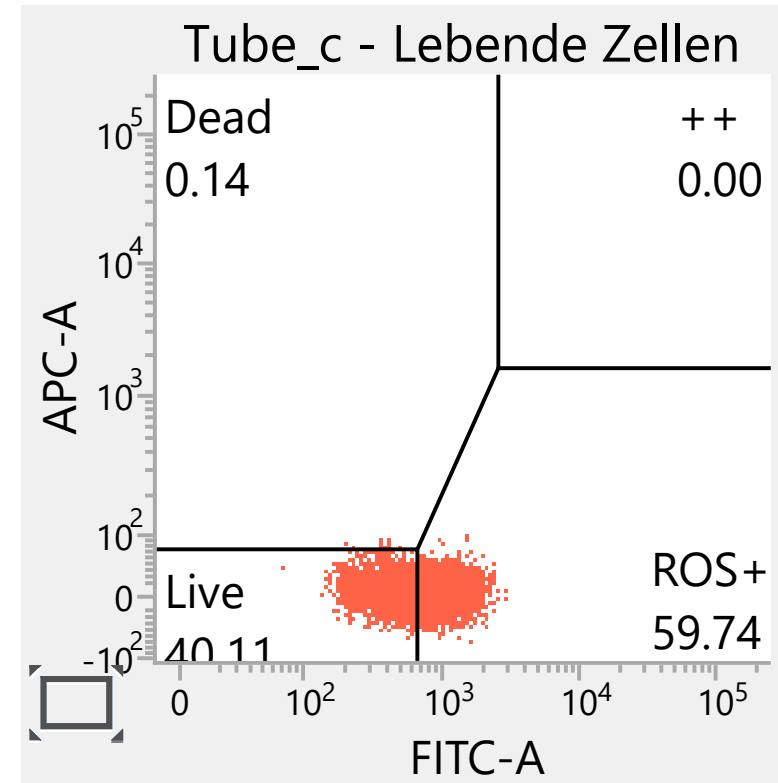

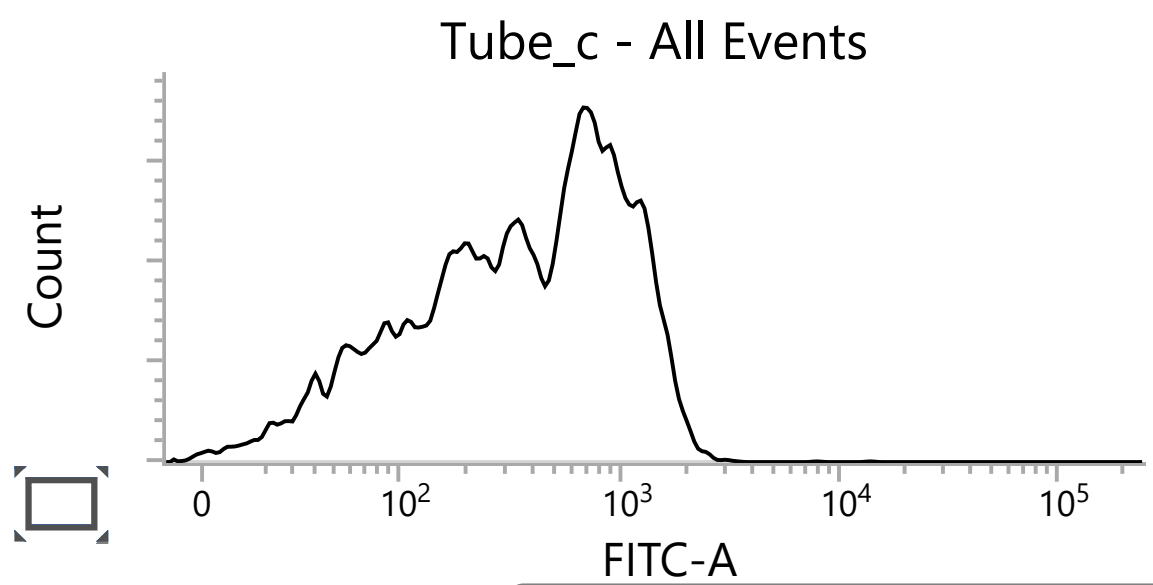

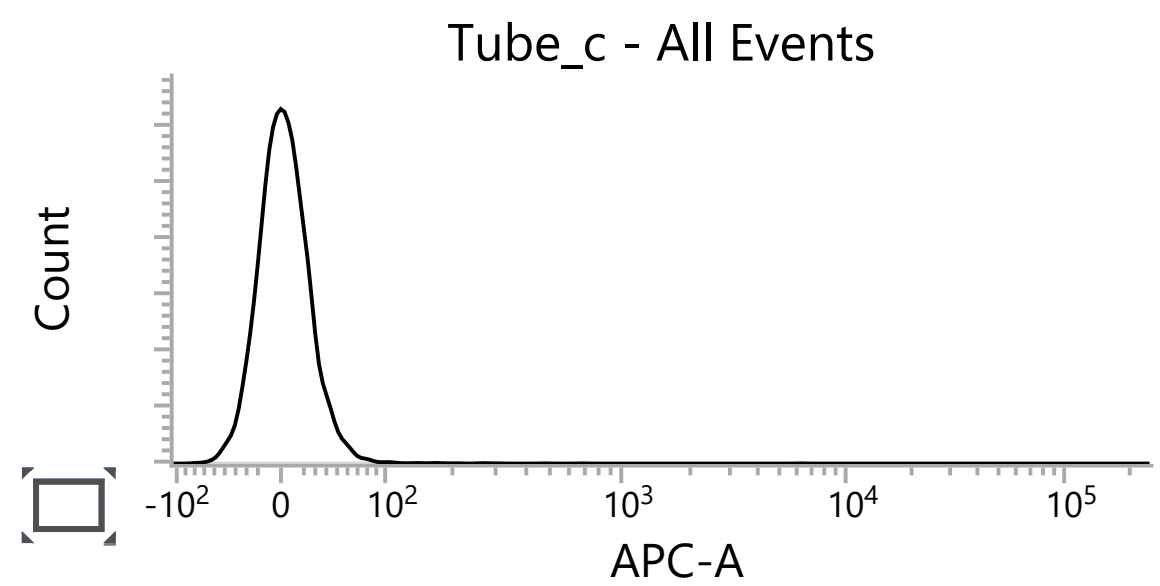

Statistics

Name	Events	% Parent	% Grandparent	% Total
Tube_a:All Events	10,000	***	***	100.00
Tube_b:All Events	10,000	***	***	100.00
Tube_c:All Events	10,000	***	***	100.00
Tube_a:Lebende Zellen	7,046	70.46	***	70.46
Tube_b:Lebende Zellen	6,301	63.01	***	63.01
Tube_c:Lebende Zellen	5,599	55.99	***	55.99
Tube_a:Dead	45	0.45	***	0.45
Tube_a:++	1	0.01	***	0.01
Tube_a:Live	6,513	65.13	***	65.13
Tube_a:ROS+	3,441	34.41	***	34.41
Tube_a:Dead	4	0.06	0.04	0.04
Tube_a:++	0	0.00	0.00	0.00
Tube_a:Live	3,615	51.31	36.15	36.15
Tube_a:ROS+	3,427	48.64	34.27	34.27
Tube_b:Dead	80	0.80	***	0.80
Tube_b:++	1	0.01	***	0.01
Tube_b:Live	6,973	69.73	***	69.73
Tube_b:ROS+	2,946	29.46	***	29.46
Tube_b:Dead	4	0.06	0.04	0.04
Tube_b:++	0	0.00	0.00	0.00
Tube_b:Live	3,363	53.37	33.63	33.63
Tube_b:ROS+	2,934	46.56	29.34	29.34
Tube_c:Dead	69	0.69	***	0.69
Tube_c:++	1	0.01	***	0.01
Tube_c:Live	6,546	65.46	***	65.46
Tube_c:ROS+	3,384	33.84	***	33.84
Tube_c:Dead	8	0.14	0.08	0.08
Tube_c:++	0	0.00	0.00	0.00
Tube_c:Live	2,246	40.11	22.46	22.46
Tube_c:ROS+	3,345	59.74	33.45	33.45

LDT

CellROX Assay

Sample ID: N6-ATM_D

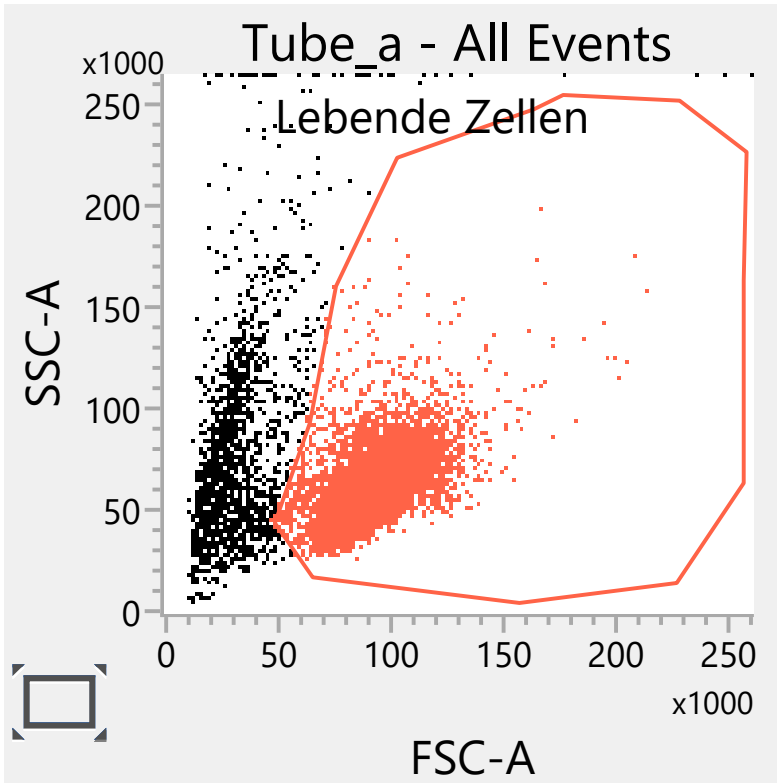

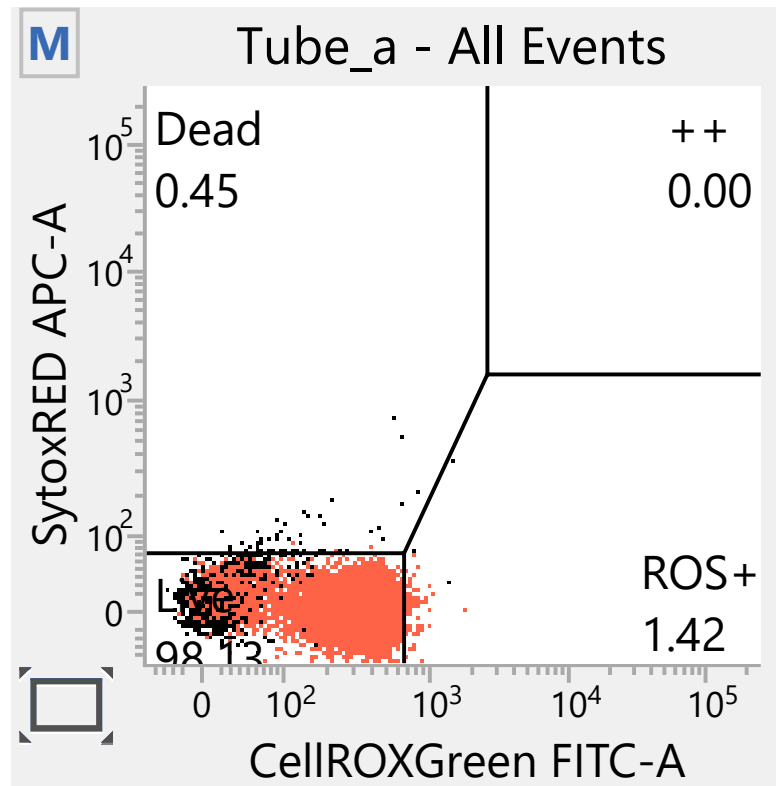

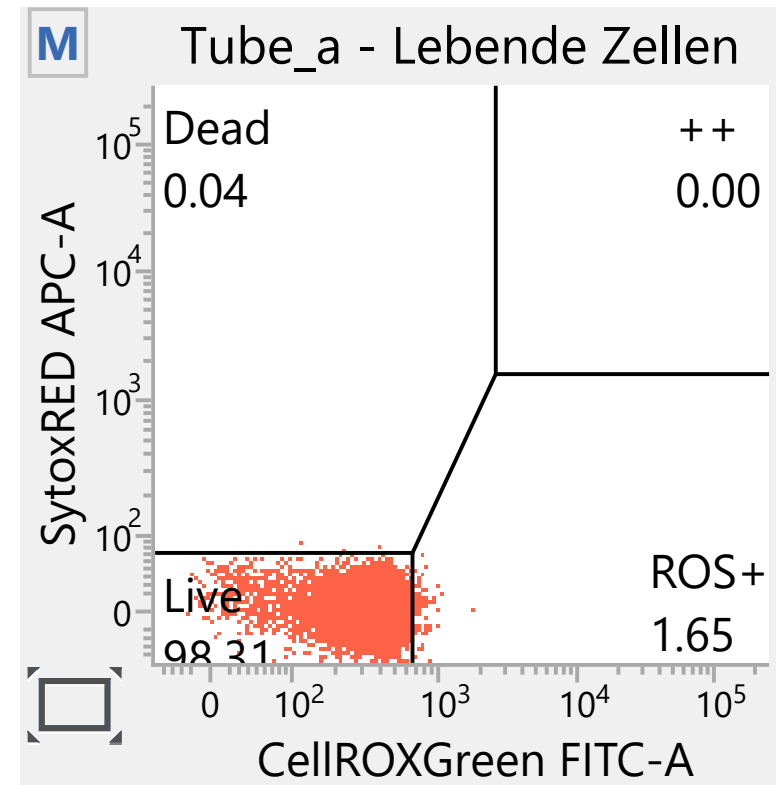

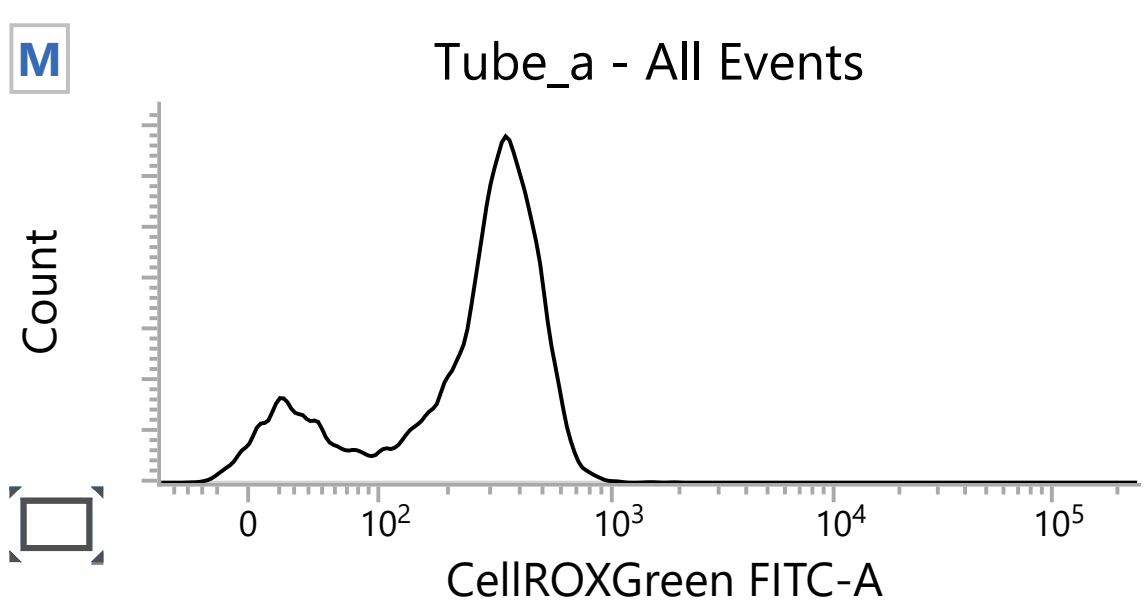

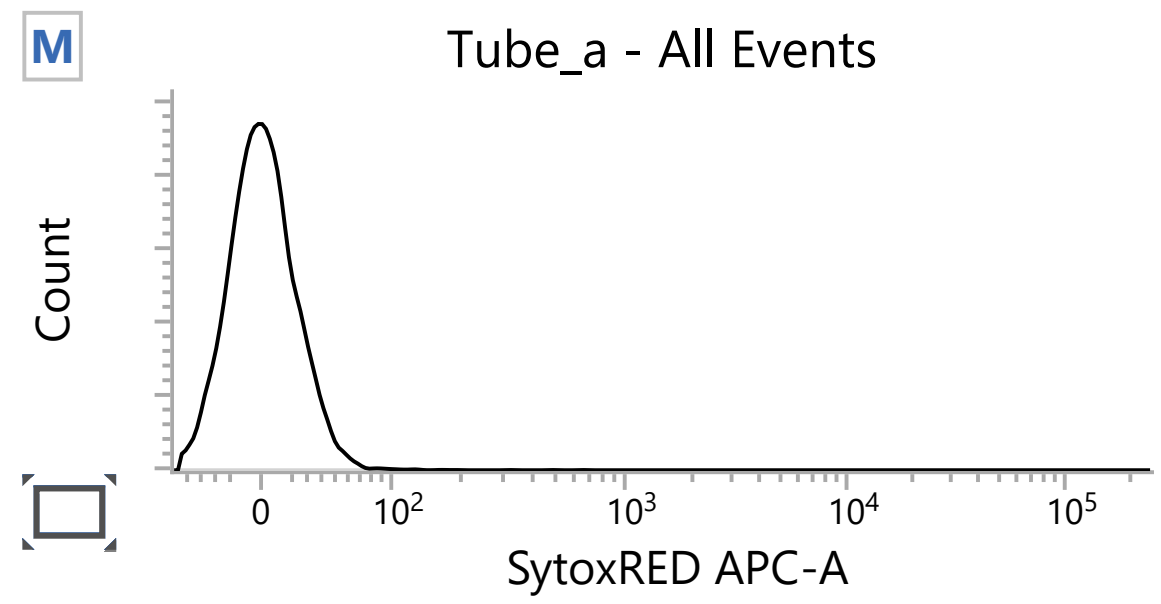

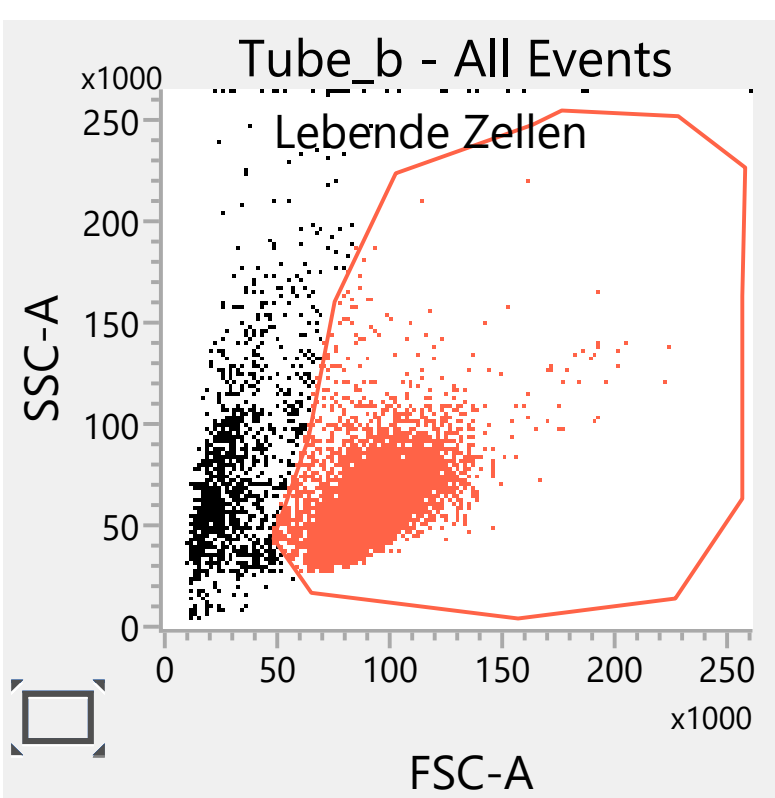

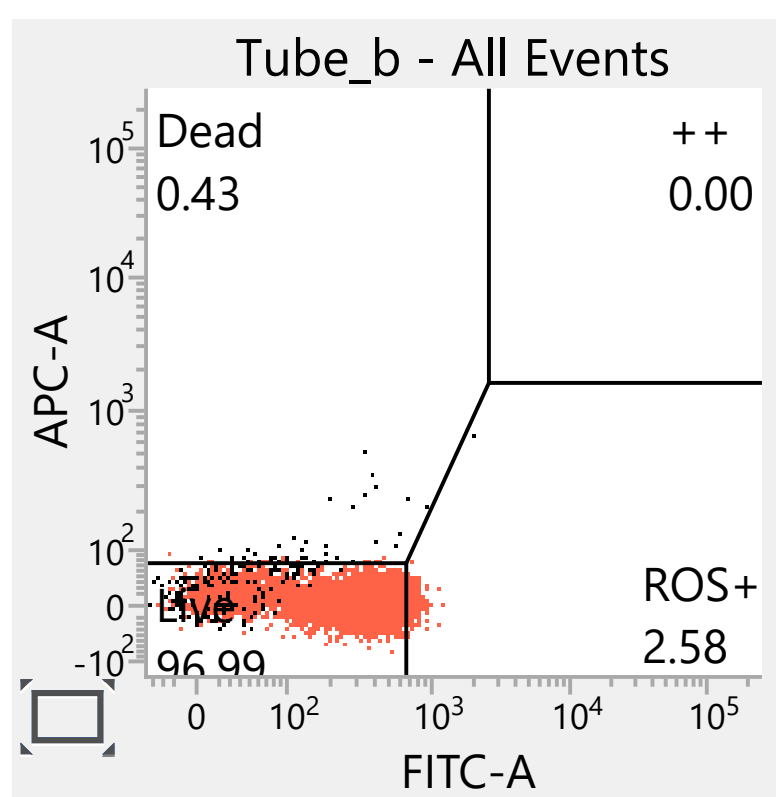

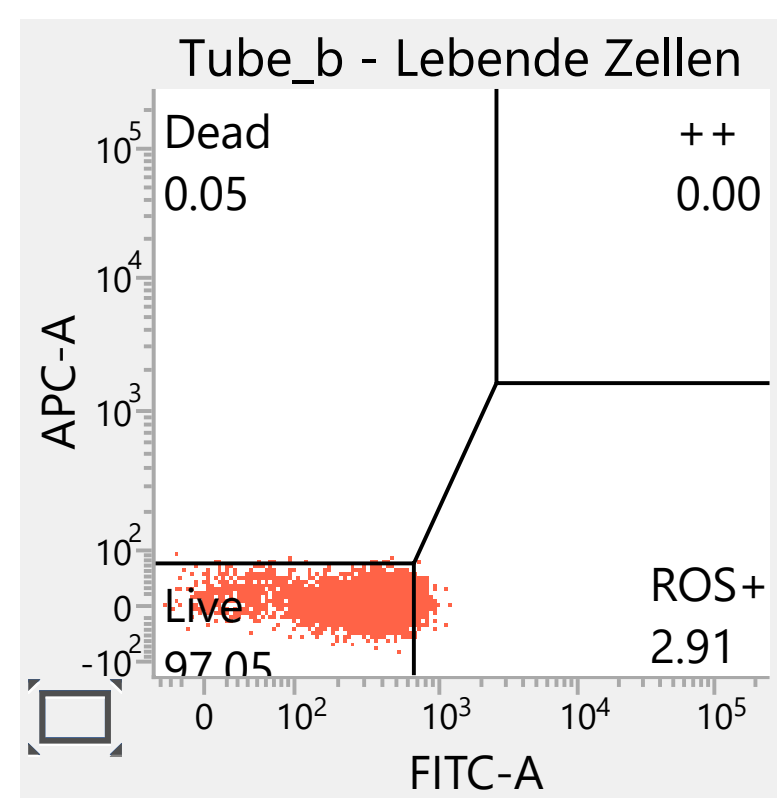

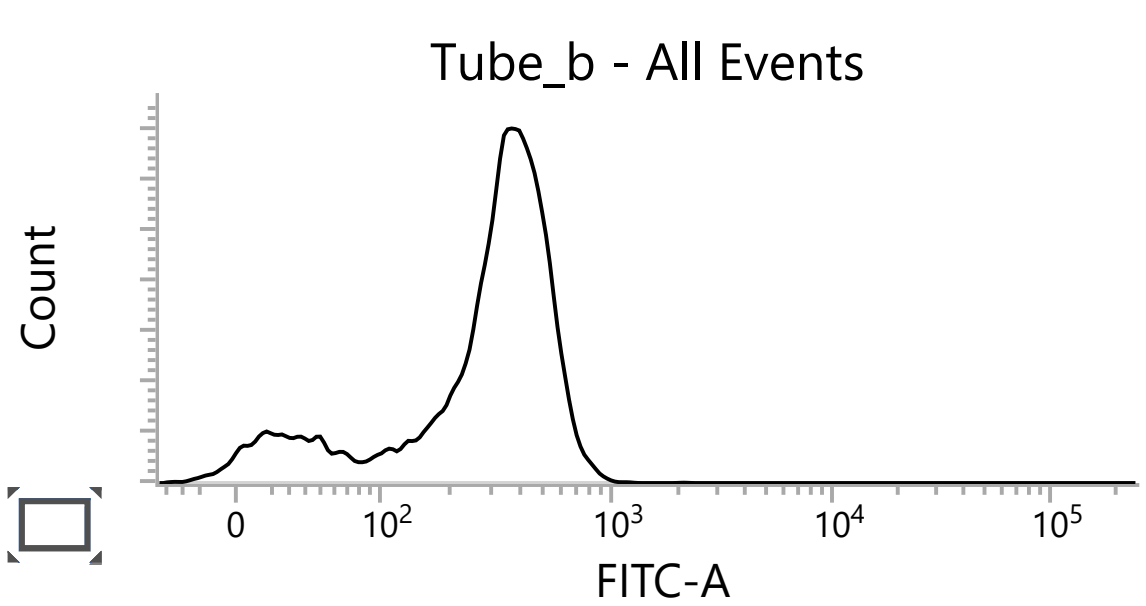

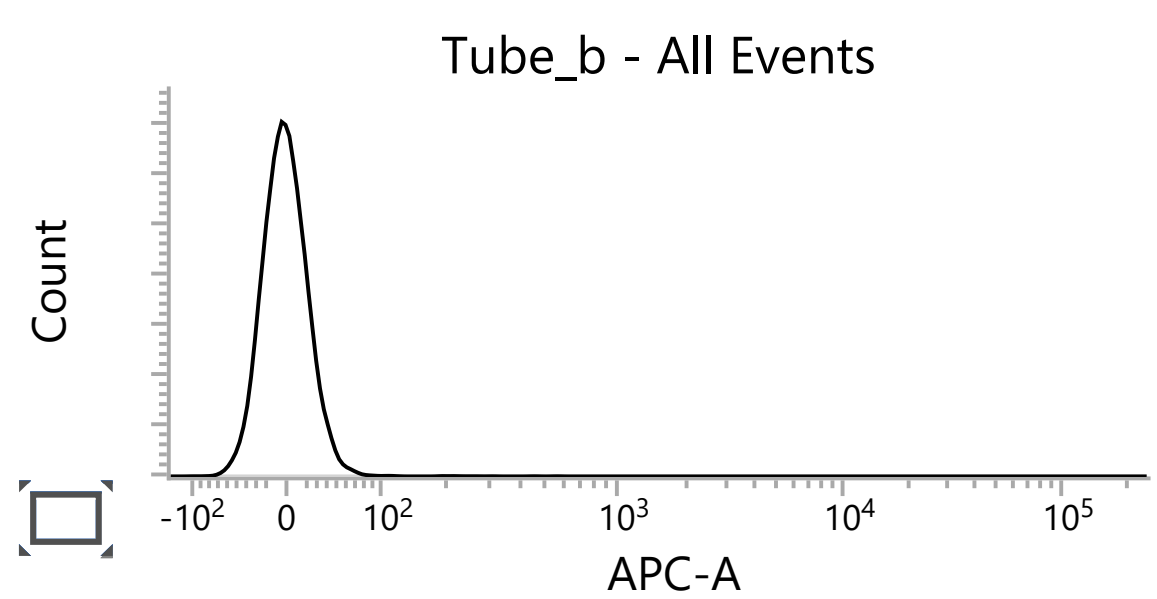

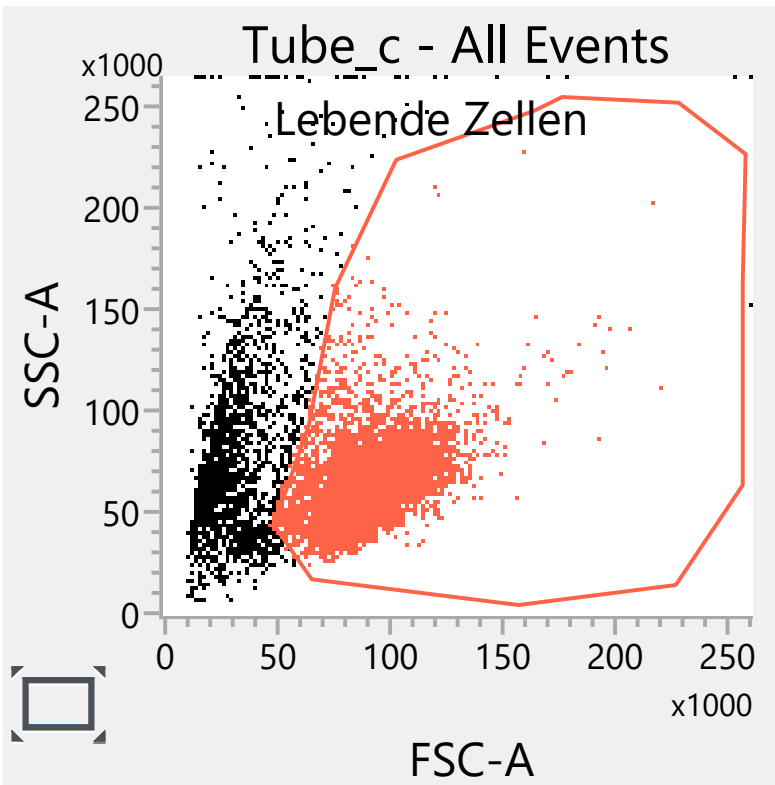

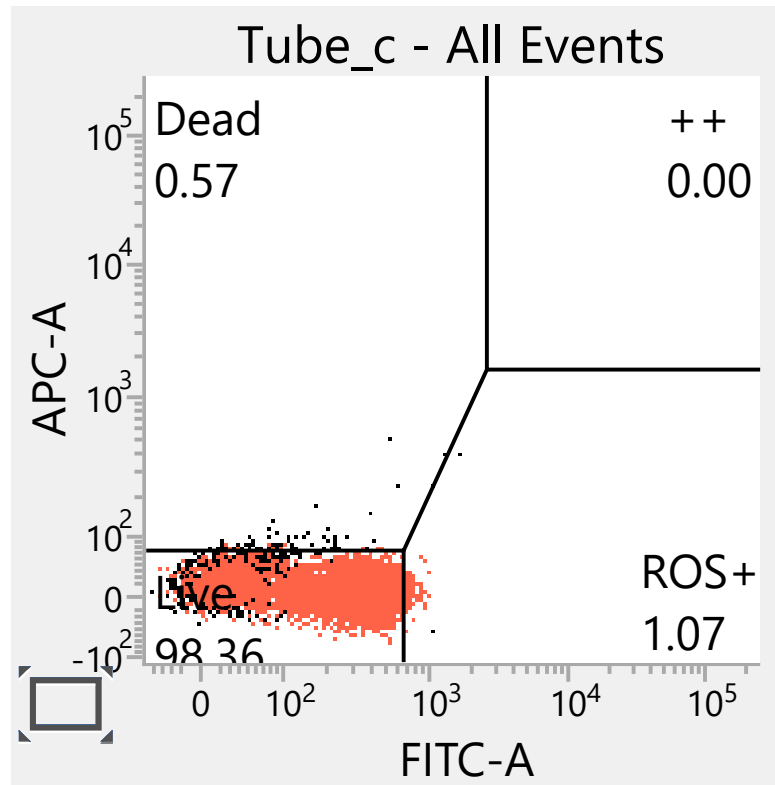

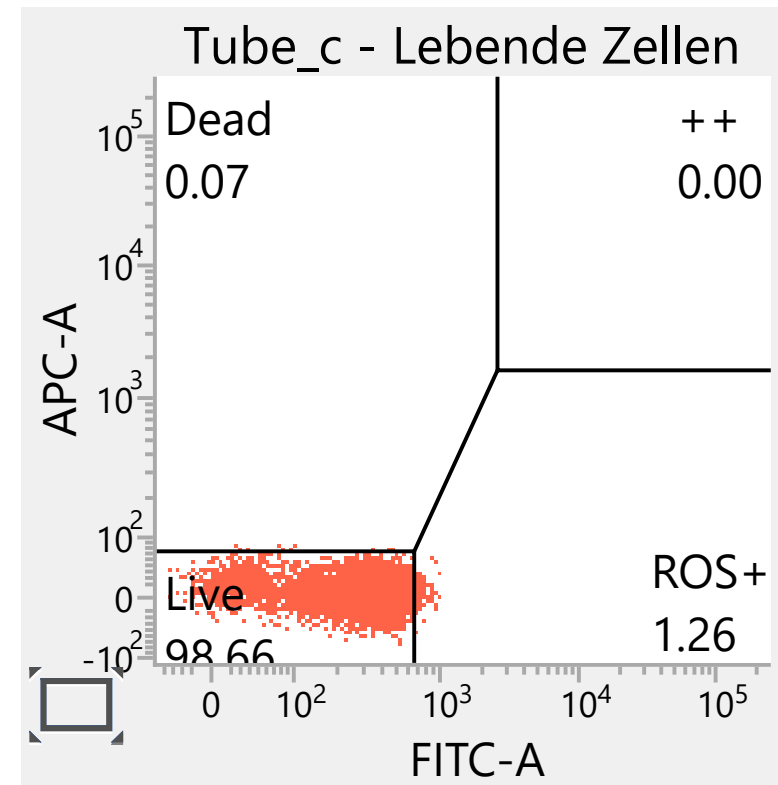

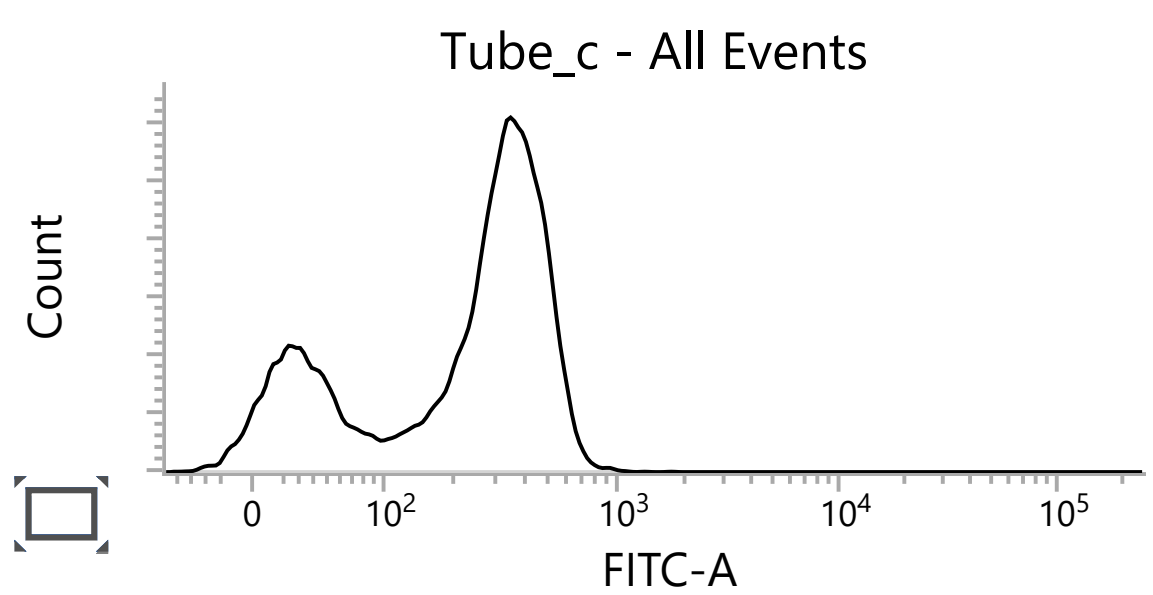

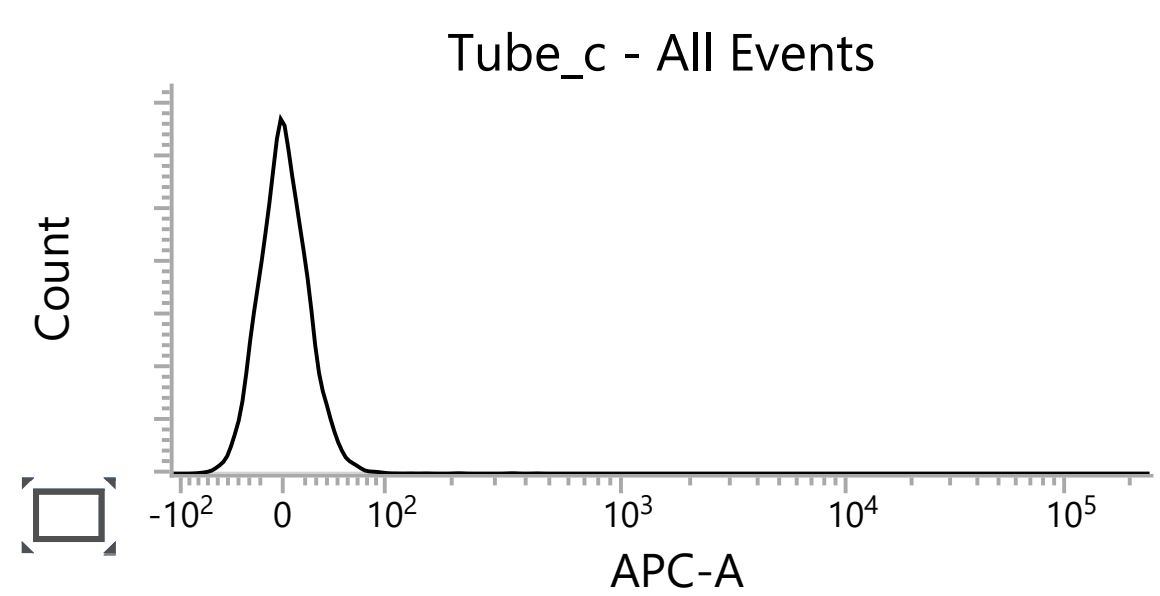

Statistics

Name	Events	% Parent	% Grandparent	% Total
Tube_b:All Events	10,000	***	***	100.00
Tube_c:All Events	10,000	***	***	100.00
Tube_a:All Events	10,000	***	***	100.00
Tube_a:Lebende Zellen	8,359	83.59	***	83.59
Tube_b:Lebende Zellen	8,834	88.34	***	88.34
Tube_c:Lebende Zellen	8,151	81.51	***	81.51

CellROX Assay

Sample ID: N6-ATM_H2O2 3yM NAC

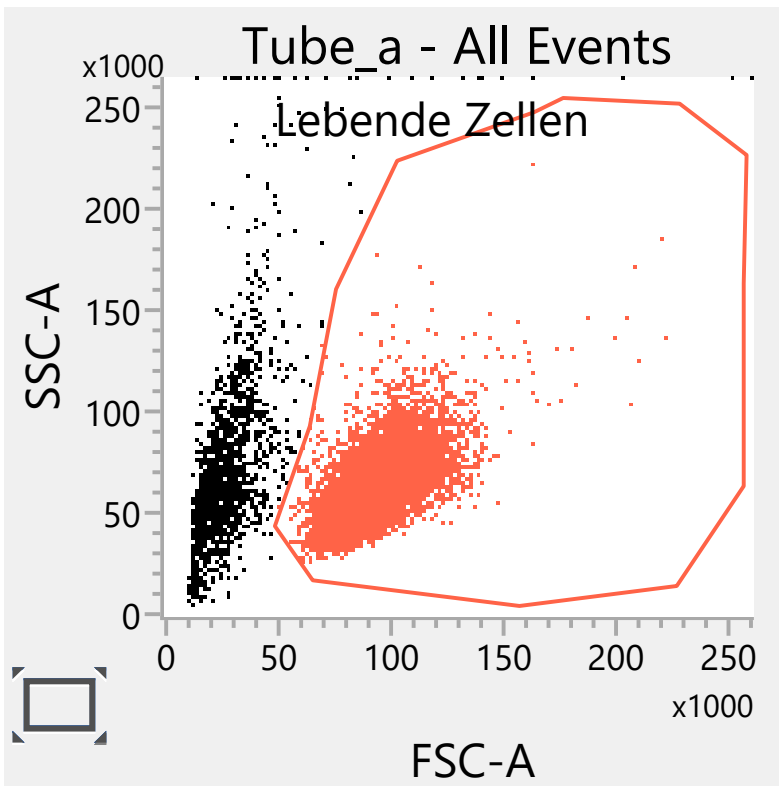

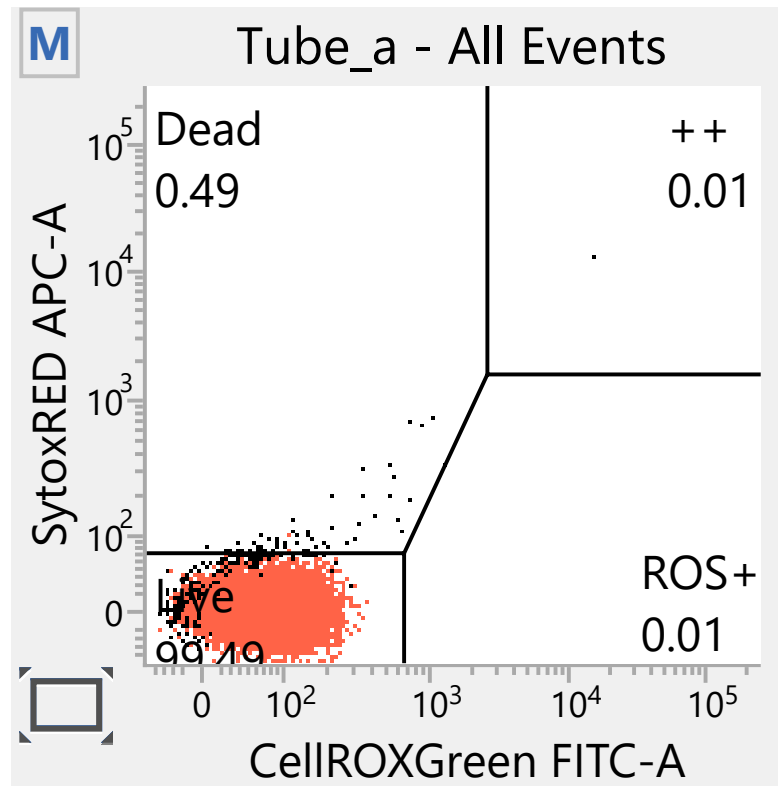

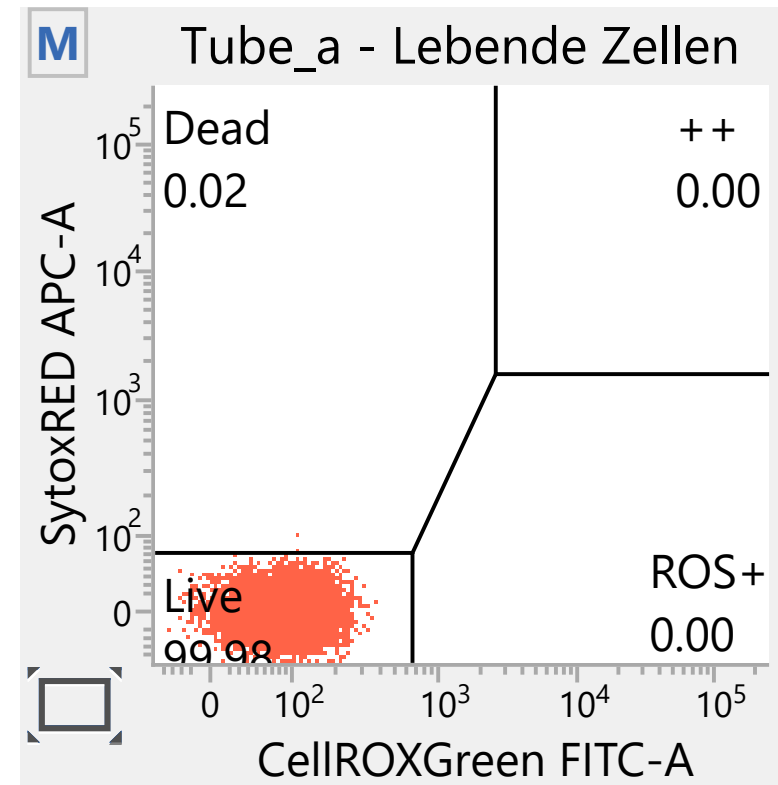

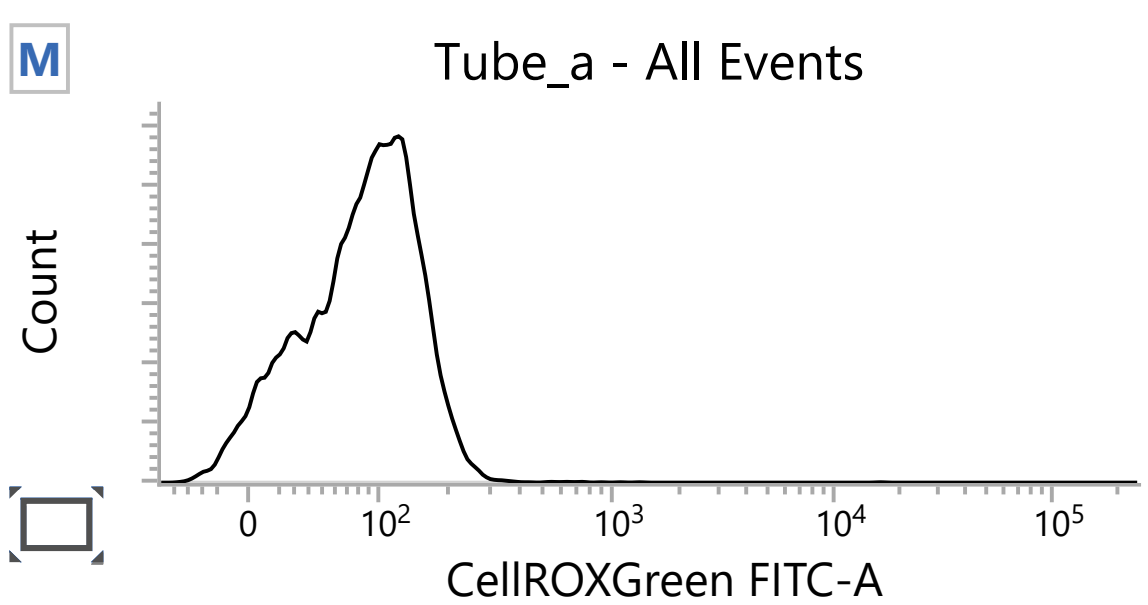

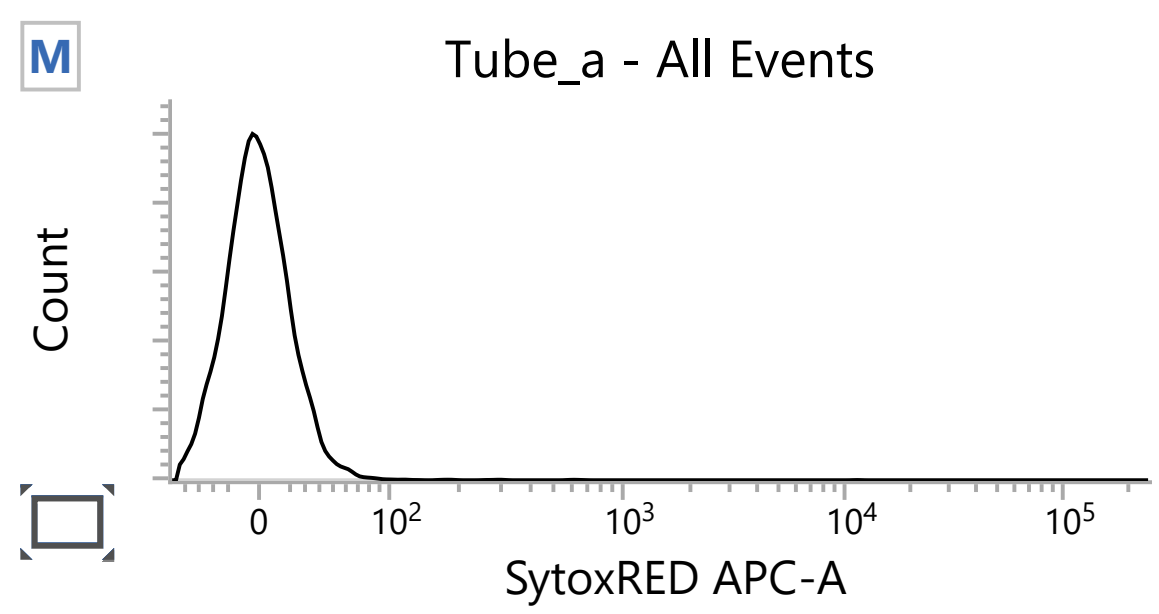

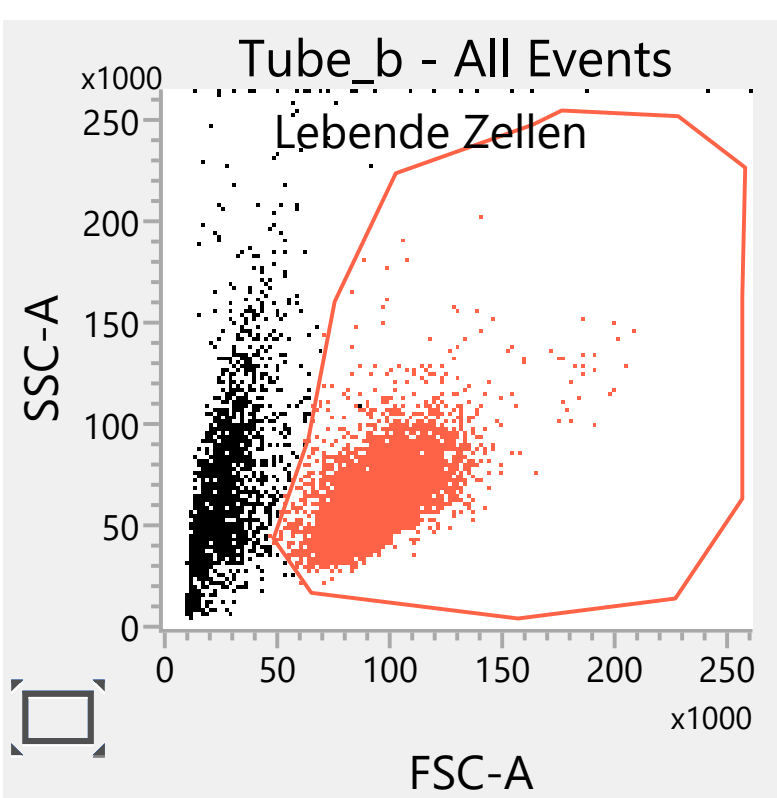

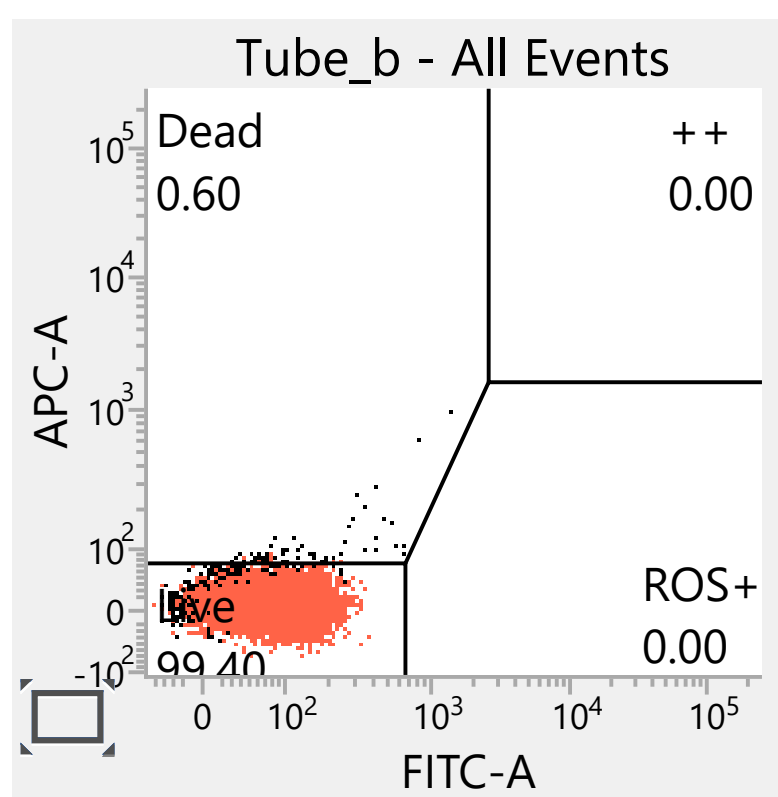

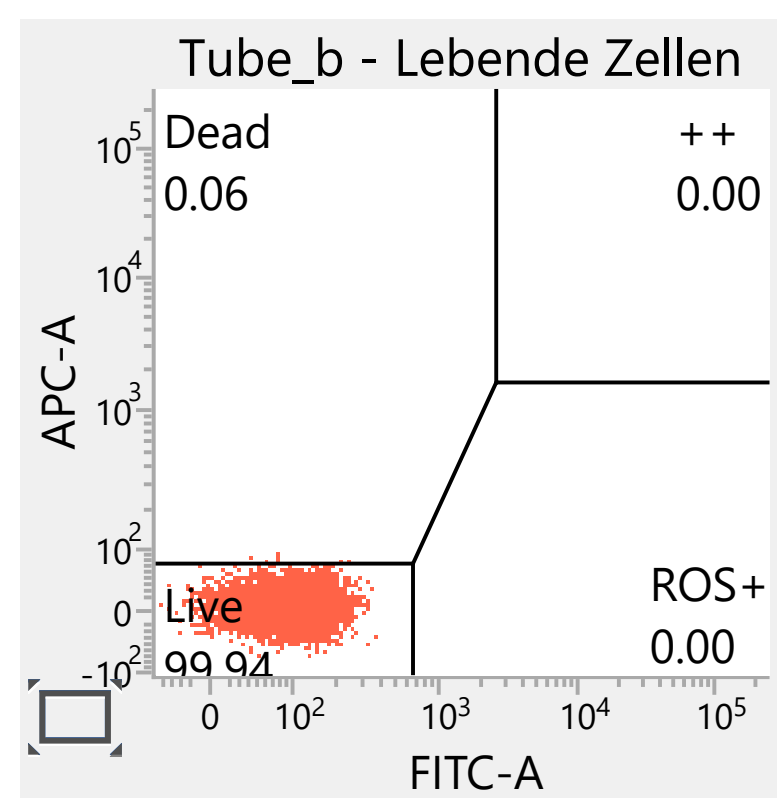

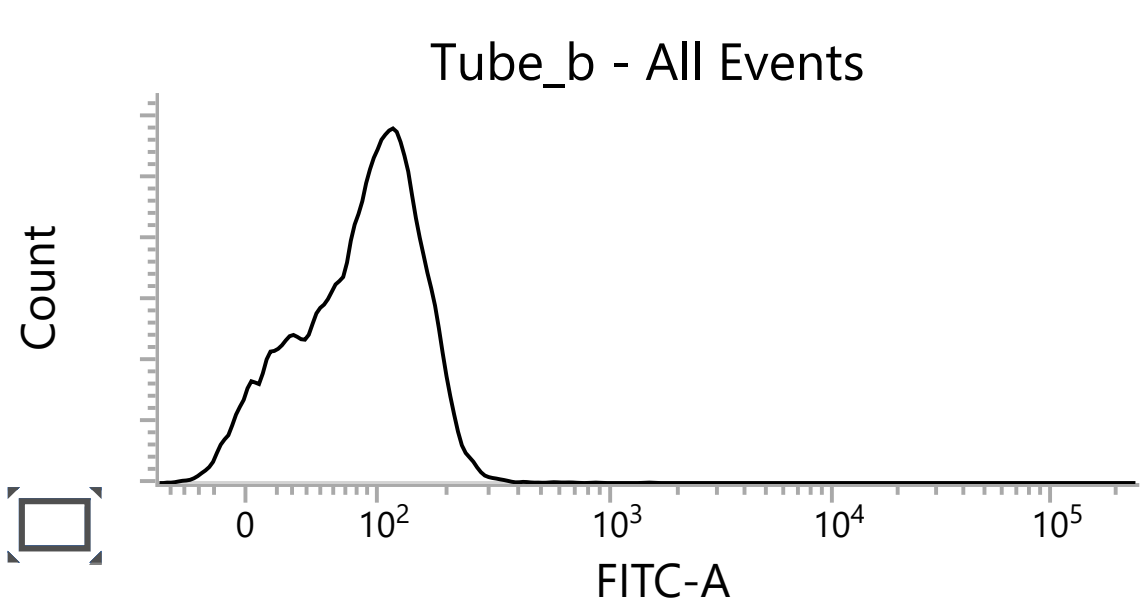

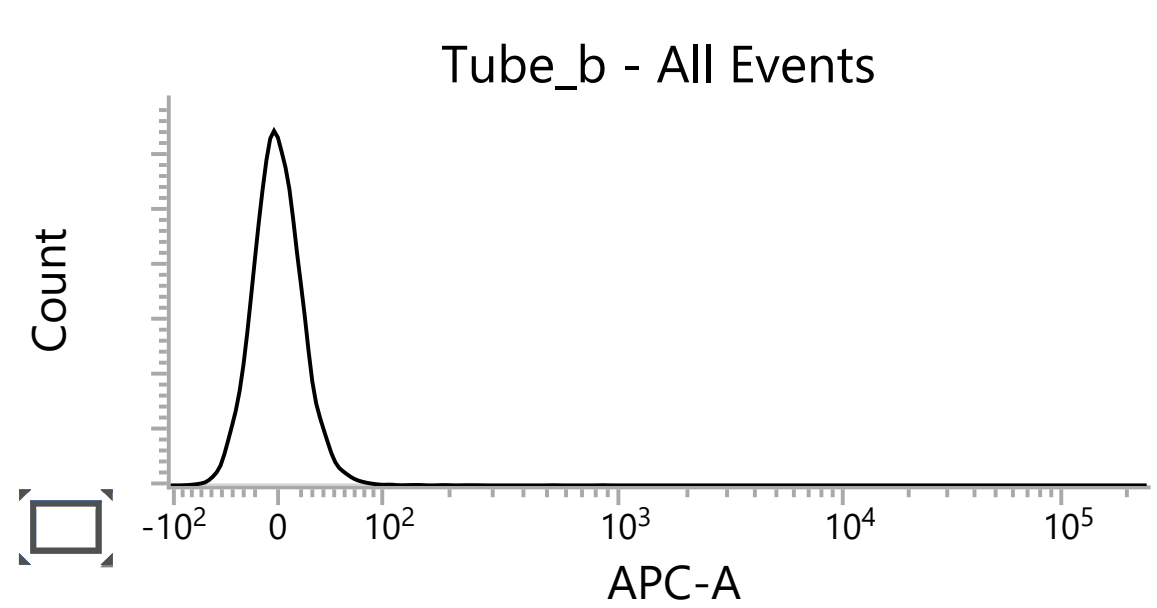

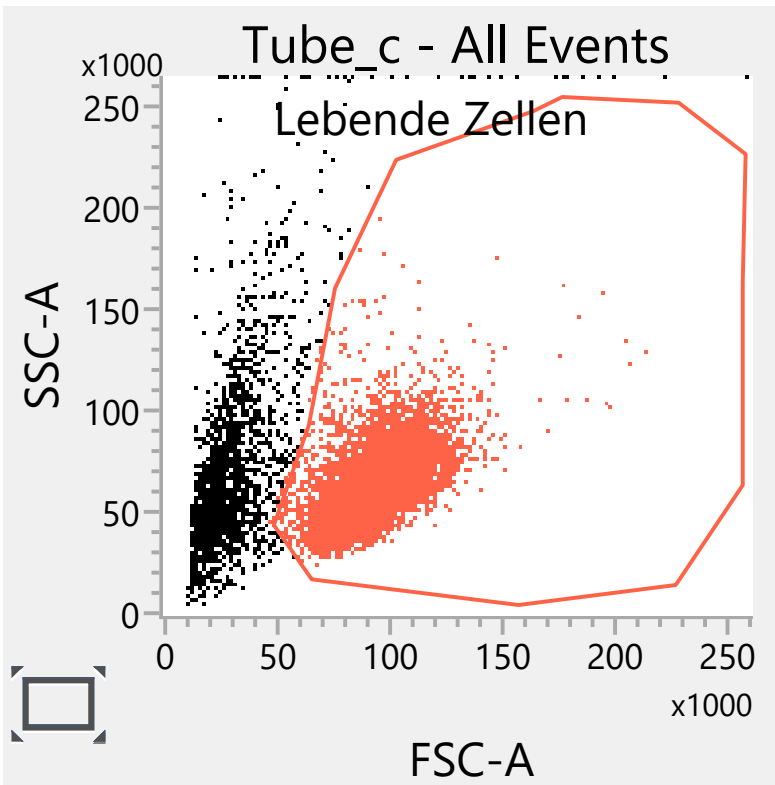

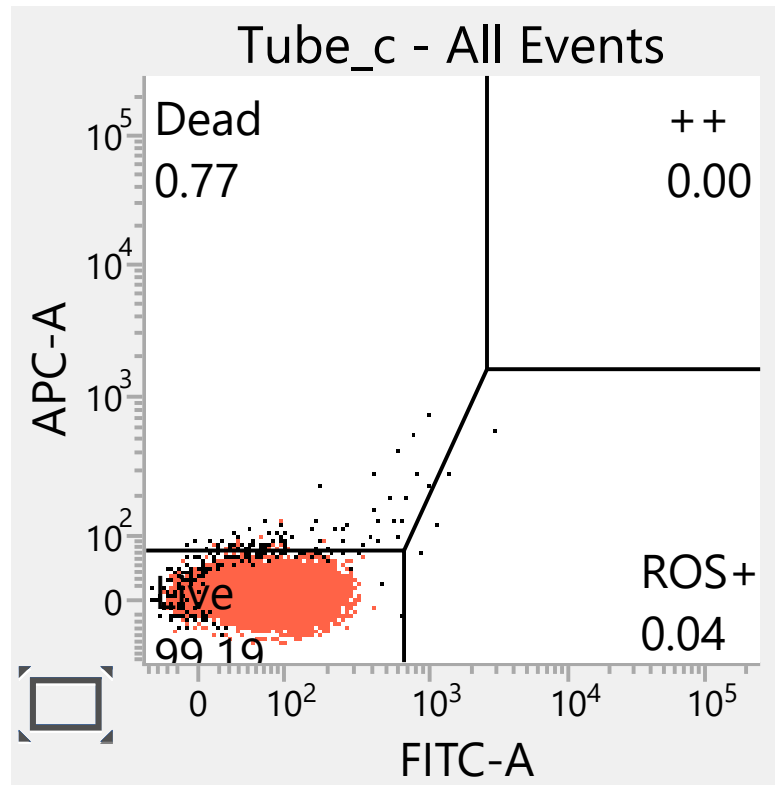

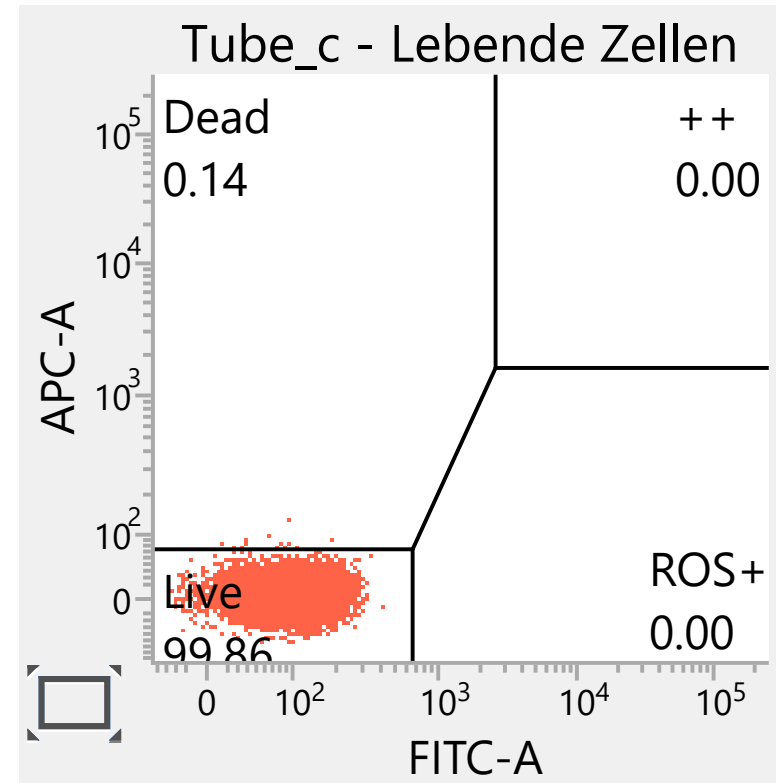

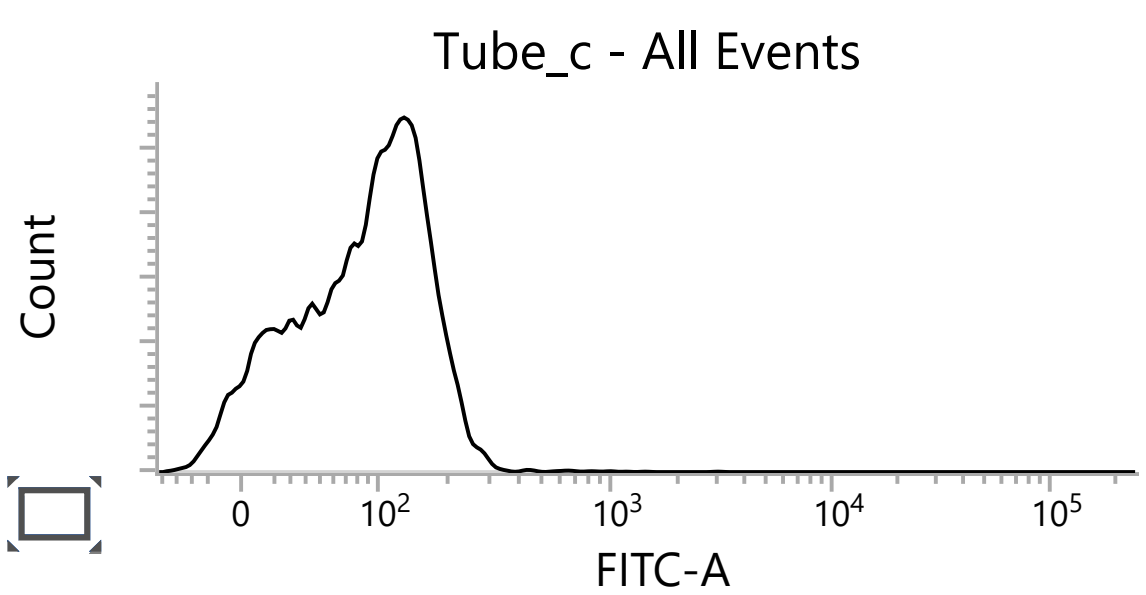

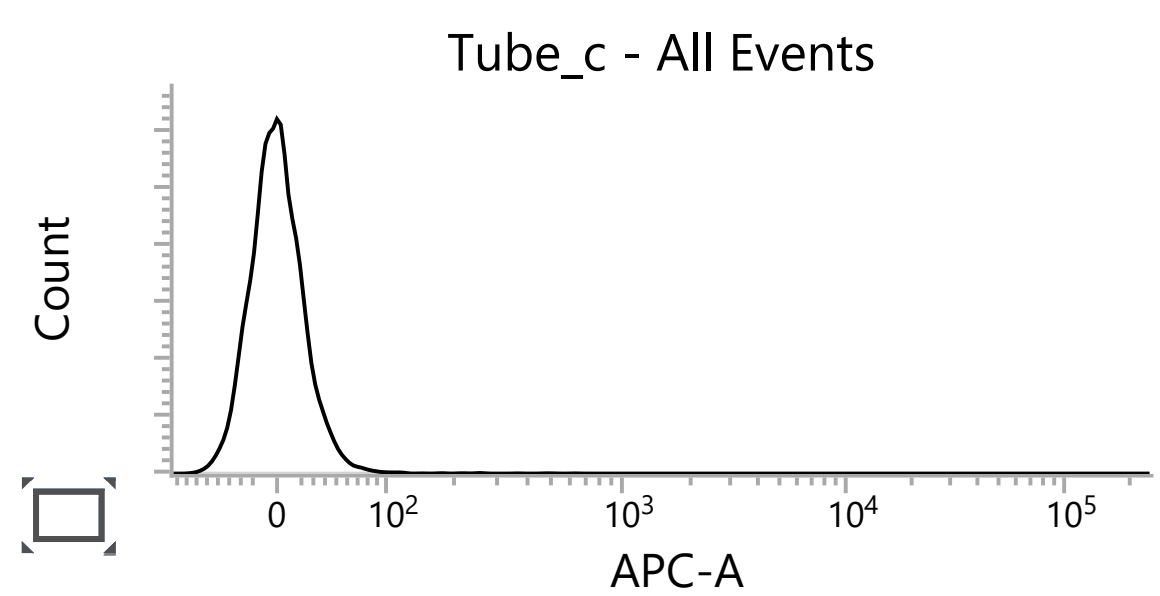

Statistics

Name	Events	% Parent	% Grandparent	% Total
Tube_a:All Events	10,000	***	***	100.00
Tube_b:All Events	10,000	***	***	100.00
Tube_c:All Events	10,000	***	***	100.00
Tube_a:Lebende Zellen	8,526	85.26	***	85.26
Tube_b:Lebende Zellen	8,209	82.09	***	82.09
Tube_c:Lebende Zellen	8,136	81.36	***	81.36

CellROX Assay

Sample ID: N6-ATM_H2O2 3yM

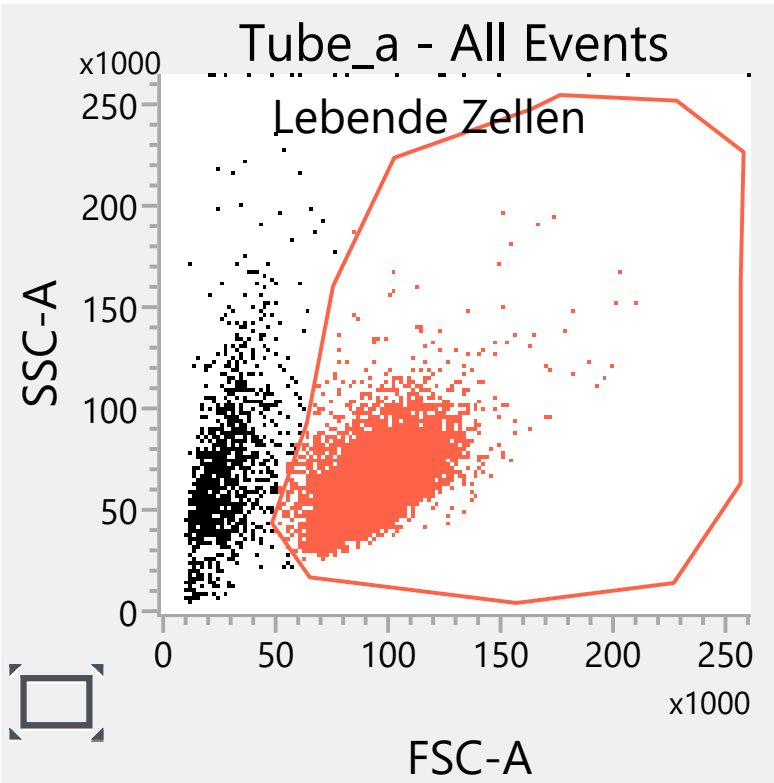

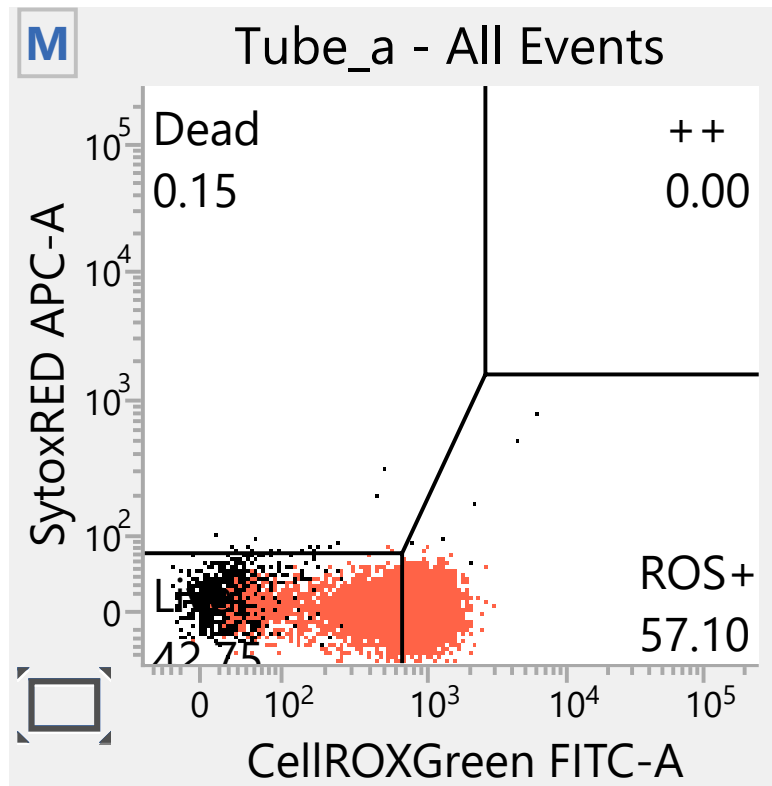

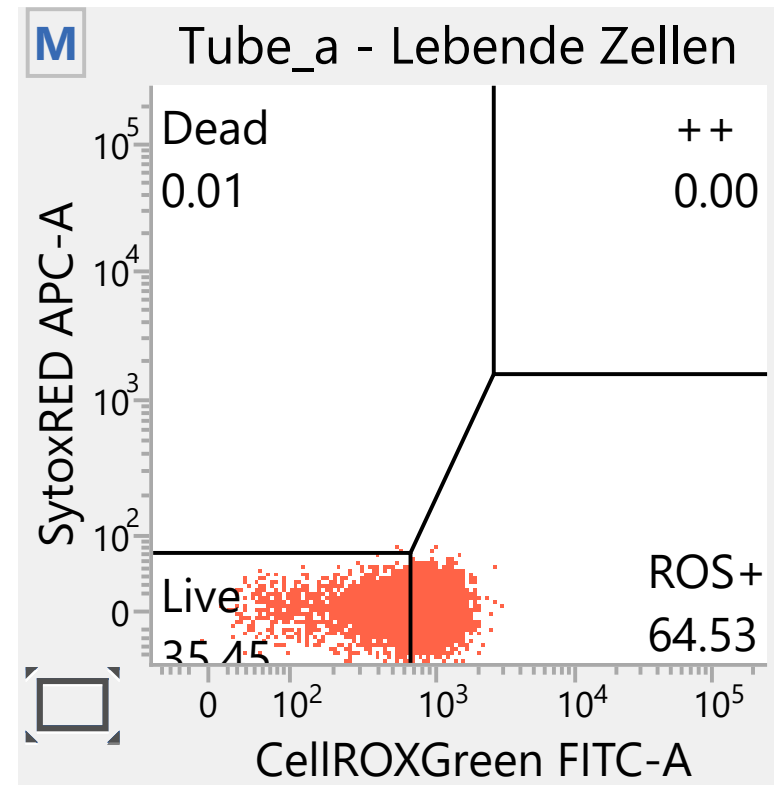

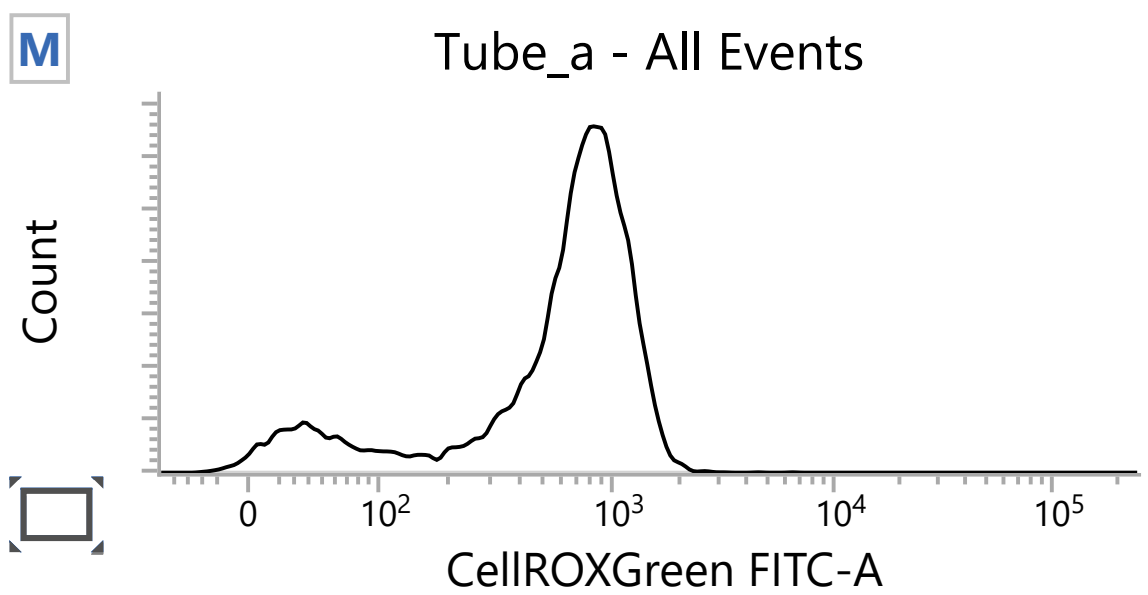

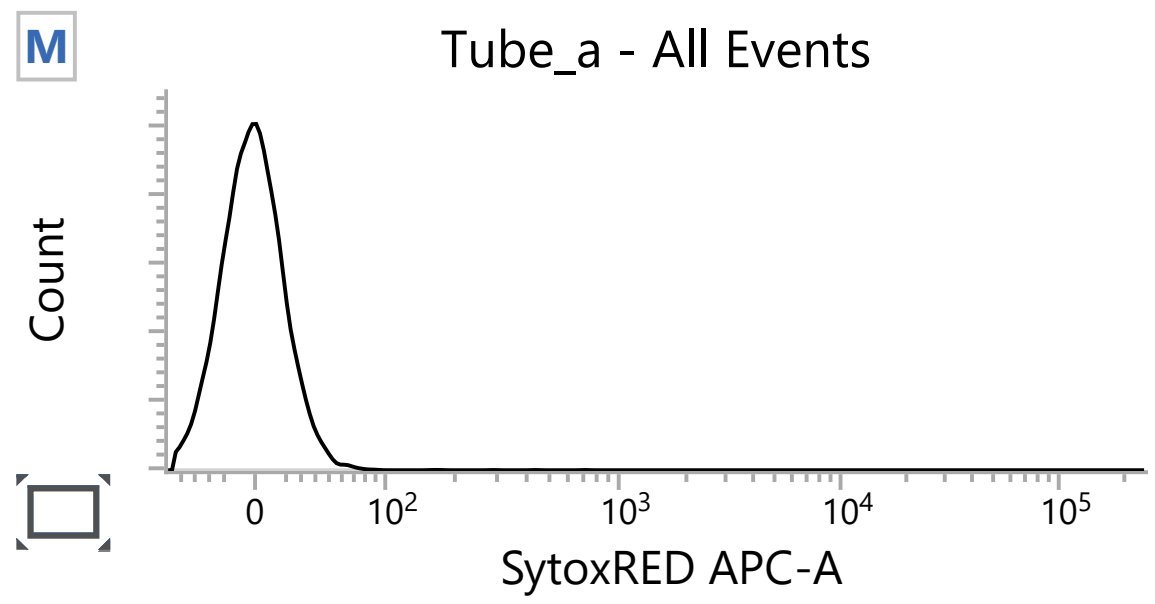

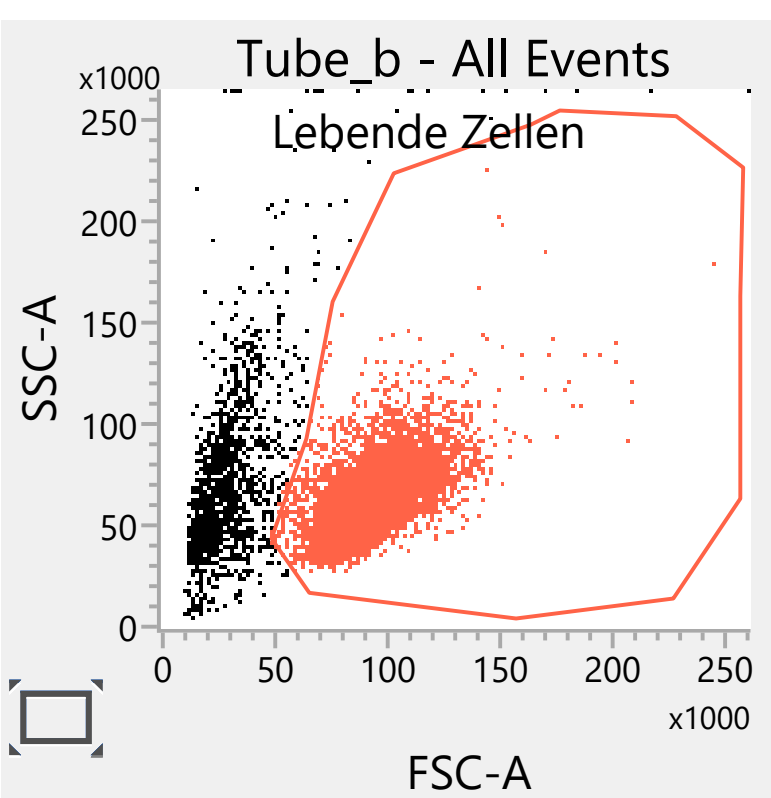

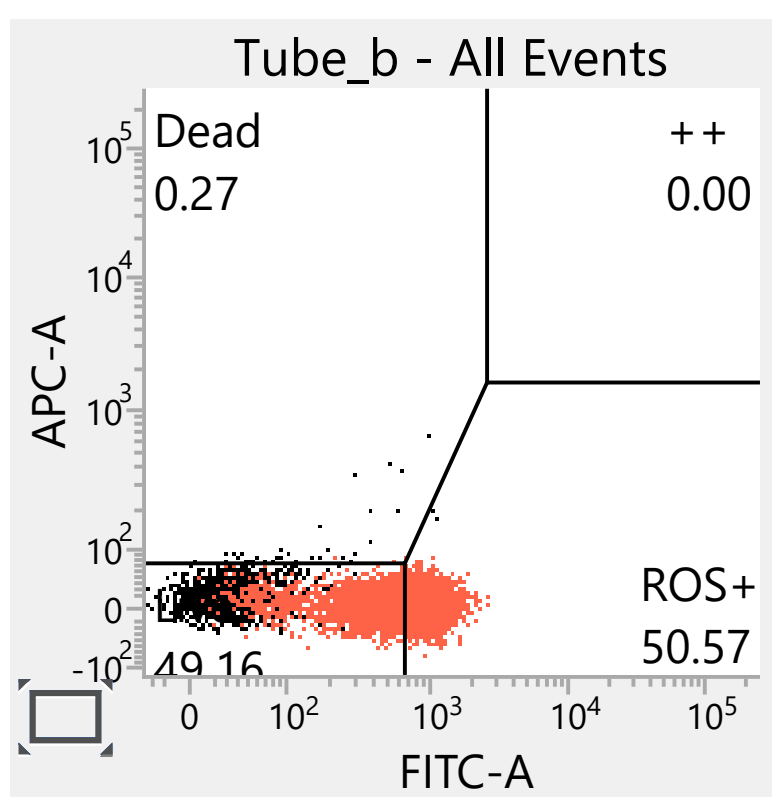

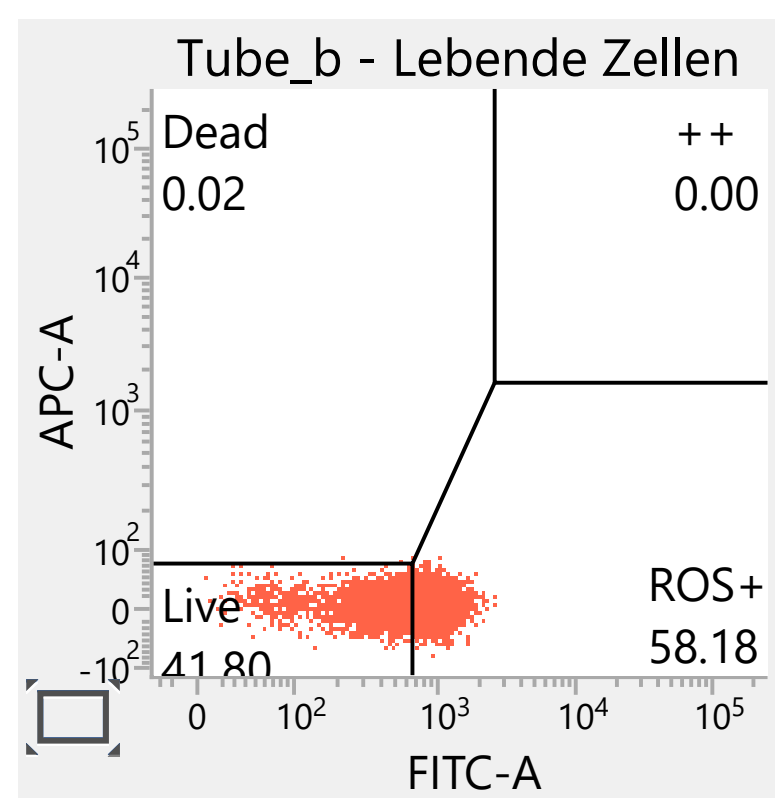

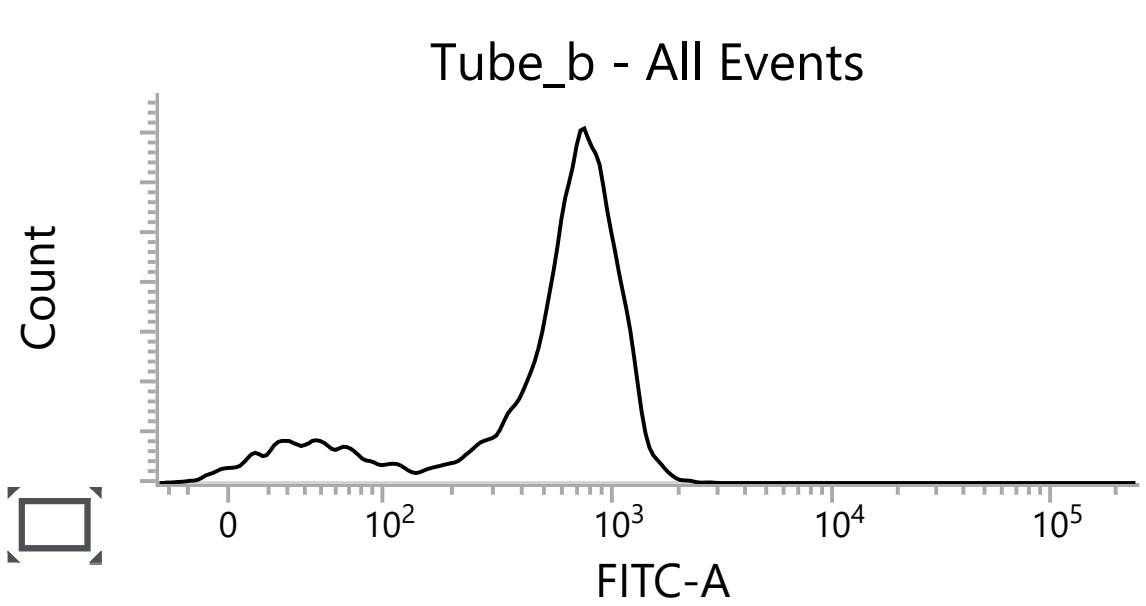

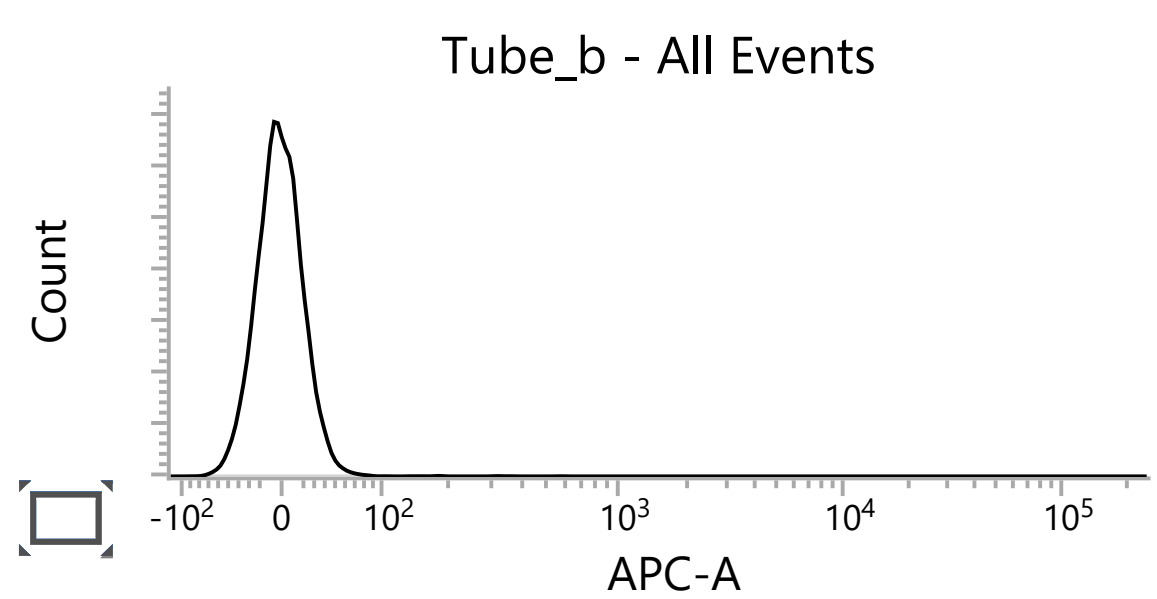

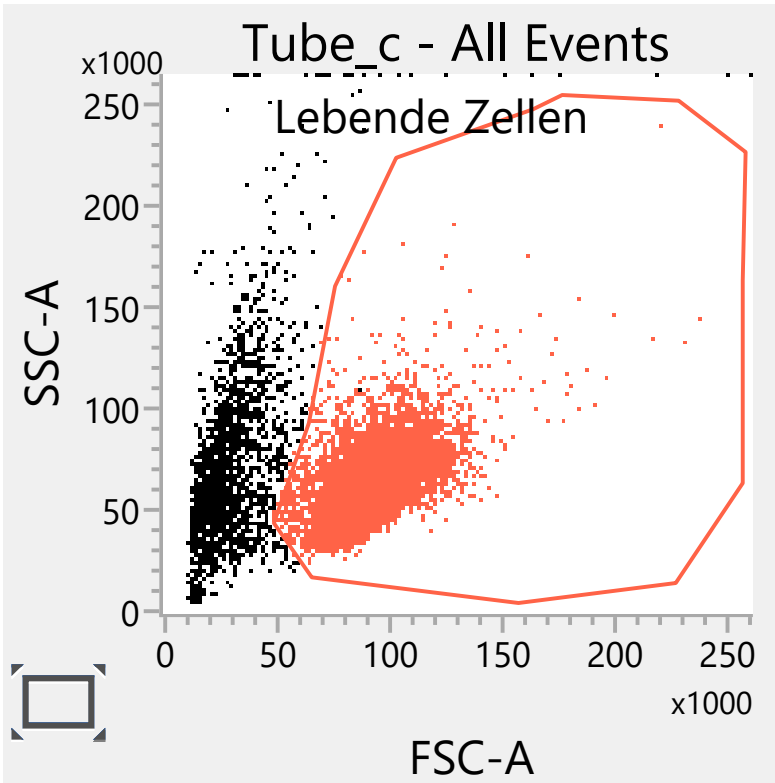

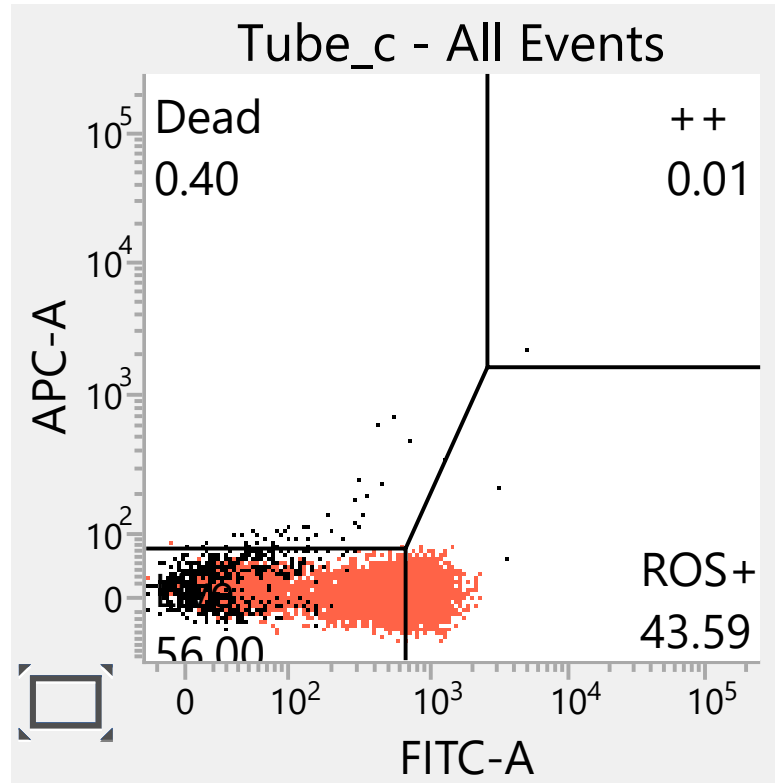

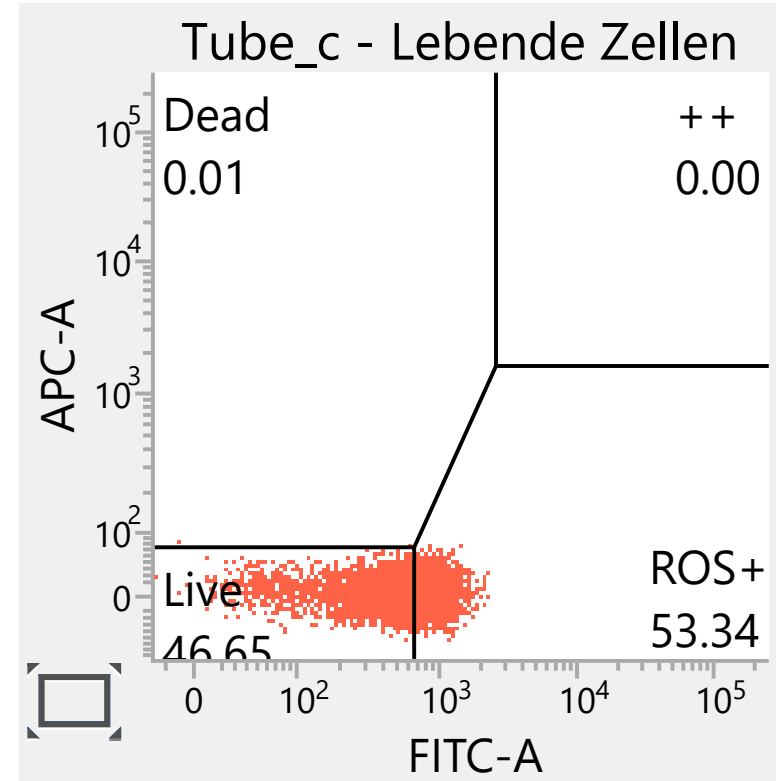

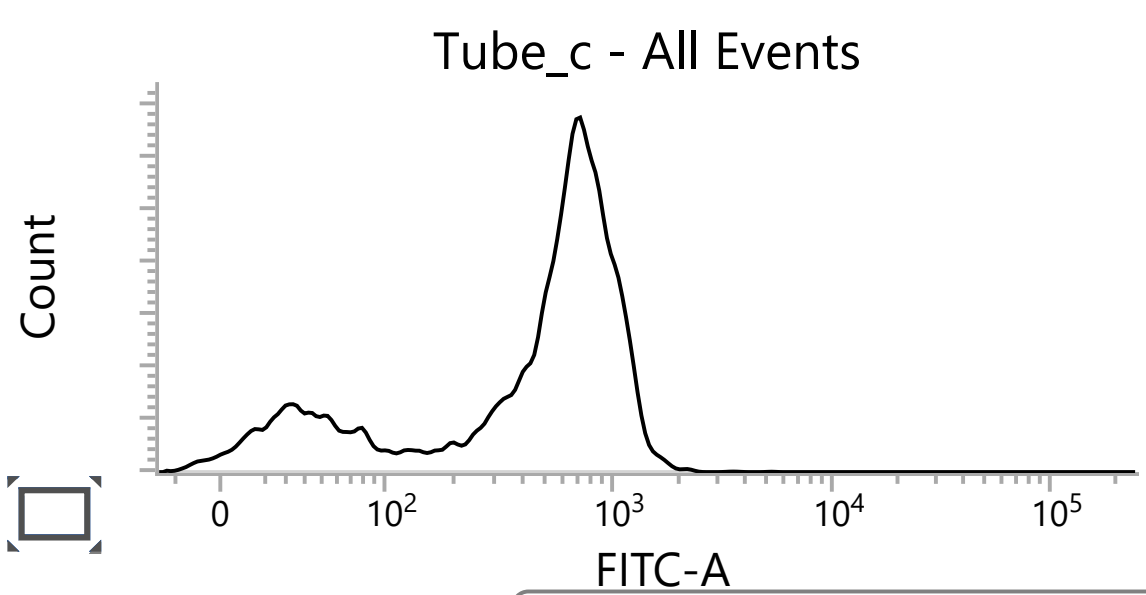

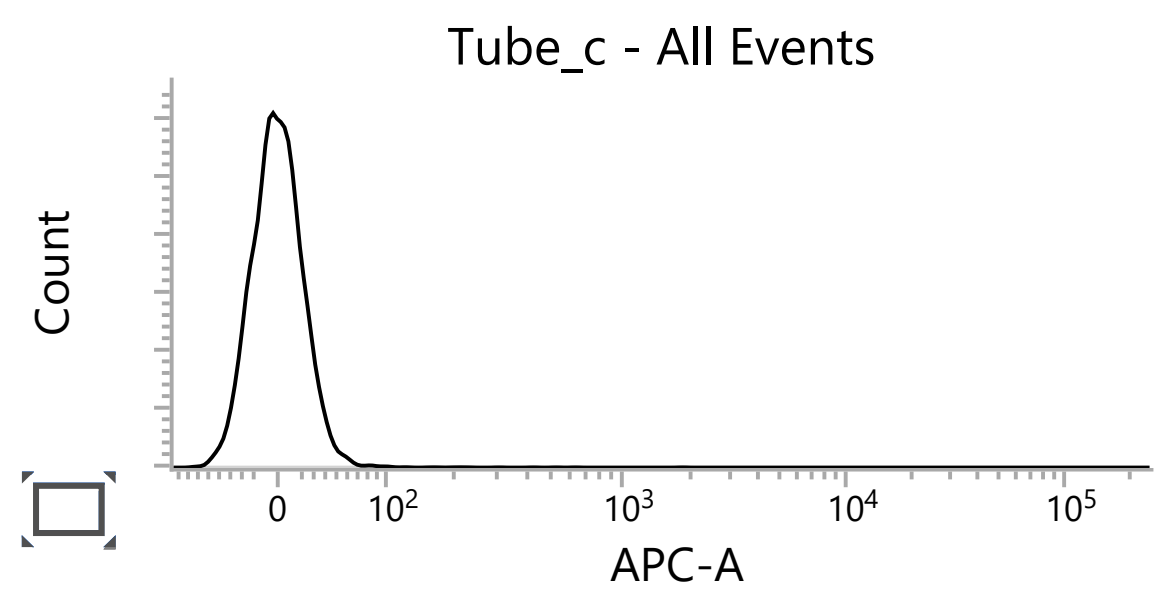

Statistics

Name	Events	% Parent	% Grandparent	% Total
Tube_a:All Events	10,000	***	***	100.00
Tube_b:All Events	10,000	***	***	100.00
Tube_c:All Events	10,000	***	***	100.00
Tube_a:Lebende Zellen	8,834	88.34	***	88.34
Tube_b:Lebende Zellen	8,684	86.84	***	86.84
Tube_c:Lebende Zellen	8,163	81.63	***	81.63

CellROX Assay

Sample ID: N6-ATM_H2O2 4yM NAC

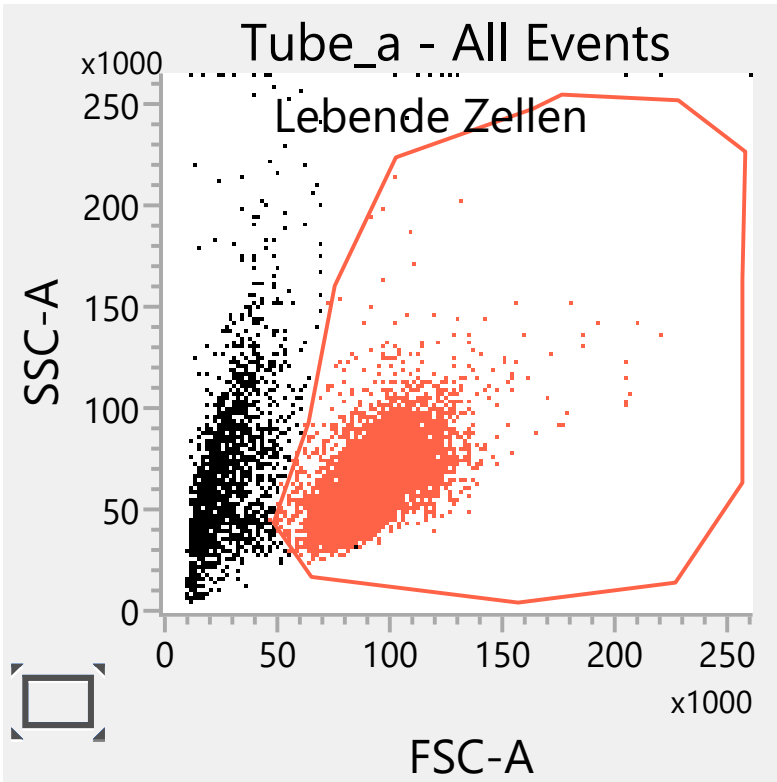

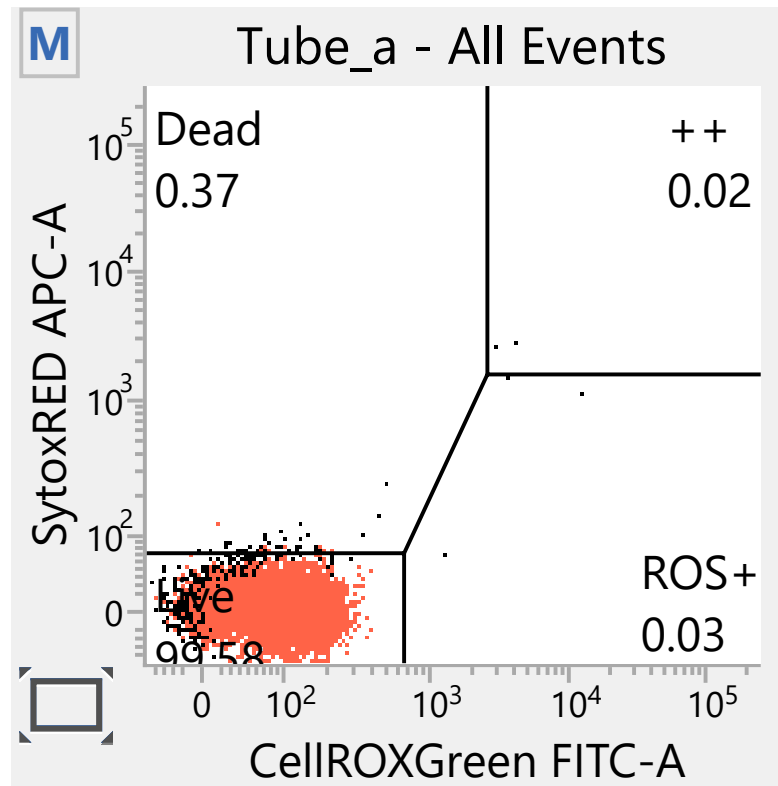

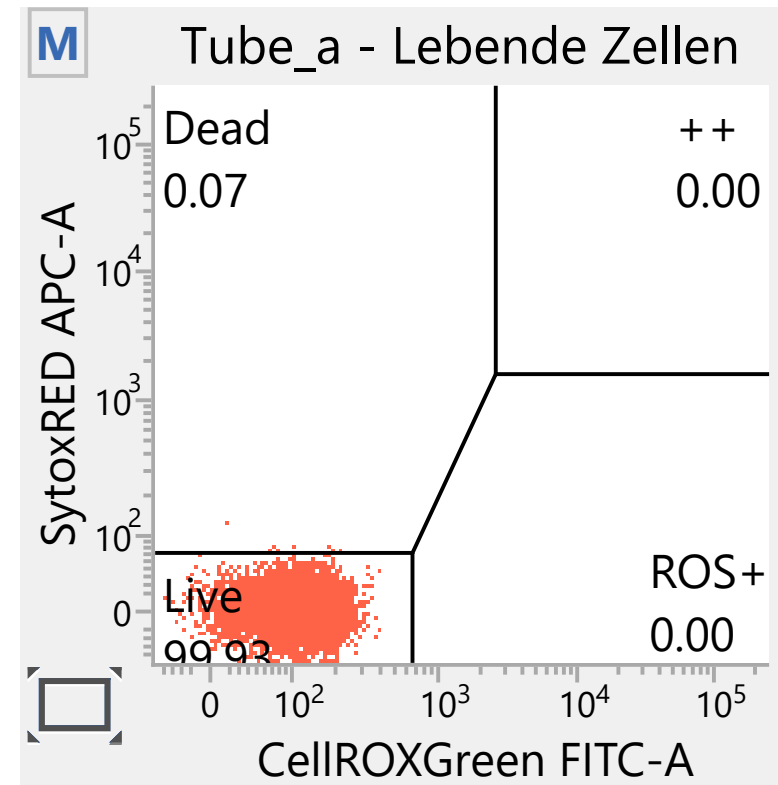

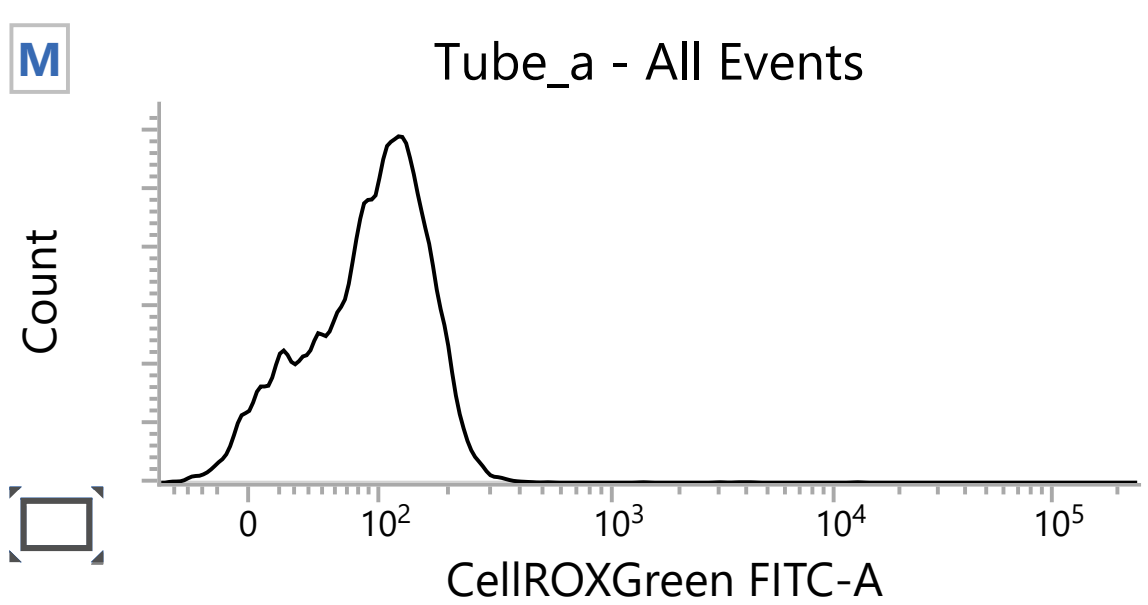

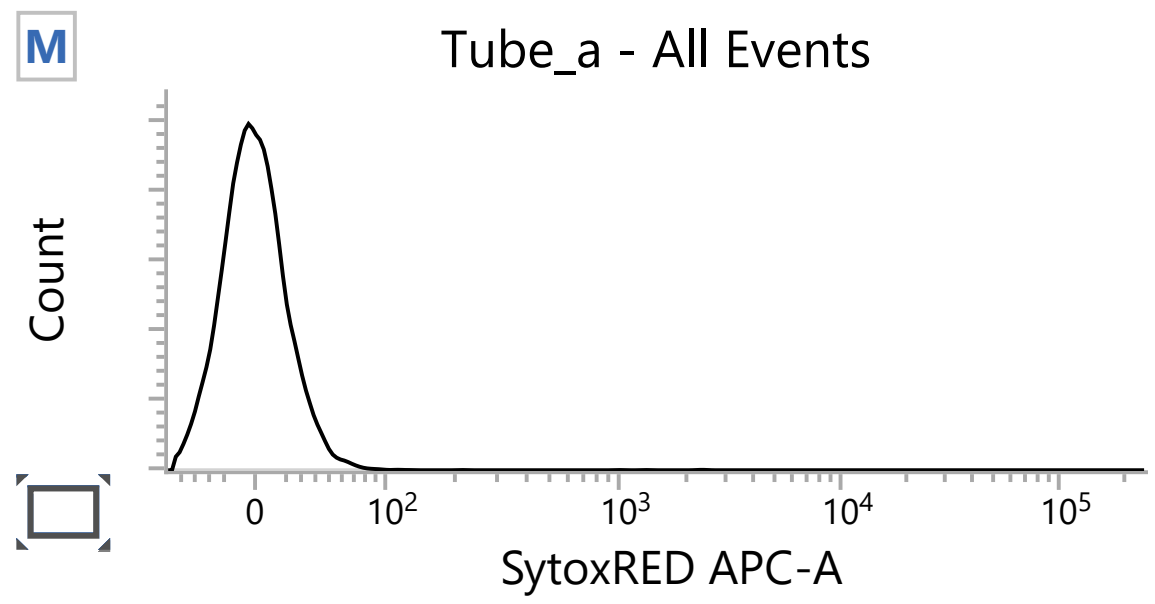

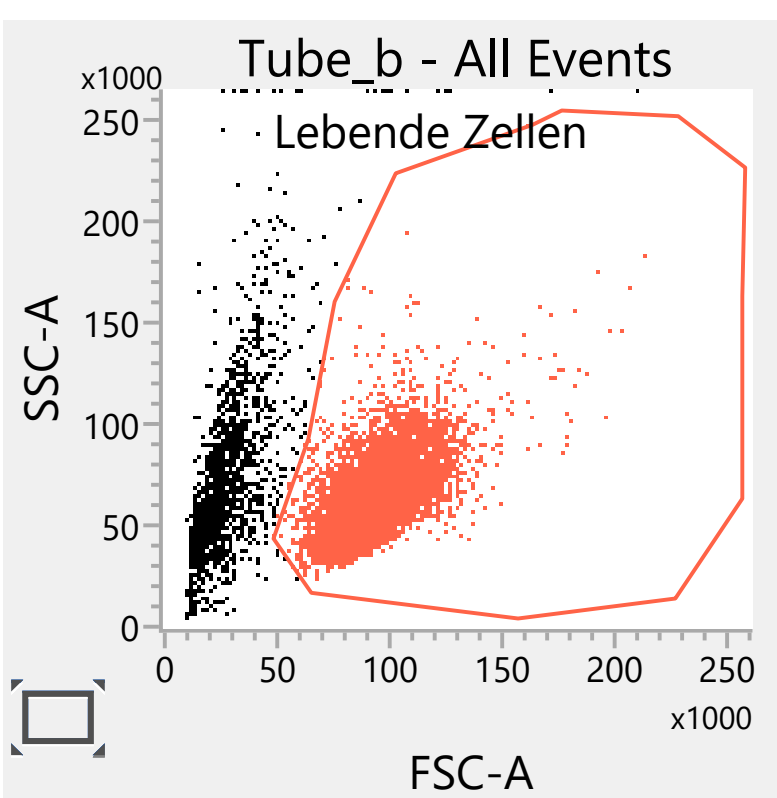

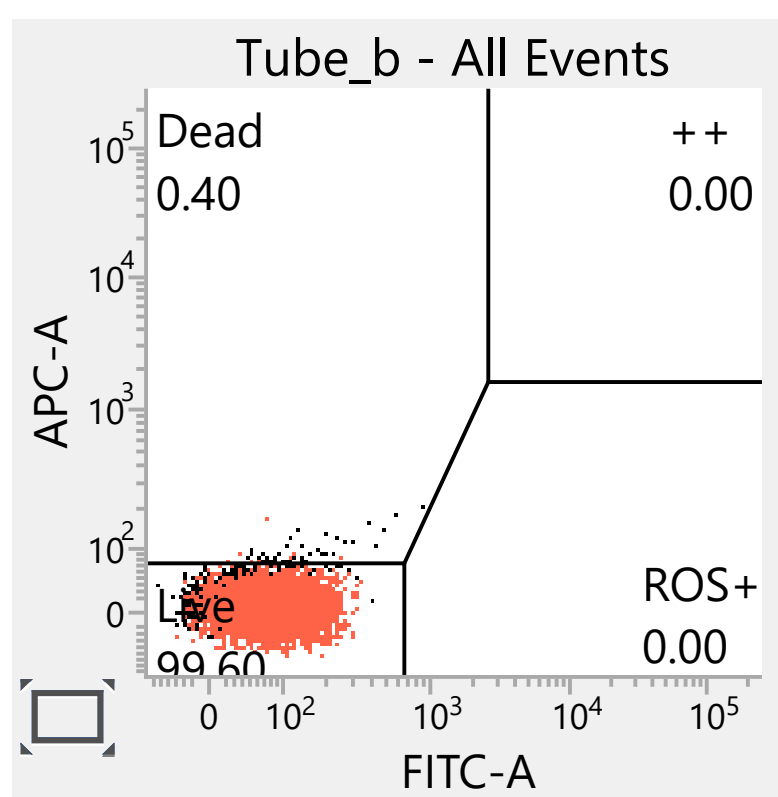

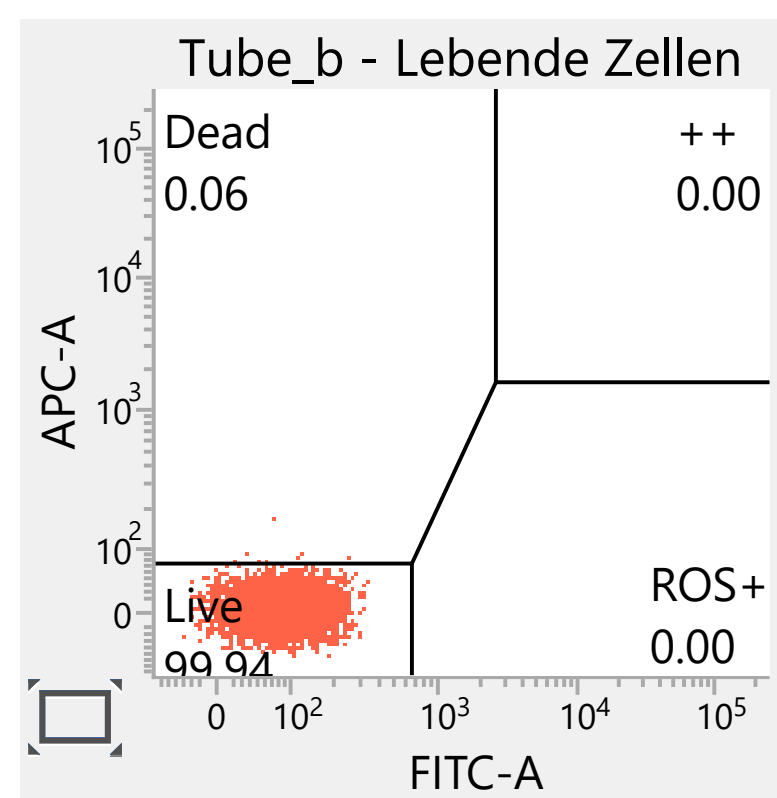

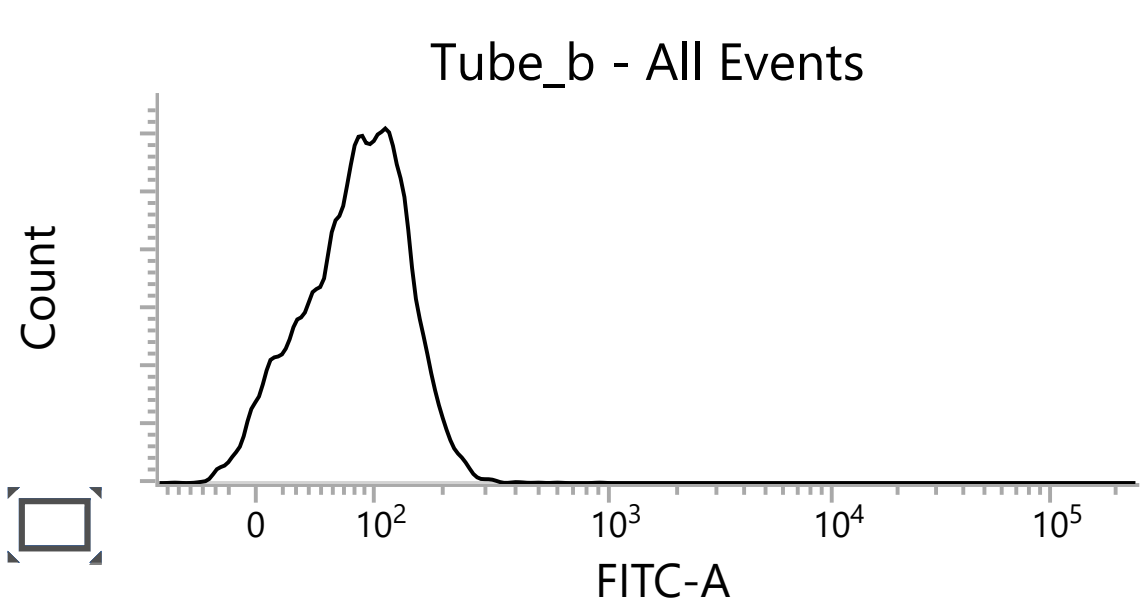

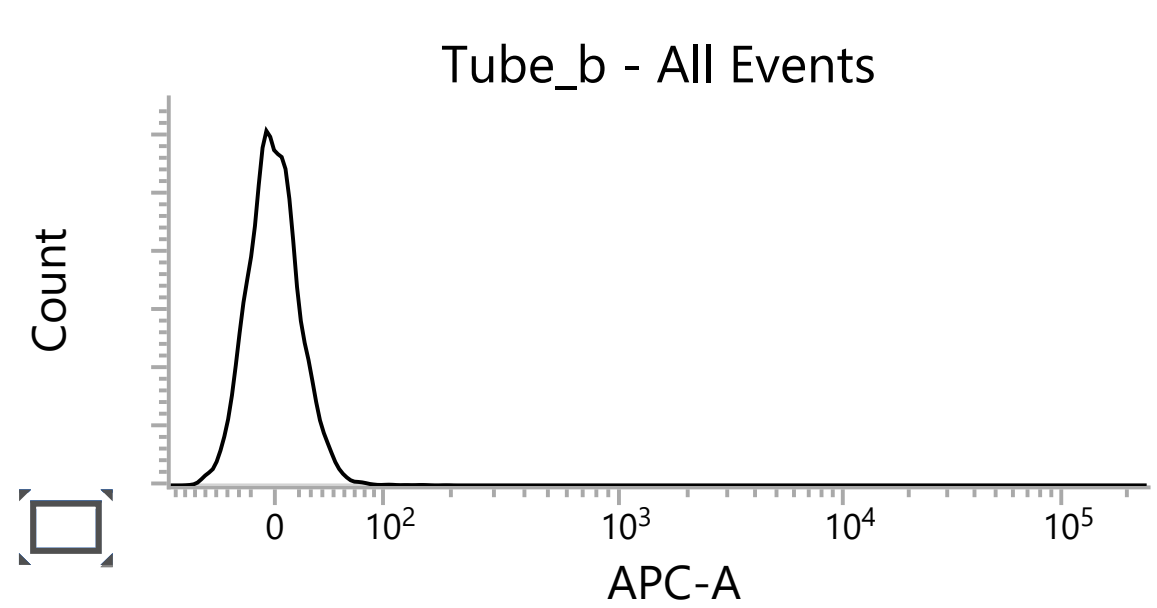

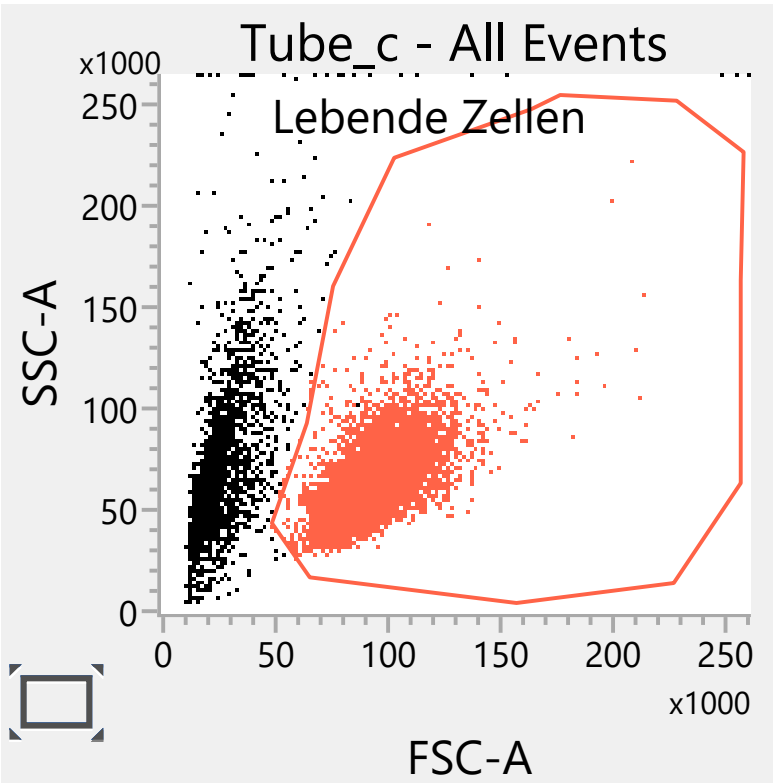

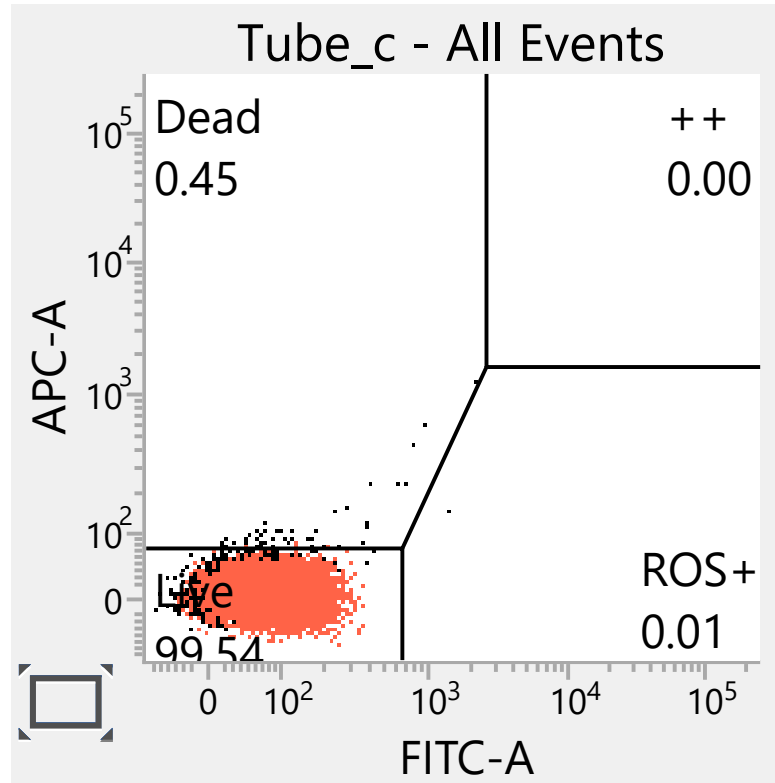

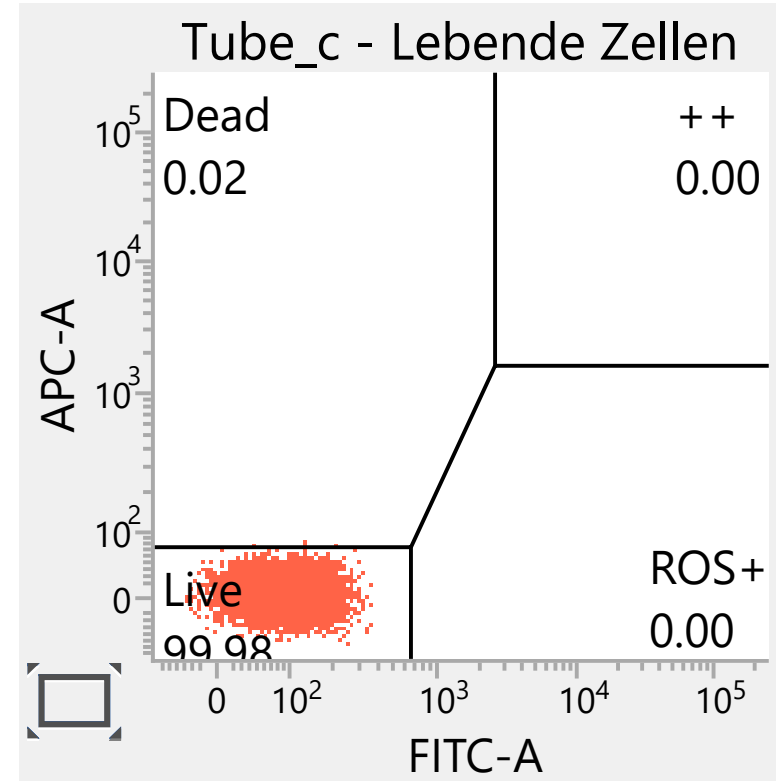

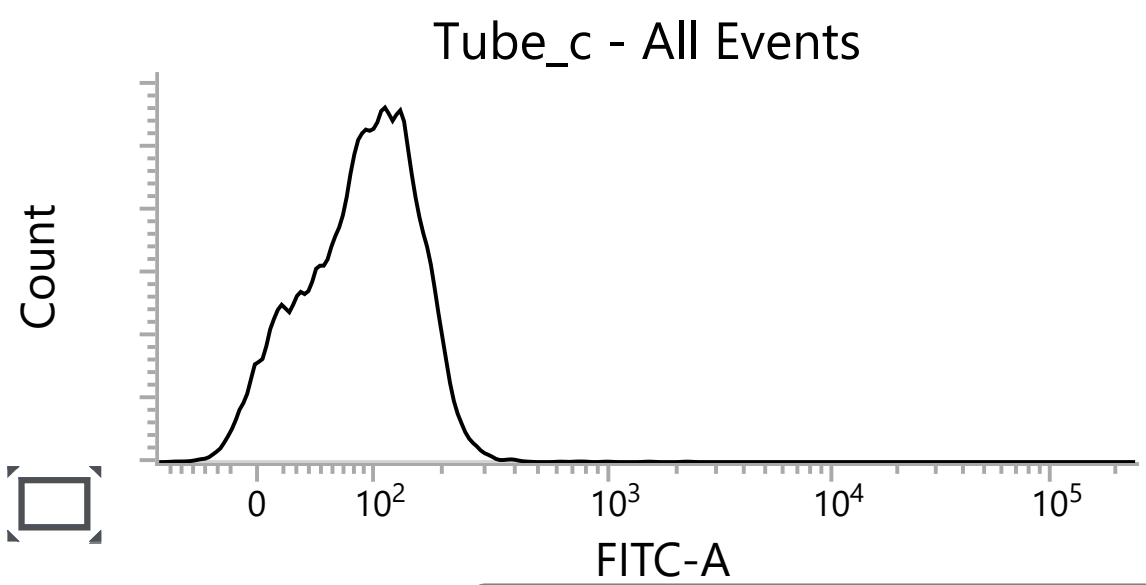

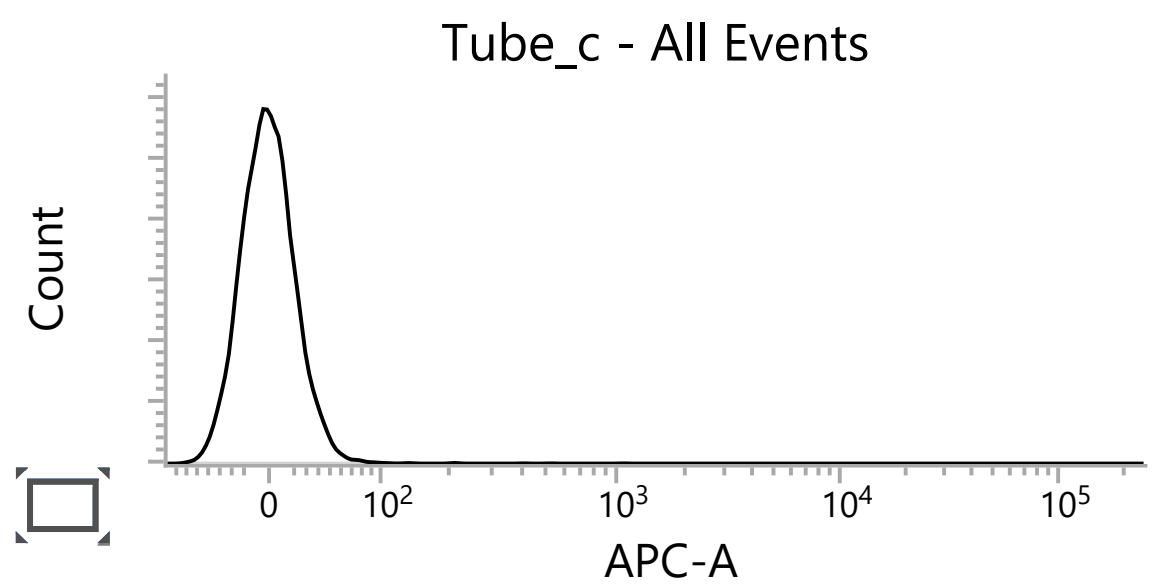

Statistics

Name	Events	% Parent	% Grandparent	% Total
Tube_a:All Events	10,000	***	***	100.00
Tube_b:All Events	10,000	***	***	100.00
Tube_c:All Events	10,000	***	***	100.00
Tube_a:Lebende Zellen	8,427	84.27	***	84.27
Tube_b:Lebende Zellen	8,542	85.42	***	85.42
Tube_c:Lebende Zellen	8,264	82.64	***	82.64

CellROX Assay

Sample ID: N6-ATM_H2O2 4yM

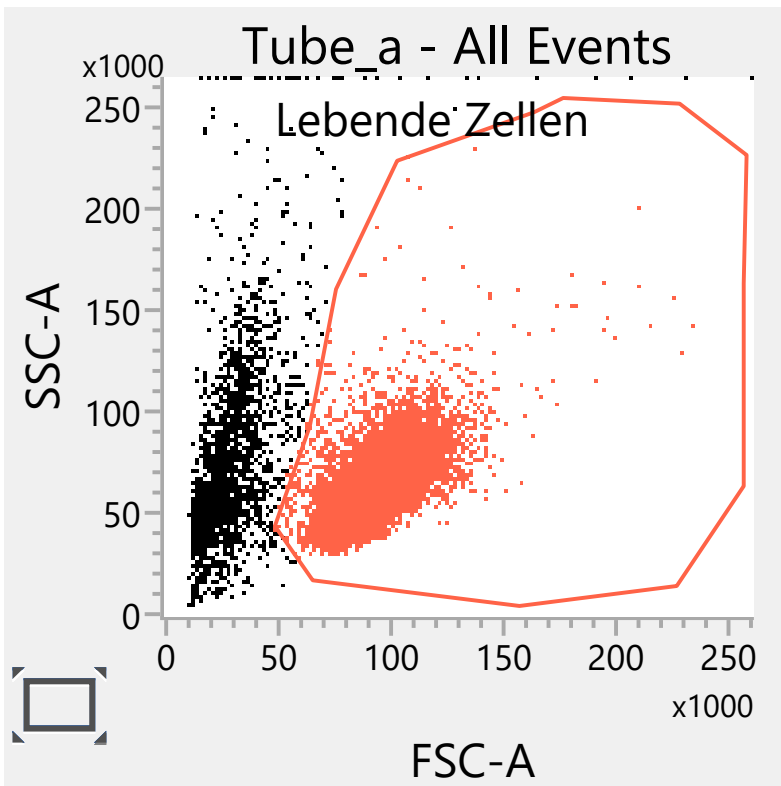

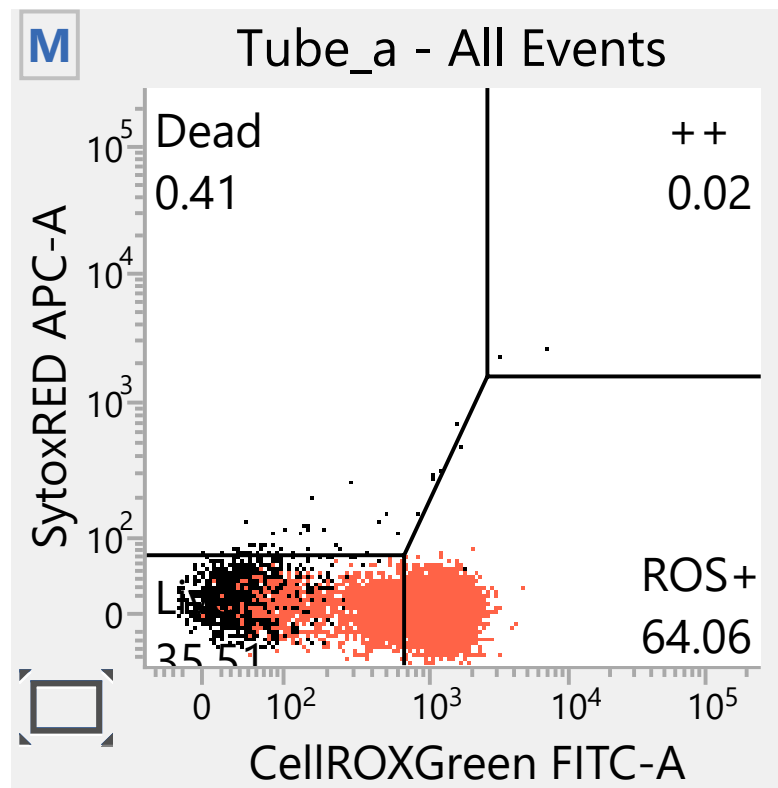

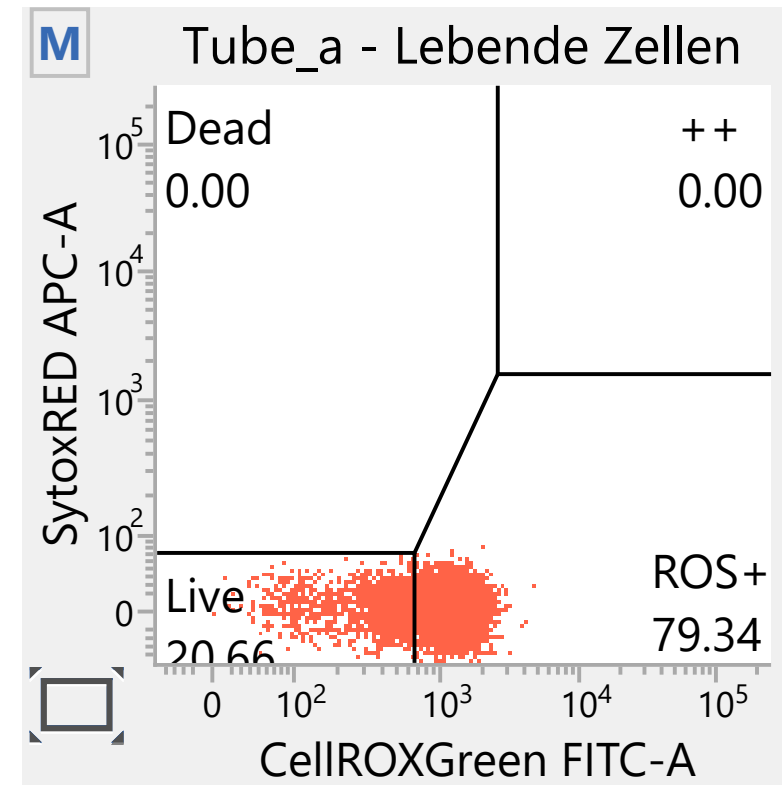

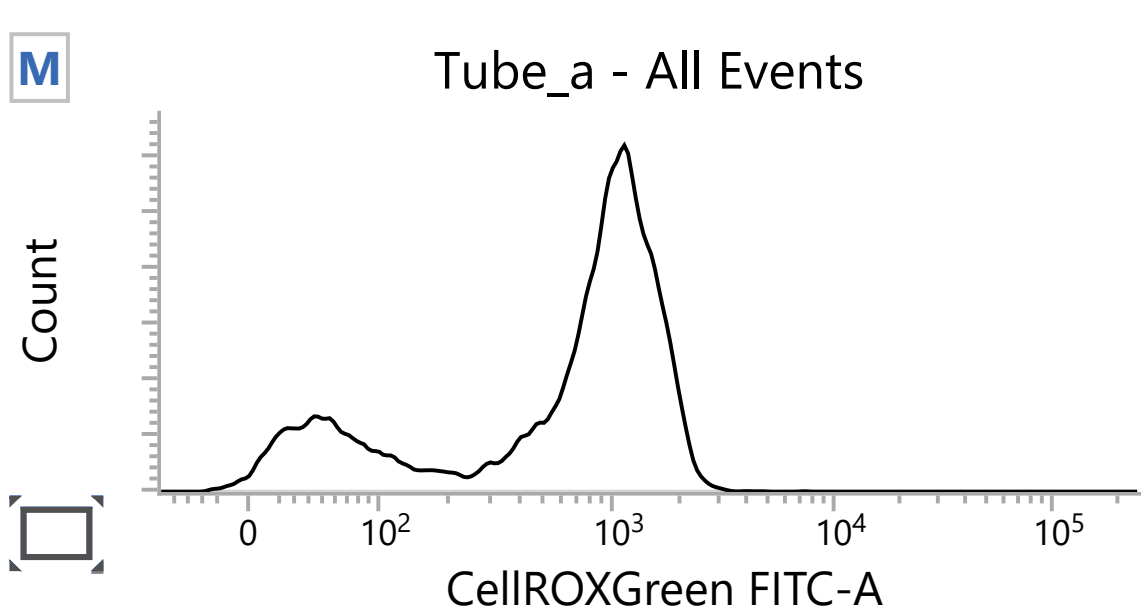

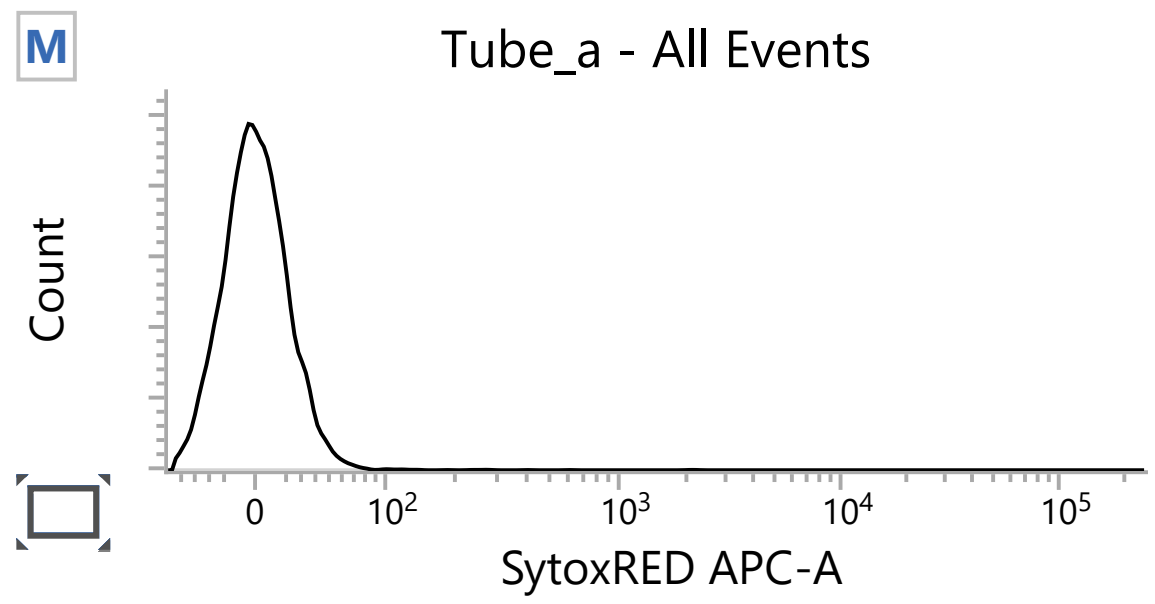

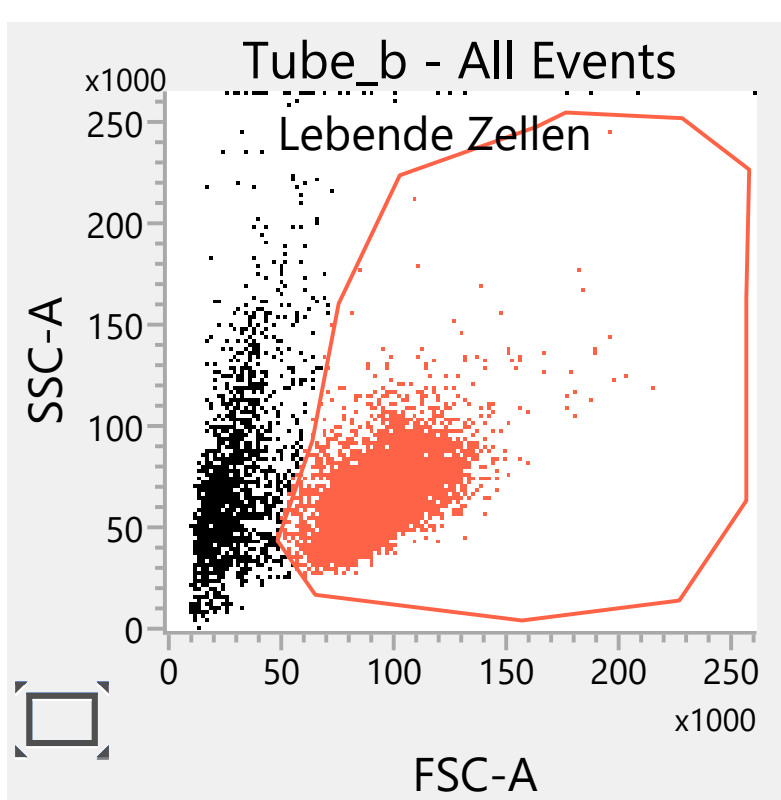

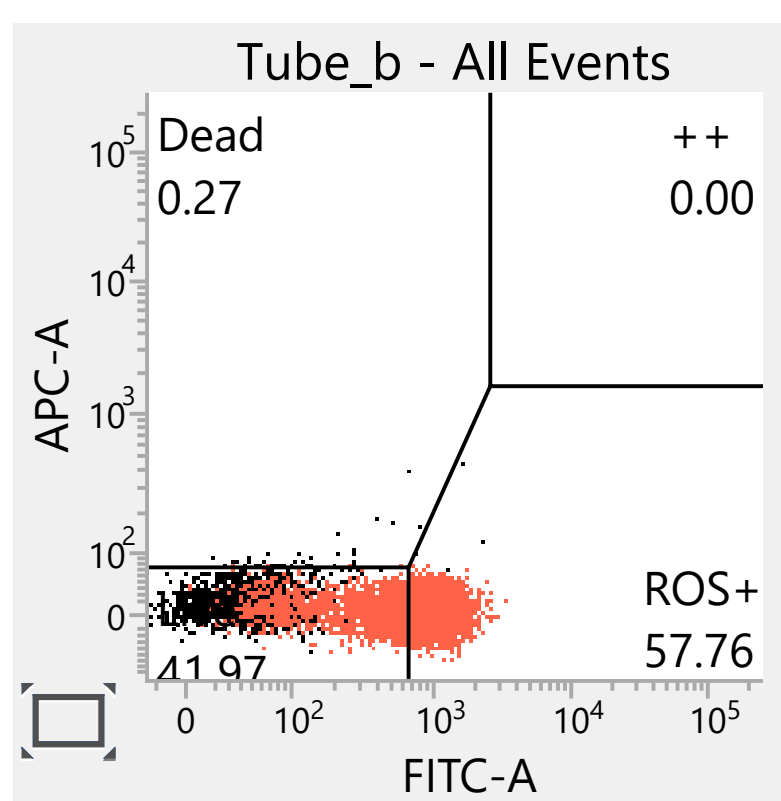

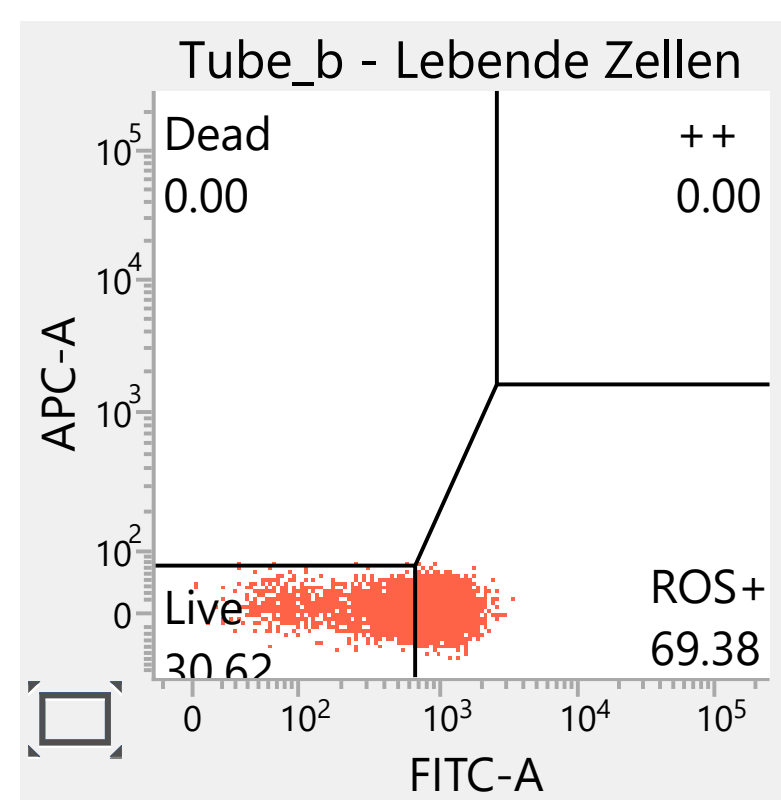

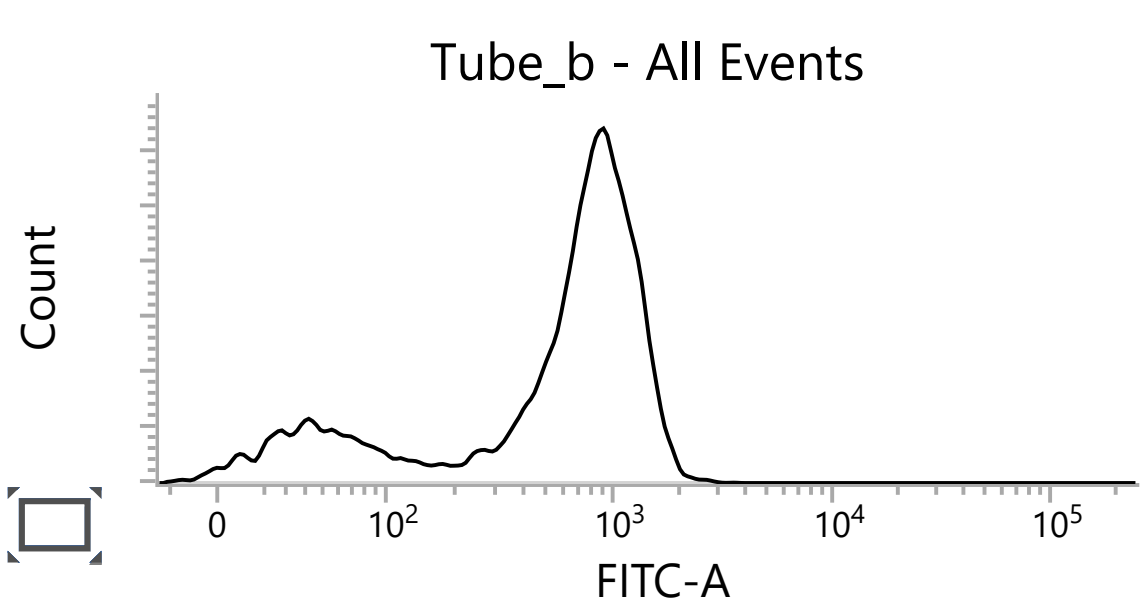

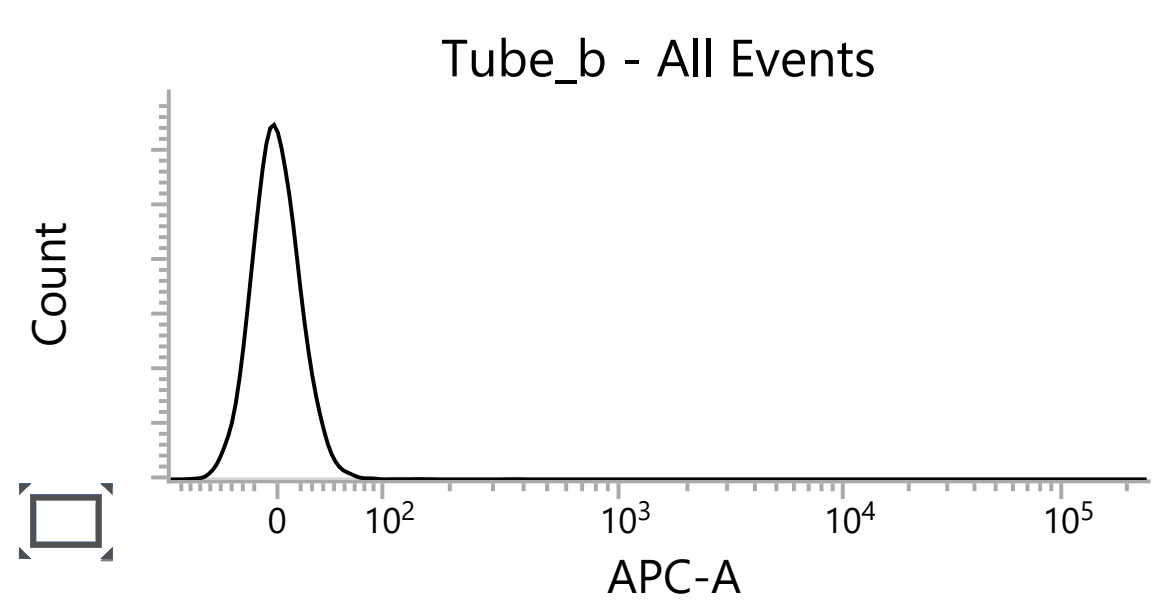

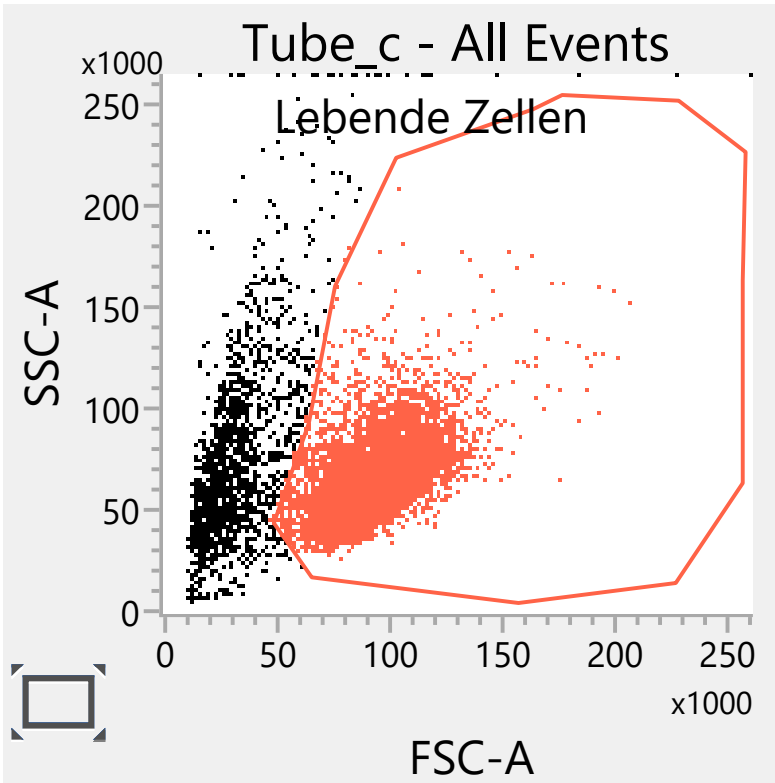

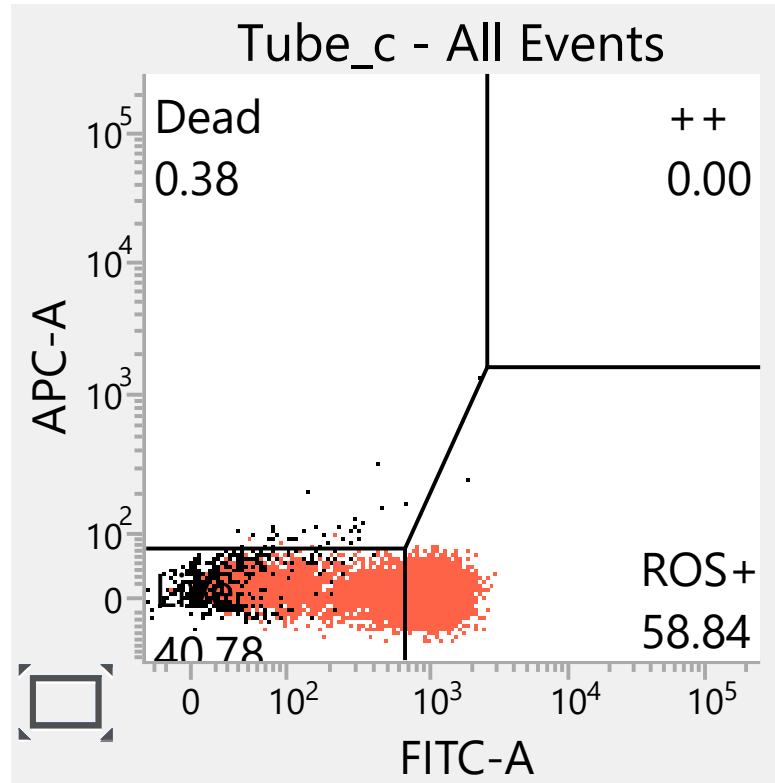

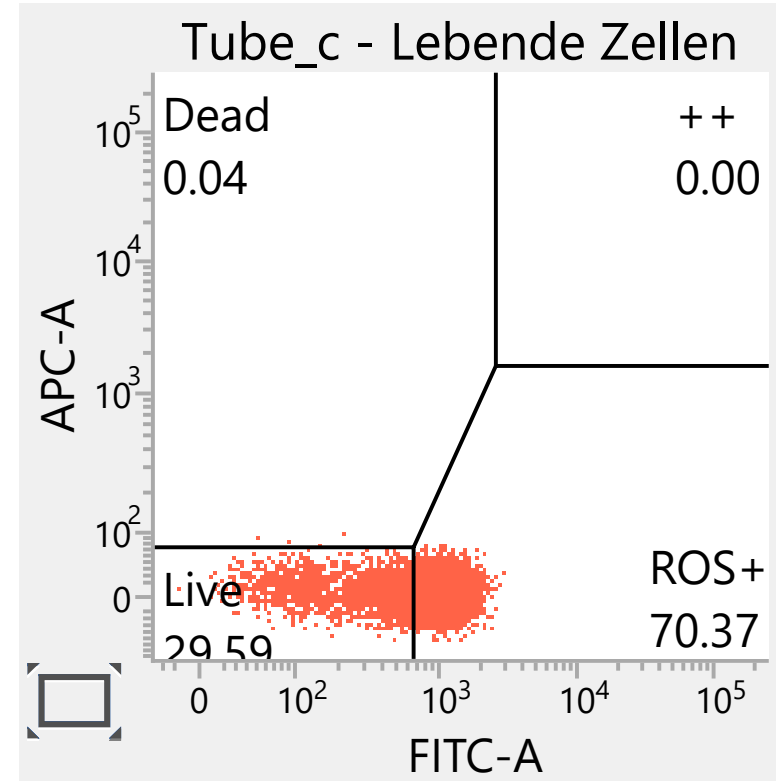

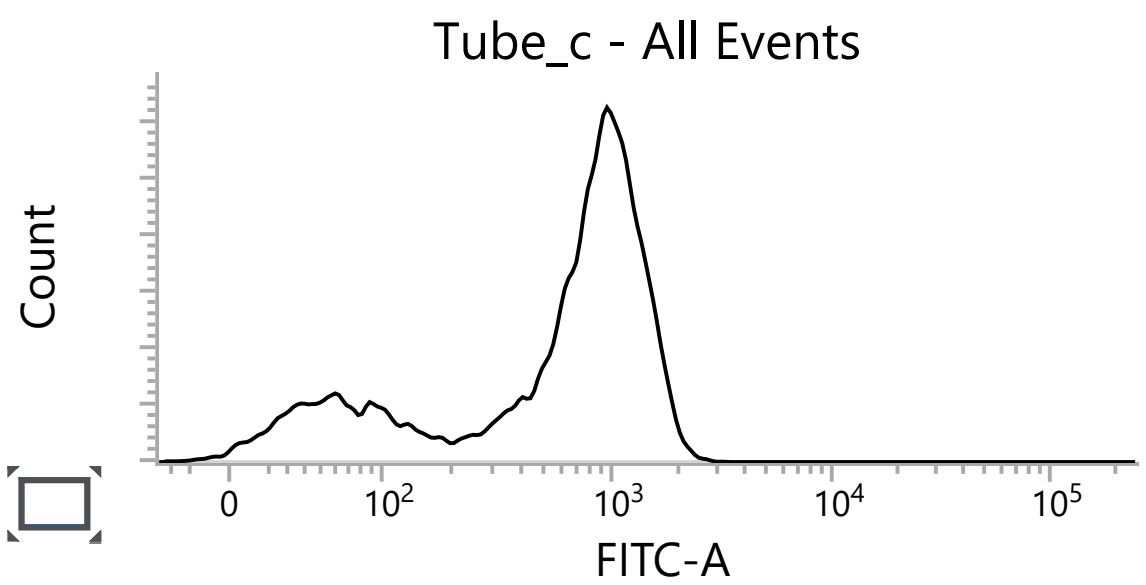

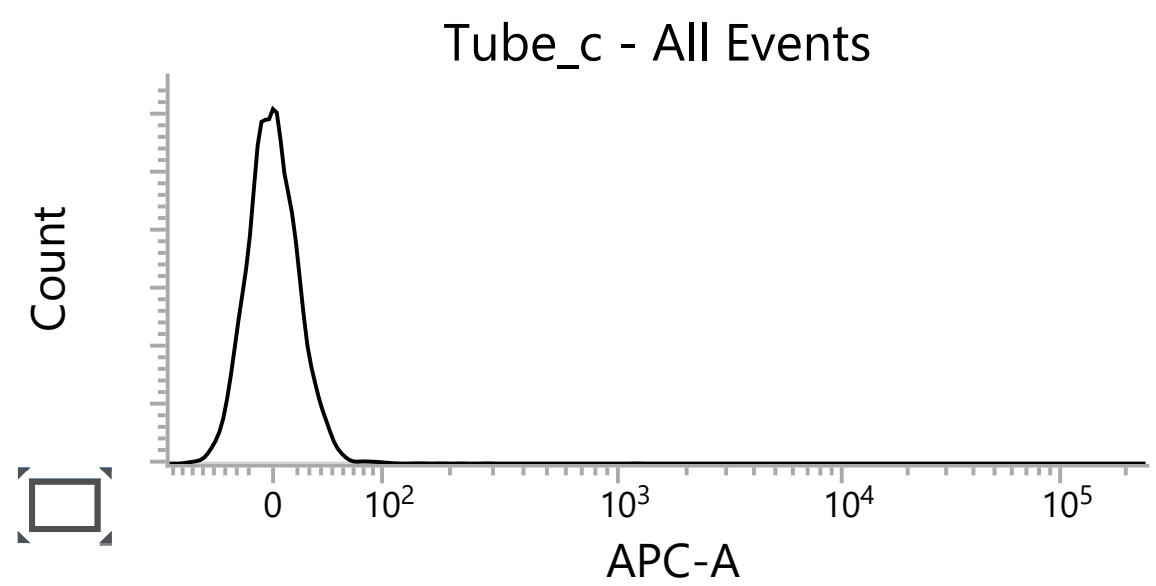

Statistics

Name	Events	% Parent	% Grandparent	% Total
Tube_a:All Events	10,000	***	***	100.00
Tube_b:All Events	10,000	***	***	100.00
Tube_c:All Events	10,000	***	***	100.00
Tube_a:Lebende Zellen	8,060	80.60	***	80.60
Tube_b:Lebende Zellen	8,312	83.12	***	83.12
Tube_c:Lebende Zellen	8,343	83.43	***	83.43

CellROX Assay

Sample ID: N6-ATM_KO

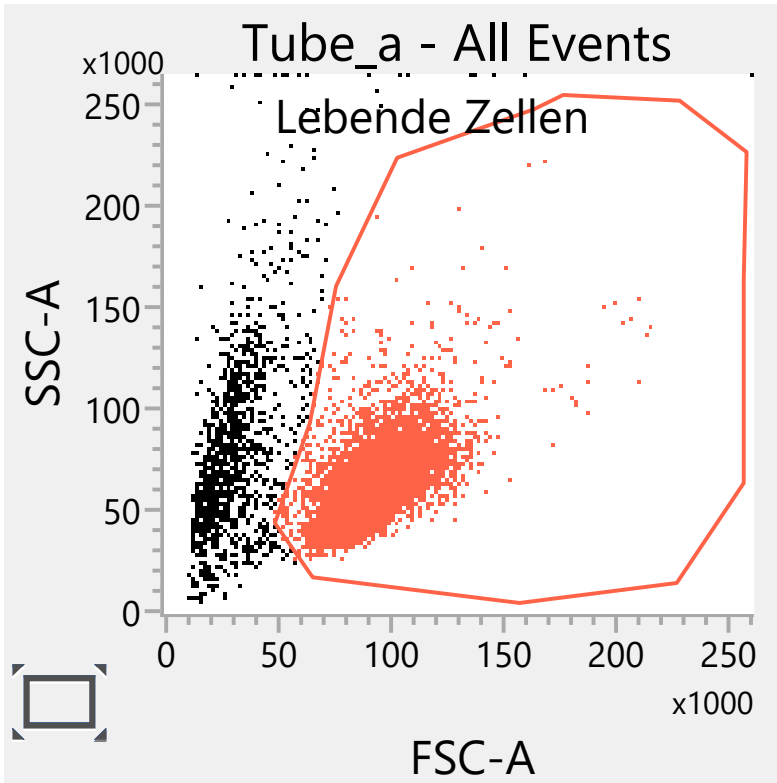

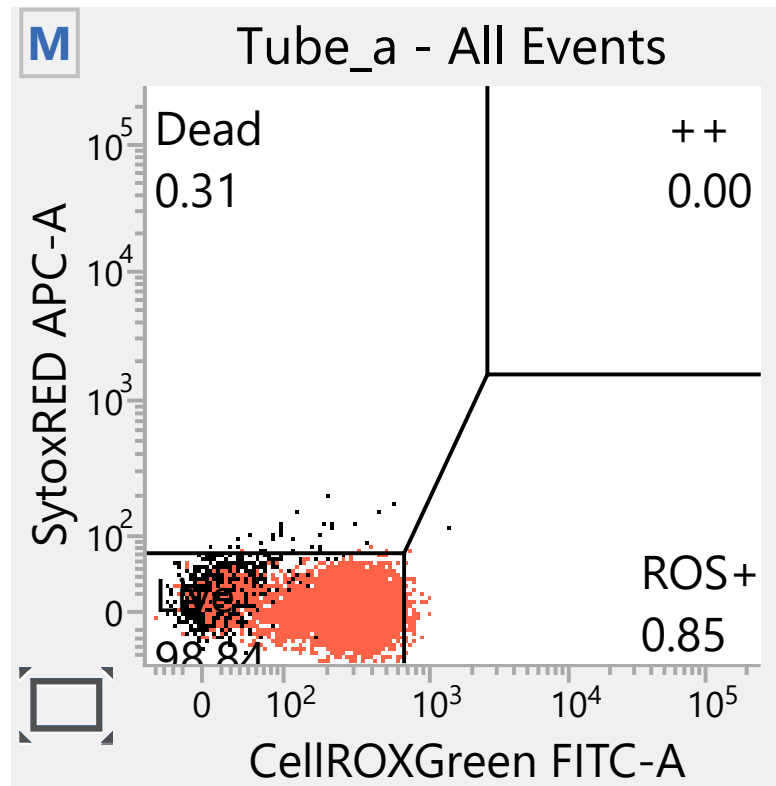

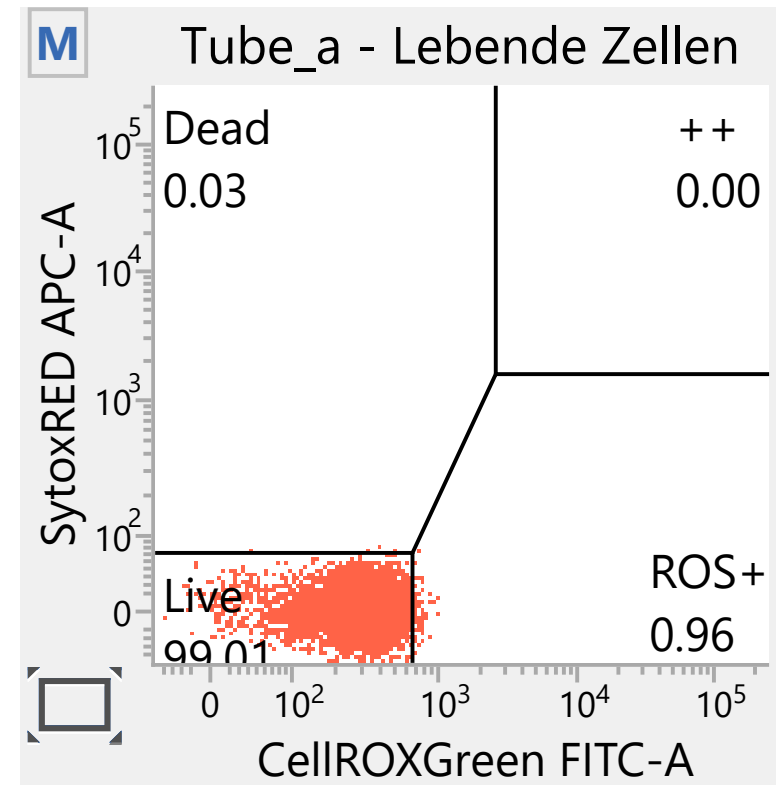

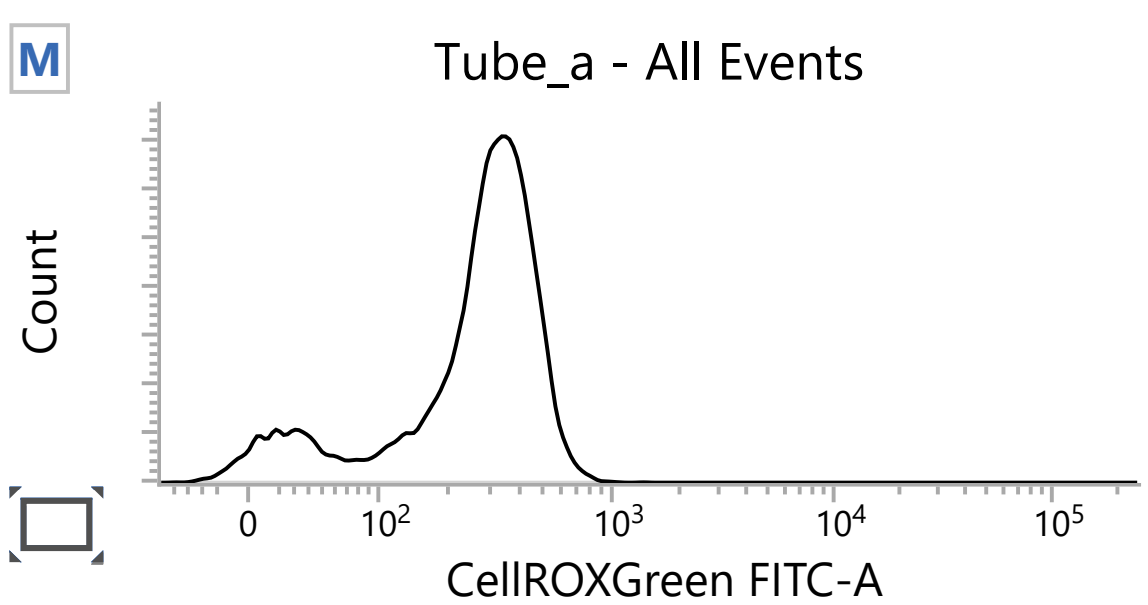

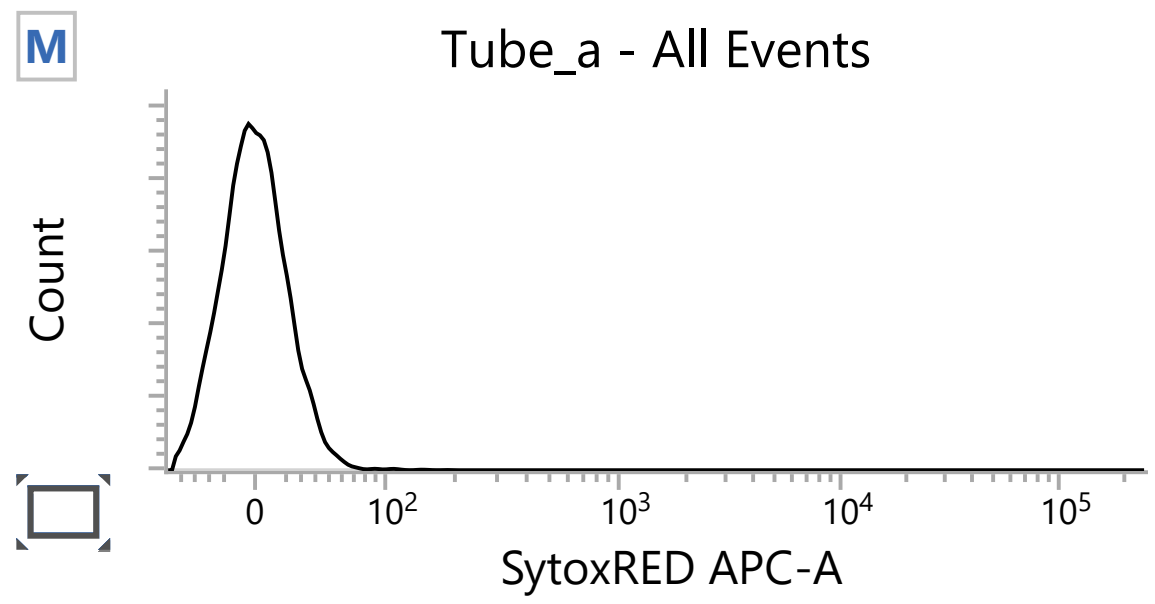

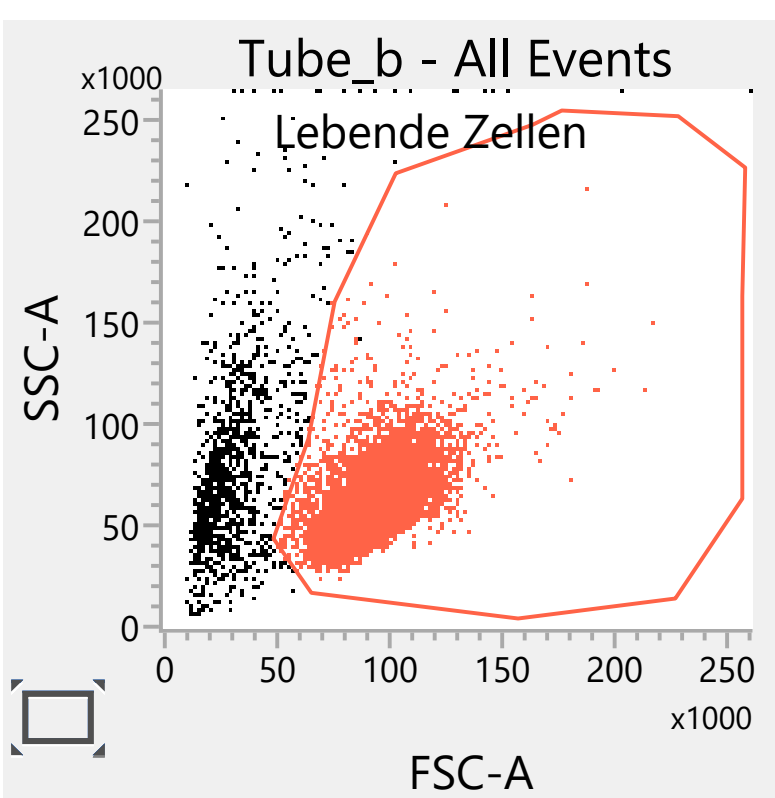

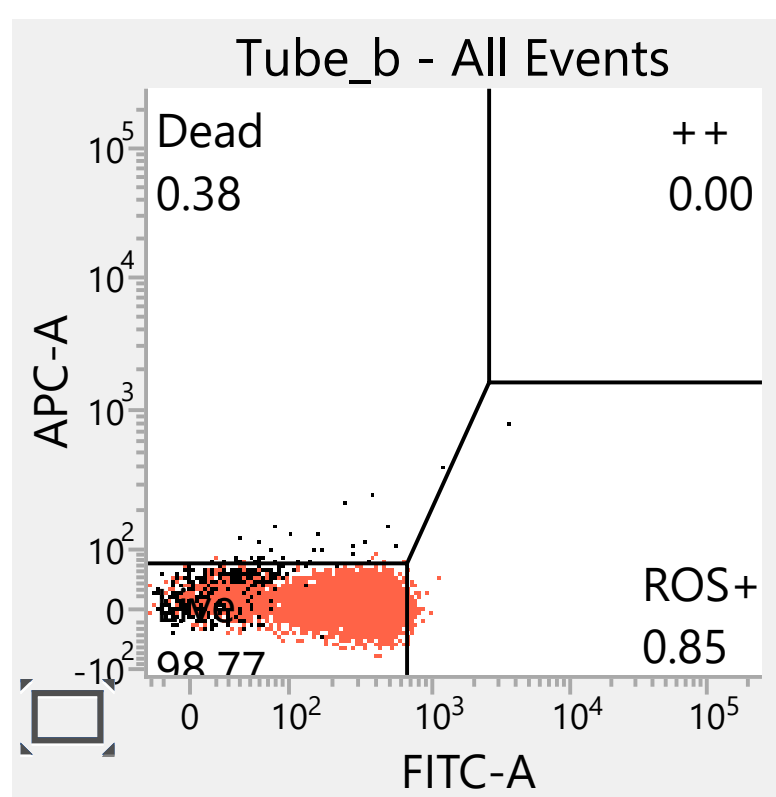

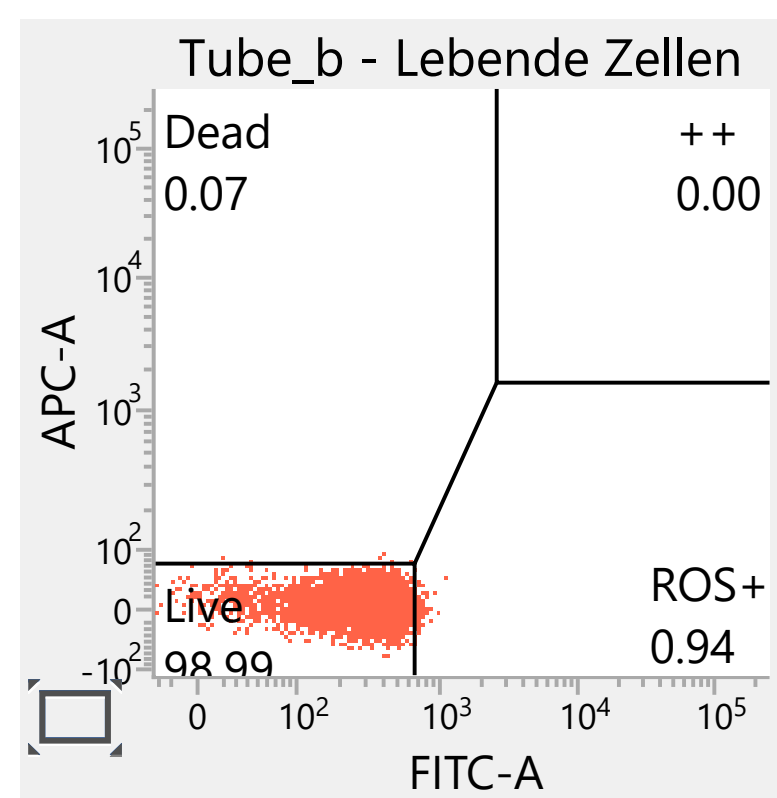

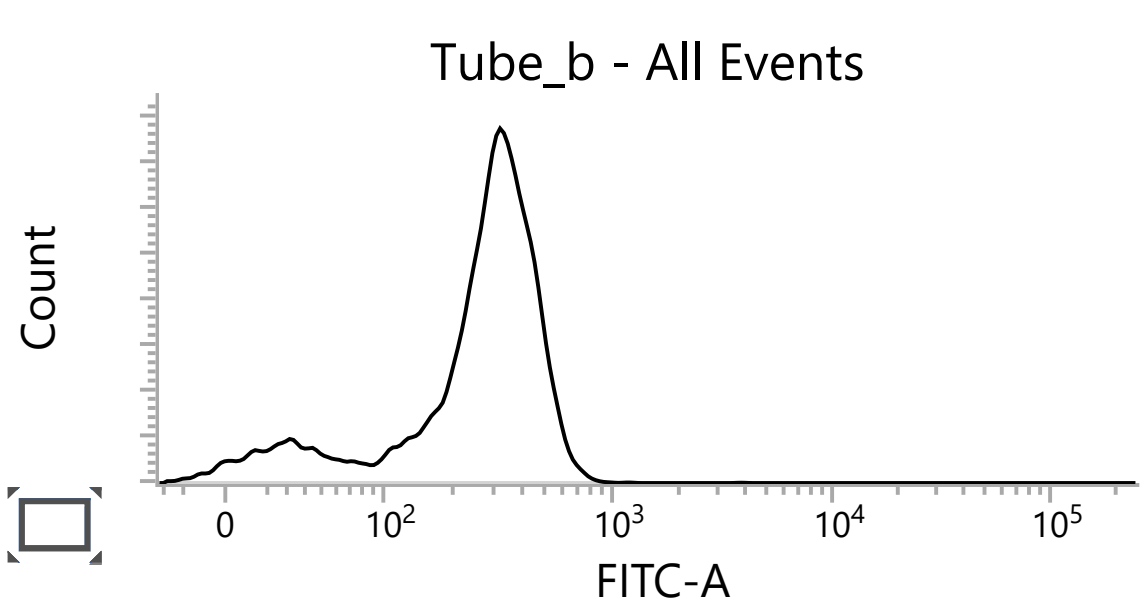

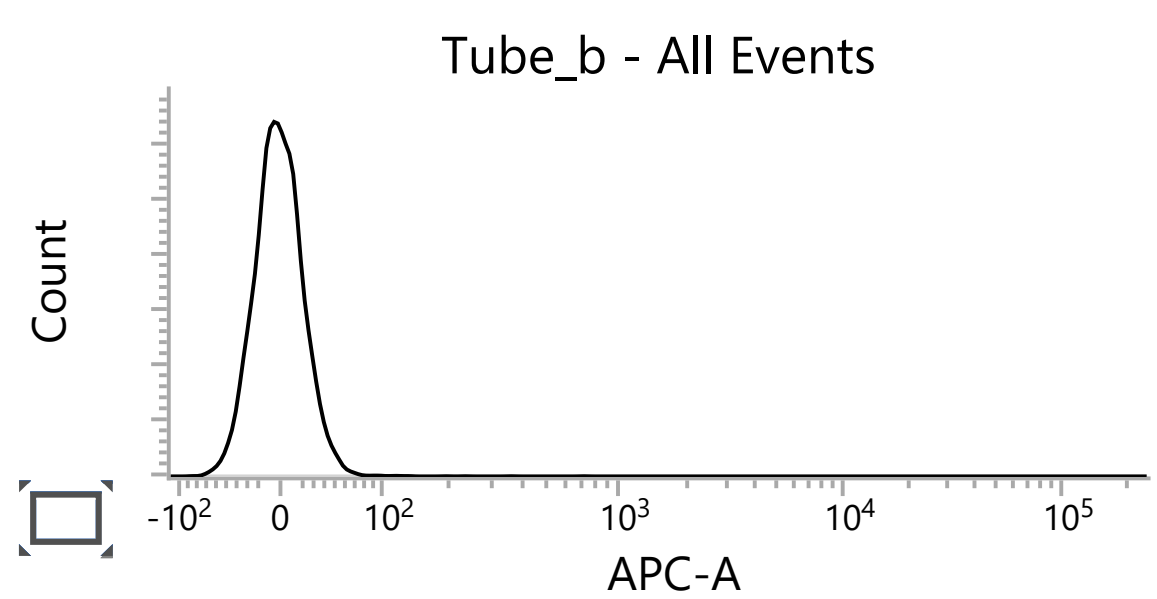

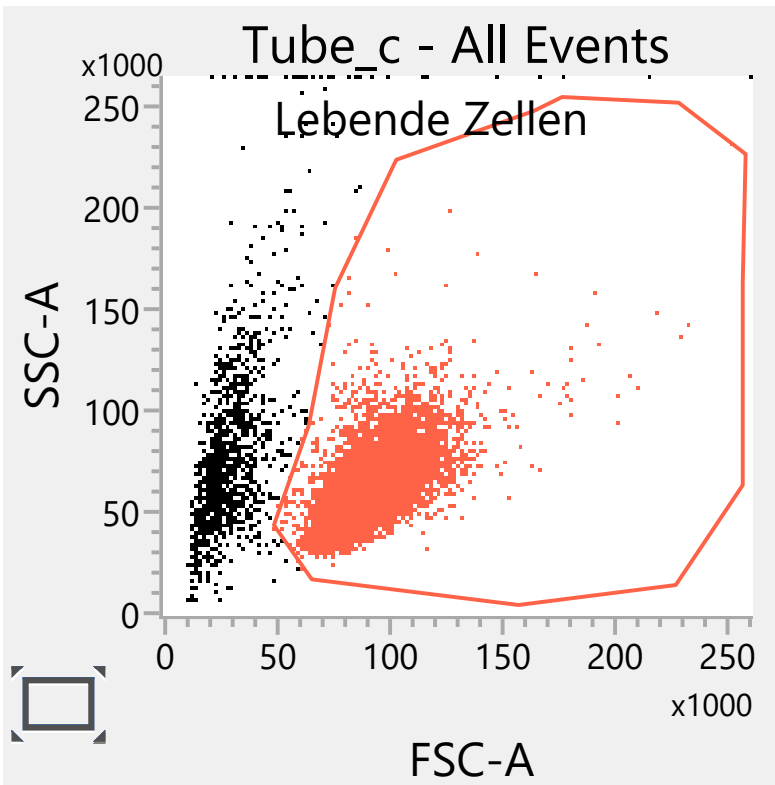

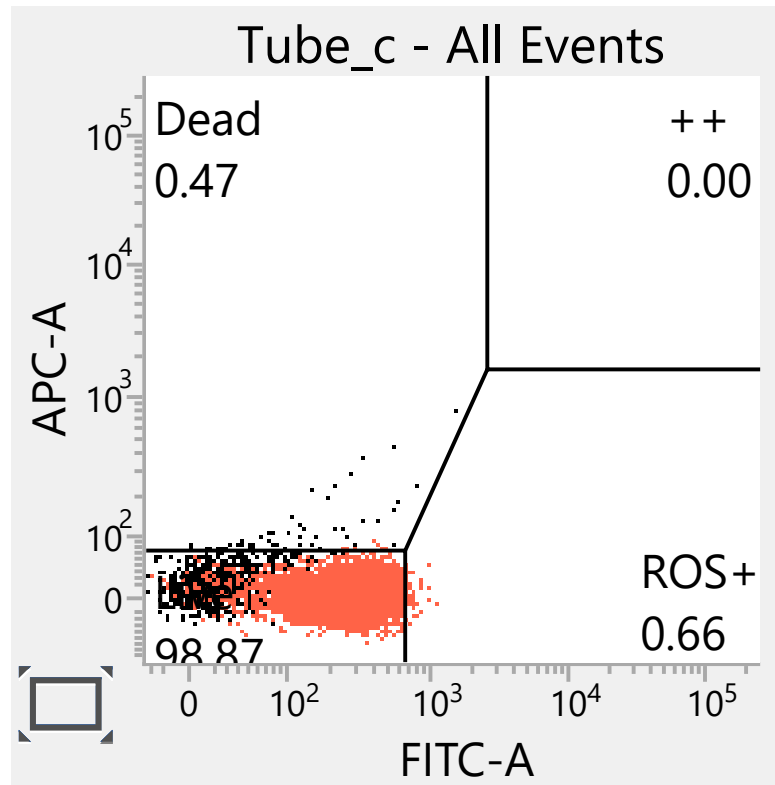

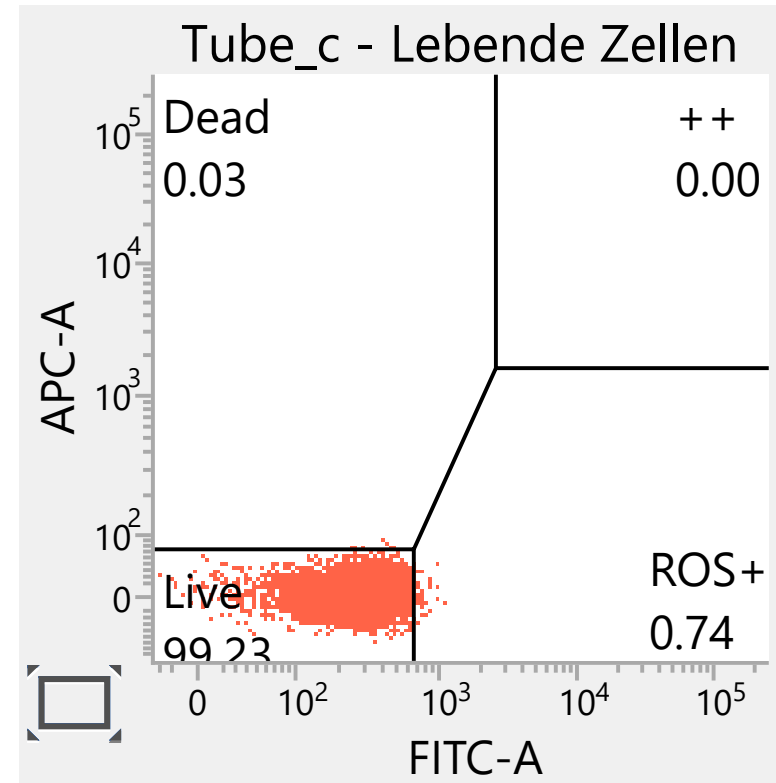

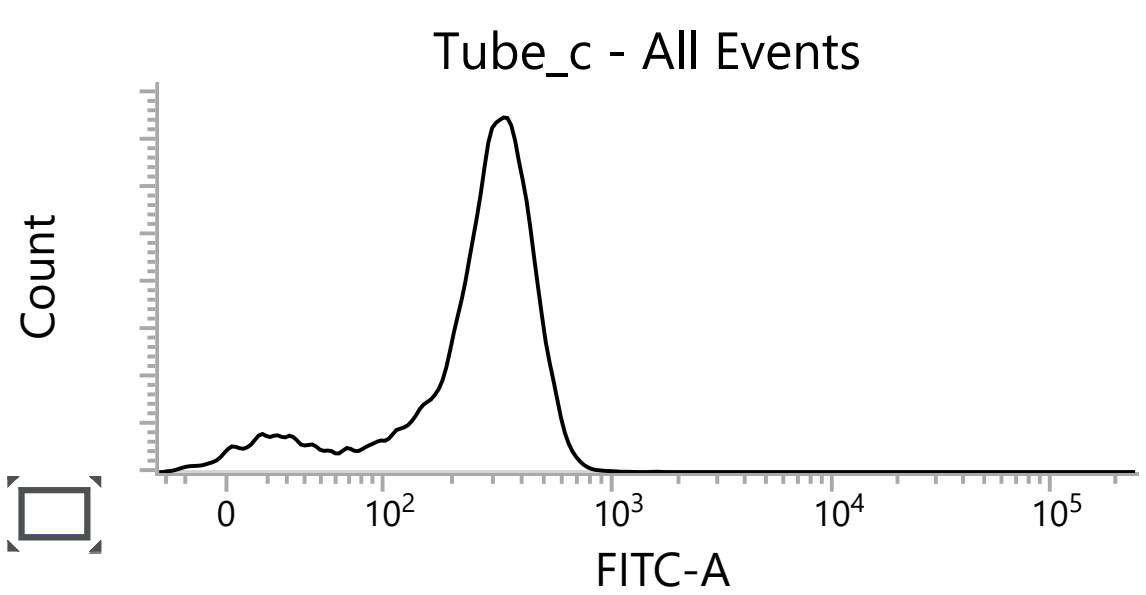

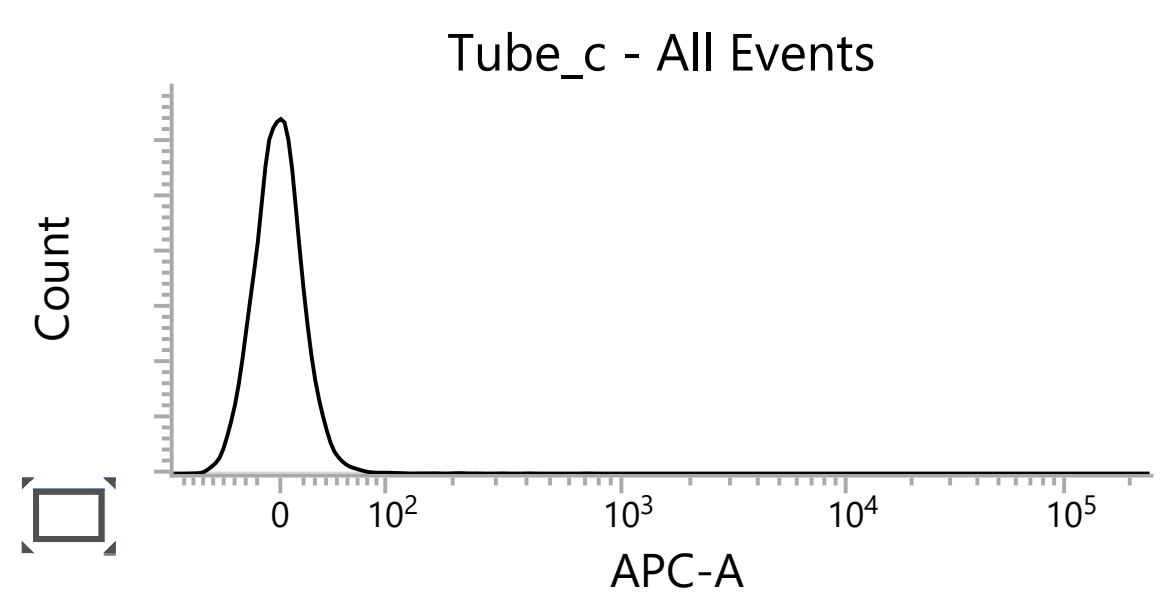

Statistics

Name	Events	% Parent	% Grandparent	% Total
Tube_a:All Events	10,000	***	***	100.00
Tube_a:Lebende Zellen	8,757	87.57	***	87.57
Tube_b:All Events	10,000	***	***	100.00
Tube_b:Lebende Zellen	8,924	89.24	***	89.24
Tube_c:All Events	10,000	***	***	100.00
Tube_c:Lebende Zellen	8,916	89.16	***	89.16

CellROX Assay

Sample ID: N6-ATM_NAC

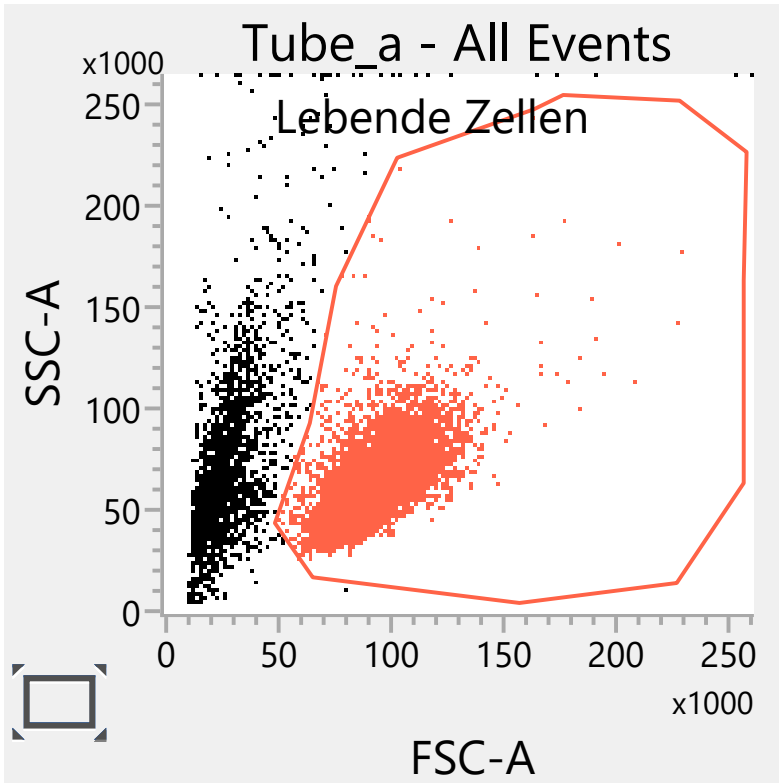

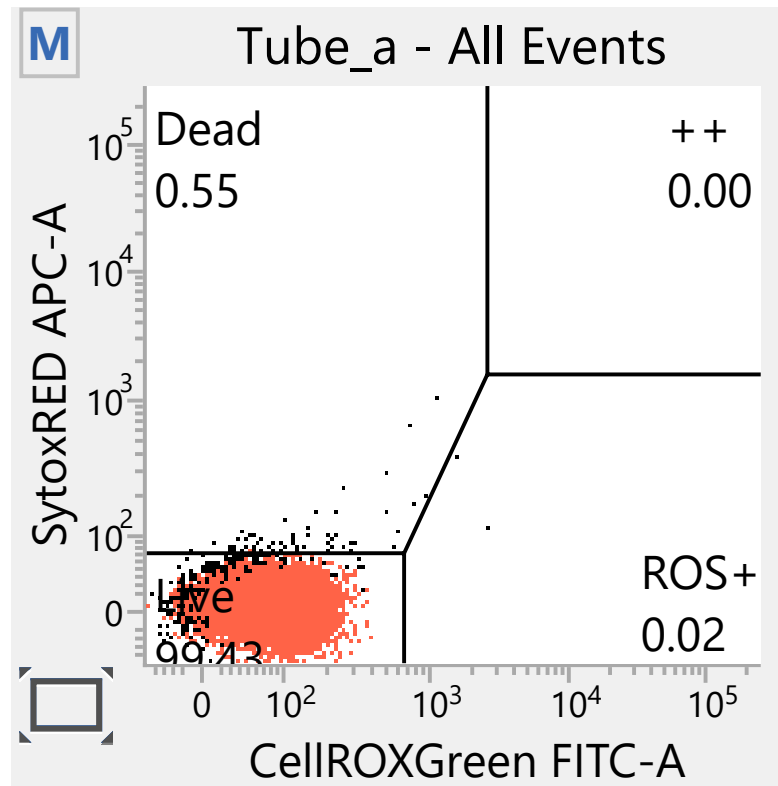

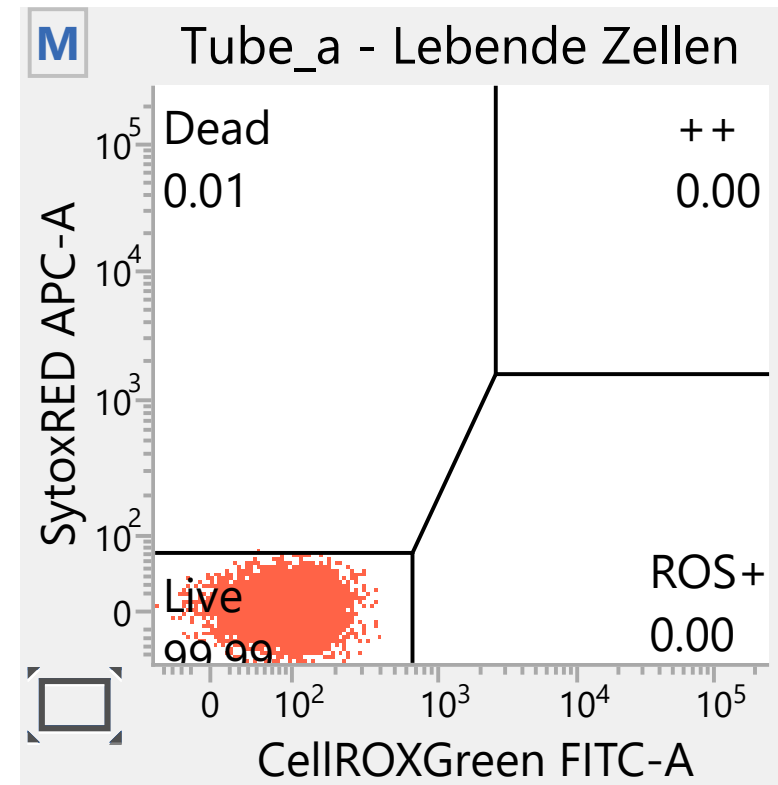

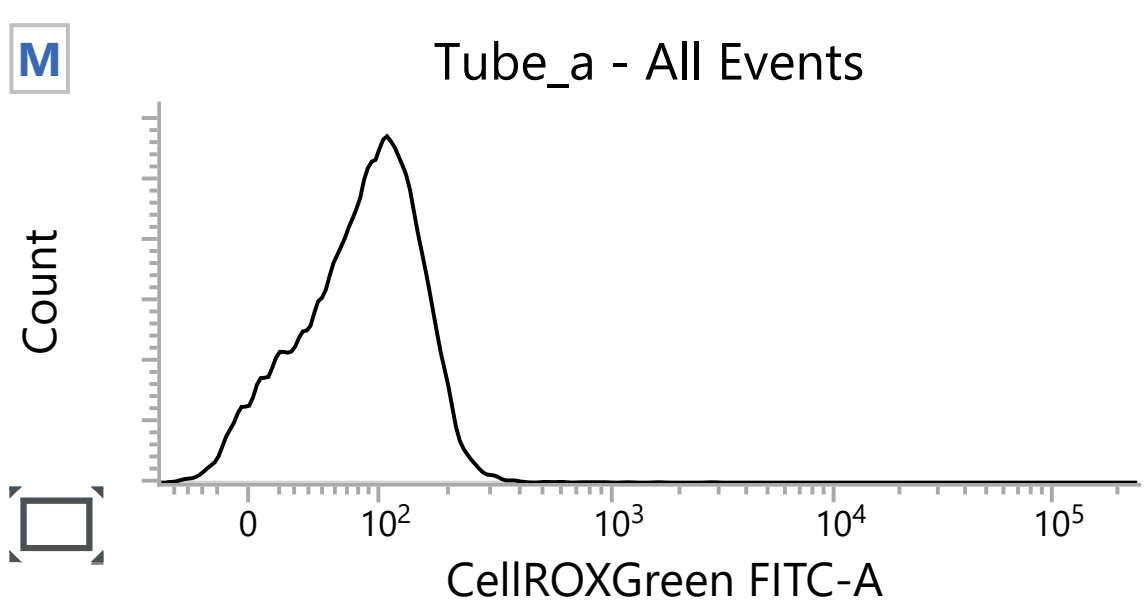

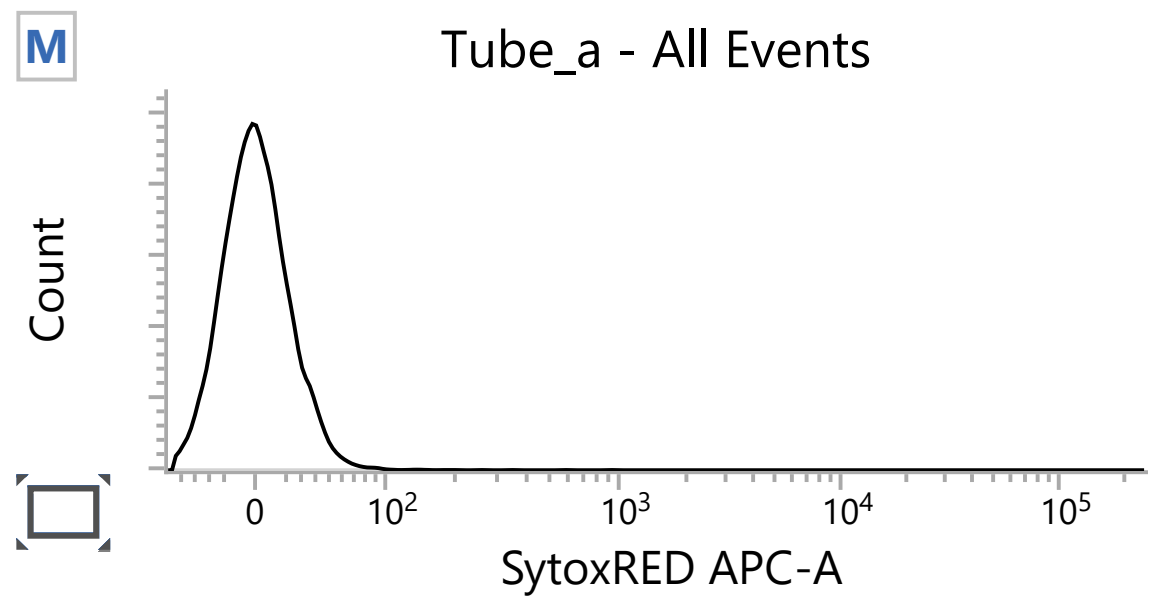

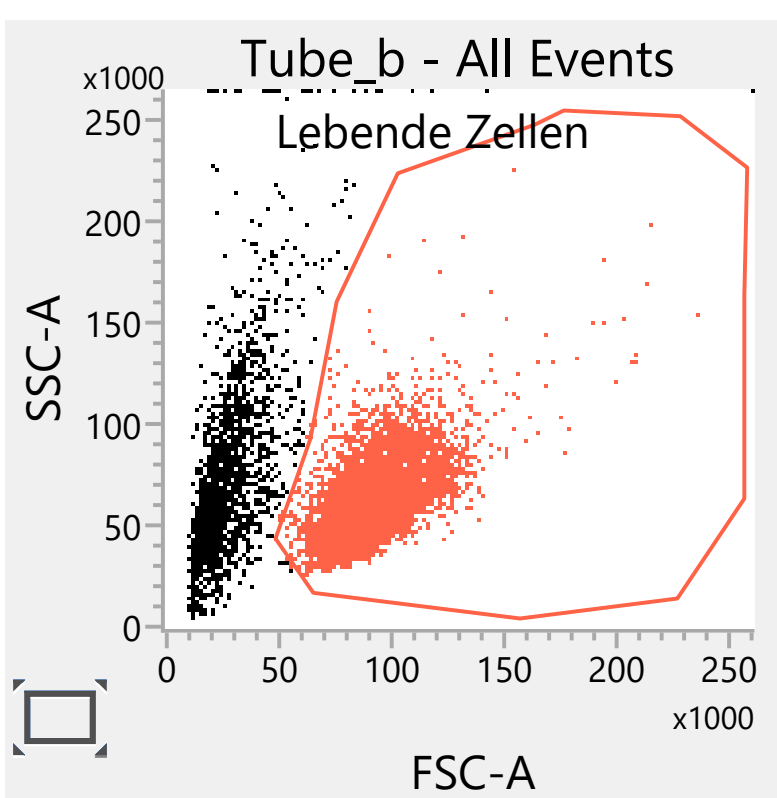

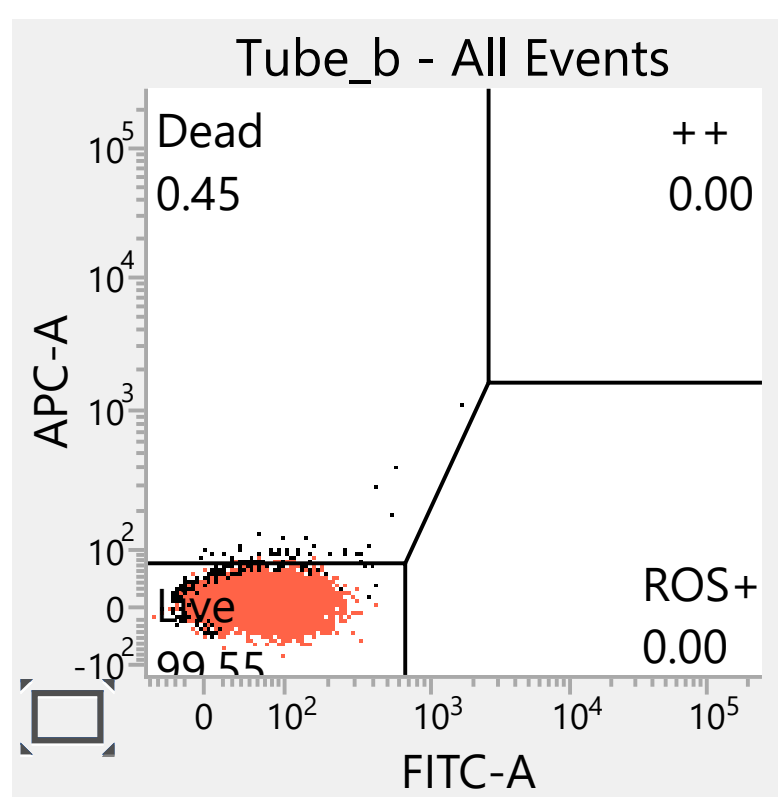

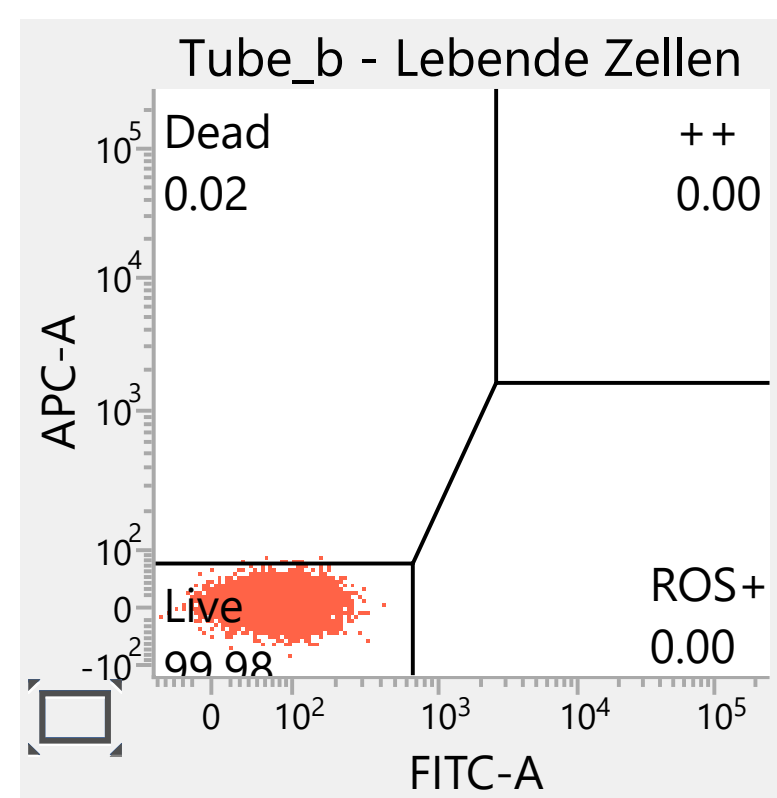

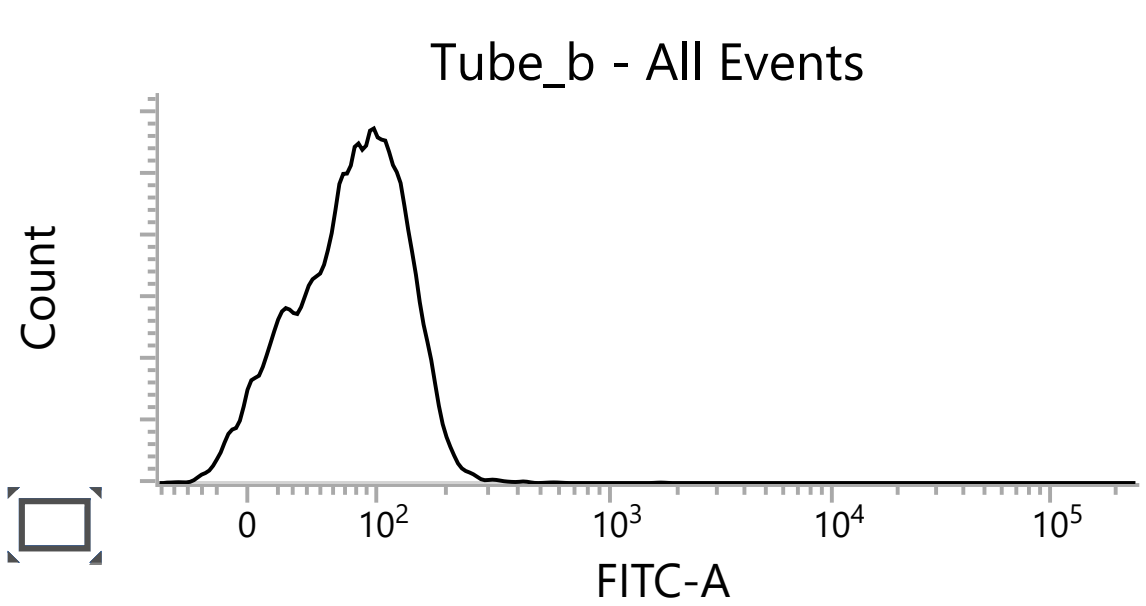

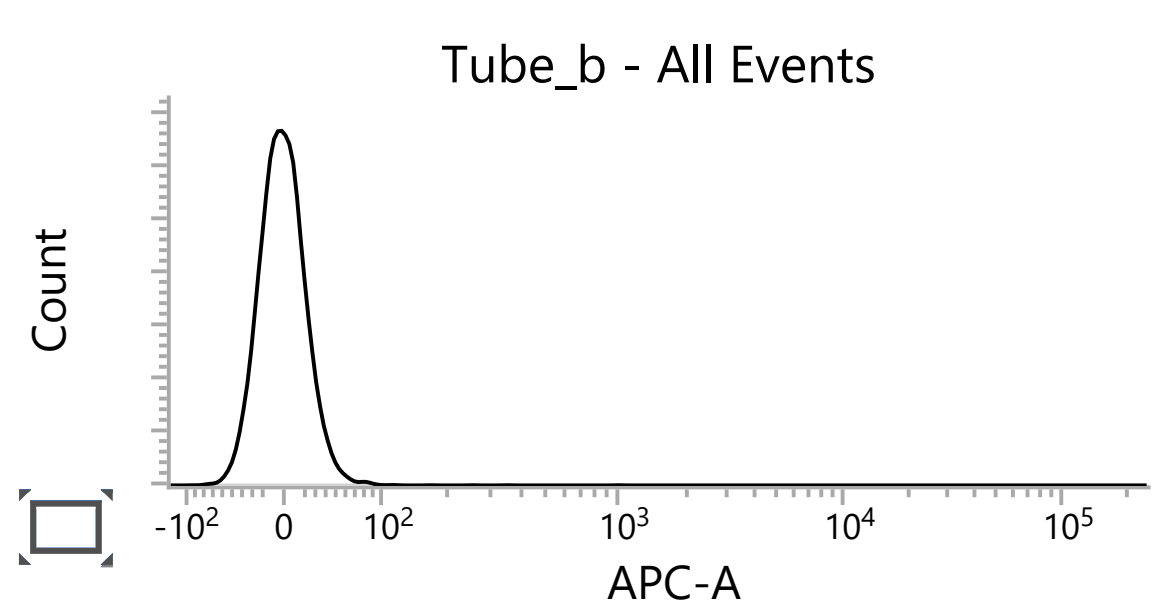

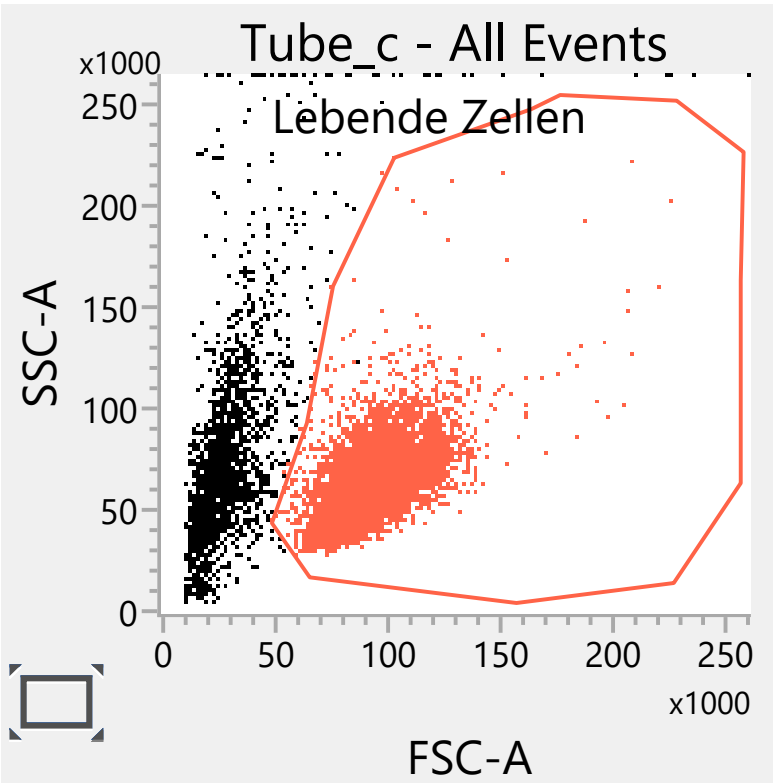

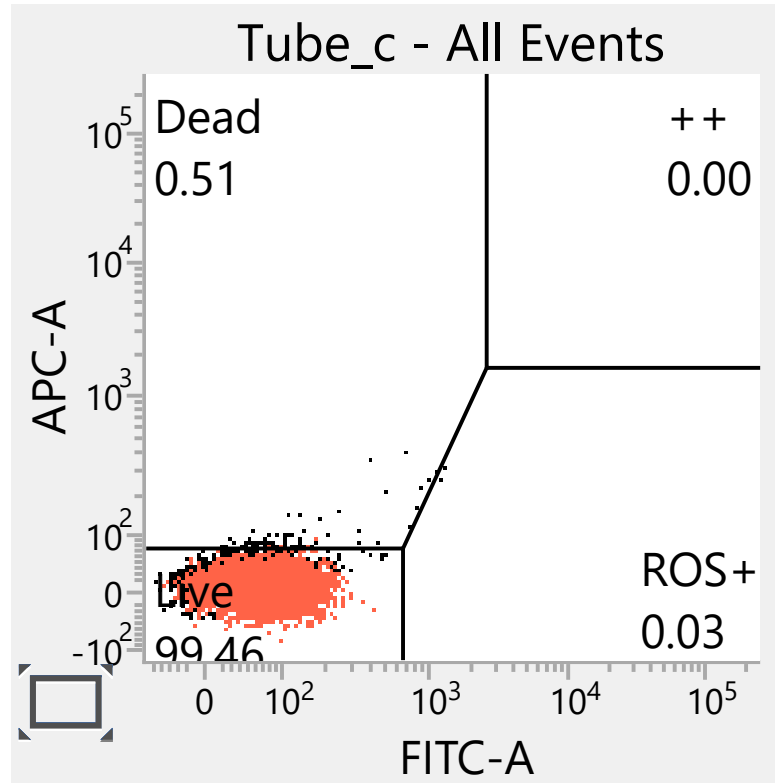

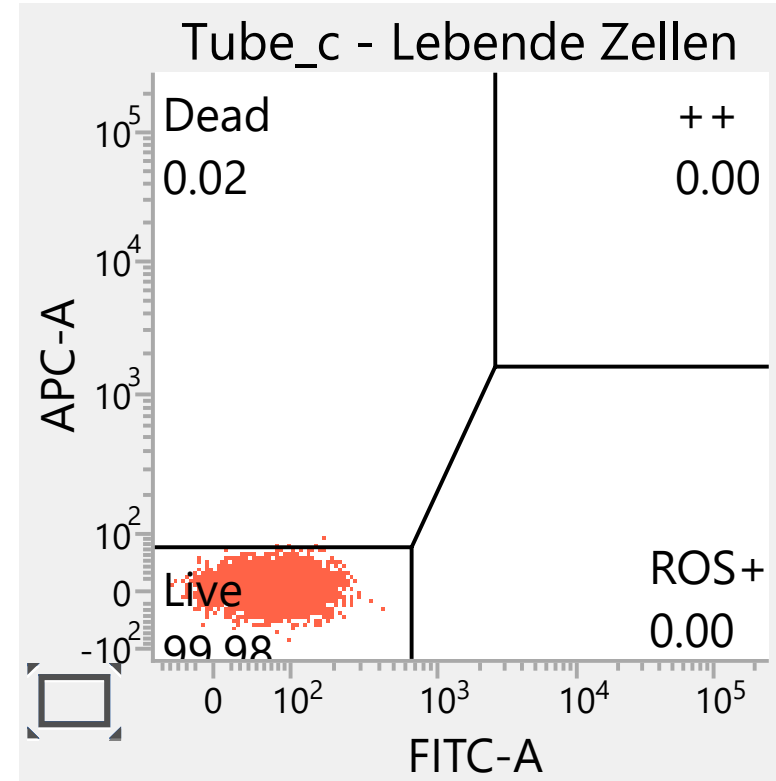

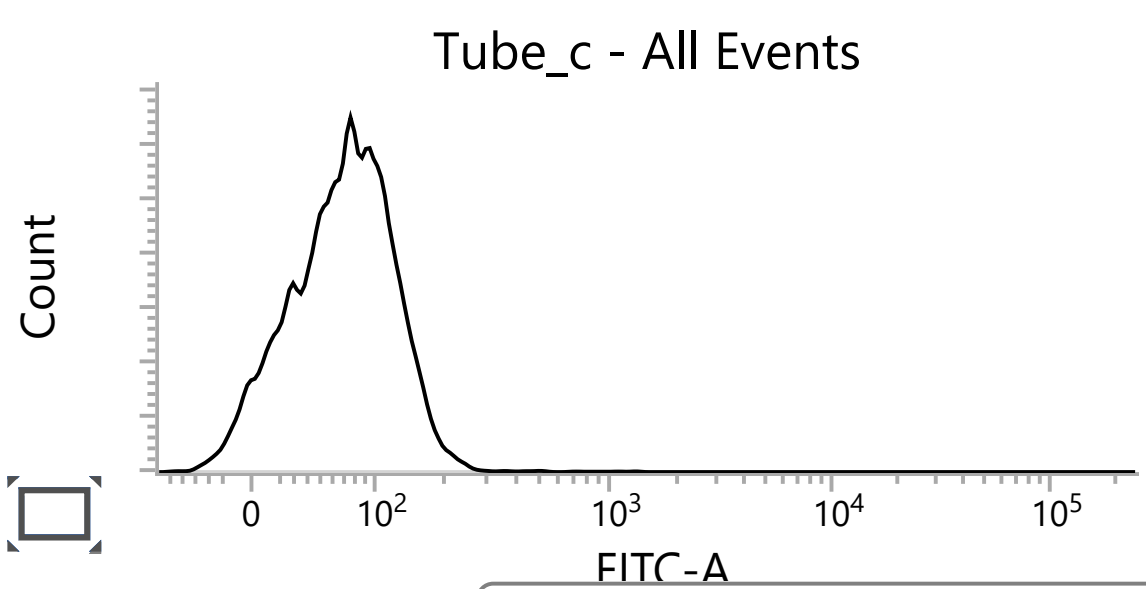

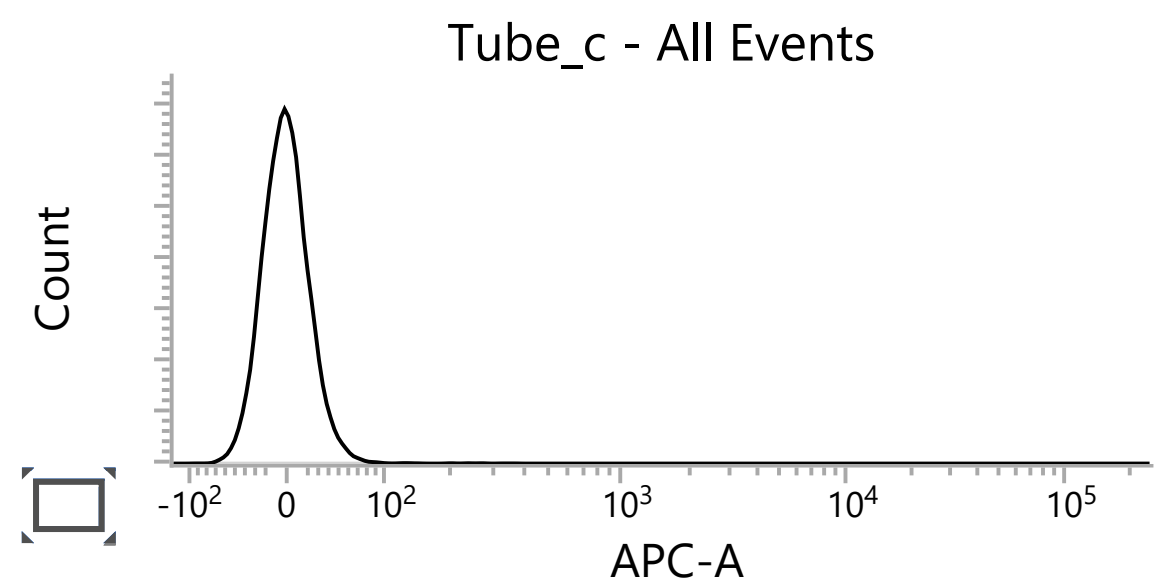

Statistics

Name	Events	% Parent	% Grandparent	% Total
Tube_a:All Events	10,000	***	***	100.00
Tube_b:All Events	10,000	***	***	100.00
Tube_c:All Events	10,000	***	***	100.00
Tube_a:Lebende Zellen	8,129	81.29	***	81.29
Tube_b:Lebende Zellen	8,200	82.00	***	82.00
Tube_c:Lebende Zellen	8,160	81.60	***	81.60

CellROX Assay

Sample ID: 0,5 ATM +NAC

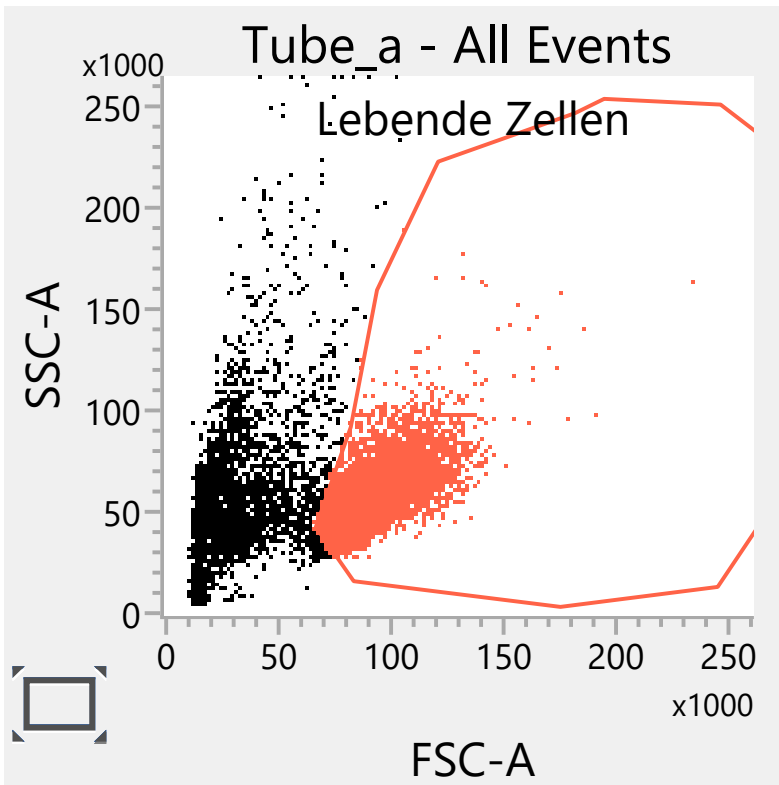

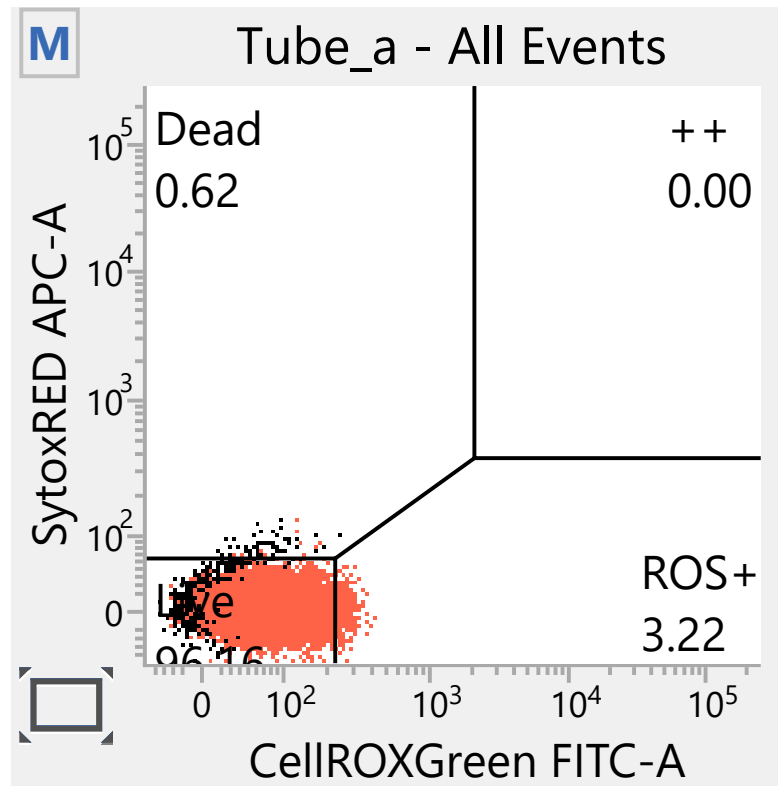

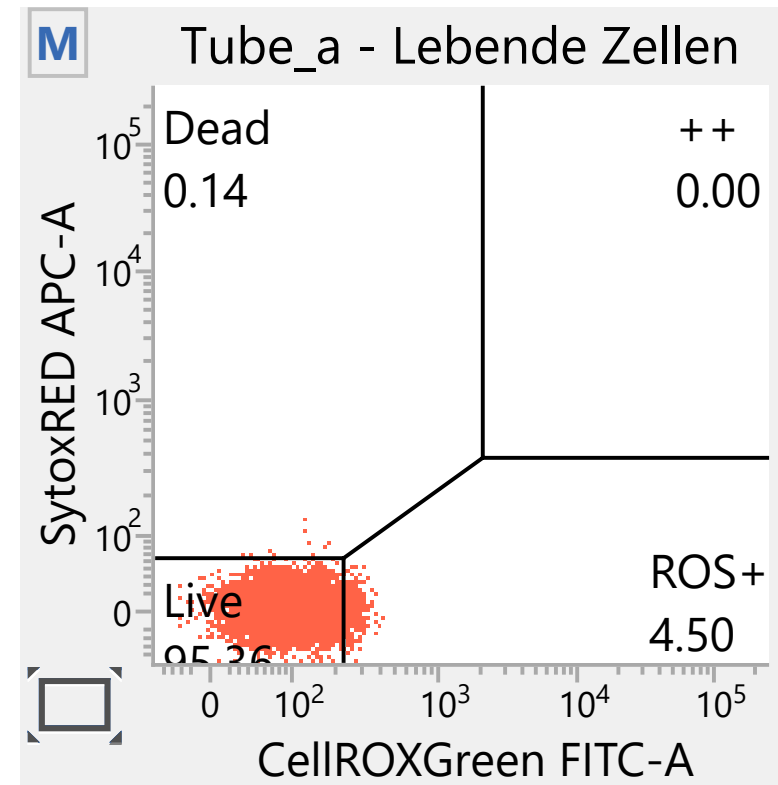

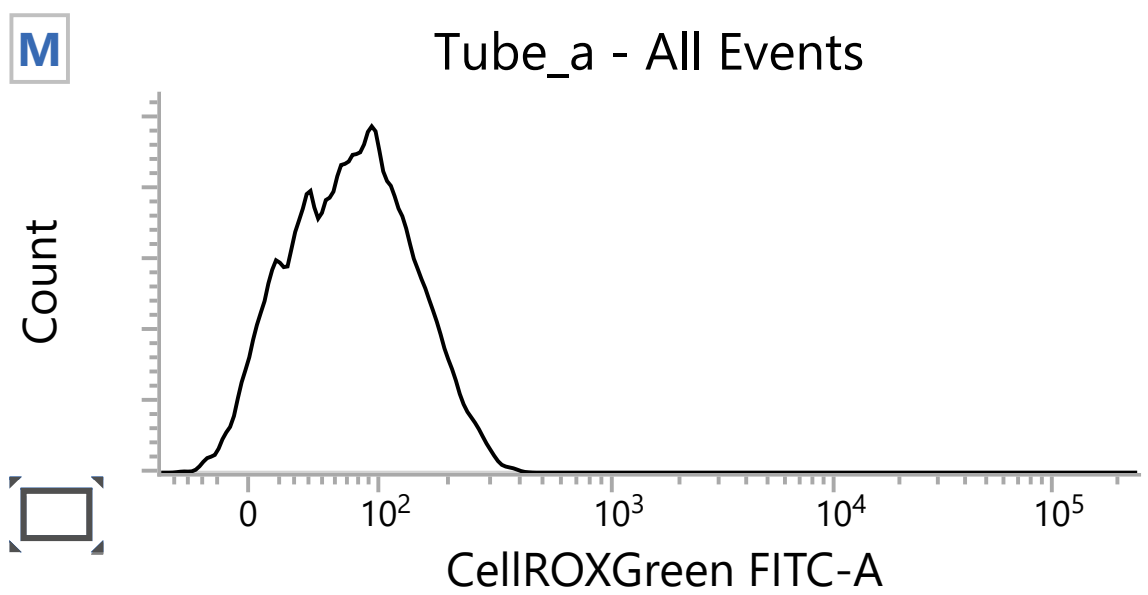

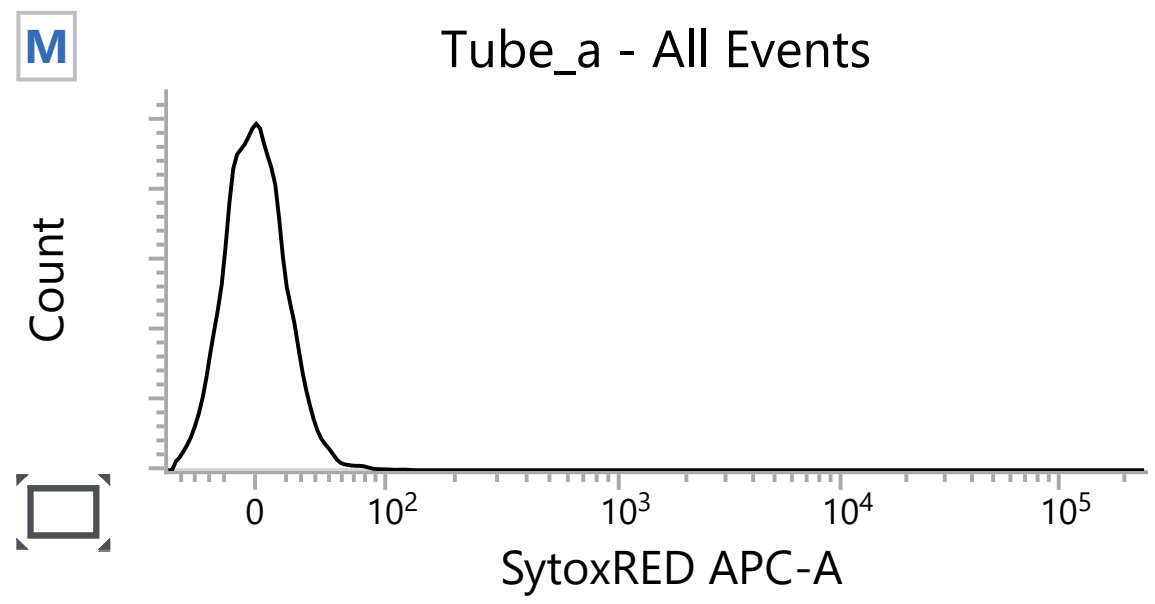

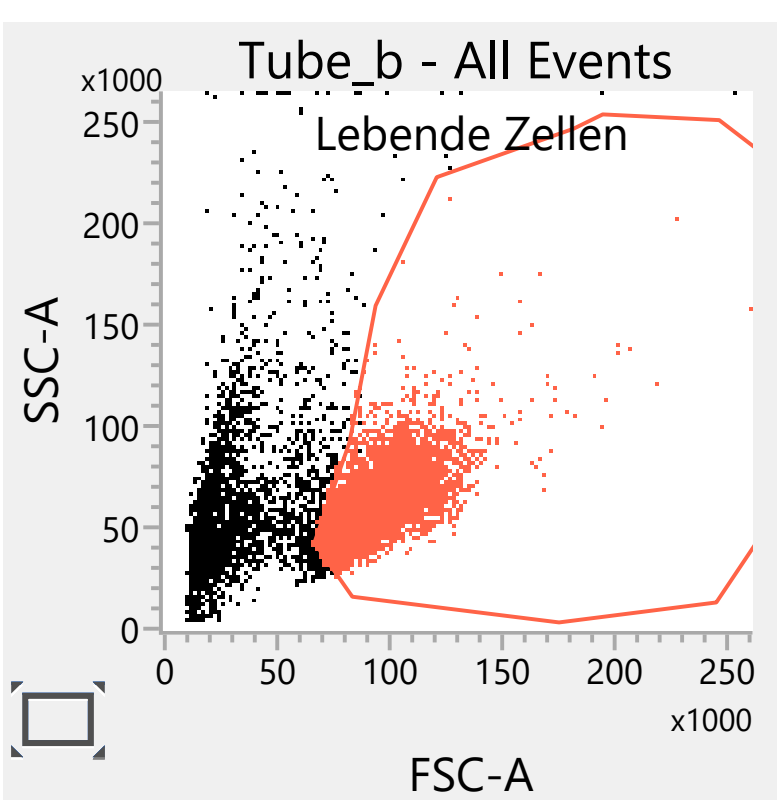

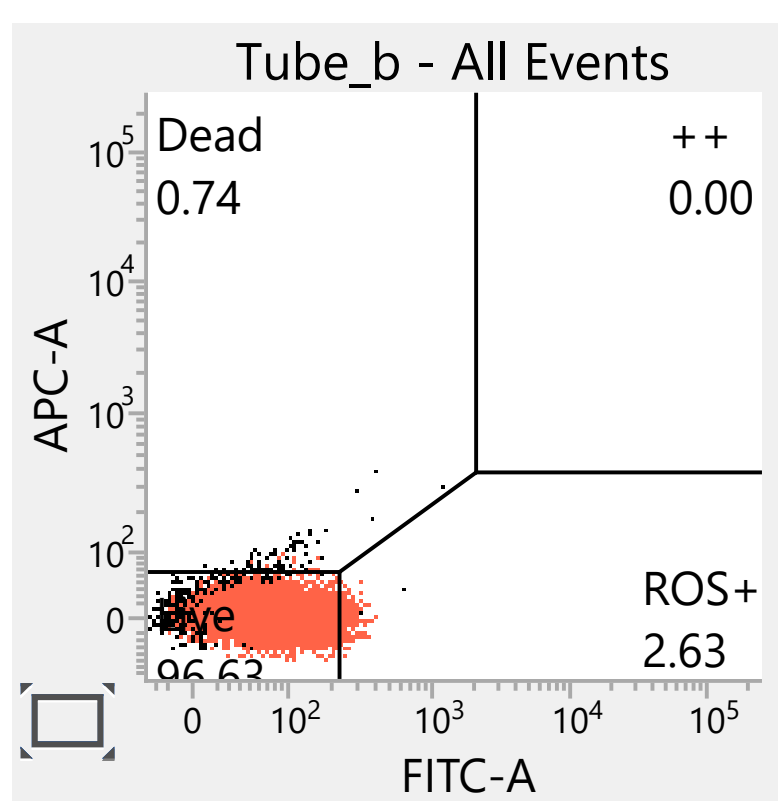

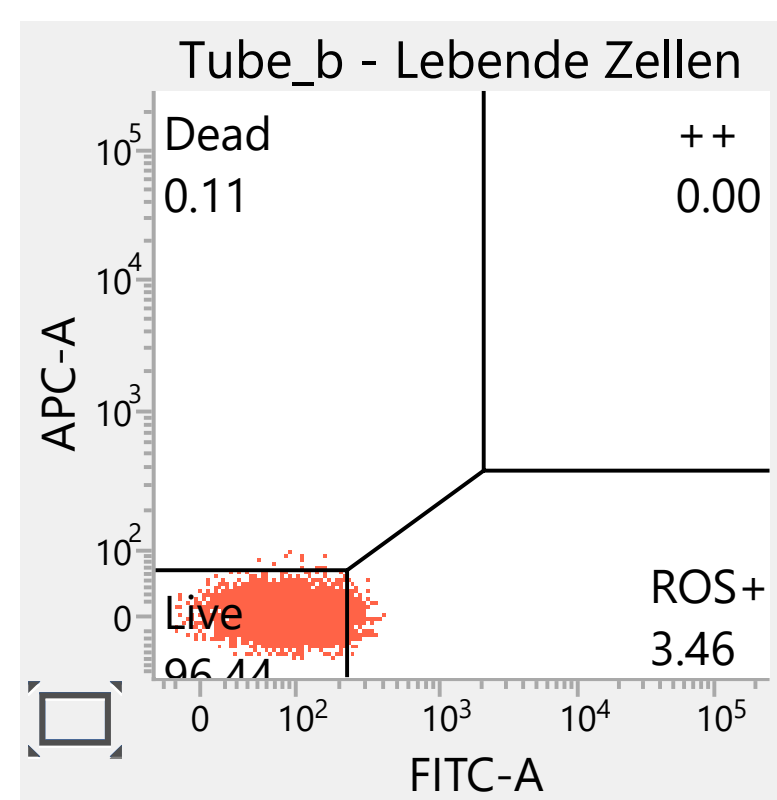

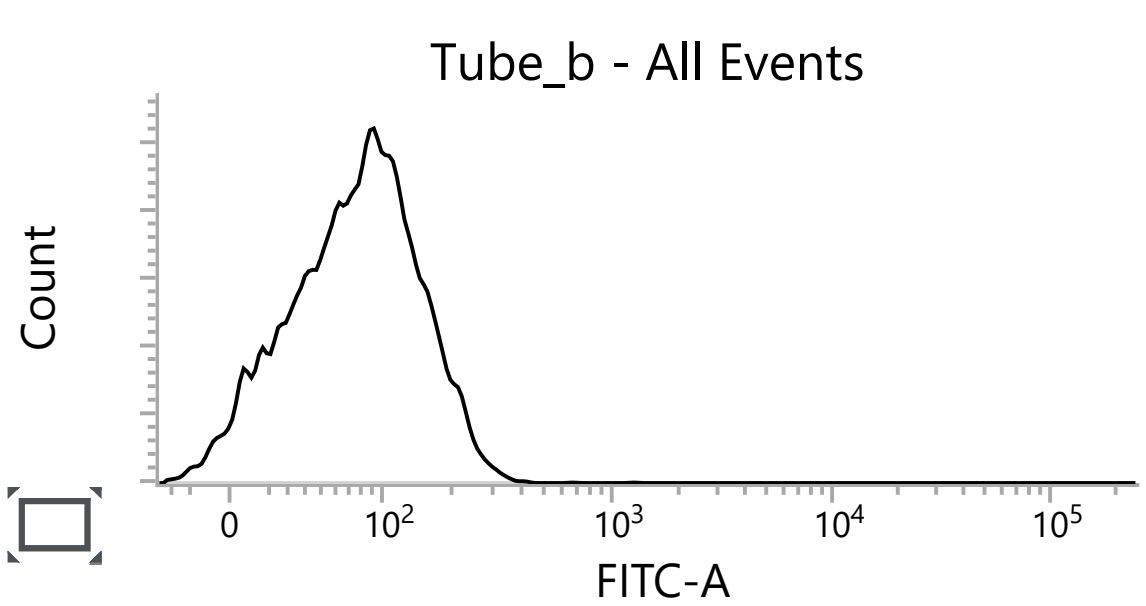

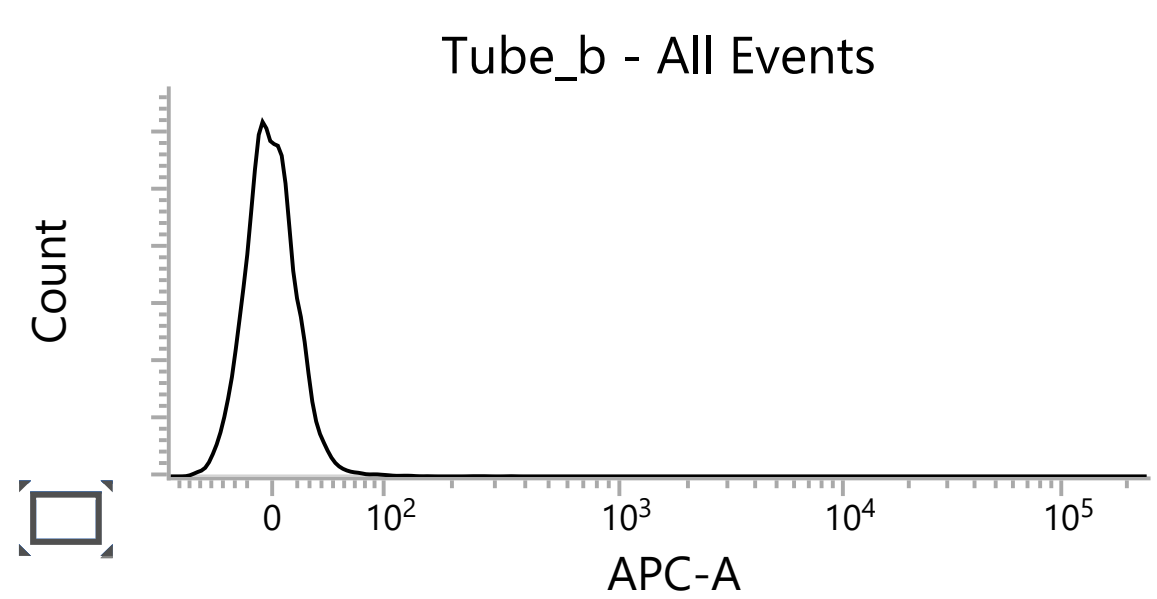

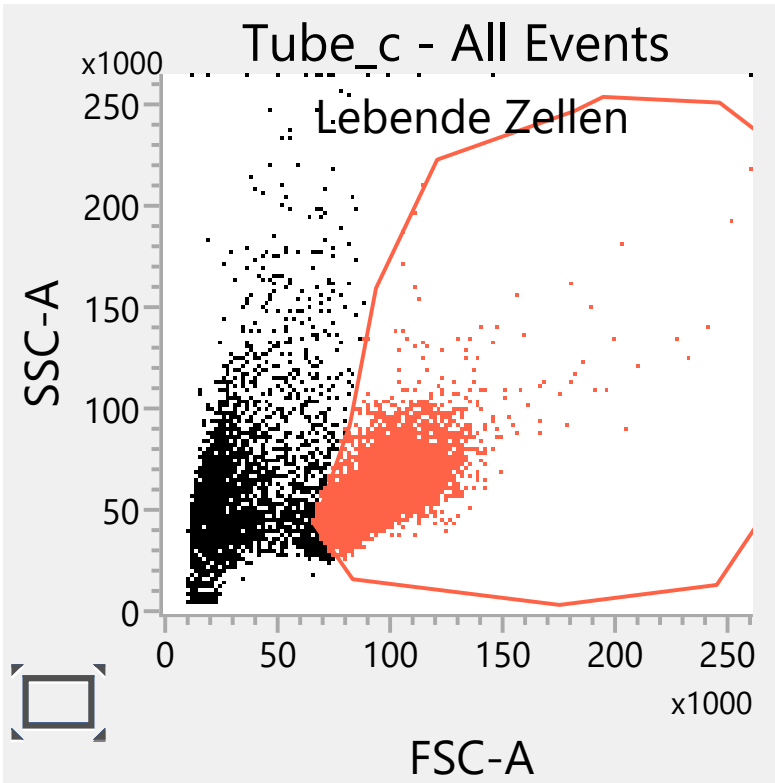

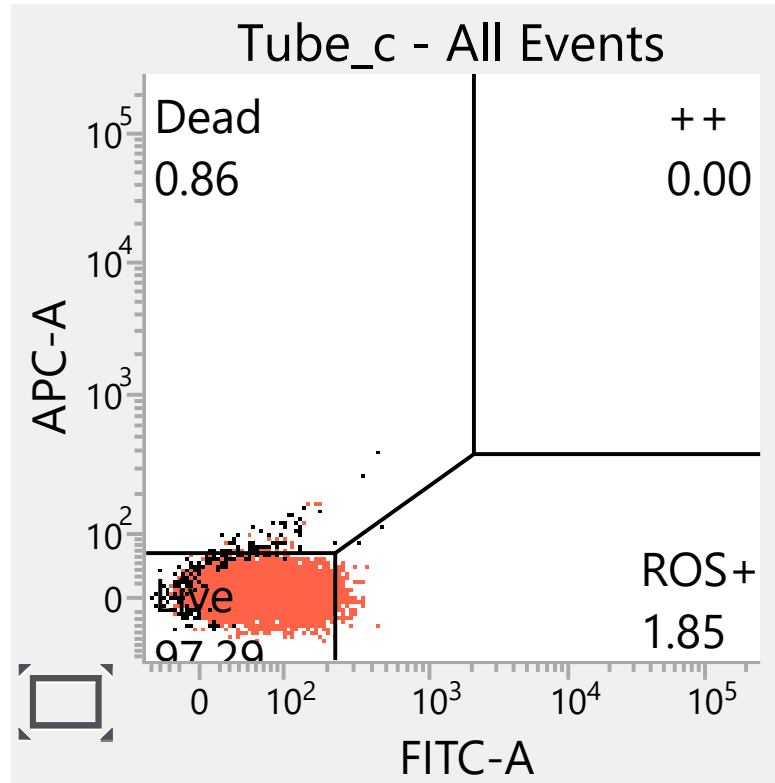

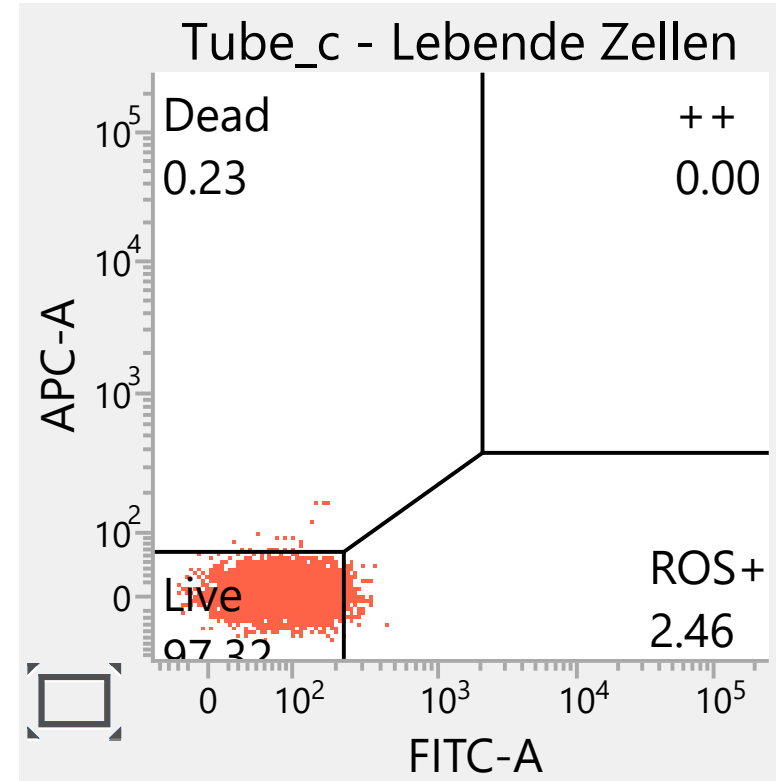

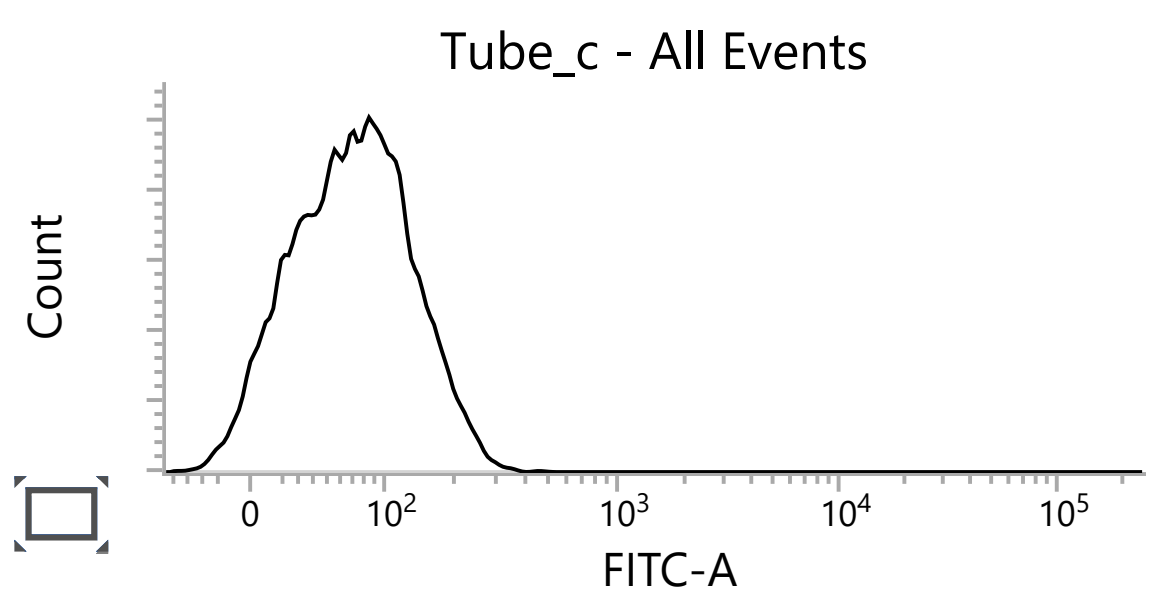

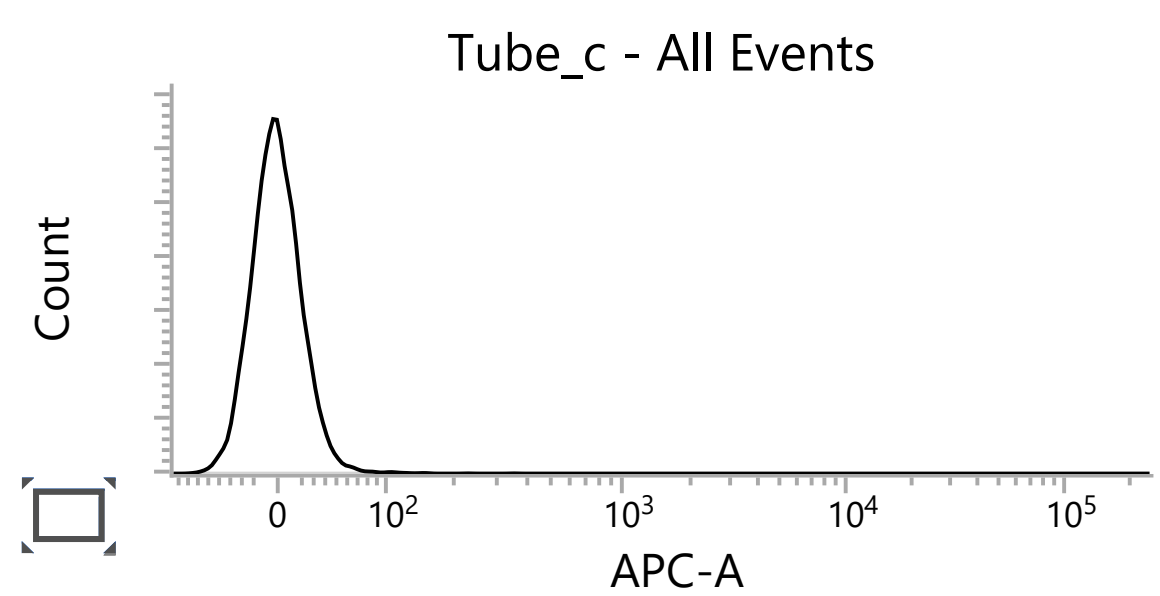

Statistics

Name	Events	% Parent	% Grandparent	% Total
Tube_a:All Events	10,000	***	***	100.00
Tube_a:Lebende Zellen	7,149	71.49	***	71.49
Tube_b:All Events	10,000	***	***	100.00
Tube_b:Lebende Zellen	7,550	75.50	***	75.50
Tube_c:All Events	10,000	***	***	100.00
Tube_c:Lebende Zellen	7,453	74.53	***	74.53

CellROX Assay

Sample ID: 0,5 ATM

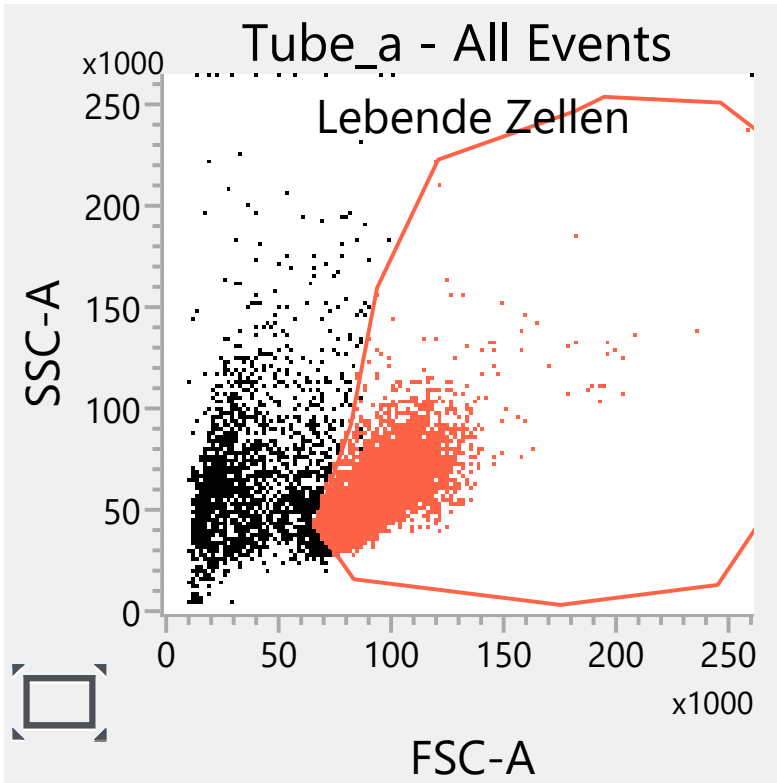

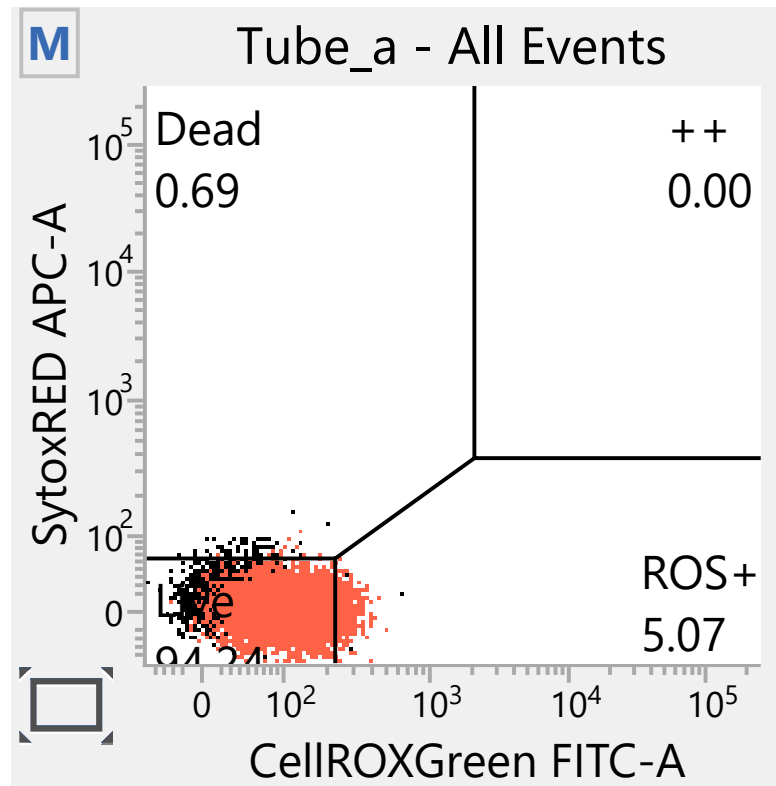

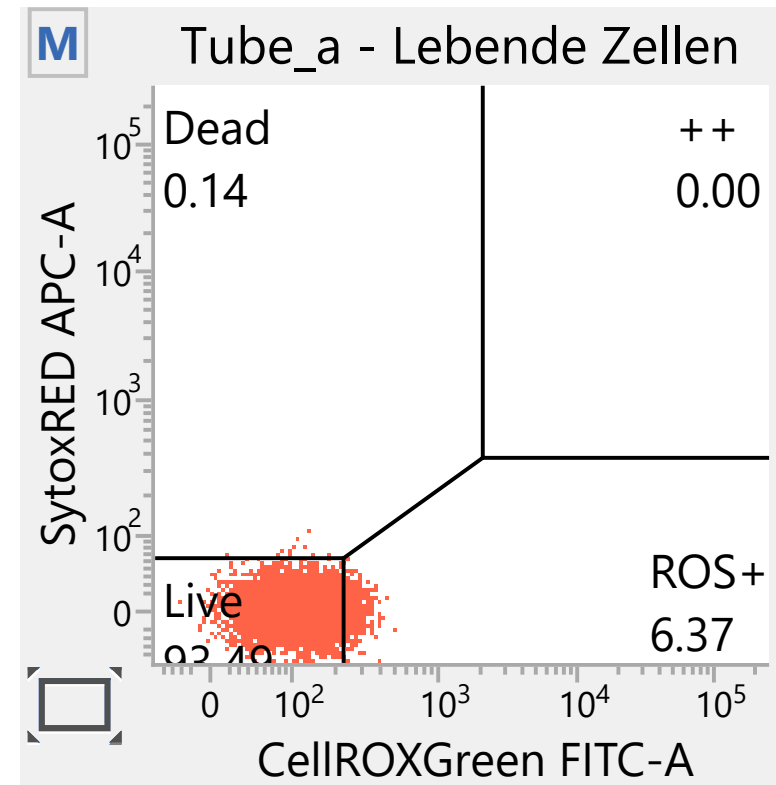

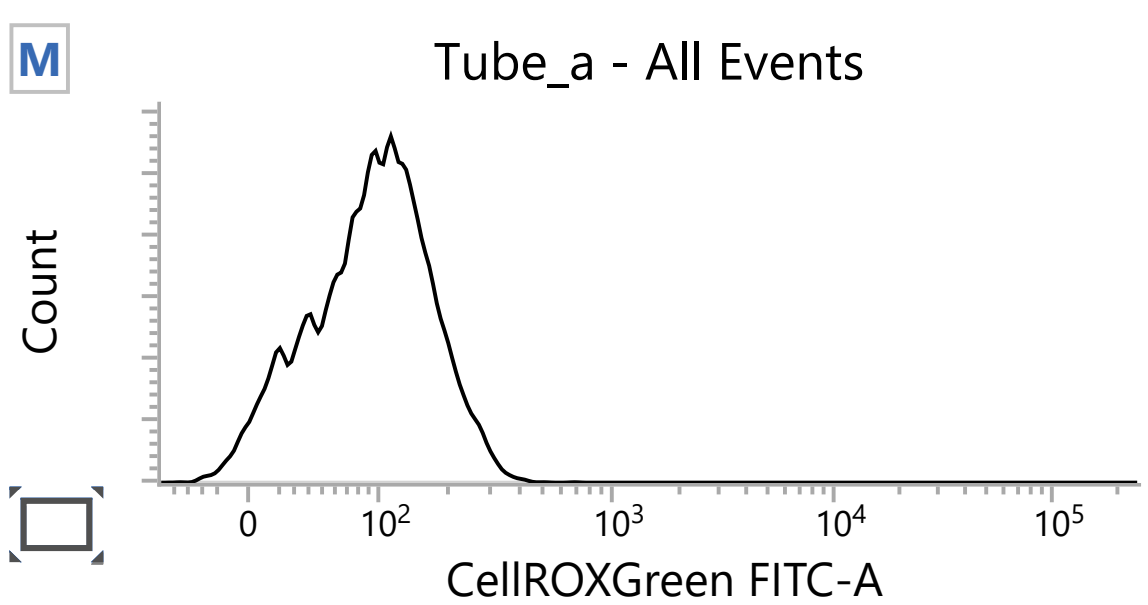

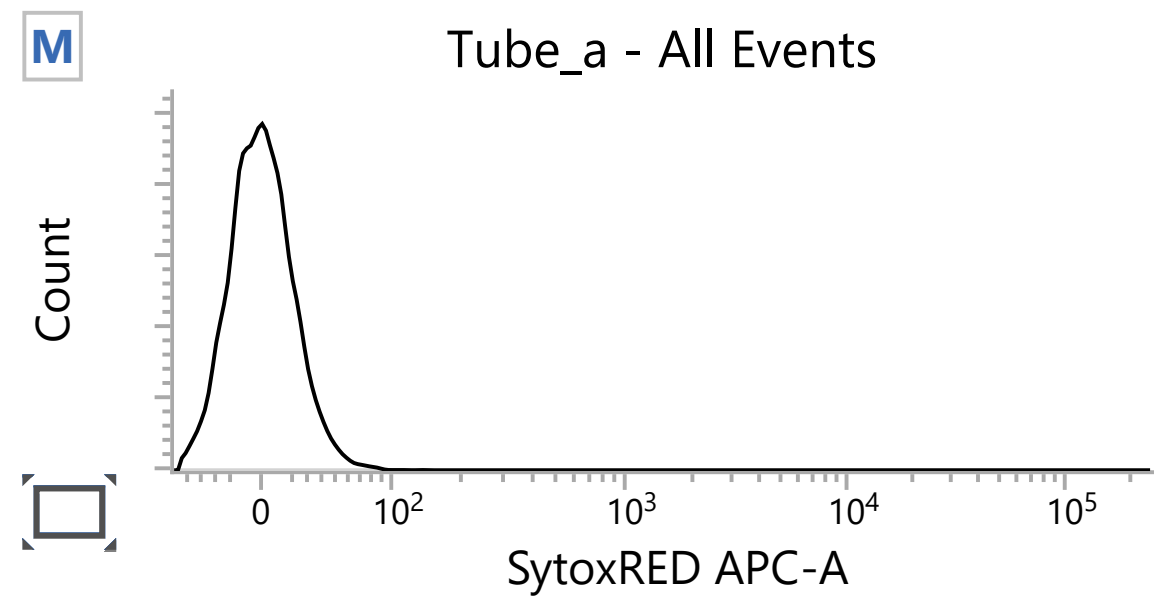

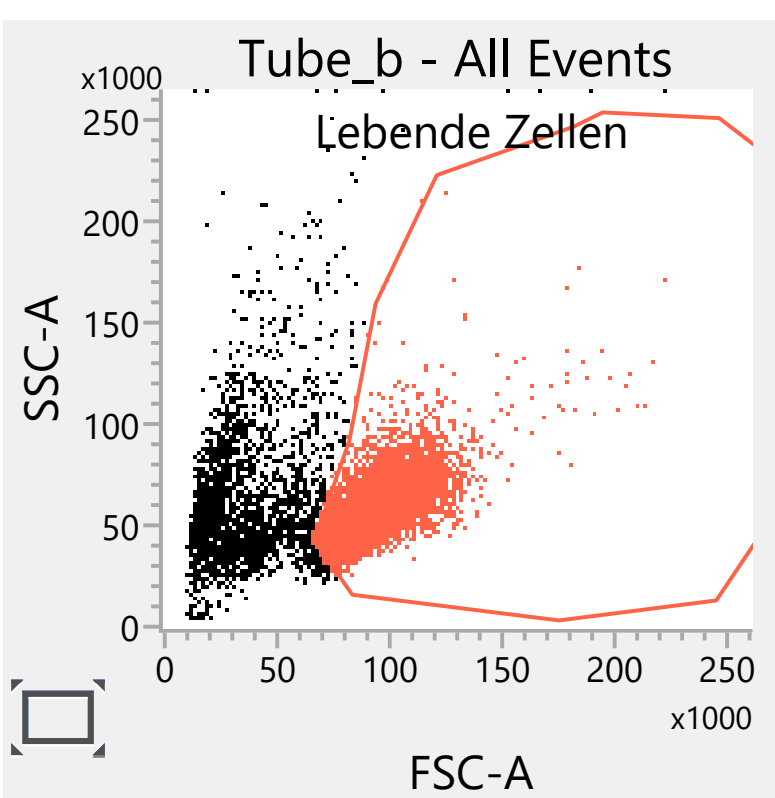

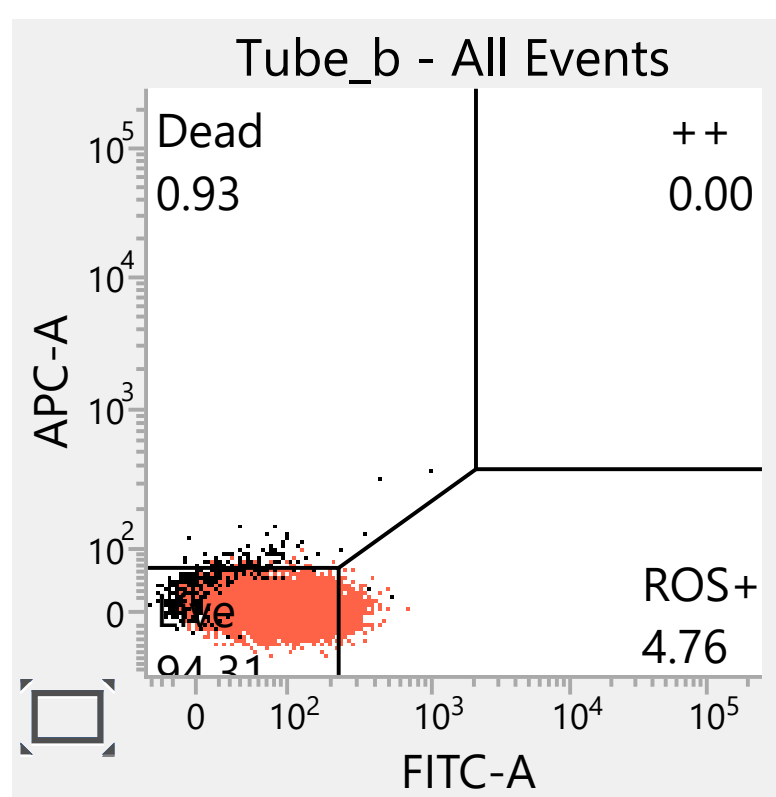

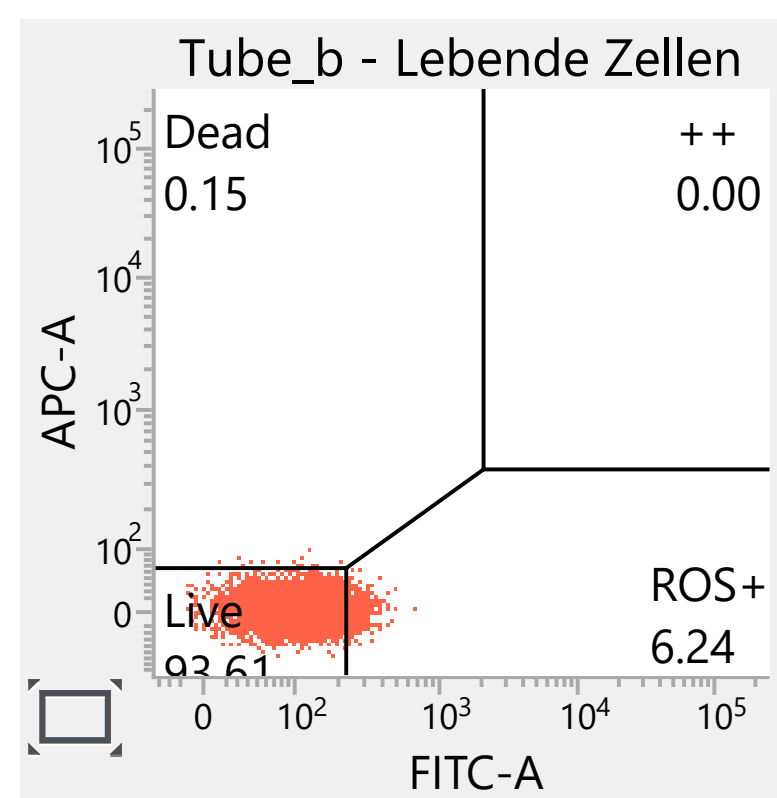

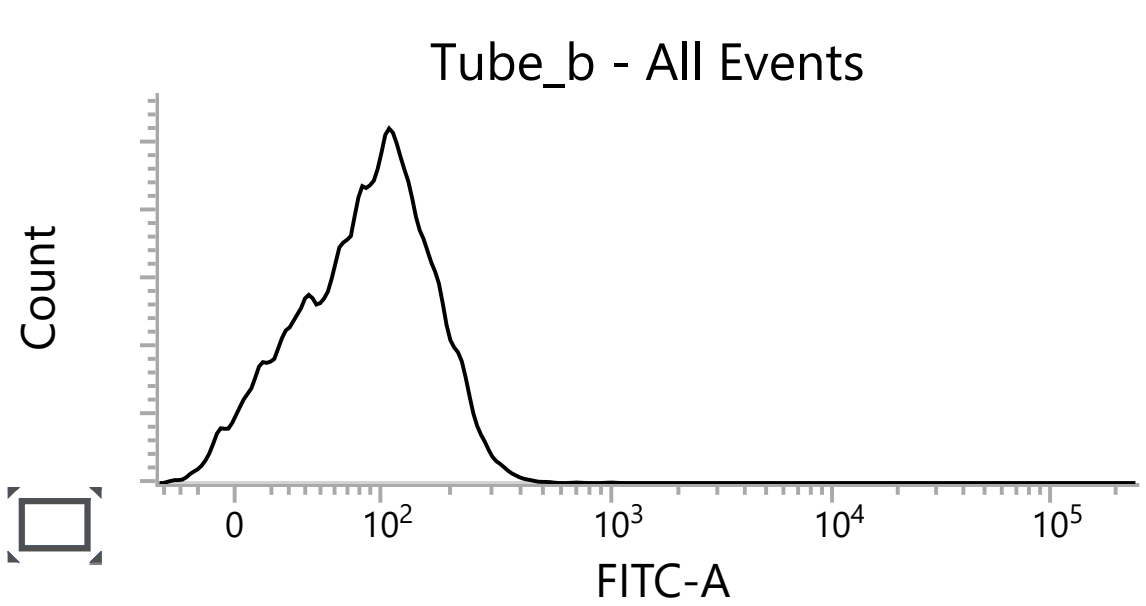

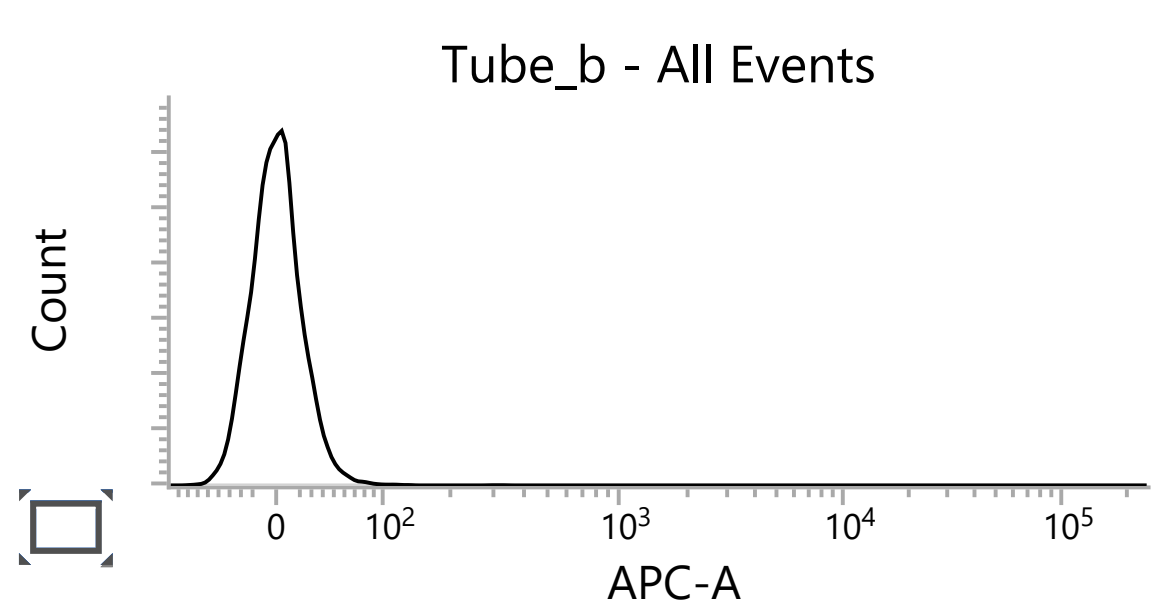

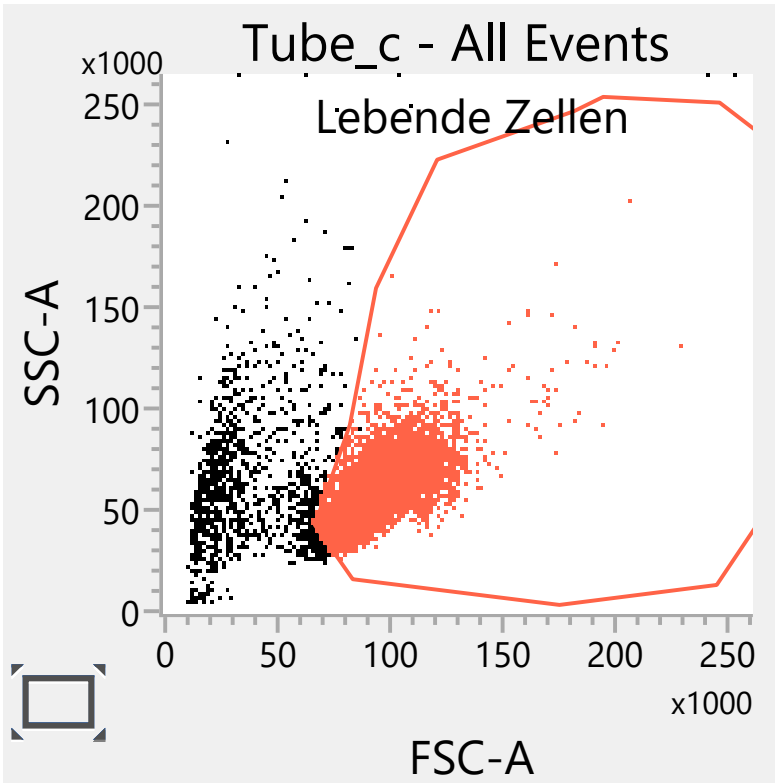

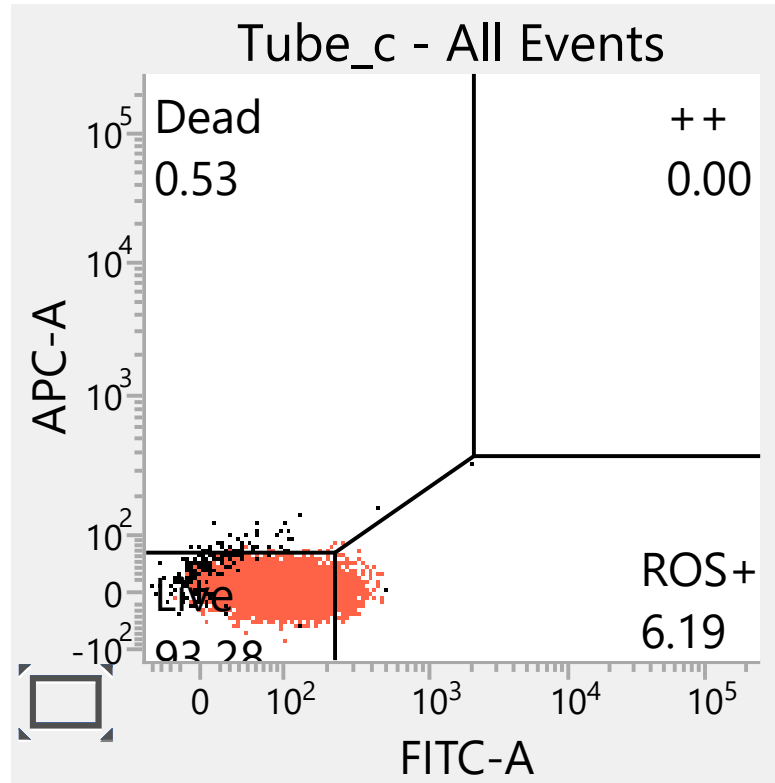

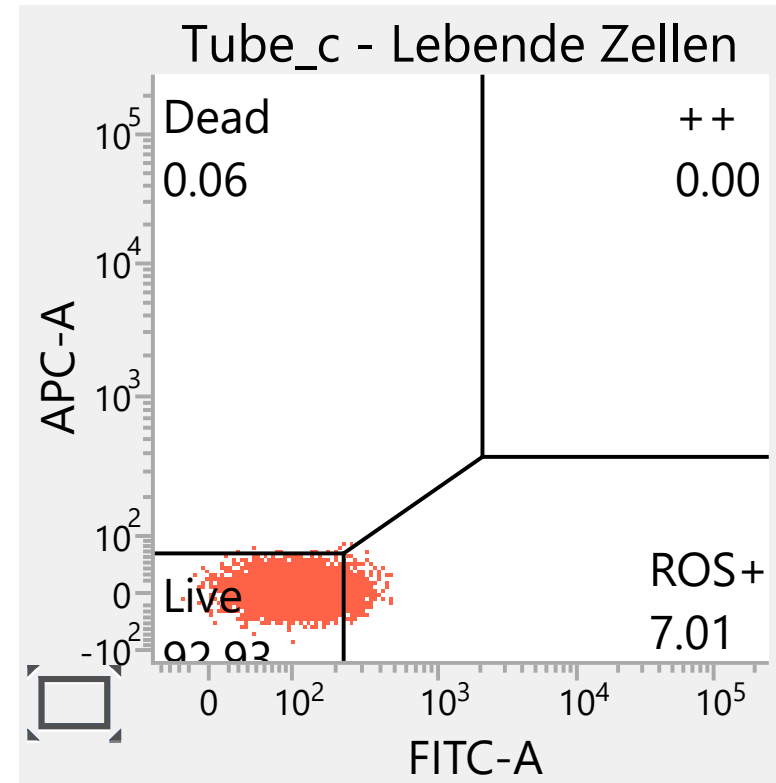

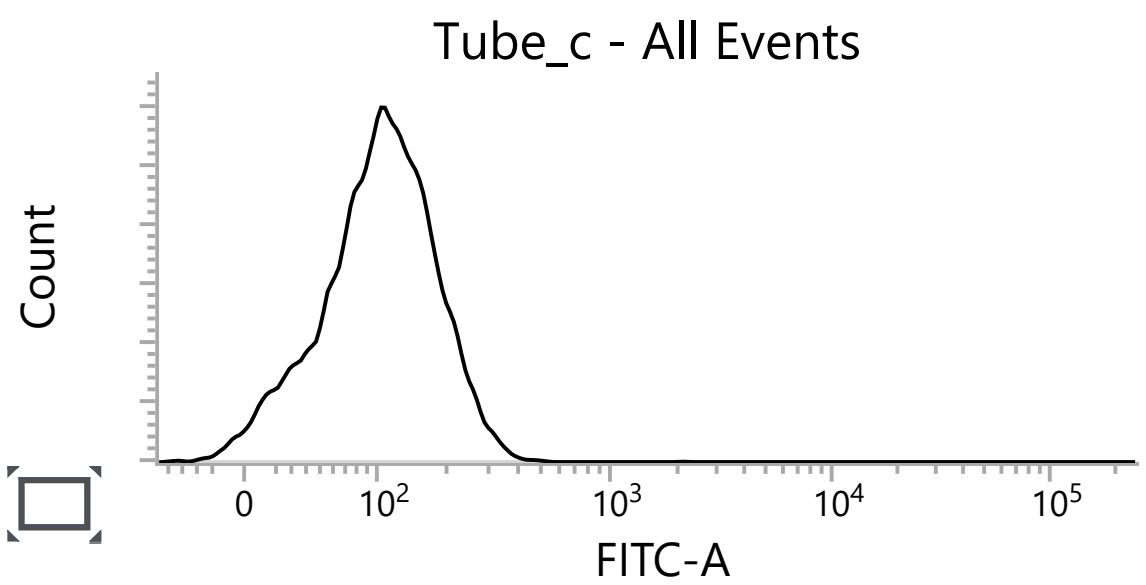

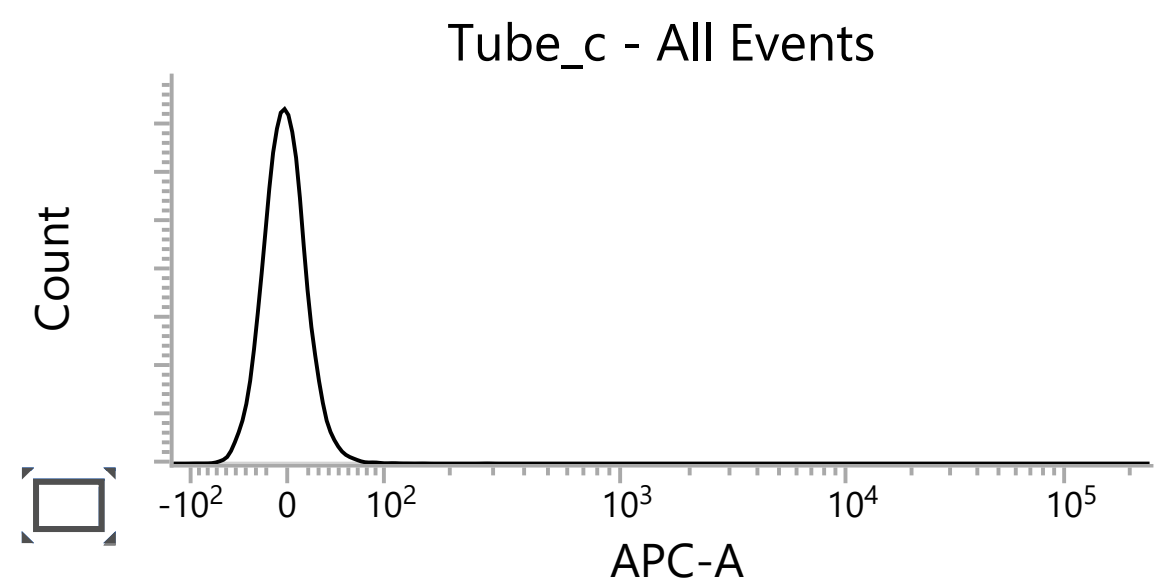

Statistics

Name	Events	% Parent	% Grandparent	% Total
Tube_a:All Events	10,000	***	***	100.00
Tube_a:Lebende Zellen	7,912	79.12	***	79.12
Tube_b:All Events	10,000	***	***	100.00
Tube_b:Lebende Zellen	7,575	75.75	***	75.75
Tube_c:All Events	10,000	***	***	100.00
Tube_c:Lebende Zellen	8,744	87.44	***	87.44

CellROX Assay

Sample ID: 1 ATM

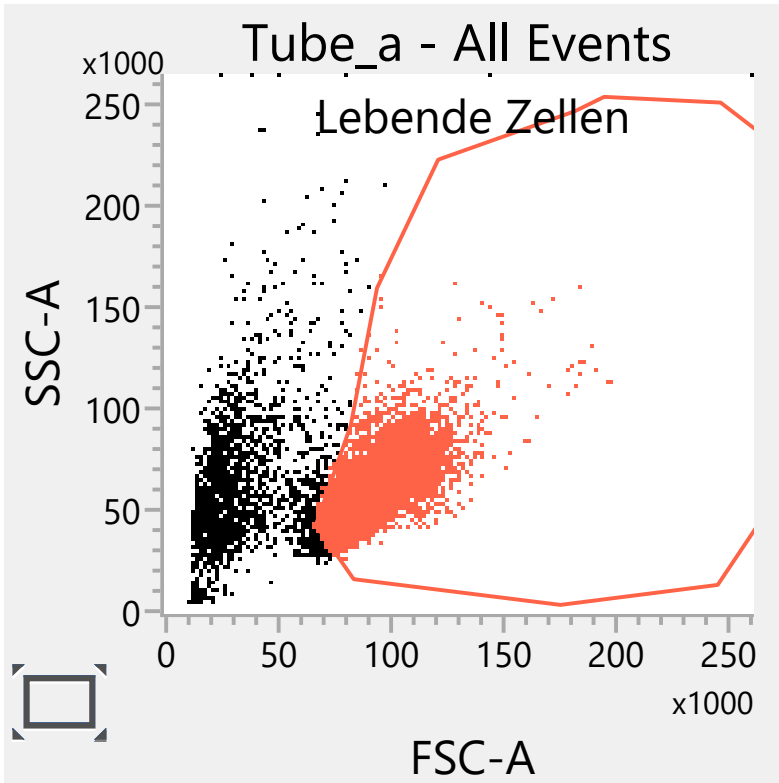

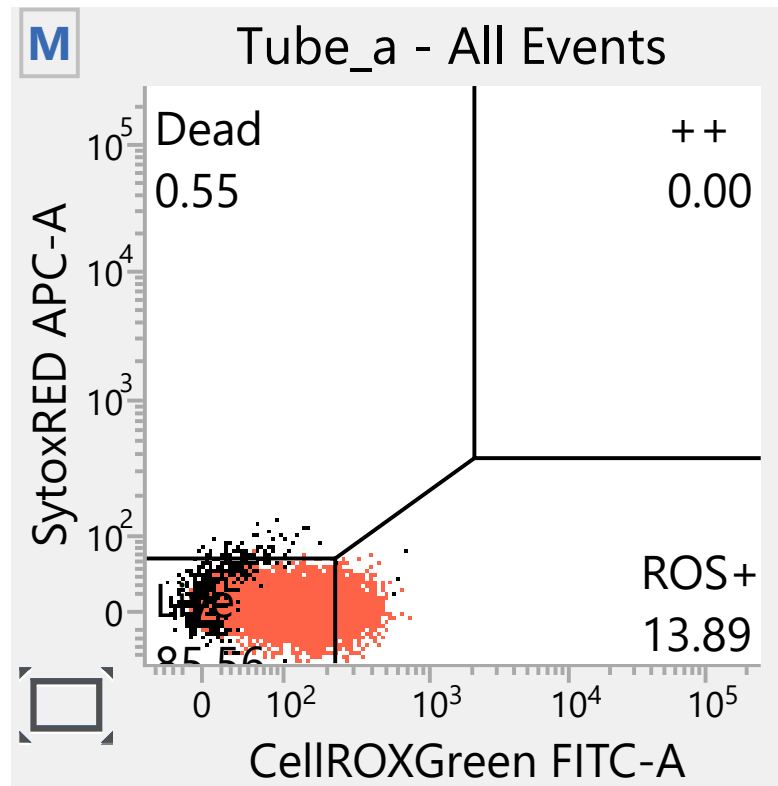

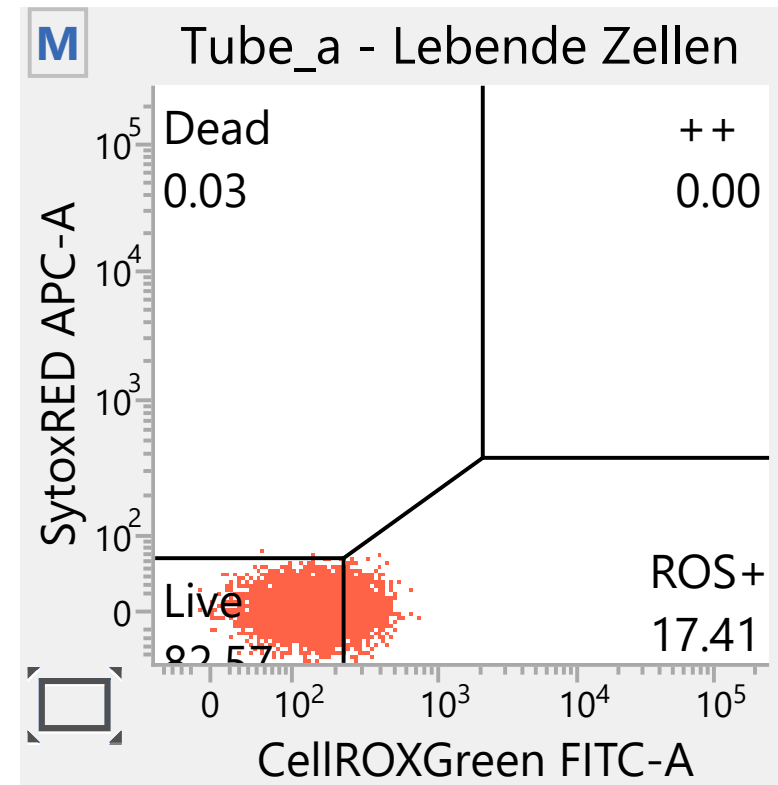

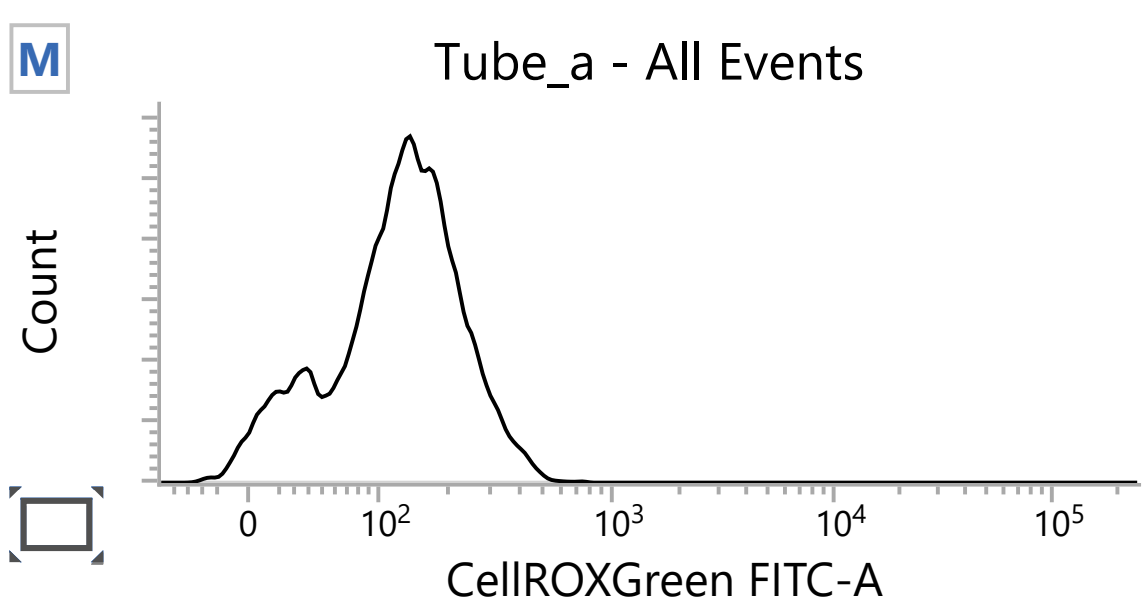

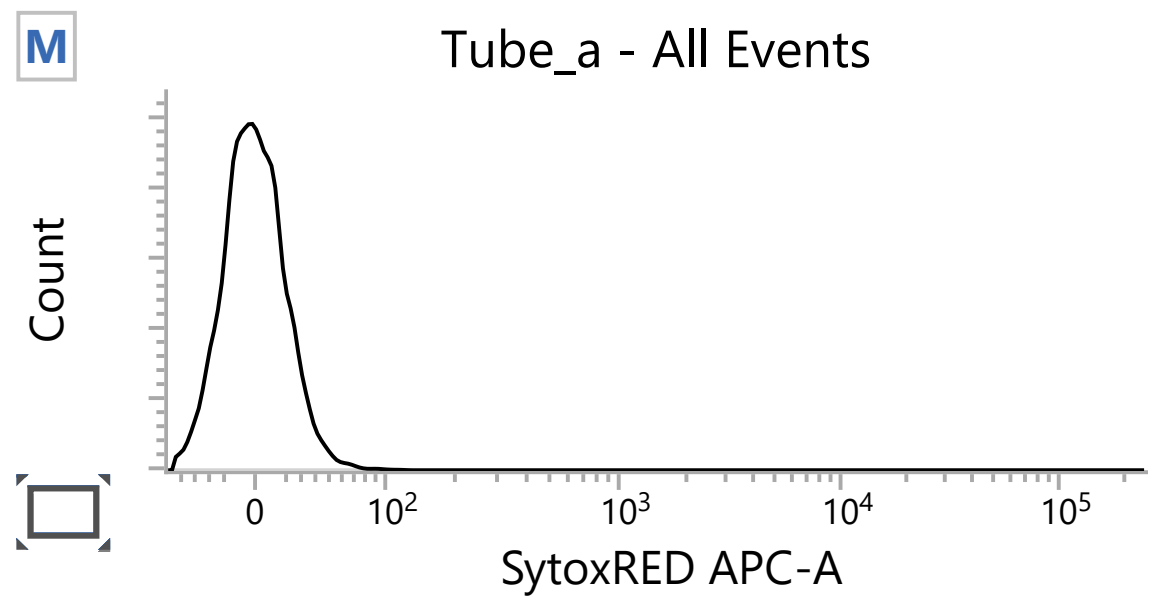

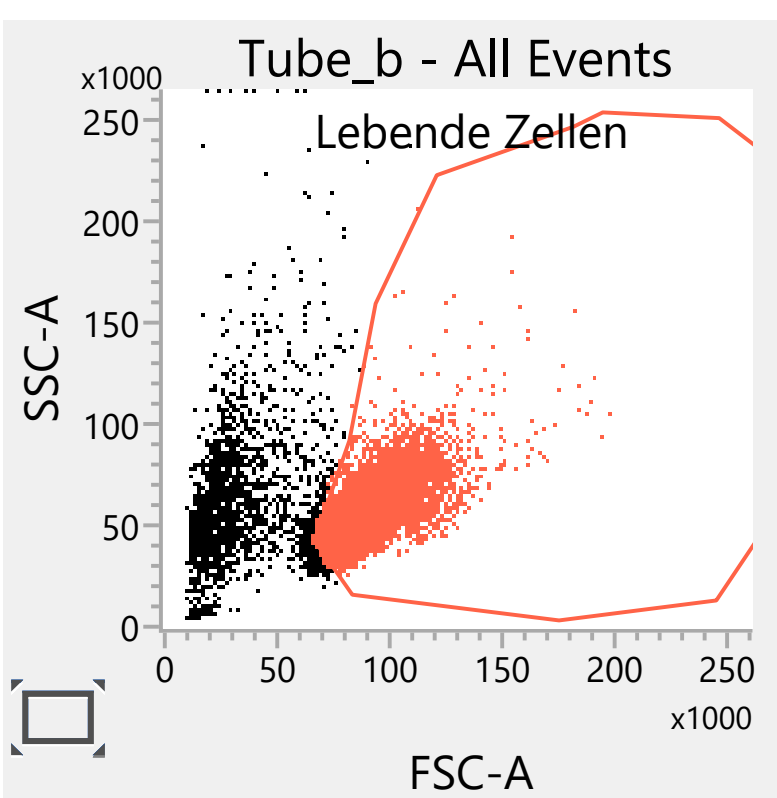

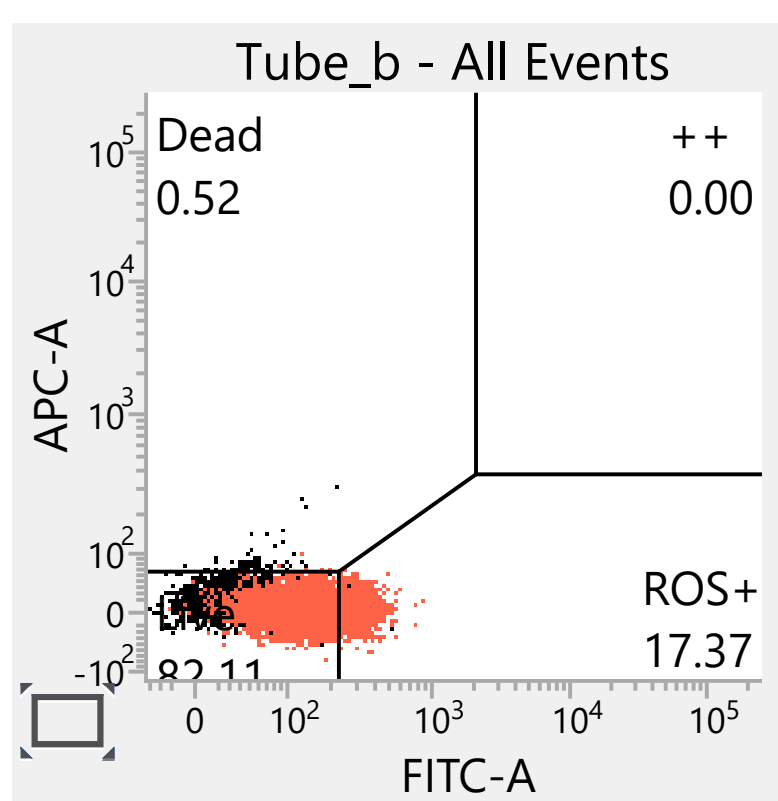

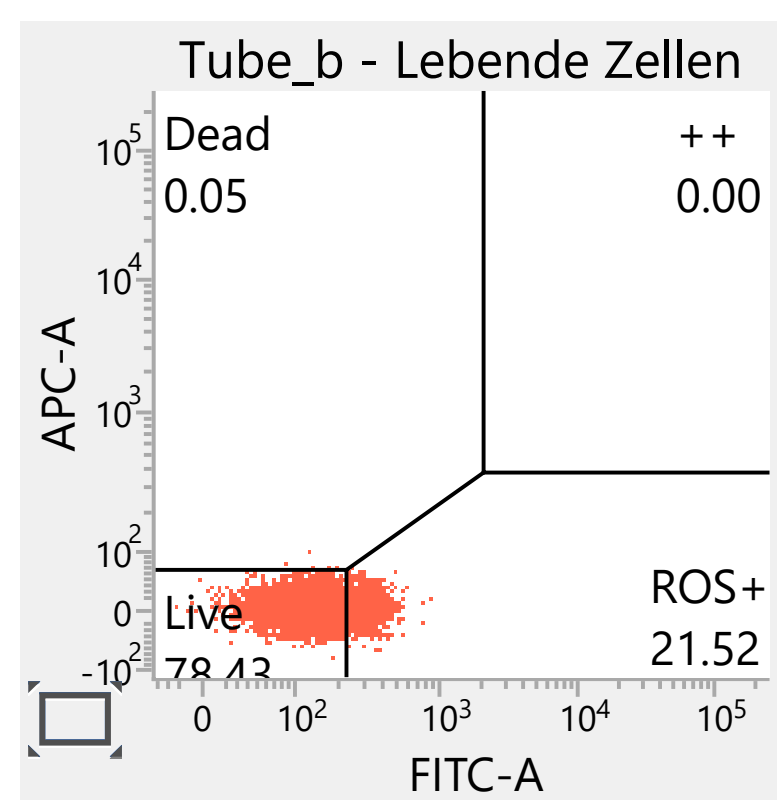

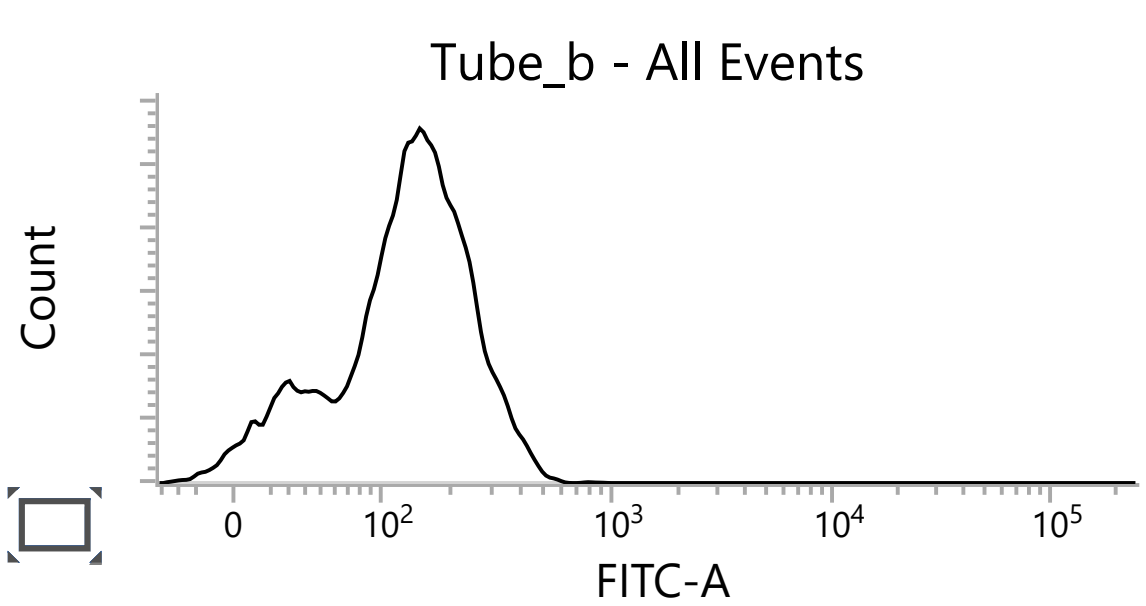

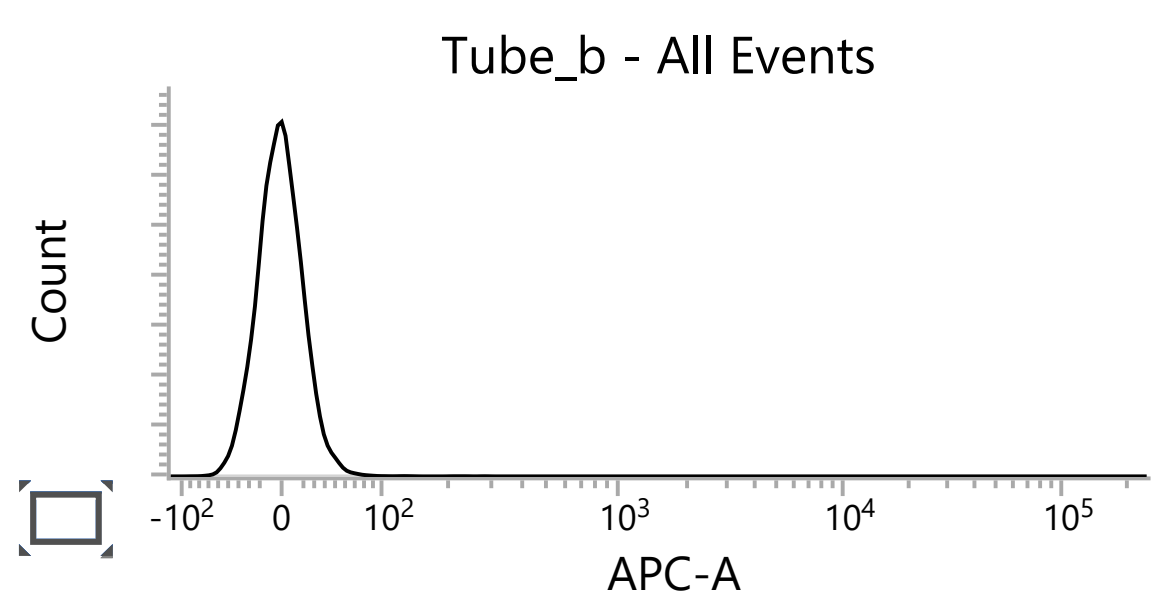

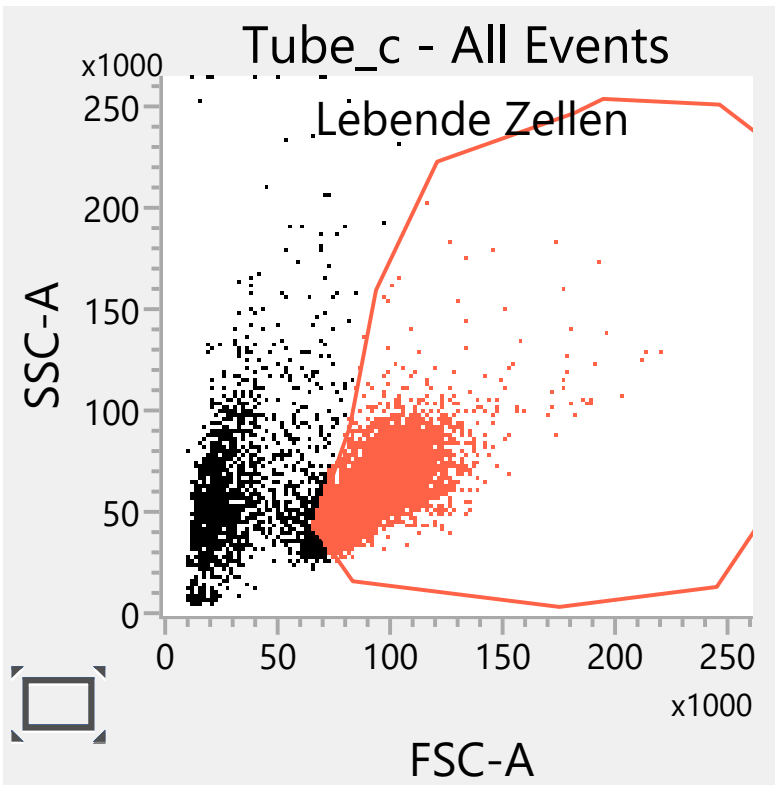

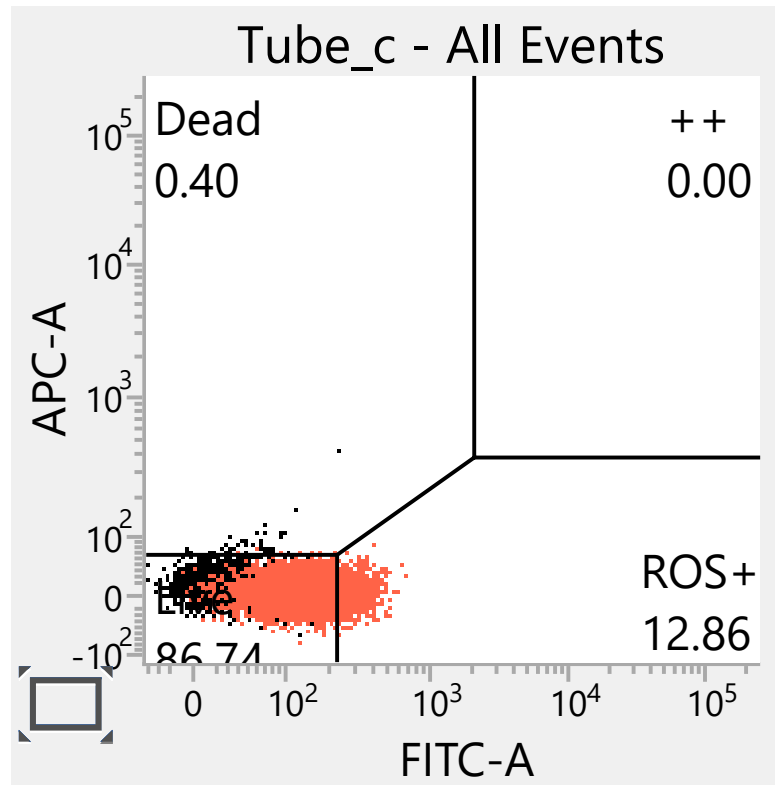

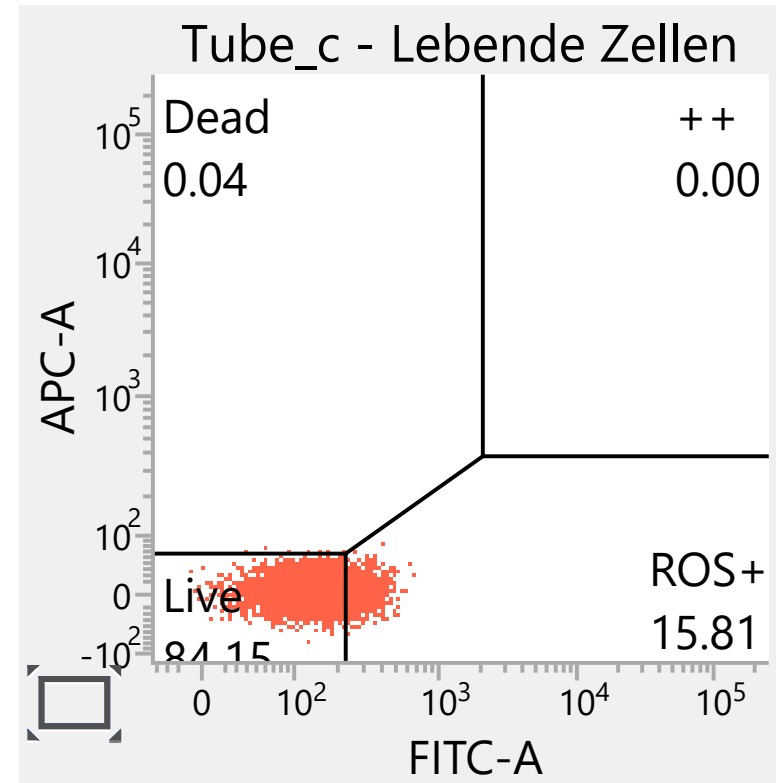

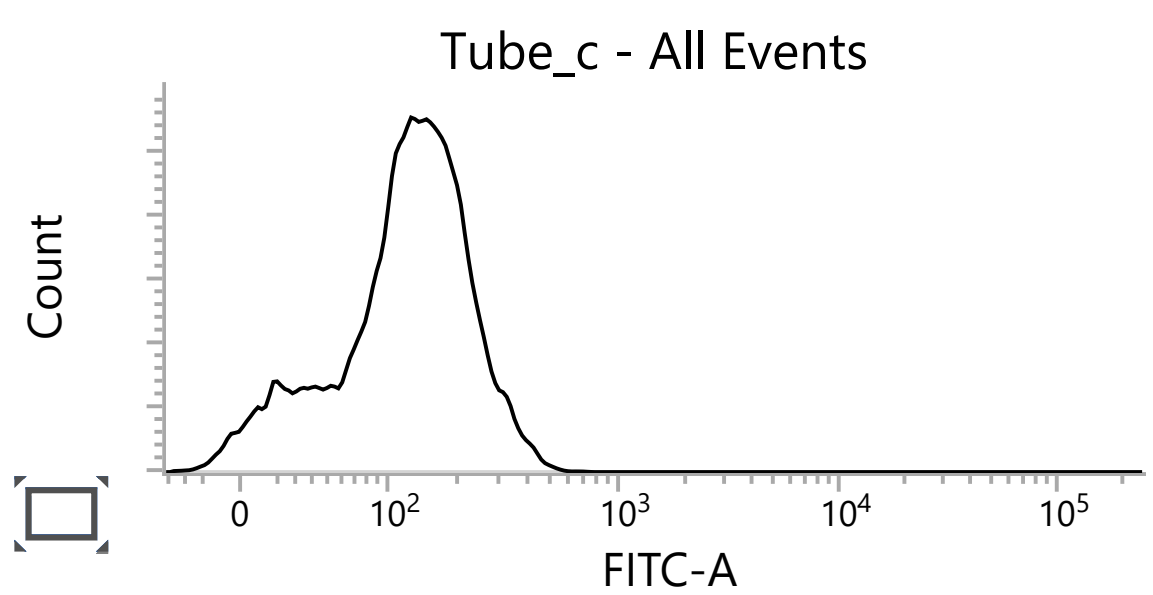

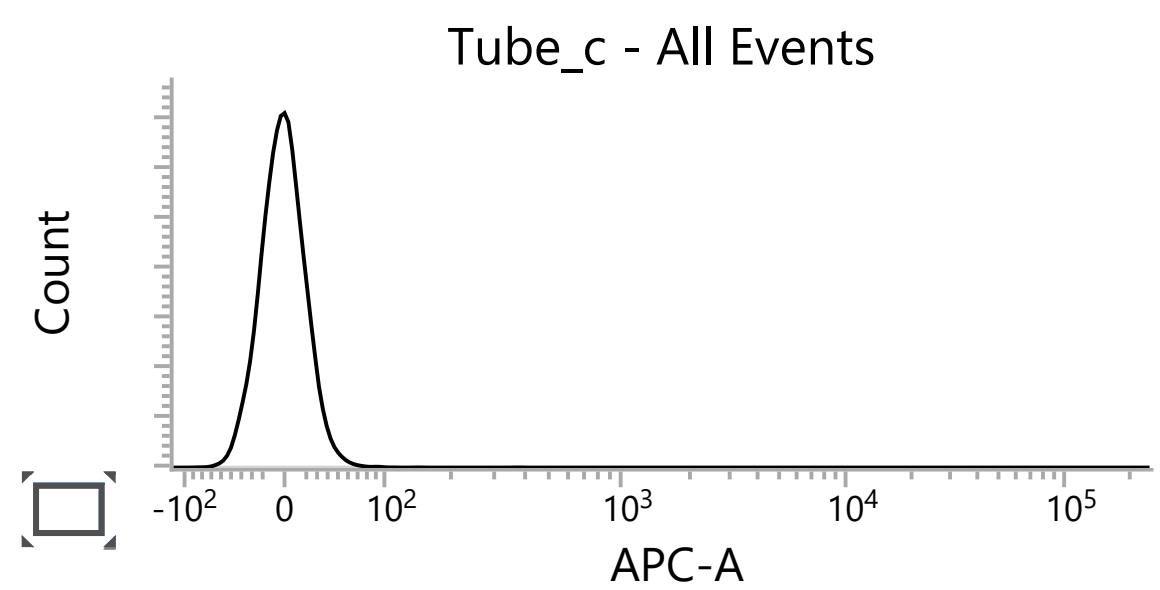

Statistics

Name	Events	% Parent	% Grandparent	% Total
Tube_a:All Events	10,000	***	***	100.00
Tube_a:Lebende Zellen	7,893	78.93	***	78.93
Tube_b:All Events	10,000	***	***	100.00
Tube_b:Lebende Zellen	7,980	79.80	***	79.80
Tube_c:All Events	10,000	***	***	100.00
Tube_c:Lebende Zellen	8,033	80.33	***	80.33

CellROX Assay

Sample ID: 1ATM +NAC

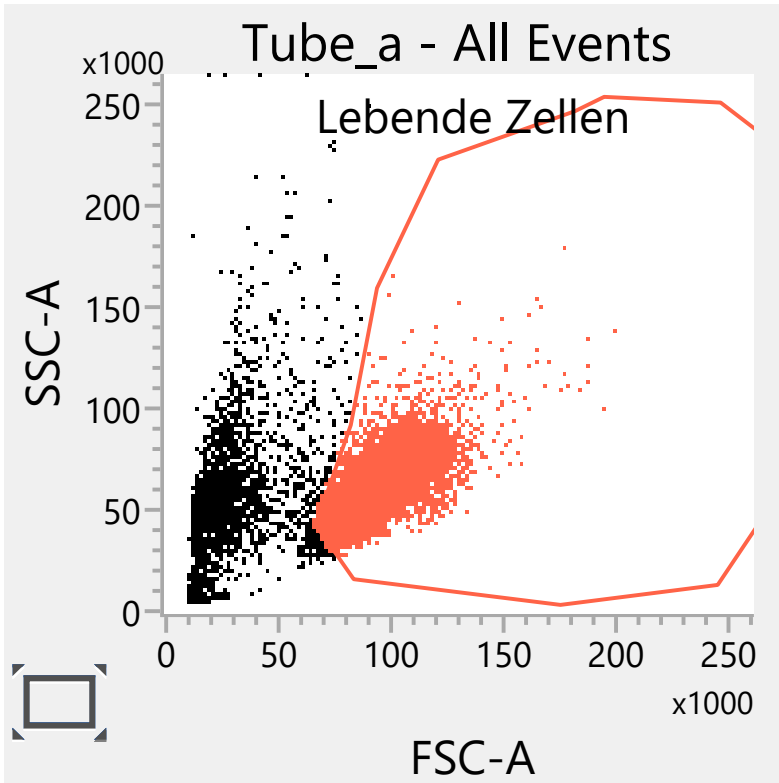

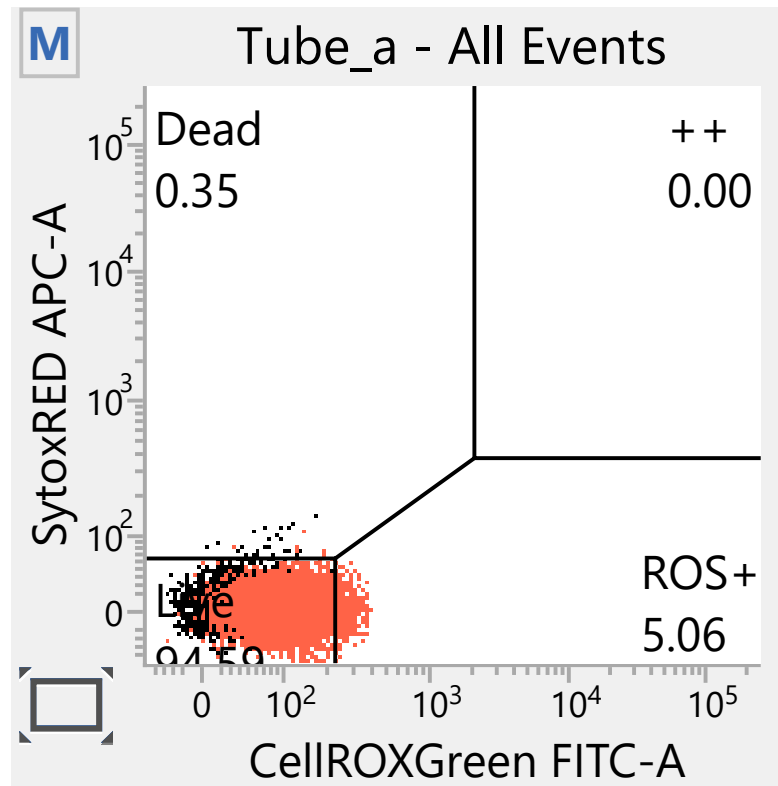

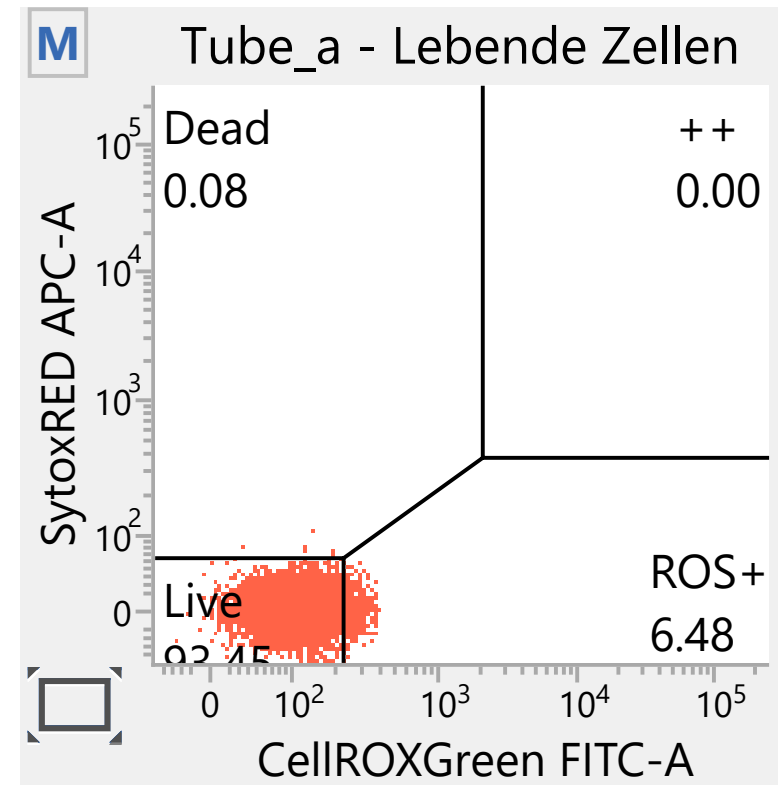

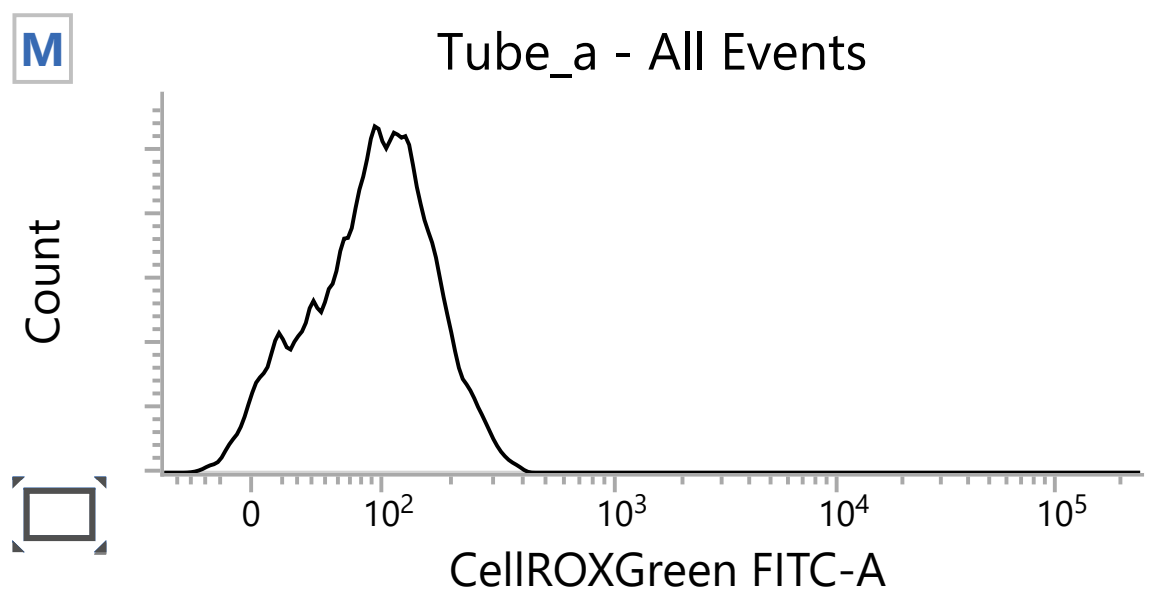

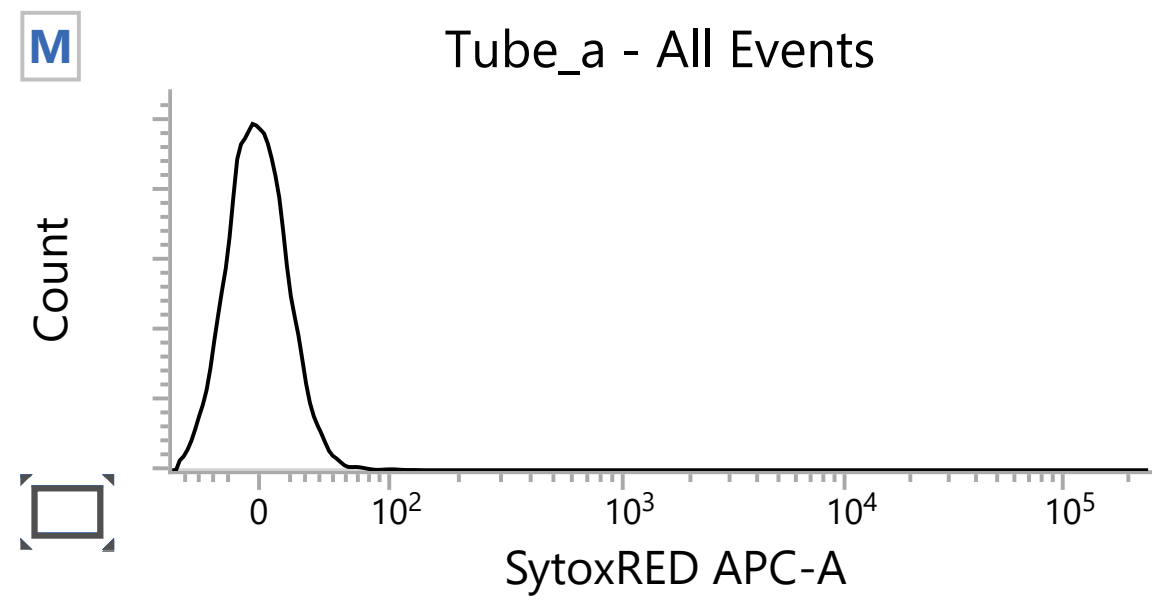

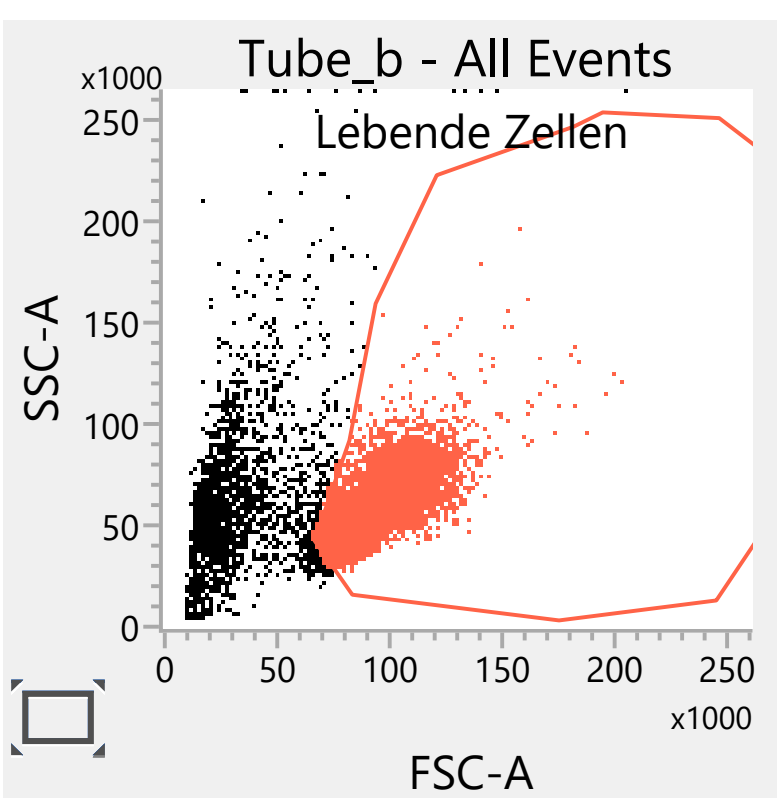

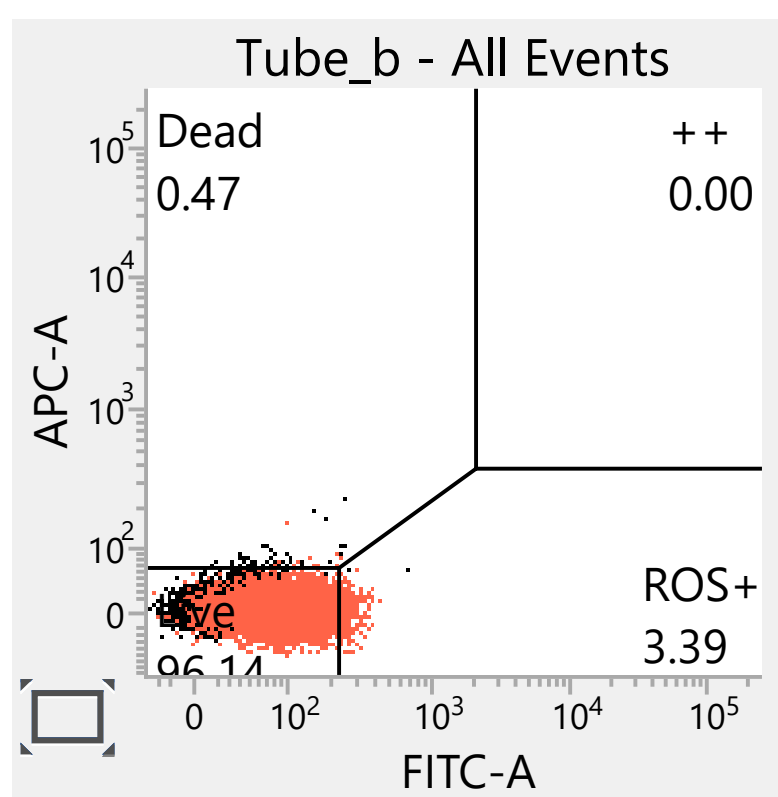

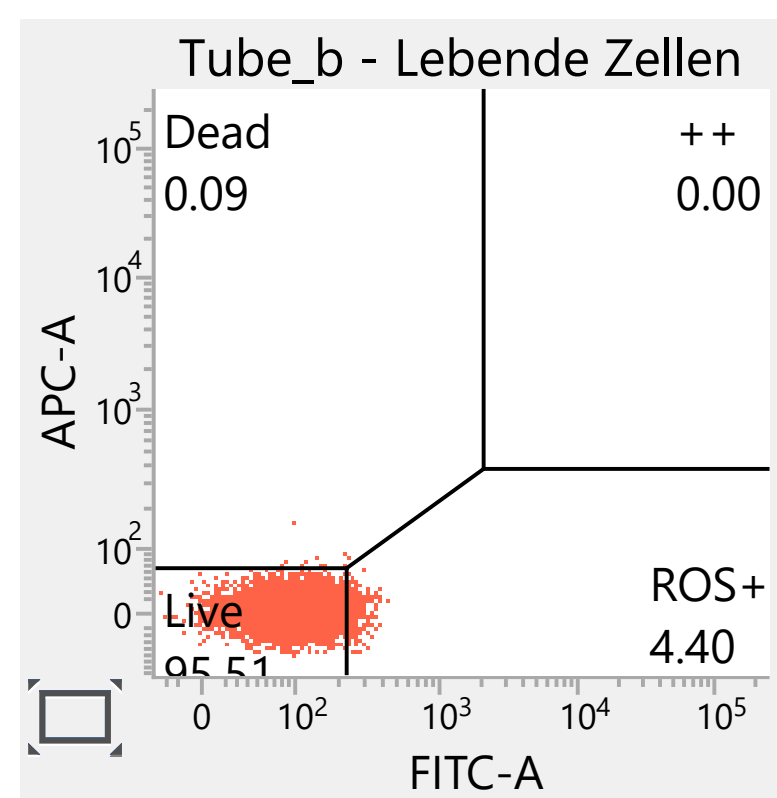

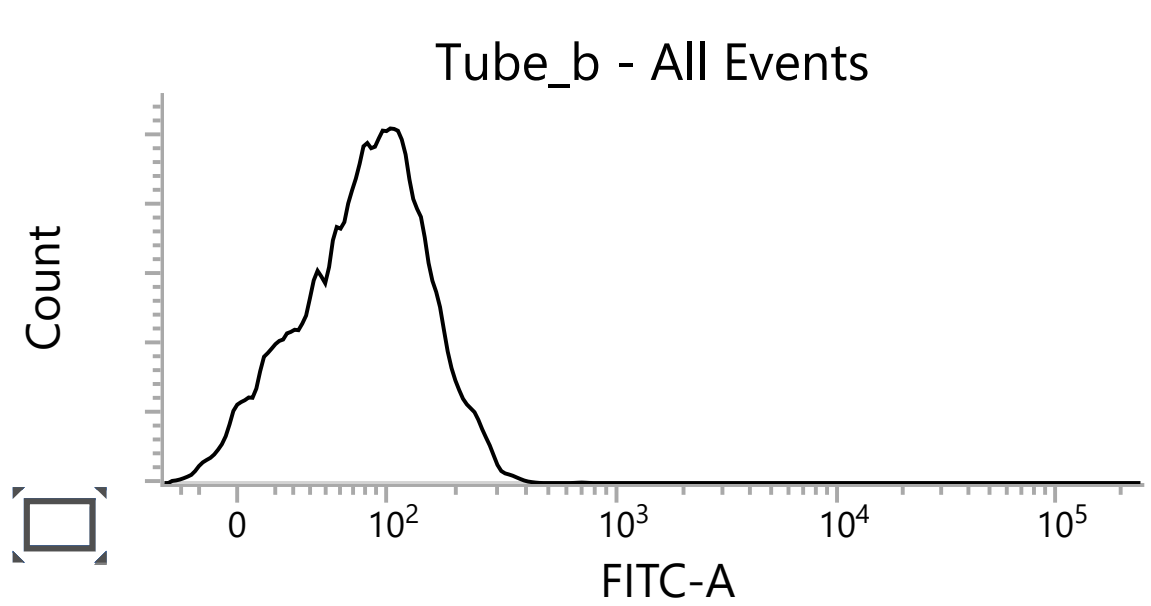

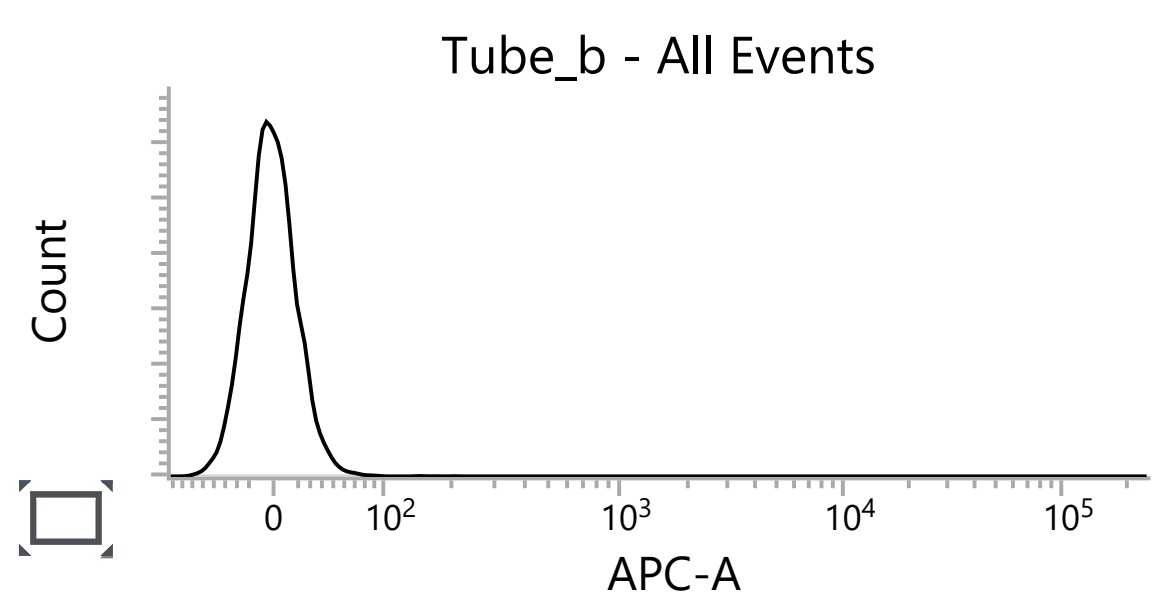

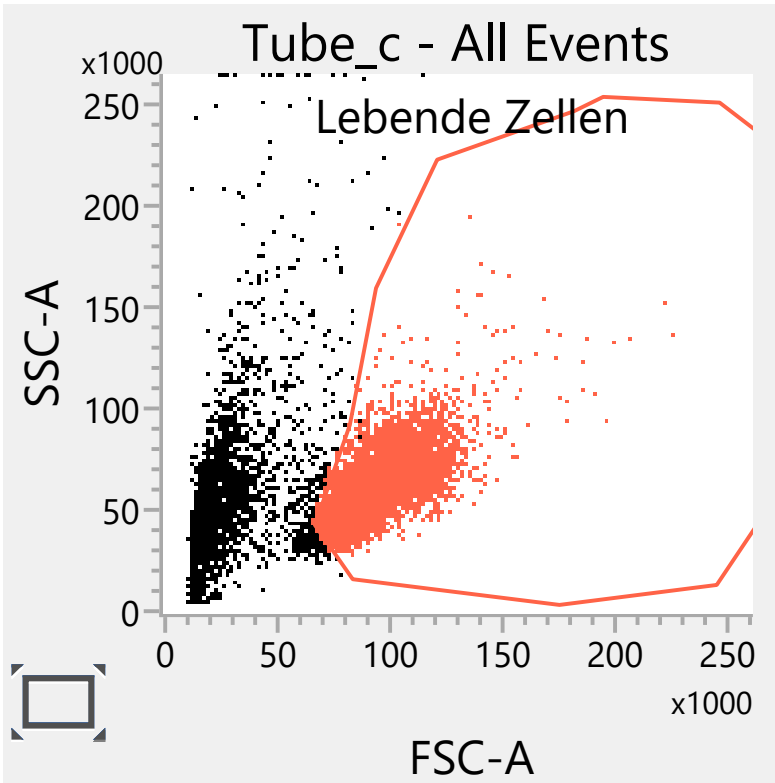

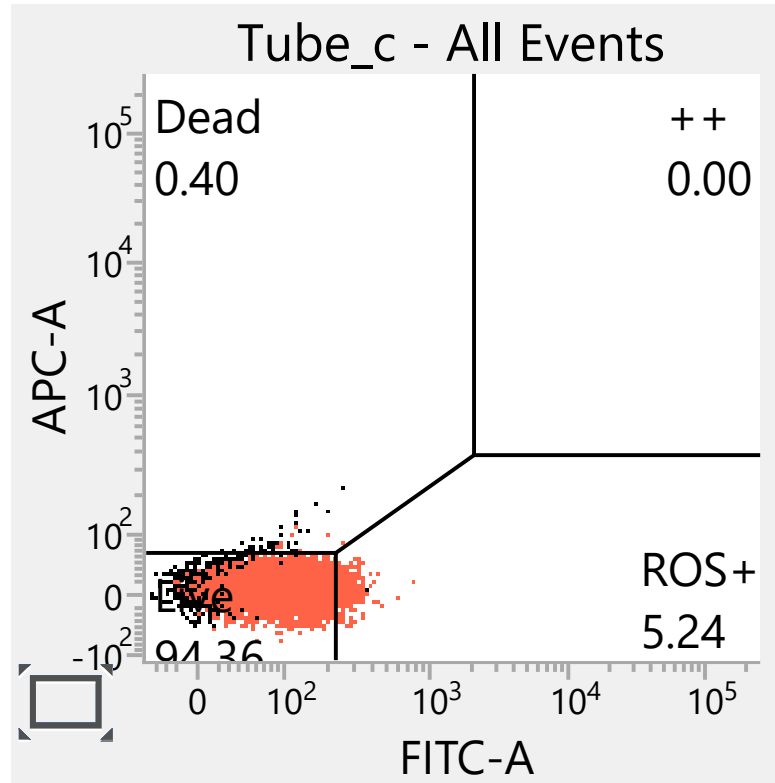

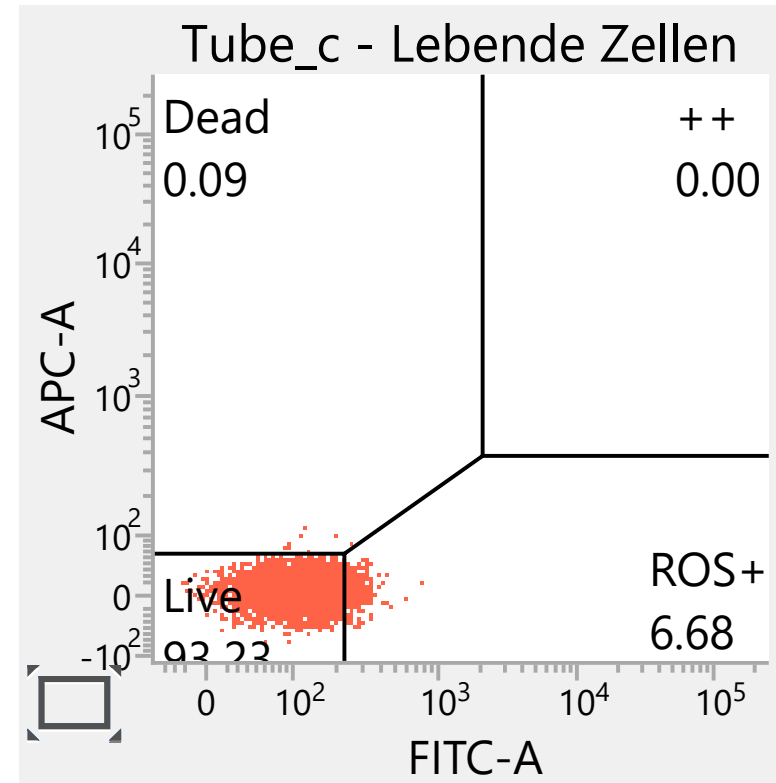

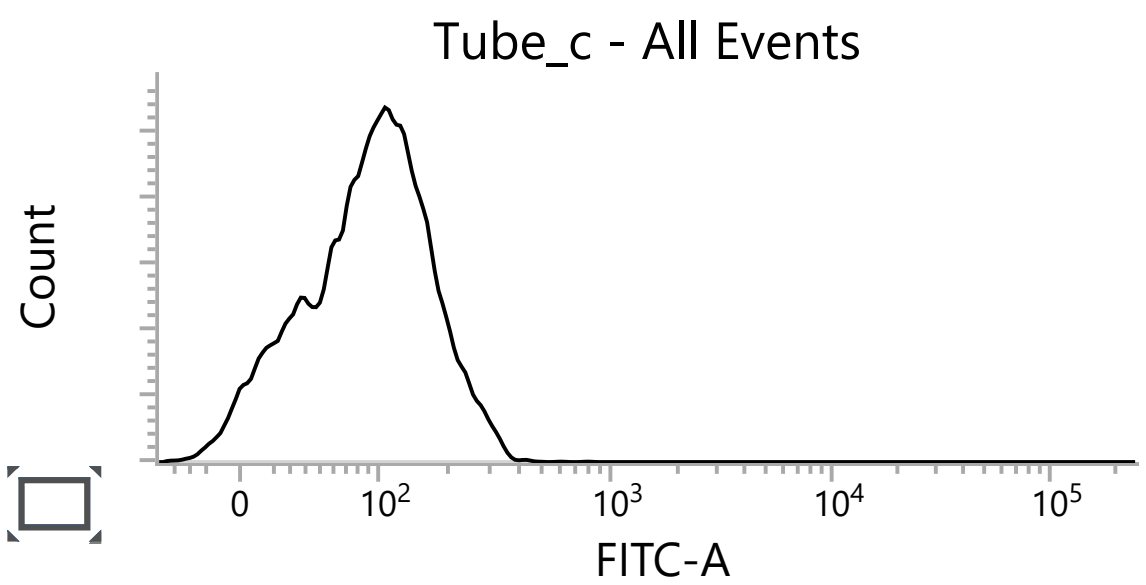

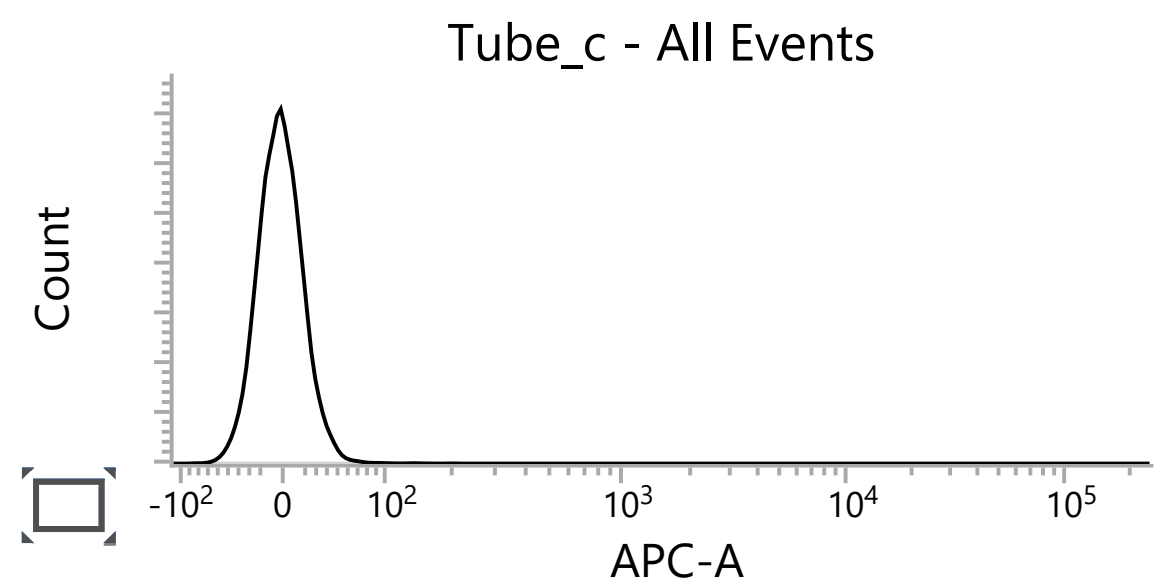

Statistics

Name	Events	% Parent	% Grandparent	% Total
Tube_a:All Events	10,000	***	***	100.00
Tube_a:Lebende Zellen	7,797	77.97	***	77.97
Tube_b:All Events	10,000	***	***	100.00
Tube_b:Lebende Zellen	7,597	75.97	***	75.97
Tube_c:All Events	10,000	***	***	100.00
Tube_c:Lebende Zellen	7,768	77.68	***	77.68

CellROX Assay

Sample ID: 2,5 ATM + NAC

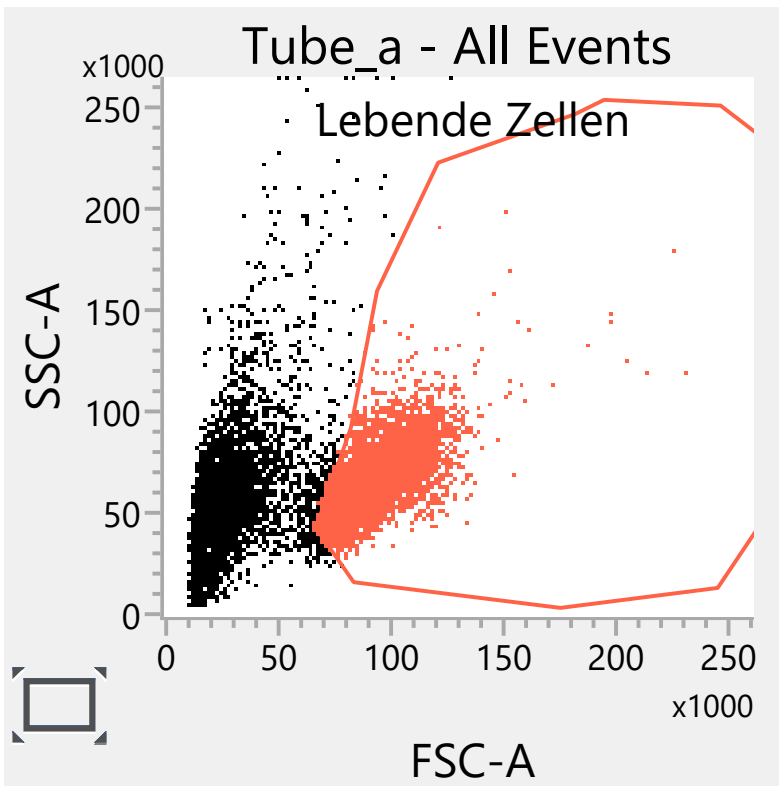

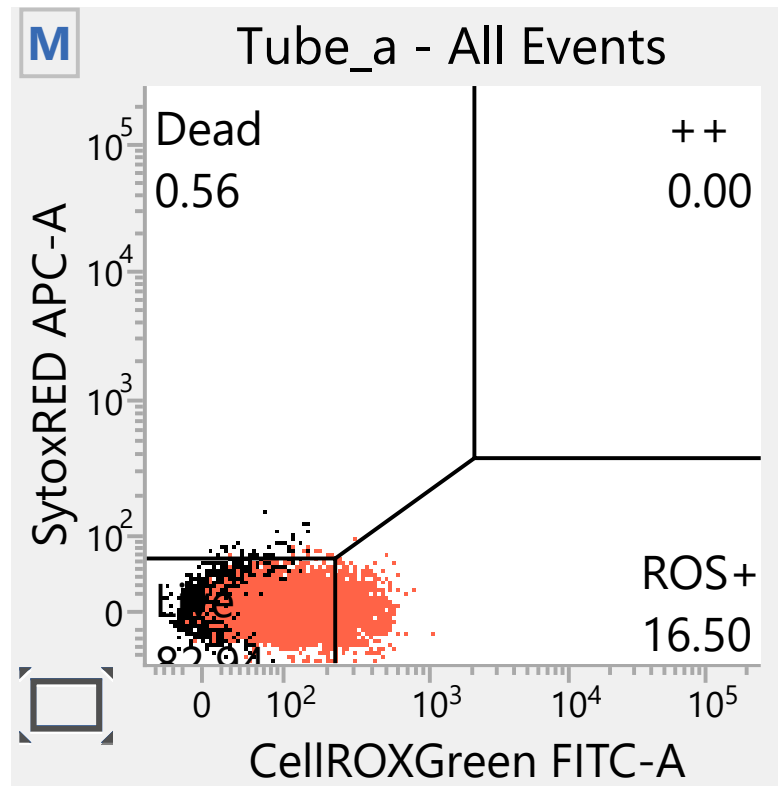

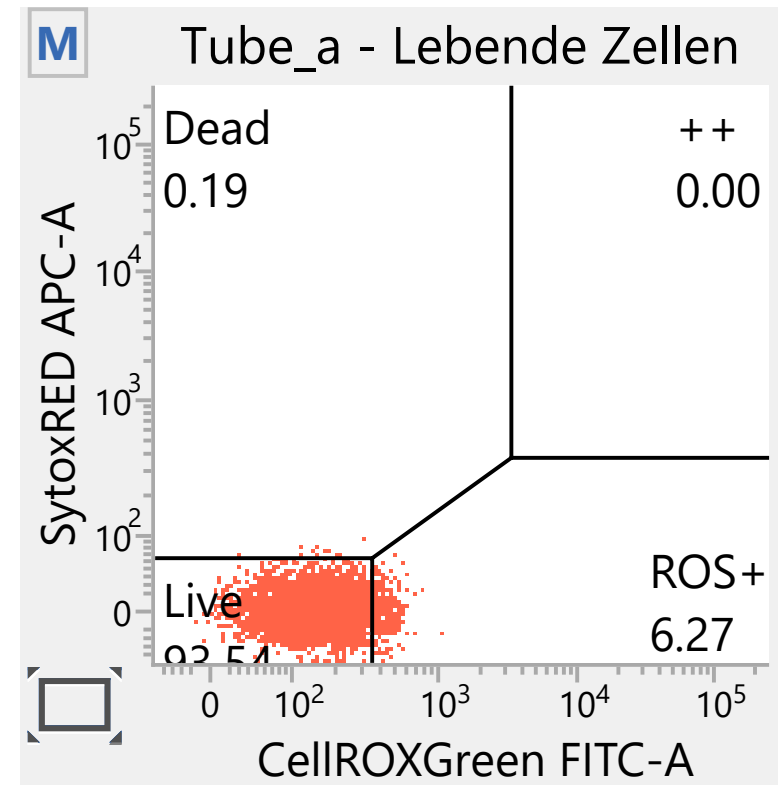

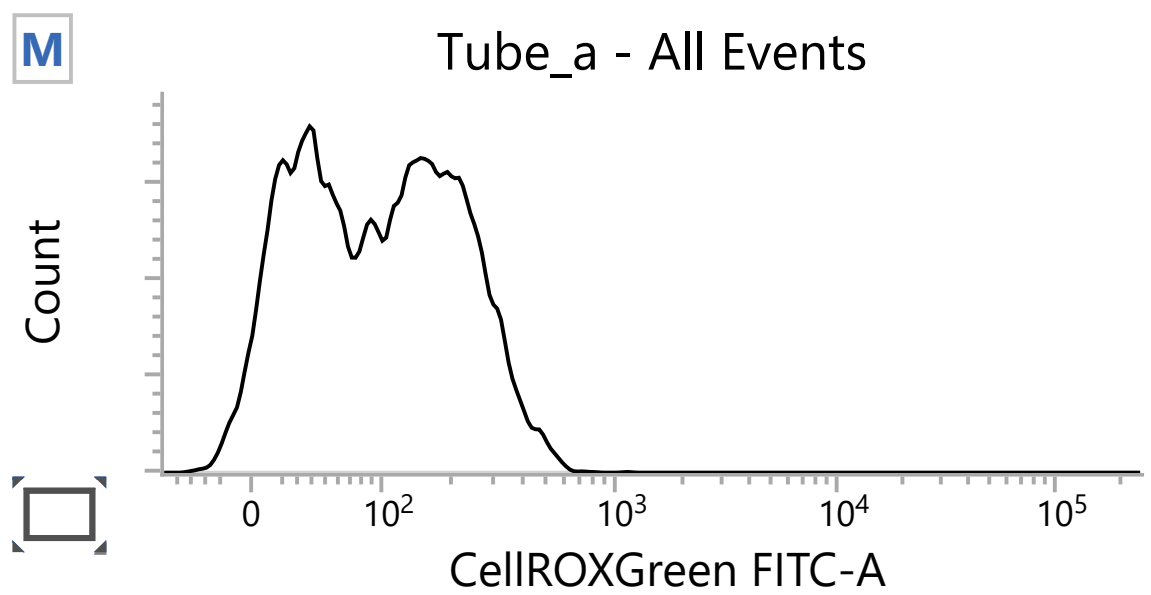

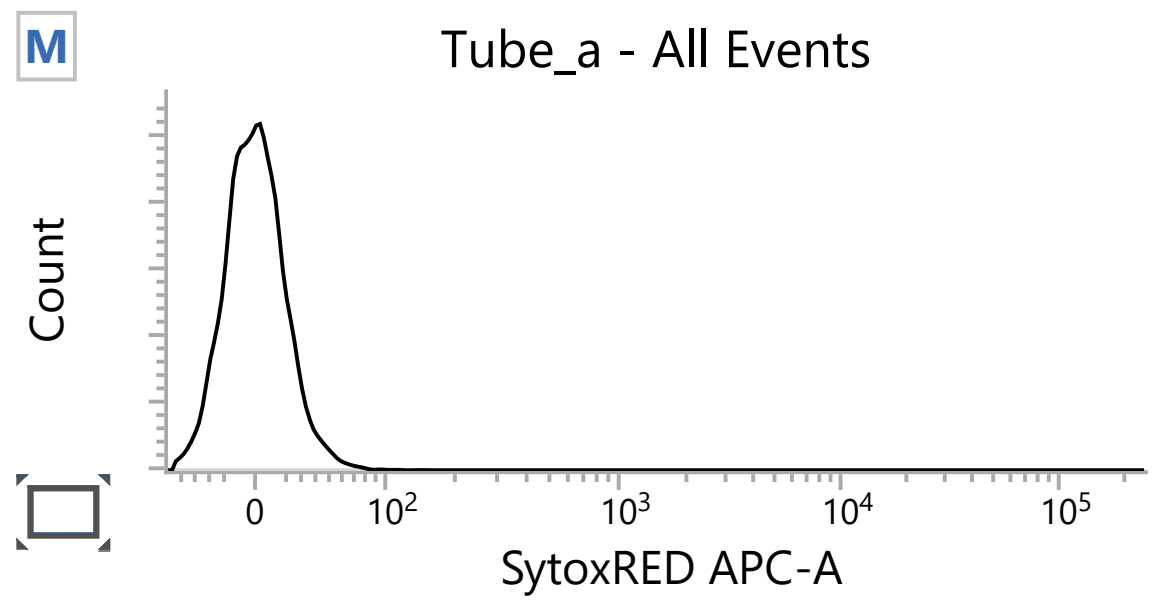

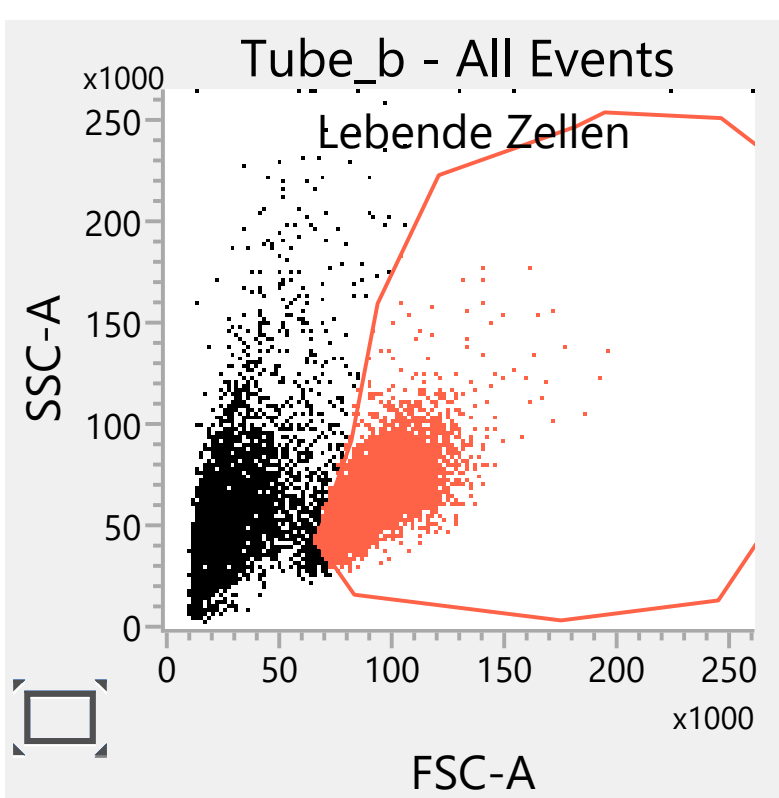

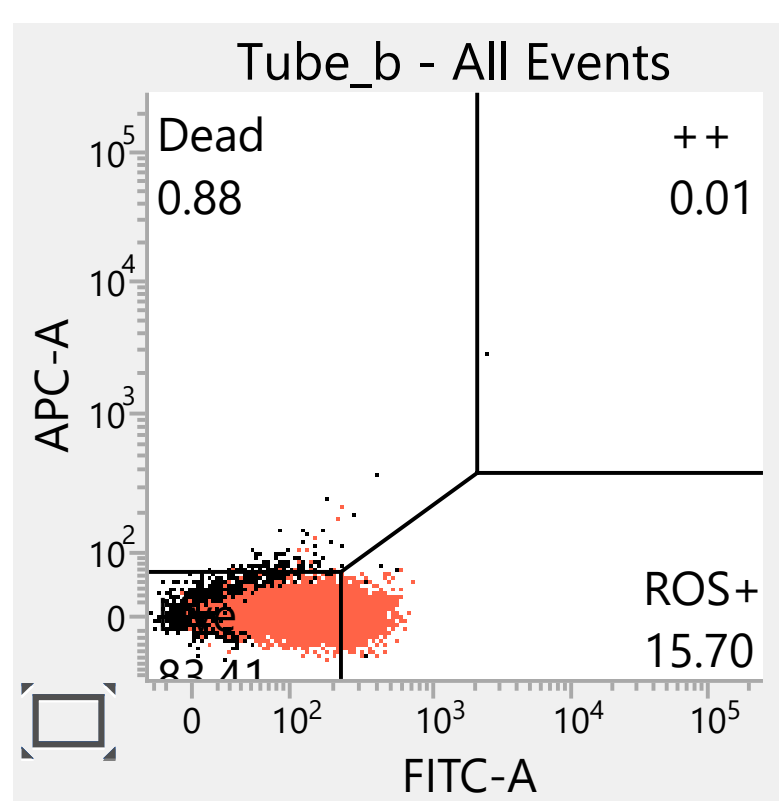

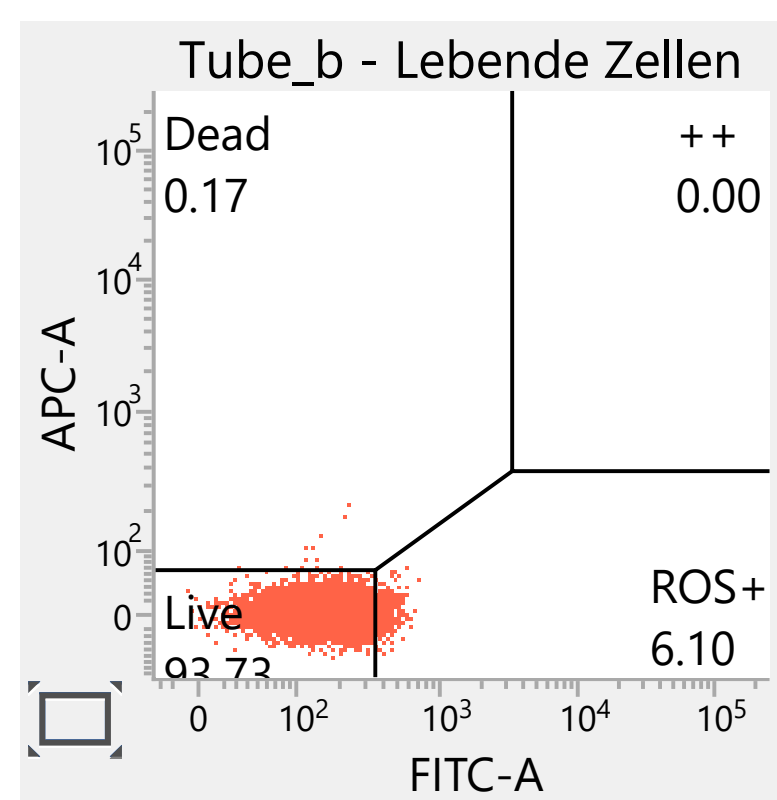

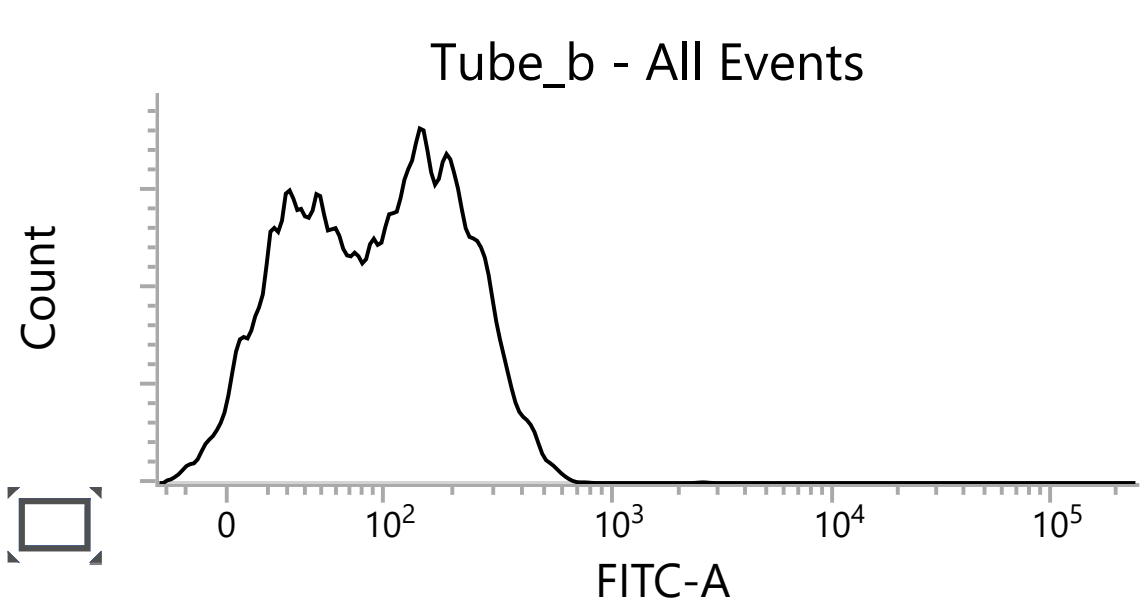

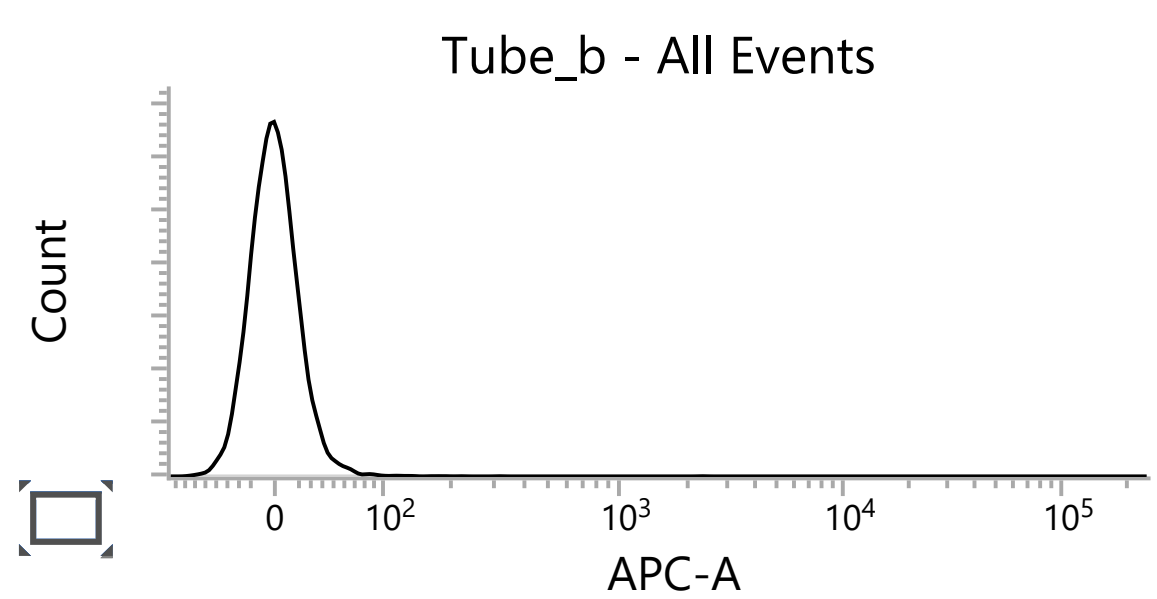

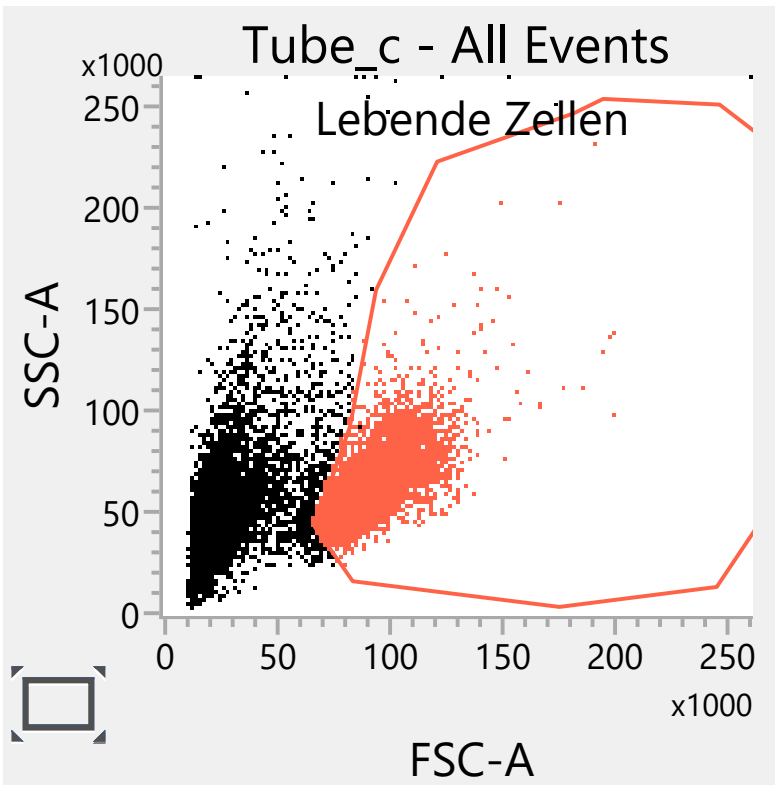

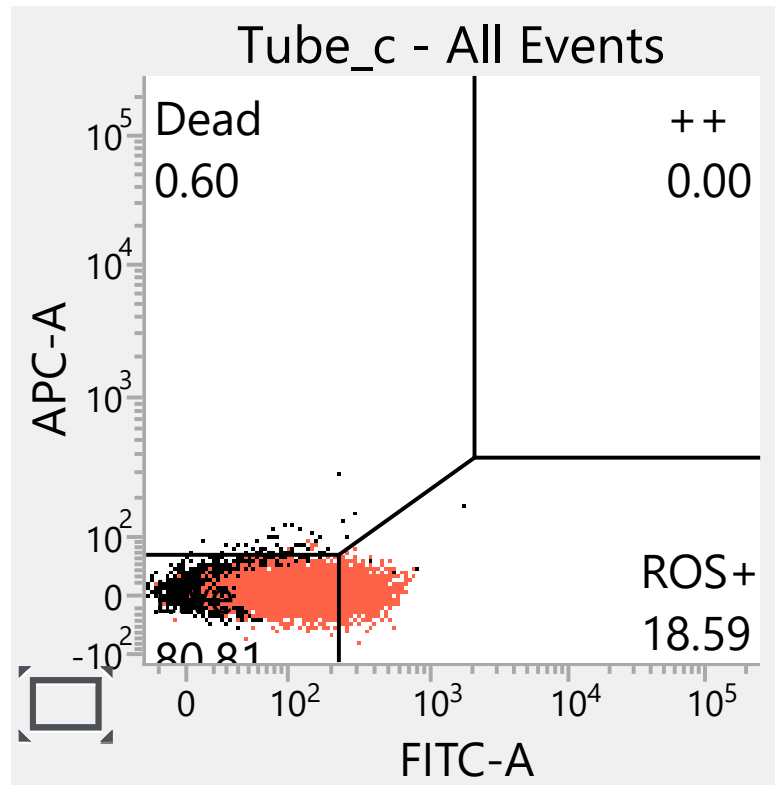

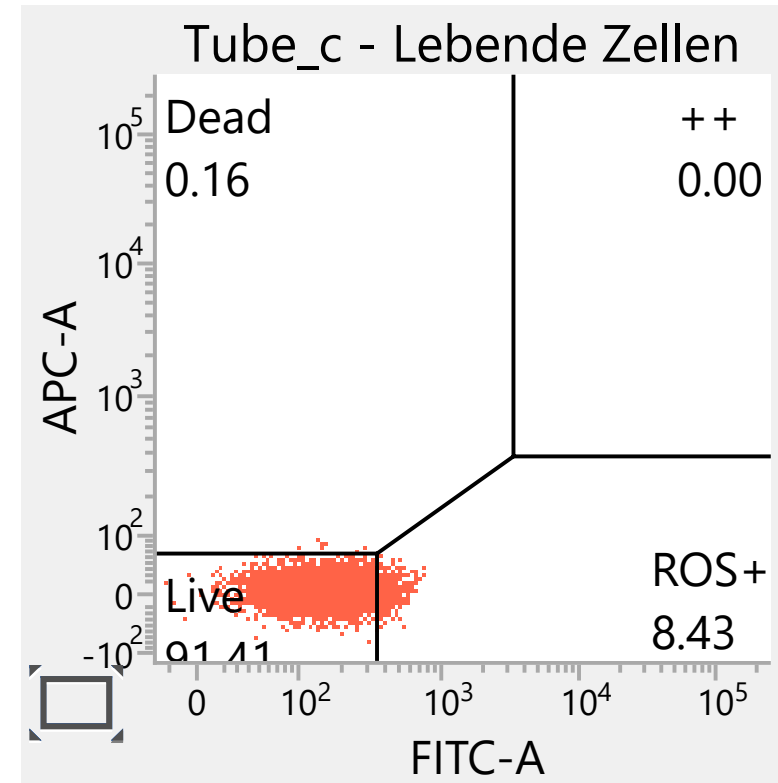

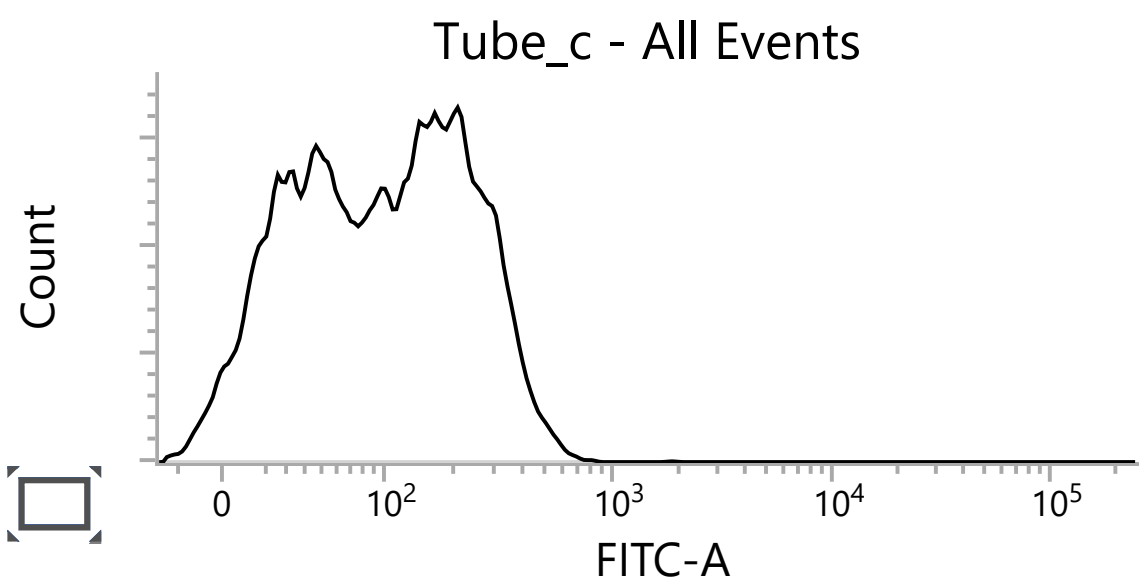

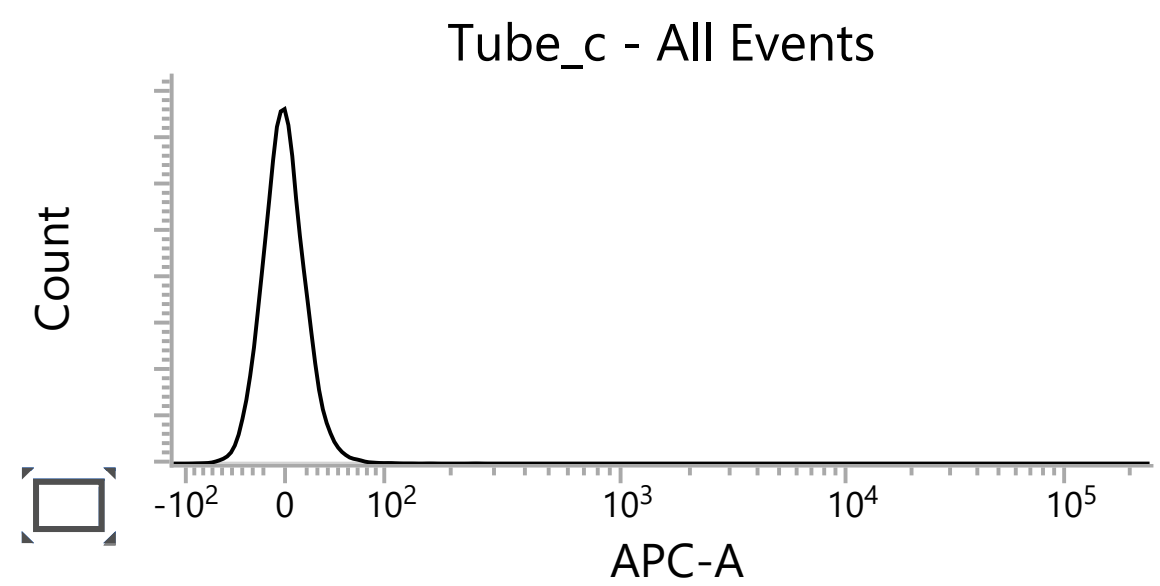

Statistics

Name	Events	% Parent	% Grandparent	% Total
Tube_a:All Events	10,000	***	***	100.00
Tube_a:Lebende Zellen	5,818	58.18	***	58.18
Tube_b:All Events	10,000	***	***	100.00
Tube_b:Lebende Zellen	5,757	57.57	***	57.57
Tube_c:All Events	10,000	***	***	100.00
Tube_c:Lebende Zellen	5,624	56.24	***	56.24

CellROX Assay

Sample ID: 2,5 ATM

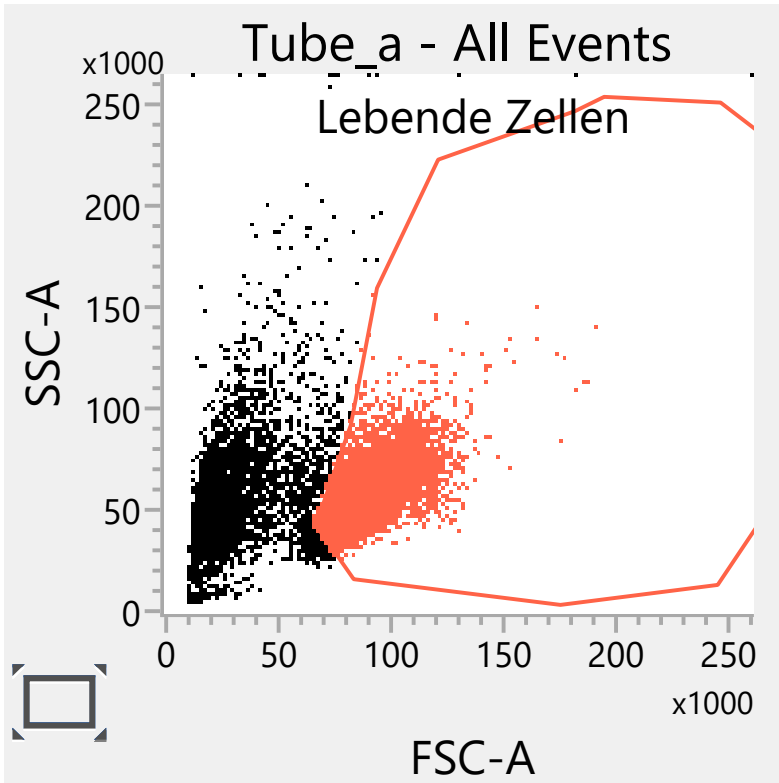

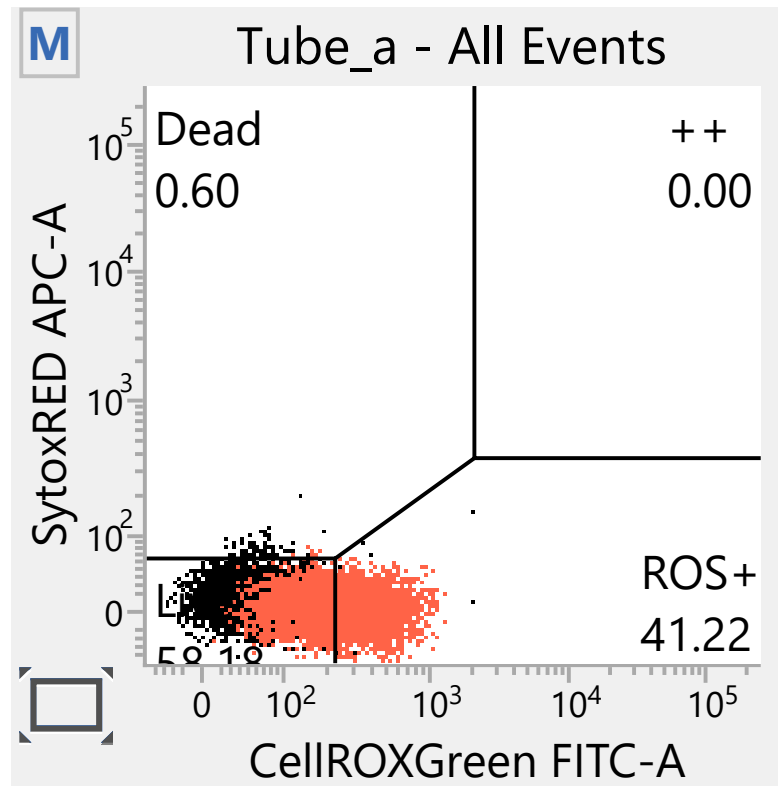

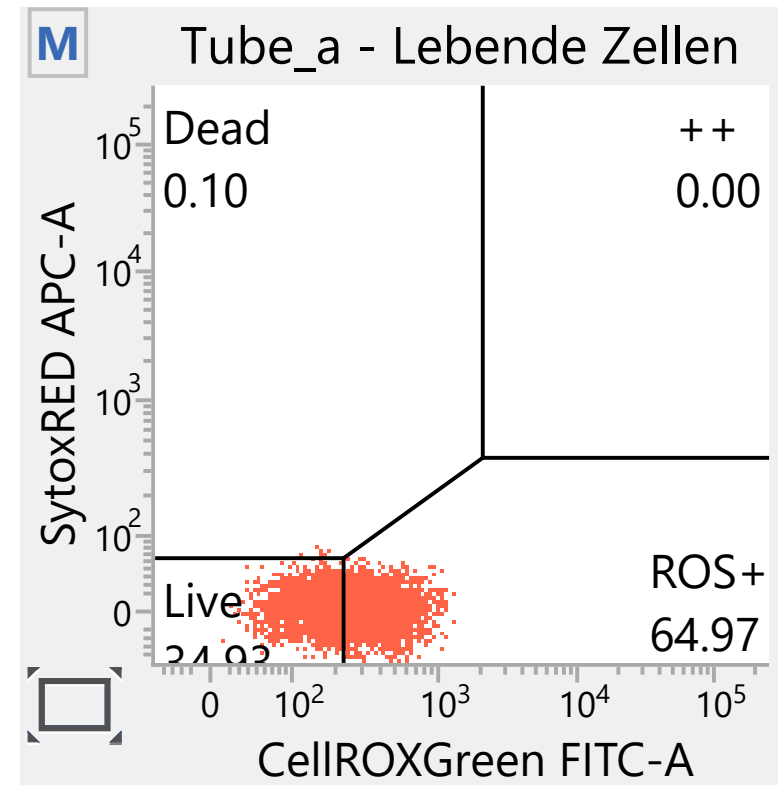

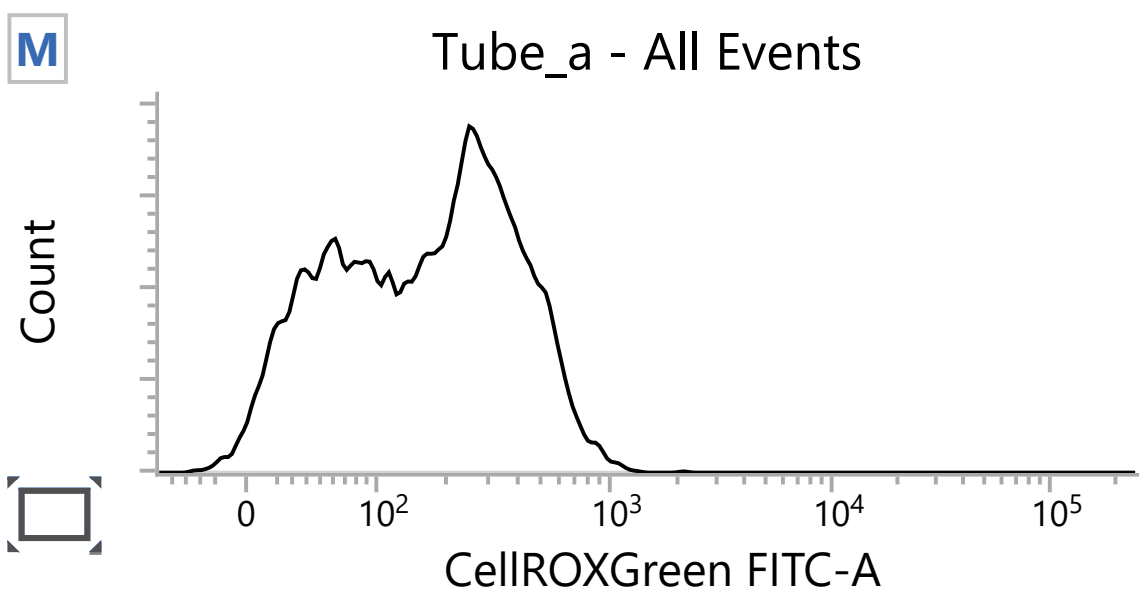

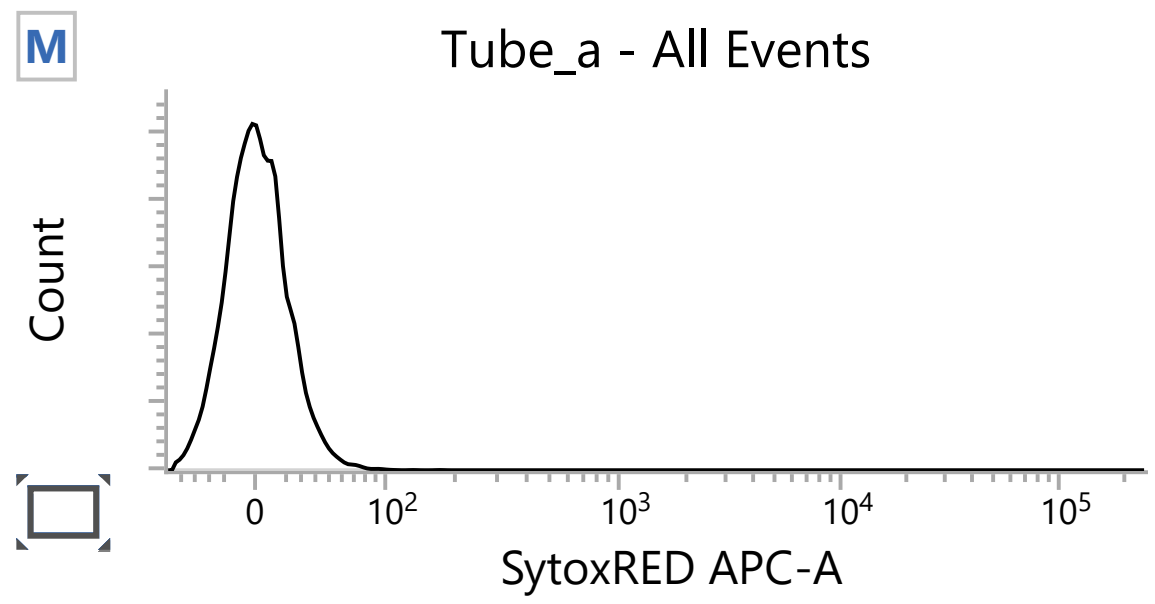

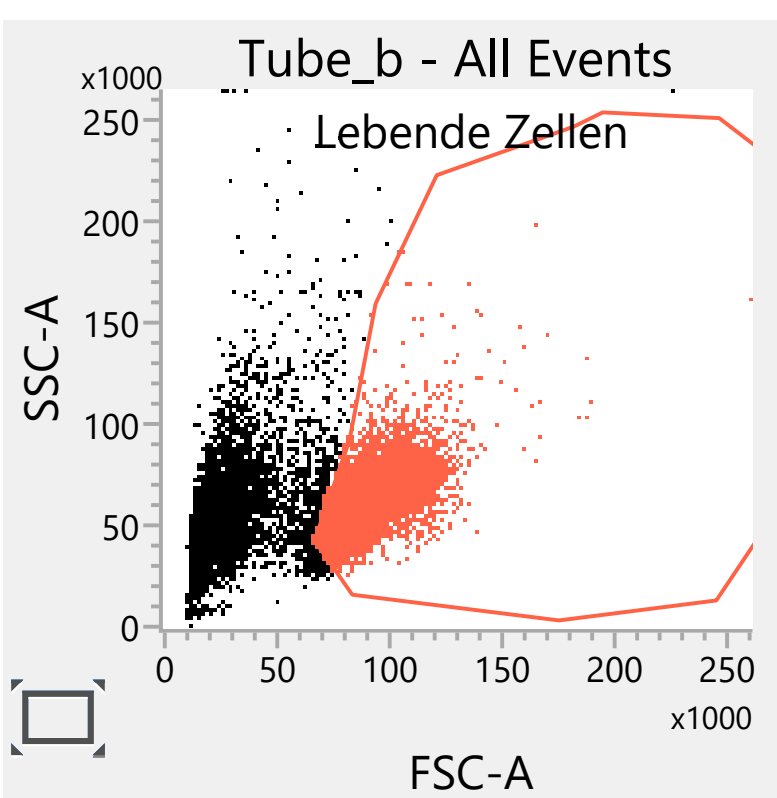

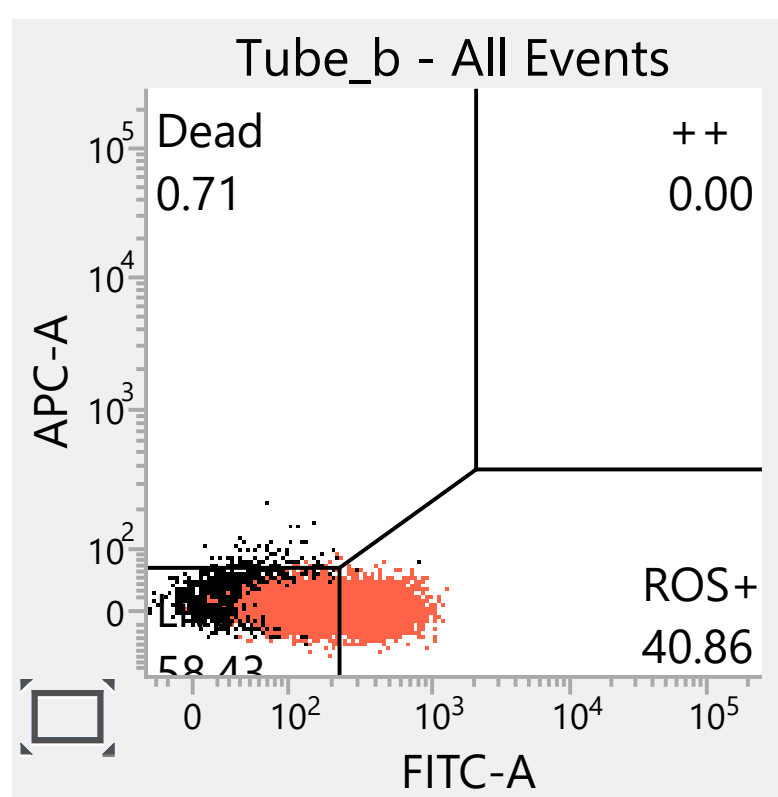

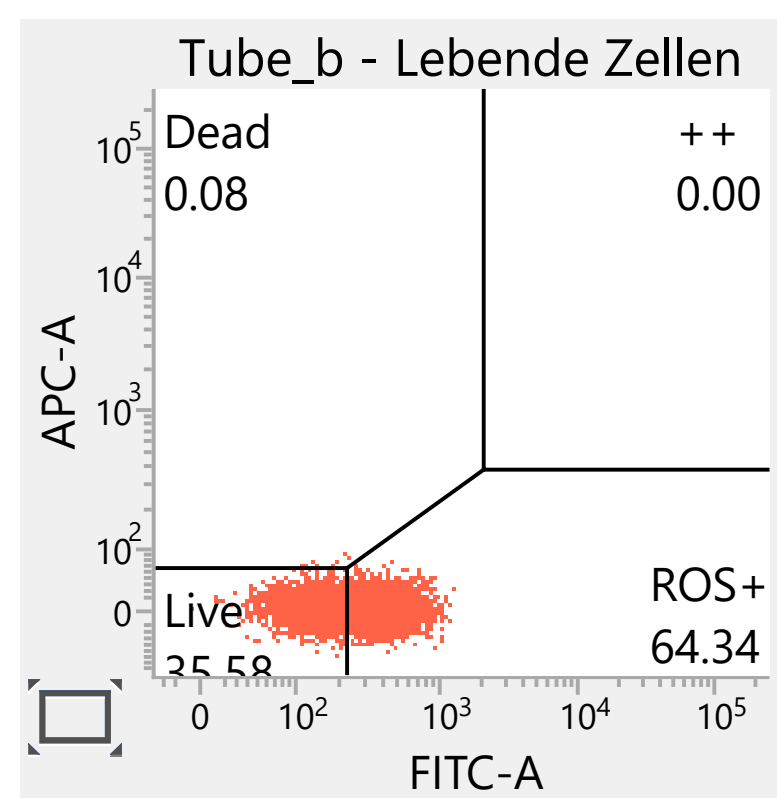

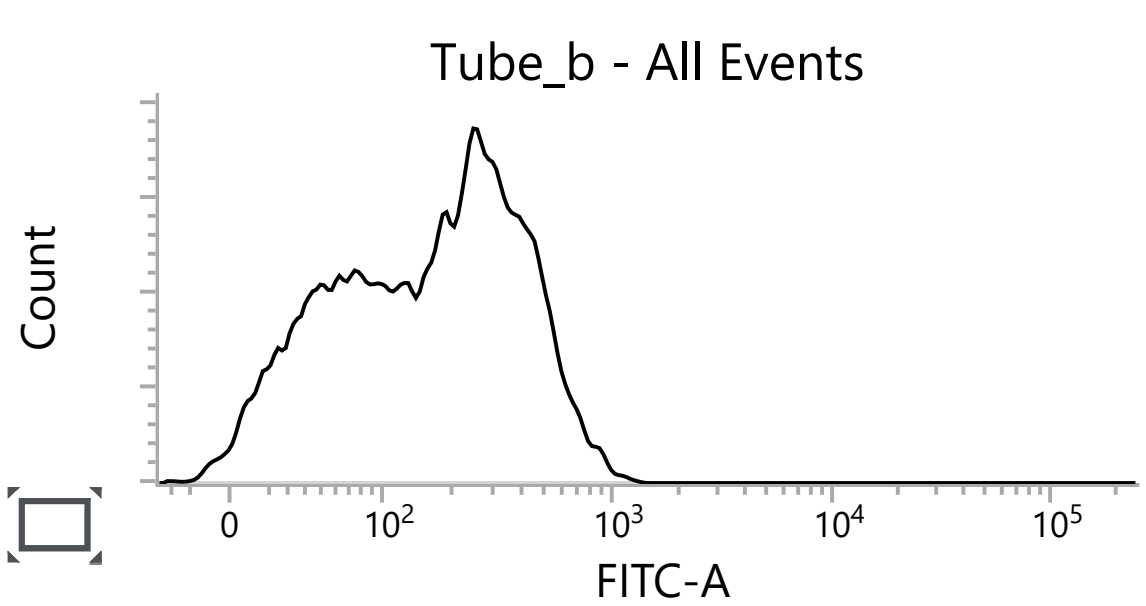

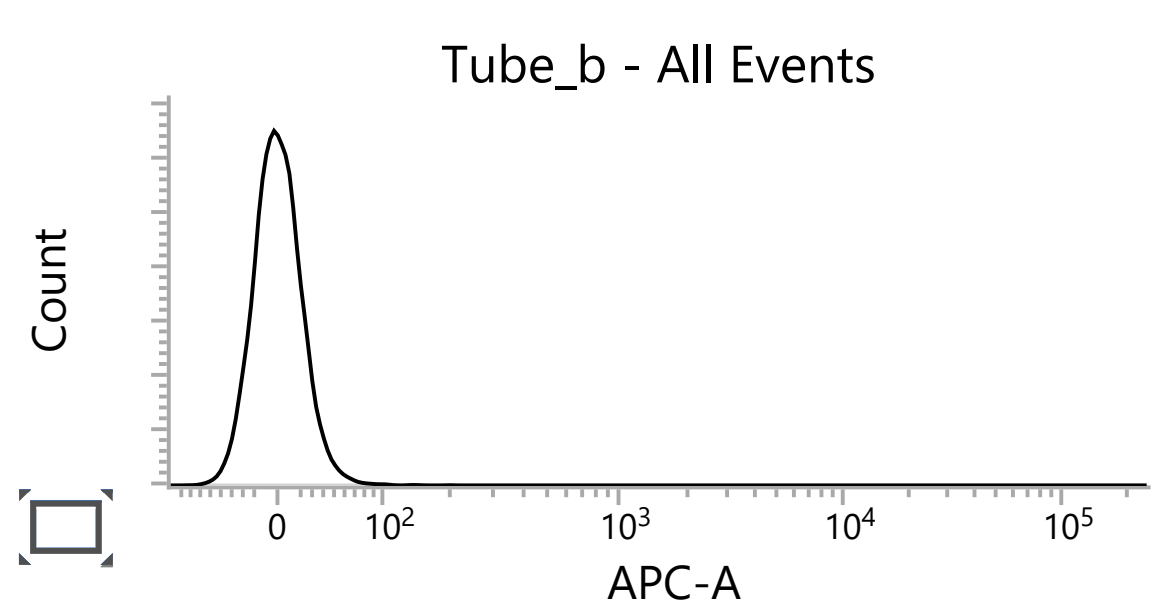

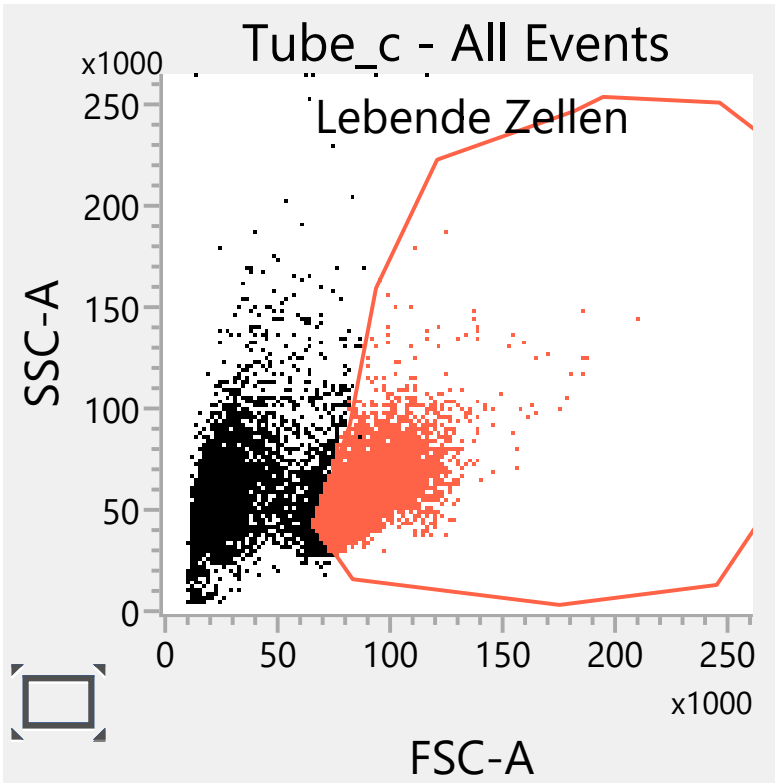

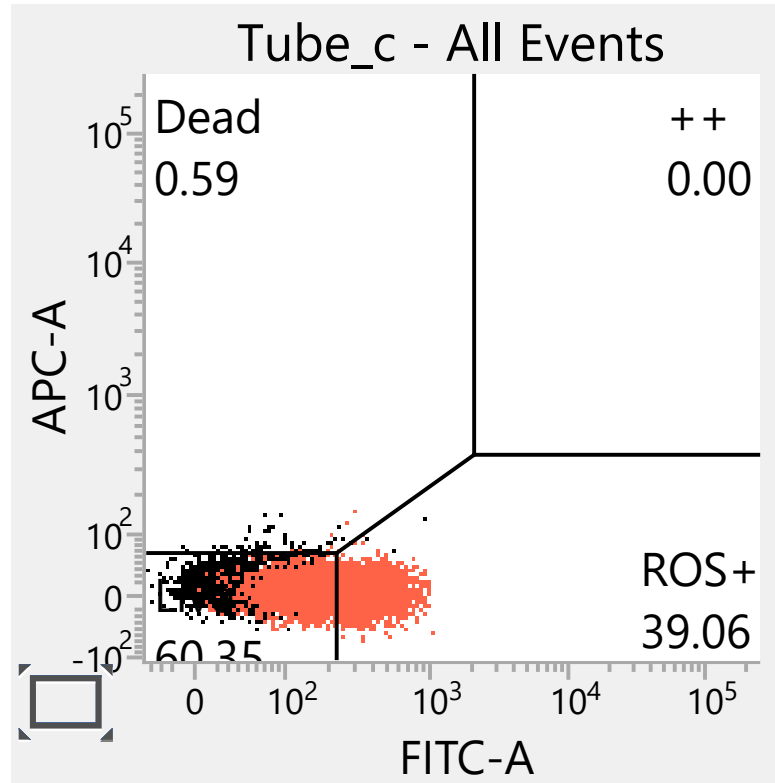

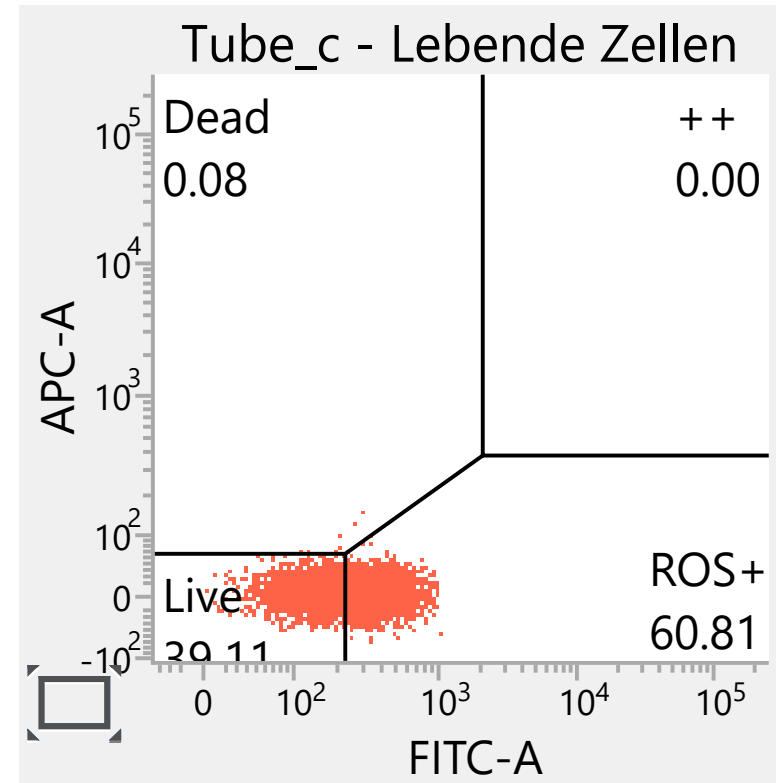

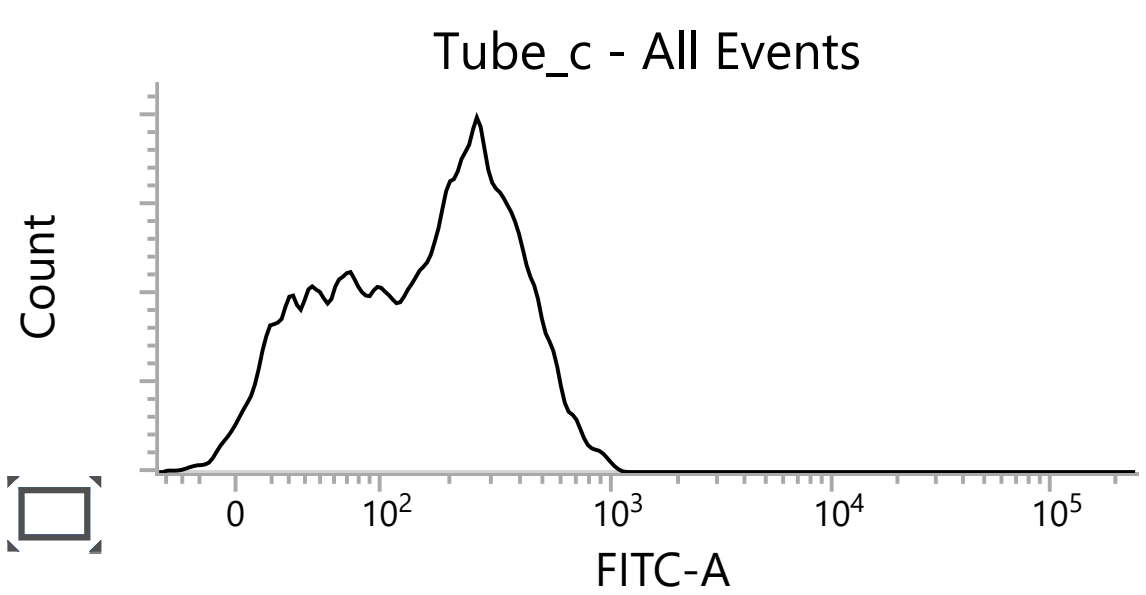

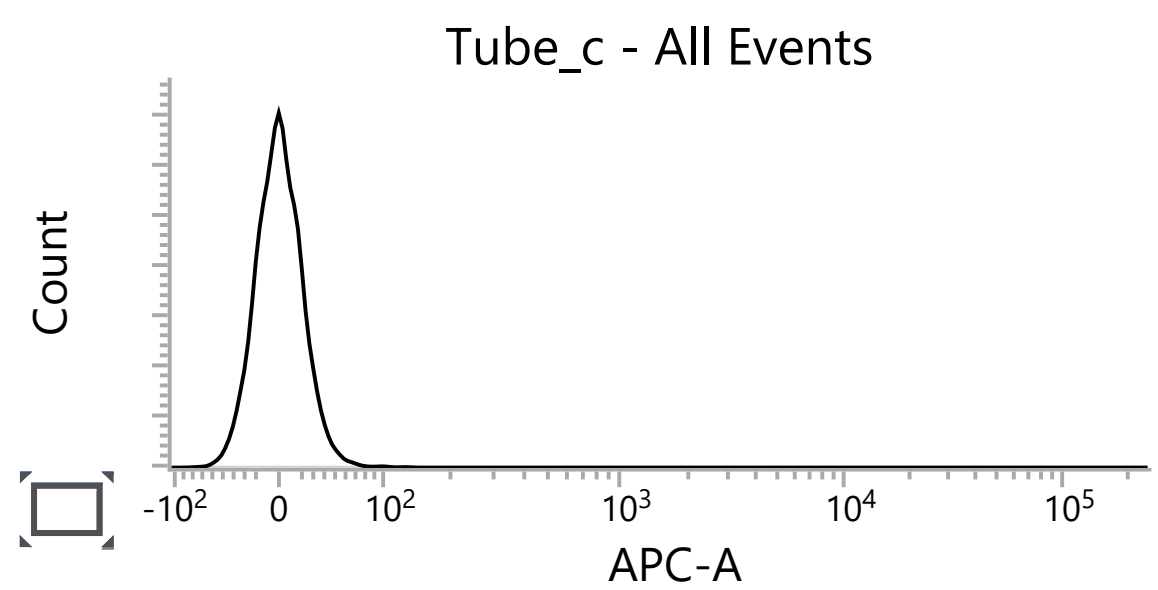

Statistics

Name	Events	% Parent	% Grandparent	% Total
Tube_a:All Events	10,000	***	***	100.00
Tube_a:Lebende Zellen	6,081	60.81	***	60.81
Tube_b:All Events	10,000	***	***	100.00
Tube_b:Lebende Zellen	6,085	60.85	***	60.85
Tube_c:All Events	10,000	***	***	100.00
Tube_c:Lebende Zellen	6,236	62.36	***	62.36

CellROX Assay

Sample ID: 5 ATM +NAC

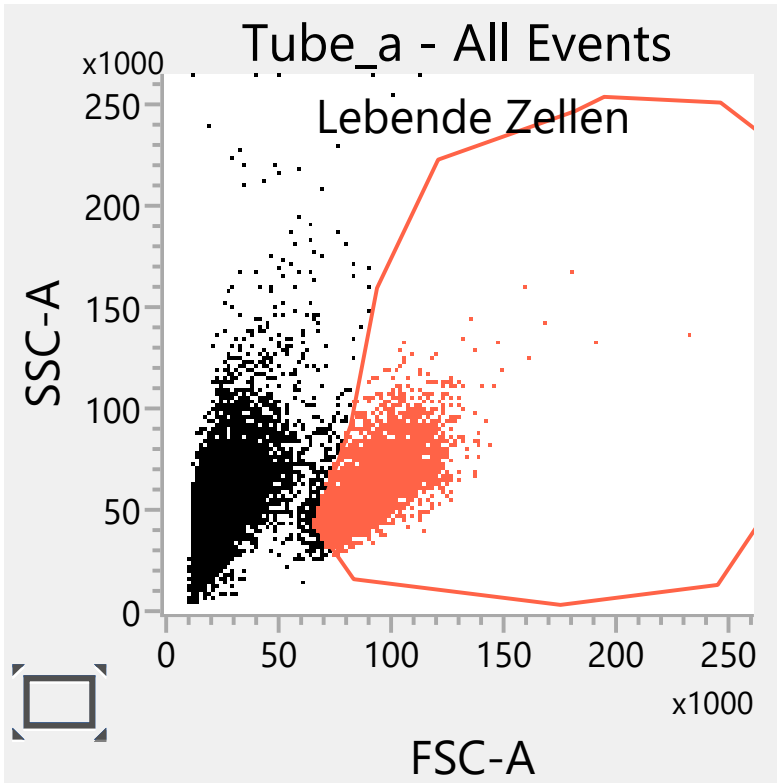

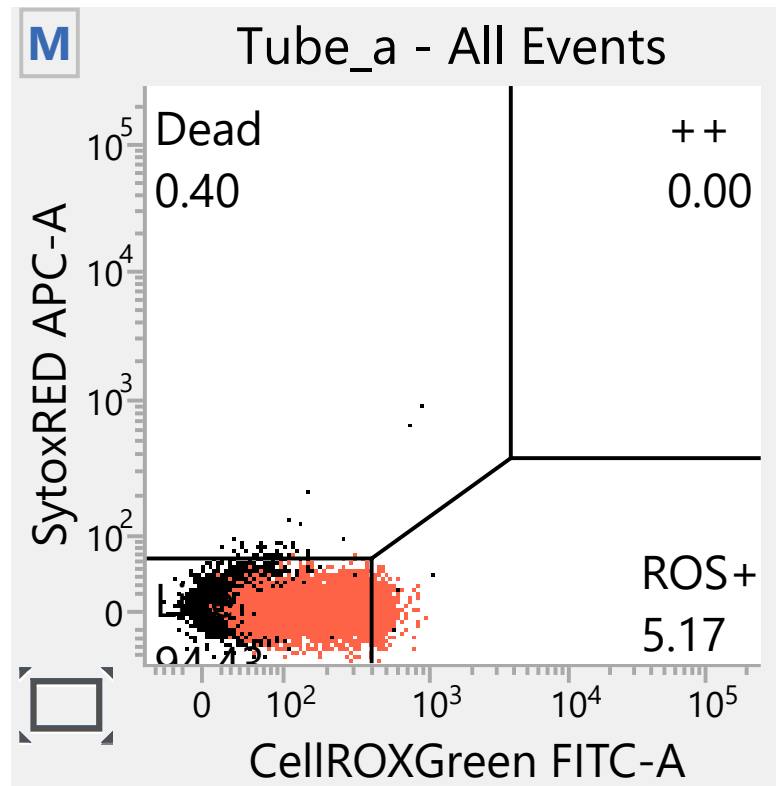

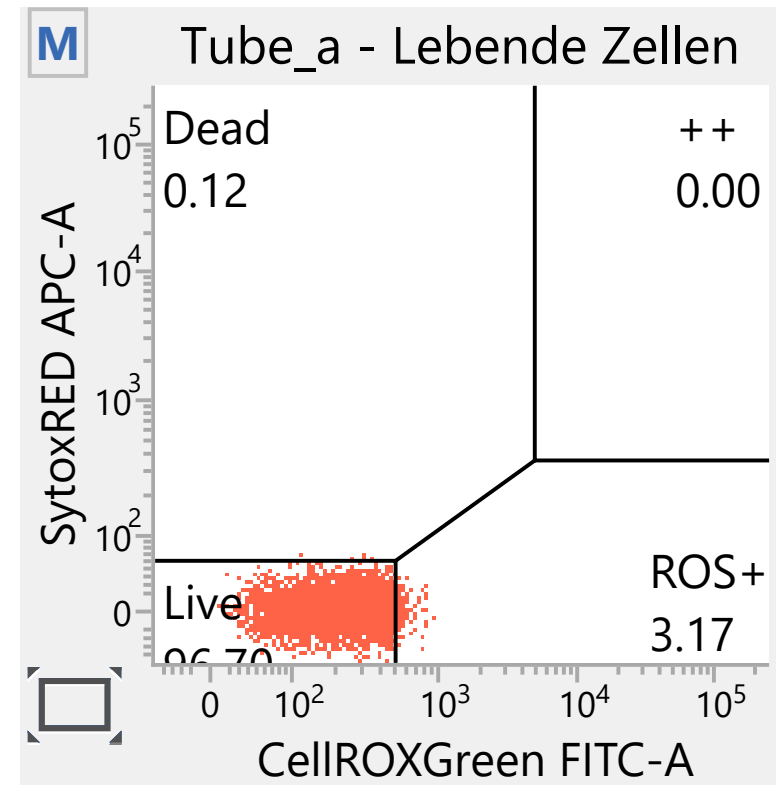

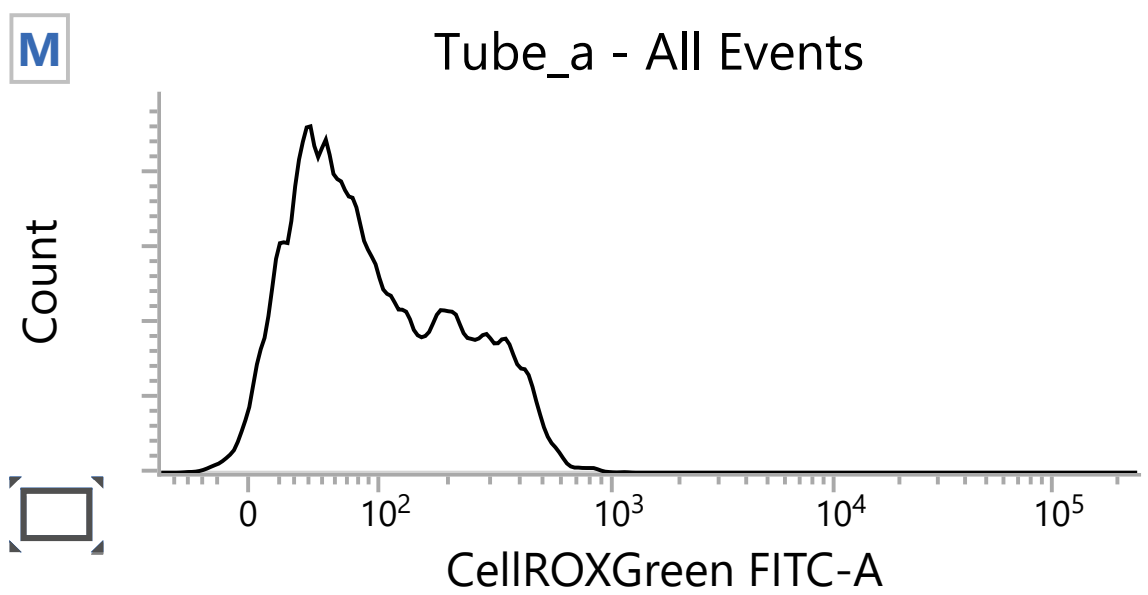

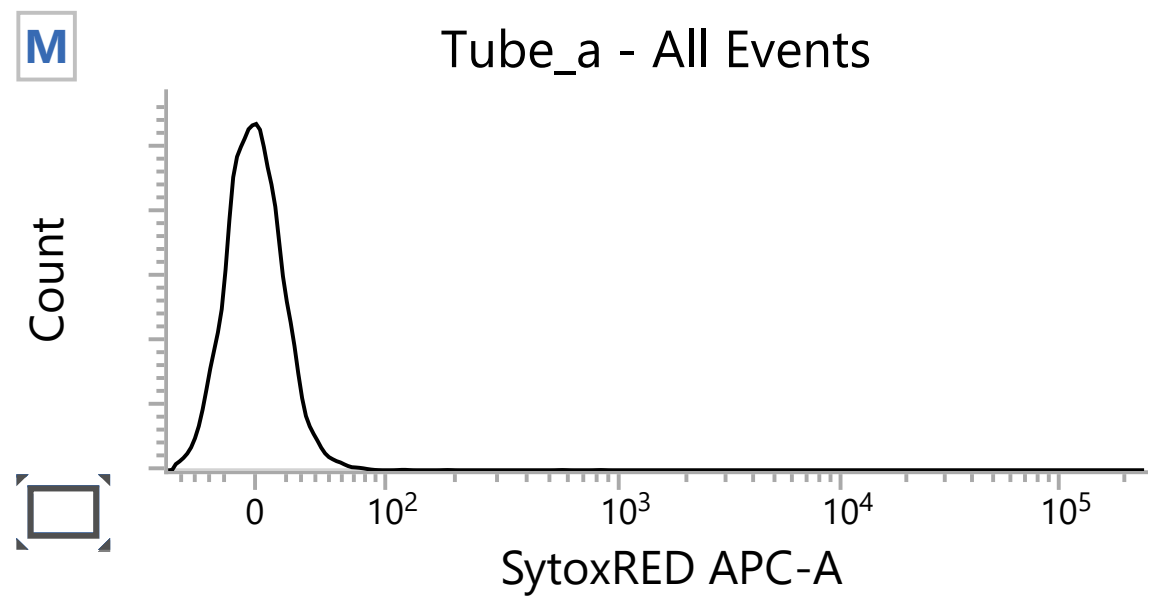

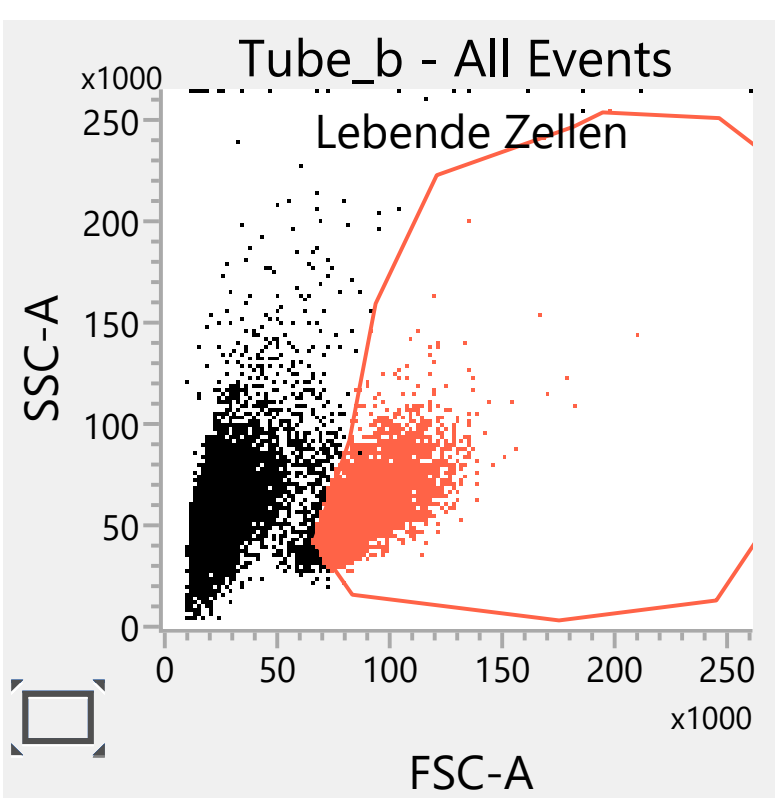

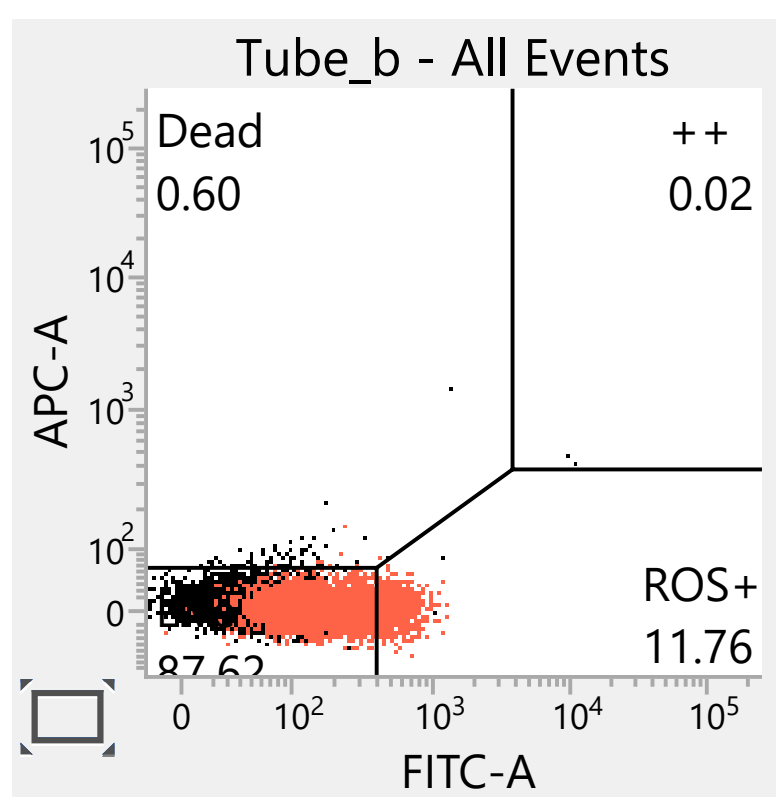

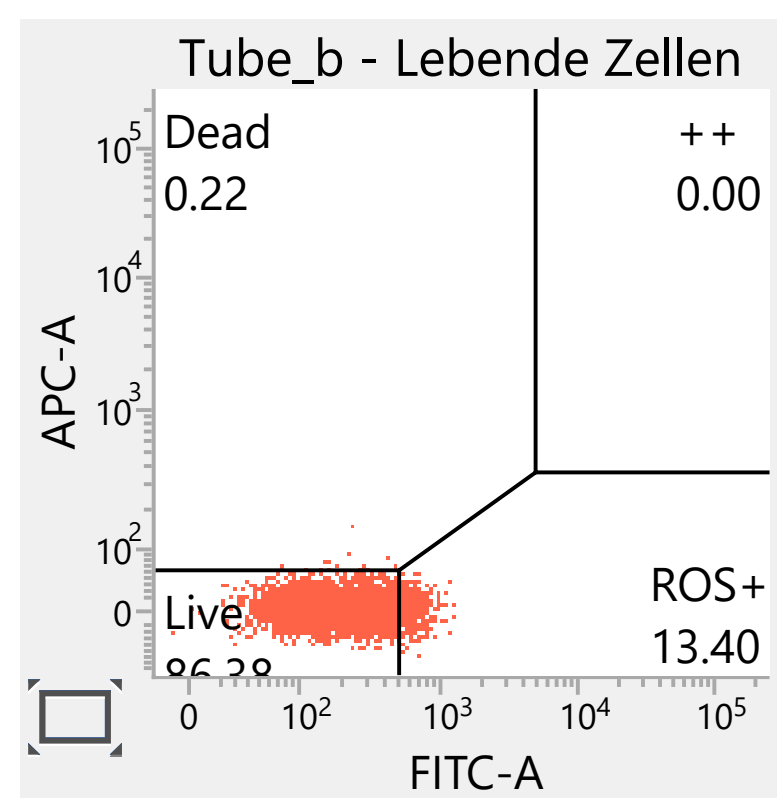

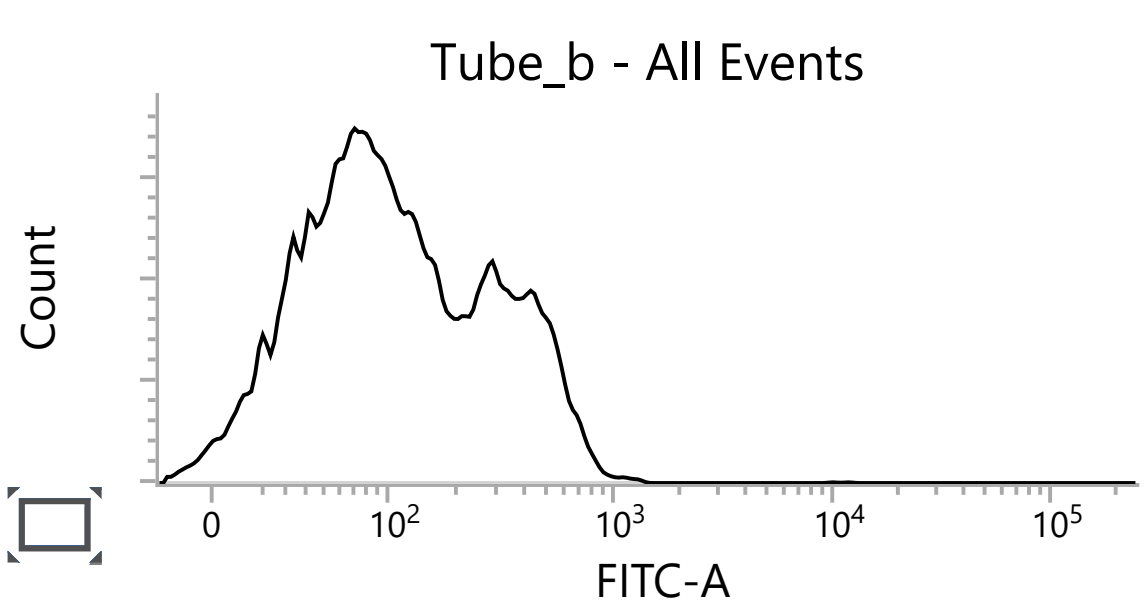

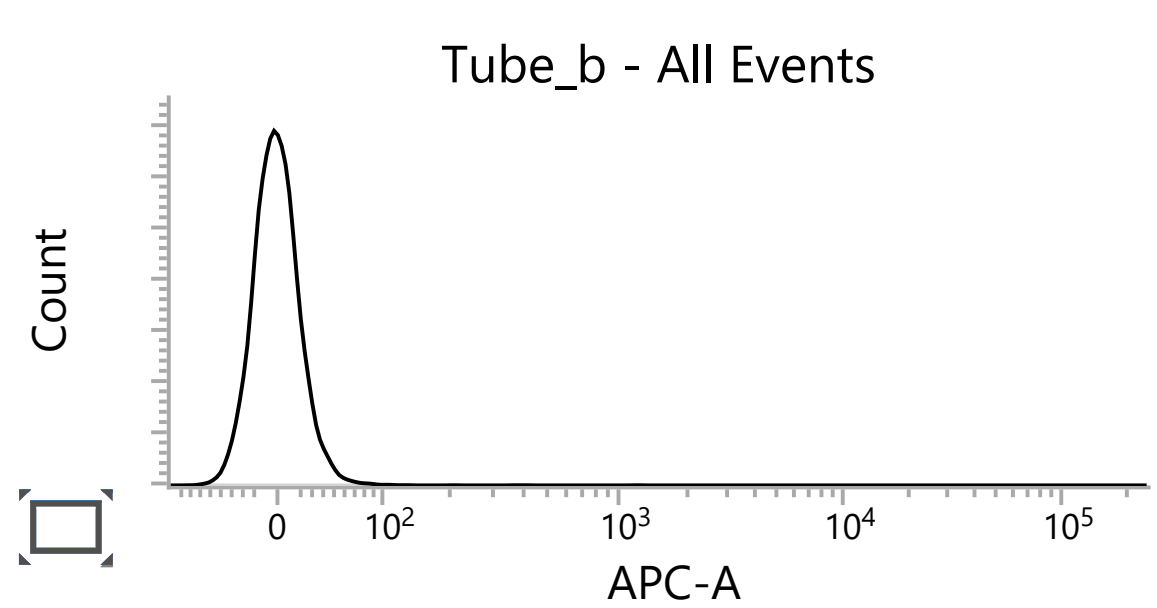

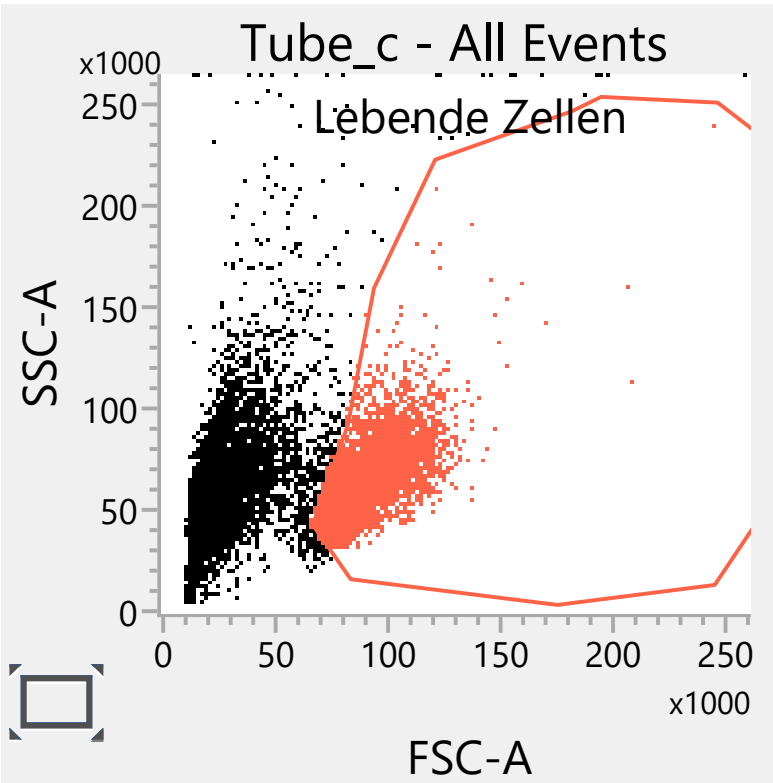

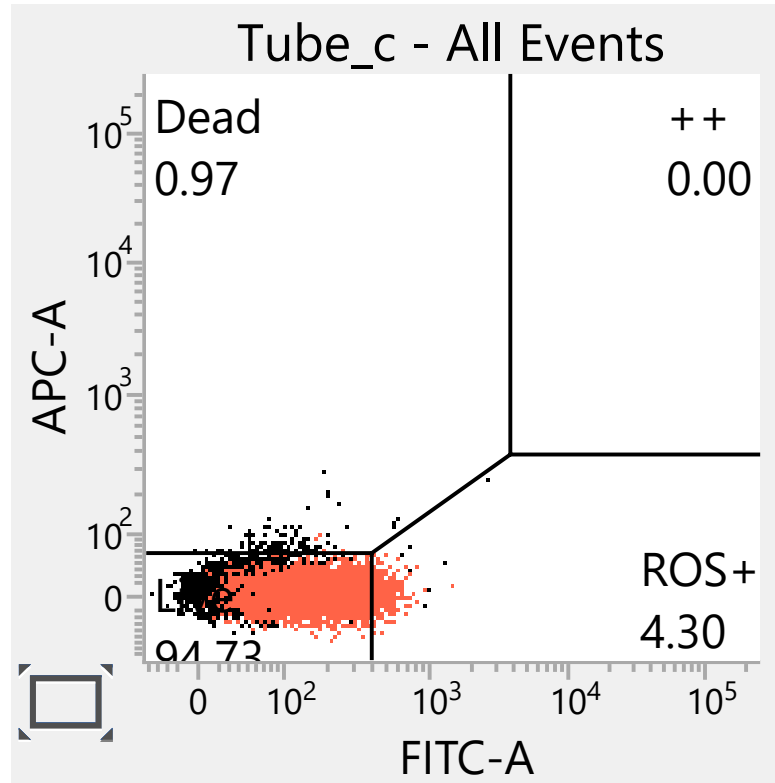

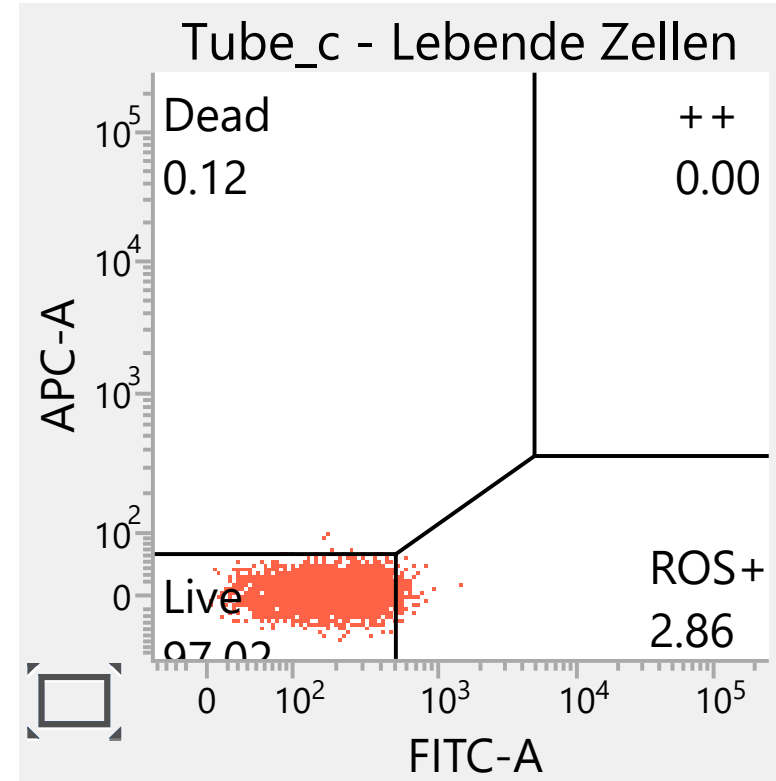

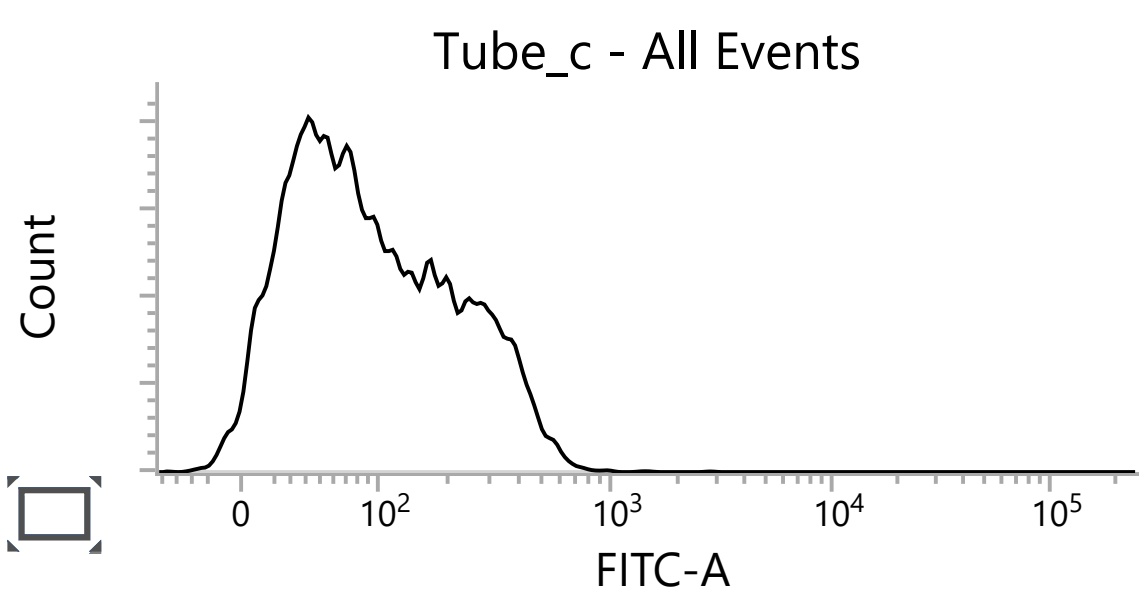

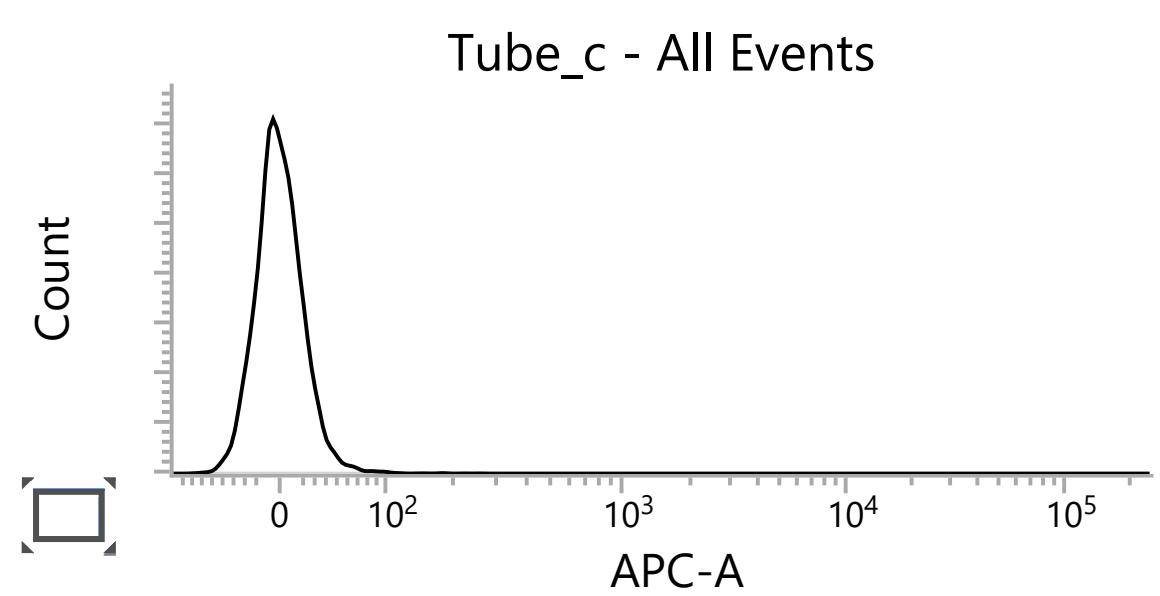

Statistics

Name	Events	% Parent	% Grandparent	% Total
Tube_a:All Events	10,000	***	***	100.00
Tube_a:Lebende Zellen	4,066	40.66	***	40.66
Tube_b:All Events	10,000	***	***	100.00
Tube_b:Lebende Zellen	4,448	44.48	***	44.48
Tube_c:All Events	10,000	***	***	100.00
Tube_c:Lebende Zellen	4,233	42.33	***	42.33

CellROX Assay

Sample ID: 5 ATM

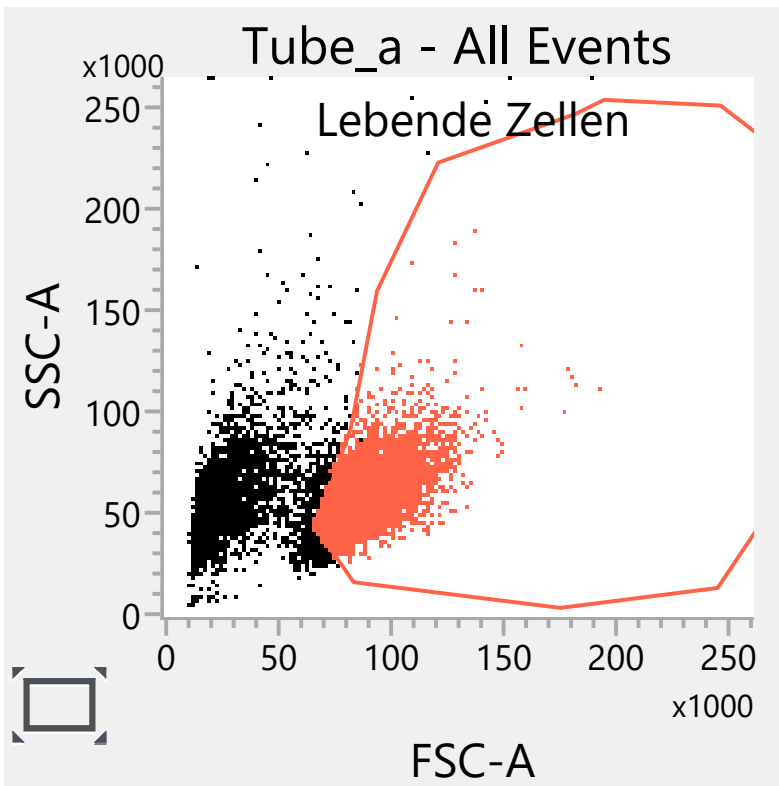

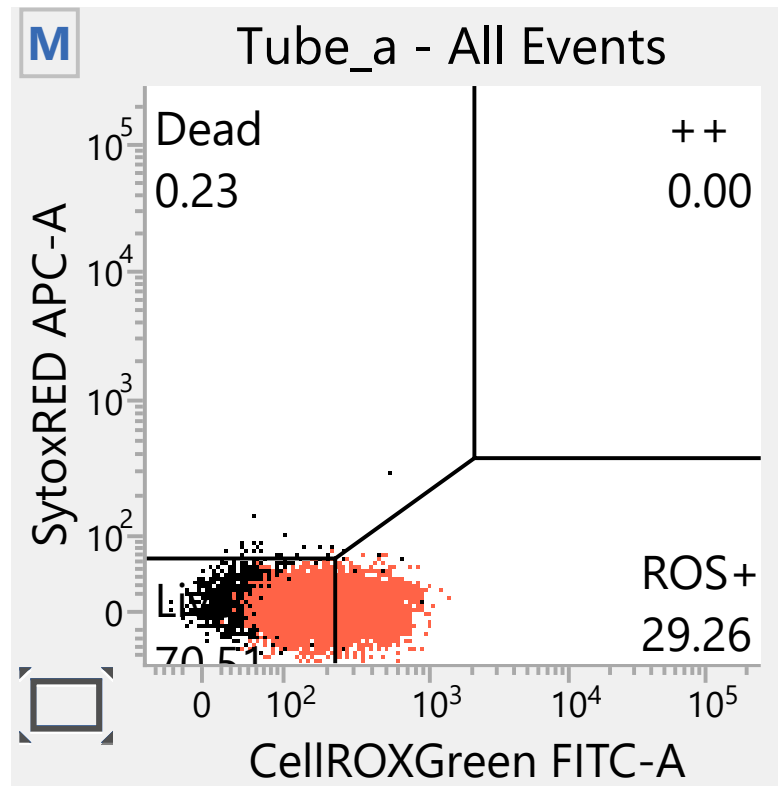

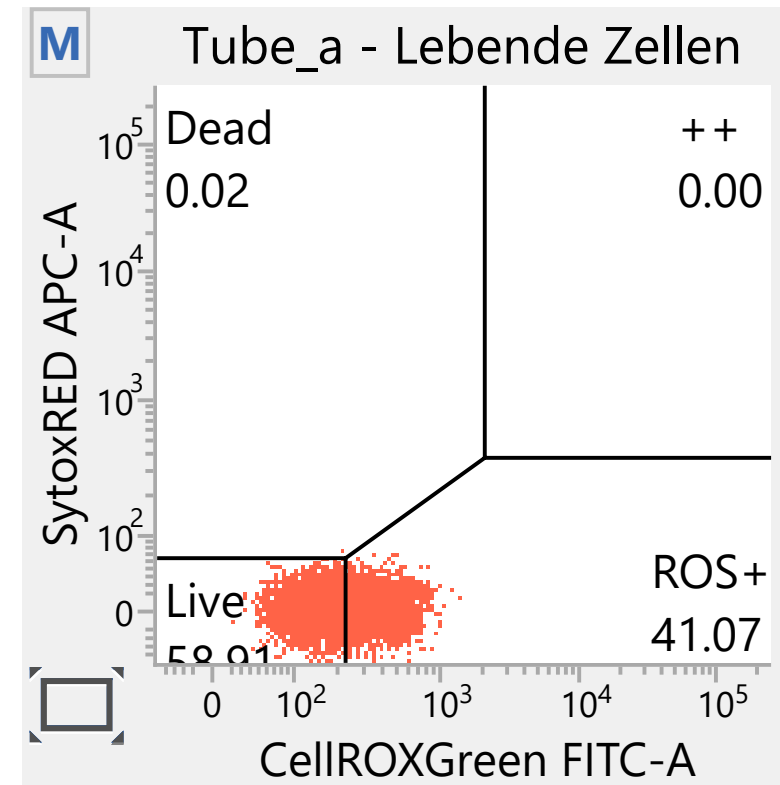

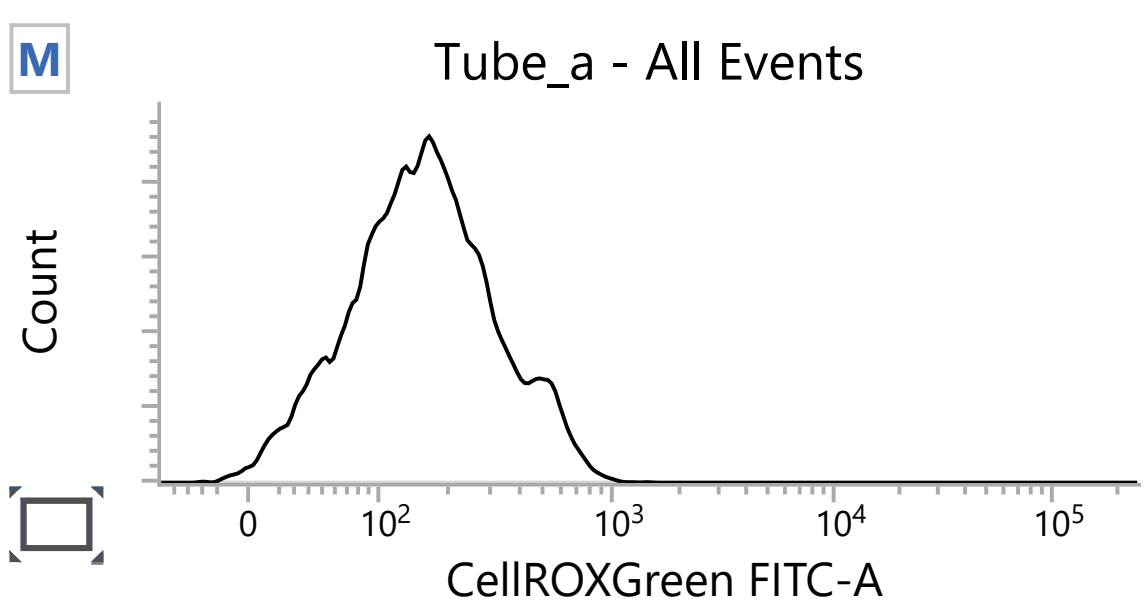

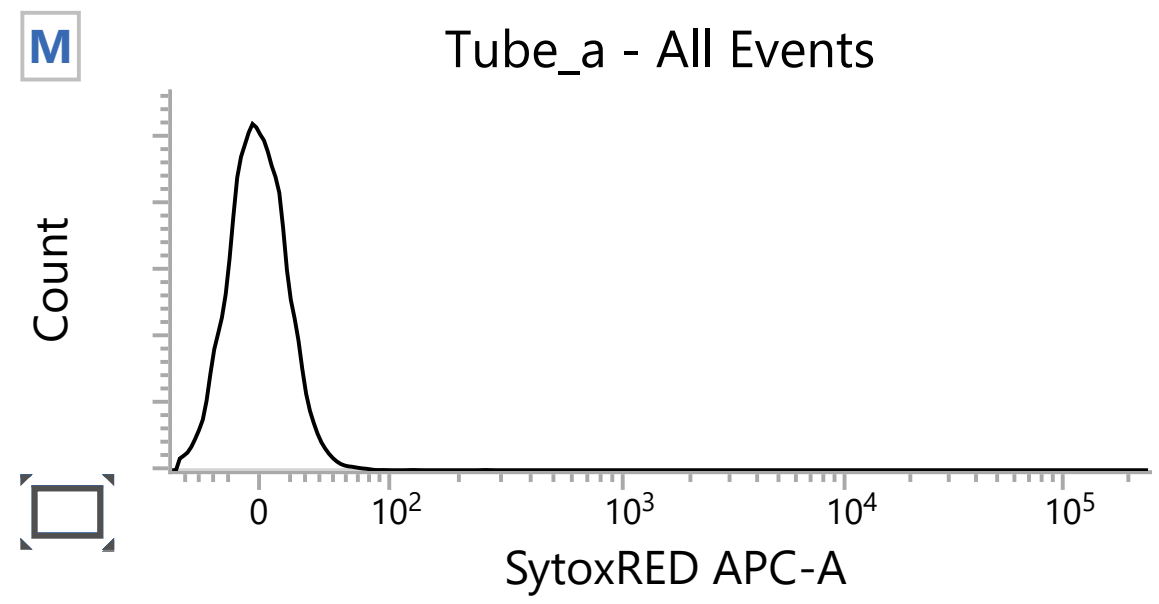

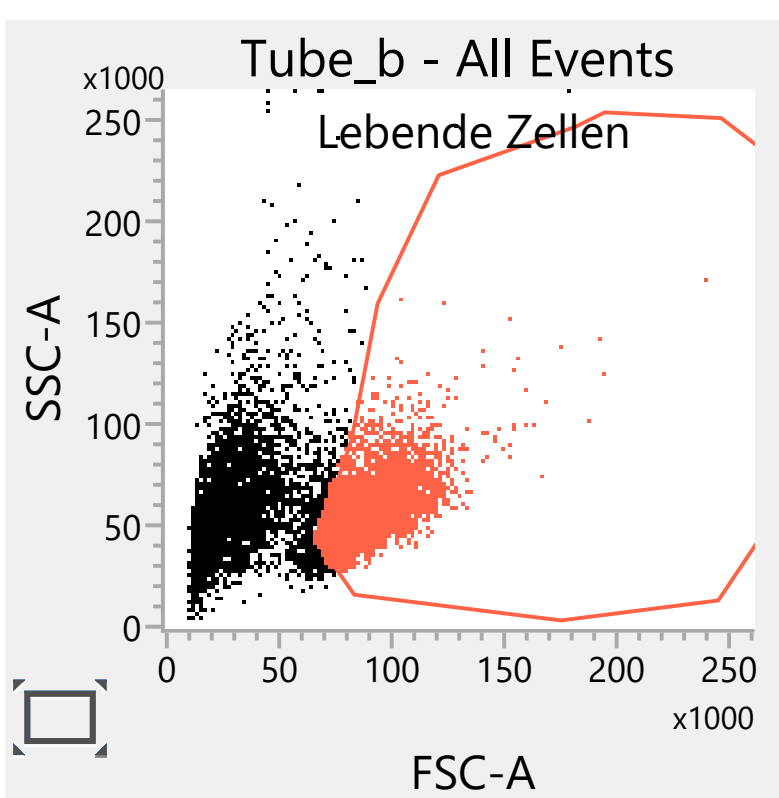

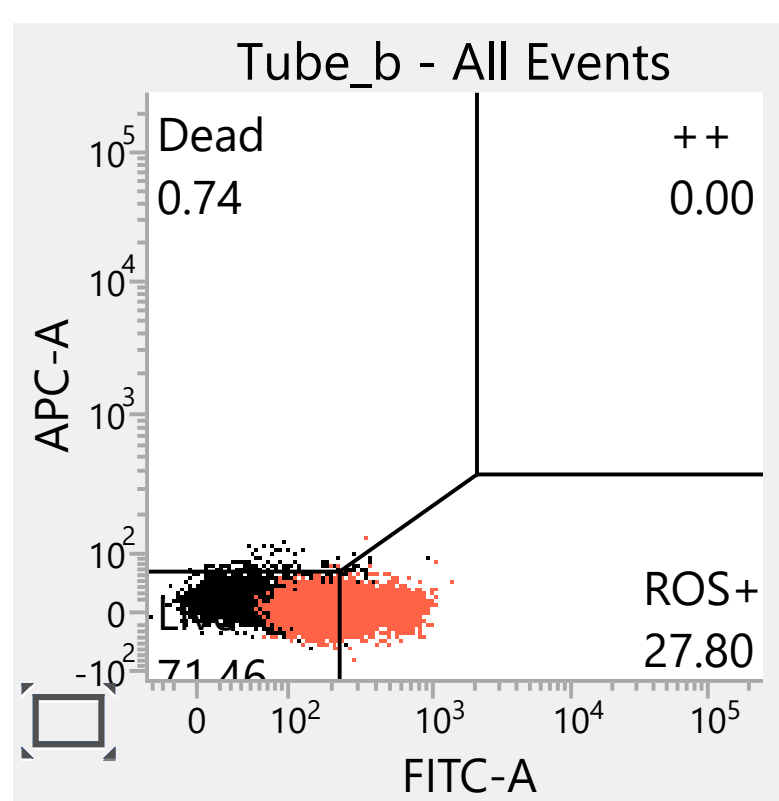

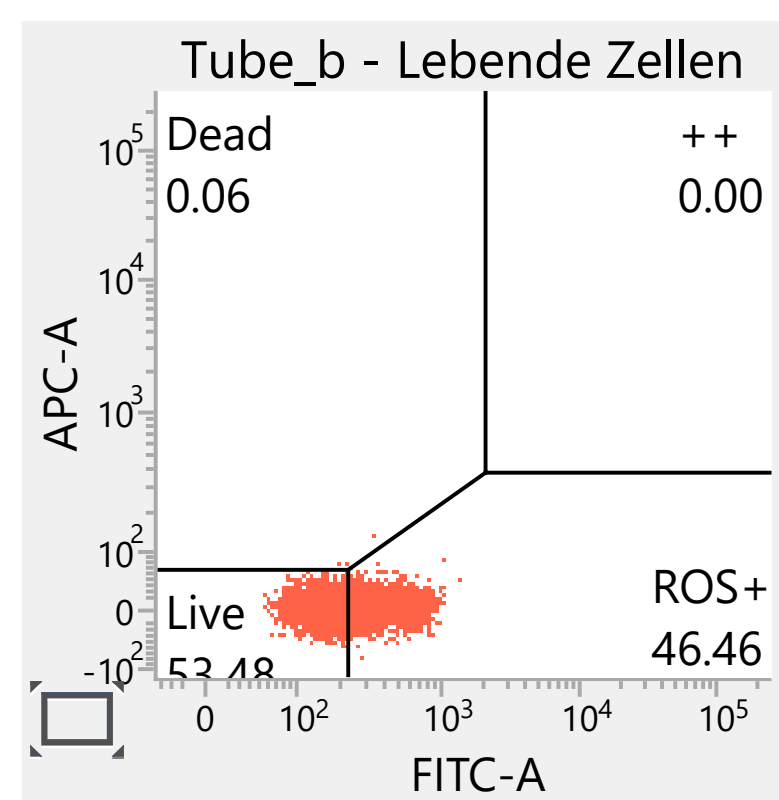

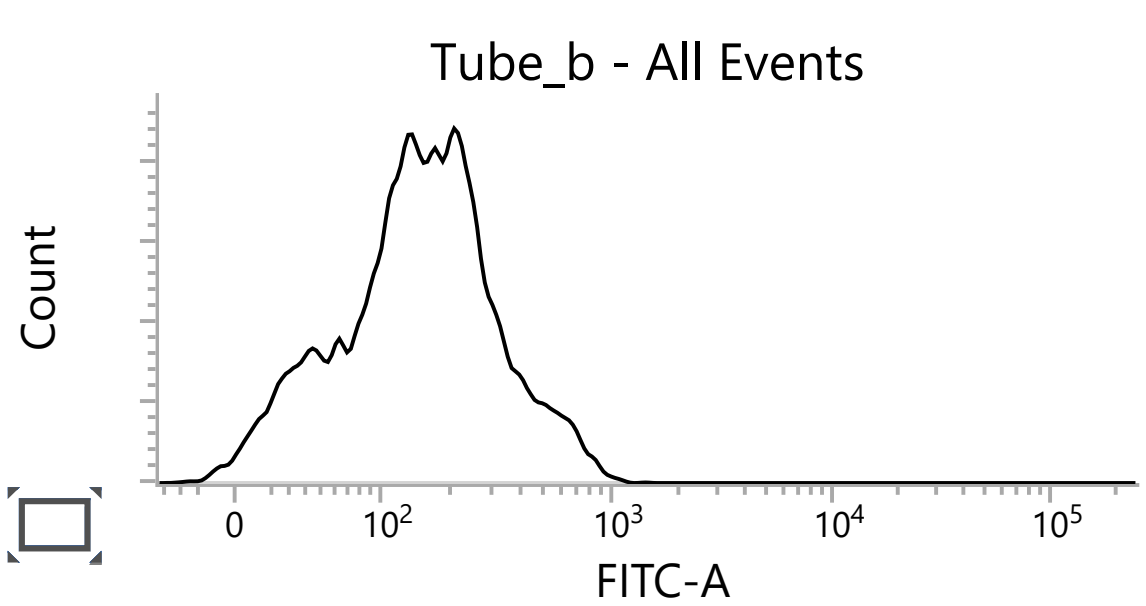

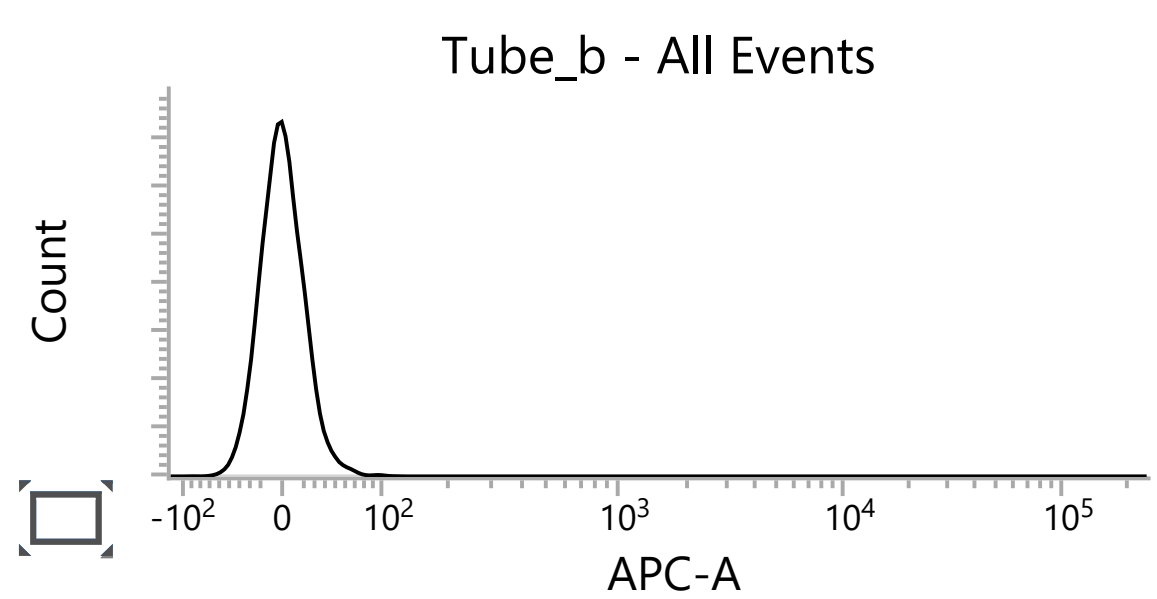

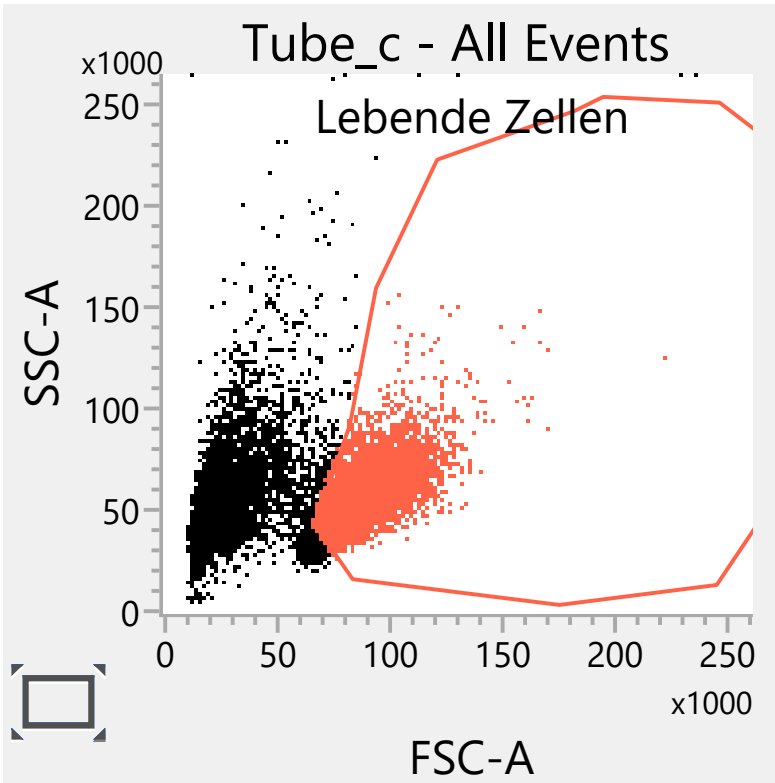

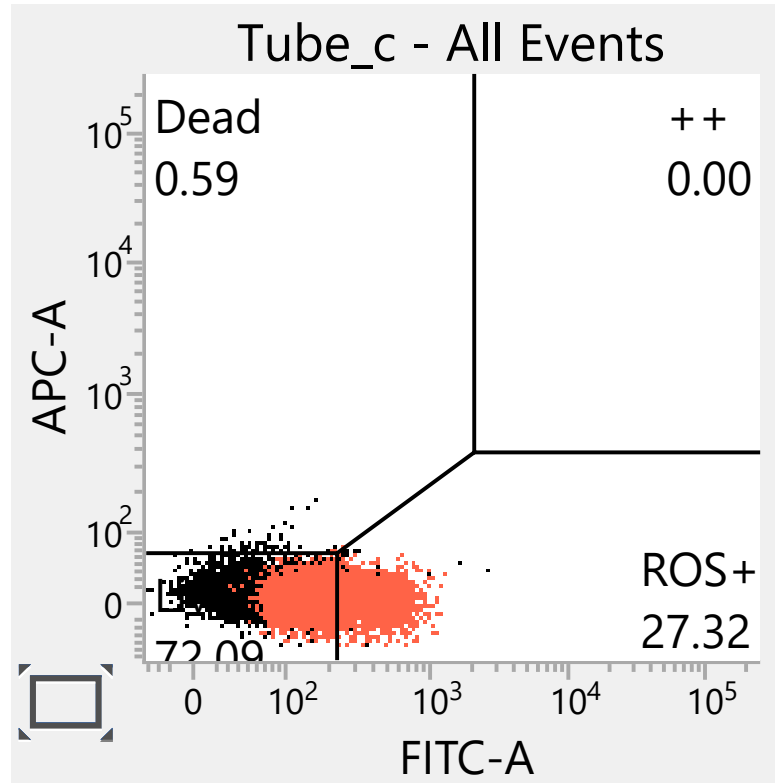

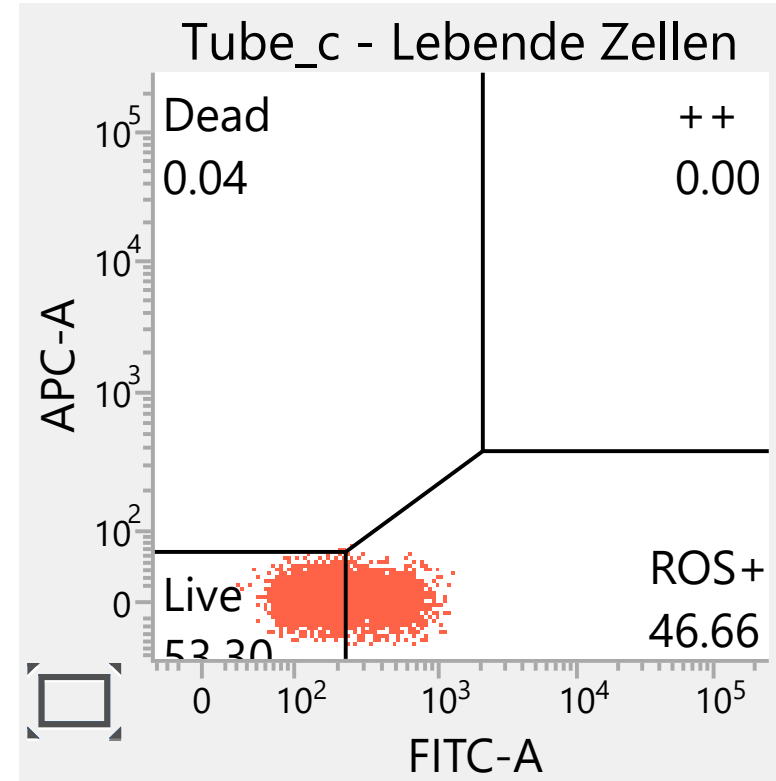

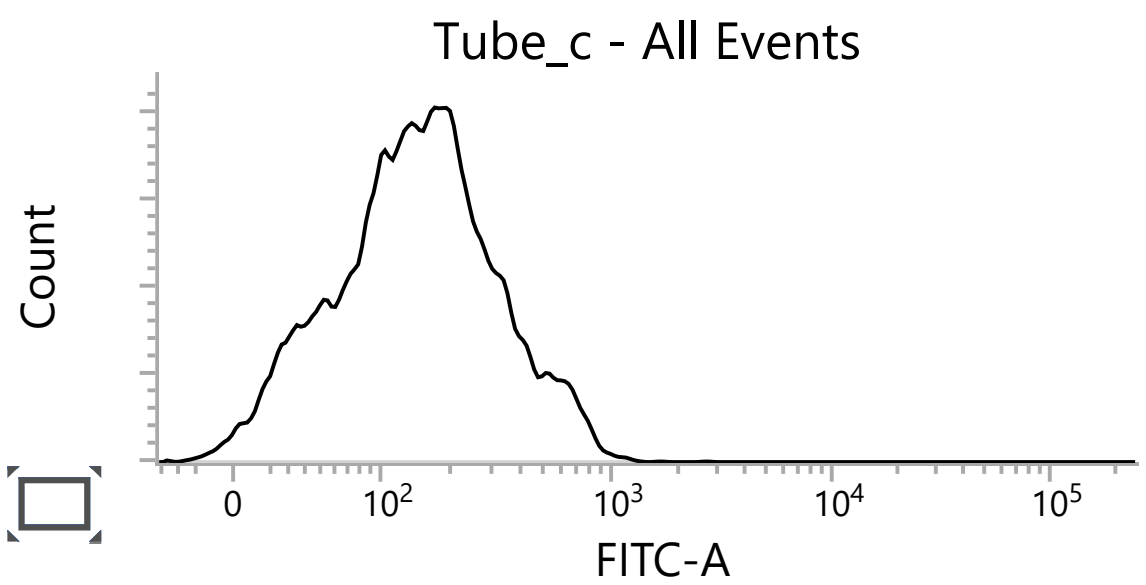

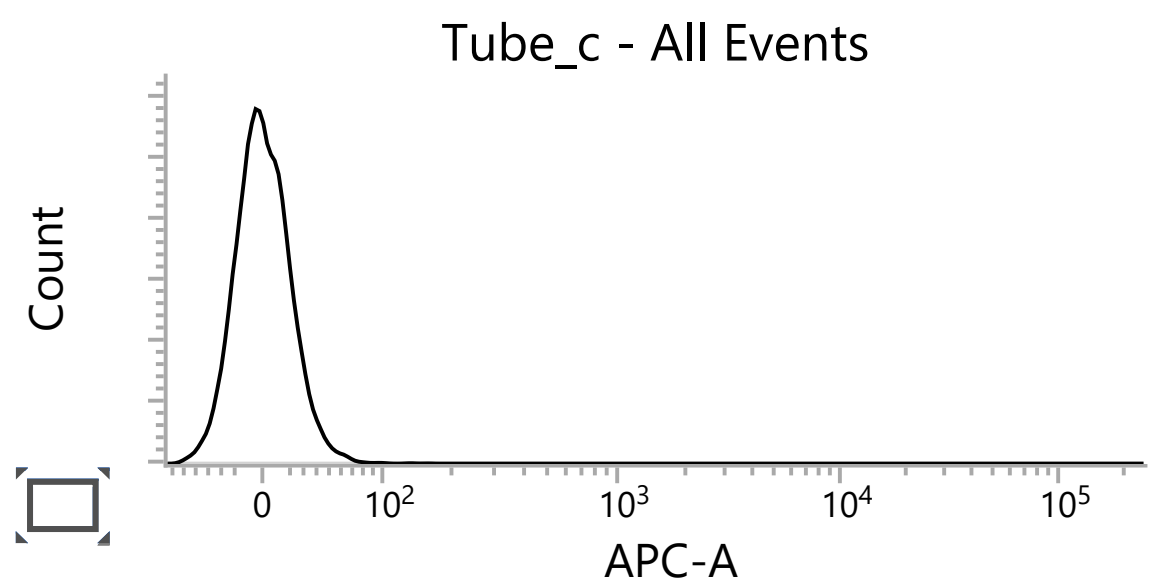

Statistics

Name	Events	% Parent	% Grandparent	% Total
Tube_a:All Events	10,000	***	***	100.00
Tube_a:Lebende Zellen	6,625	66.25	***	66.25
Tube_b:All Events	10,000	***	***	100.00
Tube_b:Lebende Zellen	5,204	52.04	***	52.04
Tube_c:All Events	10,000	***	***	100.00
Tube_c:Lebende Zellen	5,334	53.34	***	53.34

CellROX Assay

Sample ID: DMSO

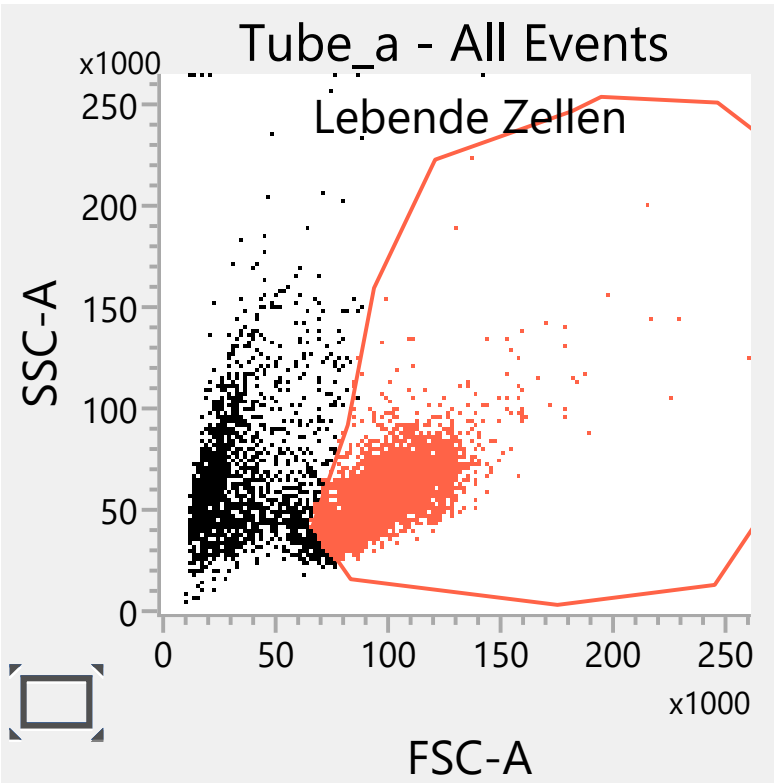

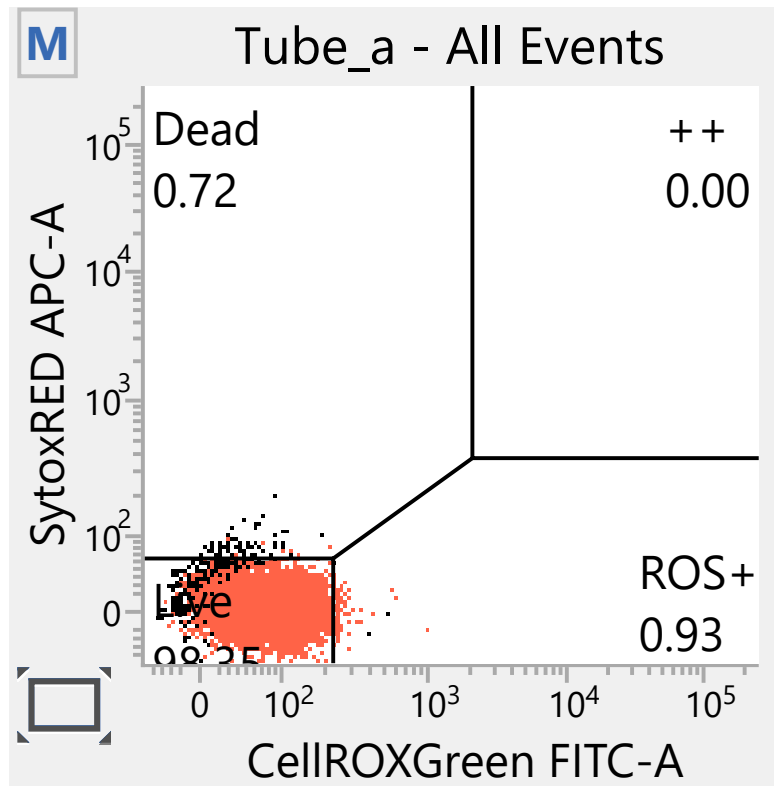

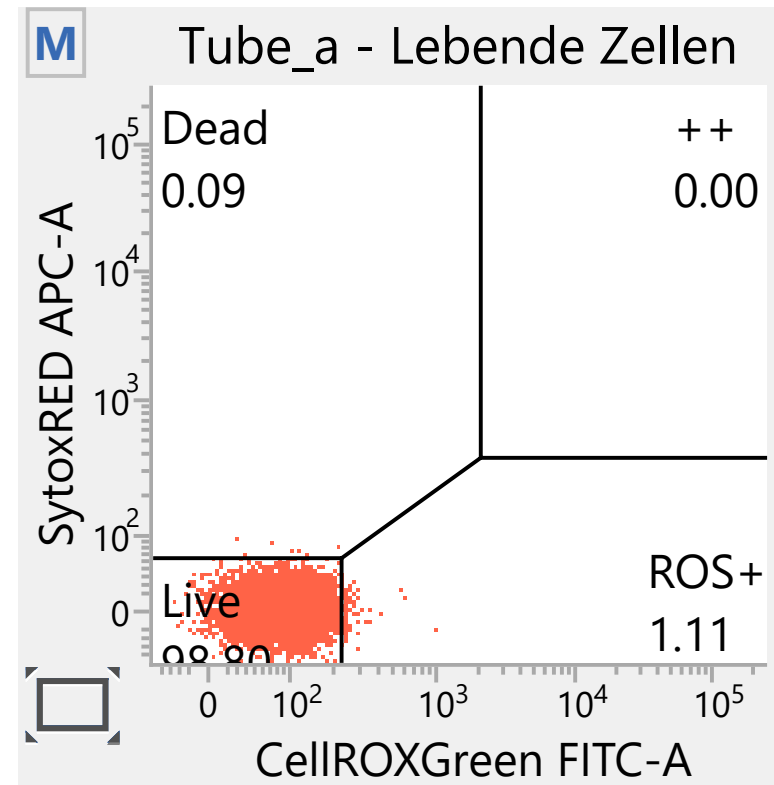

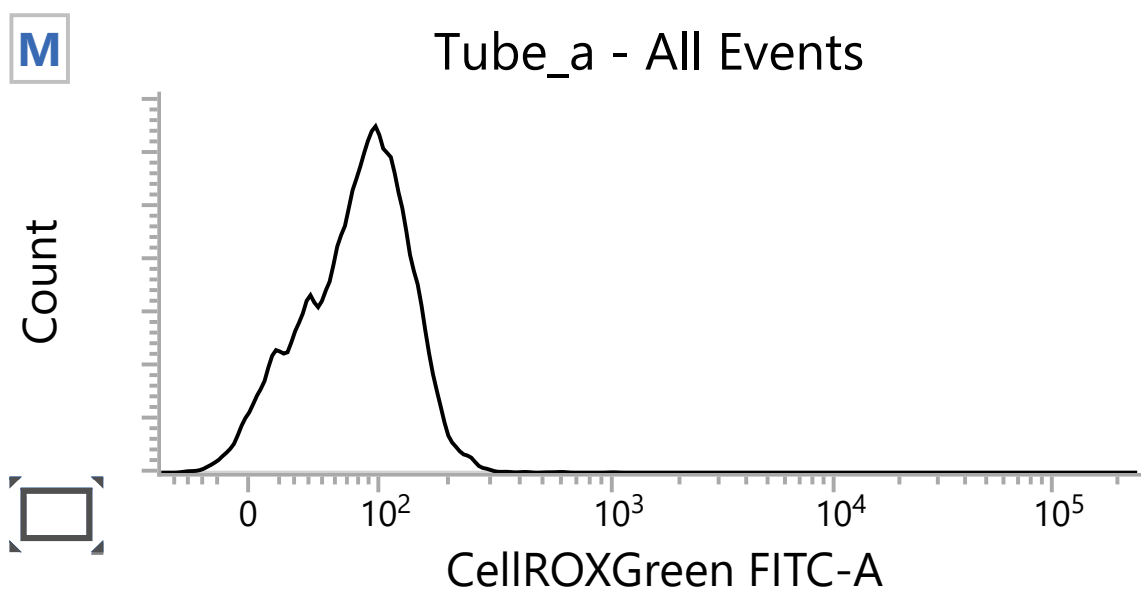

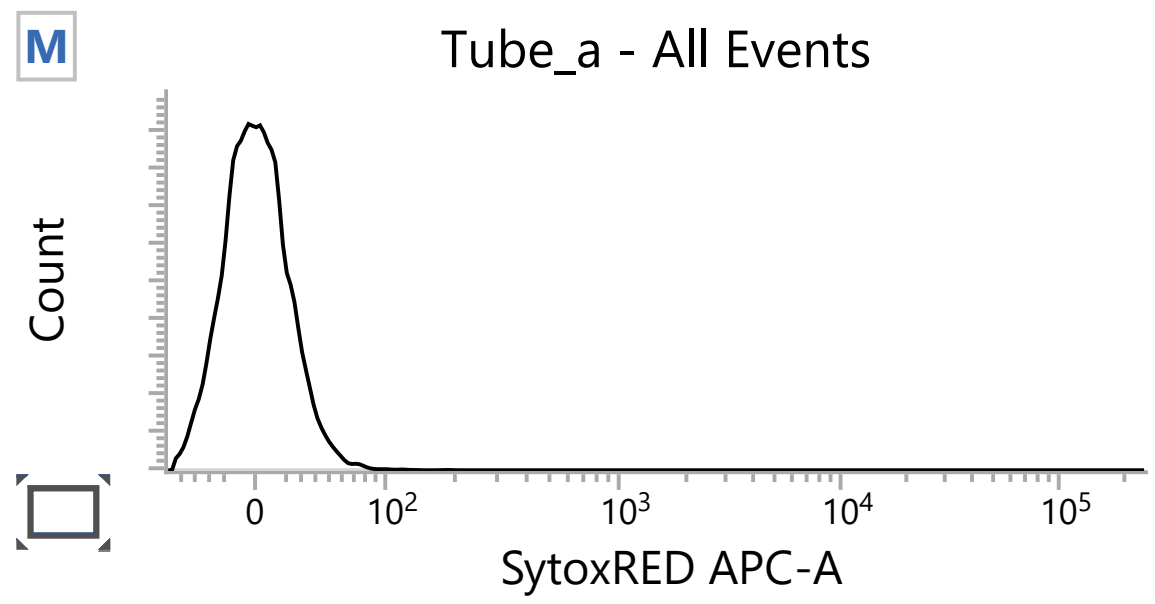

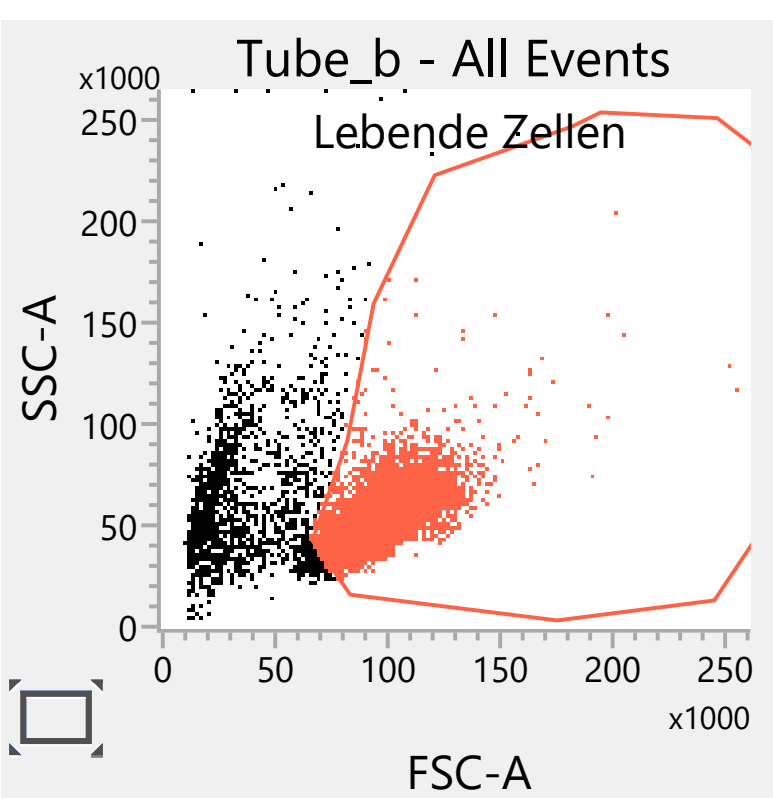

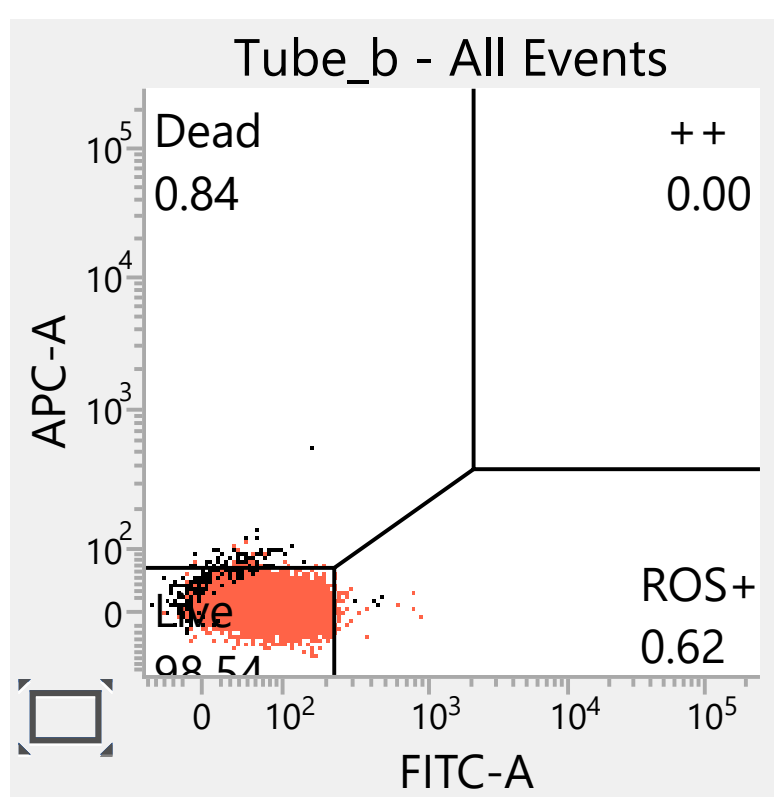

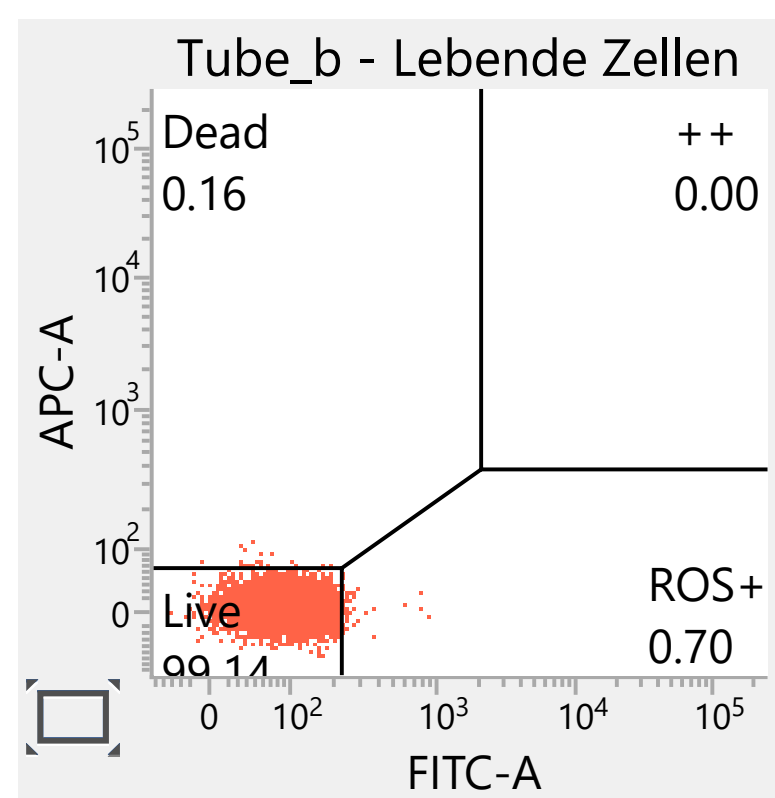

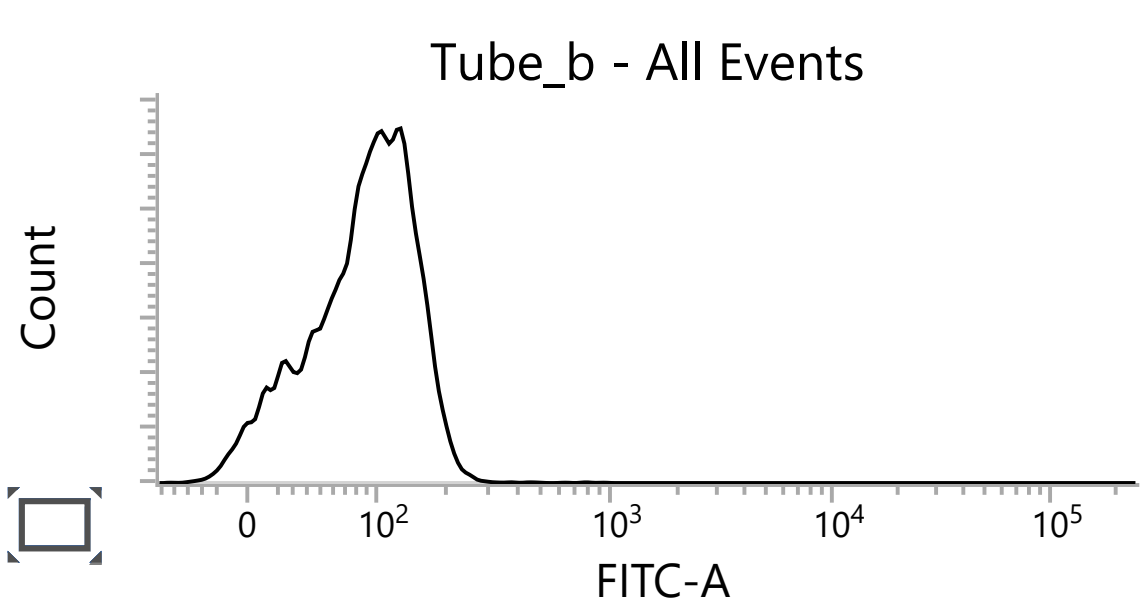

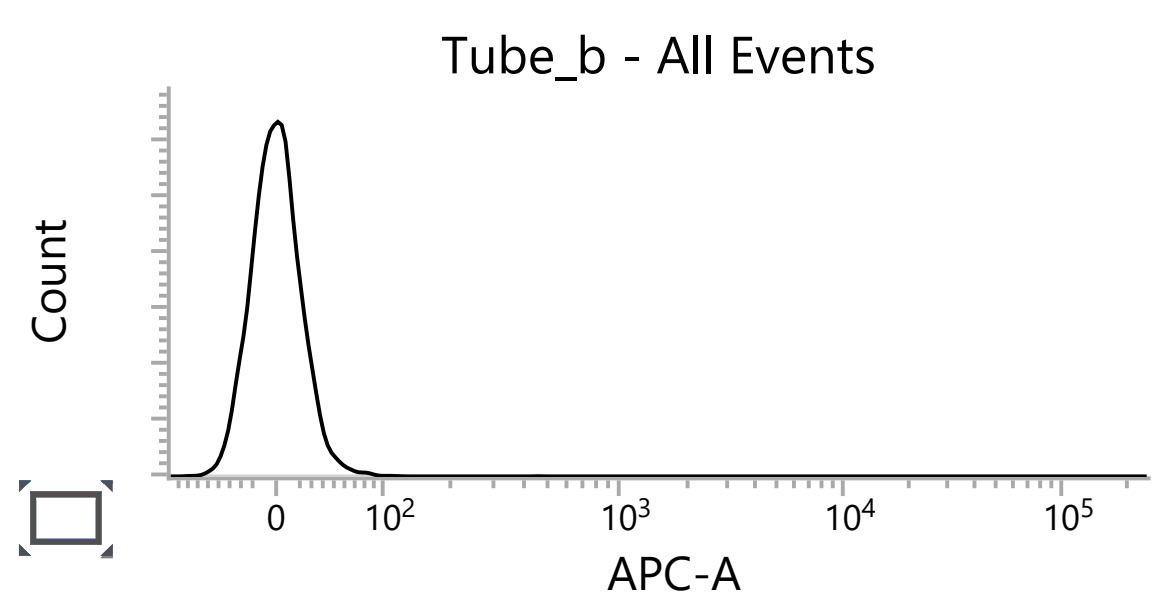

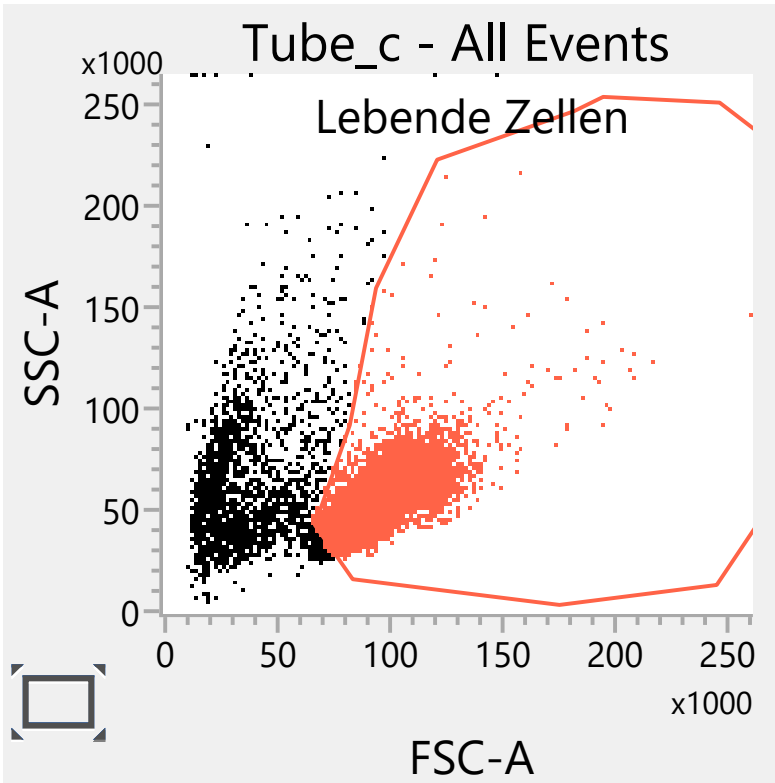

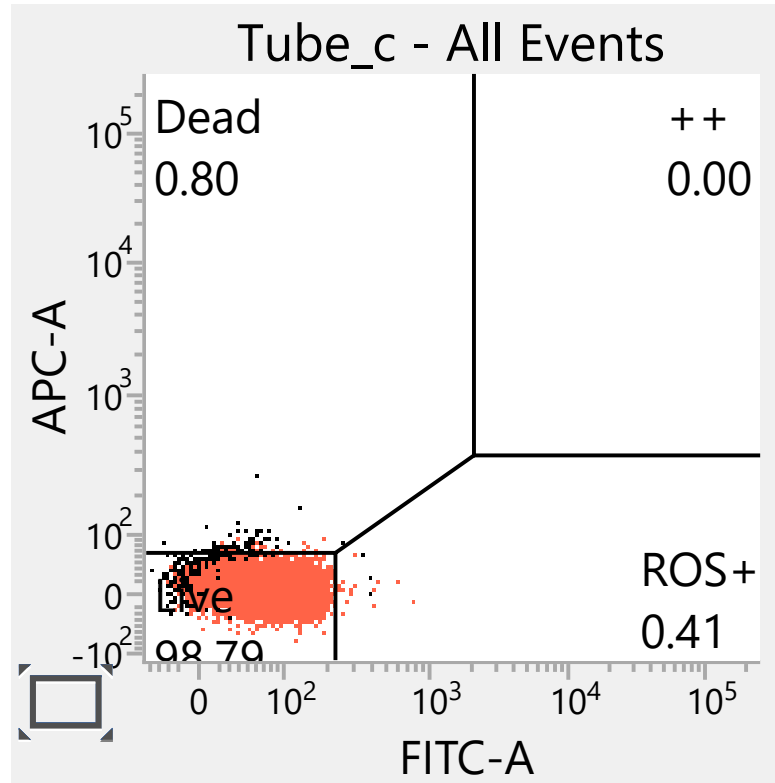

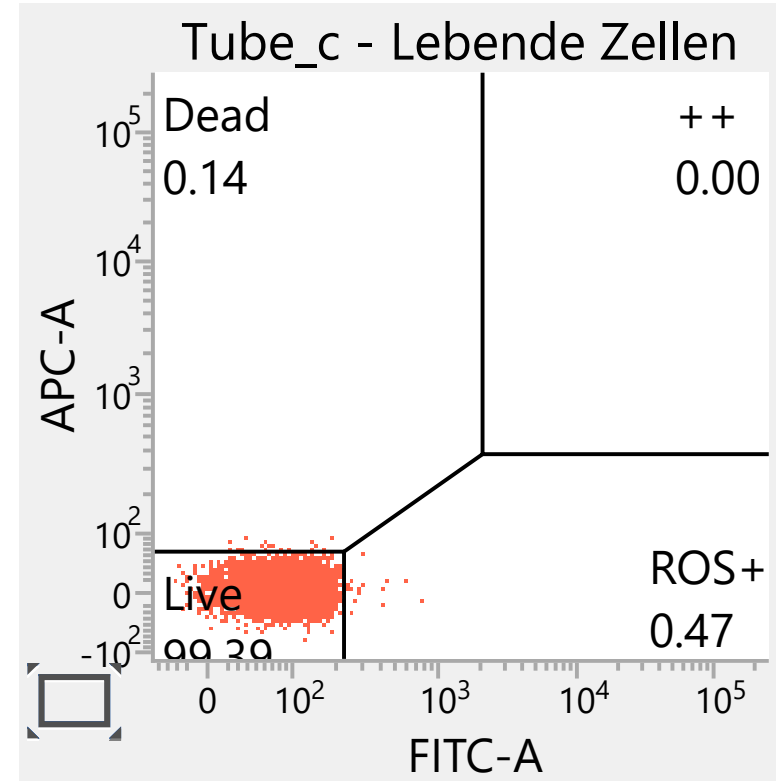

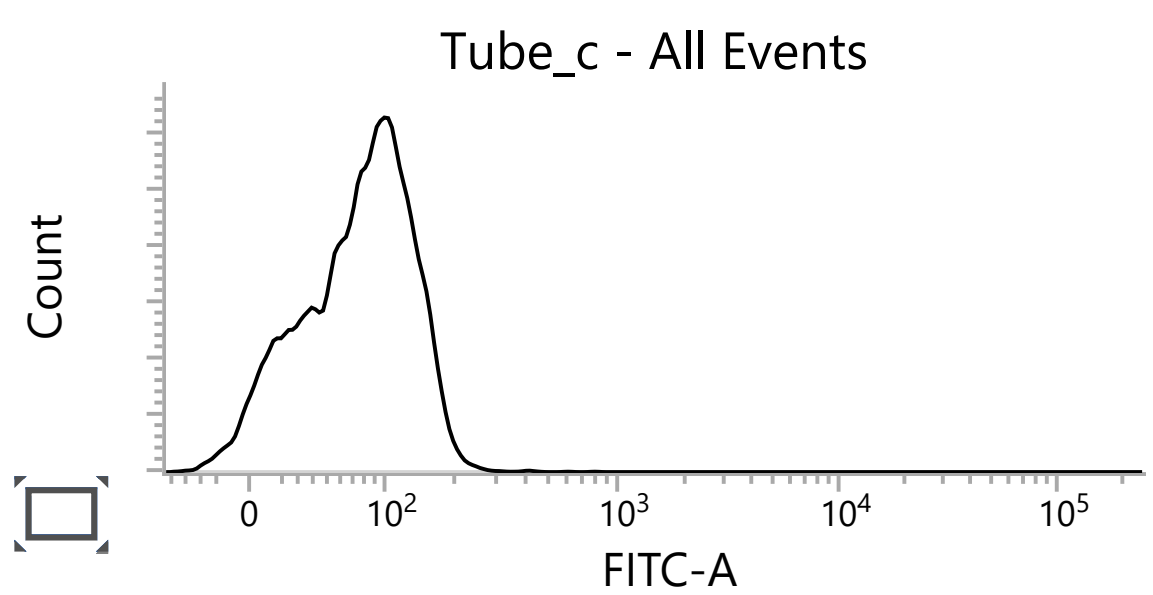

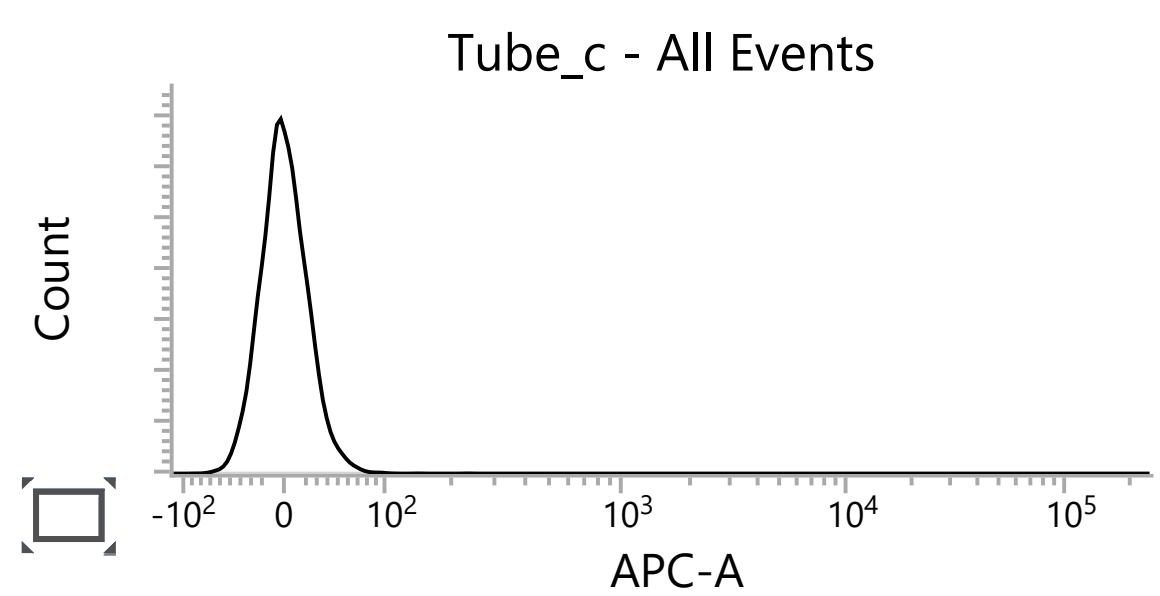

Statistics

Name	Events	% Parent	% Grandparent	% Total
Tube_a:All Events	10,000	***	***	100.00
Tube_a:Lebende Zellen	8,112	81.12	***	81.12
Tube_b:All Events	10,000	***	***	100.00
Tube_b:Lebende Zellen	8,304	83.04	***	83.04
Tube_c:All Events	10,000	***	***	100.00
Tube_c:Lebende Zellen	7,908	79.08	***	79.08

CellROX Assay

Sample ID: H_2O_2 3 +NAC

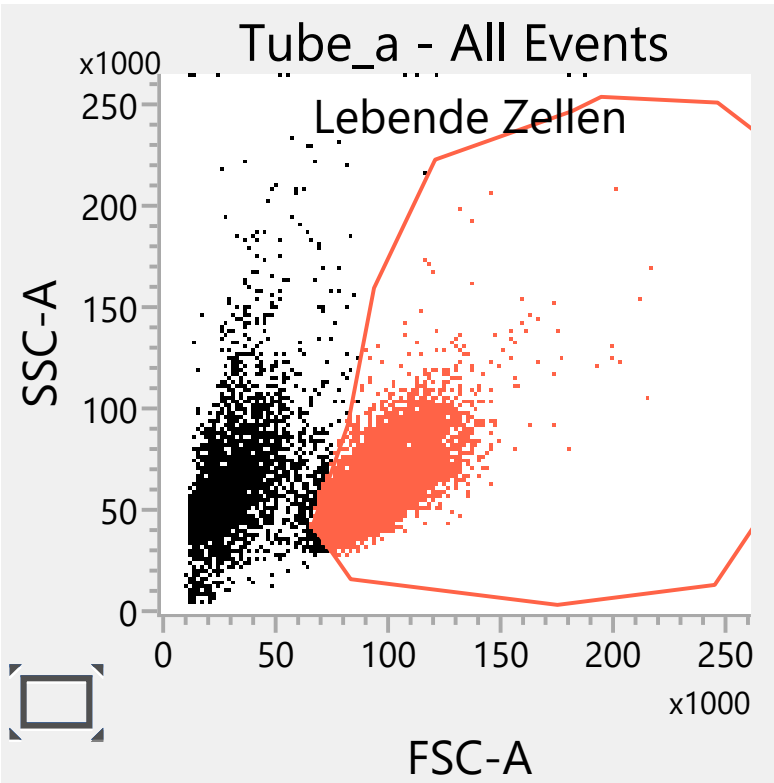

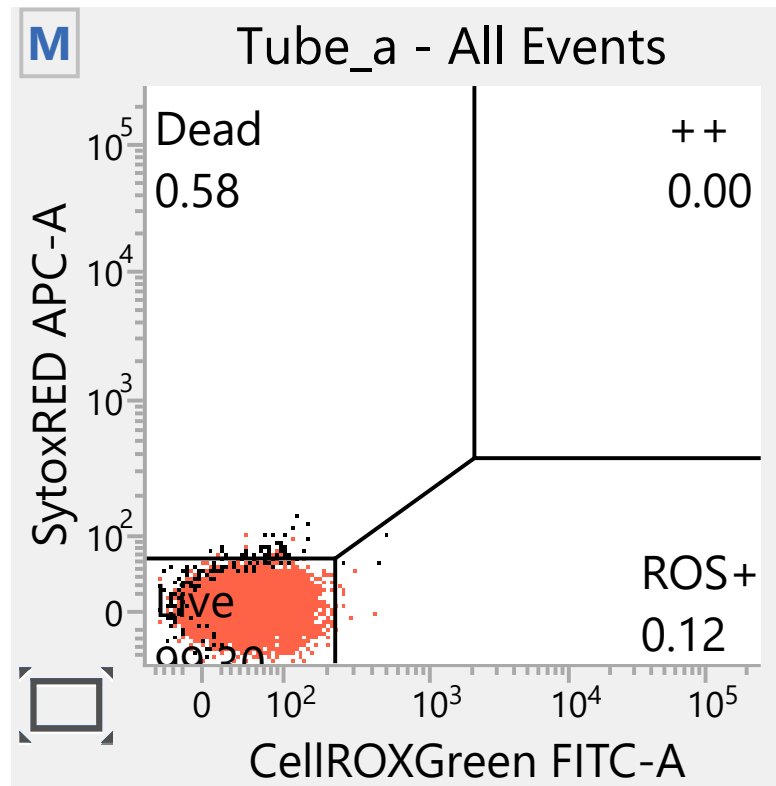

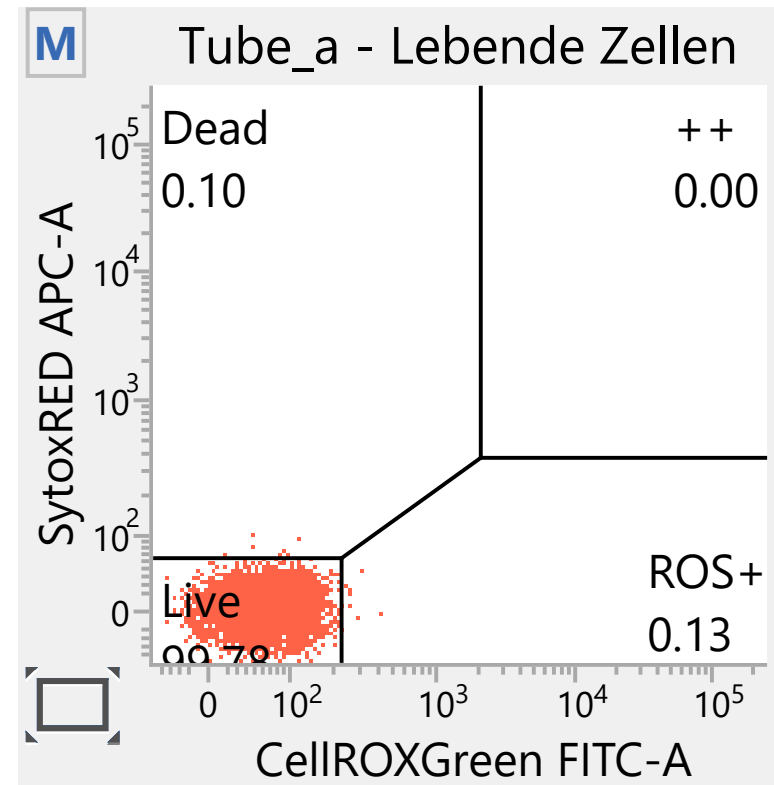

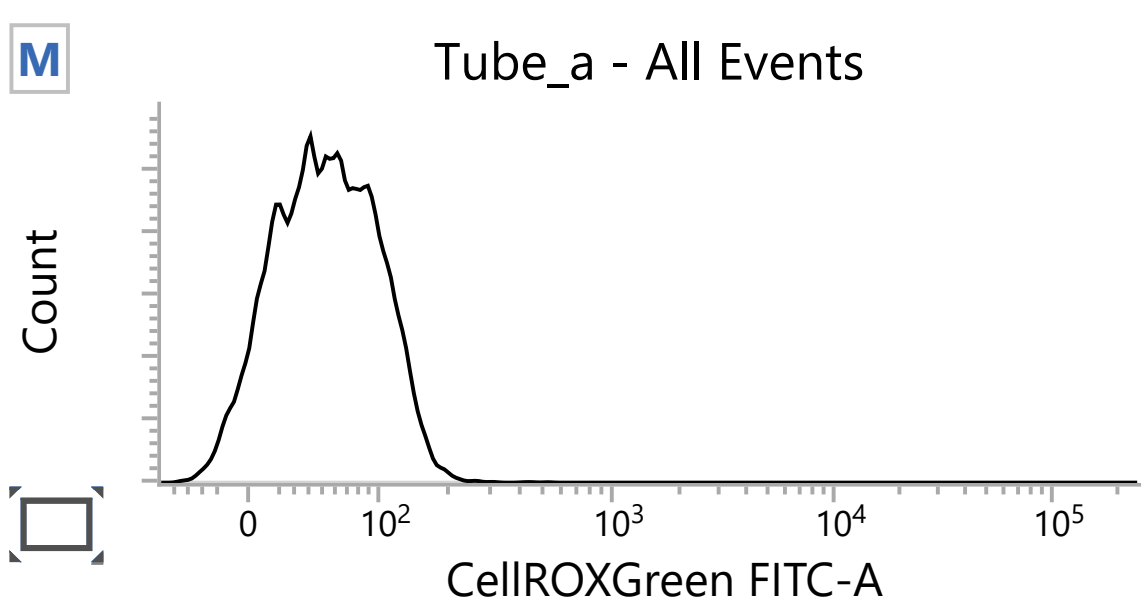

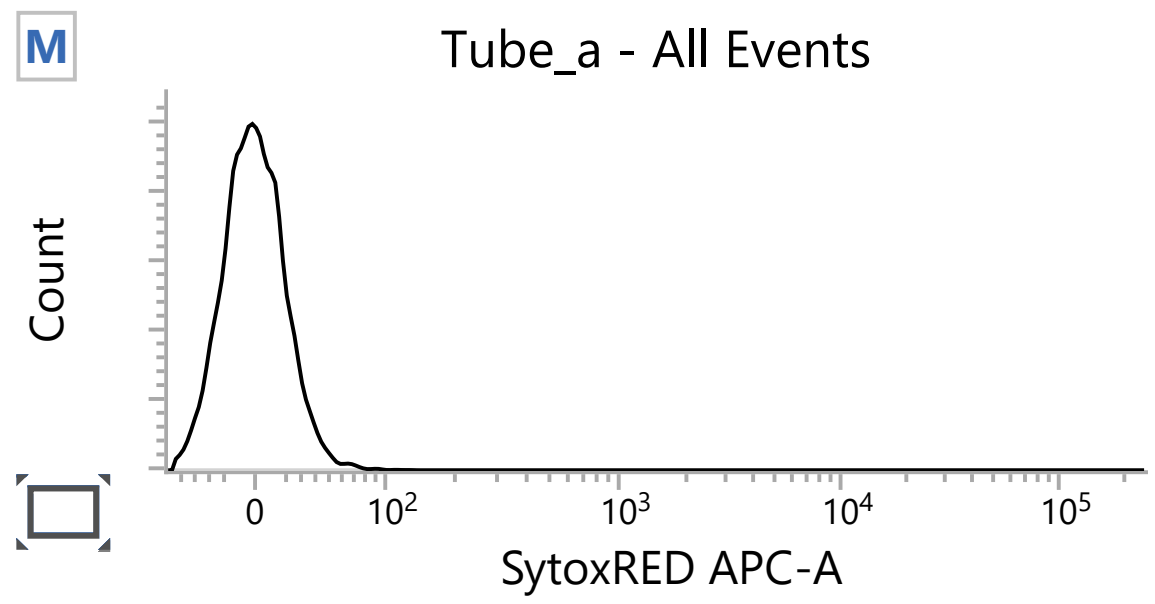

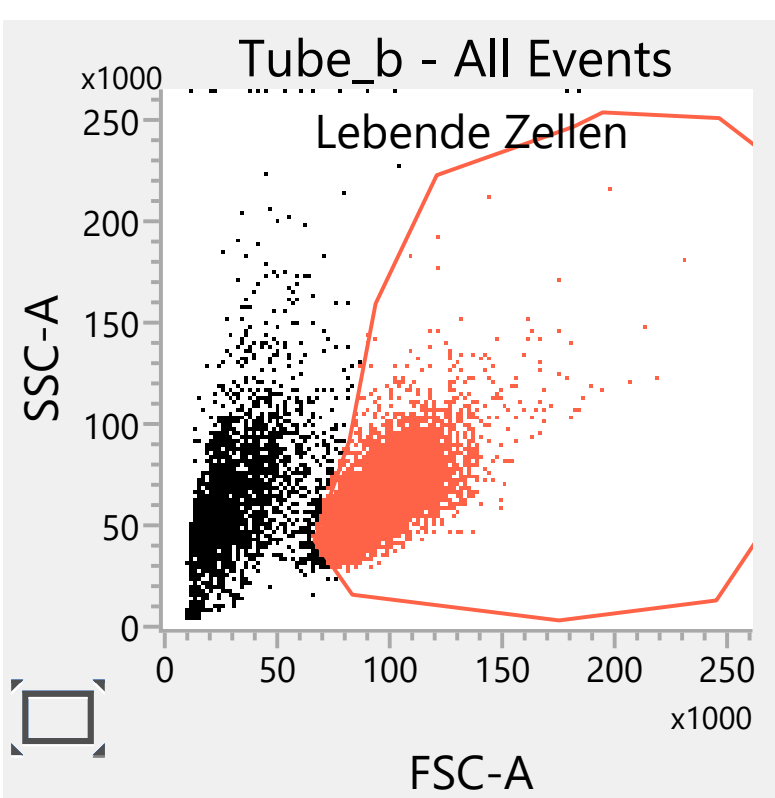

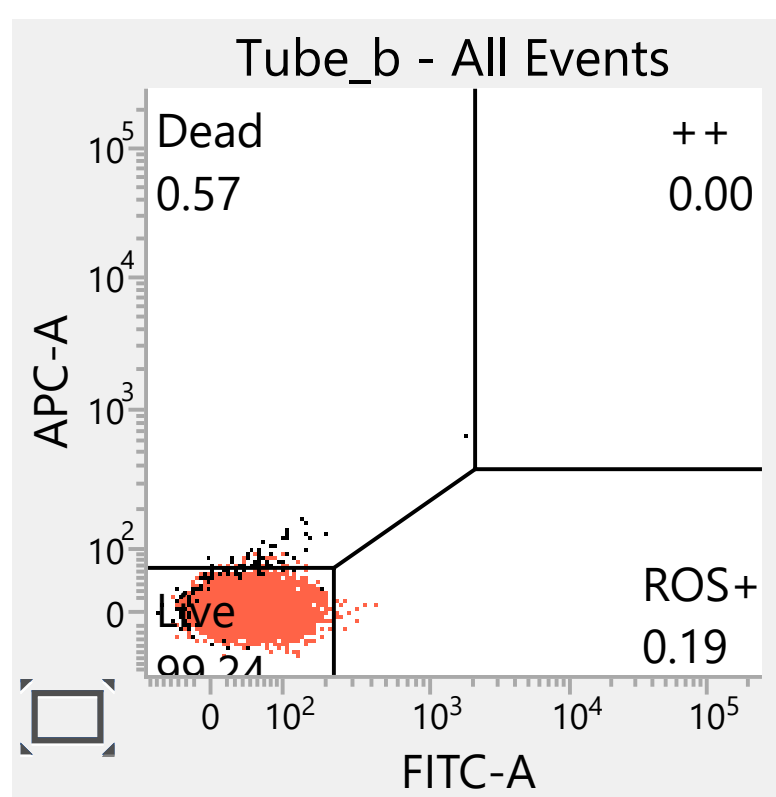

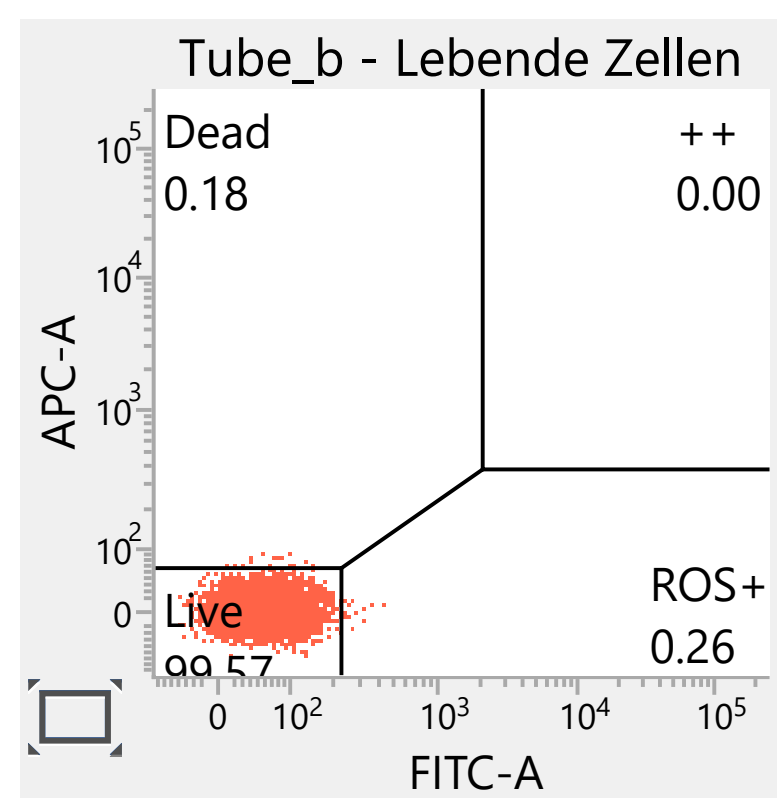

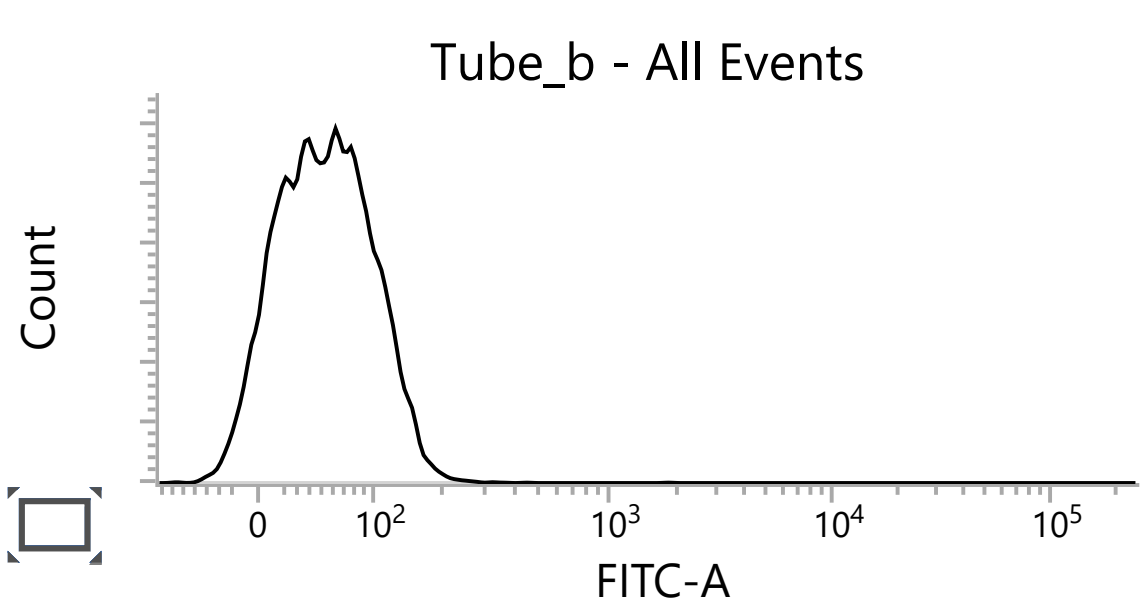

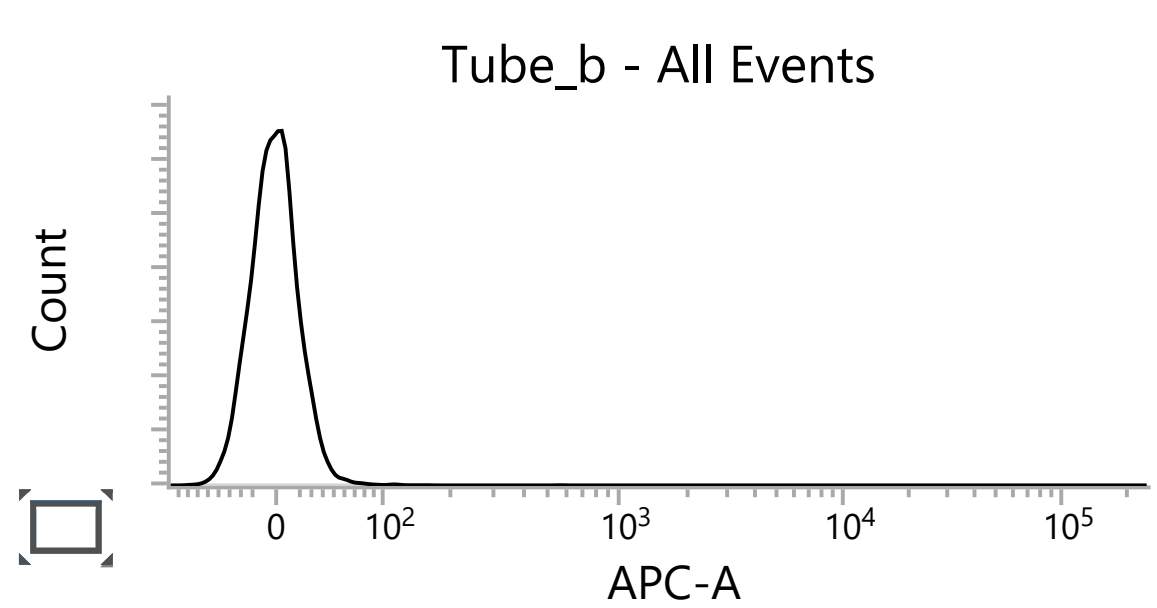

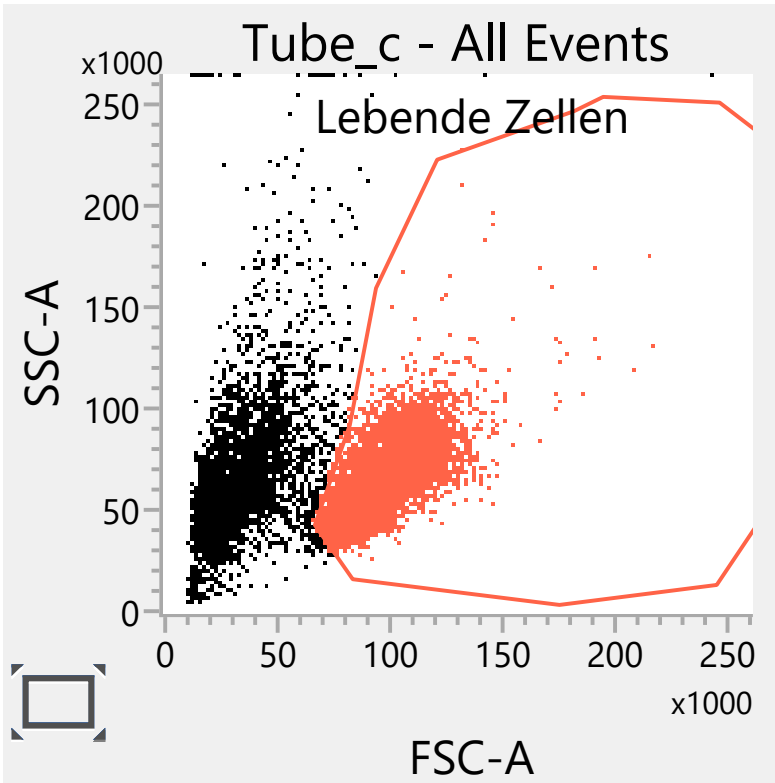

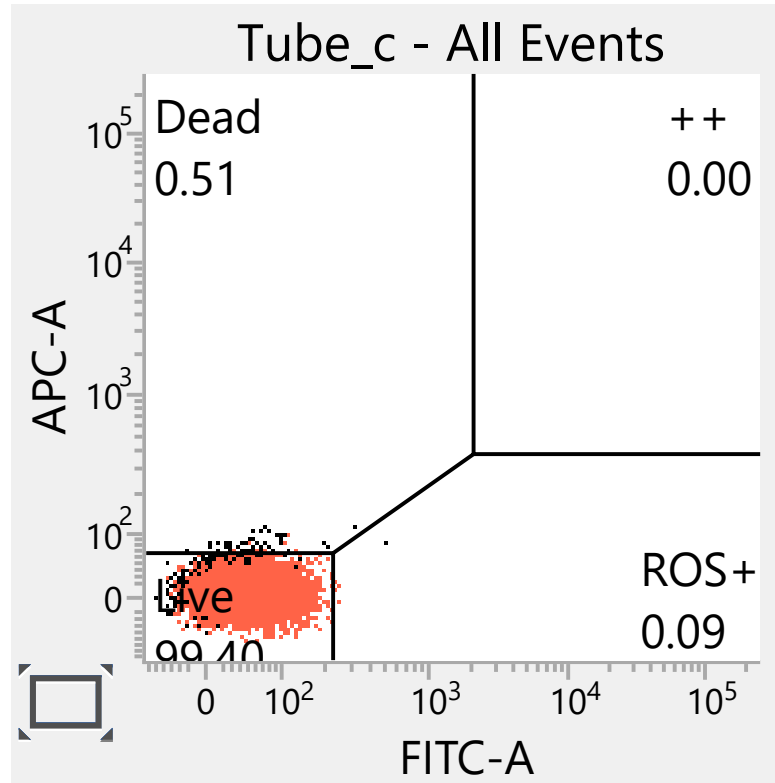

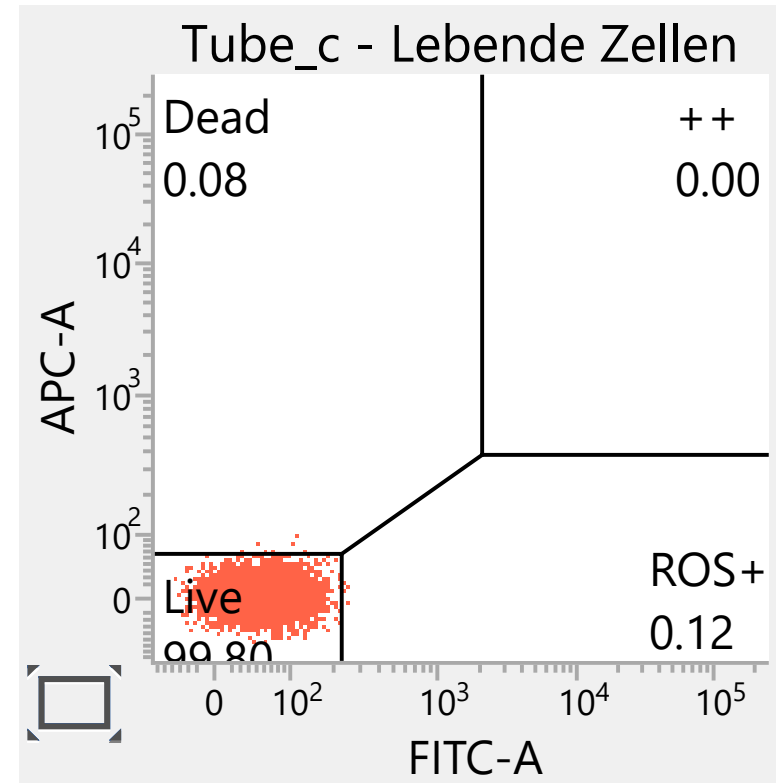

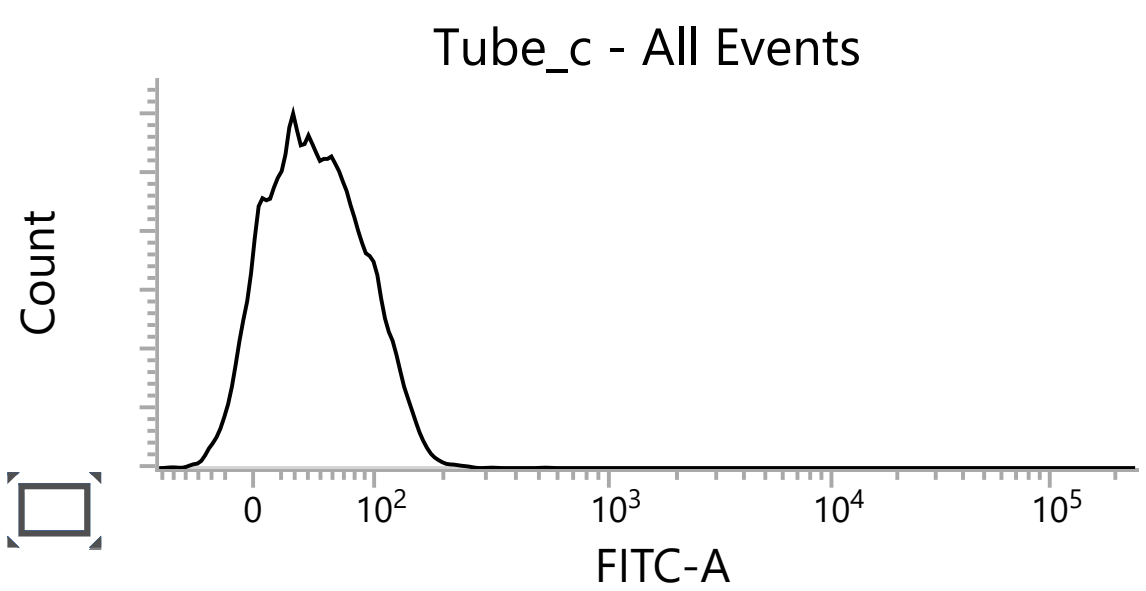

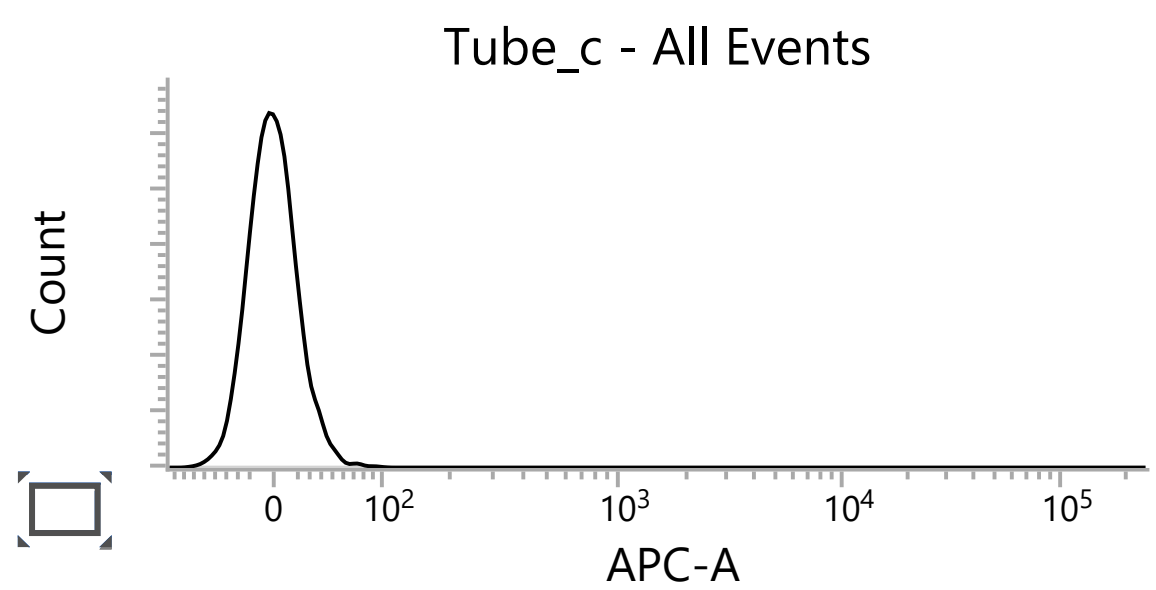

Statistics

Name	Events	% Parent	% Grandparent	% Total
Tube_a:All Events	10,000	***	***	100.00
Tube_a:Lebende Zellen	7,155	71.55	***	71.55
Tube_b:All Events	10,000	***	***	100.00
Tube_b:Lebende Zellen	7,418	74.18	***	74.18
Tube_c:All Events	10,000	***	***	100.00
Tube_c:Lebende Zellen	6,480	64.80	***	64.80

CellROX Assay

Sample ID: H_2O_2 3

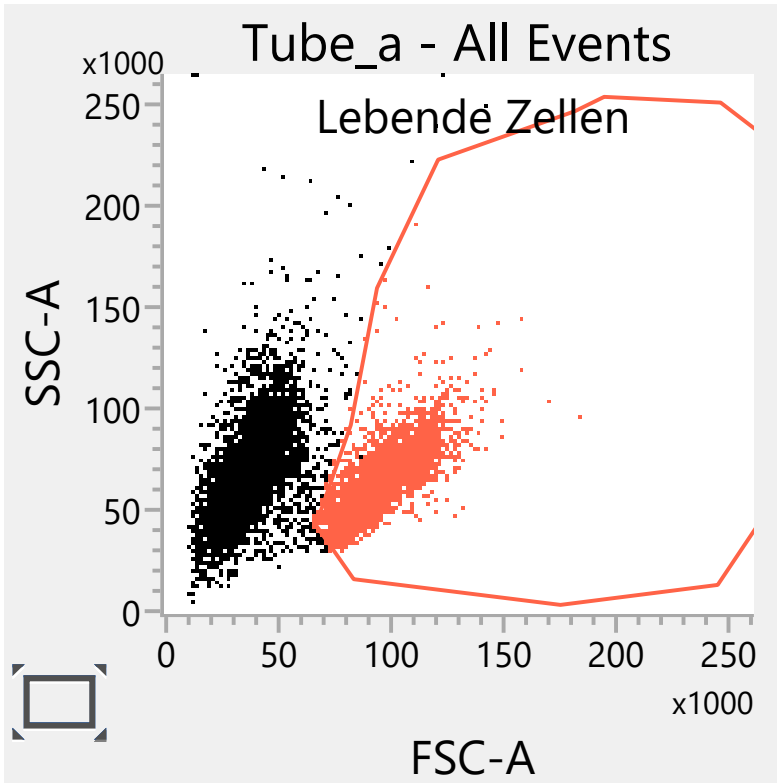

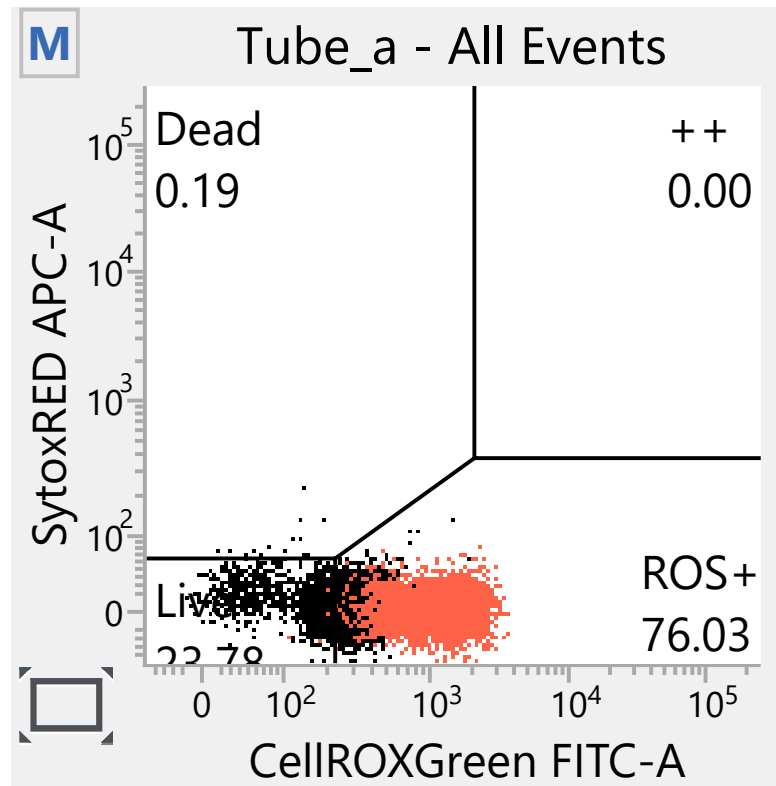

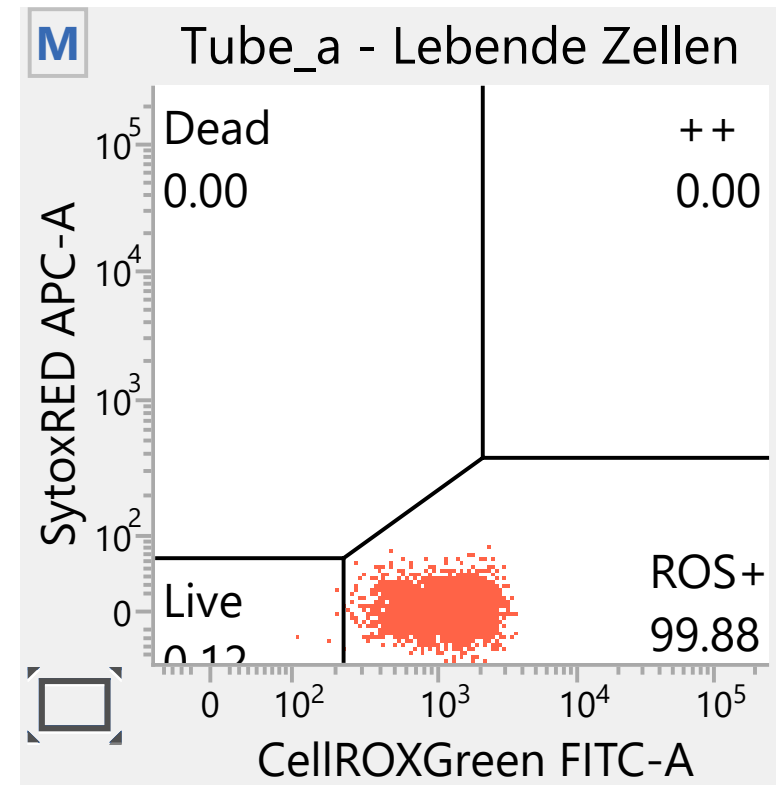

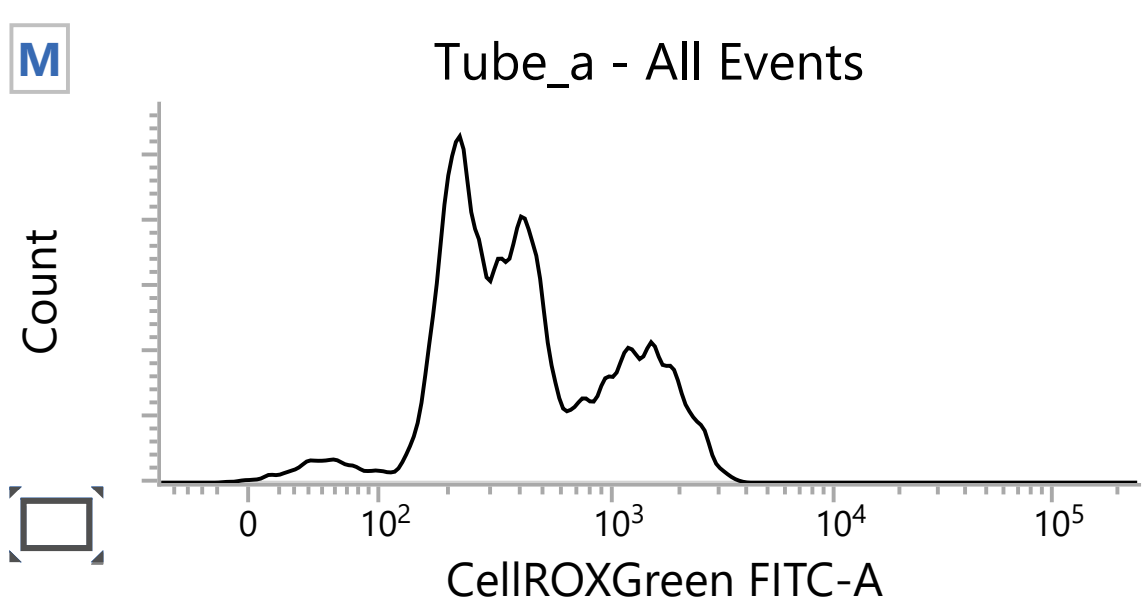

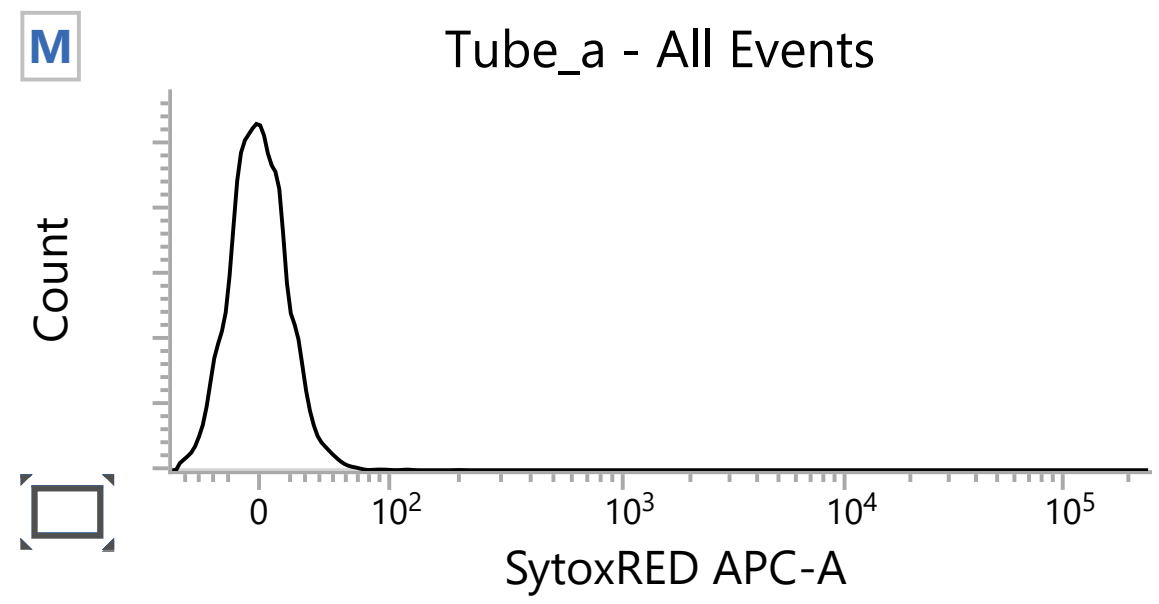

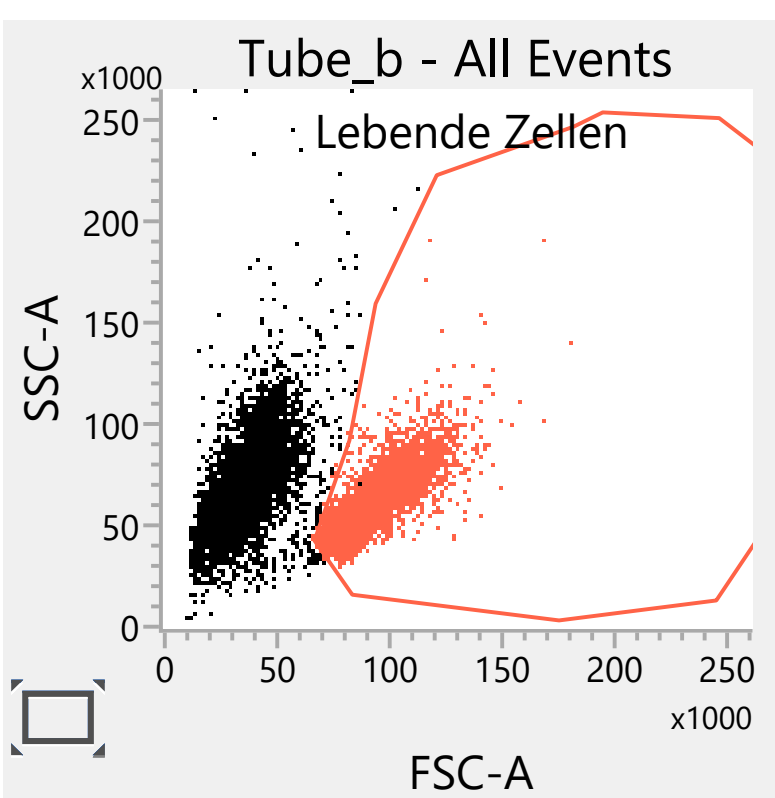

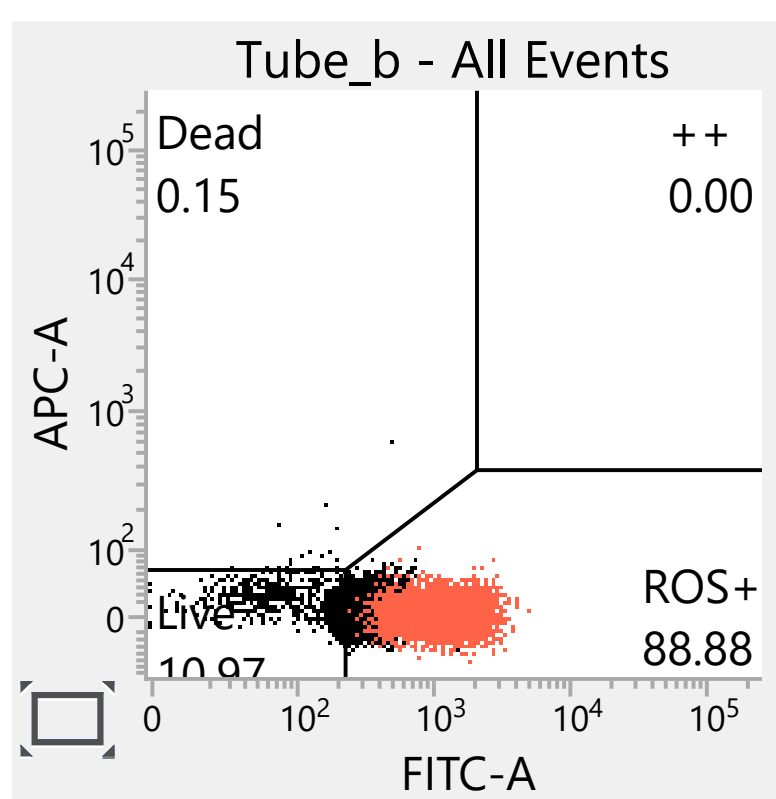

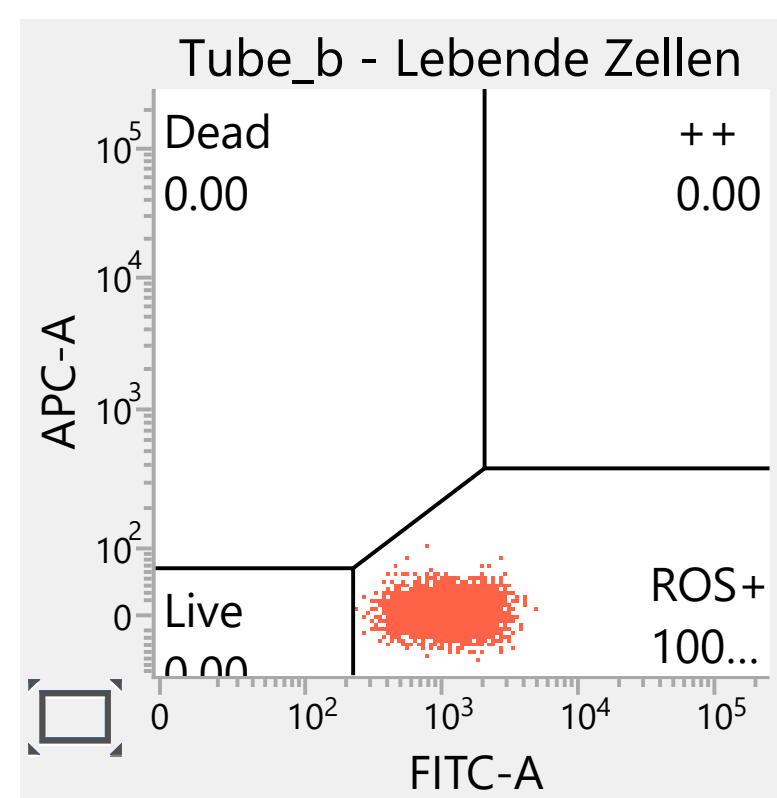

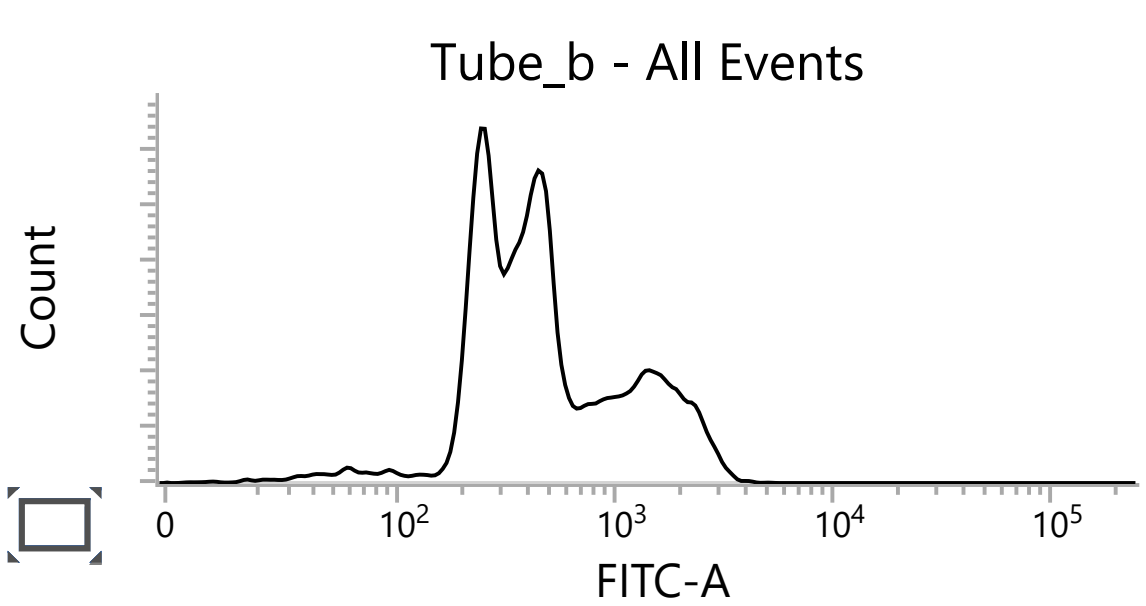

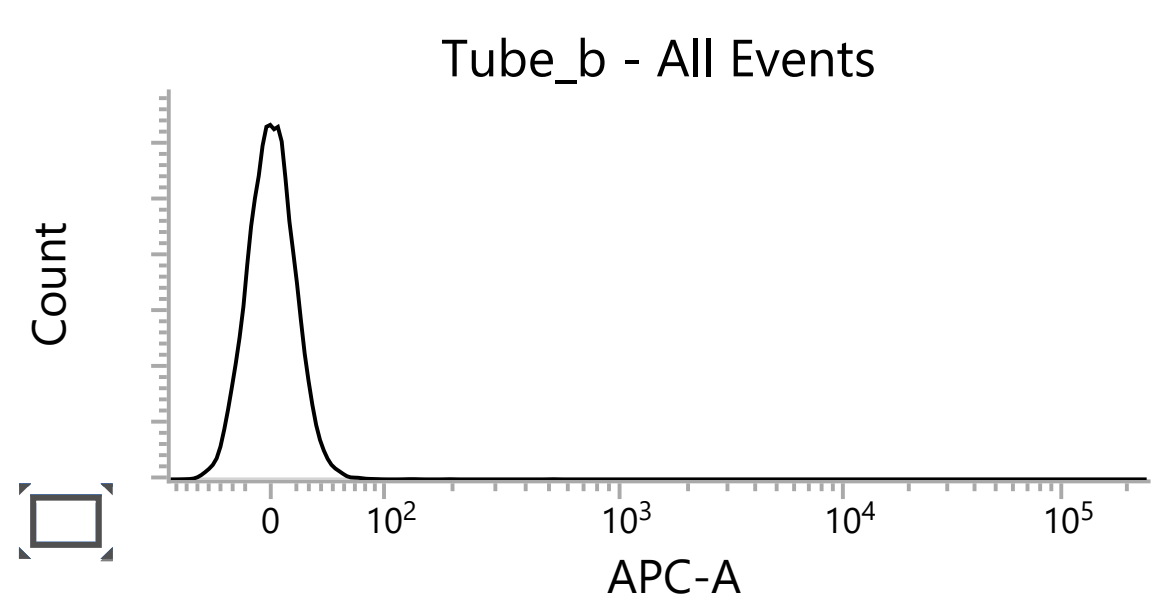

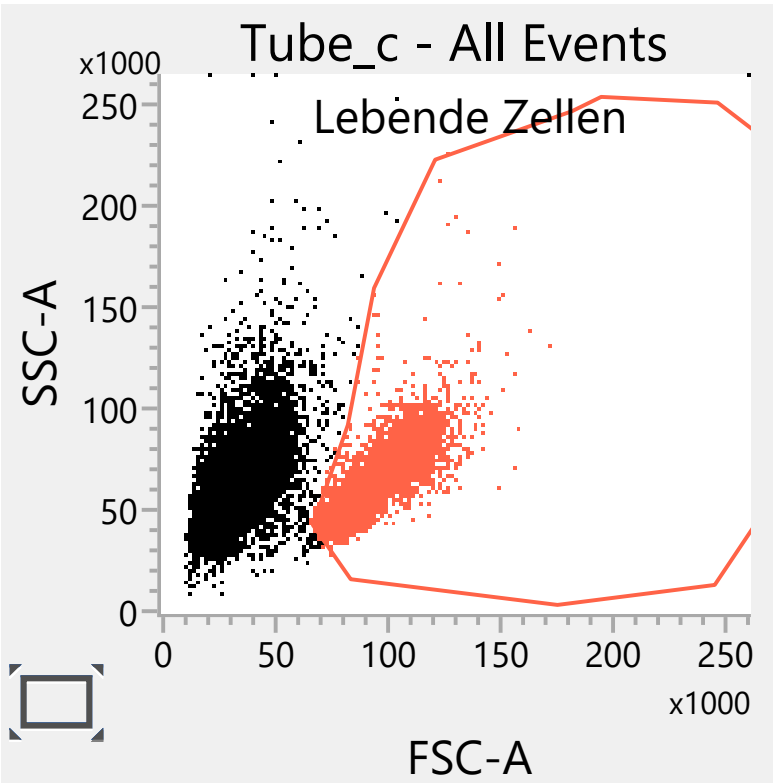

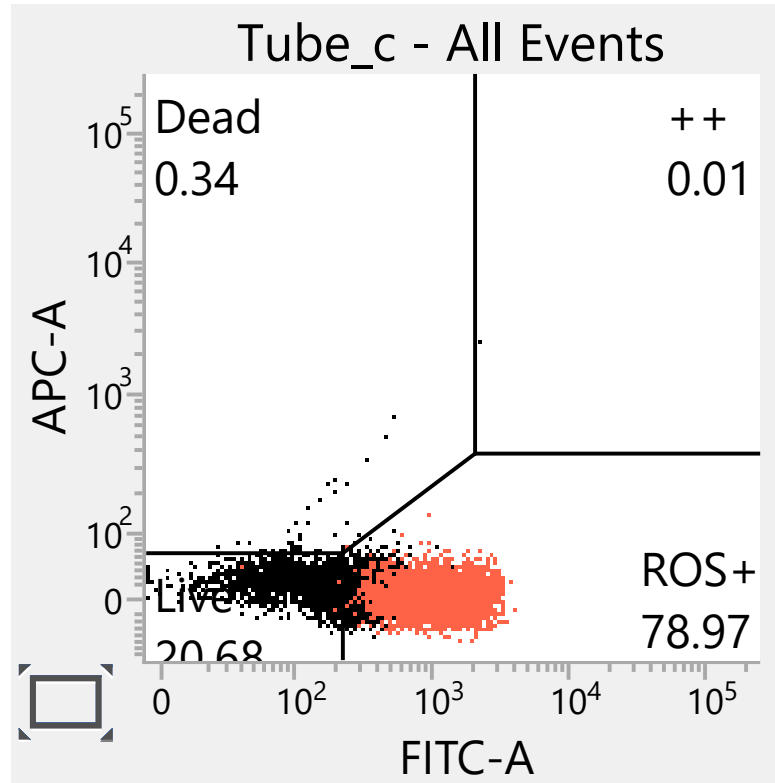

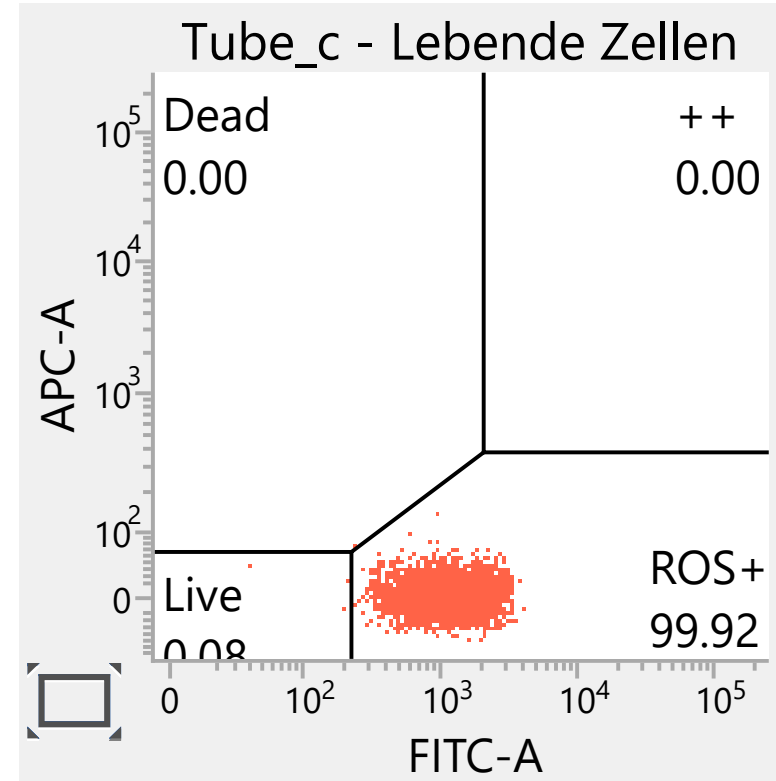

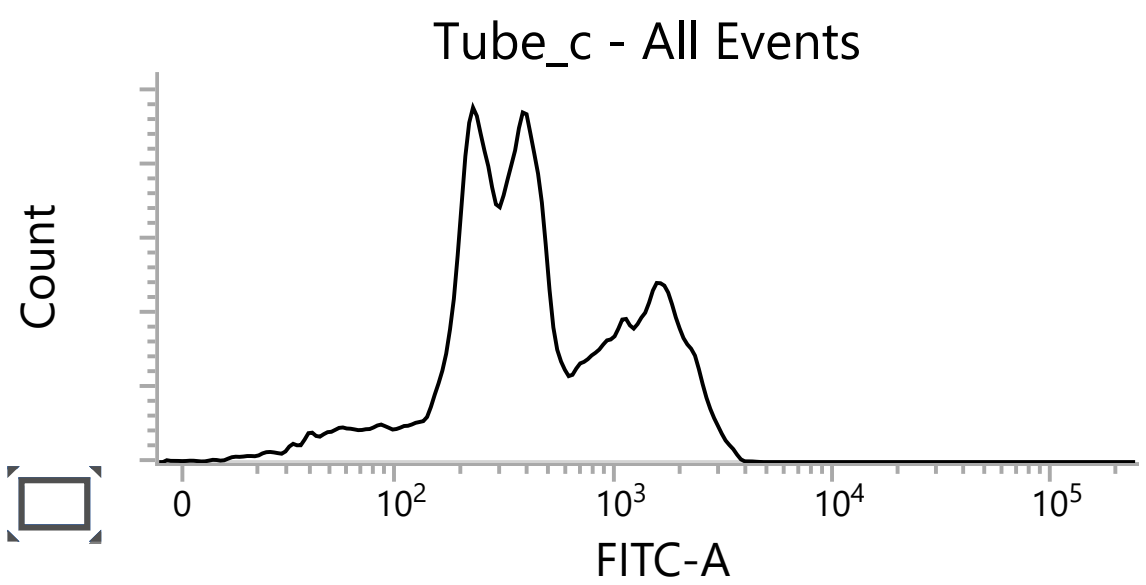

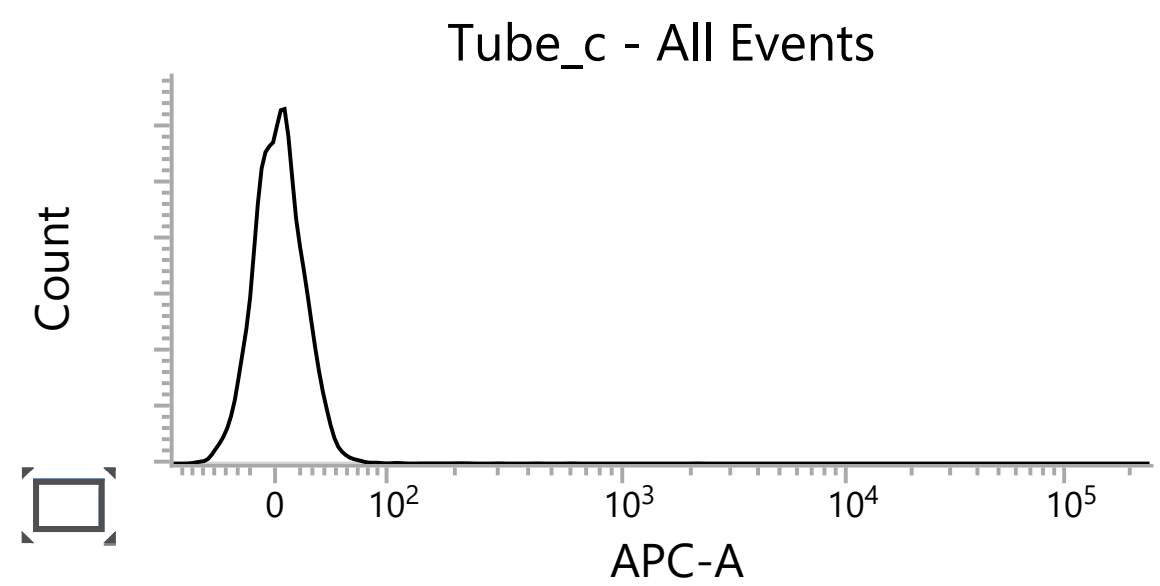

Statistics

Name	Events	% Parent	% Grandparent	% Total
Tube_a:All Events	10,000	***	***	100.00
Tube_a:Lebende Zellen	3,349	33.49	***	33.49
Tube_b:All Events	10,000	***	***	100.00
Tube_b:Lebende Zellen	3,329	33.29	***	33.29
Tube_c:All Events	10,000	***	***	100.00
Tube_c:Lebende Zellen	3,658	36.58	***	36.58

CellROX Assay

Sample ID: H2O2 4 + NAC

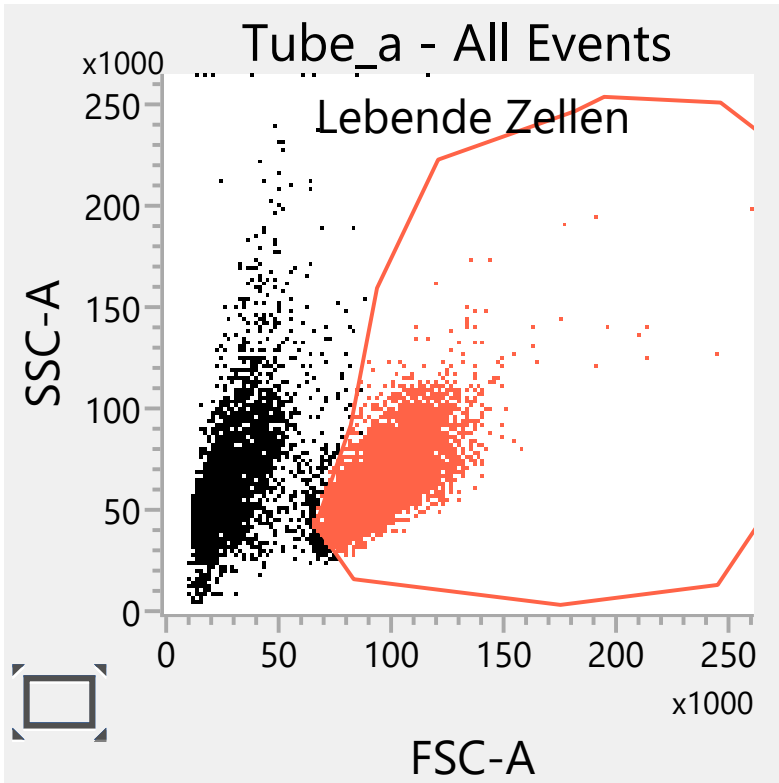

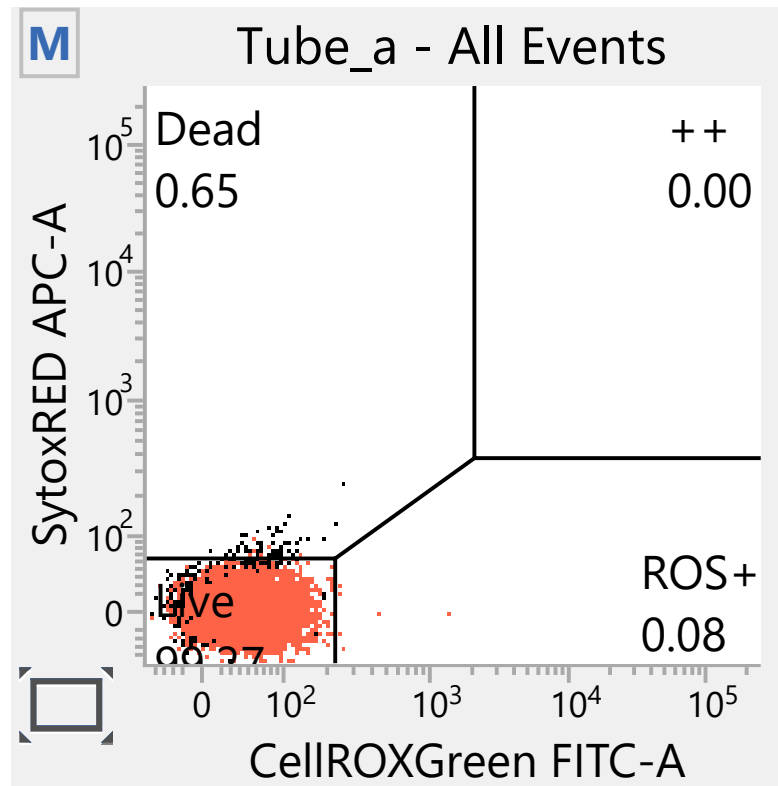

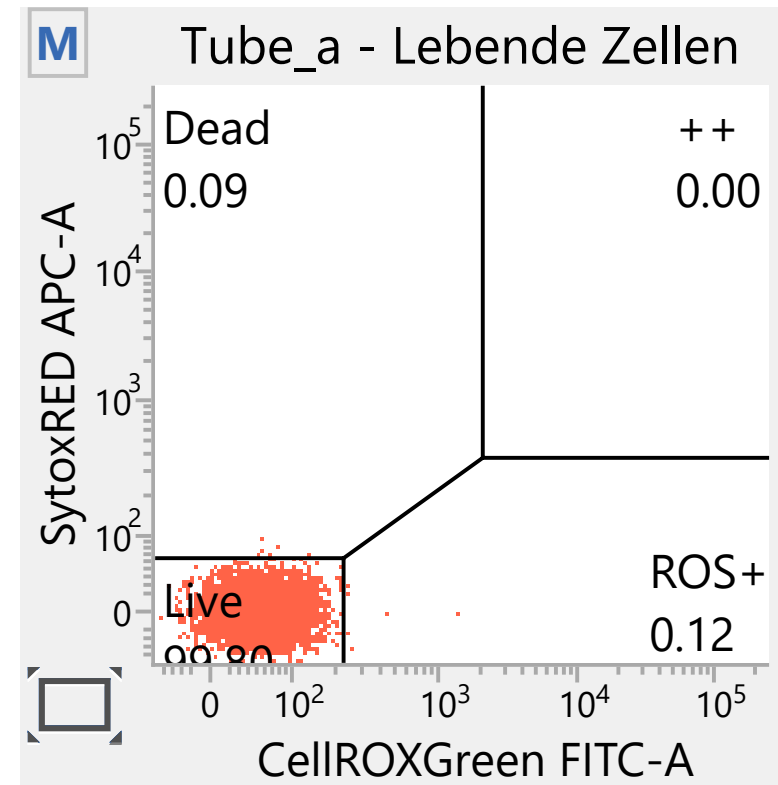

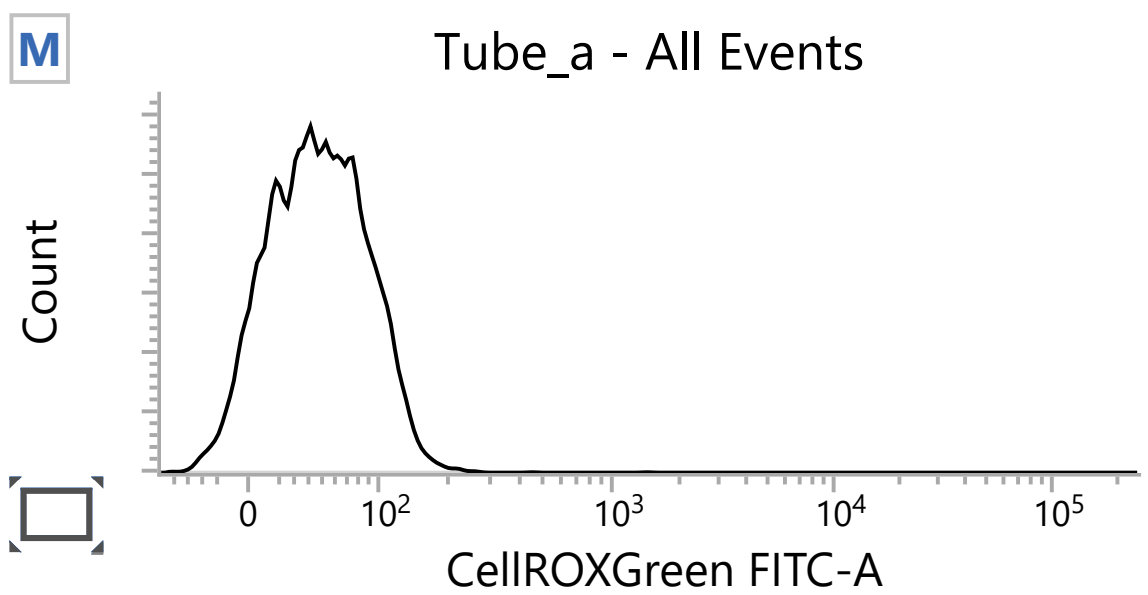

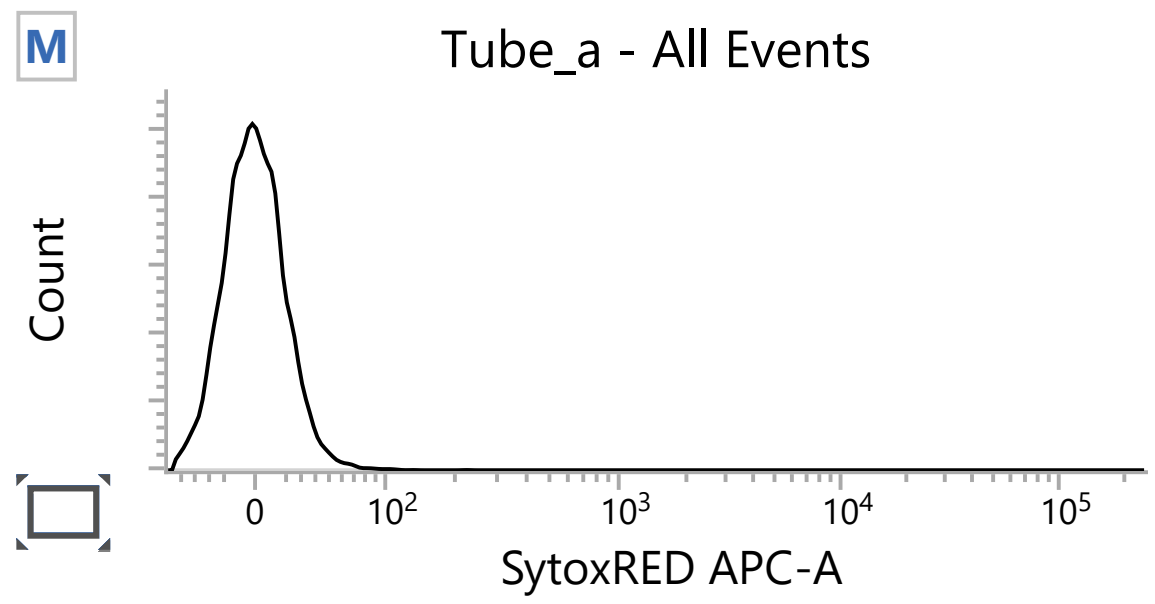

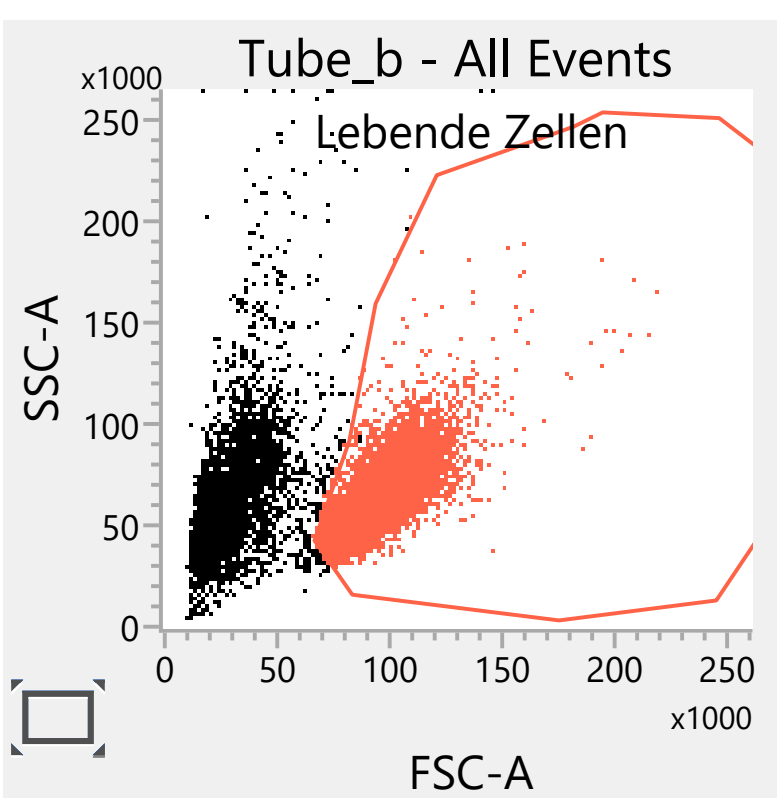

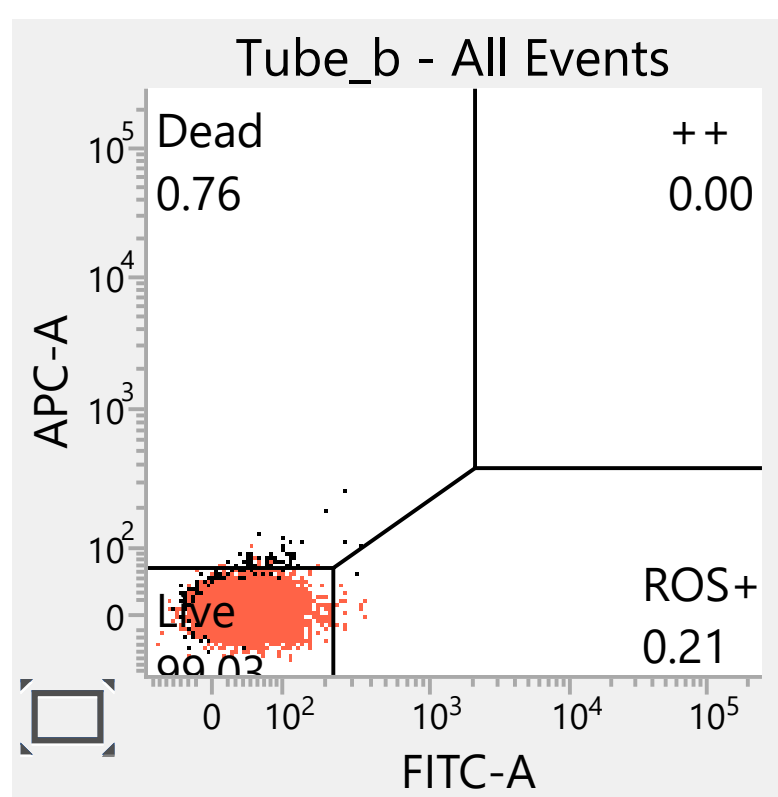

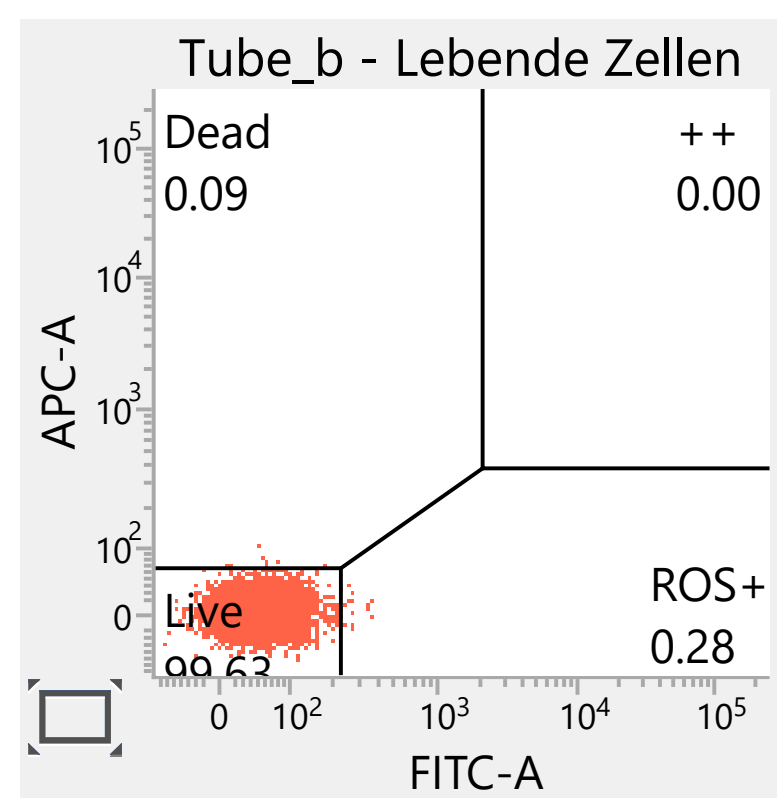

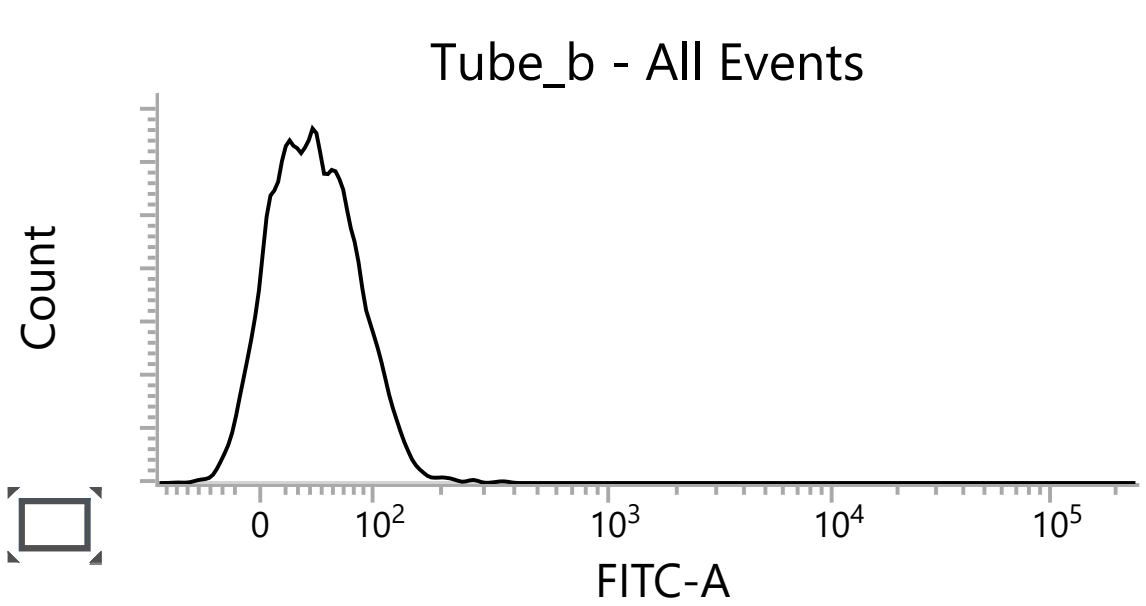

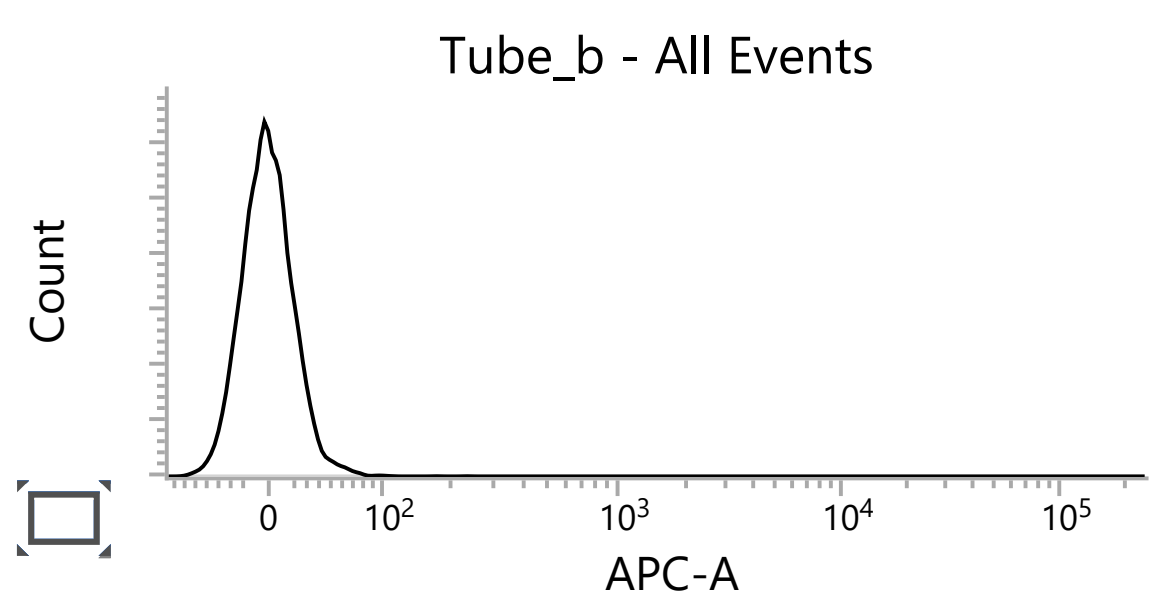

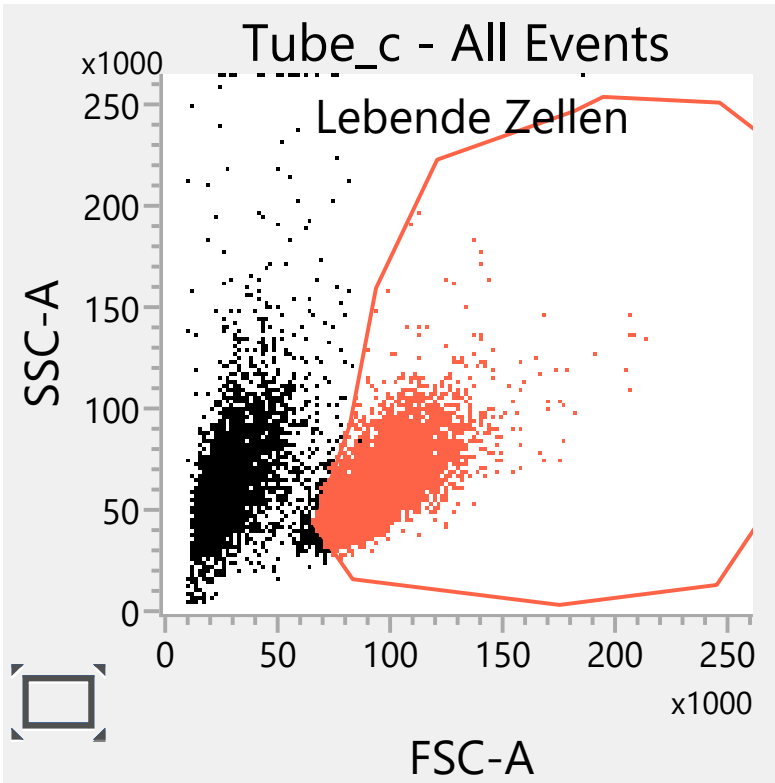

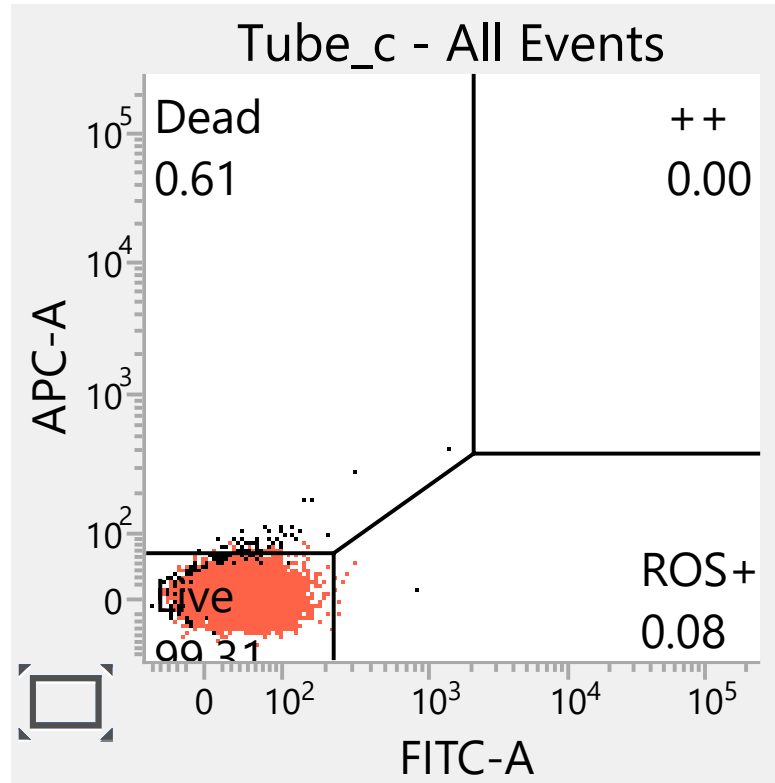

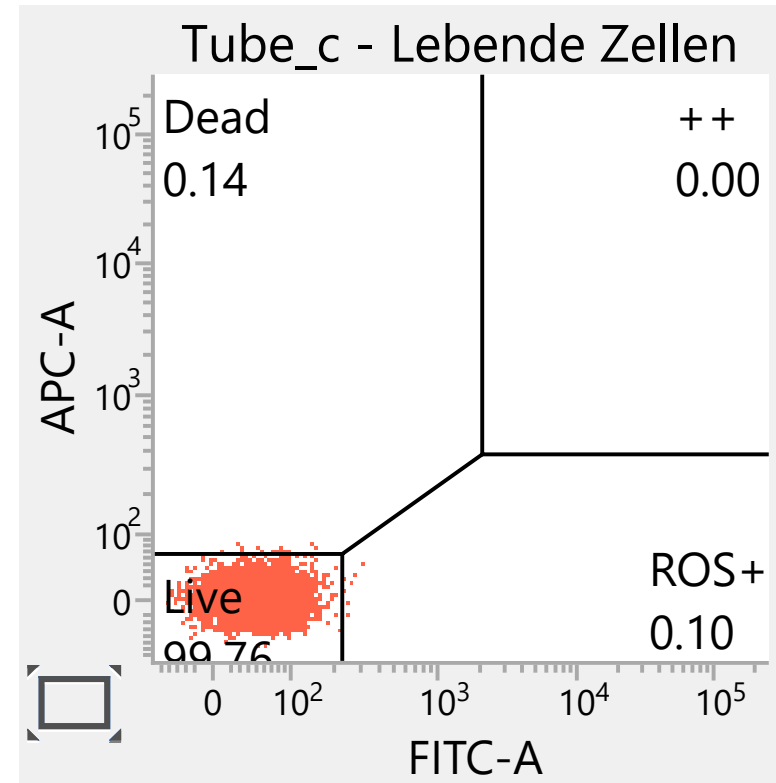

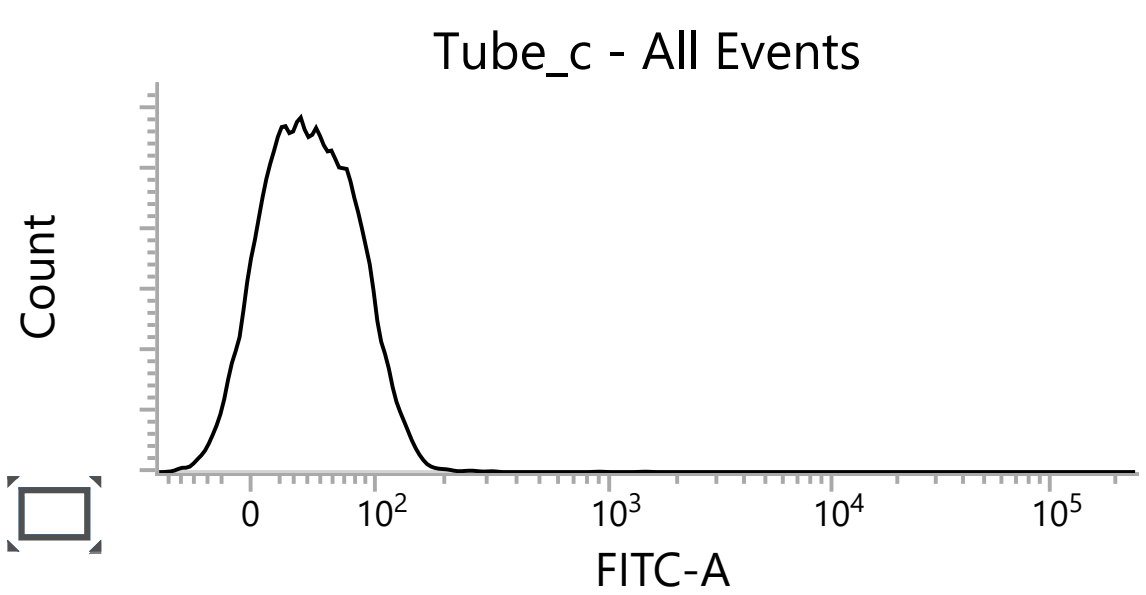

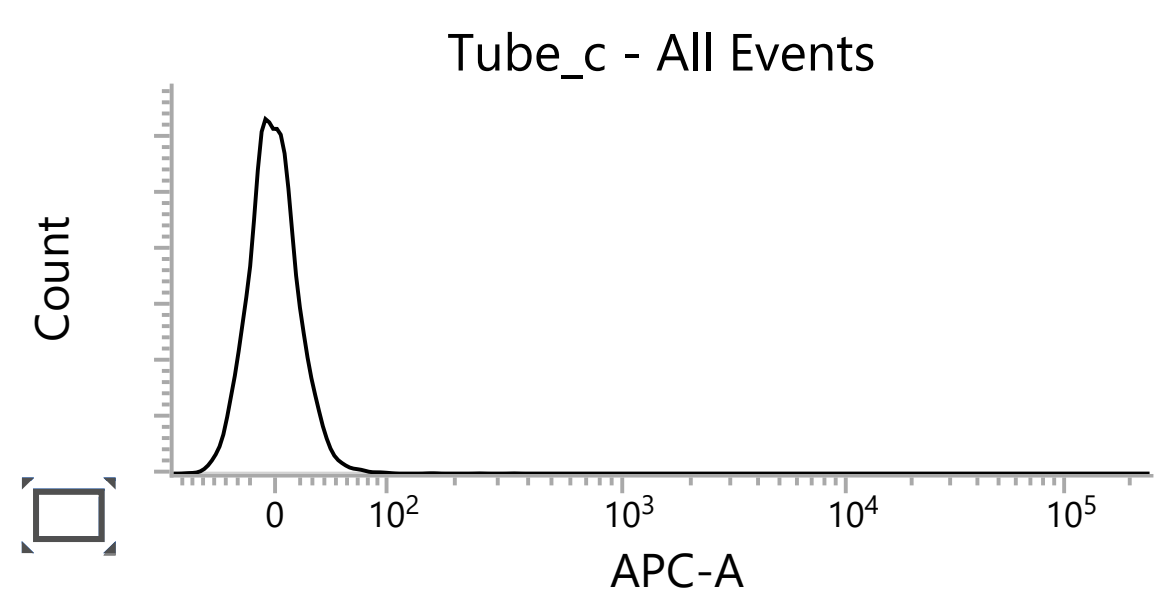

Statistics

Name	Events	% Parent	% Grandparent	% Total
Tube_a:All Events	10,000	***	***	100.00
Tube_a:Lebende Zellen	6,949	69.49	***	69.49
Tube_b:All Events	10,000	***	***	100.00
Tube_b:Lebende Zellen	6,848	68.48	***	68.48
Tube_c:All Events	10,000	***	***	100.00
Tube_c:Lebende Zellen	6,940	69.40	***	69.40

CellROX Assay

Sample ID: H2O2 4

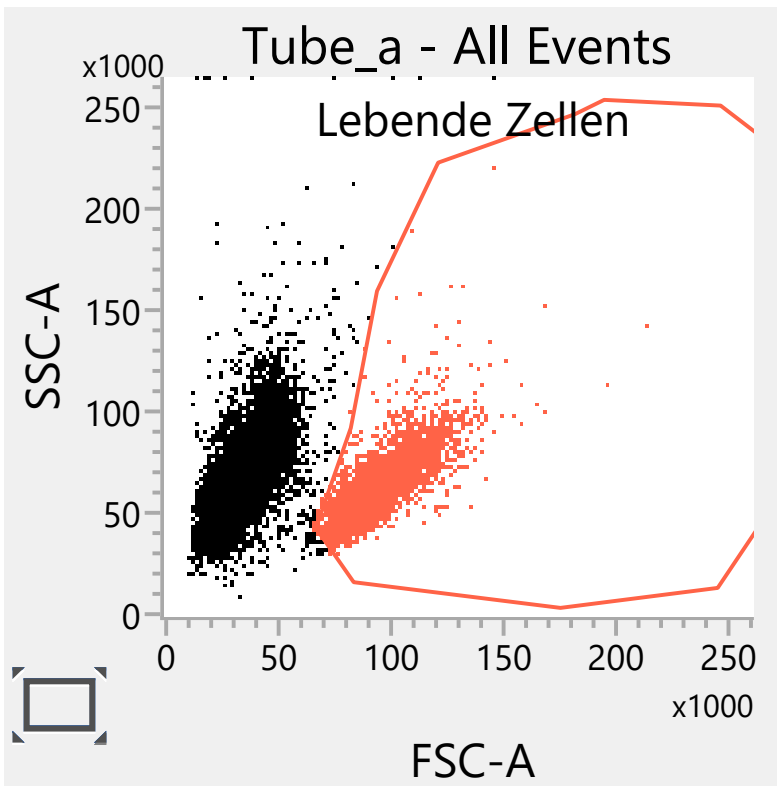

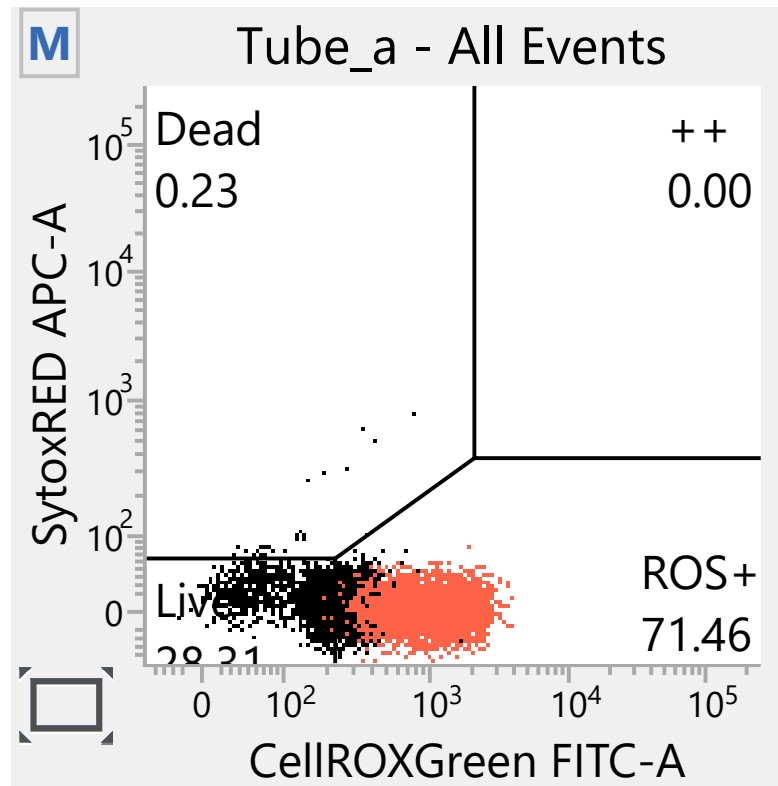

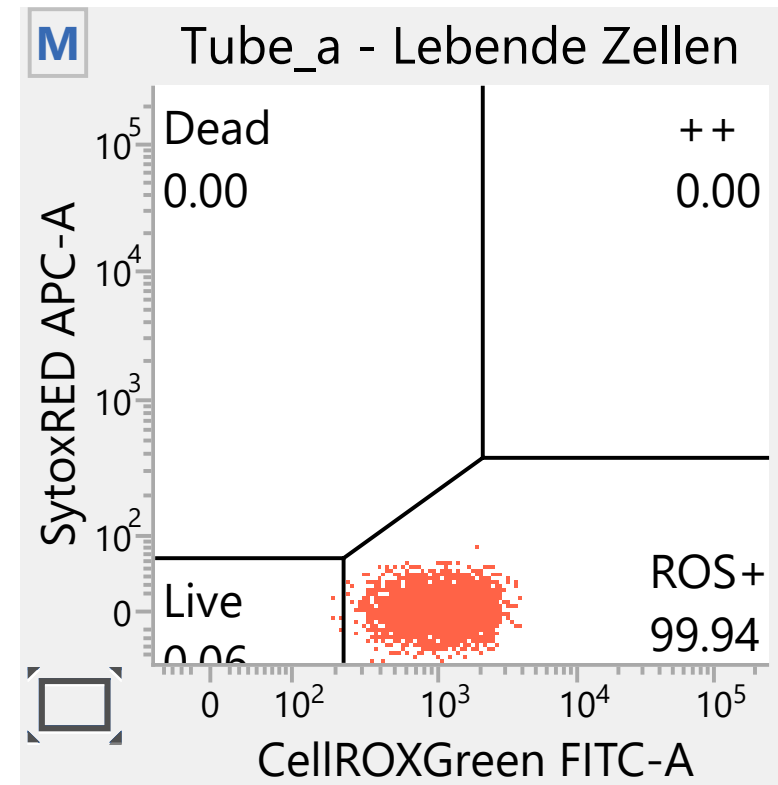

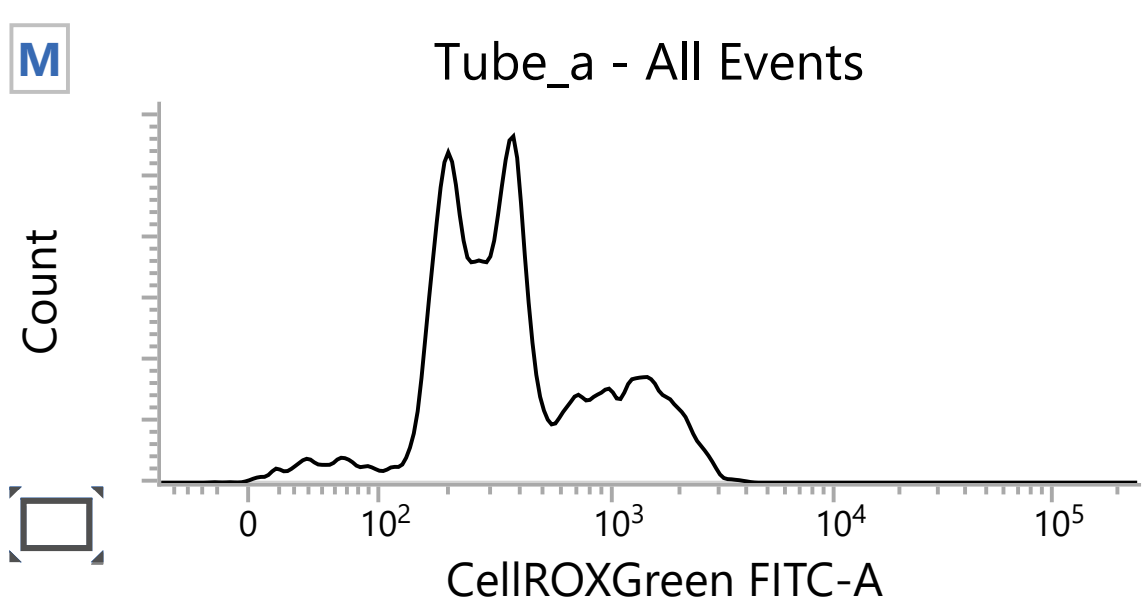

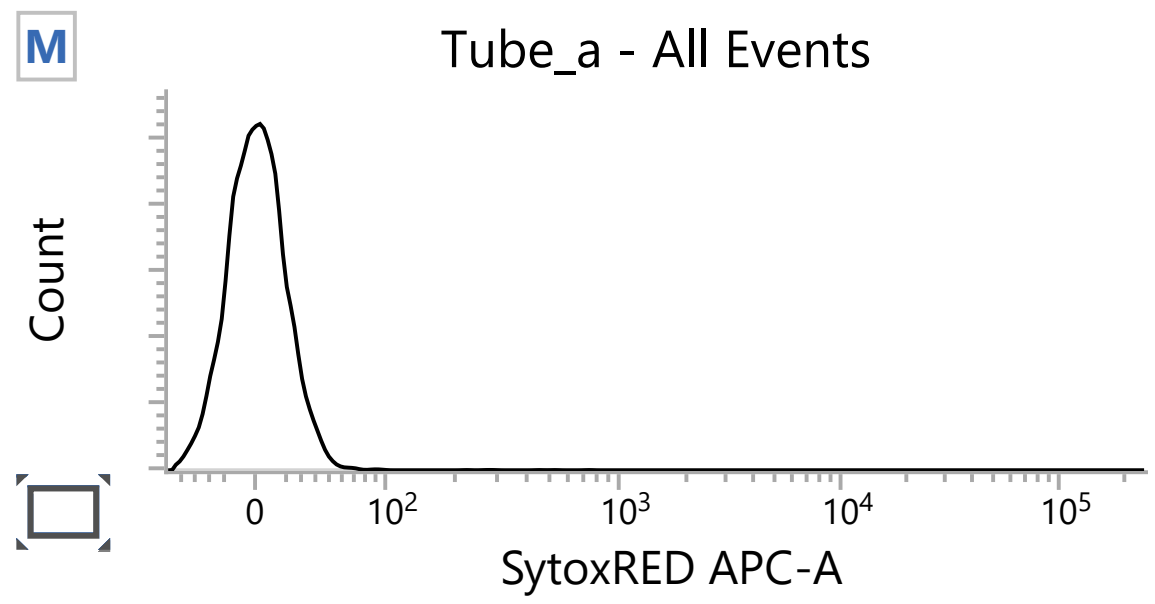

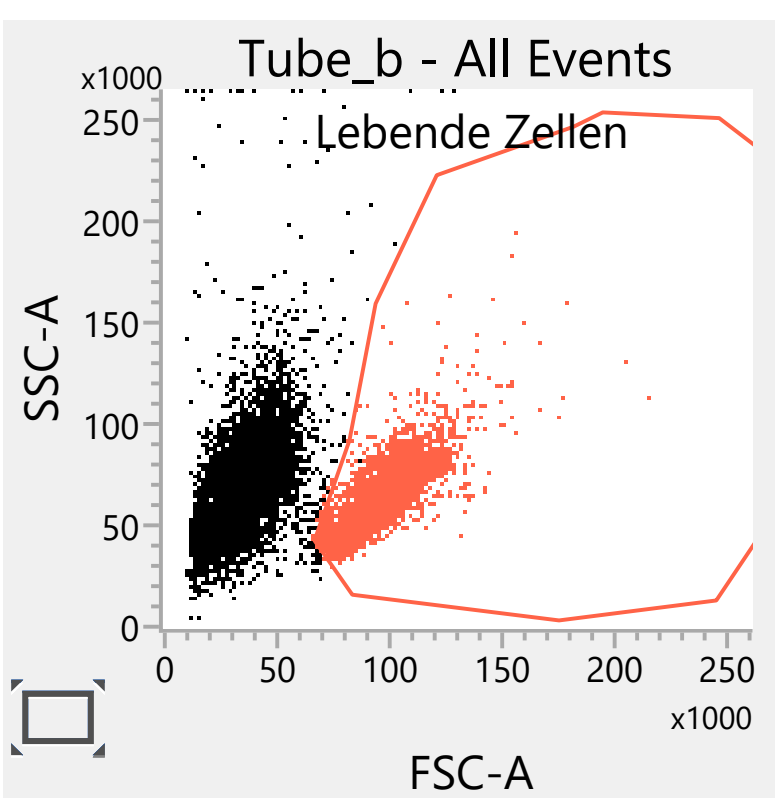

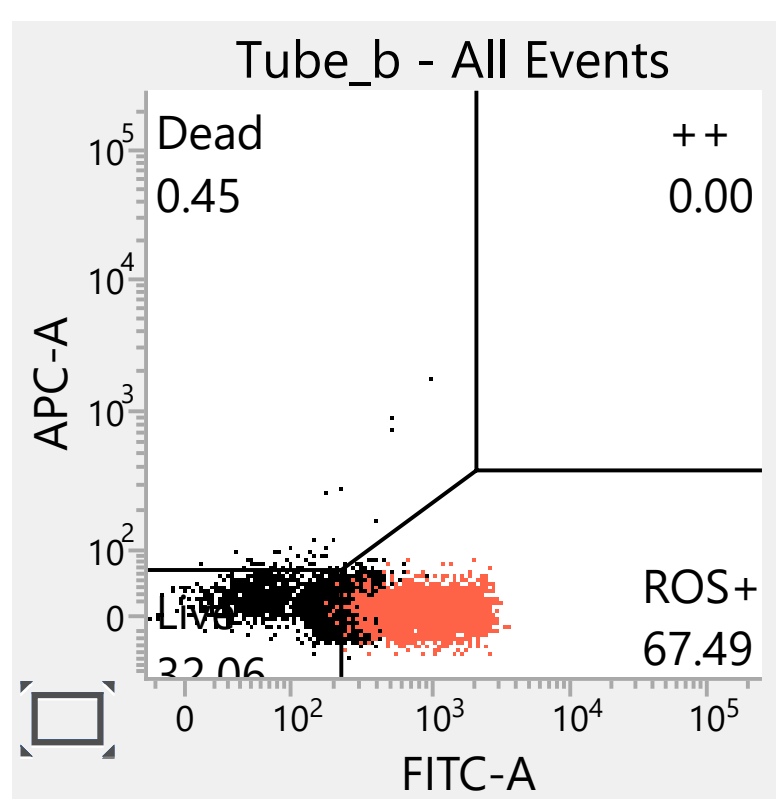

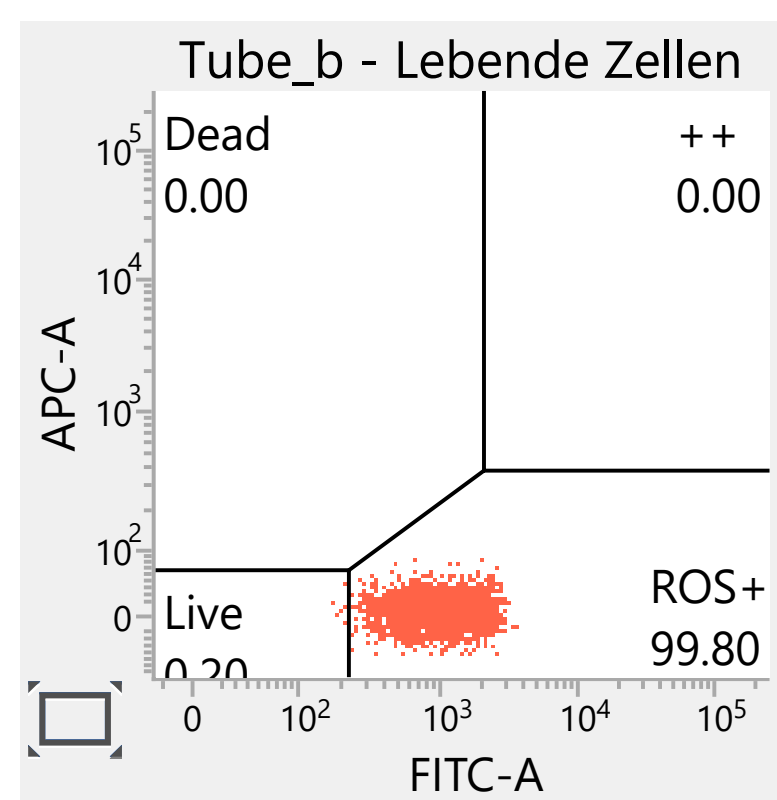

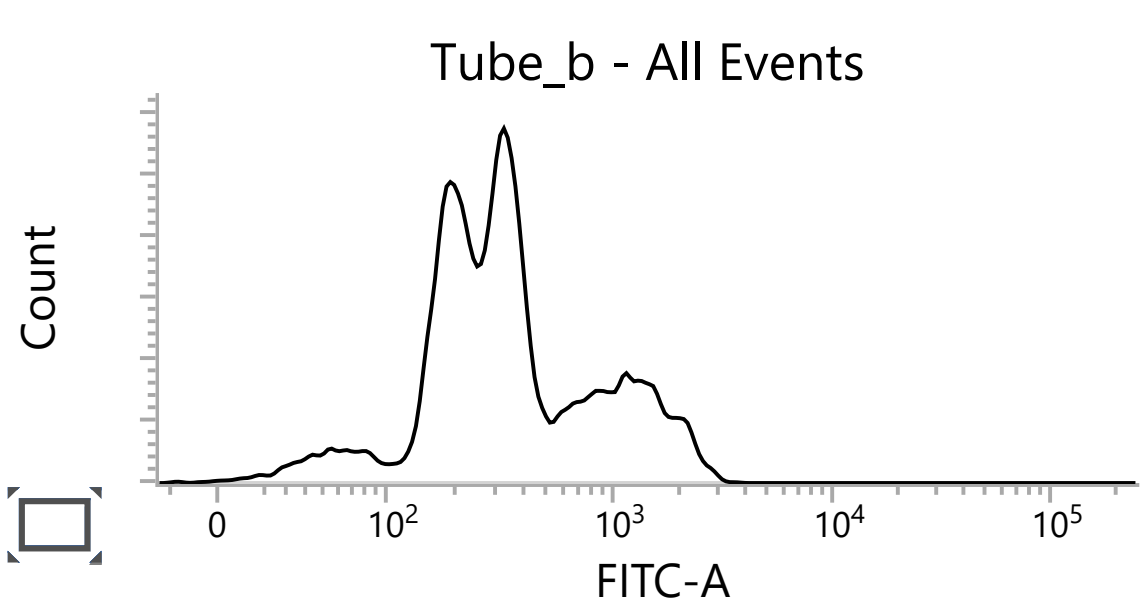

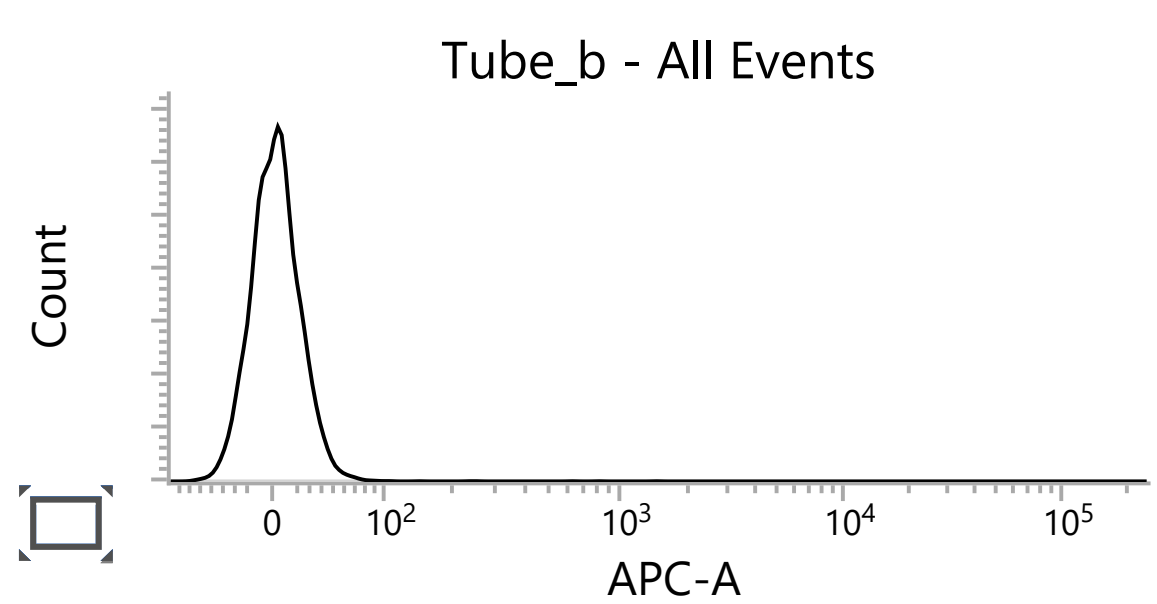

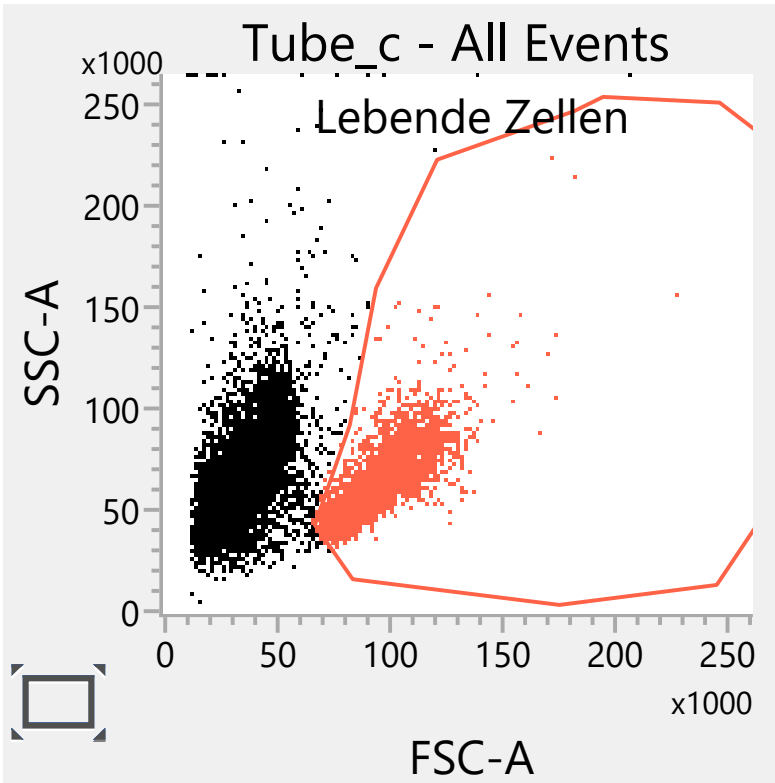

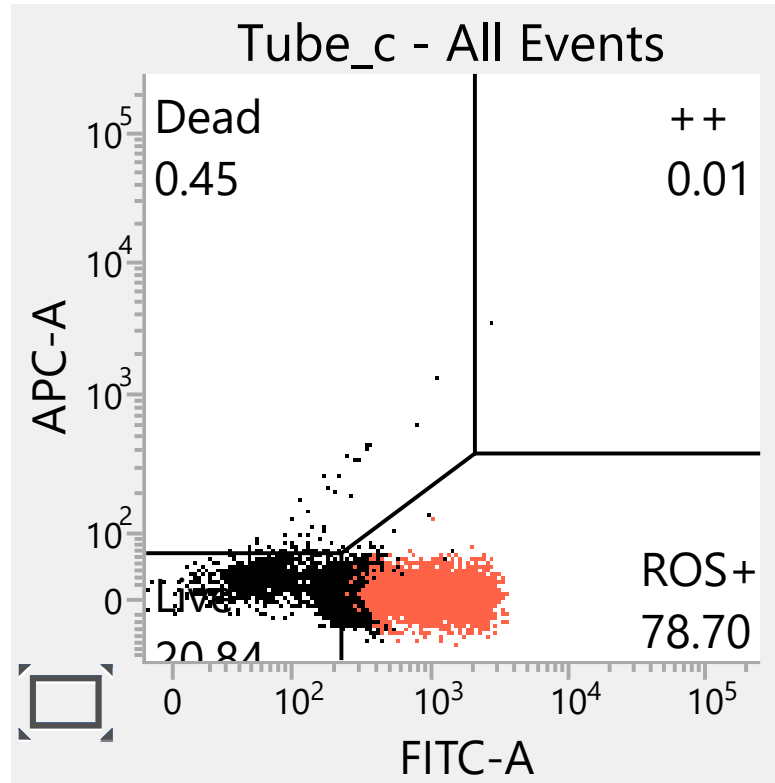

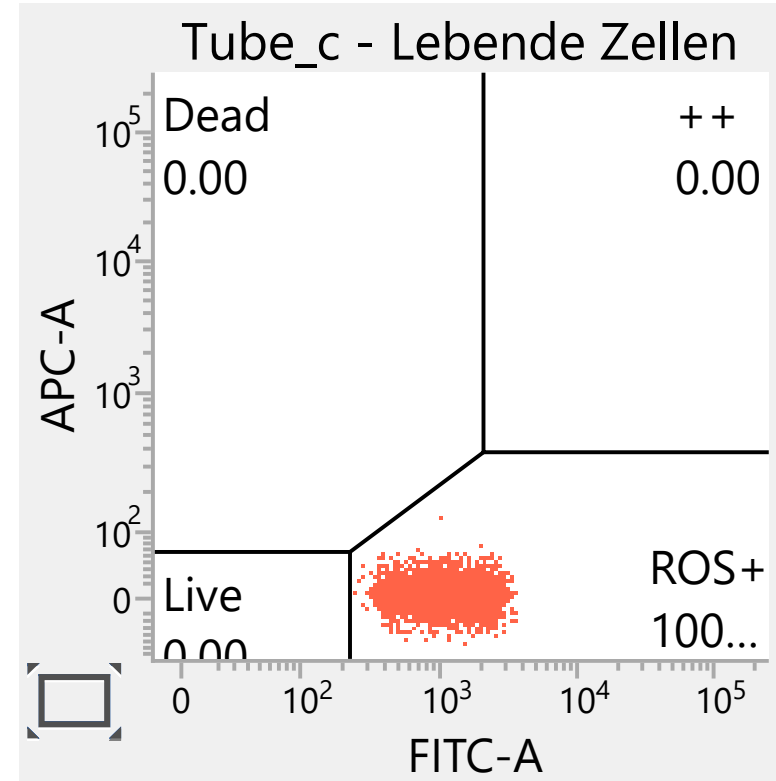

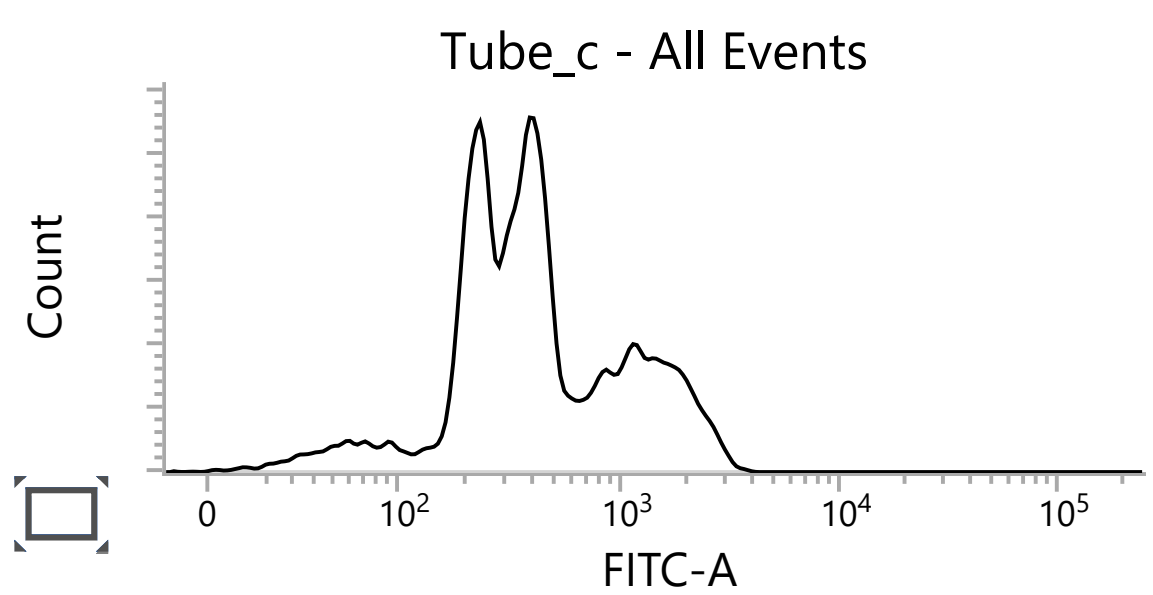

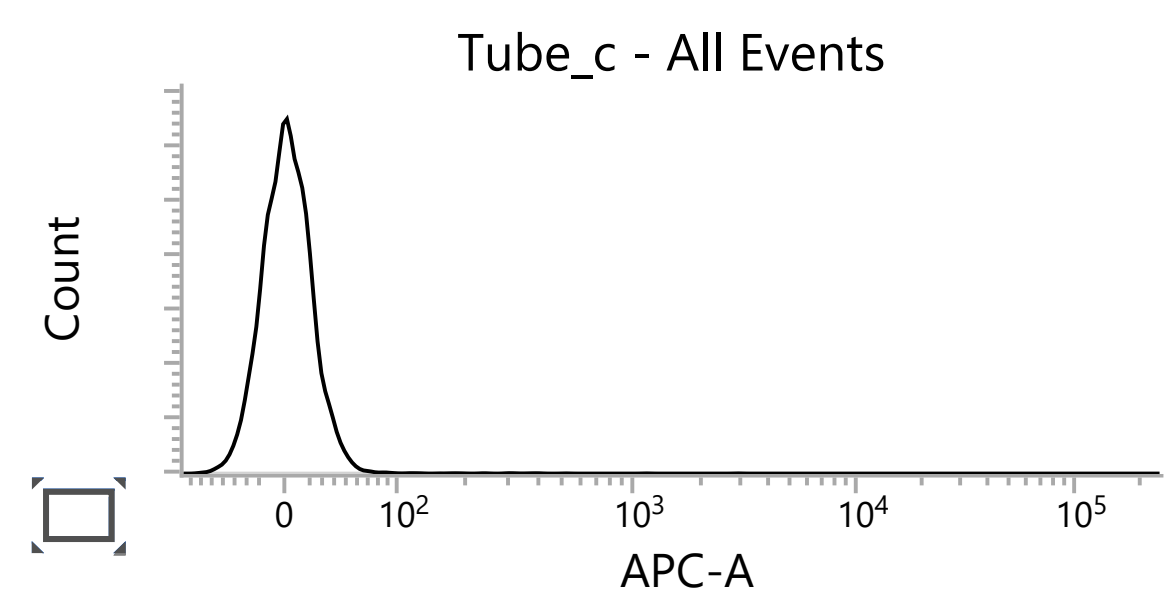

Statistics

Name	Events	% Parent	% Grandparent	% Total
Tube_a:All Events	10,000	***	***	100.00
Tube_a:Lebende Zellen	3,085	30.85	***	30.85
Tube_b:All Events	10,000	***	***	100.00
Tube_b:Lebende Zellen	3,005	30.05	***	30.05
Tube_c:All Events	10,000	***	***	100.00
Tube_c:Lebende Zellen	3,262	32.62	***	32.62

CellROX Assay

Sample ID: Kontrolle Nalm6

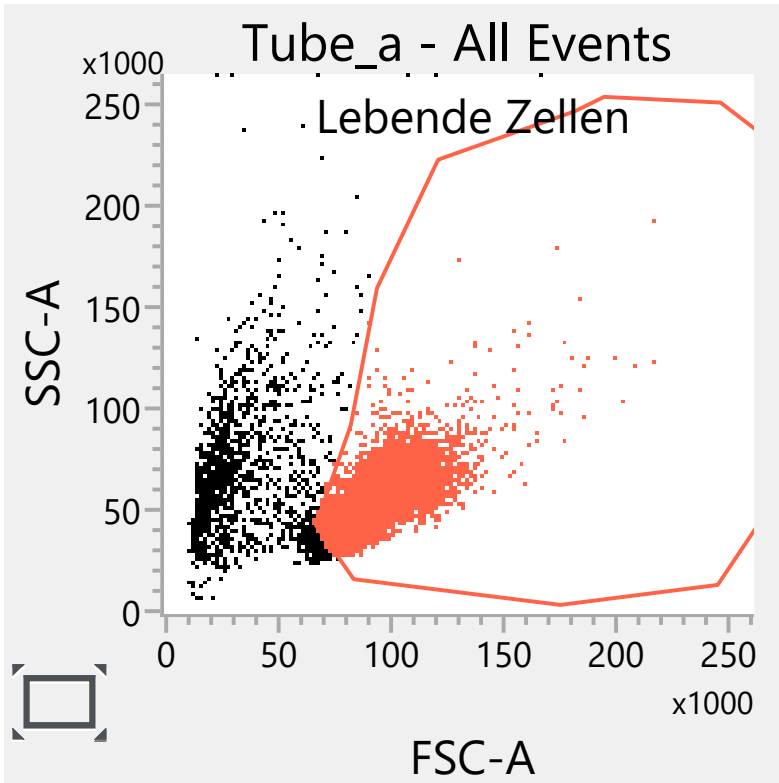

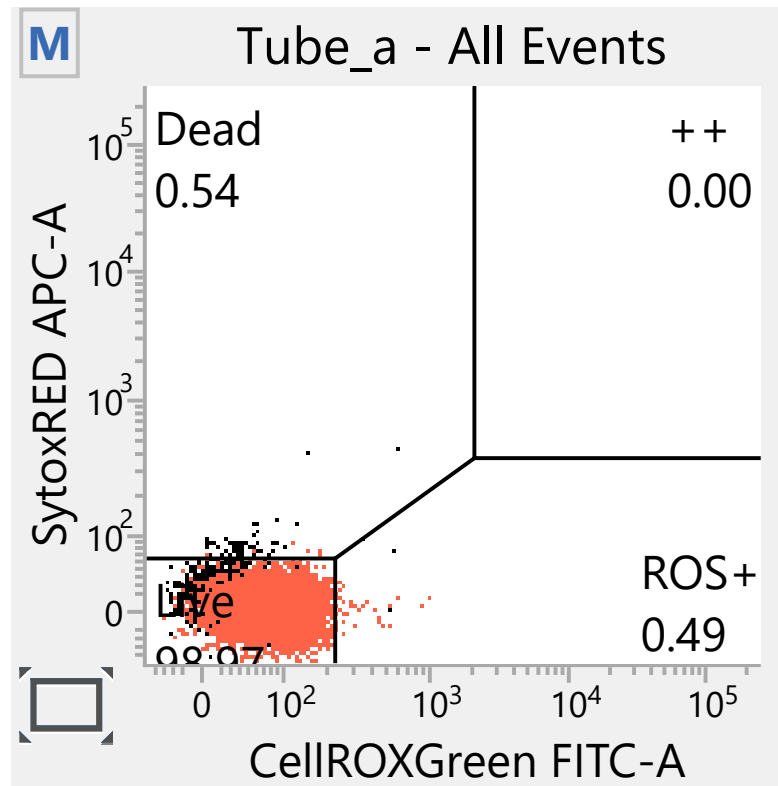

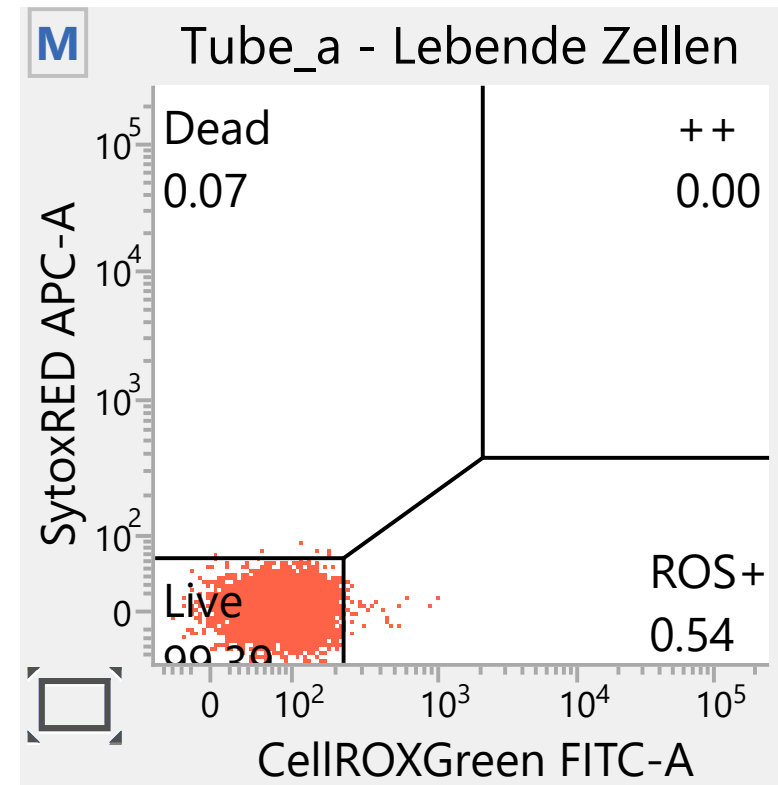

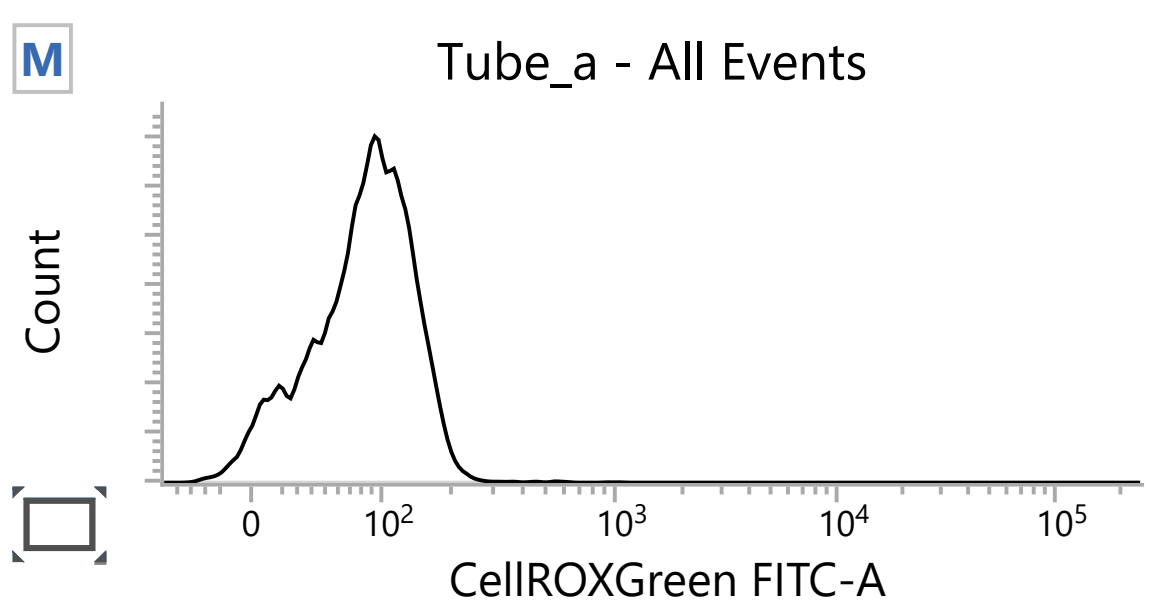

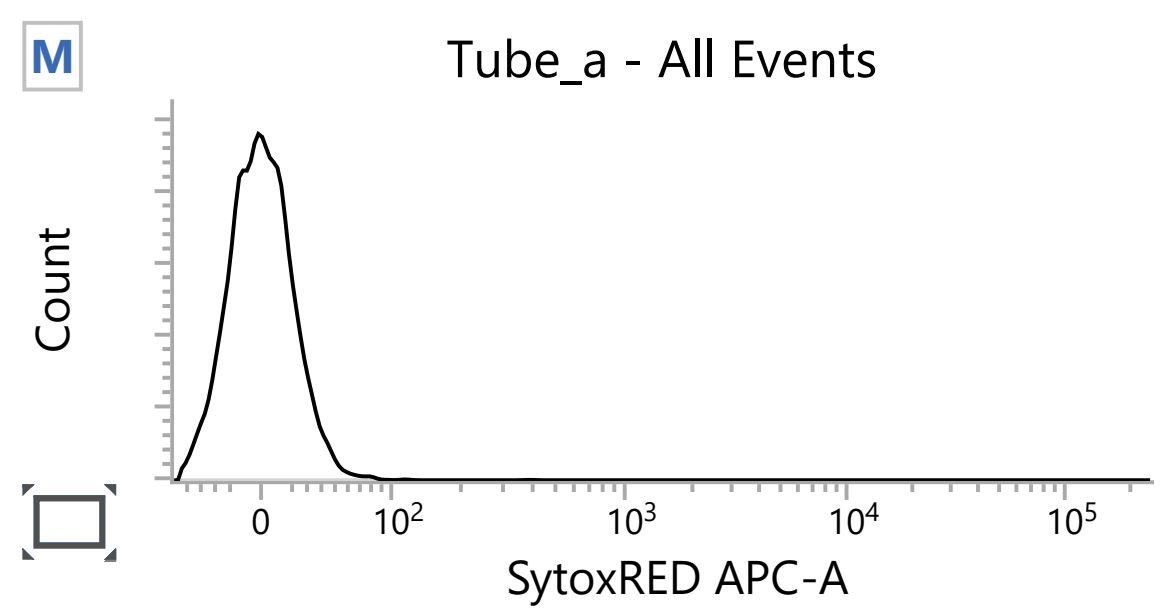

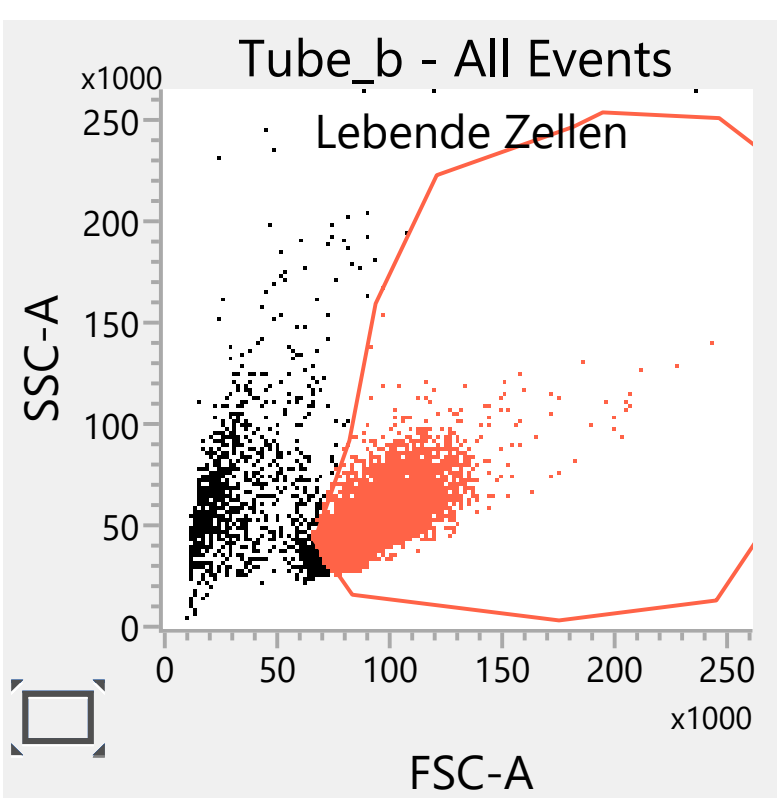

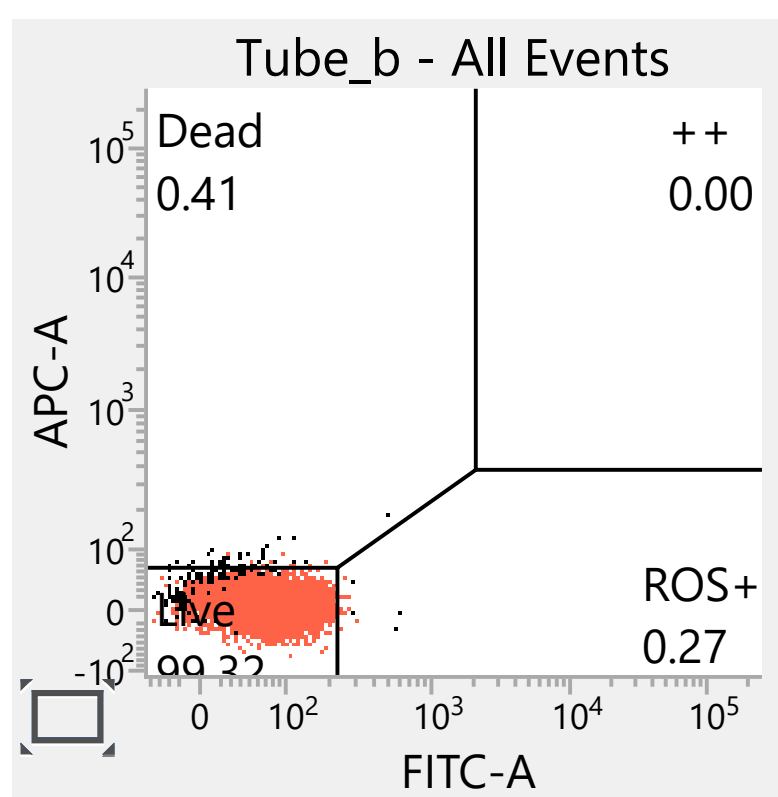

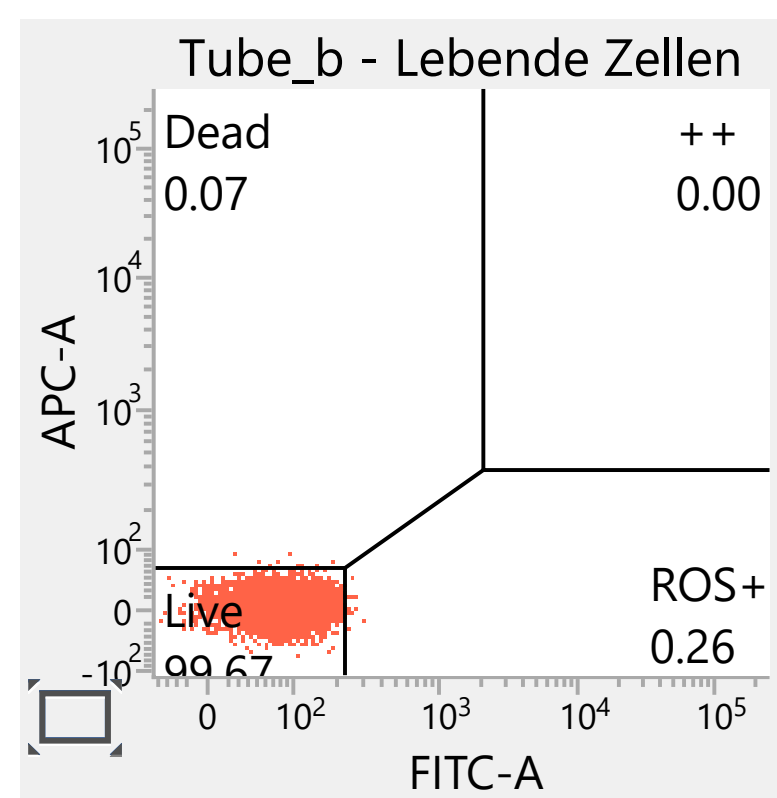

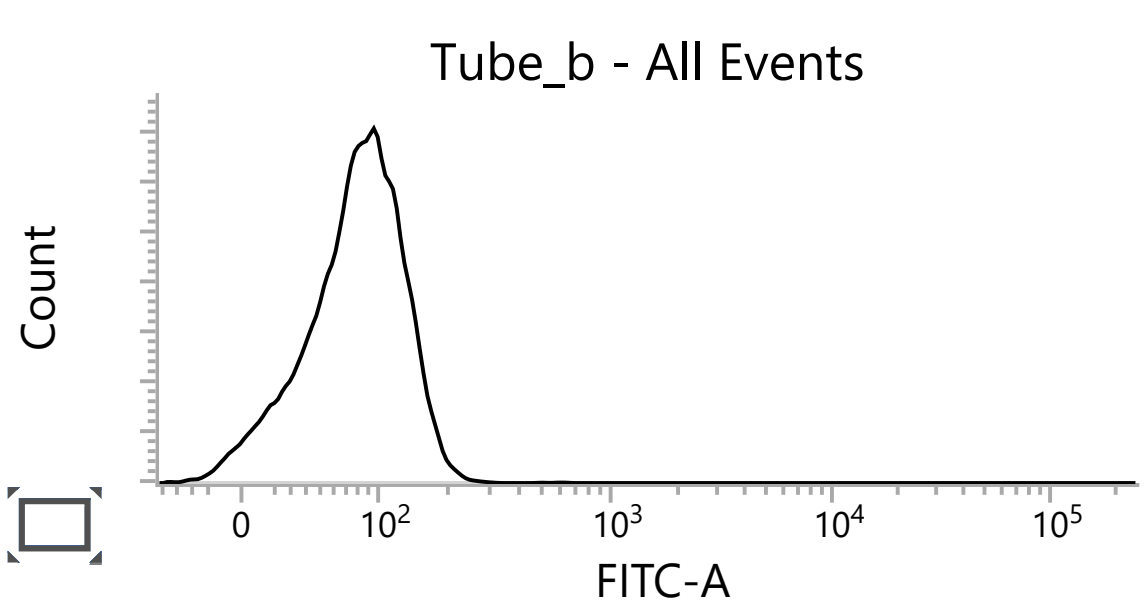

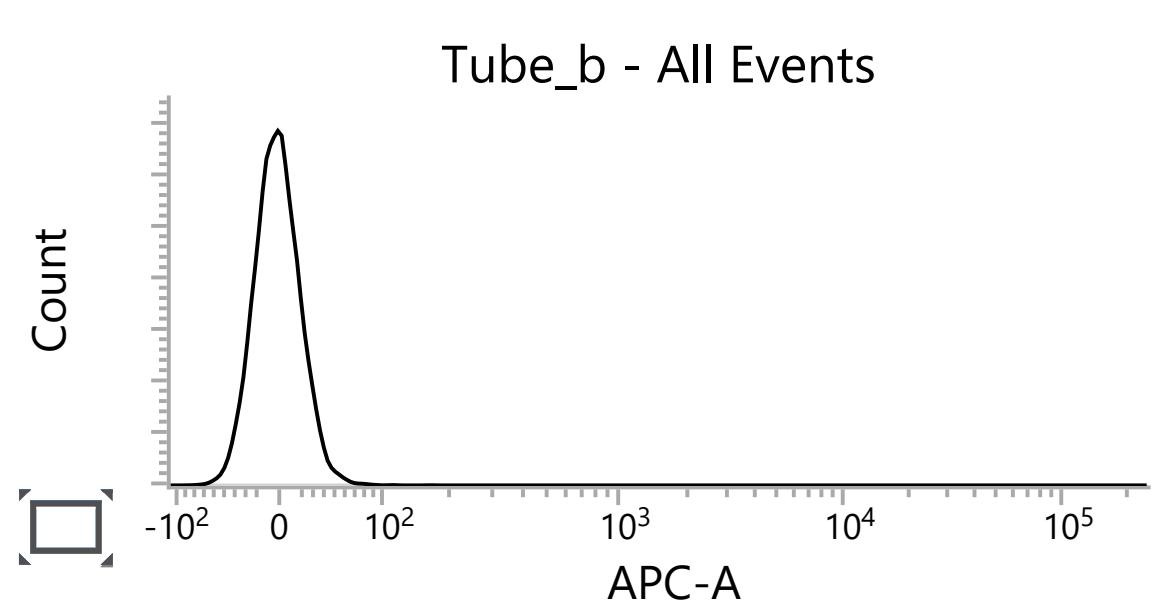

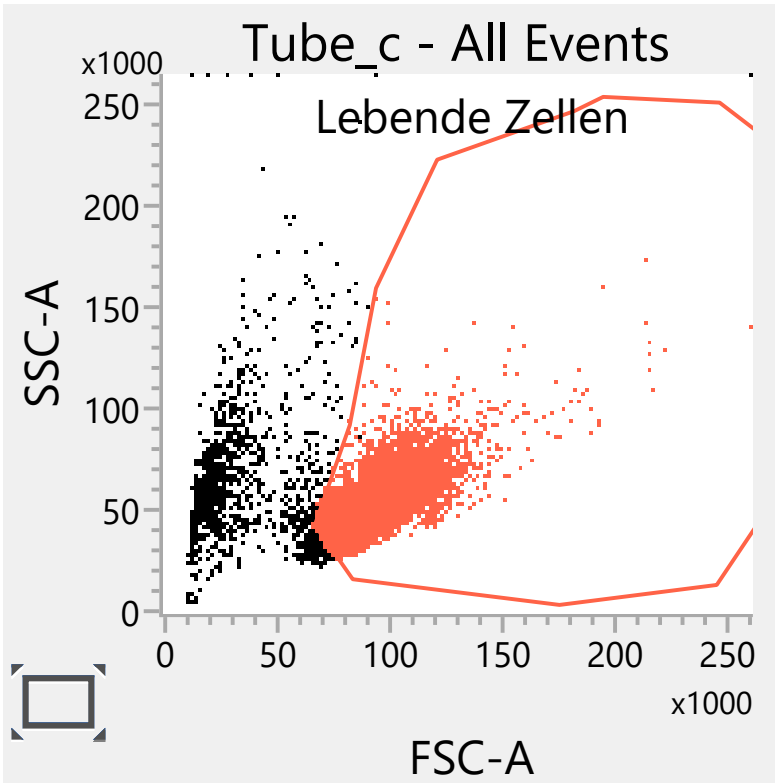

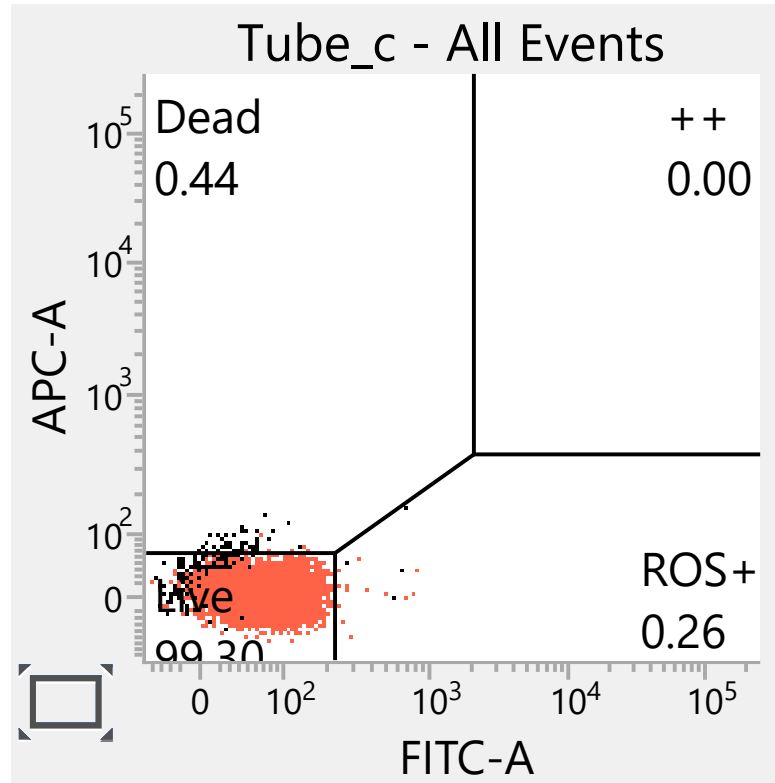

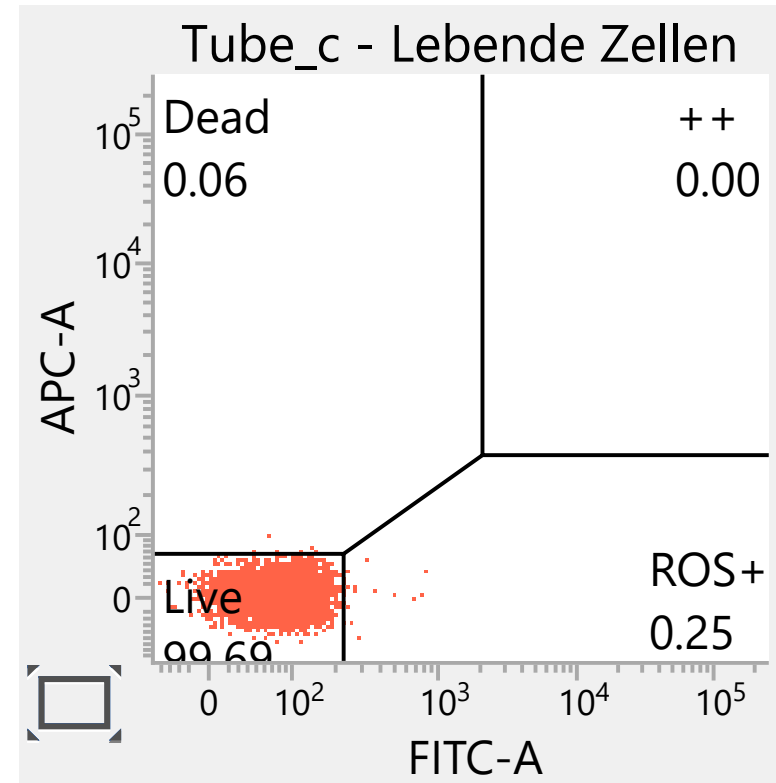

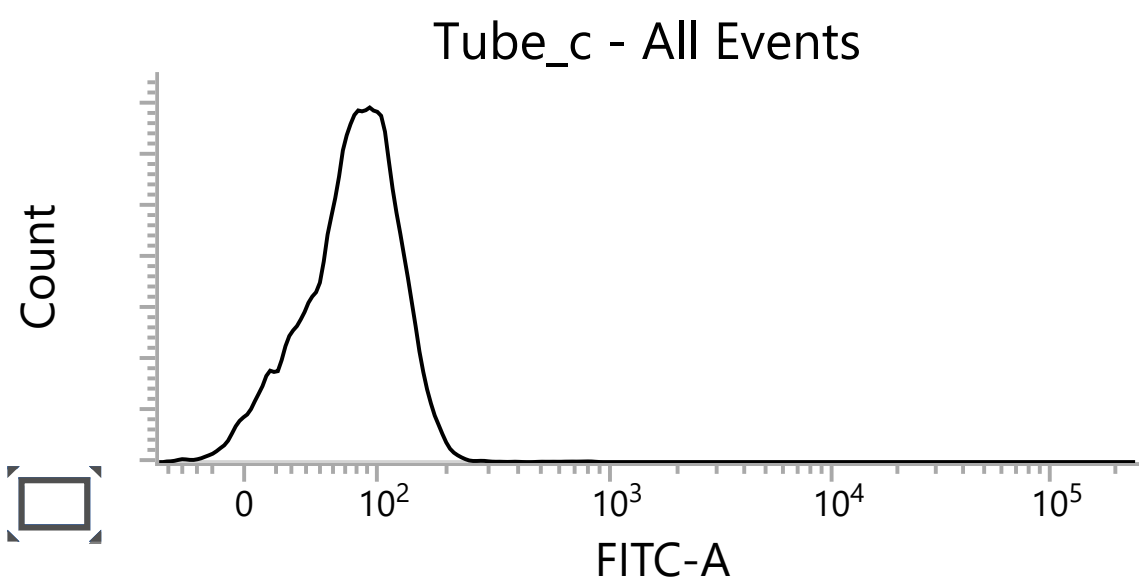

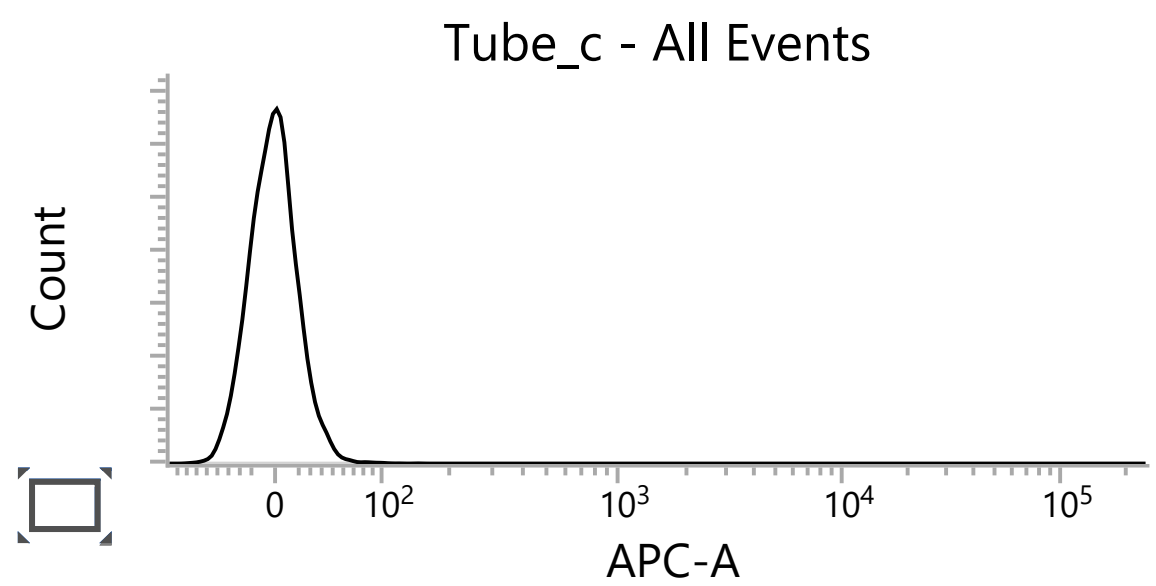

Statistics

Name	Events	% Parent	% Grandparent	% Total
Tube_a:All Events	10,000	***	***	100.00
Tube_a:Lebende Zellen	8,315	83.15	***	83.15
Tube_b:All Events	10,000	***	***	100.00
Tube_b:Lebende Zellen	8,688	86.88	***	86.88
Tube_c:All Events	10,000	***	***	100.00
Tube_c:Lebende Zellen	8,628	86.28	***	86.28

CellROX Assay

Sample ID: NAC

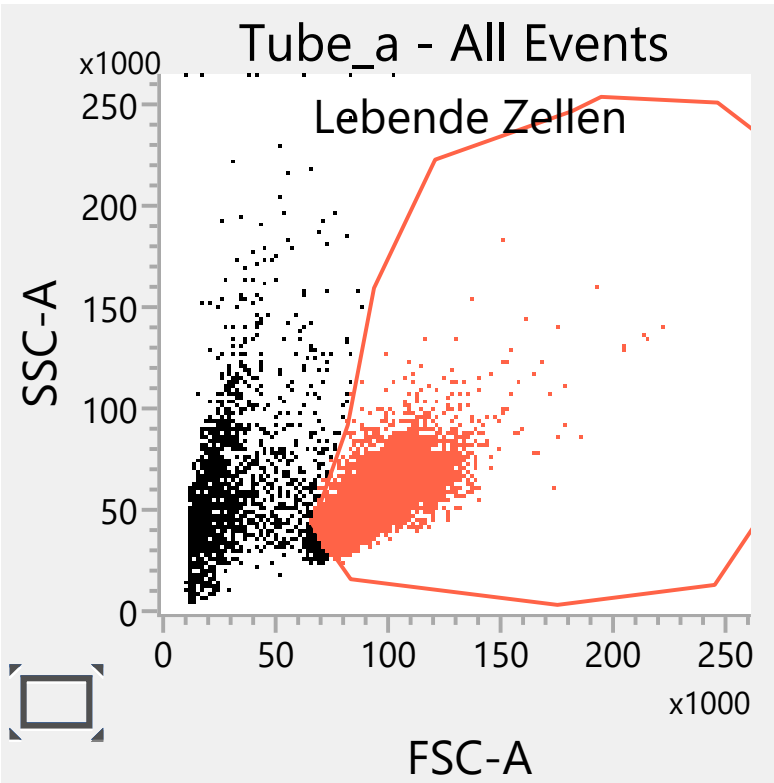

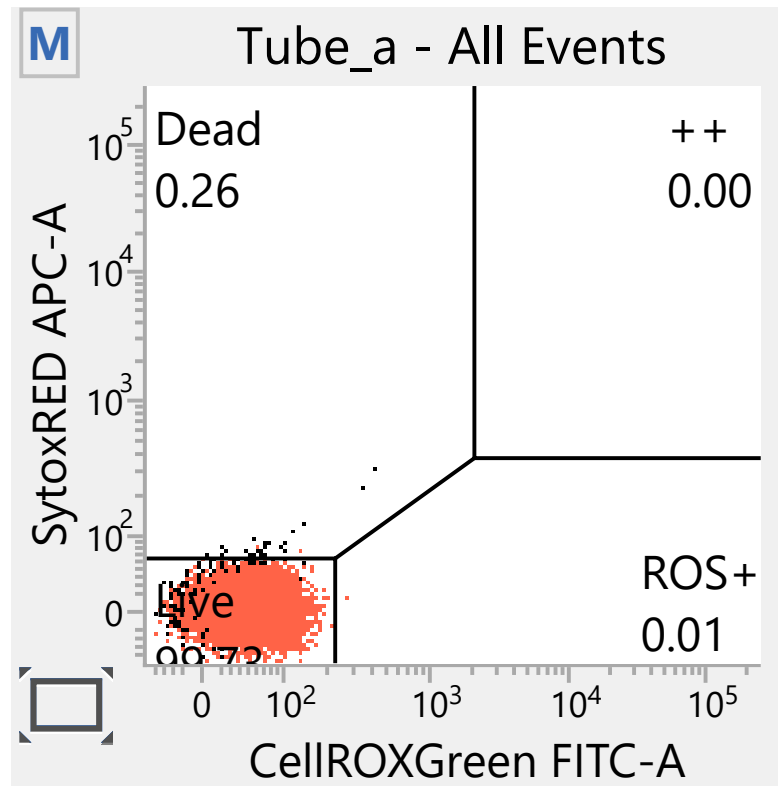

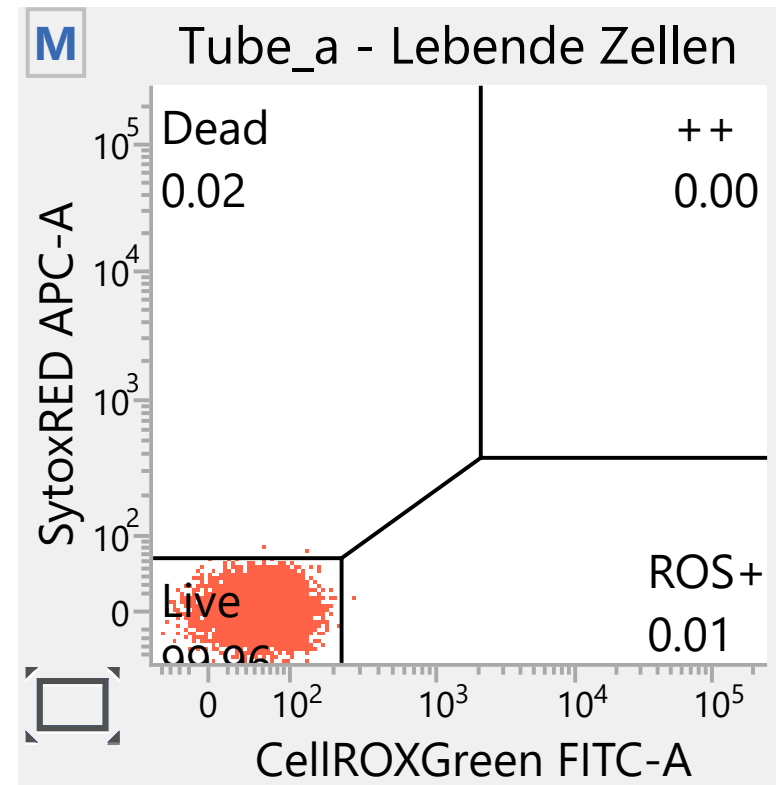

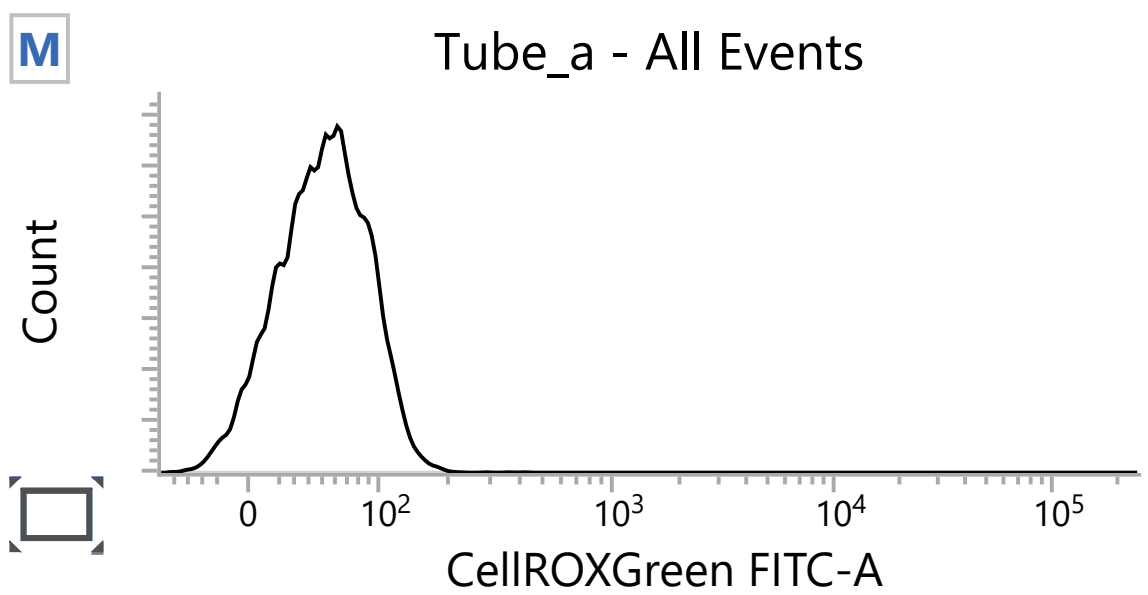

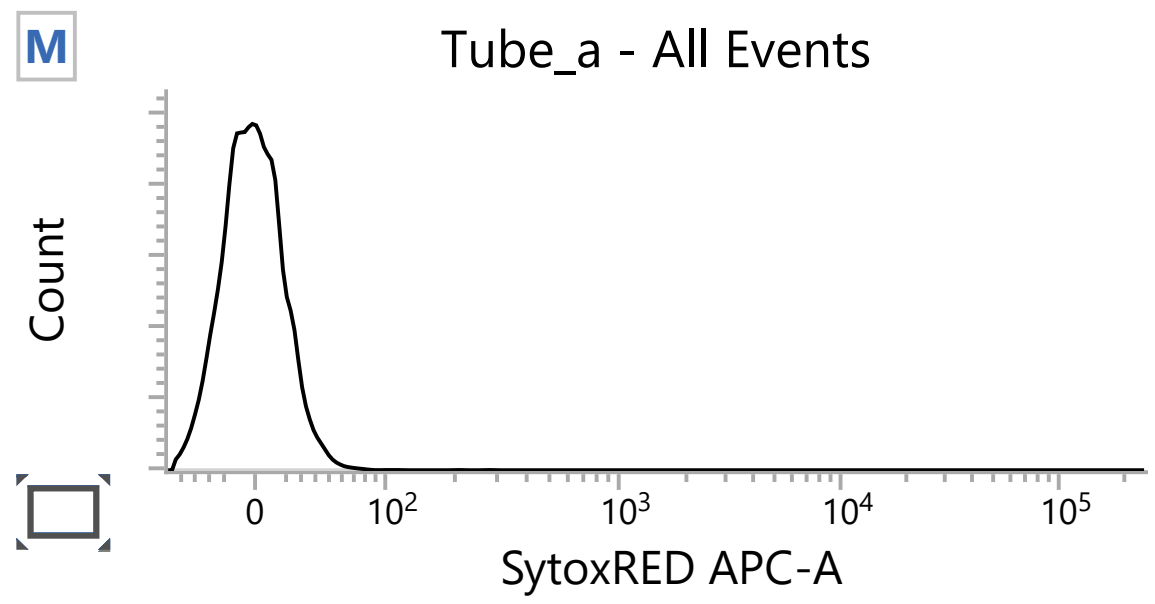

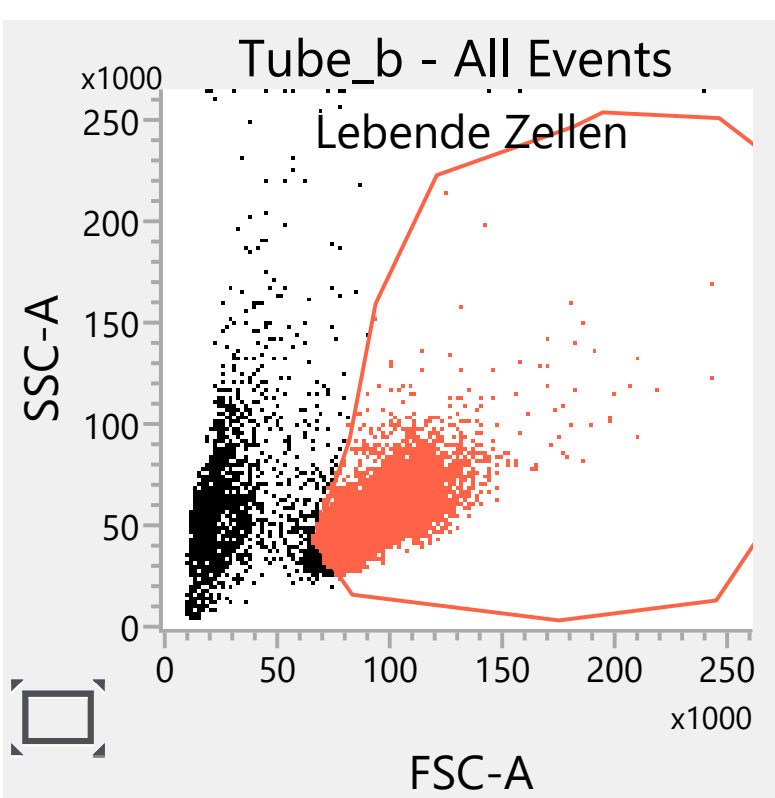

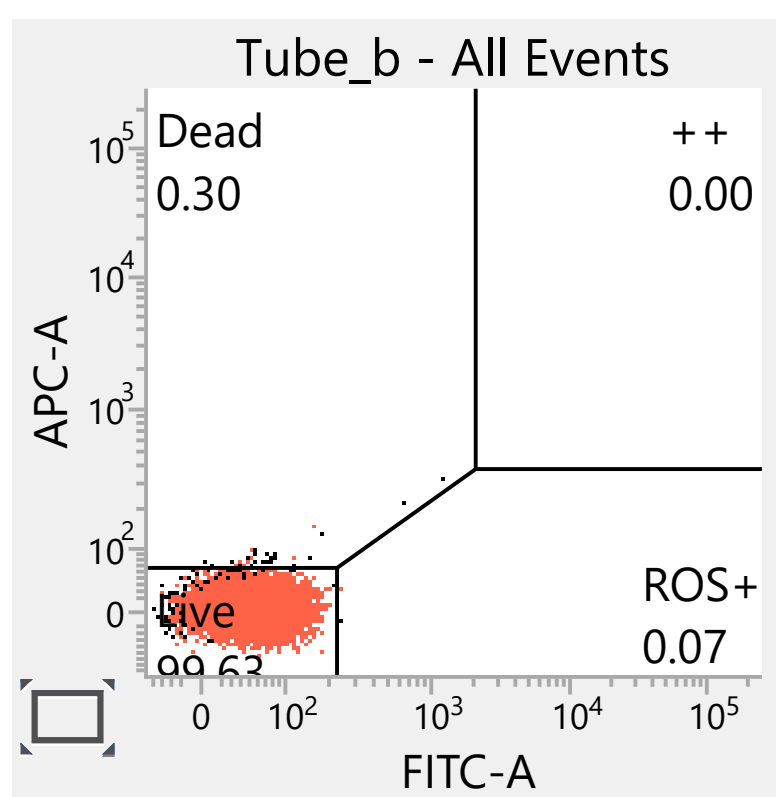

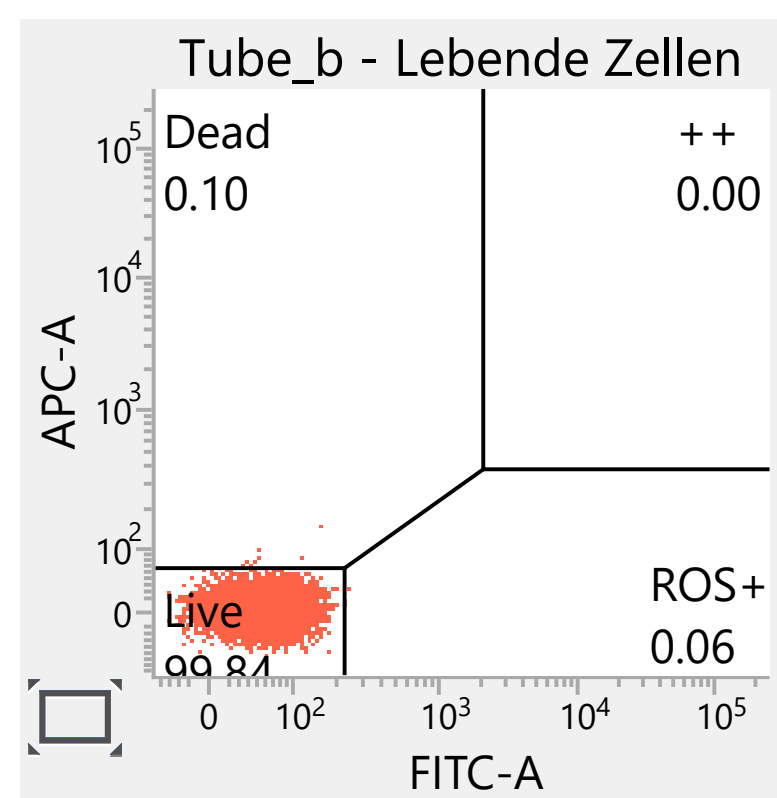

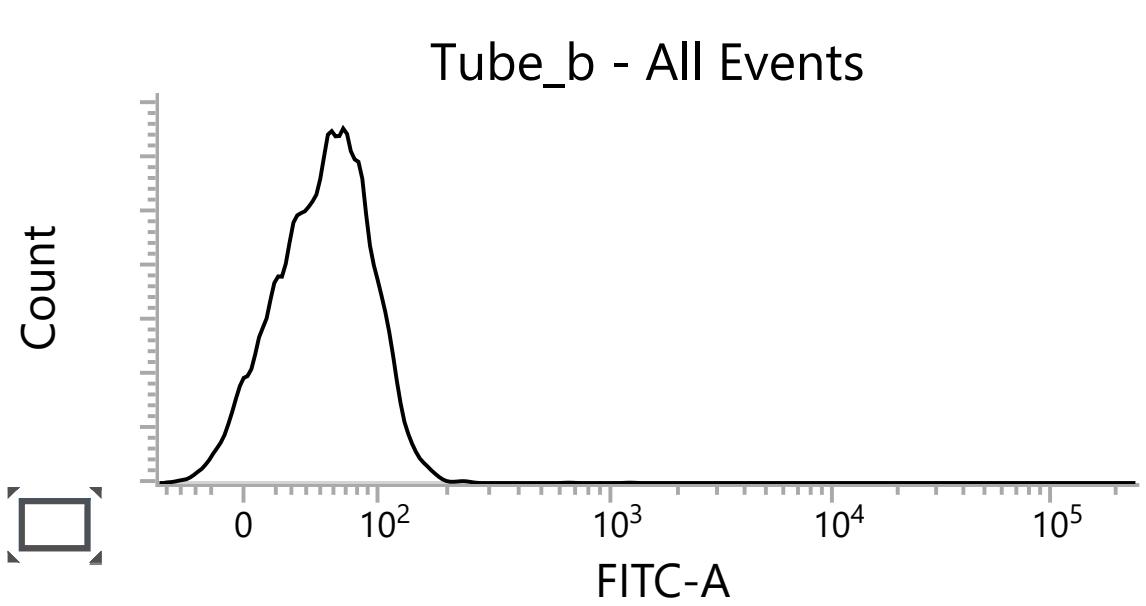

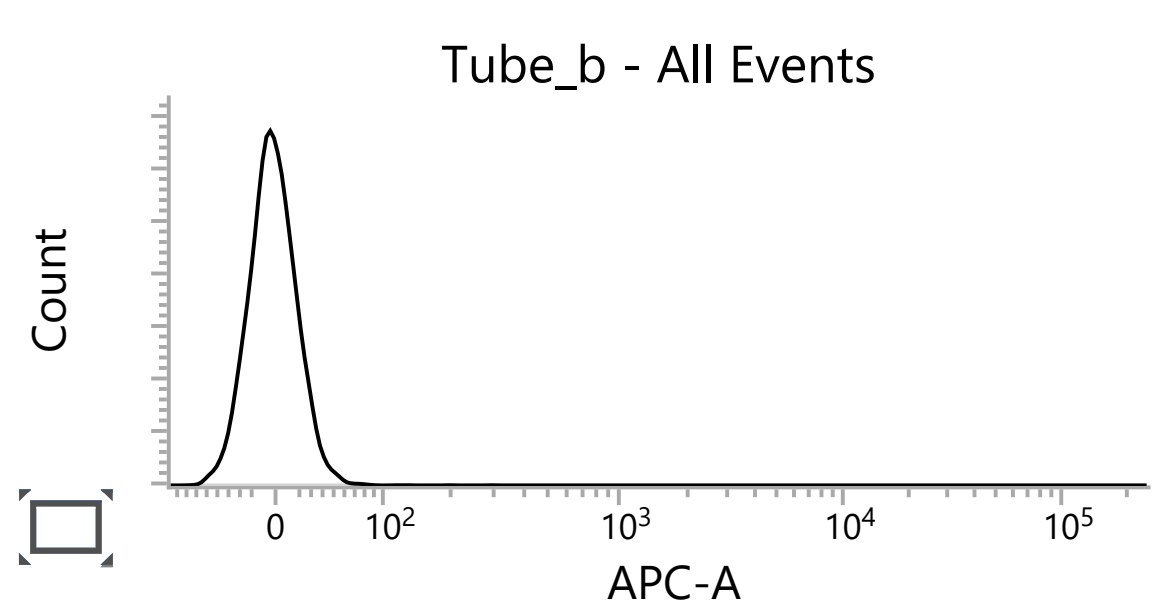

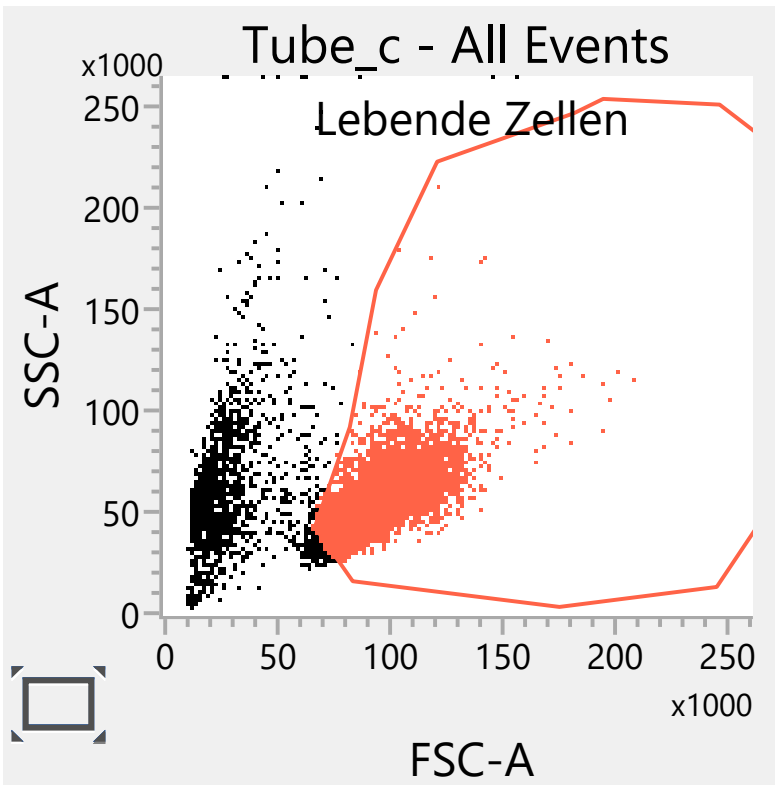

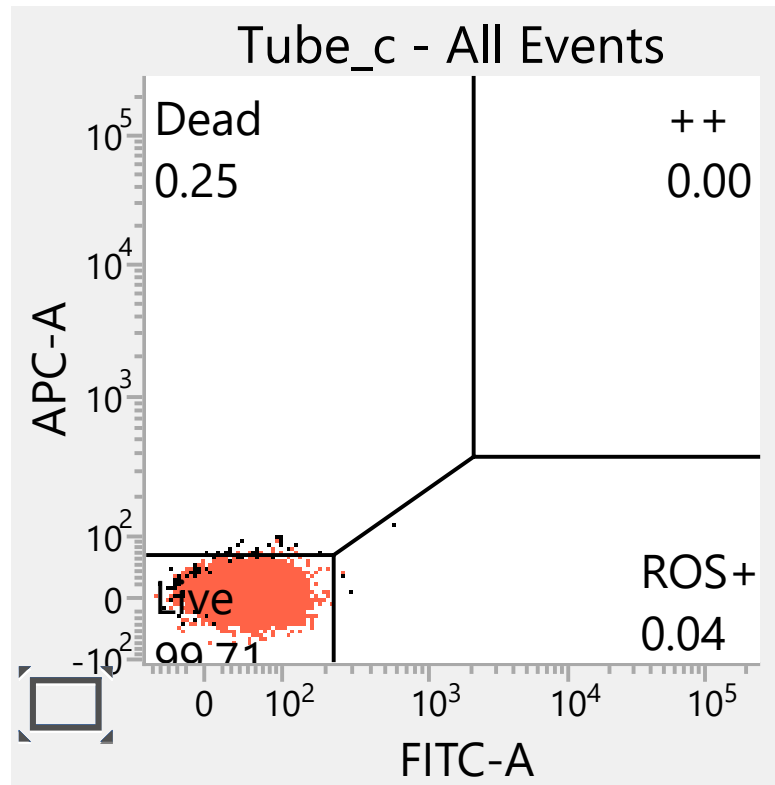

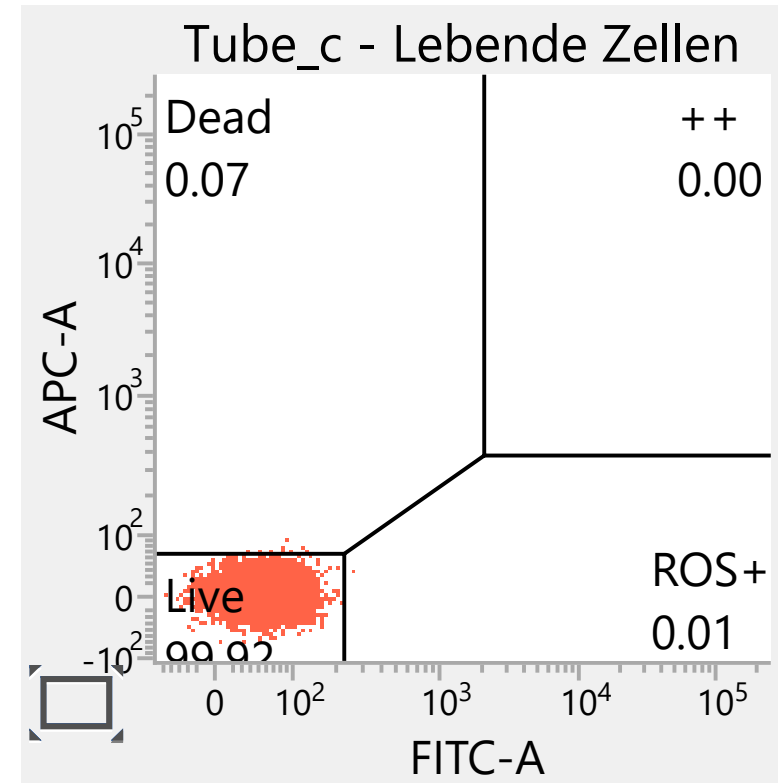

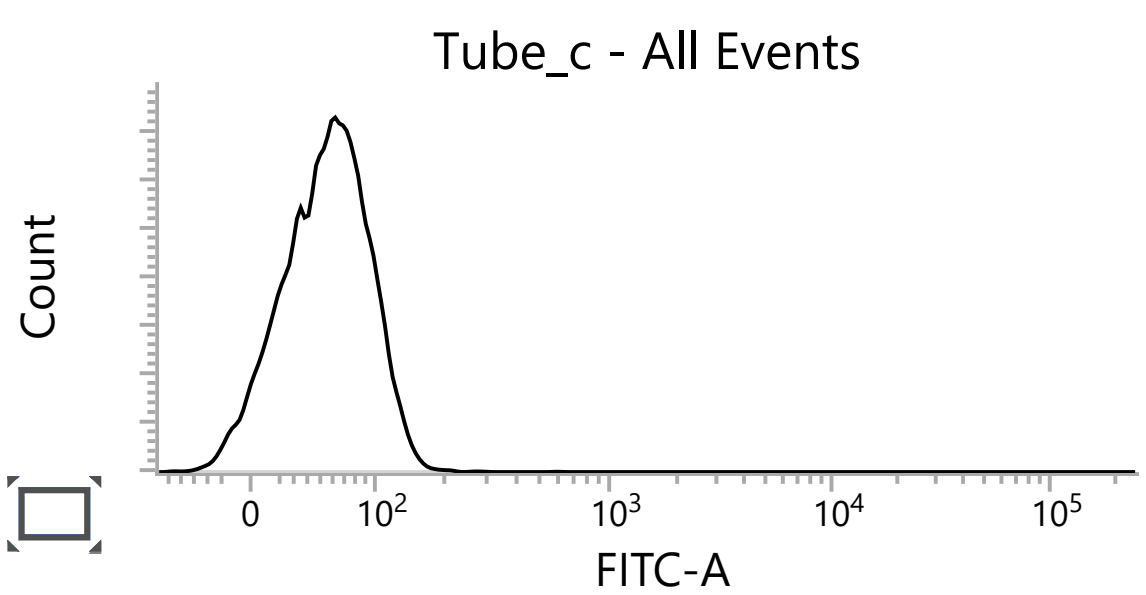

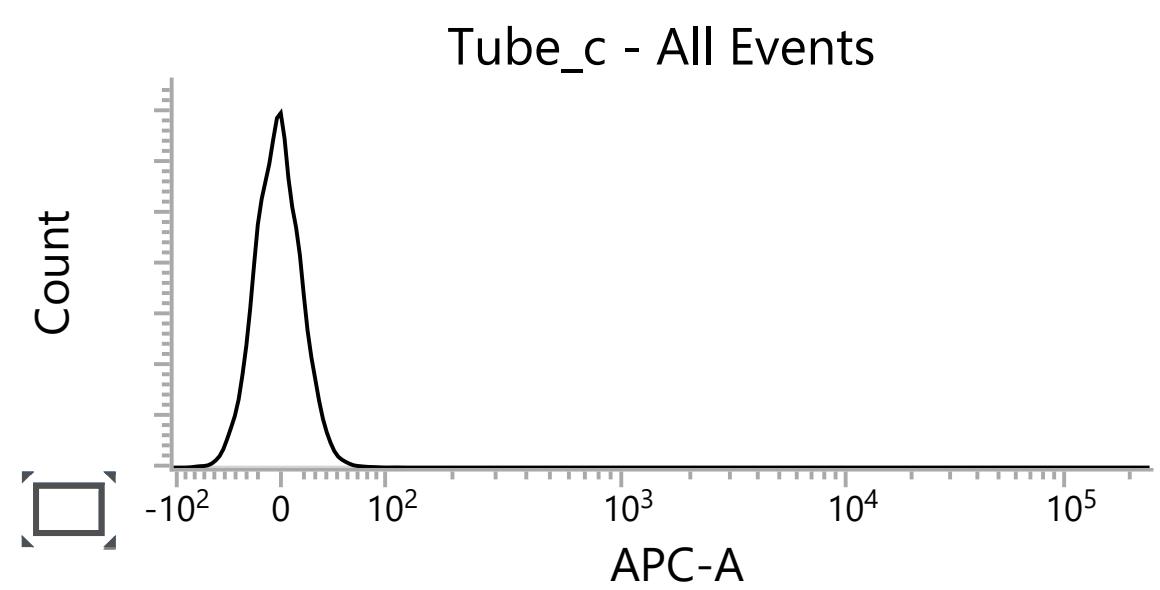

Statistics

Name	Events	% Parent	% Grandparent	% Total
Tube_a:All Events	10,000	***	***	100.00
Tube_a:Lebende Zellen	8,172	81.72	***	81.72
Tube_b:All Events	10,000	***	***	100.00
Tube_b:Lebende Zellen	8,280	82.80	***	82.80
Tube_c:All Events	10,000	***	***	100.00
Tube_c:Lebende Zellen	8,458	84.58	***	84.58

JC-1 Assay

Sample ID: N6_0,5

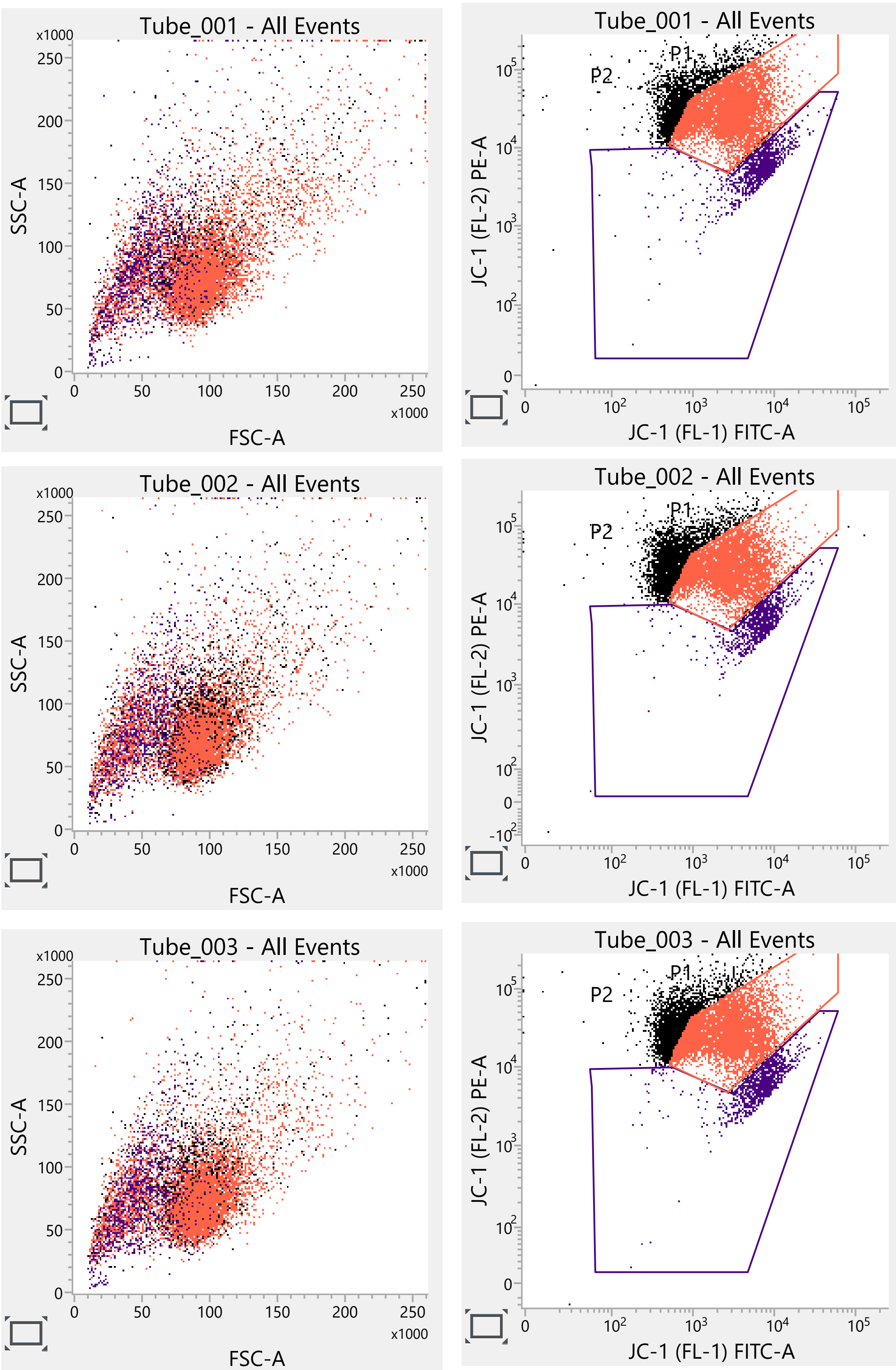

Statistics

Name	Tube_001:All Events	Tube_001:P2	Tube_001:P1	Tube_002:All Events	Tube_002:P2	Tube_002:P1	Tube_003:All Events	Tube_003:P2	Tube_003:P1
Events	10,000	1,141	6,600	10,000	992	5,308	10,000	1,062	5,906
% Parent	***	11.4	66.0	***	9.9	53.1	***	10.6	59.1
% Grandparent	***	***	***	***	***	***	***	***	***
% Total	100.0	11.4	66.0	100.0	9.9	53.1	100.0	10.6	59.1

JC-1 Assay

Sample ID: N6_1

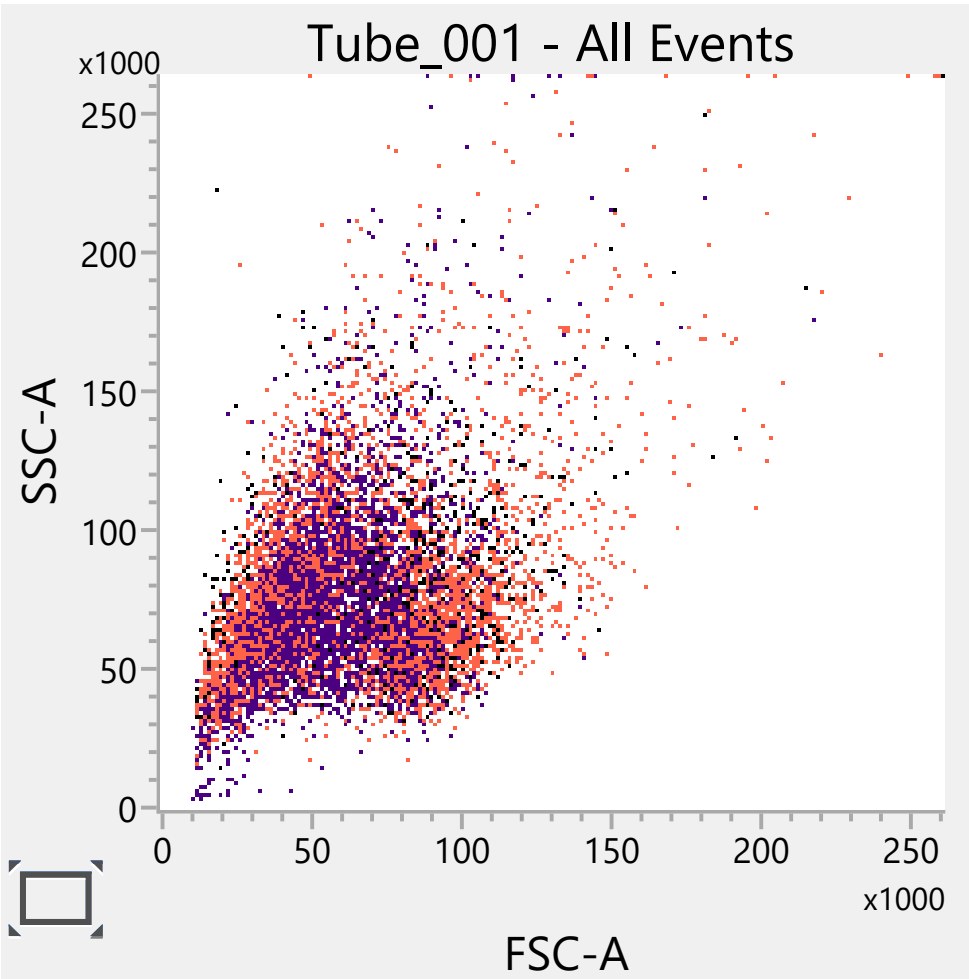

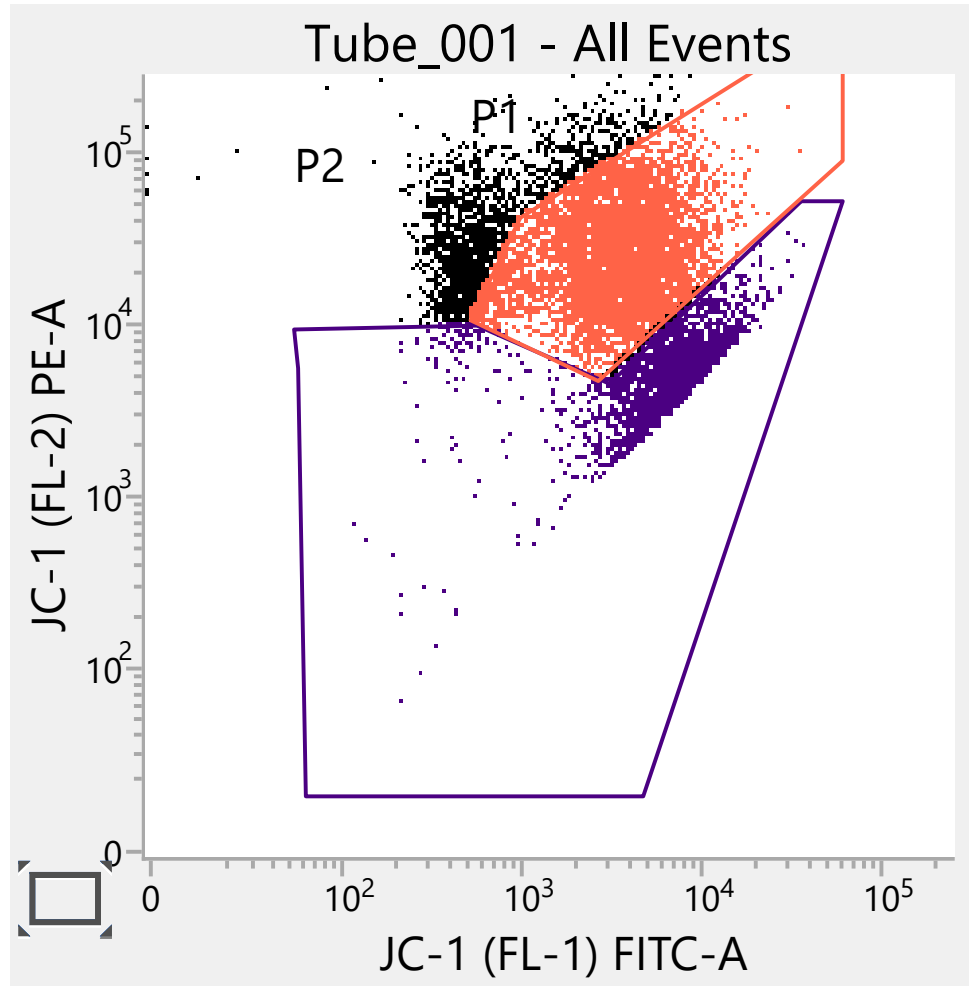

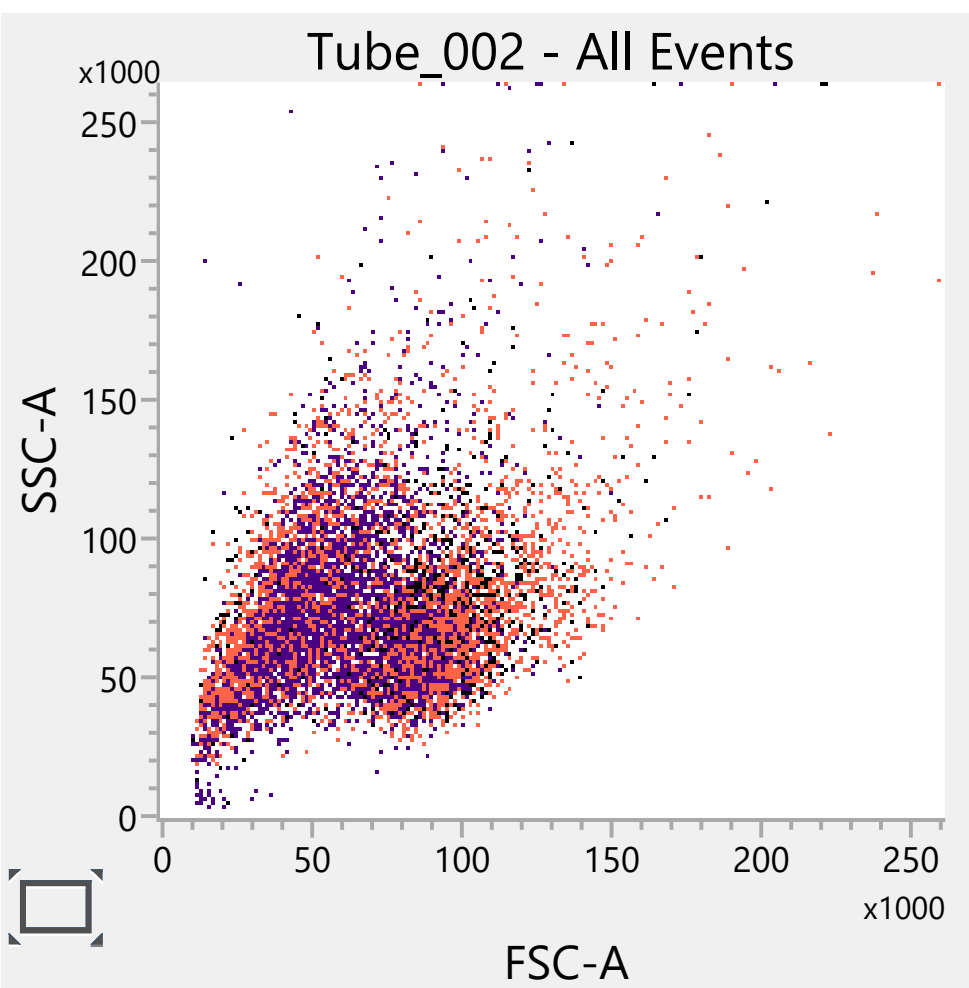

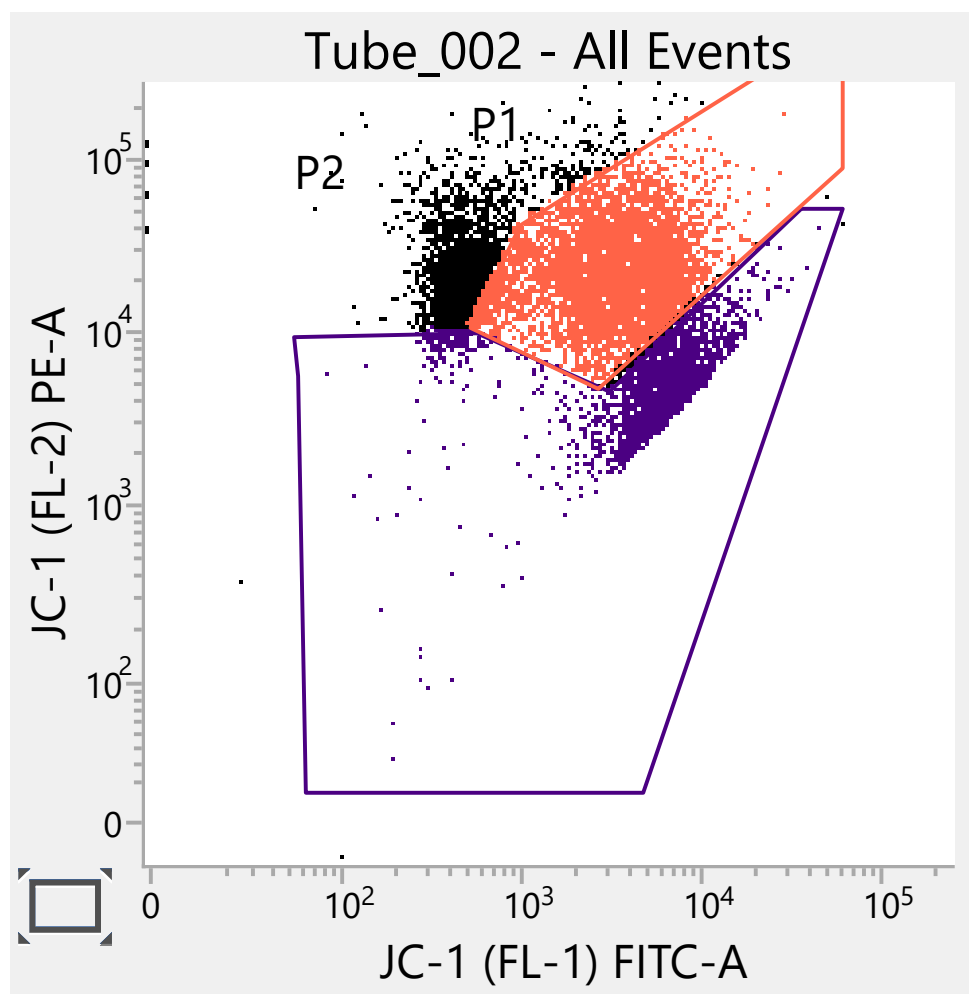

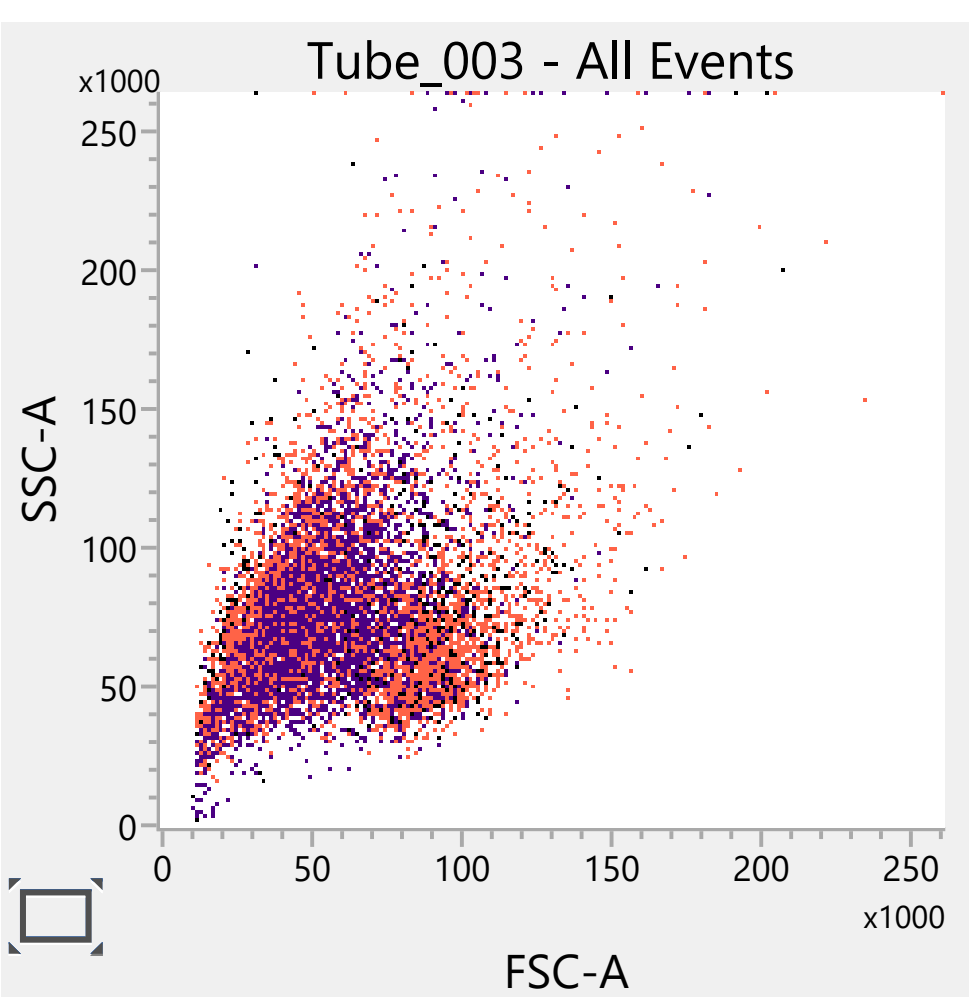

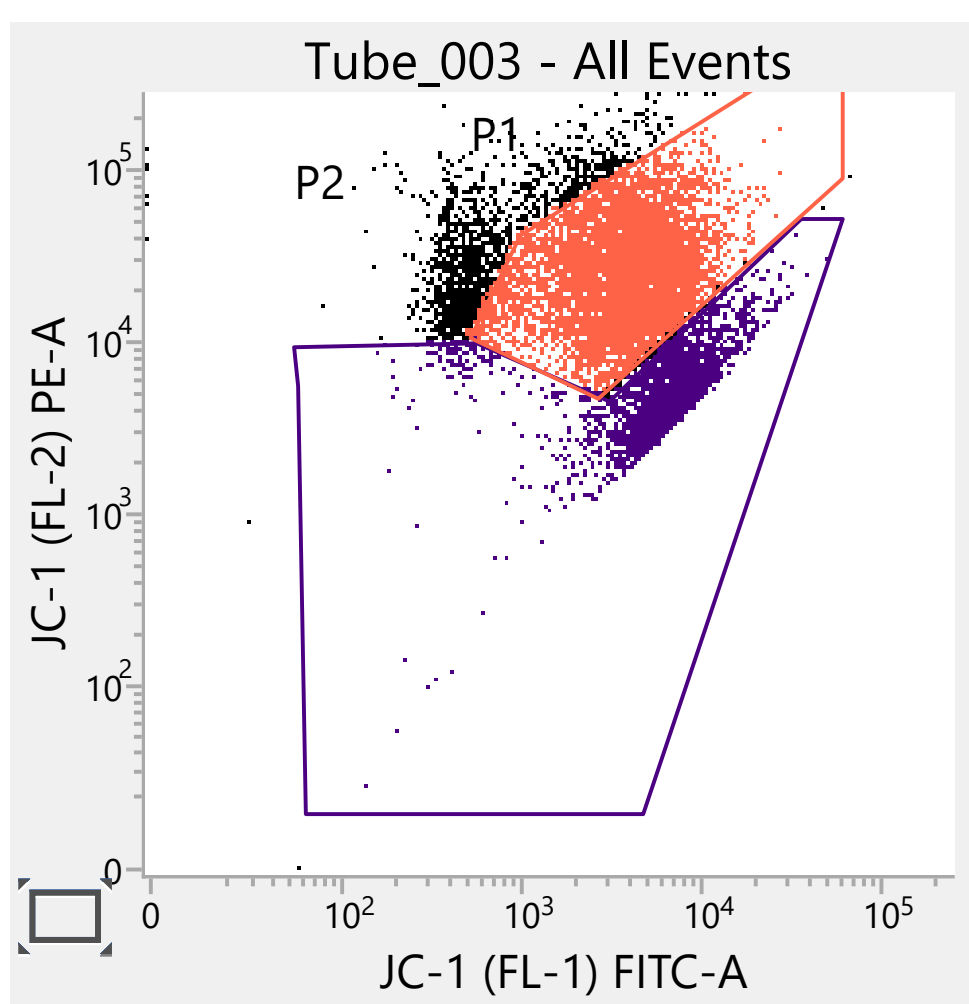

Statistics

Name	Tube_001:All Events	Tube_001:P2	Tube_001:P1	Tube_002:All Events	Tube_002:P2	Tube_002:P1	Tube_003:All Events	Tube_003:P2	Tube_003:P1
Events	10,000	2,580	5,739	10,000	2,476	5,258	10,000	2,567	5,987
% Parent	***	25.8	57.4	***	24.8	52.6	***	25.7	59.9
% Grandparent	***	***	***	***	***	***	***	***	***
% Total	100.0	25.8	57.4	100.0	24.8	52.6	100.0	25.7	59.9

JC-1 Assay

Sample ID: N6_2,5

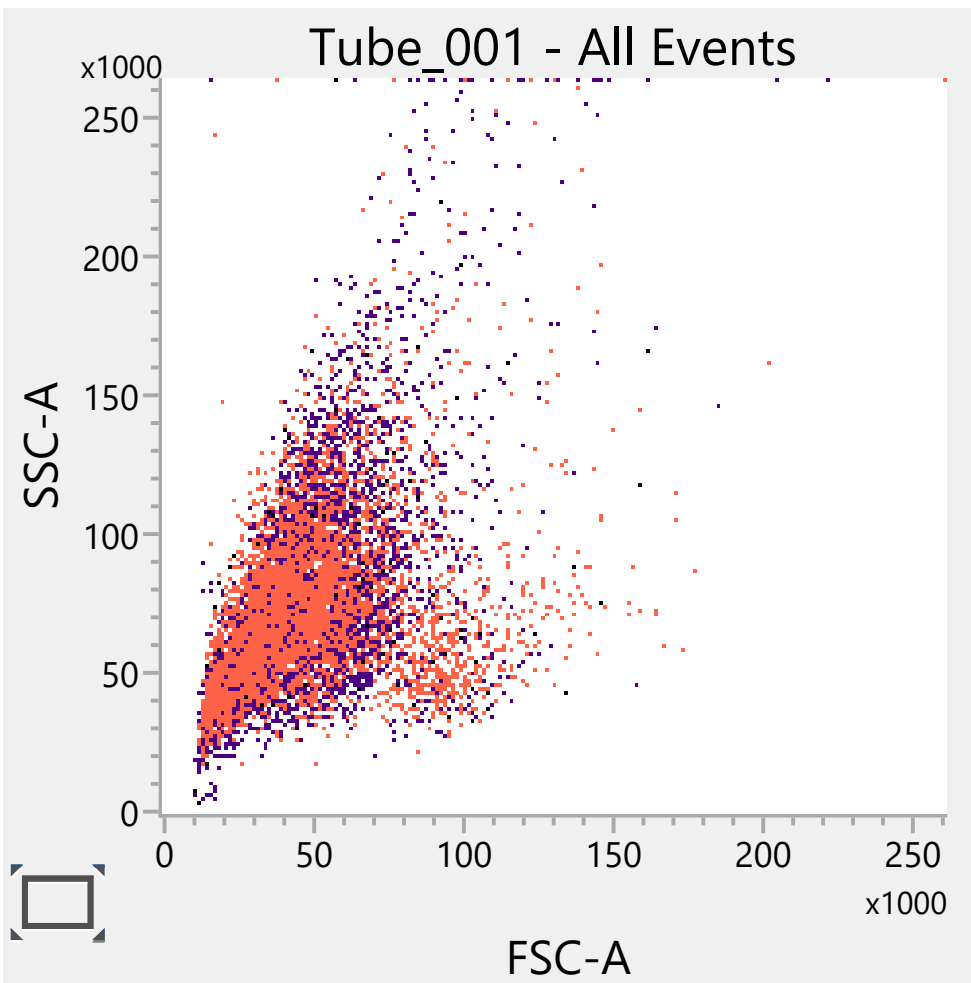

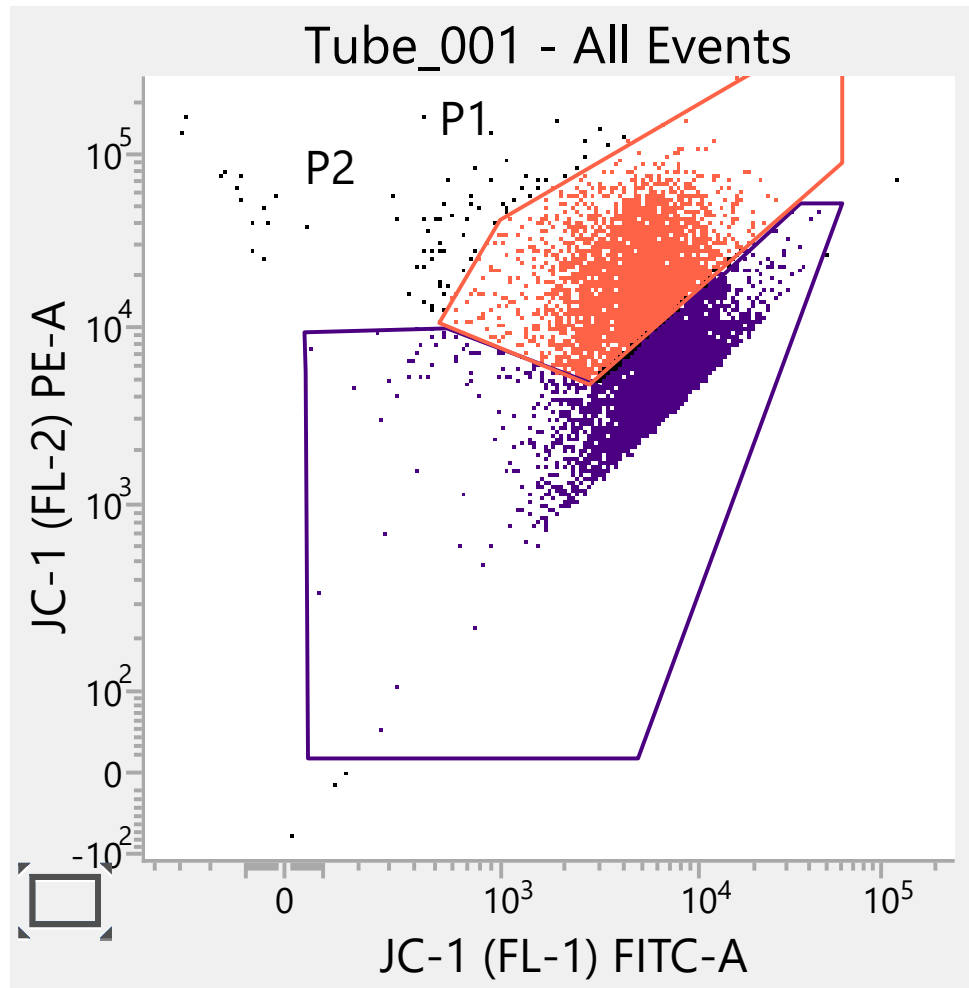

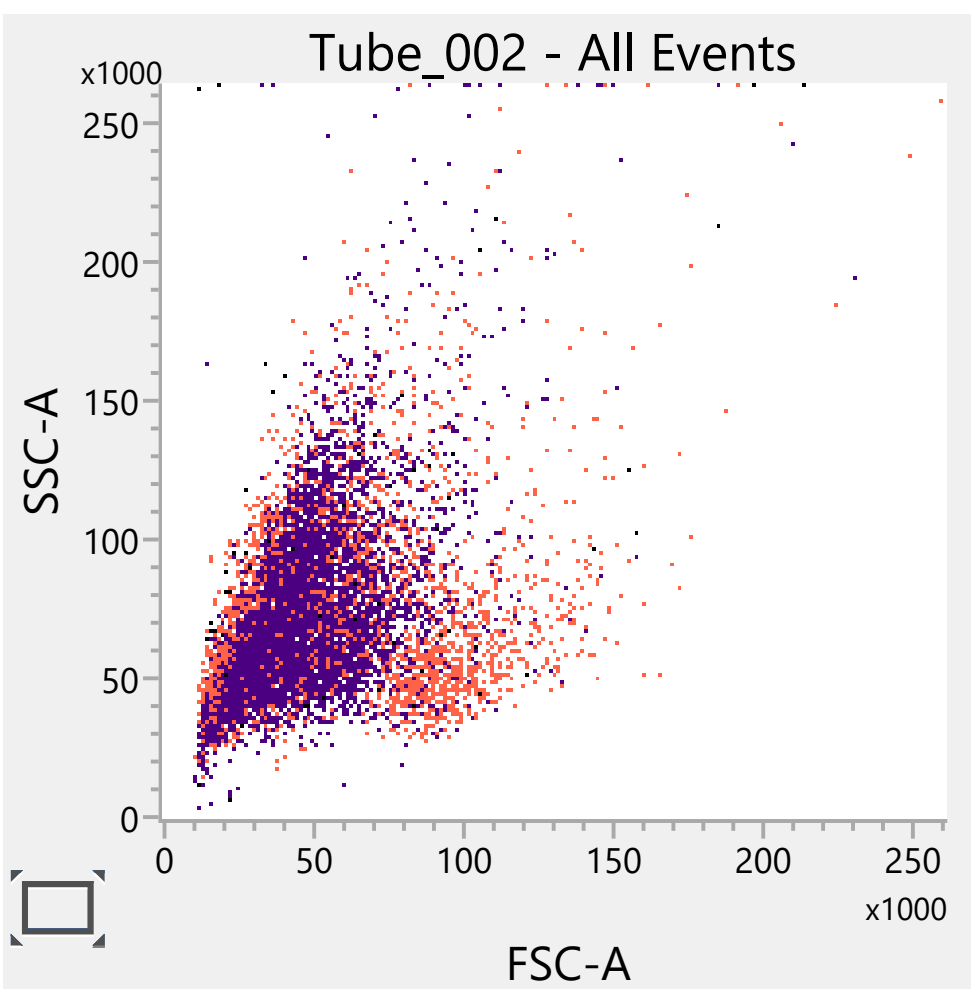

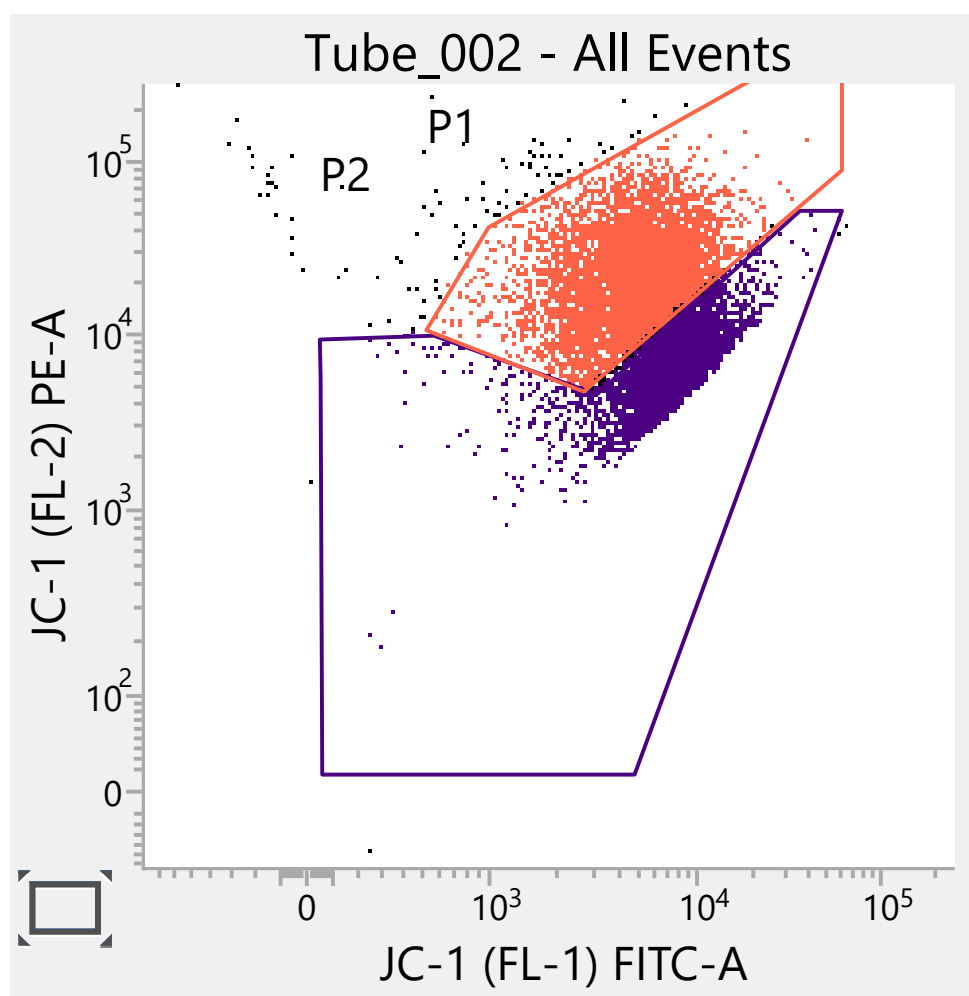

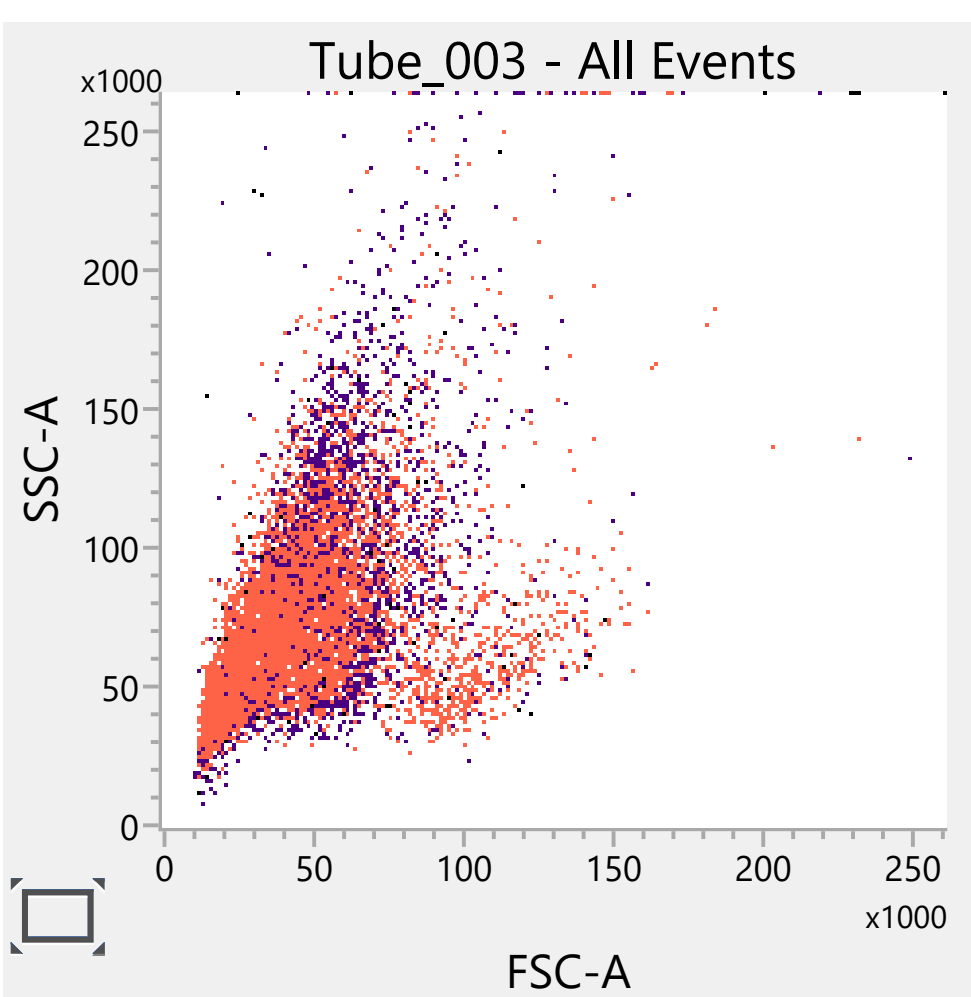

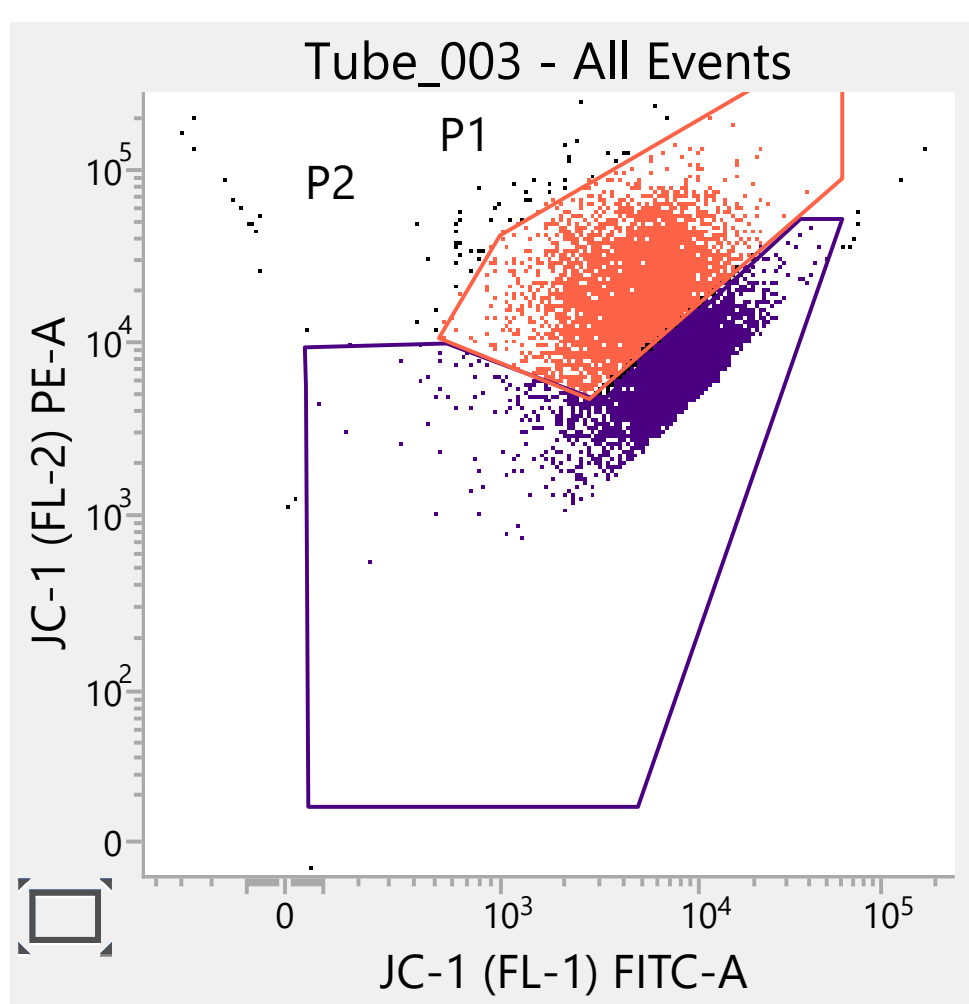

Statistics

Name	Tube_001:All Events	Tube_001:P2	Tube_001:P1	Tube_002:All Events	Tube_002:P2	Tube_002:P1	Tube_003:All Events	Tube_003:P2	Tube_003:P1
Events	10,000	5,743	3,856	10,000	3,998	5,642	10,000	4,850	4,771
% Parent	***	57.4	38.6	***	40.0	56.4	***	48.5	47.7
% Grandparent	***	***	***	***	***	***	***	***	***
% Total	100.0	57.4	38.6	100.0	40.0	56.4	100.0	48.5	47.7

JC-1 Assay

Sample ID: N6_5

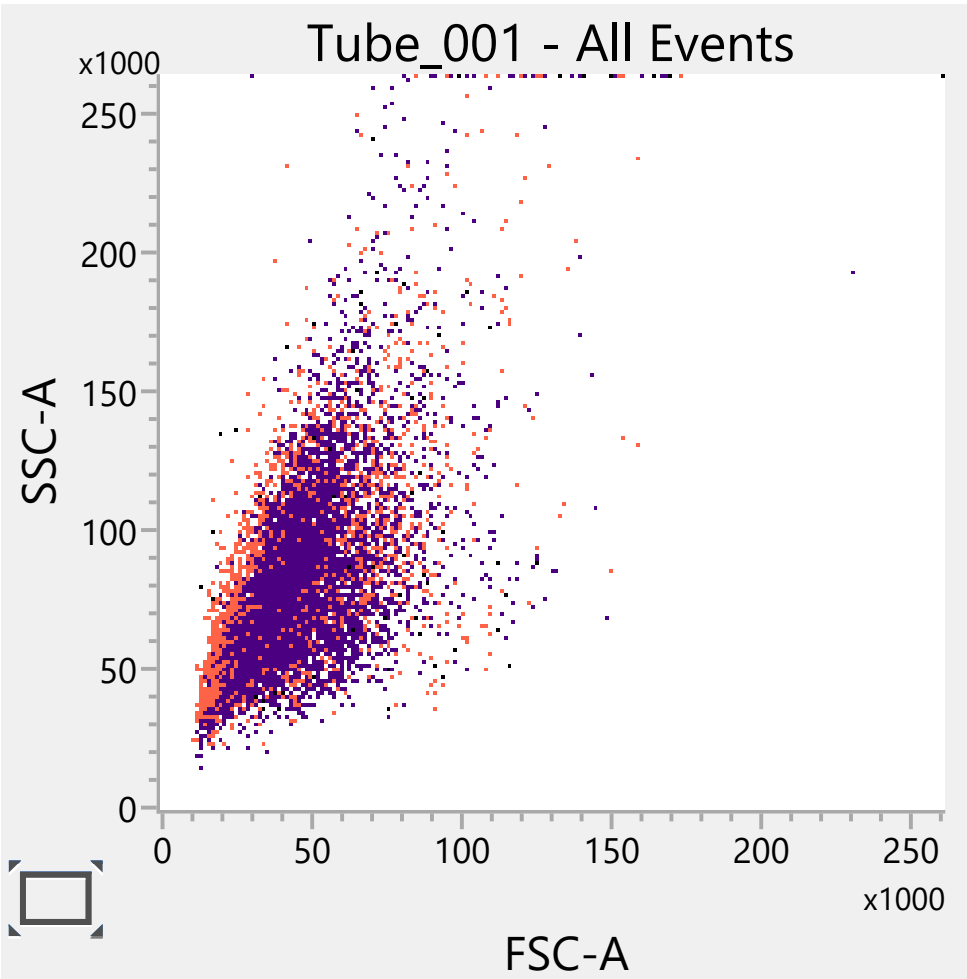

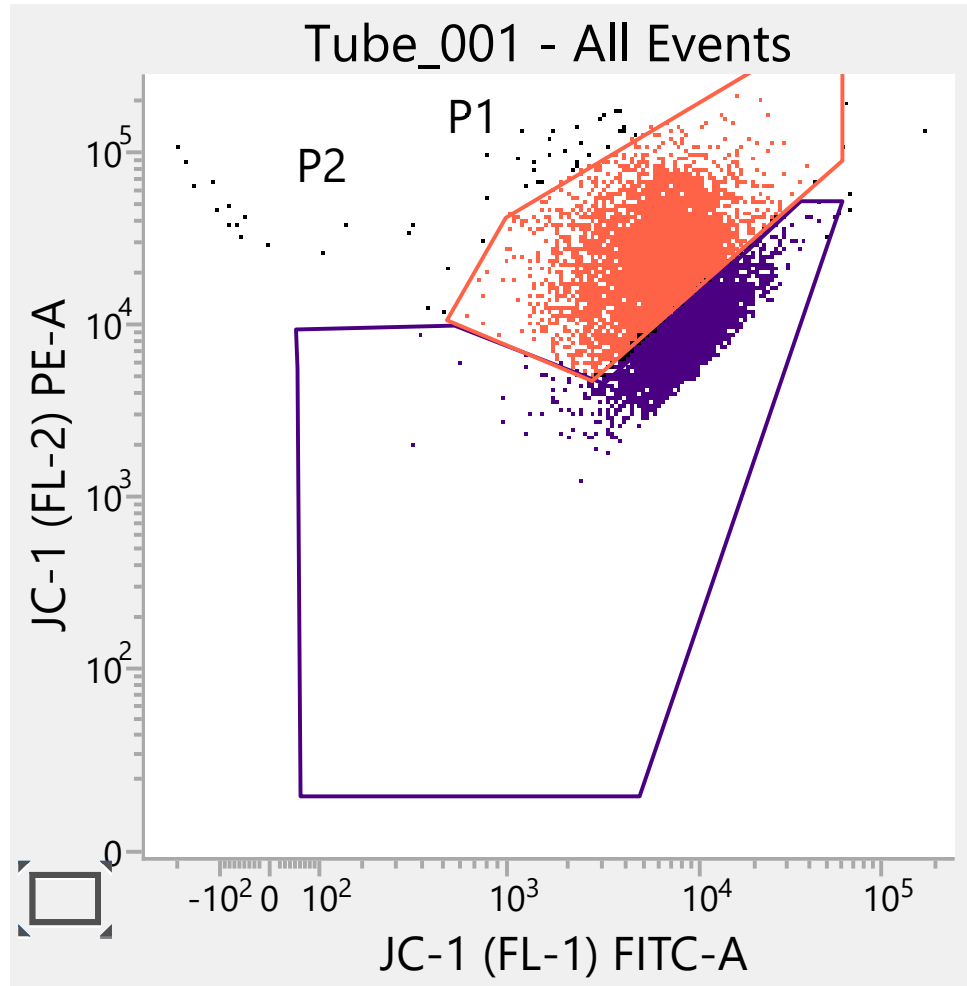

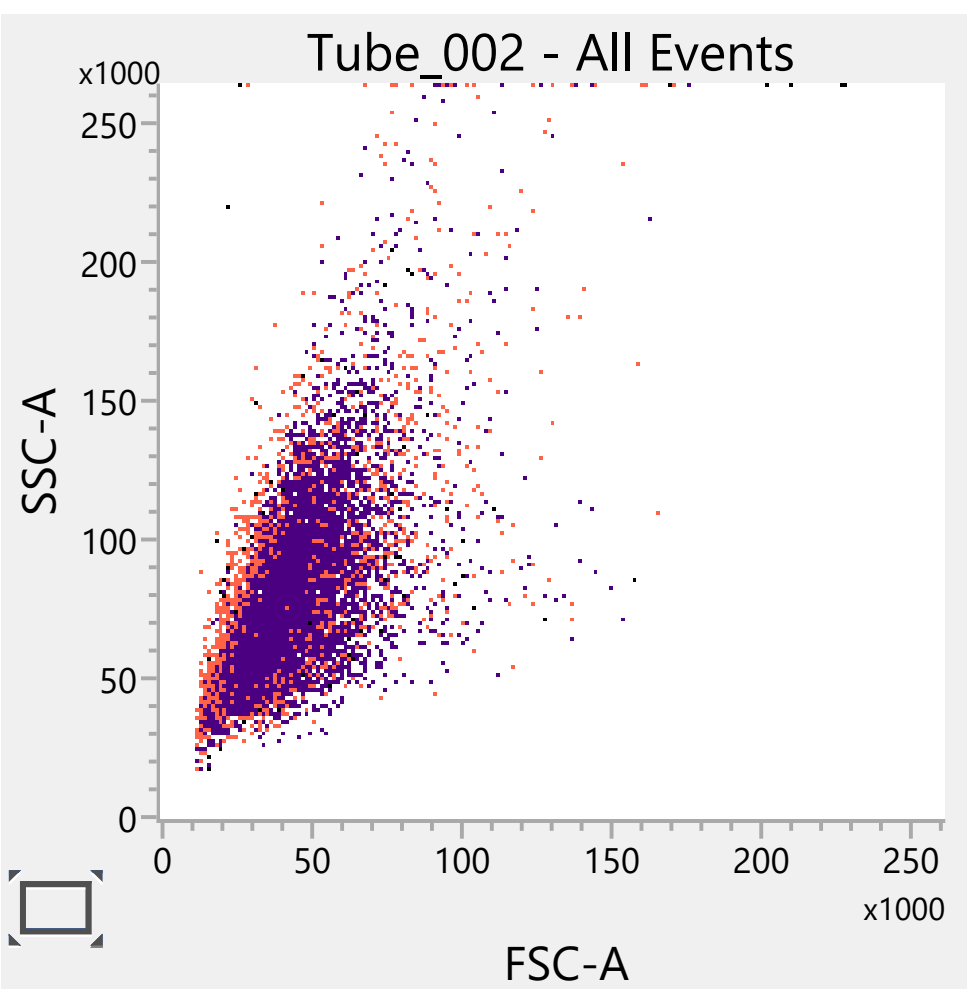

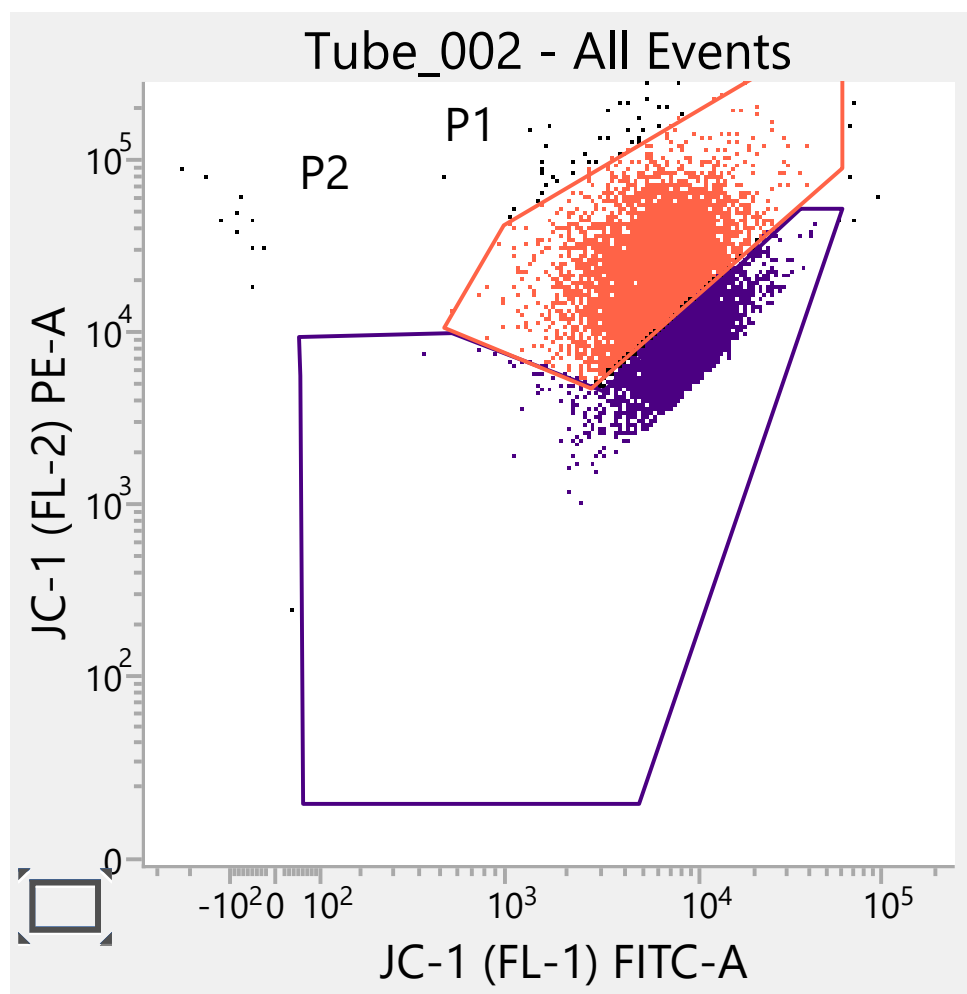

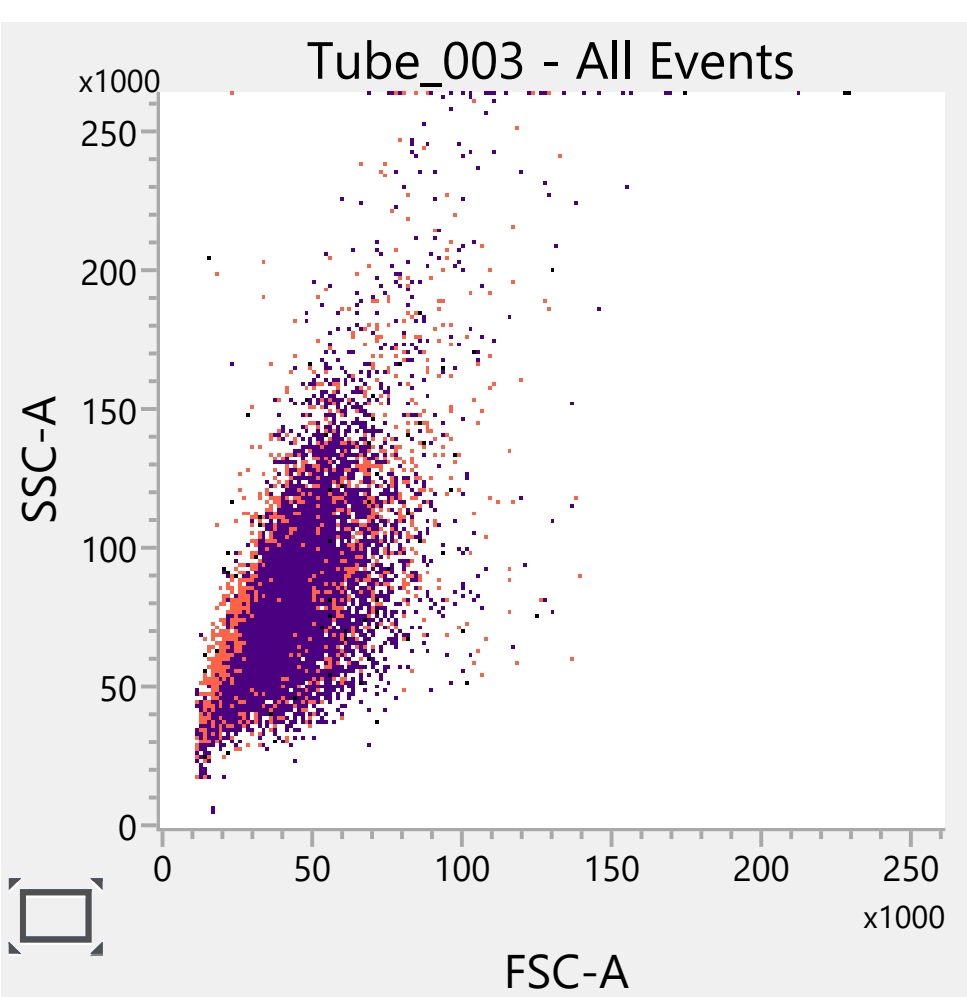

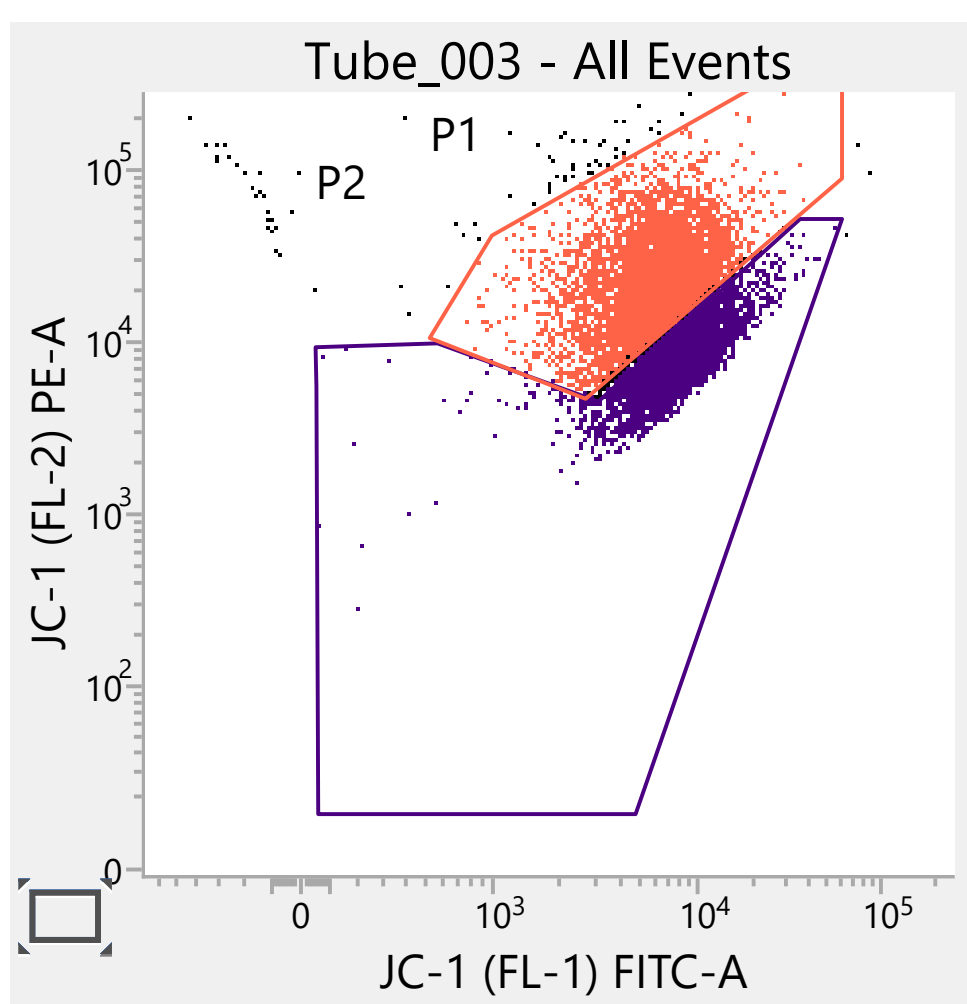

Statistics

Name	Tube_001:All Events	Tube_001:P2	Tube_001:P1	Tube_002:All Events	Tube_002:P2	Tube_002:P1	Tube_003:All Events	Tube_003:P2	Tube_003:P1
Events	10,000	4,157	5,423	10,000	4,135	5,443	10,000	4,379	5,112
% Parent	***	41.6	54.2	***	41.3	54.4	***	43.8	51.1
% Grandparent	***	***	***	***	***	***	***	***	***
% Total	100.0	41.6	54.2	100.0	41.3	54.4	100.0	43.8	51.1

JC-1 Assay

Sample ID: N6_CCCP

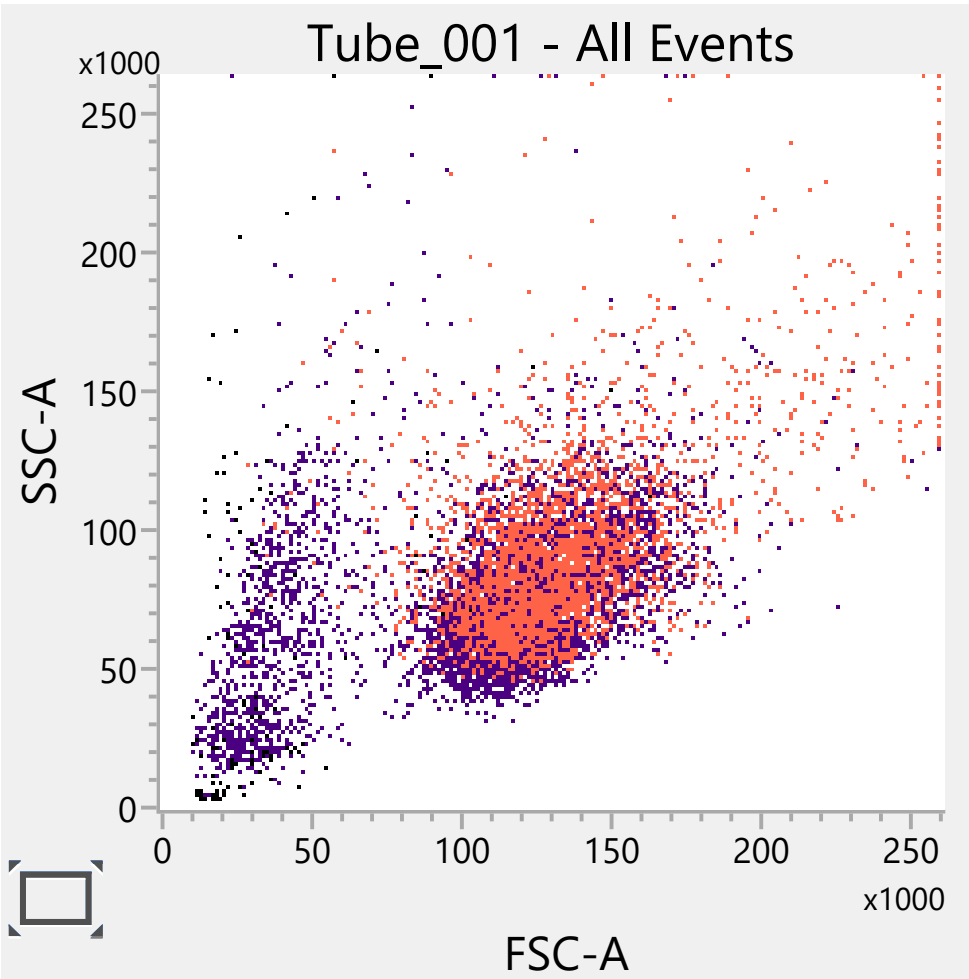

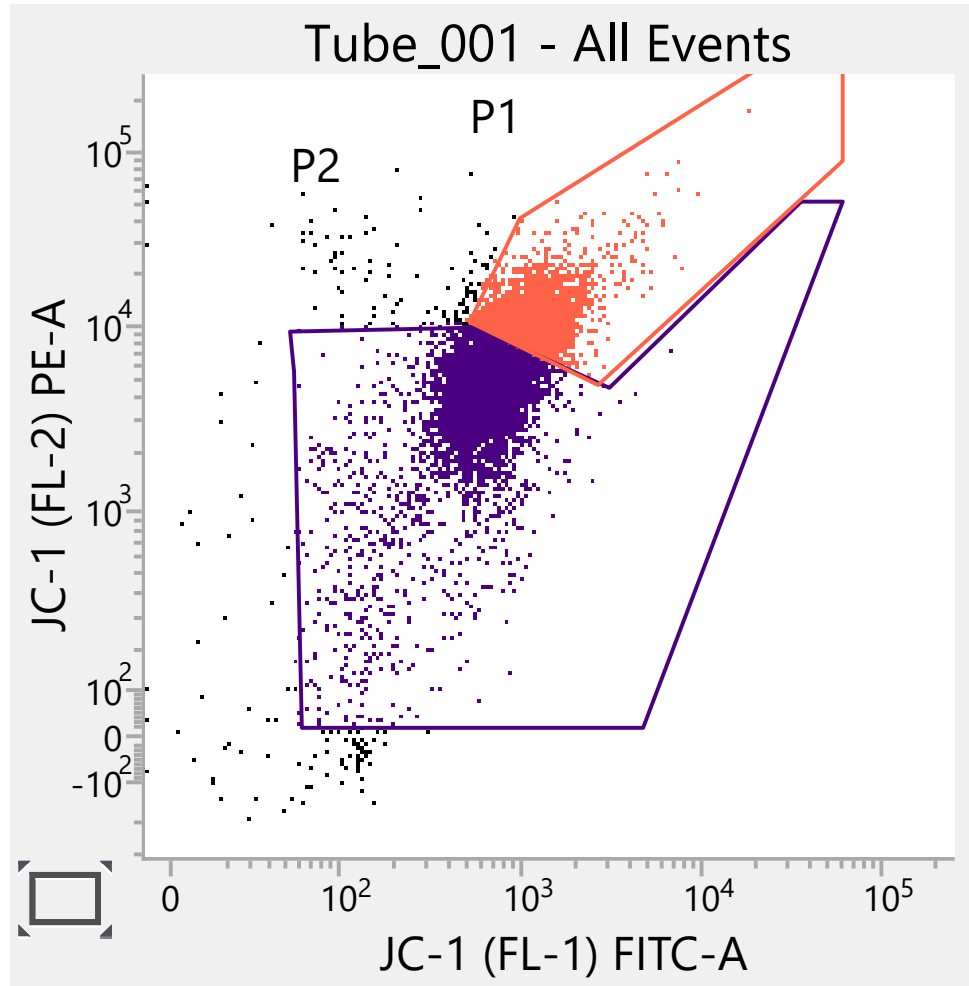

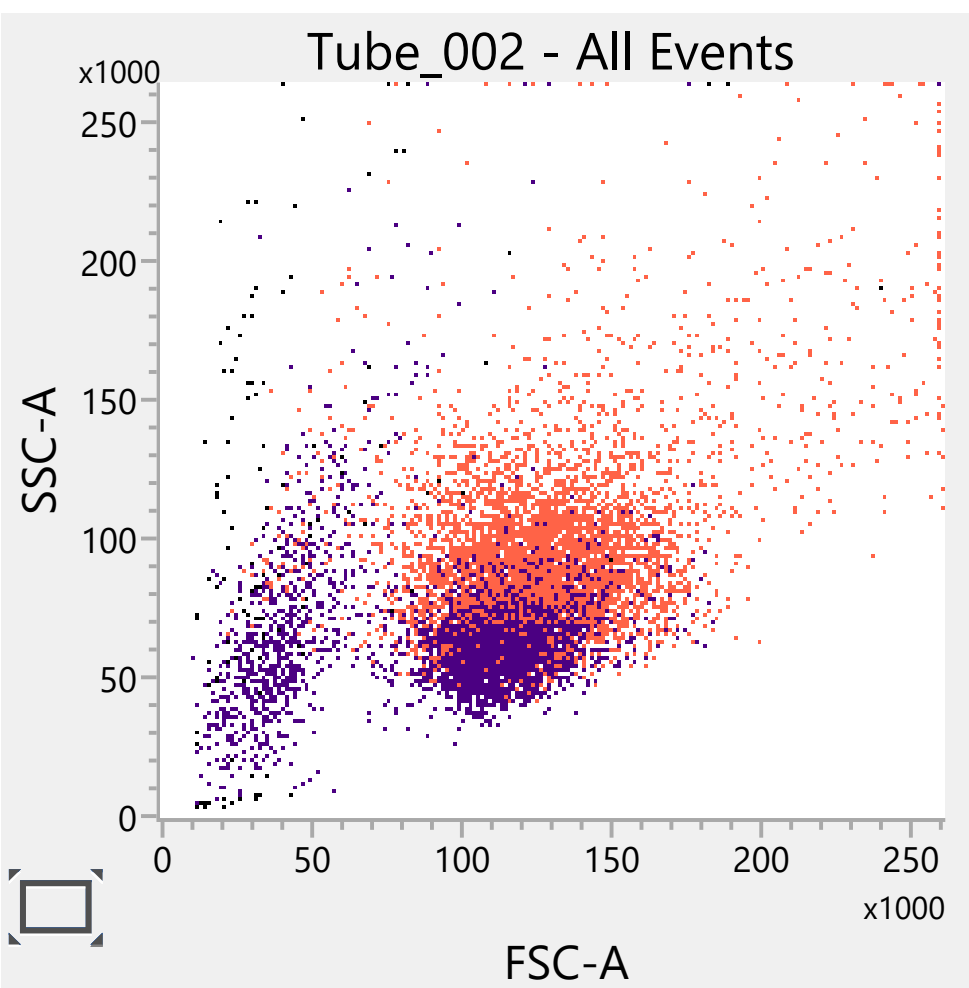

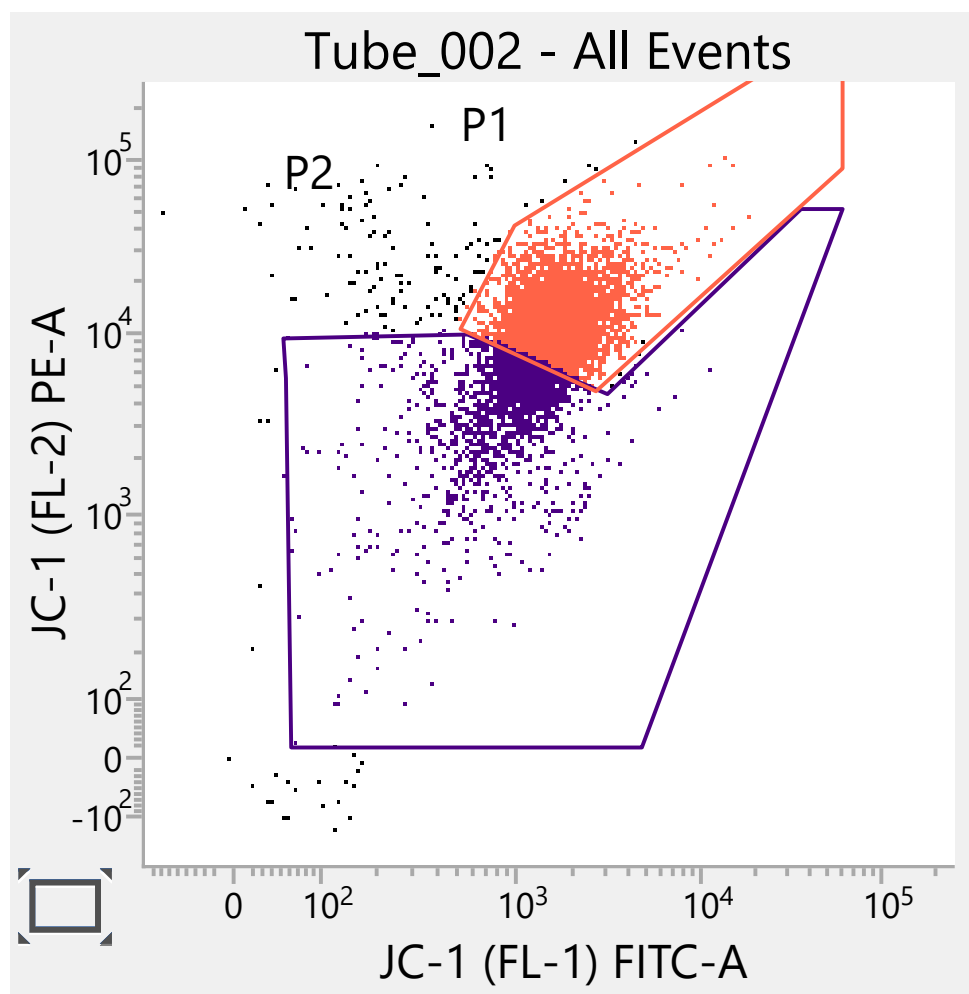

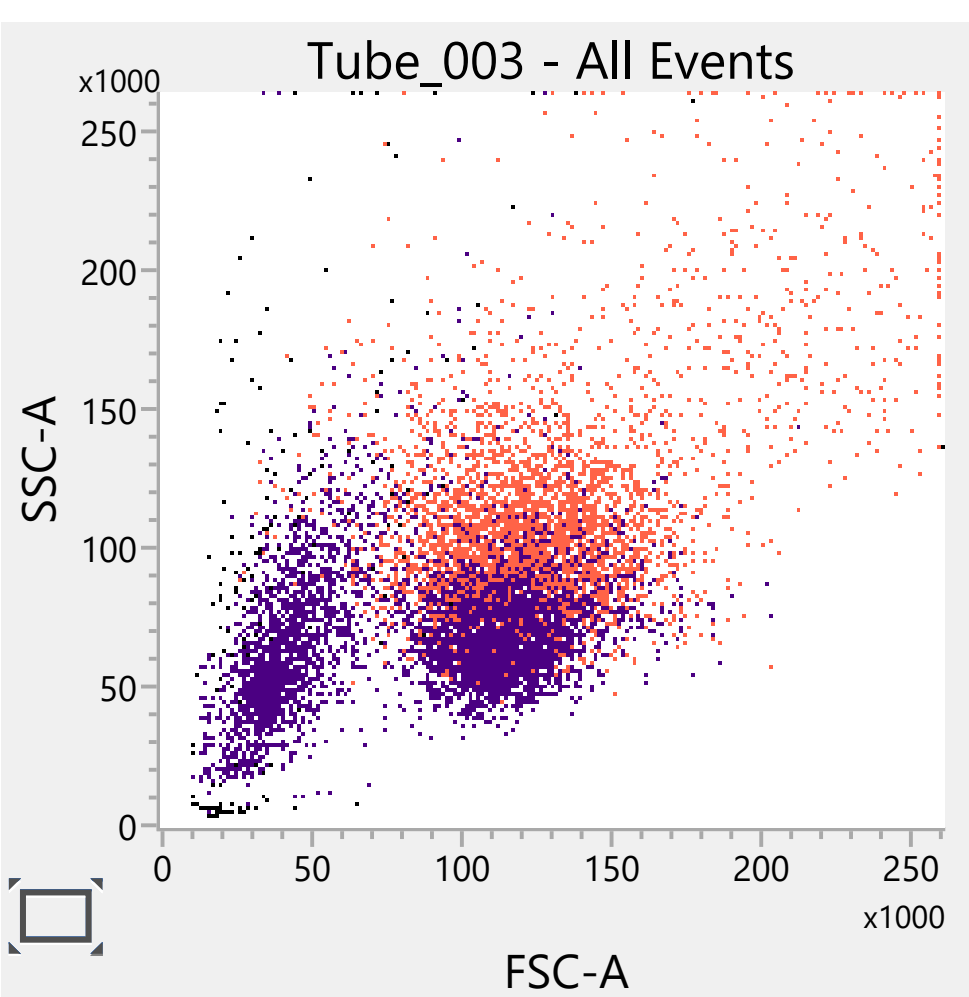

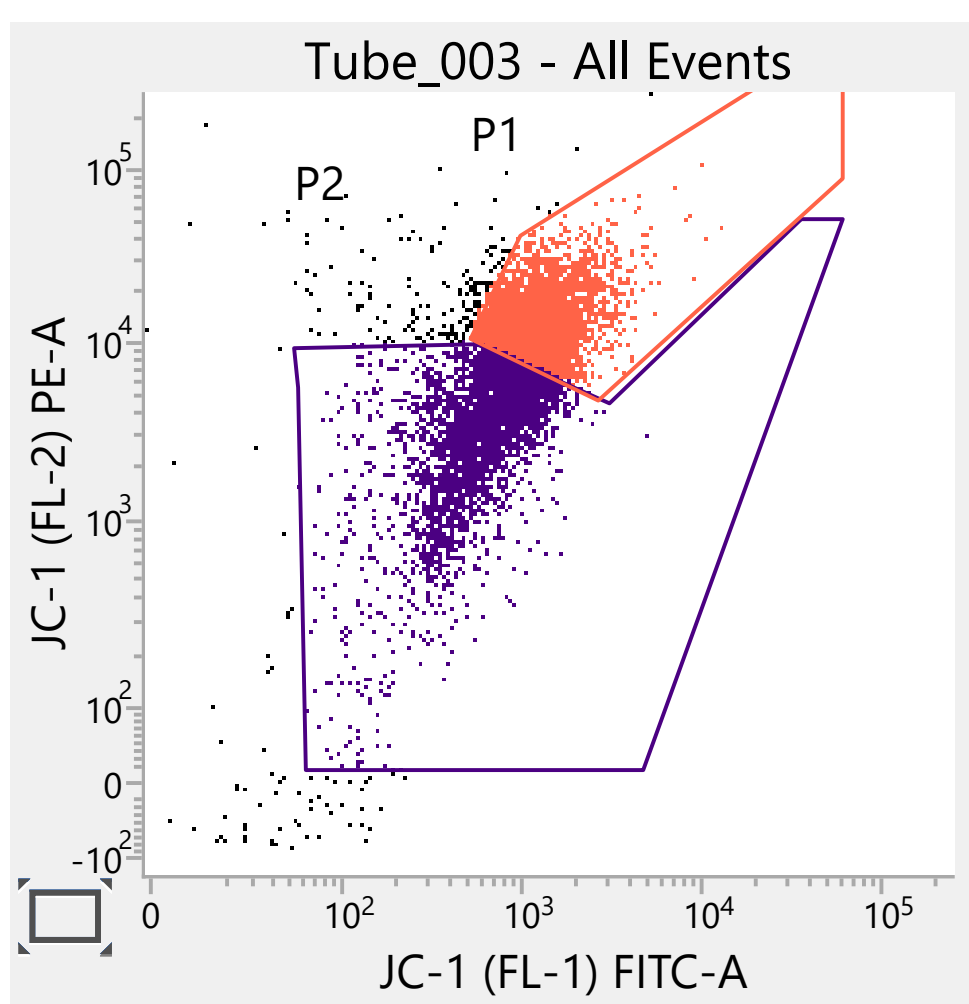

Statistics

Name	Tube_001:All Events	Tube_001:P2	Tube_001:P1	Tube_002:All Events	Tube_002:P2	Tube_002:P1	Tube_003:All Events	Tube_003:P2	Tube_003:P1
Events	10,000	6,856	3,047	10,000	2,768	7,249	10,000	4,596	5,232
% Parent	***	68.6	30.5	***	27.7	72.5	***	46.0	52.3
% Grandparent	***	***	***	***	***	***	***	***	***
% Total	100.0	68.6	30.5	100.0	27.7	72.5	100.0	46.0	52.3

JC-1 Assay

Sample ID: N6_DMSO

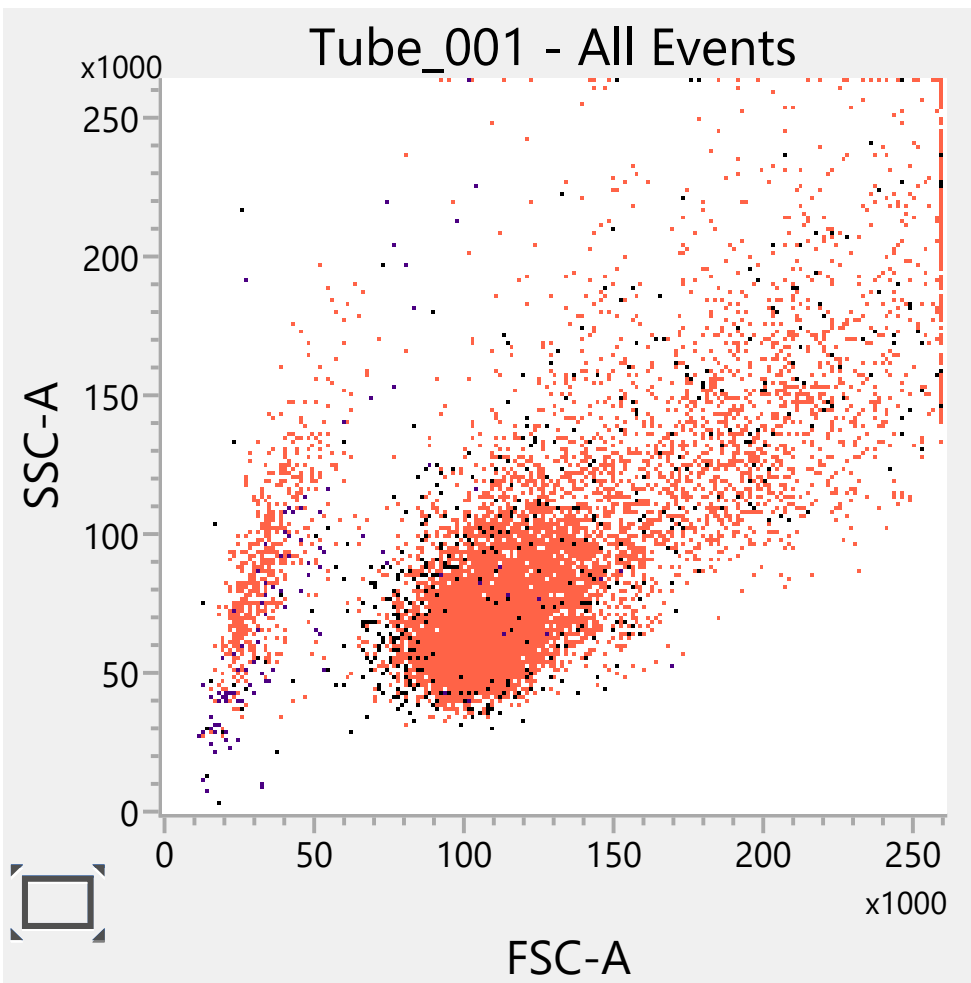

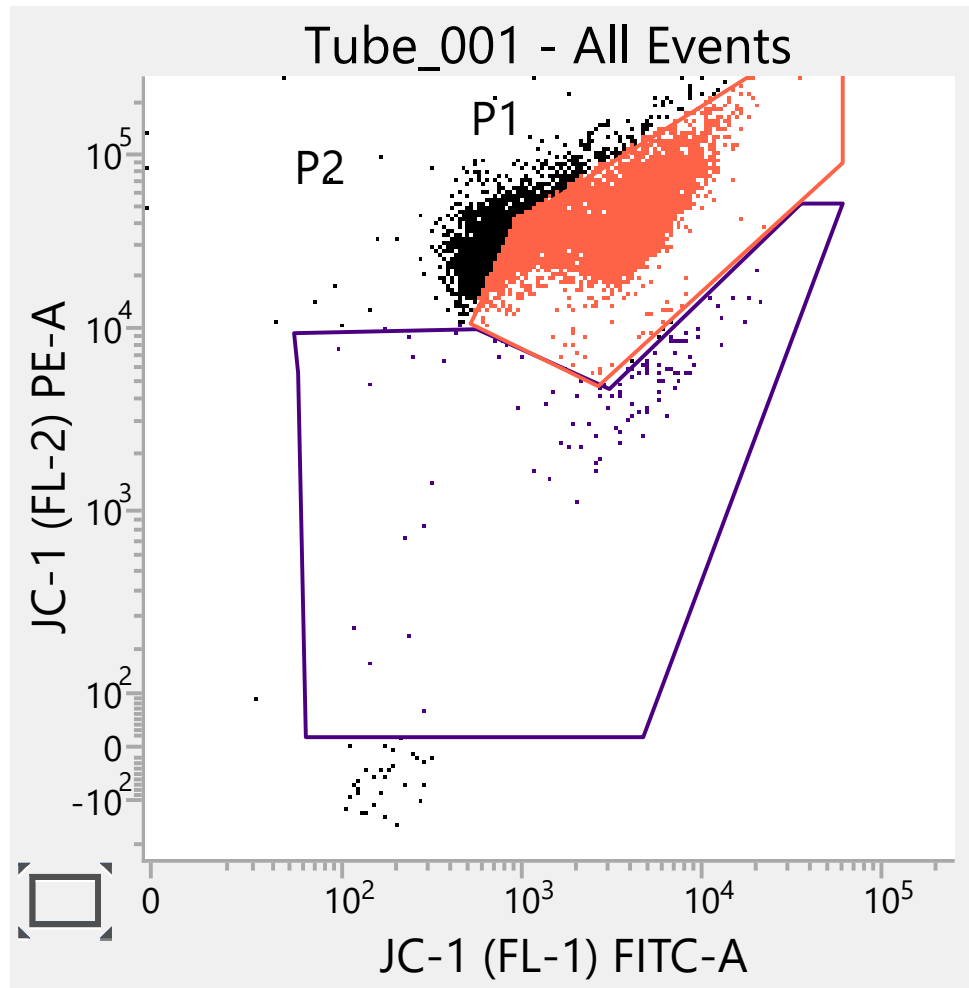

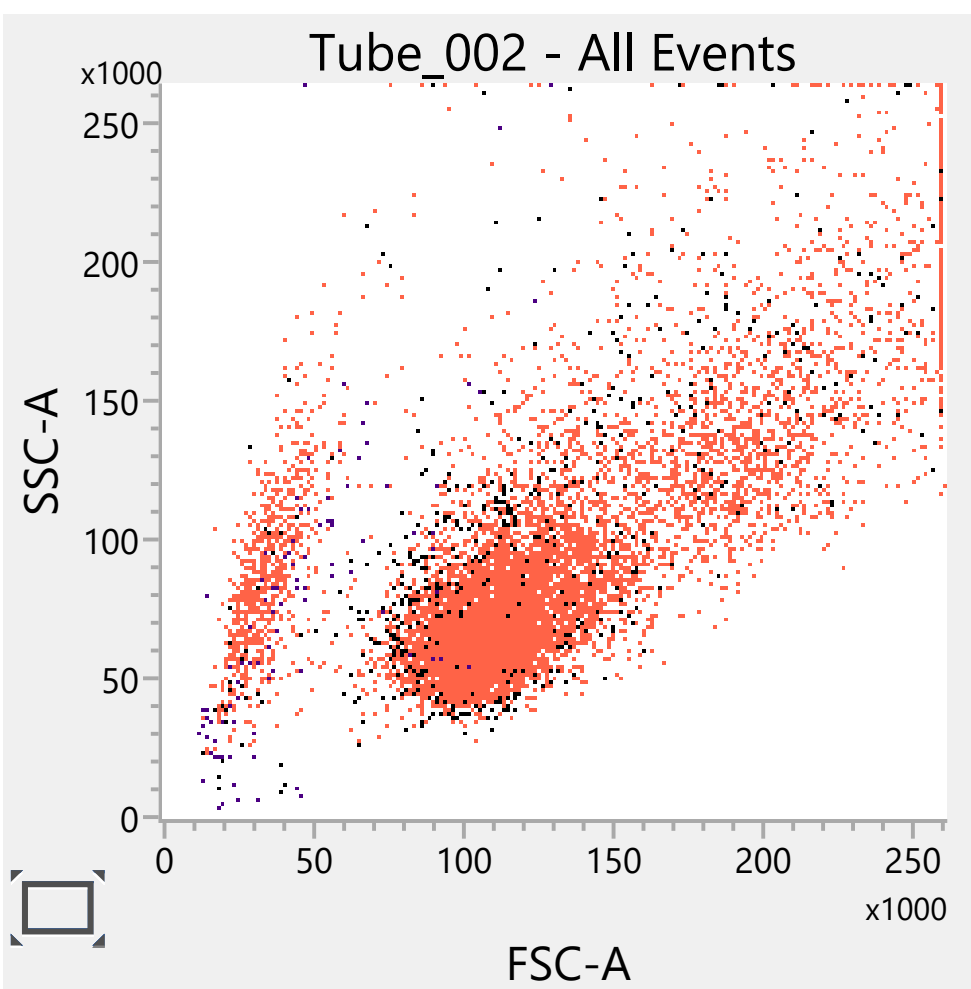

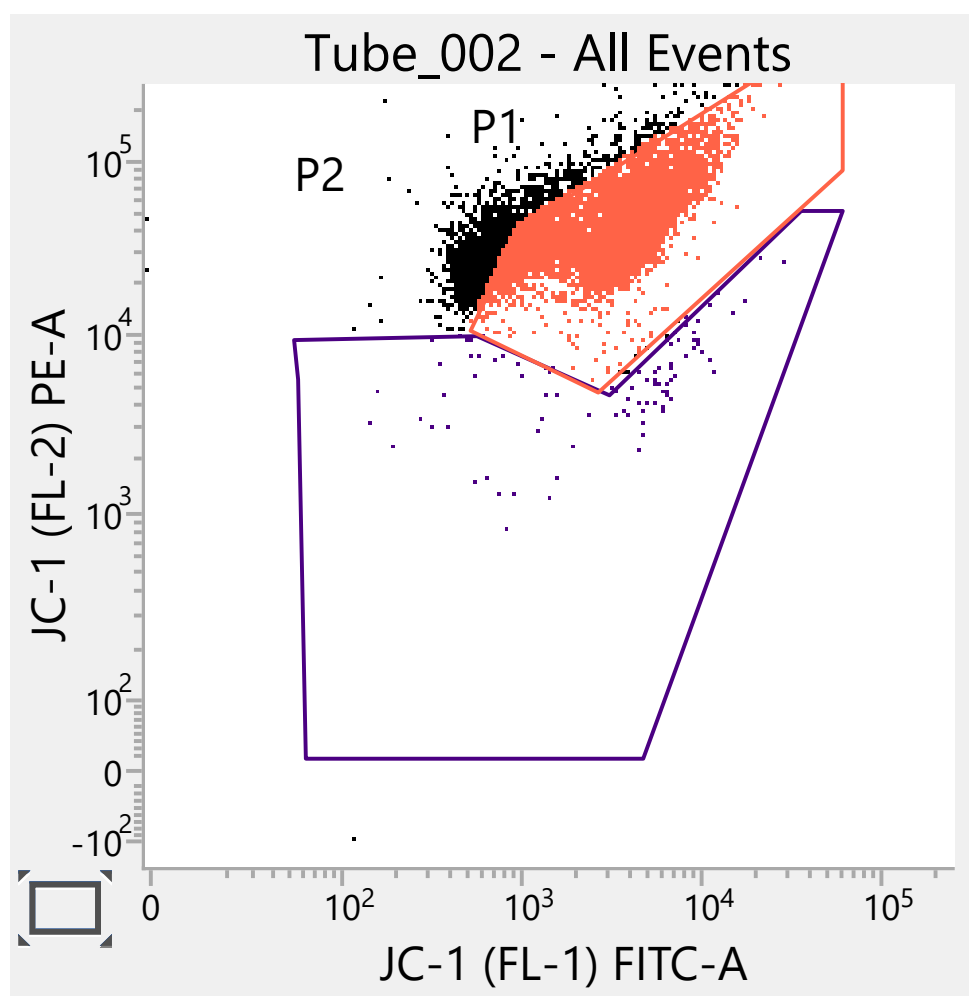

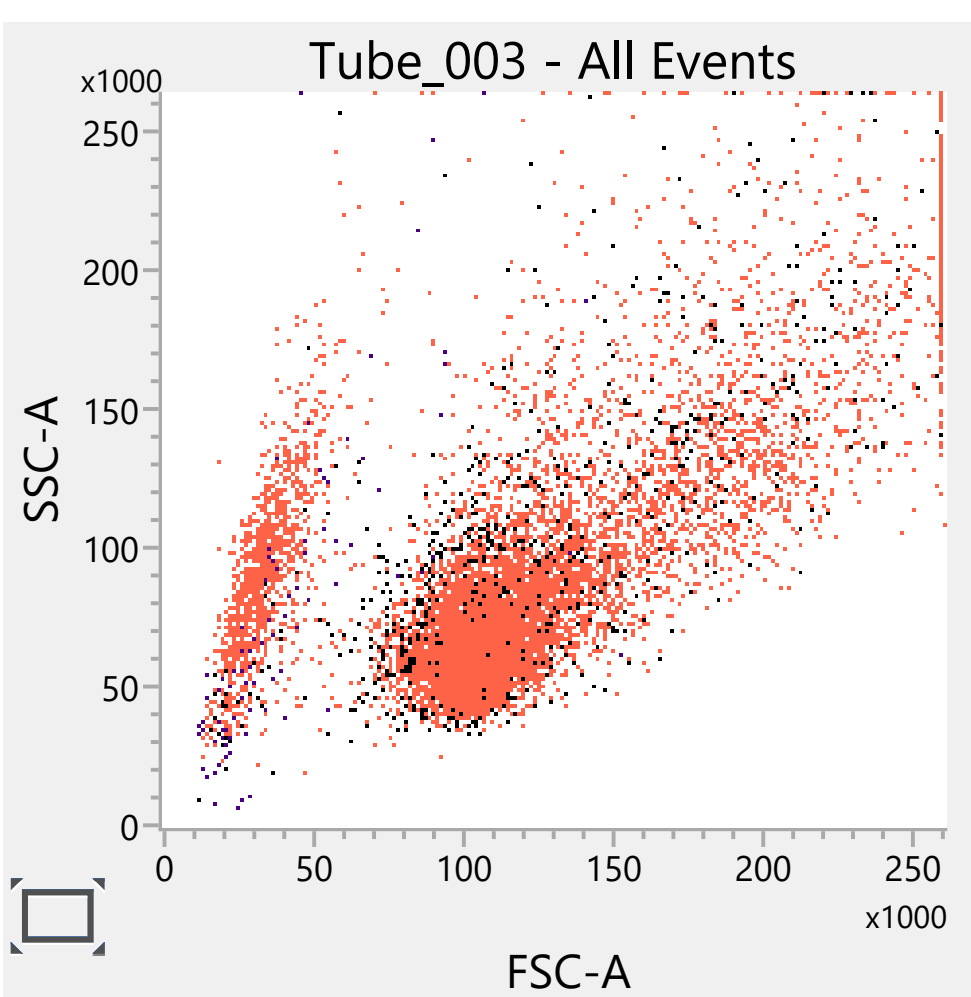

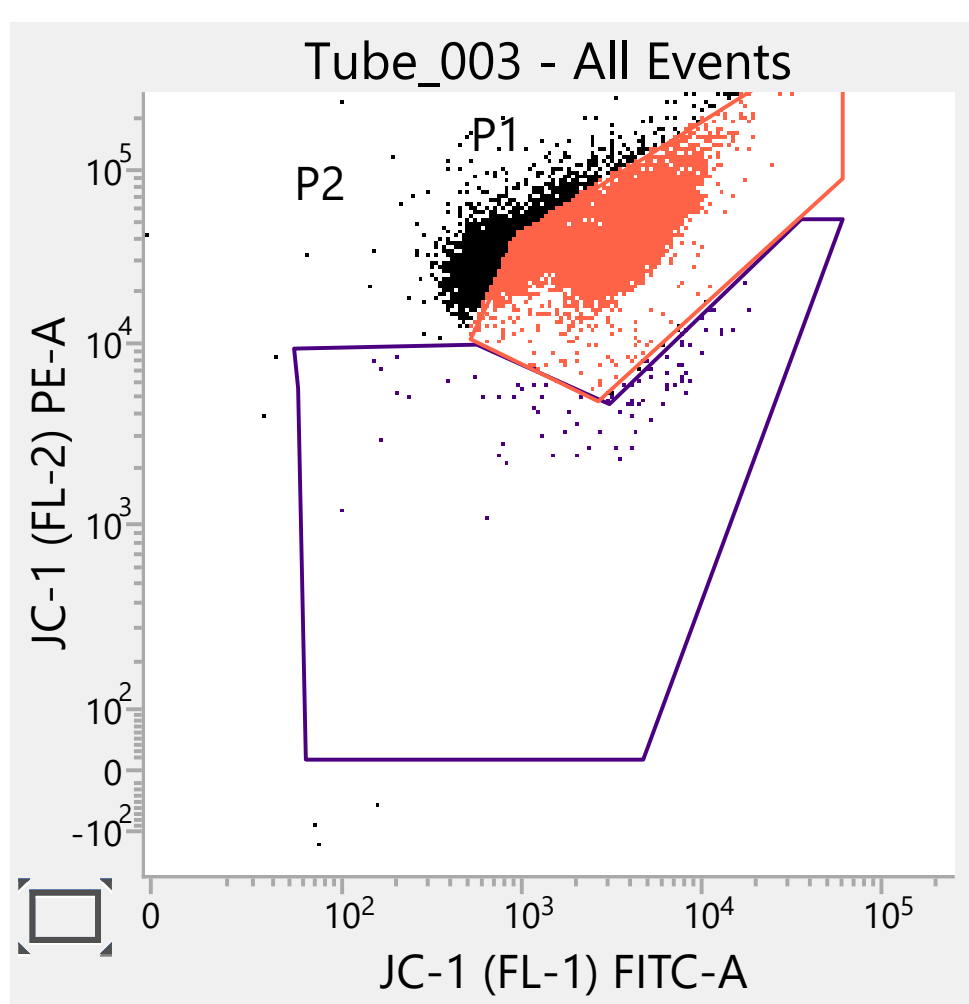

Statistics

Name	Tube_001:All Events	Tube_001:P2	Tube_001:P1	Tube_002:All Events	Tube_002:P2	Tube_002:P1	Tube_003:All Events	Tube_003:P2	Tube_003:P1
Events	10,000	102	7,906	10,000	94	7,763	10,000	85	7,535
% Parent	***	1.0	79.1	***	0.9	77.6	***	0.9	75.4
% Grandparent	***	***	***	***	***	***	***	***	***
% Total	100.0	1.0	79.1	100.0	0.9	77.6	100.0	0.9	75.4

JC-1 Assay

Sample ID: N6_K

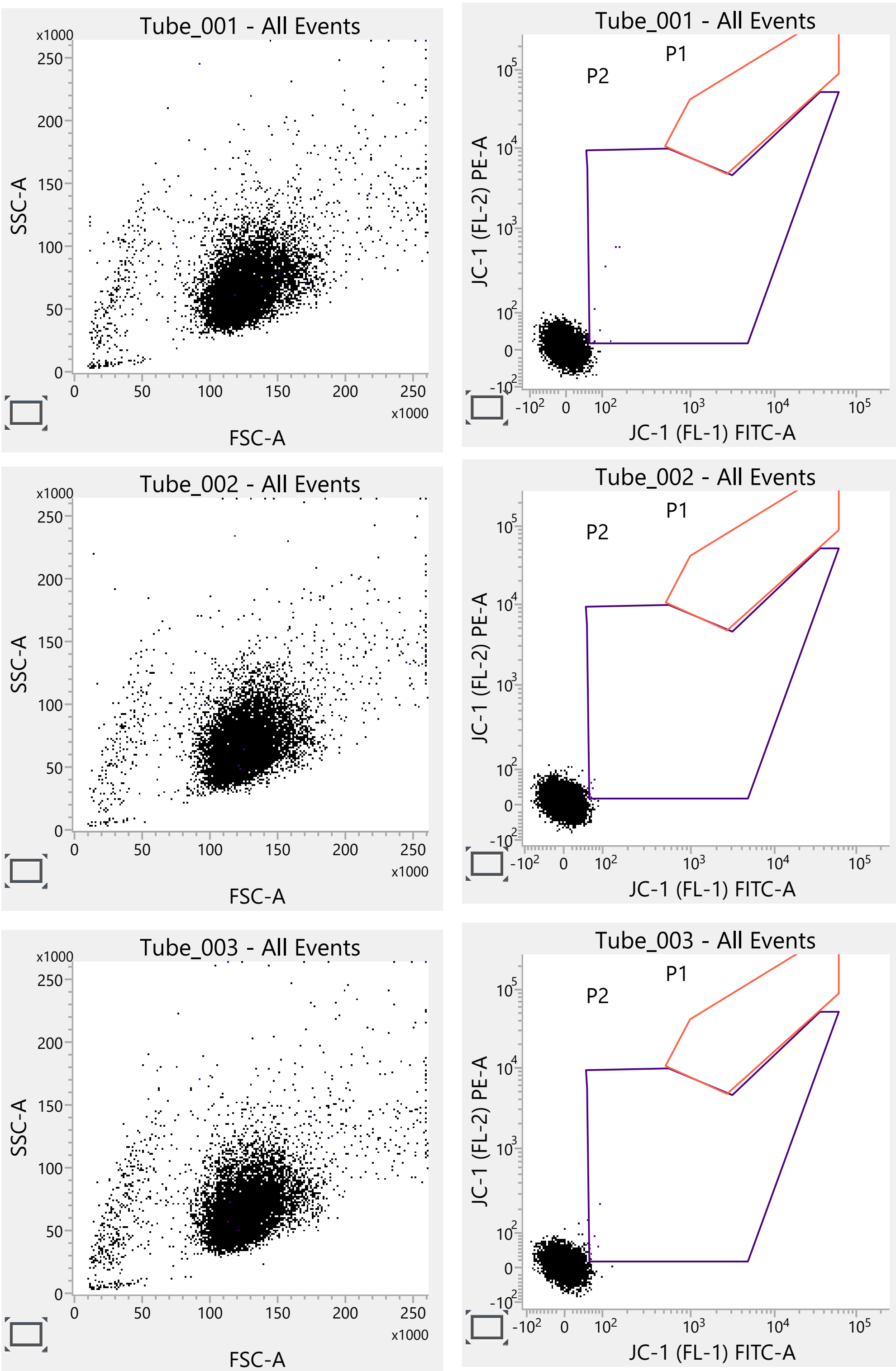

Statistics

Name	Tube_001:All Events	Tube_001:P2	Tube_001:P1	Tube_002:All Events	Tube_002:P2	Tube_002:P1	Tube_003:All Events	Tube_003:P2	Tube_003:P1
Events	10,000	13	0	10,000	8	0	10,000	10	0
% Parent	***	0.1	0.0	***	0.1	0.0	***	0.1	0.0
% Grandparent	***	***	***	***	***	***	***	***	***
% Total	100.0	0.1	0.0	100.0	0.1	0.0	100.0	0.1	0.0

JC-1 Assay

Sample ID: N6_K+

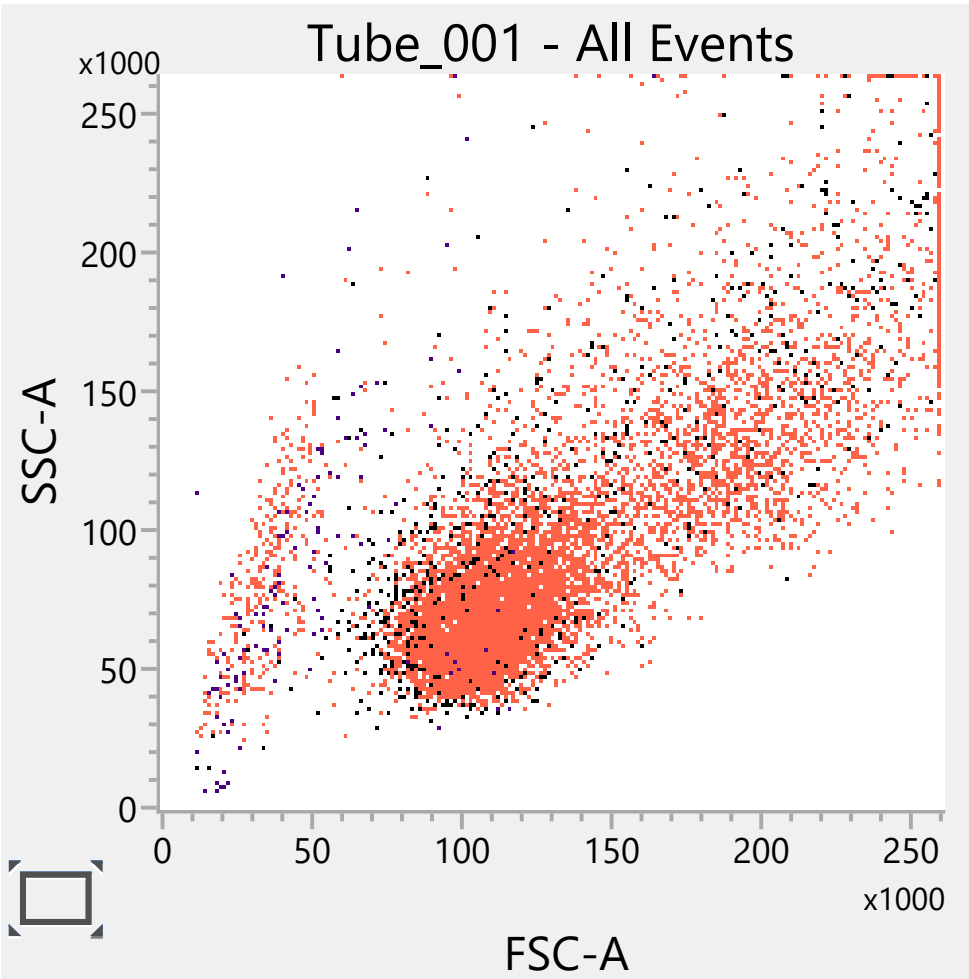

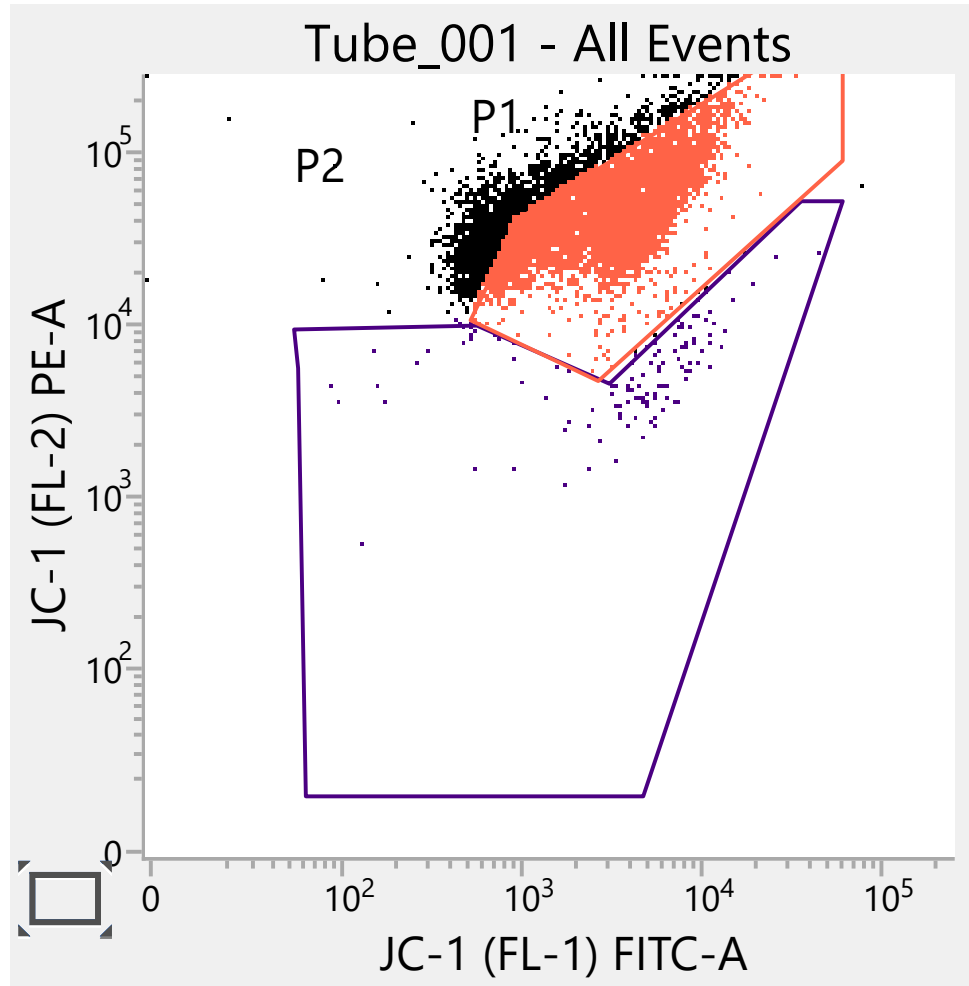

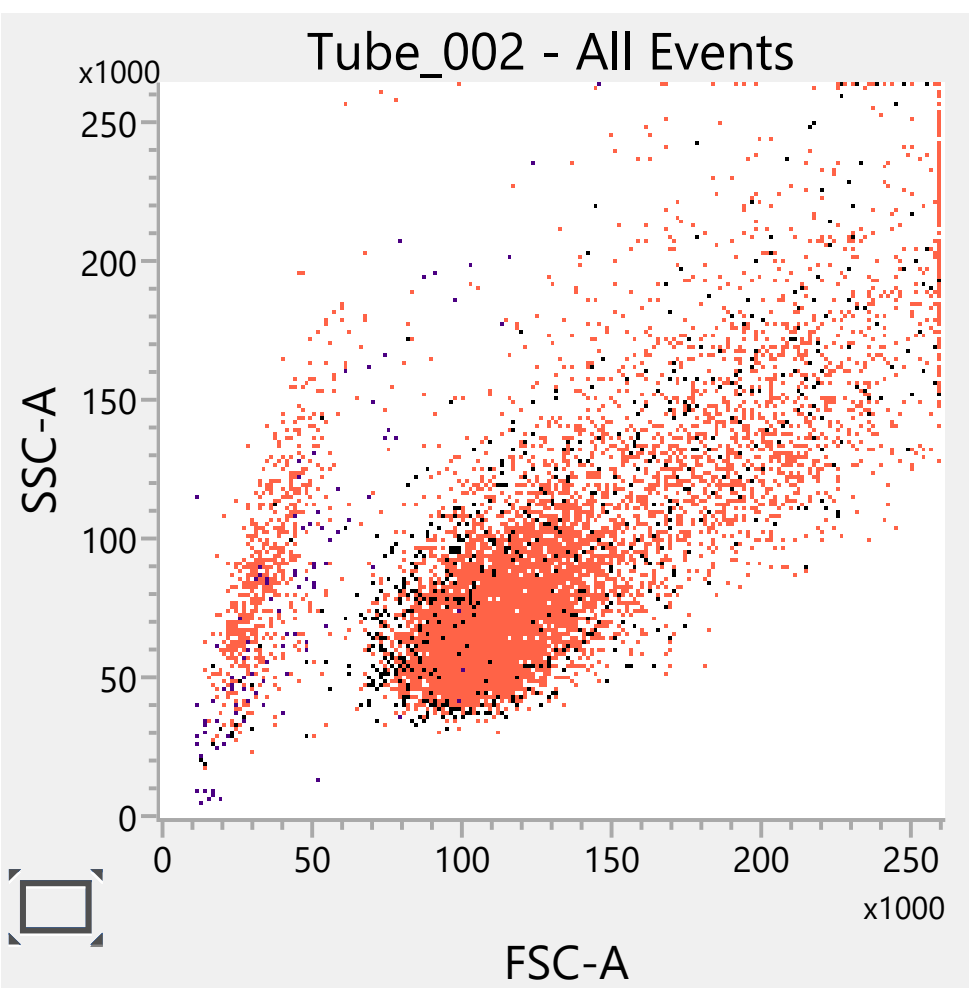

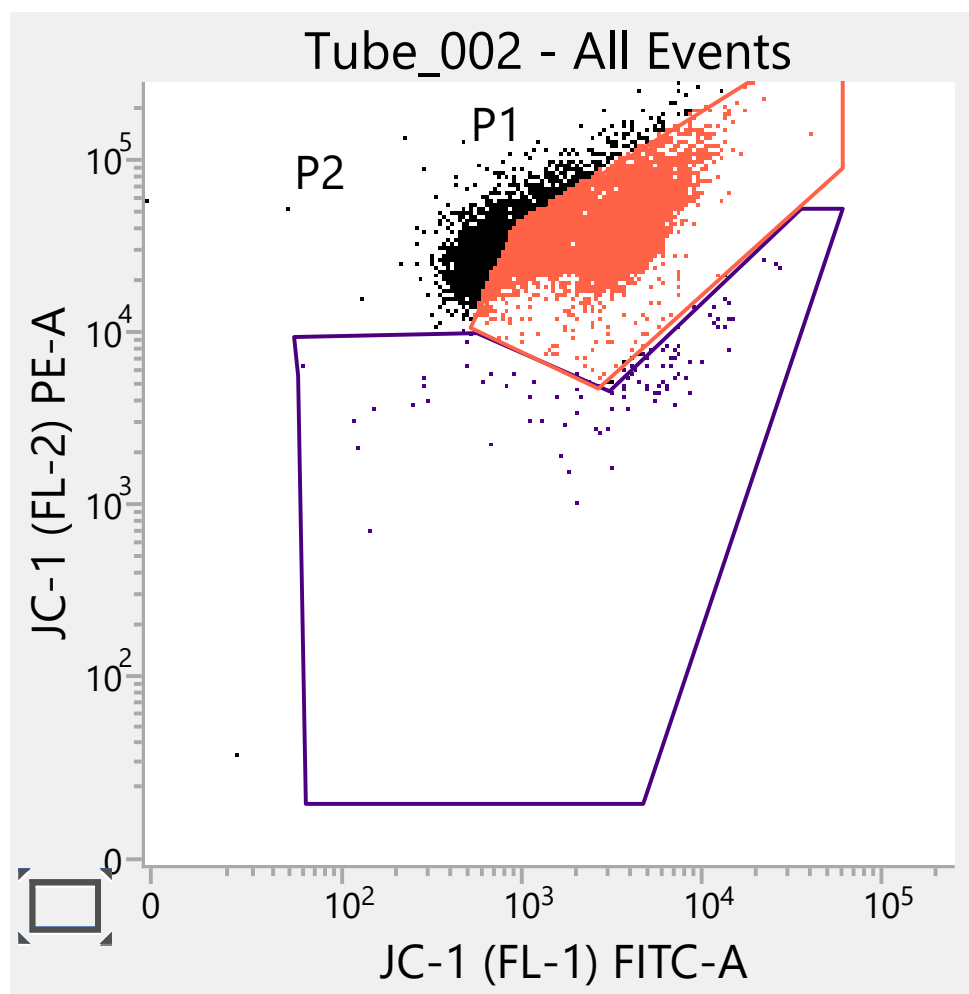

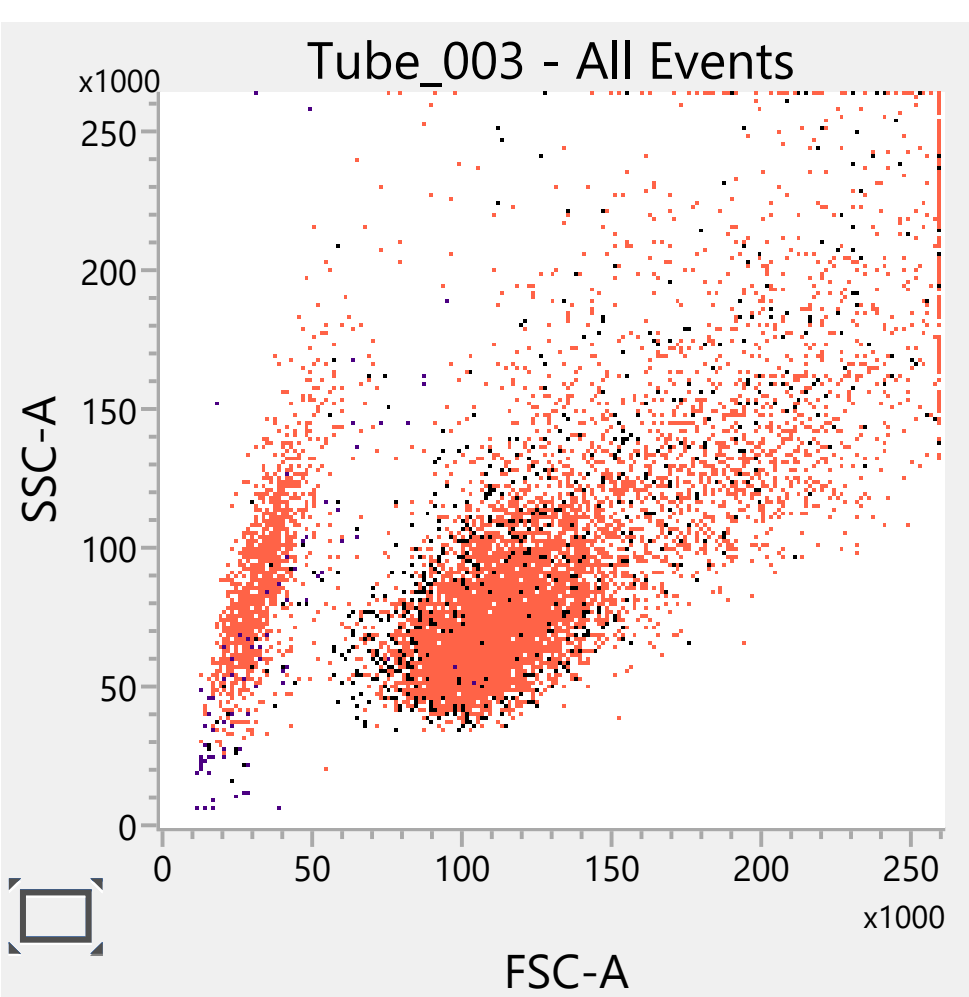

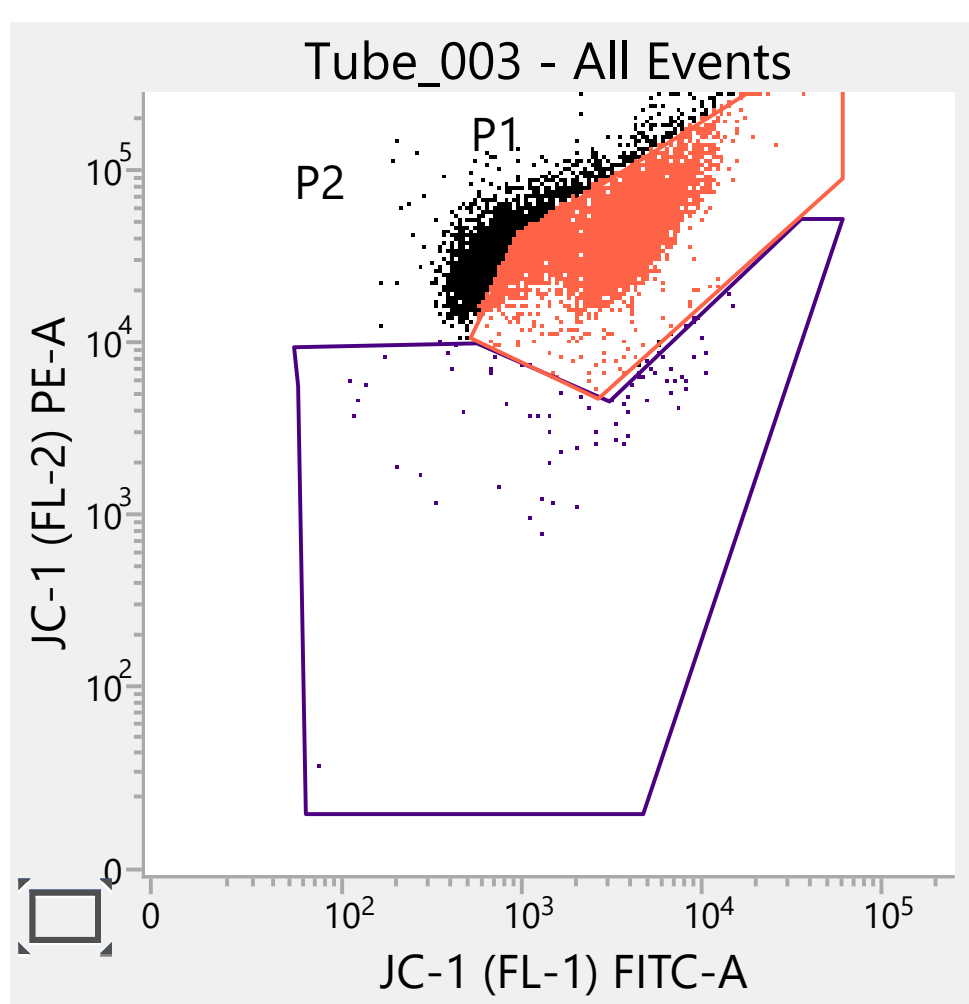

Statistics

Name	Tube_001:All Events	Tube_001:P2	Tube_001:P1	Tube_002:All Events	Tube_002:P2	Tube_002:P1	Tube_003:All Events	Tube_003:P2	Tube_003:P1
Events	10,000	122	7,511	10,000	100	7,295	10,000	77	7,322
% Parent	***	1.2	75.1	***	1.0	73.0	***	0.8	73.2
% Grandparent	***	***	***	***	***	***	***	***	***
% Total	100.0	1.2	75.1	100.0	1.0	73.0	100.0	0.8	73.2

JC-1 Assay

Sample ID: N6_0,5

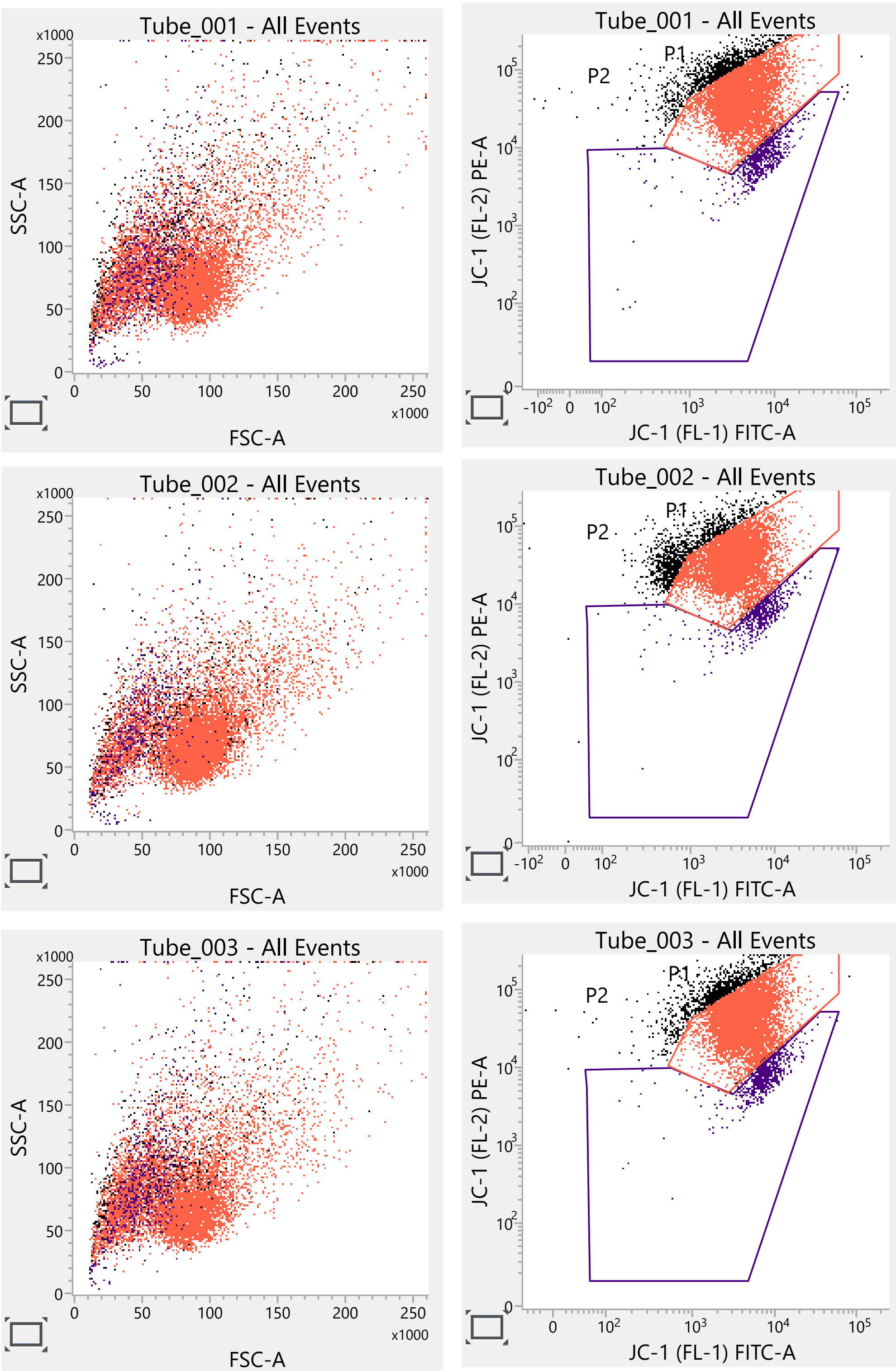

Statistics

Name	Tube_001:All Events	Tube_001:P2	Tube_001:P1	Tube_002:All Events	Tube_002:P2	Tube_002:P1	Tube_003:All Events	Tube_003:P2	Tube_003:P1
Events	10,000	631	8,280	10,000	531	8,352	10,000	643	8,336
% Parent	***	6.3	82.8	***	5.3	83.5	***	6.4	83.4
% Grandparent	***	***	***	***	***	***	***	***	***
% Total	100.0	6.3	82.8	100.0	5.3	83.5	100.0	6.4	83.4

JC-1 Assay

Sample ID: N6_1

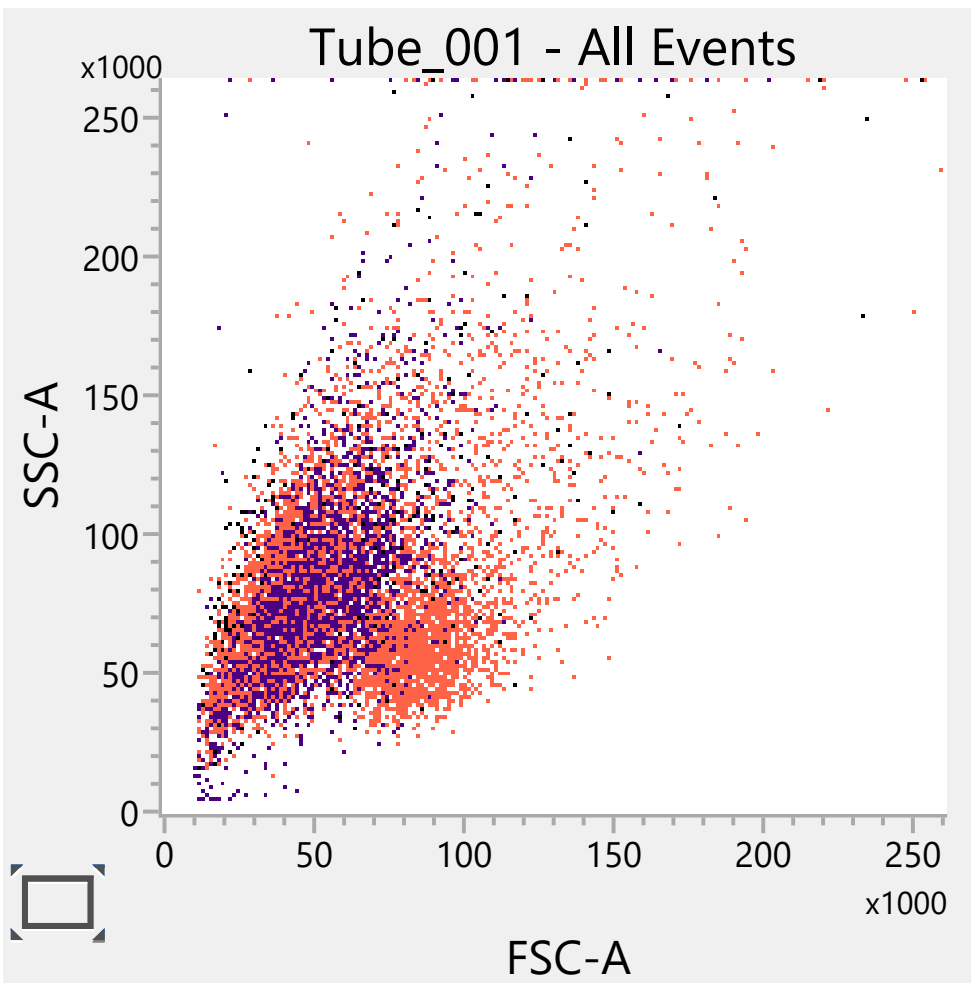

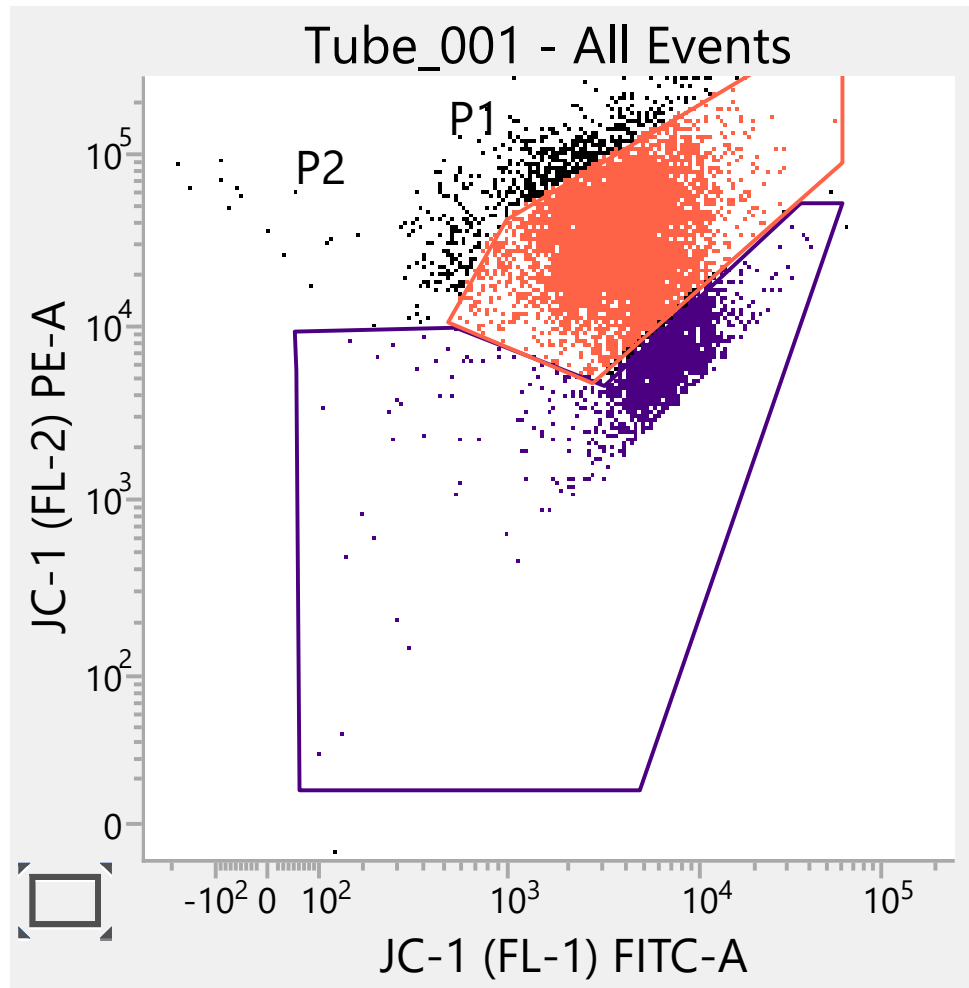

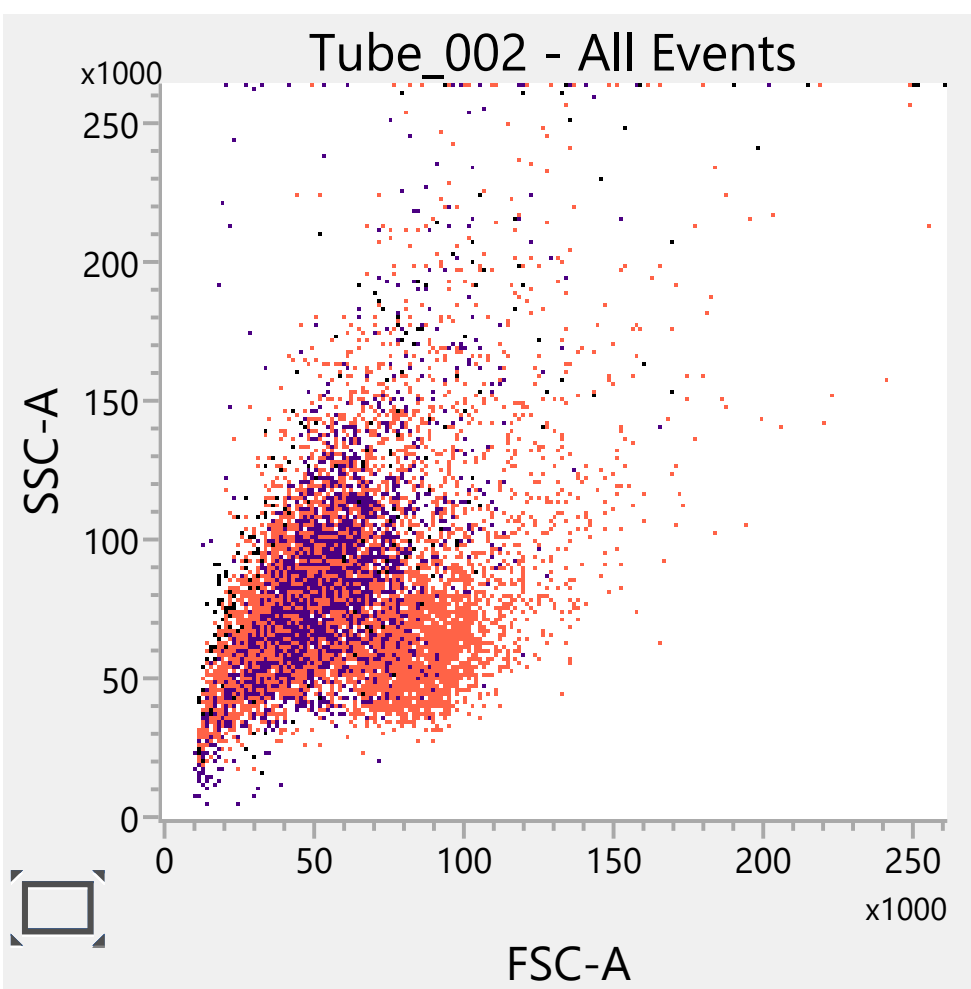

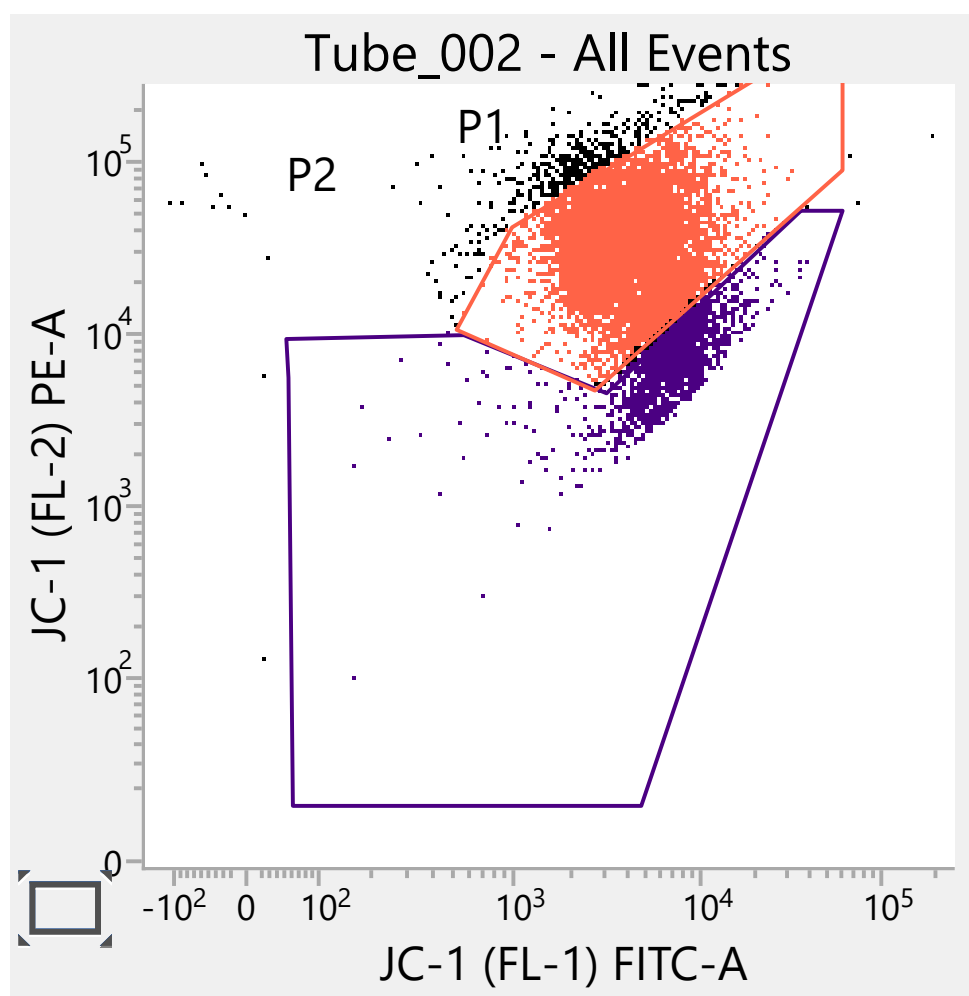

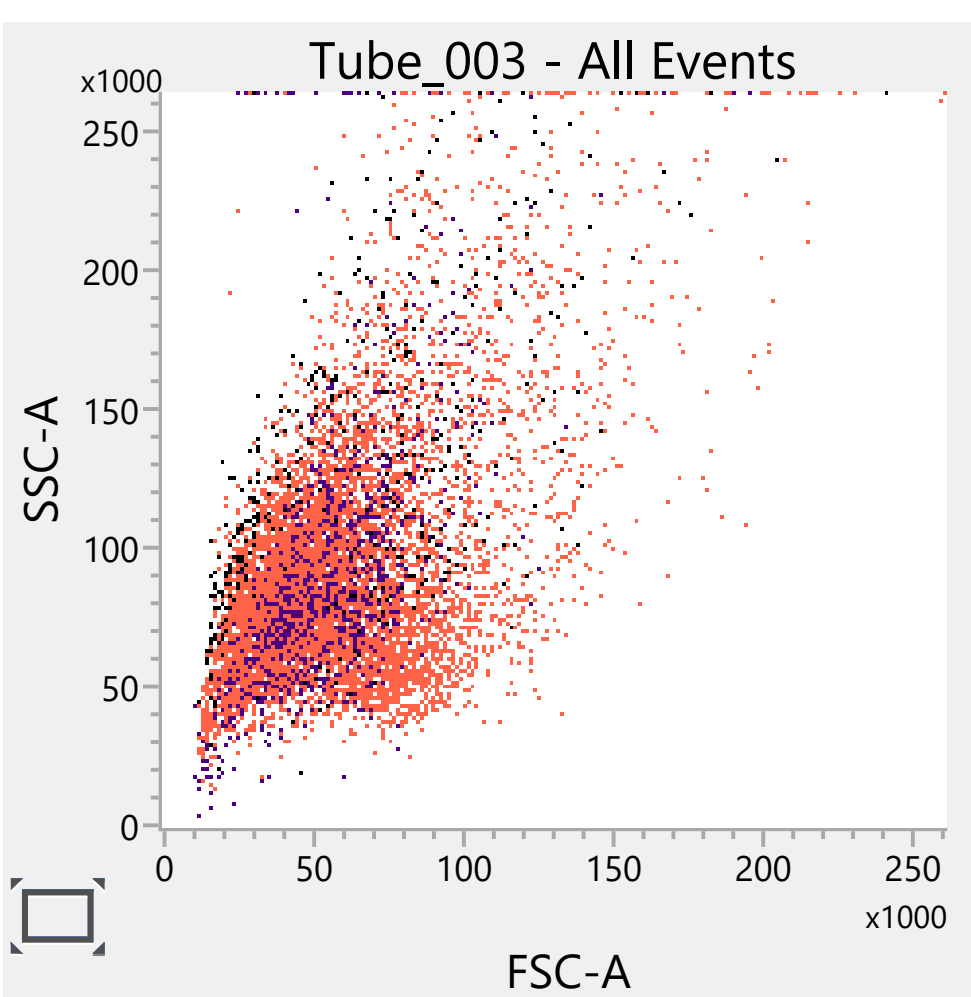

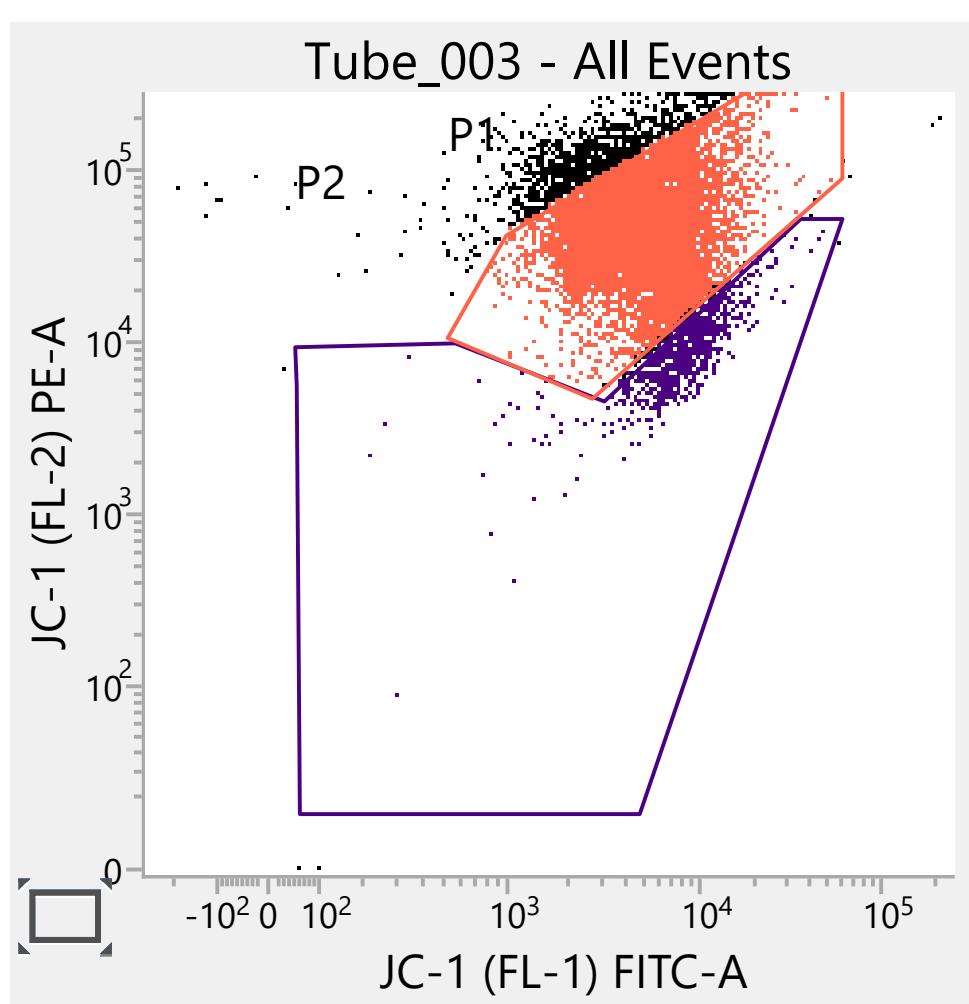

Statistics

Name	Tube_001:All Events	Tube_001:P2	Tube_001:P1	Tube_002:All Events	Tube_002:P2	Tube_002:P1	Tube_003:All Events	Tube_003:P2	Tube_003:P1
Events	10,000	1,863	7,335	10,000	1,656	7,719	10,000	845	7,905
% Parent	***	18.6	73.4	***	16.6	77.2	***	8.5	79.1
% Grandparent	***	***	***	***	***	***	***	***	***
% Total	100.0	18.6	73.4	100.0	16.6	77.2	100.0	8.5	79.1

JC-1 Assay

Sample ID: N6_2,5

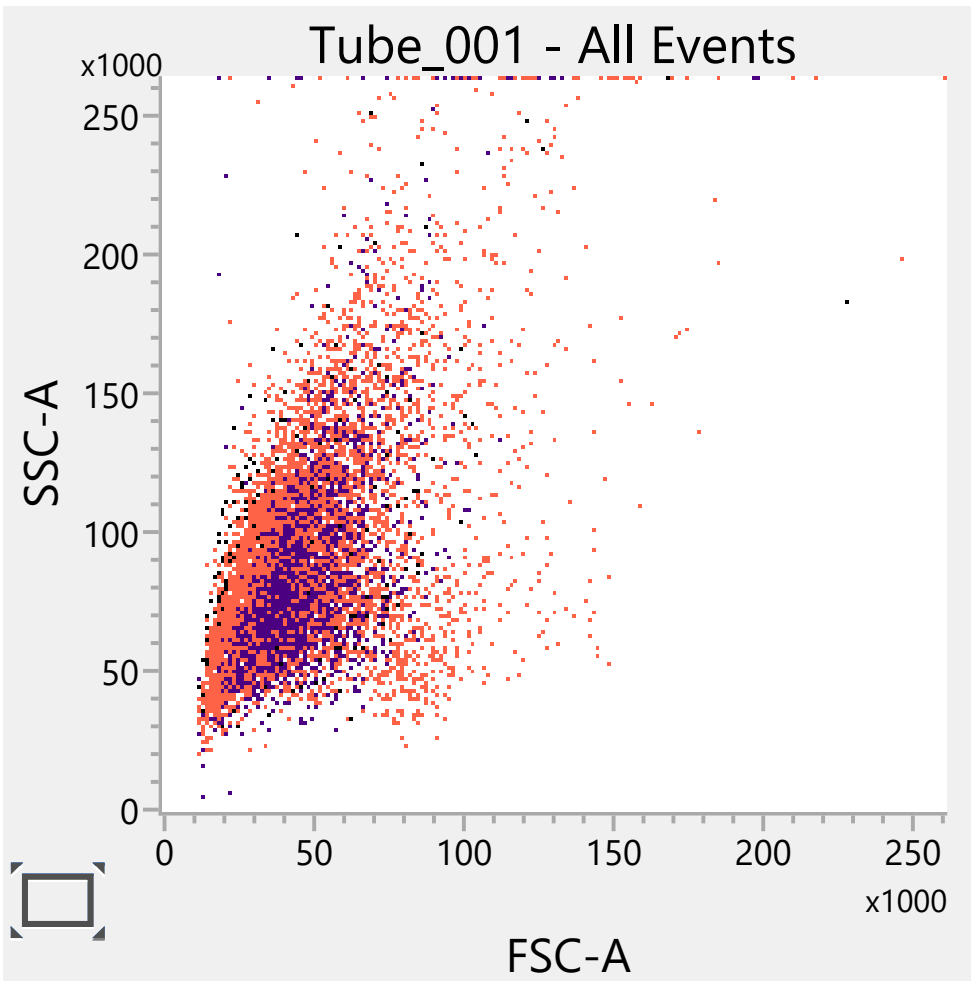

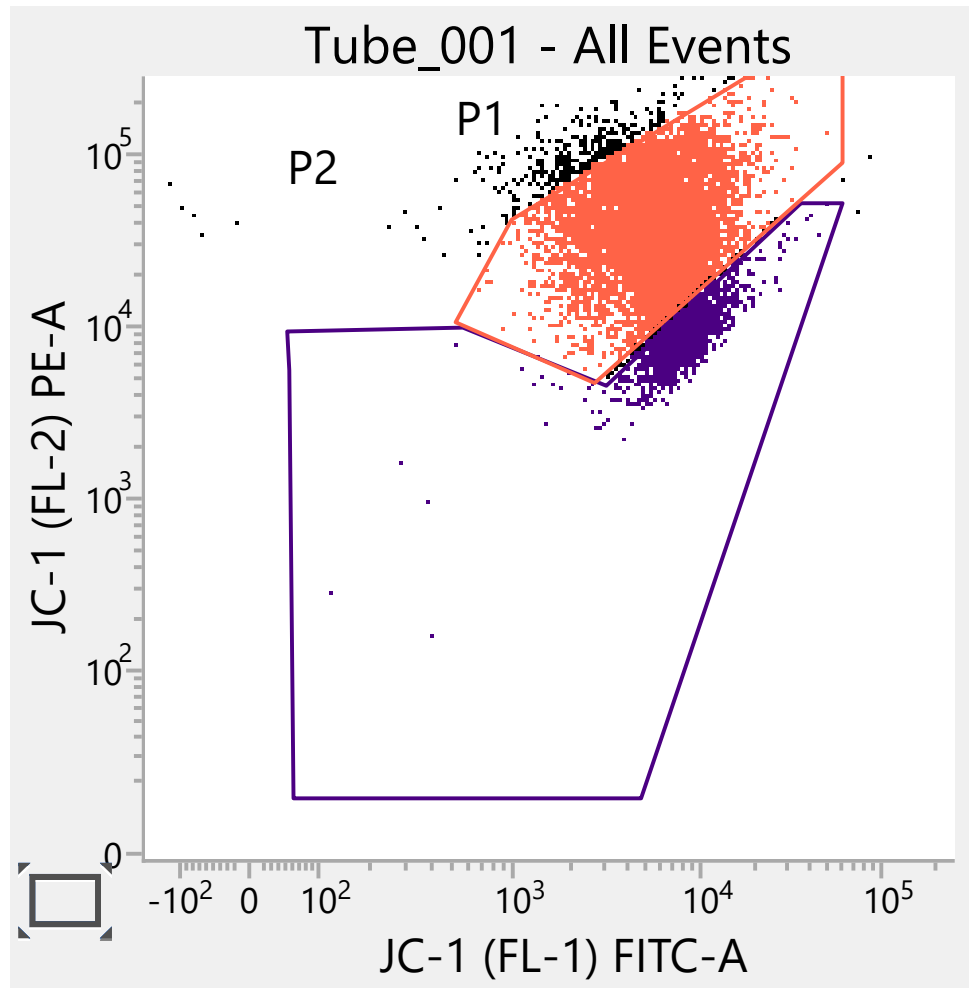

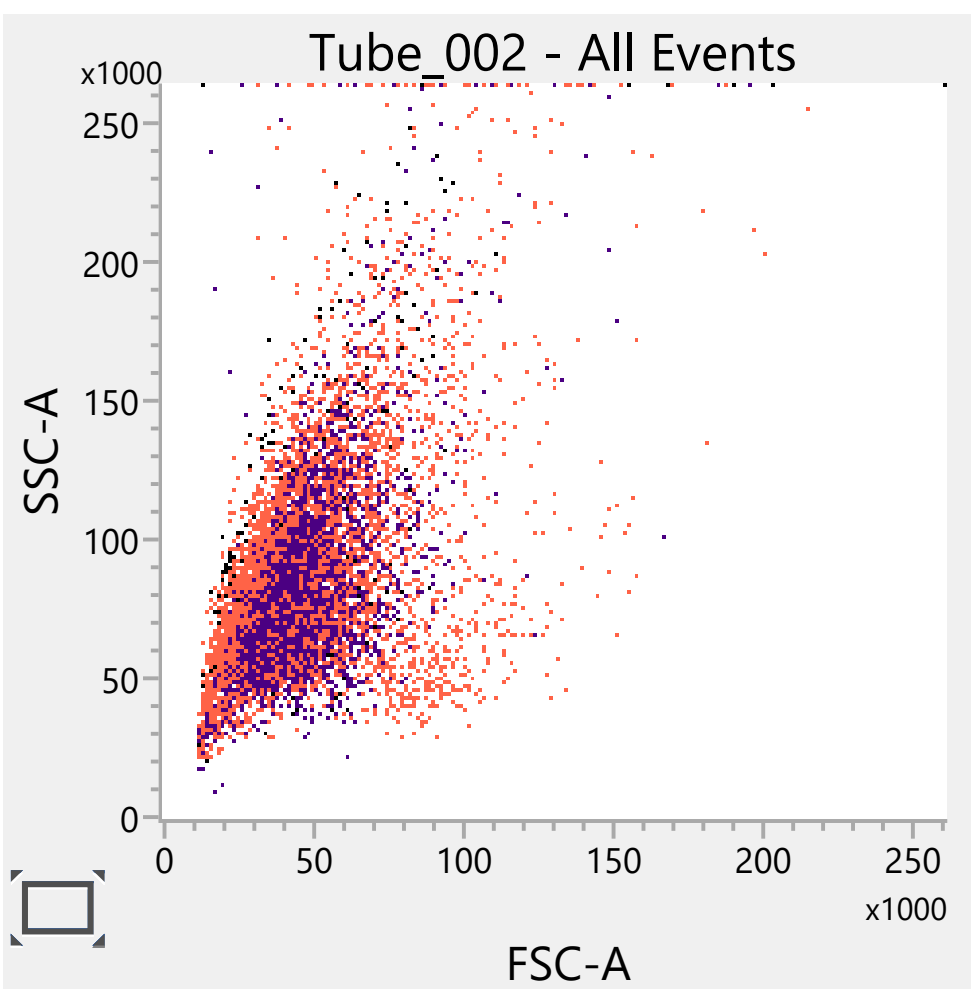

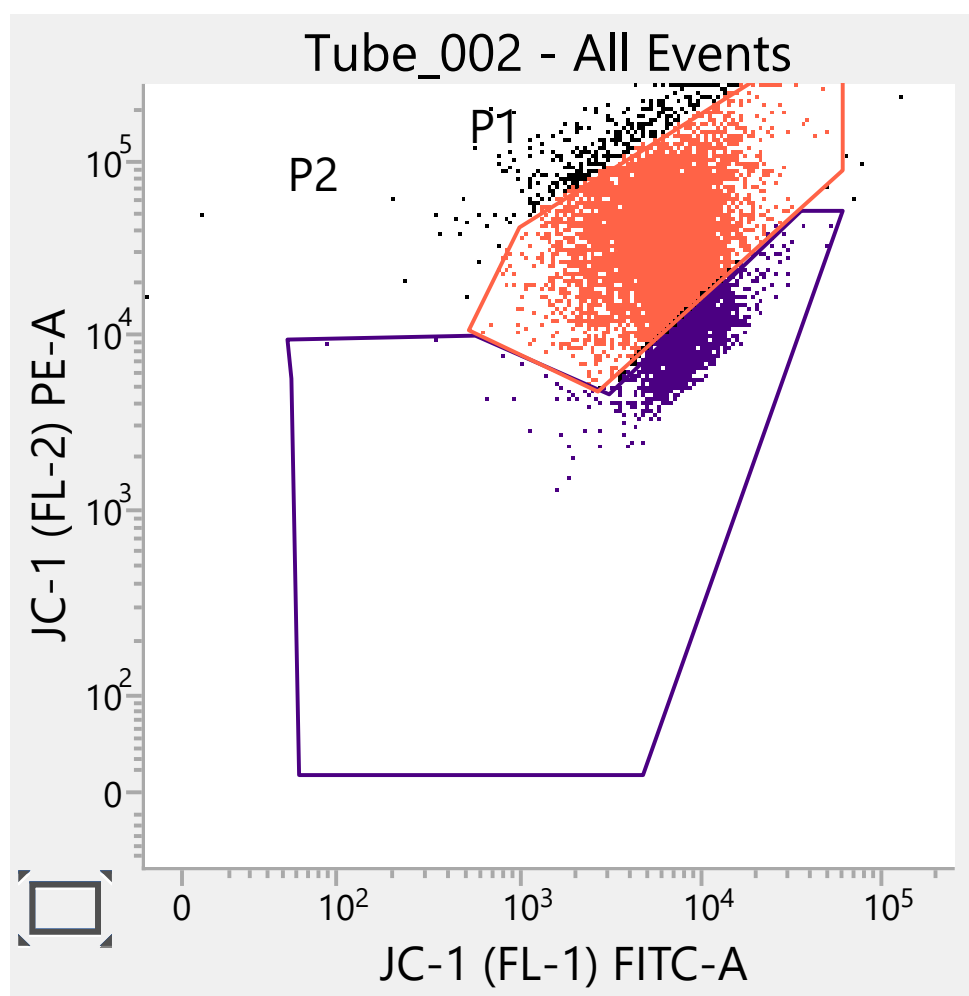

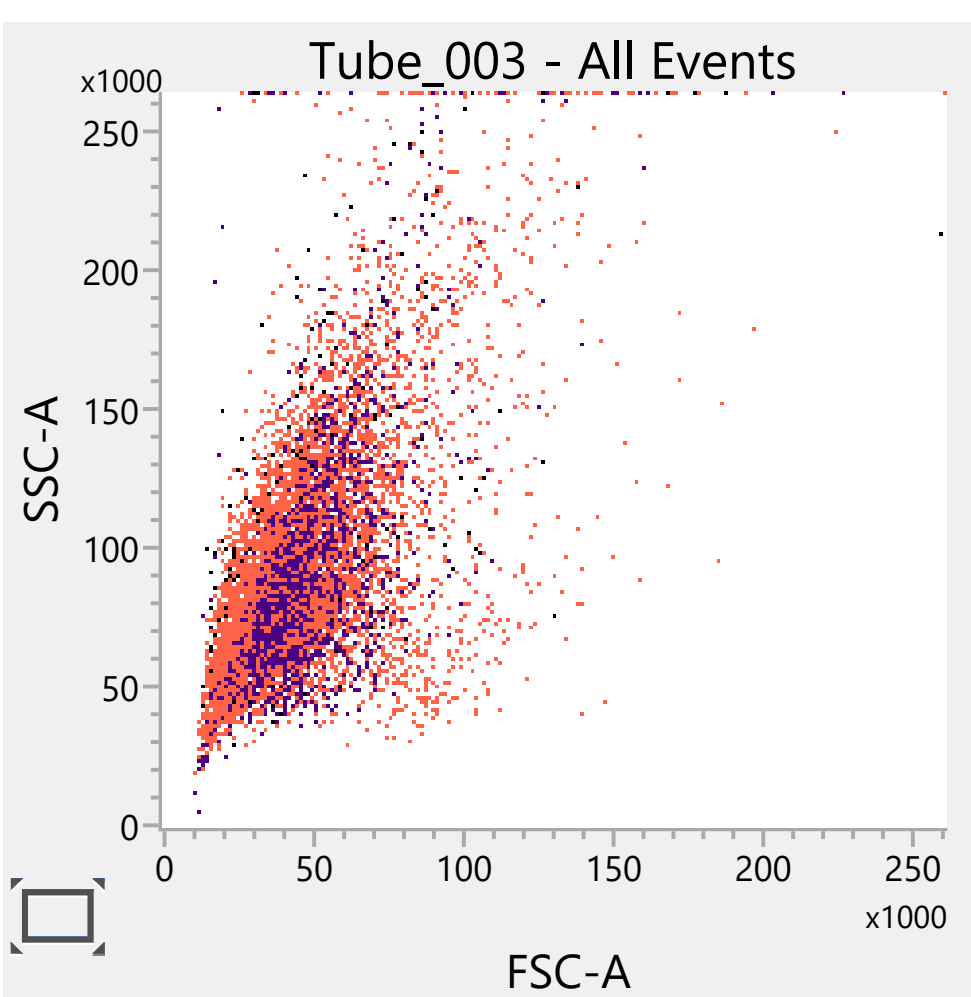

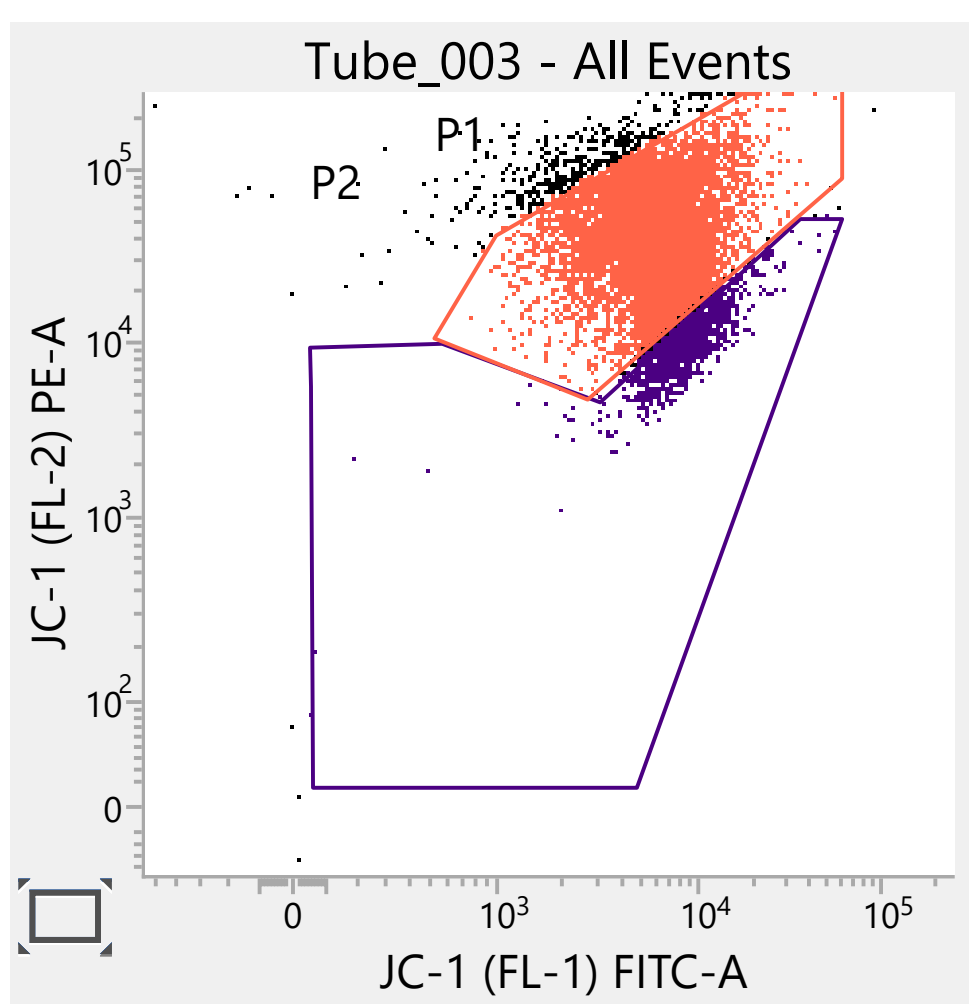

Statistics

Name	Tube_001:All Events	Tube_001:P2	Tube_001:P1	Tube_002:All Events	Tube_002:P2	Tube_002:P1	Tube_003:All Events	Tube_003:P2	Tube_003:P1
Events	10,000	1,416	7,992	10,000	1,756	7,642	10,000	1,257	8,122
% Parent	***	14.2	79.9	***	17.6	76.4	***	12.6	81.2
% Grandparent	***	***	***	***	***	***	***	***	***
% Total	100.0	14.2	79.9	100.0	17.6	76.4	100.0	12.6	81.2

JC-1 Assay

Sample ID: N6_5

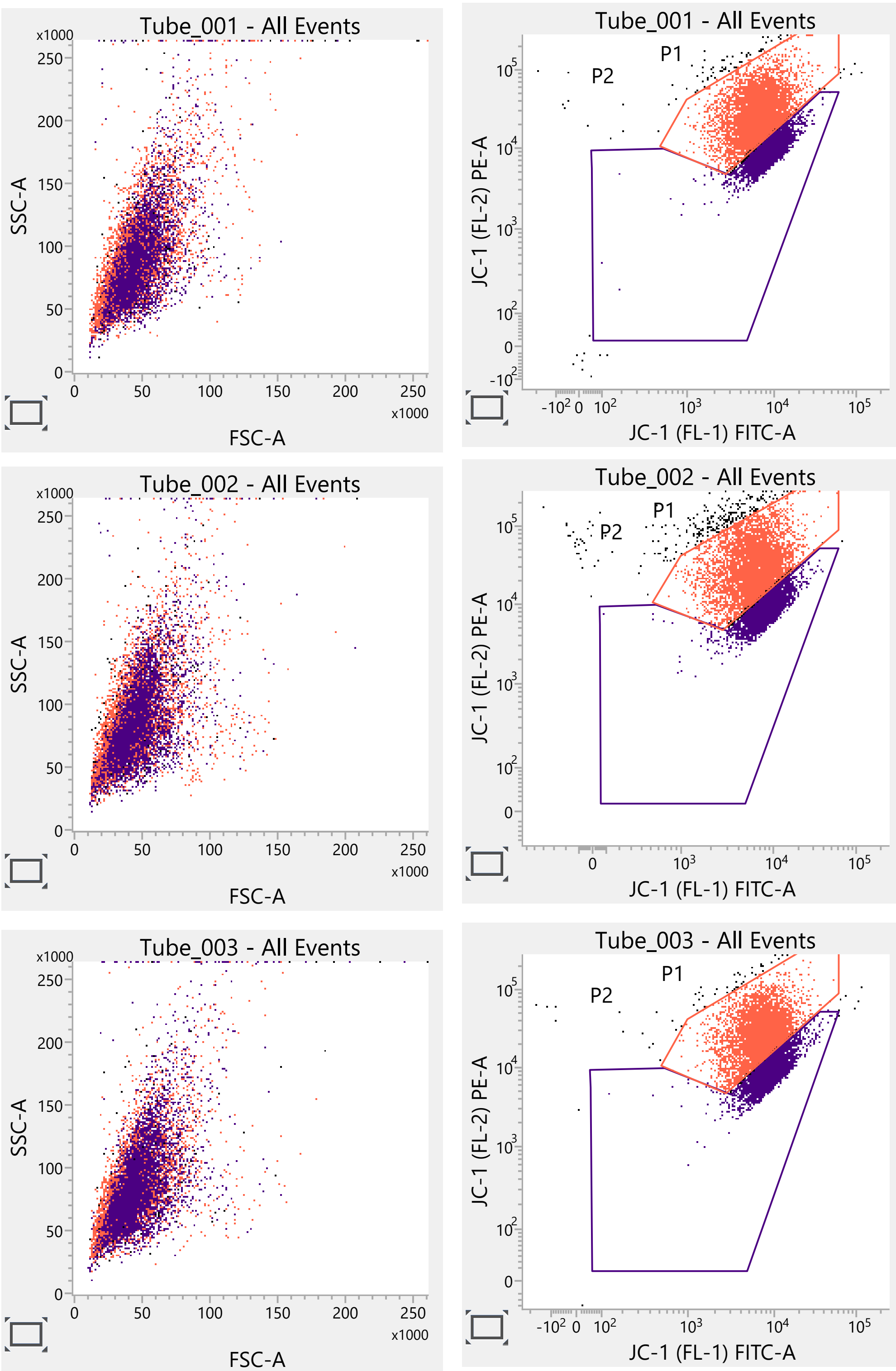

Statistics

Name	Tube_001:All Events	Tube_001:P2	Tube_001:P1	Tube_002:All Events	Tube_002:P2	Tube_002:P1	Tube_003:All Events	Tube_003:P2	Tube_003:P1
Events	10,000	3,301	6,146	10,000	3,703	5,627	10,000	4,441	5,152
% Parent	***	33.0	61.5	***	37.0	56.3	***	44.4	51.5
% Grandparent	***	***	***	***	***	***	***	***	***
% Total	100.0	33.0	61.5	100.0	37.0	56.3	100.0	44.4	51.5

JC-1 Assay

Sample ID: N6_CCCP

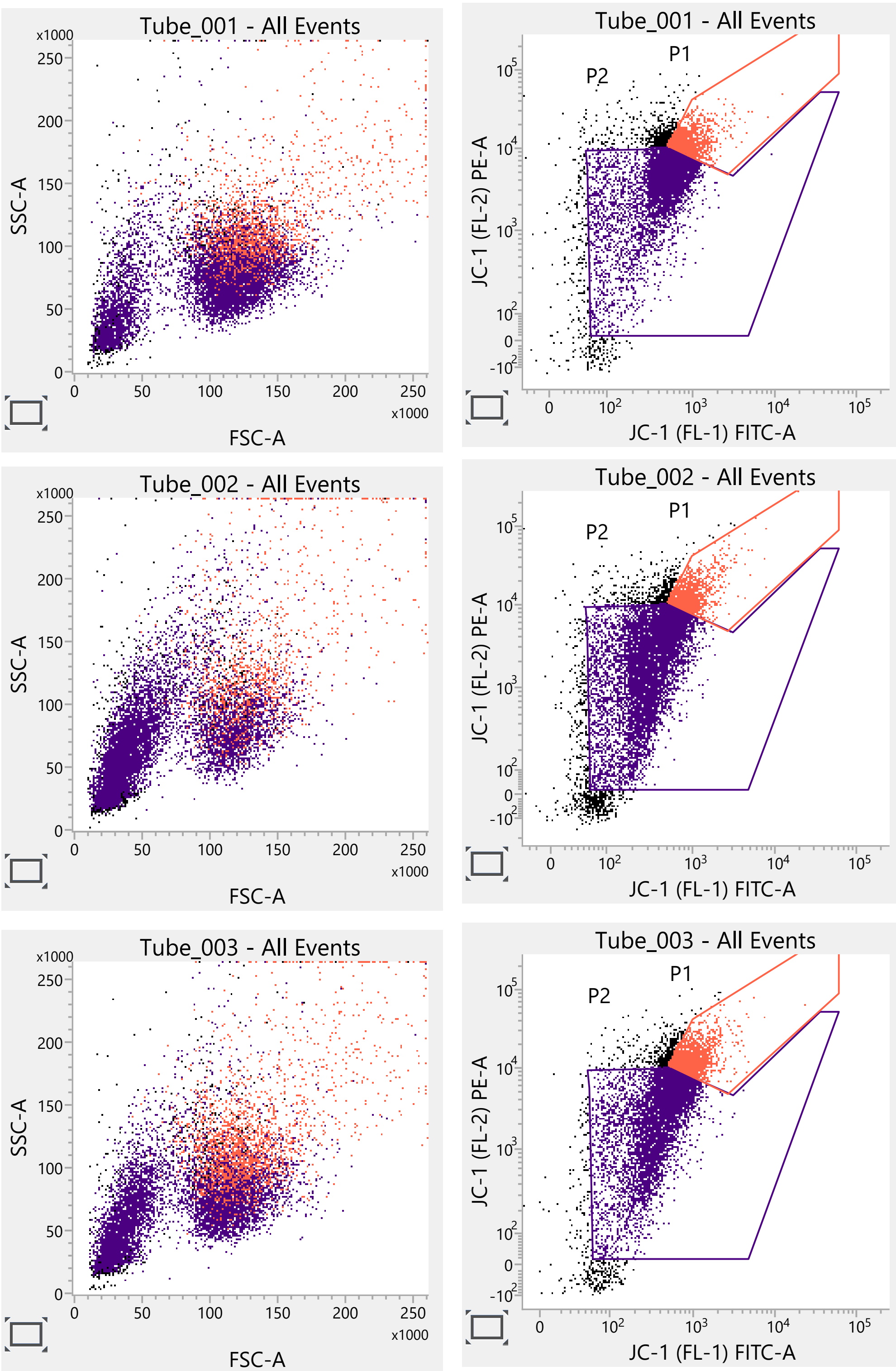

Statistics

Name	Tube_001:All Events	Tube_001:P2	Tube_001:P1	Tube_002:All Events	Tube_002:P2	Tube_002:P1	Tube_003:All Events	Tube_003:P2	Tube_003:P1
Events	10,000	7,815	1,473	10,000	7,853	1,131	10,000	7,355	1,961
% Parent	***	78.1	14.7	***	78.5	11.3	***	73.6	19.6
% Grandparent	***	***	***	***	***	***	***	***	***
% Total	100.0	78.1	14.7	100.0	78.5	11.3	100.0	73.6	19.6

JC-1 Assay

Sample ID: N6_DMSO

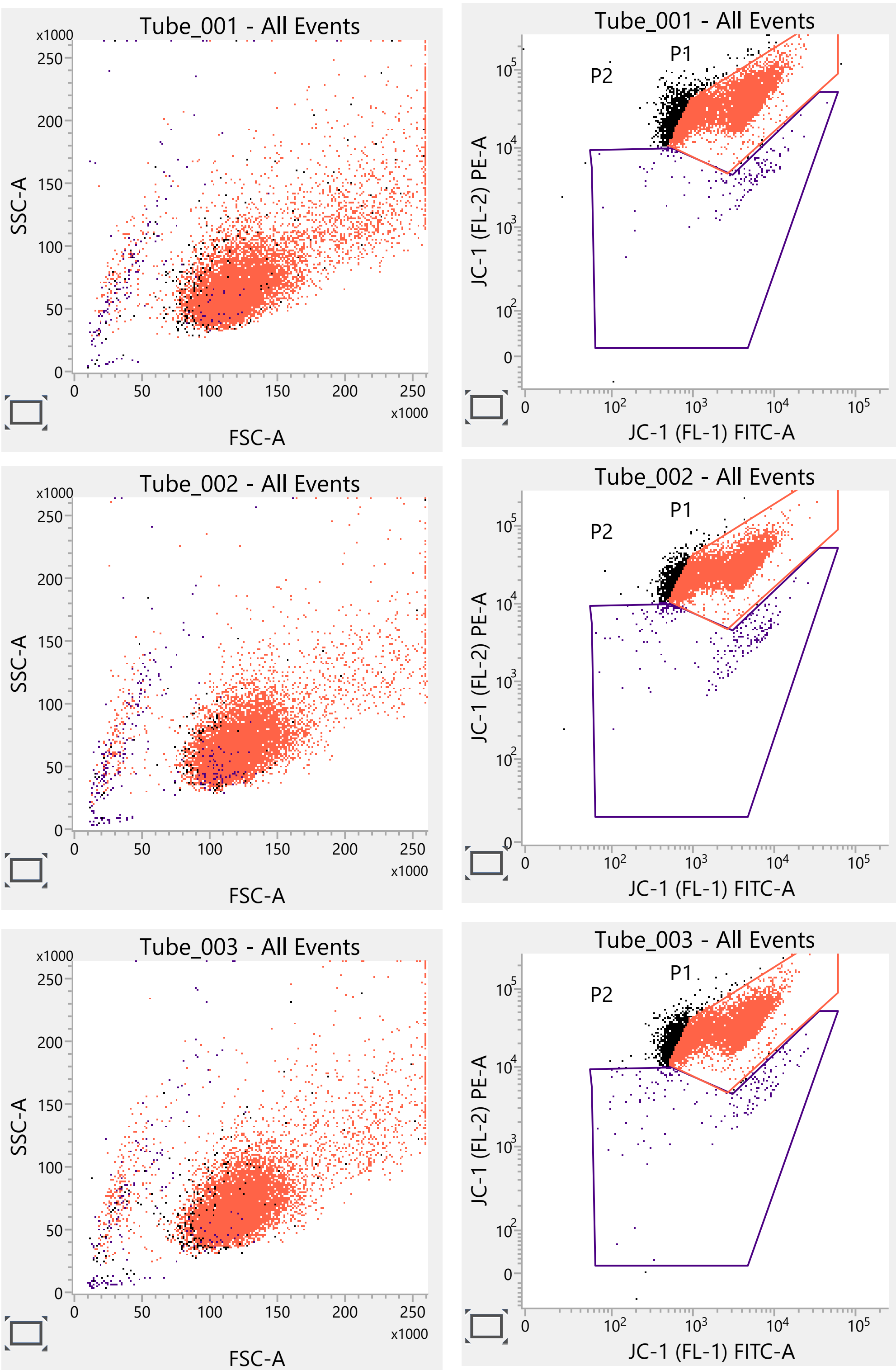

Statistics

Name	Tube_001:All Events	Tube_001:P2	Tube_001:P1	Tube_002:All Events	Tube_002:P2	Tube_002:P1	Tube_003:All Events	Tube_003:P2	Tube_003:P1
Events	10,000	159	8,577	10,000	248	8,882	10,000	179	8,561
% Parent	***	1.6	85.8	***	2.5	88.8	***	1.8	85.6
% Grandparent	***	***	***	***	***	***	***	***	***
% Total	100.0	1.6	85.8	100.0	2.5	88.8	100.0	1.8	85.6

JC-1 Assay

Sample ID: N6_JC1

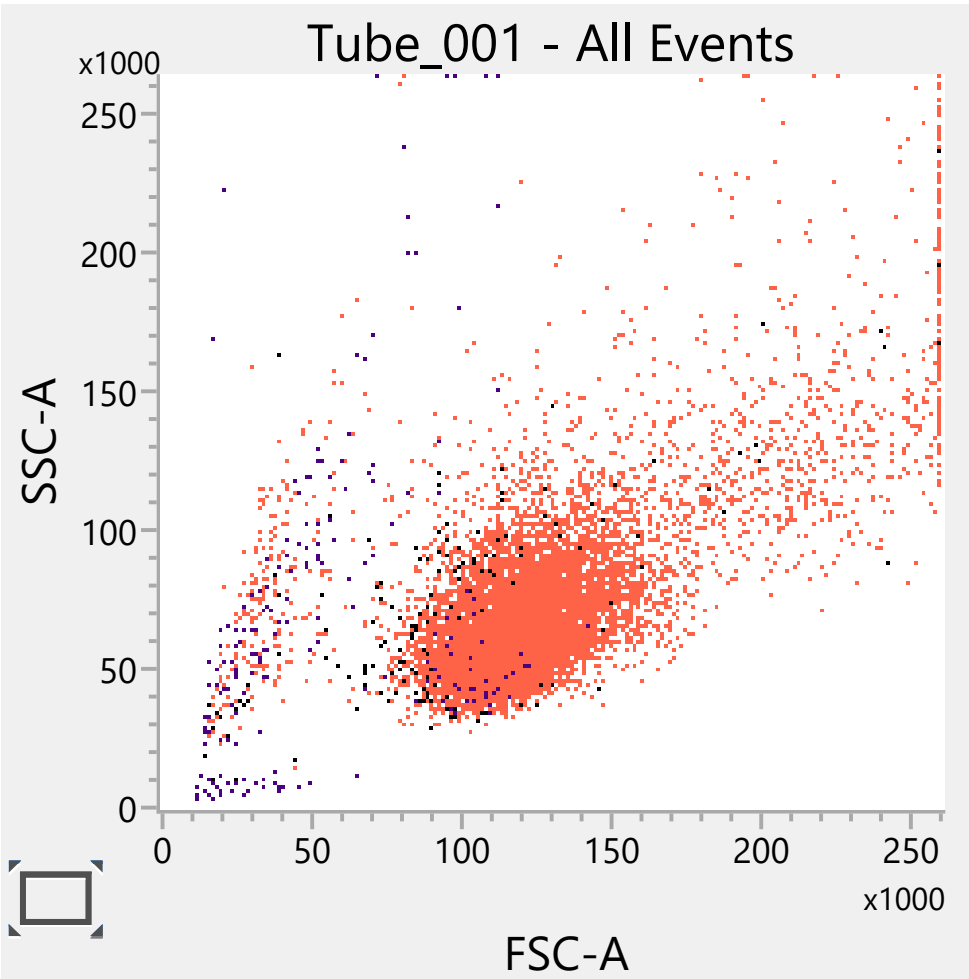

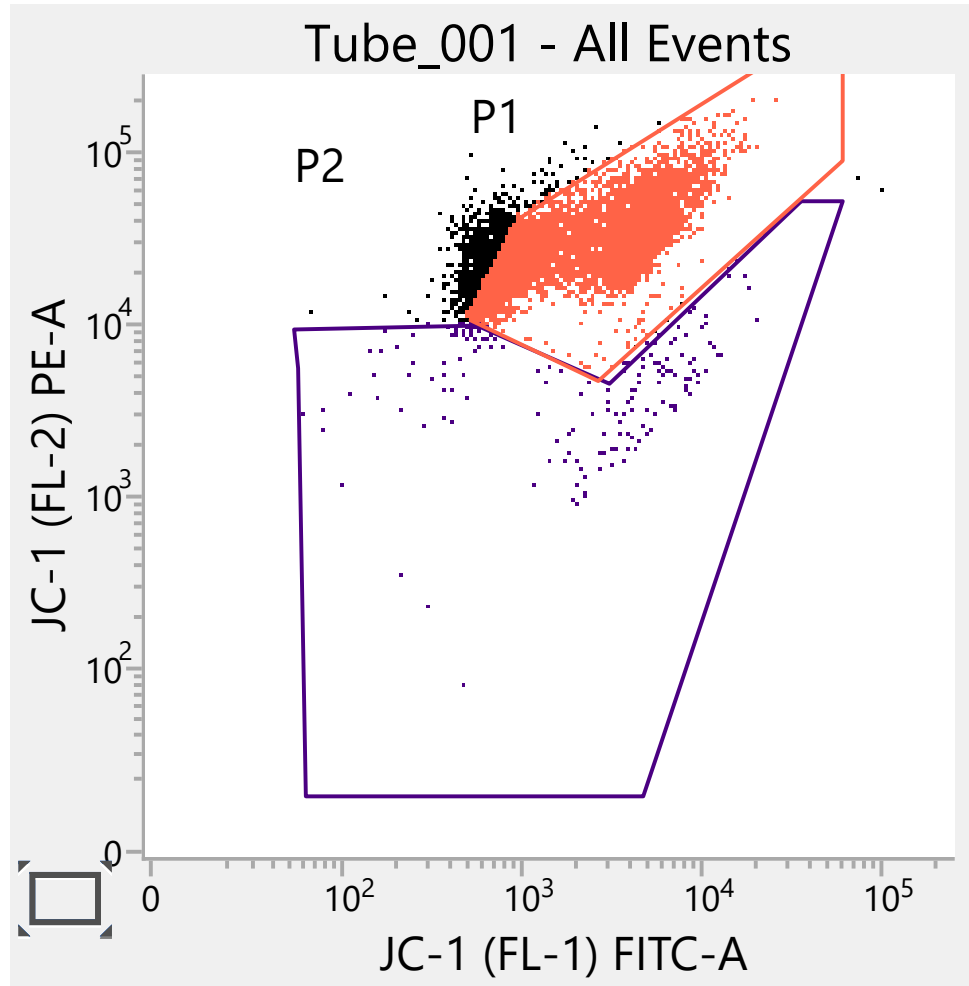

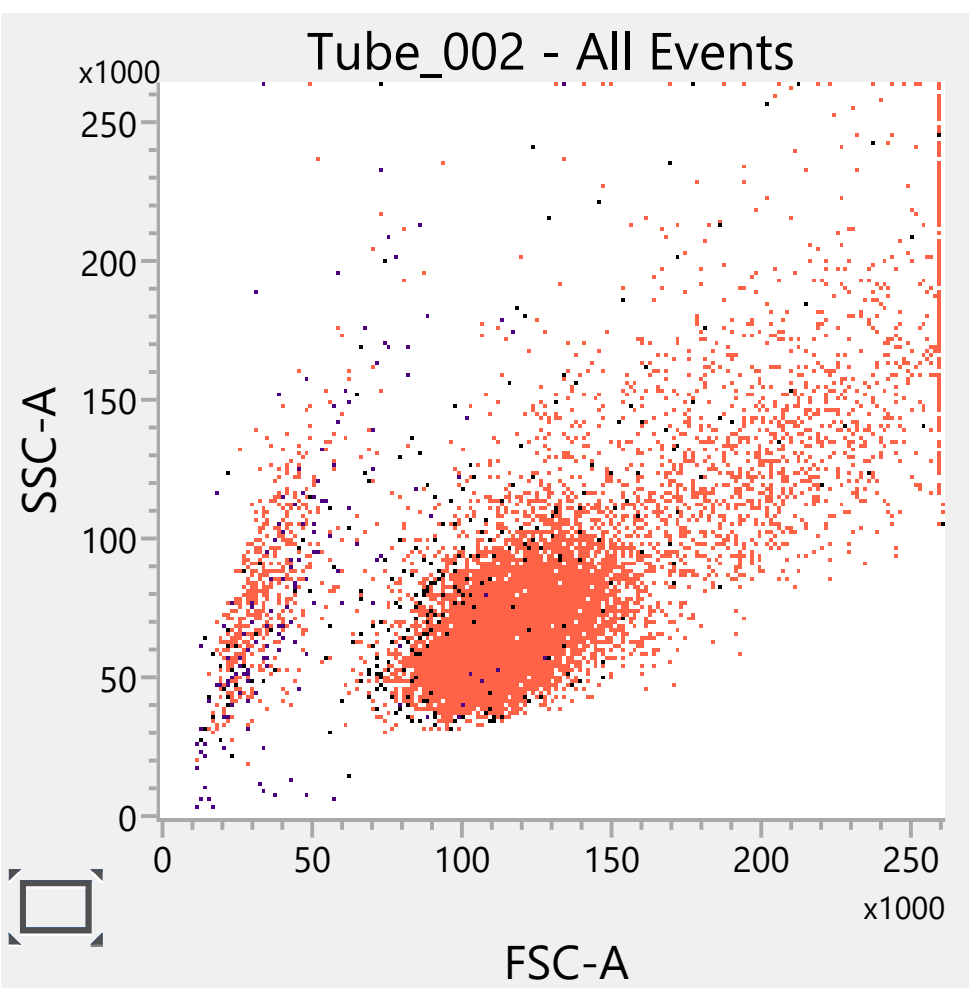

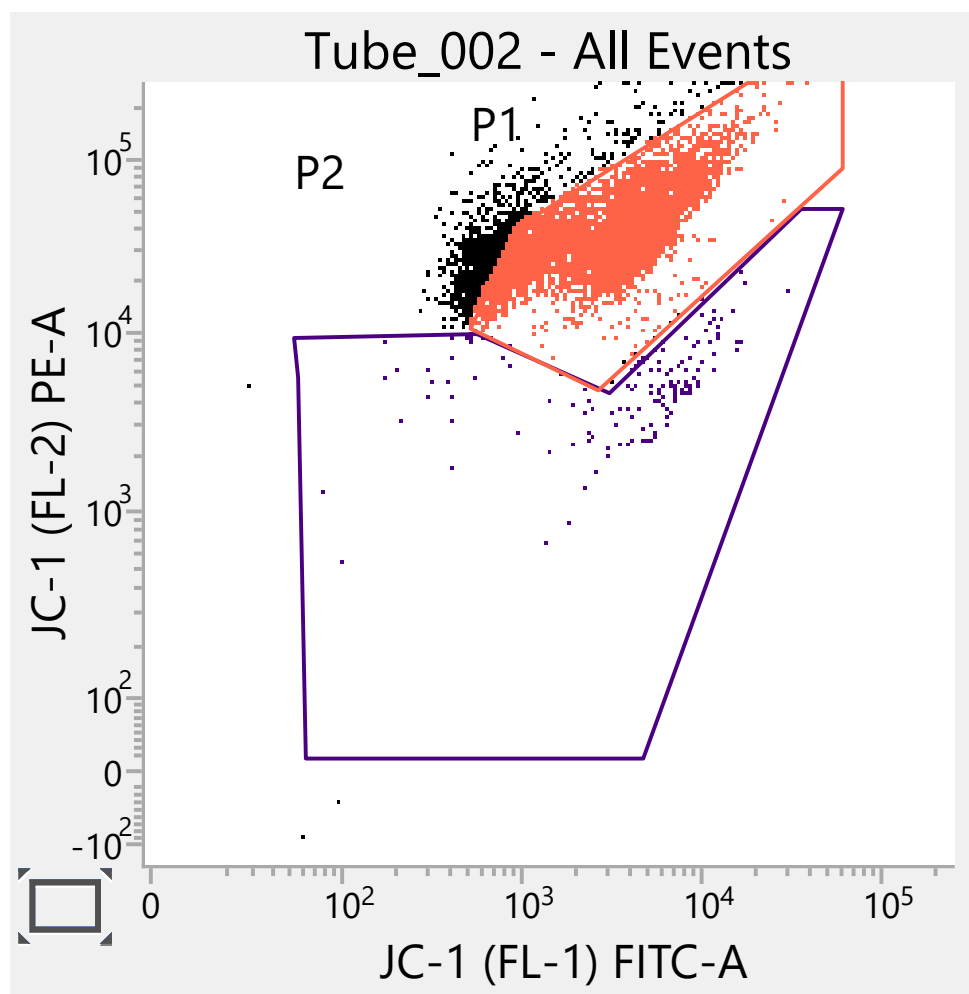

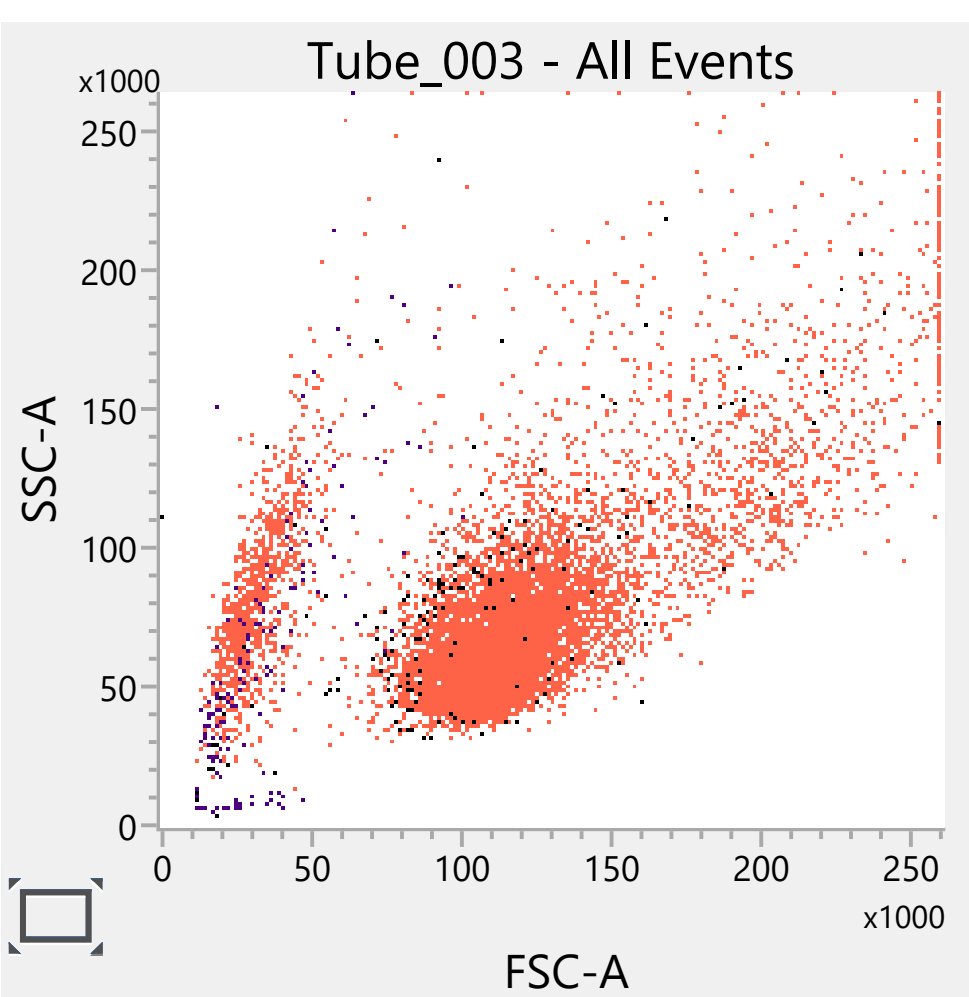

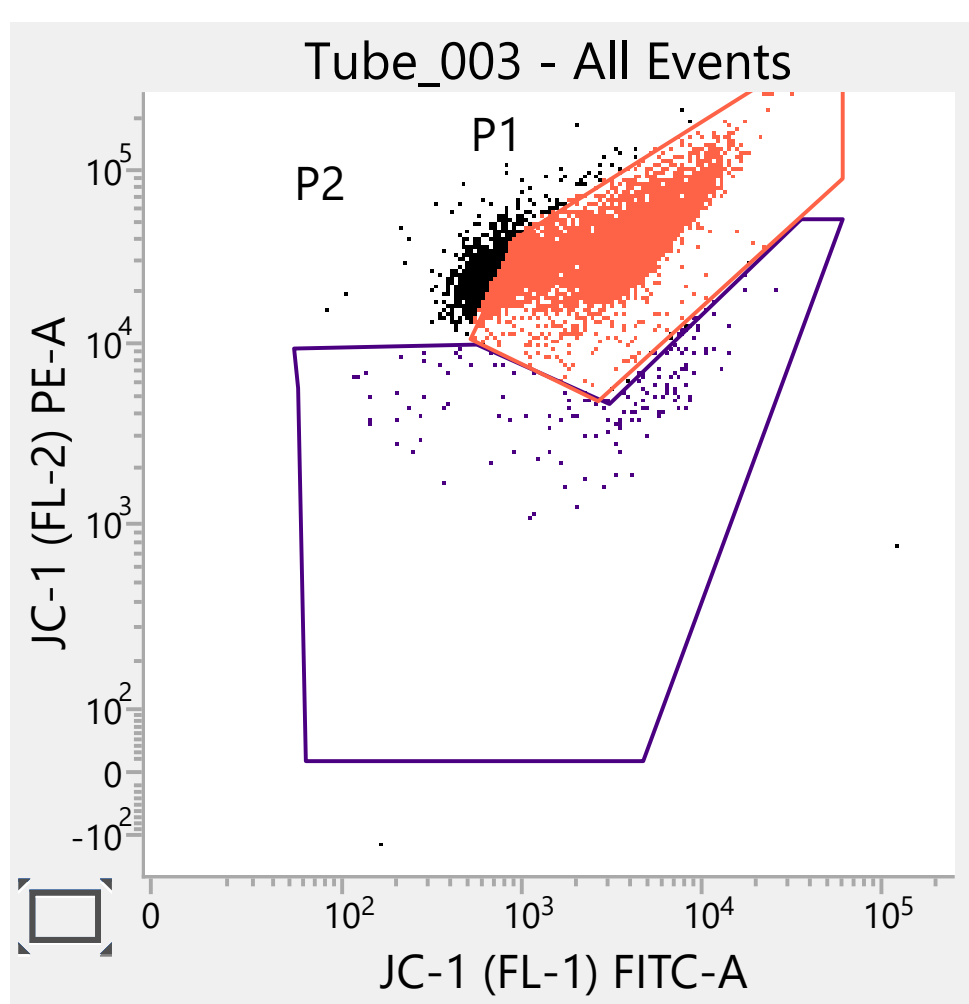

Statistics

Name	Tube_001:All Events	Tube_001:P2	Tube_001:P1	Tube_002:All Events	Tube_002:P2	Tube_002:P1	Tube_003:All Events	Tube_003:P2	Tube_003:P1
Events	10,000	189	8,864	10,000	145	8,550	10,000	151	8,736
% Parent	***	1.9	88.6	***	1.5	85.5	***	1.5	87.4
% Grandparent	***	***	***	***	***	***	***	***	***
% Total	100.0	1.9	88.6	100.0	1.5	85.5	100.0	1.5	87.4

JC-1 Assay

Sample ID: N6_K

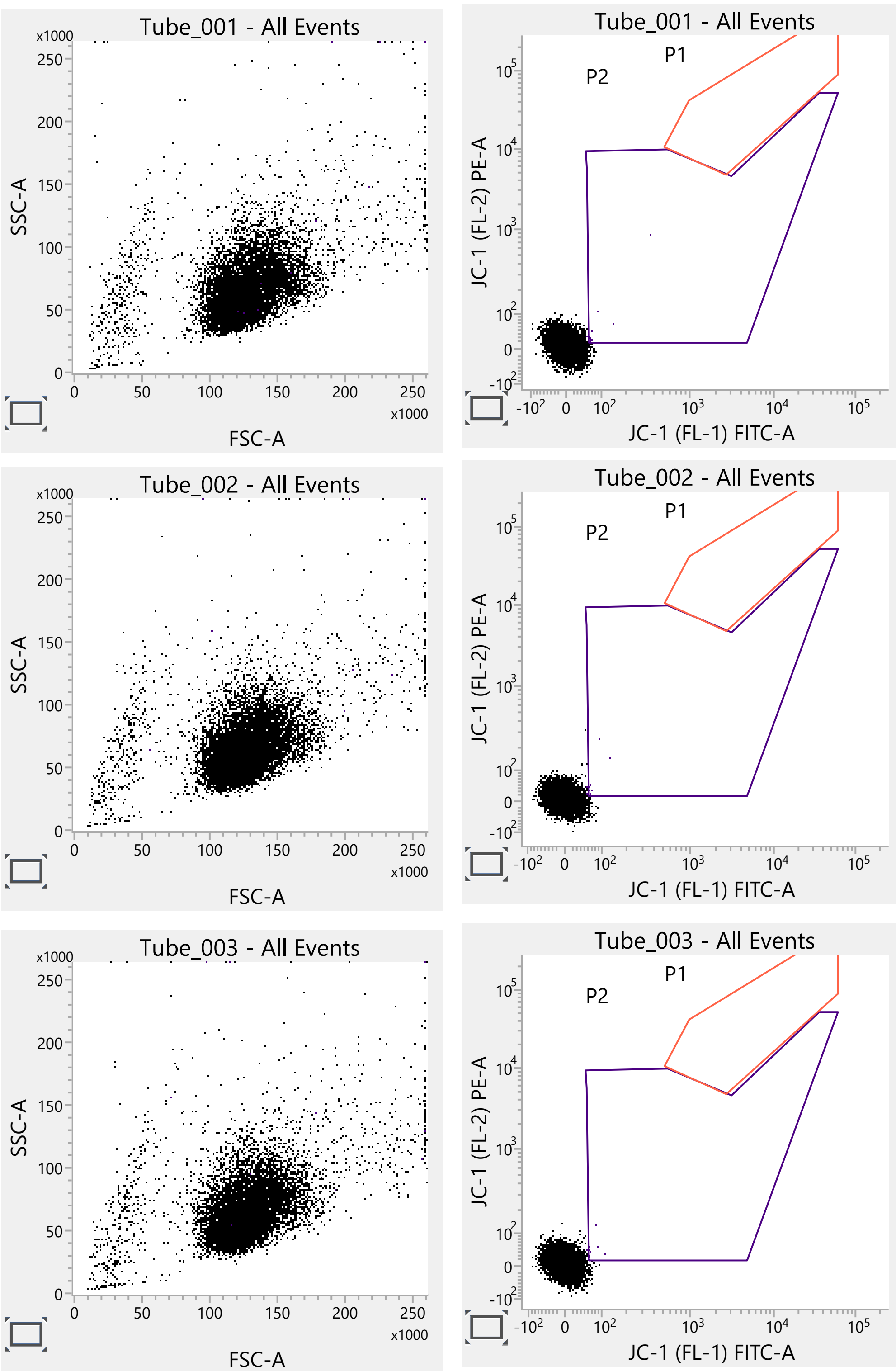

Statistics

Name	Tube_001:All Events	Tube_001:P2	Tube_001:P1	Tube_002:All Events	Tube_002:P2	Tube_002:P1	Tube_003:All Events	Tube_003:P2	Tube_003:P1
Events	10,000	11	0	10,000	10	0	10,000	10	0
% Parent	***	0.1	0.0	***	0.1	0.0	***	0.1	0.0
% Grandparent	***	***	***	***	***	***	***	***	***
% Total	100.0	0.1	0.0	100.0	0.1	0.0	100.0	0.1	0.0

Annexin V FITC PI Assay

Sample ID: N6_0,5

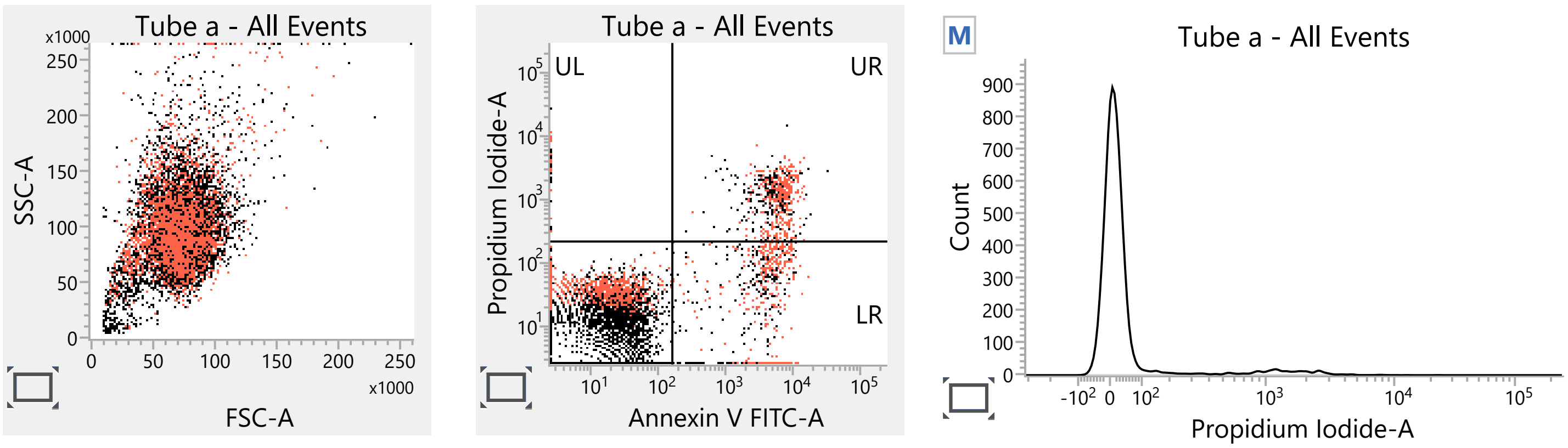

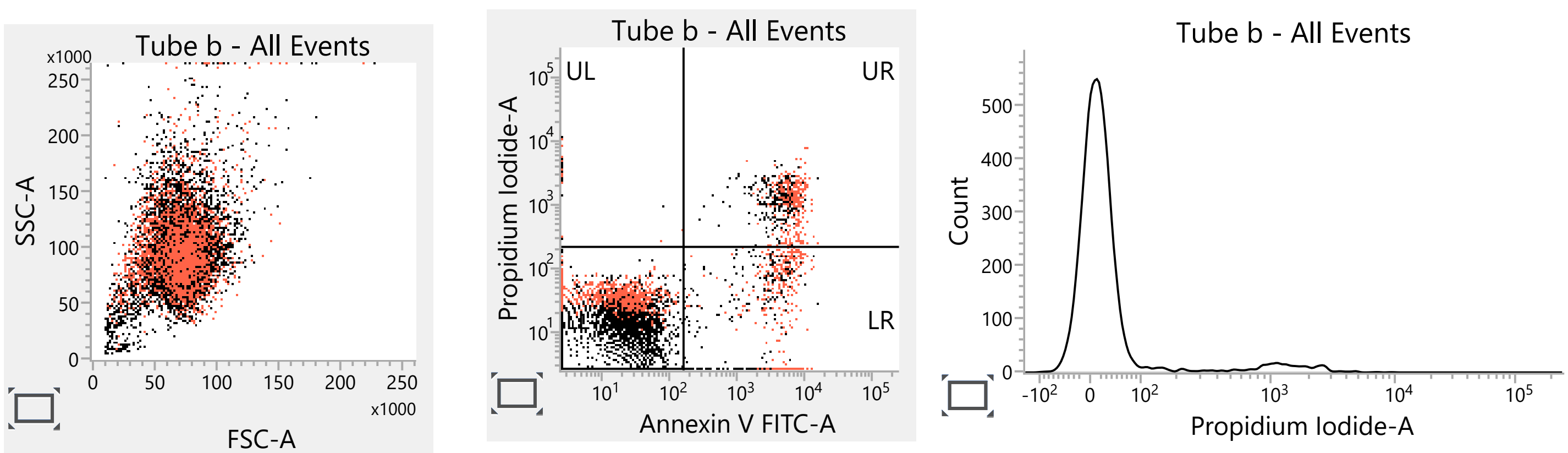

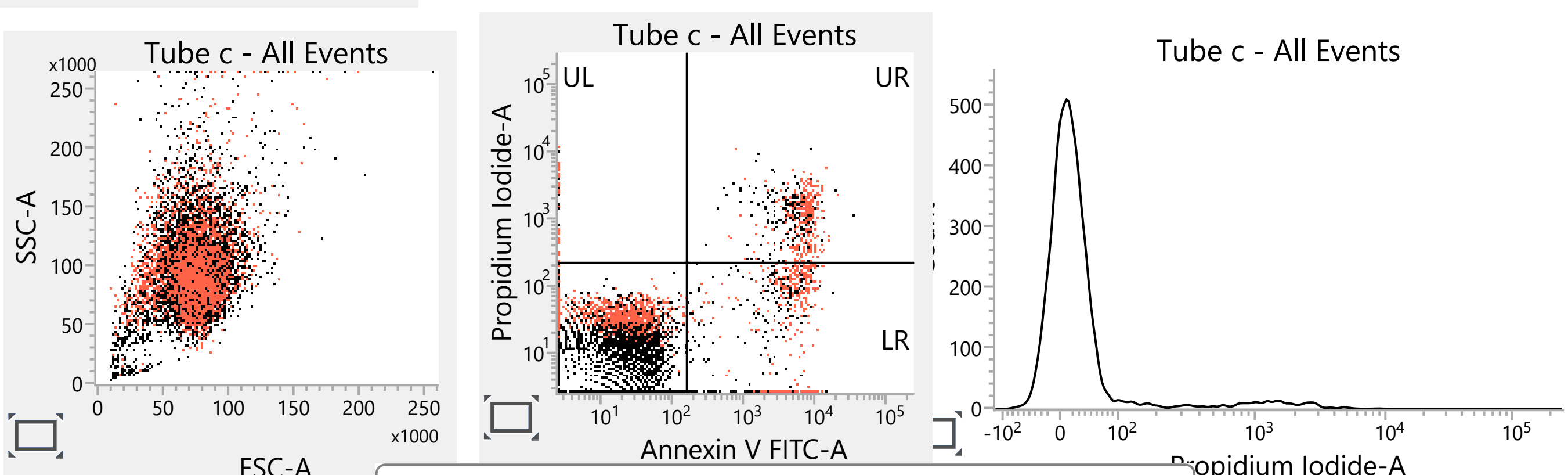

Statistics

Name	Events	% Parent	% Grandparent	% Total
Tube a:All Events	10,000	***	***	100.00
Tube b:All Events	10,000	***	***	100.00
Tube c:All Events	10,000	***	***	100.00
Tube a:UL	80	0.80	***	0.80
Tube a:UR	600	6.00	***	6.00
Tube a:LL	8,738	87.38	***	87.38
Tube a:LR	582	5.82	***	5.82
Tube b:UL	74	0.74	***	0.74
Tube b:UR	565	5.65	***	5.65
Tube b:LL	8,785	87.85	***	87.85
Tube b:LR	576	5.76	***	5.76
Tube c:UL	85	0.85	***	0.85
Tube c:UR	507	5.07	***	5.07
Tube c:LL	8,837	88.37	***	88.37
Tube c:LR	571	5.71	***	5.71

LDT

Annexin V FITC PI Assay

Sample ID: N6_1

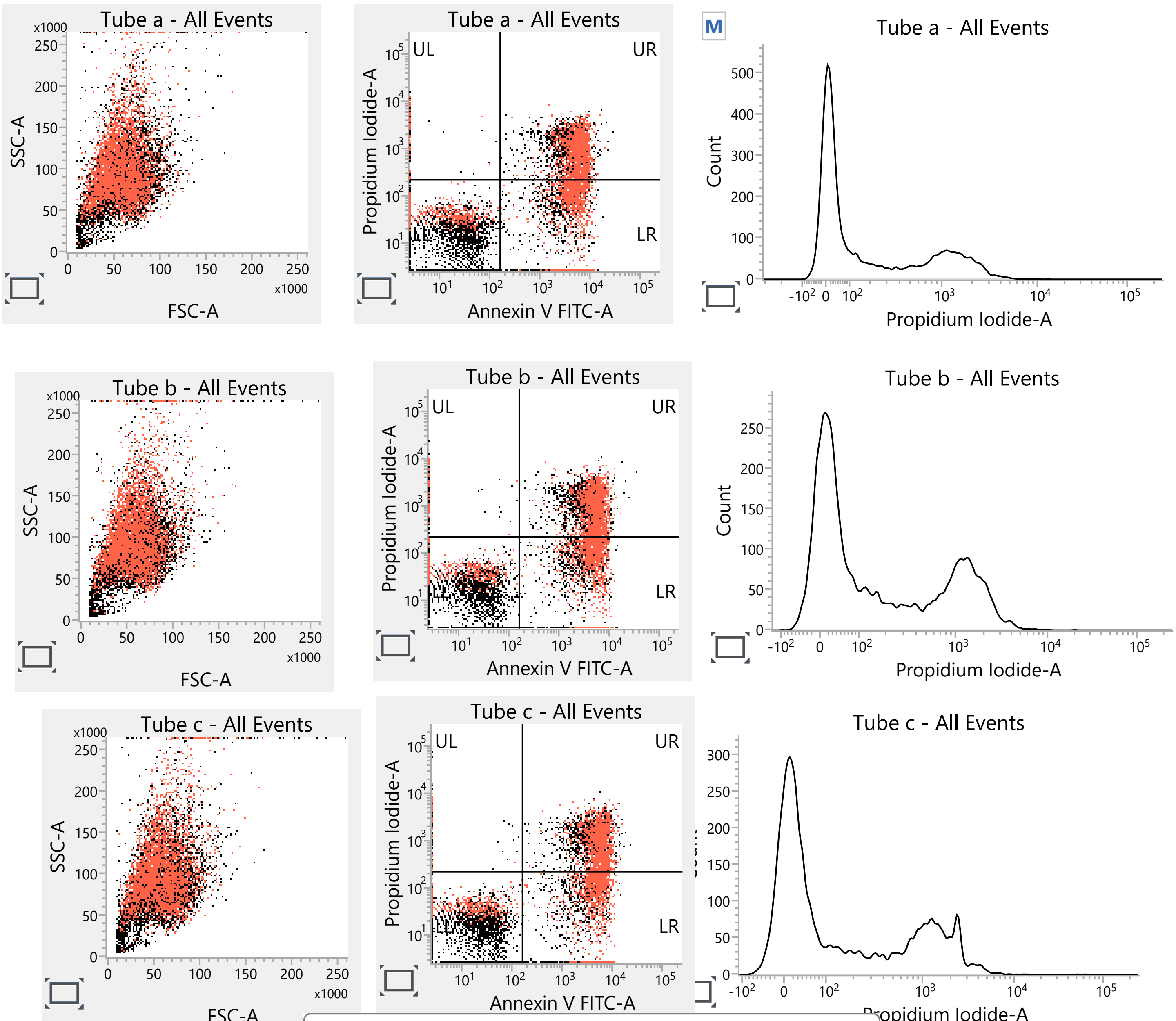

Statistics

Name	Events	% Parent	% Grandparent	% Total
Tube a:All Events	10,000	***	***	100.00
Tube b:All Events	10,000	***	***	100.00
Tube c:All Events	10,000	***	***	100.00
Tube a:UL	198	1.98	***	1.98
Tube a:UR	3,197	31.97	***	31.97
Tube a:LL	4,591	45.91	***	45.91
Tube a:LR	2,014	20.14	***	20.14
Tube b:UL	174	1.74	***	1.74
Tube b:UR	3,539	35.39	***	35.39
Tube b:LL	4,053	40.53	***	40.53
Tube b:LR	2,234	22.34	***	22.34
Tube c:UL	462	4.62	***	4.62
Tube c:UR	2,959	29.59	***	29.59
Tube c:LL	4,732	47.32	***	47.32
Tube c:LR	1,847	18.47	***	18.47

LDT

Annexin V FITC PI Assay

Sample ID: N6_2,5

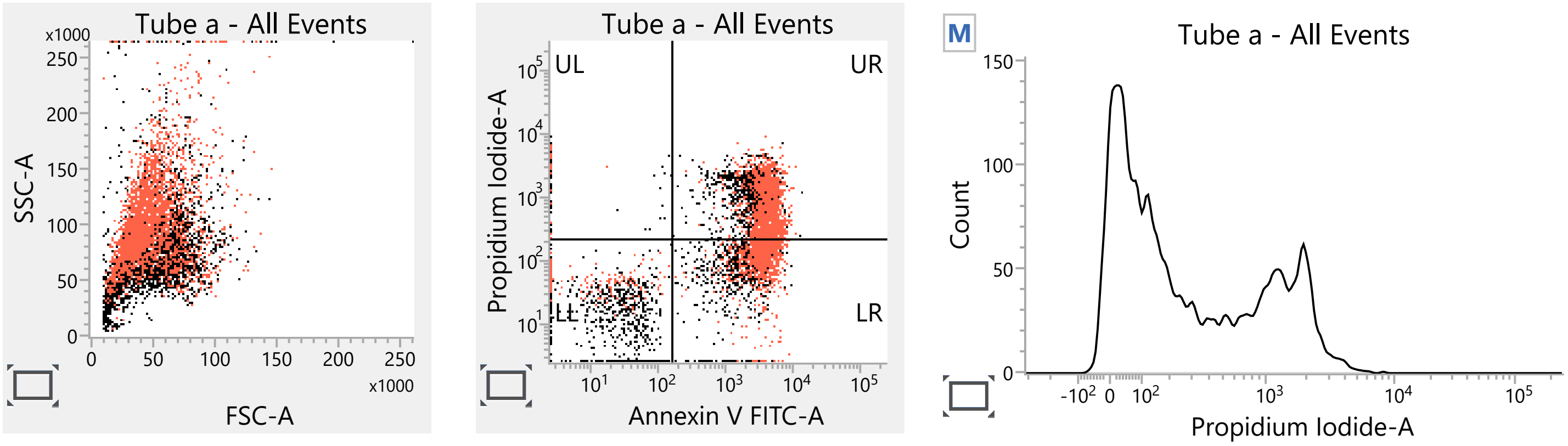

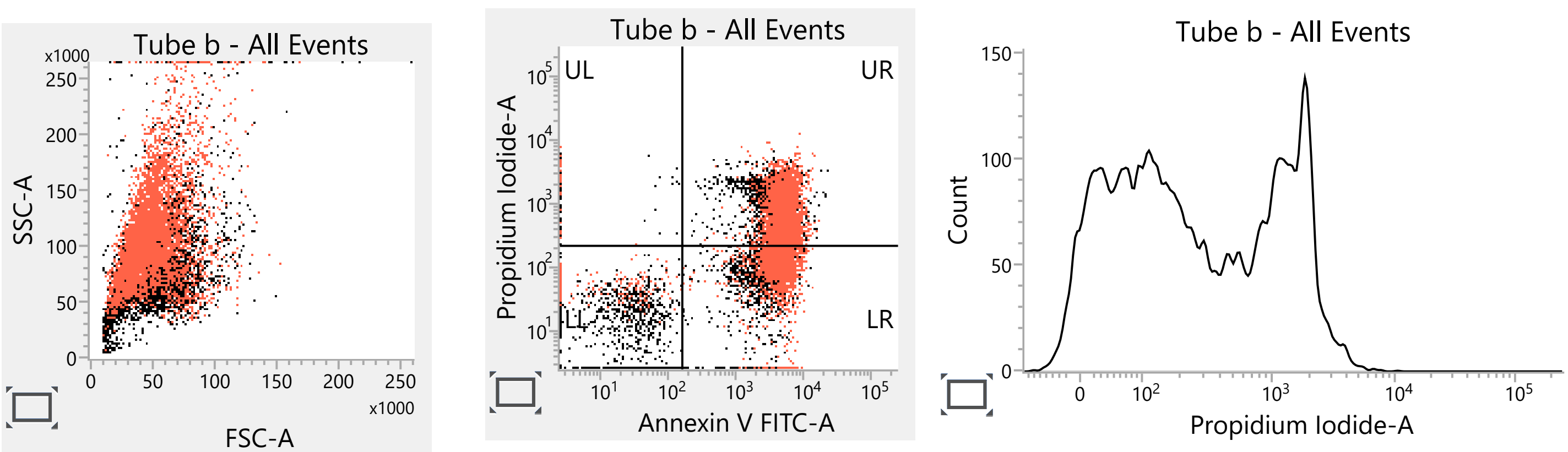

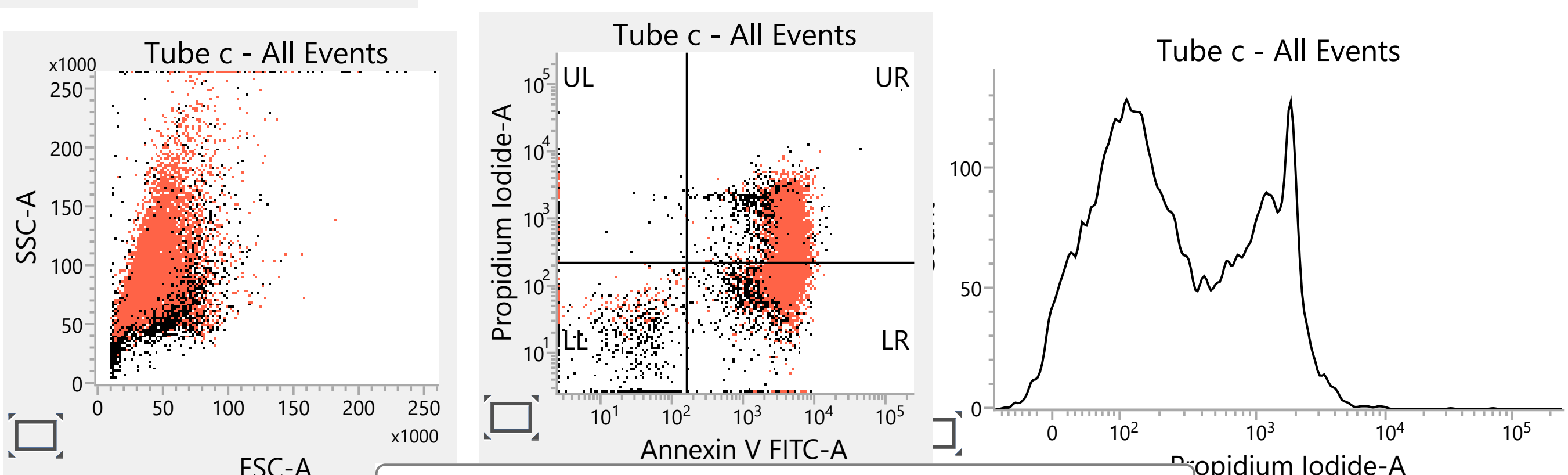

Statistics

Name	Events	% Parent	% Grandparent	% Total
Tube a:All Events	5,828	***	***	100.00
Tube b:All Events	10,000	***	***	100.00
Tube c:All Events	10,000	***	***	100.00
Tube a:UL	94	1.61	***	1.61
Tube a:UR	2,615	44.87	***	44.87
Tube a:LL	1,186	20.35	***	20.35
Tube a:LR	1,933	33.17	***	33.17
Tube b:UL	93	0.93	***	0.93
Tube b:UR	5,054	50.54	***	50.54
Tube b:LL	1,359	13.59	***	13.59
Tube b:LR	3,494	34.94	***	34.94
Tube c:UL	115	1.15	***	1.15
Tube c:UR	5,000	50.00	***	50.00
Tube c:LL	820	8.20	***	8.20
Tube c:LR	4,065	40.65	***	40.65

LDT

Annexin V FITC PI Assay

Sample ID: N6_5

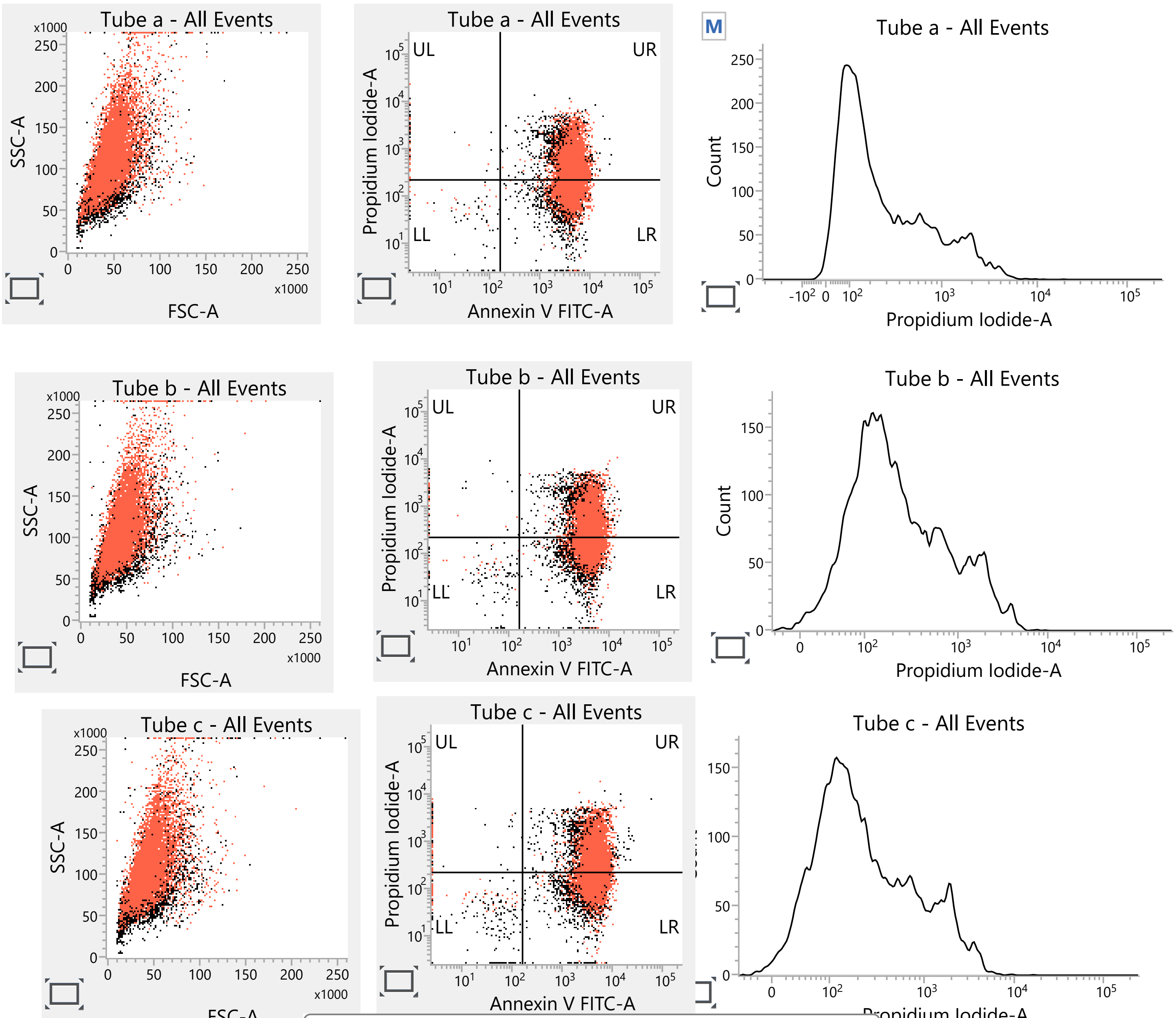

Statistics

Name	Events	% Parent	% Grandparent	% Total
Tube a:All Events	10,000	***	***	100.00
Tube b:All Events	10,000	***	***	100.00
Tube c:All Events	10,000	***	***	100.00
Tube a:UL	69	0.69	***	0.69
Tube a:UR	4,731	47.31	***	47.31
Tube a:LL	71	0.71	***	0.71
Tube a:LR	5,129	51.29	***	51.29
Tube b:UL	74	0.74	***	0.74
Tube b:UR	4,683	46.83	***	46.83
Tube b:LL	111	1.11	***	1.11
Tube b:LR	5,132	51.32	***	51.32
Tube c:UL	129	1.29	***	1.29
Tube c:UR	4,673	46.73	***	46.73
Tube c:LL	196	1.96	***	1.96
Tube c:LR	5,002	50.02	***	50.02

LDT

Annexin V FITC PI Assay

Sample ID: N6_K+P

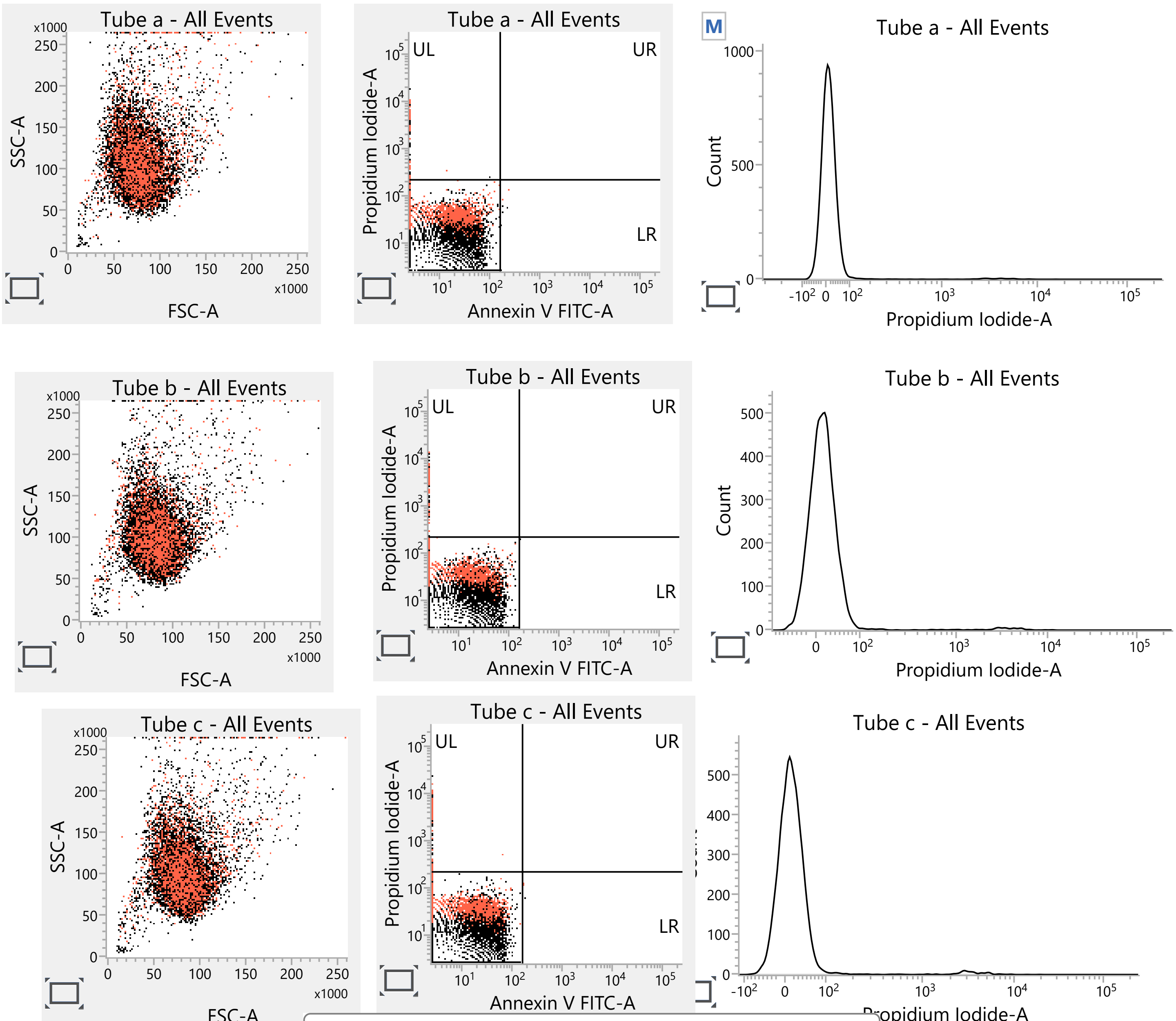

Statistics

Name	Events	% Parent	% Grandparent	% Total
Tube a:All Events	10,000	***	***	100.00
Tube b:All Events	10,000	***	***	100.00
Tube c:All Events	10,000	***	***	100.00
Tube a:UL	125	1.25	***	1.25
Tube a:UR	0	0.00	***	0.00
Tube a:LL	9,864	98.64	***	98.64
Tube a:LR	11	0.11	***	0.11
Tube b:UL	154	1.54	***	1.54
Tube b:UR	0	0.00	***	0.00
Tube b:LL	9,845	98.45	***	98.45
Tube b:LR	1	0.01	***	0.01
Tube c:UL	190	1.90	***	1.90
Tube c:UR	0	0.00	***	0.00
Tube c:LL	9,807	98.07	***	98.07
Tube c:LR	3	0.03	***	0.03

LDT

Annexin V FITC PI Assay

Sample ID: N6_KAP

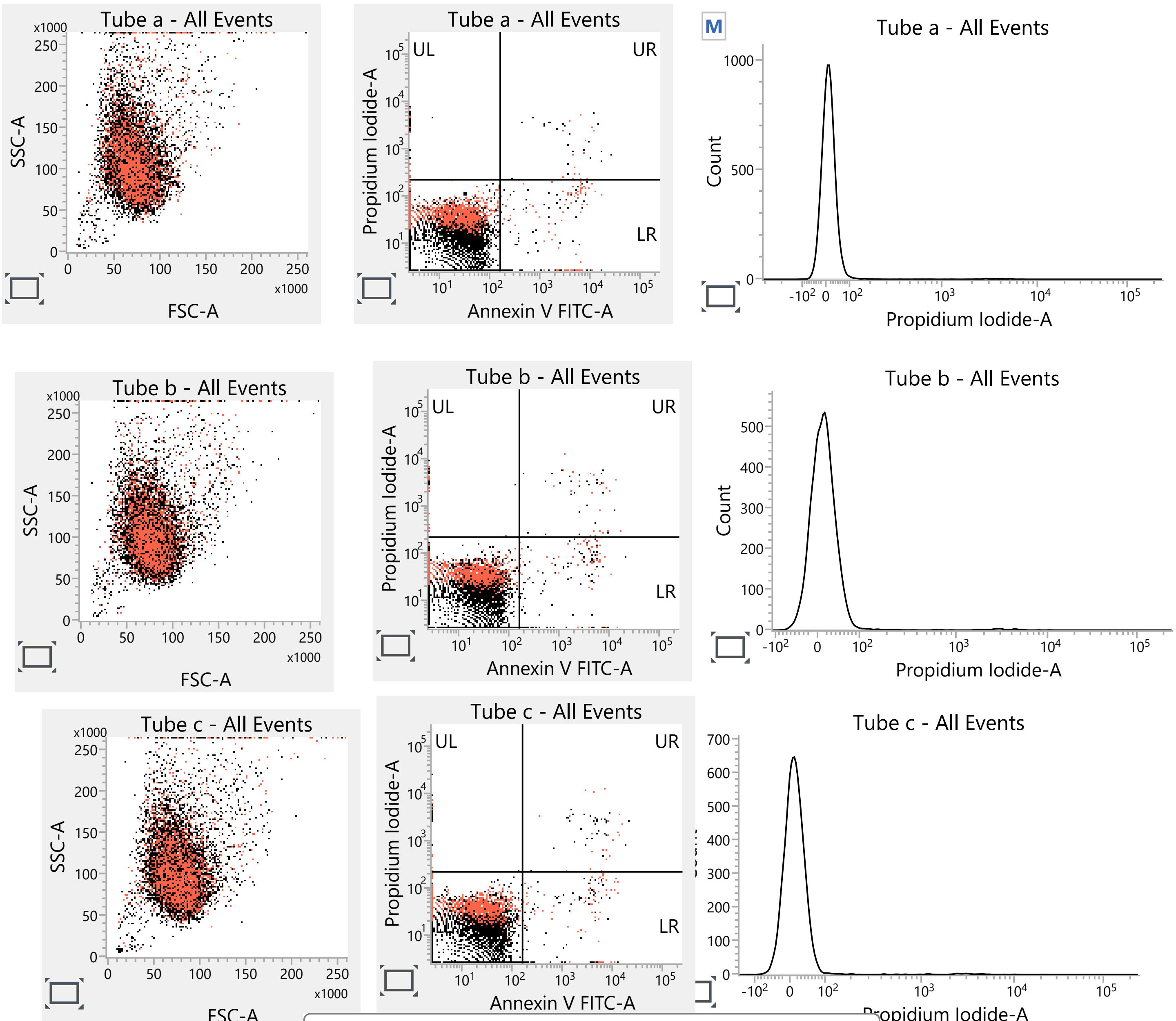

Statistics

Name	Events	% Parent	% Grandparent	% Total
Tube a:All Events	10,000	***	***	100.00
Tube b:All Events	10,000	***	***	100.00
Tube c:All Events	10,000	***	***	100.00
Tube a:UL	47	0.47	***	0.47
Tube a:UR	38	0.38	***	0.38
Tube a:LL	9,747	97.47	***	97.47
Tube a:LR	168	1.68	***	1.68
Tube b:UL	36	0.36	***	0.36
Tube b:UR	50	0.50	***	0.50
Tube b:LL	9,750	97.50	***	97.50
Tube b:LR	164	1.64	***	1.64
Tube c:UL	40	0.40	***	0.40
Tube c:UR	52	0.52	***	0.52
Tube c:LL	9,750	97.50	***	97.50
Tube c:LR	158	1.58	***	1.58

LDT

Annexin V FITC PI Assay

Sample ID: N6-DMSO

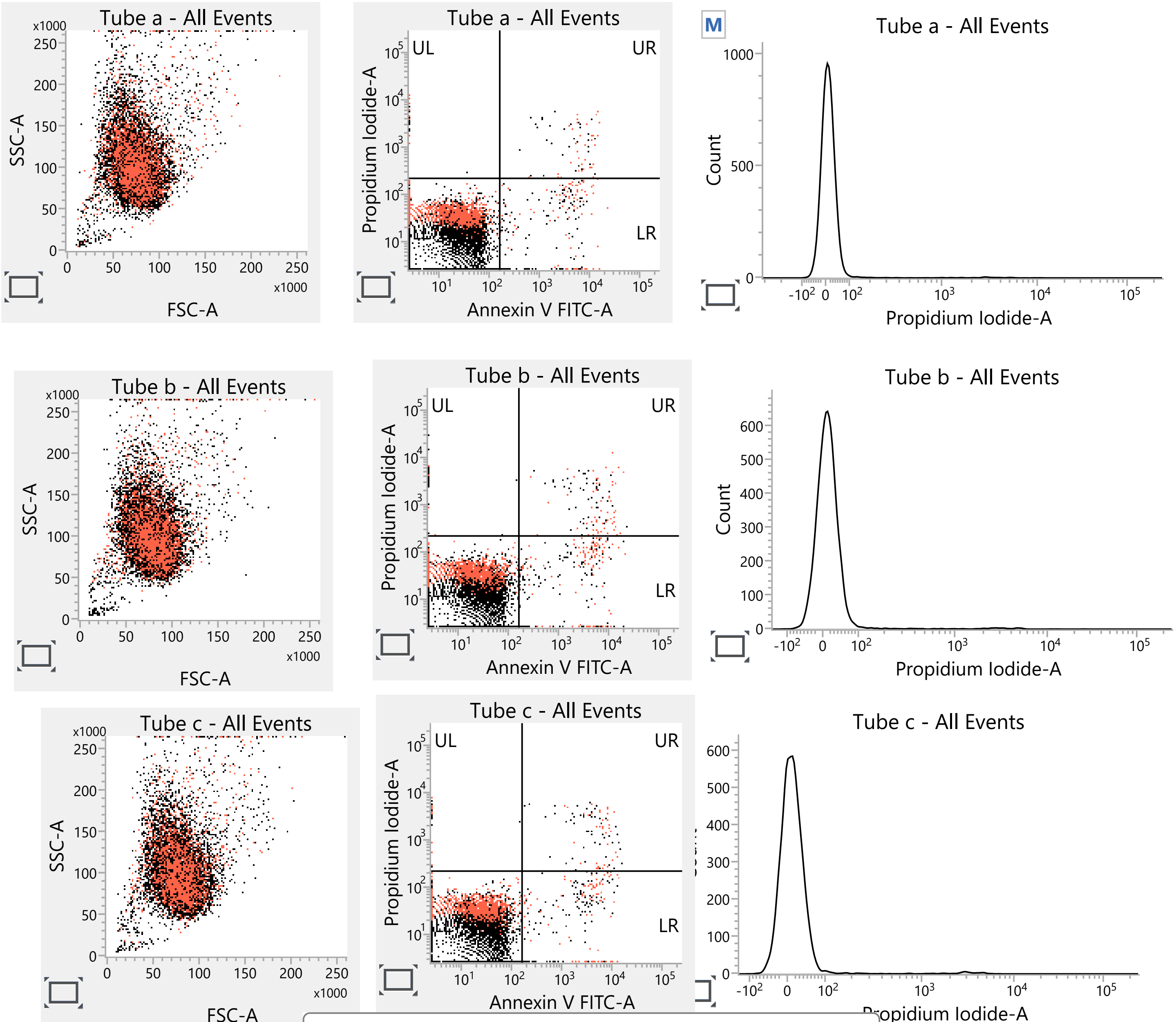

Name	Events	% Parent	% Grandparent	% Total
Tube a:All Events	10,000	***	***	100.00
Tube b:All Events	10,000	***	***	100.00
Tube c:All Events	10,000	***	***	100.00
Tube a:UL	41	0.41	***	0.41
Tube a:UR	65	0.65	***	0.65
Tube a:LL	9,688	96.88	***	96.88
Tube a:LR	206	2.06	***	2.06
Tube b:UL	43	0.43	***	0.43
Tube b:UR	103	1.03	***	1.03
Tube b:LL	9,599	95.99	***	95.99
Tube b:LR	255	2.55	***	2.55
Tube c:UL	47	0.47	***	0.47
Tube c:UR	85	0.85	***	0.85
Tube c:LL	9,644	96.44	***	96.44
Tube c:LR	224	2.24	***	2.24

Statistics

LDT

Annexin V FITC PI Assay

Sample ID: N6-K

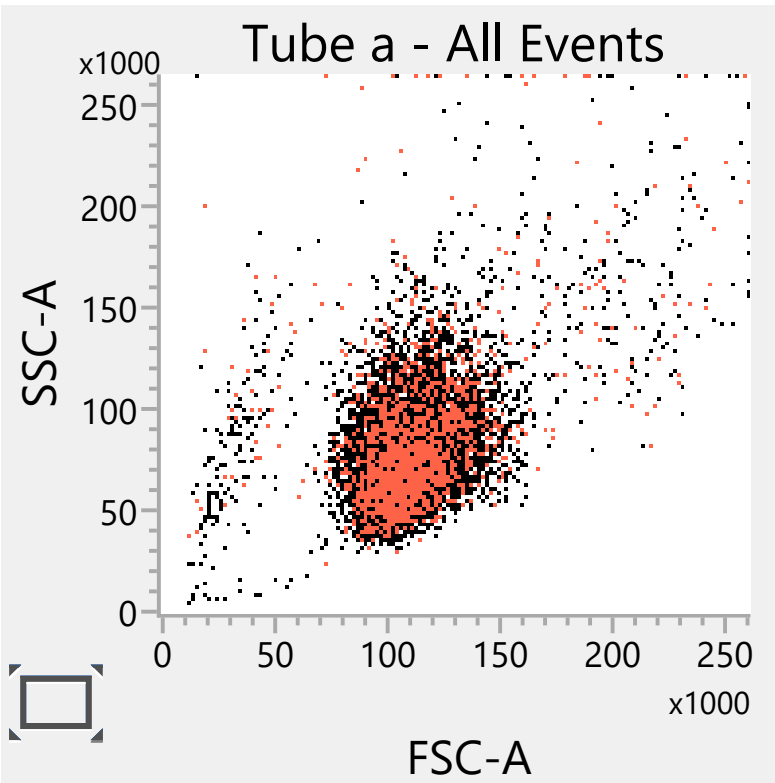

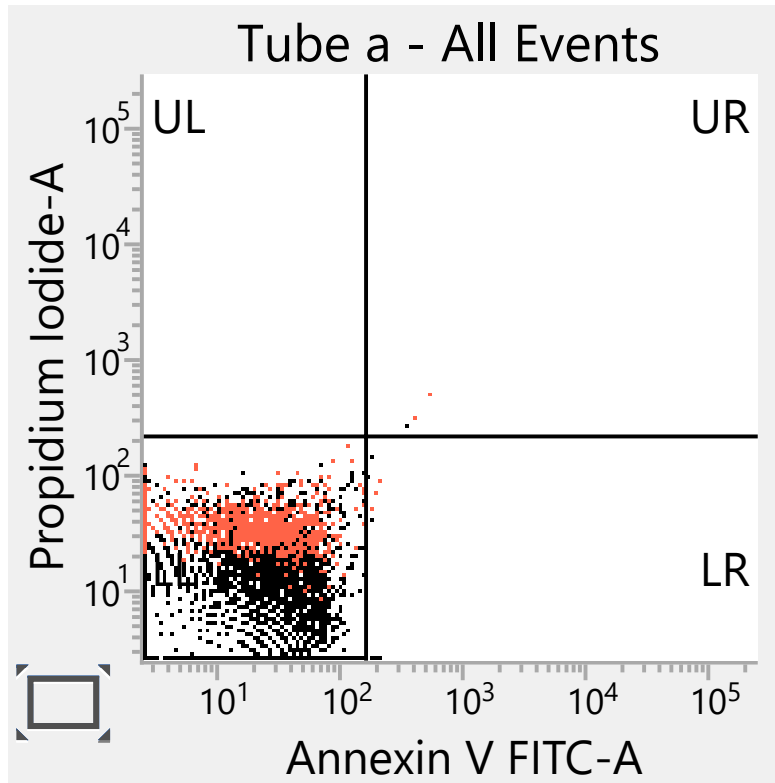

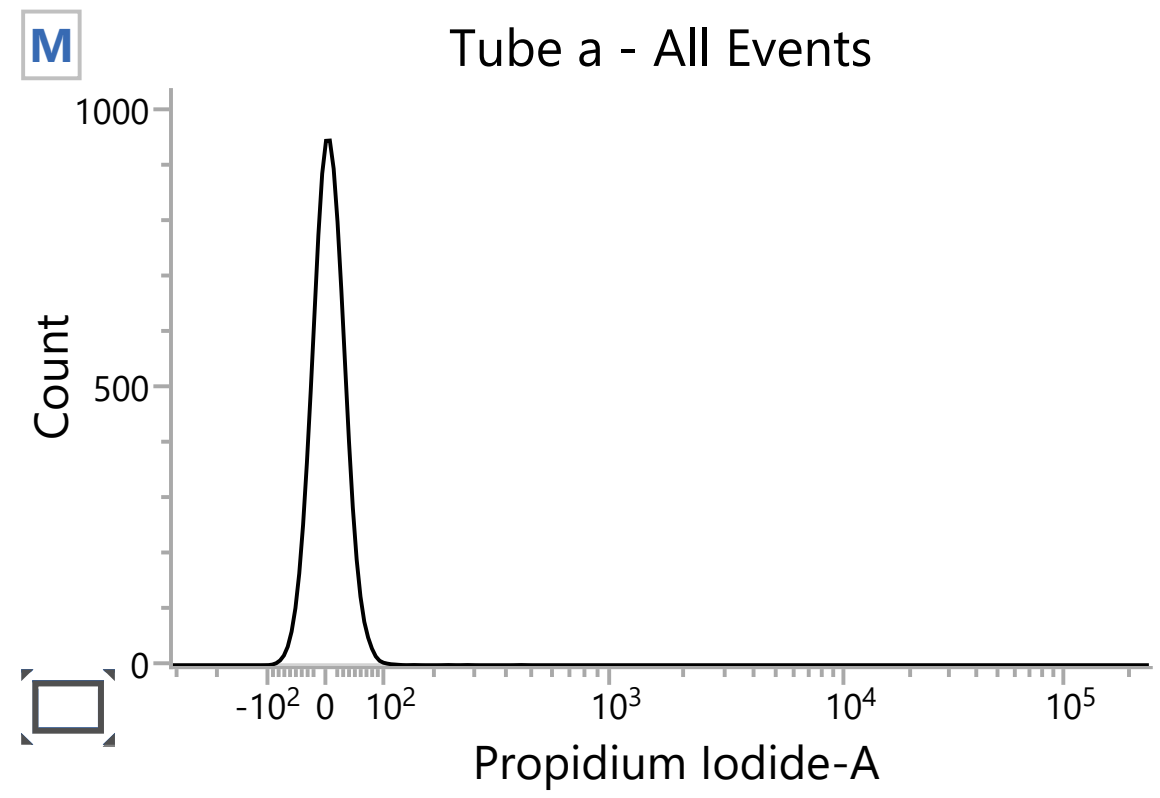

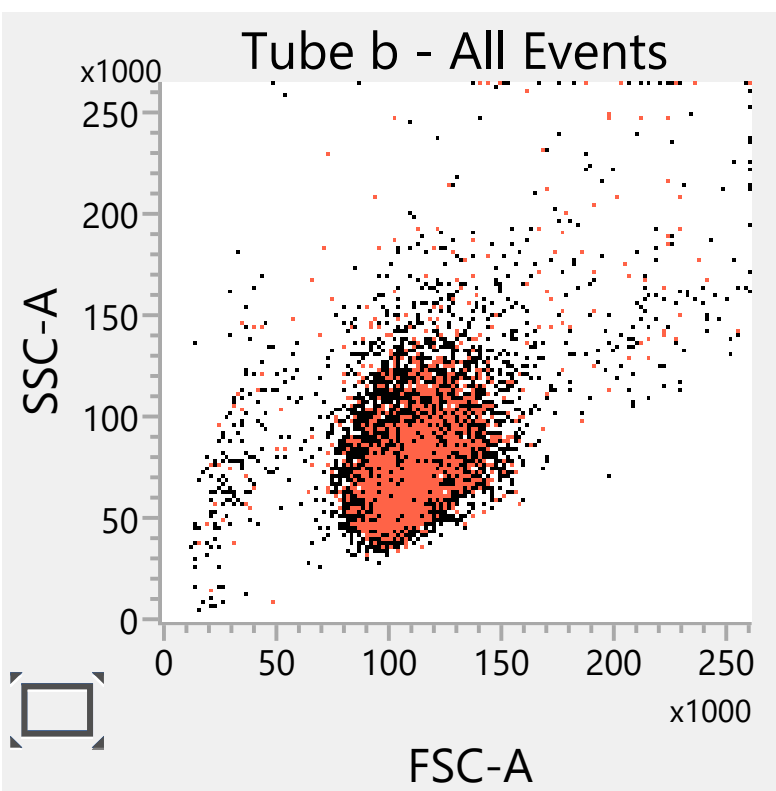

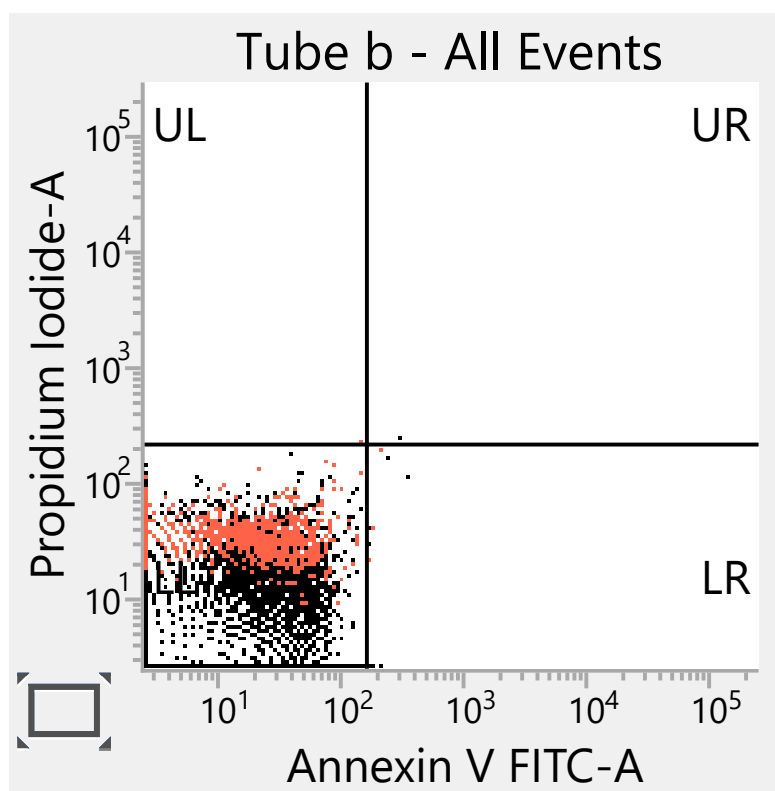

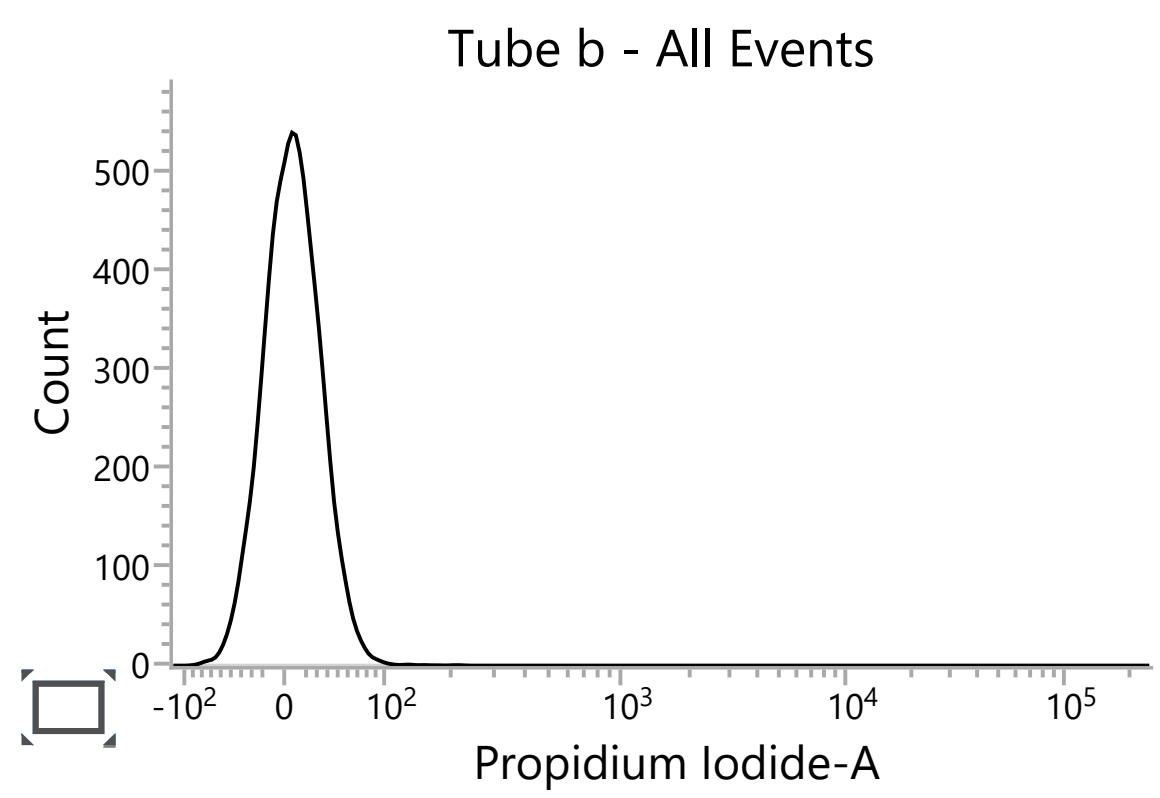

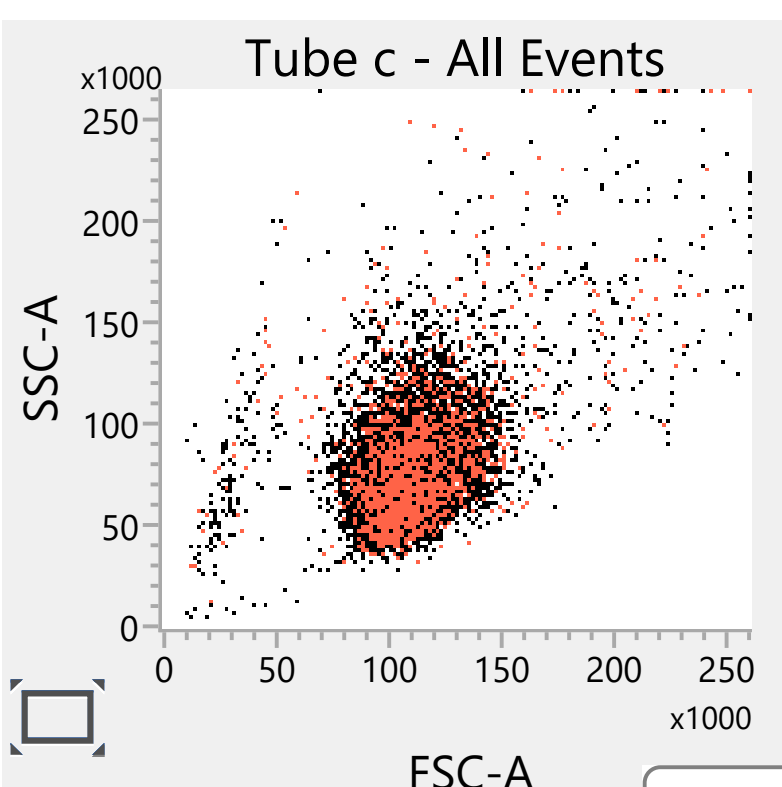

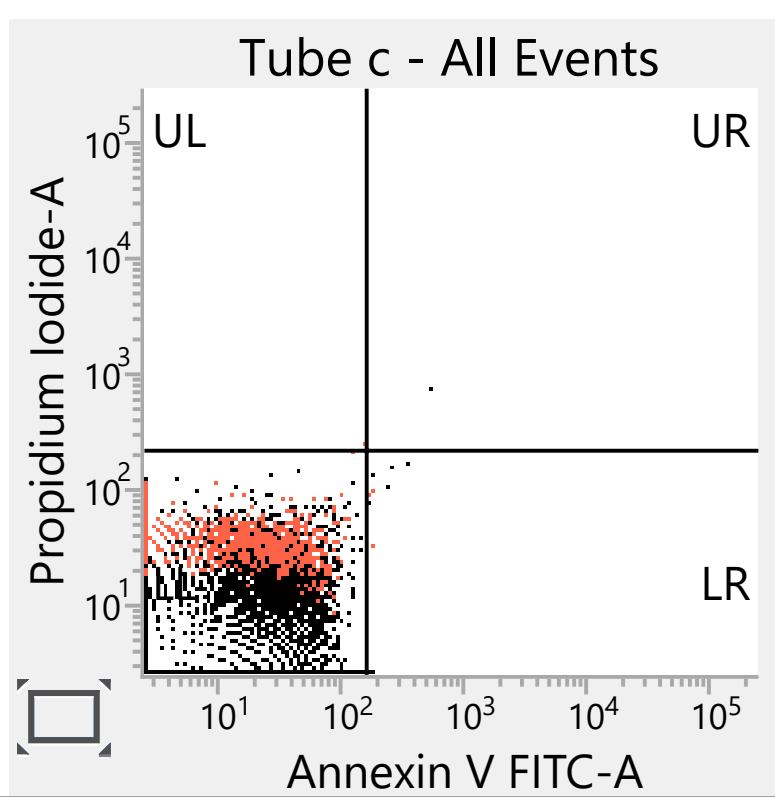

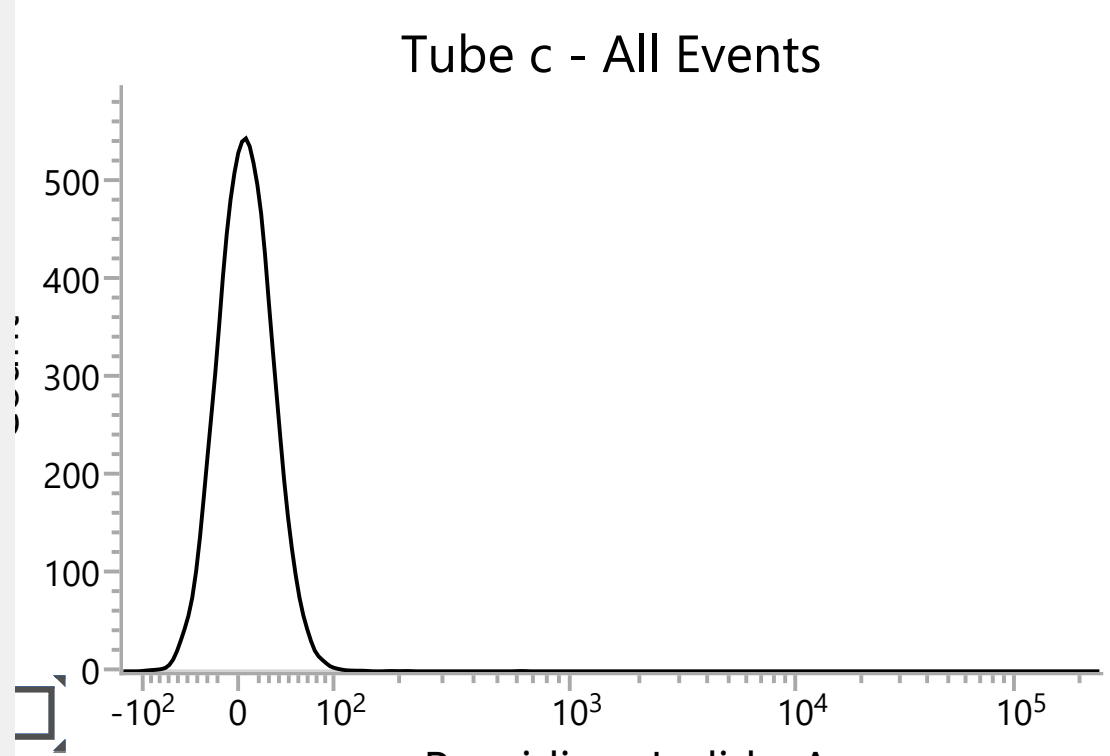

Statistics

Name	Events	% Parent	% Grandparent	% Total
Tube a:All Events	10,000	***	***	100.00
Tube b:All Events	10,000	***	***	100.00
Tube c:All Events	10,000	***	***	100.00
Tube a:UL	0	0.00	***	0.00
Tube a:UR	3	0.03	***	0.03
Tube a:LL	9,982	99.82	***	99.82
Tube a:LR	15	0.15	***	0.15
Tube b:UL	1	0.01	***	0.01
Tube b:UR	1	0.01	***	0.01
Tube b:LL	9,988	99.88	***	99.88
Tube b:LR	10	0.10	***	0.10
Tube c:UL	0	0.00	***	0.00
Tube c:UR	2	0.02	***	0.02
Tube c:LL	9,987	99.87	***	99.87
Tube c:LR	11	0.11	***	0.11

LDT

Annexin V FITC PI Assay

Sample ID: N6-K+A

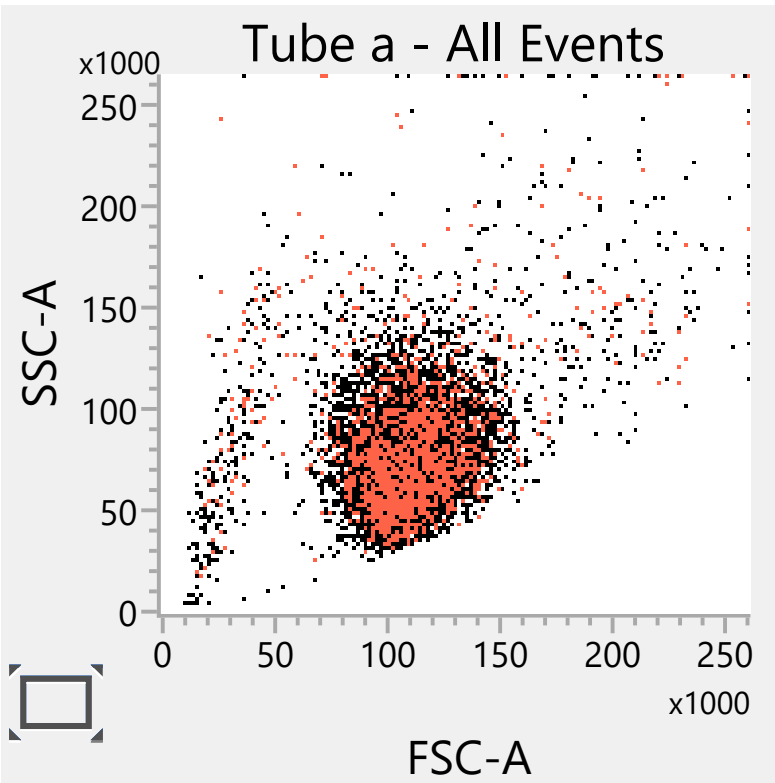

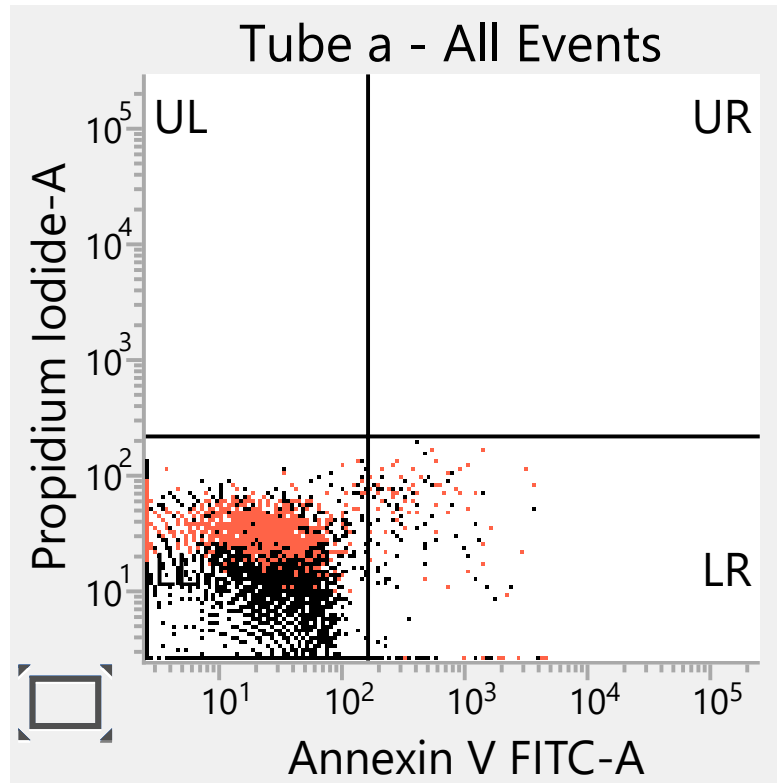

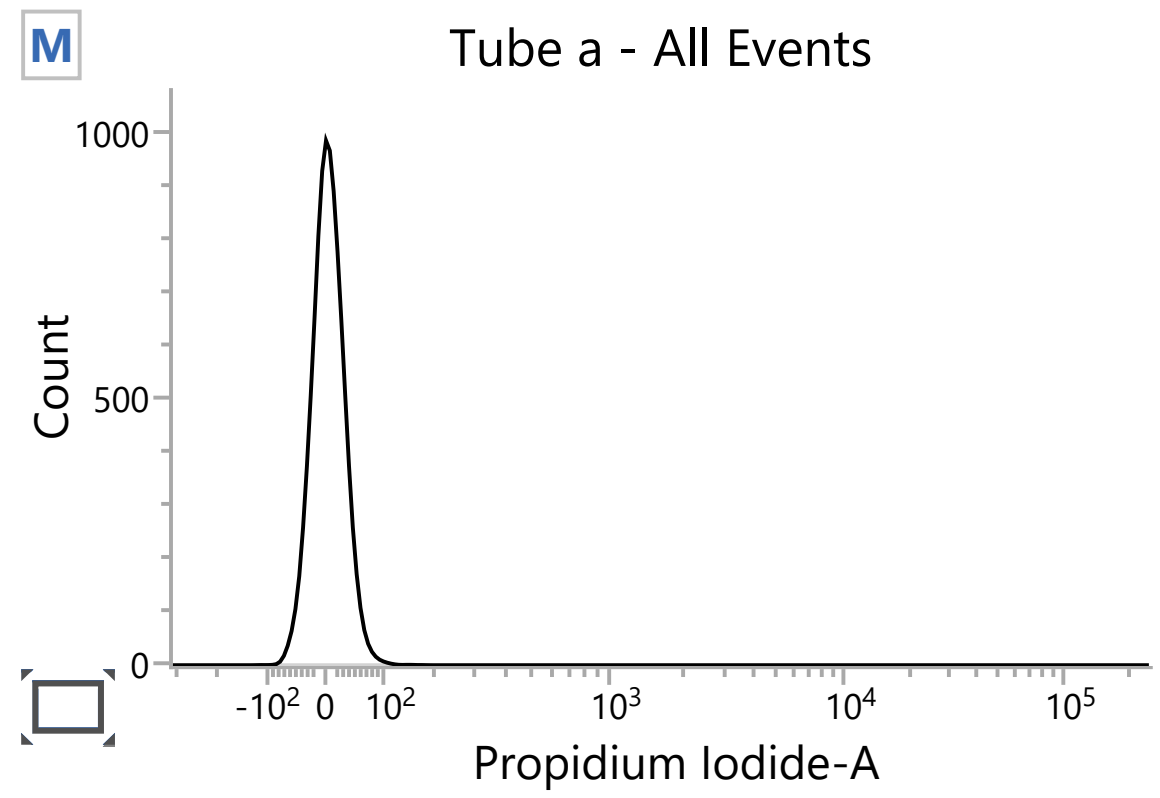

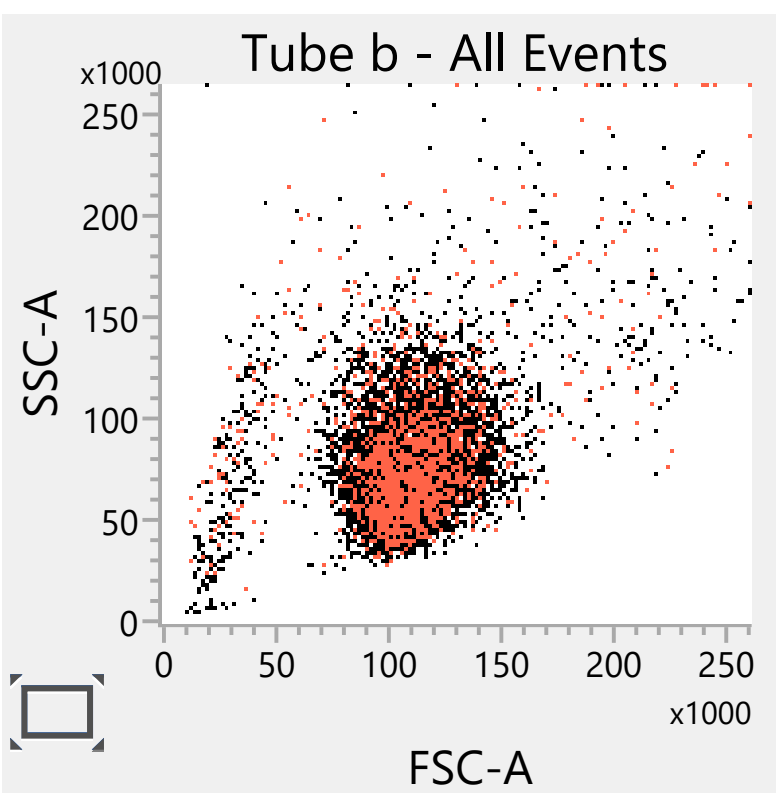

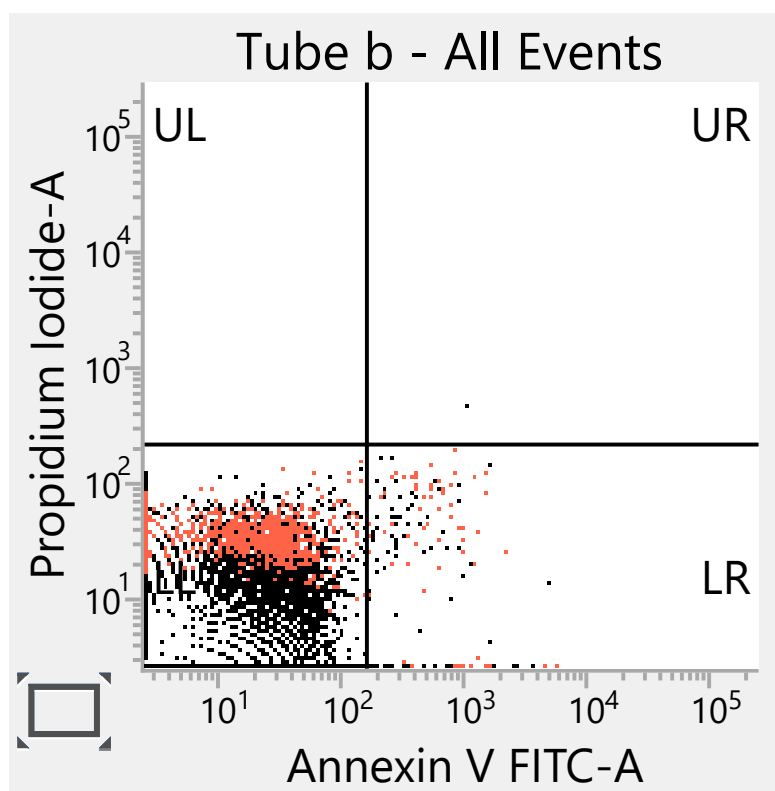

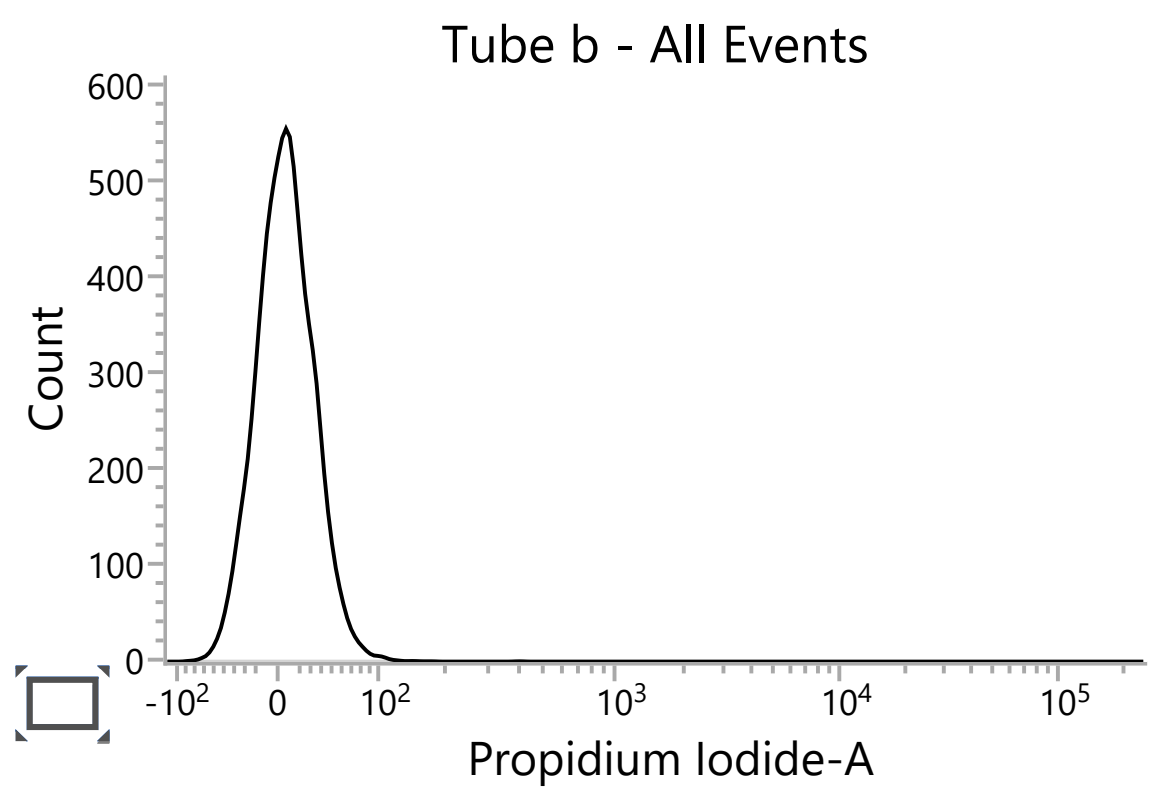

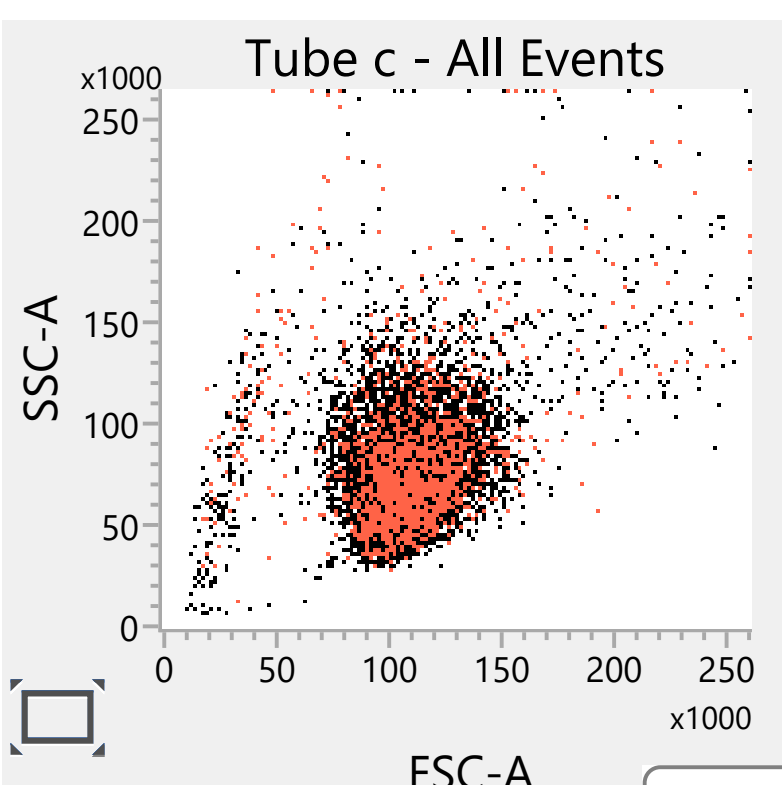

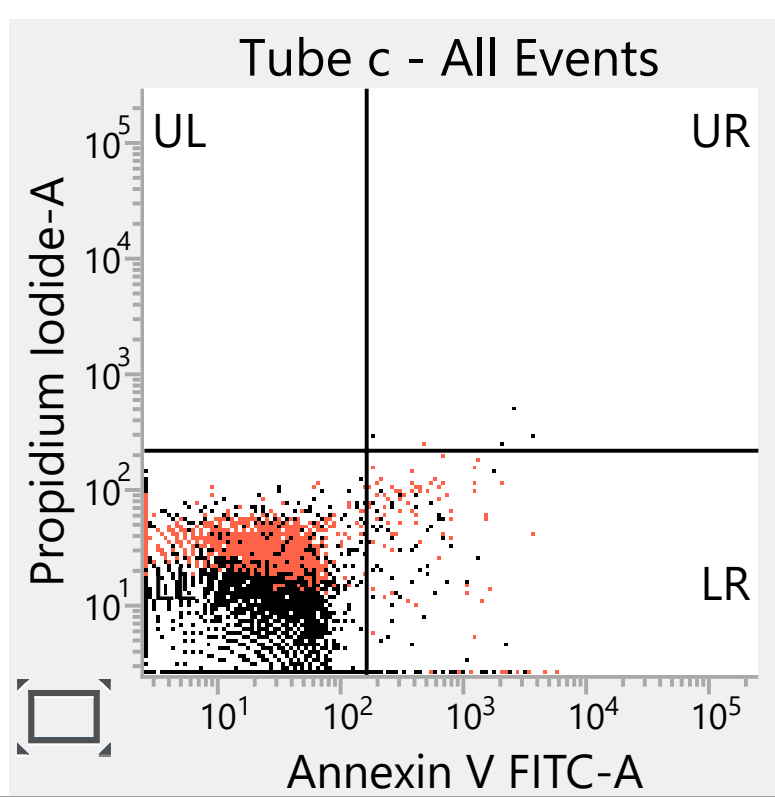

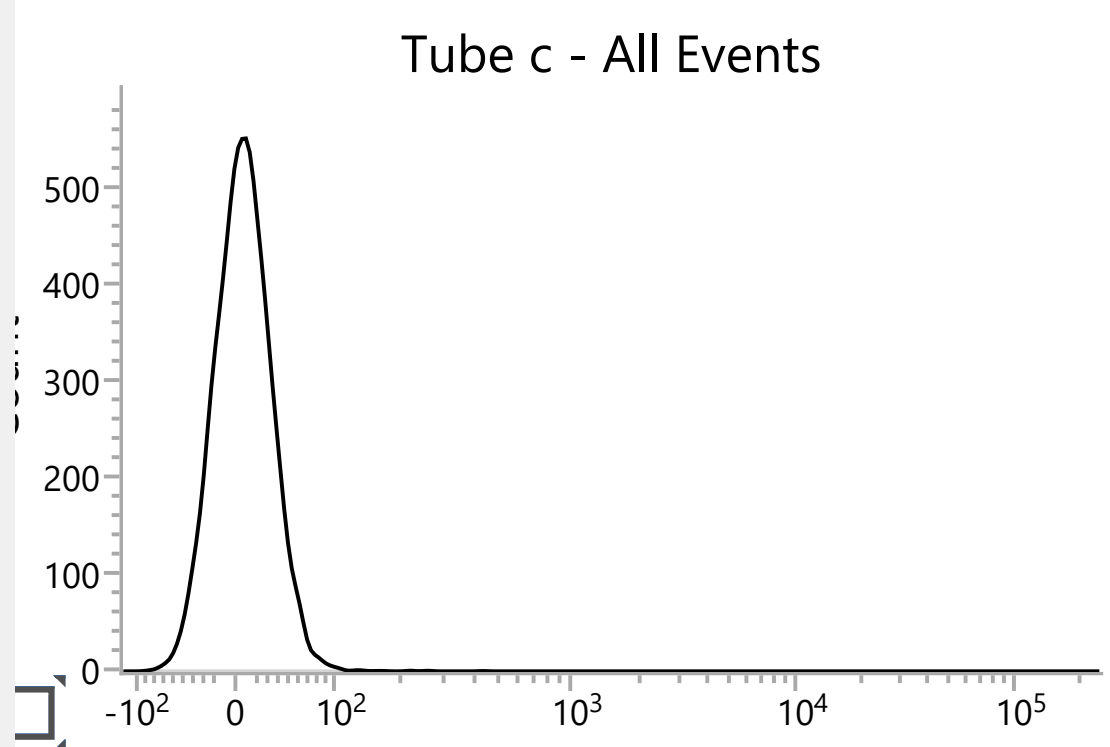

Statistics

Name	Events	% Parent	% Grandparent	% Total
Tube a:All Events	10,000	***	***	100.00
Tube b:All Events	10,000	***	***	100.00
Tube c:All Events	10,000	***	***	100.00
Tube b:UL	0	0.00	***	0.00
Tube b:UR	1	0.01	***	0.01
Tube b:LL	9,836	98.36	***	98.36
Tube b:LR	163	1.63	***	1.63
Tube a:UL	0	0.00	***	0.00
Tube a:UR	0	0.00	***	0.00
Tube a:LL	9,822	98.22	***	98.22
Tube a:LR	178	1.78	***	1.78
Tube c:UL	0	0.00	***	0.00
Tube c:UR	5	0.05	***	0.05
Tube c:LL	9,813	98.13	***	98.13
Tube c:LR	182	1.82	***	1.82

LDT

Annexin V FITC PI Assay

Sample ID: 0,5

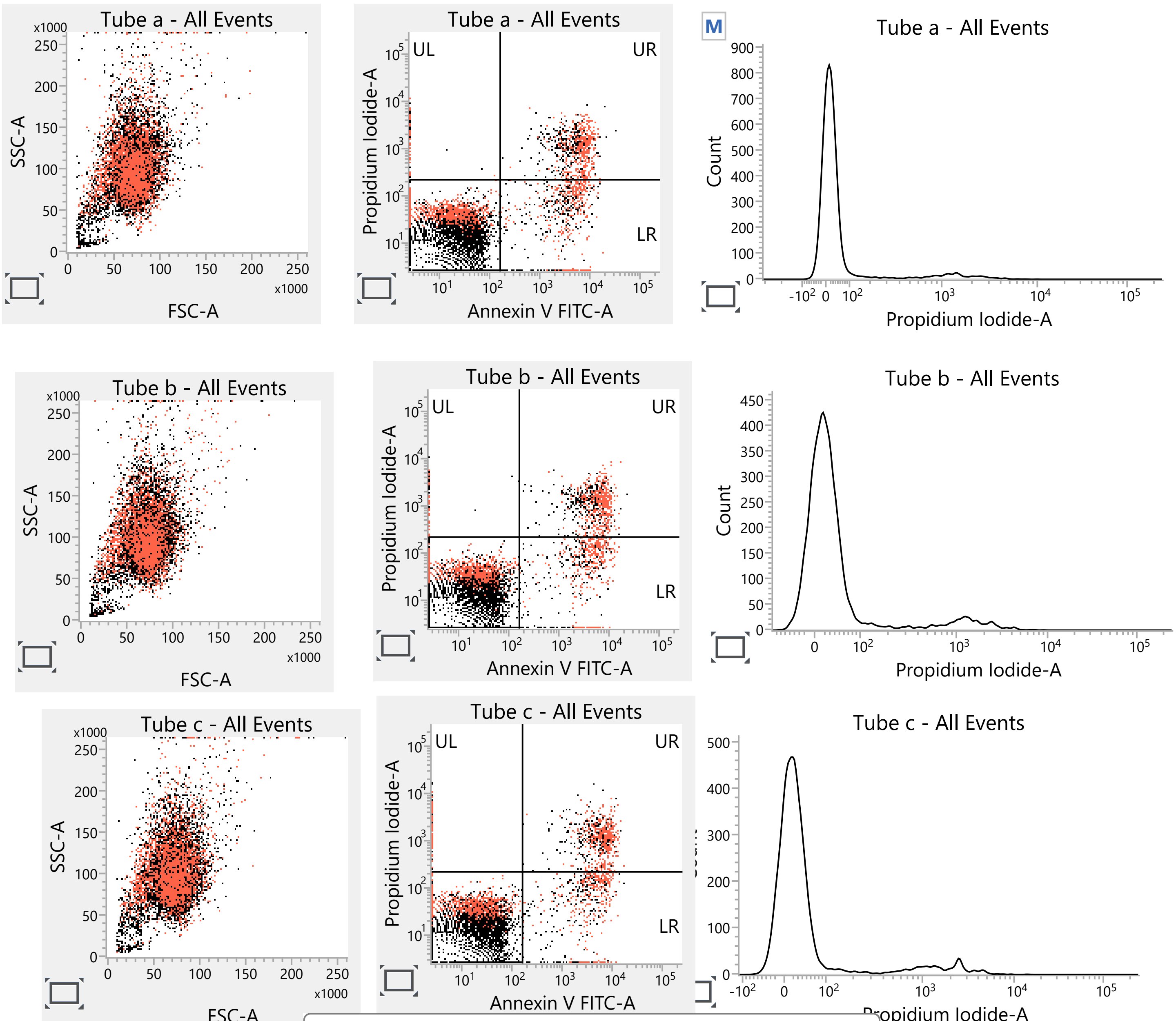

Statistics

Name	Events	% Parent	% Grandparent	% Total
Tube a:All Events	10,000	***	***	100.00
Tube b:All Events	10,000	***	***	100.00
Tube c:All Events	10,000	***	***	100.00
Tube a:UL	122	1.22	***	1.22
Tube a:UR	774	7.74	***	7.74
Tube a:LL	8,477	84.77	***	84.77
Tube a:LR	627	6.27	***	6.27
Tube b:UL	113	1.13	***	1.13
Tube b:UR	794	7.94	***	7.94
Tube b:LL	8,526	85.26	***	85.26
Tube b:LR	567	5.67	***	5.67
Tube c:UL	297	2.97	***	2.97
Tube c:UR	657	6.57	***	6.57
Tube c:LL	8,512	85.12	***	85.12
Tube c:LR	534	5.34	***	5.34

LDT

Annexin V FITC PI Assay

Sample ID: 1

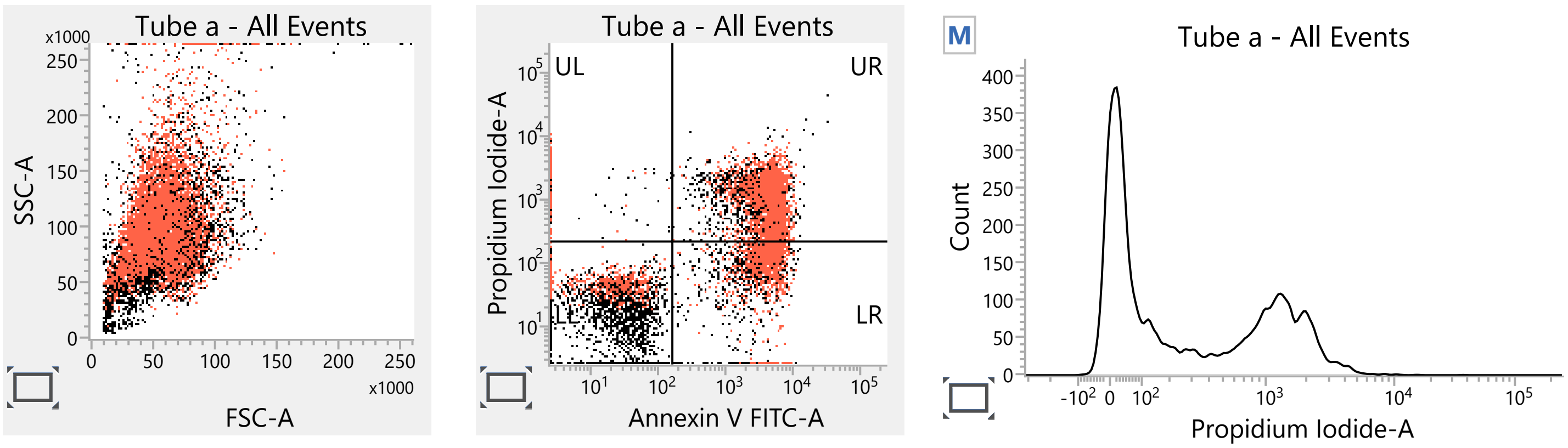

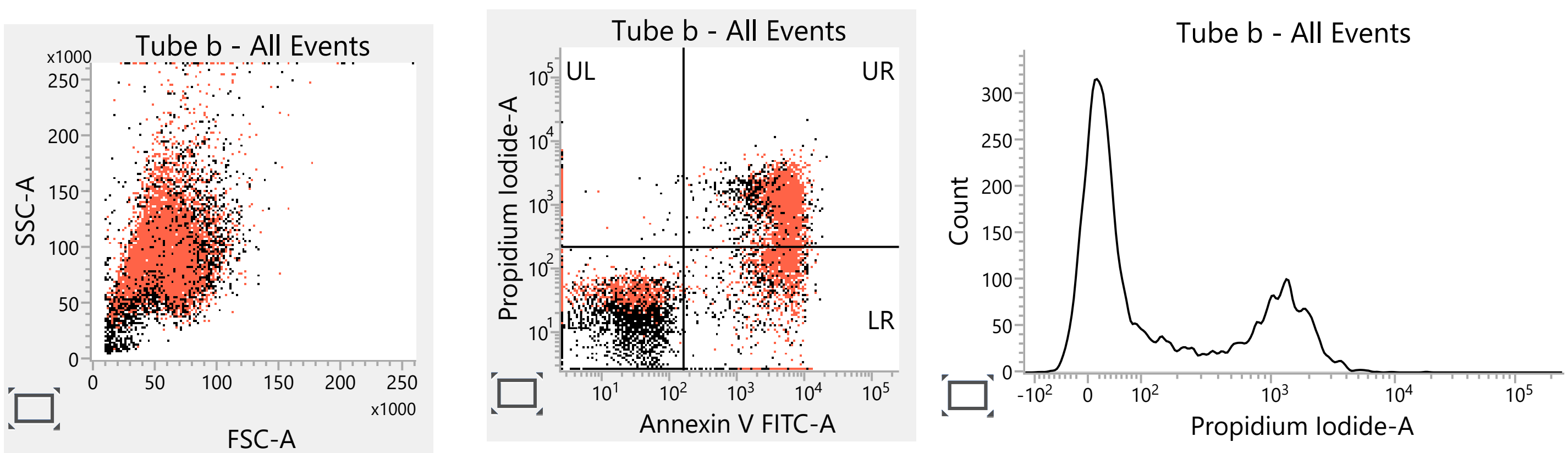

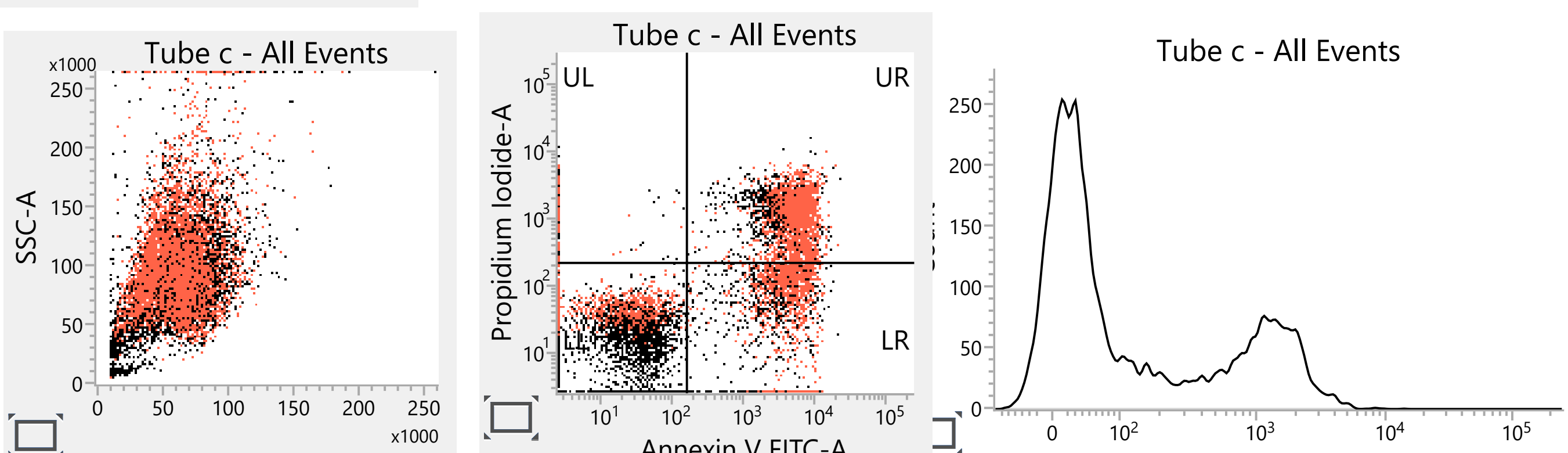

Statistics

Name	Events	% Parent	% Grandparent	% Total
Tube a:All Events	10,000	***	***	100.00
Tube b:All Events	10,000	***	***	100.00
Tube c:All Events	10,000	***	***	100.00
Tube a:UL	378	3.78	***	3.78
Tube a:UR	4,038	40.38	***	40.38
Tube a:LL	3,776	37.76	***	37.76
Tube a:LR	1,808	18.08	***	18.08
Tube b:UL	184	1.84	***	1.84
Tube b:UR	3,234	32.34	***	32.34
Tube b:LL	4,949	49.49	***	49.49
Tube b:LR	1,633	16.33	***	16.33
Tube c:UL	190	1.90	***	1.90
Tube c:UR	3,000	30.00	***	30.00
Tube c:LL	5,070	50.70	***	50.70
Tube c:LR	1,740	17.40	***	17.40

LDT

Annexin V FITC PI Assay

Sample ID: 2,5

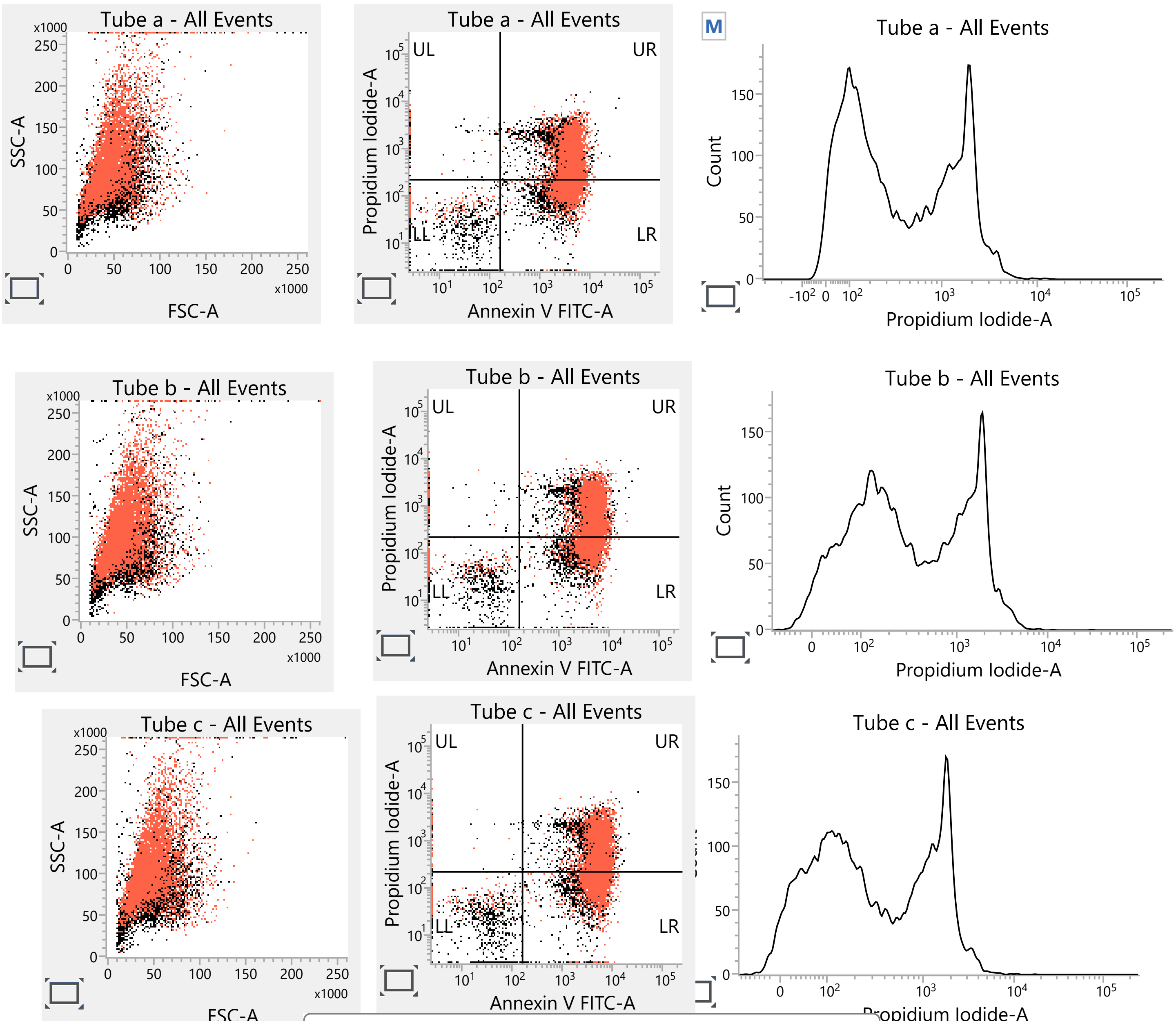

Statistics

Name	Events	% Parent	% Grandparent	% Total
Tube a:All Events	10,000	***	***	100.00
Tube b:All Events	10,000	***	***	100.00
Tube c:All Events	10,000	***	***	100.00
Tube a:UL	183	1.83	***	1.83
Tube a:UR	5,746	57.46	***	57.46
Tube a:LL	804	8.04	***	8.04
Tube a:LR	3,267	32.67	***	32.67
Tube b:UL	161	1.61	***	1.61
Tube b:UR	5,496	54.96	***	54.96
Tube b:LL	868	8.68	***	8.68
Tube b:LR	3,475	34.75	***	34.75
Tube c:UL	117	1.17	***	1.17
Tube c:UR	5,263	52.63	***	52.63
Tube c:LL	1,036	10.36	***	10.36
Tube c:LR	3,584	35.84	***	35.84

LDT

Annexin V FITC PI Assay

Sample ID: 5

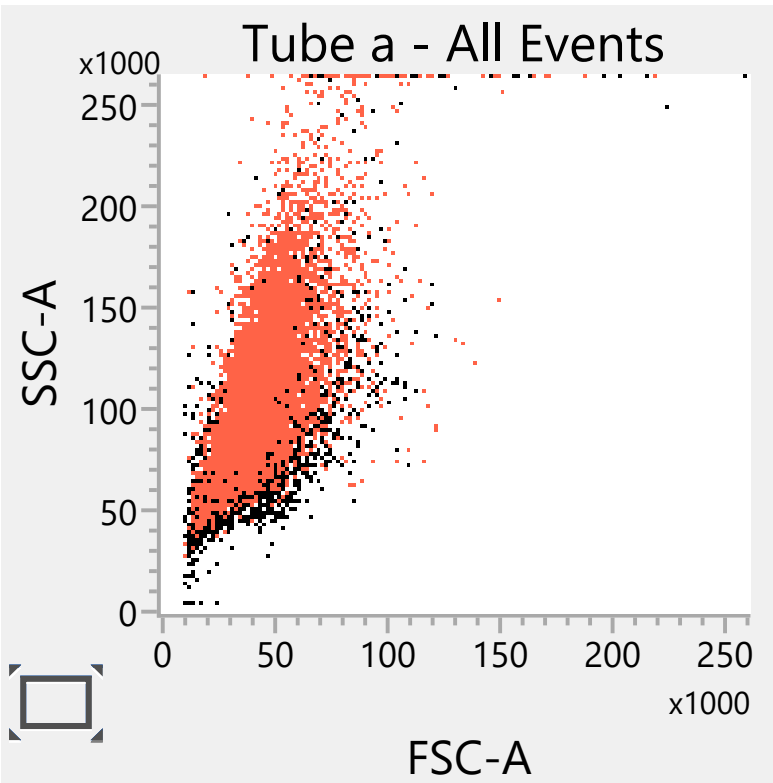

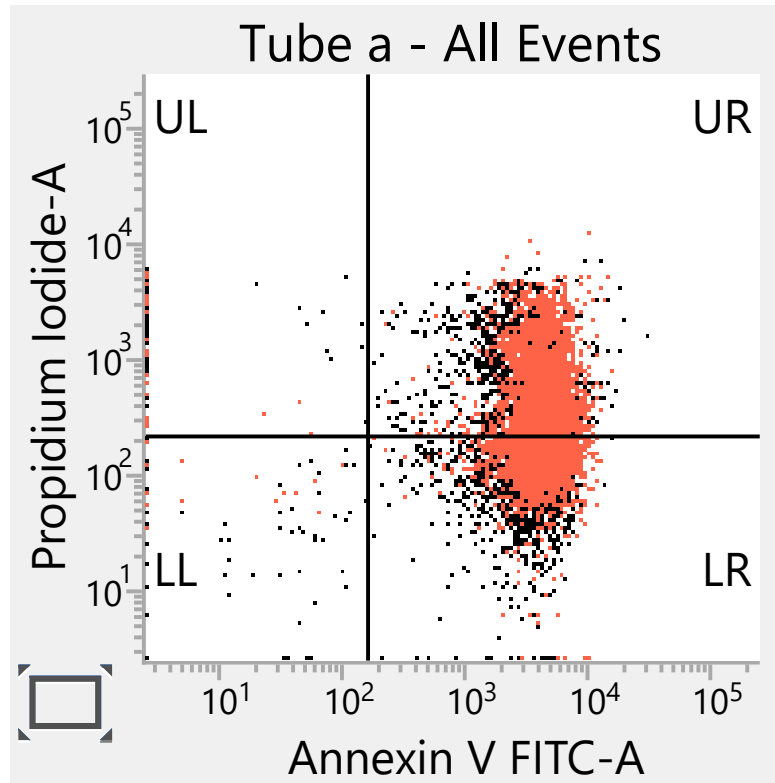

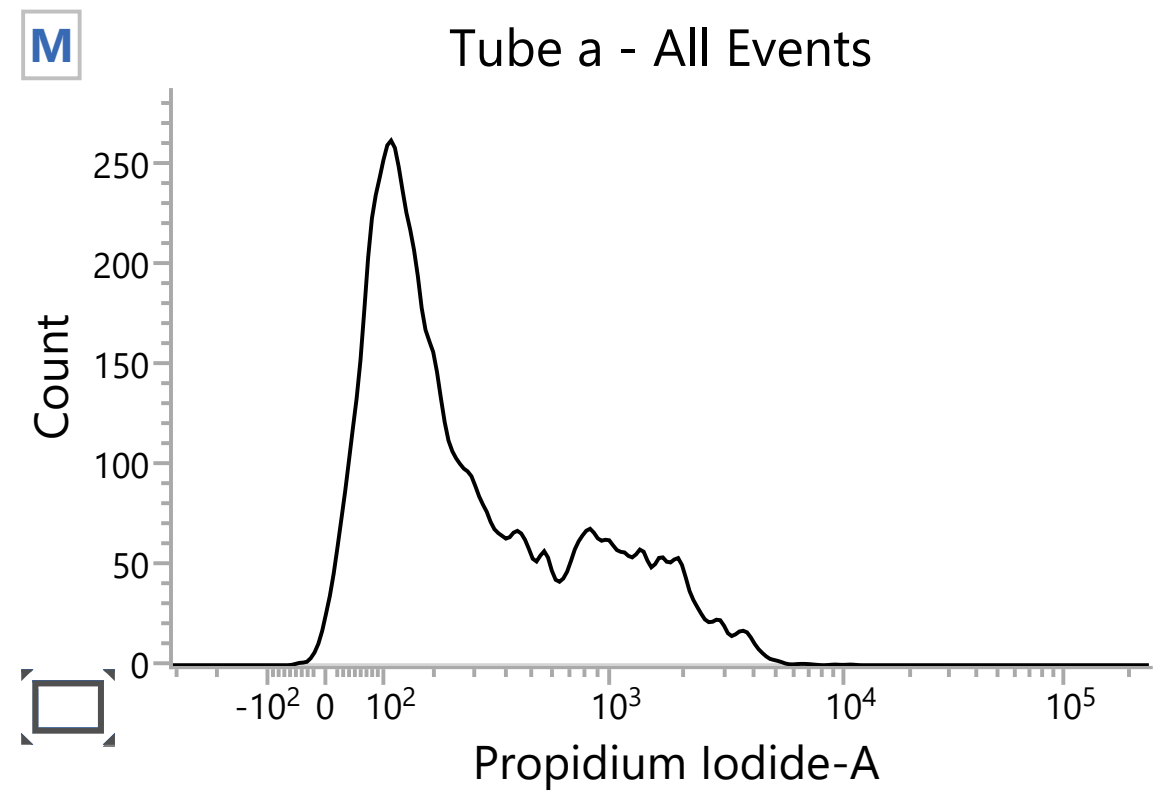

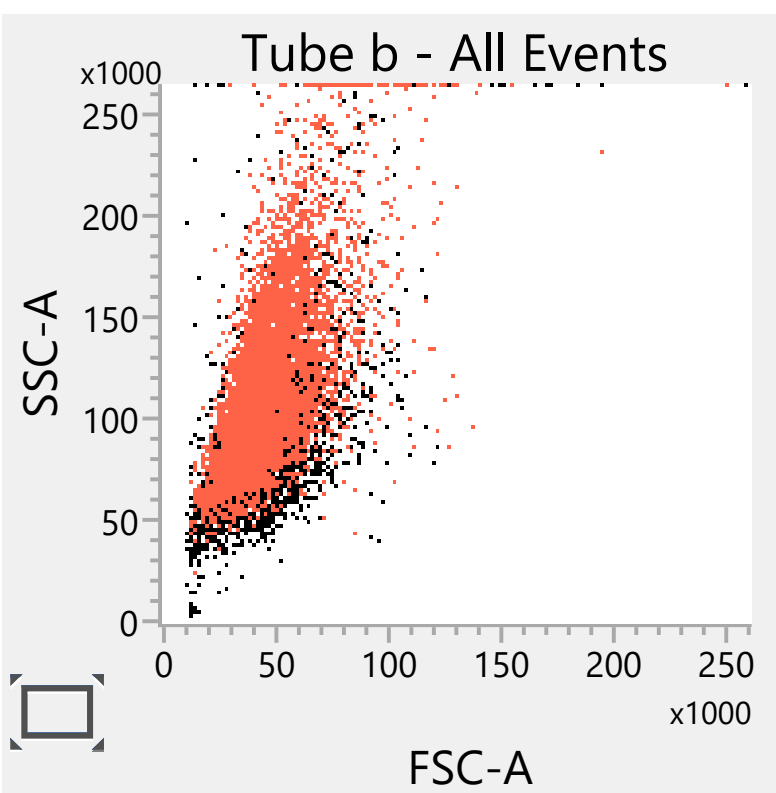

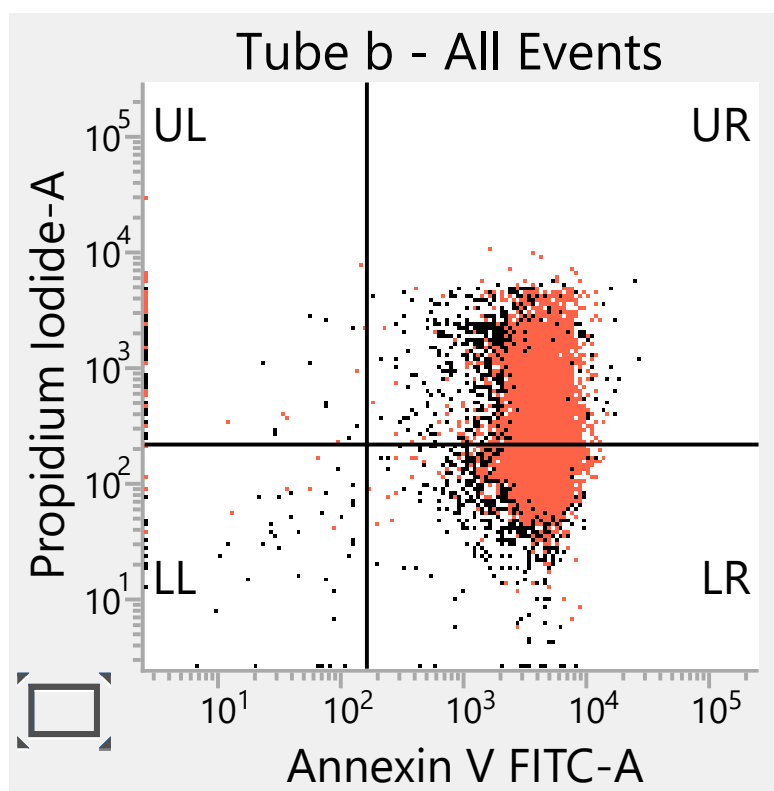

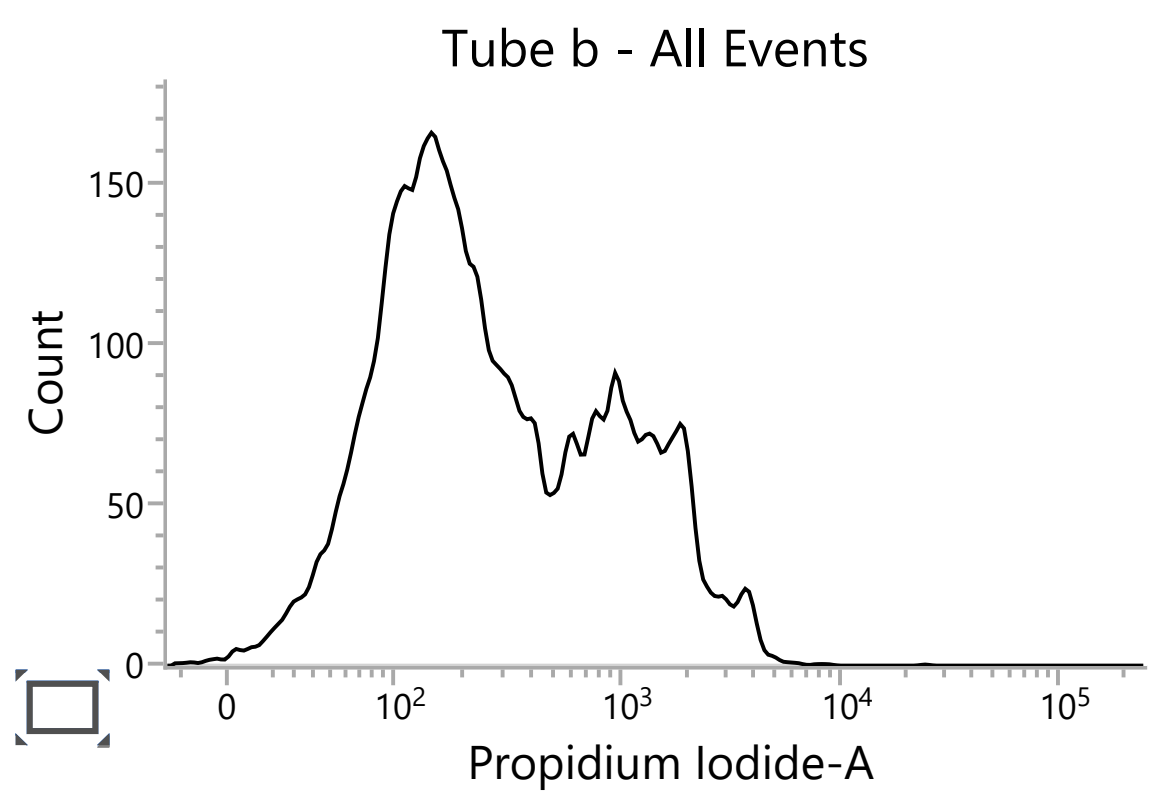

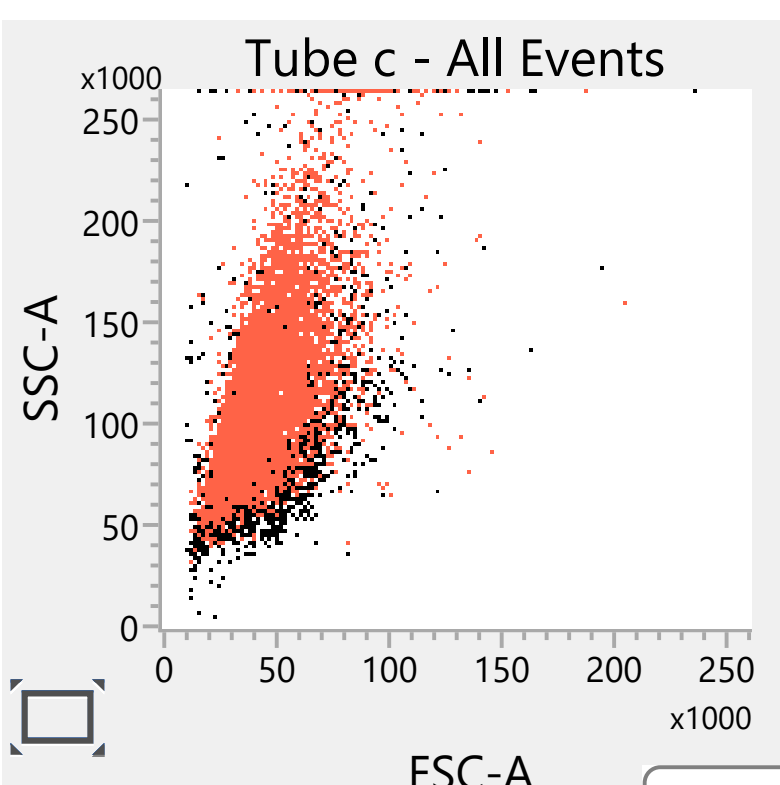

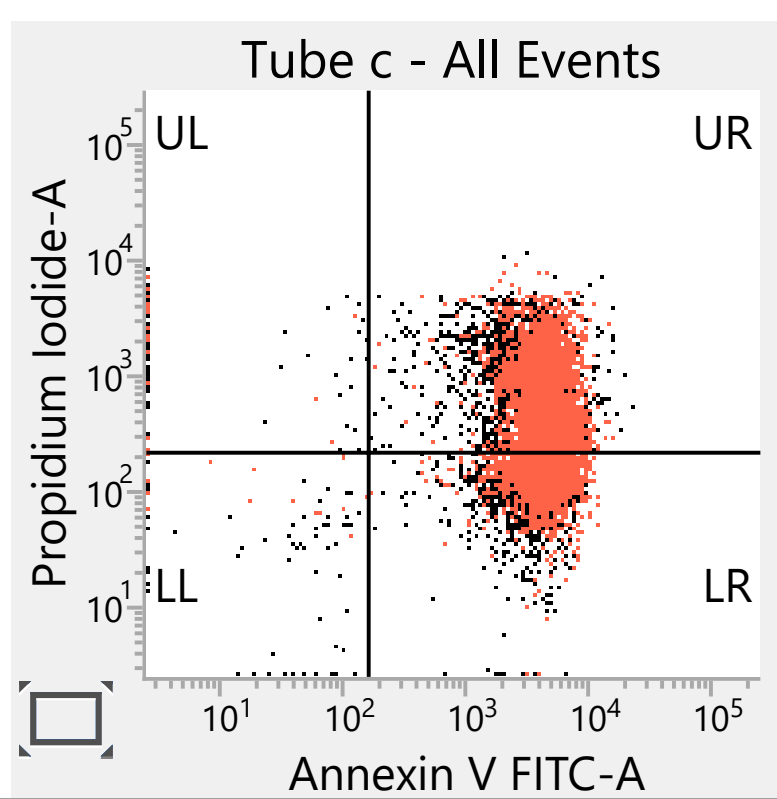

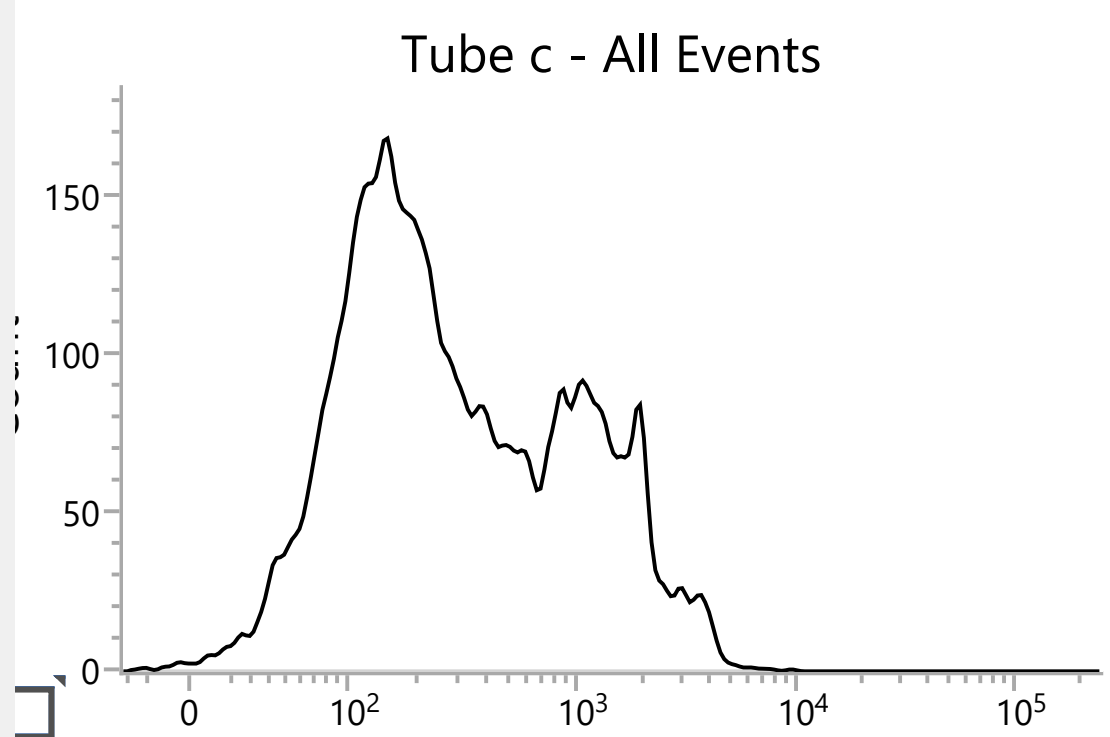

Propidium Iodide-A

Statistics

Name	Events	% Parent	% Grandparent	% Total
Tube a:All Events	10,000	***	***	100.00
Tube b:All Events	10,000	***	***	100.00
Tube c:All Events	10,000	***	***	100.00
Tube a:UL	83	0.83	***	0.83
Tube a:UR	4,883	48.83	***	48.83
Tube a:LL	75	0.75	***	0.75
Tube a:LR	4,959	49.59	***	49.59
Tube b:UL	93	0.93	***	0.93
Tube b:UR	5,225	52.25	***	52.25
Tube b:LL	53	0.53	***	0.53
Tube b:LR	4,629	46.29	***	46.29
Tube c:UL	94	0.94	***	0.94
Tube c:UR	5,539	55.39	***	55.39
Tube c:LL	73	0.73	***	0.73
Tube c:LR	4,294	42.94	***	42.94

LDT

Annexin V FITC PI Assay

Sample ID: DMSO

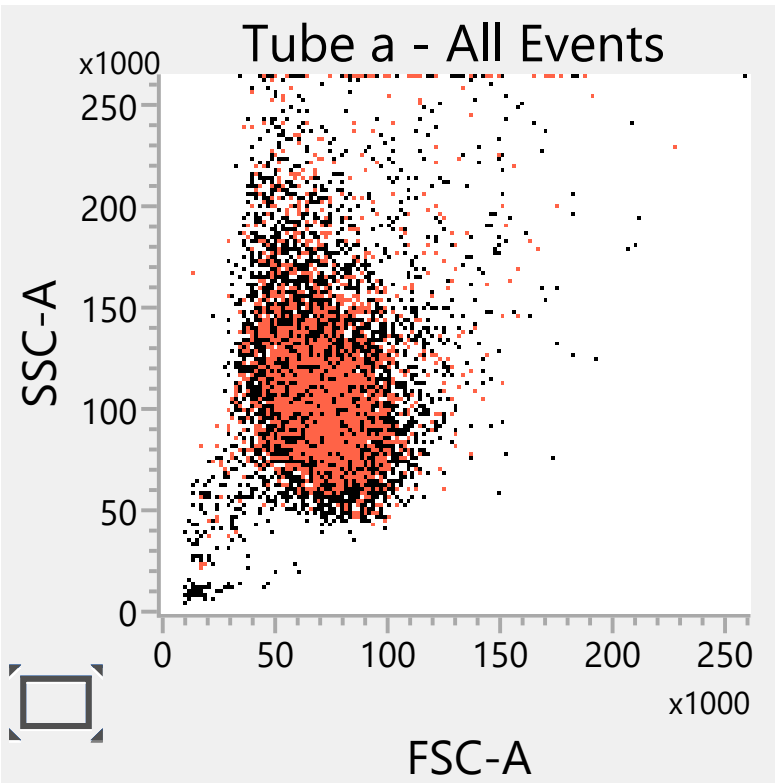

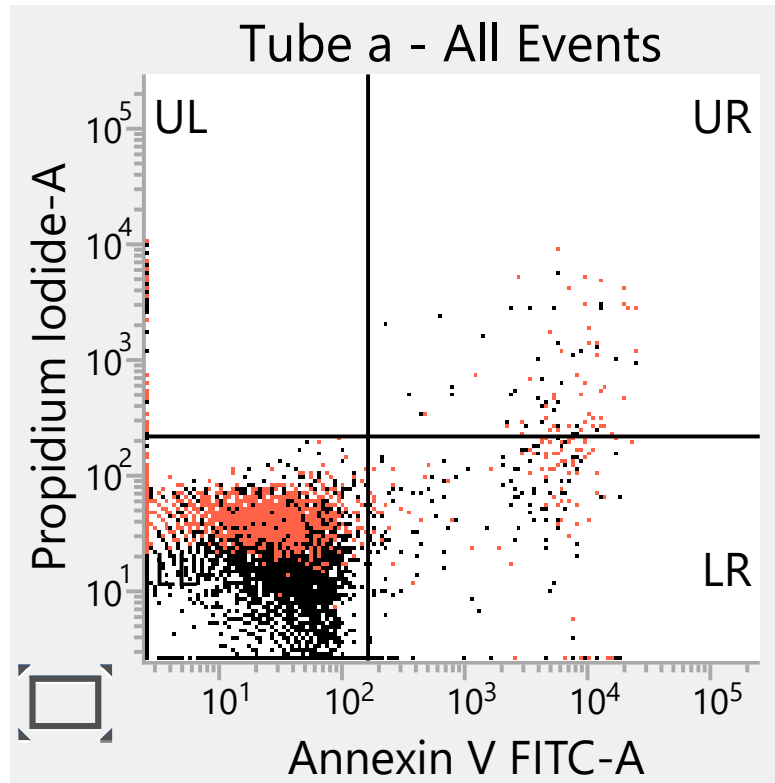

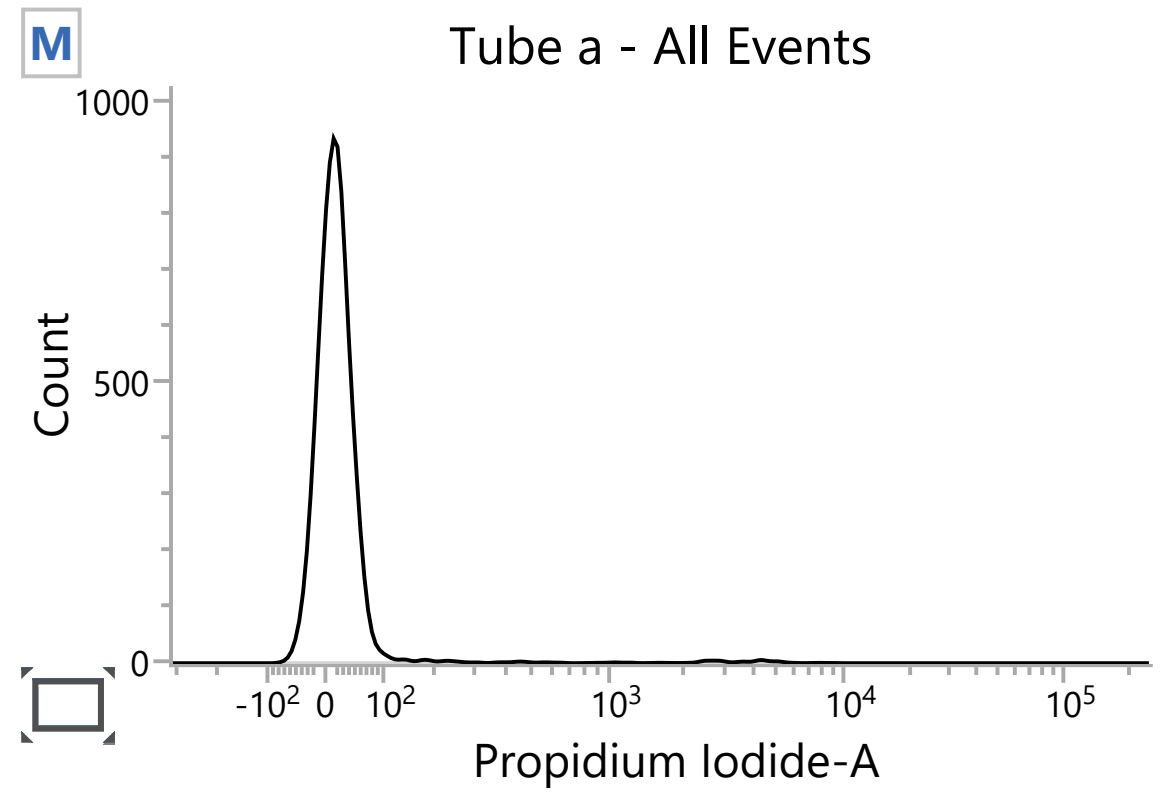

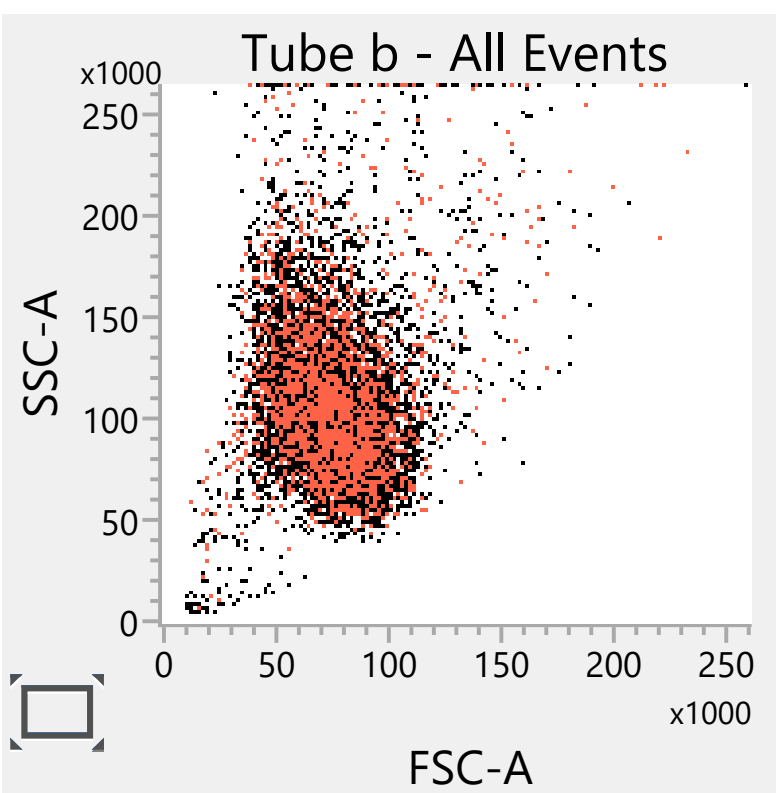

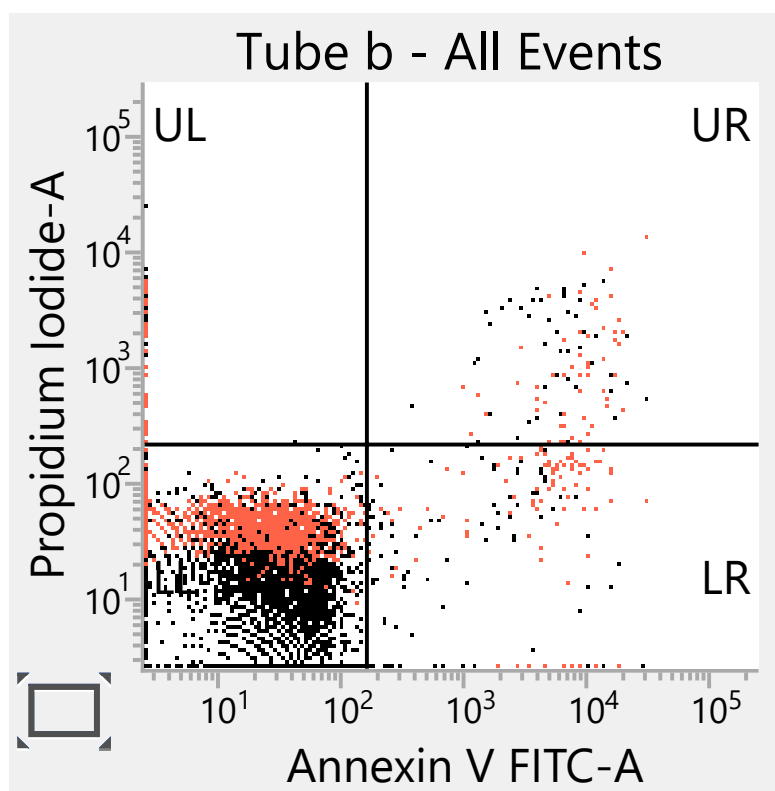

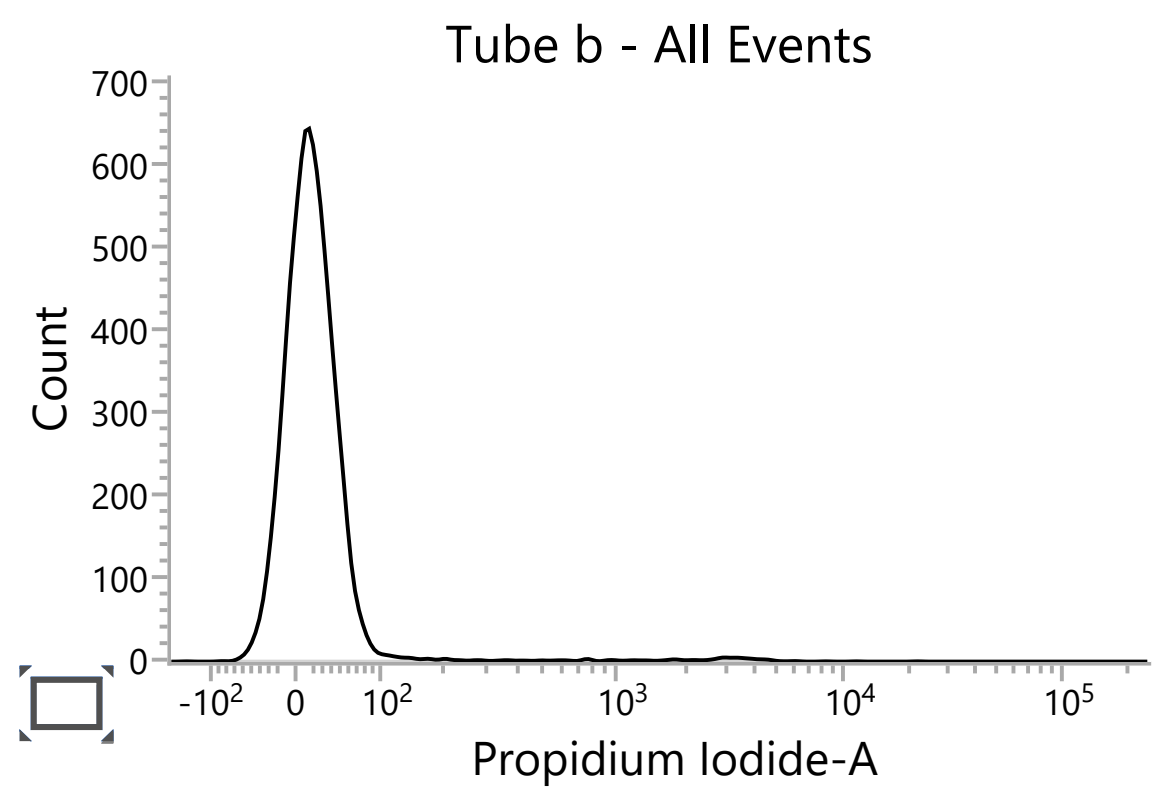

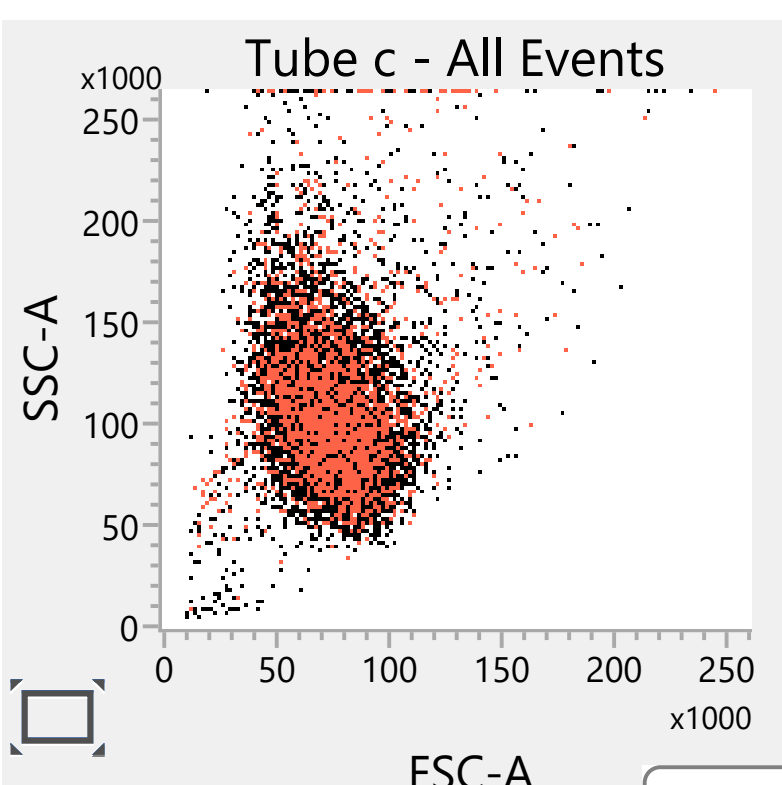

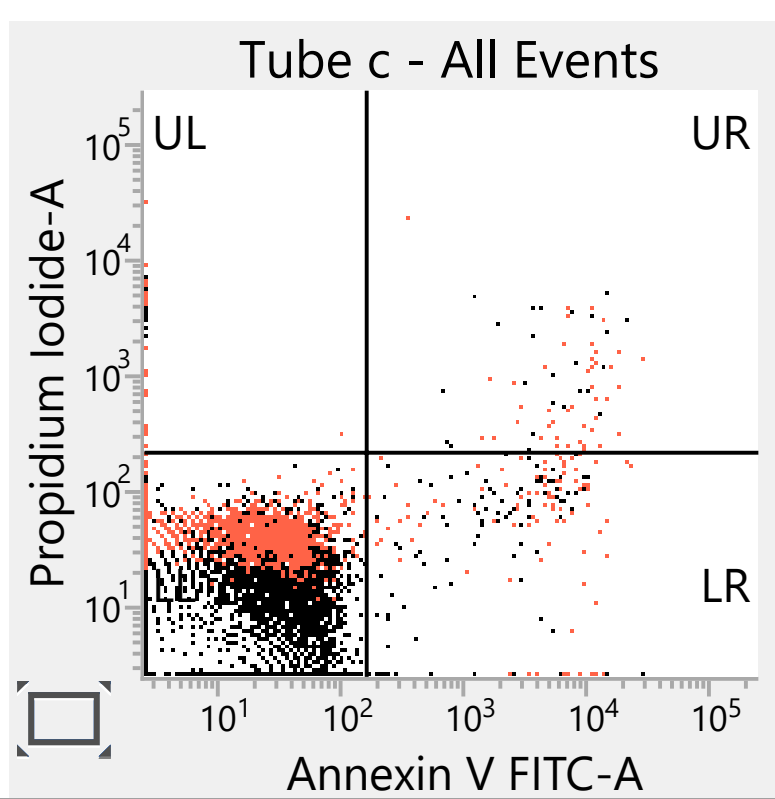

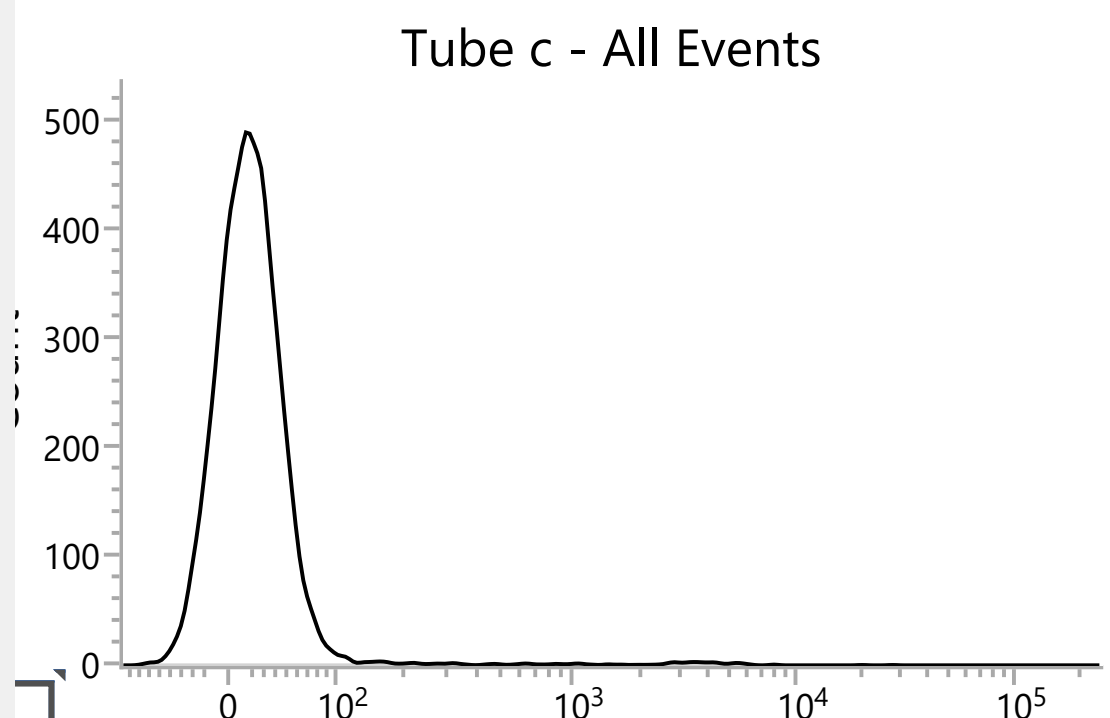

Propidium Iodide-A

Statistics

Name	Events	% Parent	% Grandparent	% Total
Tube a:All Events	10,000	***	***	100.00
Tube b:All Events	10,000	***	***	100.00
Tube c:All Events	10,000	***	***	100.00
Tube a:UL	94	0.94	***	0.94
Tube a:UR	84	0.84	***	0.84
Tube a:LL	9,579	95.79	***	95.79
Tube a:LR	243	2.43	***	2.43
Tube b:UL	80	0.80	***	0.80
Tube b:UR	102	1.02	***	1.02
Tube b:LL	9,623	96.23	***	96.23
Tube b:LR	195	1.95	***	1.95
Tube c:UL	62	0.62	***	0.62
Tube c:UR	69	0.69	***	0.69
Tube c:LL	9,670	96.70	***	96.70
Tube c:LR	199	1.99	***	1.99

LDT

Annexin V FITC PI Assay

Sample ID: K+A

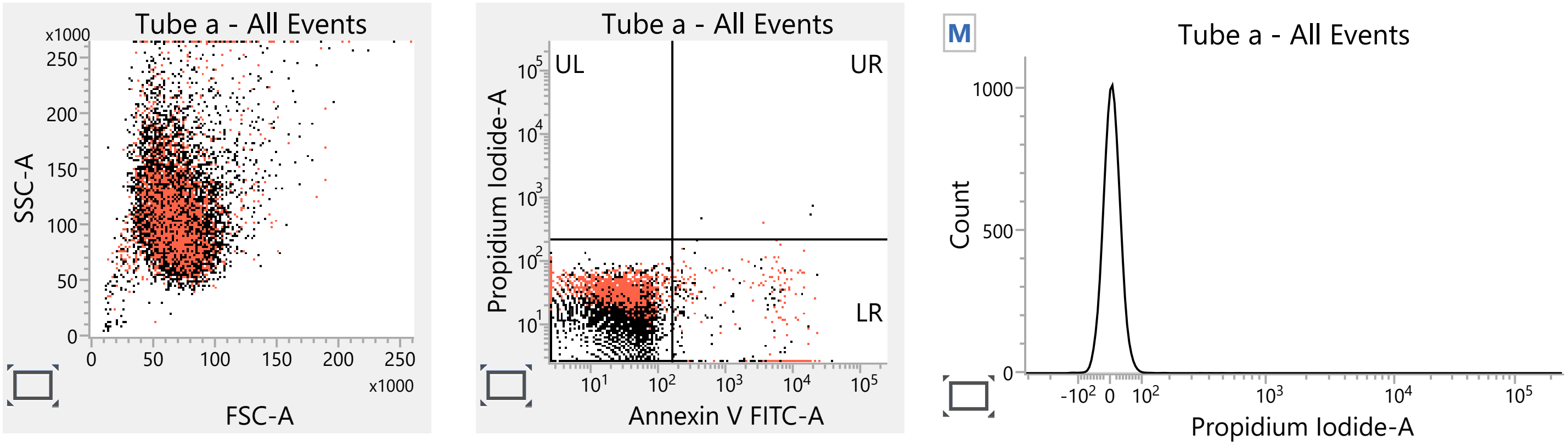

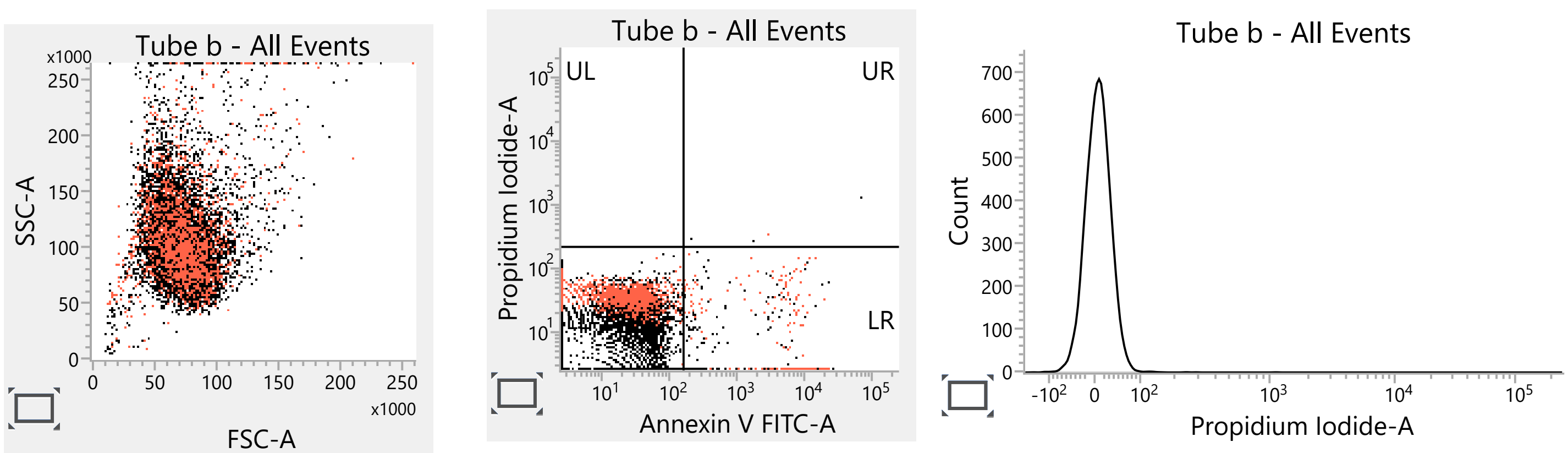

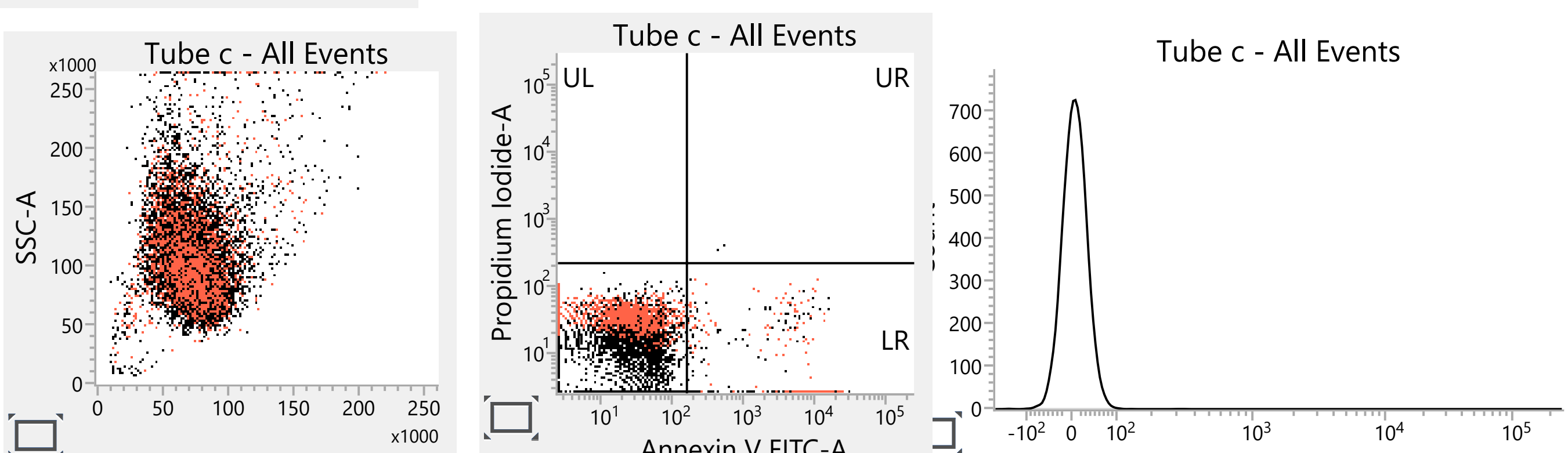

Statistics

Name	Events	% Parent	% Grandparent	% Total
Tube a:All Events	10,000	***	***	100.00
Tube b:All Events	10,000	***	***	100.00
Tube c:All Events	10,000	***	***	100.00
Tube a:UL	0	0.00	***	0.00
Tube a:UR	4	0.04	***	0.04
Tube a:LL	9,643	96.43	***	96.43
Tube a:LR	353	3.53	***	3.53
Tube b:UL	0	0.00	***	0.00
Tube b:UR	4	0.04	***	0.04
Tube b:LL	9,614	96.14	***	96.14
Tube b:LR	382	3.82	***	3.82
Tube c:UL	0	0.00	***	0.00
Tube c:UR	2	0.02	***	0.02
Tube c:LL	9,684	96.84	***	96.84
Tube c:LR	314	3.14	***	3.14

LDT

Annexin V FITC PI Assay

Sample ID: K+P

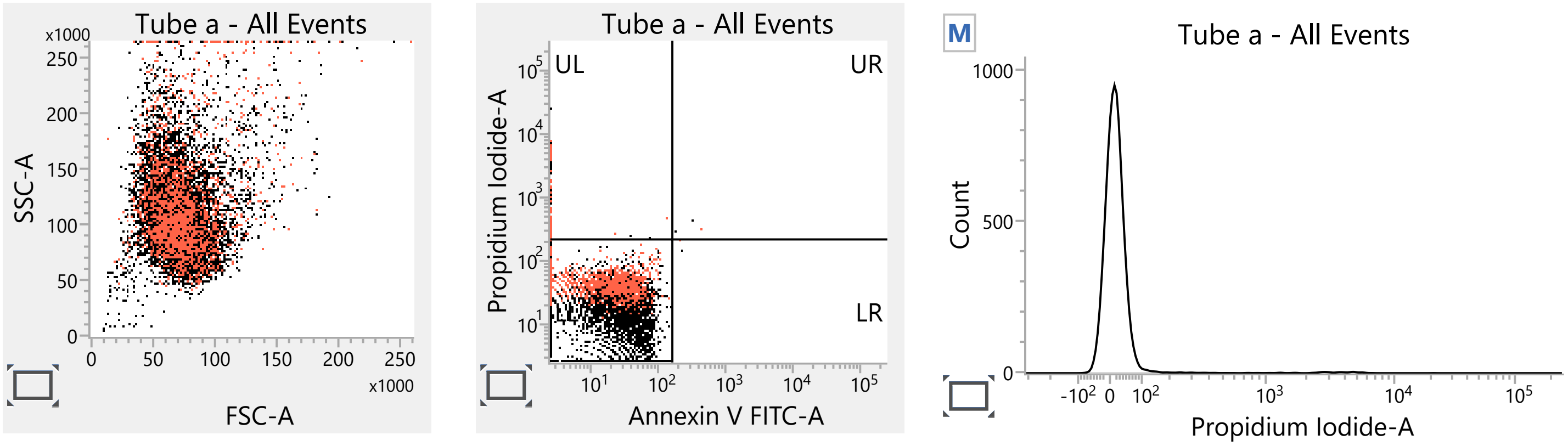

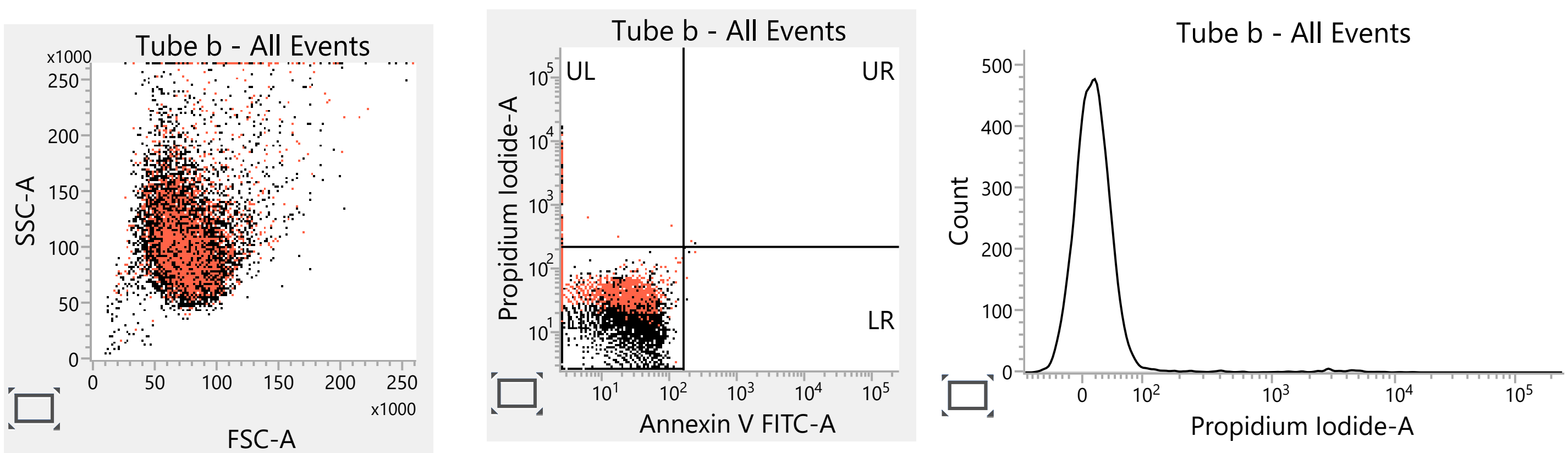

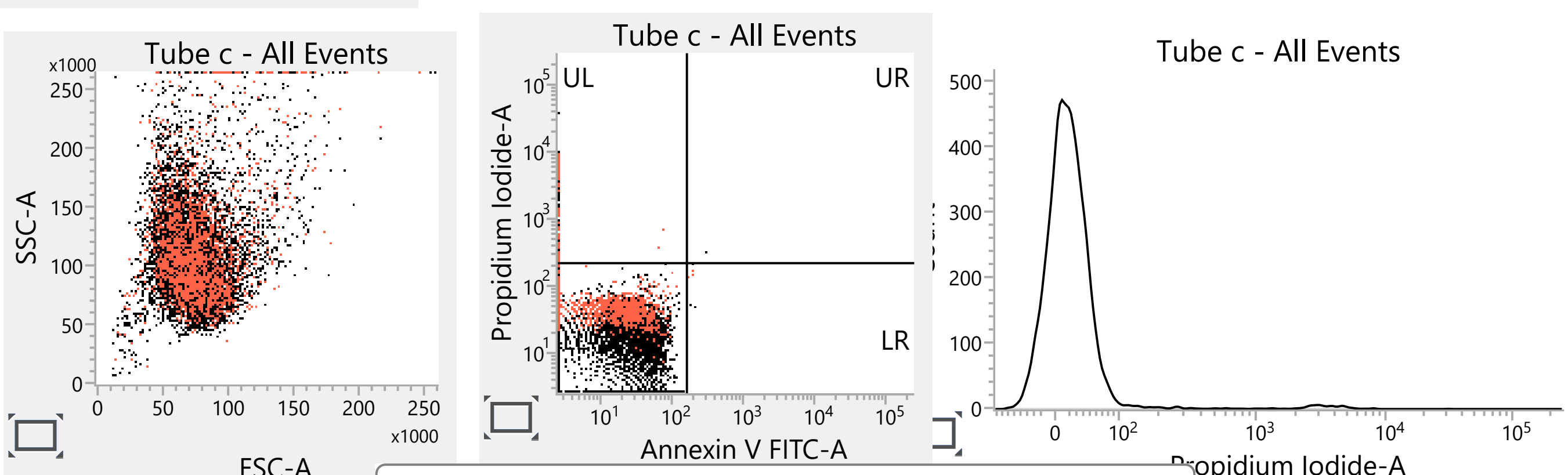

Statistics

Name	Events	% Parent	% Grandparent	% Total
Tube a:All Events	10,000	***	***	100.00
Tube b:All Events	10,000	***	***	100.00
Tube c:All Events	10,000	***	***	100.00
Tube a:UL	131	1.31	***	1.31
Tube a:UR	3	0.03	***	0.03
Tube a:LL	9,863	98.63	***	98.63
Tube a:LR	3	0.03	***	0.03
Tube b:UL	152	1.52	***	1.52
Tube b:UR	2	0.02	***	0.02
Tube b:LL	9,839	98.39	***	98.39
Tube b:LR	7	0.07	***	0.07
Tube c:UL	181	1.81	***	1.81
Tube c:UR	1	0.01	***	0.01
Tube c:LL	9,812	98.12	***	98.12
Tube c:LR	6	0.06	***	0.06

LDT

Annexin V FITC PI Assay

Sample ID: KAP

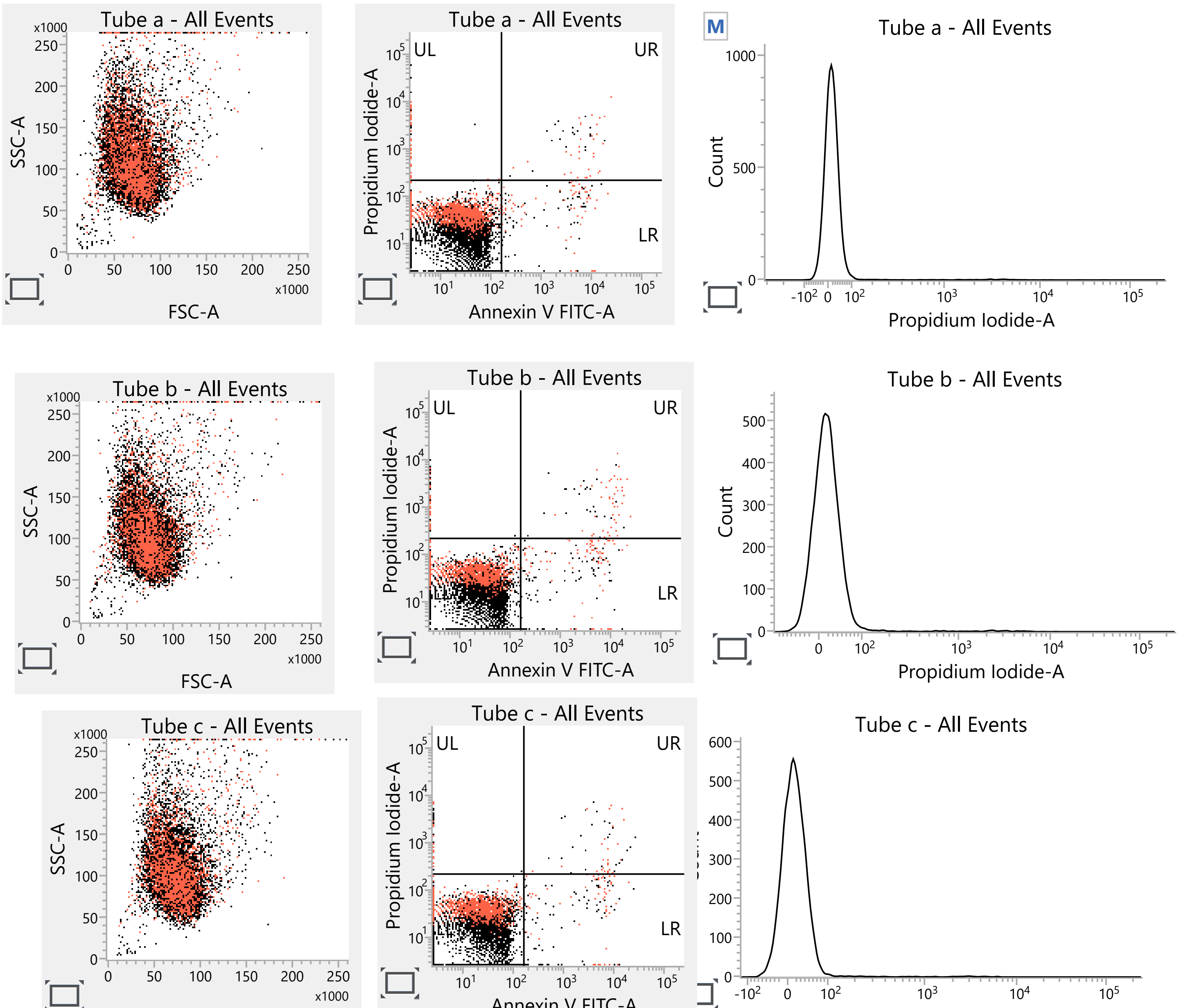

Statistics

Name	Events	% Parent	% Grandparent	% Total
Tube a:All Events	10,000	***	***	100.00
Tube b:All Events	10,000	***	***	100.00
Tube c:All Events	10,000	***	***	100.00
Tube a:UL	65	0.65	***	0.65
Tube a:UR	52	0.52	***	0.52
Tube a:LL	9,735	97.35	***	97.35
Tube a:LR	148	1.48	***	1.48
Tube b:UL	58	0.58	***	0.58
Tube b:UR	68	0.68	***	0.68
Tube b:LL	9,696	96.96	***	96.96
Tube b:LR	178	1.78	***	1.78
Tube c:UL	40	0.40	***	0.40
Tube c:UR	50	0.50	***	0.50
Tube c:LL	9,769	97.69	***	97.69
Tube c:LR	141	1.41	***	1.41

LDT

Annexin V FITC PI Assay

Sample ID: N6_K

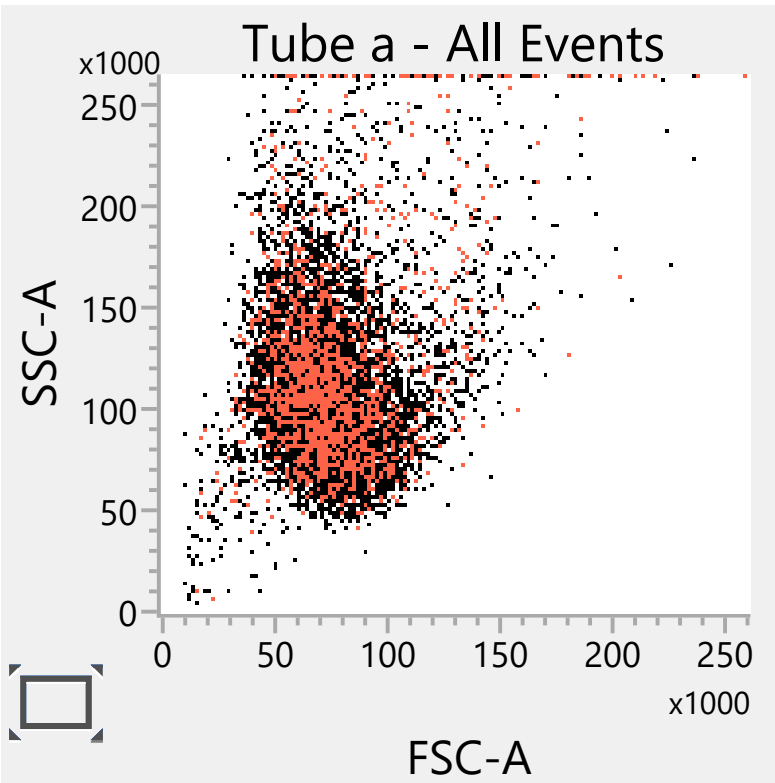

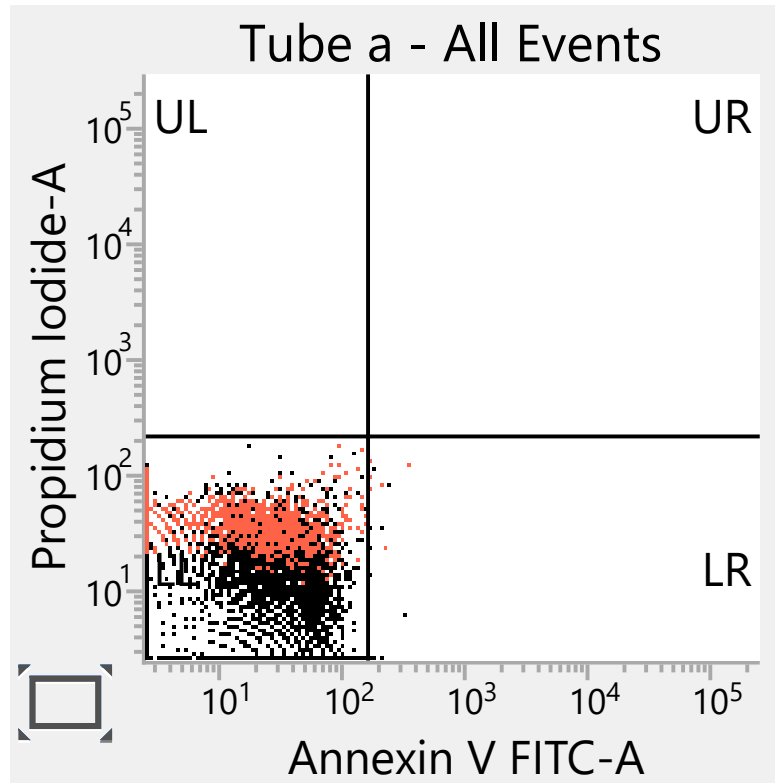

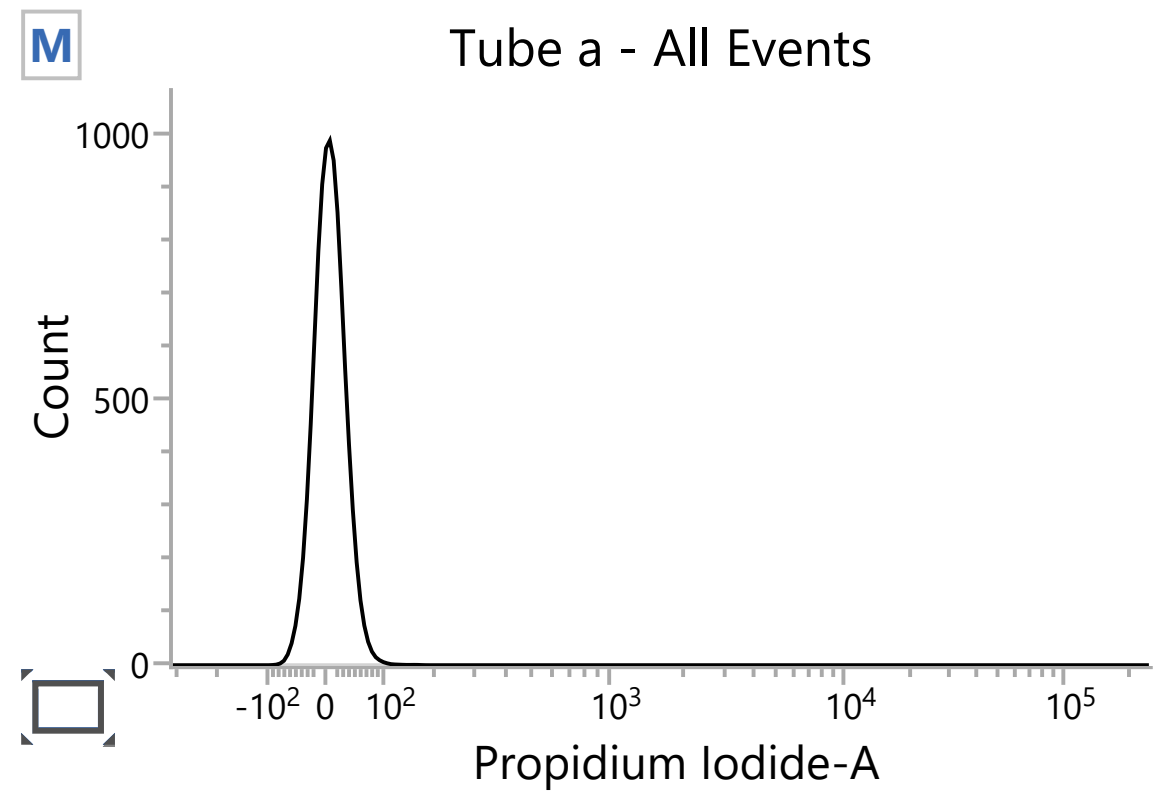

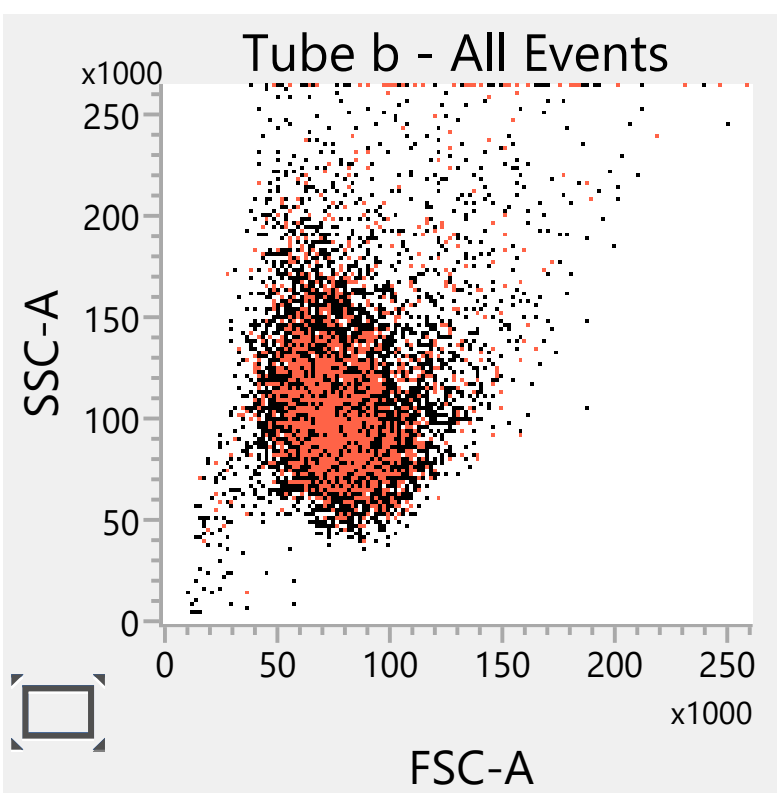

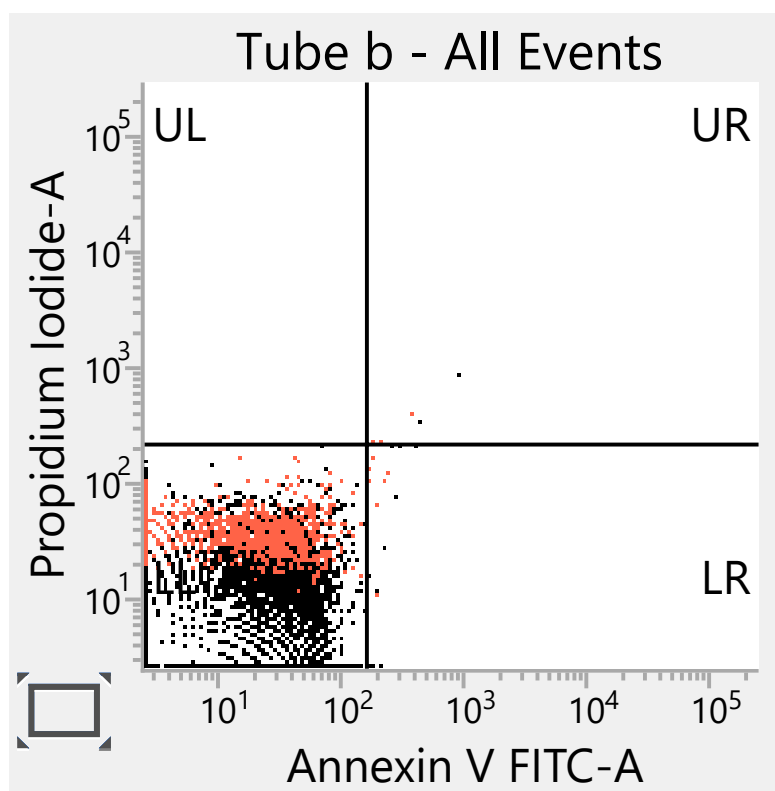

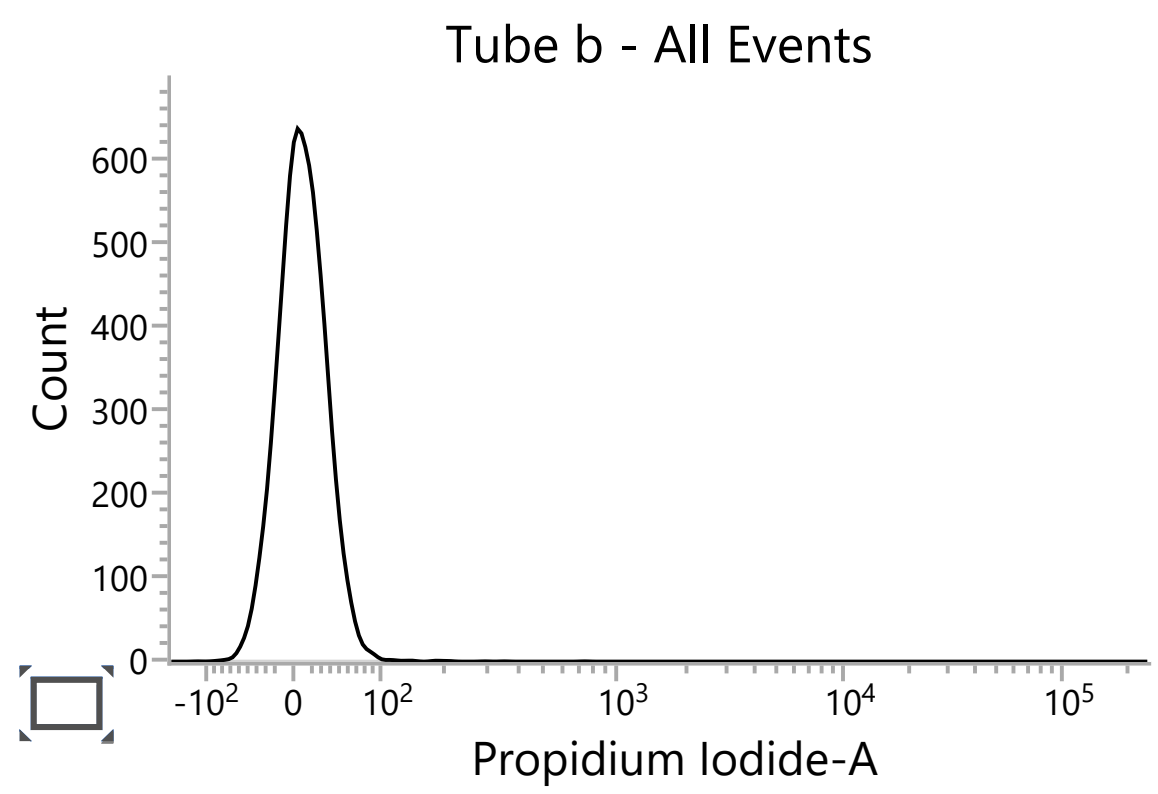

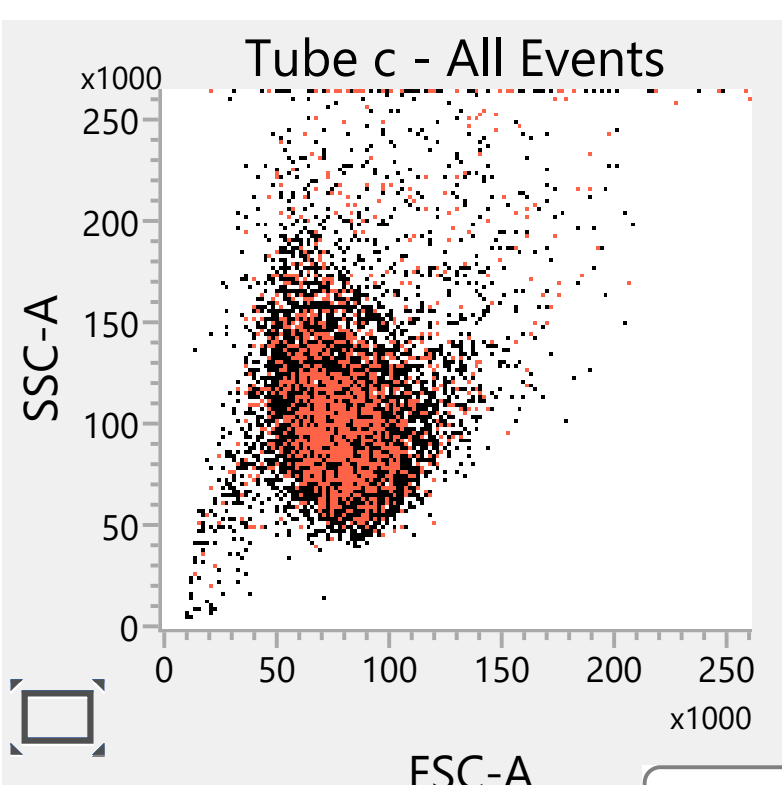

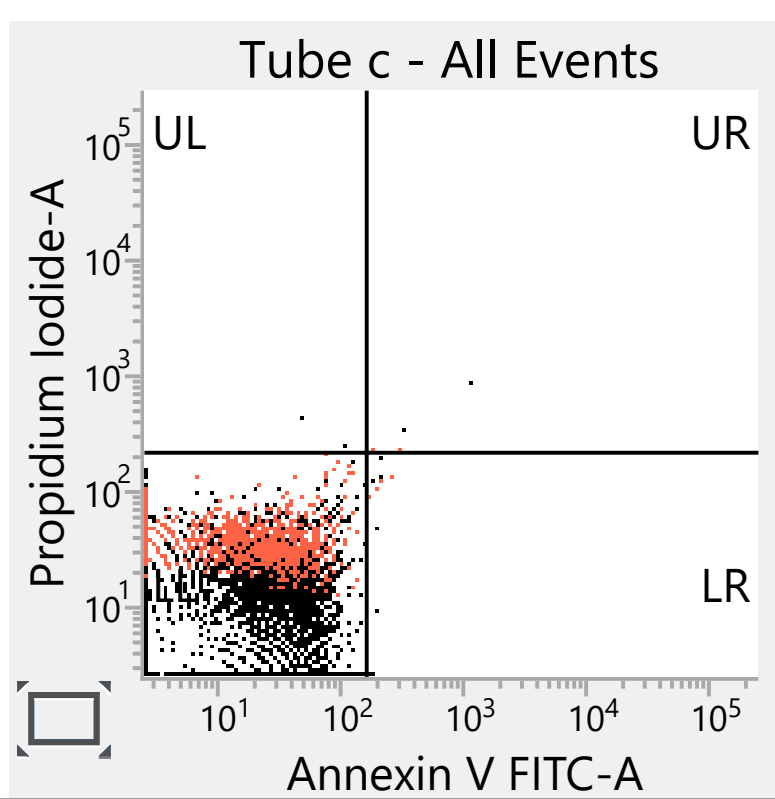

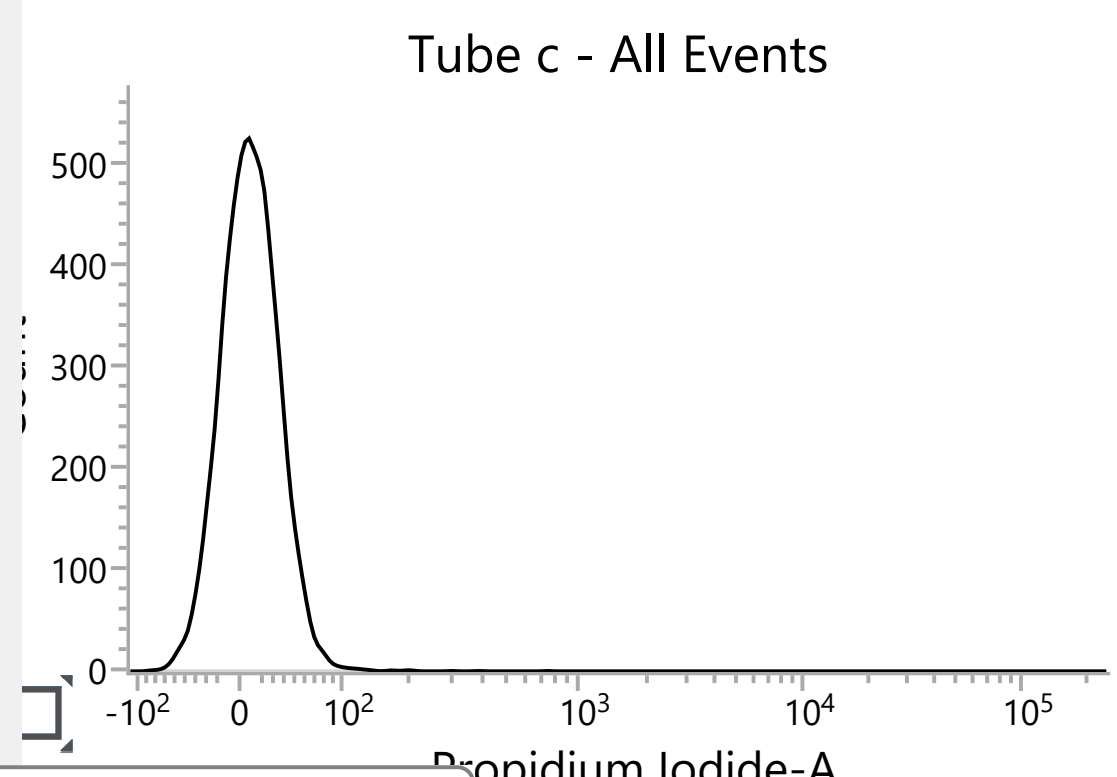

Statistics

Name	Events	% Parent	% Grandparent	% Total
Tube a:All Events	10,000	***	***	100.00
Tube b:All Events	10,000	***	***	100.00
Tube c:All Events	10,000	***	***	100.00
Tube a:UL	0	0.00	***	0.00
Tube a:UR	0	0.00	***	0.00
Tube a:LL	9,975	99.75	***	99.75
Tube a:LR	25	0.25	***	0.25
Tube b:UL	0	0.00	***	0.00
Tube b:UR	5	0.05	***	0.05
Tube b:LL	9,974	99.74	***	99.74
Tube b:LR	21	0.21	***	0.21
Tube c:UL	2	0.02	***	0.02
Tube c:UR	2	0.02	***	0.02
Tube c:LL	9,981	99.81	***	99.81
Tube c:LR	15	0.15	***	0.15

LDT

DNA Fragmentierung

Sample ID: JeFri - ATM N6 - 0,1

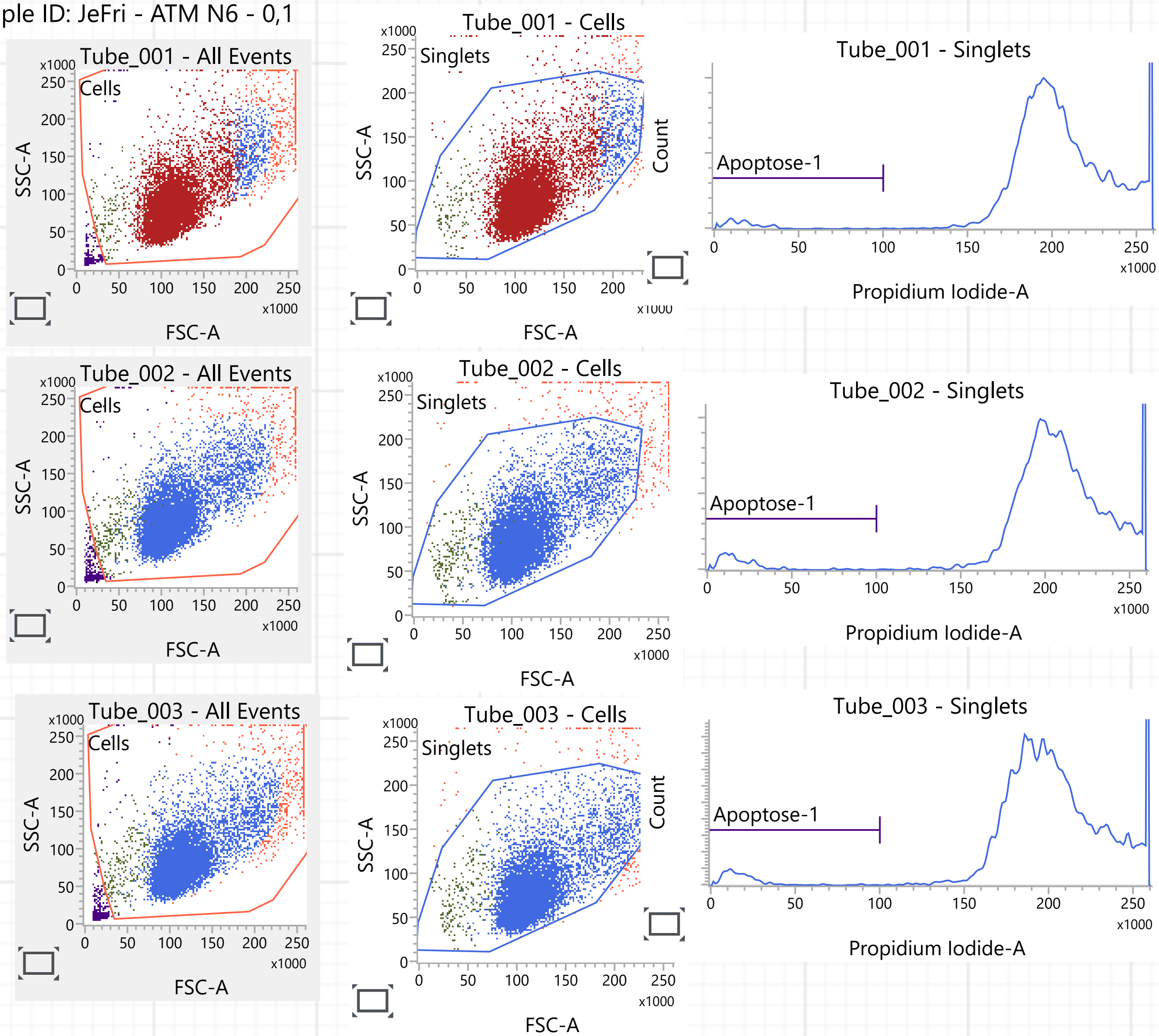

Statistics

Prozent apoptotische Zellen: Prozent apoptotische Zellen No Value % von Cells

Name	Events	% Total	% Parent	% Grandparent
Tube_001:All Events	10,167	100.00	***	***
Tube_001:Cells	10,000	98.36	98.36	***
Tube_001:Singlets	9,283	91.31	92.83	91.31
Tube_002:All Events	10,000	100.00	***	***
Tube_002:Cells	9,755	97.55	97.55	***
Tube_002:Singlets	9,226	92.26	94.58	92.26
Tube_001:Apoptose-1	323	3.18	3.18	***
Tube_002:Apoptose-1	505	5.05	5.05	***
Tube_002:Apoptose-2	241	2.41	2.61	2.47

DNA Fragmentierung

Sample ID: JeFri - ATM N6 - 0,05

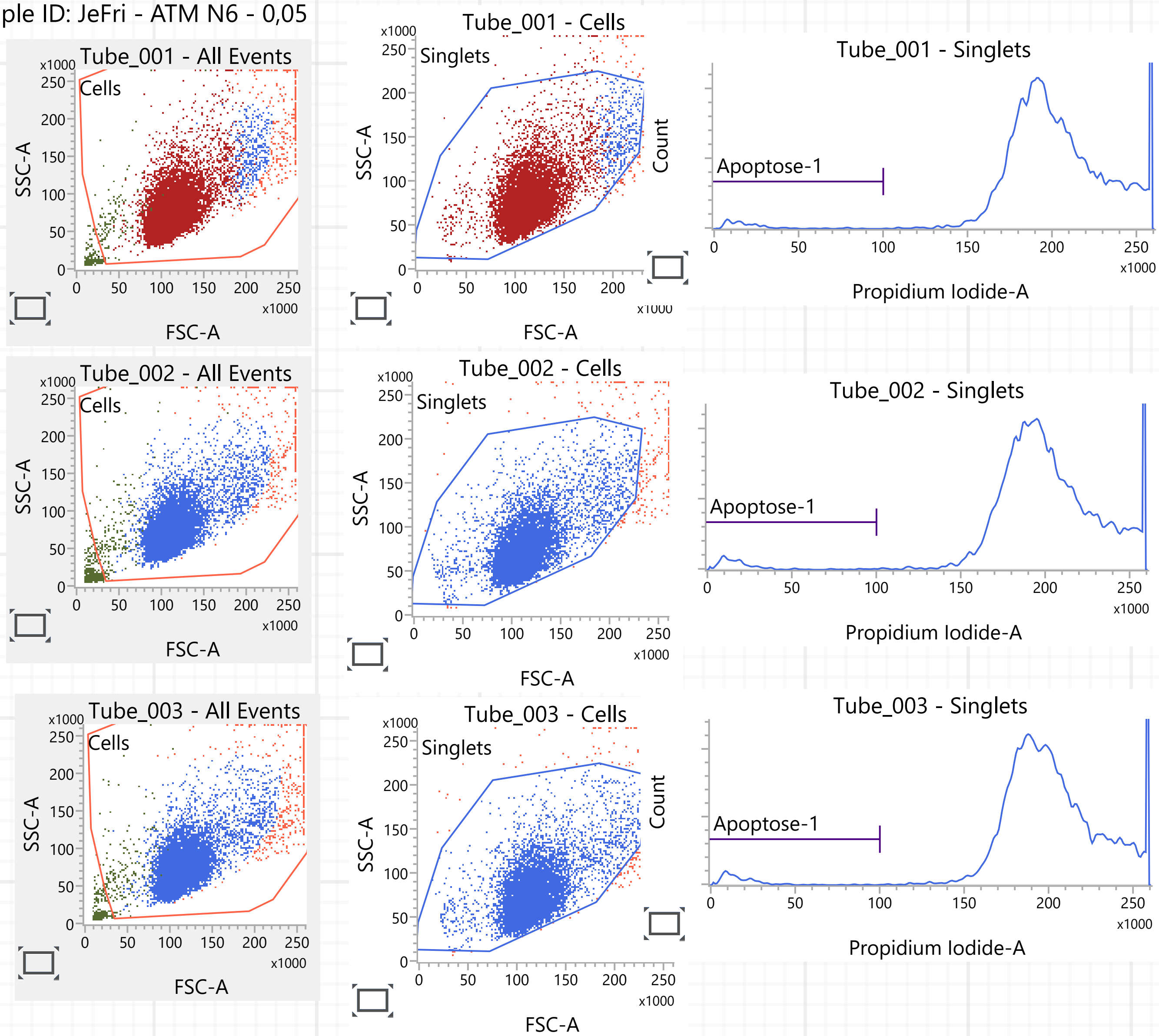

Statistics

Prozent apoptotische Zellen: Prozent apoptotische Zellen No Value % von Cells

Name	Events	% Total	% Parent	% Grandparent
Tube_001:All Events	10,244	100.00	***	***
Tube_001:Cells	10,000	97.62	97.62	***
Tube_001:Singlets	9,557	93.29	95.57	93.29
Tube_002:All Events	10,000	100.00	***	***
Tube_002:Cells	9,592	95.92	95.92	***
Tube_002:Singlets	9,240	92.40	96.33	92.40
Tube_003:All Events	10,000	100.00	***	***
Tube_003:Cells	9,784	97.84	97.84	***
Tube_003:Singlets	9,295	92.95	95.00	92.95
Tube_001:Apoptose-1	374	3.65	3.65	***
Tube_002:Apoptose-1	605	6.05	6.05	***
Tube_003:Apoptose-1	404	4.04	4.04	***

DNA Fragmentierung

Sample ID: JeFri - ATM N6 - 0,5

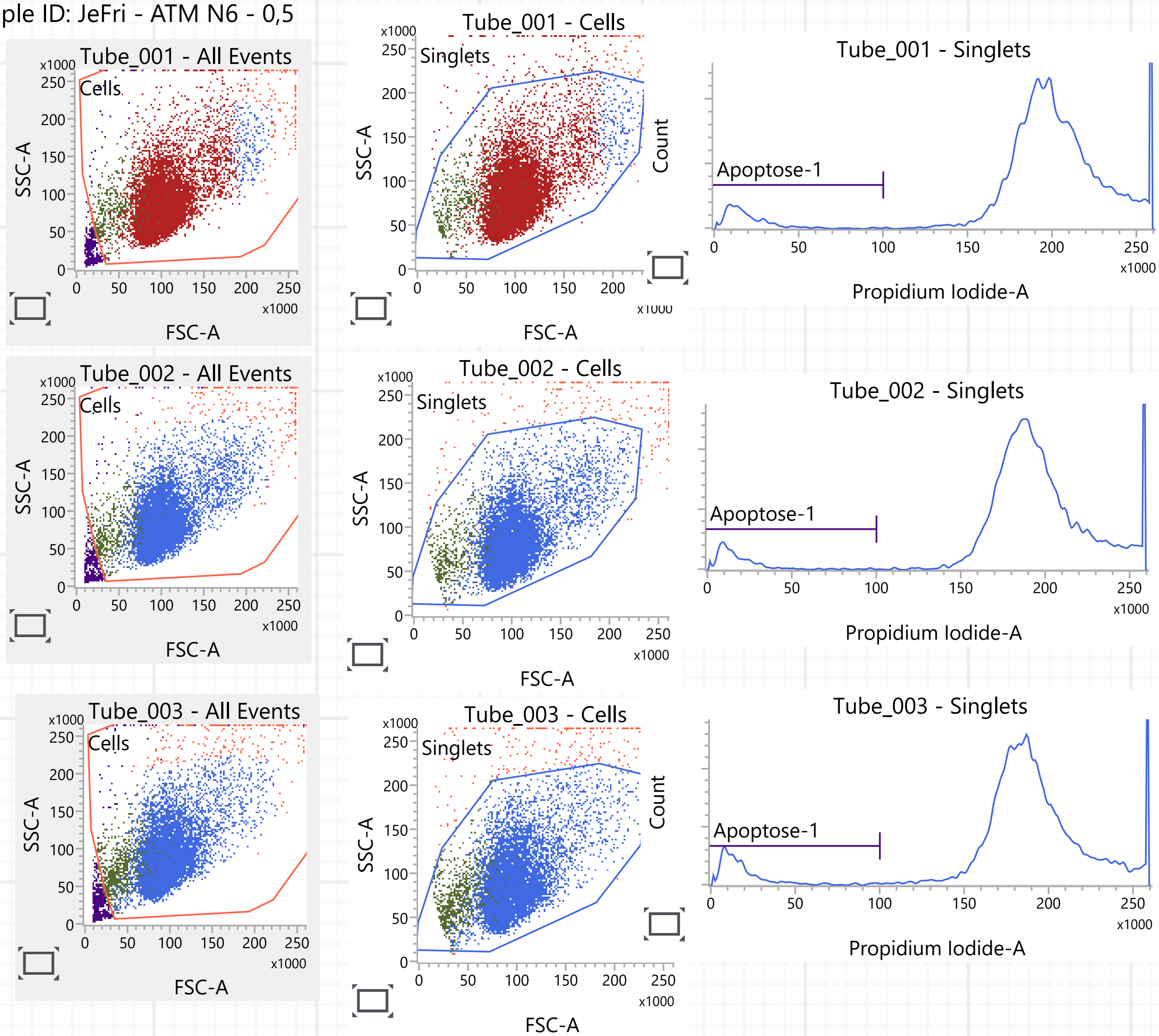

Statistics

Prozent apoptotische Zellen: Prozent apoptotische Zellen No Value % von Cells

Name	Events	% Total	% Parent	% Grandparent
Tube_001:All Events	10,337	100.00	***	***
Tube_001:Cells	10,000	96.74	96.74	***
Tube_001:Singlets	9,688	93.72	96.88	93.72
Tube_002:All Events	10,000	100.00	***	***
Tube_002:Cells	9,598	95.98	95.98	***
Tube_002:Singlets	9,329	93.29	97.20	93.29
Tube_003:All Events	10,000	100.00	***	***
Tube_003:Cells	9,403	94.03	94.03	***
Tube_003:Singlets	9,129	91.29	97.09	91.29
Tube_001:Apoptose-1	795	7.69	7.69	***
Tube_002:Apoptose-1	888	8.88	8.88	***
Tube_003:Apoptose-1	1,298	12.98	12.98	***

DNA Fragmentierung

Sample ID: JeFri - ATM N6 - 0,25

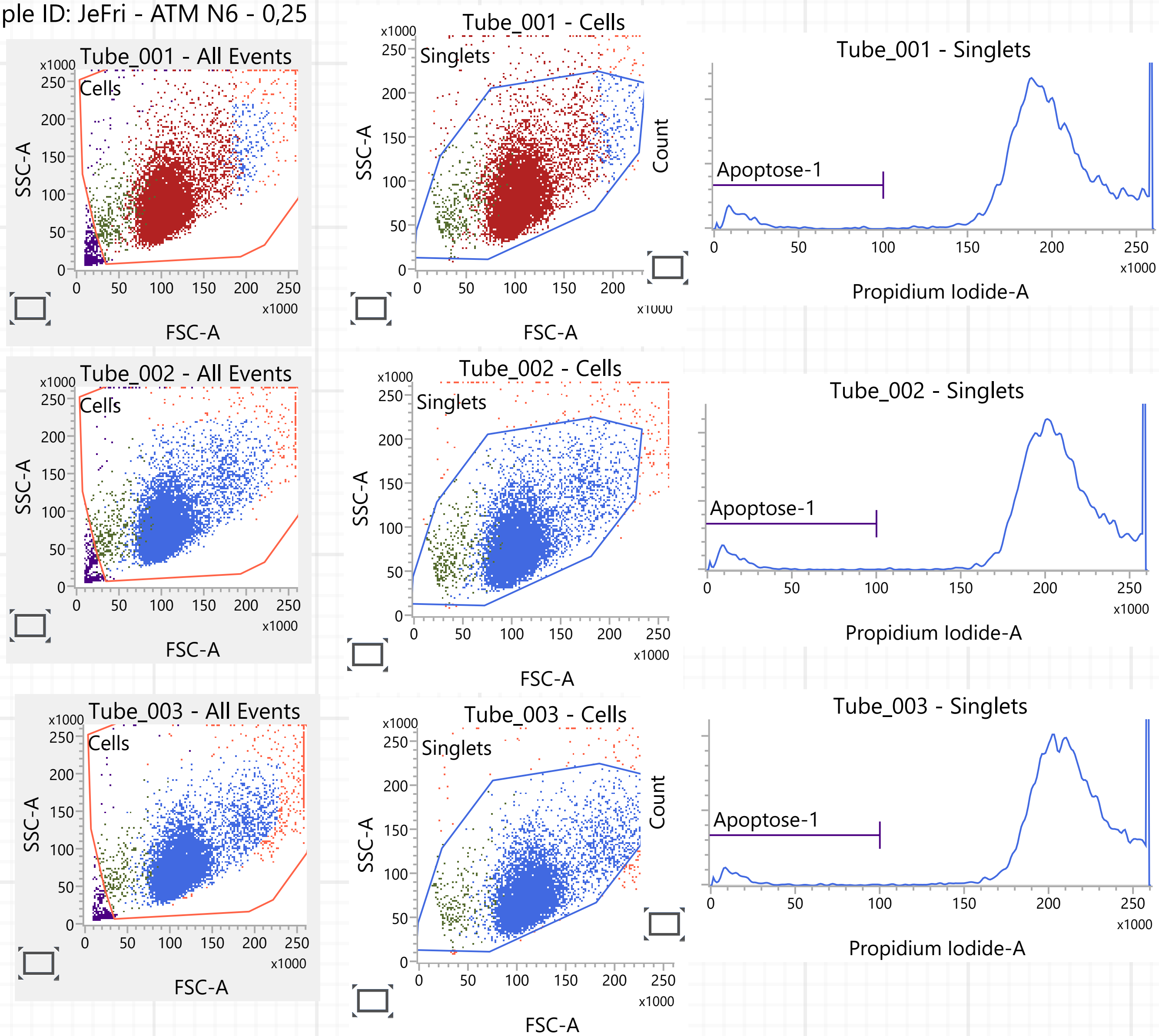

Statistics

Prozent apoptotische Zellen: Prozent apoptotische Zellen No Value % von Cells

Name	Events	% Total	% Parent	% Grandparent
Tube_001:All Events	10,411	100.00	***	***
Tube_001:Cells	10,000	96.05	96.05	***
Tube_001:Singlets	9,726	93.42	97.26	93.42
Tube_002:All Events	10,000	100.00	***	***
Tube_002:Cells	9,722	97.22	97.22	***
Tube_002:Singlets	9,437	94.37	97.07	94.37
Tube_003:All Events	10,000	100.00	***	***
Tube_003:Cells	9,708	97.08	97.08	***
Tube_003:Singlets	9,276	92.76	95.55	92.76
Tube_001:Apoptose-1	794	7.63	7.63	***
Tube_002:Apoptose-1	628	6.28	6.28	***
Tube_003:Apoptose-1	535	5.35	5.35	***

DNA Fragmentierung

Sample ID: JeFri - ATM N6 - 1

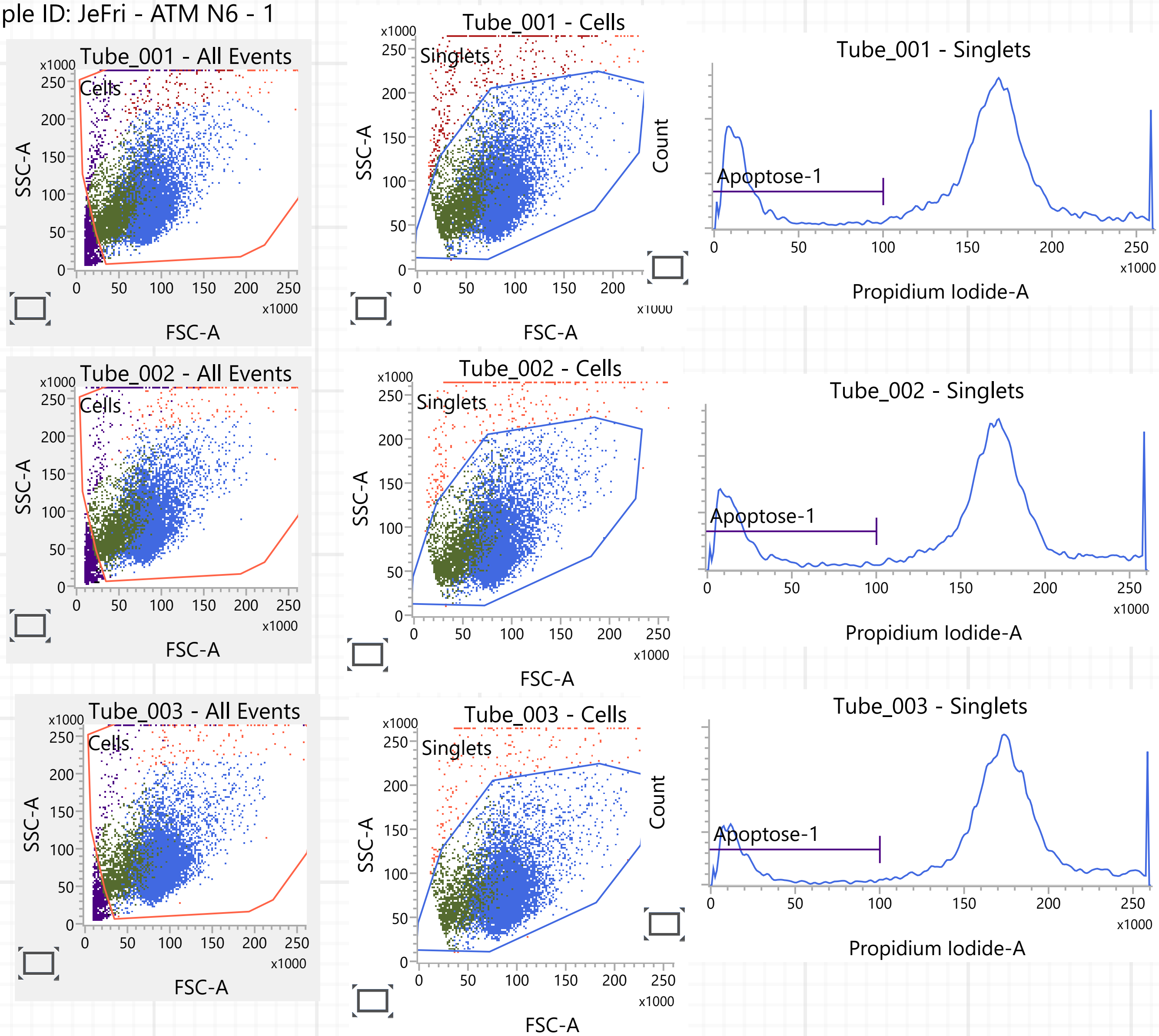

Statistics

Prozent apoptotische Zellen: Prozent apoptotische Zellen No Value % von Cells

Name	Events	% Total	% Parent	% Grandparent
Tube_001:All Events	11,095	100.00	***	***
Tube_001:Cells	10,000	90.13	90.13	***
Tube_001:Singlets	9,534	85.93	95.34	85.93
Tube_002:All Events	10,000	100.00	***	***
Tube_002:Cells	9,170	91.70	91.70	***
Tube_002:Singlets	8,839	88.39	96.39	88.39
Tube_003:All Events	10,000	100.00	***	***
Tube_003:Cells	9,304	93.04	93.04	***
Tube_003:Singlets	9,062	90.62	97.40	90.62
Tube_001:Apoptose-1	3,515	31.68	31.68	***
Tube_002:Apoptose-1	2,791	27.91	27.91	***
Tube_003:Apoptose-1	2,153	21.53	21.53	***

DNA Fragmentierung

Sample ID: JeFri - ATM N6 - 2,5

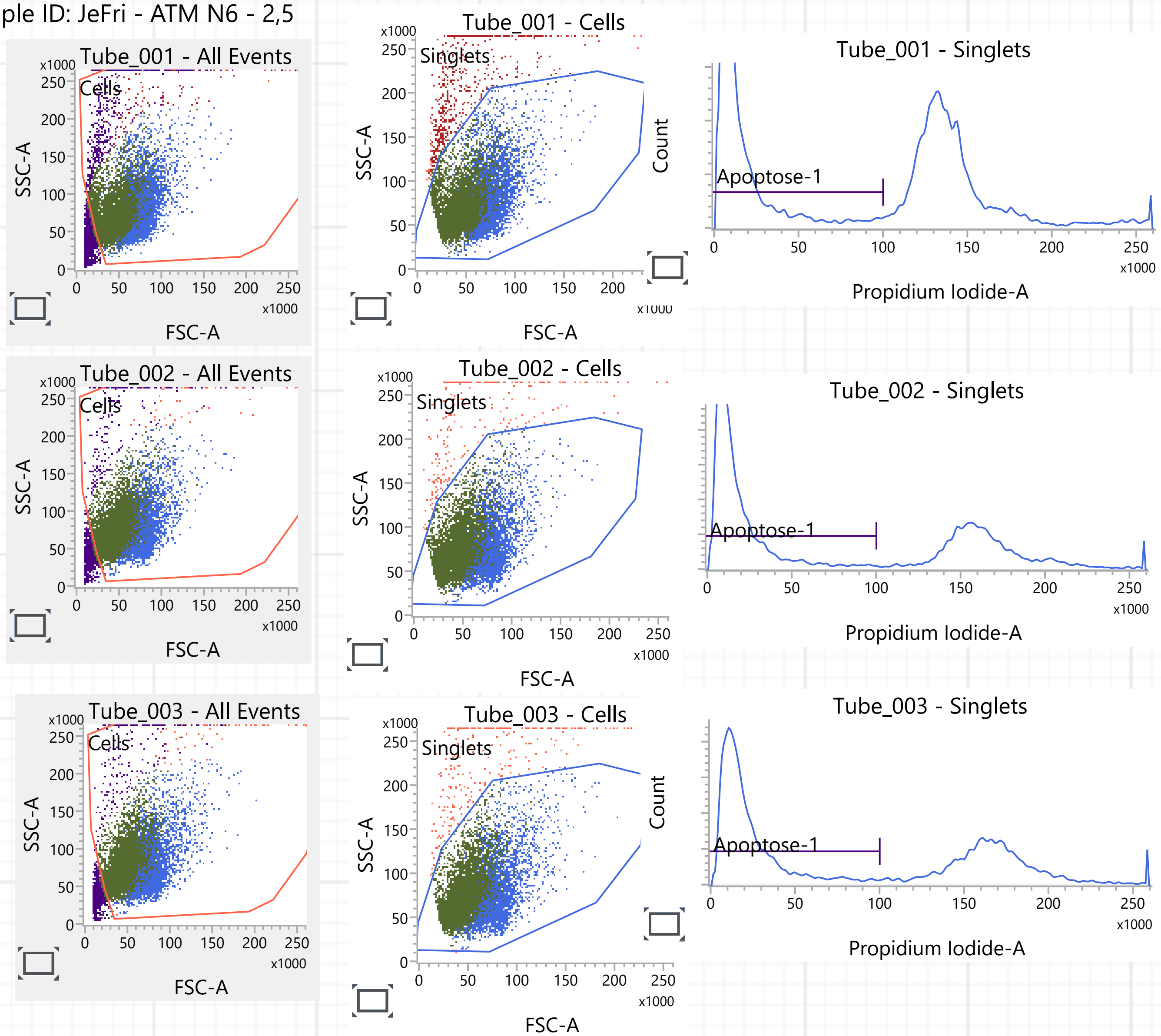

Statistics

Prozent apoptotische Zellen: Prozent apoptotische Zellen No Value % von Cells

Name	Events	% Total	% Parent	% Grandparent
Tube_001:All Events	11,579	100.00	***	***
Tube_001:Cells	10,000	86.36	86.36	***
Tube_001:Singlets	9,386	81.06	93.86	81.06
Tube_002:All Events	10,000	100.00	***	***
Tube_002:Cells	8,913	89.13	89.13	***
Tube_002:Singlets	8,655	86.55	97.11	86.55
Tube_003:All Events	10,000	100.00	***	***
Tube_003:Cells	9,078	90.78	90.78	***
Tube_003:Singlets	8,817	88.17	97.12	88.17
Tube_001:Apoptose-1	6,525	56.35	56.35	***
Tube_002:Apoptose-1	6,349	63.49	63.49	***
Tube_003:Apoptose-1	6,005	60.05	60.05	***

DNA Fragmentierung

Sample ID: JeFri - ATM N6 - 5

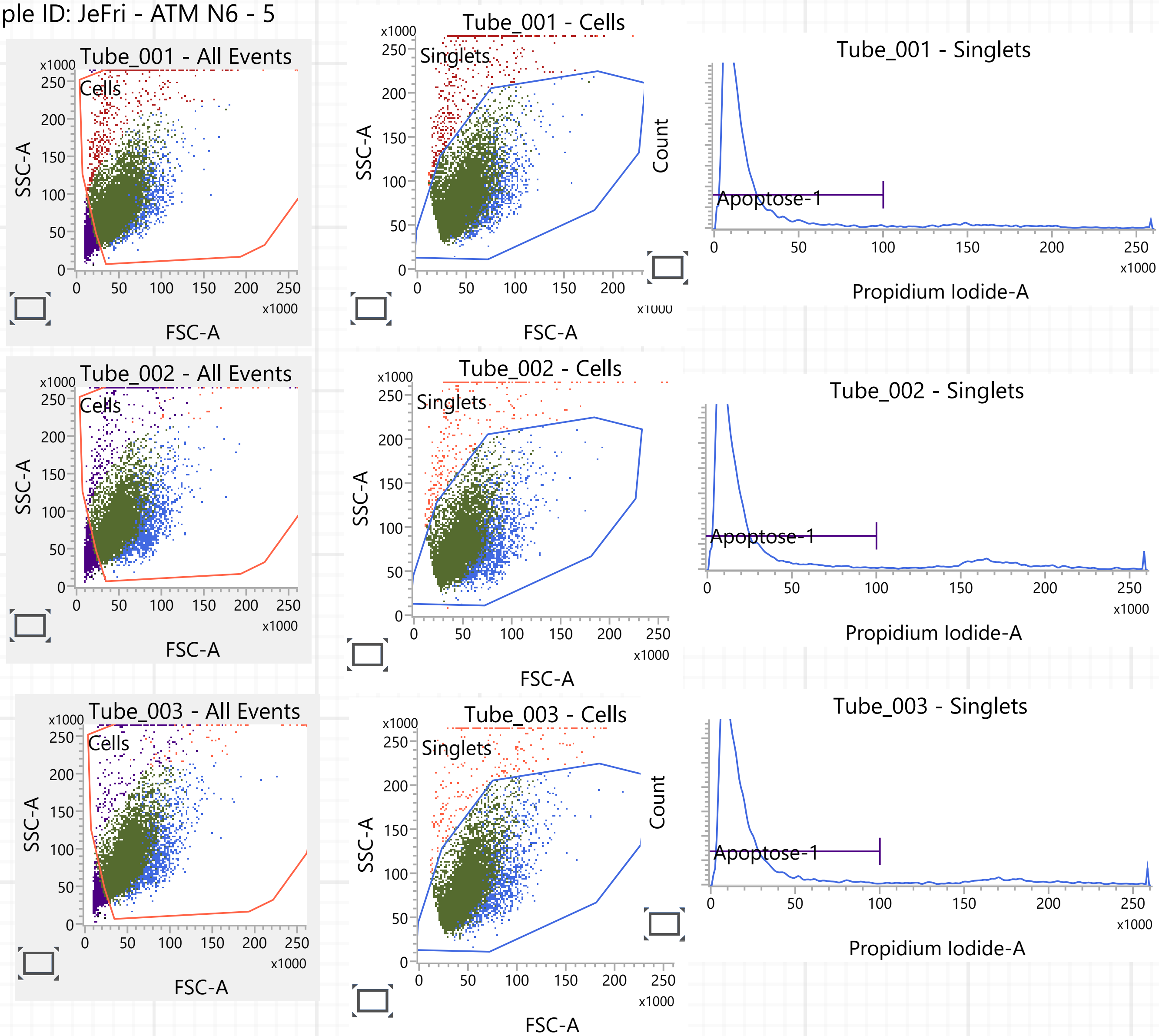

Statistics

Prozent apoptotische Zellen: Prozent apoptotische Zellen No Value % von Cells

Name	Events	% Total	% Parent	% Grandparent
Tube_001:All Events	11,908	100.00	***	***
Tube_001:Cells	10,000	83.98	83.98	***
Tube_001:Singlets	9,529	80.02	95.29	80.02
Tube_002:All Events	10,000	100.00	***	***
Tube_002:Cells	8,748	87.48	87.48	***
Tube_002:Singlets	8,421	84.21	96.26	84.21
Tube_003:All Events	10,000	100.00	***	***
Tube_003:Cells	8,857	88.57	88.57	***
Tube_003:Singlets	8,586	85.86	96.94	85.86
Tube_001:Apoptose-1	10,690	89.77	89.77	***
Tube_002:Apoptose-1	8,357	83.57	83.57	***
Tube_003:Apoptose-1	8,488	84.88	84.88	***

DNA Fragmentierung

Sample ID: JeFri - ATM N6 - DMSO

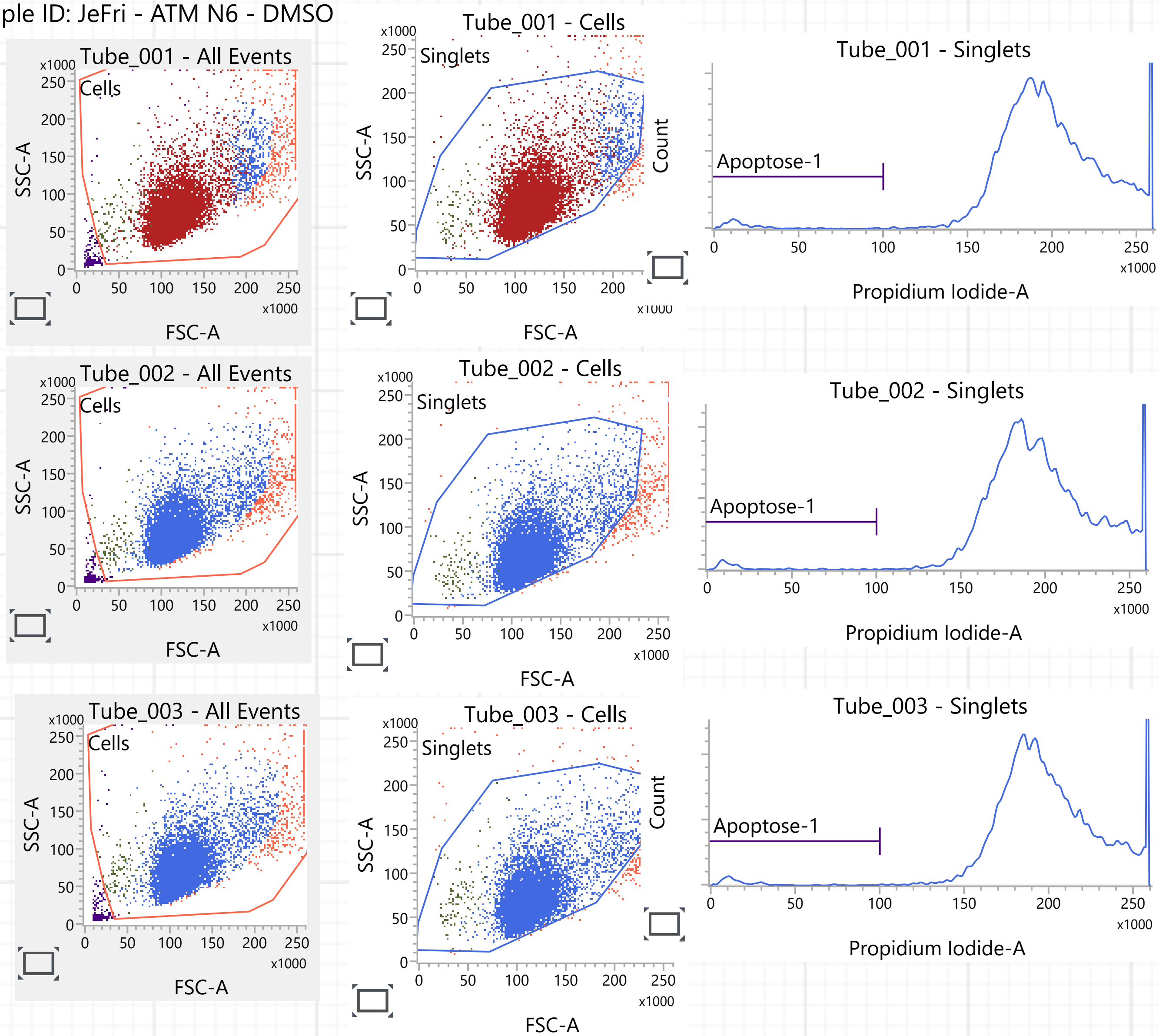

Statistics

Prozent apoptotische Zellen: Prozent apoptotische Zellen No Value % von Cells

Name	Events	% Total	% Parent	% Grandparent
Tube_001:All Events	10,185	100.00	***	***
Tube_001:Cells	10,000	98.18	98.18	***
Tube_001:Singlets	9,244	90.76	92.44	90.76
Tube_002:All Events	10,000	100.00	***	***
Tube_002:Cells	9,771	97.71	97.71	***
Tube_002:Singlets	9,099	90.99	93.12	90.99
Tube_003:All Events	10,000	100.00	***	***
Tube_003:Cells	9,768	97.68	97.68	***
Tube_003:Singlets	9,315	93.15	95.36	93.15
Tube_001:Apoptose-1	313	3.07	3.07	***
Tube_002:Apoptose-1	353	3.53	3.53	***
Tube_003:Apoptose-1	364	3.64	3.64	***

DNA Fragmentierung

Sample ID: JeFri - ATM N6 - Etoposi

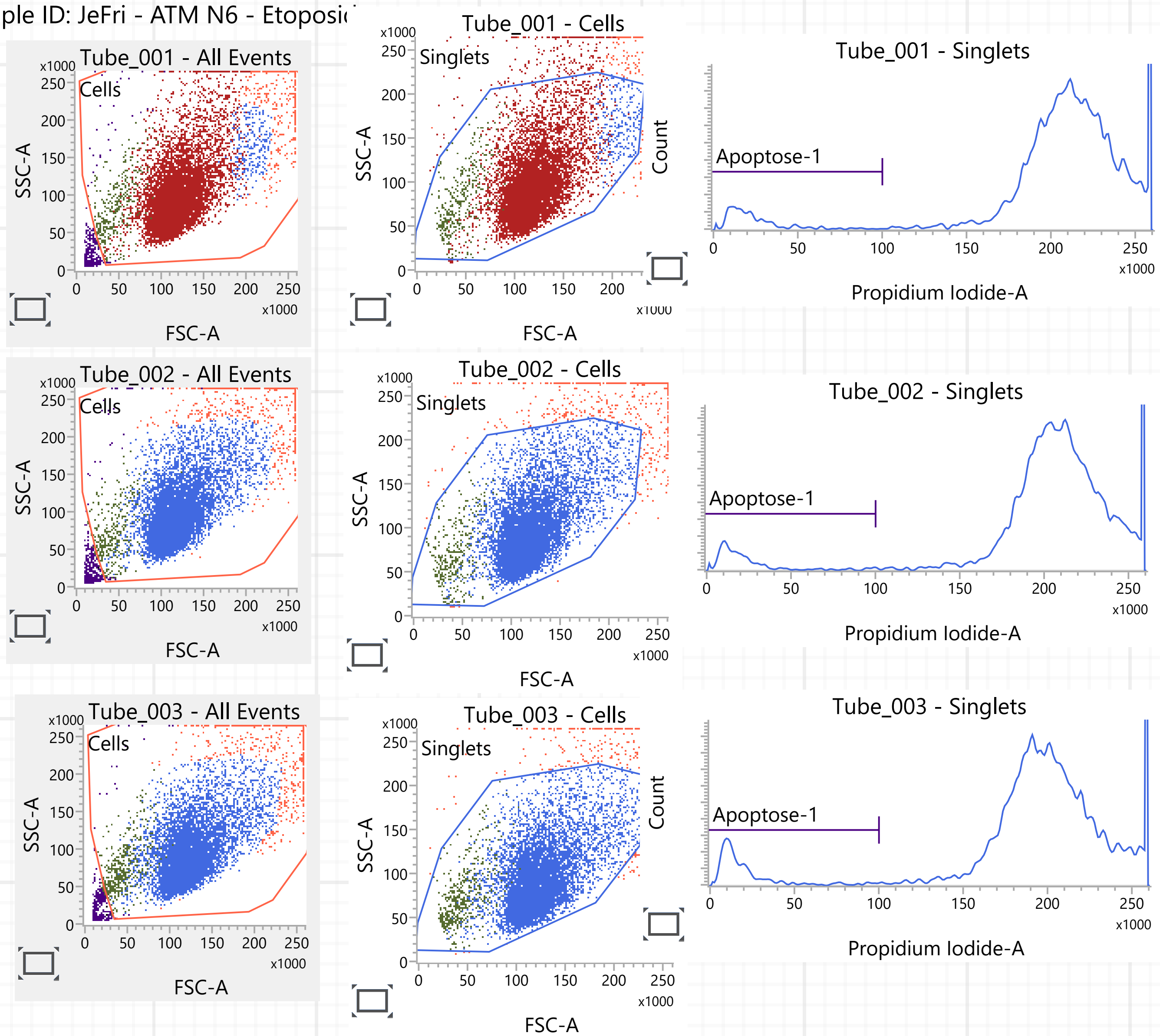

Statistics

Prozent apoptotische Zellen: Prozent apoptotische Zellen No Value % von Cells

Name	Events	% Total	% Parent	% Grandparent
Tube_001:All Events	10,321	100.00	***	***
Tube_001:Cells	10,000	96.89	96.89	***
Tube_001:Singlets	9,306	90.17	93.06	90.17
Tube_002:All Events	10,000	100.00	***	***
Tube_002:Cells	9,645	96.45	96.45	***
Tube_002:Singlets	9,014	90.14	93.46	90.14
Tube_003:All Events	10,000	100.00	***	***
Tube_003:Cells	9,683	96.83	96.83	***
Tube_003:Singlets	9,053	90.53	93.49	90.53
Tube_001:Apoptose-1	710	6.88	6.88	***
Tube_002:Apoptose-1	738	7.38	7.38	***
Tube_003:Apoptose-1	825	8.25	8.25	***

DNA Fragmentierung

Sample ID: JeFri - ATM N6 - KO

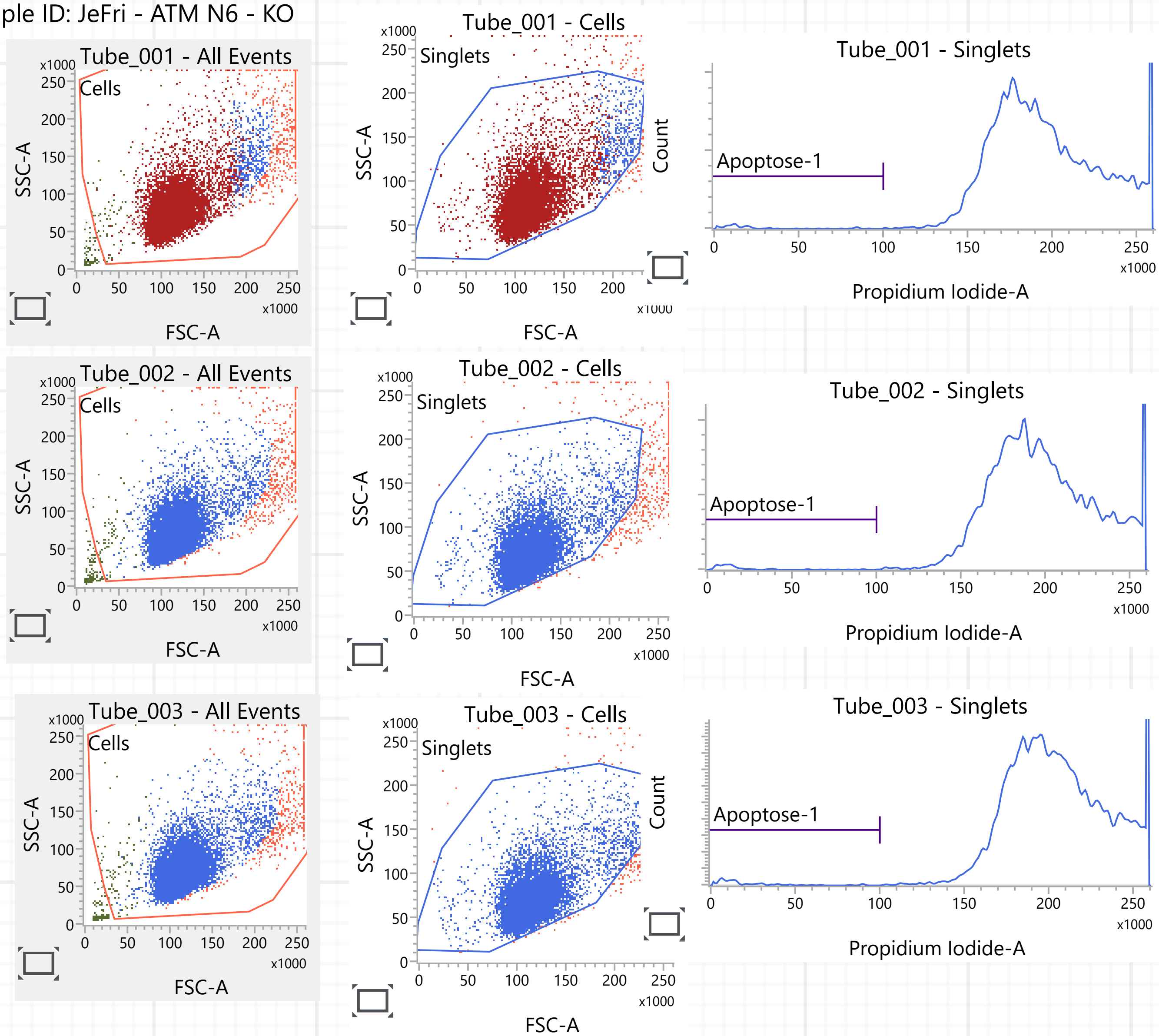

Statistics

Prozent apoptotische Zellen: Prozent apoptotische Zellen No Value % von Cells

Name	Events	% Total	% Parent	% Grandparent
Tube_001:All Events	10,061	100.00	***	***
Tube_001:Cells	10,000	99.39	99.39	***
Tube_001:Singlets	9,231	91.75	92.31	91.75
Tube_002:All Events	10,000	100.00	***	***
Tube_002:Cells	9,888	98.88	98.88	***
Tube_002:Singlets	9,212	92.12	93.16	92.12
Tube_003:All Events	10,000	100.00	***	***
Tube_003:Cells	9,883	98.83	98.83	***
Tube_003:Singlets	9,364	93.64	94.75	93.64
Tube_001:Apoptose-1	126	1.25	1.25	***
Tube_002:Apoptose-1	185	1.85	1.85	***
Tube_003:Apoptose-1	208	2.08	2.08	***

DNA Fragmentierung

Sample ID: J DMSO

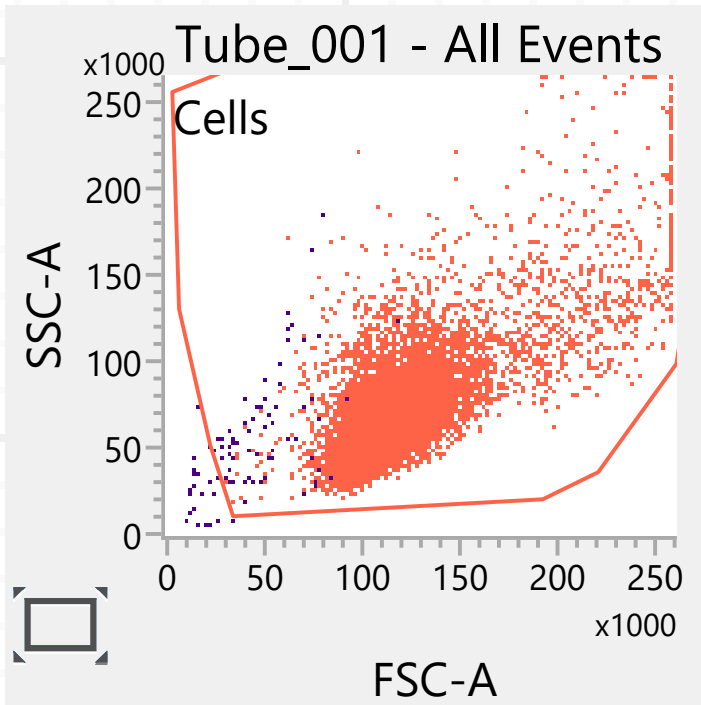

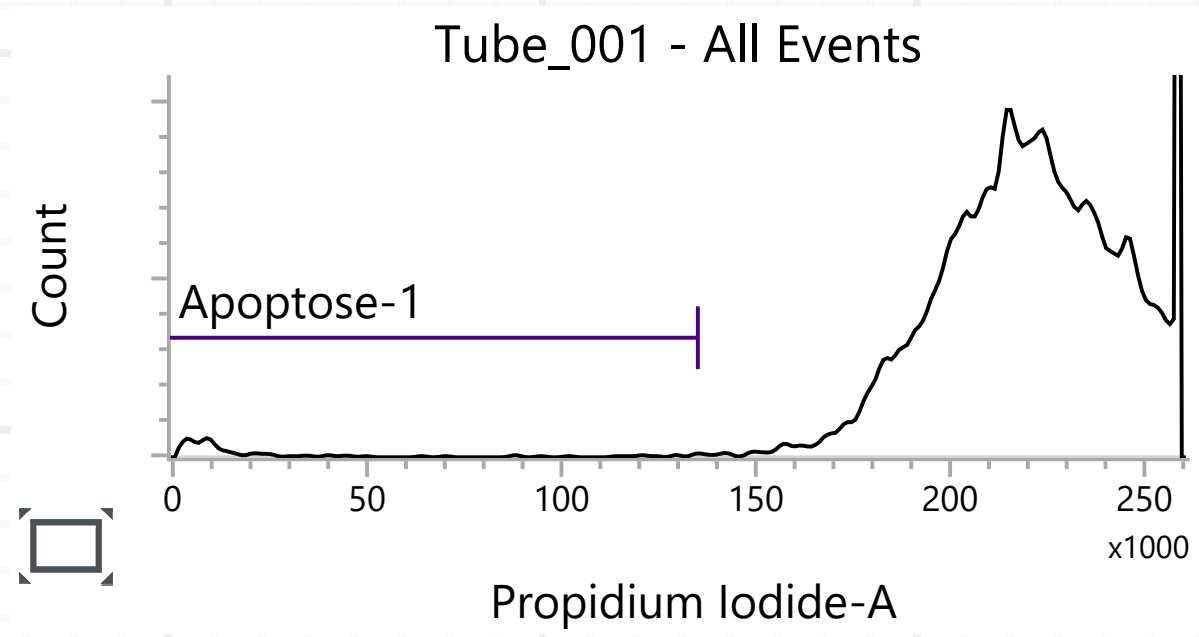

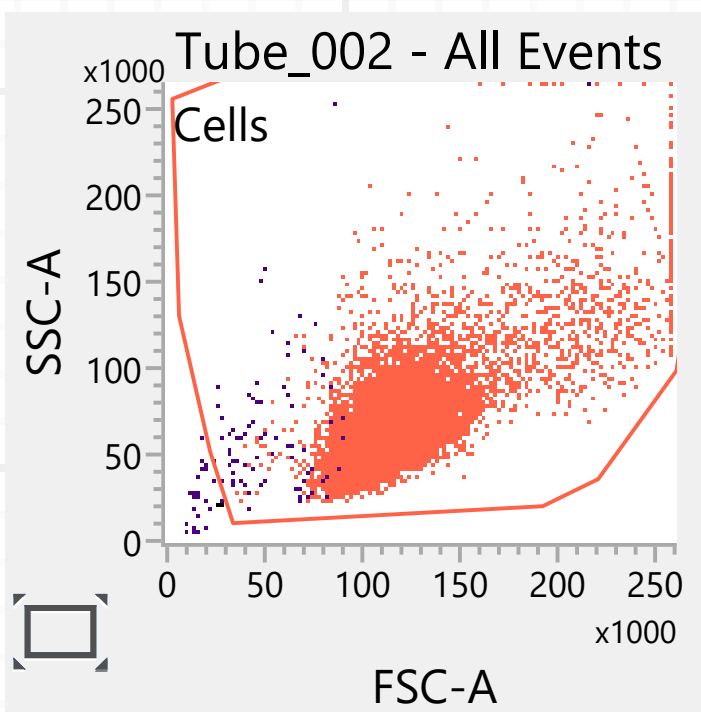

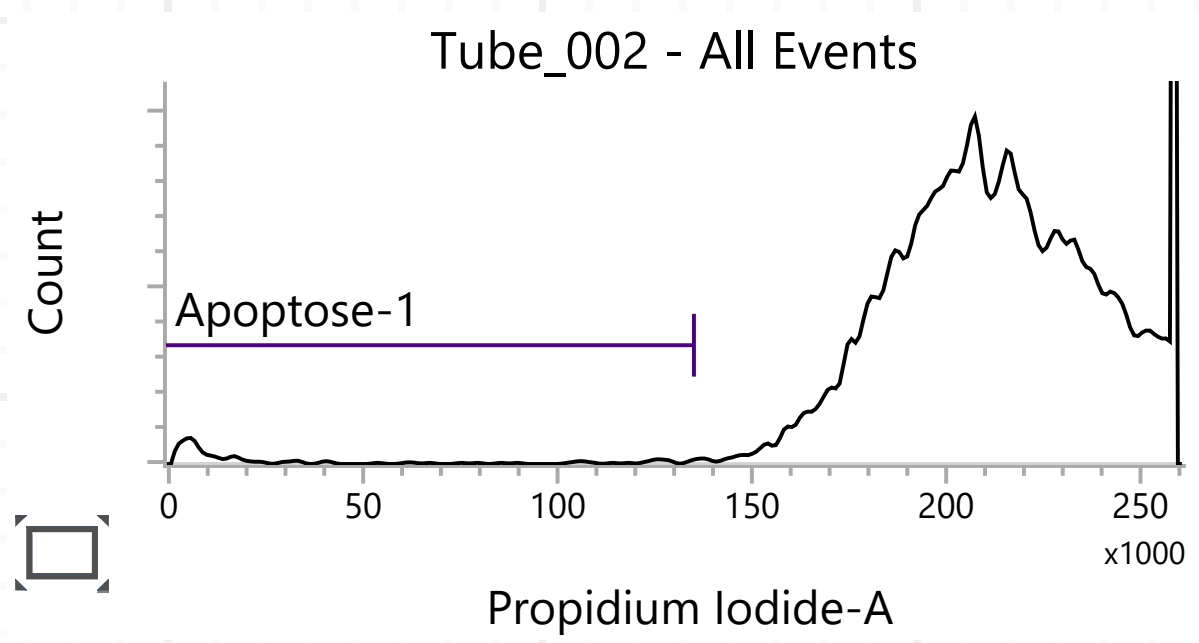

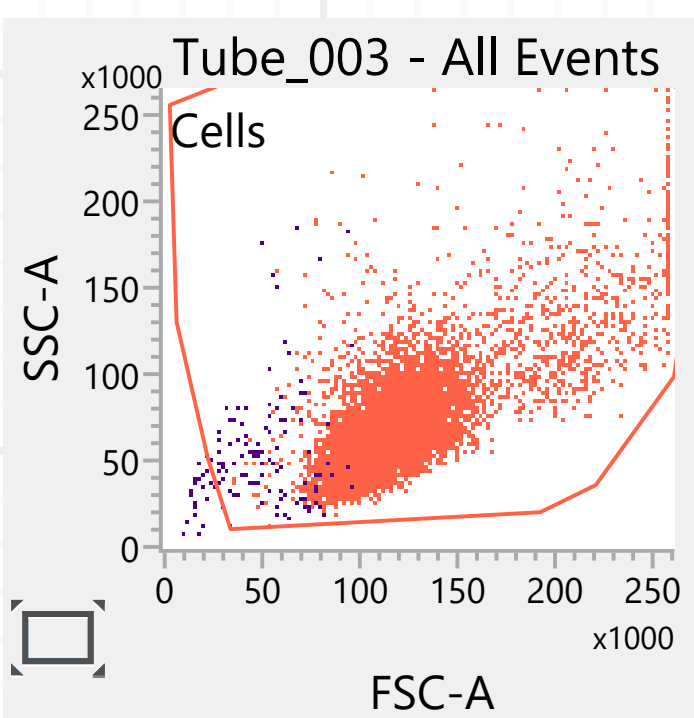

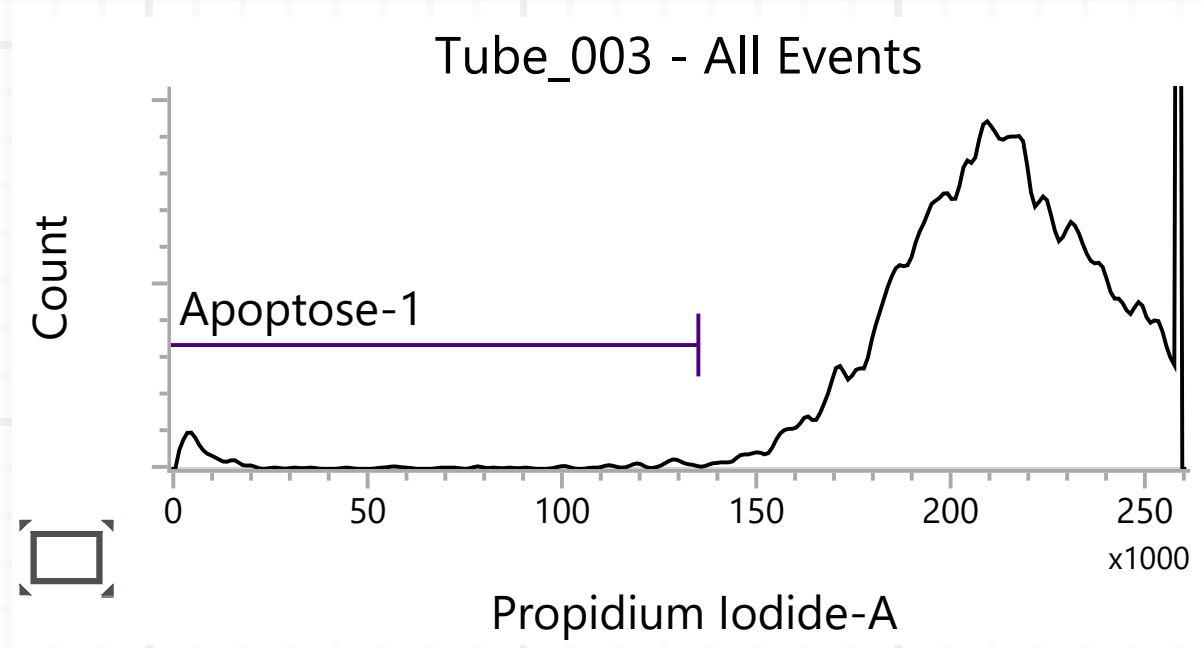

Statistics

Prozent apoptotische Zellen: Prozent apoptotische Zellen No Value % von Cells

Name	Events	% Total	% Parent	% Grandparent
Tube_001:All Events	10,019	100.00	***	***
Tube_001:Cells	10,000	99.81	99.81	***
Tube_002:All Events	10,000	100.00	***	***
Tube_002:Cells	9,972	99.72	99.72	***
Tube_003:All Events	10,000	100.00	***	***
Tube_003:Cells	9,981	99.81	99.81	***
Tube_001:Apoptose-1	87	0.87	0.87	***
Tube_002:Apoptose-1	105	1.05	1.05	***
Tube_003:Apoptose-1	131	1.31	1.31	***

DNA Fragmentierung

Sample ID: J EtoGry

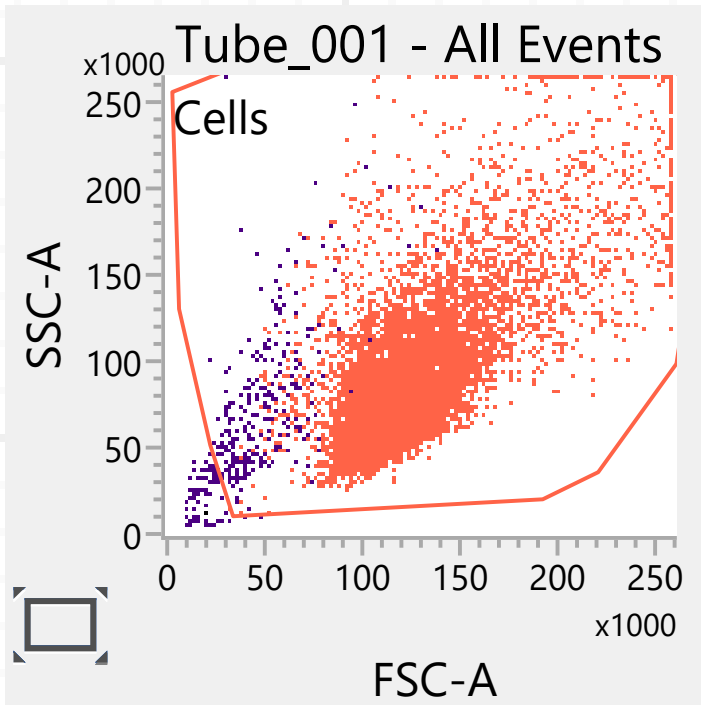

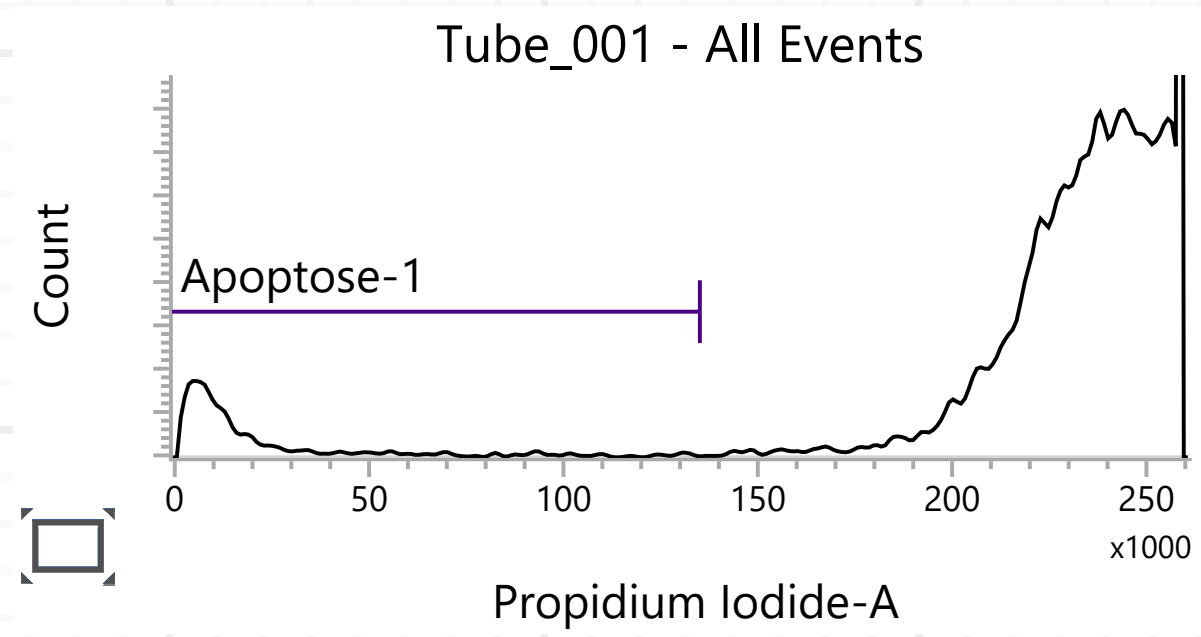

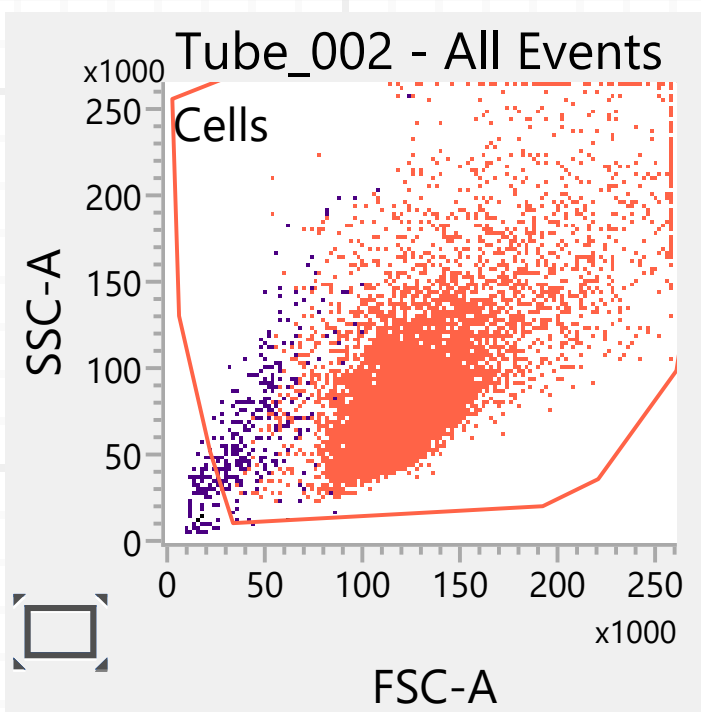

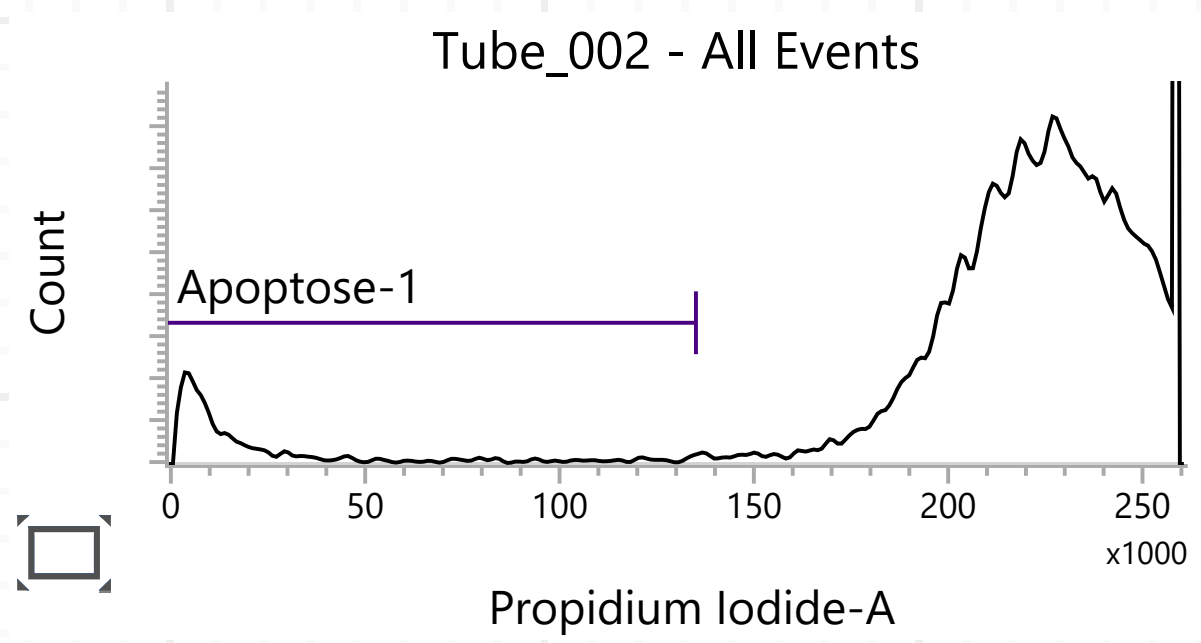

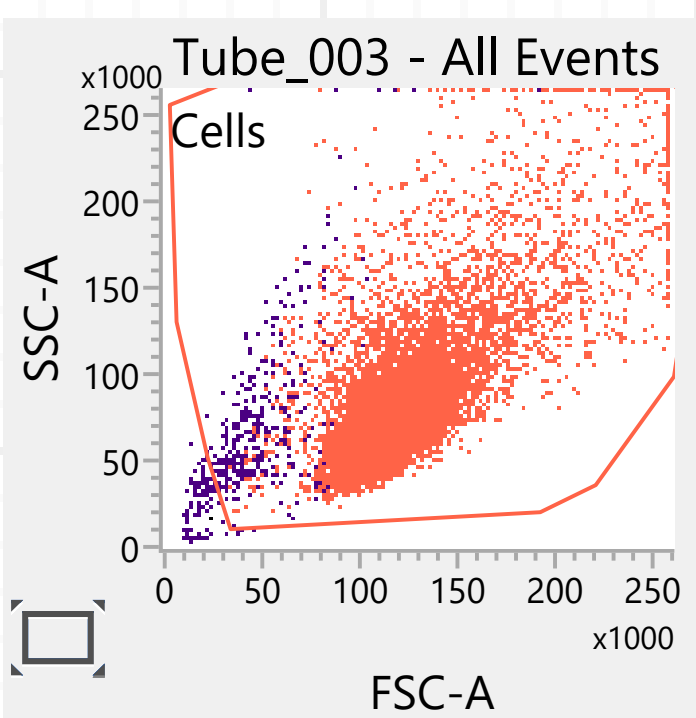

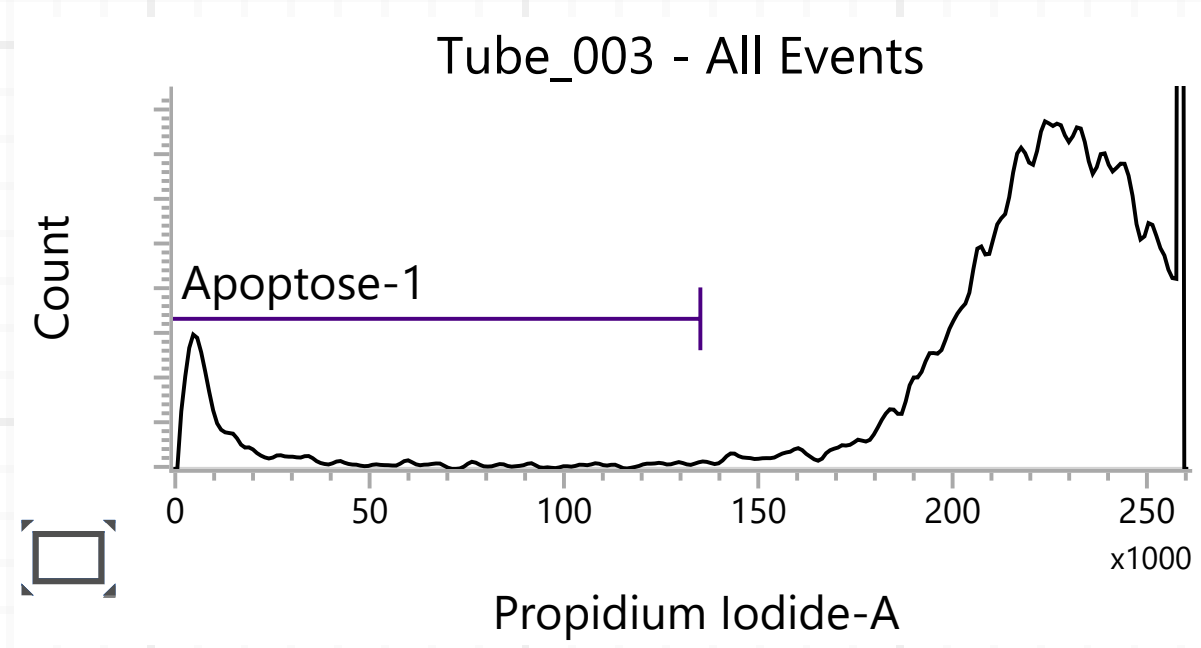

Statistics

Prozent apoptotische Zellen: Prozent apoptotische Zellen No Value % von Cells

Name	Events	% Total	% Parent	% Grandparent
Tube_001:All Events	10,070	100.00	***	***
Tube_001:Cells	10,000	99.30	99.30	***
Tube_002:All Events	10,000	100.00	***	***
Tube_002:Cells	9,922	99.22	99.22	***
Tube_003:All Events	10,000	100.00	***	***
Tube_003:Cells	9,912	99.12	99.12	***
Tube_001:Apoptose-1	319	3.17	3.17	***
Tube_002:Apoptose-1	353	3.53	3.53	***
Tube_003:Apoptose-1	409	4.09	4.09	***

DNA Fragmentierung

Sample ID: Jefri 0,1

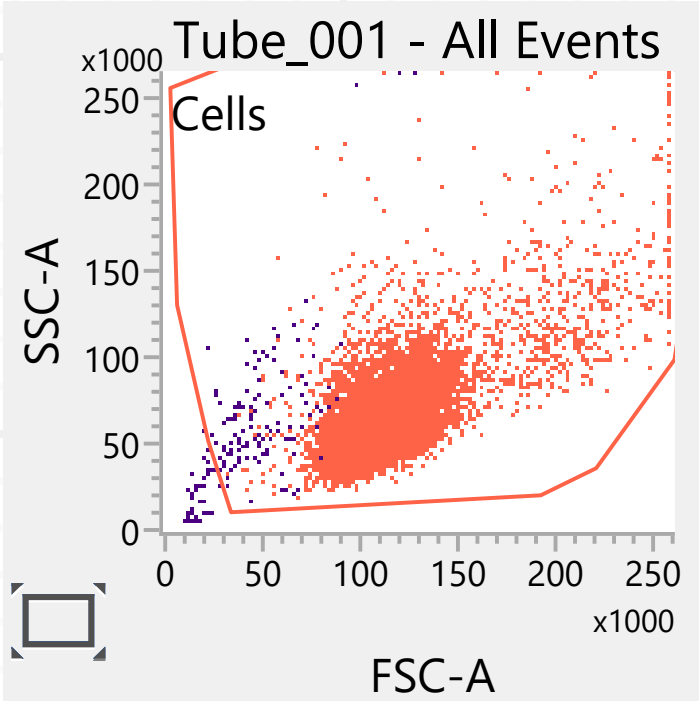

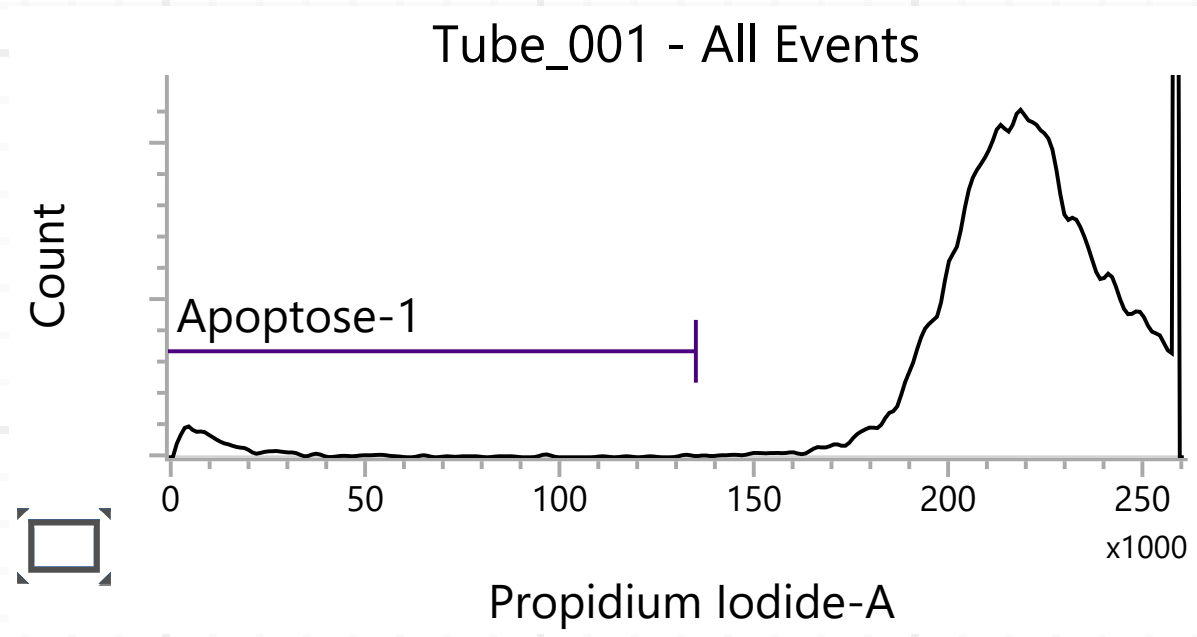

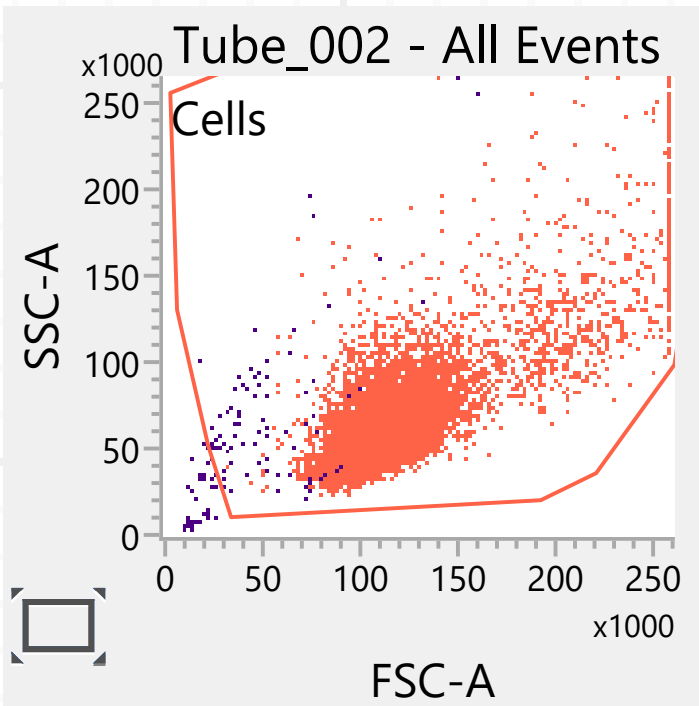

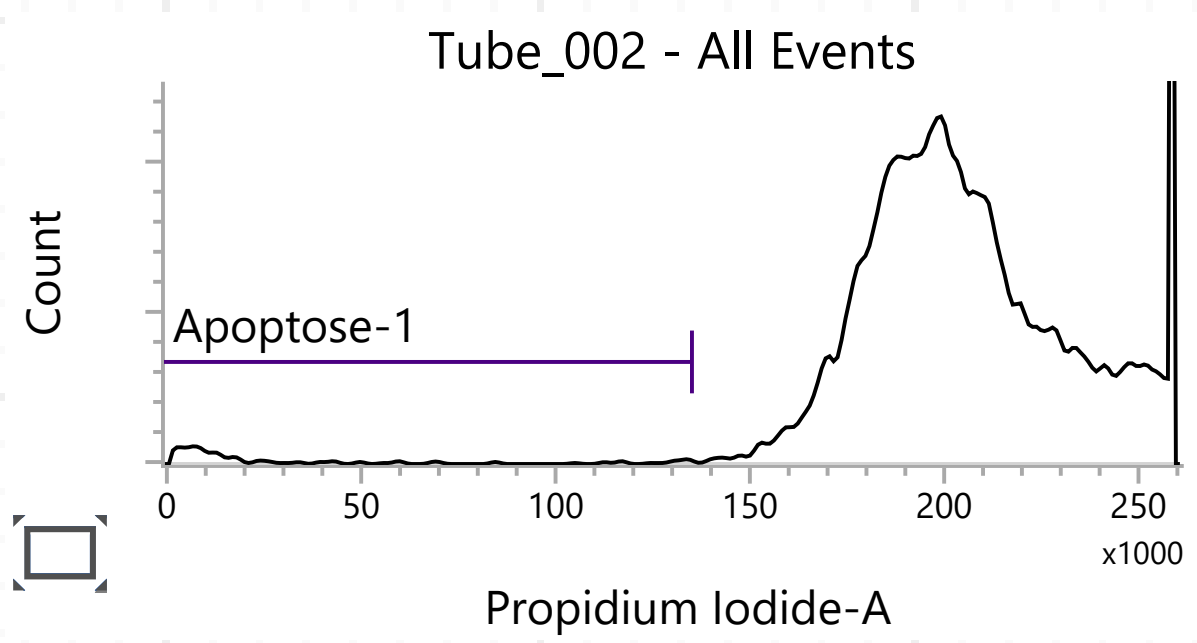

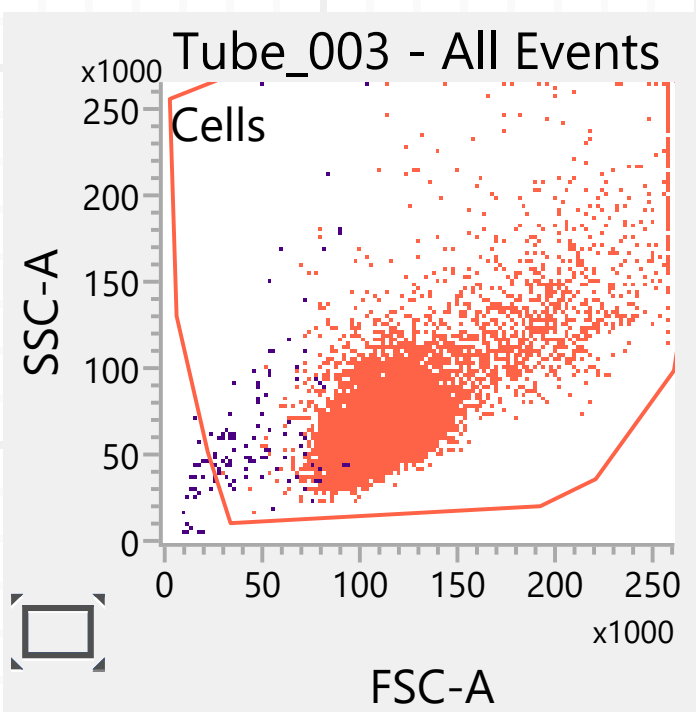

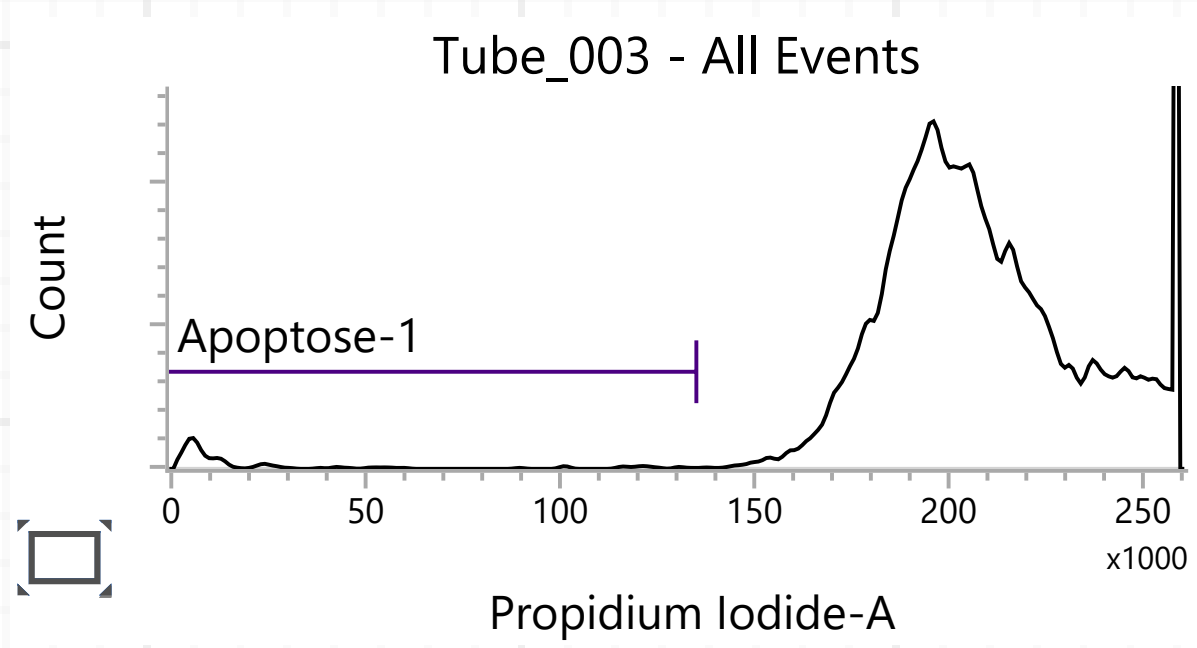

Statistics

Prozent apoptotische Zellen: Prozent apoptotische Zellen No Value % von Cells

Name	Events	% Total	% Parent	% Grandparent
Tube_001:All Events	10,040	100.00	***	***
Tube_001:Cells	10,000	99.60	99.60	***
Tube_002:All Events	10,000	100.00	***	***
Tube_002:Cells	9,964	99.64	99.64	***
Tube_003:All Events	10,000	100.00	***	***
Tube_003:Cells	9,974	99.74	99.74	***
Tube_001:Apoptose-1	167	1.66	1.66	***
Tube_002:Apoptose-1	119	1.19	1.19	***
Tube_003:Apoptose-1	119	1.19	1.19	***

DNA Fragmentierung

Sample ID: Jefri 0,5

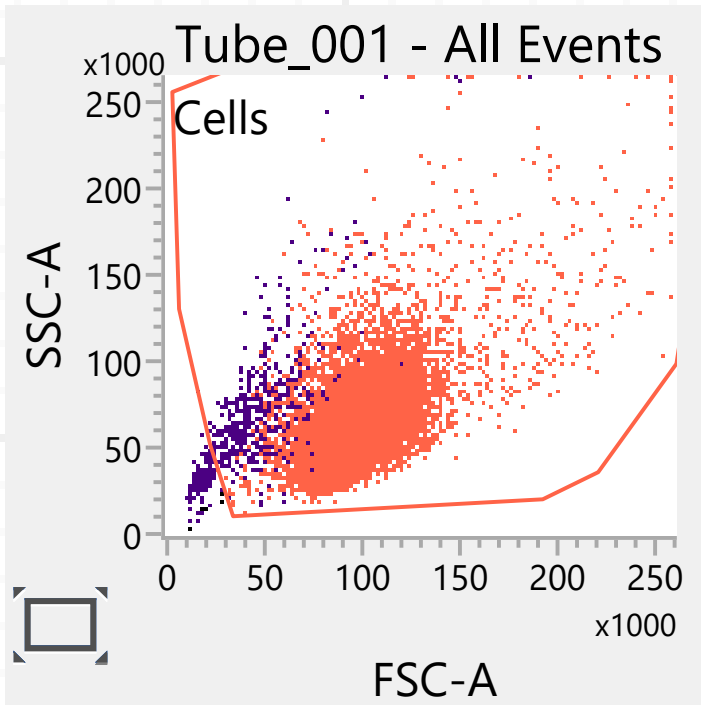

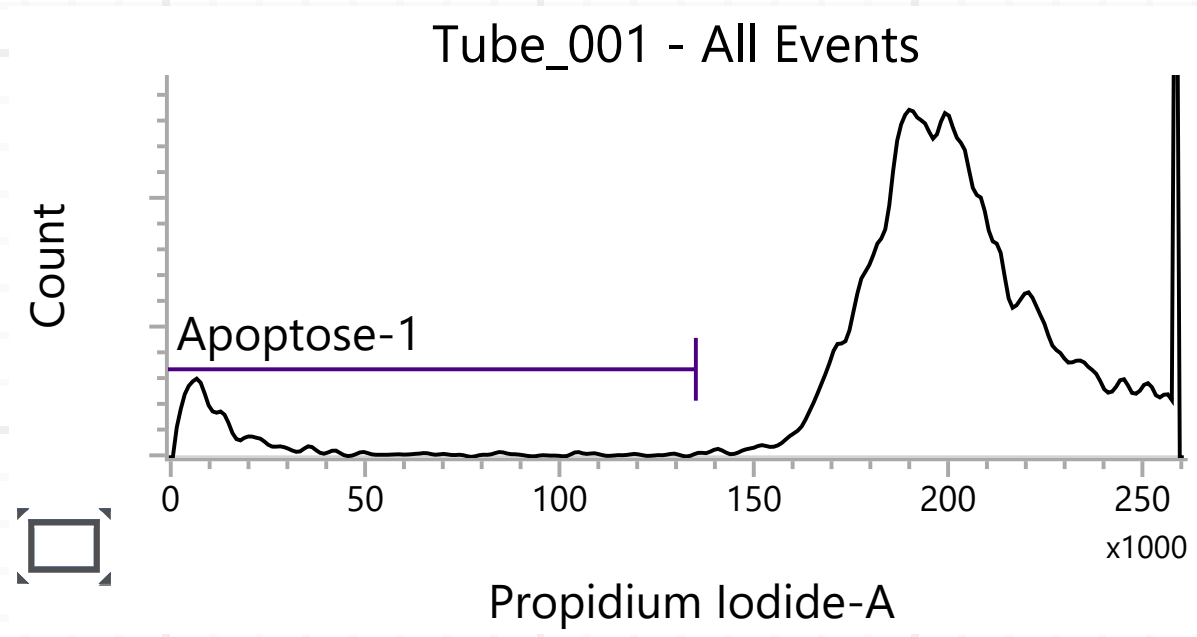

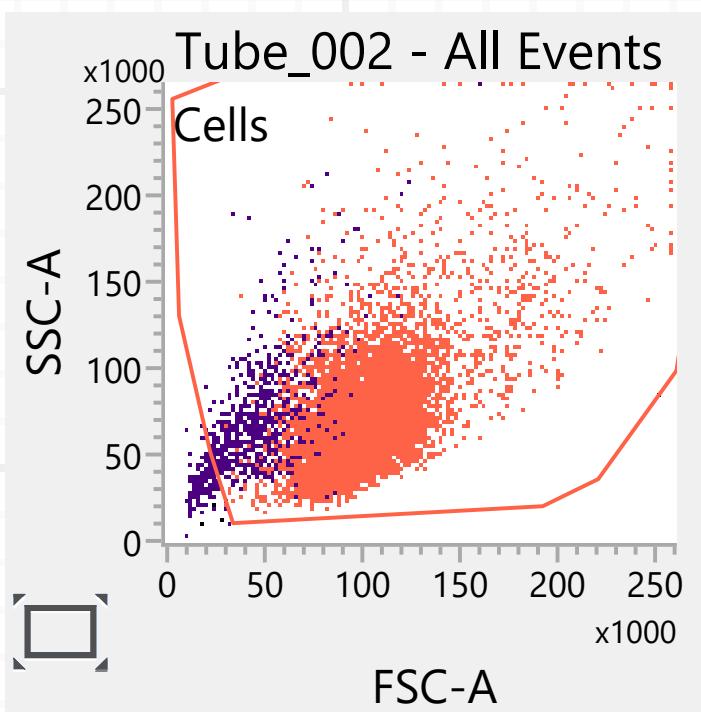

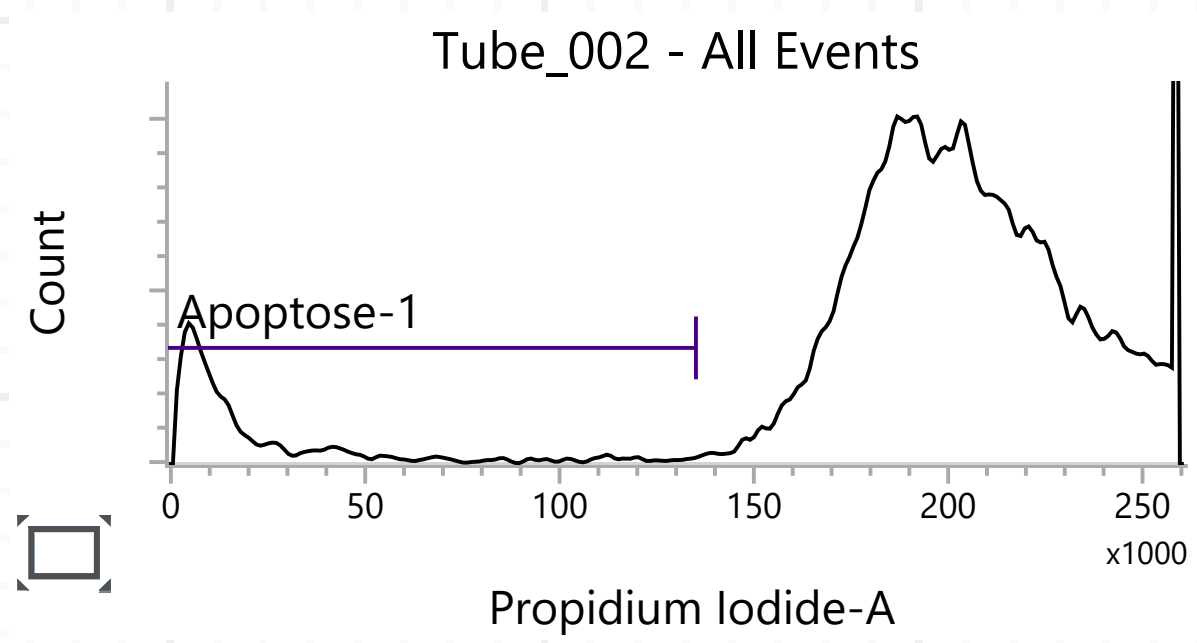

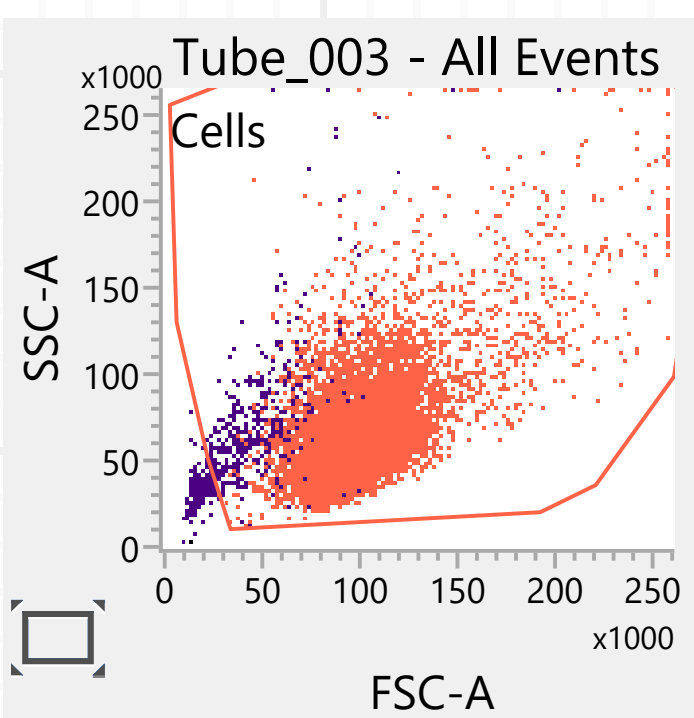

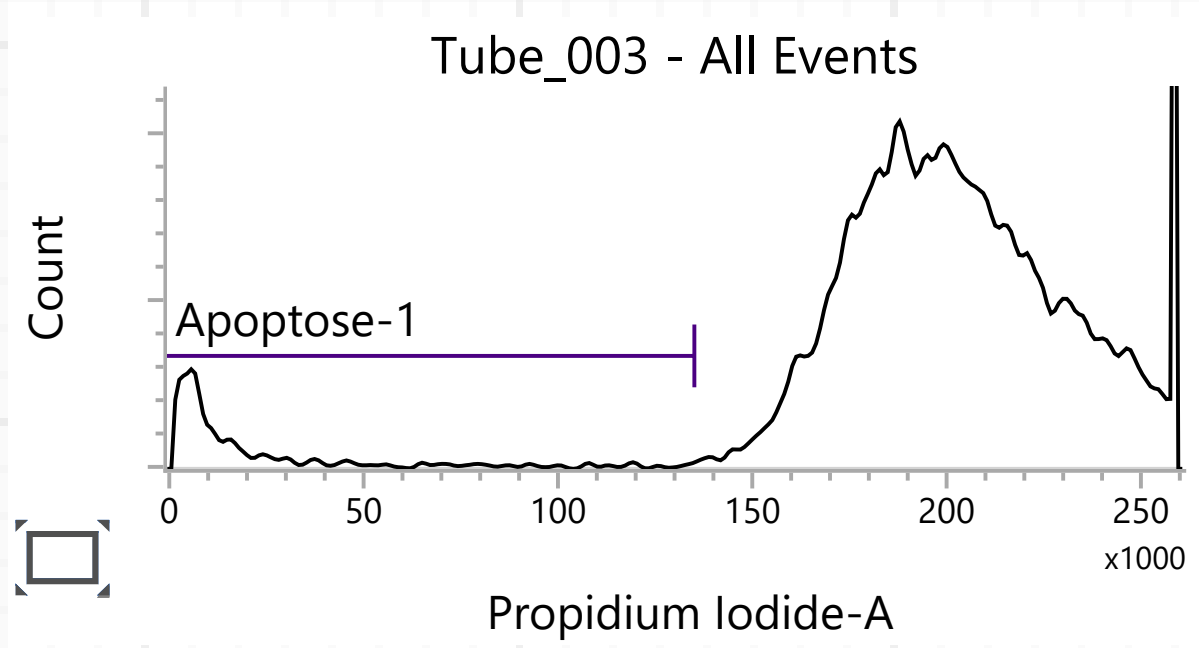

Statistics

Prozent apoptotische Zellen: Prozent apoptotische Zellen No Value % von Cells

Name	Events	% Total	% Parent	% Grandparent
Tube_001:All Events	10,104	100.00	***	***
Tube_001:Cells	10,000	98.97	98.97	***
Tube_002:All Events	10,000	100.00	***	***
Tube_002:Cells	9,865	98.65	98.65	***
Tube_003:All Events	10,000	100.00	***	***
Tube_003:Cells	9,889	98.89	98.89	***
Tube_001:Apoptose-1	530	5.25	5.25	***
Tube_002:Apoptose-1	707	7.07	7.07	***
Tube_003:Apoptose-1	462	4.62	4.62	***

DNA Fragmentierung

Sample ID: Jefri 1

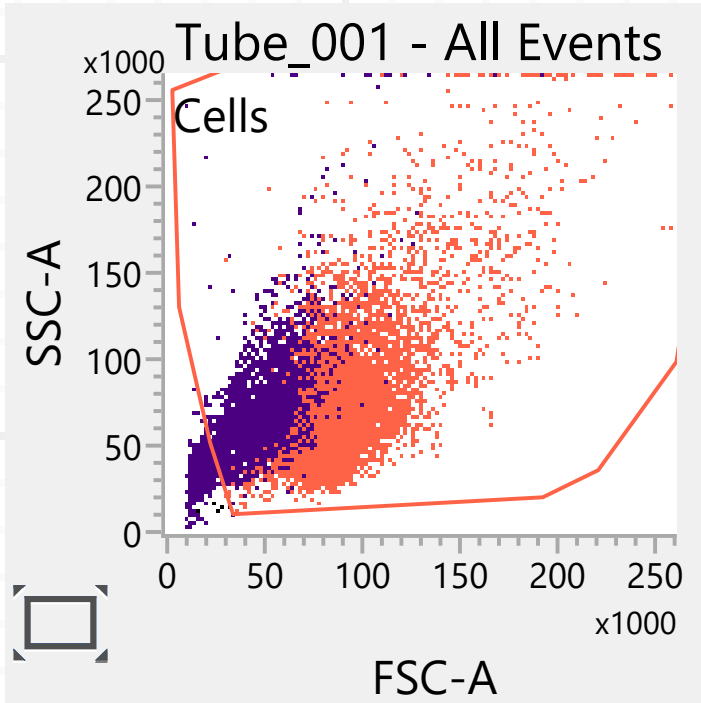

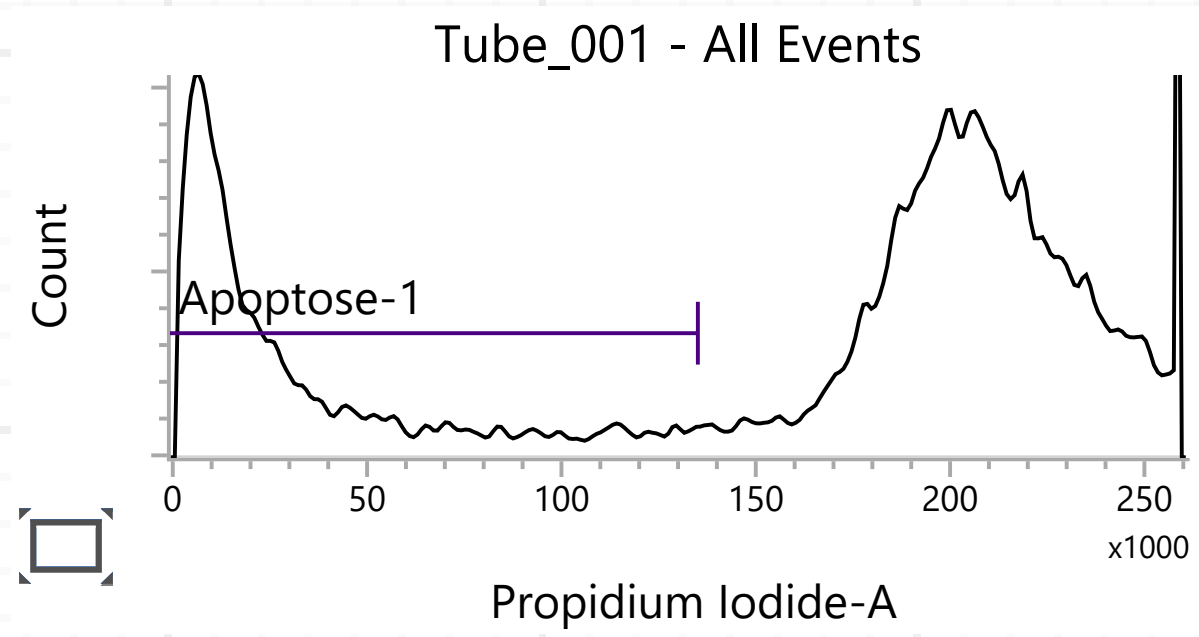

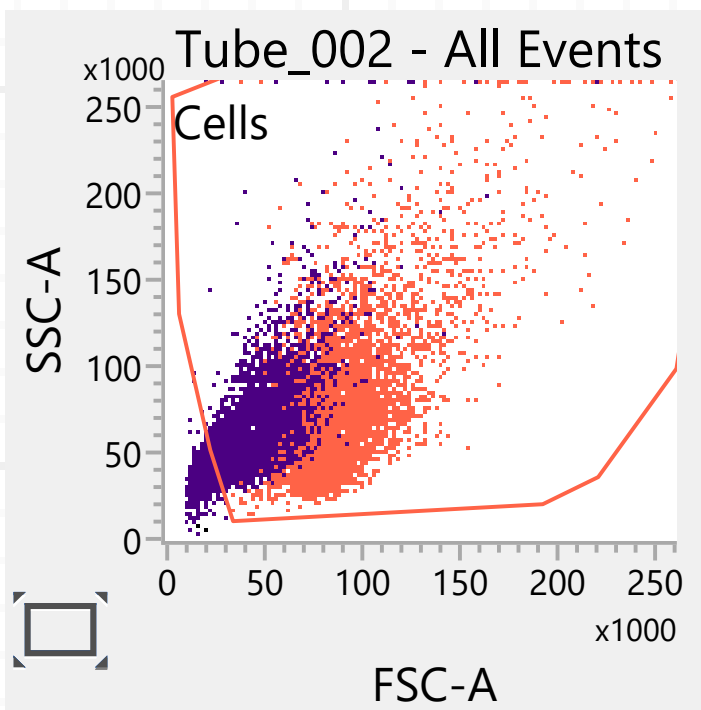

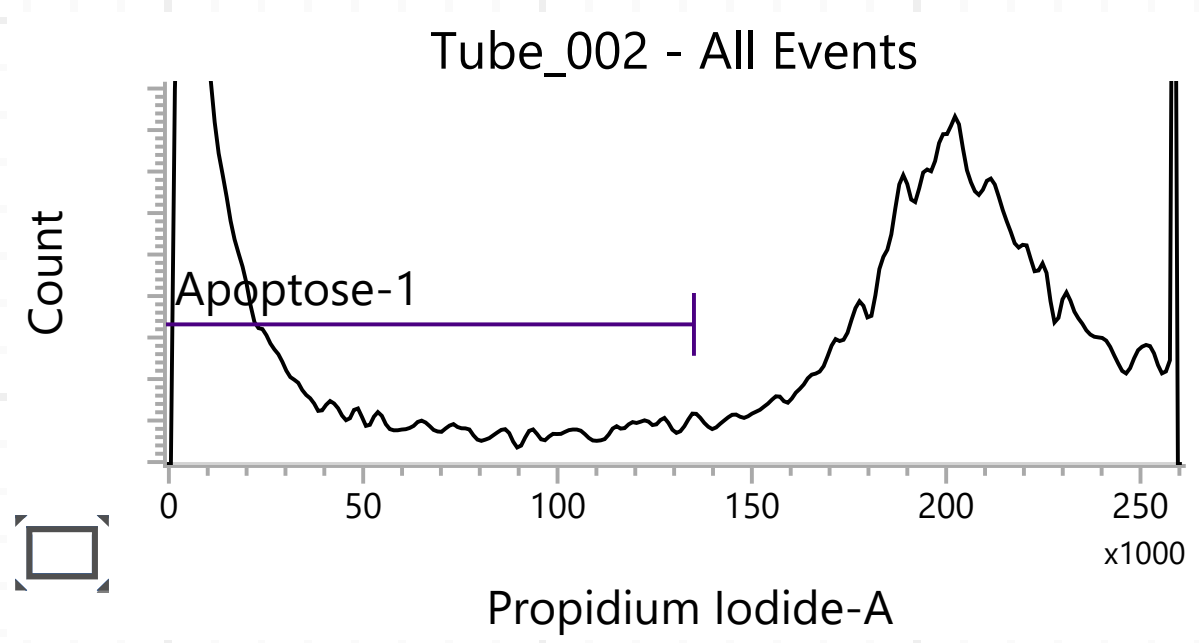

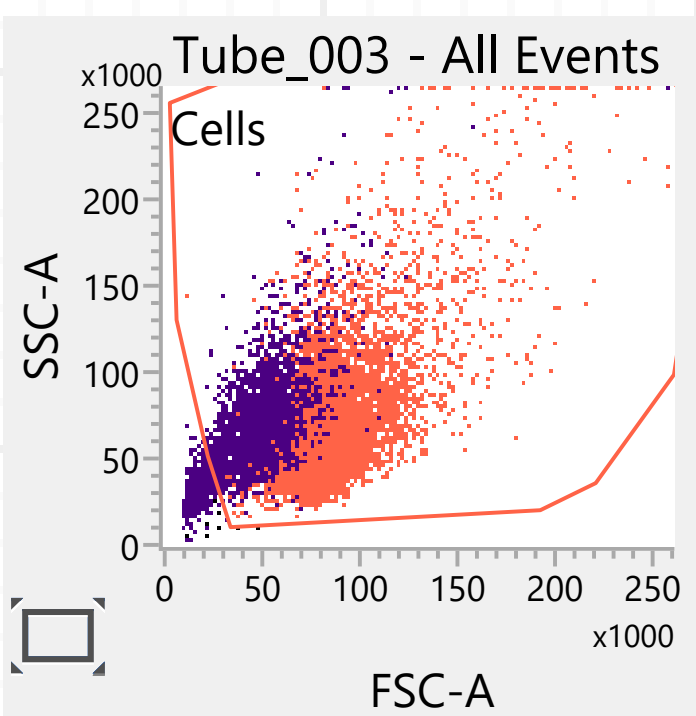

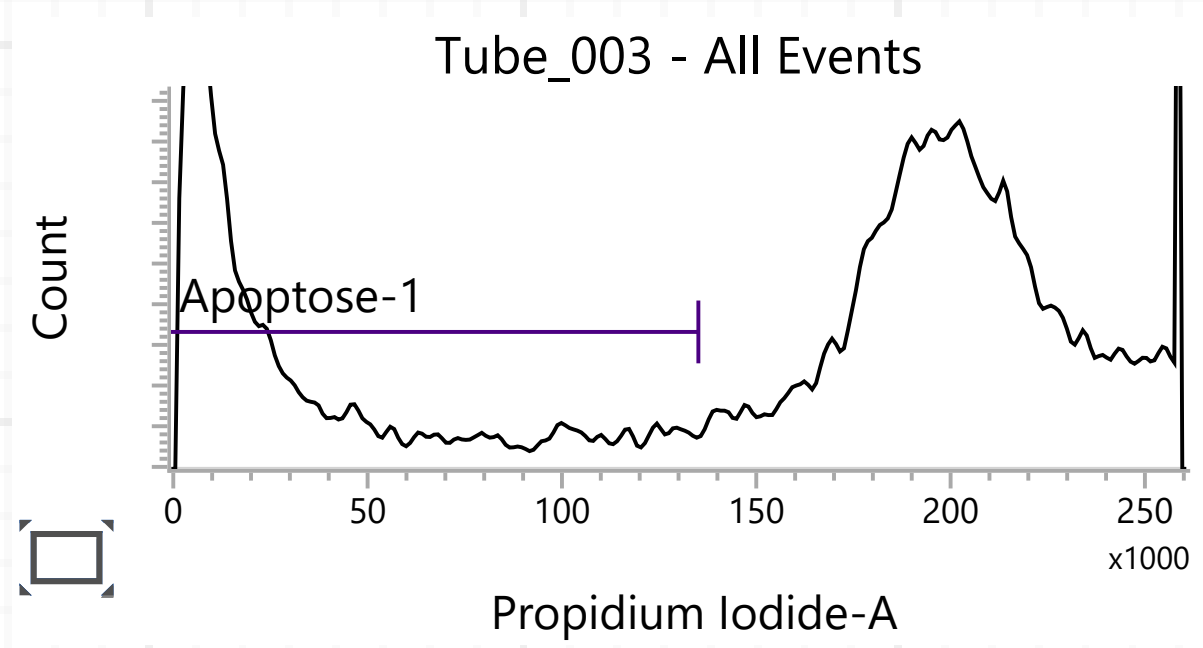

Statistics

Prozent apoptotische Zellen: Prozent apoptotische Zellen No Value % von Cells

Name	Events	% Total	% Parent	% Grandparent
Tube_001:All Events	10,466	100.00	***	***
Tube_001:Cells	10,000	95.55	95.55	***
Tube_002:All Events	10,000	100.00	***	***
Tube_002:Cells	9,409	94.09	94.09	***
Tube_003:All Events	10,000	100.00	***	***
Tube_003:Cells	9,472	94.72	94.72	***
Tube_001:Apoptose-1	2,634	25.17	25.17	***
Tube_002:Apoptose-1	3,071	30.71	30.71	***
Tube_003:Apoptose-1	2,806	28.06	28.06	***

DNA Fragmentierung

Sample ID: Jefri 2,5

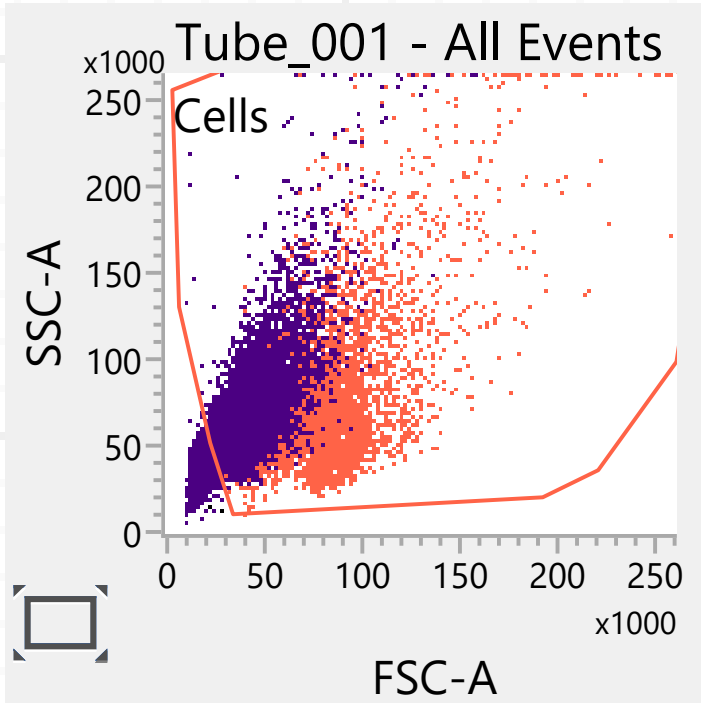

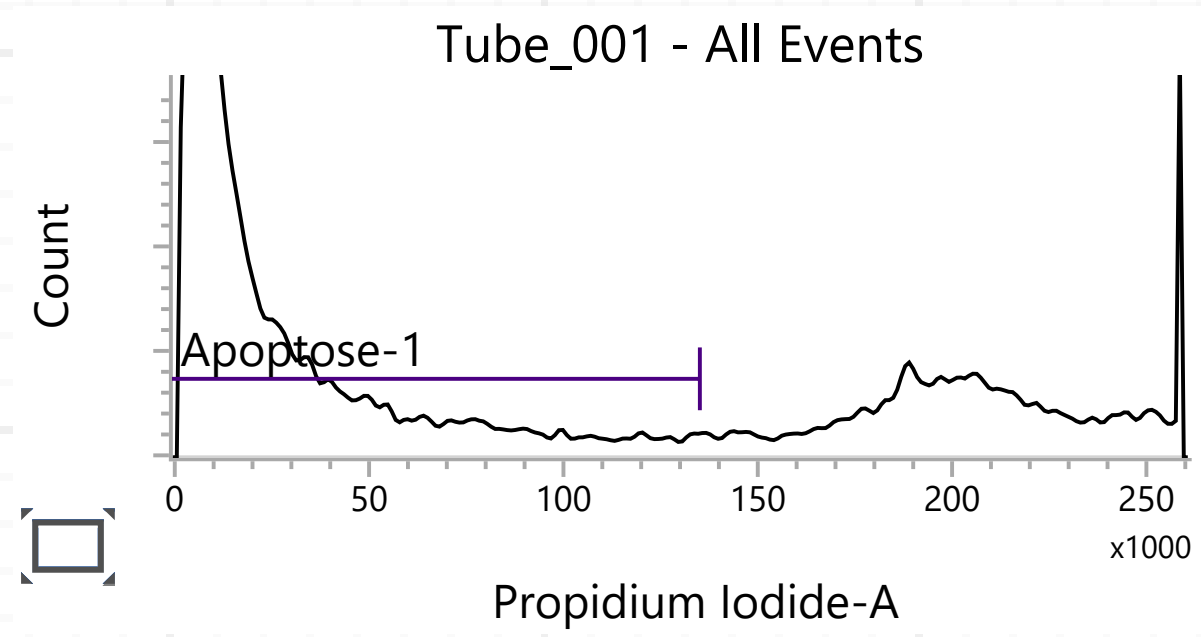

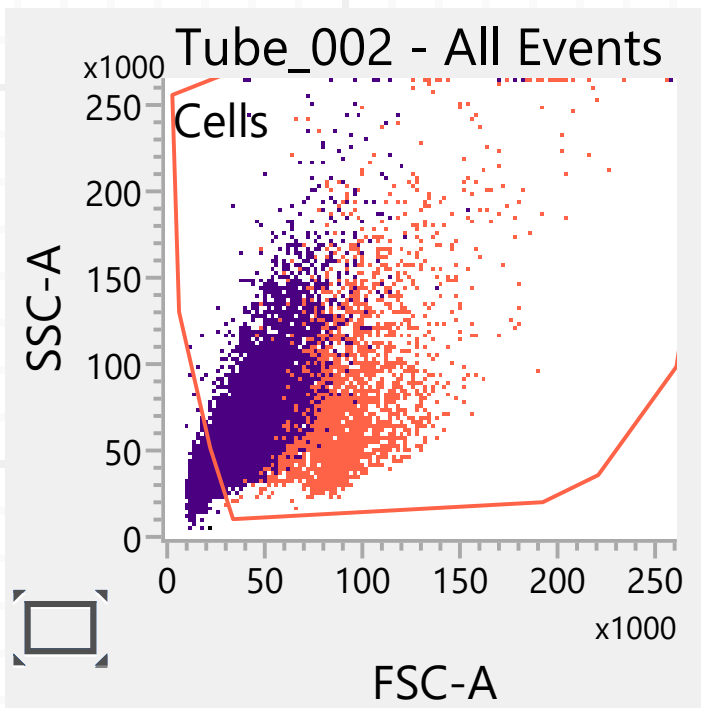

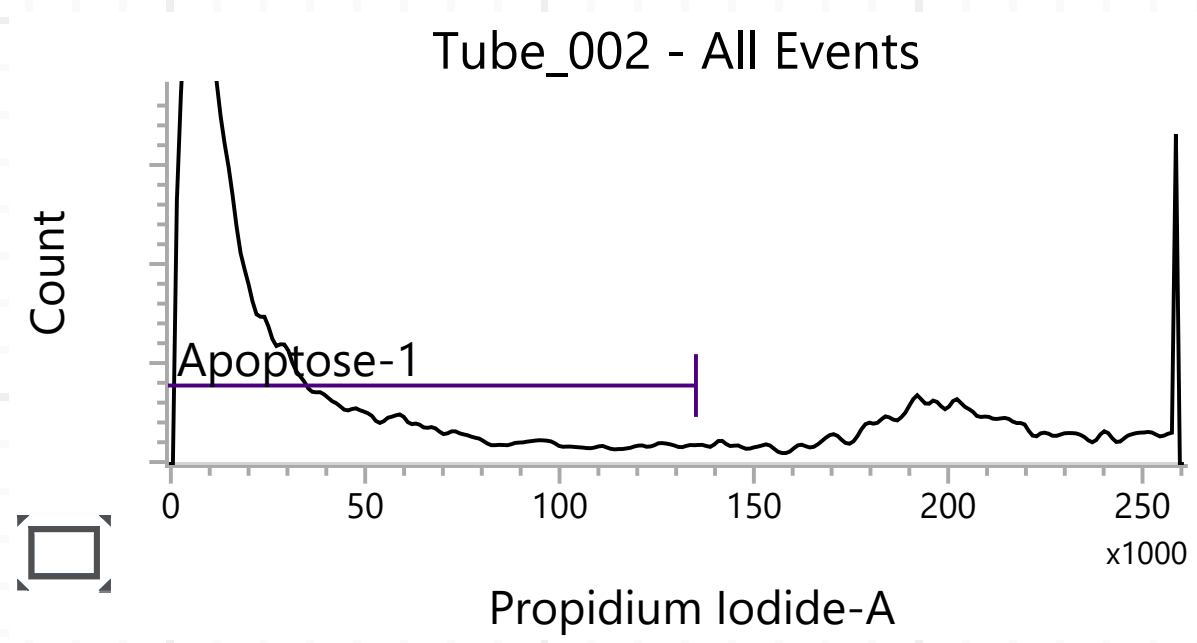

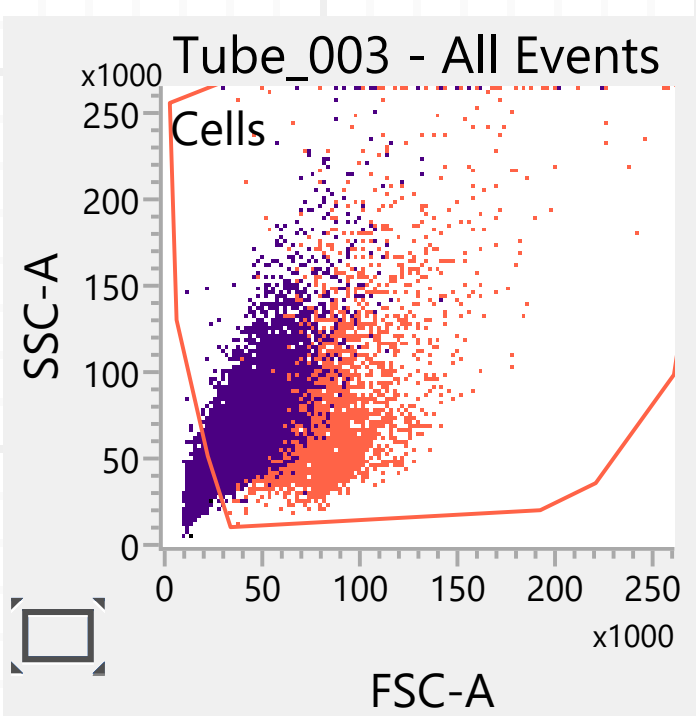

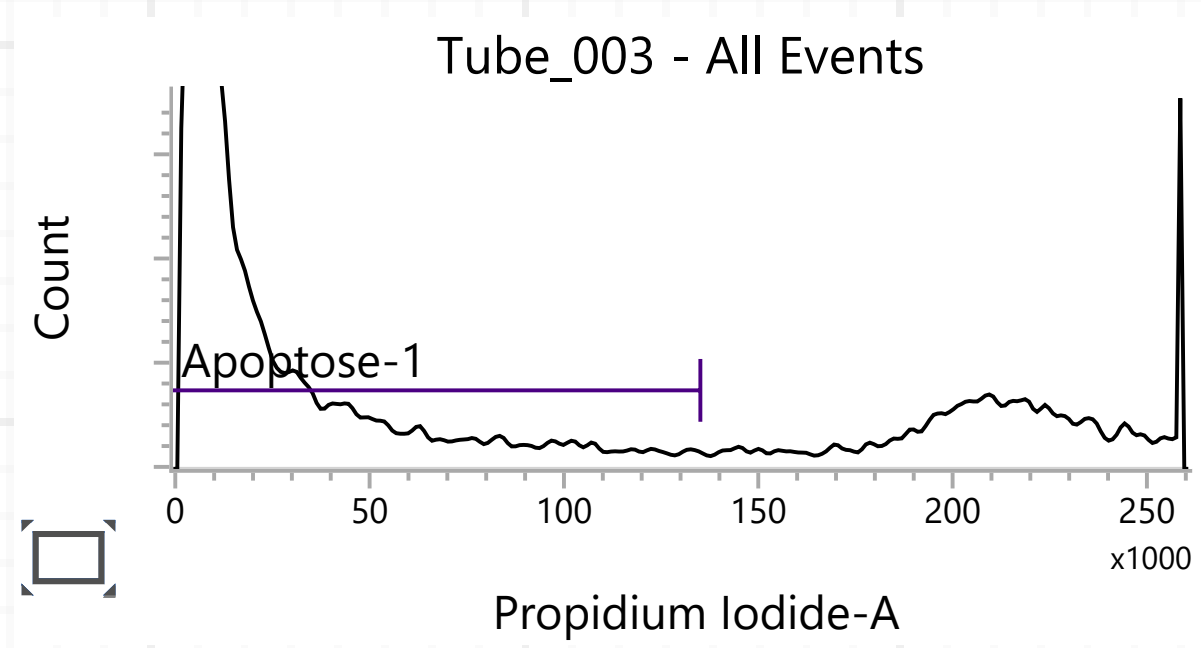

Statistics

Prozent apoptotische Zellen: Prozent apoptotische Zellen No Value % von Cells

Name	Events	% Total	% Parent	% Grandparent
Tube_001:All Events	11,045	100.00	***	***
Tube_001:Cells	10,000	90.54	90.54	***
Tube_002:All Events	10,000	100.00	***	***
Tube_002:Cells	8,890	88.90	88.90	***
Tube_003:All Events	10,000	100.00	***	***
Tube_003:Cells	8,932	89.32	89.32	***
Tube_001:Apoptose-1	6,183	55.98	55.98	***
Tube_002:Apoptose-1	6,126	61.26	61.26	***
Tube_003:Apoptose-1	5,874	58.74	58.74	***

DNA Fragmentierung

Sample ID: Jefri 5

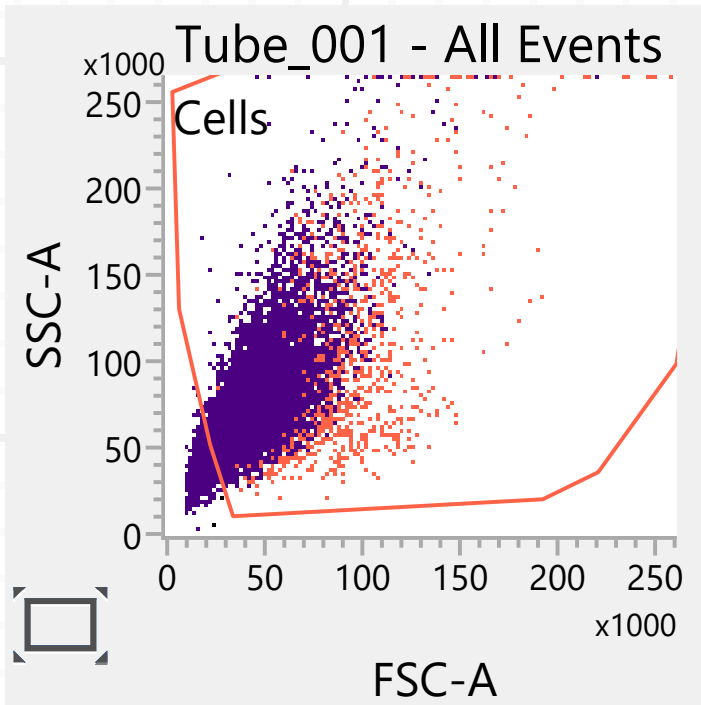

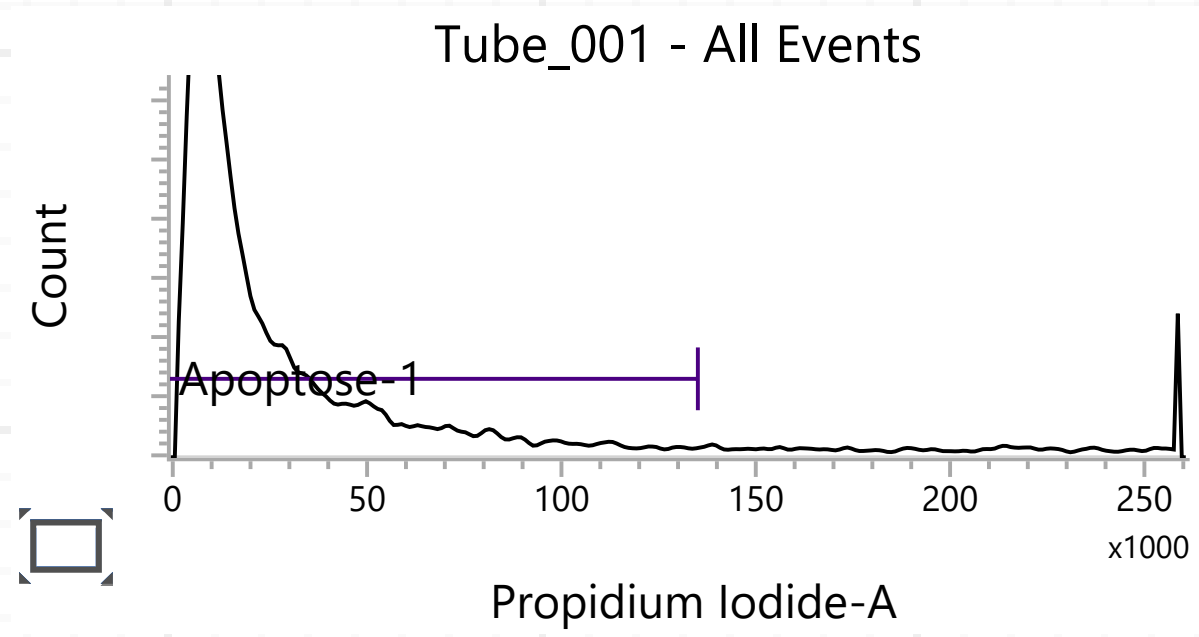

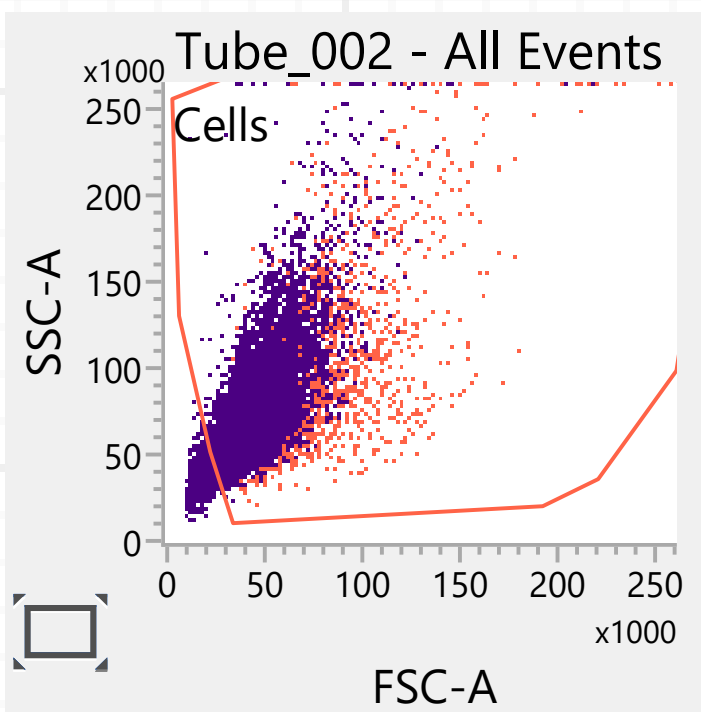

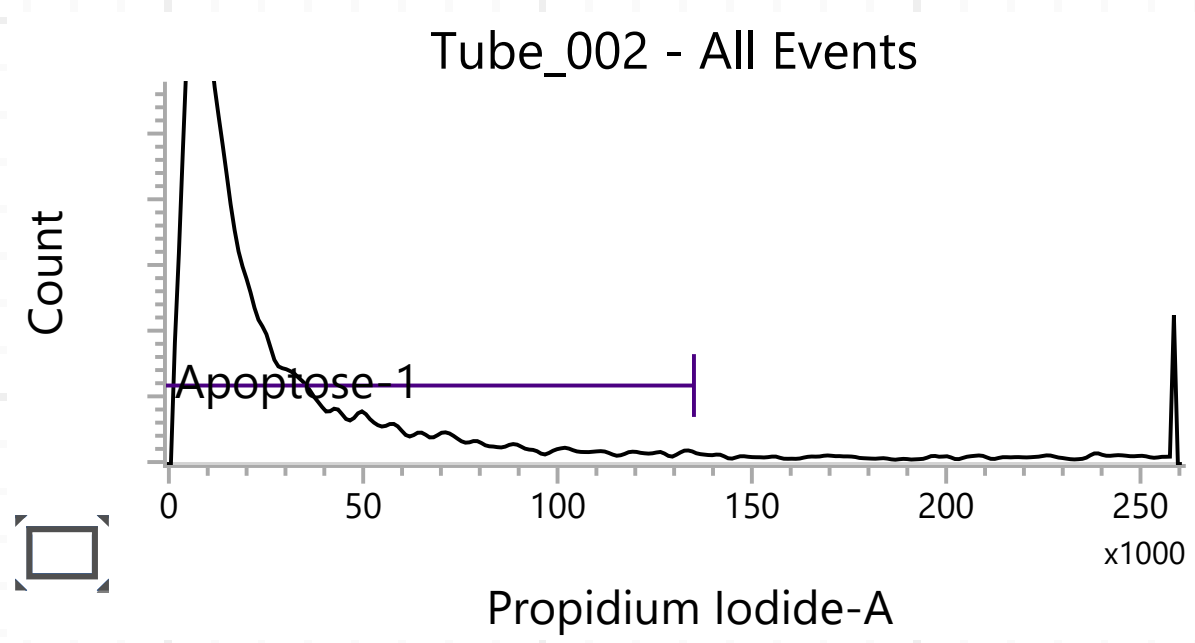

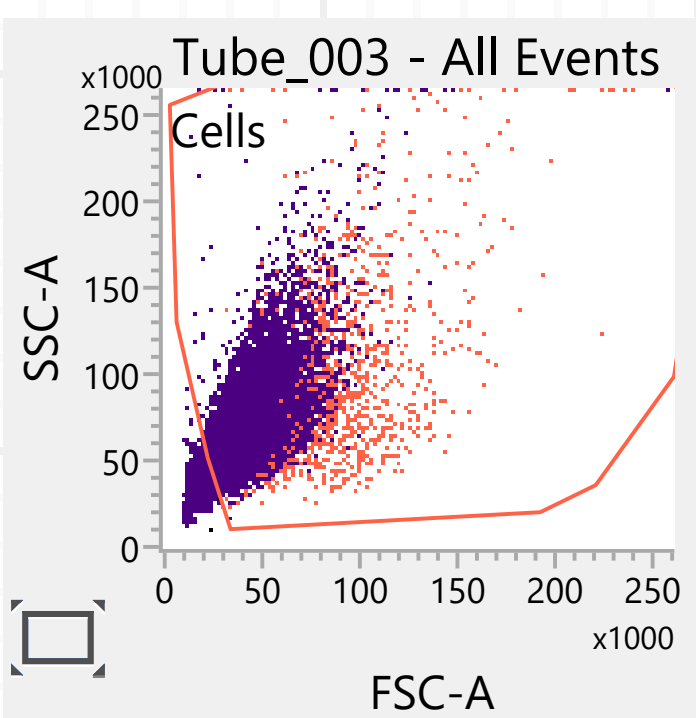

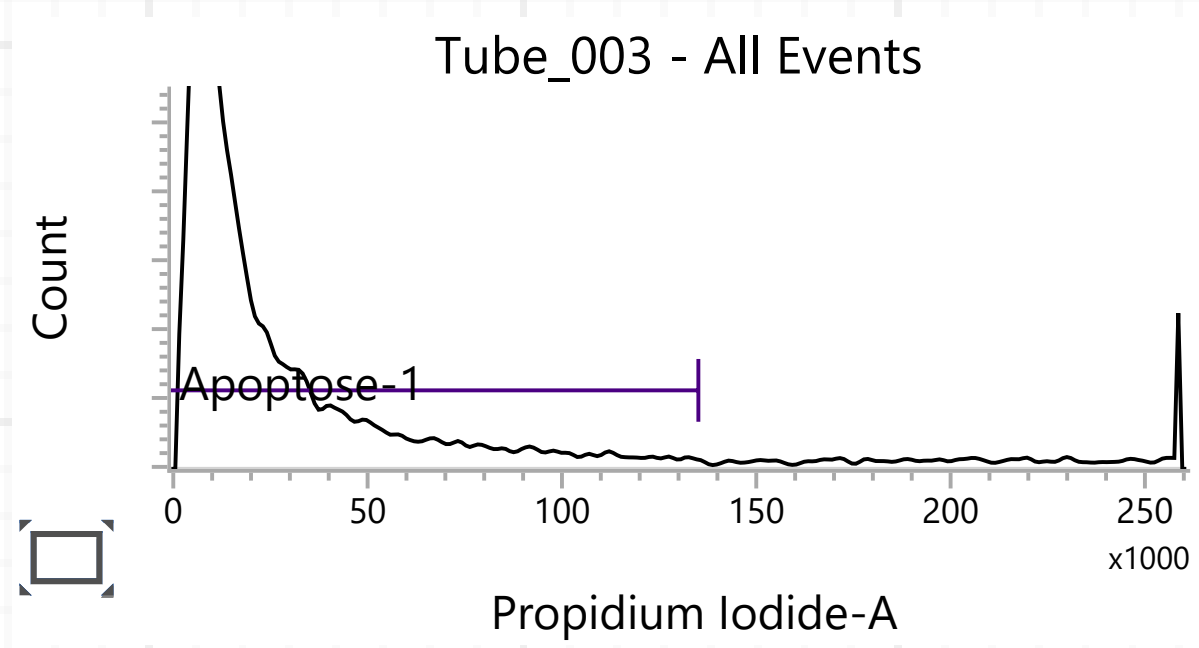

Statistics

Prozent apoptotische Zellen: Prozent apoptotische Zellen No Value % von Cells

Name	Events	% Total	% Parent	% Grandparent	
Tube_001:All Events	11,403	100.00	***	***	
Tube_001:Cells	10,000	87.70	87.70	***	
Tube_002:All Events	10,000	100.00	***	***	
Tube_002:Cells	9,035	90.35	90.35	***	
Tube_003:All Events	10,000	100.00	***	***	
Tube_003:Cells	8,846	88.46	88.46	***	
Tube_001:Apoptose-1	9,196	80.65	80.65	***	
Tube_002:Apoptose-1	8,067	80.67	80.67	***	
Tube_003:Apoptose-1	8,025	80.25	80.25	***	

<u>DNA Fragmentierung</u>

Sample ID: Jefri EtoNeu

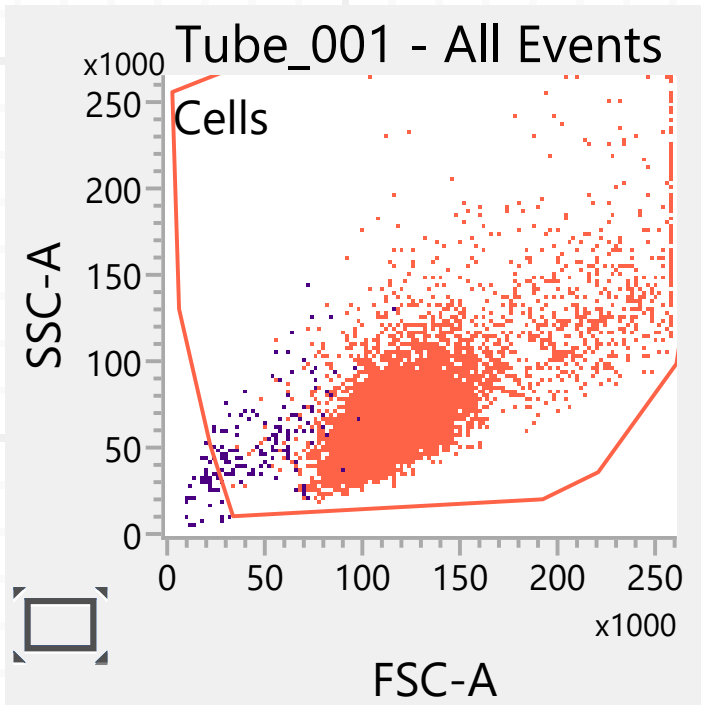

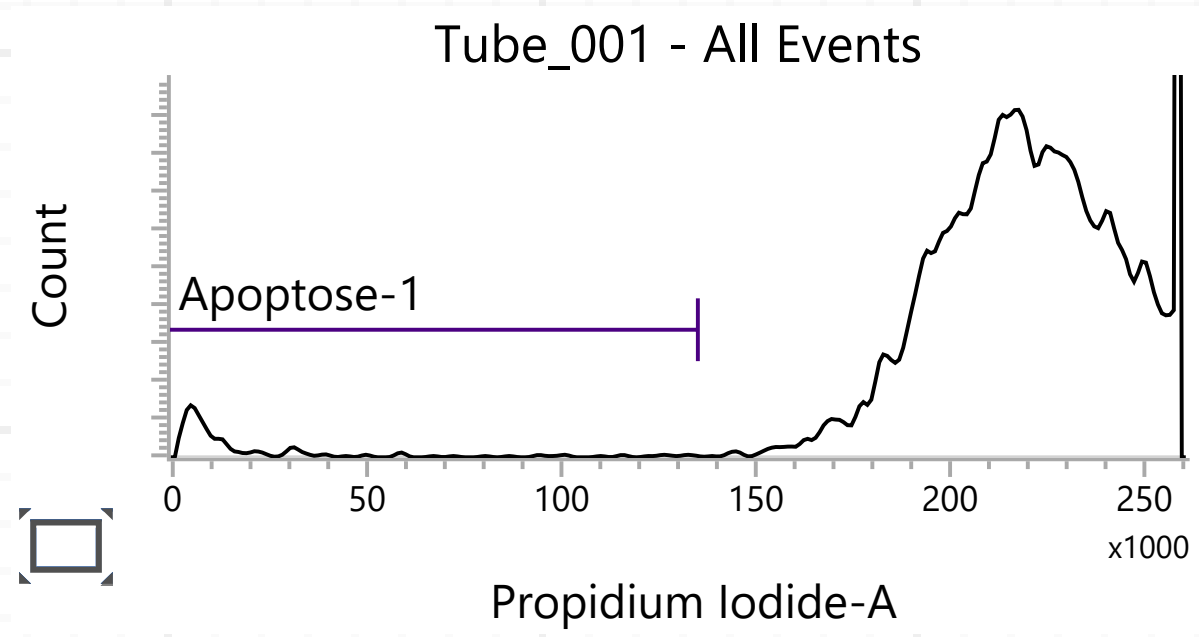

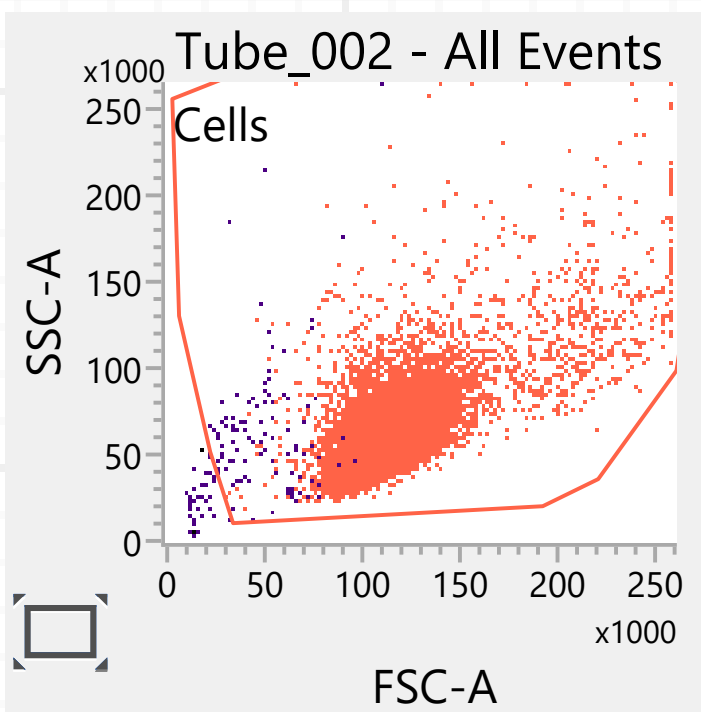

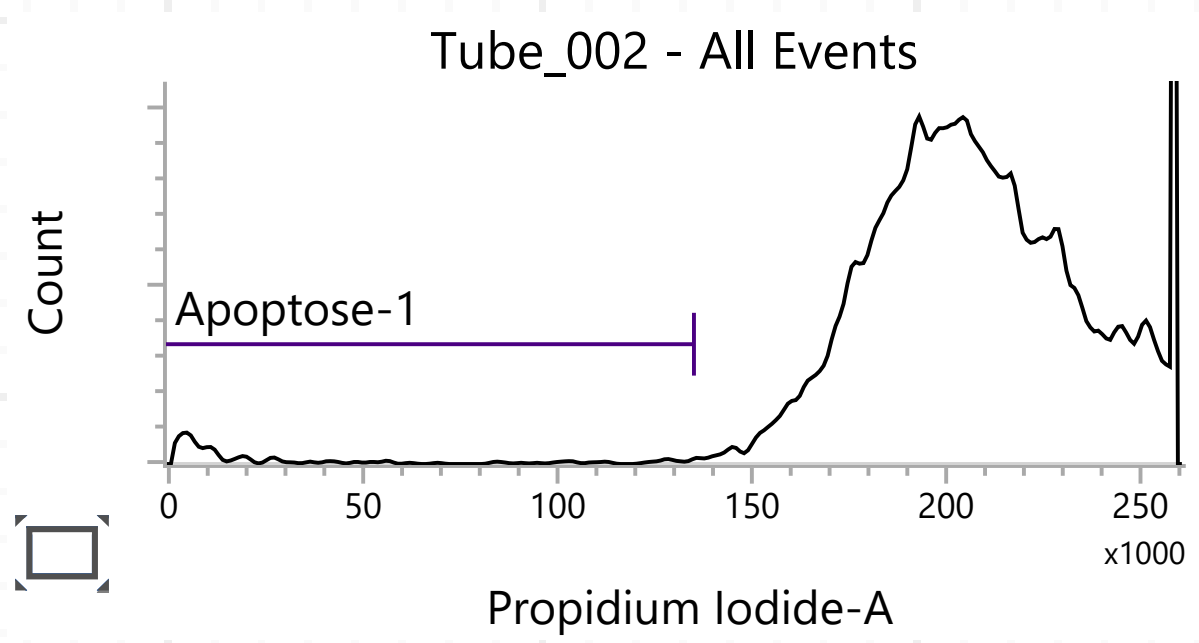

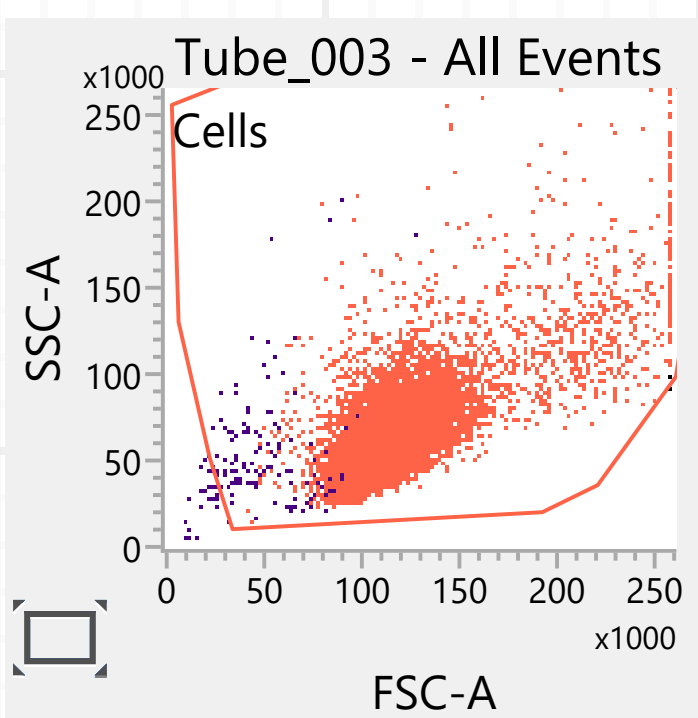

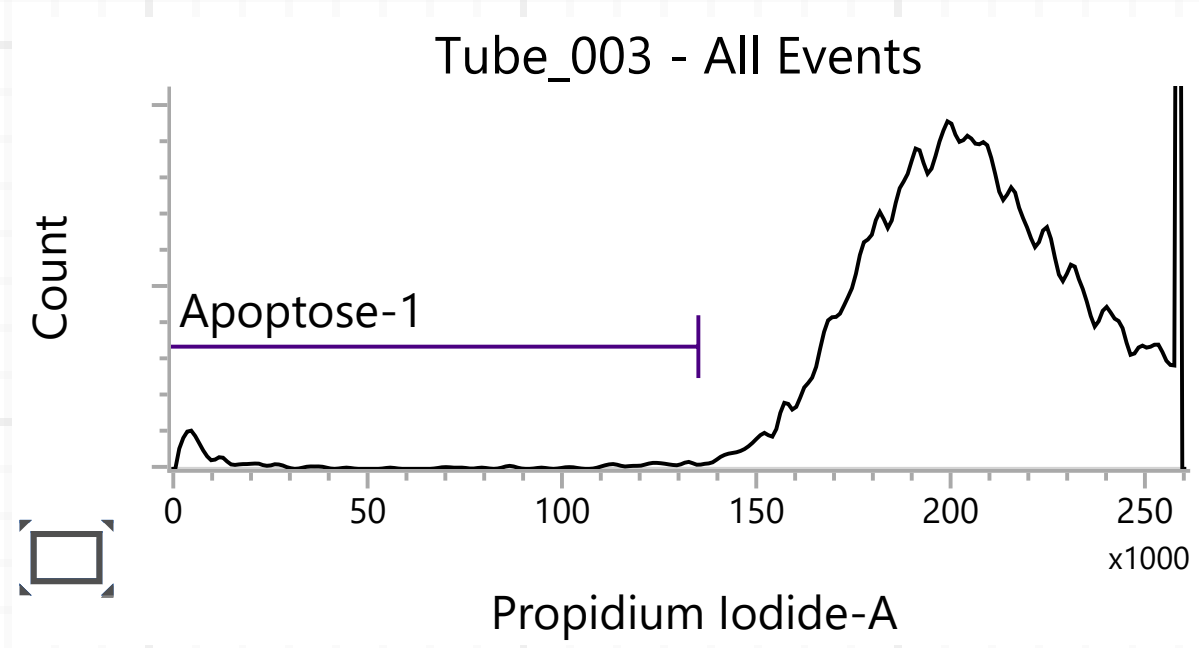

Statistics

Prozent apoptotische Zellen: Prozent apoptotische Zellen No Value % von Cells

Name	Events	% Total	% Parent	% Grandparent	
Tube_001:All Events	10,038	100.00	***	***	
Tube_001:Cells	10,000	99.62	99.62	***	
Tube_002:All Events	10,000	100.00	***	***	
Tube_002:Cells	9,965	99.65	99.65	***	
Tube_003:All Events	10,000	100.00	***	***	
Tube_003:Cells	9,975	99.75	99.75	***	
Tube_001:Apoptose-1	166	1.65	1.65	***	
Tube_002:Apoptose-1	138	1.38	1.38	***	
Tube_003:Apoptose-1	141	1.41	1.41	***	

DNA Fragmentierung

Sample ID: Jefri K

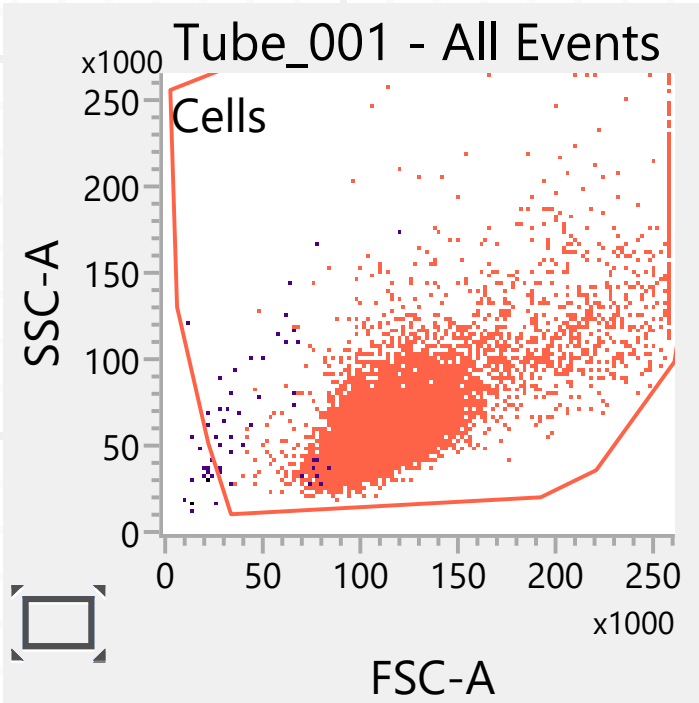

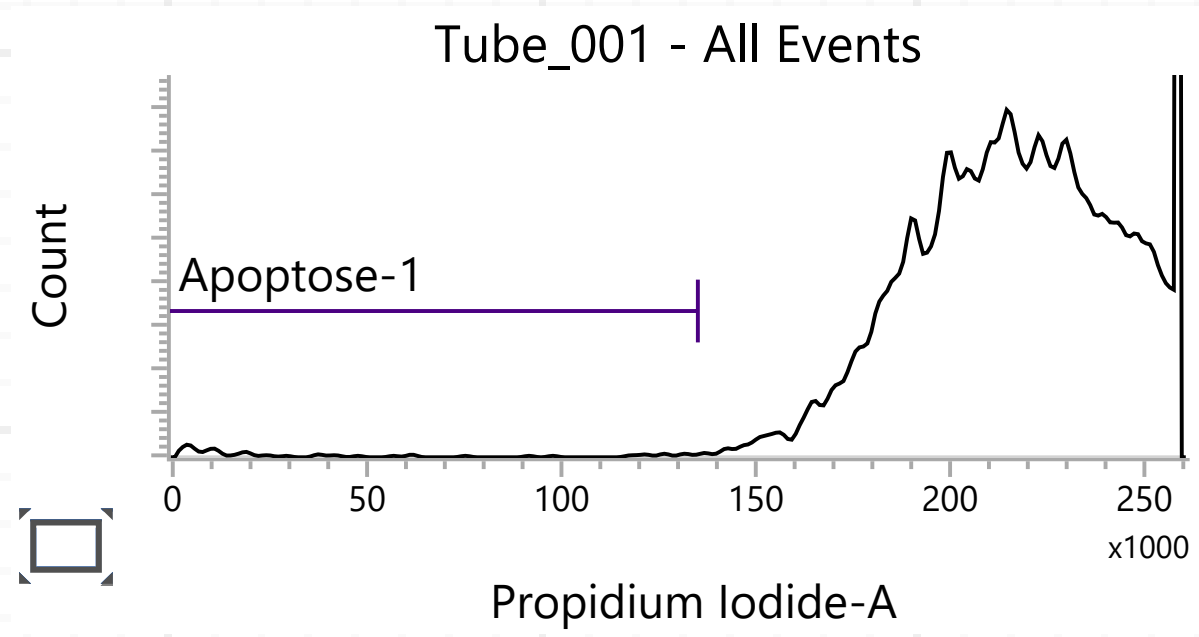

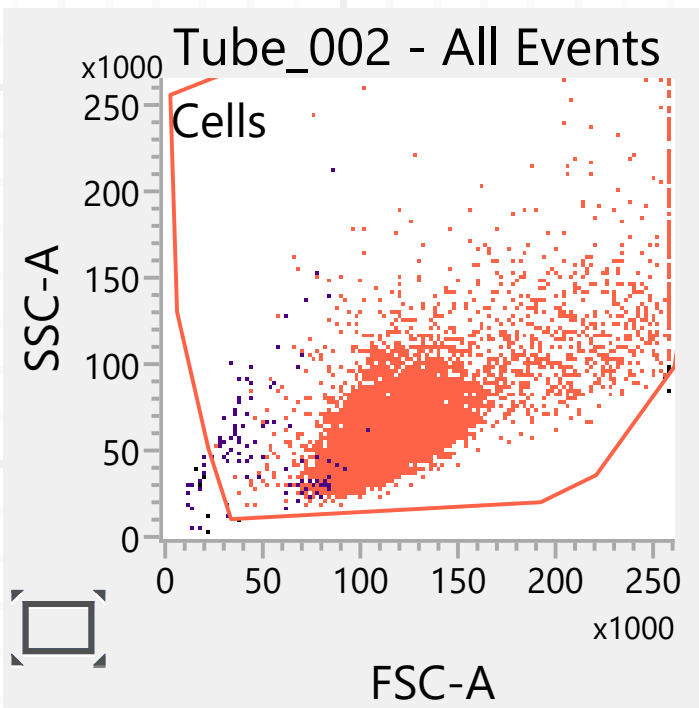

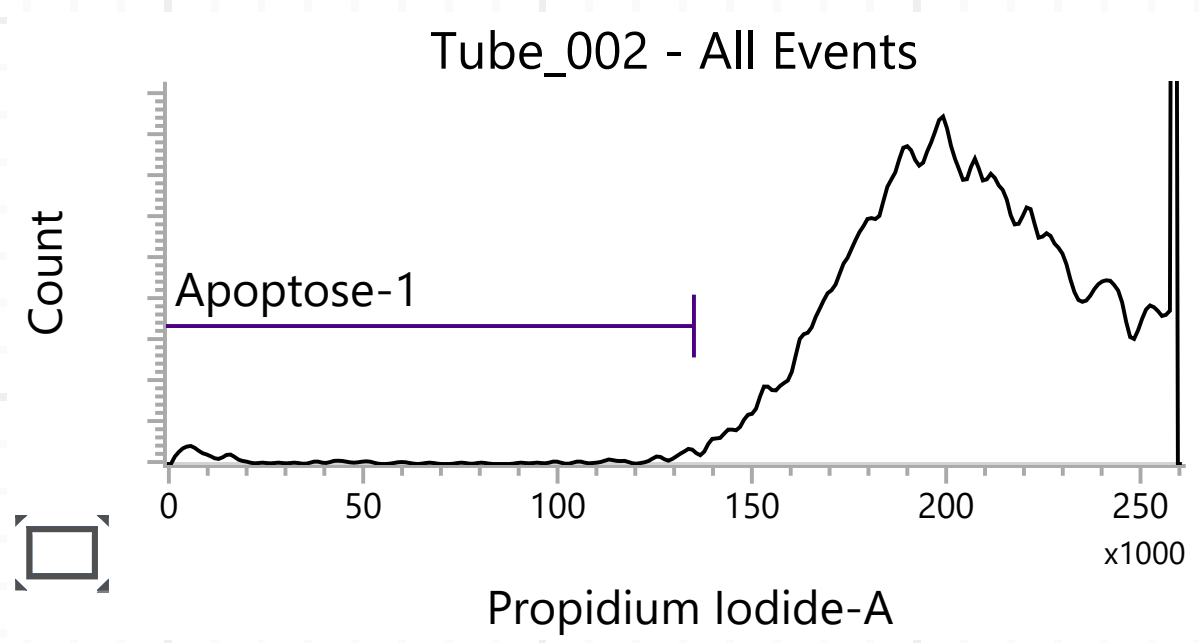

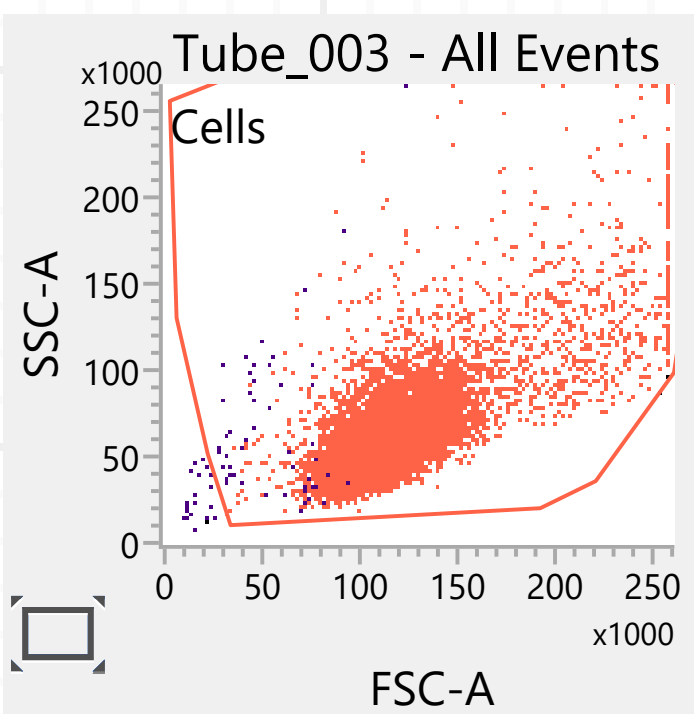

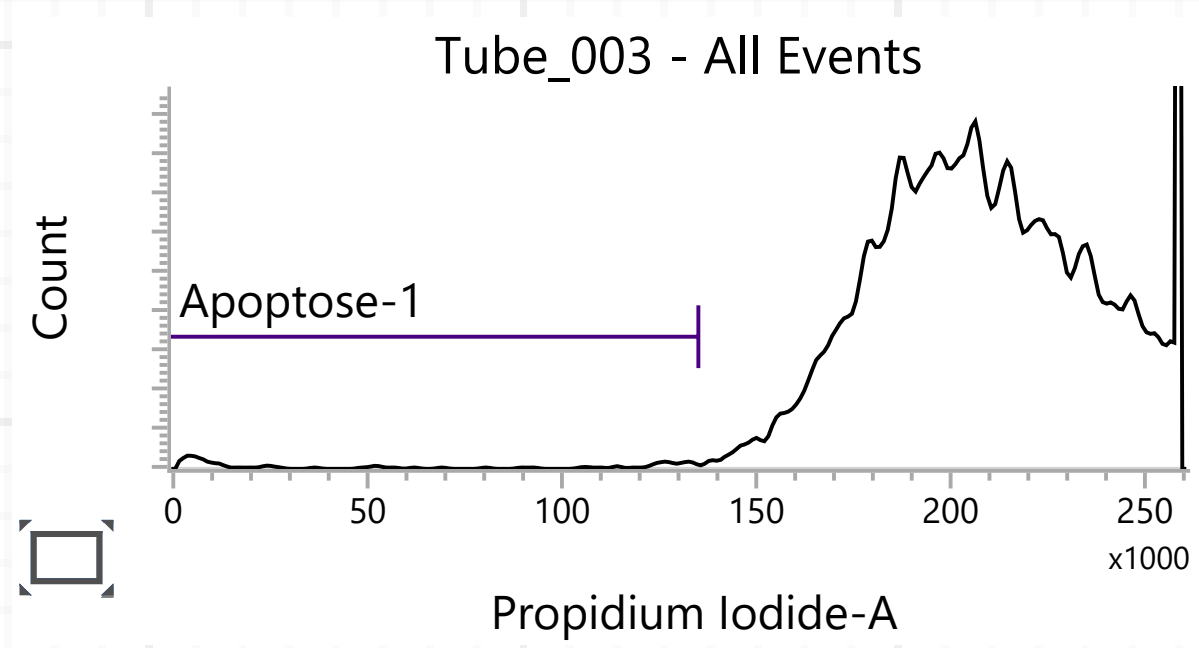

Statistics

Prozent apoptotische Zellen: Prozent apoptotische Zellen No Value % von Cells

Name	Events	% Total	% Parent	% Grandparent
Tube_001:All Events	10,014	100.00	***	***
Tube_001:Cells	10,000	99.86	99.86	***
Tube_002:All Events	10,000	100.00	***	***
Tube_002:Cells	9,975	99.75	99.75	***
Tube_003:All Events	10,000	100.00	***	***
Tube_003:Cells	9,977	99.77	99.77	***
Tube_001:Apoptose-1	54	0.54	0.54	***
Tube_002:Apoptose-1	99	0.99	0.99	***
Tube_003:Apoptose-1	73	0.73	0.73	***

DNA Fragmentierung

Sample ID: N6-0,1

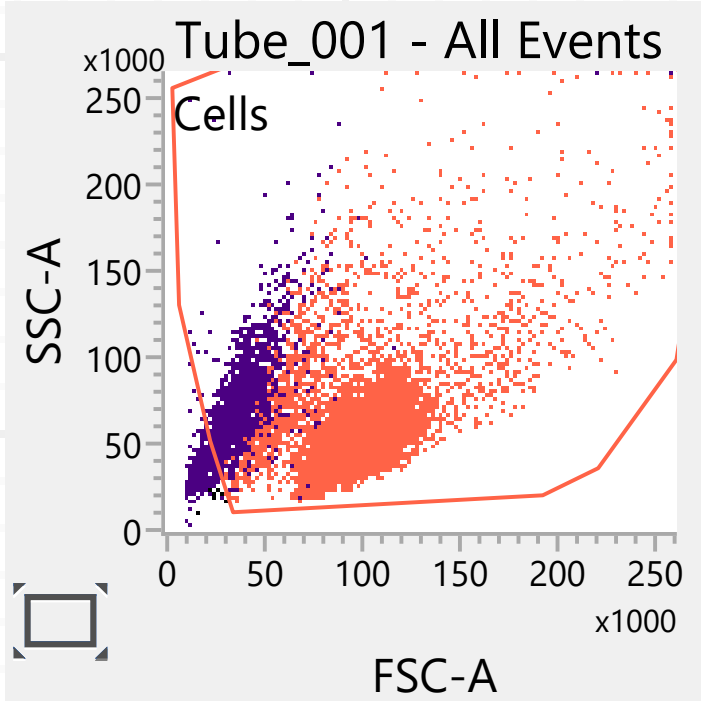

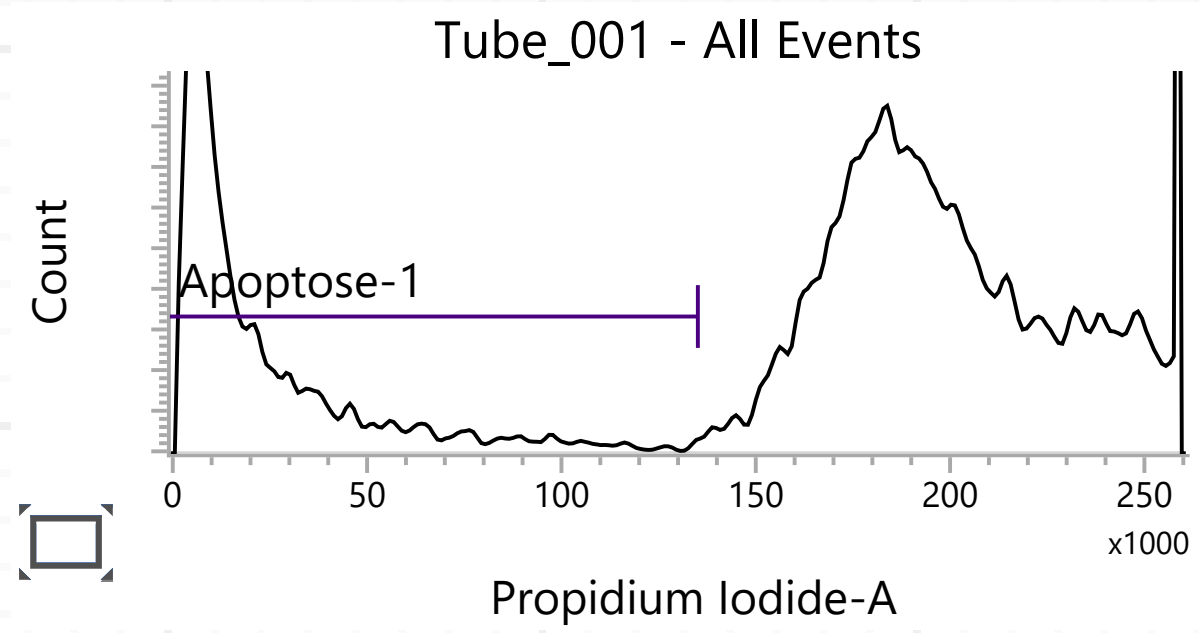

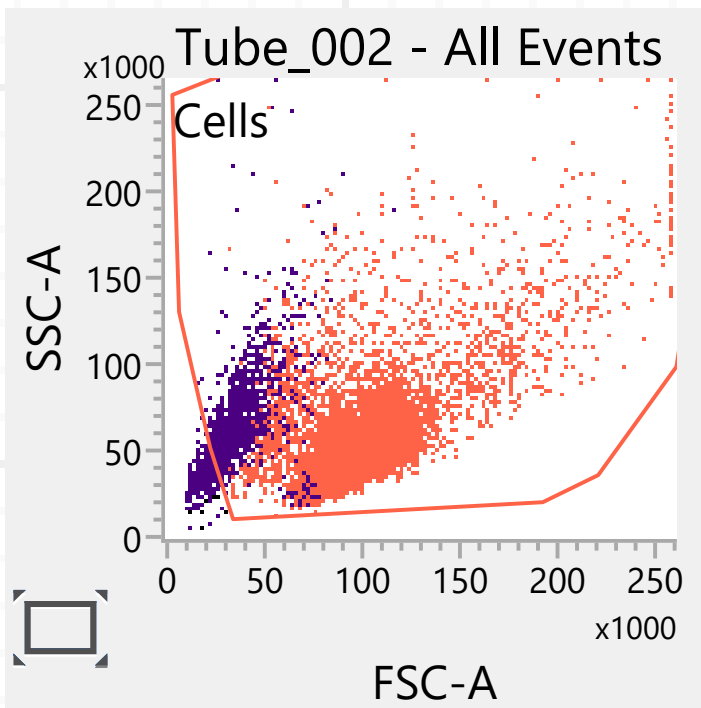

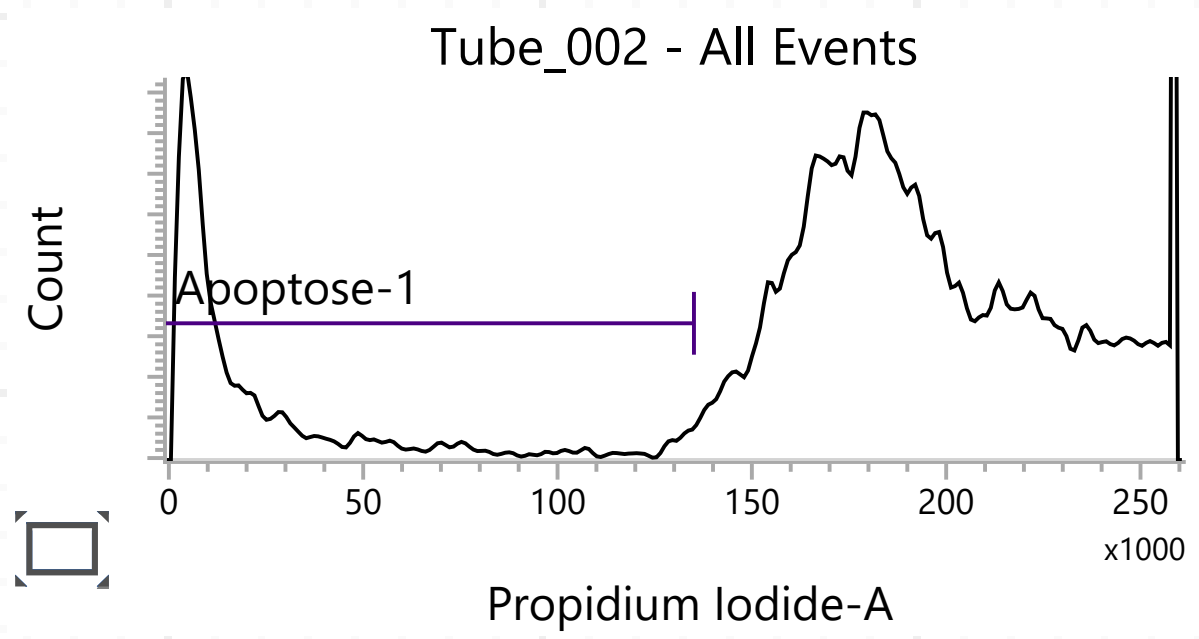

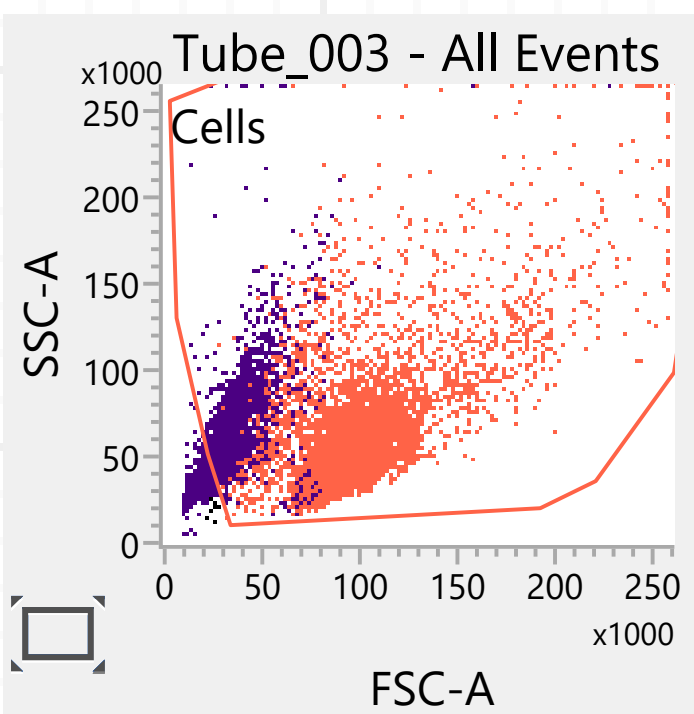

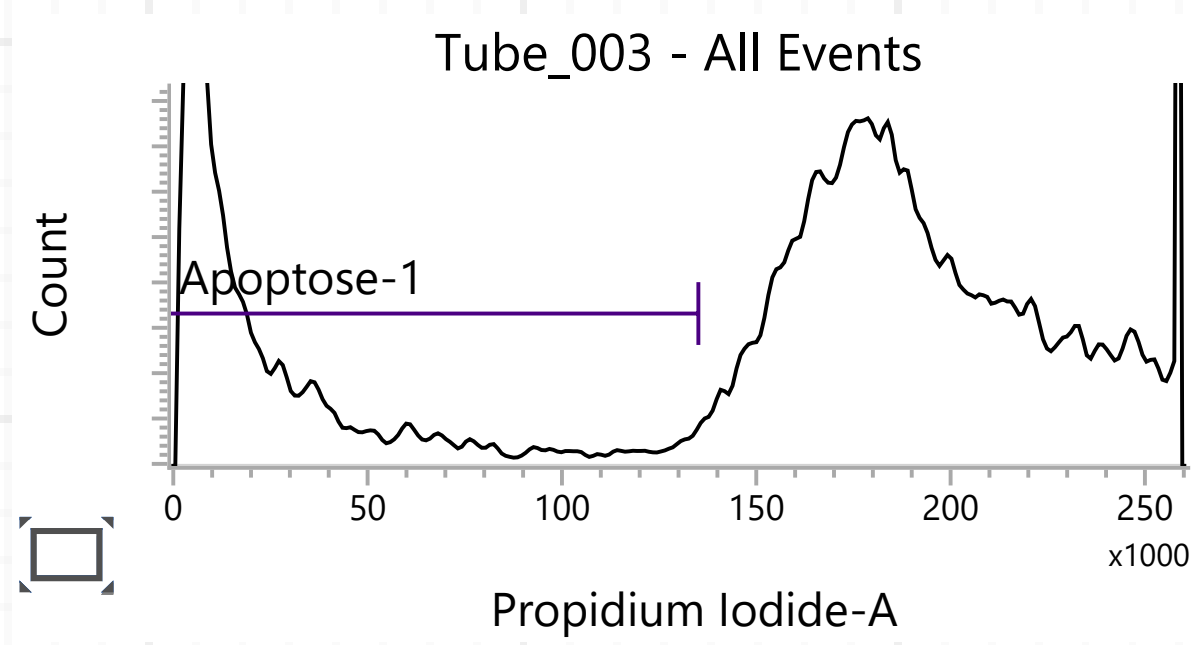

Statistics

Prozent apoptotische Zellen: Prozent apoptotische Zellen No Value % von Cells

Name	Events	% Total	% Parent	% Grandparent
Tube_001:All Events	10,389	100.00	***	***
Tube_001:Cells	10,000	96.26	96.26	***
Tube_002:All Events	10,000	100.00	***	***
Tube_002:Cells	9,698	96.98	96.98	***
Tube_003:All Events	10,000	100.00	***	***
Tube_003:Cells	9,592	95.92	95.92	***
Tube_001:Apoptose-1	2,112	20.33	20.33	***
Tube_002:Apoptose-1	1,383	13.83	13.83	***
Tube_003:Apoptose-1	2,184	21.84	21.84	***

DNA Fragmentierung

Sample ID: N6-0,5

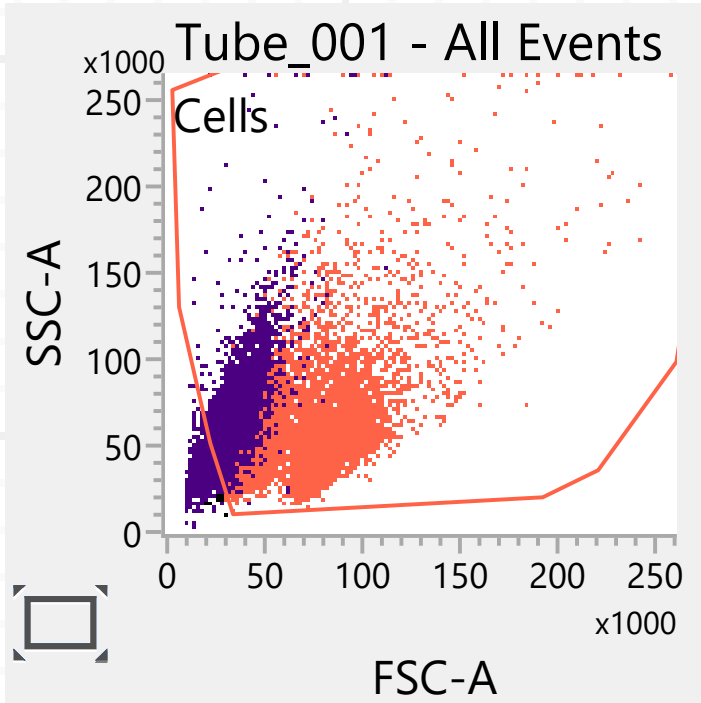

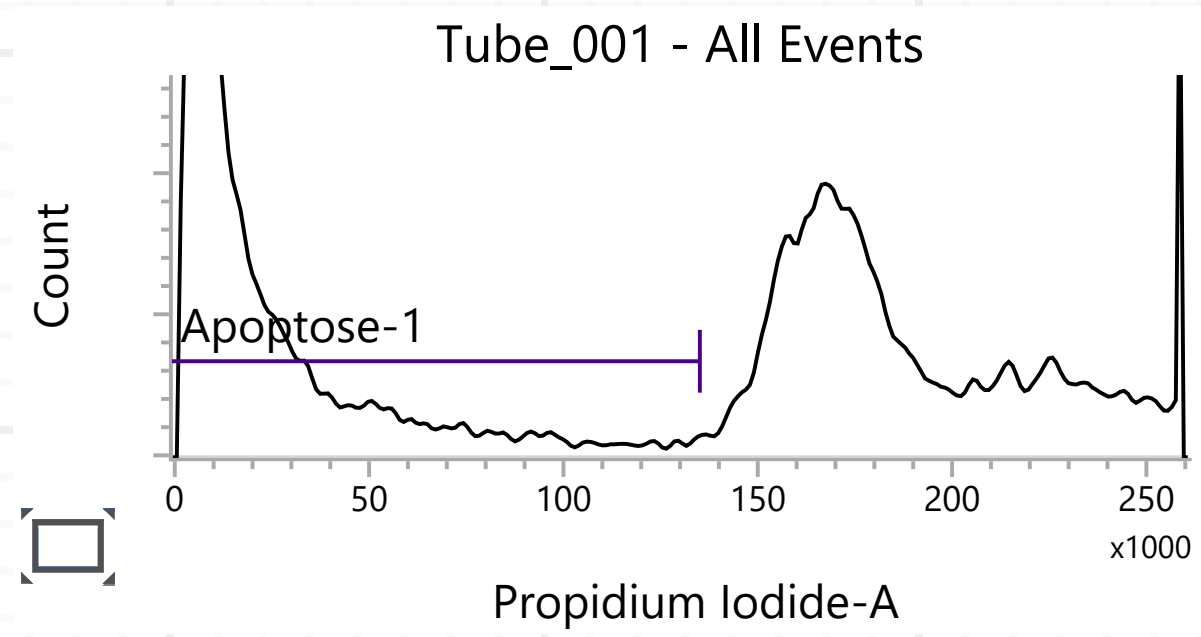

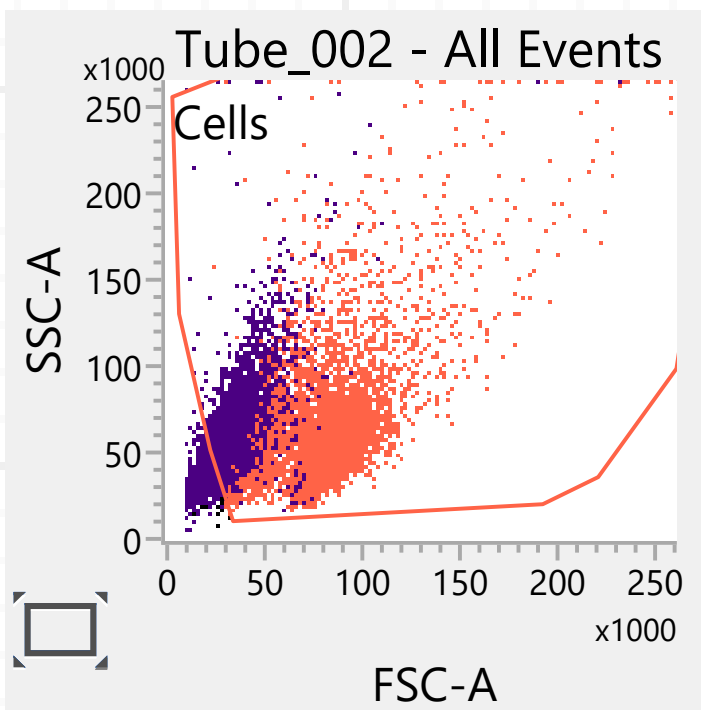

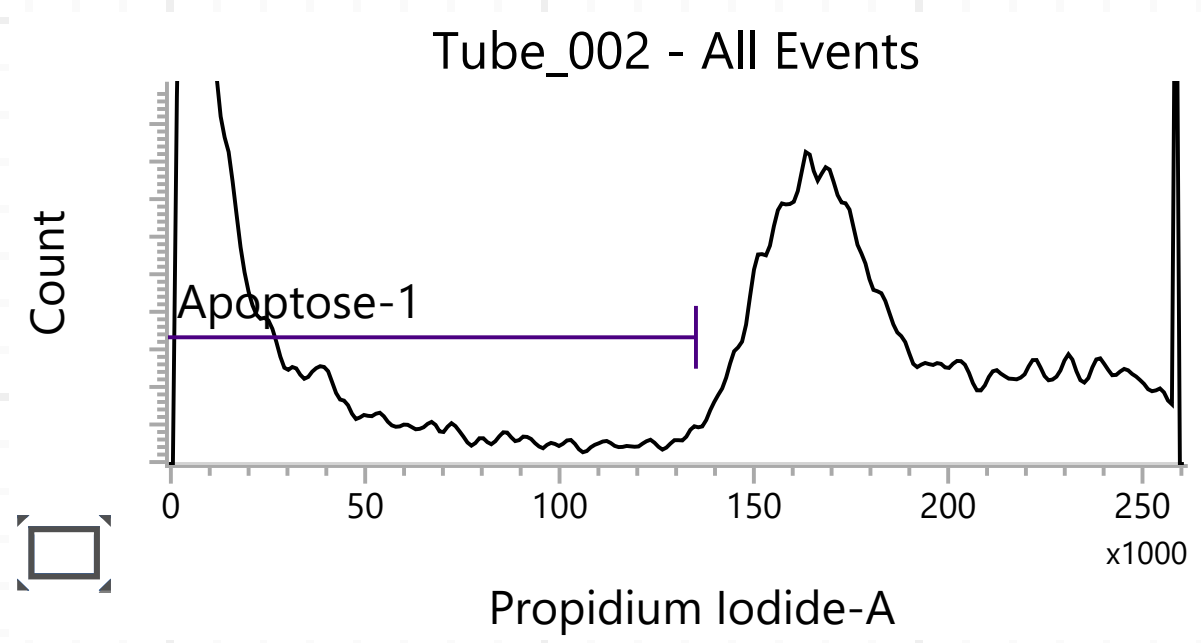

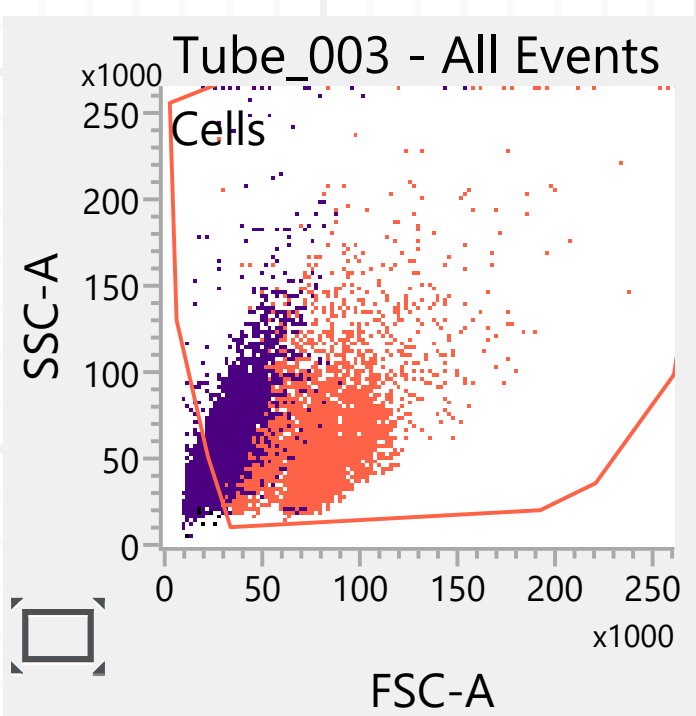

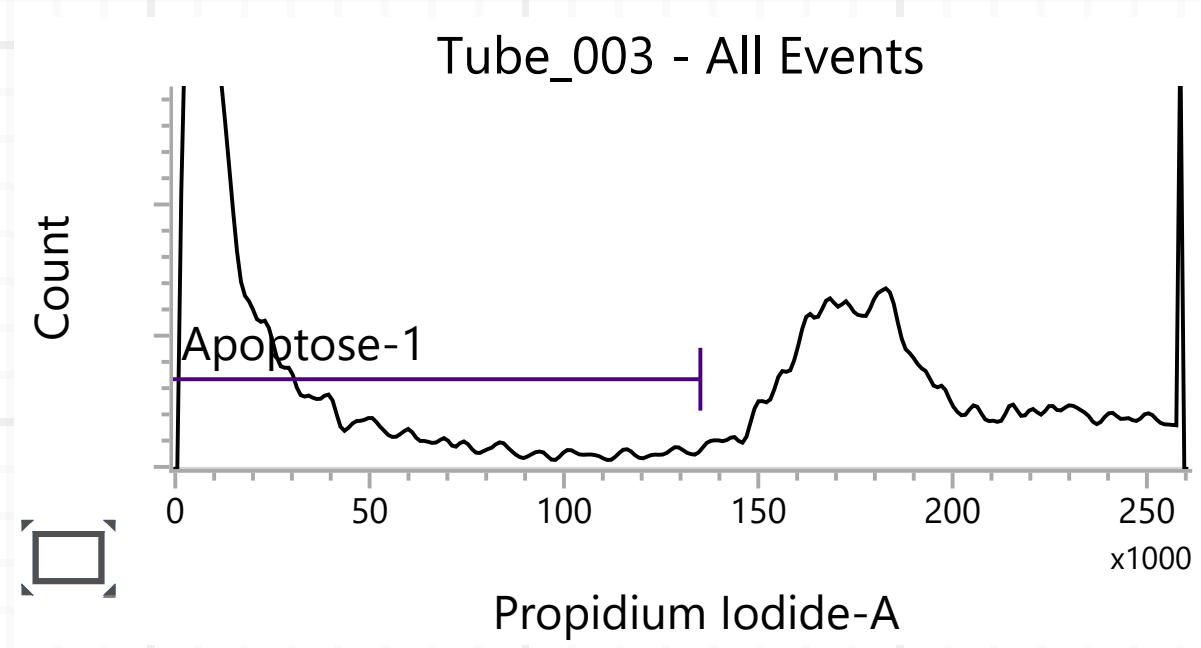

Statistics

Prozent apoptotische Zellen: Prozent apoptotische Zellen No Value % von Cells

Name	Events	% Total	% Parent	% Grandparent
Tube_001:All Events	11,160	100.00	***	***
Tube_001:Cells	10,000	89.61	89.61	***
Tube_002:All Events	10,000	100.00	***	***
Tube_002:Cells	8,989	89.89	89.89	***
Tube_003:All Events	10,000	100.00	***	***
Tube_003:Cells	8,822	88.22	88.22	***
Tube_001:Apoptose-1	4,414	39.55	39.55	***
Tube_002:Apoptose-1	3,782	37.82	37.82	***
Tube_003:Apoptose-1	4,429	44.29	44.29	***

DNA Fragmentierung

Sample ID: N6-1

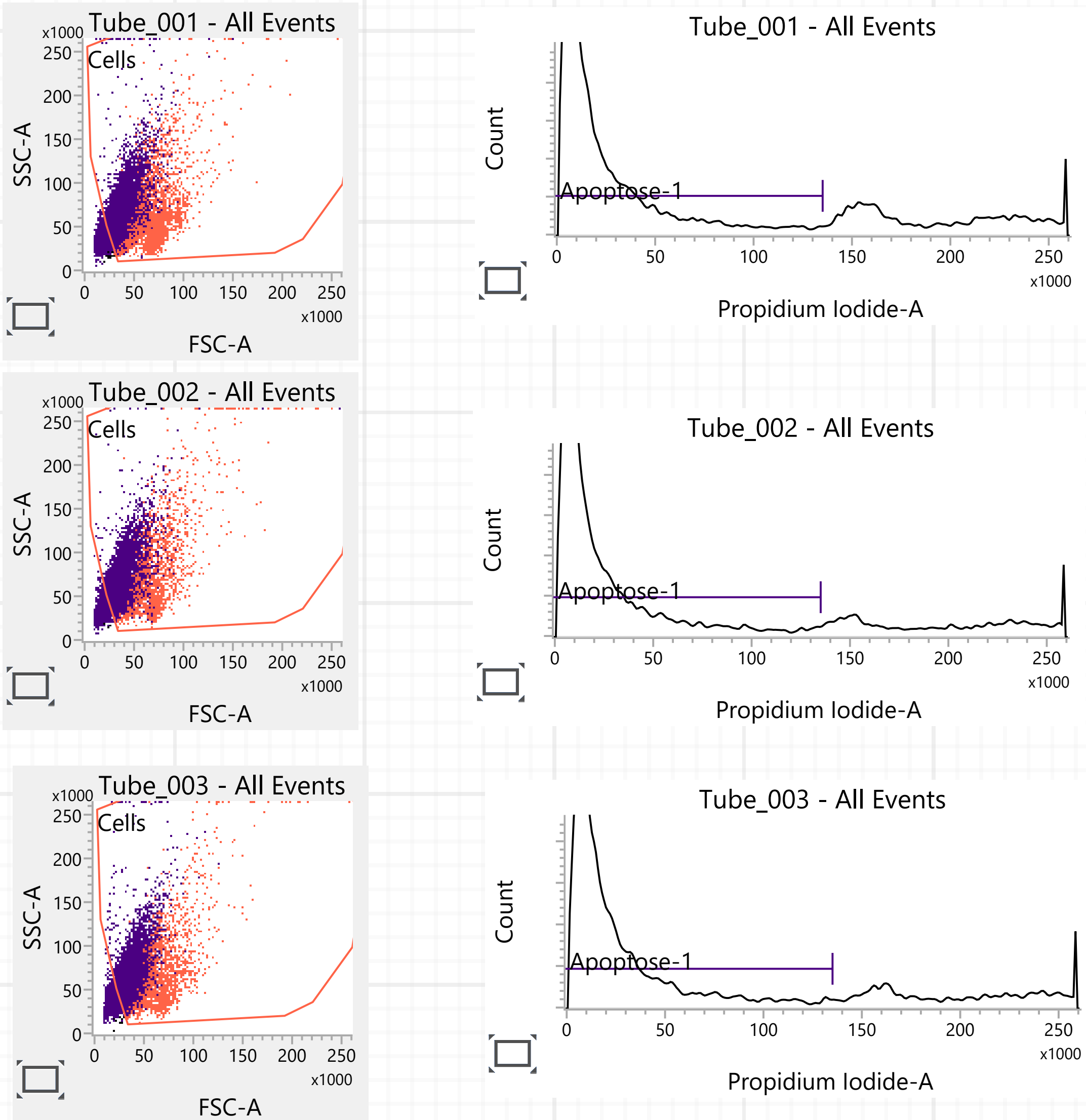

Statistics

Prozent apoptotische Zellen: Prozent apoptotische Zellen No Value % von Cells

Name	Events	% Total	% Parent	% Grandparent
Tube_001:All Events	12,112	100.00	***	***
Tube_001:Cells	10,000	82.56	82.56	***
Tube_002:All Events	10,000	100.00	***	***
Tube_002:Cells	8,327	83.27	83.27	***
Tube_003:All Events	10,000	100.00	***	***
Tube_003:Cells	8,191	81.91	81.91	***
Tube_001:Apoptose-1	8,354	68.97	68.97	***
Tube_002:Apoptose-1	7,223	72.23	72.23	***
Tube_003:Apoptose-1	7,140	71.40	71.40	***

DNA Fragmentierung

Sample ID: N6-2,5

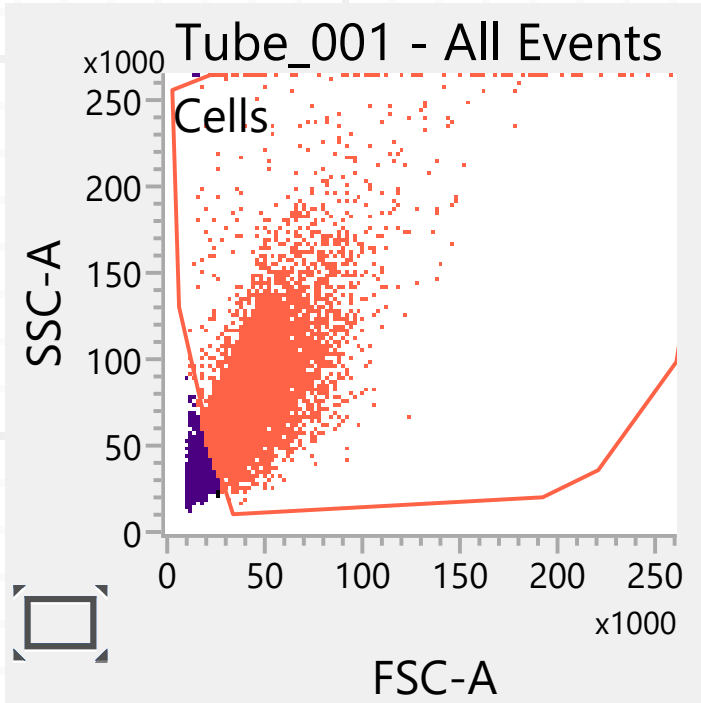

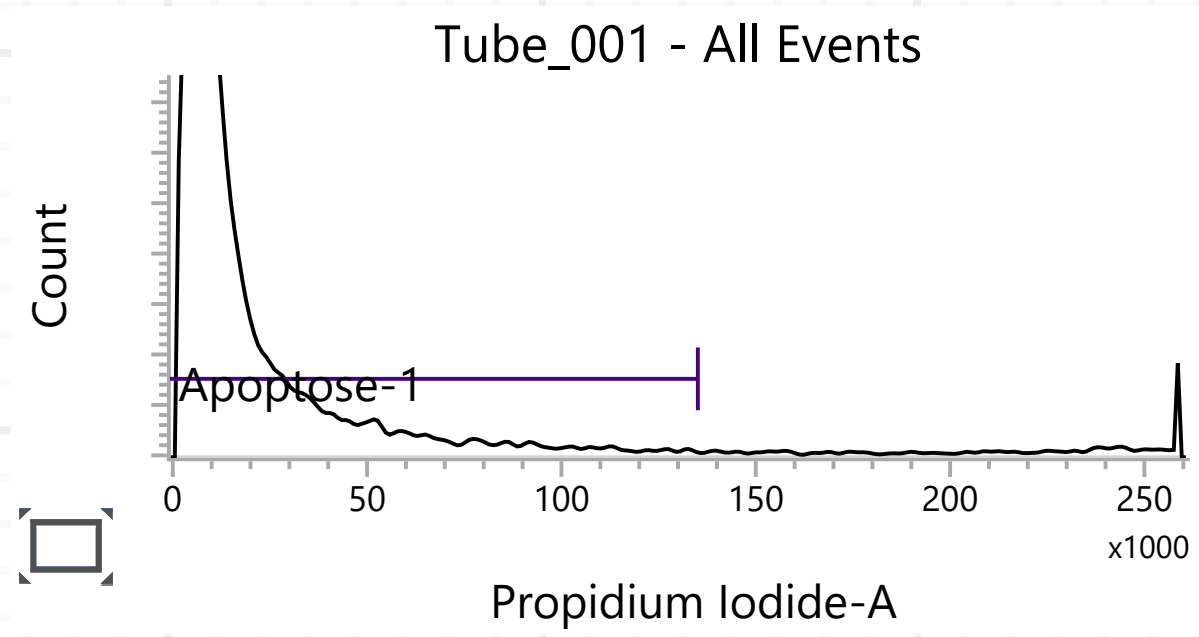

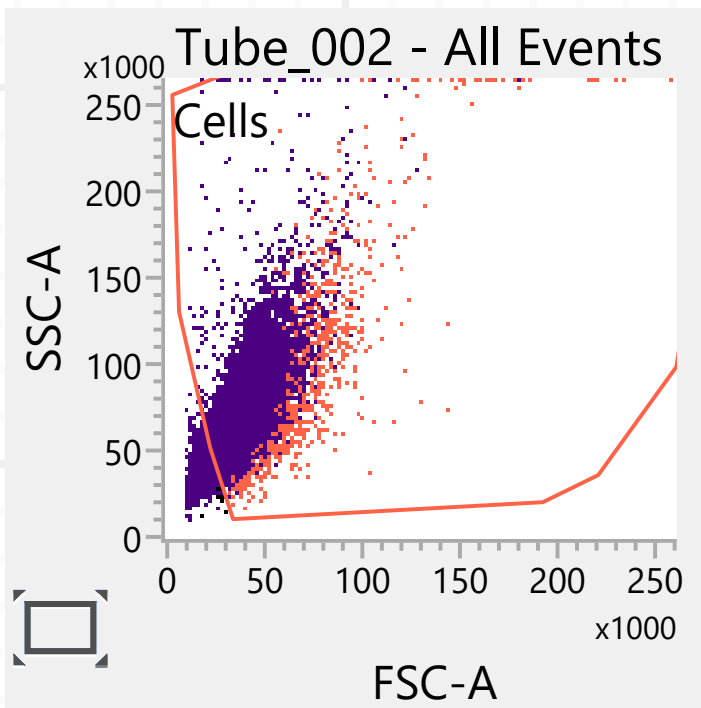

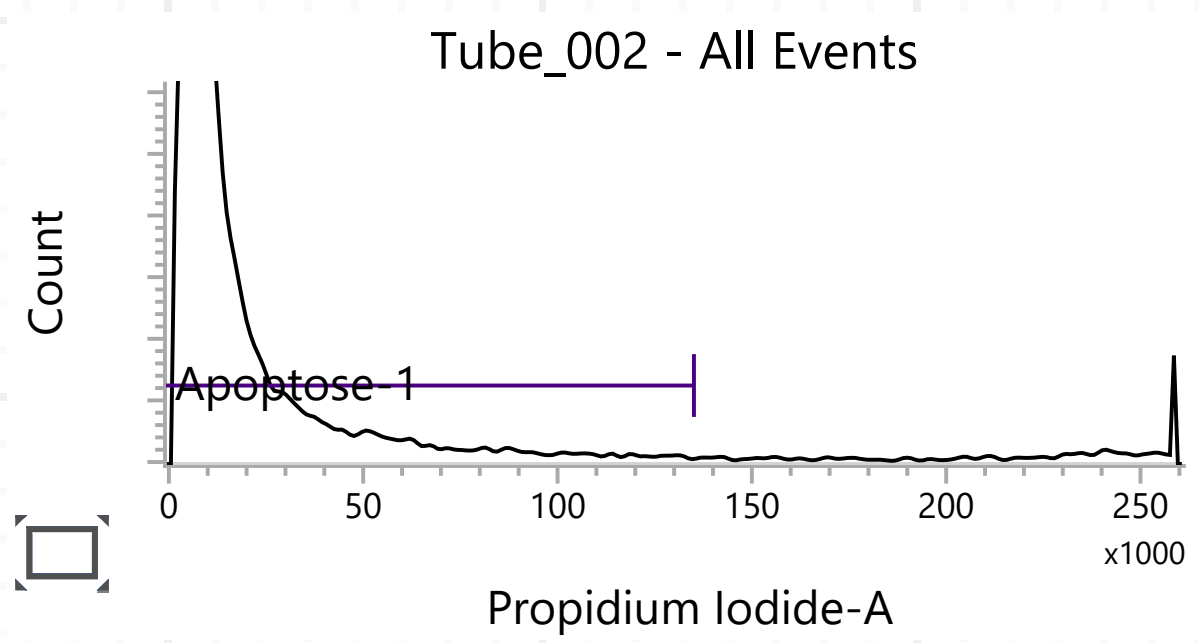

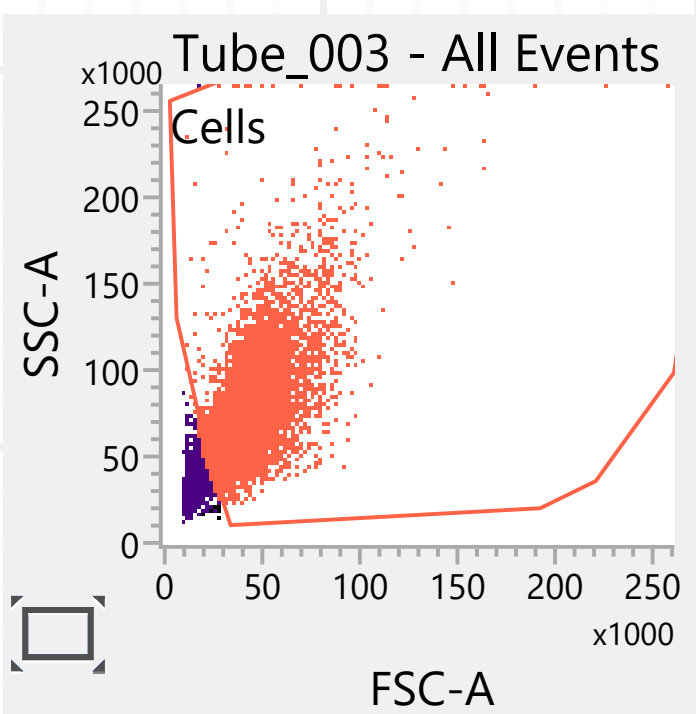

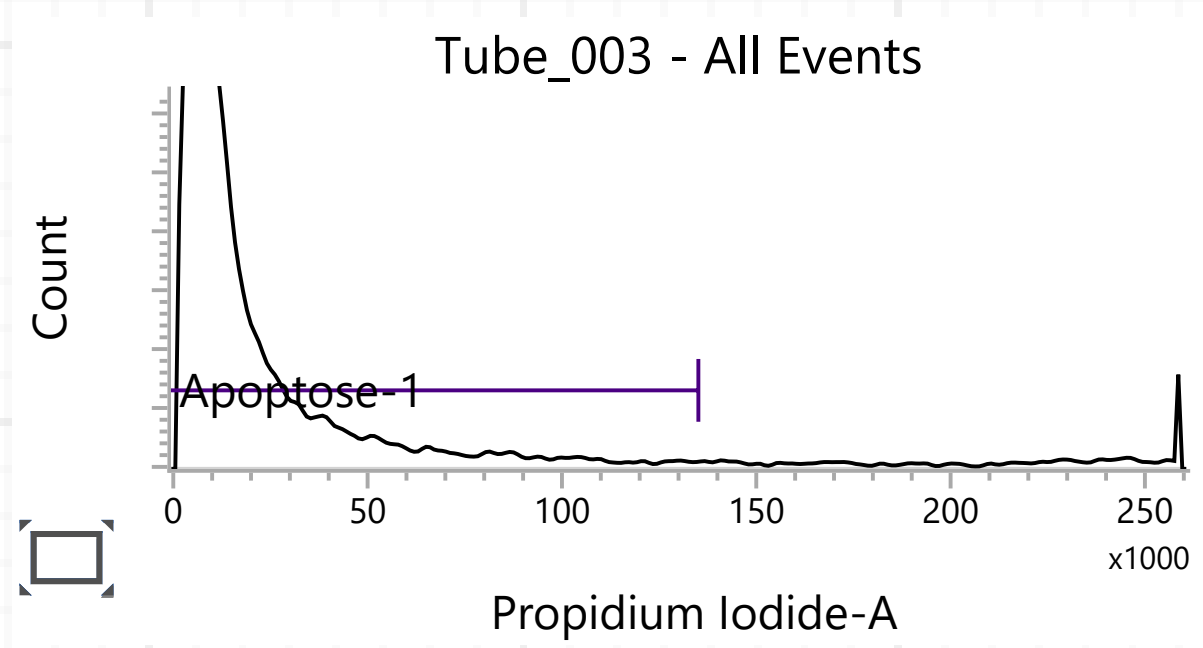

Statistics

Prozent apoptotische Zellen: Prozent apoptotische Zellen No Value % von Cells

Name	Events	% Total	% Parent	% Grandparent
Tube_001:All Events	12,091	100.00	***	***
Tube_001:Cells	10,000	82.71	82.71	***
Tube_002:All Events	10,000	100.00	***	***
Tube_002:Cells	8,434	84.34	84.34	***
Tube_003:All Events	10,000	100.00	***	***
Tube_003:Cells	8,154	81.54	81.54	***
Tube_001:Apoptose-1	10,390	85.93	85.93	***
Tube_002:Apoptose-1	8,366	83.66	83.66	***
Tube_003:Apoptose-1	8,484	84.84	84.84	***

DNA Fragmentierung

Sample ID: N6-5

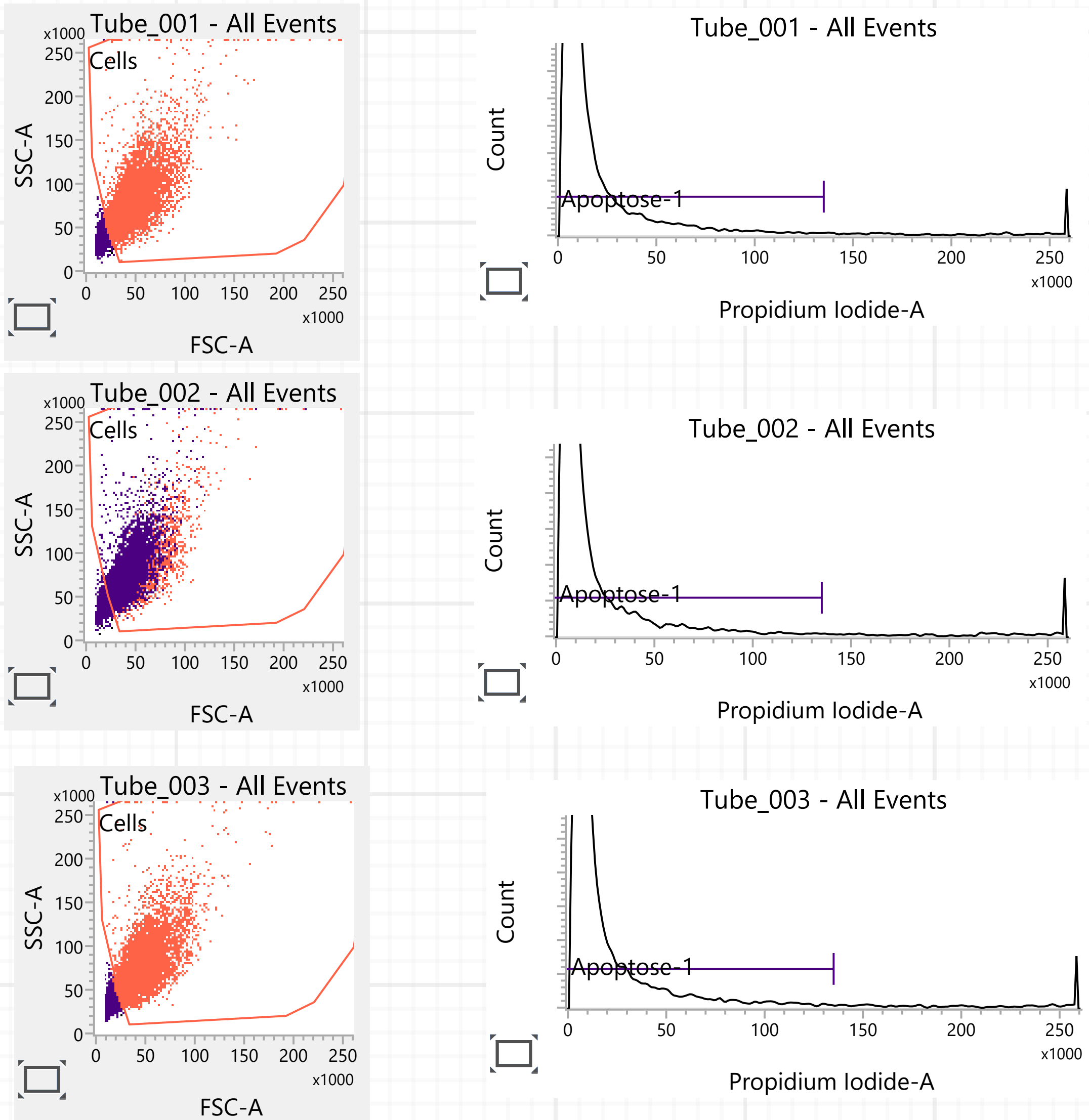

Statistics

Prozent apoptotische Zellen: Prozent apoptotische Zellen No Value % von Cells

Name	Events	% Total	% Parent	% Grandparent
Tube_001:All Events	11,640	100.00	***	***
Tube_001:Cells	10,000	85.91	85.91	***
Tube_002:All Events	10,000	100.00	***	***
Tube_002:Cells	8,586	85.86	85.86	***
Tube_003:All Events	10,000	100.00	***	***
Tube_003:Cells	8,498	84.98	84.98	***
Tube_001:Apoptose-1	10,141	87.12	87.12	***
Tube_002:Apoptose-1	8,577	85.77	85.77	***
Tube_003:Apoptose-1	8,671	86.71	86.71	***

DNA Fragmentierung

Sample ID: N6-AraC

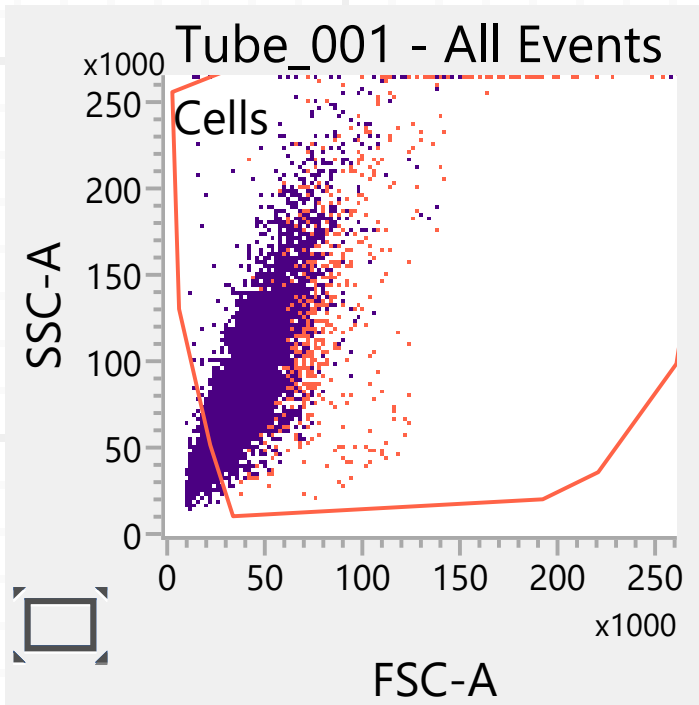

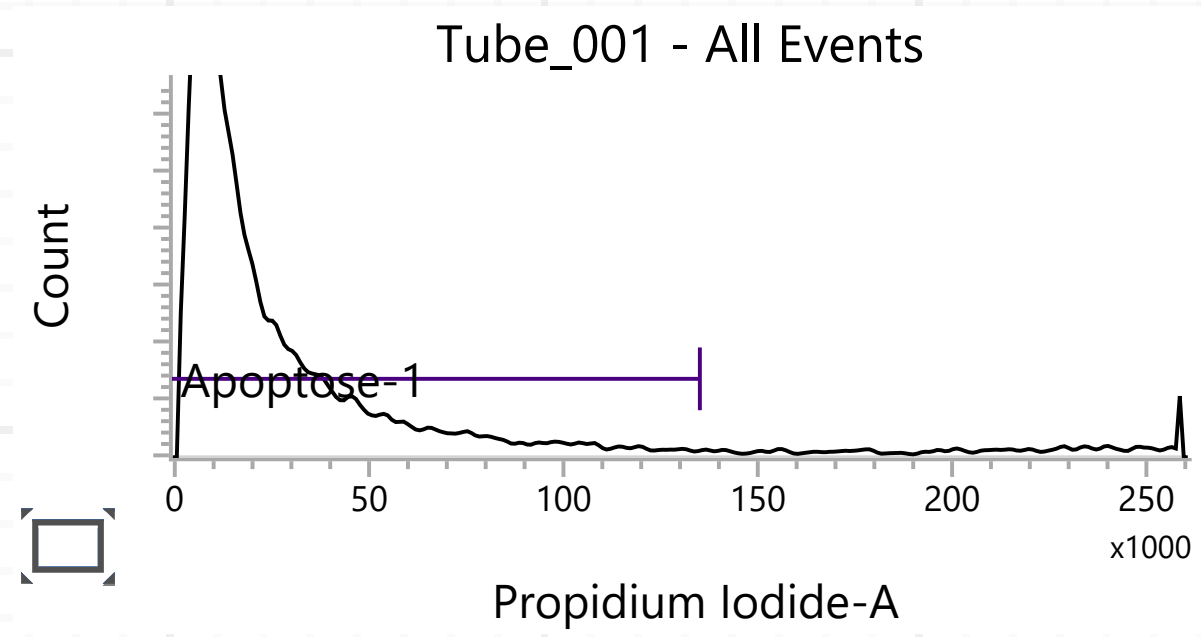

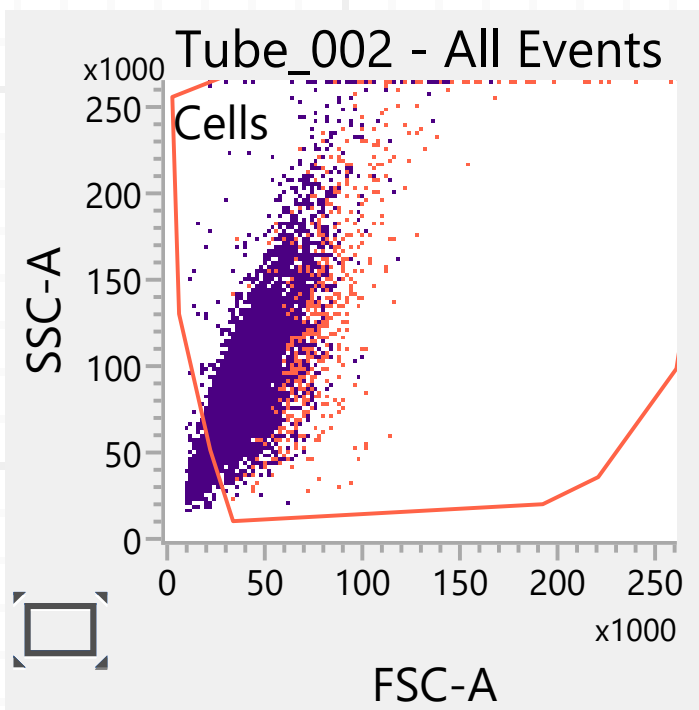

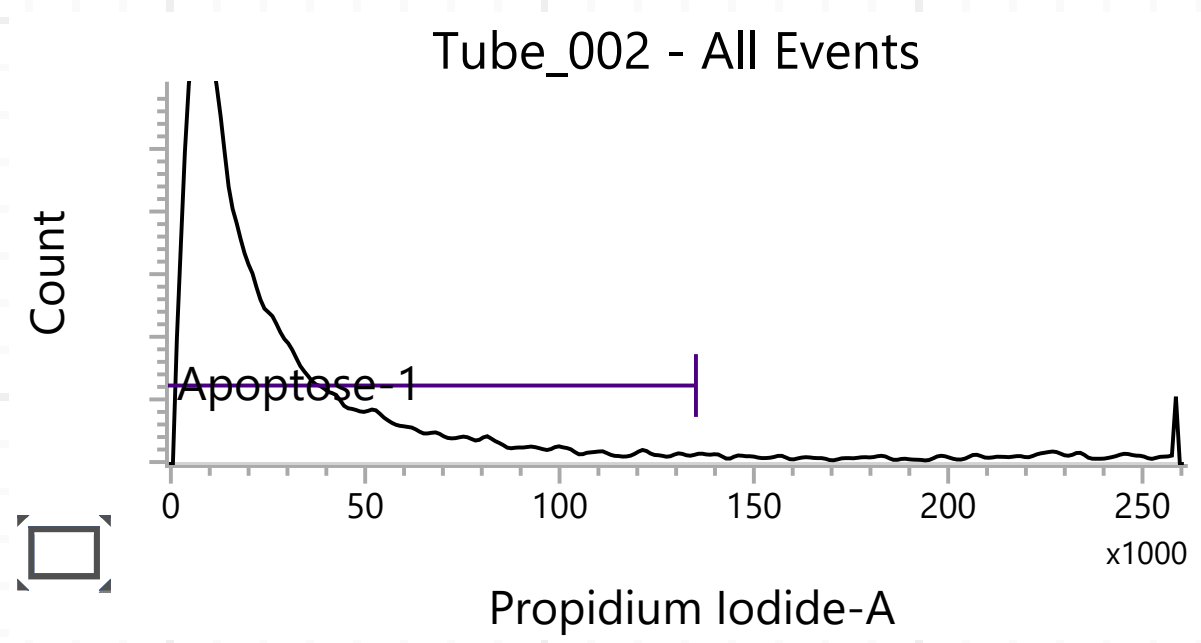

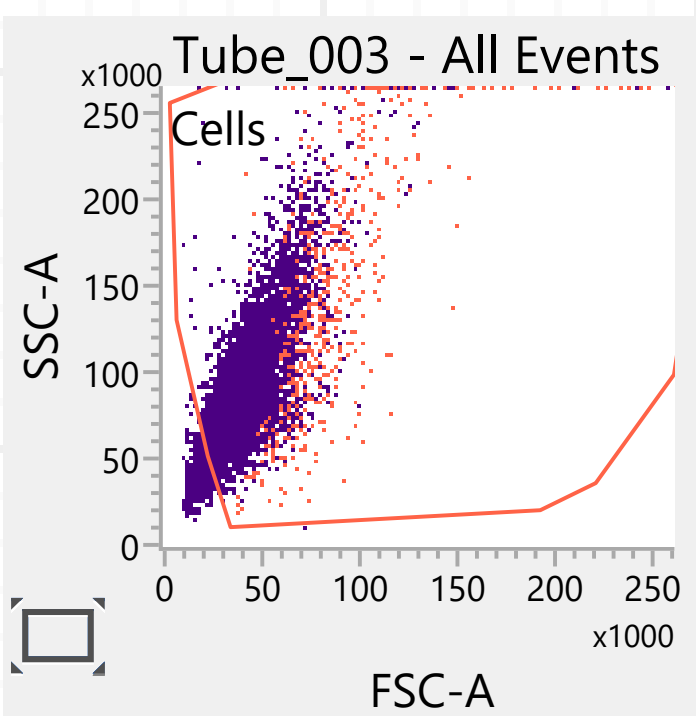

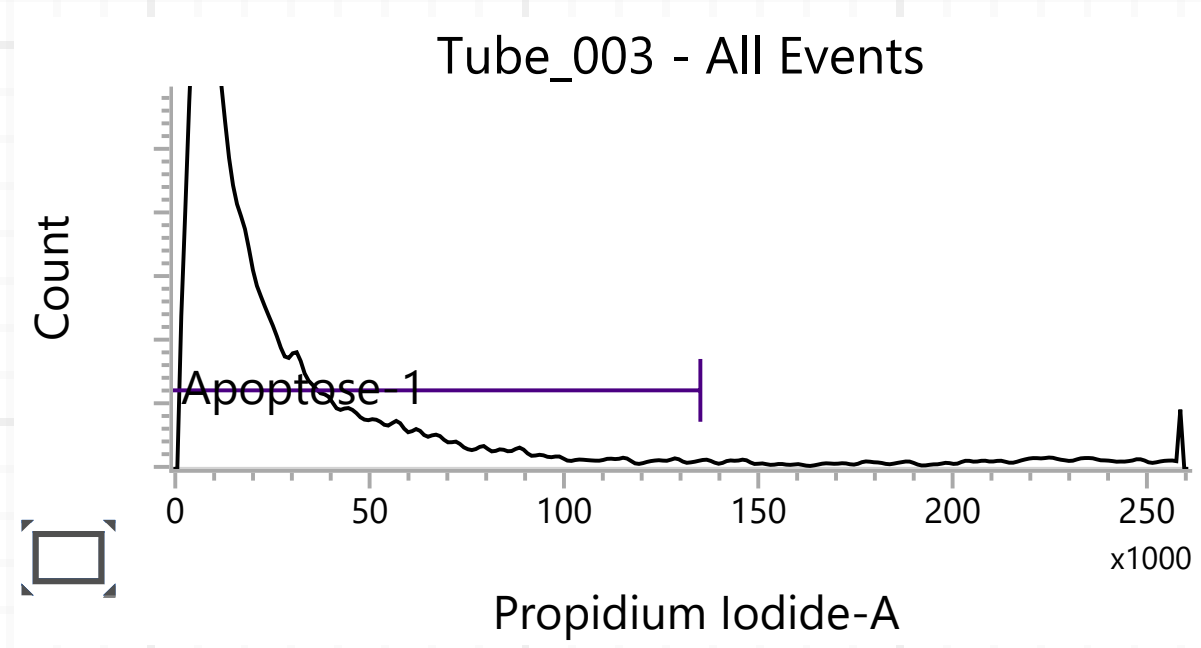

Statistics

Prozent apoptotische Zellen: Prozent apoptotische Zellen No Value % von Cells

Name	Events	% Total	% Parent	% Grandparent
Tube_001:All Events	10,981	100.00	***	***
Tube_001:Cells	10,000	91.07	91.07	***
Tube_002:All Events	10,000	100.00	***	***
Tube_002:Cells	9,169	91.69	91.69	***
Tube_003:All Events	10,000	100.00	***	***
Tube_003:Cells	9,146	91.46	91.46	***
Tube_001:Apoptose-1	9,672	88.08	88.08	***
Tube_002:Apoptose-1	8,736	87.36	87.36	***
Tube_003:Apoptose-1	8,834	88.34	88.34	***

DNA Fragmentierung

Sample ID: N6-D

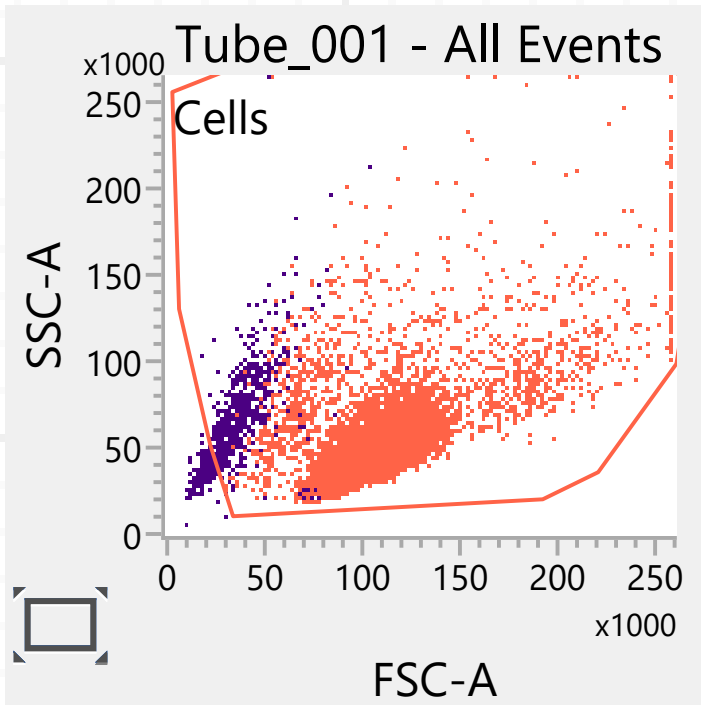

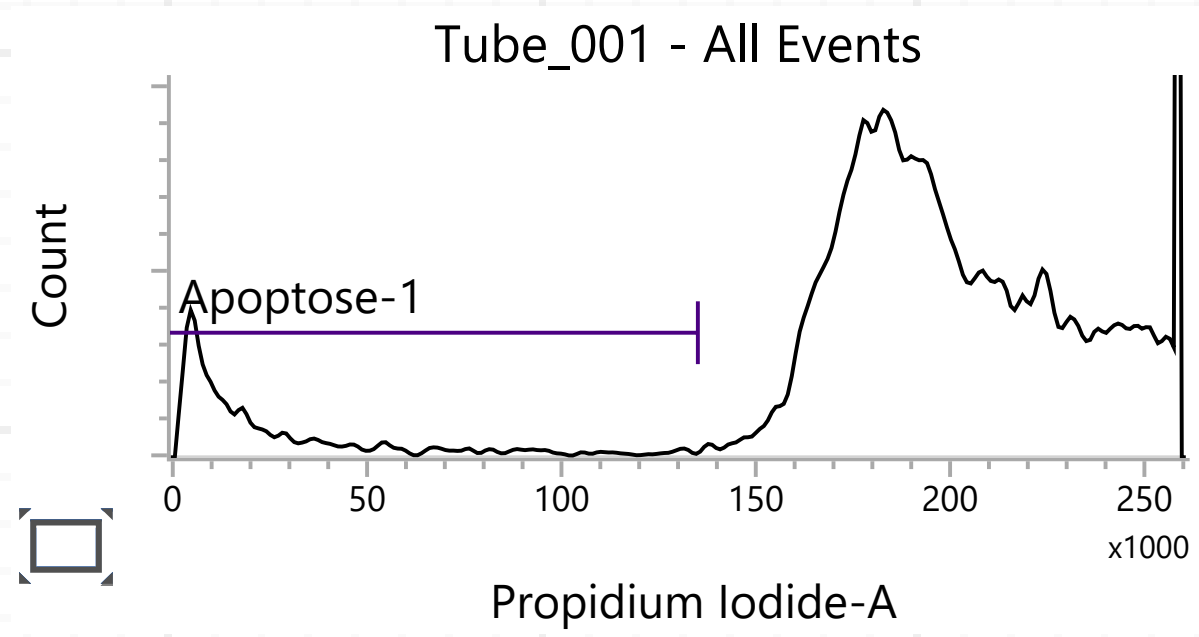

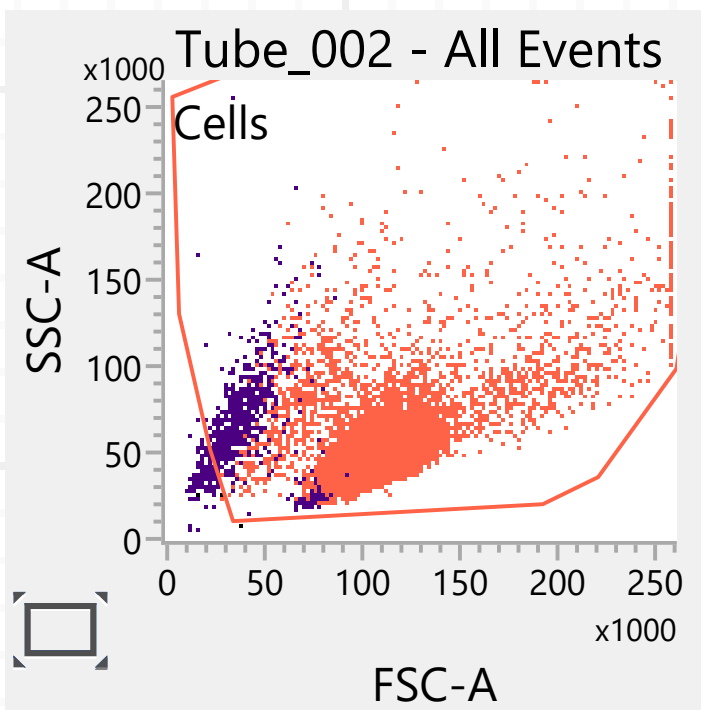

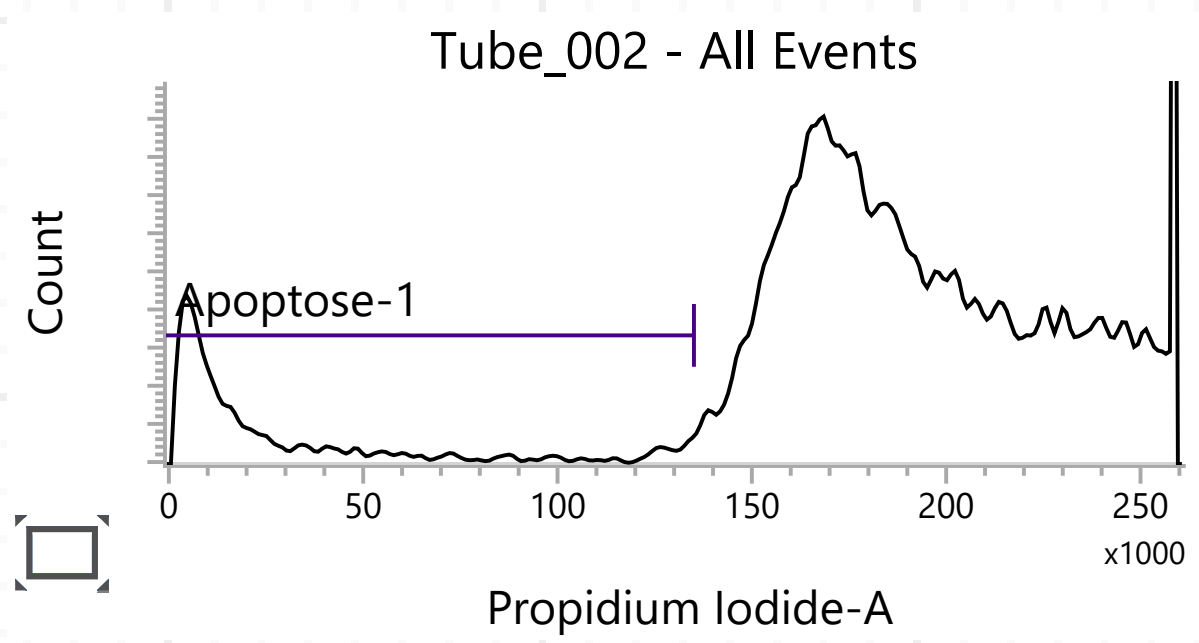

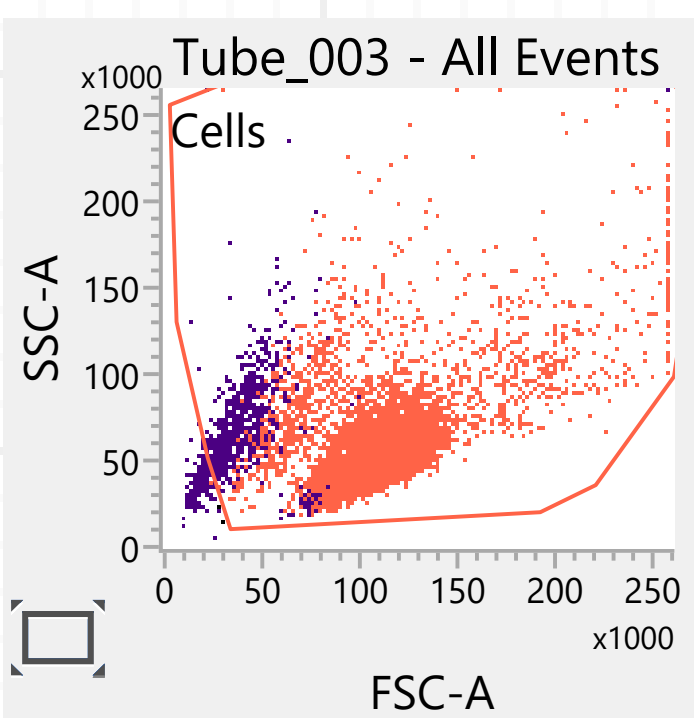

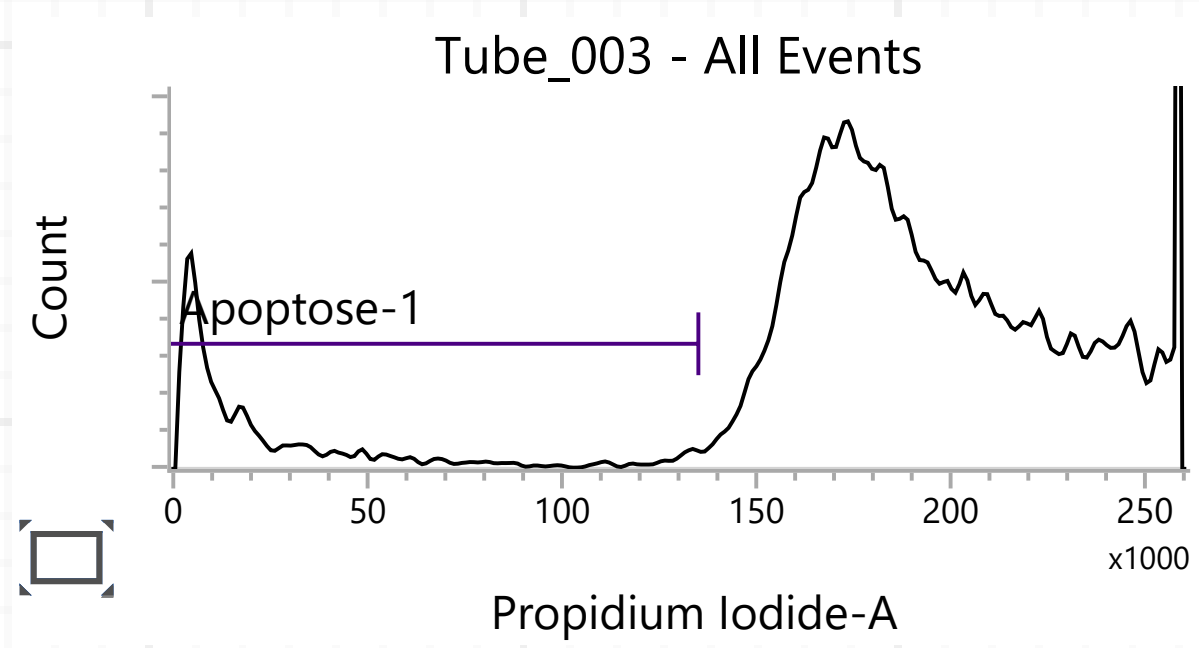

Statistics

Prozent apoptotische Zellen: Prozent apoptotische Zellen No Value % von Cells

Name	Events	% Total	% Parent	% Grandparent
Tube_001:All Events	10,121	100.00	***	***
Tube_001:Cells	10,000	98.80	98.80	***
Tube_002:All Events	10,000	100.00	***	***
Tube_002:Cells	9,859	98.59	98.59	***
Tube_003:All Events	10,000	100.00	***	***
Tube_003:Cells	9,858	98.58	98.58	***
Tube_001:Apoptose-1	663	6.55	6.55	***
Tube_002:Apoptose-1	784	7.84	7.84	***
Tube_003:Apoptose-1	850	8.50	8.50	***

DNA Fragmentierung

Sample ID: N6-EtoGry

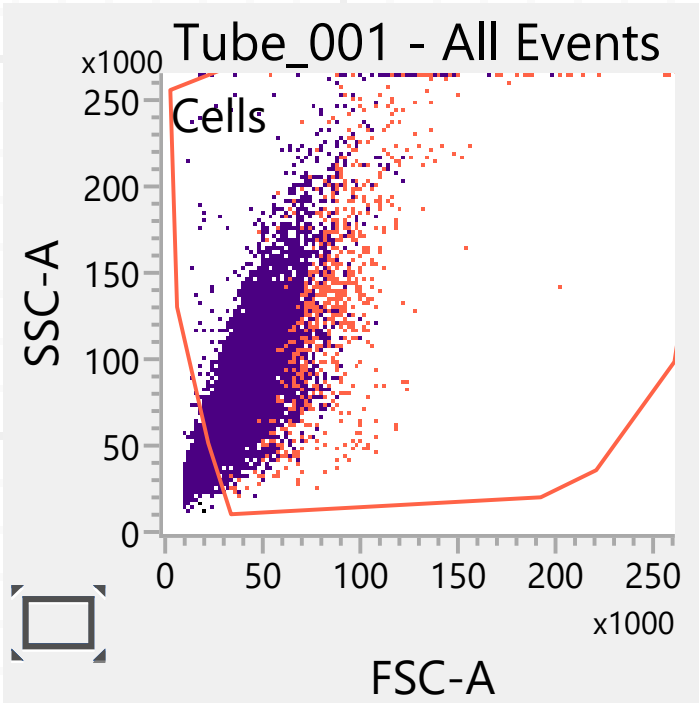

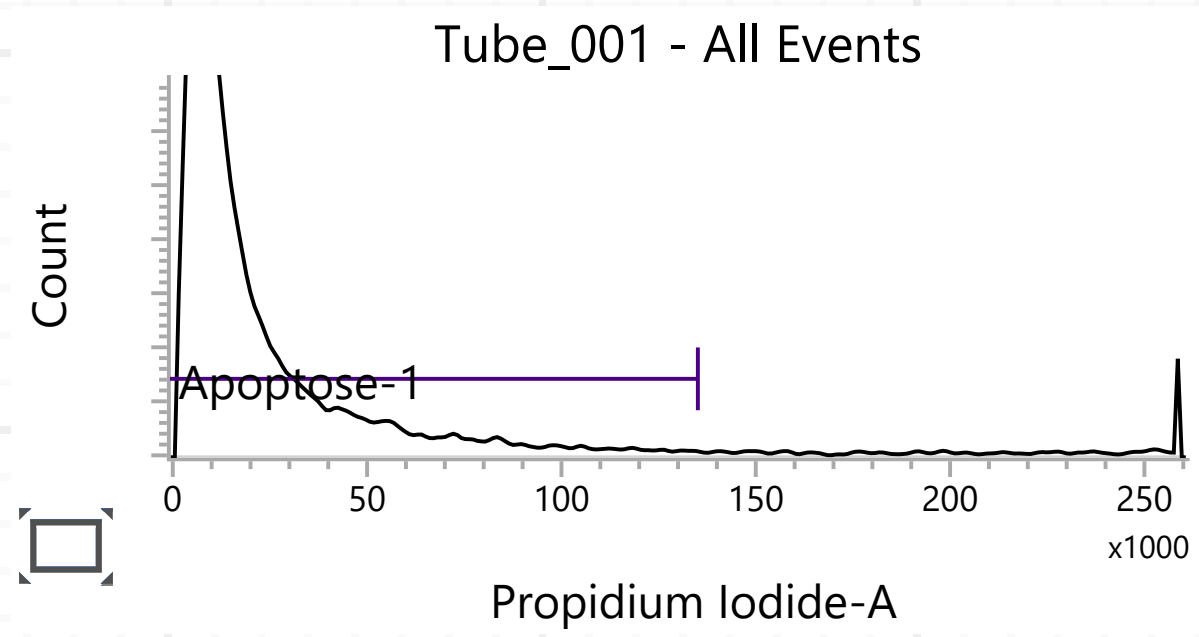

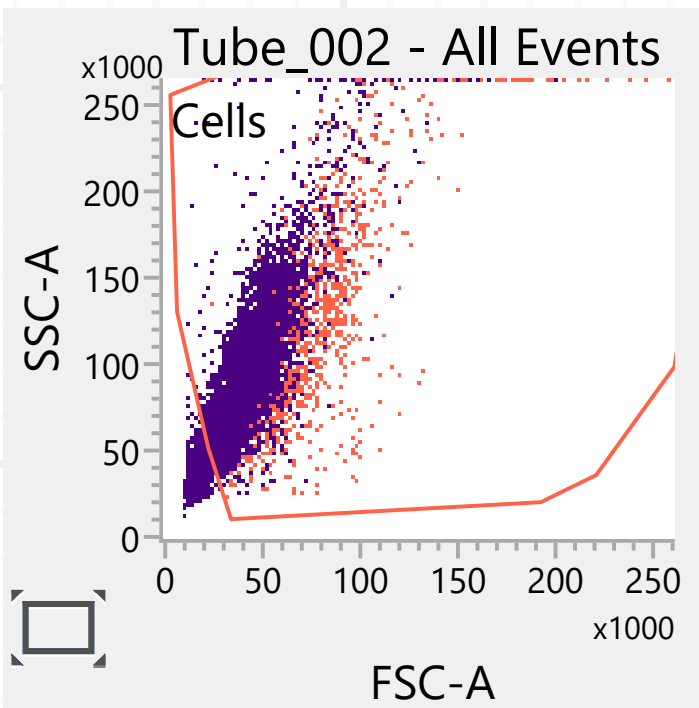

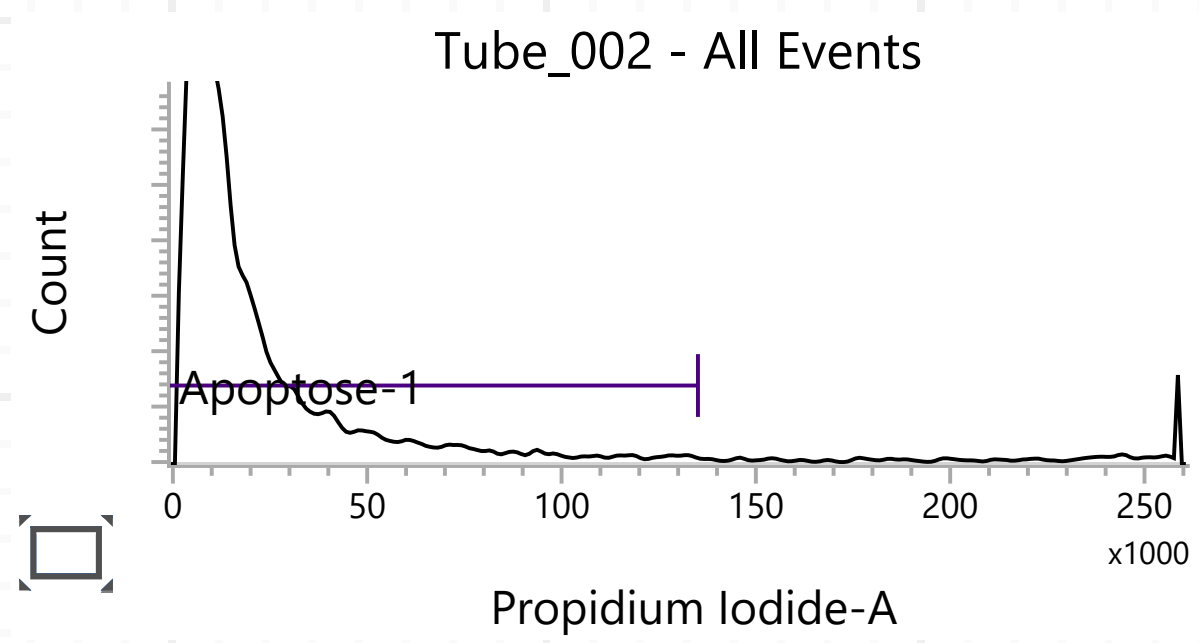

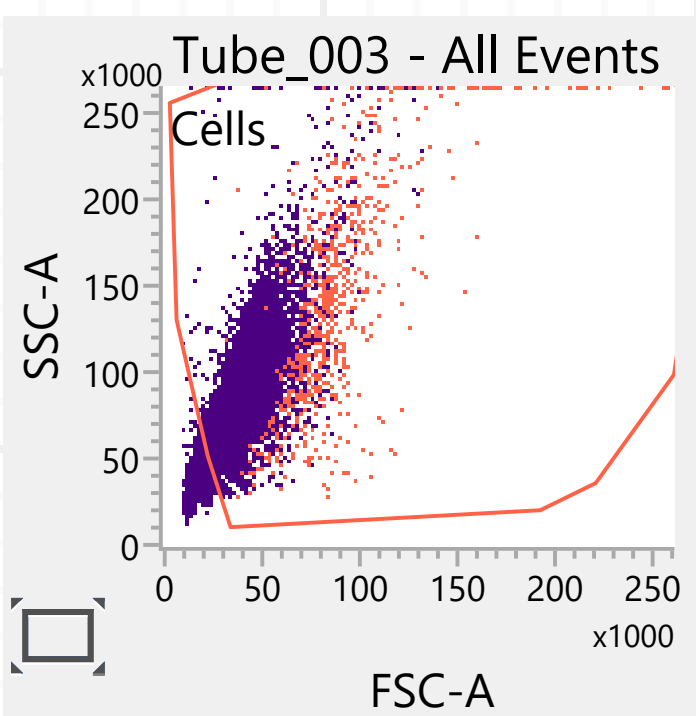

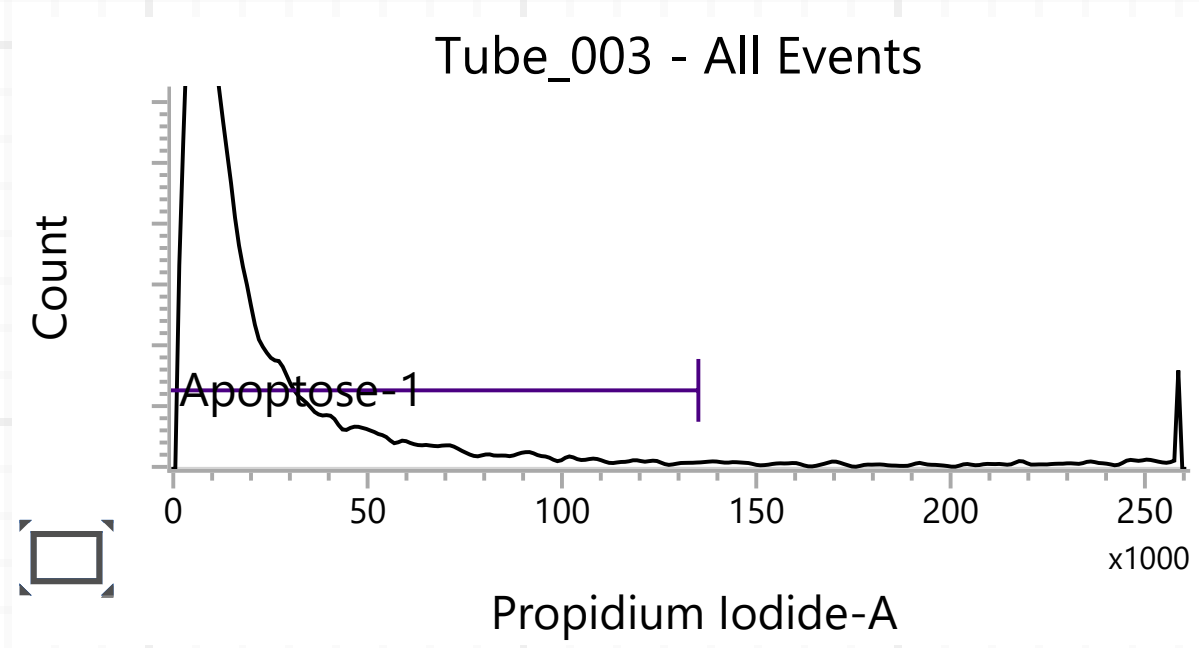

Statistics

Prozent apoptotische Zellen: Prozent apoptotische Zellen No Value % von Cells

Name	Events	% Total	% Parent	% Grandparent
Tube_001:All Events	11,128	100.00	***	***
Tube_001:Cells	10,000	89.86	89.86	***
Tube_002:All Events	10,000	100.00	***	***
Tube_002:Cells	8,989	89.89	89.89	***
Tube_003:All Events	10,000	100.00	***	***
Tube_003:Cells	8,908	89.08	89.08	***
Tube_001:Apoptose-1	9,562	85.93	85.93	***
Tube_002:Apoptose-1	8,582	85.82	85.82	***
Tube_003:Apoptose-1	8,592	85.92	85.92	***

DNA Fragmentierung

Sample ID: N6-EtoNeu

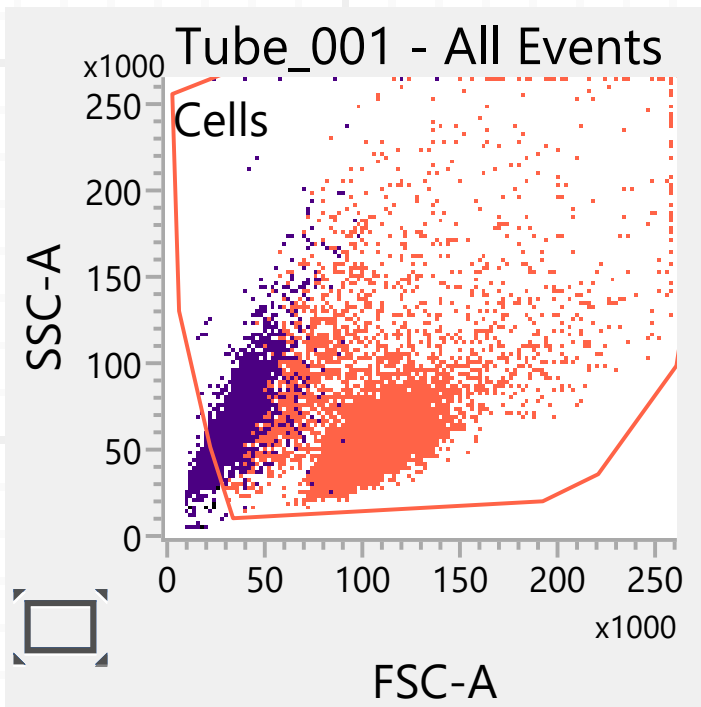

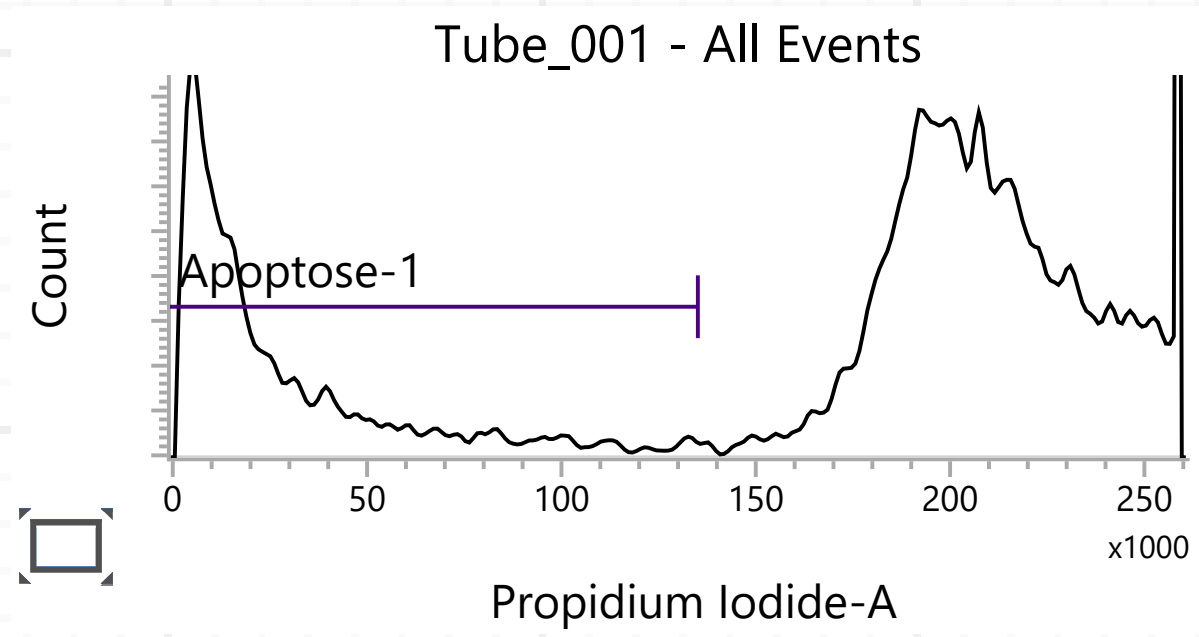

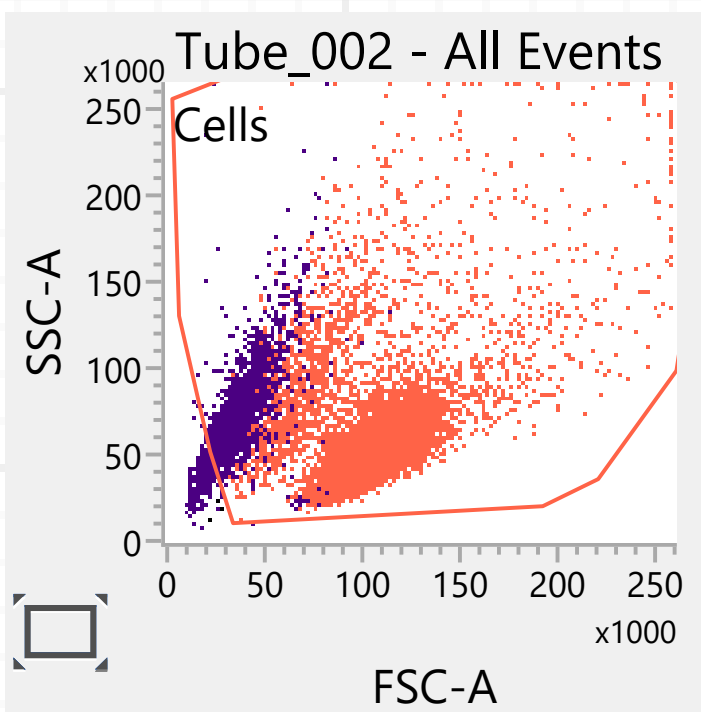

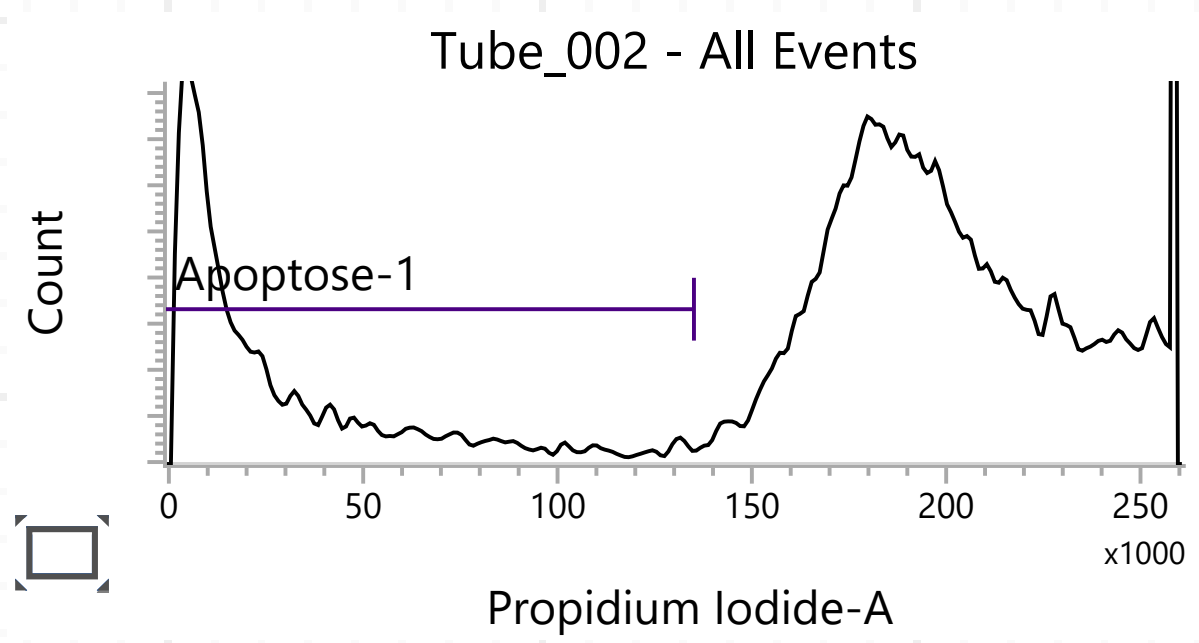

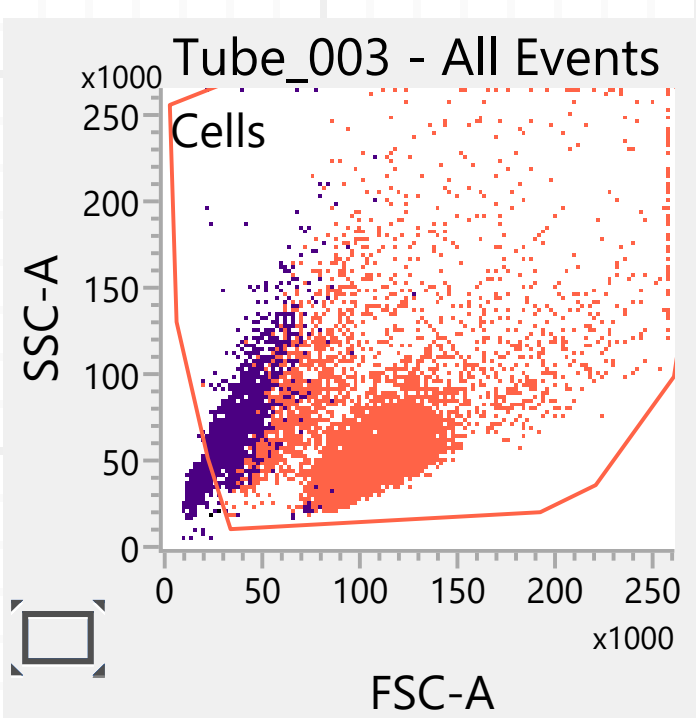

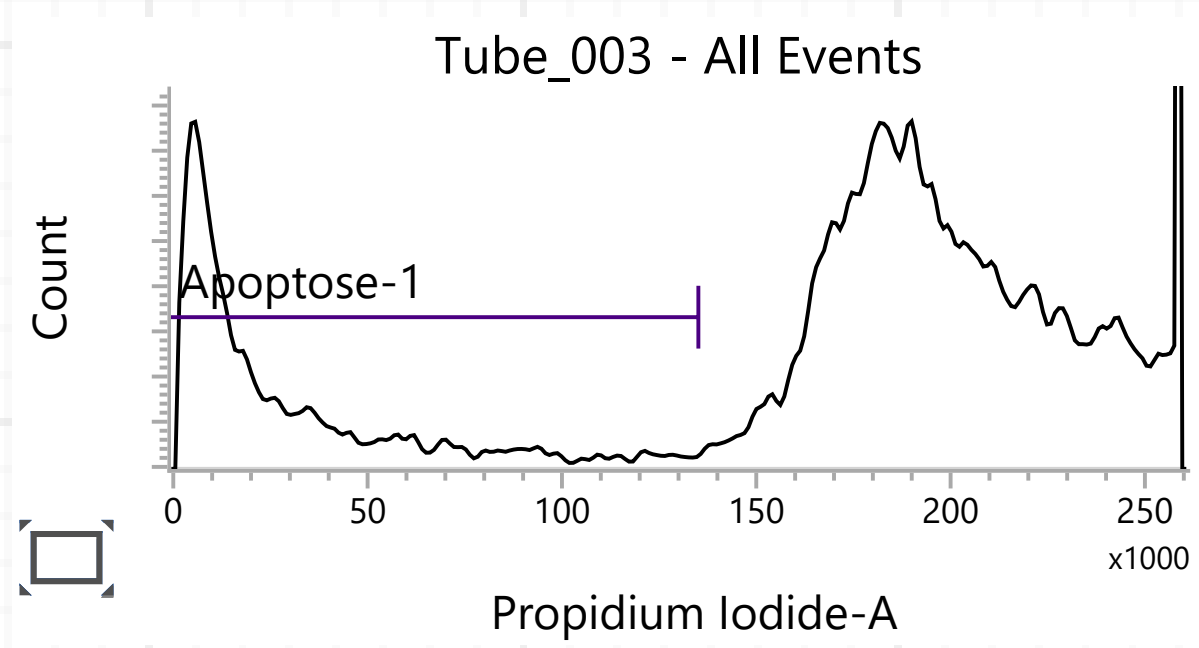

Statistics

Prozent apoptotische Zellen: Prozent apoptotische Zellen No Value % von Cells

Name	Events	% Total	% Parent	% Grandparent
Tube_001:All Events	10,317	100.00	***	***
Tube_001:Cells	10,000	96.93	96.93	***
Tube_002:All Events	10,000	100.00	***	***
Tube_002:Cells	9,707	97.07	97.07	***
Tube_003:All Events	10,000	100.00	***	***
Tube_003:Cells	9,723	97.23	97.23	***
Tube_001:Apoptose-1	1,891	18.33	18.33	***
Tube_002:Apoptose-1	1,829	18.29	18.29	***
Tube_003:Apoptose-1	1,583	15.83	15.83	***

DNA Fragmentierung

Sample ID: N6-K

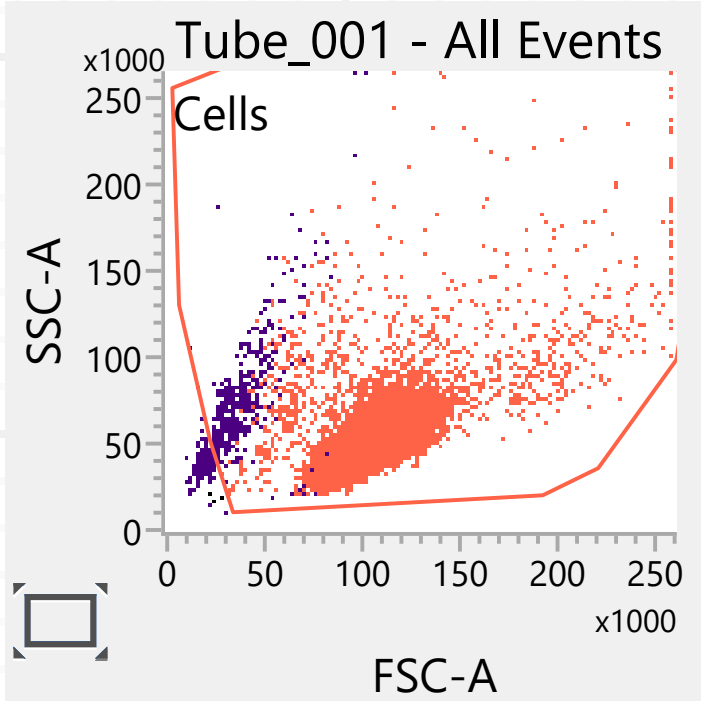

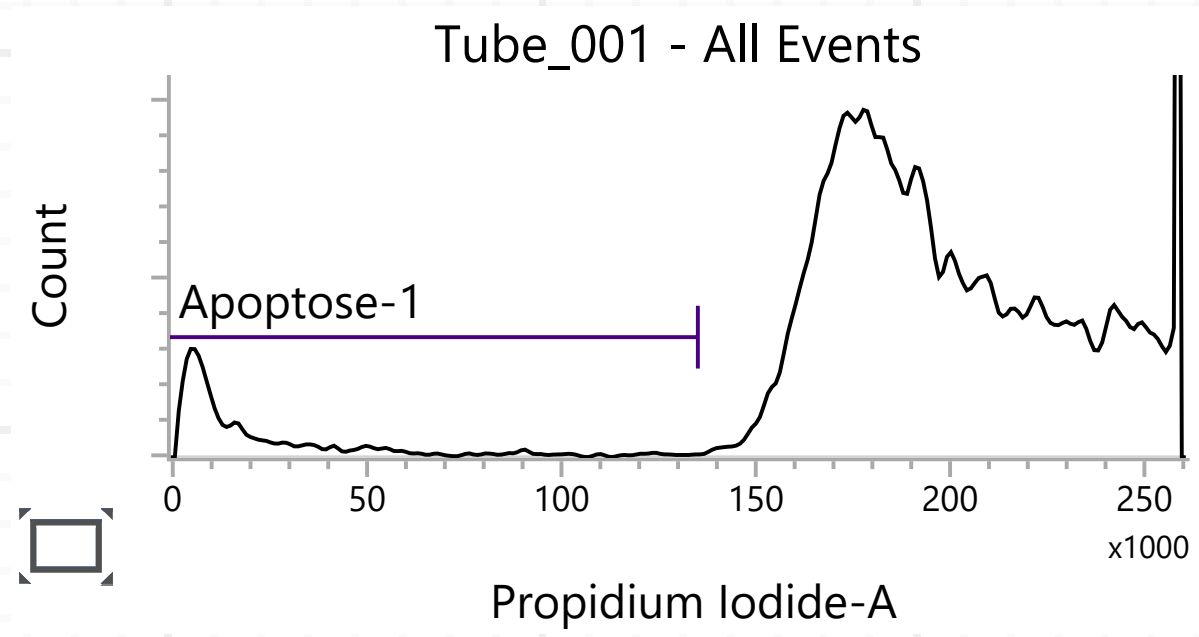

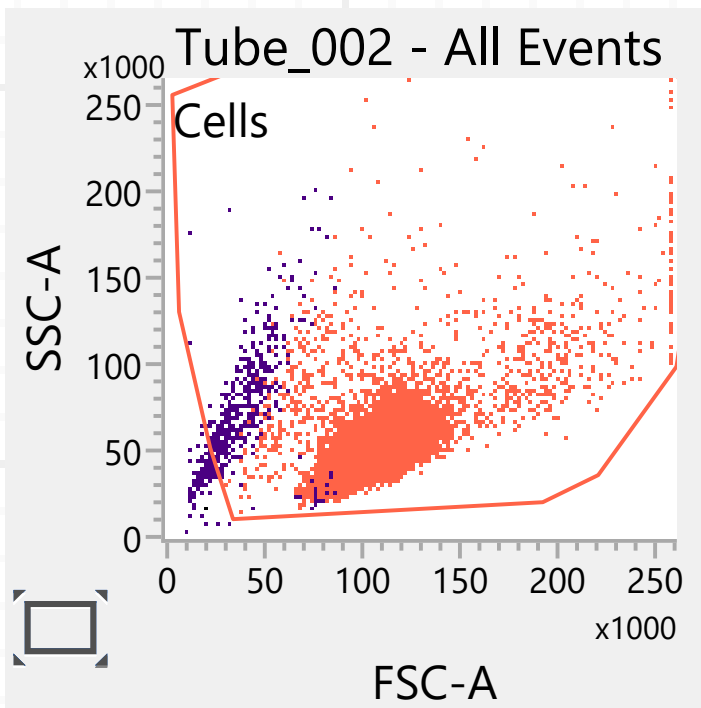

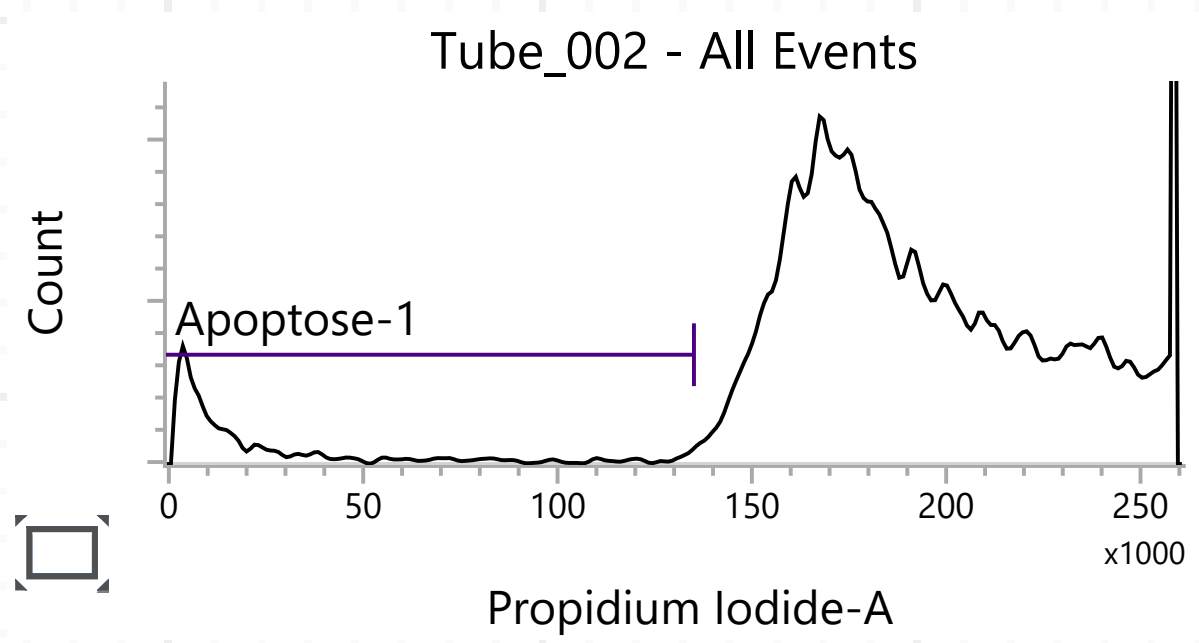

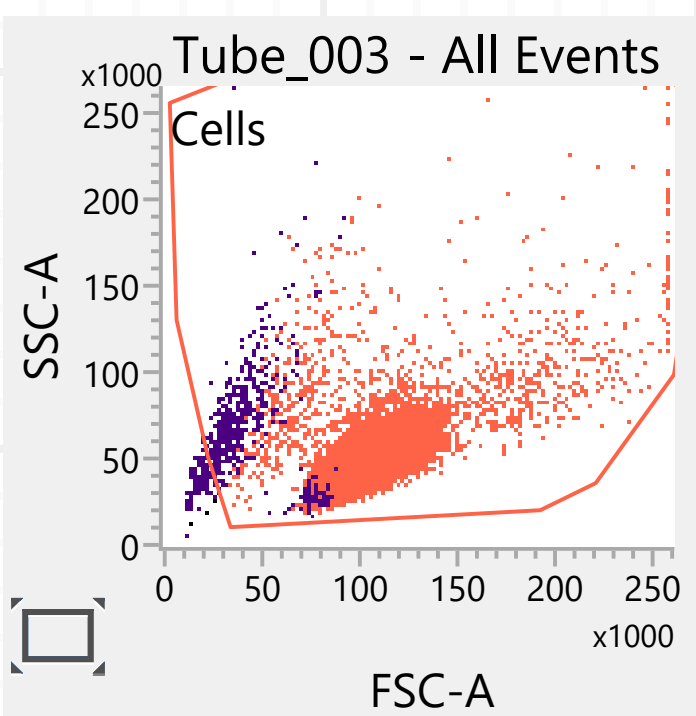

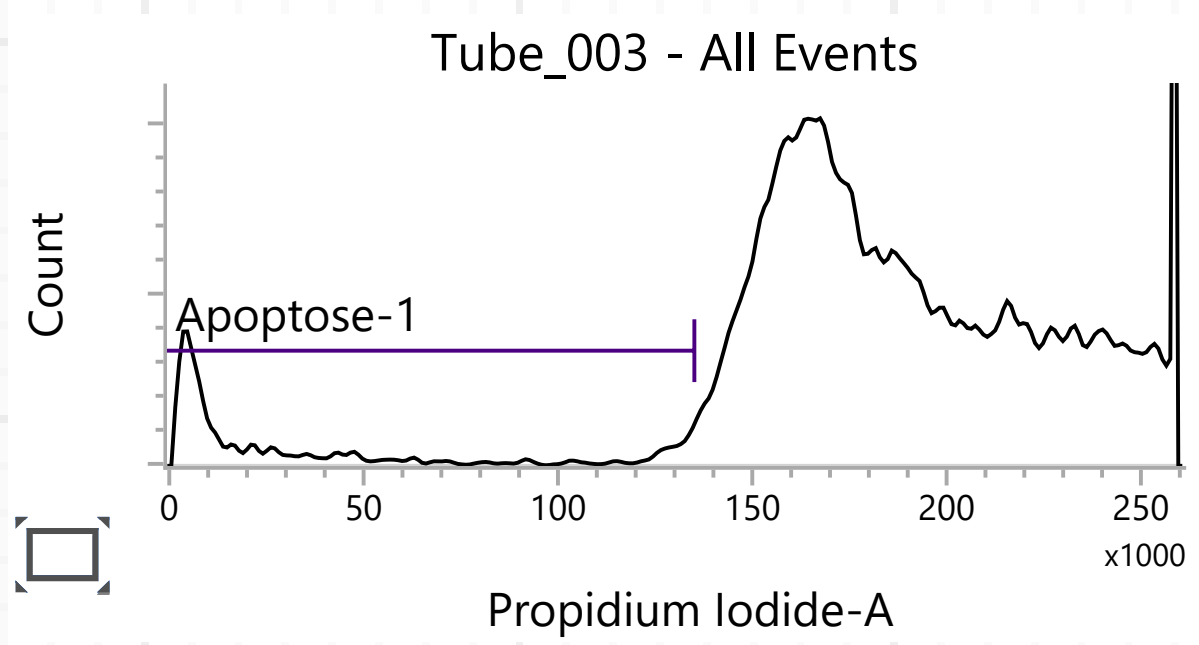

Statistics

Prozent apoptotische Zellen: Prozent apoptotische Zellen No Value % von Cells

Name	Events	% Total	% Parent	% Grandparent
Tube_001:All Events	10,097	100.00	***	***
Tube_001:Cells	10,000	99.04	99.04	***
Tube_002:All Events	10,000	100.00	***	***
Tube_002:Cells	9,904	99.04	99.04	***
Tube_003:All Events	10,000	100.00	***	***
Tube_003:Cells	9,891	98.91	98.91	***
Tube_001:Apoptose-1	499	4.94	4.94	***
Tube_002:Apoptose-1	520	5.20	5.20	***
Tube_003:Apoptose-1	572	5.72	5.72	***

DNA Fragmentierung

Sample ID: N6-Pred

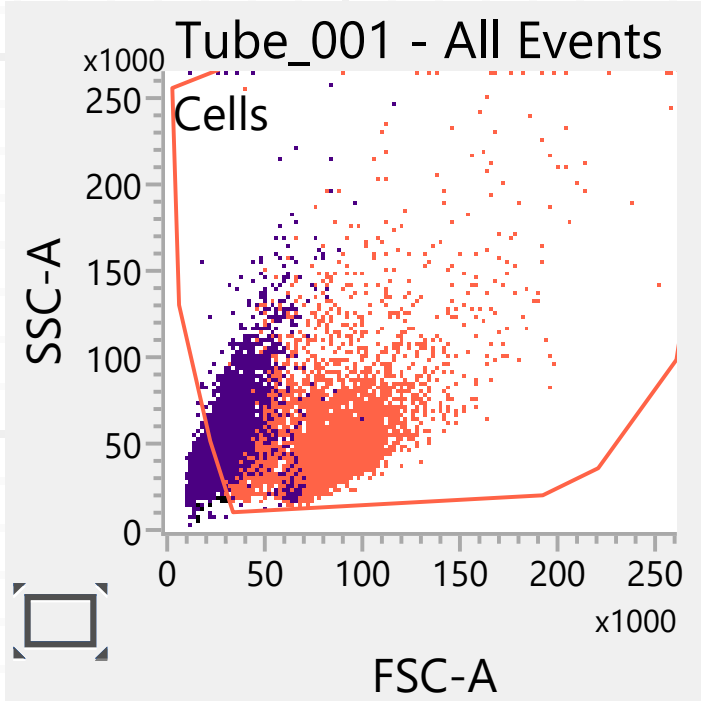

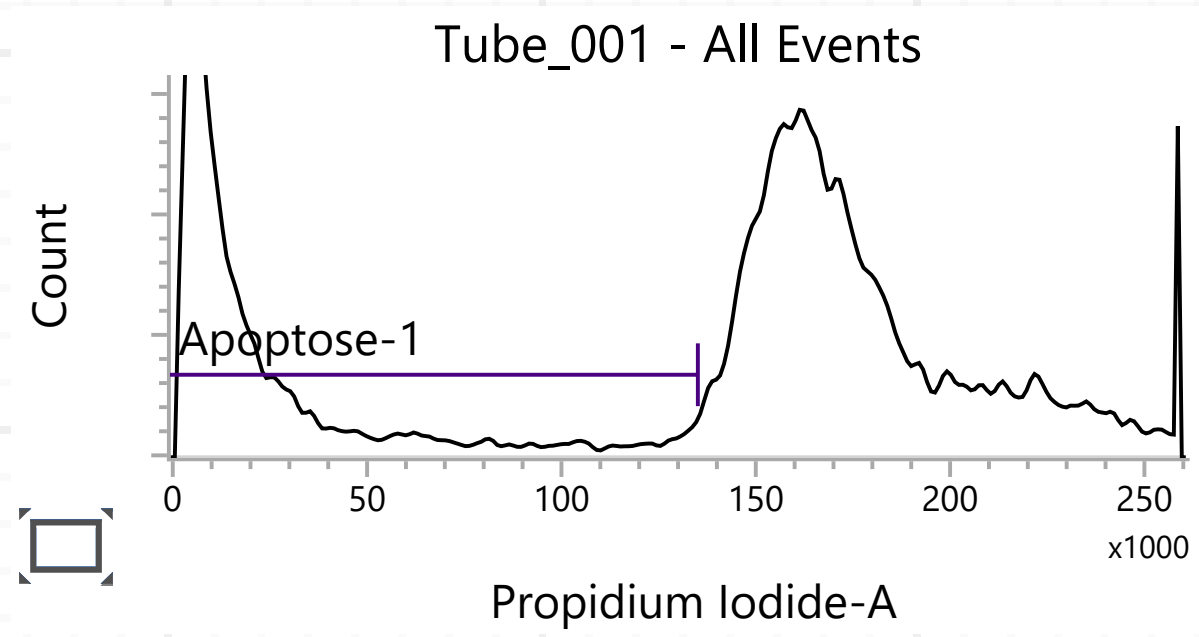

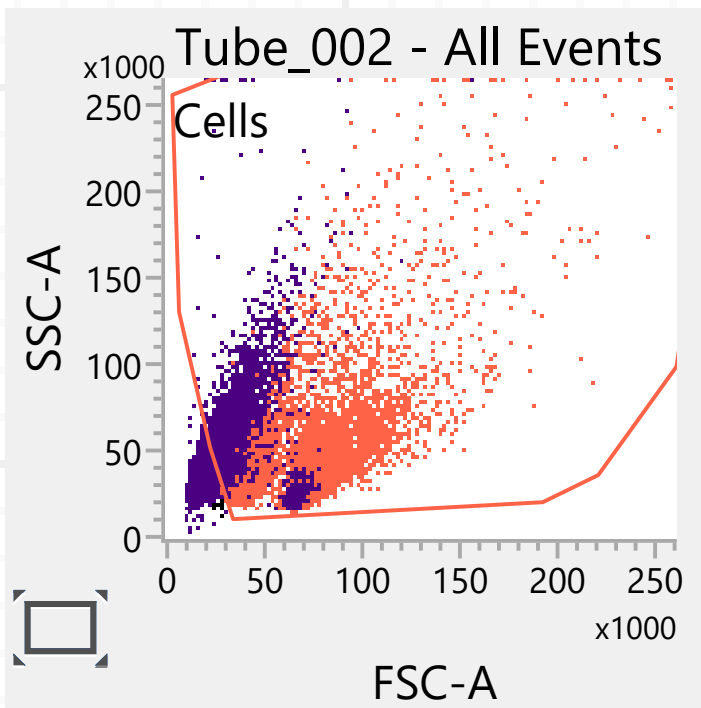

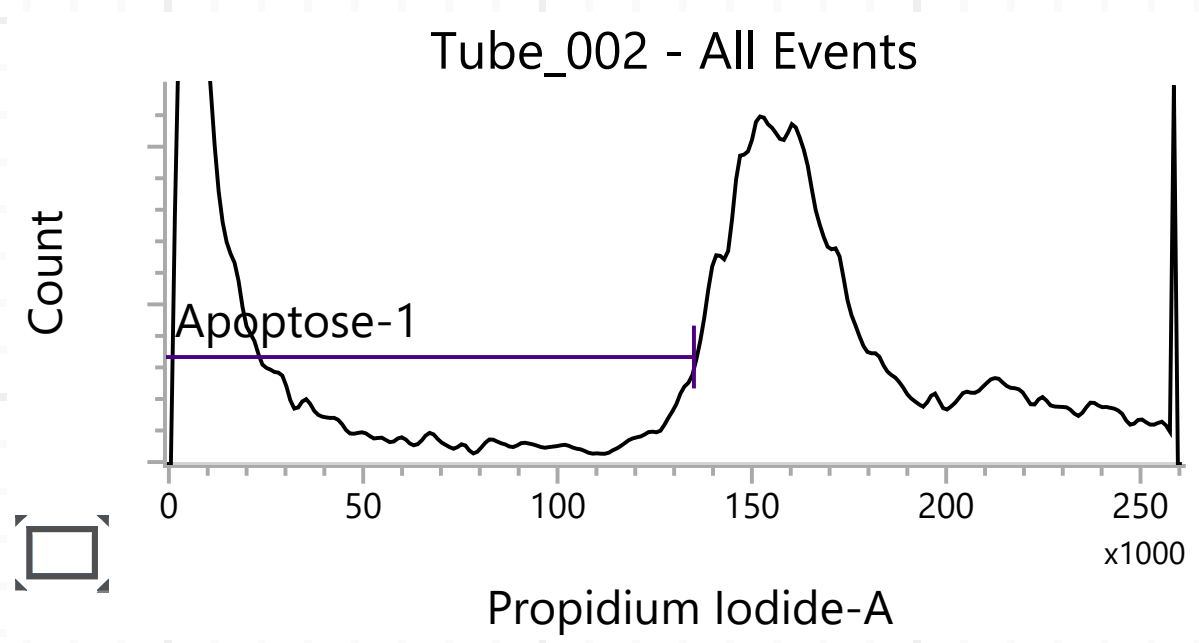

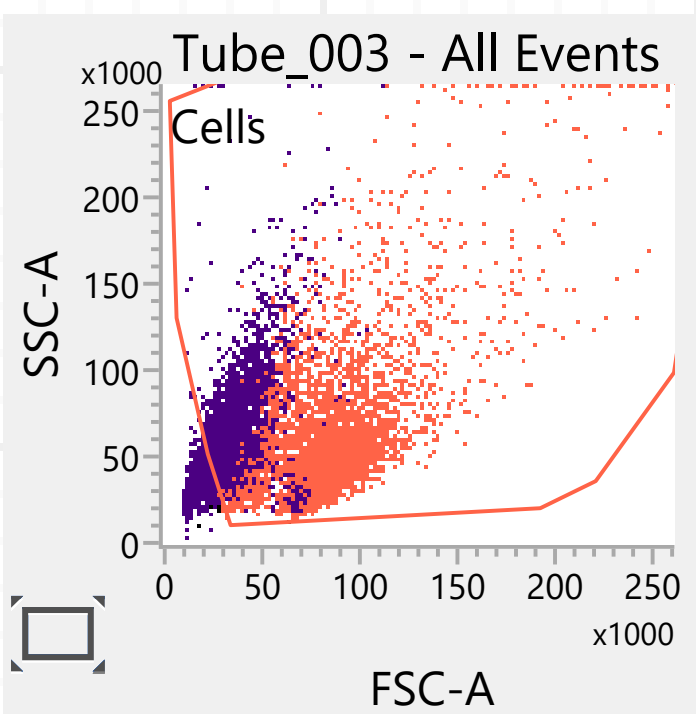

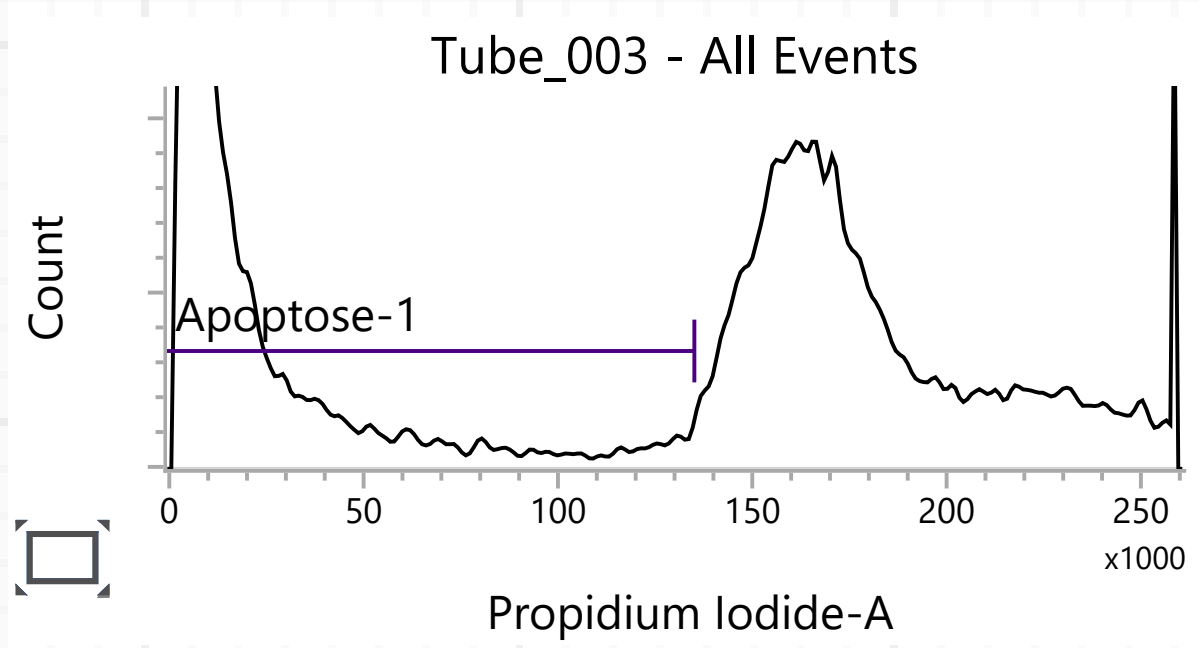

Statistics

Prozent apoptotische Zellen: Prozent apoptotische Zellen No Value % von Cells

Name	Events	% Total	% Parent	% Grandparent
Tube_001:All Events	11,434	100.00	***	***
Tube_001:Cells	10,000	87.46	87.46	***
Tube_002:All Events	10,000	100.00	***	***
Tube_002:Cells	8,641	86.41	86.41	***
Tube_003:All Events	10,000	100.00	***	***
Tube_003:Cells	8,684	86.84	86.84	***
Tube_001:Apoptose-1	3,487	30.50	30.50	***
Tube_002:Apoptose-1	3,595	35.95	35.95	***
Tube_003:Apoptose-1	3,730	37.30	37.30	***

DNA Fragmentierung

Sample ID: N6-Pred

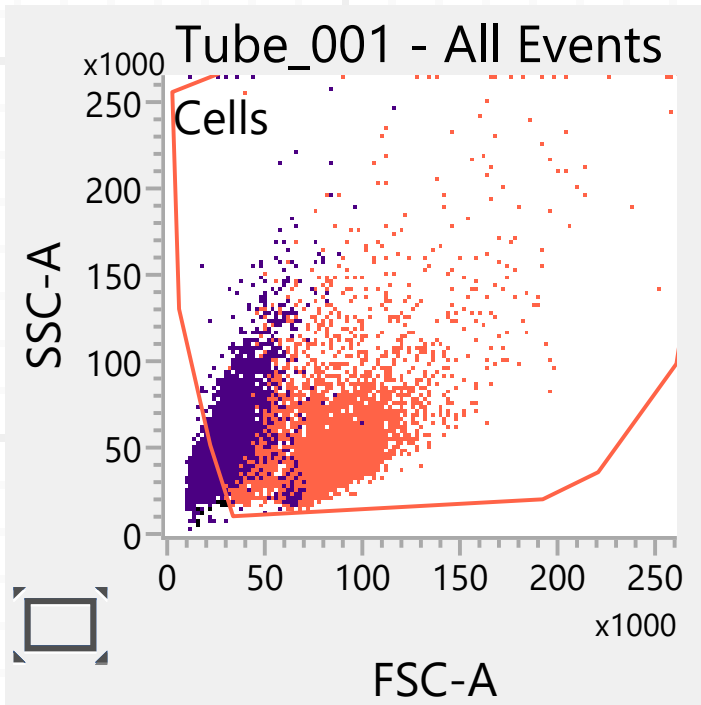

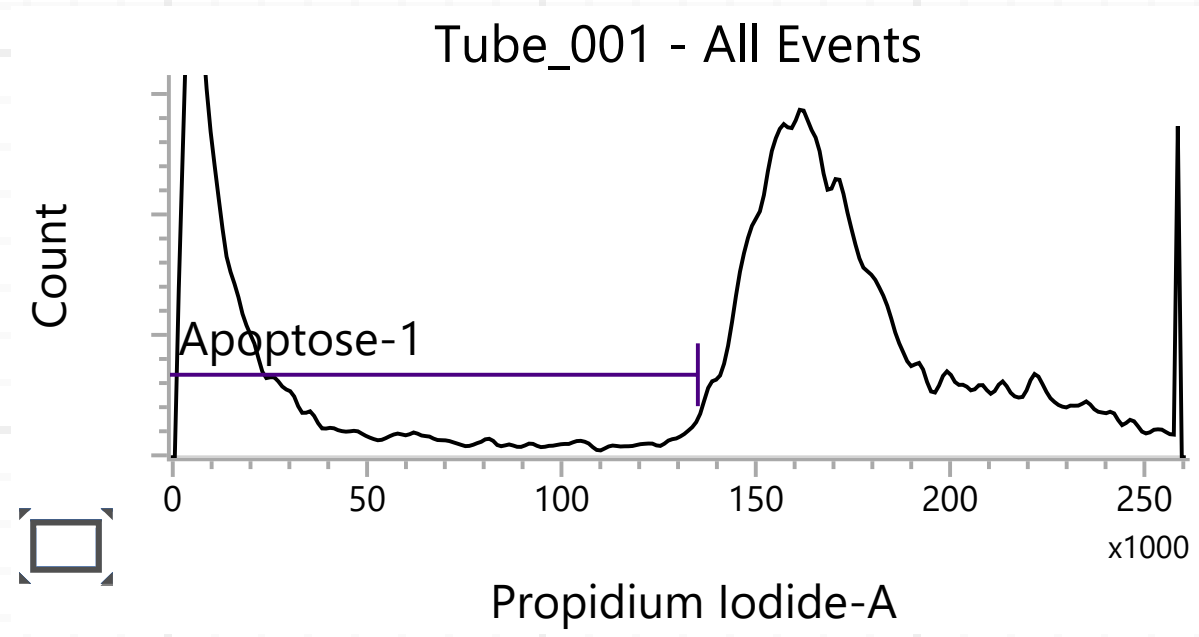

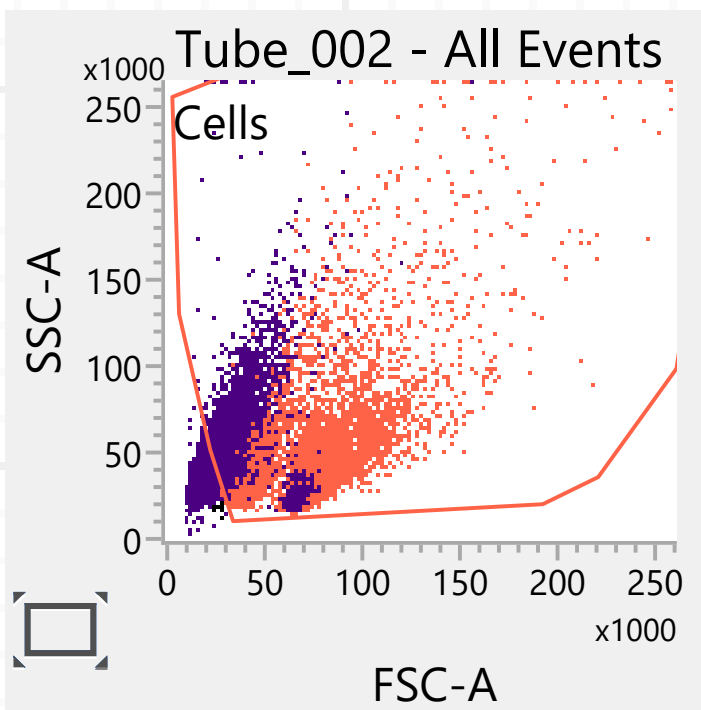

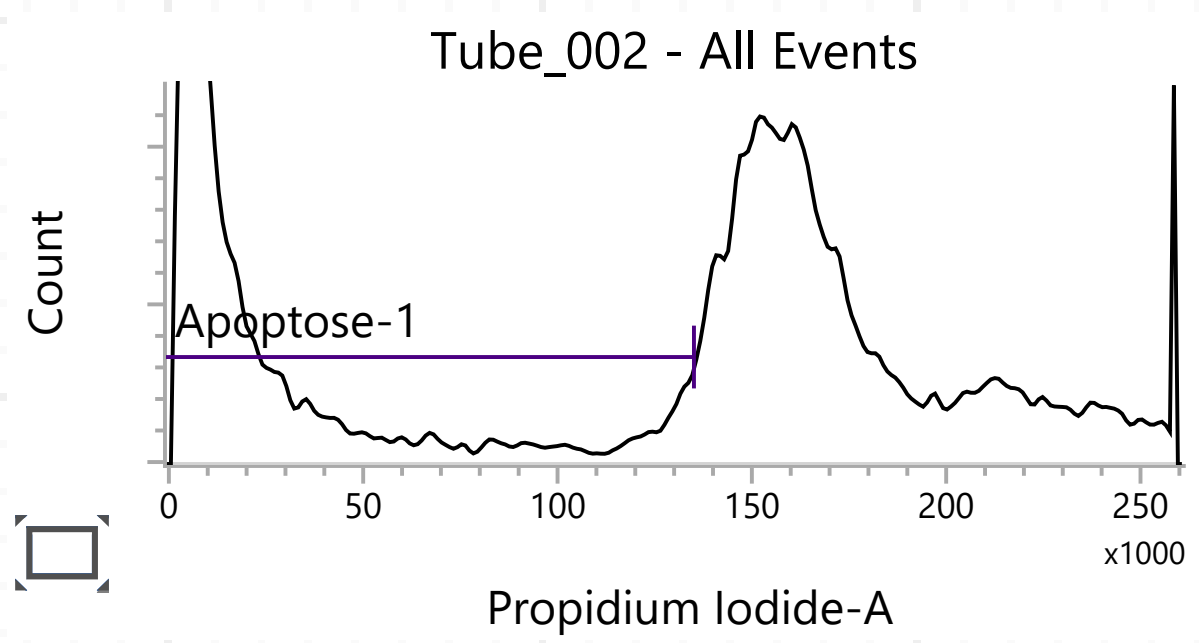

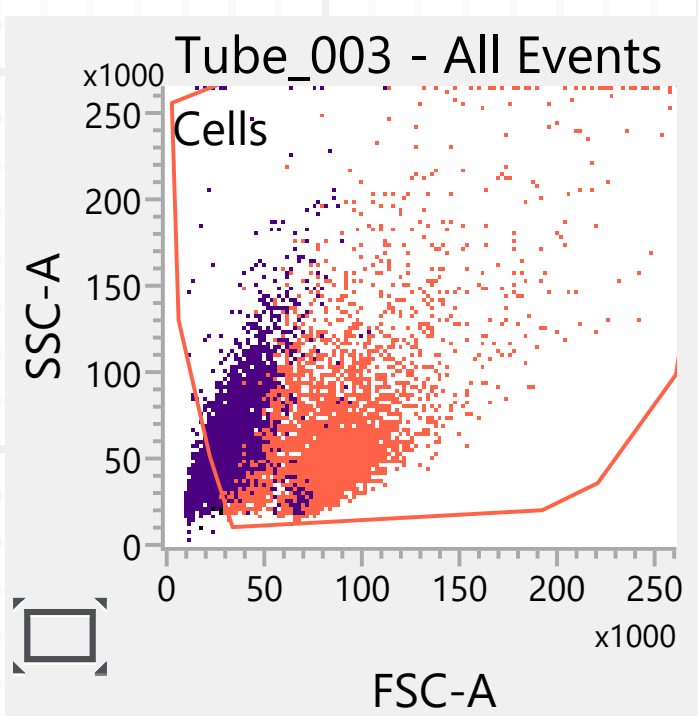

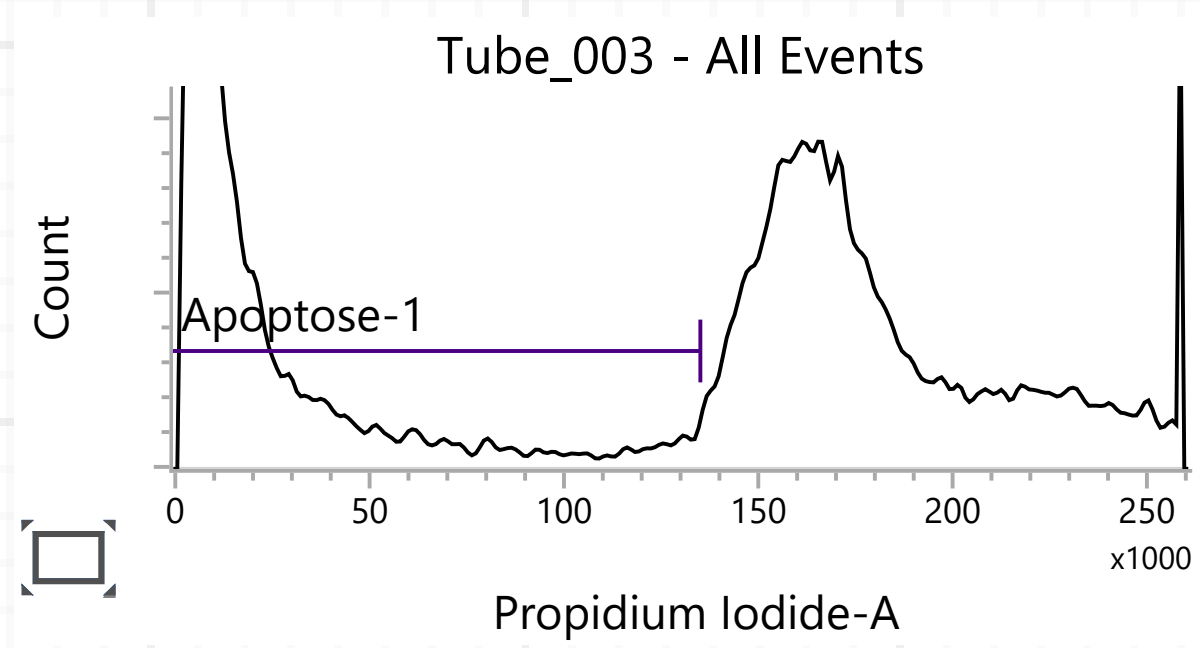

Statistics

Prozent apoptotische Zellen: Prozent apoptotische Zellen No Value % von Cells

Name	Events	% Total	% Parent	% Grandparent	
Tube_001:All Events	11,434	100.00	***	***	
Tube_001:Cells	10,000	87.46	87.46	***	
Tube_002:All Events	10,000	100.00	***	***	
Tube_002:Cells	8,641	86.41	86.41	***	
Tube_003:All Events	10,000	100.00	***	***	
Tube_003:Cells	8,684	86.84	86.84	***	
Tube_001:Apoptose-1	3,487	30.50	30.50	***	
Tube_002:Apoptose-1	3,595	35.95	35.95	***	
Tube_003:Apoptose-1	3,730	37.30	37.30	***	

DNA Fragmentierung

Sample ID: Naku 0,5

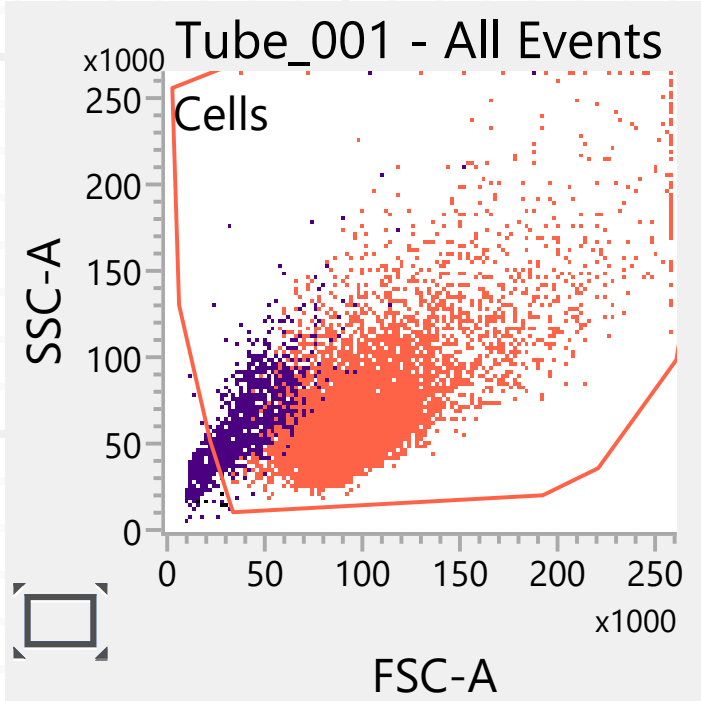

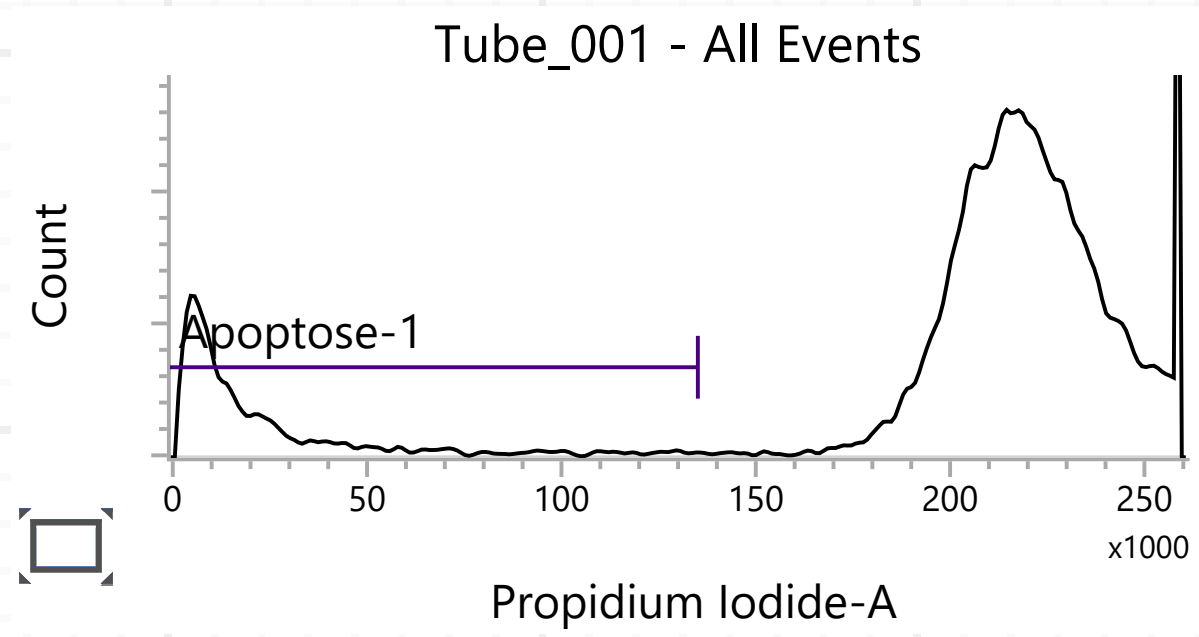

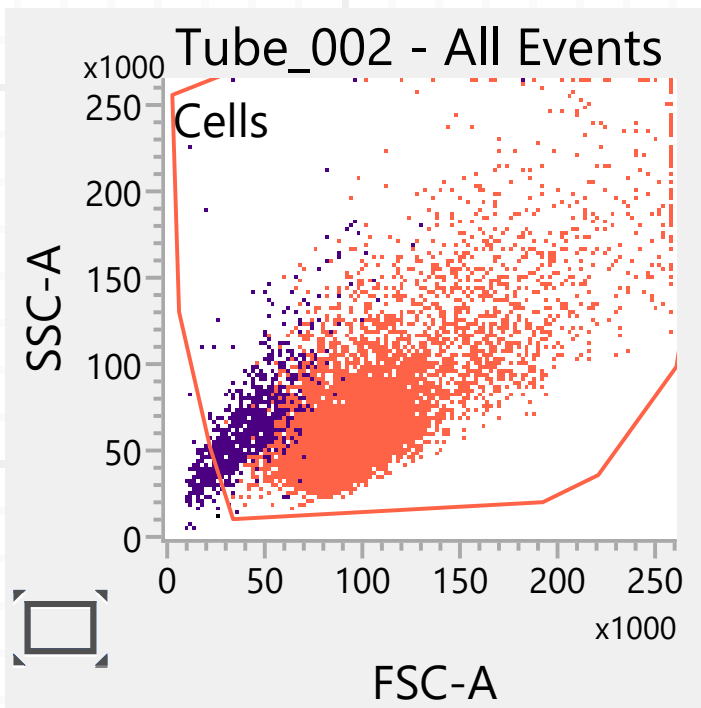

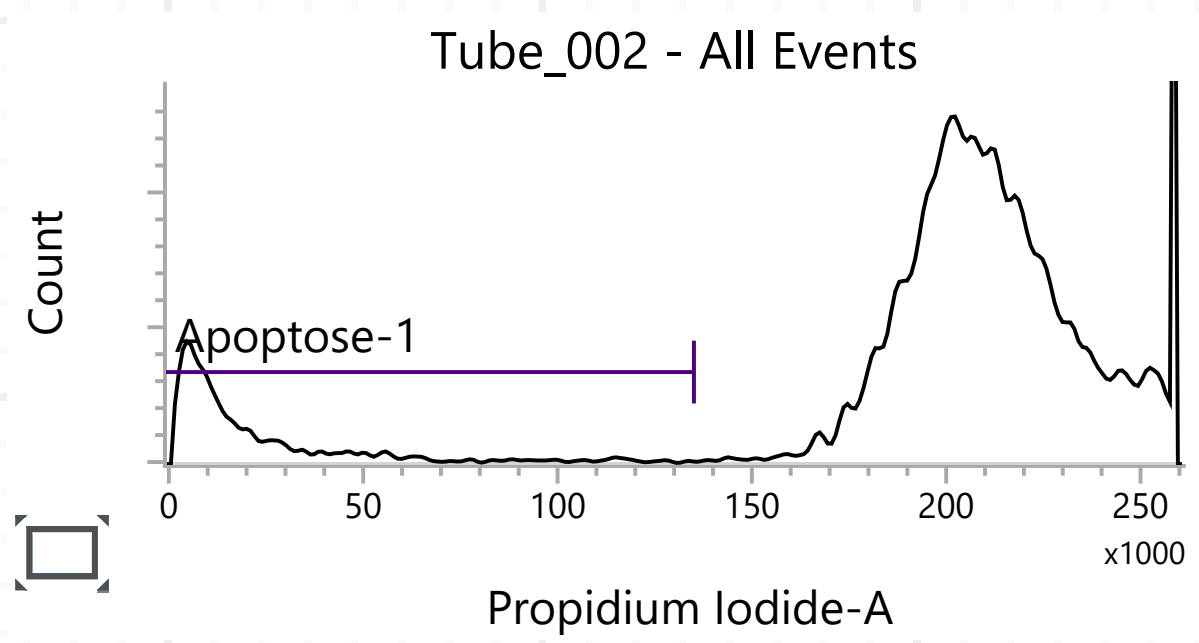

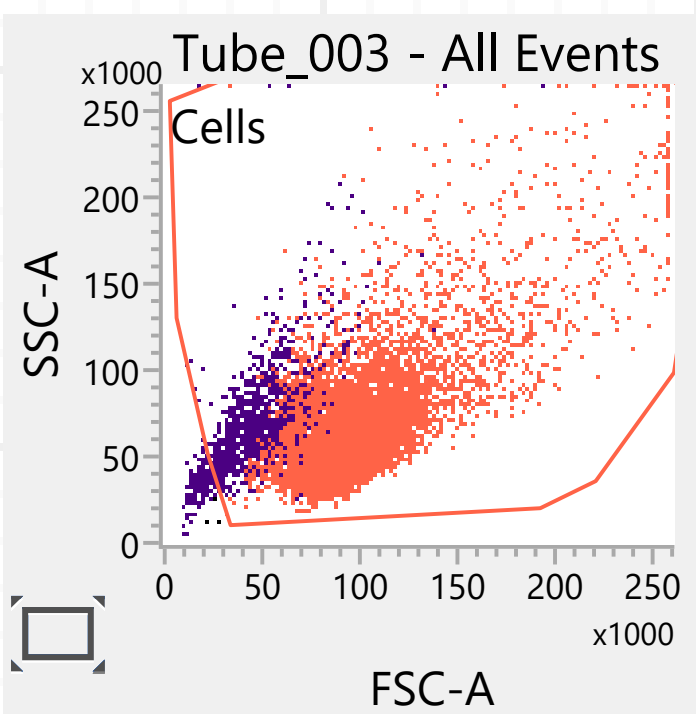

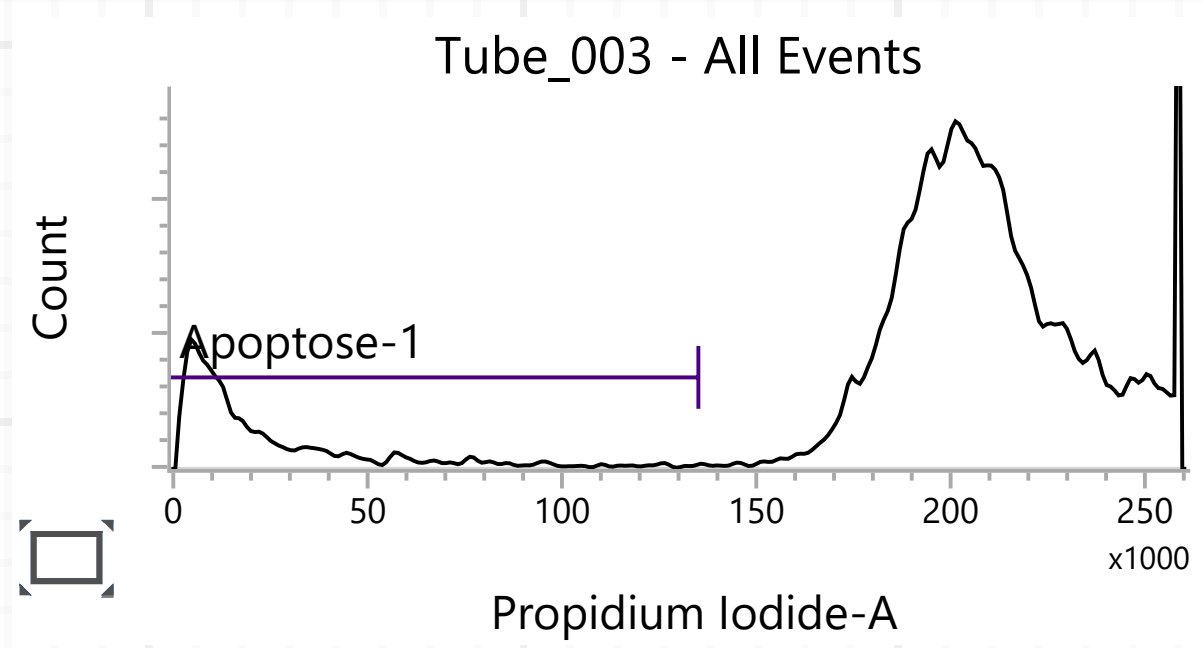

Statistics

Prozent apoptotische Zellen: Prozent apoptotische Zellen No Value % von Cells

Name	Events	% Total	% Parent	% Grandparent
Tube_001:All Events	10,215	100.00	***	***
Tube_001:Cells	10,000	97.90	97.90	***
Tube_002:All Events	10,000	100.00	***	***
Tube_002:Cells	9,862	98.62	98.62	***
Tube_003:All Events	10,000	100.00	***	***
Tube_003:Cells	9,869	98.69	98.69	***
Tube_001:Apoptose-1	1,110	10.87	10.87	***
Tube_002:Apoptose-1	845	8.45	8.45	***
Tube_003:Apoptose-1	990	9.90	9.90	***

DNA Fragmentierung

Sample ID: Naku 1

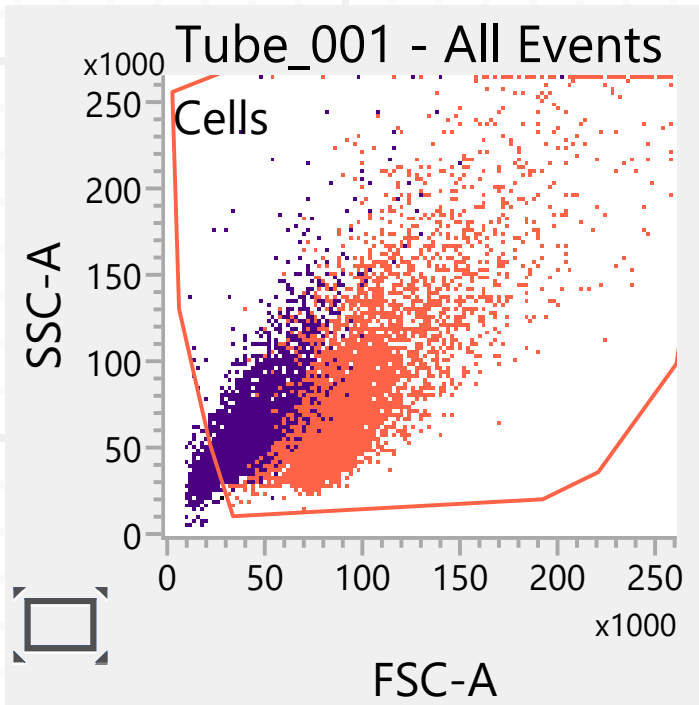

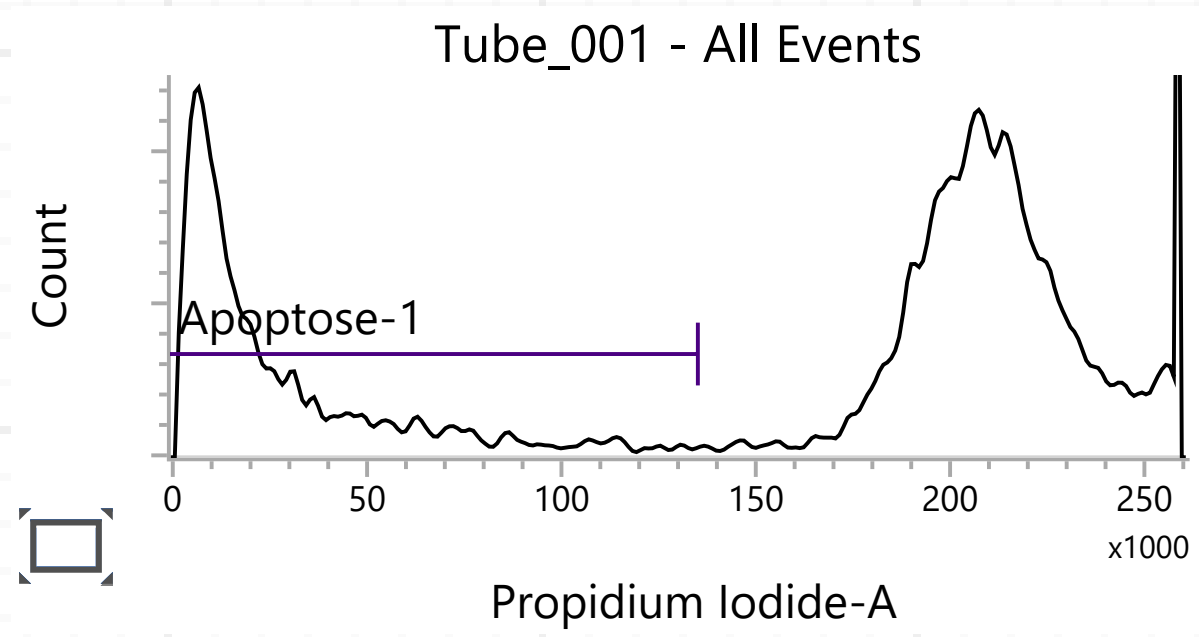

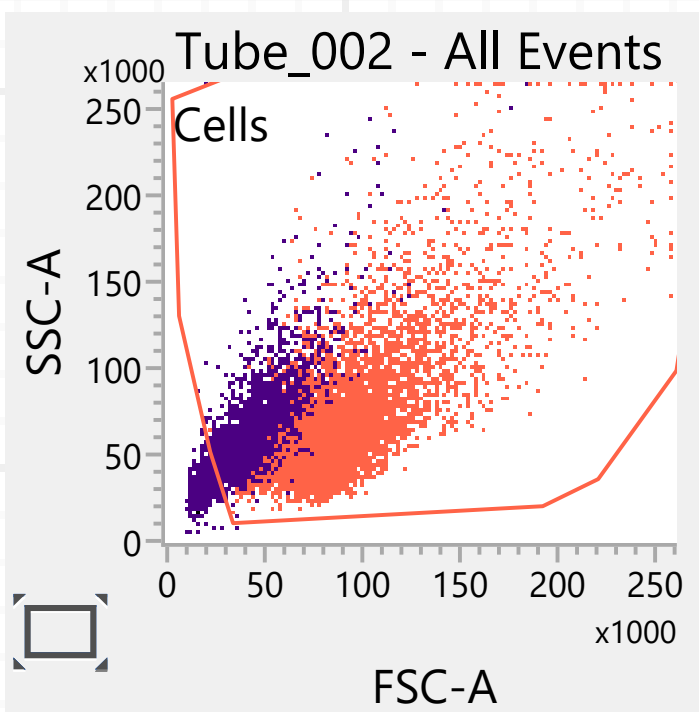

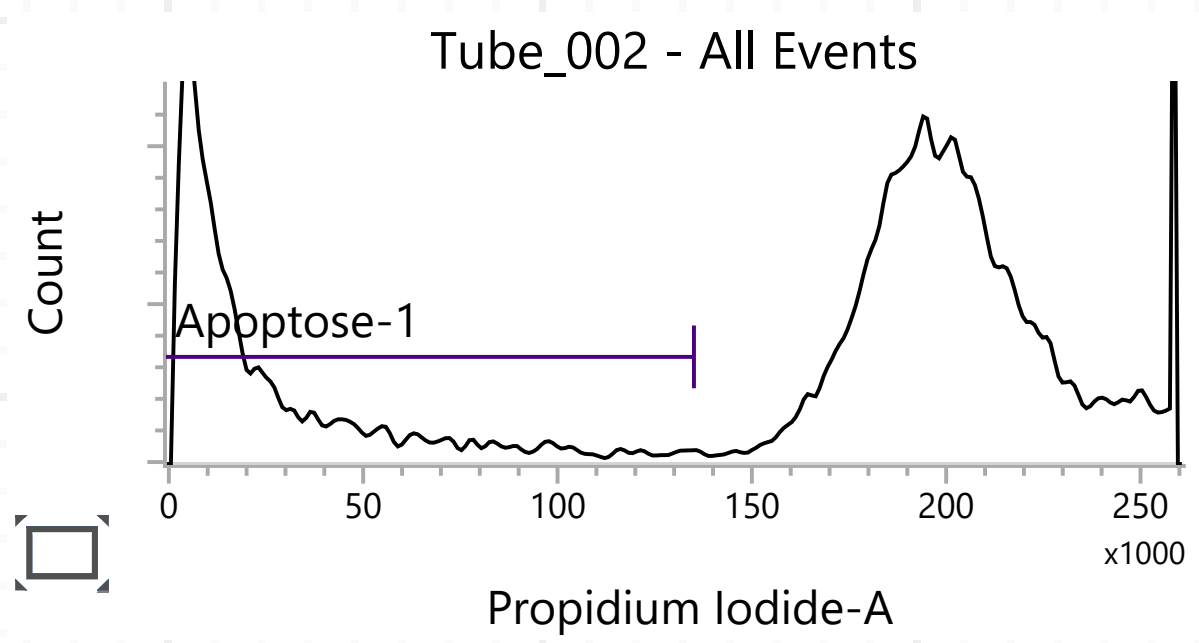

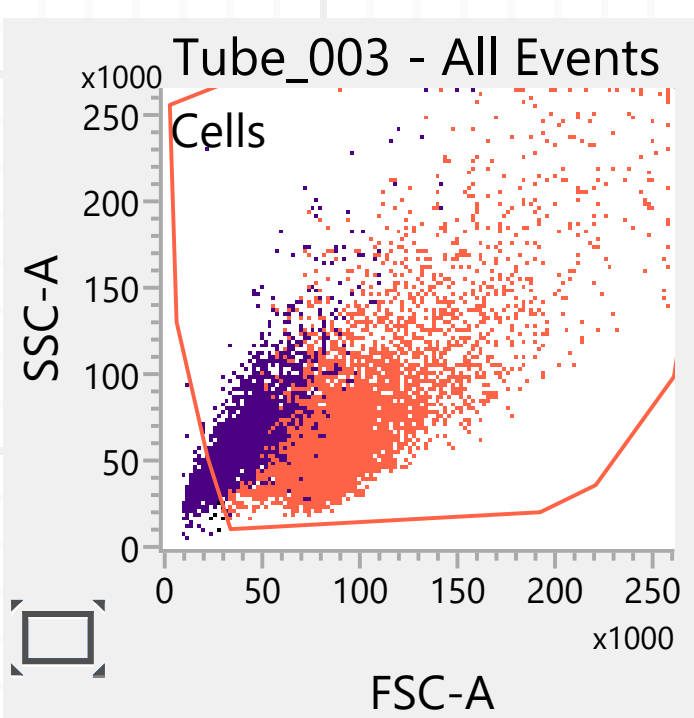

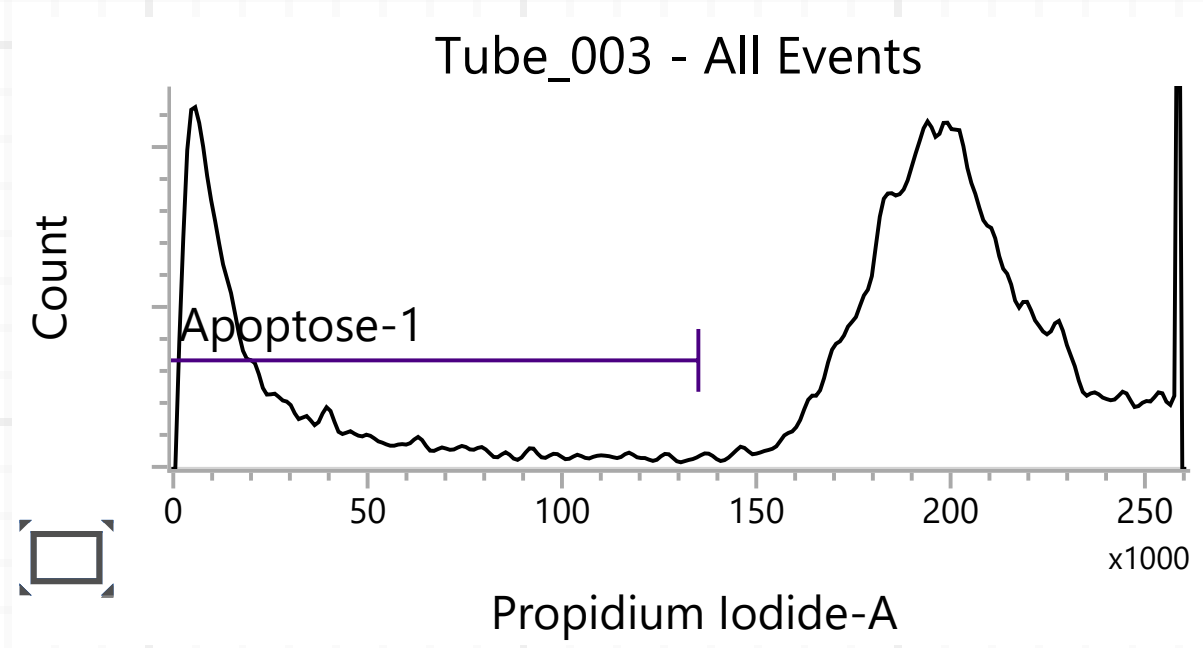

Statistics

Prozent apoptotische Zellen: Prozent apoptotische Zellen No Value % von Cells

Name	Events	% Total	% Parent	% Grandparent
Tube_001:All Events	10,405	100.00	***	***
Tube_001:Cells	10,000	96.11	96.11	***
Tube_002:All Events	10,000	100.00	***	***
Tube_002:Cells	9,519	95.19	95.19	***
Tube_003:All Events	10,000	100.00	***	***
Tube_003:Cells	9,603	96.03	96.03	***
Tube_001:Apoptose-1	2,662	25.58	25.58	***
Tube_002:Apoptose-1	2,566	25.66	25.66	***
Tube_003:Apoptose-1	2,322	23.22	23.22	***

DNA Fragmentierung

Sample ID: Naku 2,5

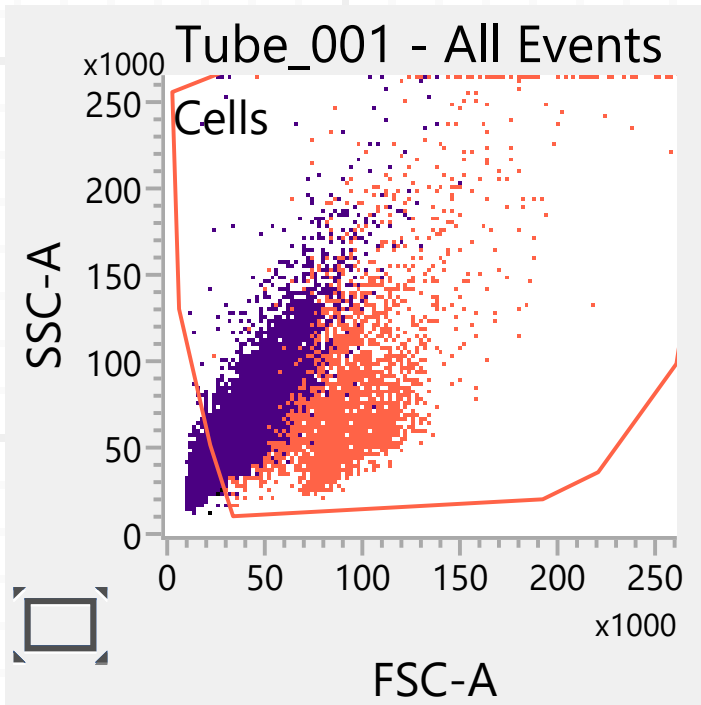

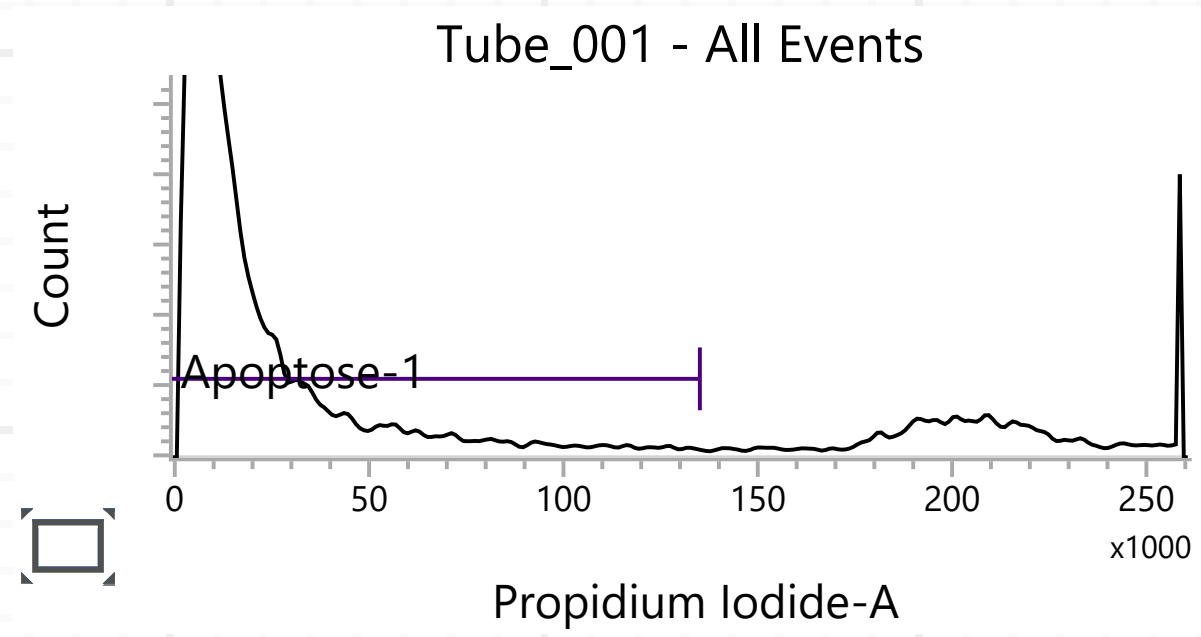

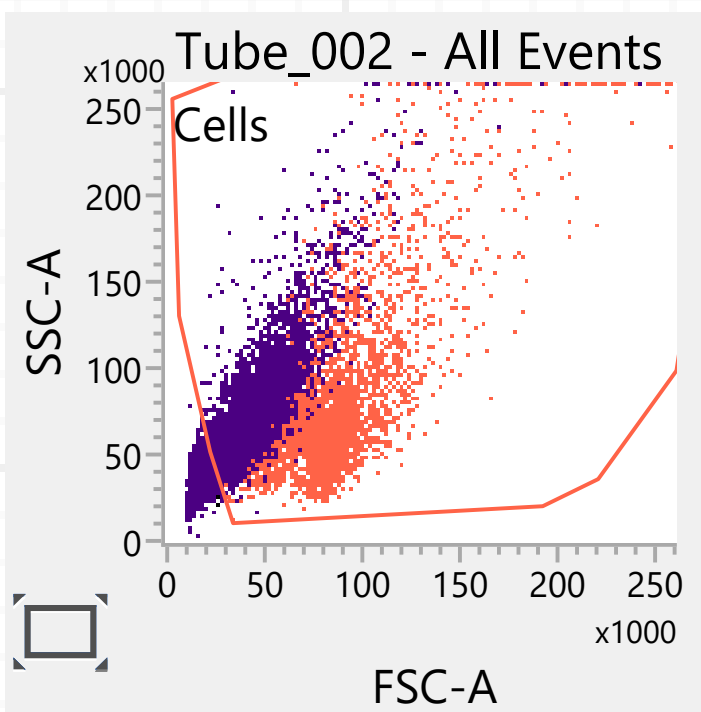

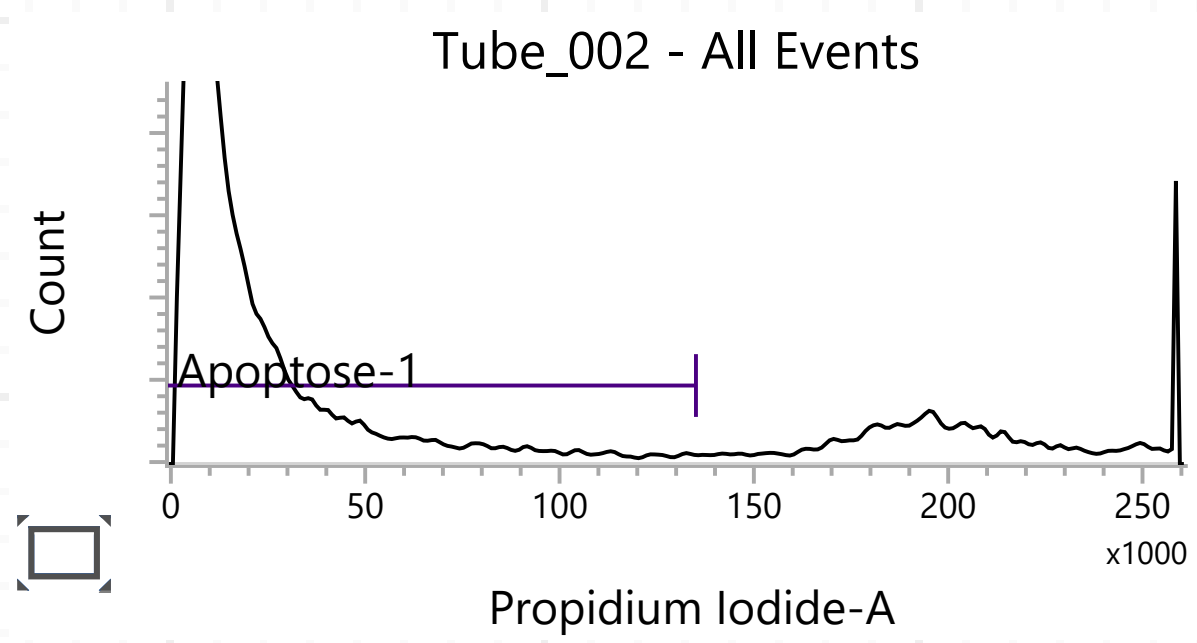

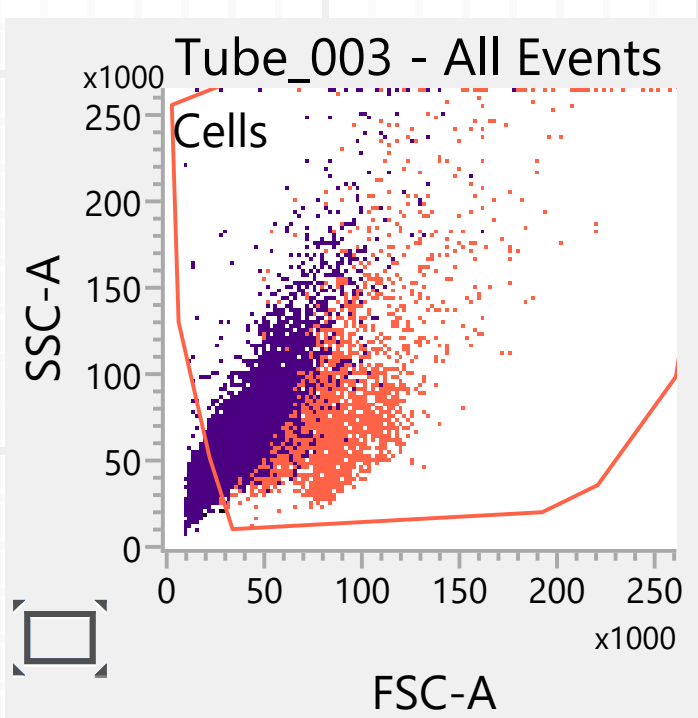

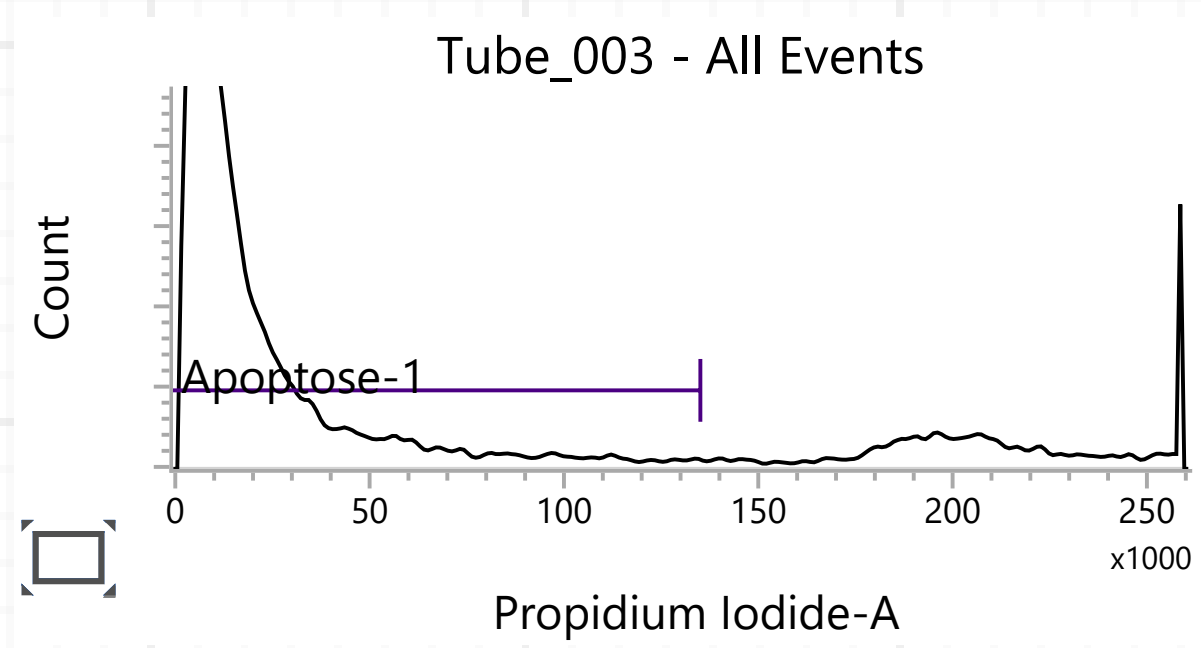

Statistics

Prozent apoptotische Zellen: Prozent apoptotische Zellen No Value % von Cells

Name	Events	% Total	% Parent	% Grandparent	
Tube_001:All Events	11,843	100.00	***	***	
Tube_001:Cells	10,000	84.44	84.44	***	
Tube_002:All Events	10,000	100.00	***	***	
Tube_002:Cells	8,547	85.47	85.47	***	
Tube_003:All Events	10,000	100.00	***	***	
Tube_003:Cells	8,581	85.81	85.81	***	
Tube_001:Apoptose-1	7,915	66.83	66.83	***	
Tube_002:Apoptose-1	6,425	64.25	64.25	***	
Tube_003:Apoptose-1	6,848	68.48	68.48	***	

DNA Fragmentierung

Sample ID: Naku 5

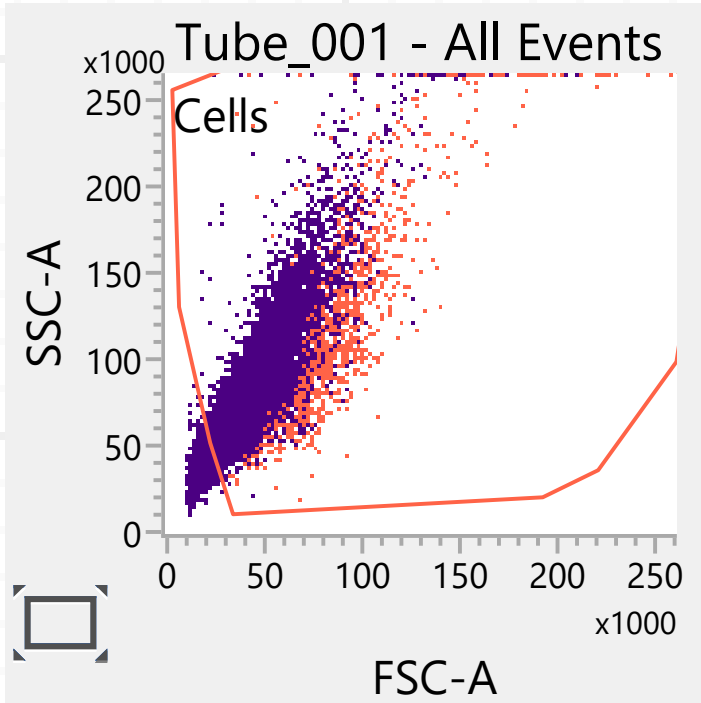

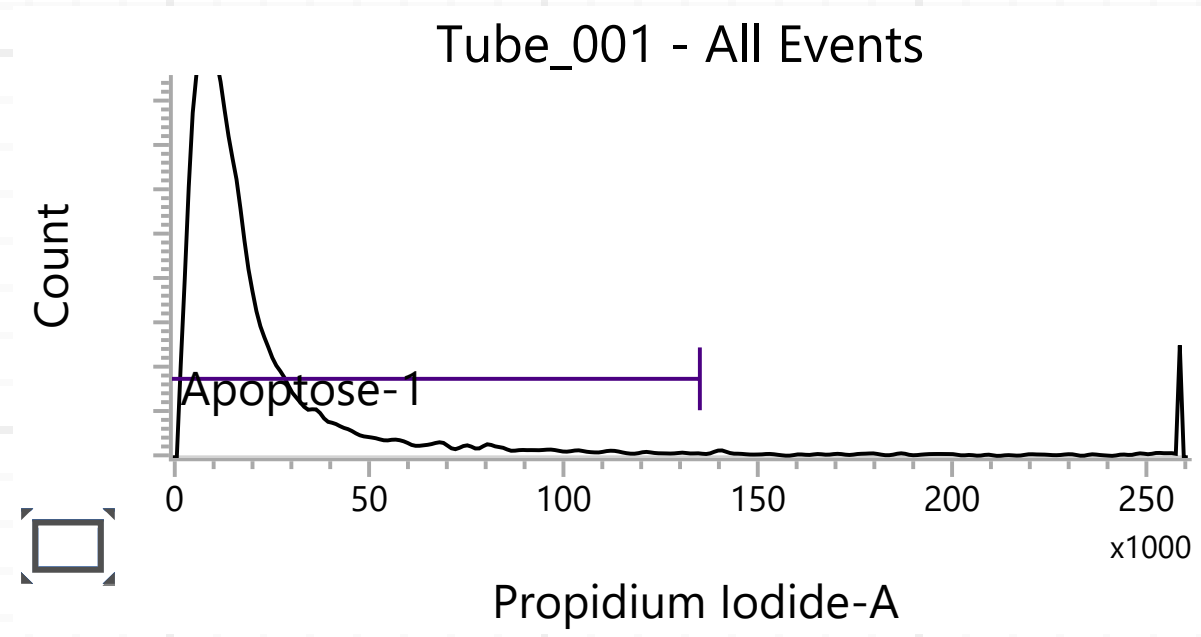

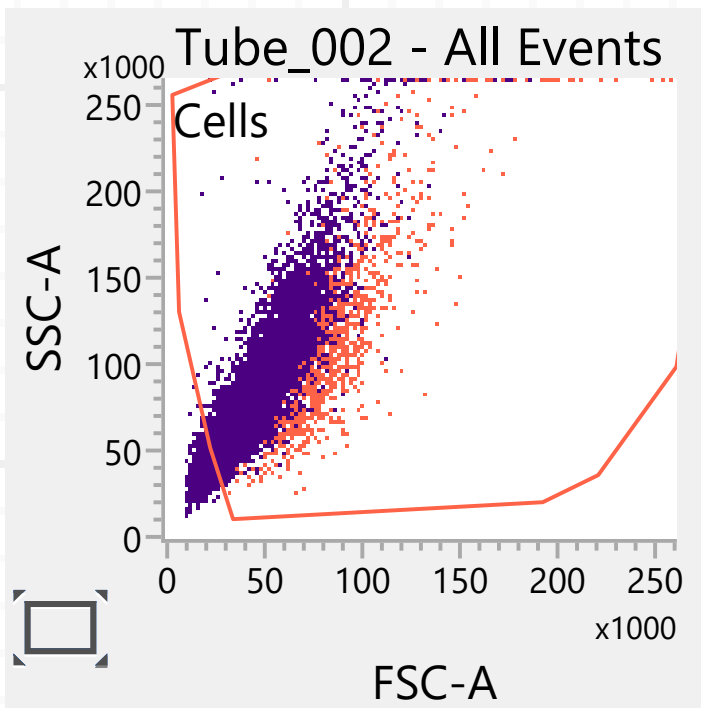

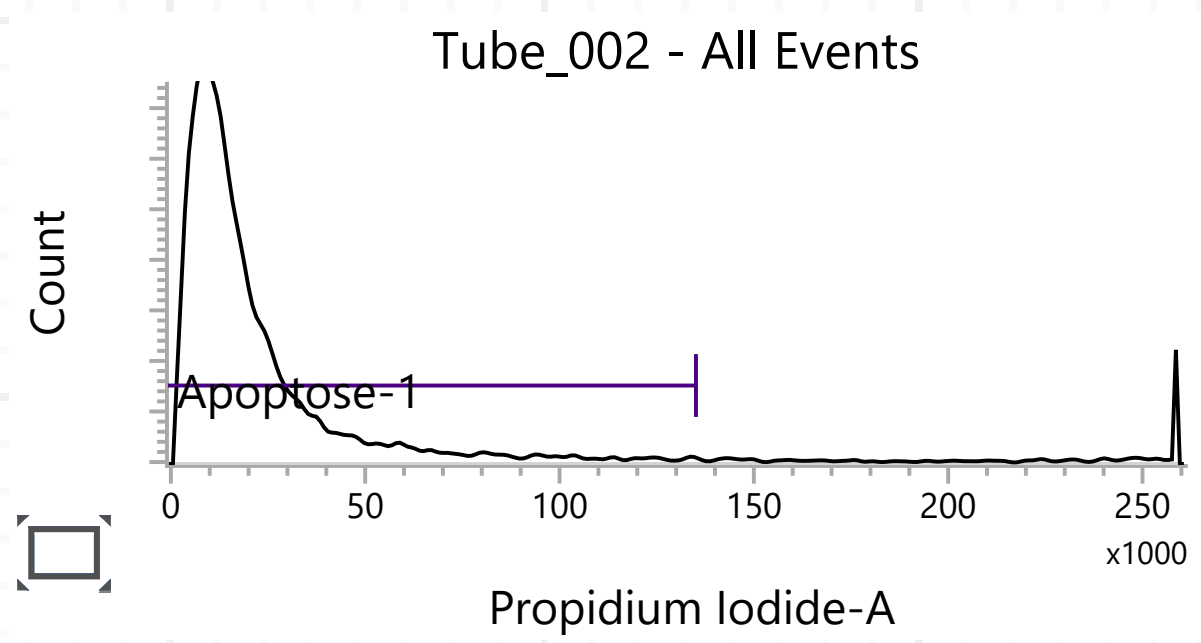

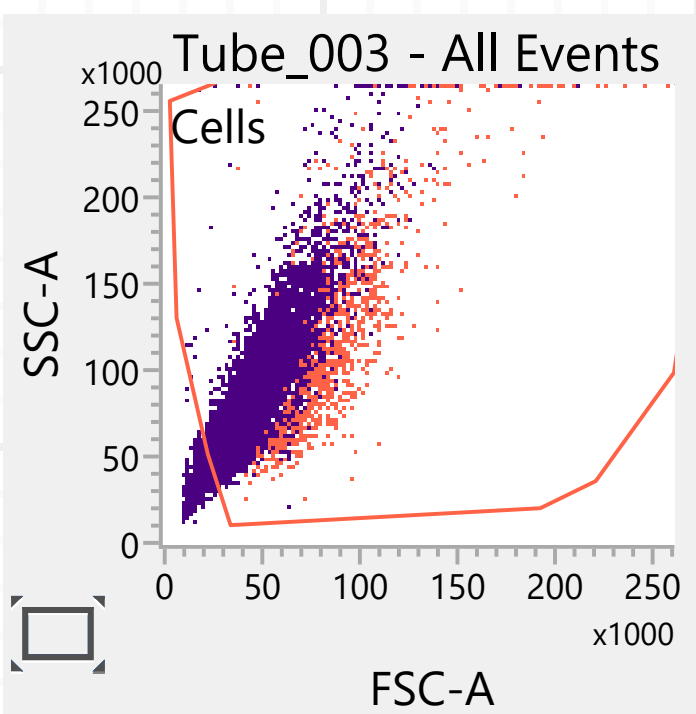

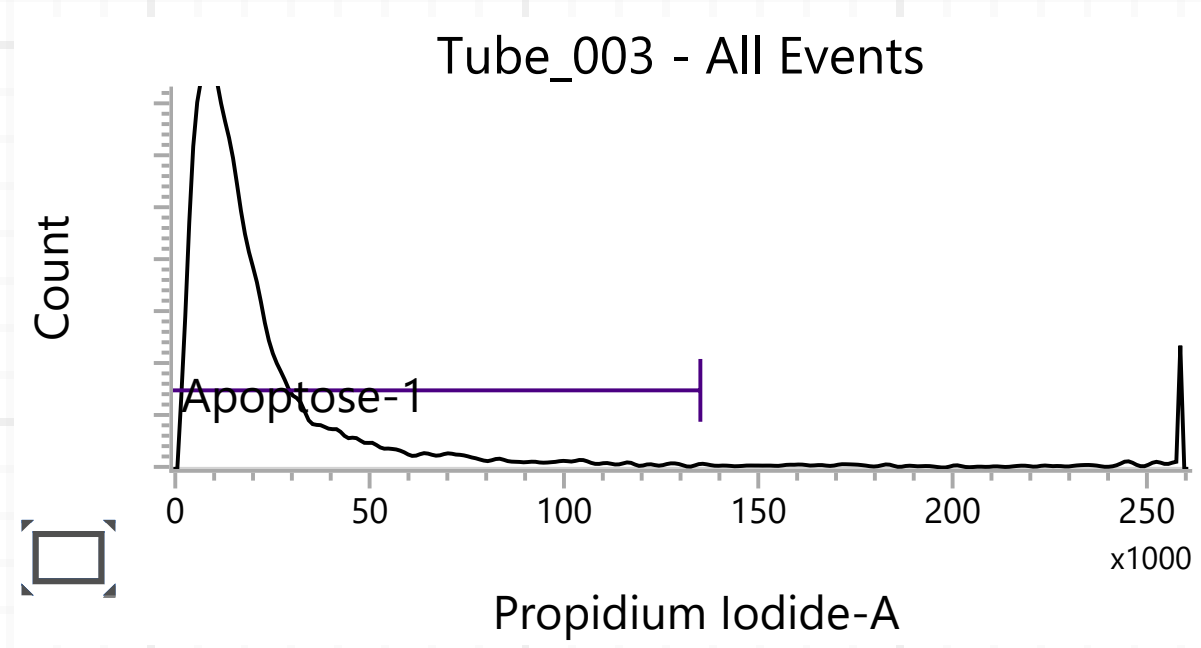

Statistics

Prozent apoptotische Zellen: Prozent apoptotische Zellen No Value % von Cells

Name	Events	% Total	% Parent	% Grandparent
Tube_001:All Events	11,231	100.00	***	***
Tube_001:Cells	10,000	89.04	89.04	***
Tube_002:All Events	10,000	100.00	***	***
Tube_002:Cells	9,033	90.33	90.33	***
Tube_003:All Events	10,000	100.00	***	***
Tube_003:Cells	9,141	91.41	91.41	***
Tube_001:Apoptose-1	9,367	83.40	83.40	***
Tube_002:Apoptose-1	8,284	82.84	82.84	***
Tube_003:Apoptose-1	8,264	82.64	82.64	***

DNA Fragmentierung

Sample ID: Naku-0,1

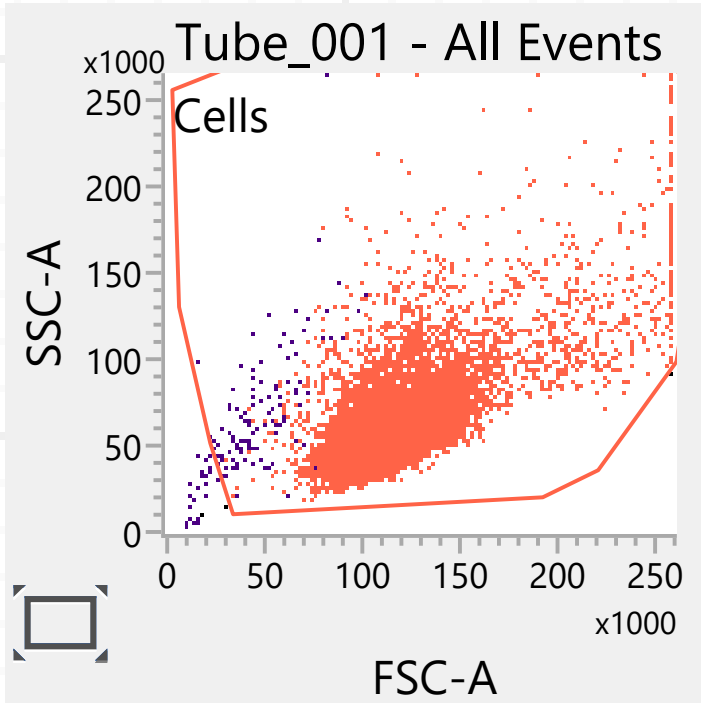

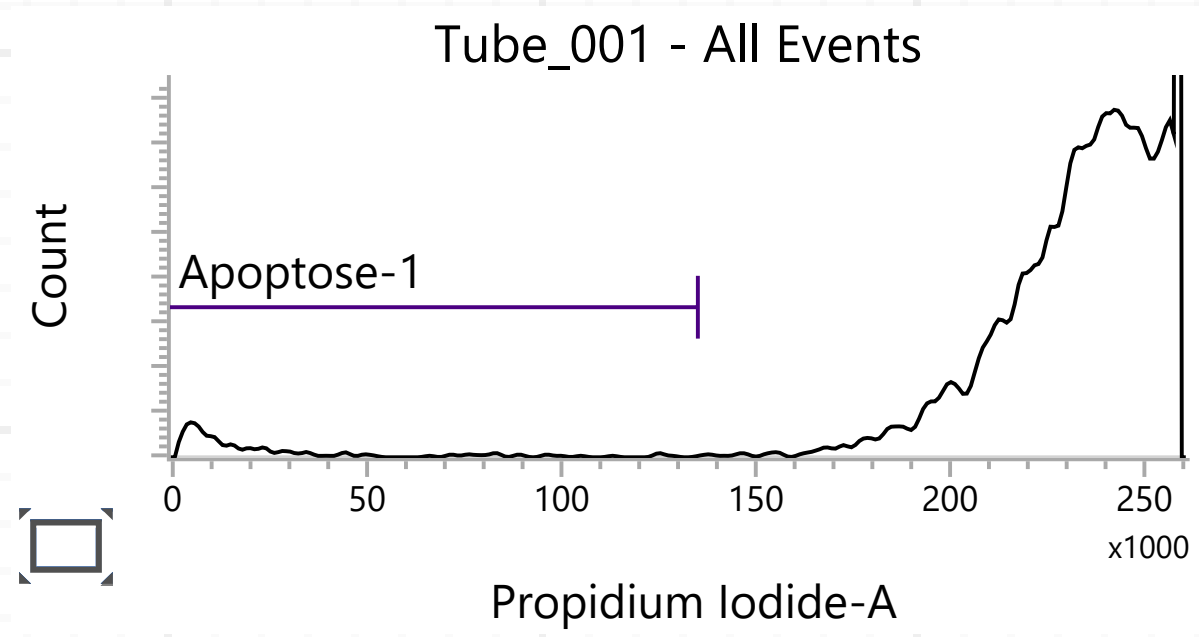

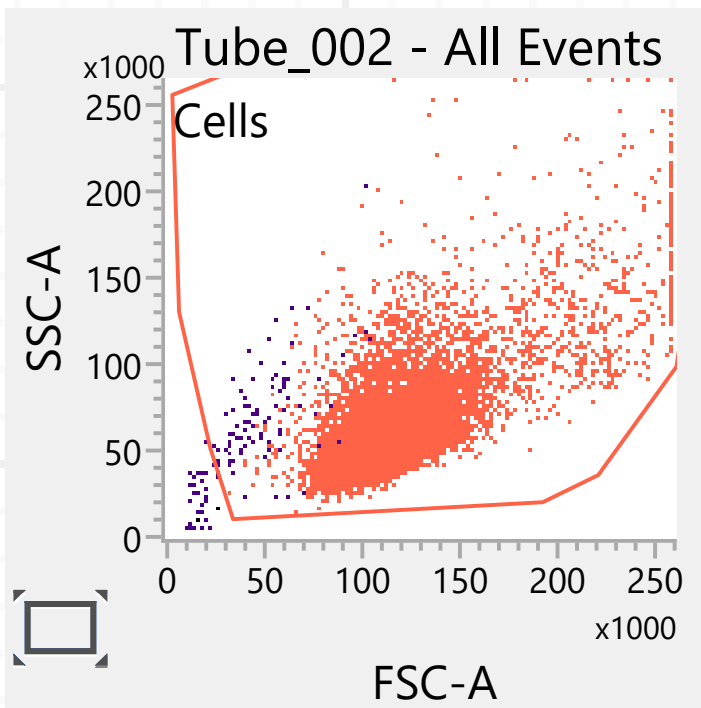

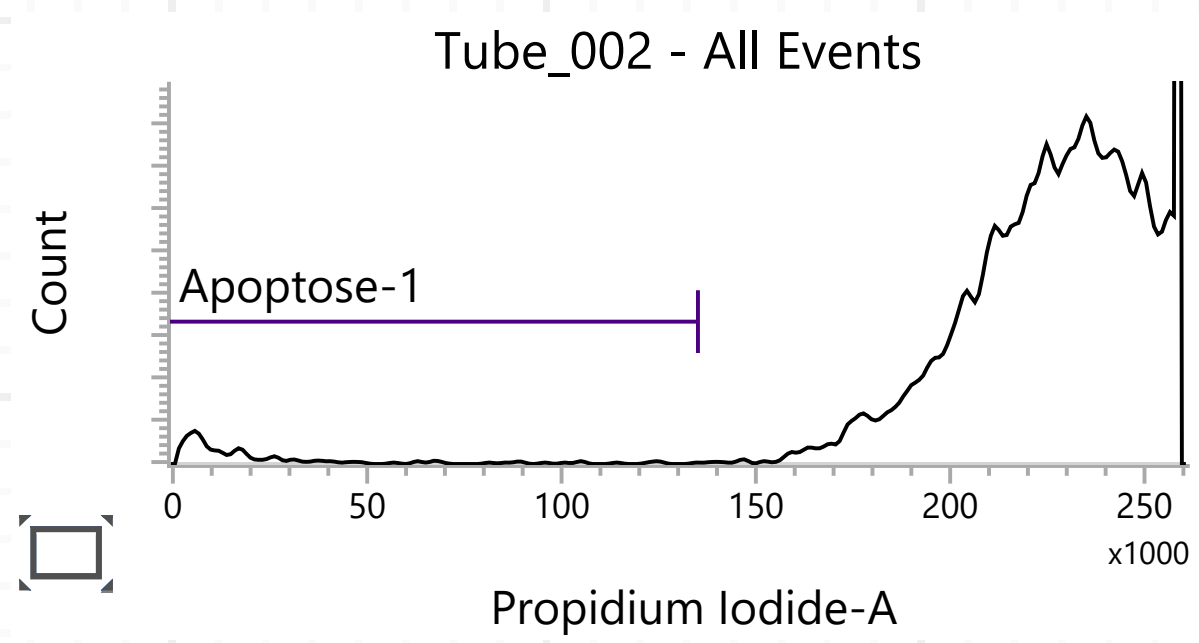

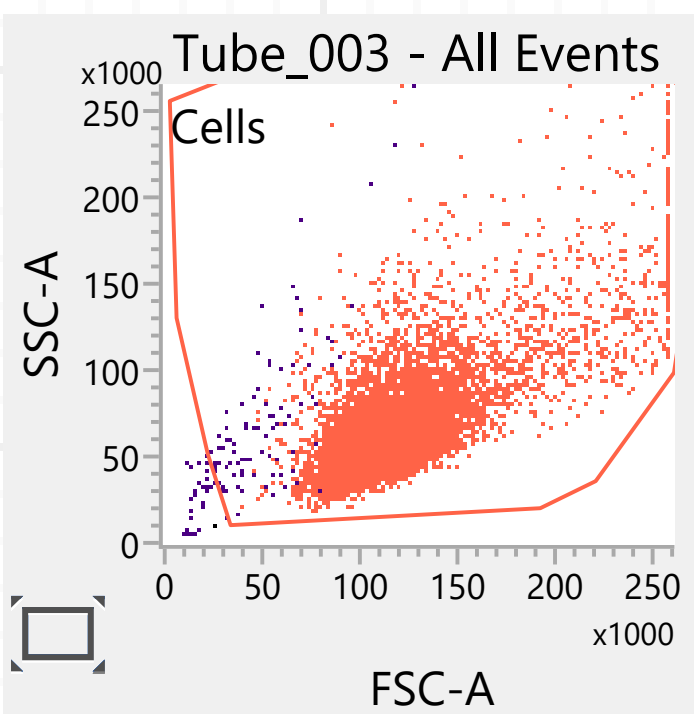

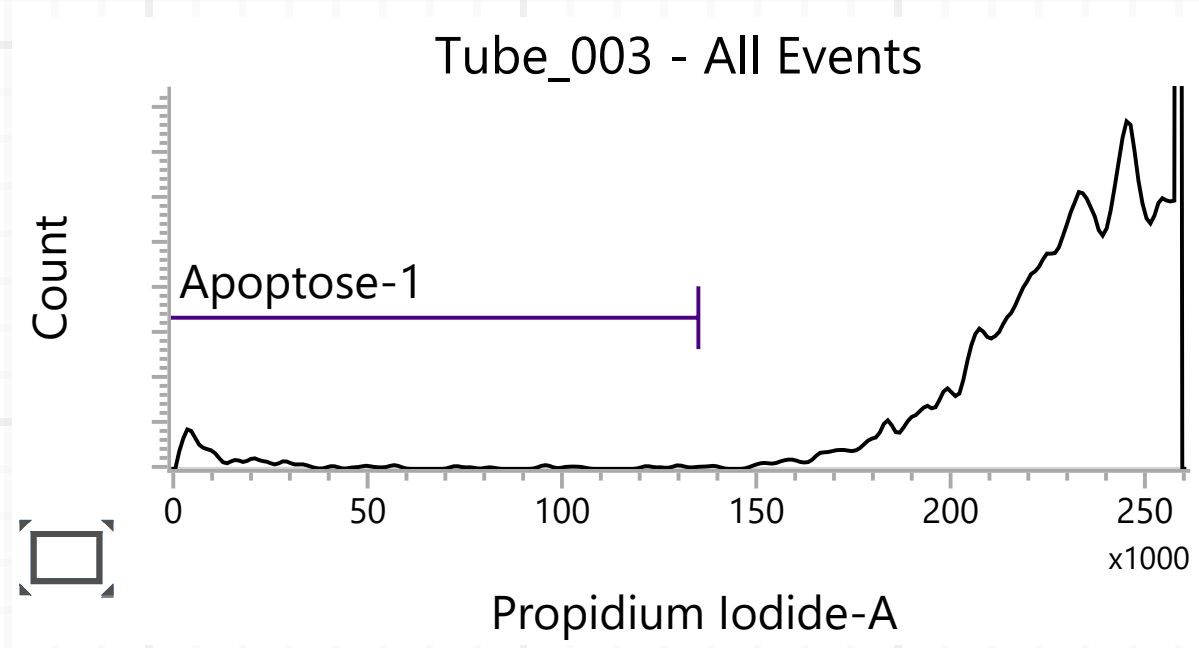

Statistics

Prozent apoptotische Zellen: Prozent apoptotische Zellen No Value % von Cells

Name	Events	% Total	% Parent	% Grandparent
Tube_001:All Events	10,030	100.00	***	***
Tube_001:Cells	10,000	99.70	99.70	***
Tube_002:All Events	10,000	100.00	***	***
Tube_002:Cells	9,969	99.69	99.69	***
Tube_003:All Events	10,000	100.00	***	***
Tube_003:Cells	9,968	99.68	99.68	***
Tube_001:Apoptose-1	134	1.34	1.34	***
Tube_002:Apoptose-1	123	1.23	1.23	***
Tube_003:Apoptose-1	121	1.21	1.21	***

DNA Fragmentierung

Sample ID: Naku-D

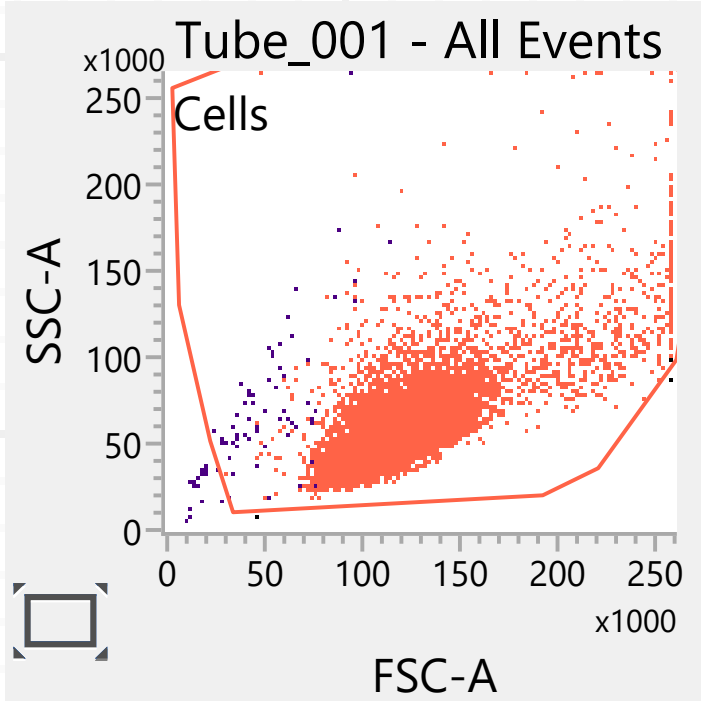

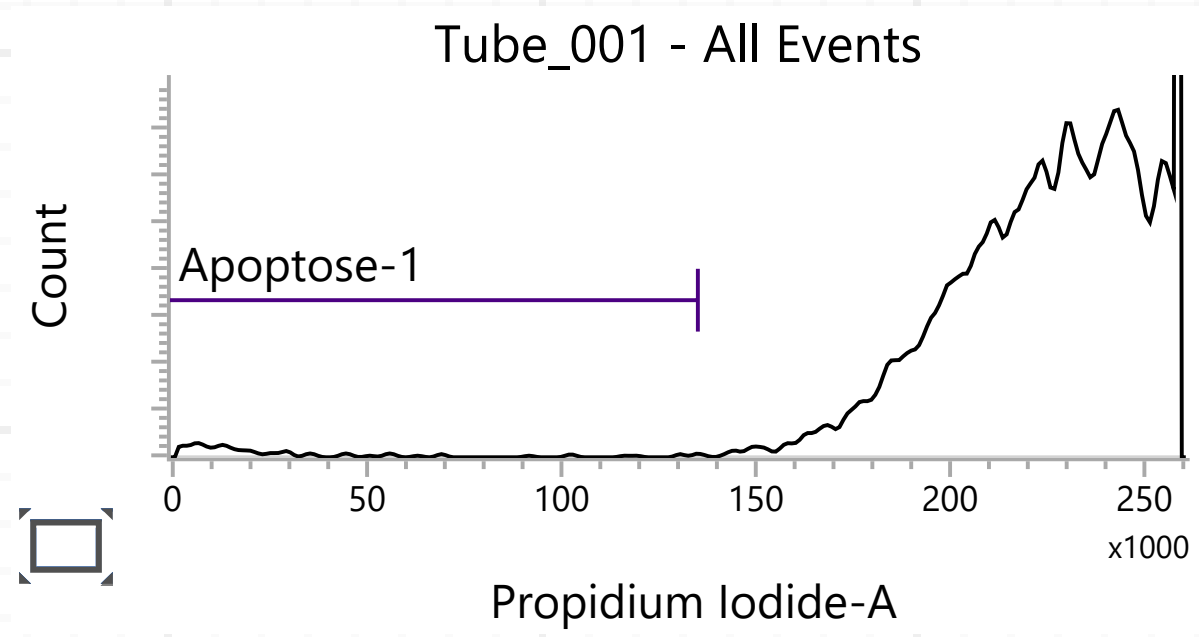

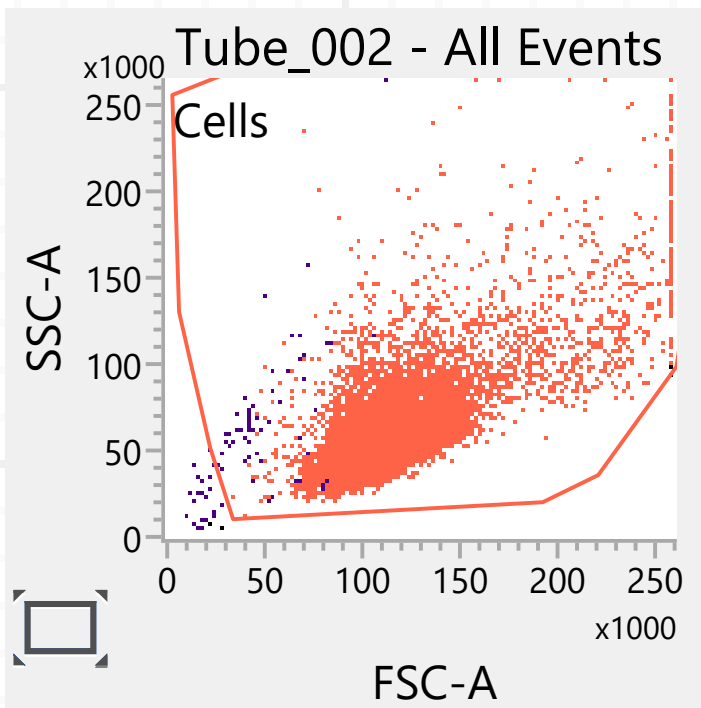

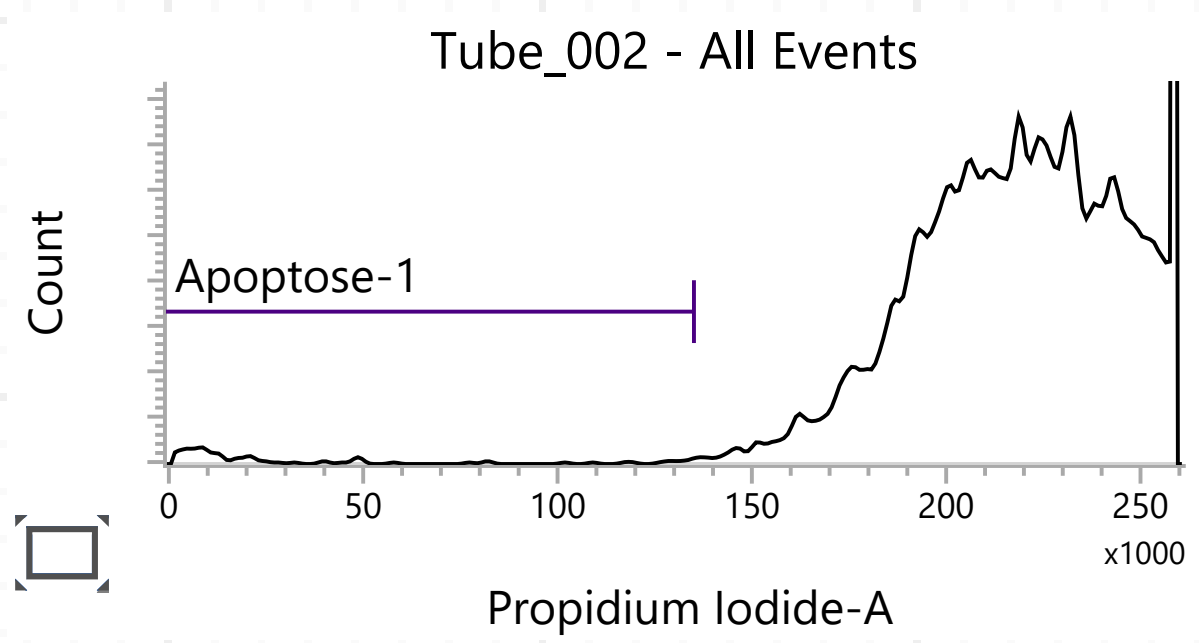

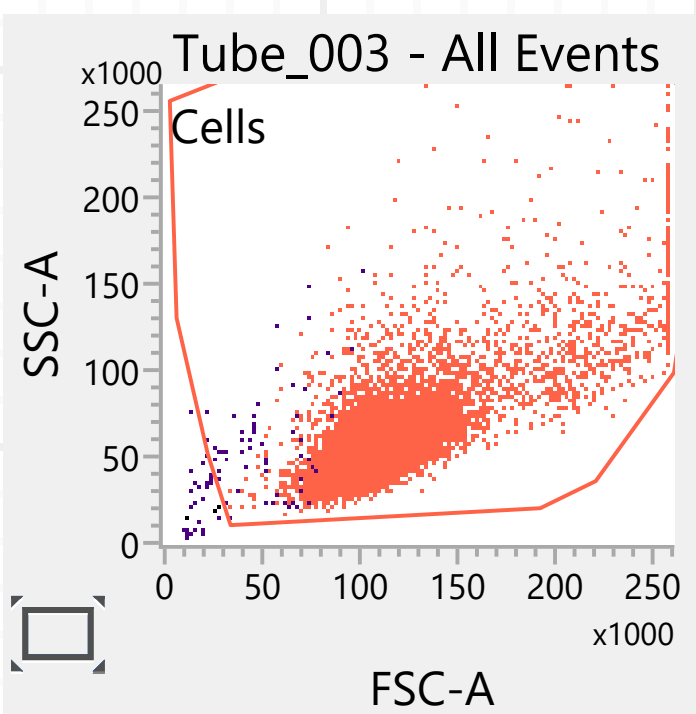

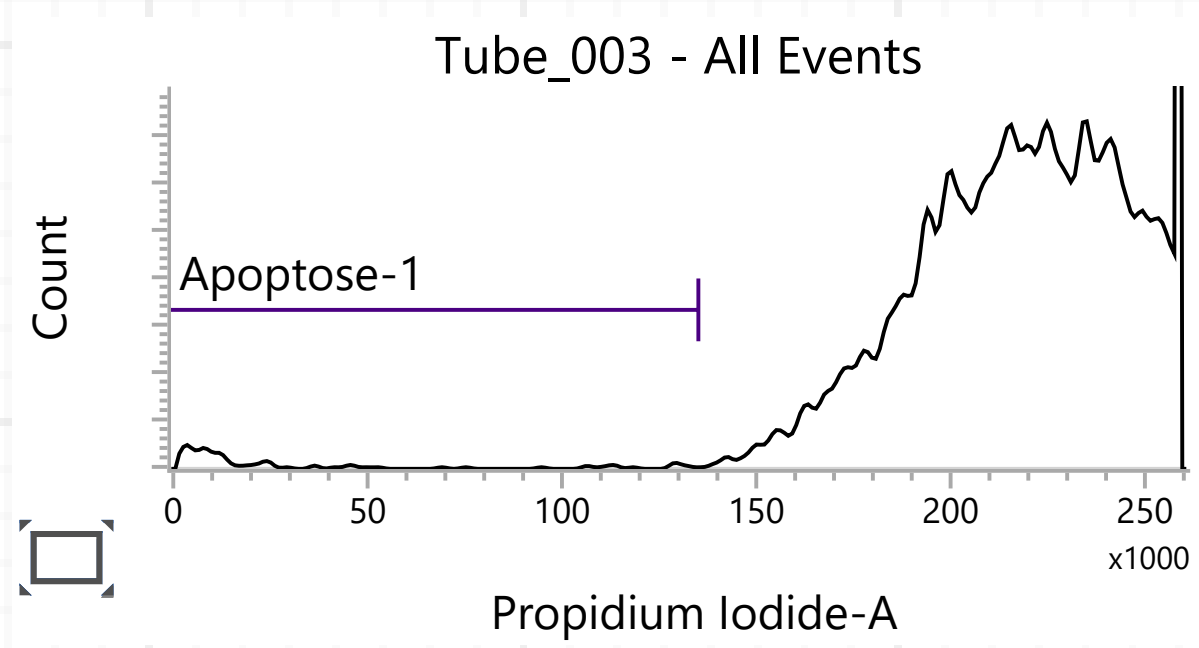

Statistics

Prozent apoptotische Zellen: Prozent apoptotische Zellen No Value % von Cells

Name	Events	% Total	% Parent	% Grandparent
Tube_001:All Events	10,020	100.00	***	***
Tube_001:Cells	10,000	99.80	99.80	***
Tube_002:All Events	10,000	100.00	***	***
Tube_002:Cells	9,975	99.75	99.75	***
Tube_003:All Events	10,000	100.00	***	***
Tube_003:Cells	9,971	99.71	99.71	***
Tube_001:Apoptose-1	77	0.77	0.77	***
Tube_002:Apoptose-1	80	0.80	0.80	***
Tube_003:Apoptose-1	89	0.89	0.89	***

DNA Fragmentierung

Sample ID: NaKu-K

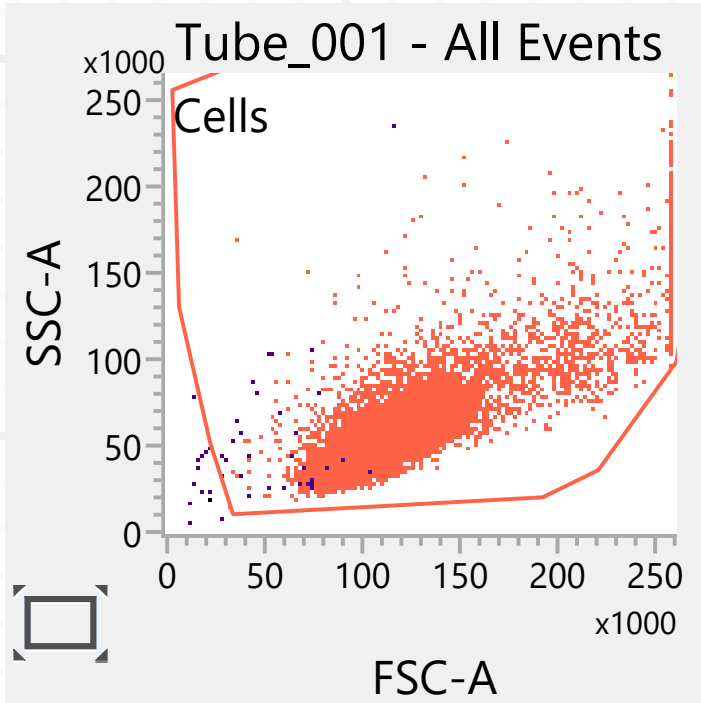

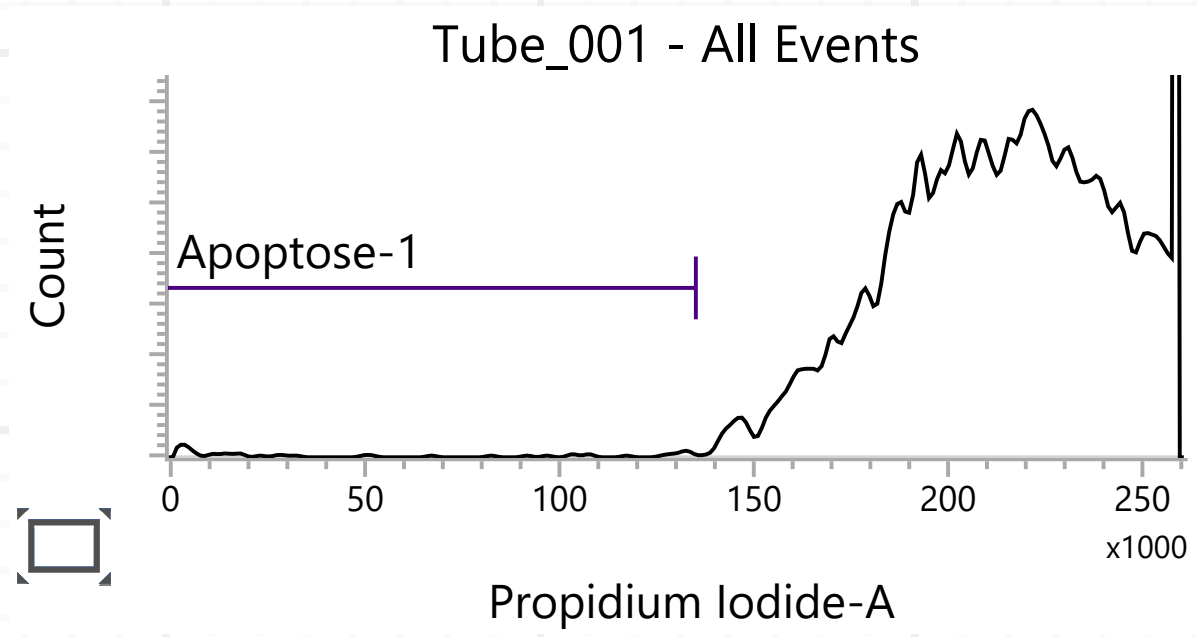

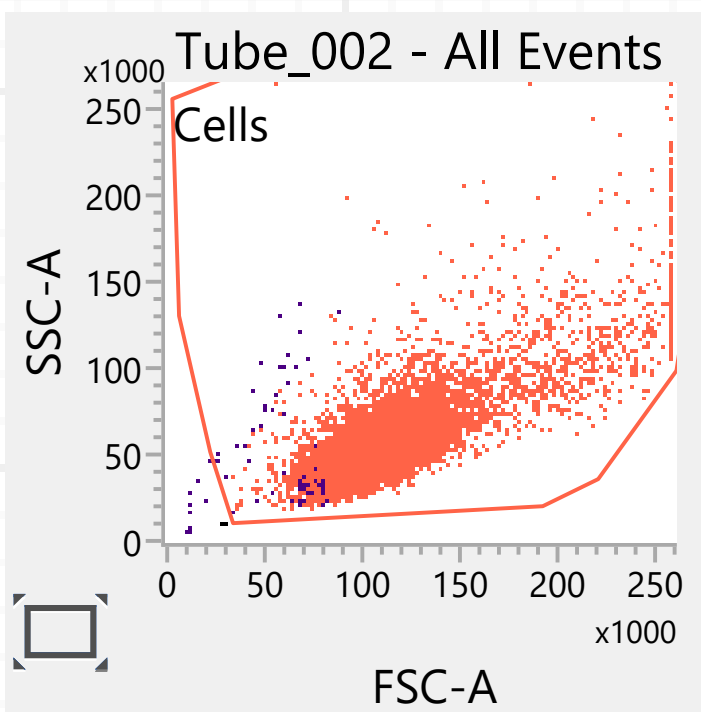

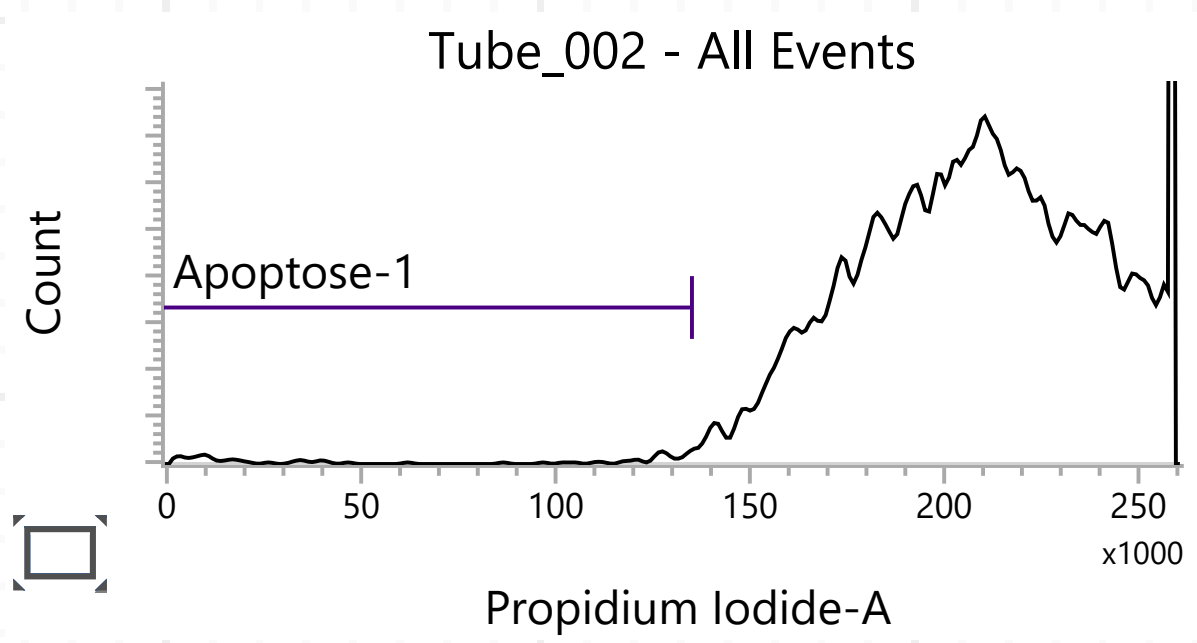

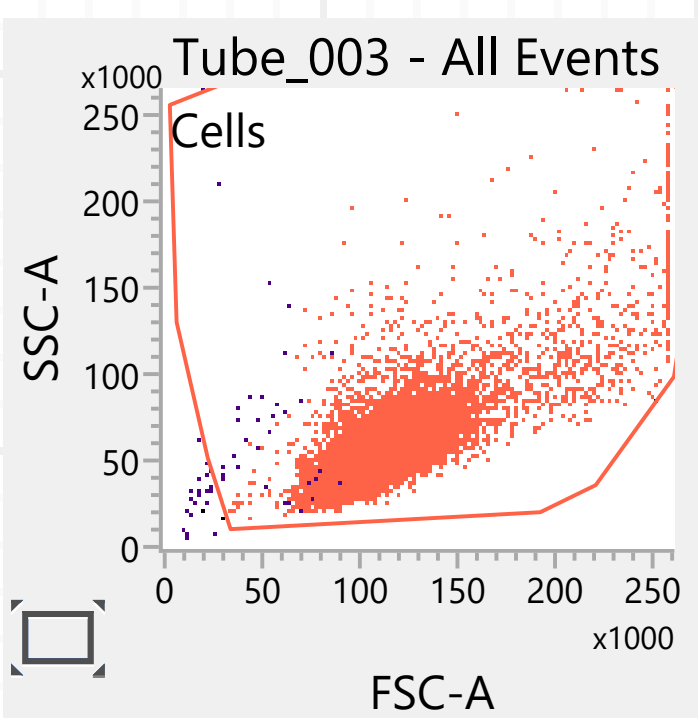

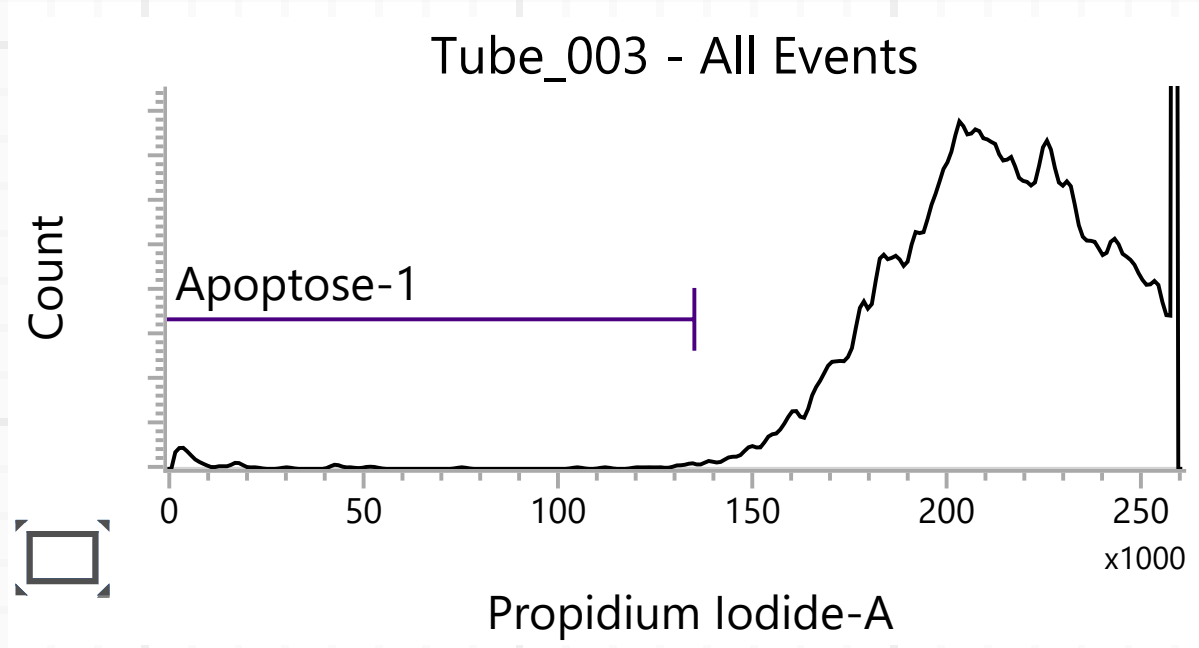

Statistics

Prozent apoptotische Zellen: Prozent apoptotische Zellen No Value % von Cells

Name	Events	% Total	% Parent	% Grandparent	
Tube_001:All Events	10,012	100.00	***	***	
Tube_001:Cells	10,000	99.88	99.88	***	
Tube_002:All Events	10,000	100.00	***	***	
Tube_002:Cells	9,989	99.89	99.89	***	
Tube_003:All Events	10,000	100.00	***	***	
Tube_003:Cells	9,977	99.77	99.77	***	
Tube_001:Apoptose-1	43	0.43	0.43	***	
Tube_002:Apoptose-1	64	0.64	0.64	***	
Tube_003:Apoptose-1	57	0.57	0.57	***	

DNA Fragmentierung

Sample ID: Naku-Pred

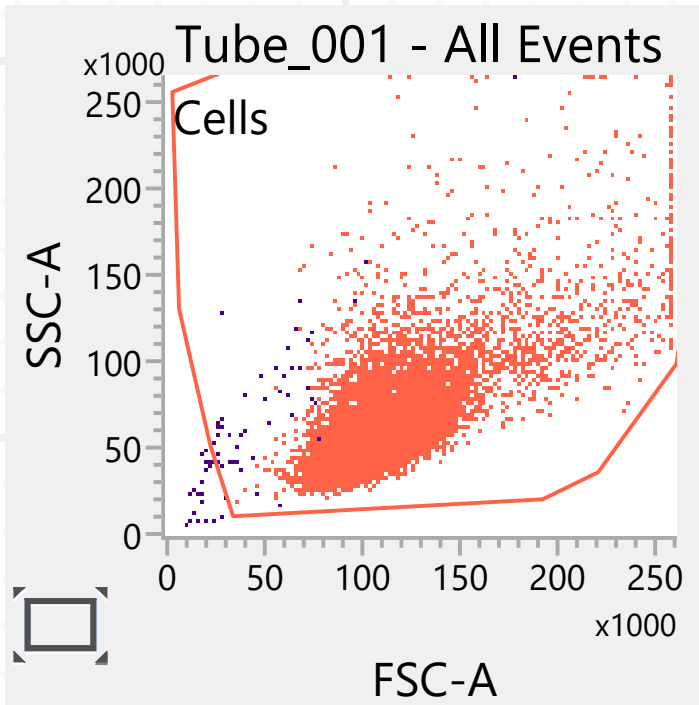

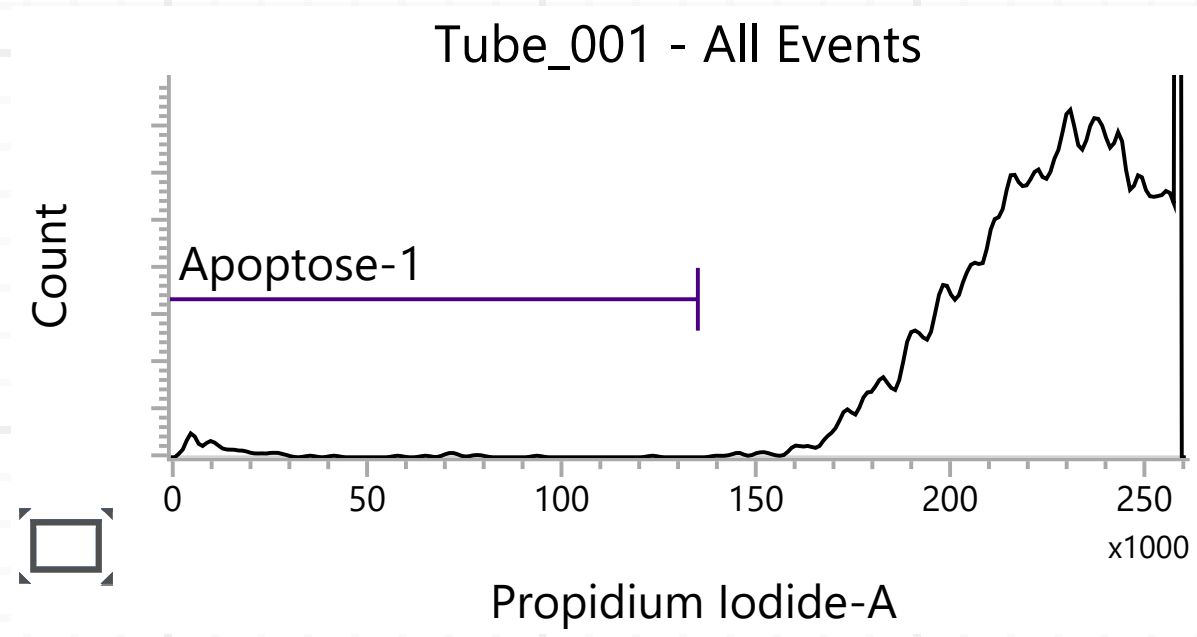

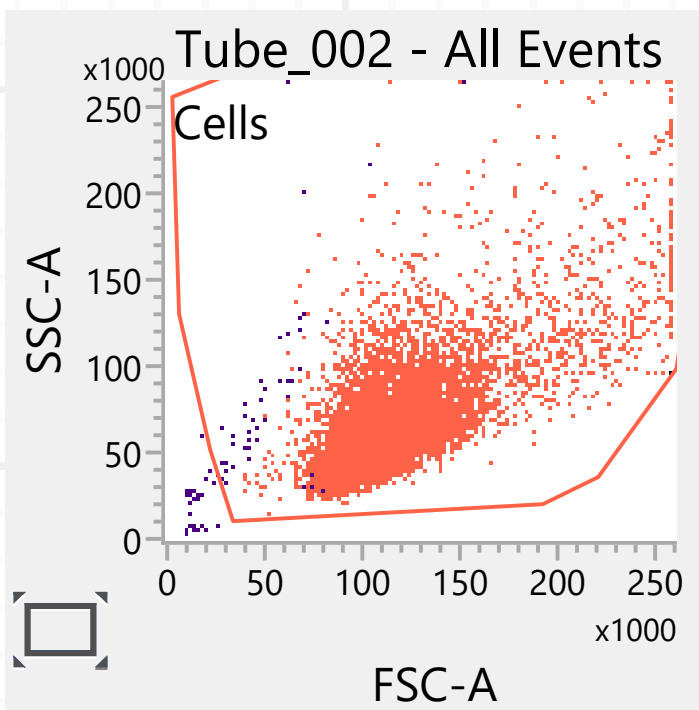

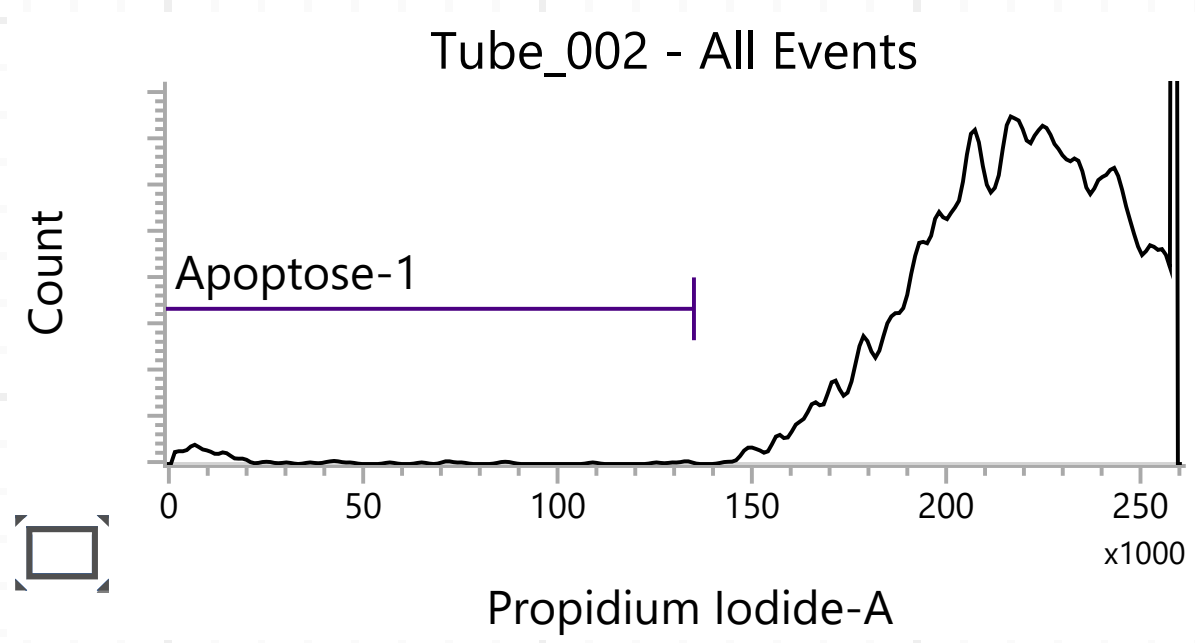

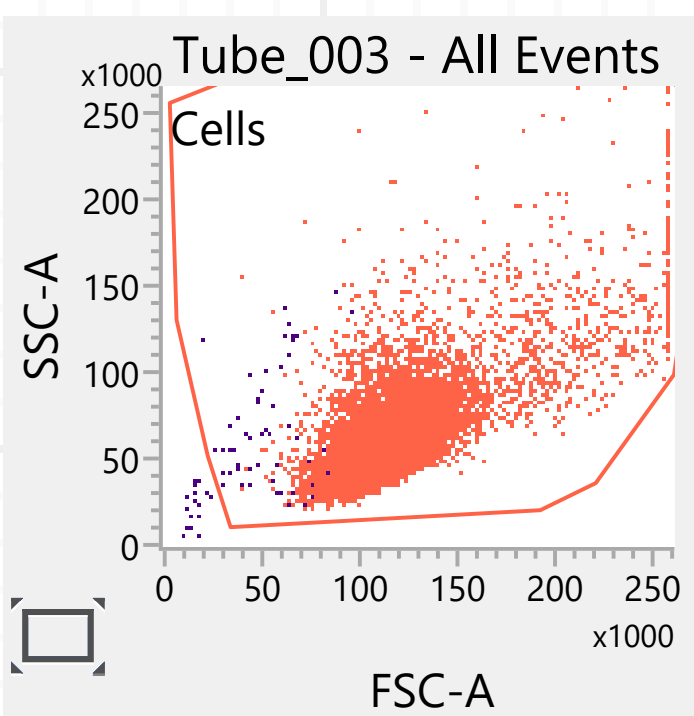

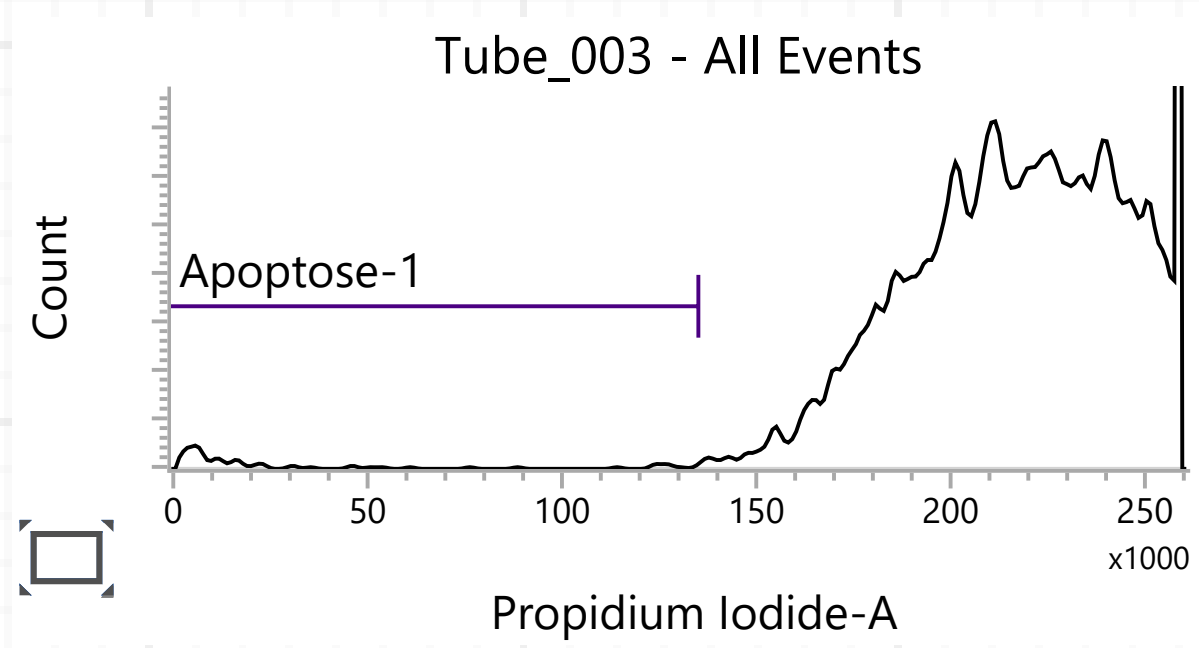

Statistics

Prozent apoptotische Zellen: Prozent apoptotische Zellen No Value % von Cells

Name	Events	% Total	% Parent	% Grandparent
Tube_001:All Events	10,020	100.00	***	***
Tube_001:Cells	10,000	99.80	99.80	***
Tube_002:All Events	10,000	100.00	***	***
Tube_002:Cells	9,973	99.73	99.73	***
Tube_003:All Events	10,000	100.00	***	***
Tube_003:Cells	9,983	99.83	99.83	***
Tube_001:Apoptose-1	66	0.66	0.66	***
Tube_002:Apoptose-1	72	0.72	0.72	***
Tube_003:Apoptose-1	69	0.69	0.69	***

DNA Fragmentierung

Sample ID: N6_0,1

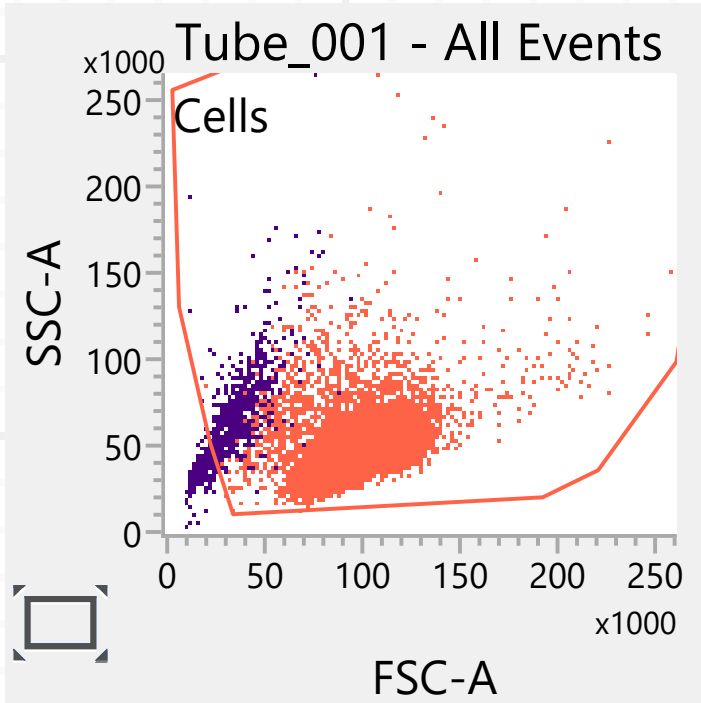

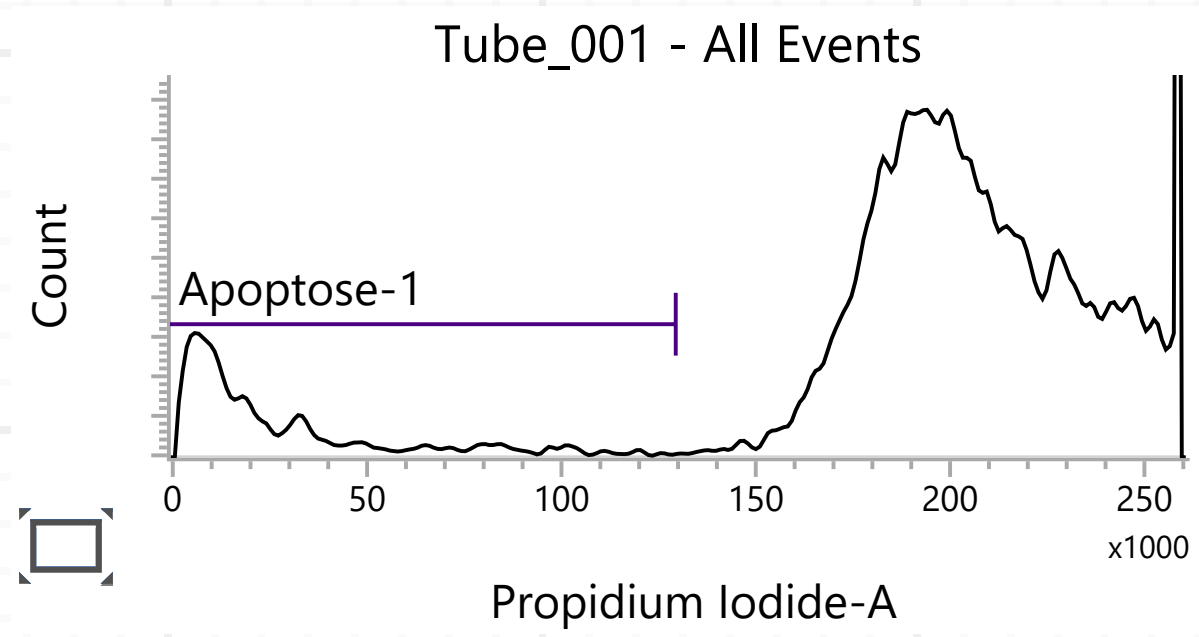

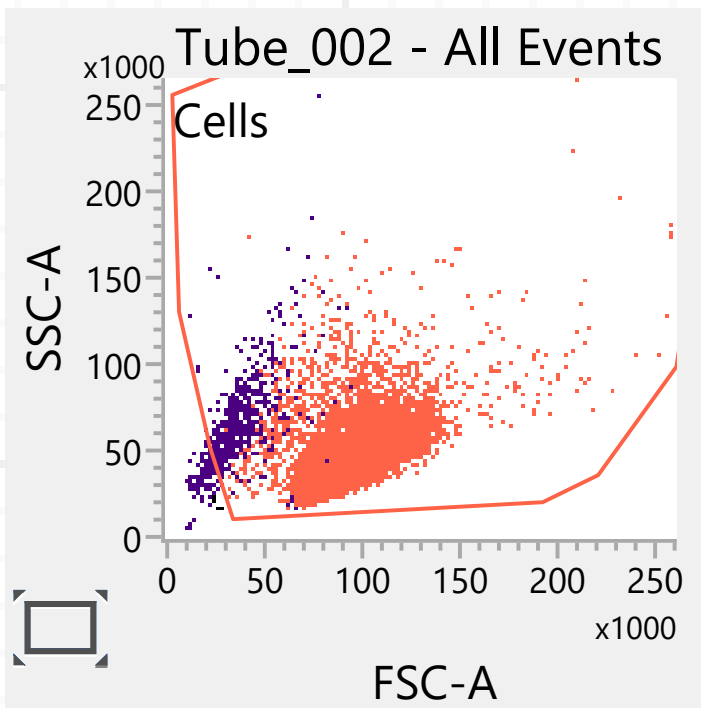

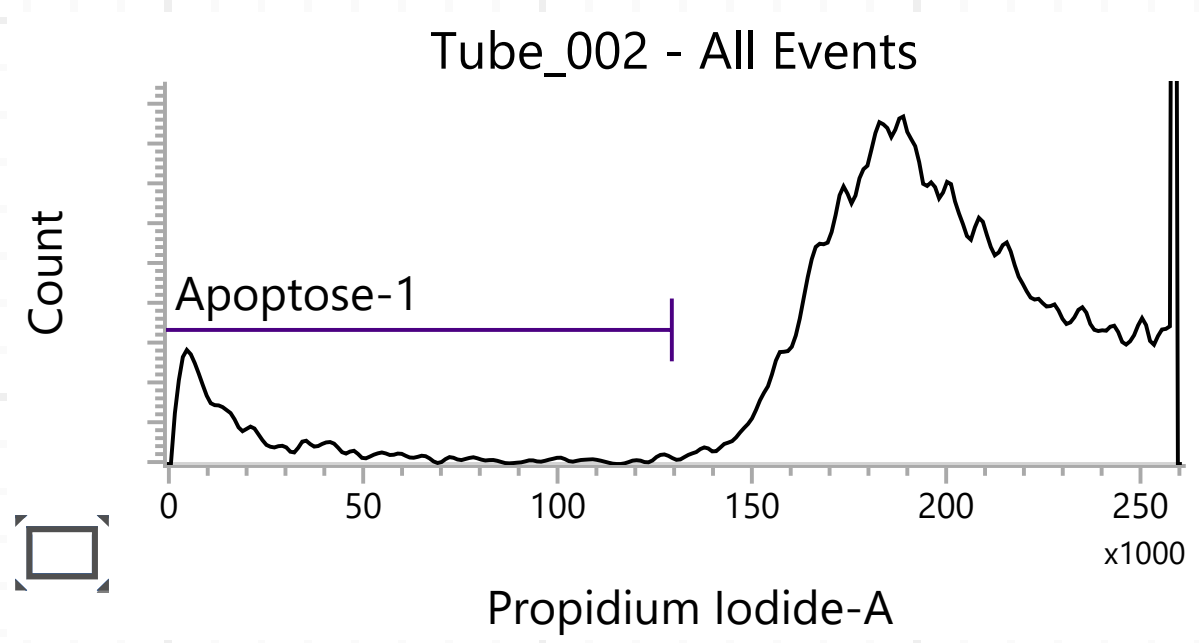

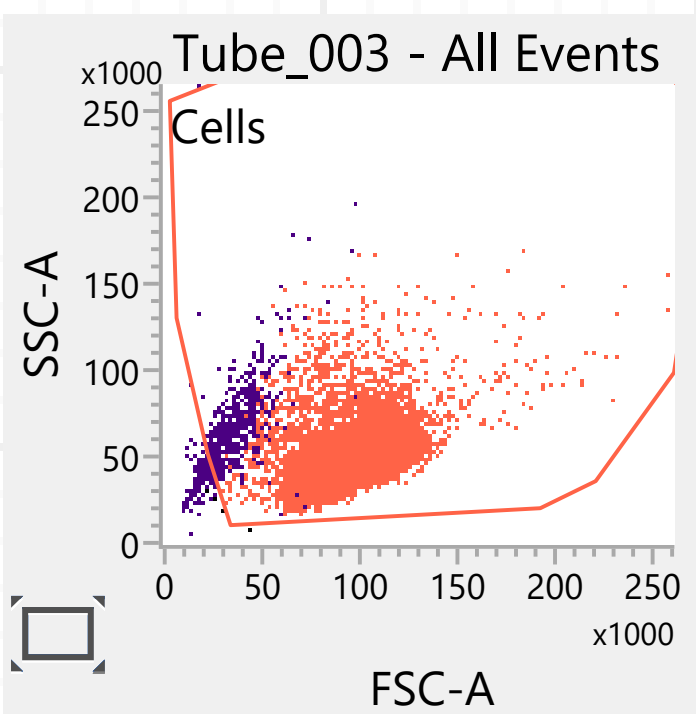

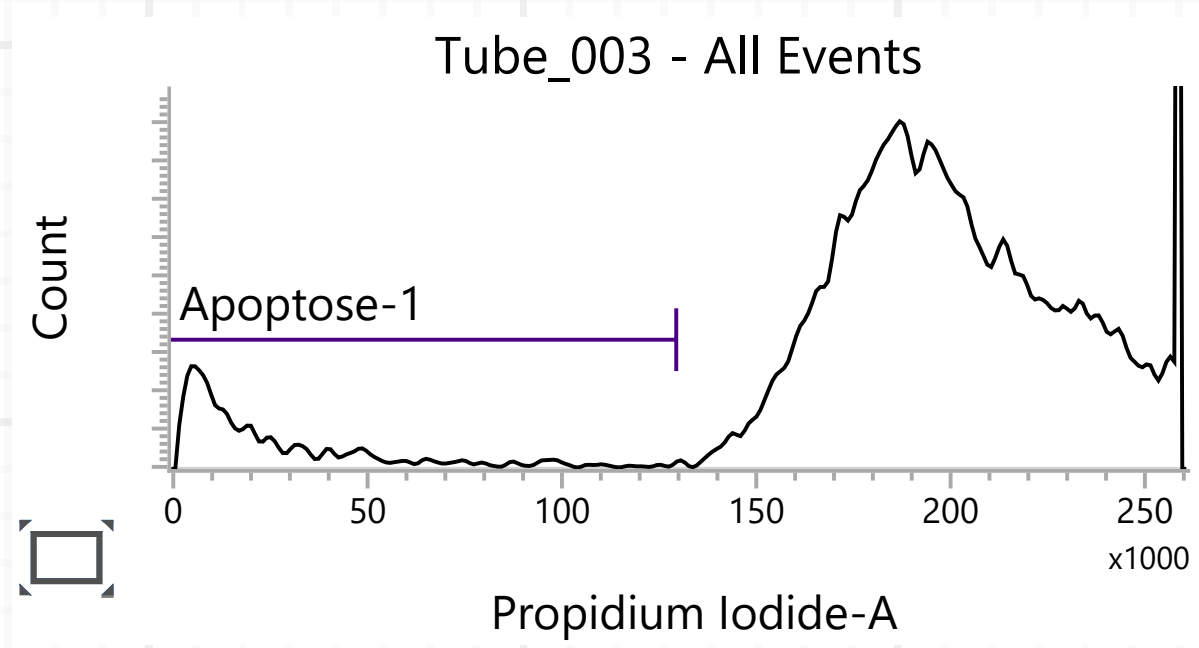

Statistics

Prozent apoptotische Zellen: Prozent apoptotische Zellen No Value % von Cells

Name	Events	% Total	% Parent	% Grandparent
Tube_001:All Events	10,143	100.00	***	***
Tube_001:Cells	10,000	98.59	98.59	***
Tube_002:All Events	10,000	100.00	***	***
Tube_002:Cells	9,896	98.96	98.96	***
Tube_003:All Events	10,000	100.00	***	***
Tube_003:Cells	9,880	98.80	98.80	***
Tube_001:Apoptose-1	765	7.54	7.54	***
Tube_002:Apoptose-1	565	5.65	5.65	***
Tube_003:Apoptose-1	598	5.98	5.98	***

DNA Fragmentierung

Sample ID: N6_0,5

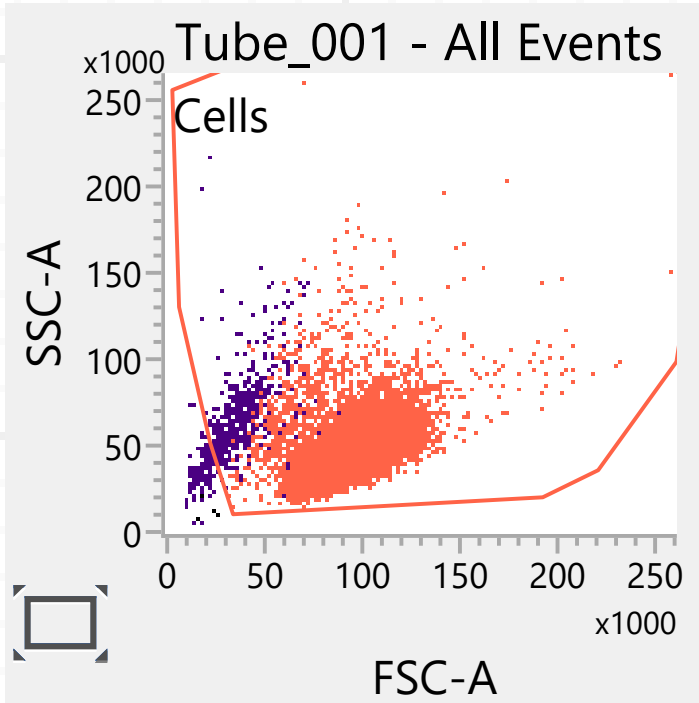

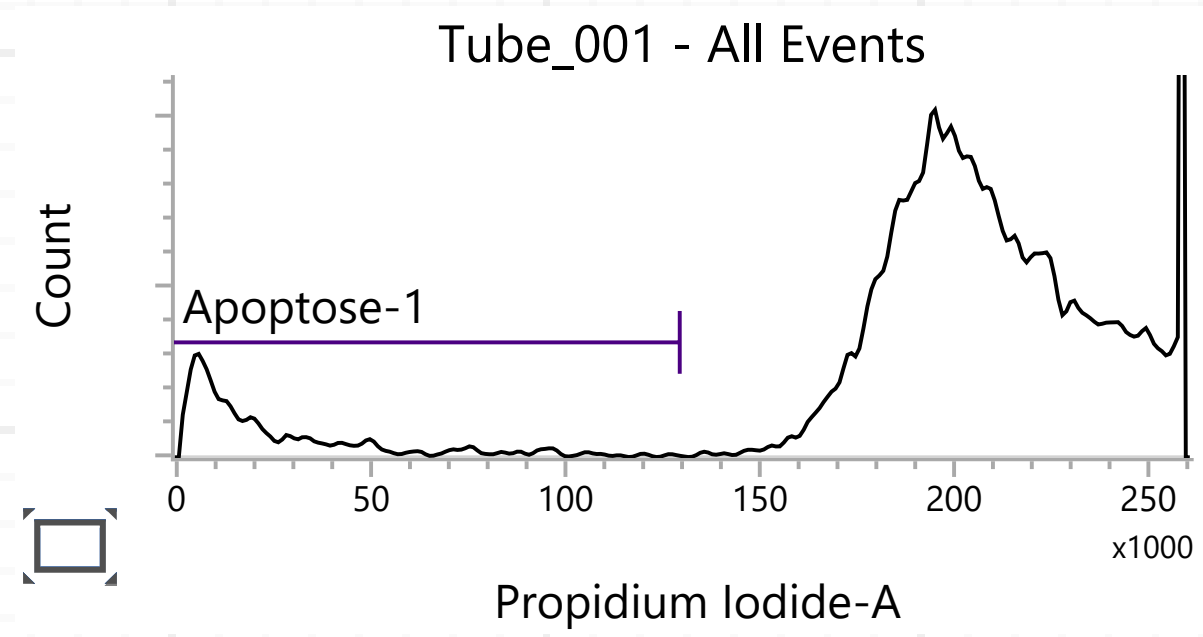

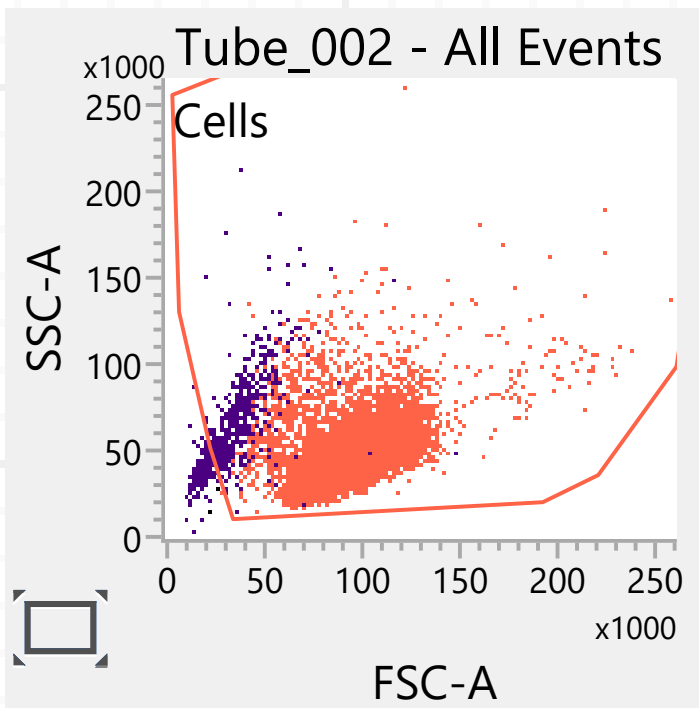

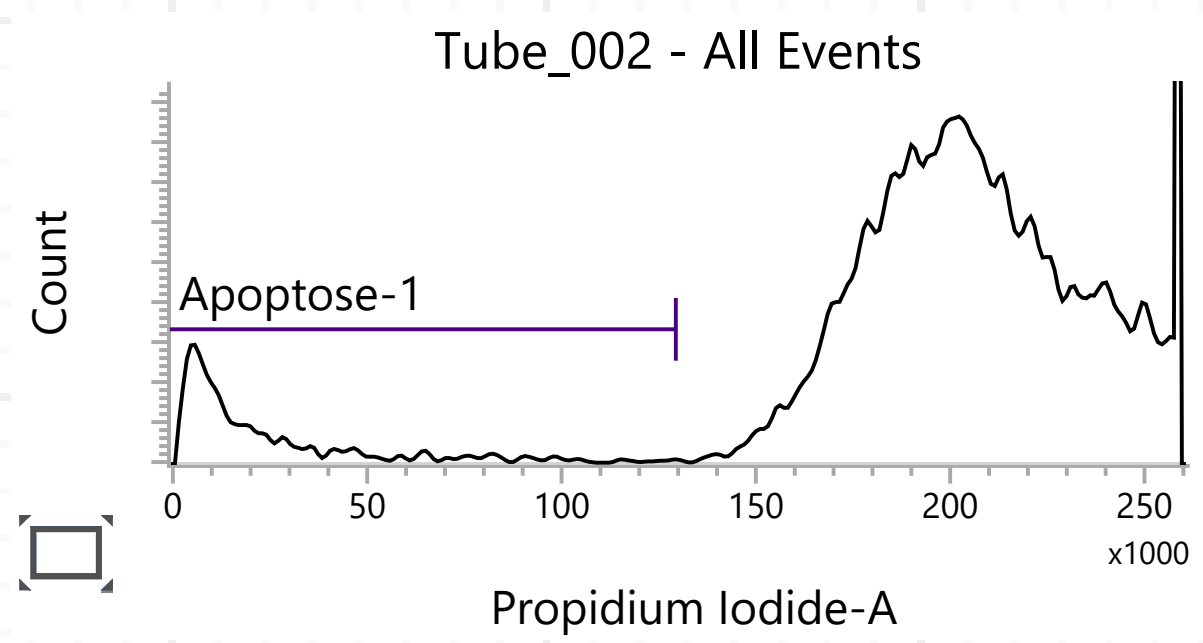

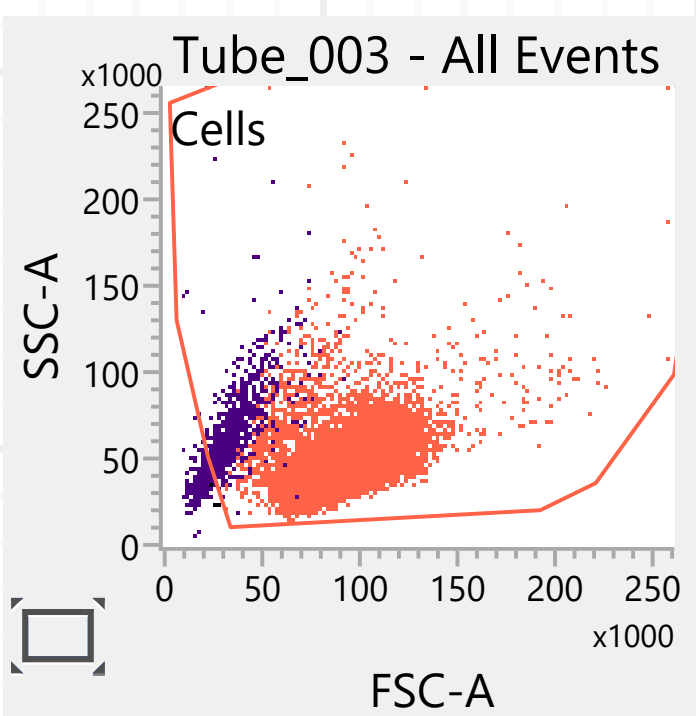

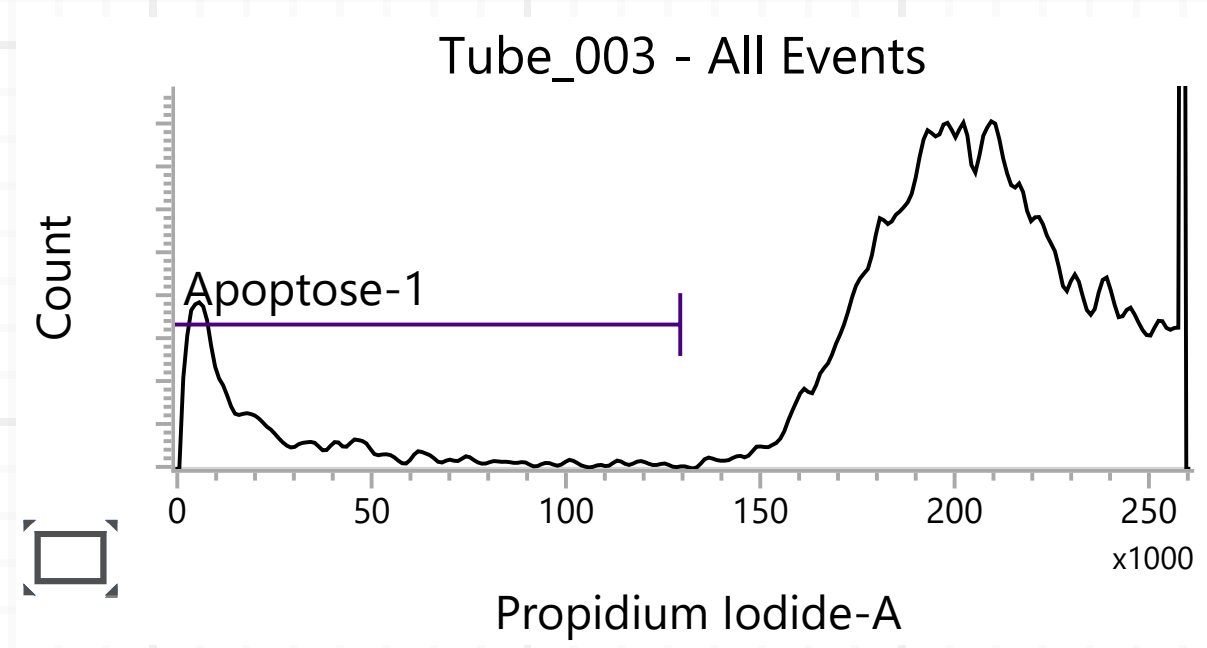

Statistics

Prozent apoptotische Zellen: Prozent apoptotische Zellen No Value % von Cells

Name	Events	% Total	% Parent	% Grandparent
Tube_001:All Events	10,123	100.00	***	***
Tube_001:Cells	10,000	98.78	98.78	***
Tube_002:All Events	10,000	100.00	***	***
Tube_002:Cells	9,892	98.92	98.92	***
Tube_003:All Events	10,000	100.00	***	***
Tube_003:Cells	9,872	98.72	98.72	***
Tube_001:Apoptose-1	616	6.09	6.09	***
Tube_002:Apoptose-1	572	5.72	5.72	***
Tube_003:Apoptose-1	780	7.80	7.80	***

DNA Fragmentierung

Sample ID: N6_1

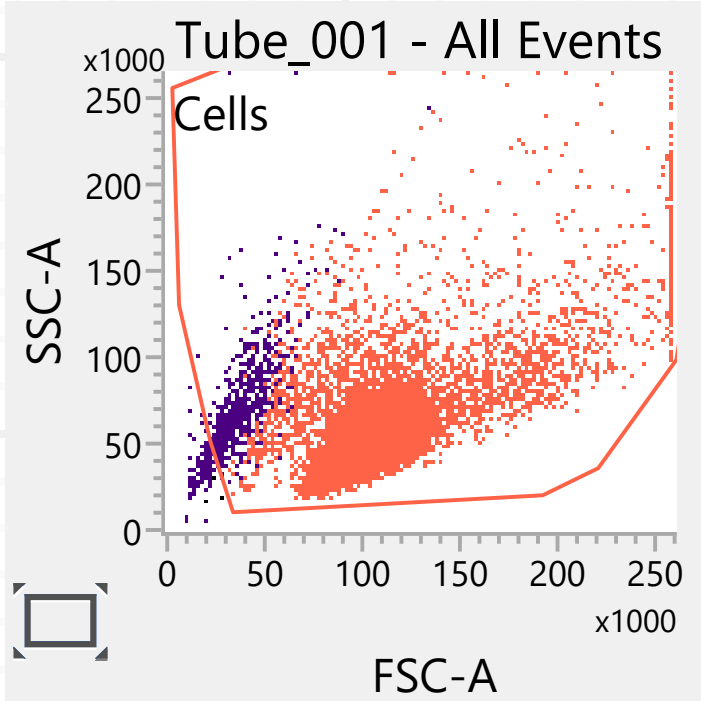

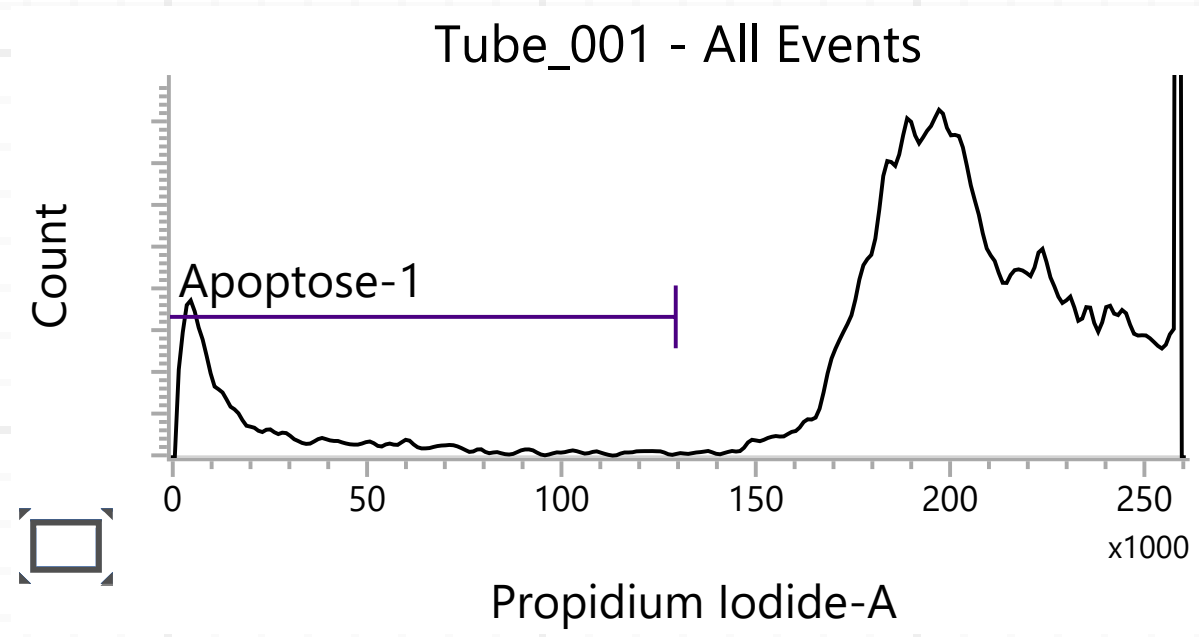

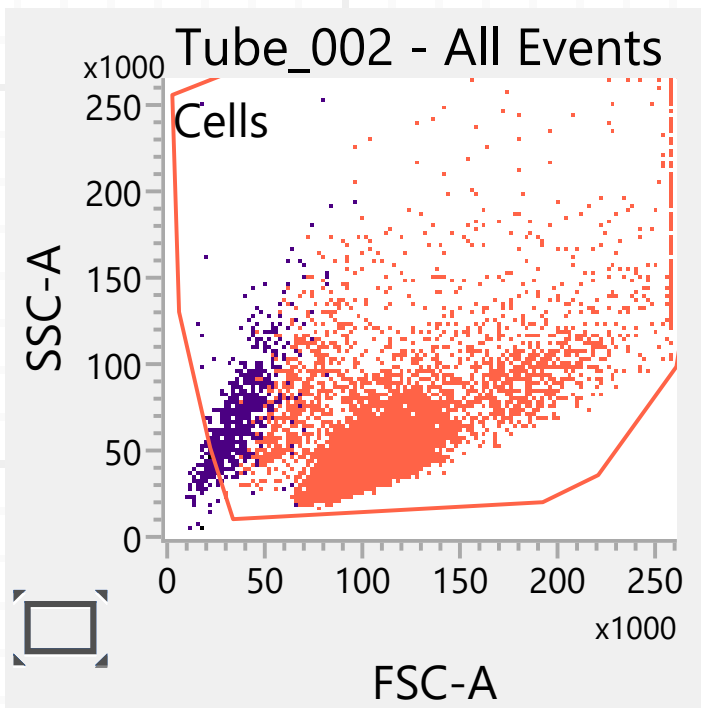

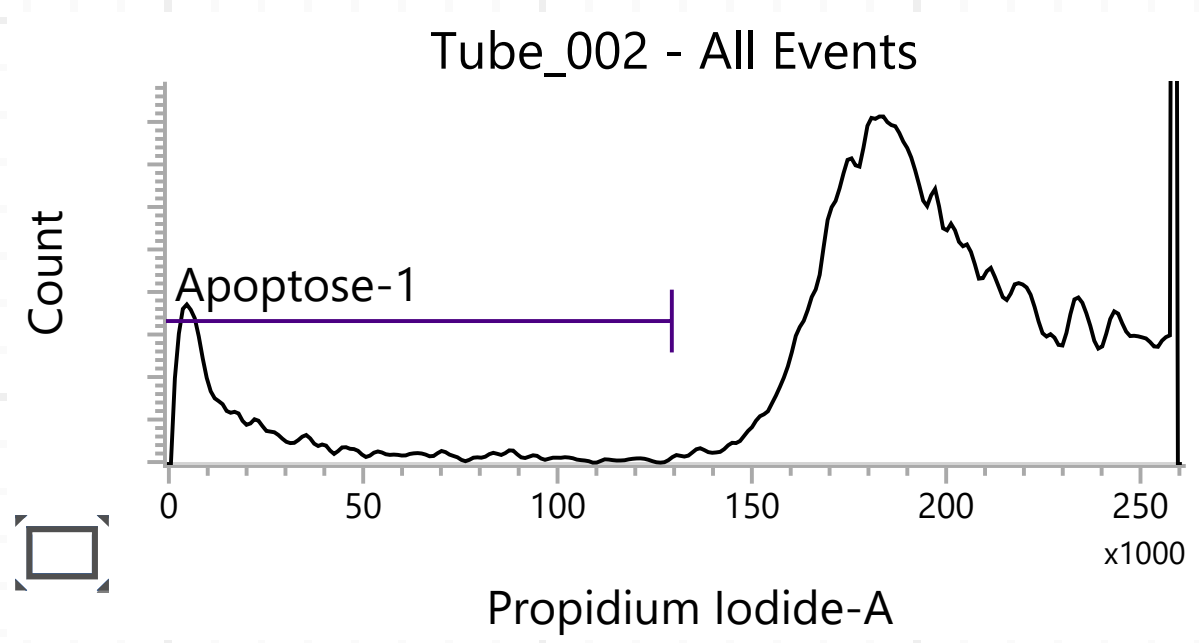

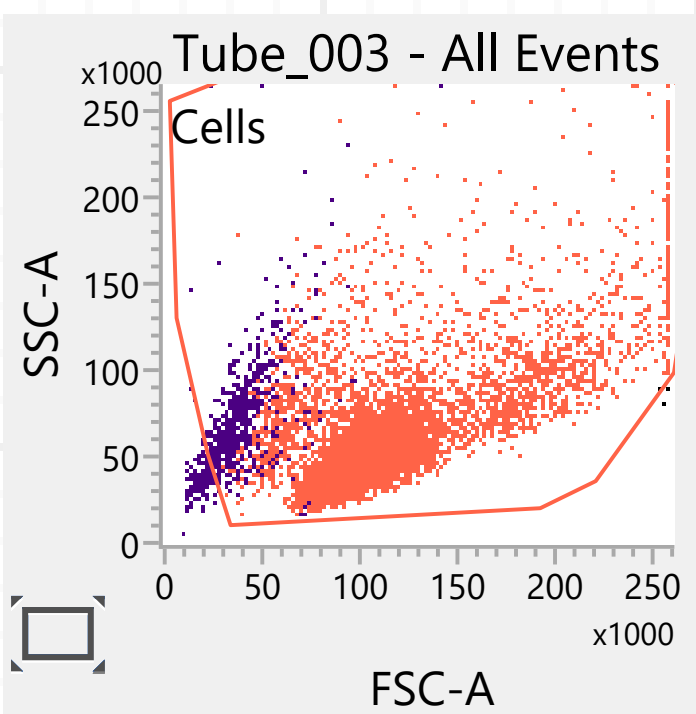

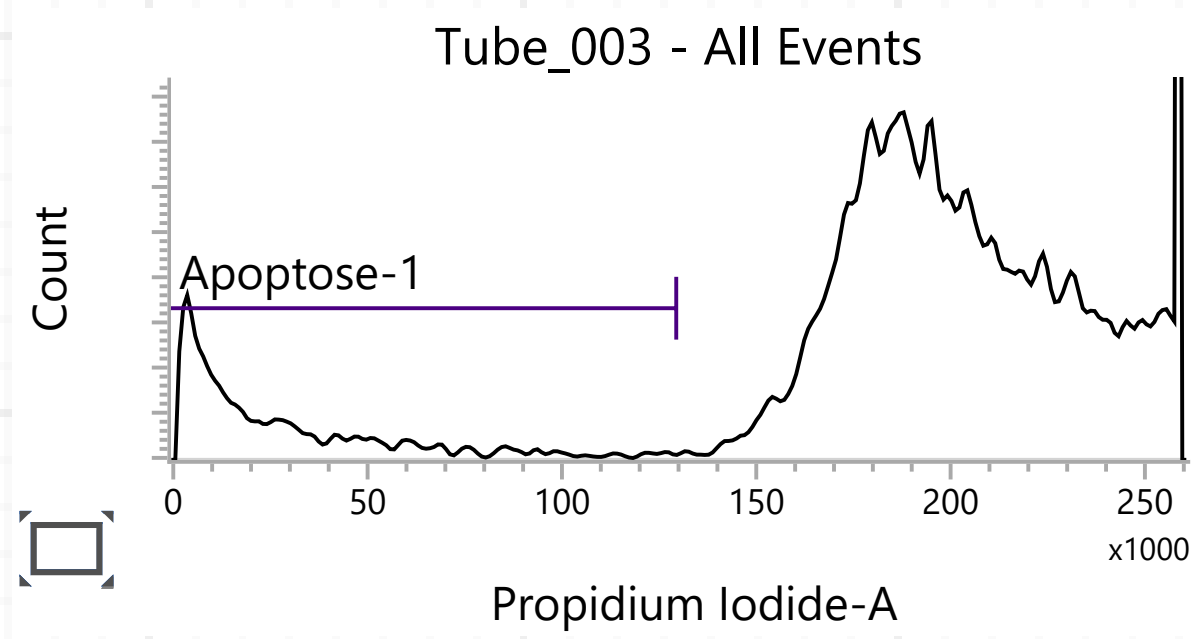

Statistics

Prozent apoptotische Zellen: Prozent apoptotische Zellen No Value % von Cells

Name	Events	% Total	% Parent	% Grandparent
Tube_001:All Events	10,116	100.00	***	***
Tube_001:Cells	10,000	98.85	98.85	***
Tube_002:All Events	10,000	100.00	***	***
Tube_002:Cells	9,896	98.96	98.96	***
Tube_003:All Events	10,000	100.00	***	***
Tube_003:Cells	9,882	98.82	98.82	***
Tube_001:Apoptose-1	675	6.67	6.67	***
Tube_002:Apoptose-1	709	7.09	7.09	***
Tube_003:Apoptose-1	729	7.29	7.29	***

DNA Fragmentierung

Sample ID: N6_5

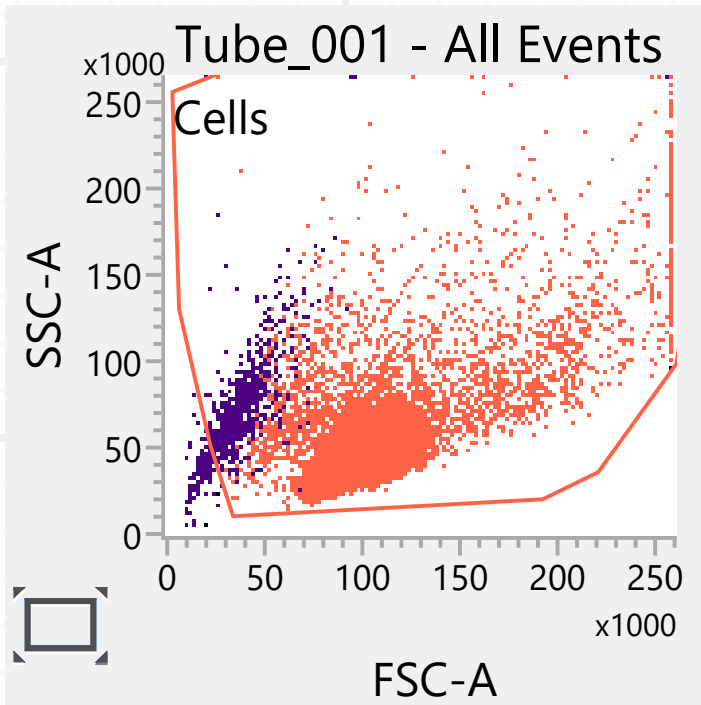

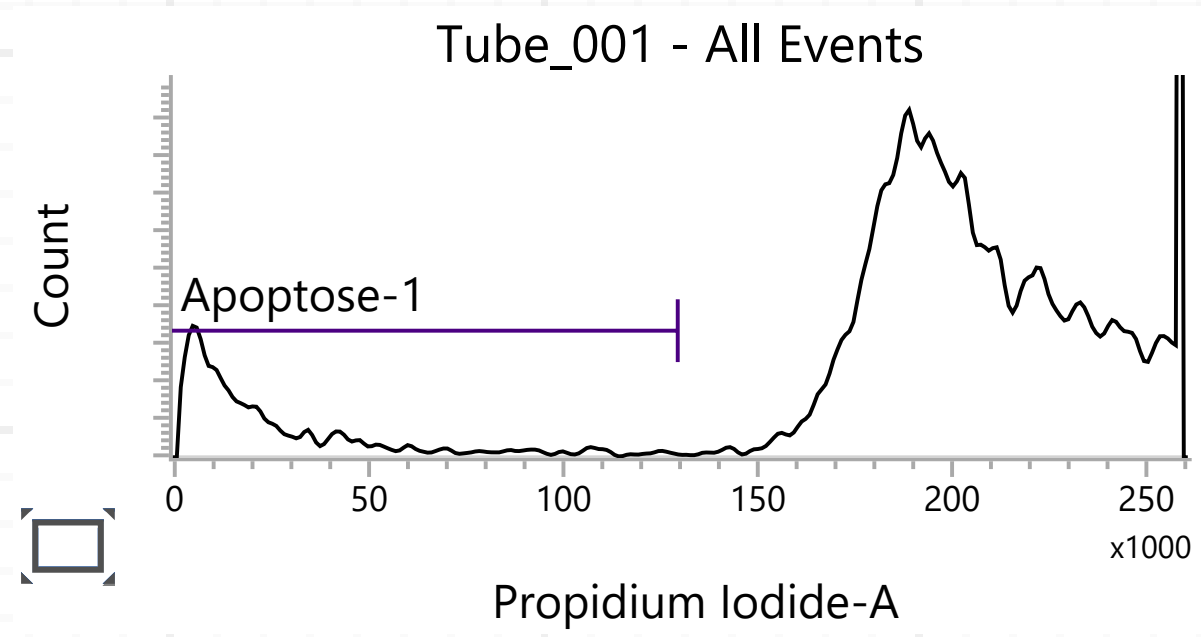

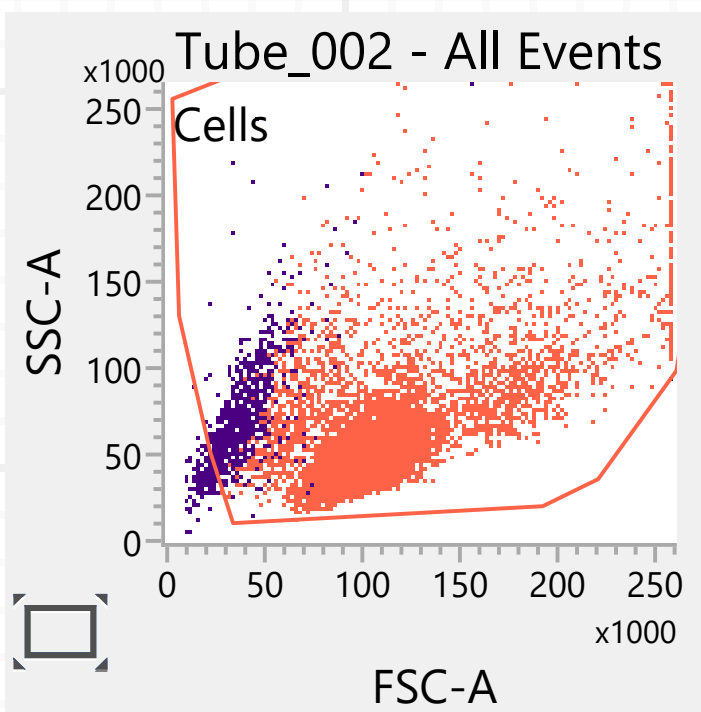

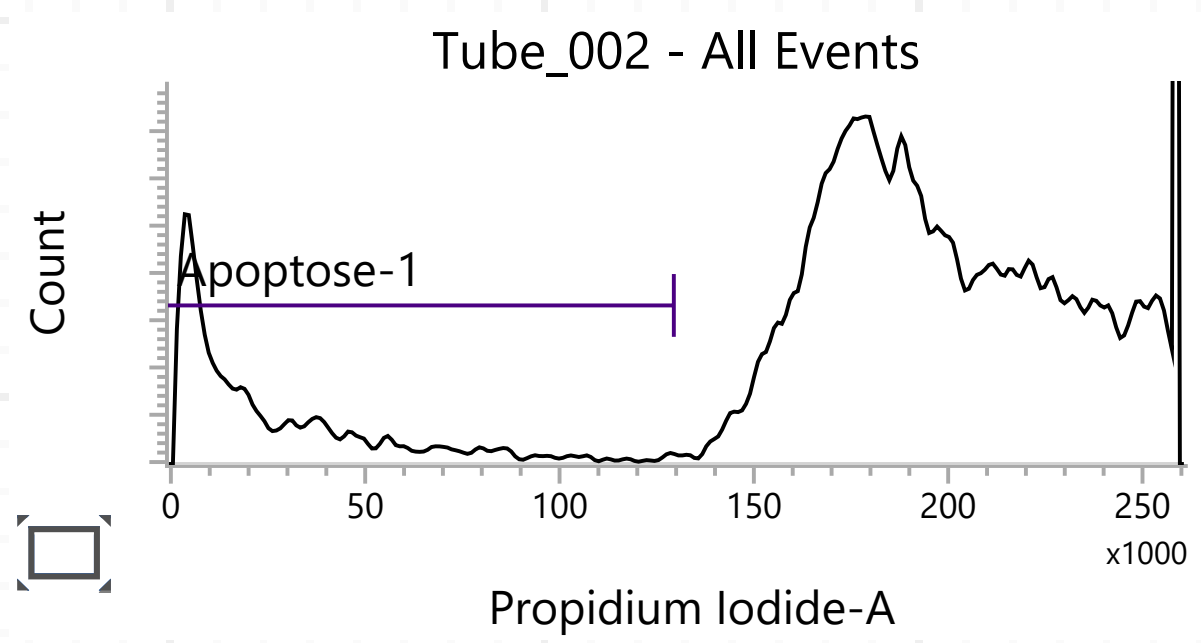

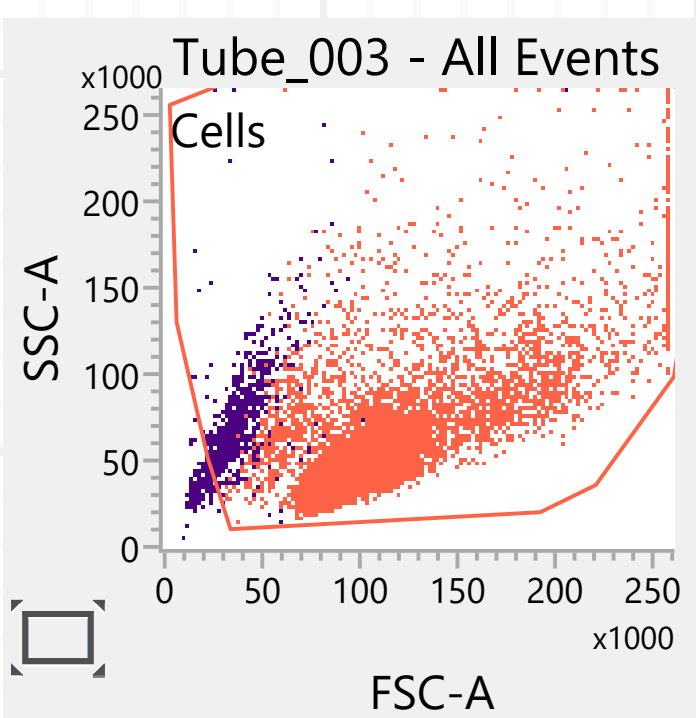

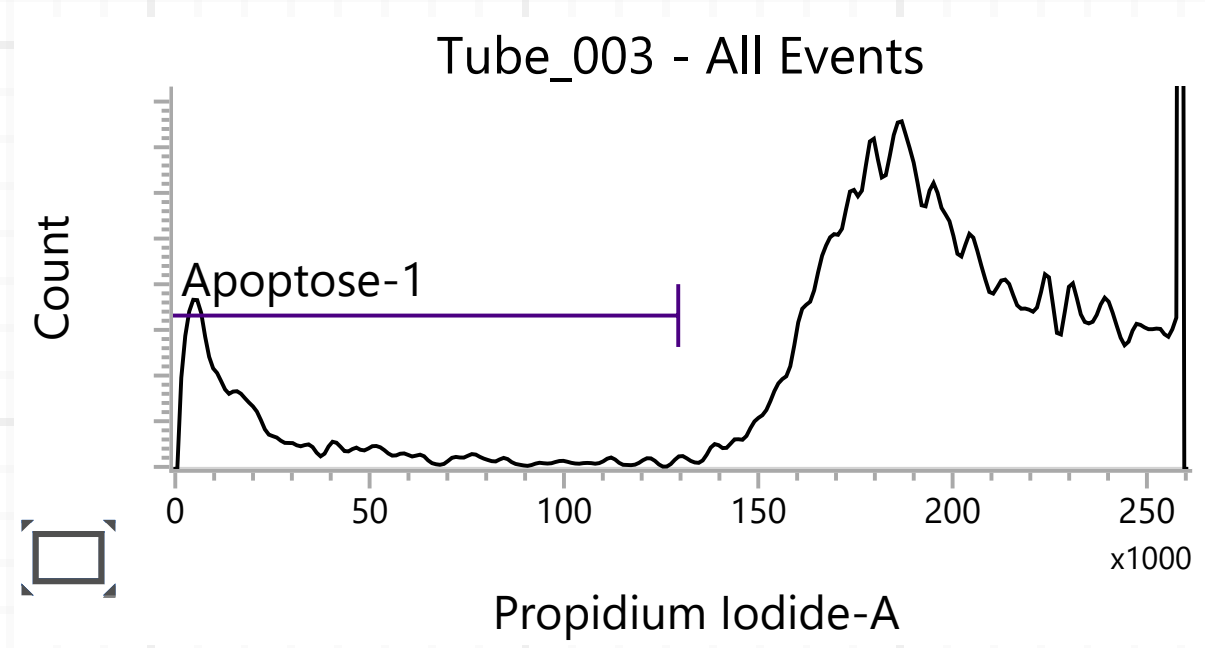

Statistics

Prozent apoptotische Zellen: Prozent apoptotische Zellen No Value % von Cells

Name	Events	% Total	% Parent	% Grandparent
Tube_001:All Events	10,131	100.00	***	***
Tube_001:Cells	10,000	98.71	98.71	***
Tube_002:All Events	10,000	100.00	***	***
Tube_002:Cells	9,852	98.52	98.52	***
Tube_003:All Events	10,000	100.00	***	***
Tube_003:Cells	9,886	98.86	98.86	***
Tube_001:Apoptose-1	758	7.48	7.48	***
Tube_002:Apoptose-1	957	9.57	9.57	***
Tube_003:Apoptose-1	769	7.69	7.69	***

DNA Fragmentierung

Sample ID: N6_10

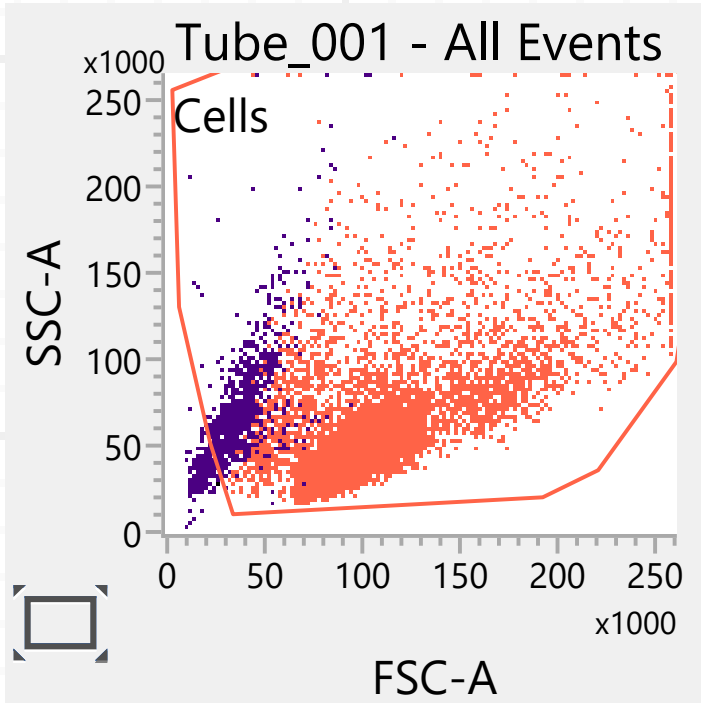

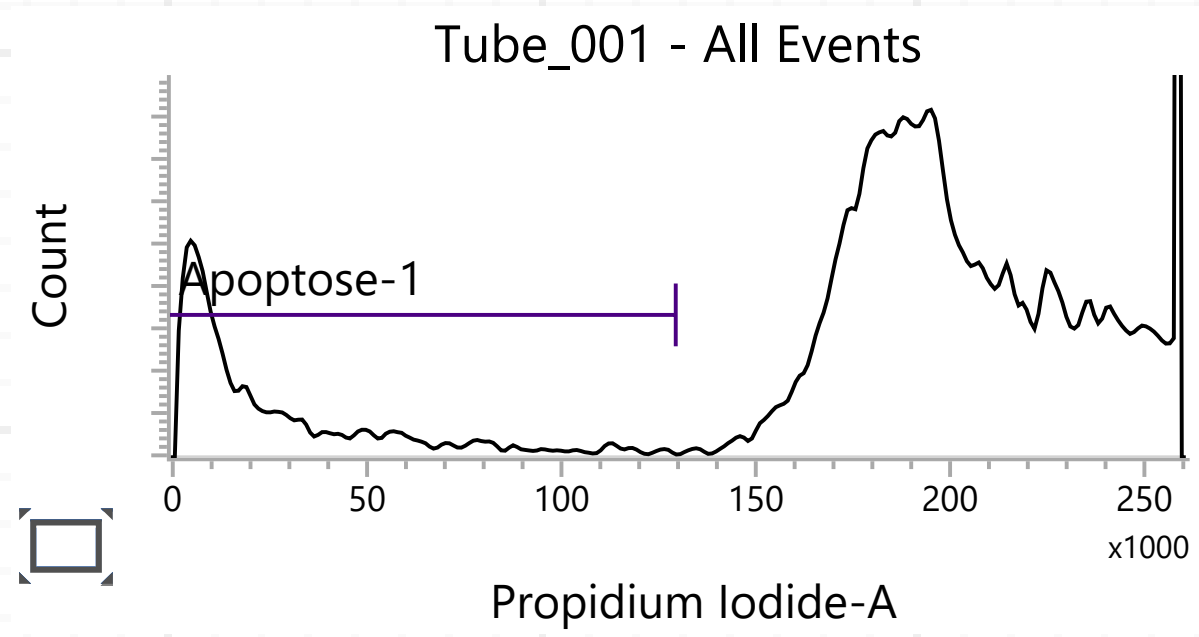

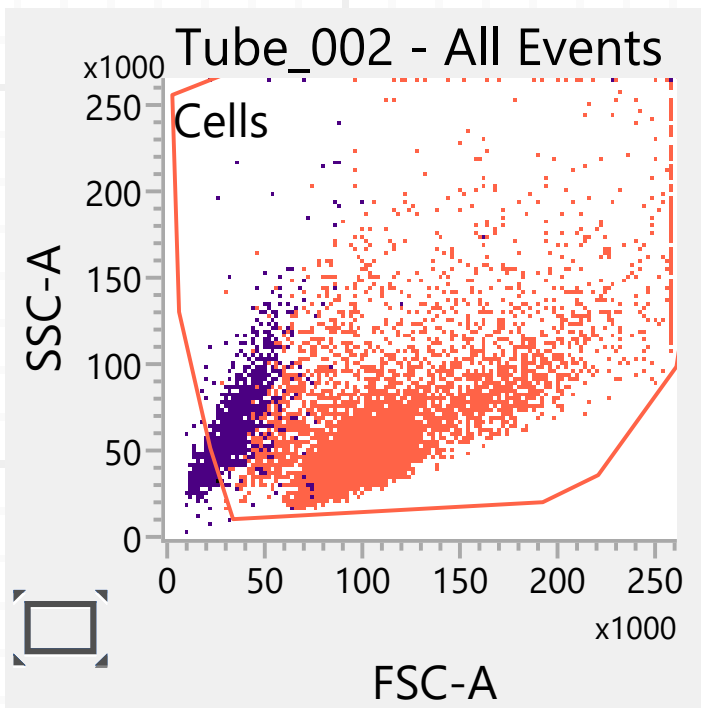

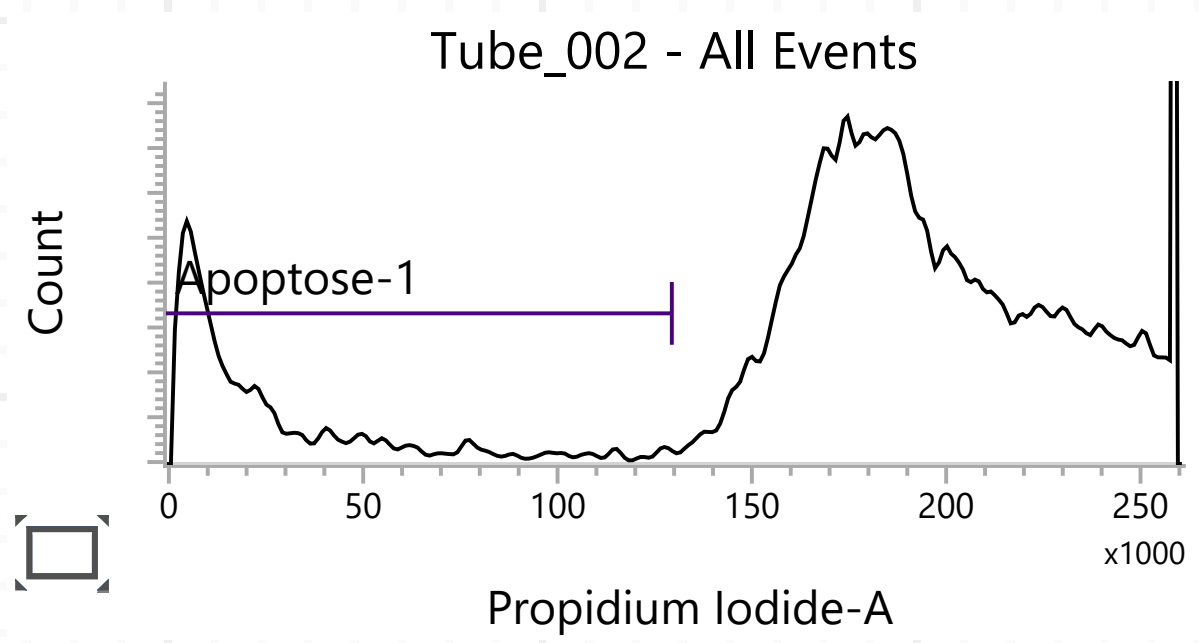

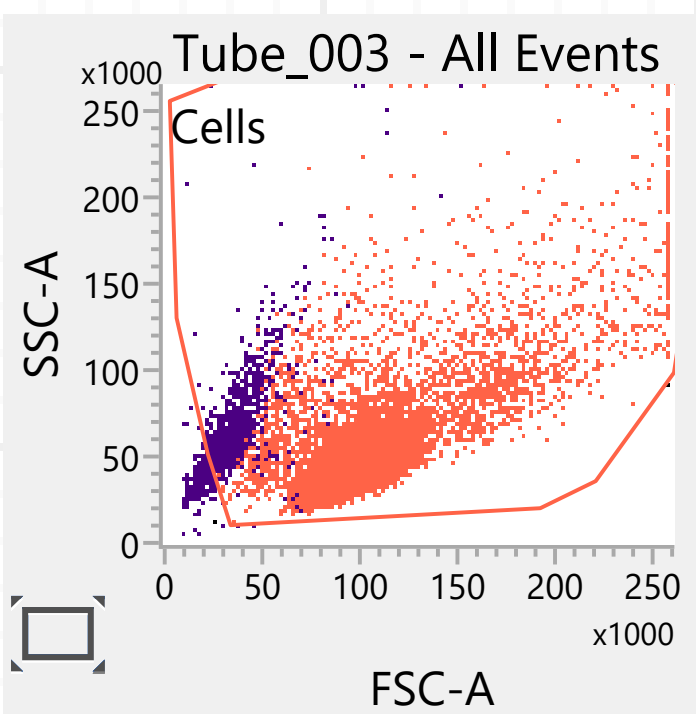

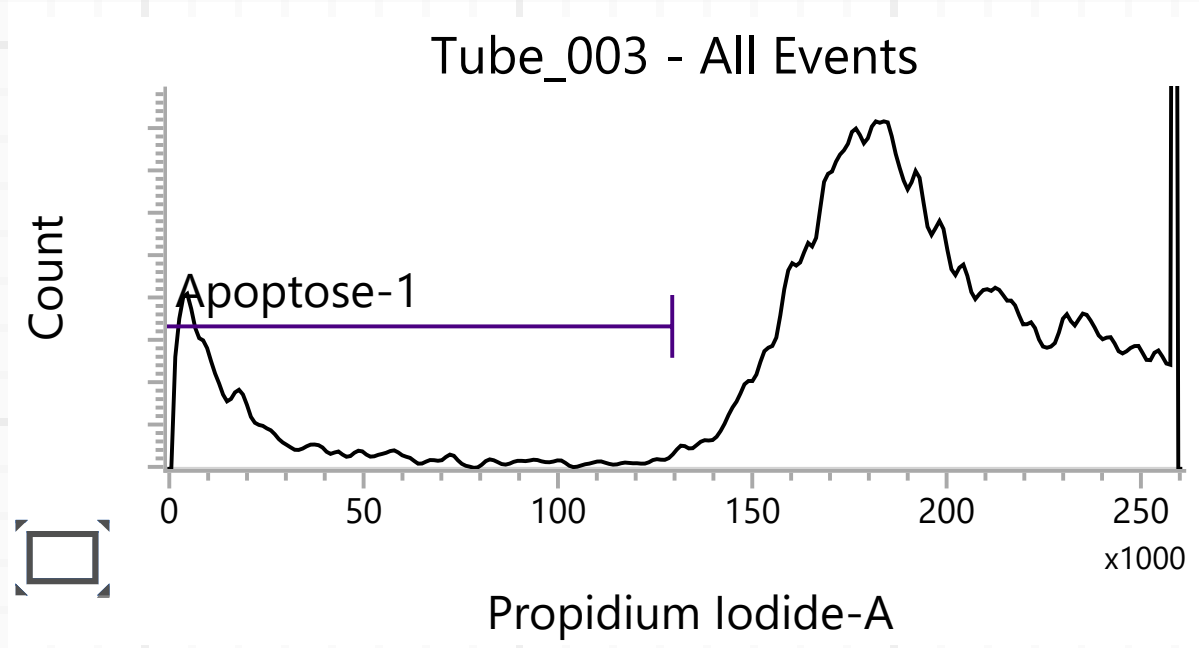

Statistics

Prozent apoptotische Zellen: Prozent apoptotische Zellen No Value % von Cells

Name	Events	% Total	% Parent	% Grandparent
Tube_001:All Events	10,195	100.00	***	***
Tube_001:Cells	10,000	98.09	98.09	***
Tube_002:All Events	10,000	100.00	***	***
Tube_002:Cells	9,813	98.13	98.13	***
Tube_003:All Events	10,000	100.00	***	***
Tube_003:Cells	9,850	98.50	98.50	***
Tube_001:Apoptose-1	1,057	10.37	10.37	***
Tube_002:Apoptose-1	1,091	10.91	10.91	***
Tube_003:Apoptose-1	842	8.42	8.42	***

DNA Fragmentierung

Sample ID: N6_20

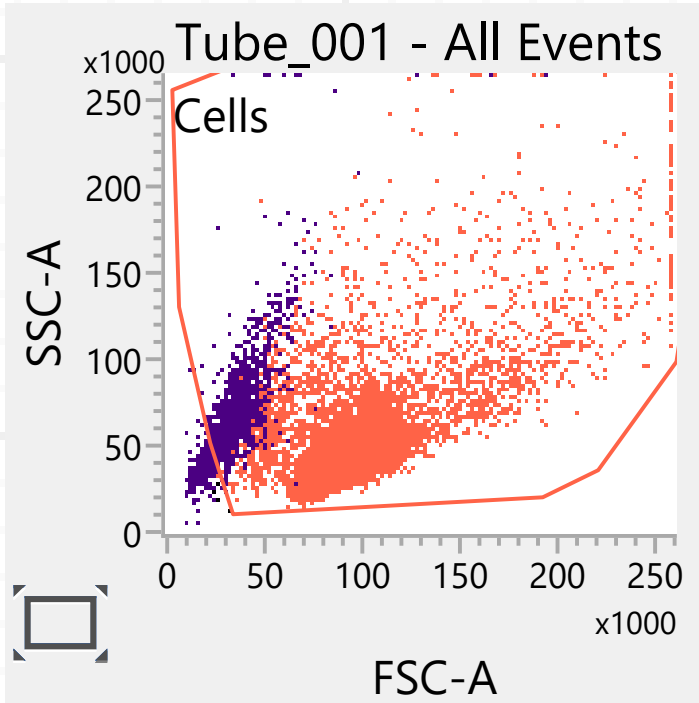

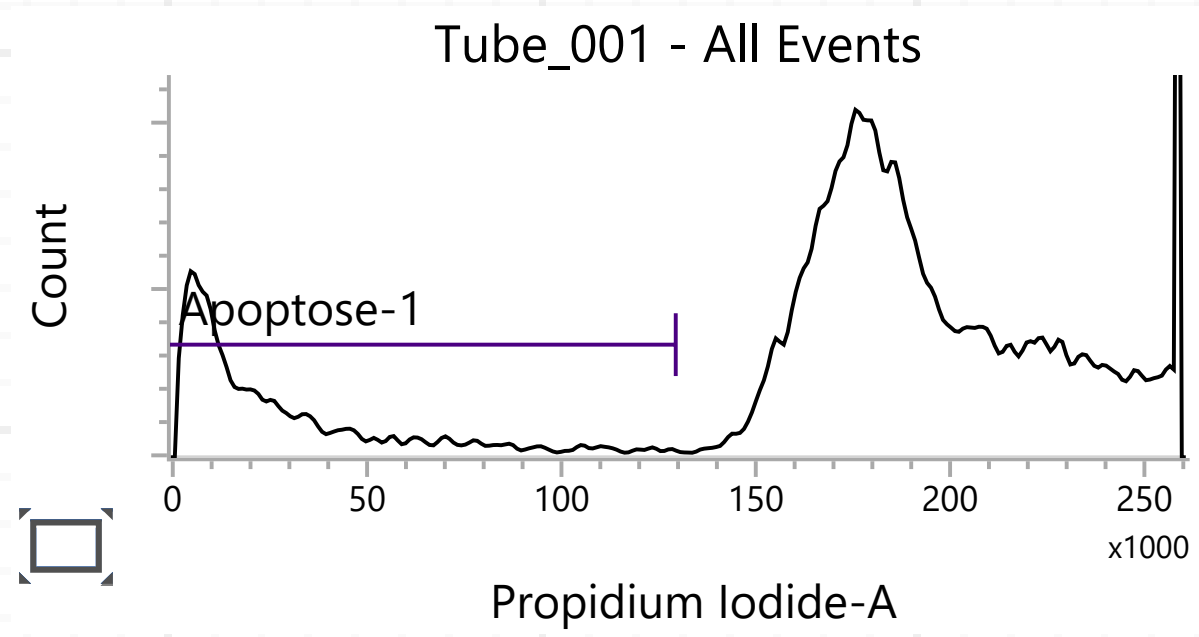

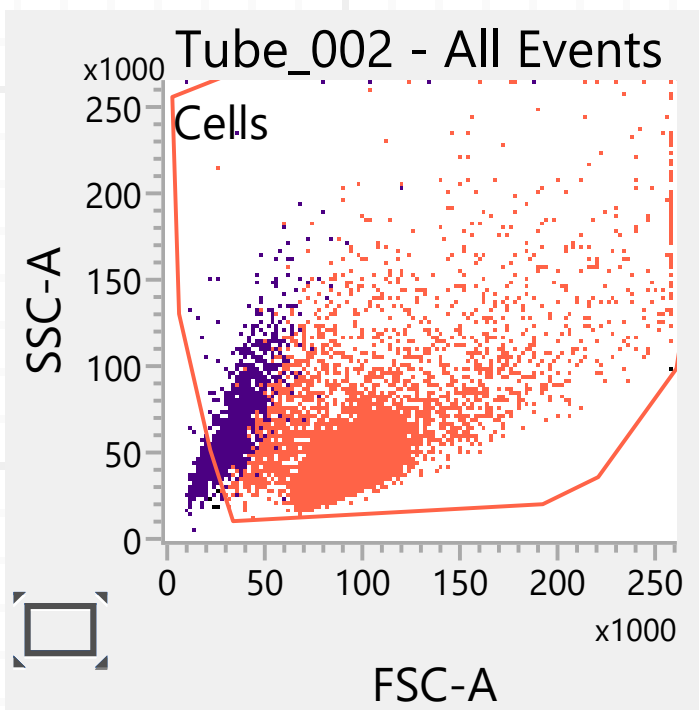

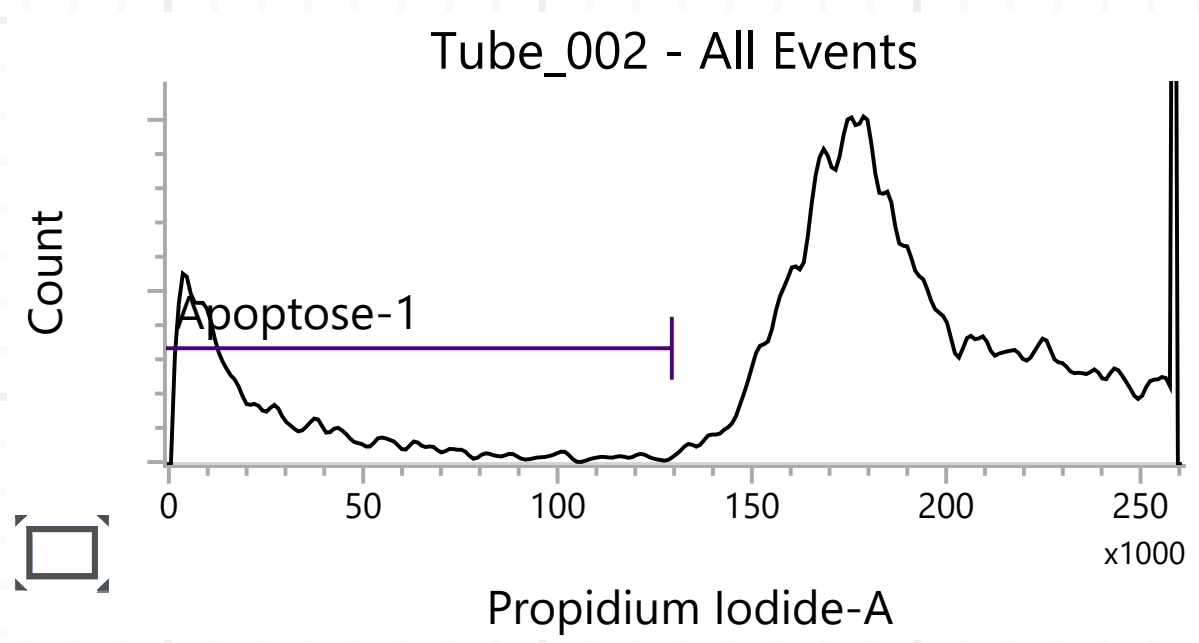

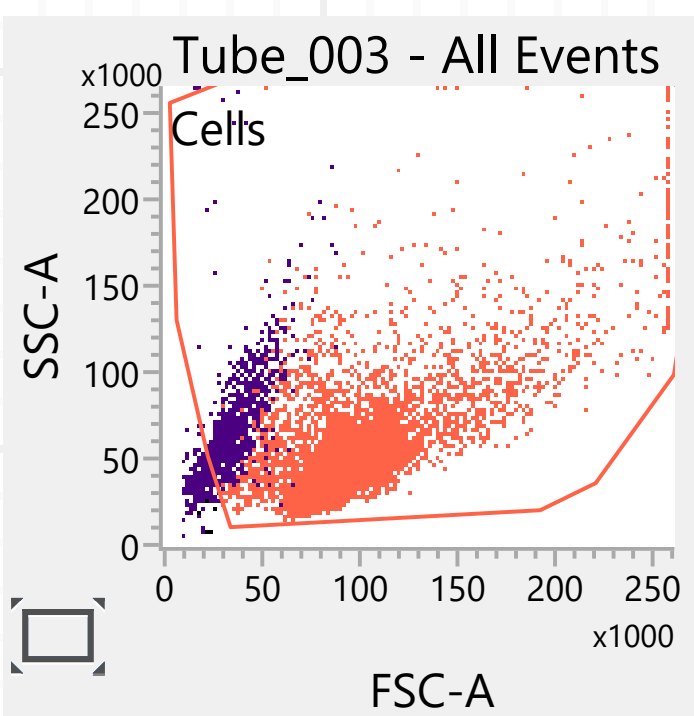

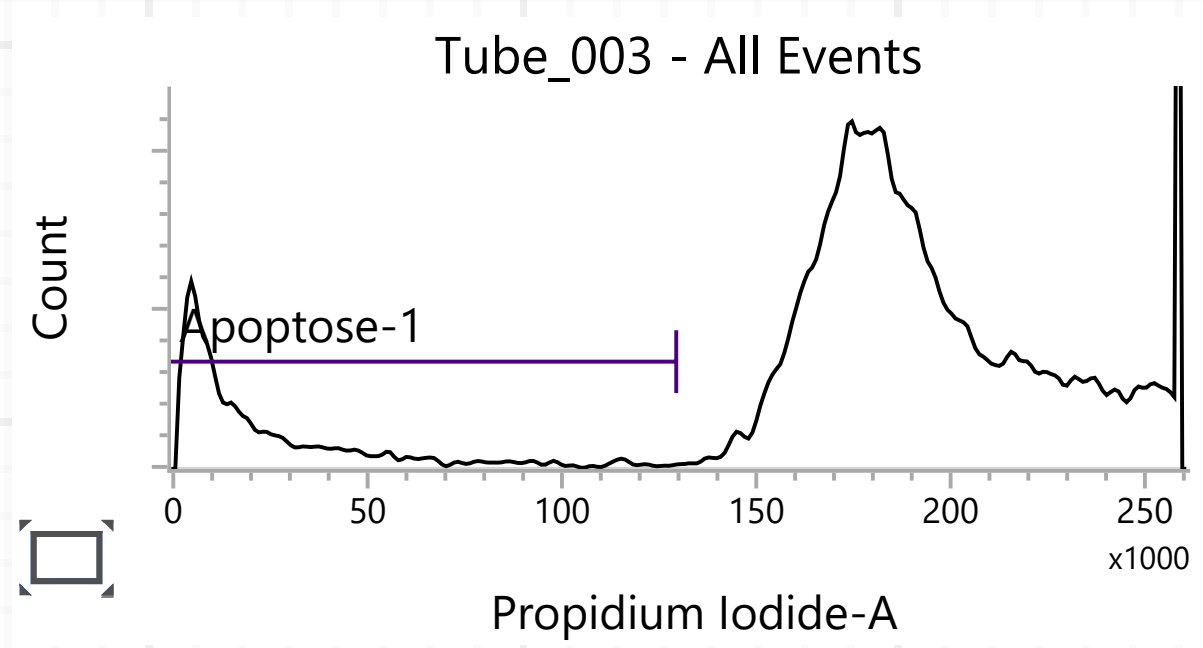

Statistics

Prozent apoptotische Zellen: Prozent apoptotische Zellen No Value % von Cells

Name	Events	% Total	% Parent	% Grandparent
Tube_001:All Events	10,226	100.00	***	***
Tube_001:Cells	10,000	97.79	97.79	***
Tube_002:All Events	10,000	100.00	***	***
Tube_002:Cells	9,755	97.55	97.55	***
Tube_003:All Events	10,000	100.00	***	***
Tube_003:Cells	9,812	98.12	98.12	***
Tube_001:Apoptose-1	1,329	13.00	13.00	***
Tube_002:Apoptose-1	1,334	13.34	13.34	***
Tube_003:Apoptose-1	1,032	10.32	10.32	***

DNA Fragmentierung

Sample ID: N6_D

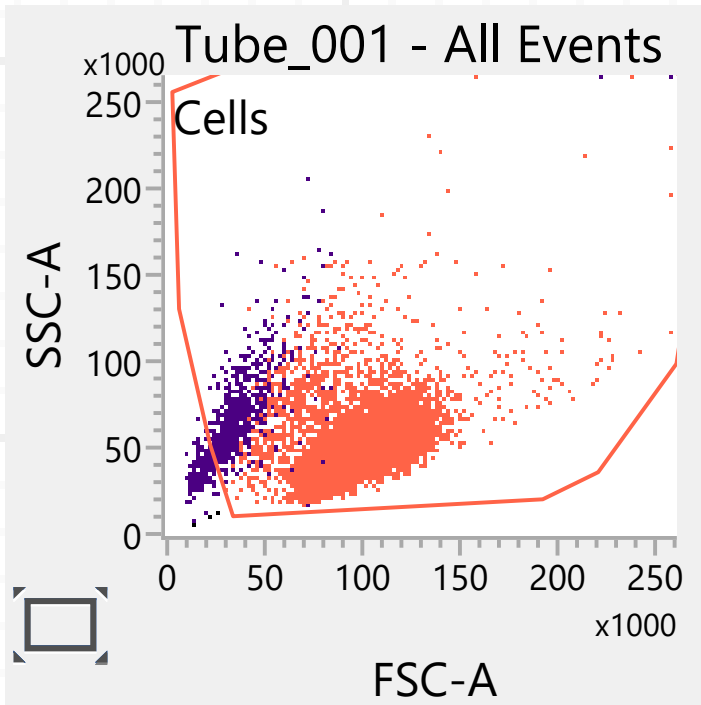

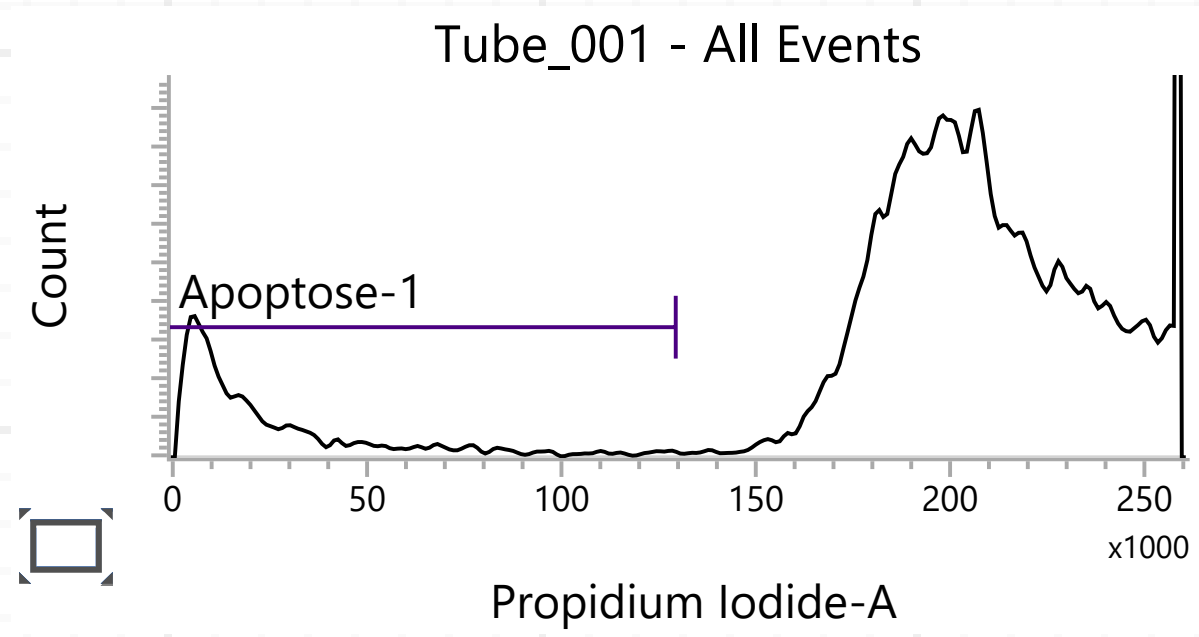

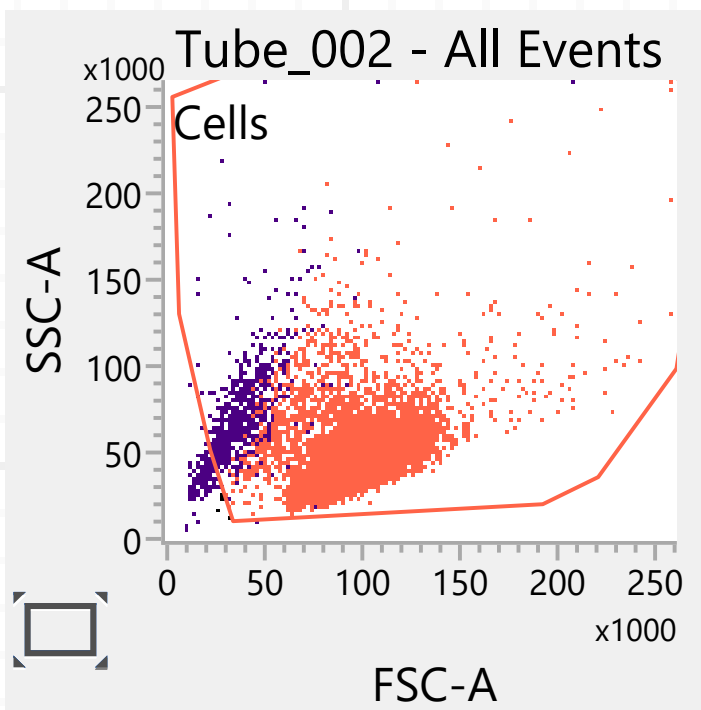

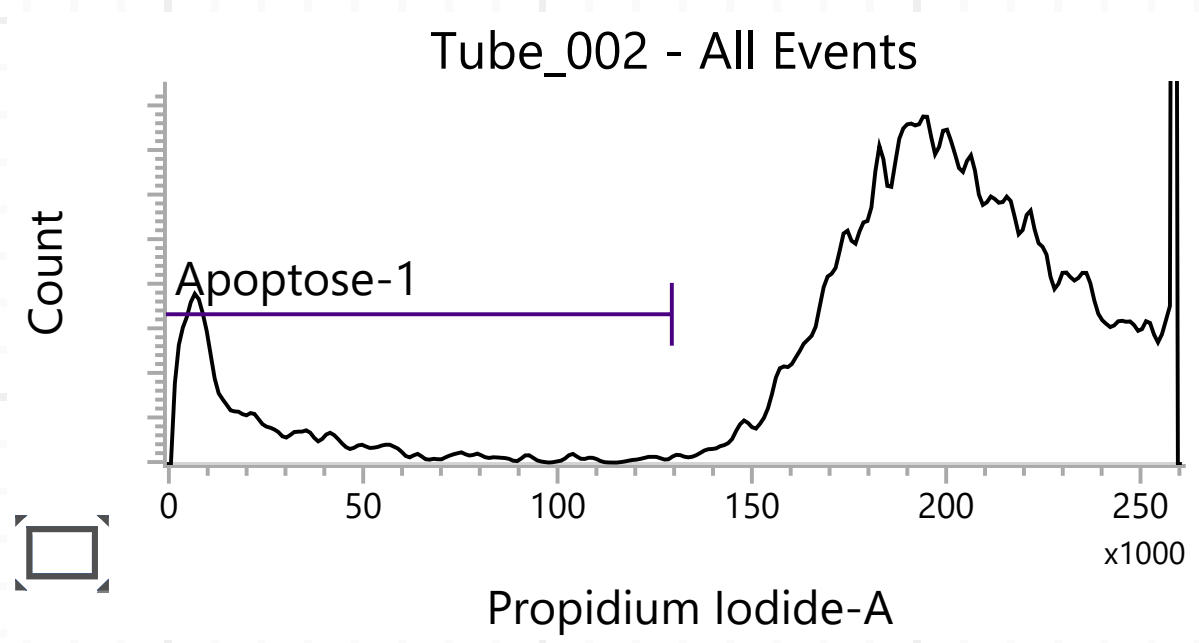

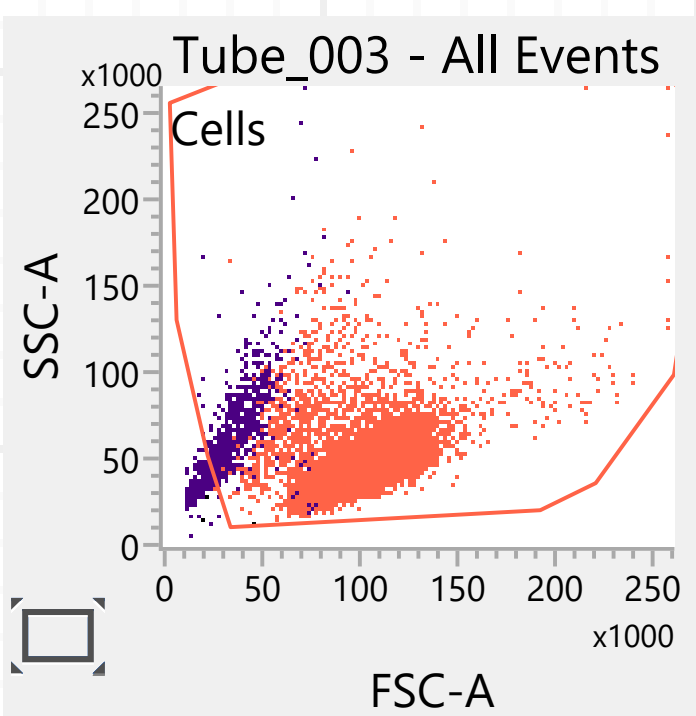

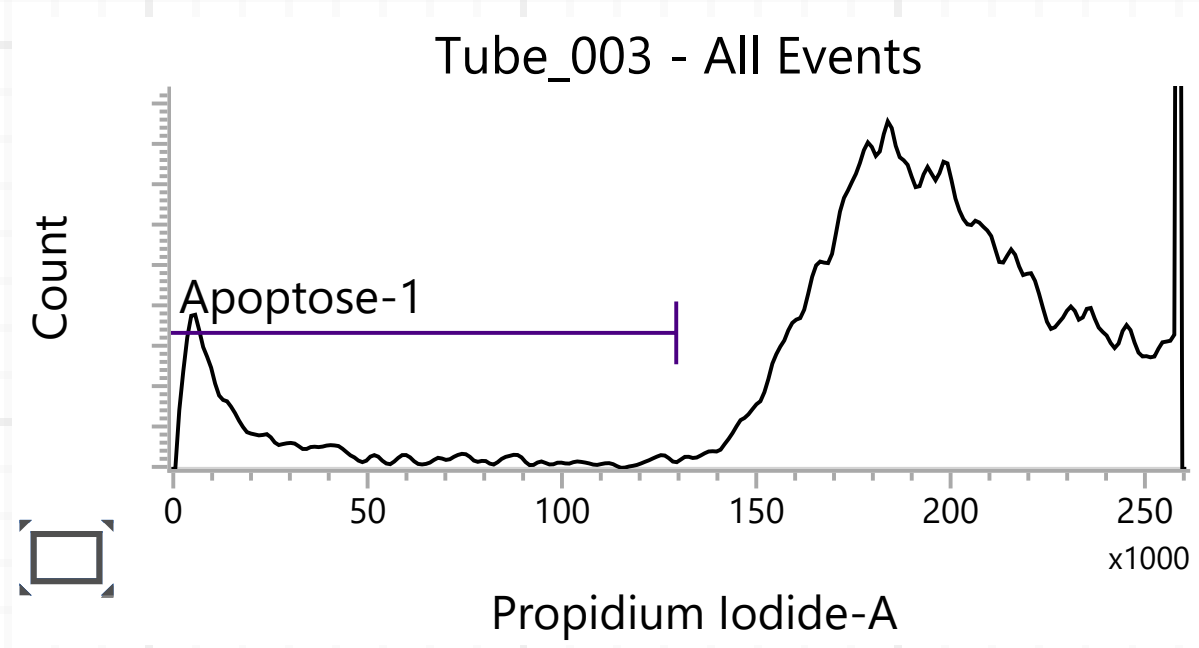

Statistics

Prozent apoptotische Zellen: Prozent apoptotische Zellen No Value % von Cells

Name	Events	% Total	% Parent	% Grandparent
Tube_001:All Events	10,155	100.00	***	***
Tube_001:Cells	10,000	98.47	98.47	***
Tube_002:All Events	10,000	100.00	***	***
Tube_002:Cells	9,872	98.72	98.72	***
Tube_003:All Events	10,000	100.00	***	***
Tube_003:Cells	9,866	98.66	98.66	***
Tube_001:Apoptose-1	767	7.55	7.55	***
Tube_002:Apoptose-1	768	7.68	7.68	***
Tube_003:Apoptose-1	736	7.36	7.36	***

DNA Fragmentierung

Sample ID: N6_K

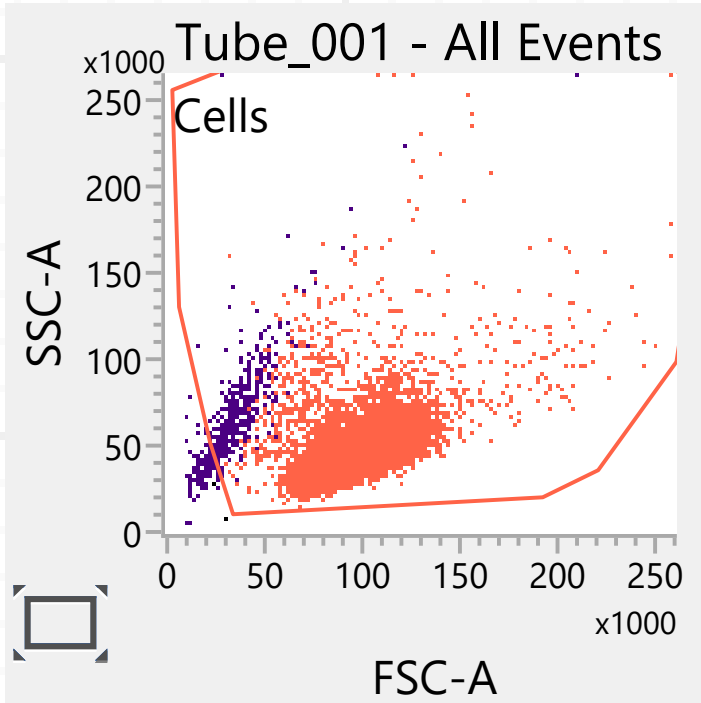

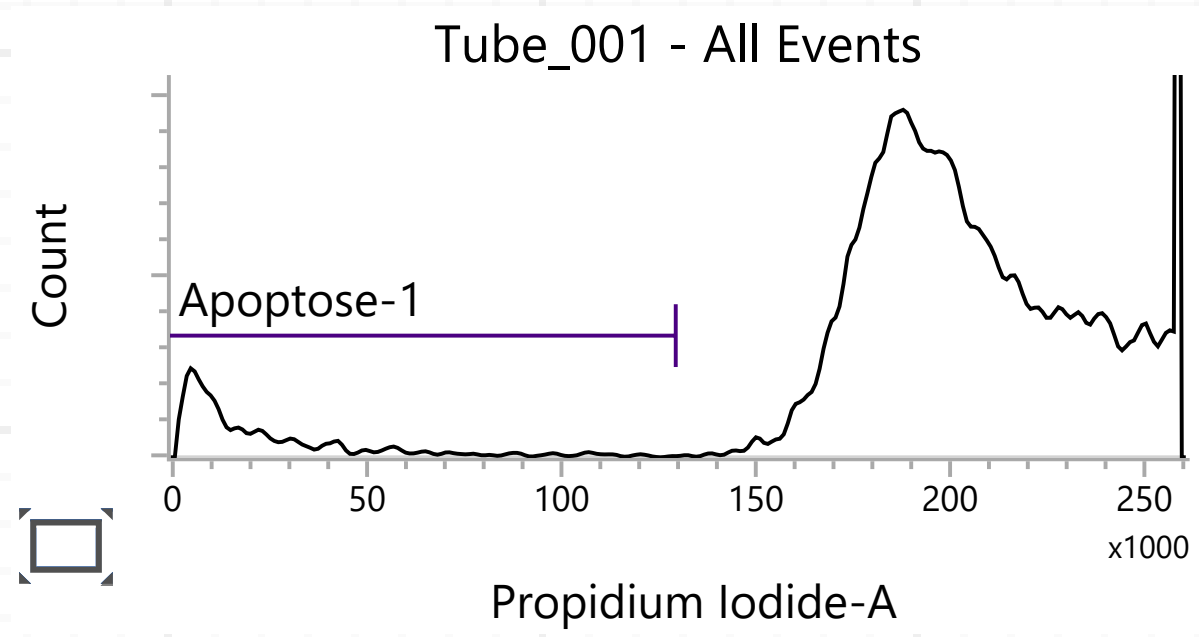

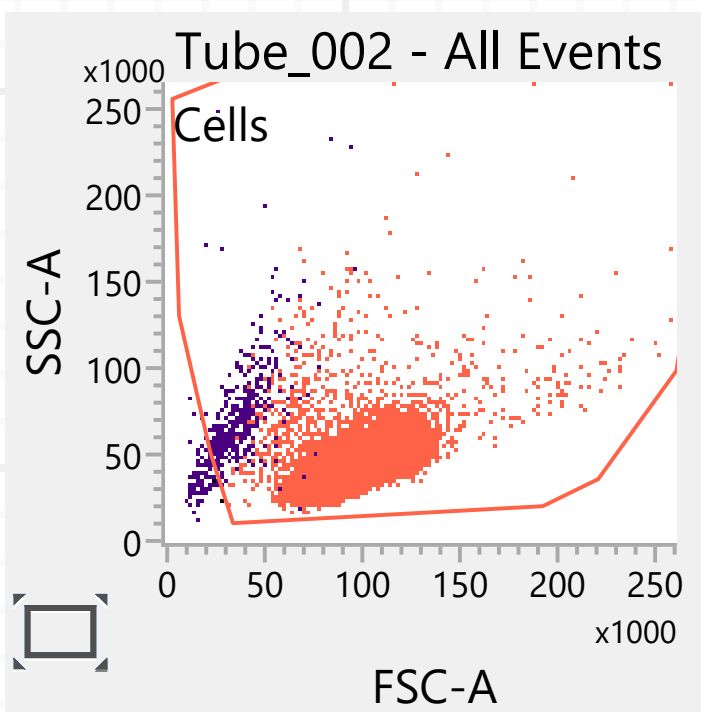

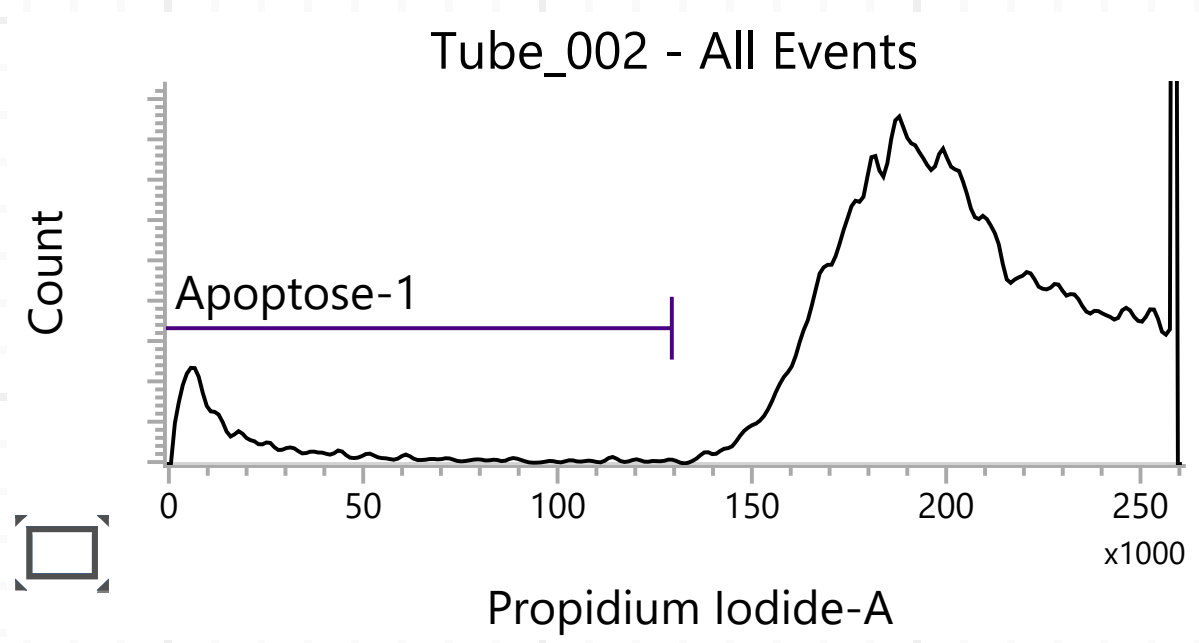

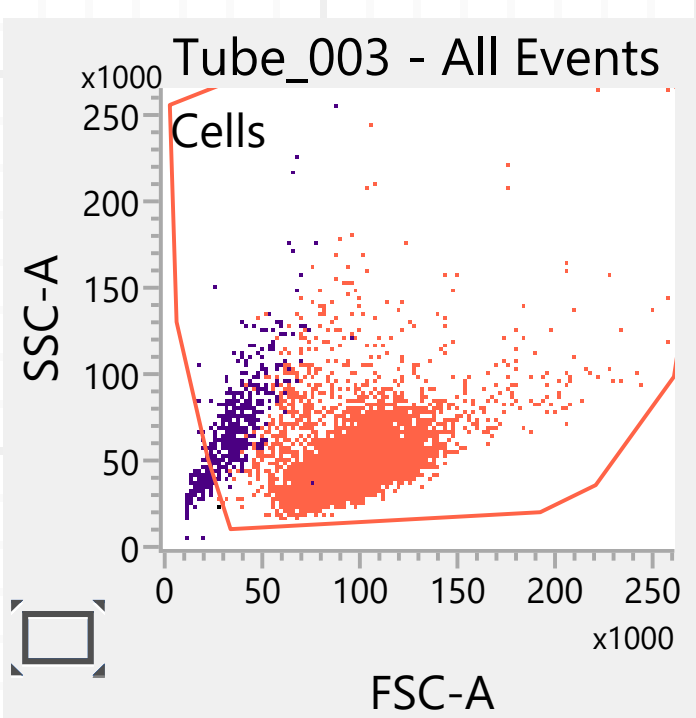

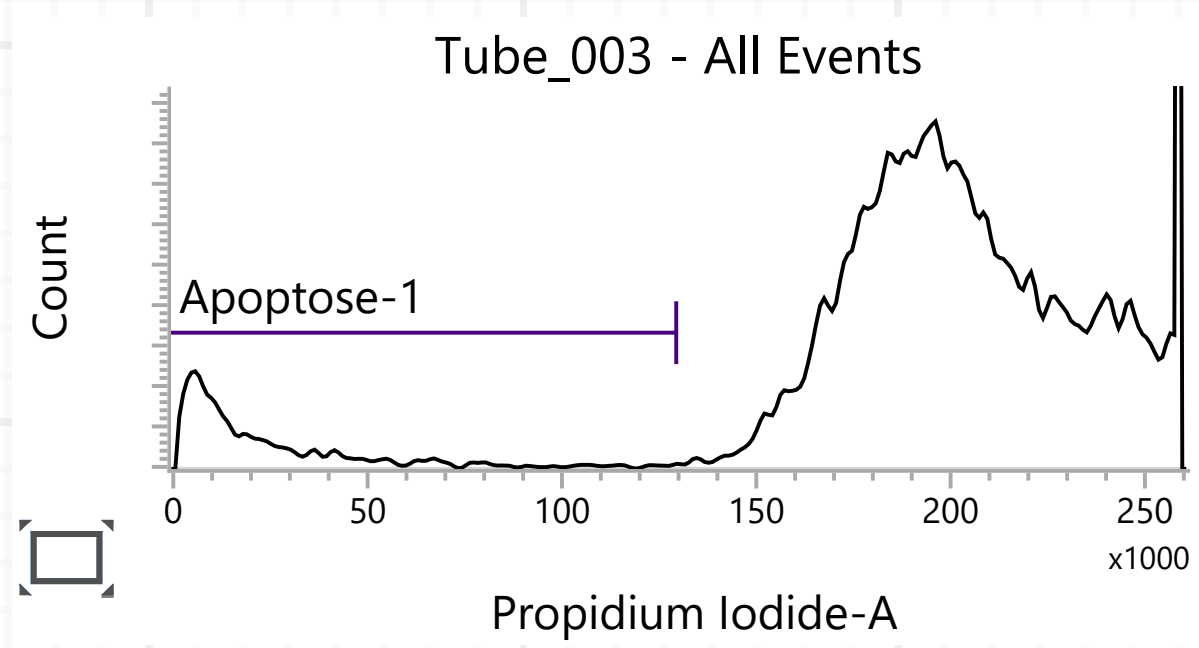

Statistics

Prozent apoptotische Zellen: Prozent apoptotische Zellen No Value % von Cells

Name	Events	% Total	% Parent	% Grandparent	
Tube_001:All Events	10,093	100.00	***	***	
Tube_001:Cells	10,000	99.08	99.08	***	
Tube_002:All Events	10,000	100.00	***	***	
Tube_002:Cells	9,925	99.25	99.25	***	
Tube_003:All Events	10,000	100.00	***	***	
Tube_003:Cells	9,917	99.17	99.17	***	
Tube_001:Apoptose-1	469	4.65	4.65	***	
Tube_002:Apoptose-1	453	4.53	4.53	***	
Tube_003:Apoptose-1	509	5.09	5.09	***	

DNA Fragmentierung

Sample ID: 0,1

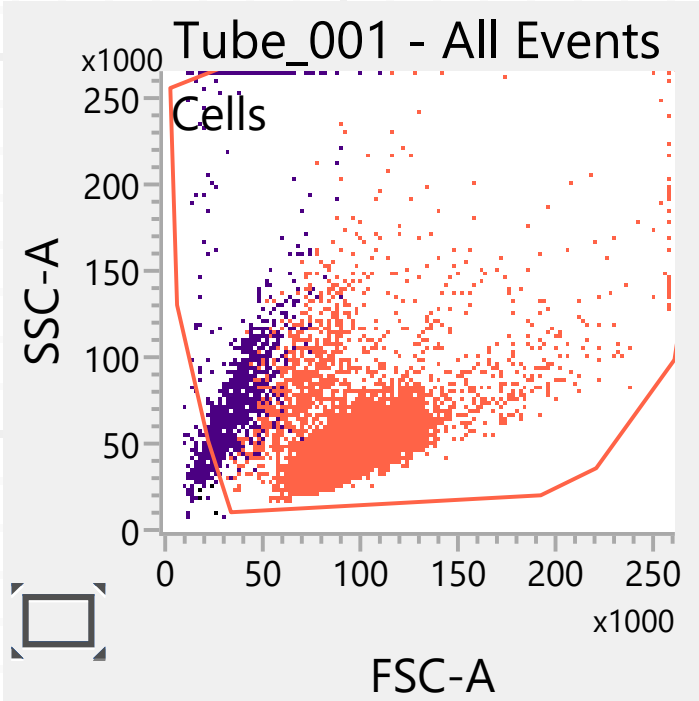

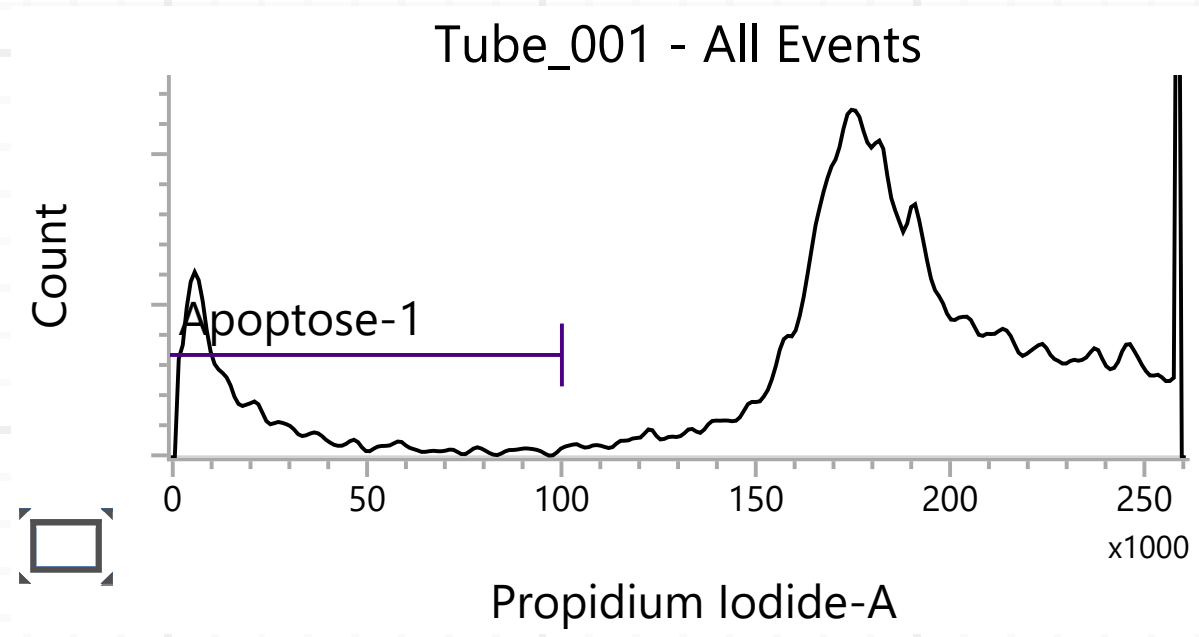

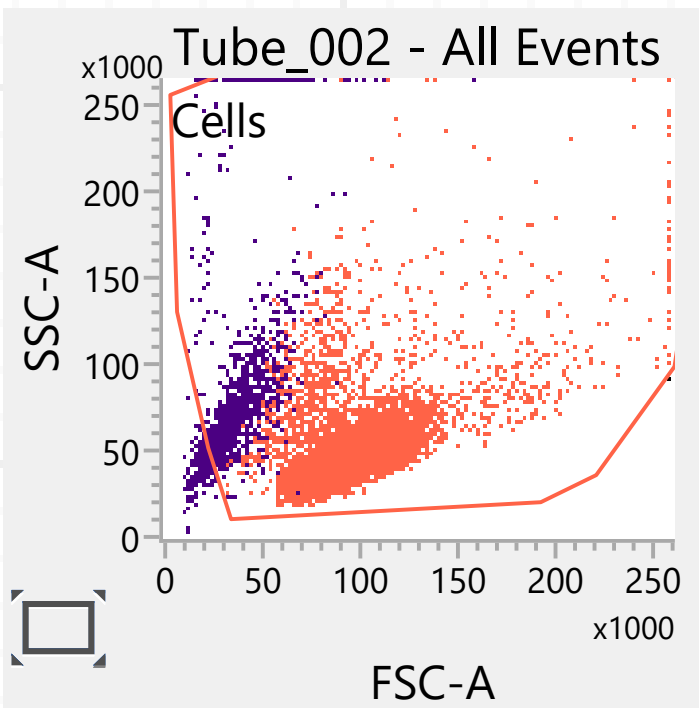

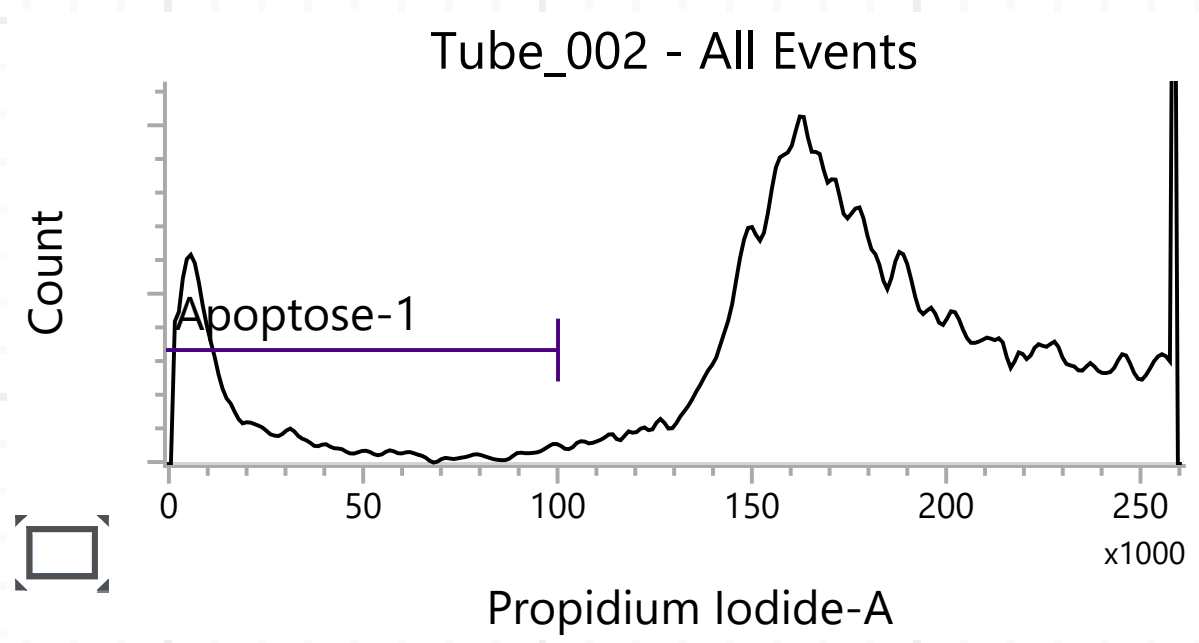

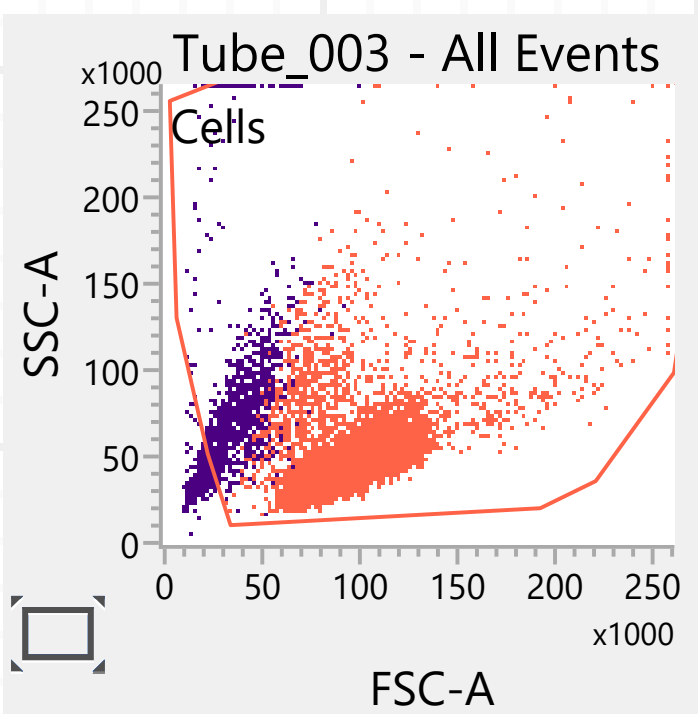

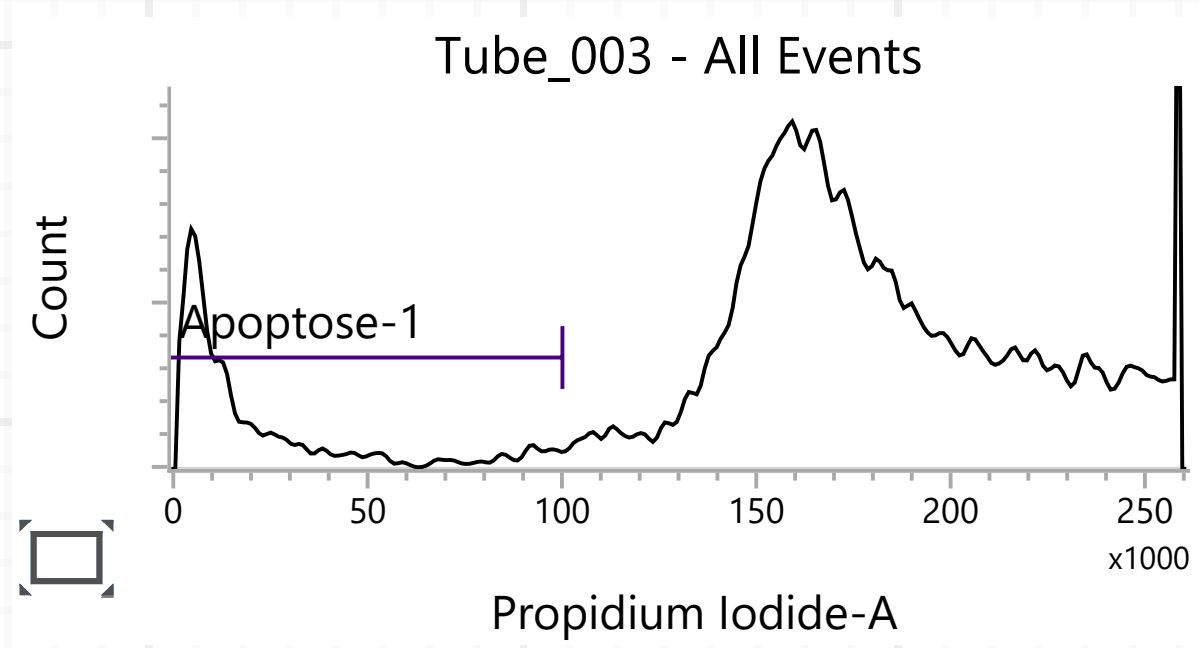

Statistics

Prozent apoptotische Zellen: Prozent apoptotische Zellen No Value % von Cells

Name	Events	% Total	% Parent	% Grandparent
Tube_001:All Events	10,187	100.00	***	***
Tube_001:Cells	10,000	98.16	98.16	***
Tube_002:All Events	10,000	100.00	***	***
Tube_002:Cells	9,846	98.46	98.46	***
Tube_003:All Events	10,000	100.00	***	***
Tube_003:Cells	9,825	98.25	98.25	***
Tube_001:Apoptose-1	1,129	11.08	11.08	***
Tube_002:Apoptose-1	1,166	11.66	11.66	***
Tube_003:Apoptose-1	1,169	11.69	11.69	***

DNA Fragmentierung

Sample ID: 0,5

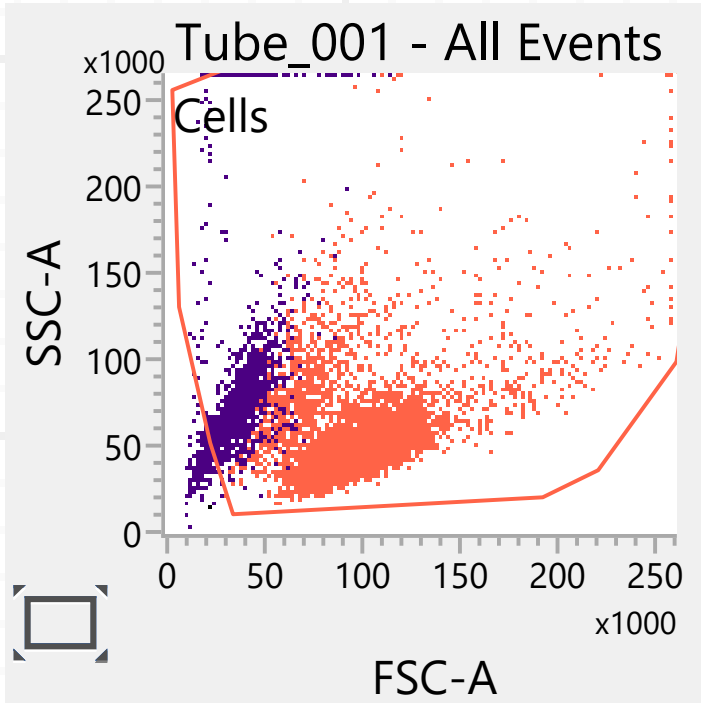

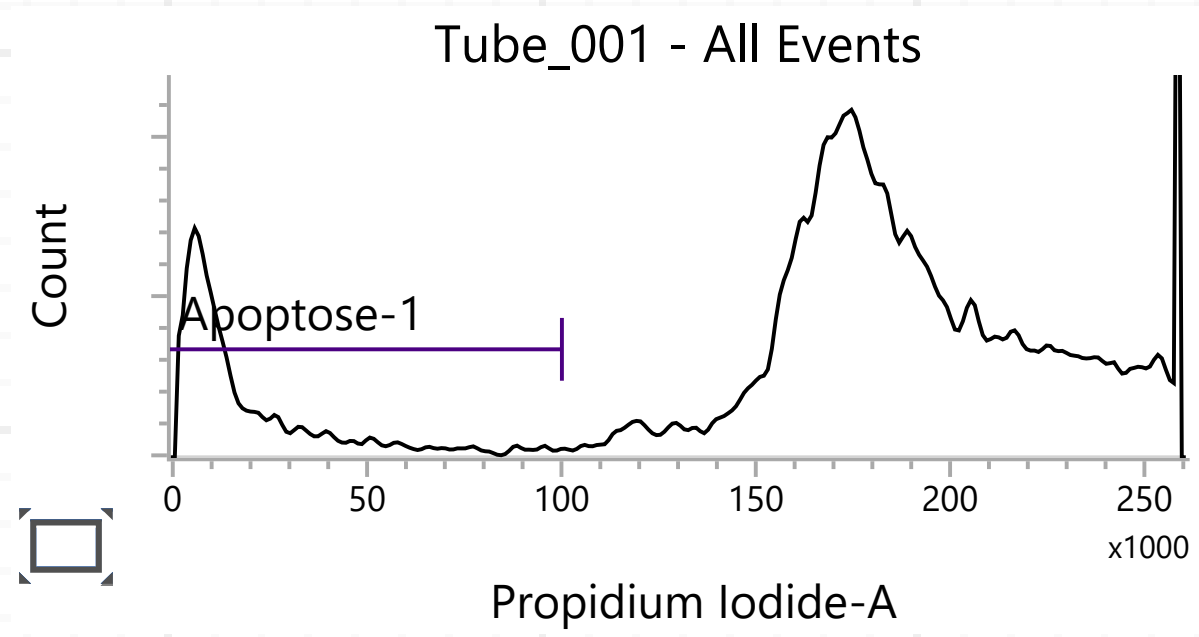

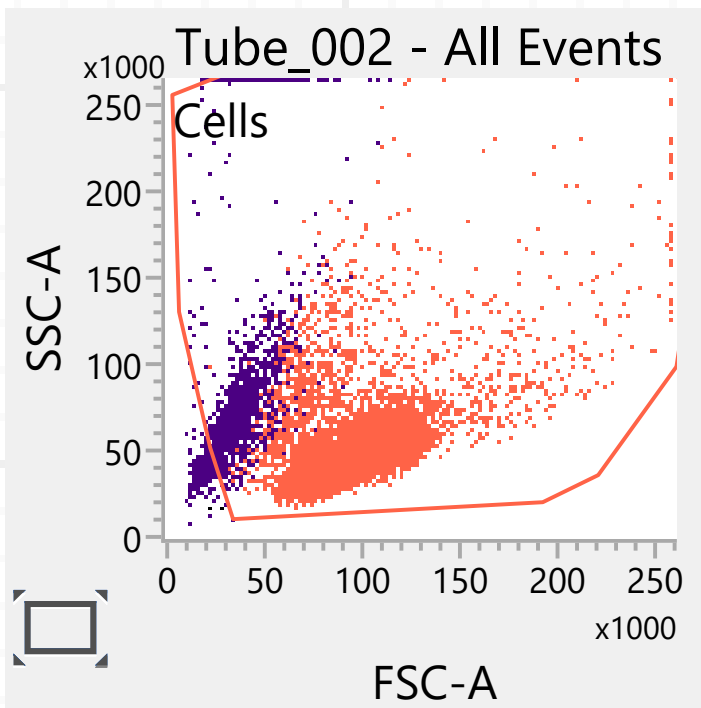

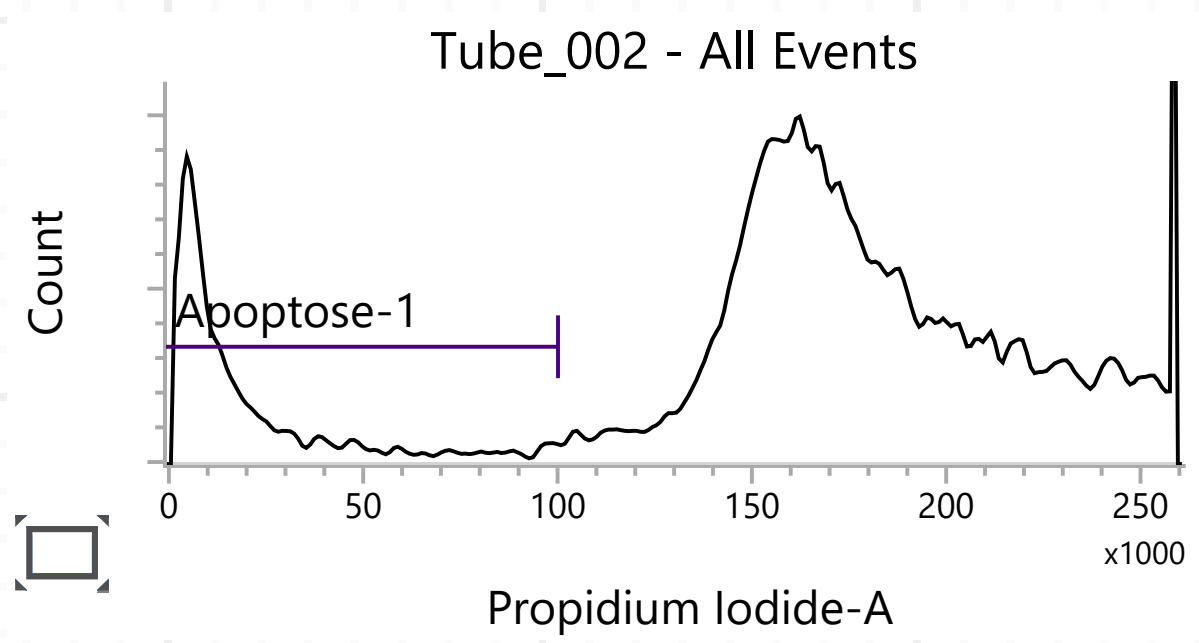

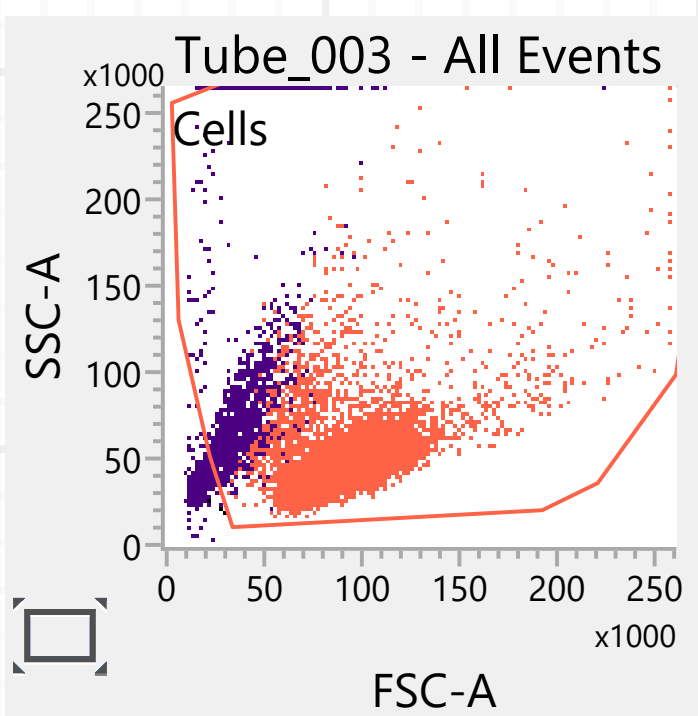

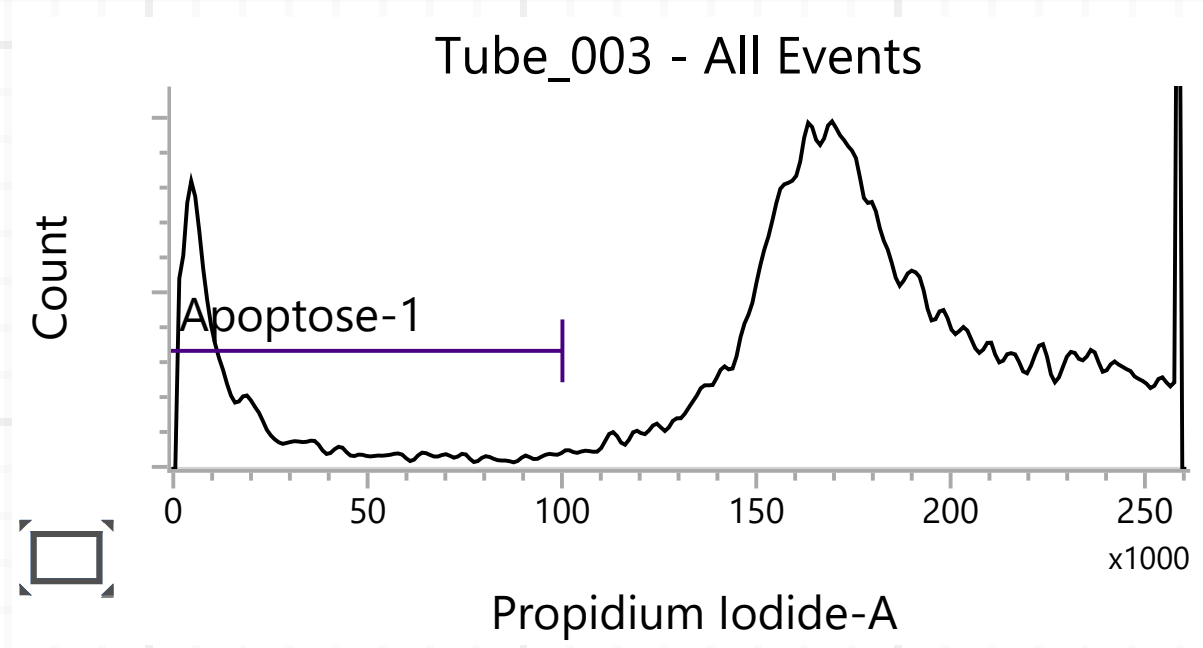

Statistics

Prozent apoptotische Zellen: Prozent apoptotische Zellen No Value % von Cells

Name	Events	% Total	% Parent	% Grandparent
Tube_001:All Events	10,184	100.00	***	***
Tube_001:Cells	10,000	98.19	98.19	***
Tube_002:All Events	10,000	100.00	***	***
Tube_002:Cells	9,780	97.80	97.80	***
Tube_003:All Events	10,000	100.00	***	***
Tube_003:Cells	9,777	97.77	97.77	***
Tube_001:Apoptose-1	1,304	12.80	12.80	***
Tube_002:Apoptose-1	1,456	14.56	14.56	***
Tube_003:Apoptose-1	1,384	13.84	13.84	***

DNA Fragmentierung

Sample ID: 1

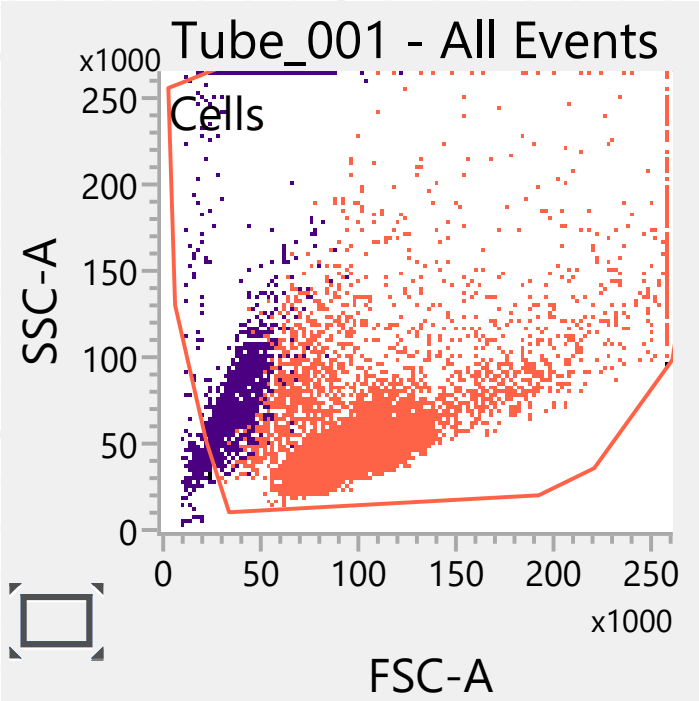

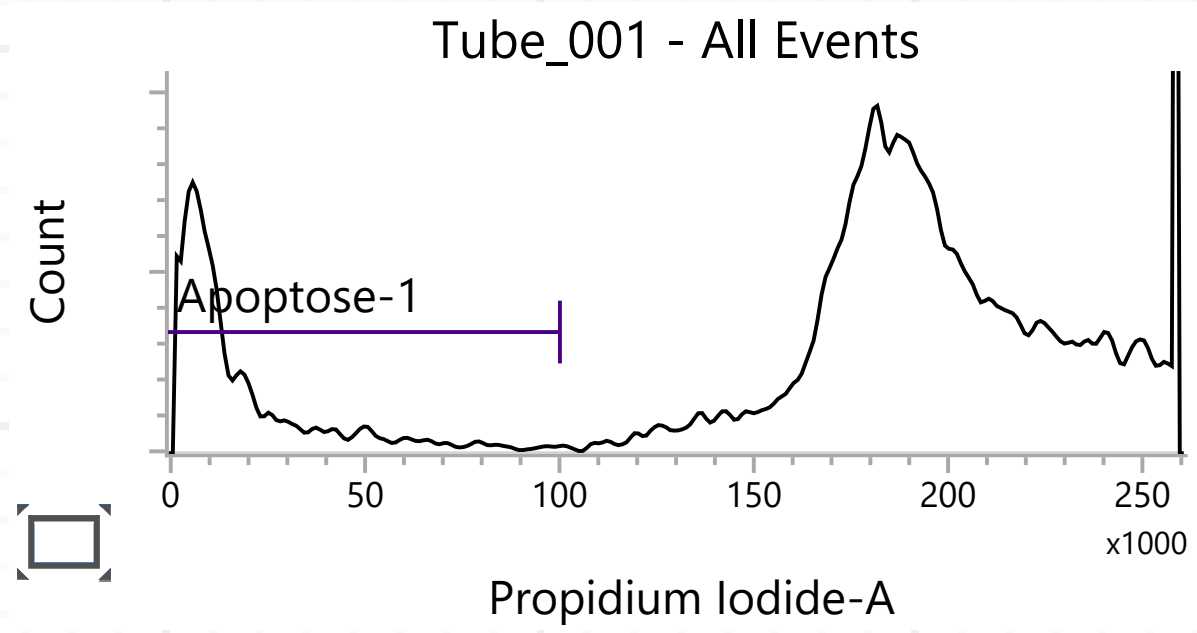

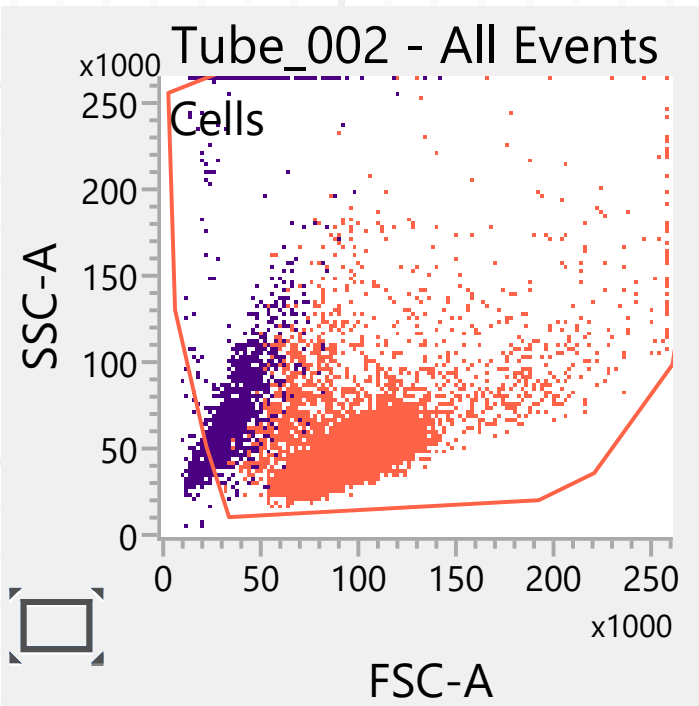

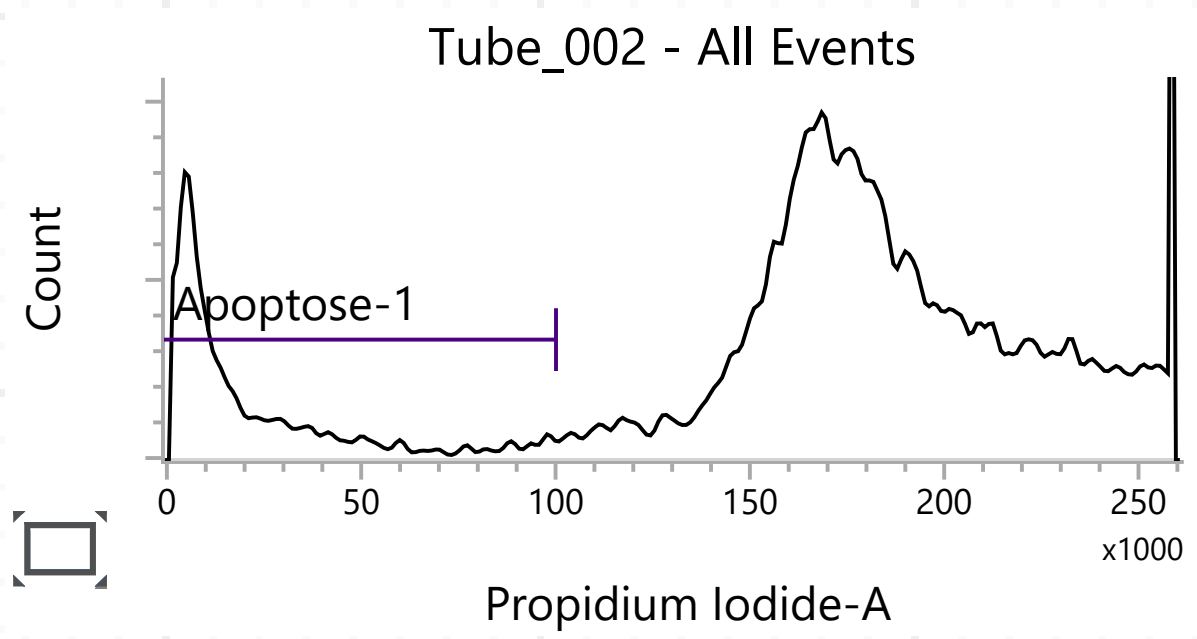

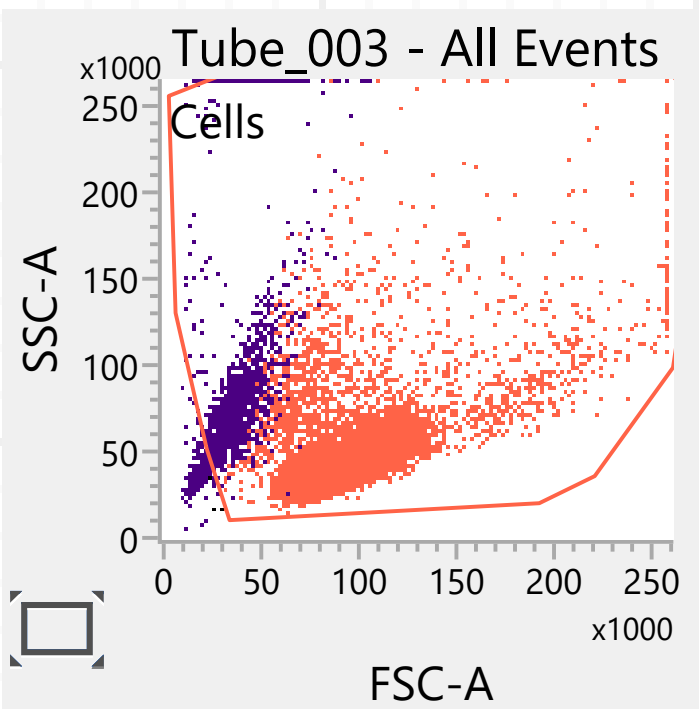

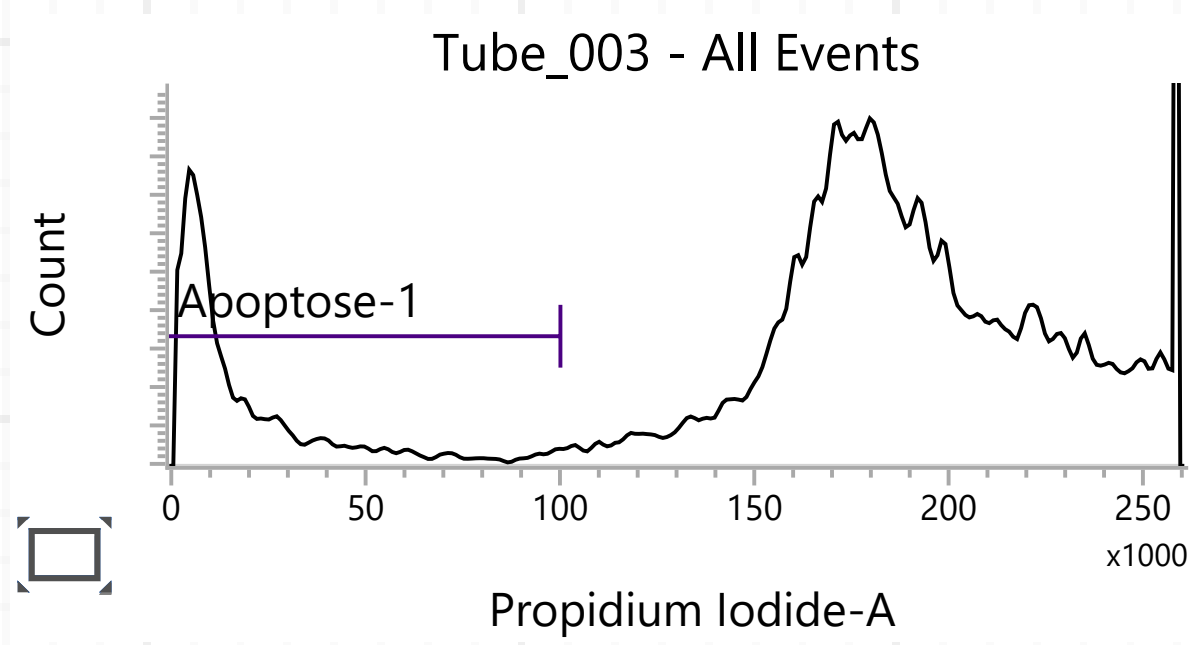

Statistics

Prozent apoptotische Zellen: Prozent apoptotische Zellen No Value % von Cells

Name	Events	% Total	% Parent	% Grandparent
Tube_001:All Events	10,196	100.00	***	***
Tube_001:Cells	10,000	98.08	98.08	***
Tube_002:All Events	10,000	100.00	***	***
Tube_002:Cells	9,834	98.34	98.34	***
Tube_003:All Events	10,000	100.00	***	***
Tube_003:Cells	9,827	98.27	98.27	***
Tube_001:Apoptose-1	1,434	14.06	14.06	***
Tube_002:Apoptose-1	1,402	14.02	14.02	***
Tube_003:Apoptose-1	1,342	13.42	13.42	***

DNA Fragmentierung

Sample ID: 5

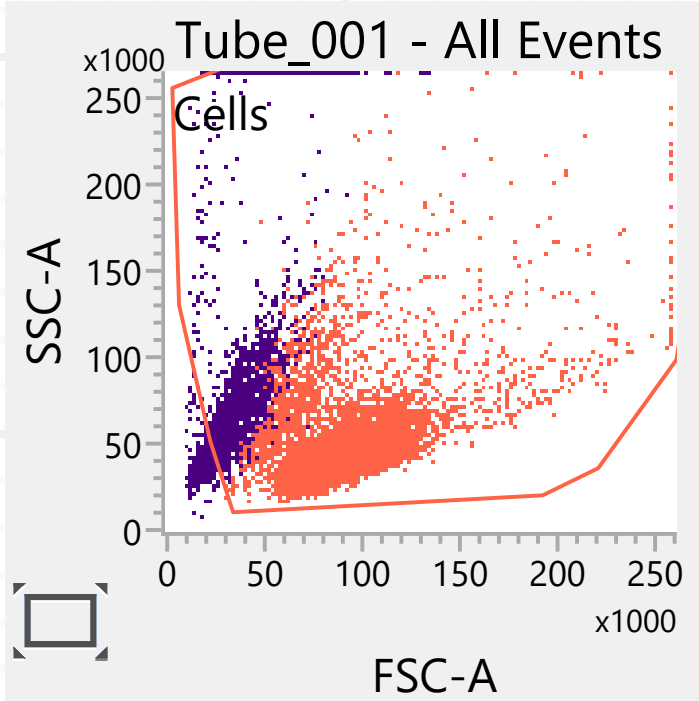

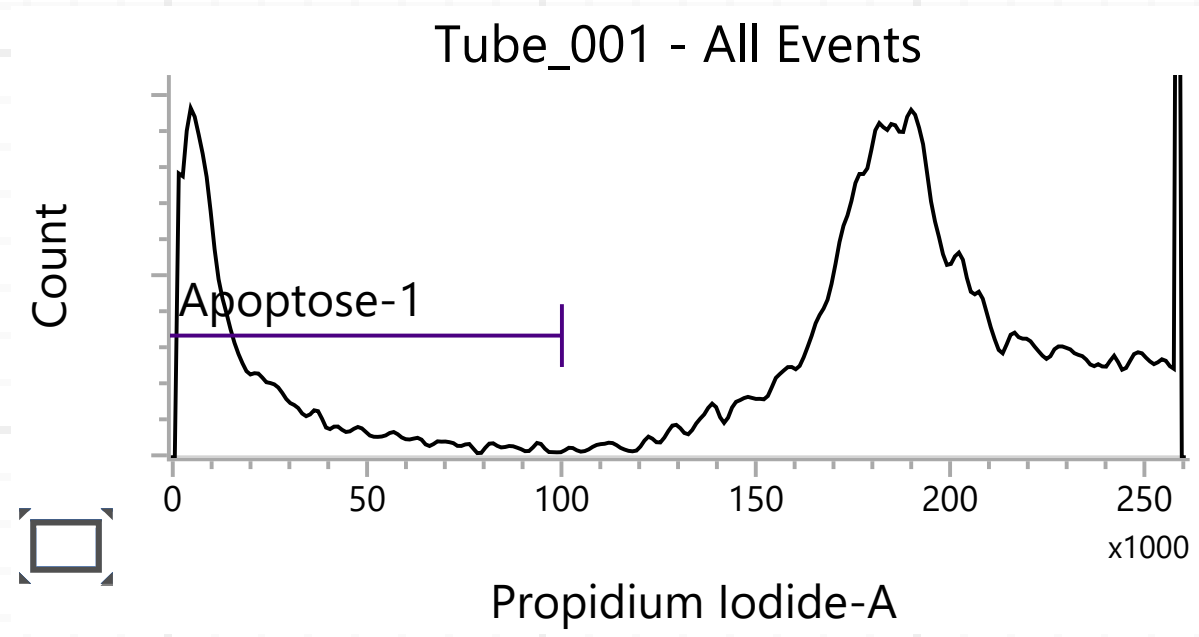

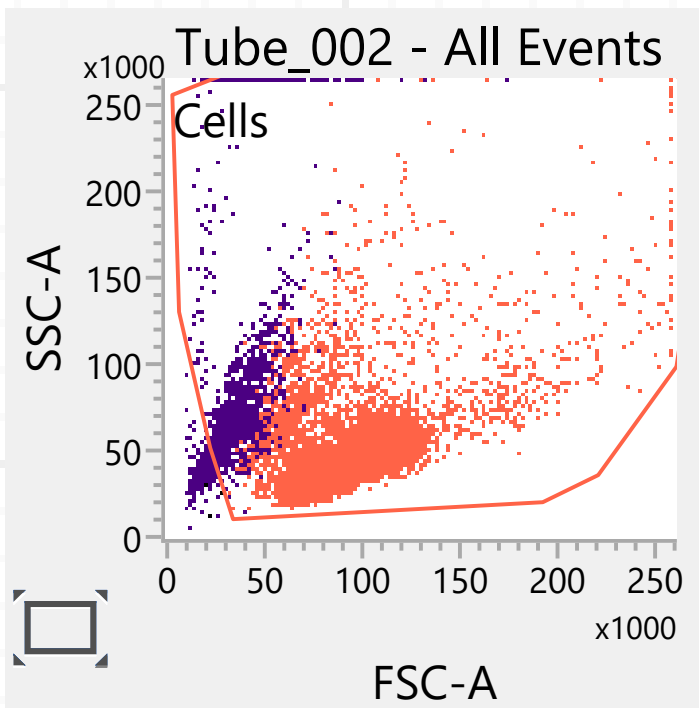

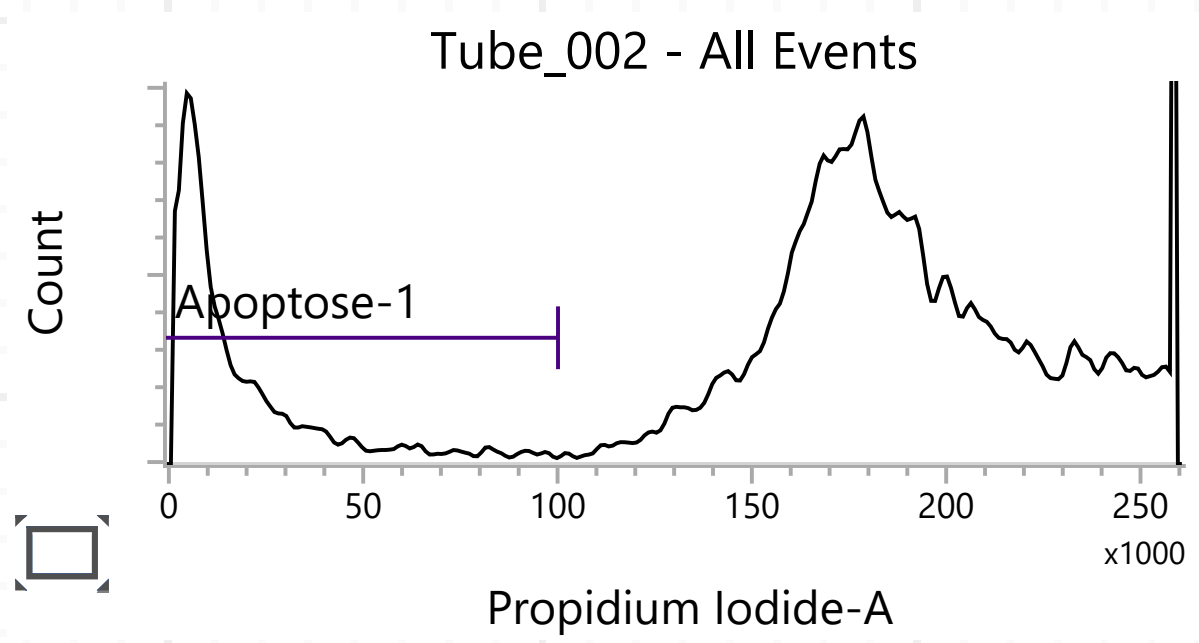

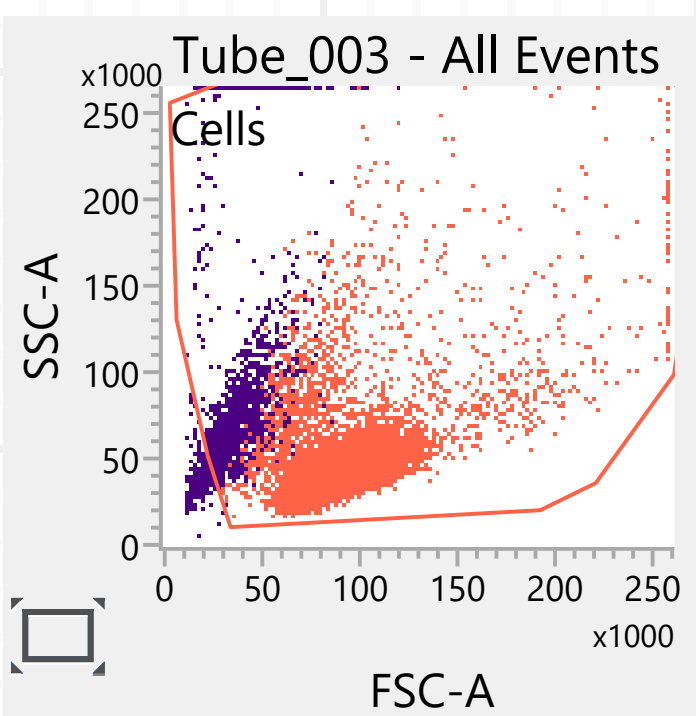

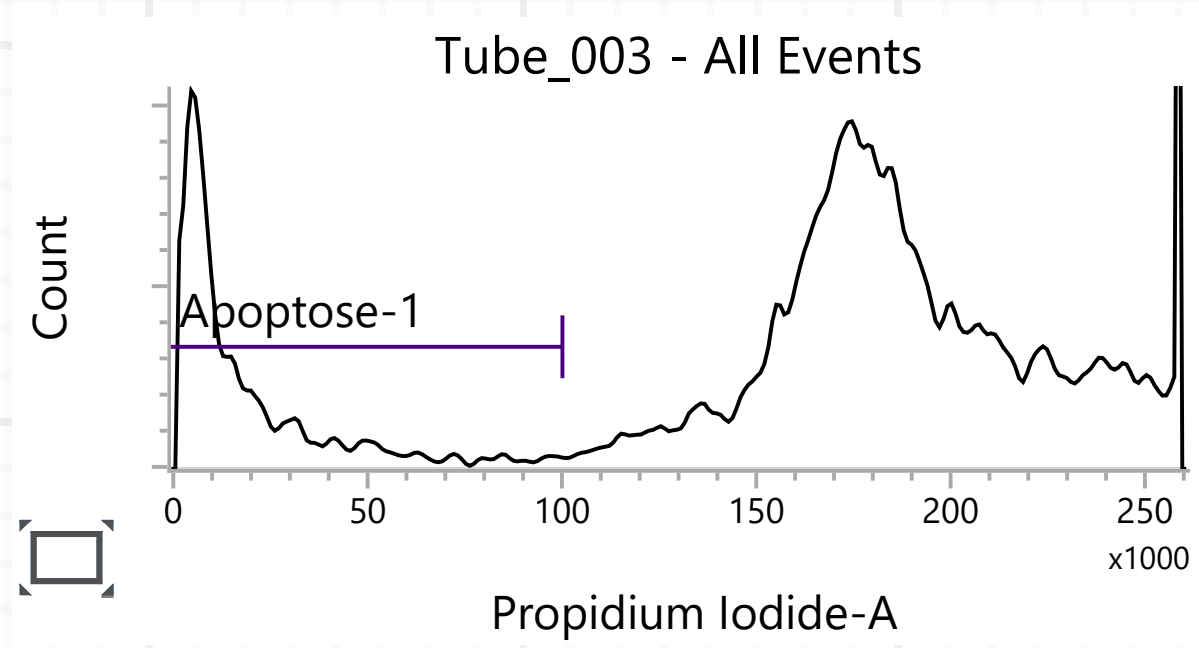

Statistics

Prozent apoptotische Zellen: Prozent apoptotische Zellen No Value % von Cells

Name	Events	% Total	% Parent	% Grandparent	
Tube_001:All Events	10,293	100.00	***	***	
Tube_001:Cells	10,000	97.15	97.15	***	
Tube_002:All Events	10,000	100.00	***	***	
Tube_002:Cells	9,746	97.46	97.46	***	
Tube_003:All Events	10,000	100.00	***	***	
Tube_003:Cells	9,760	97.60	97.60	***	
Tube_001:Apoptose-1	1,968	19.12	19.12	***	
Tube_002:Apoptose-1	1,755	17.55	17.55	***	
Tube_003:Apoptose-1	1,696	16.96	16.96	***	

DNA Fragmentierung

Sample ID: 10

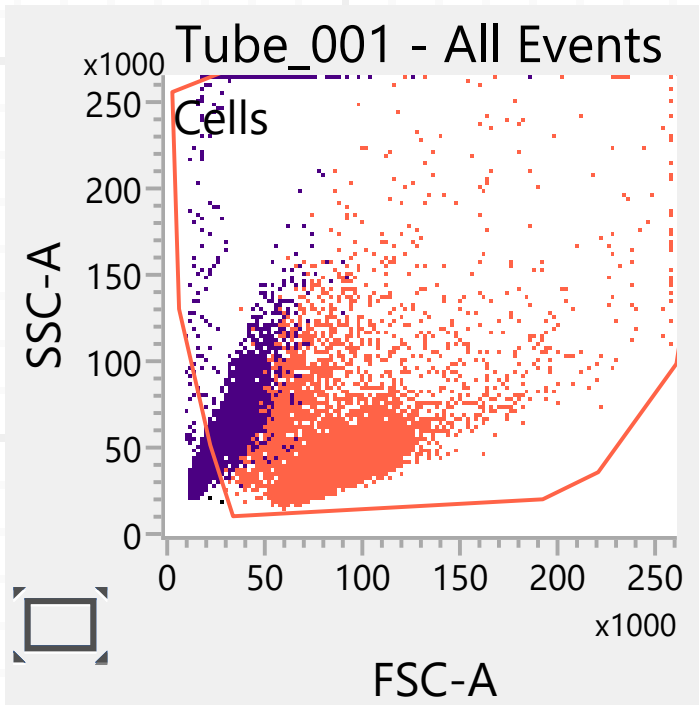

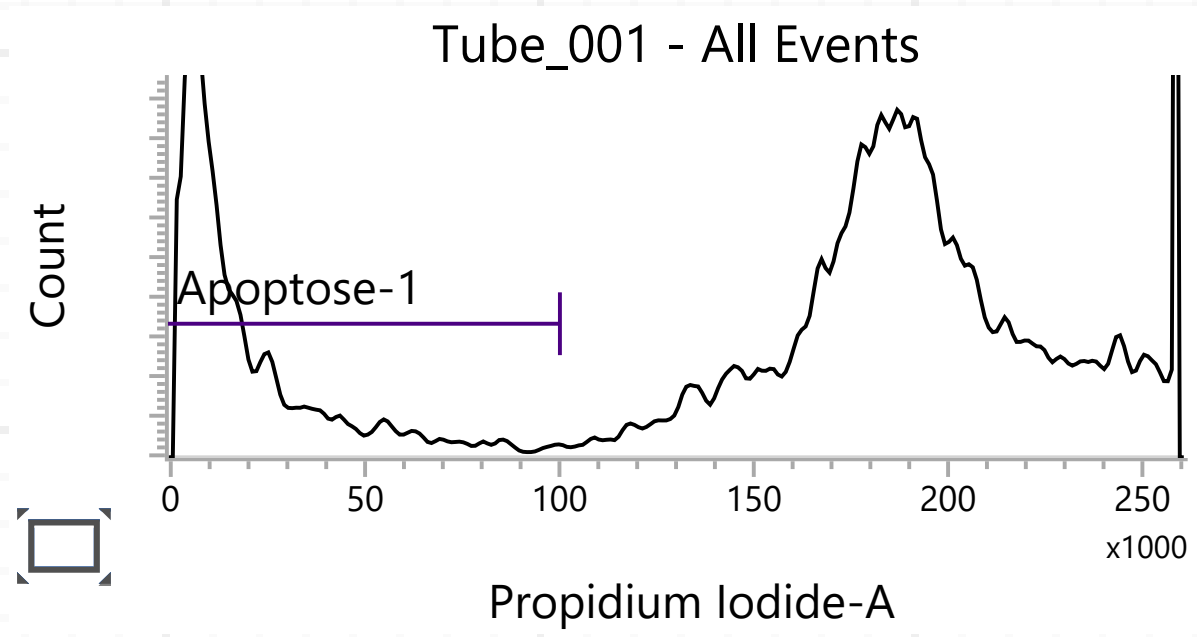

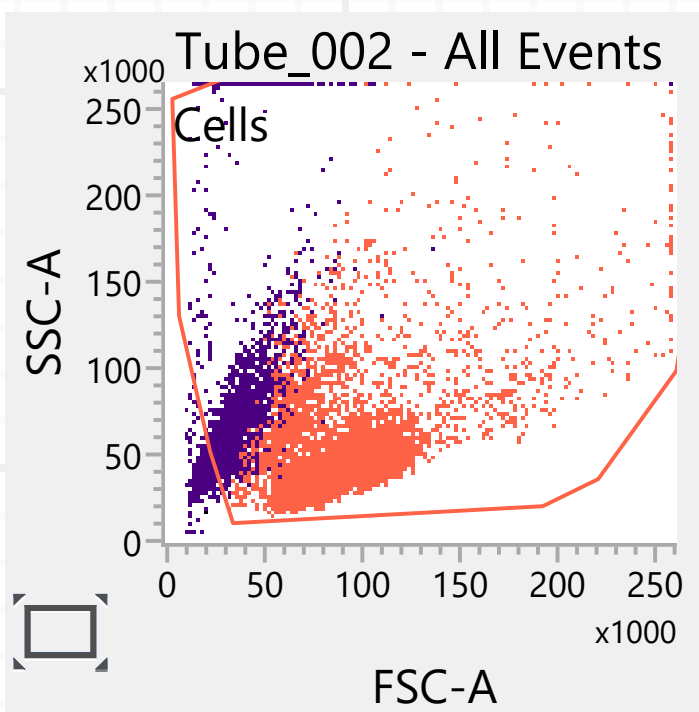

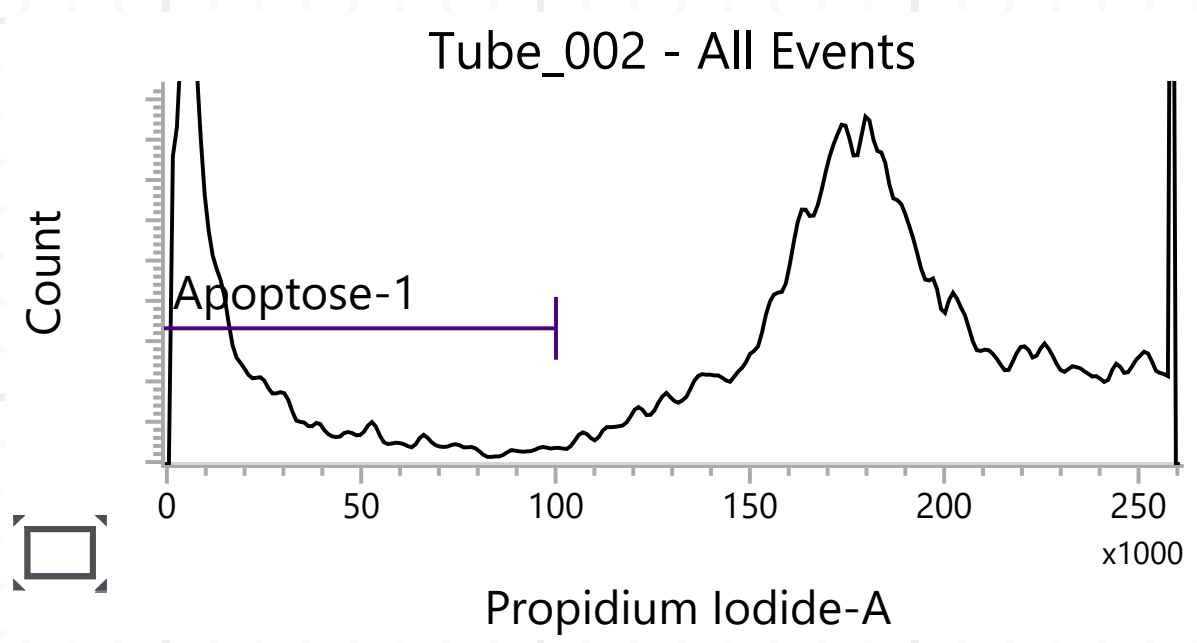

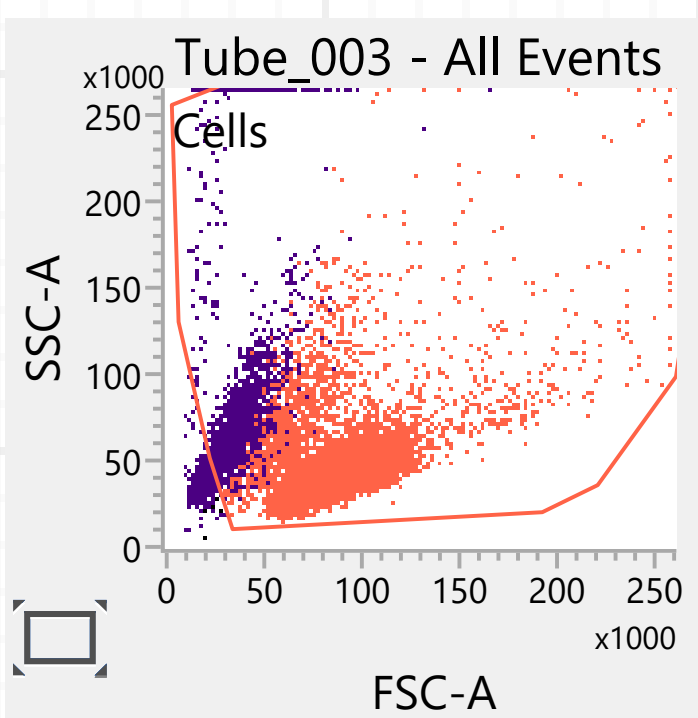

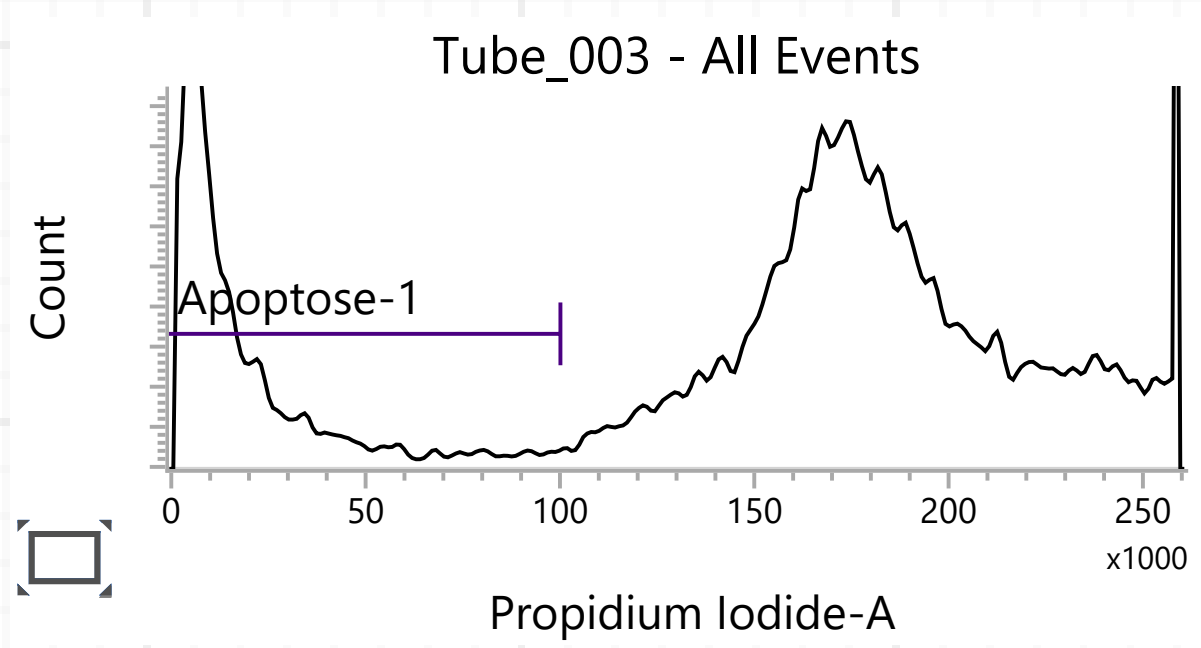

Statistics

Prozent apoptotische Zellen: Prozent apoptotische Zellen No Value % von Cells

Name	Events	% Total	% Parent	% Grandparent
Tube_001:All Events	10,322	100.00	***	***
Tube_001:Cells	10,000	96.88	96.88	***
Tube_002:All Events	10,000	100.00	***	***
Tube_002:Cells	9,672	96.72	96.72	***
Tube_003:All Events	10,000	100.00	***	***
Tube_003:Cells	9,645	96.45	96.45	***
Tube_001:Apoptose-1	2,161	20.94	20.94	***
Tube_002:Apoptose-1	2,082	20.82	20.82	***
Tube_003:Apoptose-1	2,097	20.97	20.97	***

DNA Fragmentierung

Sample ID: 20

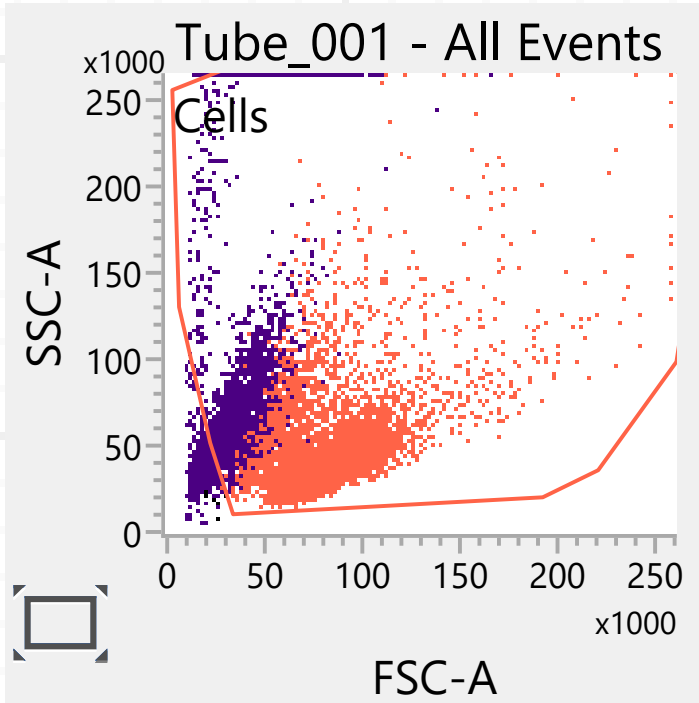

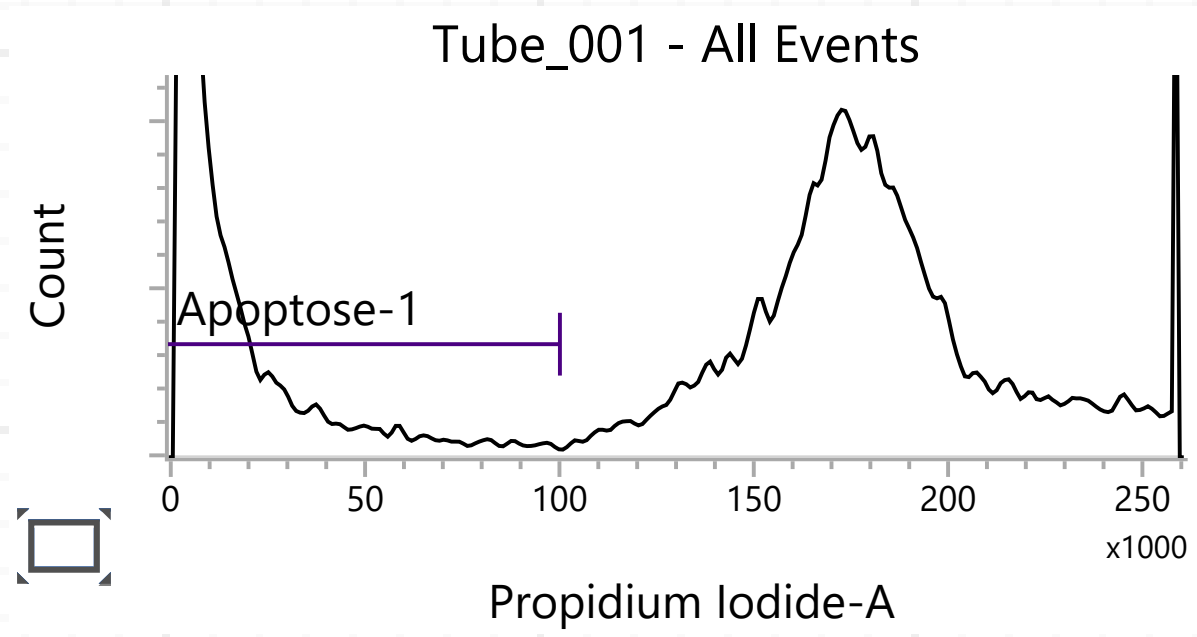

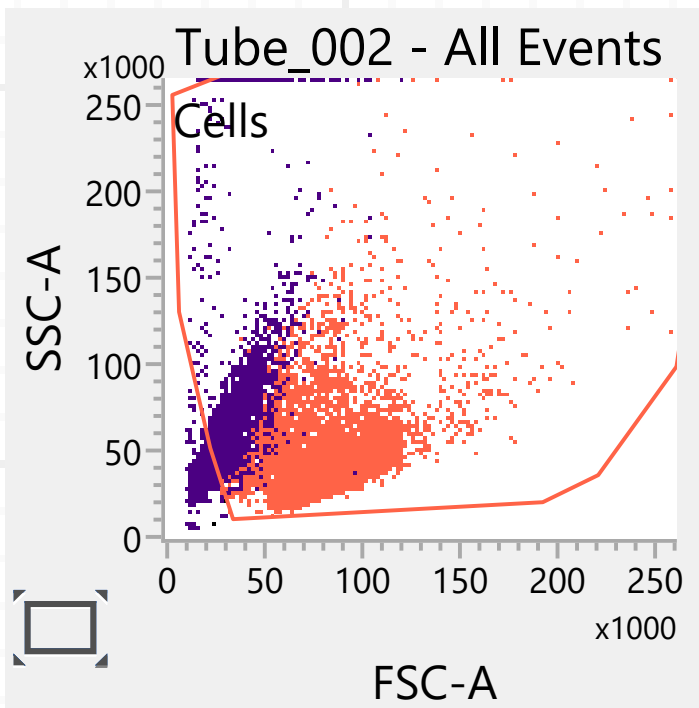

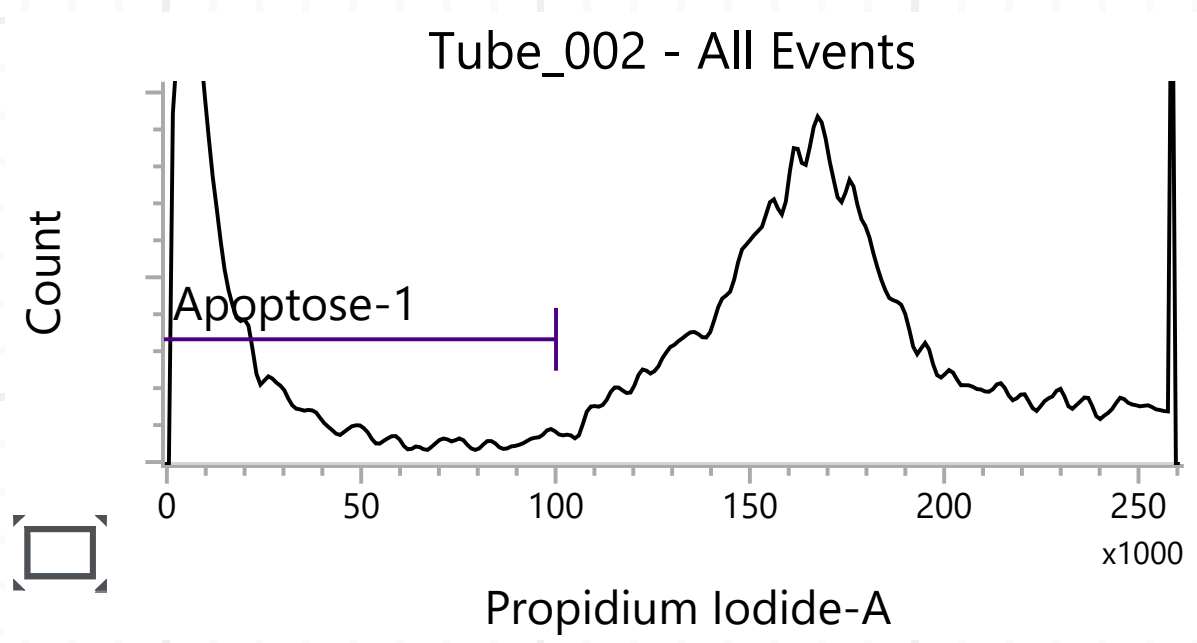

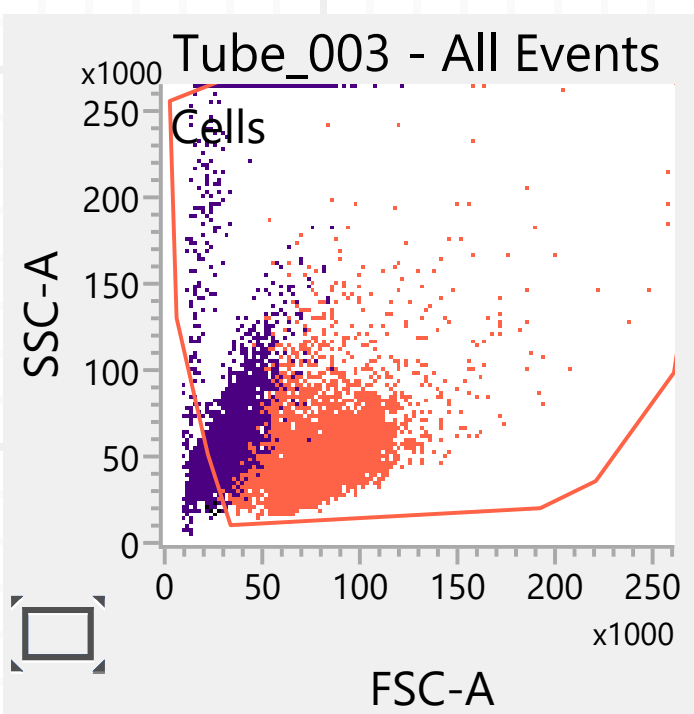

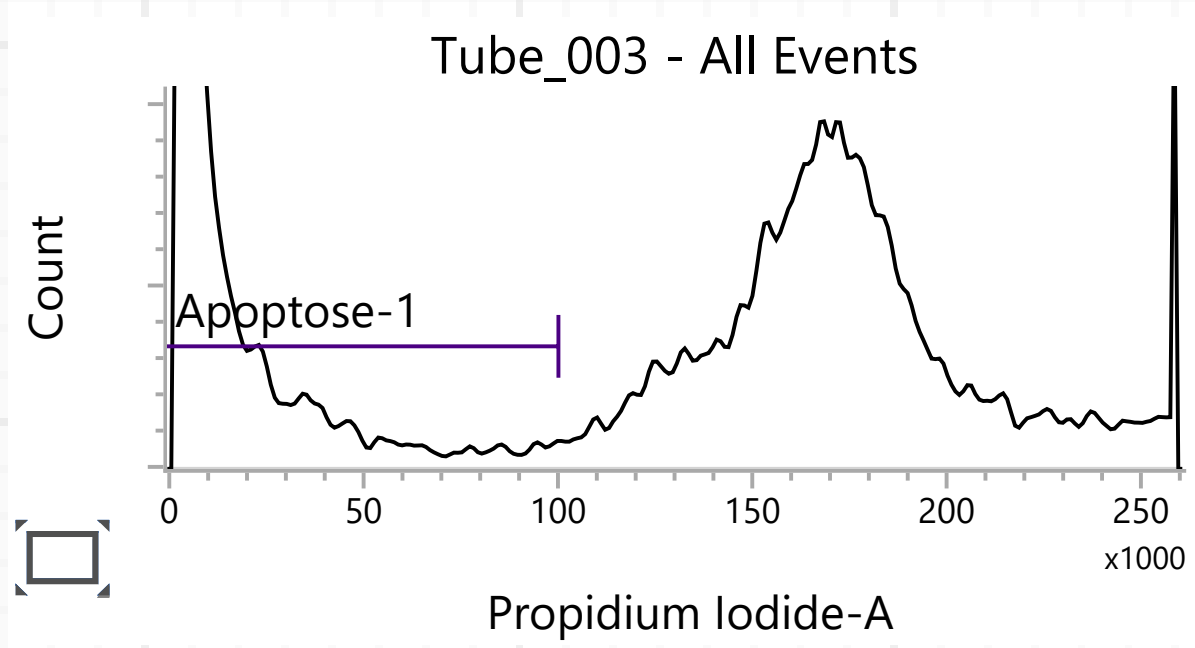

Statistics

Prozent apoptotische Zellen: Prozent apoptotische Zellen No Value % von Cells

Name	Events	% Total	% Parent	% Grandparent
Tube_001:All Events	10,554	100.00	***	***
Tube_001:Cells	10,000	94.75	94.75	***
Tube_002:All Events	10,000	100.00	***	***
Tube_002:Cells	9,423	94.23	94.23	***
Tube_003:All Events	10,000	100.00	***	***
Tube_003:Cells	9,344	93.44	93.44	***
Tube_001:Apoptose-1	3,043	28.83	28.83	***
Tube_002:Apoptose-1	2,811	28.11	28.11	***
Tube_003:Apoptose-1	3,158	31.58	31.58	***

DNA Fragmentierung

Sample ID: D

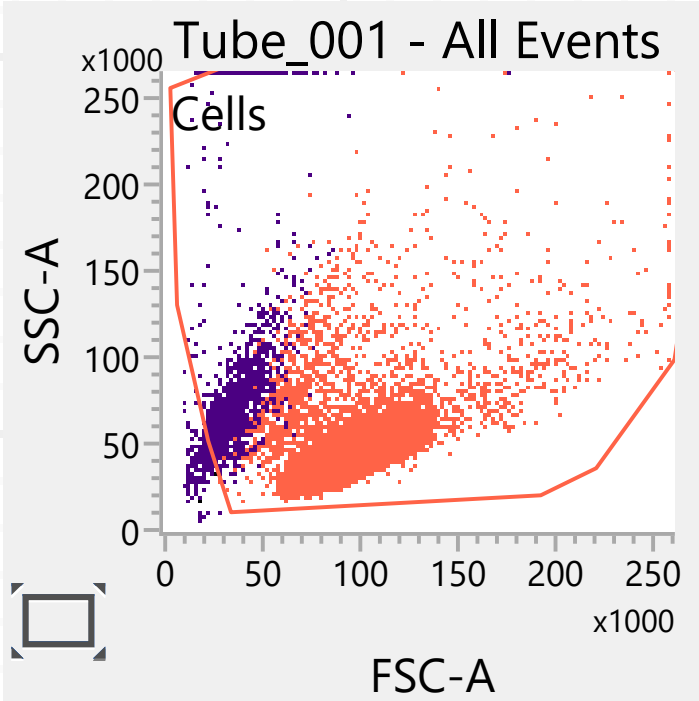

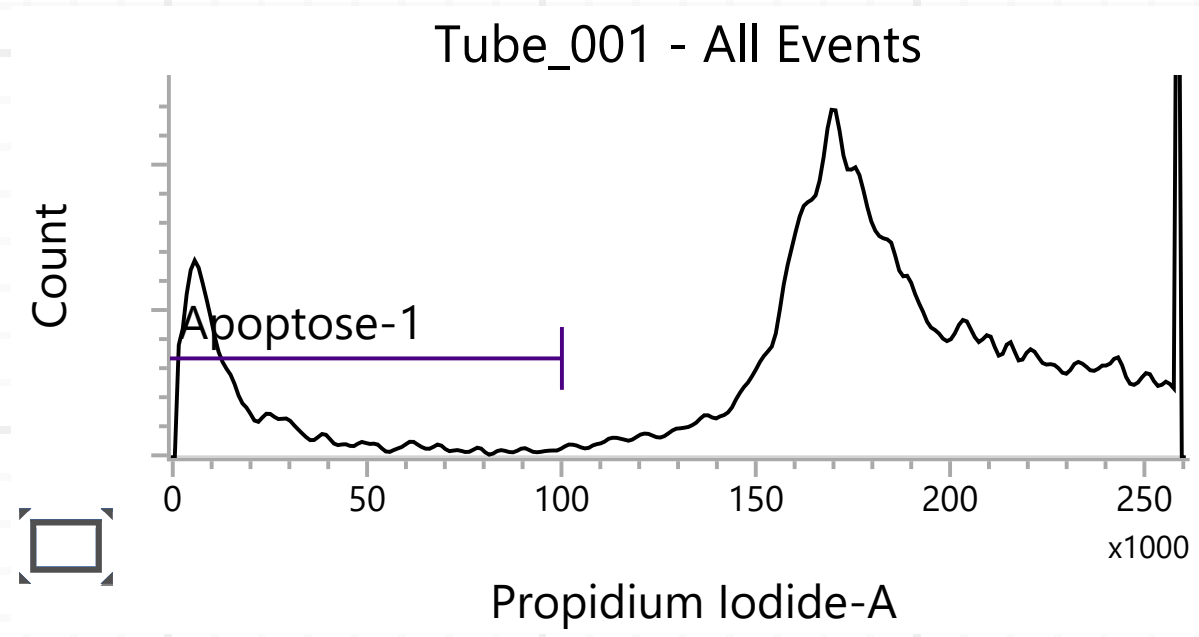

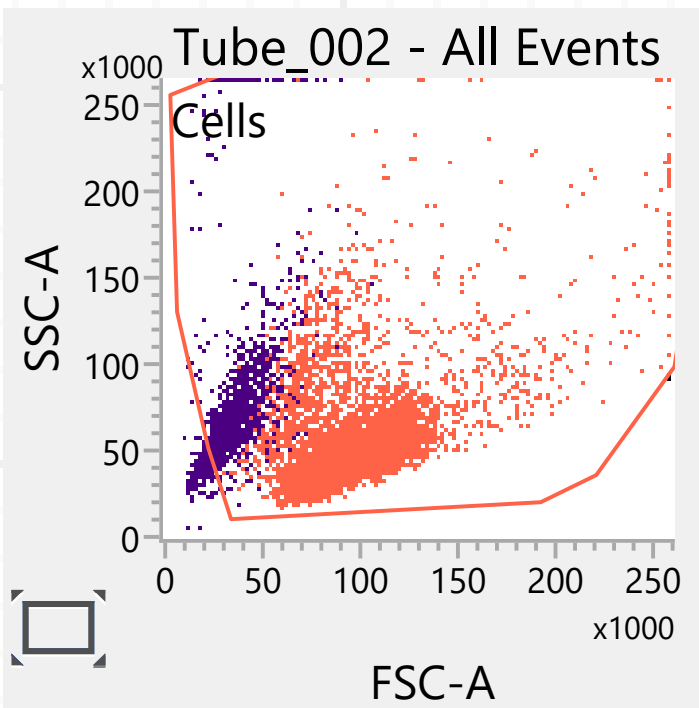

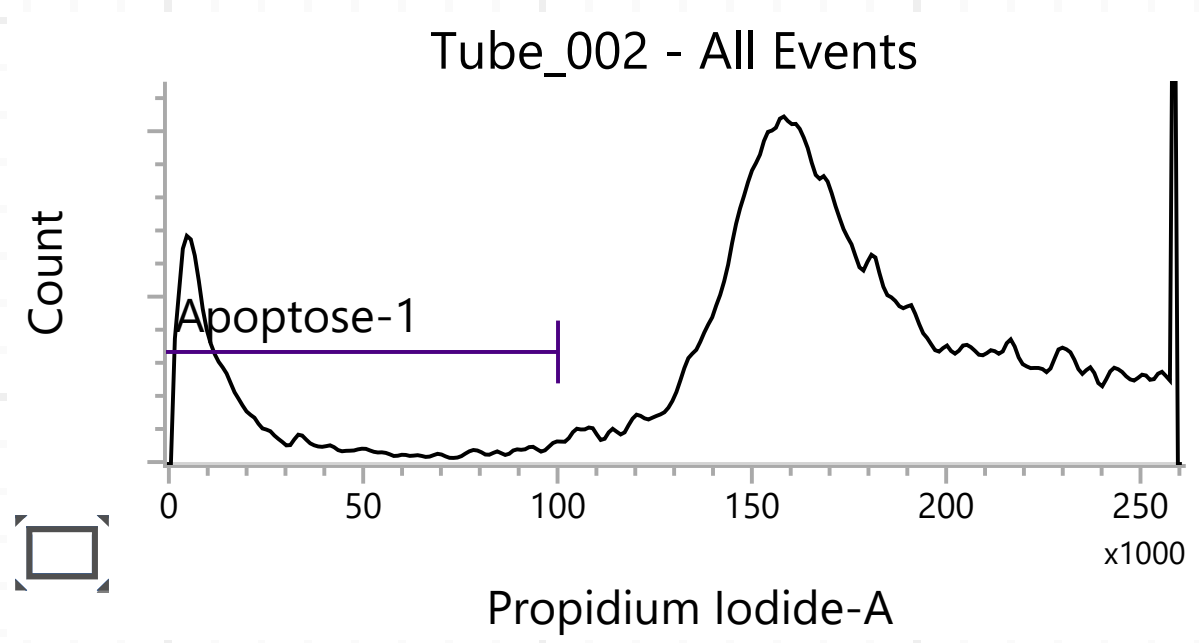

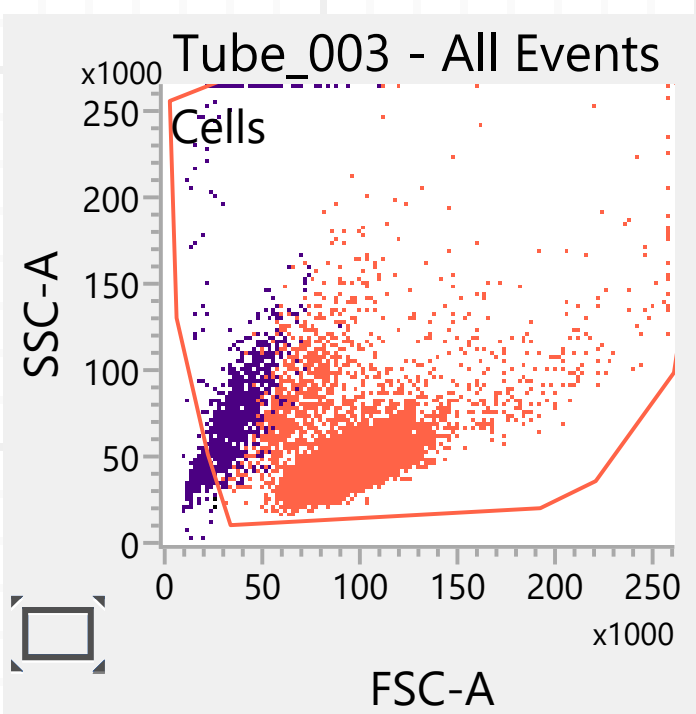

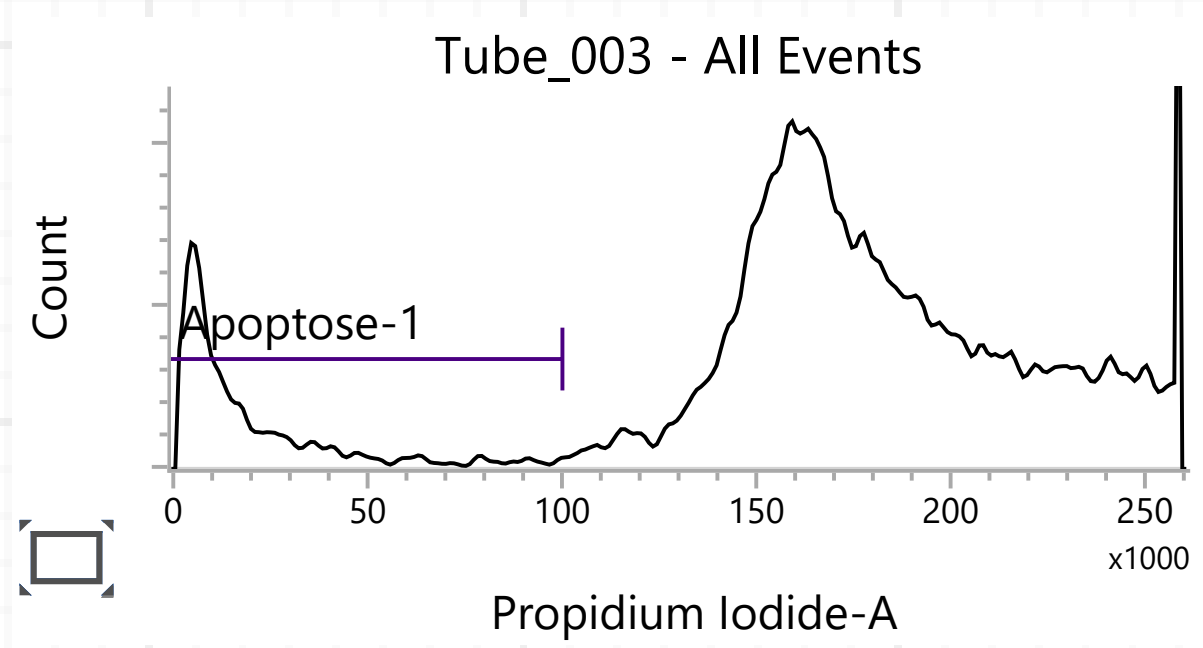

Statistics

Prozent apoptotische Zellen: Prozent apoptotische Zellen No Value % von Cells

Name	Events	% Total	% Parent	% Grandparent	
Tube_001:All Events	10,183	100.00	***	***	
Tube_001:Cells	10,000	98.20	98.20	***	
Tube_002:All Events	10,000	100.00	***	***	
Tube_002:Cells	9,820	98.20	98.20	***	
Tube_003:All Events	10,000	100.00	***	***	
Tube_003:Cells	9,841	98.41	98.41	***	
Tube_001:Apoptose-1	1,280	12.57	12.57	***	
Tube_002:Apoptose-1	1,222	12.22	12.22	***	
Tube_003:Apoptose-1	1,136	11.36	11.36	***	

DNA Fragmentierung

Sample ID: K

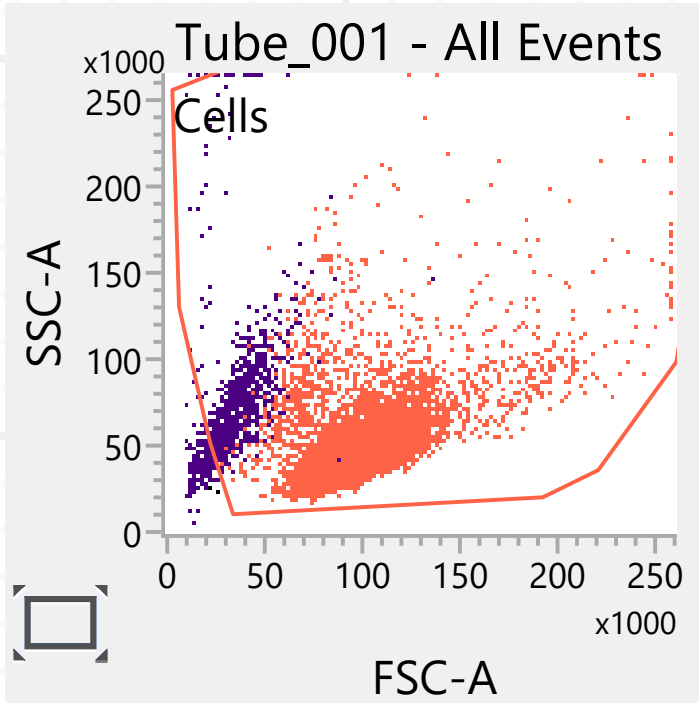

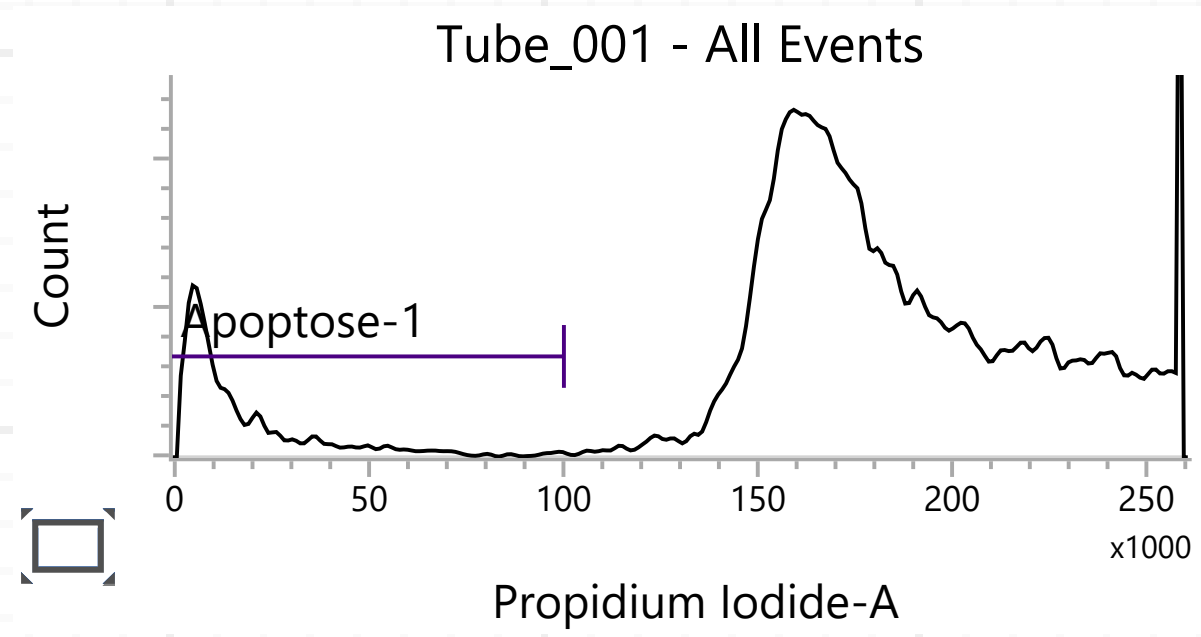

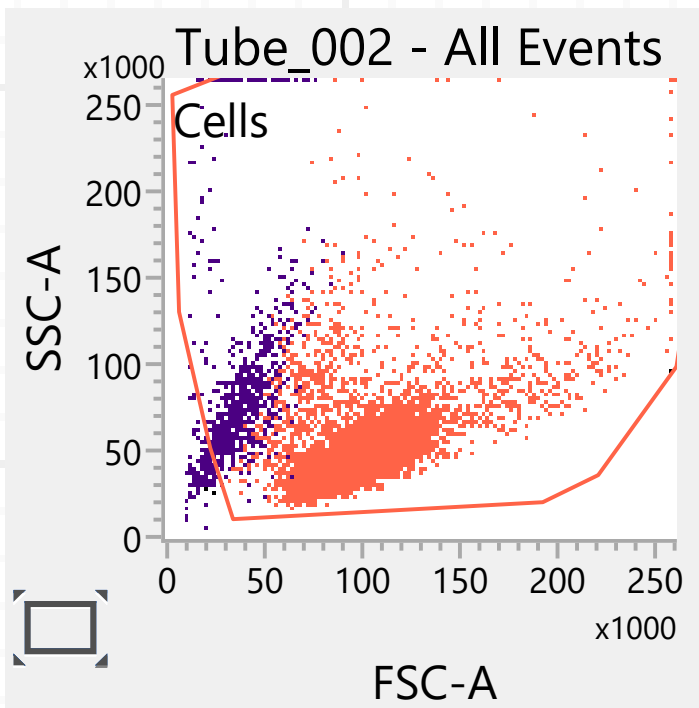

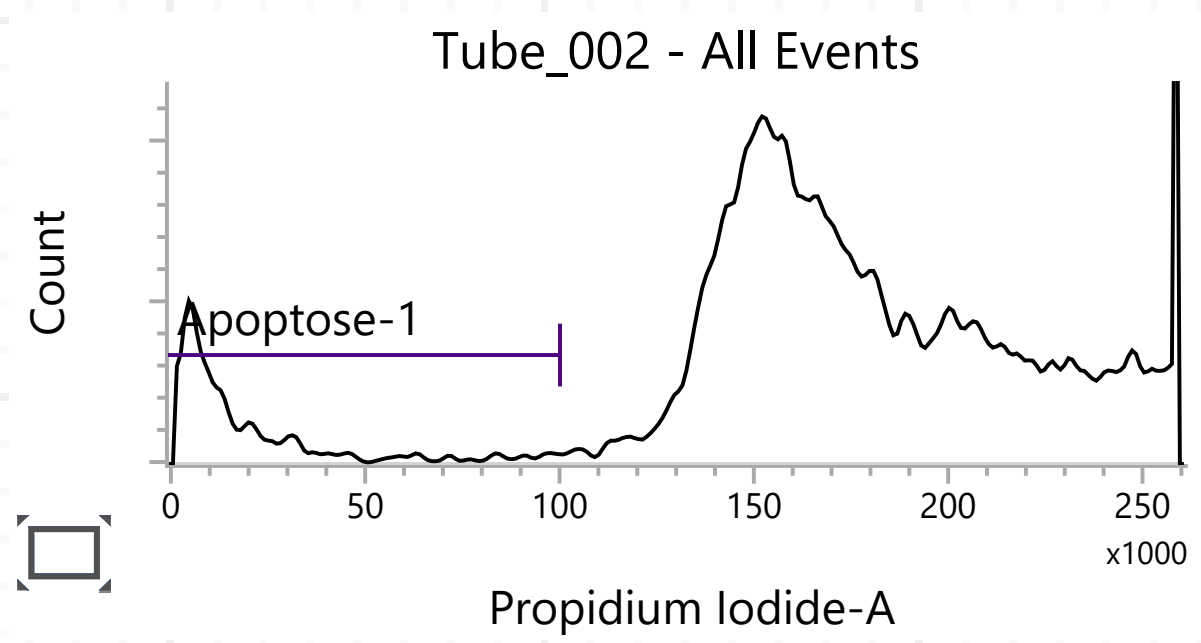

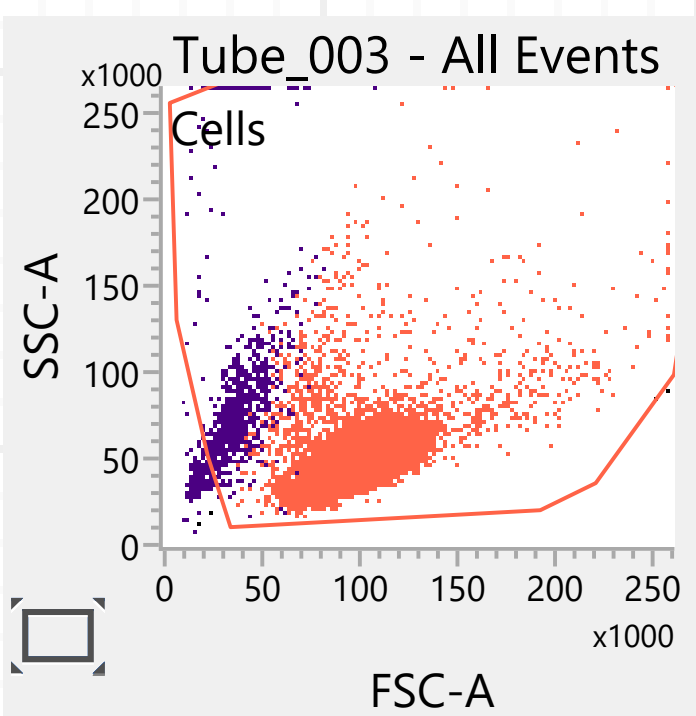

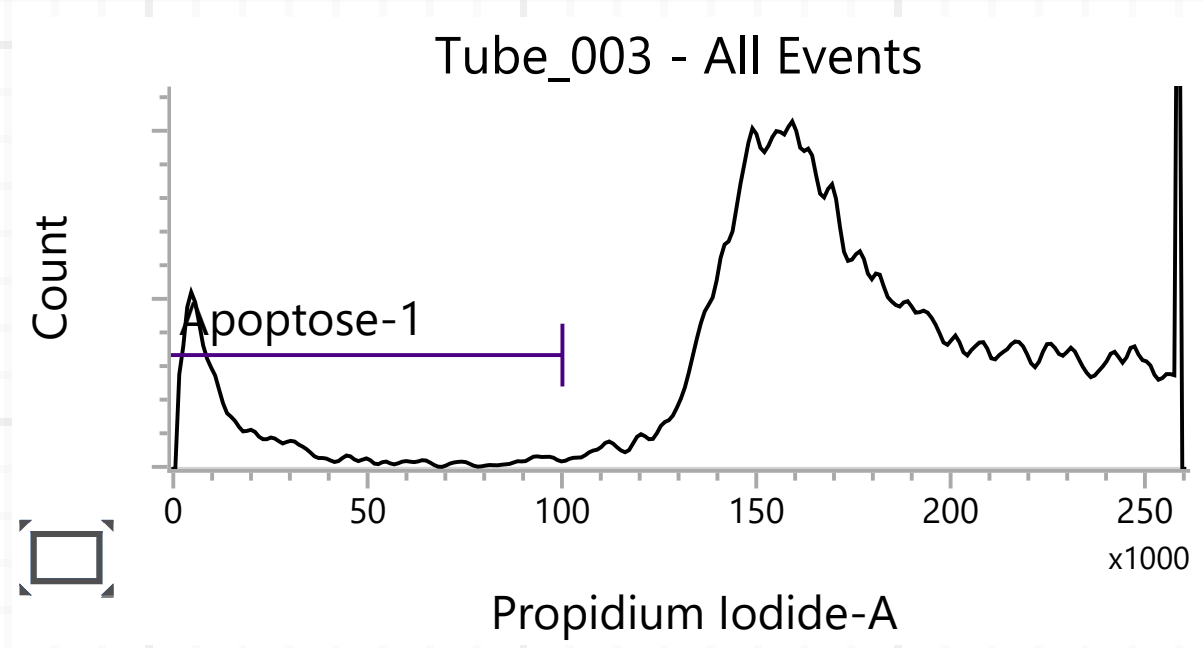

Statistics

Prozent apoptotische Zellen: Prozent apoptotische Zellen No Value % von Cells

Name	Events	% Total	% Parent	% Grandparent
Tube_001:All Events	10,133	100.00	***	***
Tube_001:Cells	10,000	98.69	98.69	***
Tube_002:All Events	10,000	100.00	***	***
Tube_002:Cells	9,883	98.83	98.83	***
Tube_003:All Events	10,000	100.00	***	***
Tube_003:Cells	9,888	98.88	98.88	***
Tube_001:Apoptose-1	902	8.90	8.90	***
Tube_002:Apoptose-1	875	8.75	8.75	***
Tube_003:Apoptose-1	875	8.75	8.75	***

DNA Fragmentierung

Sample ID: L2 0,1

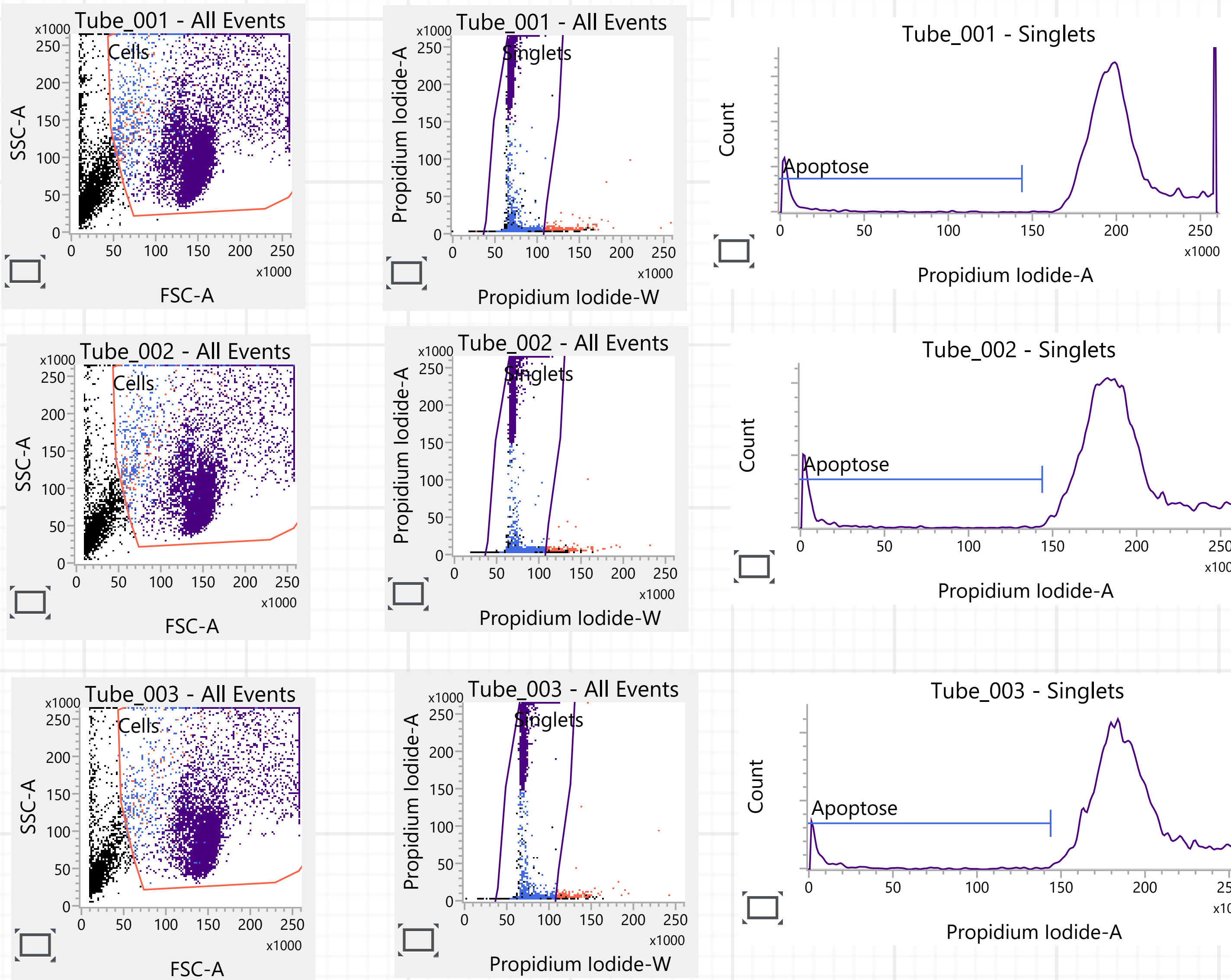

Statistics

Prozent apoptotische Zellen: Prozent apoptotische Zellen 5.5 % von Cells

Name	Events	% Total	% Parent	% Grandparent
Tube_001:All Events	13,187	100.00	***	***
Tube_001:Apoptose	538	4.08	5.46	5.38
Tube_001:Cells	10,000	75.83	75.83	***
Tube_001:Singlets	9,849	74.69	98.49	74.69
Tube_002:All Events	10,000	100.00	***	***
Tube_002:Cells	7,783	77.83	77.83	***
Tube_002:Singlets	7,657	76.57	98.38	76.57
Tube_002:Apoptose	434	4.34	5.67	5.58
Tube_003:All Events	10,000	100.00	***	***
Tube_003:Cells	8,264	82.64	82.64	***
Tube_003:Singlets	8,159	81.59	98.73	81.59
Tube_003:Apoptose	348	3.48	4.27	4.21

DNA Fragmentierung

Sample ID: L2 0,5

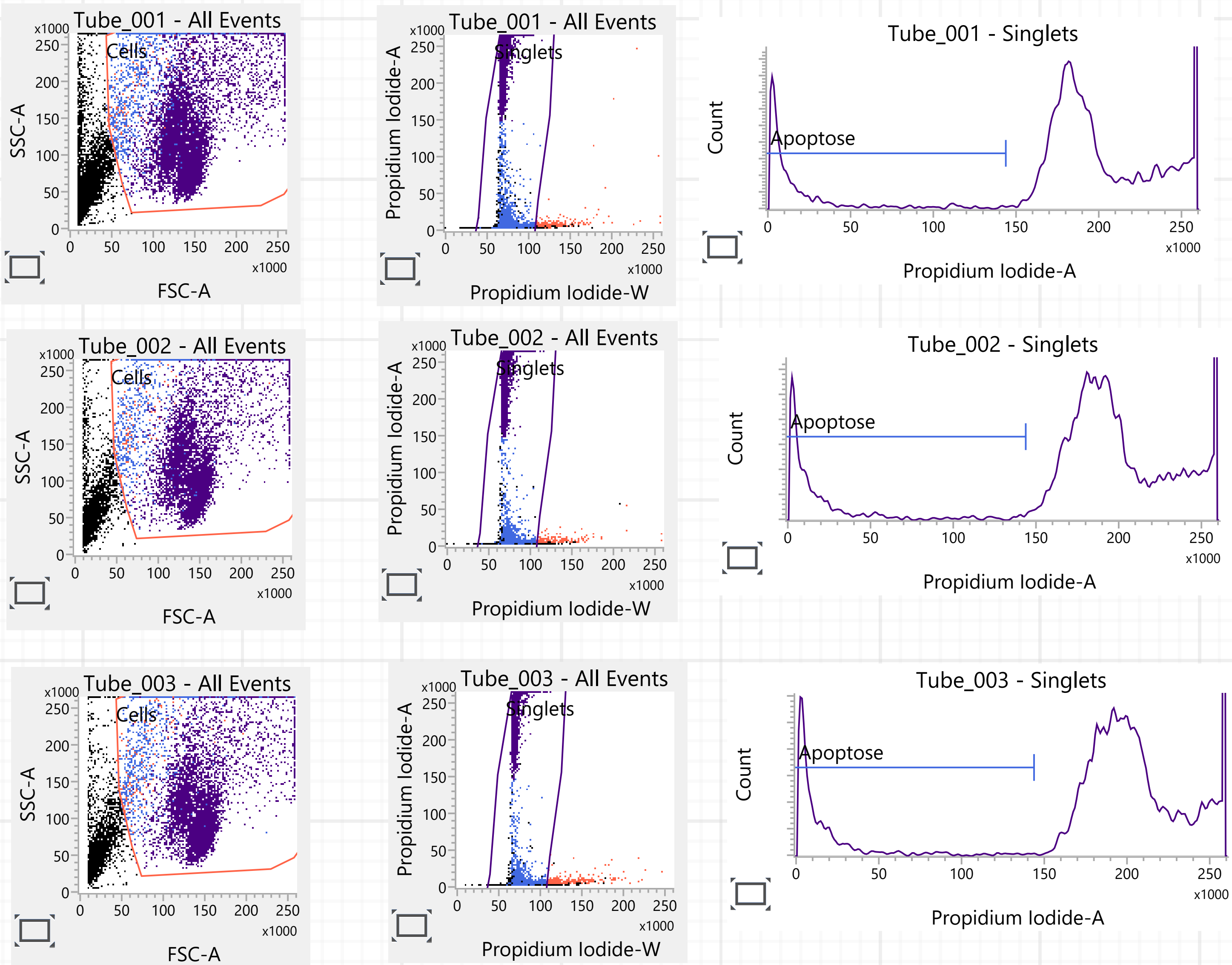

Statistics

Prozent apoptotische Zellen: Prozent apoptotische Zellen 9.9 % von Cells

Name	Events	% Total	% Parent	% Grandparent
Tube_001:All Events	14,525	100.00	***	***
Tube_001:Apoptose	971	6.69	9.88	9.71
Tube_001:Cells	10,000	68.85	68.85	***
Tube_001:Singlets	9,825	67.64	98.25	67.64
Tube_002:All Events	10,000	100.00	***	***
Tube_002:Cells	7,337	73.37	73.37	***
Tube_002:Singlets	7,176	71.76	97.81	71.76
Tube_002:Apoptose	684	6.84	9.53	9.32
Tube_003:All Events	10,000	100.00	***	***
Tube_003:Cells	7,481	74.81	74.81	***
Tube_003:Singlets	7,264	72.64	97.10	72.64
Tube_003:Apoptose	696	6.96	9.58	9.30

DNA Fragmentierung

Sample ID: L2 0,25

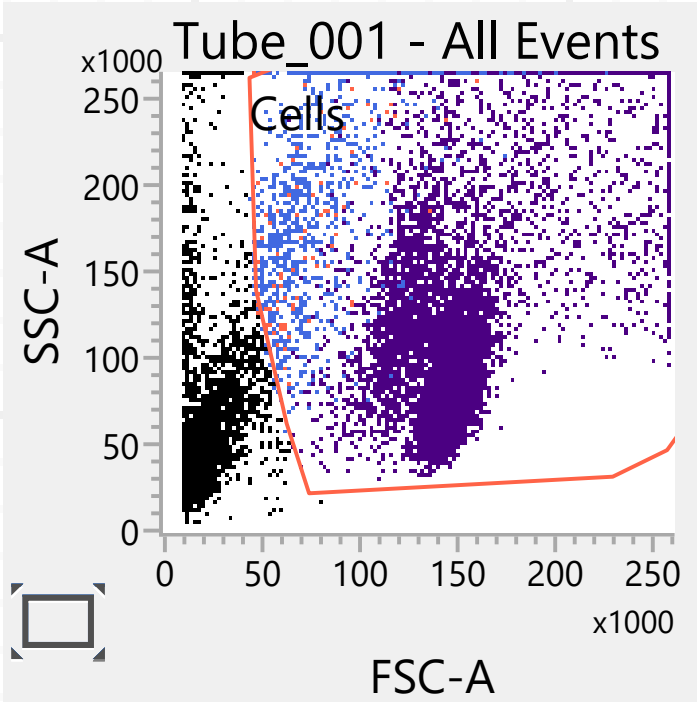

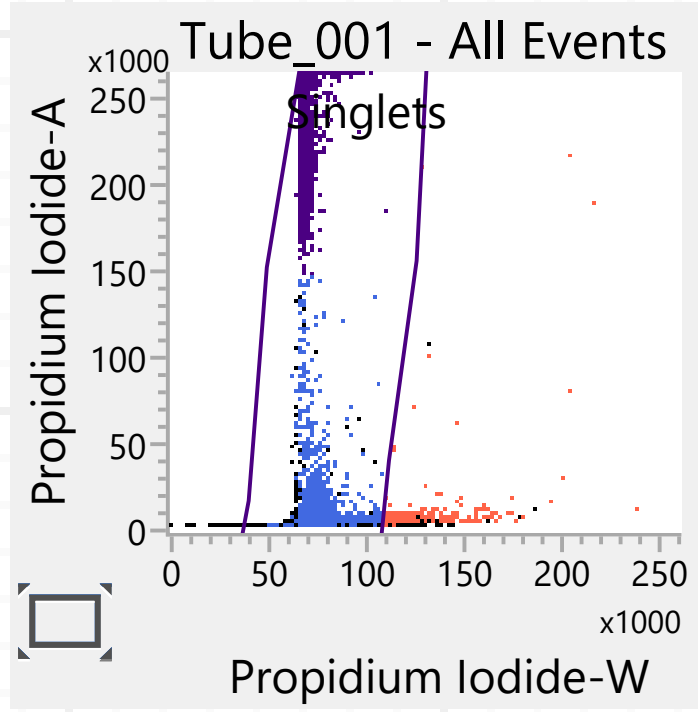

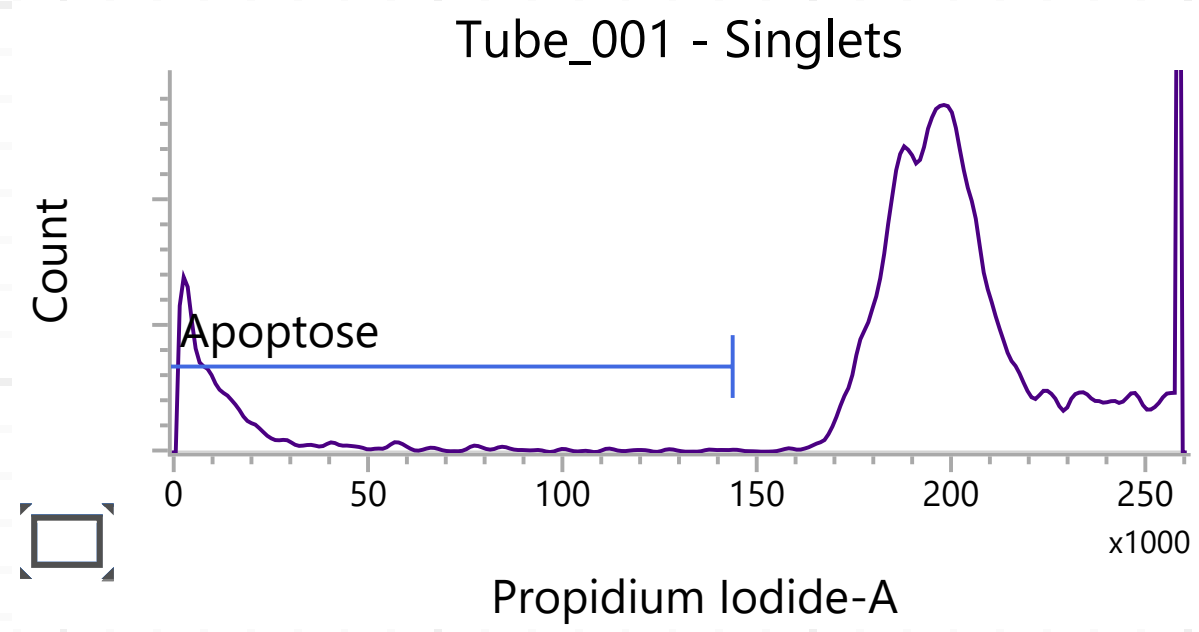

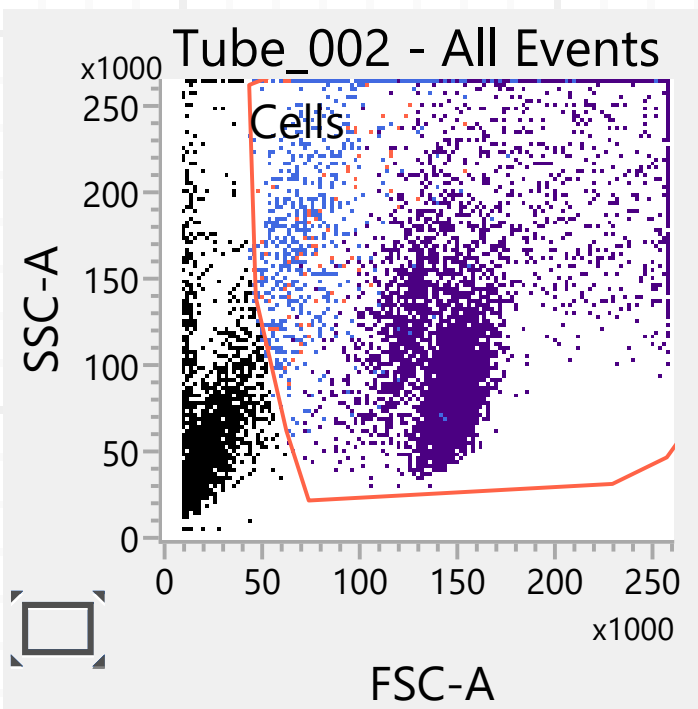

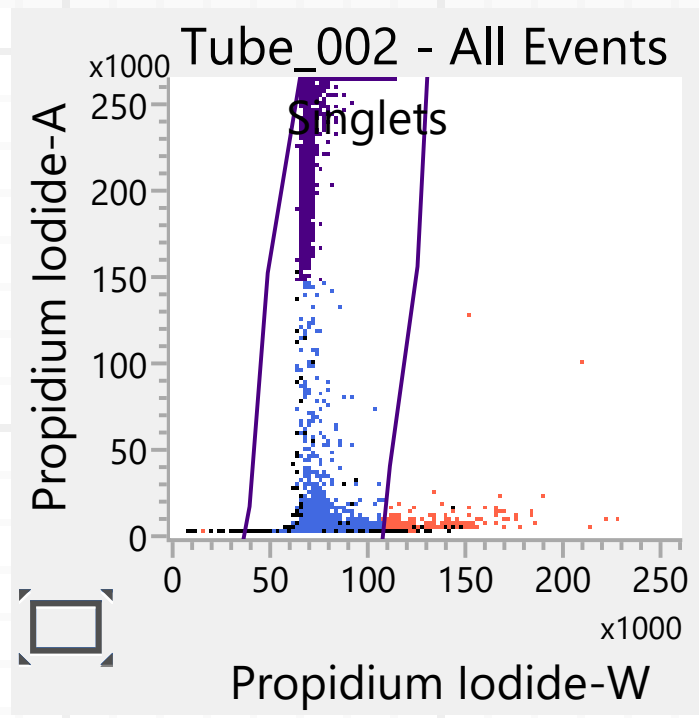

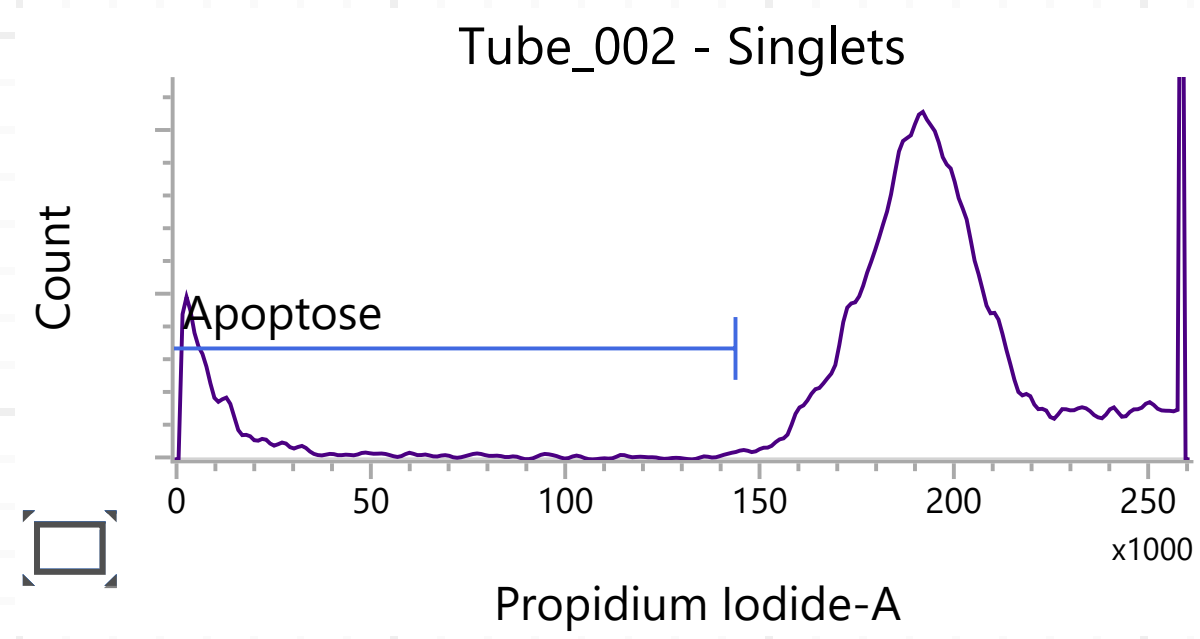

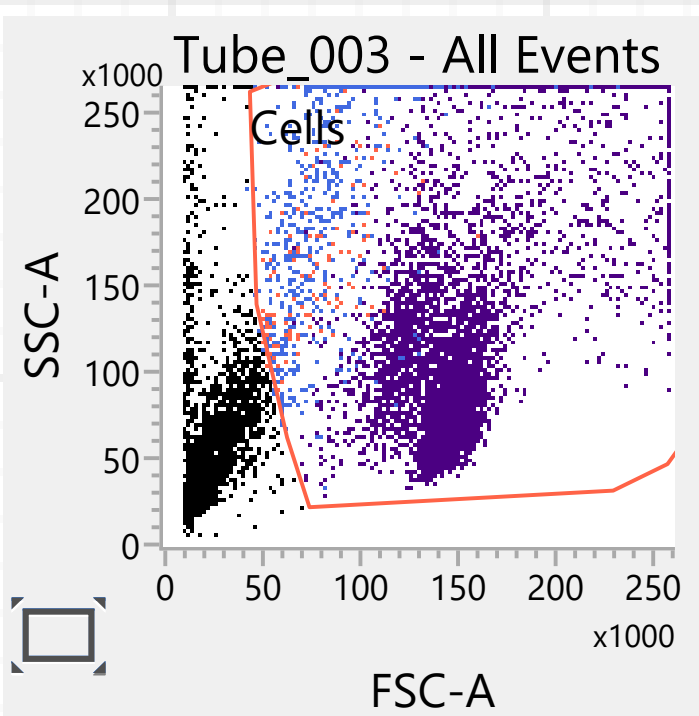

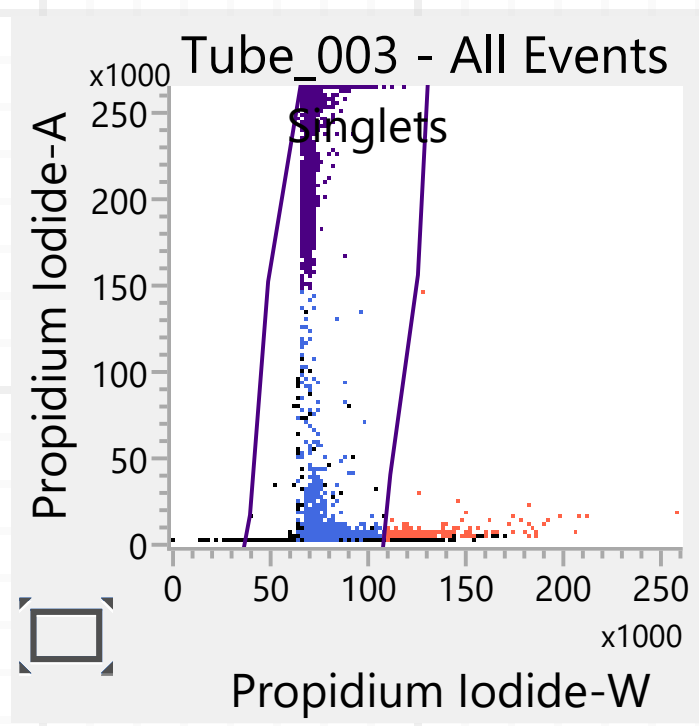

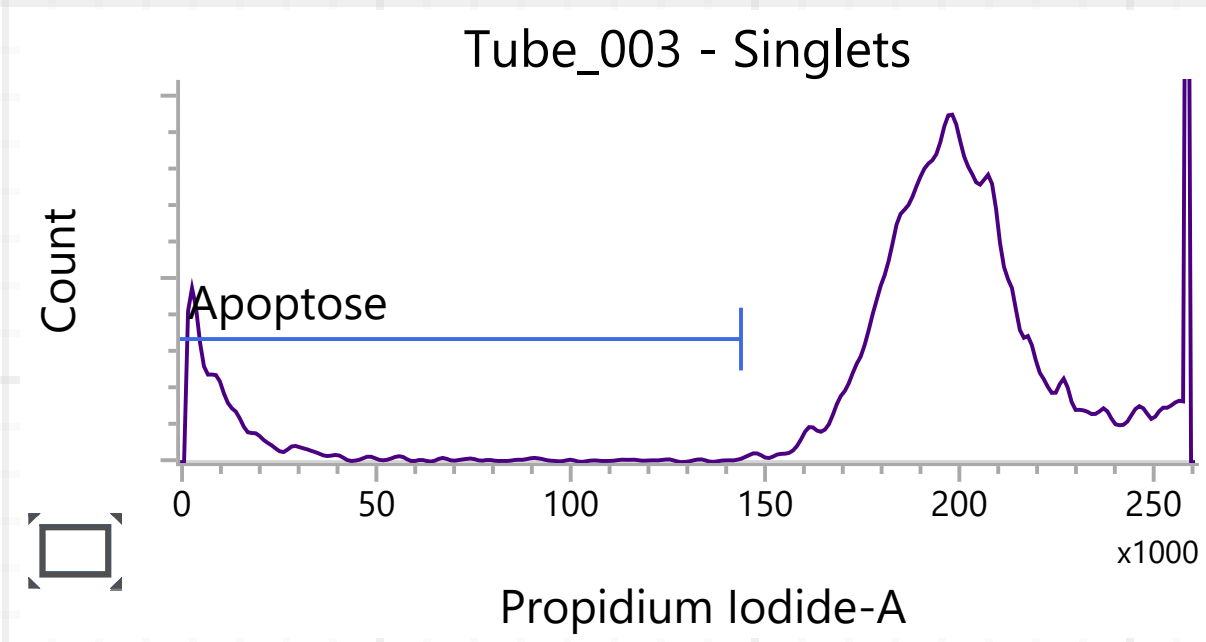

Statistics

Prozent apoptotische Zellen: Prozent apoptotische Zellen 9.3 % von Cells

Name	Events	% Total	% Parent	% Grandparent
Tube_001:All Events	13,360	100.00	***	***
Tube_001:Apoptose	910	6.81	9.26	9.10
Tube_001:Cells	10,000	74.85	74.85	***
Tube_001:Singlets	9,832	73.59	98.32	73.59
Tube_002:All Events	10,000	100.00	***	***
Tube_002:Cells	7,745	77.45	77.45	***
Tube_002:Singlets	7,586	75.86	97.95	75.86
Tube_002:Apoptose	664	6.64	8.75	8.57
Tube_003:All Events	10,000	100.00	***	***
Tube_003:Cells	7,423	74.23	74.23	***
Tube_003:Singlets	7,235	72.35	97.47	72.35
Tube_003:Apoptose	577	5.77	7.98	7.77

DNA Fragmentierung

Sample ID: L2 1

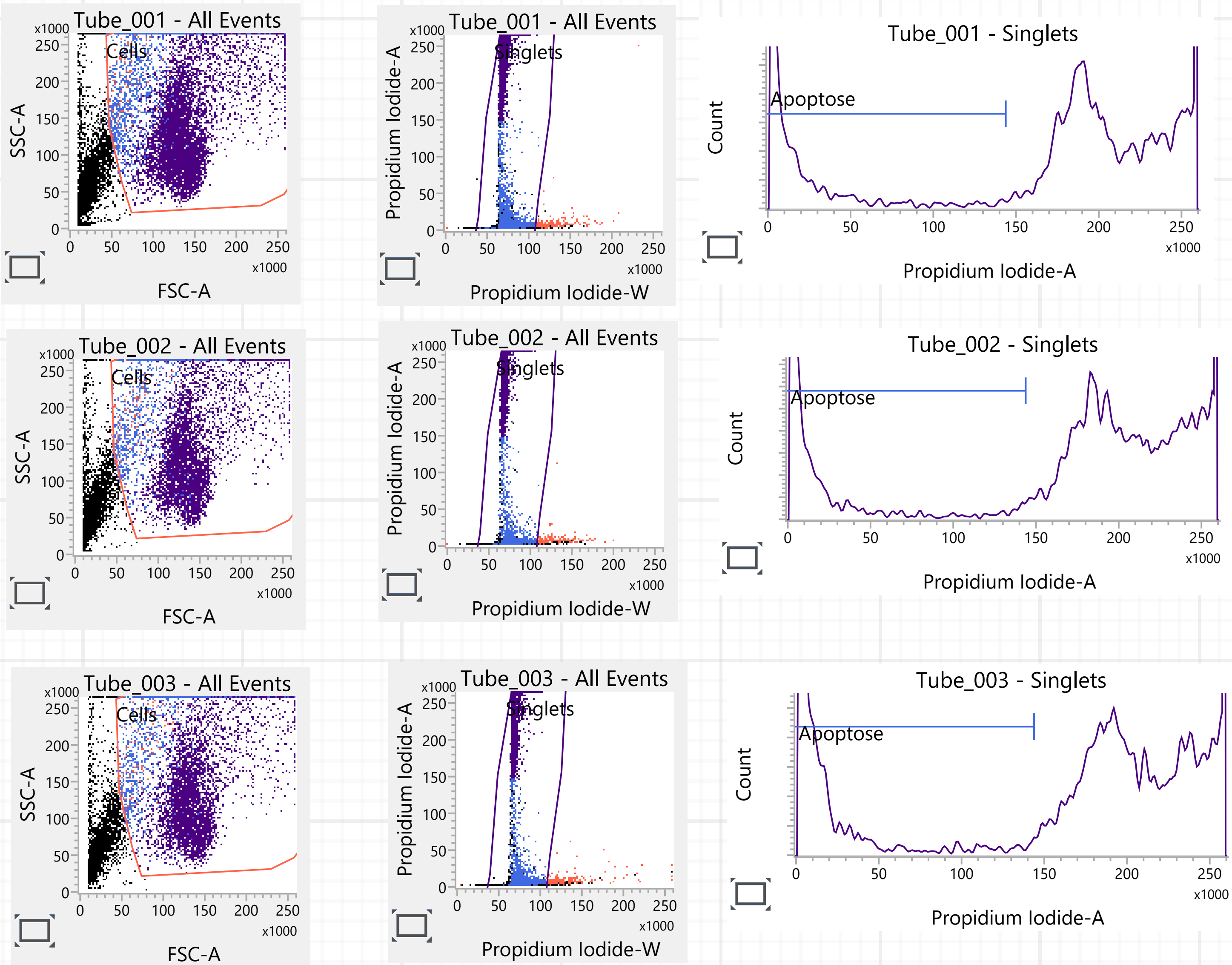

Statistics

Prozent apoptotische Zellen: Prozent apoptotische Zellen 12.7 % von Cells

Name	Events	% Total	% Parent	% Grandparent
Tube_001:All Events	14,695	100.00	***	***
Tube_001:Apoptose	1,244	8.47	12.74	12.44
Tube_001:Cells	10,000	68.05	68.05	***
Tube_001:Singlets	9,768	66.47	97.68	66.47
Tube_002:All Events	10,000	100.00	***	***
Tube_002:Cells	6,656	66.56	66.56	***
Tube_002:Singlets	6,475	64.75	97.28	64.75
Tube_002:Apoptose	852	8.52	13.16	12.80
Tube_003:All Events	10,000	100.00	***	***
Tube_003:Cells	6,470	64.70	64.70	***
Tube_003:Singlets	6,263	62.63	96.80	62.63
Tube_003:Apoptose	841	8.41	13.43	13.00

DNA Fragmentierung

Sample ID: L2 2,5

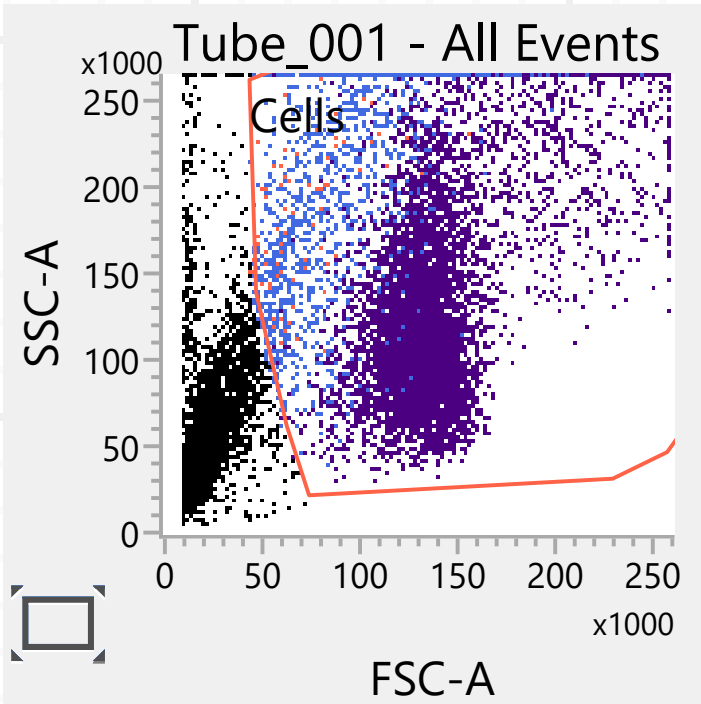

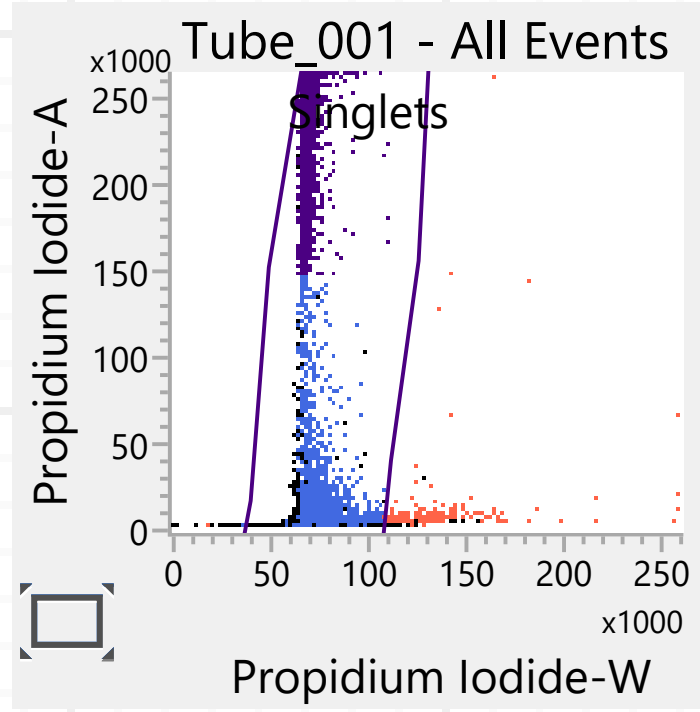

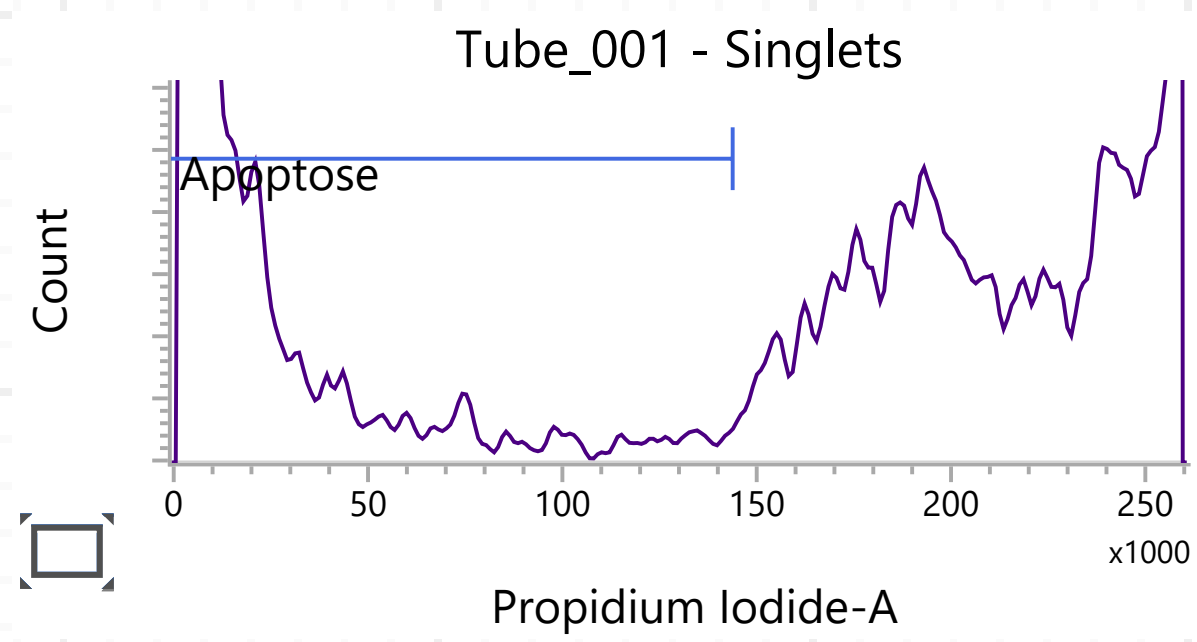

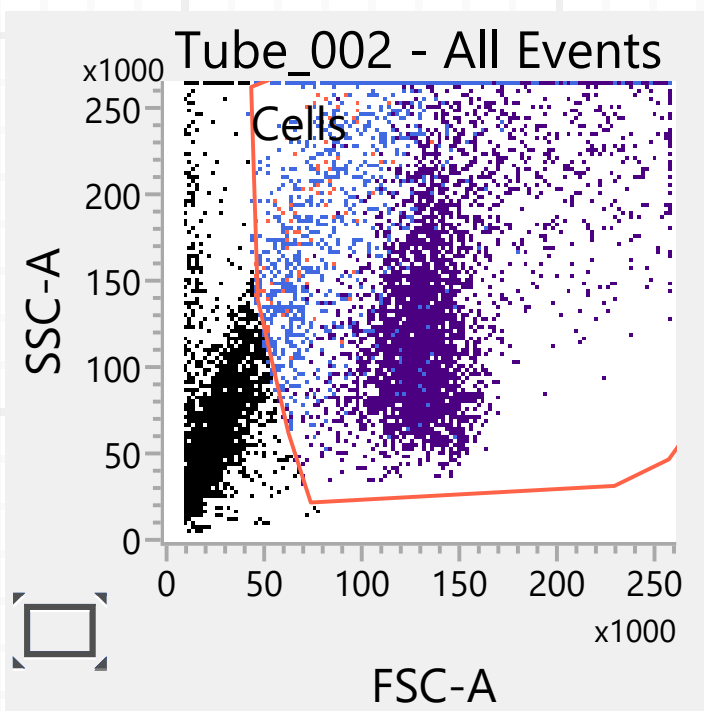

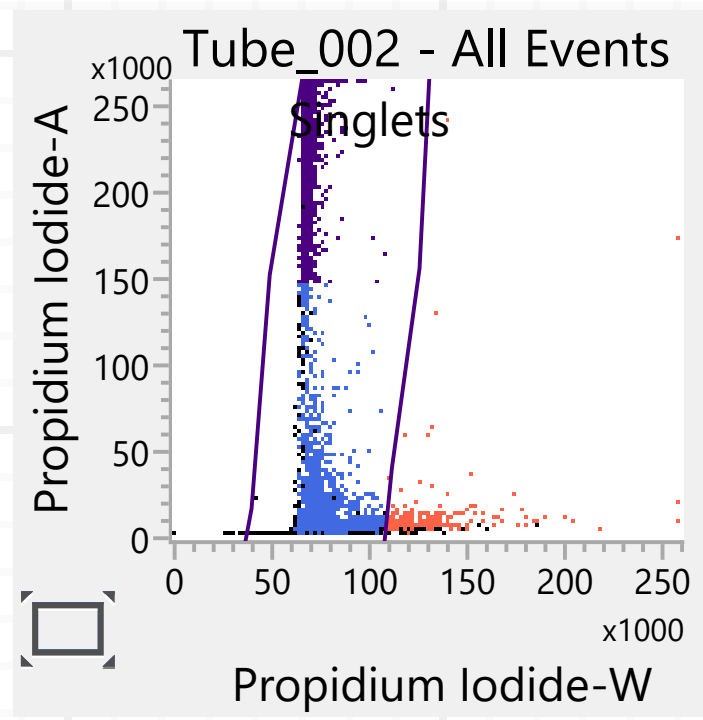

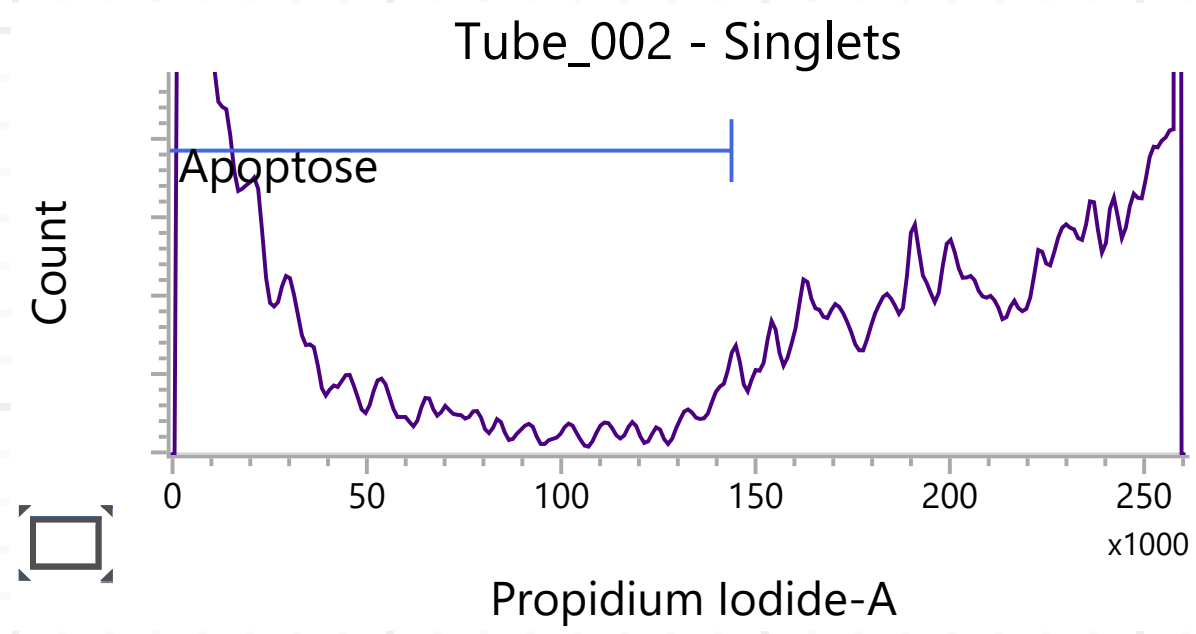

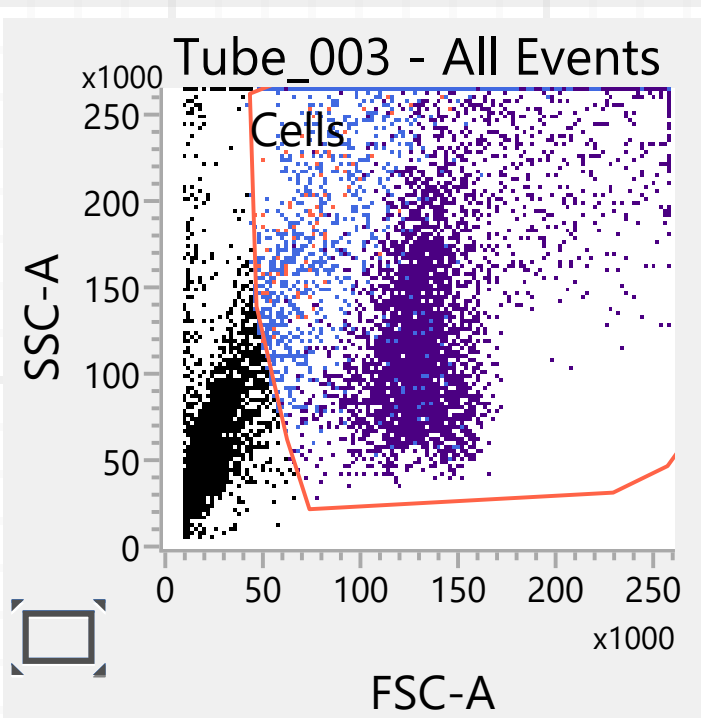

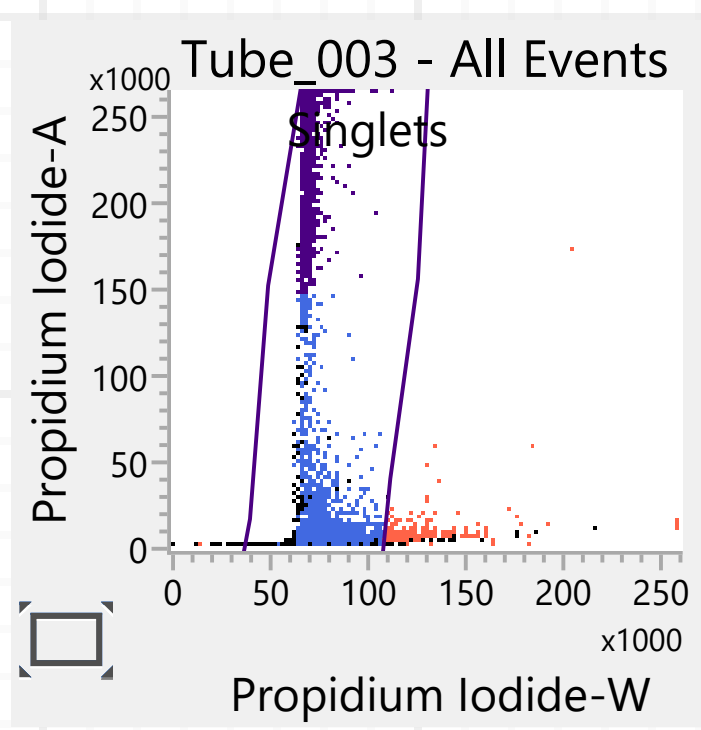

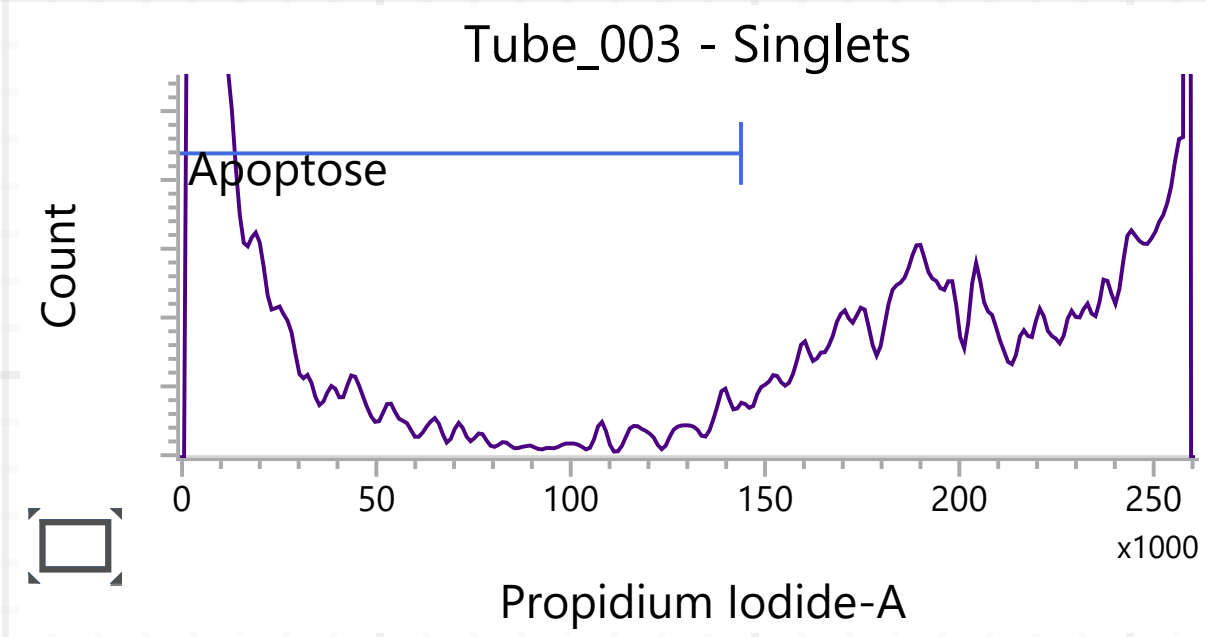

Statistics

Prozent apoptotische Zellen: Prozent apoptotische Zellen 13.9 % von Cells

Name	Events	% Total	% Parent	% Grandparent
Tube_001:All Events	15,198	100.00	***	***
Tube_001:Apoptose	1,366	8.99	13.87	13.66
Tube_001:Cells	10,000	65.80	65.80	***
Tube_001:Singlets	9,847	64.79	98.47	64.79
Tube_002:All Events	10,000	100.00	***	***
Tube_002:Cells	6,754	67.54	67.54	***
Tube_002:Singlets	6,557	65.57	97.08	65.57
Tube_002:Apoptose	1,126	11.26	17.17	16.67
Tube_003:All Events	10,000	100.00	***	***
Tube_003:Cells	6,885	68.85	68.85	***
Tube_003:Singlets	6,702	67.02	97.34	67.02
Tube_003:Apoptose	1,121	11.21	16.73	16.28

DNA Fragmentierung

Sample ID: L2 5

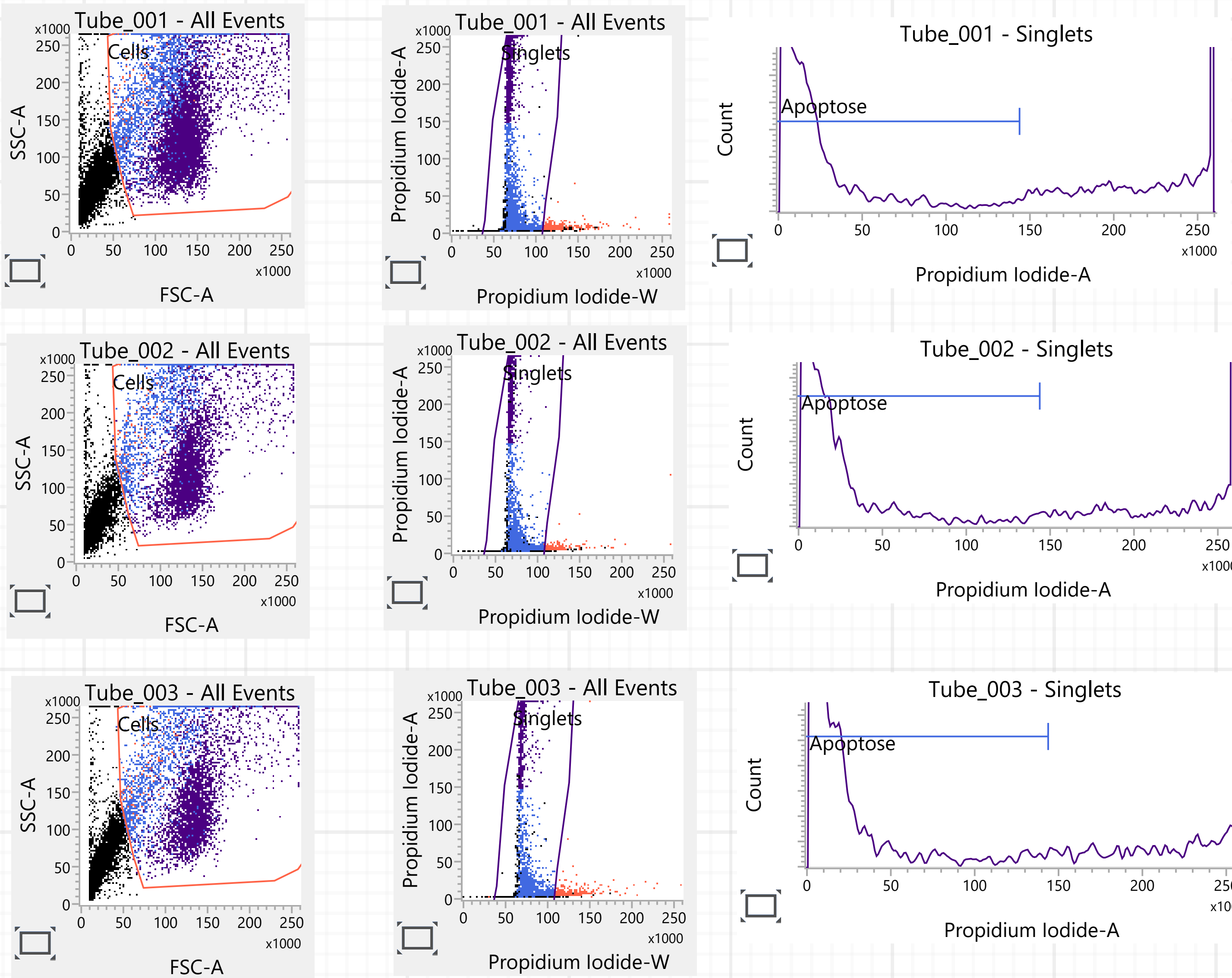

Statistics

Prozent apoptotische Zellen: Prozent apoptotische Zellen 21.6 % von Cells

Name	Events	% Total	% Parent	% Grandparent
Tube_001:All Events	17,531	100.00	***	***
Tube_001:Apoptose	2,109	12.03	21.60	21.09
Tube_001:Cells	10,000	57.04	57.04	***
Tube_001:Singlets	9,766	55.71	97.66	55.71
Tube_002:All Events	10,000	100.00	***	***
Tube_002:Cells	5,845	58.45	58.45	***
Tube_002:Singlets	5,708	57.08	97.66	57.08
Tube_002:Apoptose	1,312	13.12	22.99	22.45
Tube_003:All Events	10,000	100.00	***	***
Tube_003:Cells	5,654	56.54	56.54	***
Tube_003:Singlets	5,413	54.13	95.74	54.13
Tube_003:Apoptose	1,416	14.16	26.16	25.04

DNA Fragmentierung

Sample ID: L2 D

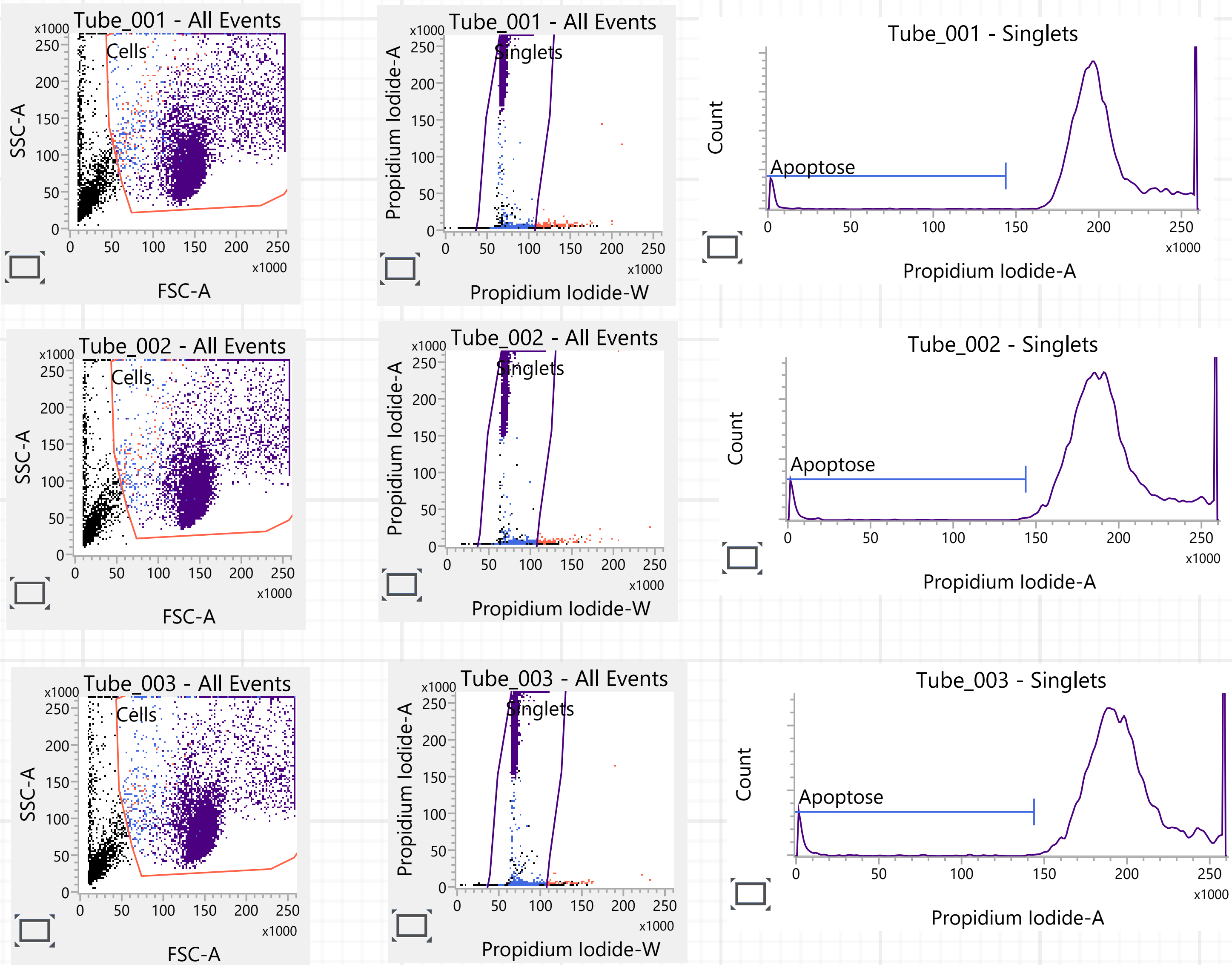

Statistics

Prozent apoptotische Zellen: Prozent apoptotische Zellen 2.6 % von Cells

Name	Events	% Total	% Parent	% Grandparent
Tube_001:All Events	12,029	100.00	***	***
Tube_001:Apoptose	256	2.13	2.59	2.56
Tube_001:Cells	10,000	83.13	83.13	***
Tube_001:Singlets	9,871	82.06	98.71	82.06
Tube_002:All Events	10,000	100.00	***	***
Tube_002:Cells	8,570	85.70	85.70	***
Tube_002:Singlets	8,476	84.76	98.90	84.76
Tube_002:Apoptose	213	2.13	2.51	2.49
Tube_003:All Events	10,000	100.00	***	***
Tube_003:Cells	8,315	83.15	83.15	***
Tube_003:Singlets	8,266	82.66	99.41	82.66
Tube_003:Apoptose	248	2.48	3.00	2.98

DNA Fragmentierung

Sample ID: Leukos1 0,1

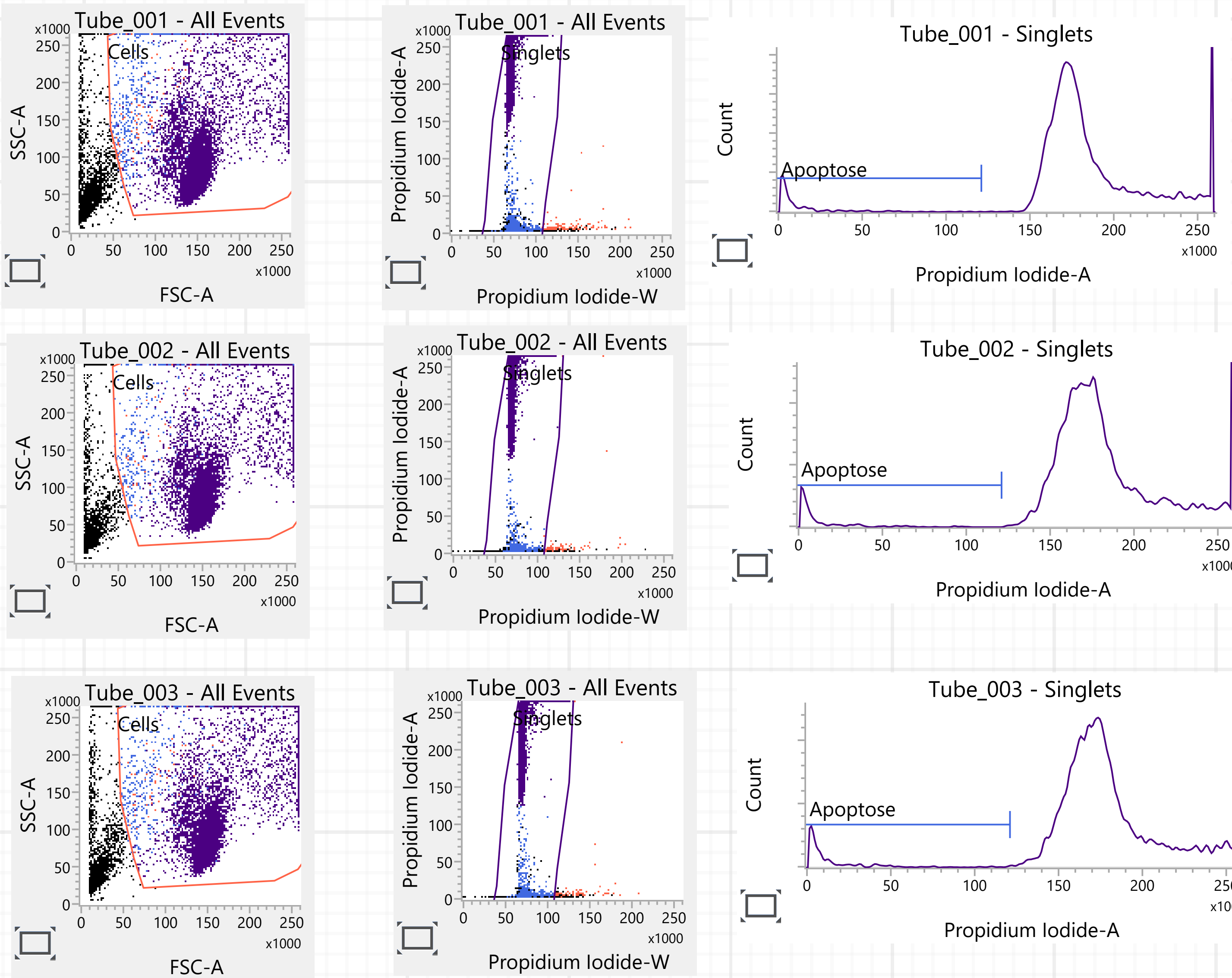

Statistics

Prozent apoptotische Zellen: Prozent apoptotische Zellen 4.6 % von Cells

Name	Events	% Total	% Parent	% Grandparent
Tube_001:All Events	12,091	100.00	***	***
Tube_001:Cells	10,000	82.71	82.71	***
Tube_001:Singlets	9,889	81.79	98.89	81.79
Tube_002:All Events	10,000	100.00	***	***
Tube_002:Cells	8,528	85.28	85.28	***
Tube_002:Singlets	8,463	84.63	99.24	84.63
Tube_003:All Events	10,000	100.00	***	***
Tube_003:Cells	8,421	84.21	84.21	***
Tube_003:Singlets	8,356	83.56	99.23	83.56
Tube_001:Apoptose	453	3.75	4.58	4.53
Tube_002:Apoptose	297	2.97	3.51	3.48
Tube_003:Apoptose	343	3.43	4.10	4.07

DNA Fragmentierung

Sample ID: Leukos1 0,5

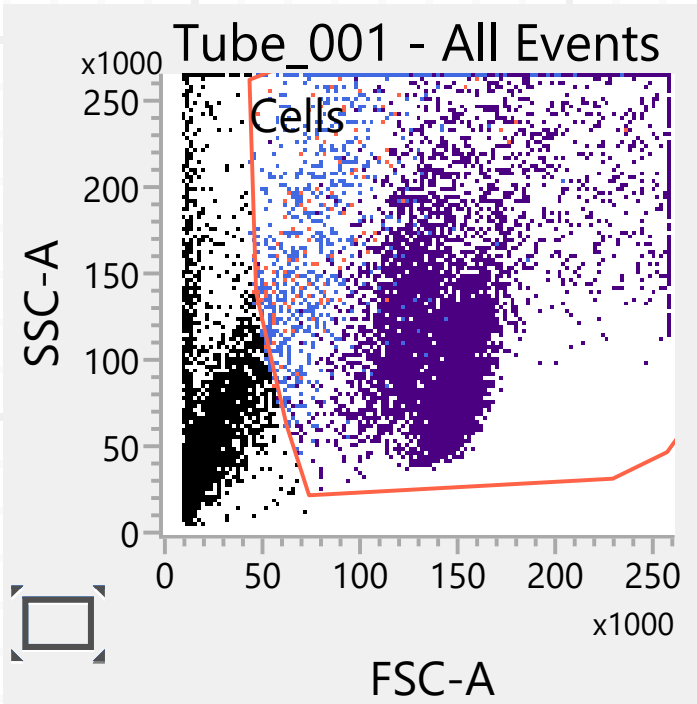

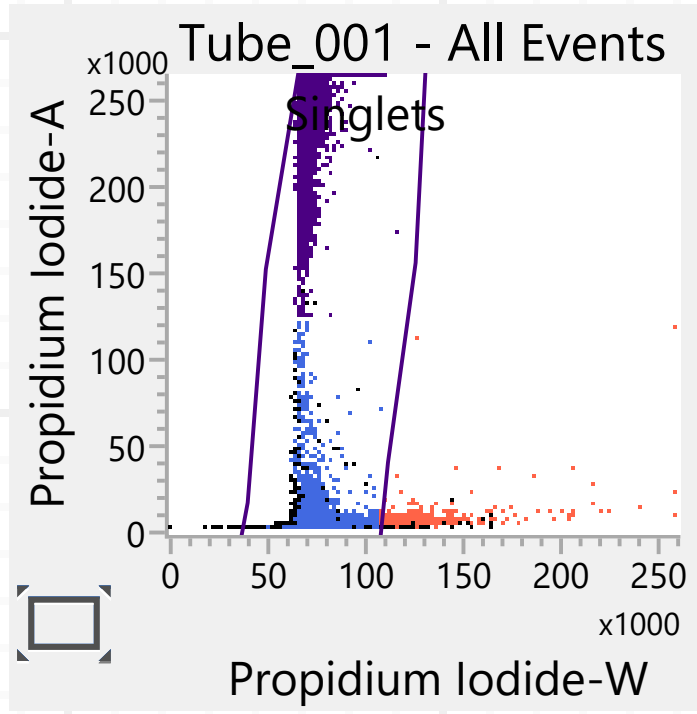

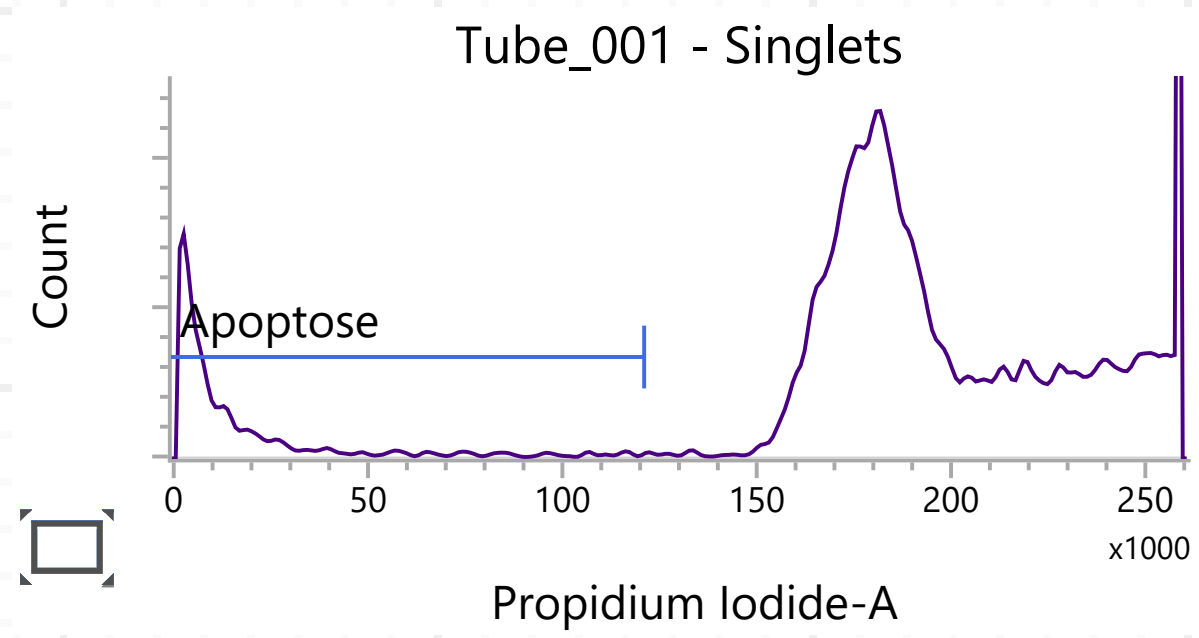

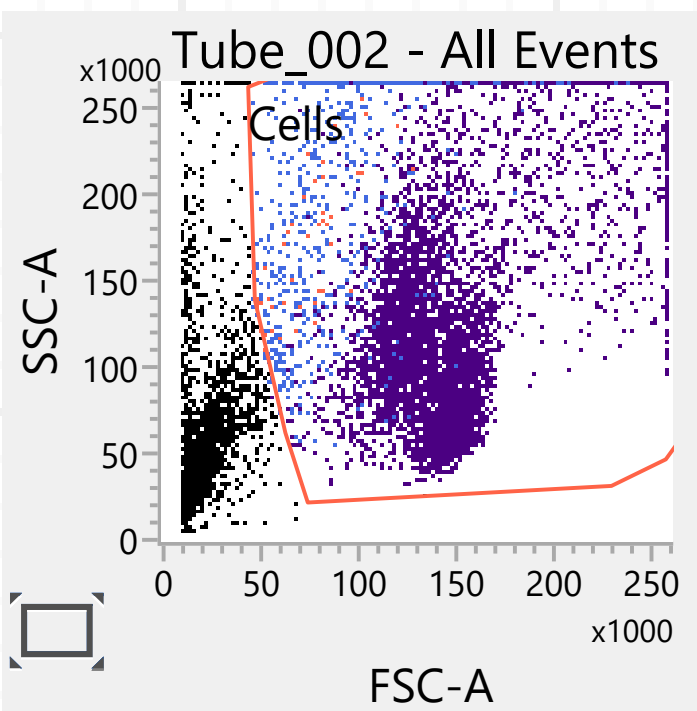

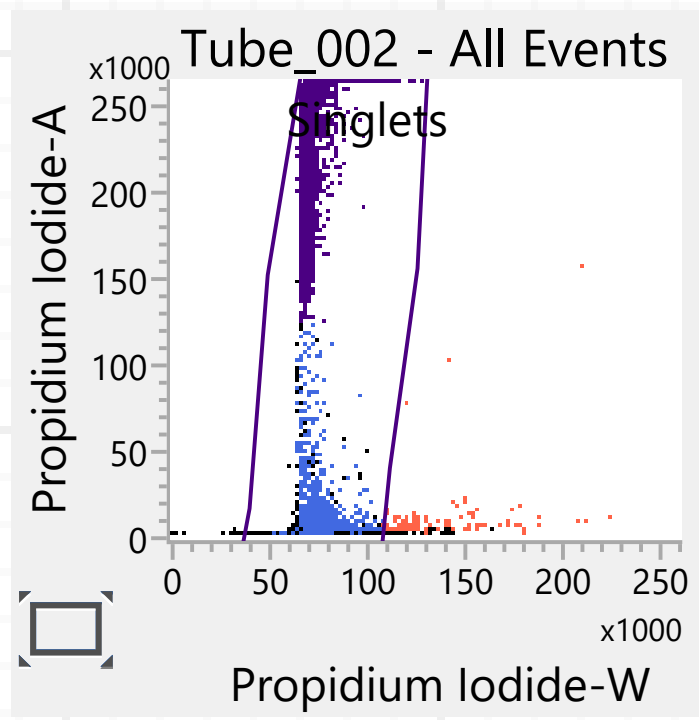

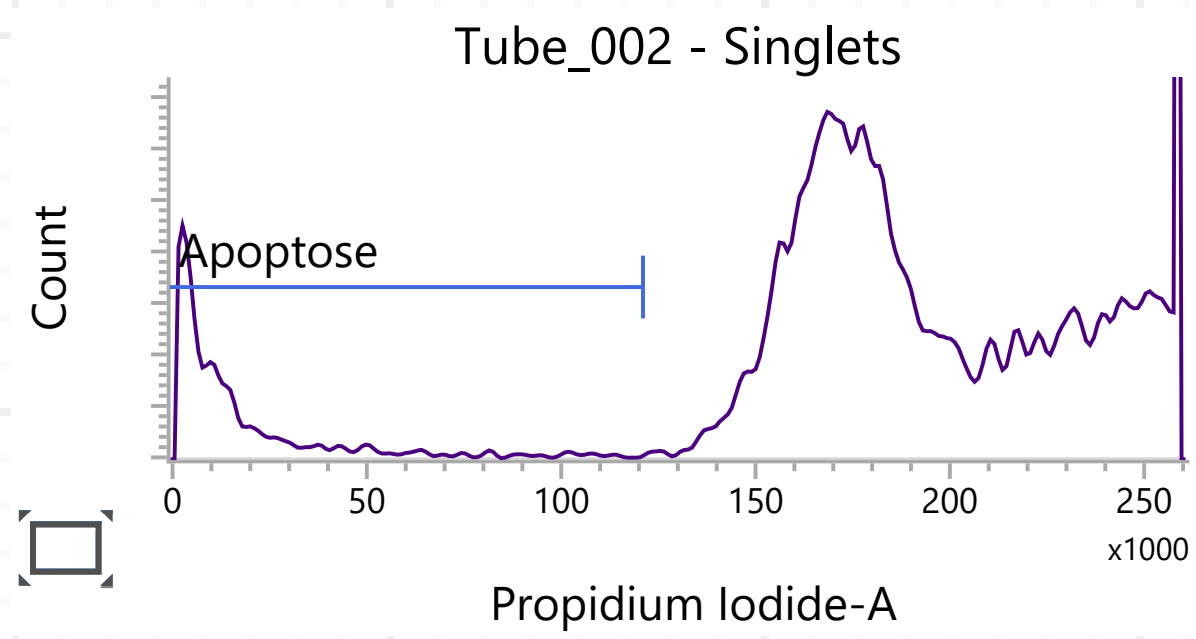

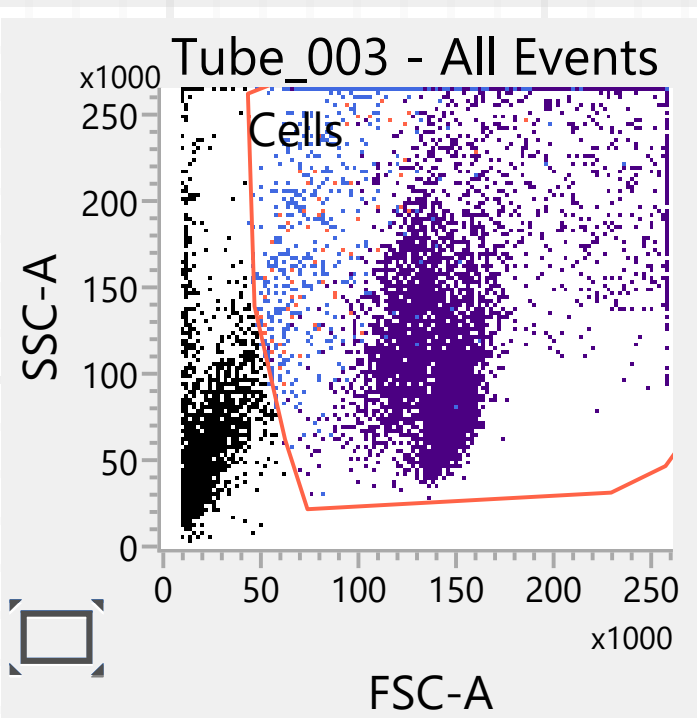

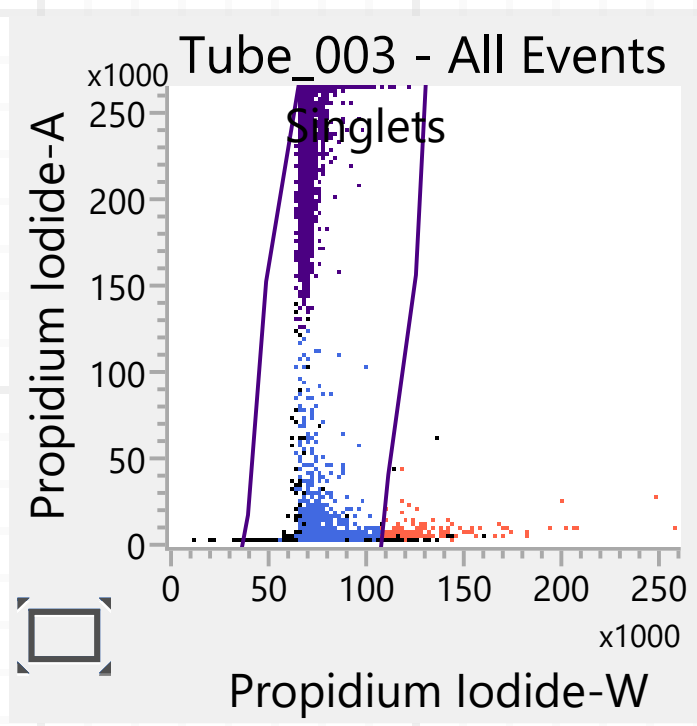

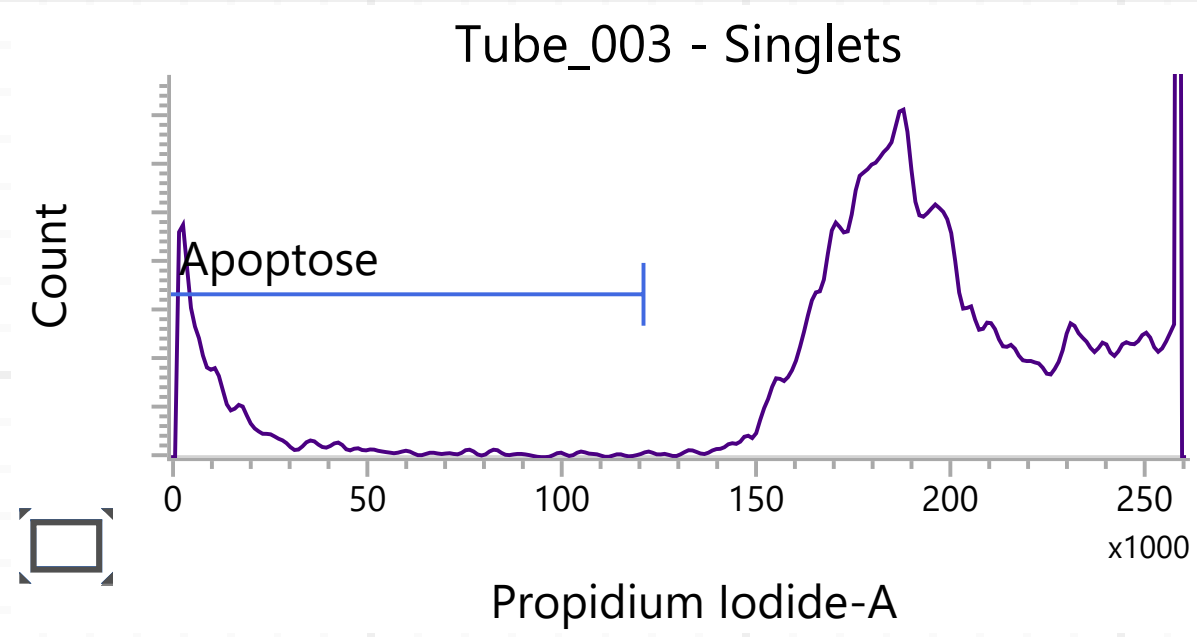

Statistics

Prozent apoptotische Zellen: Prozent apoptotische Zellen 8.5 % von Cells

Name	Events	% Total	% Parent	% Grandparent
Tube_001:All Events	13,945	100.00	***	***
Tube_001:Cells	10,000	71.71	71.71	***
Tube_001:Singlets	9,769	70.05	97.69	70.05
Tube_002:All Events	10,000	100.00	***	***
Tube_002:Cells	7,727	77.27	77.27	***
Tube_002:Singlets	7,622	76.22	98.64	76.22
Tube_003:All Events	10,000	100.00	***	***
Tube_003:Cells	7,545	75.45	75.45	***
Tube_003:Singlets	7,415	74.15	98.28	74.15
Tube_001:Apoptose	835	5.99	8.55	8.35
Tube_002:Apoptose	593	5.93	7.78	7.67
Tube_003:Apoptose	581	5.81	7.84	7.70

DNA Fragmentierung

Sample ID: Leukos1 0,25

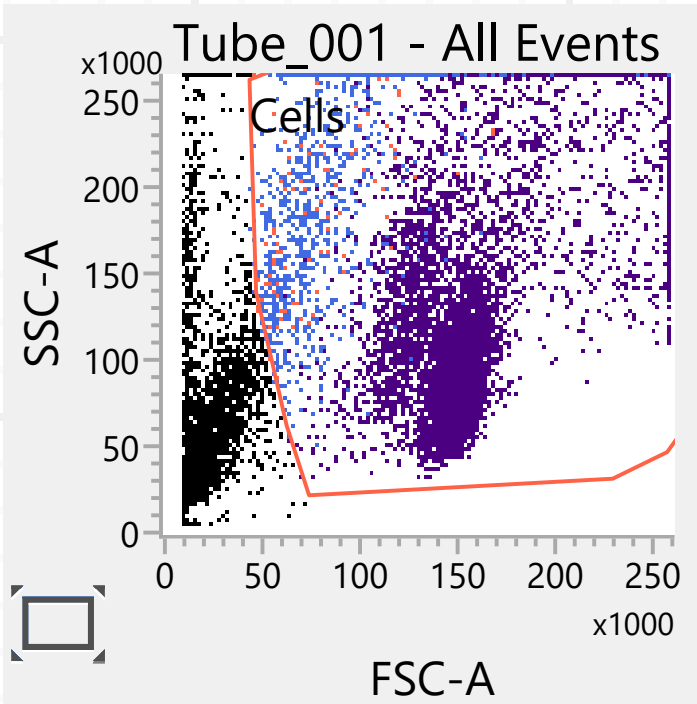

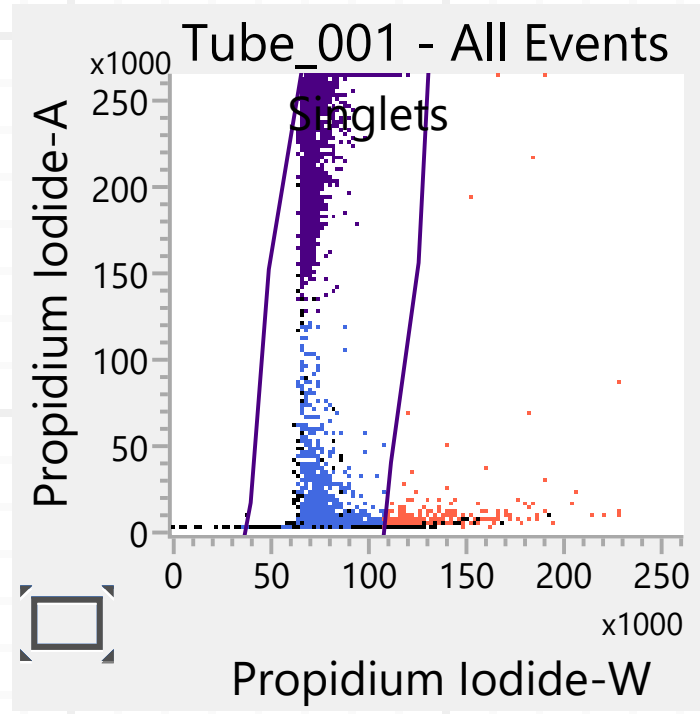

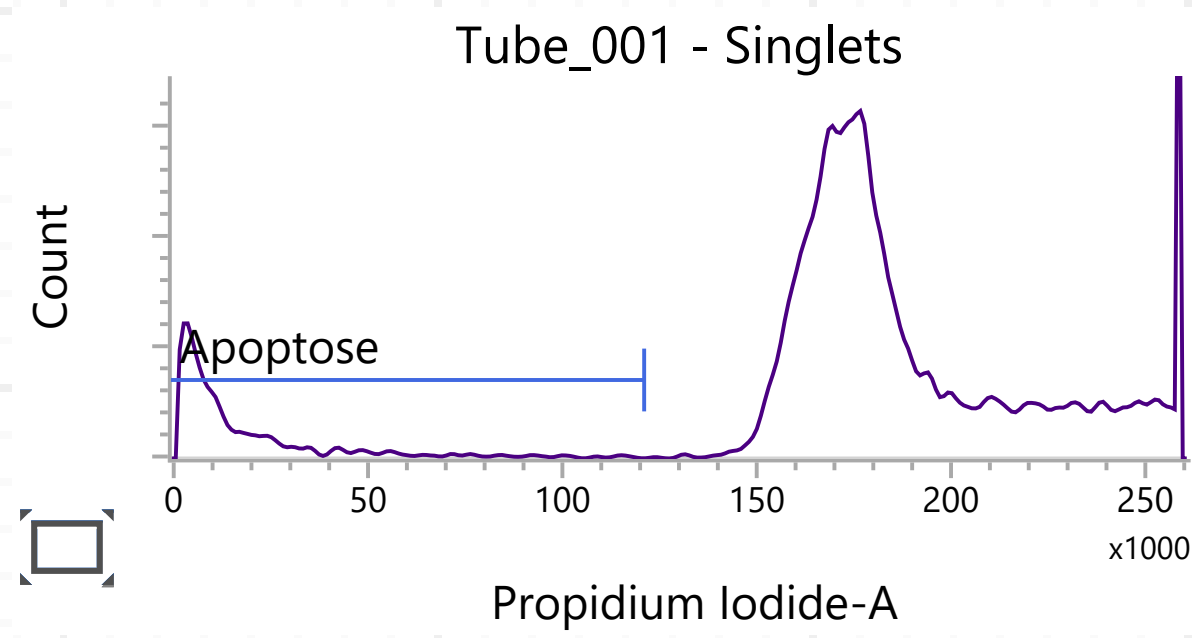

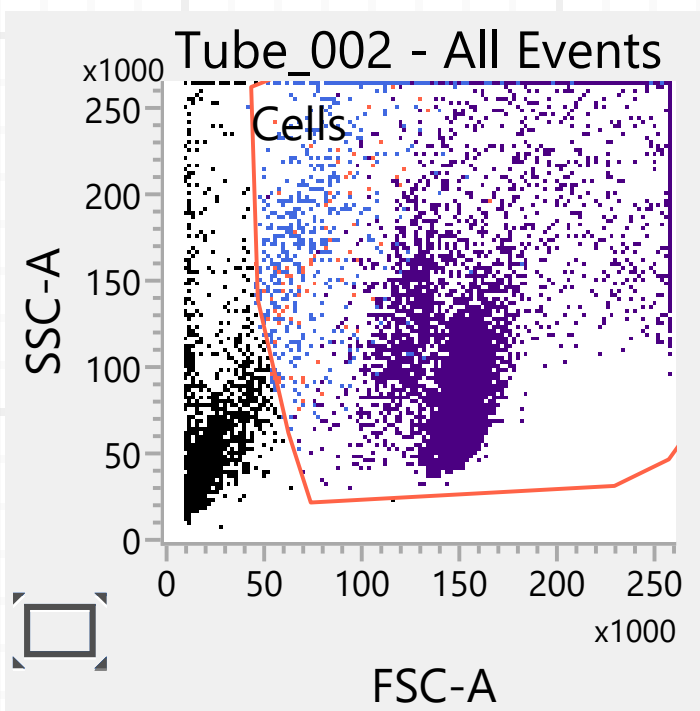

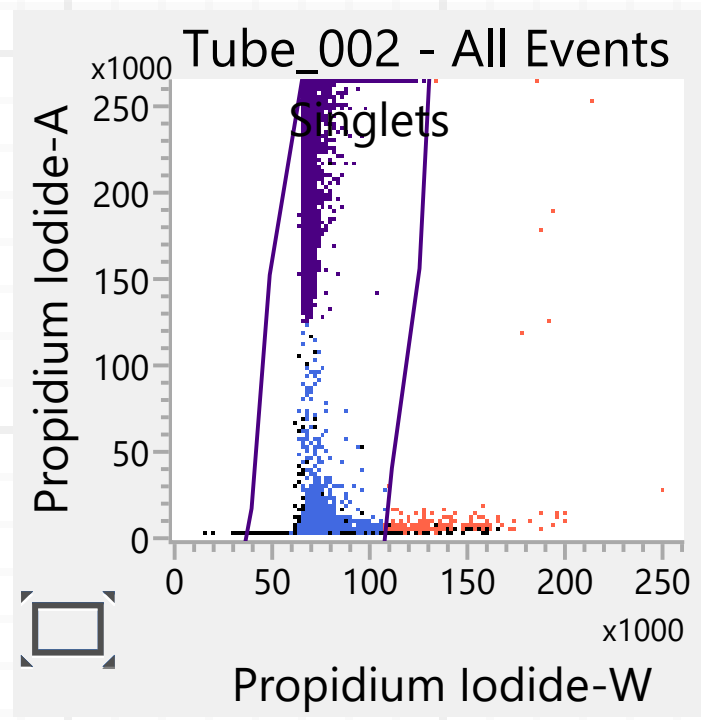

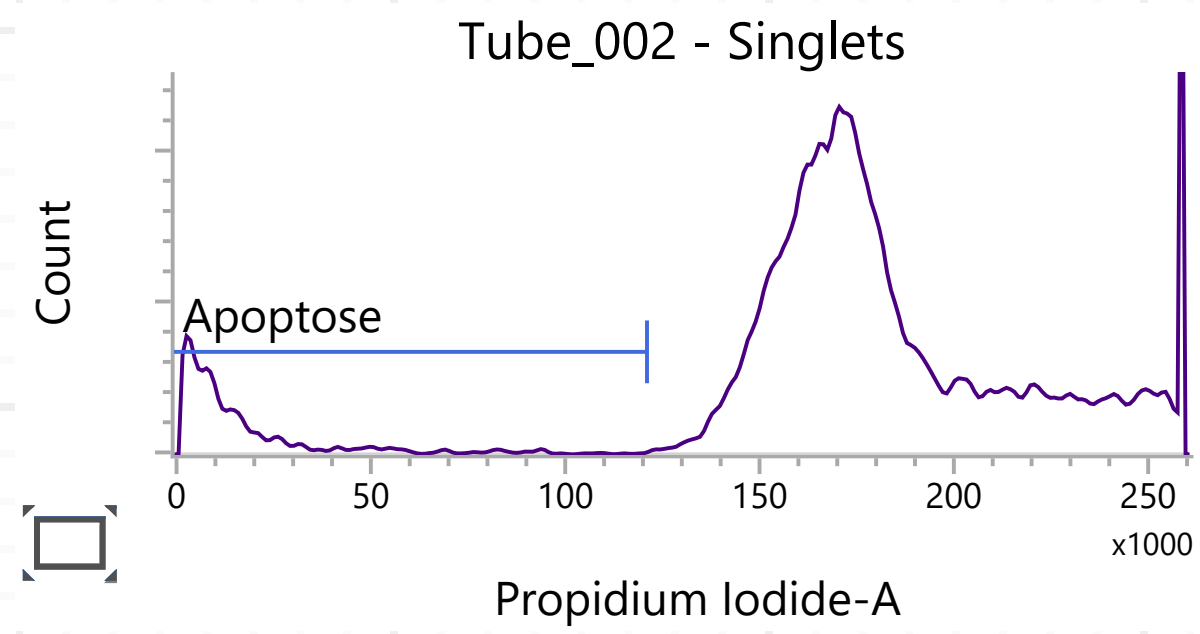

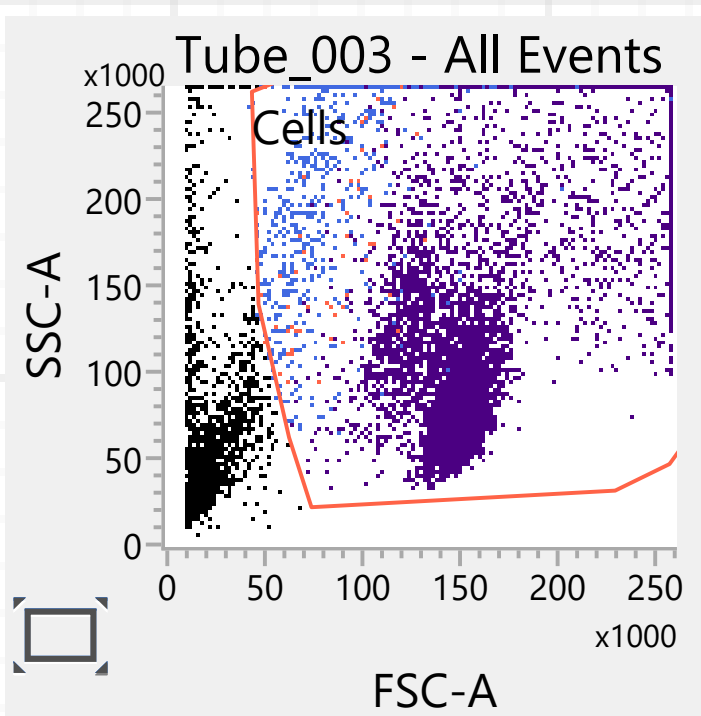

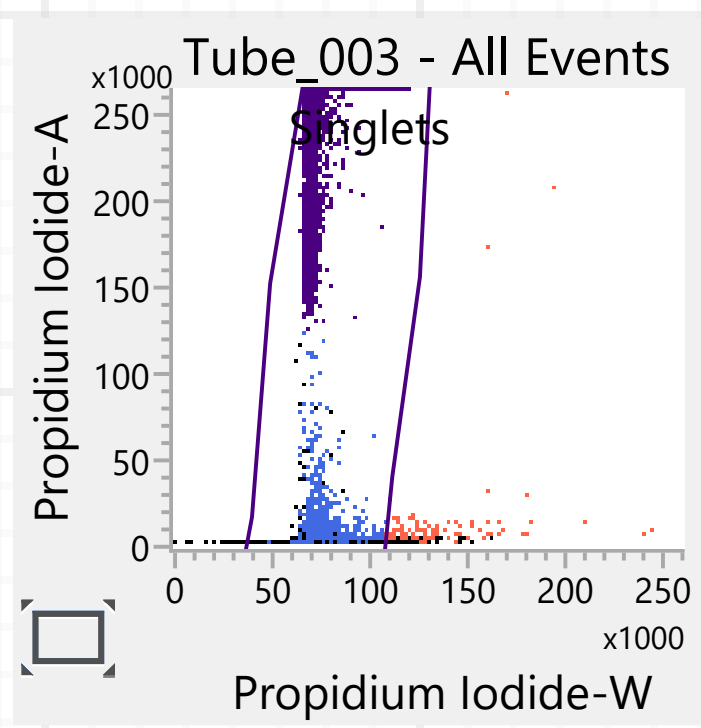

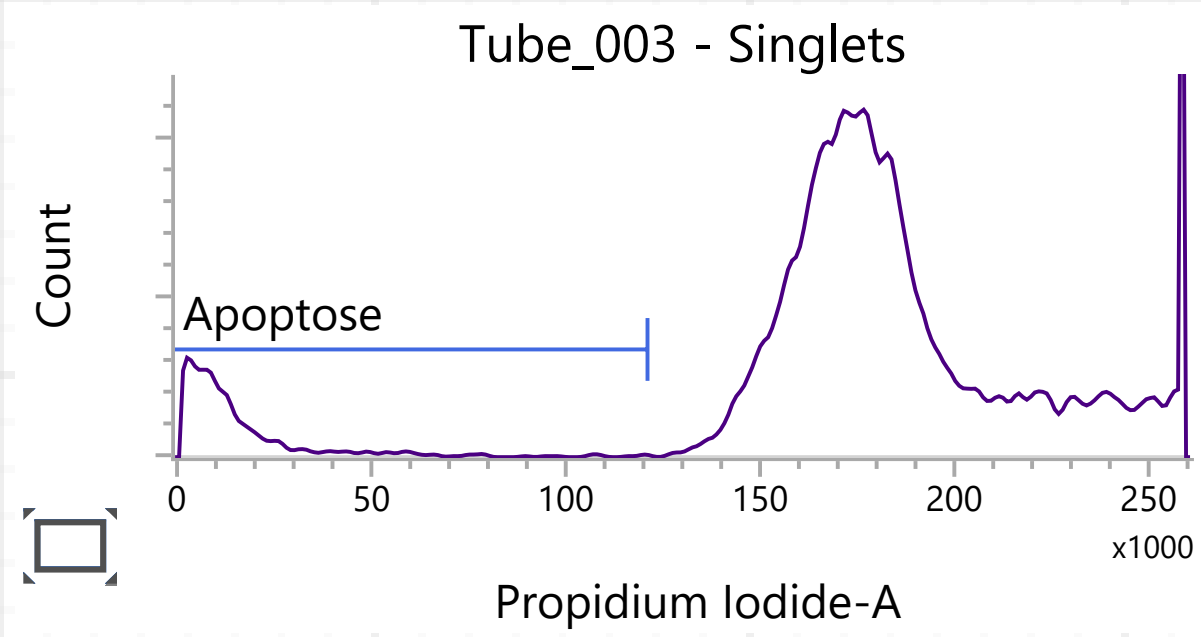

Statistics

Prozent apoptotische Zellen: Prozent apoptotische Zellen 8.8 % von Cells

Name	Events	% Total	% Parent	% Grandparent
Tube_001:All Events	12,823	100.00	***	***
Tube_001:Cells	10,000	77.98	77.98	***
Tube_001:Singlets	9,787	76.32	97.87	76.32
Tube_002:All Events	10,000	100.00	***	***
Tube_002:Cells	8,261	82.61	82.61	***
Tube_002:Singlets	8,125	81.25	98.35	81.25
Tube_003:All Events	10,000	100.00	***	***
Tube_003:Cells	8,280	82.80	82.80	***
Tube_003:Singlets	8,178	81.78	98.77	81.78
Tube_001:Apoptose	863	6.73	8.82	8.63
Tube_002:Apoptose	582	5.82	7.16	7.05
Tube_003:Apoptose	544	5.44	6.65	6.57

DNA Fragmentierung

Sample ID: Leukos1 1

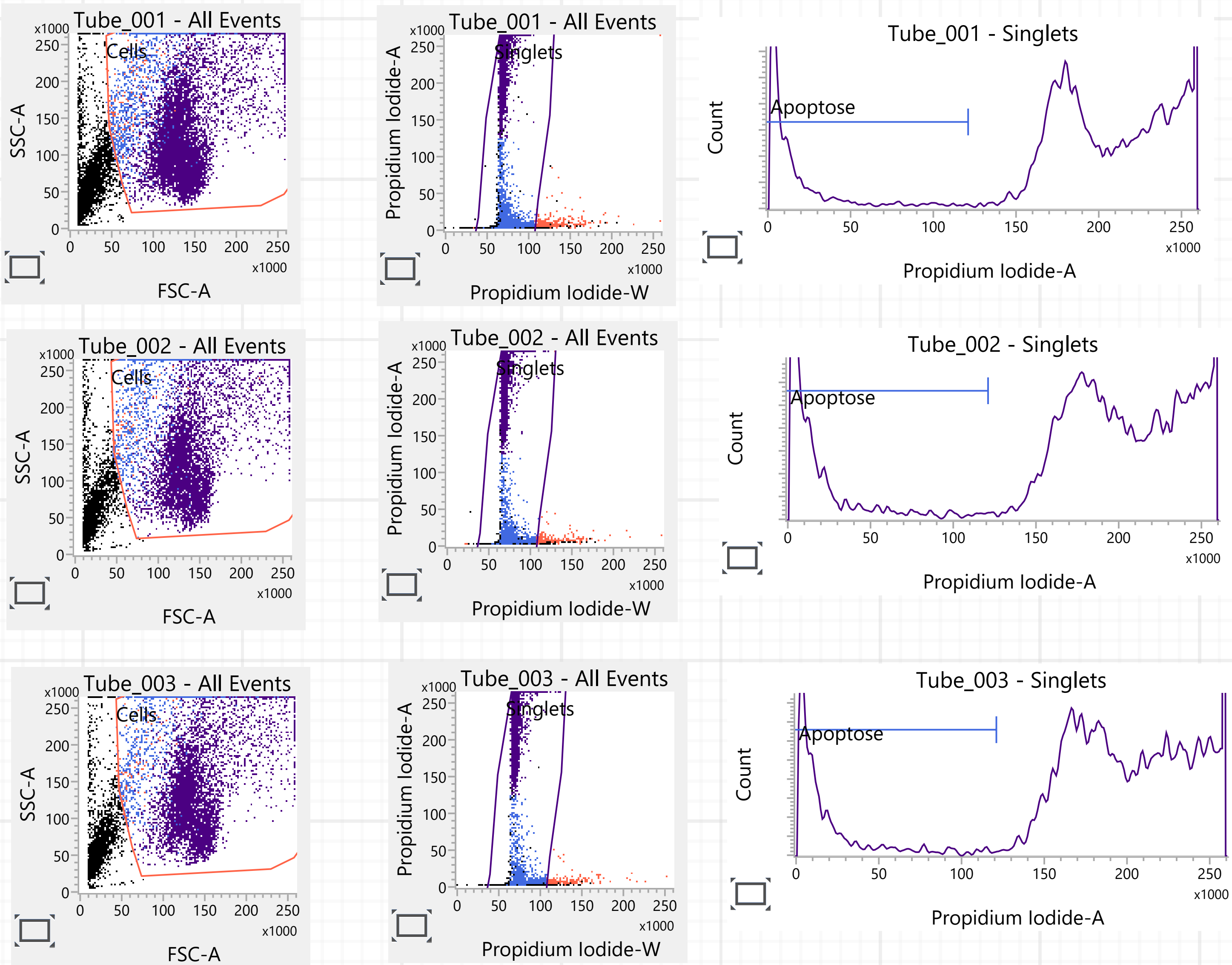

Statistics

Prozent apoptotische Zellen: Prozent apoptotische Zellen 10.1 % von Cells

Name	Events	% Total	% Parent	% Grandparent
Tube_001:All Events	14,345	100.00	***	***
Tube_001:Cells	10,000	69.71	69.71	***
Tube_001:Singlets	9,739	67.89	97.39	67.89
Tube_002:All Events	10,000	100.00	***	***
Tube_002:Cells	7,344	73.44	73.44	***
Tube_002:Singlets	7,176	71.76	97.71	71.76
Tube_003:All Events	10,000	100.00	***	***
Tube_003:Cells	7,786	77.86	77.86	***
Tube_003:Singlets	7,624	76.24	97.92	76.24
Tube_001:Apoptose	985	6.87	10.11	9.85
Tube_002:Apoptose	794	7.94	11.06	10.81
Tube_003:Apoptose	716	7.16	9.39	9.20

DNA Fragmentierung

Sample ID: Leukos1 2,5

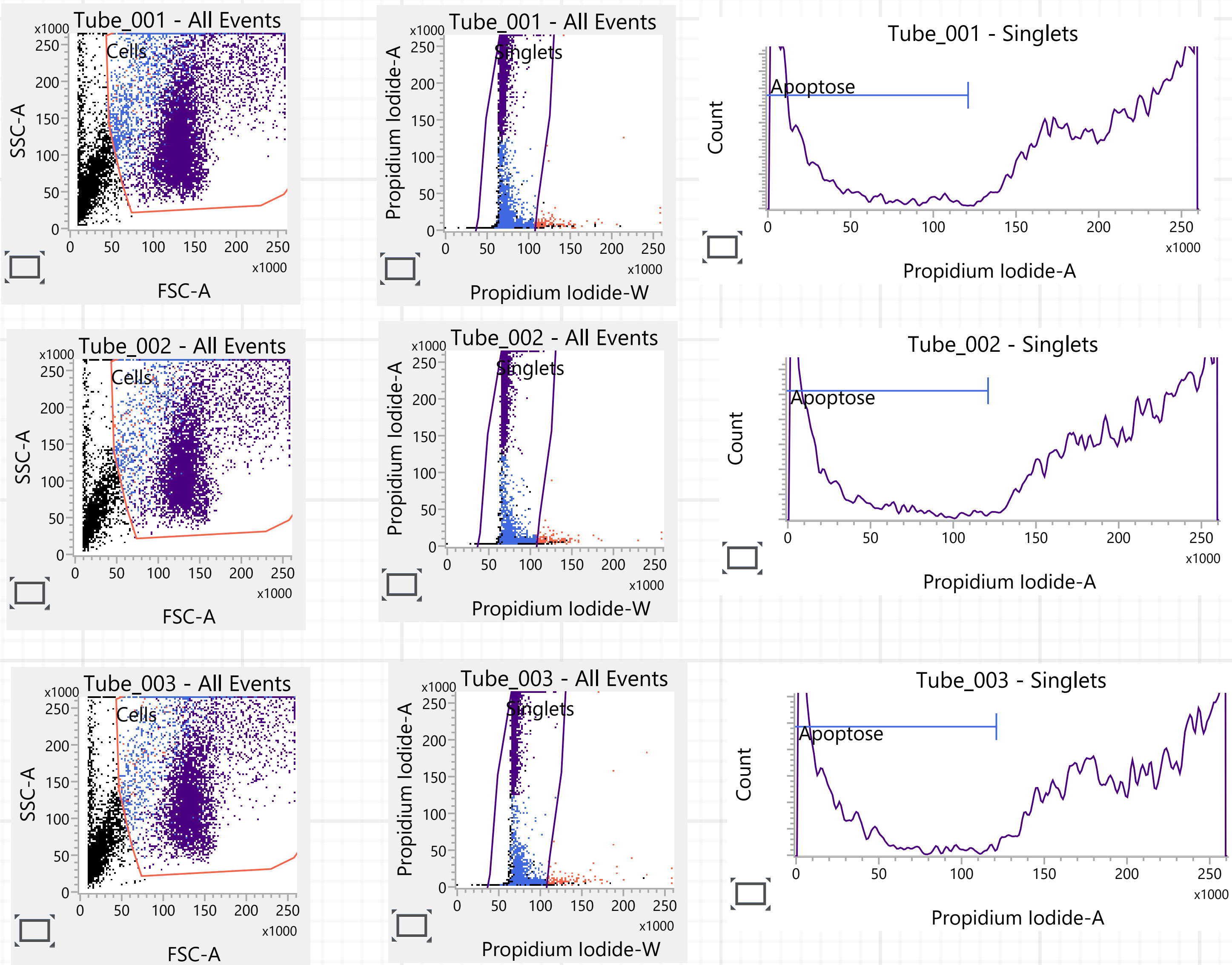

Statistics

Prozent apoptotische Zellen: Prozent apoptotische Zellen 13.5 % von Cells

Name	Events	% Total	% Parent	% Grandparent
Tube_001:All Events	13,938	100.00	***	***
Tube_001:Cells	10,000	71.75	71.75	***
Tube_001:Singlets	9,881	70.89	98.81	70.89
Tube_002:All Events	10,000	100.00	***	***
Tube_002:Cells	7,706	77.06	77.06	***
Tube_002:Singlets	7,599	75.99	98.61	75.99
Tube_003:All Events	10,000	100.00	***	***
Tube_003:Cells	6,811	68.11	68.11	***
Tube_003:Singlets	6,693	66.93	98.27	66.93
Tube_001:Apoptose	1,335	9.58	13.51	13.35
Tube_002:Apoptose	820	8.20	10.79	10.64
Tube_003:Apoptose	846	8.46	12.64	12.42

DNA Fragmentierung

Sample ID: Leukos1 5

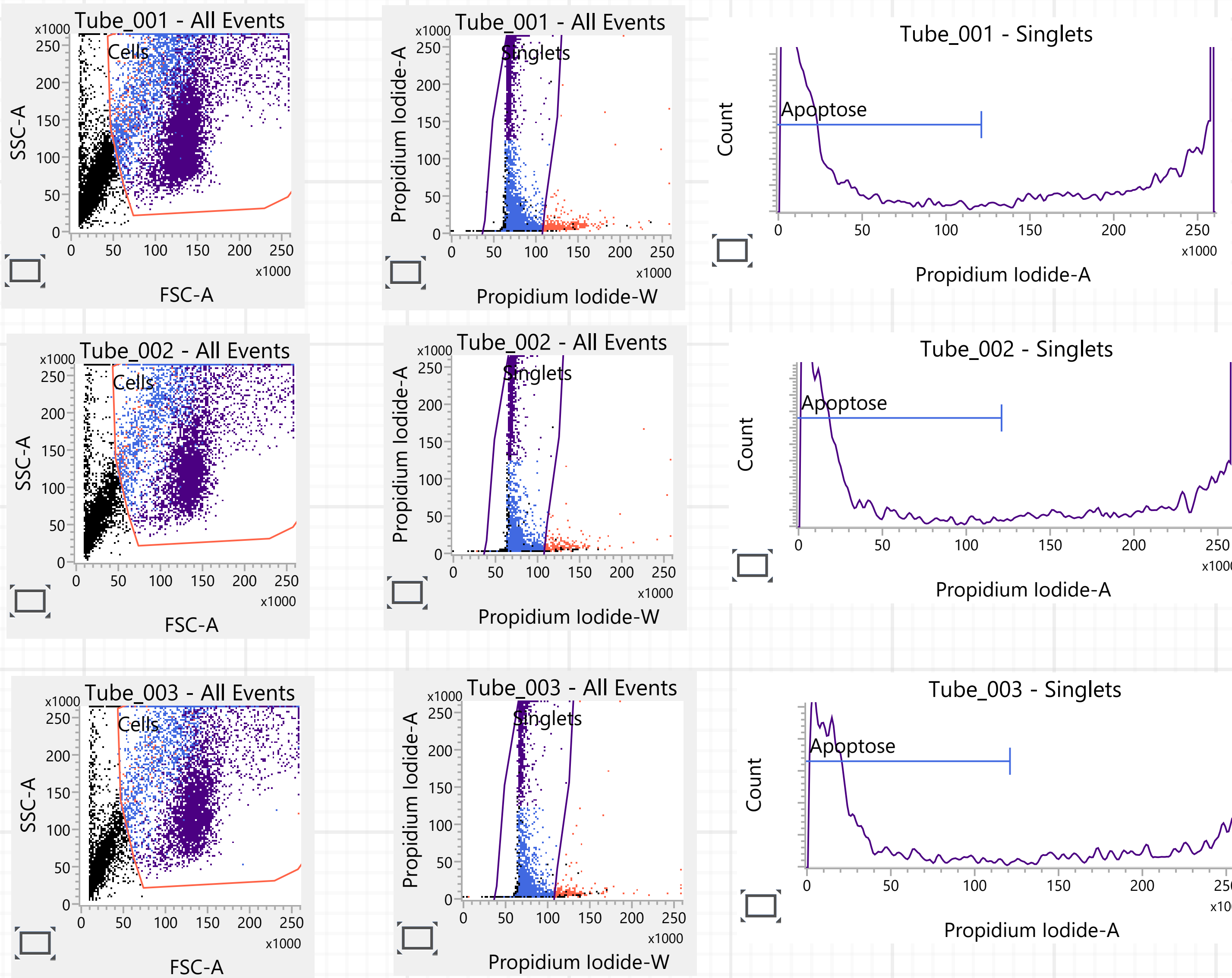

Statistics

Prozent apoptotische Zellen: Prozent apoptotische Zellen 21.3 % von Cells

Name	Events	% Total	% Parent	% Grandparent
Tube_001:All Events	15,211	100.00	***	***
Tube_001:Cells	10,000	65.74	65.74	***
Tube_001:Singlets	9,659	63.50	96.59	63.50
Tube_002:All Events	10,000	100.00	***	***
Tube_002:Cells	6,541	65.41	65.41	***
Tube_002:Singlets	6,336	63.36	96.87	63.36
Tube_003:All Events	10,000	100.00	***	***
Tube_003:Cells	6,847	68.47	68.47	***
Tube_003:Singlets	6,702	67.02	97.88	67.02
Tube_001:Apoptose	2,062	13.56	21.35	20.62
Tube_002:Apoptose	1,459	14.59	23.03	22.31
Tube_003:Apoptose	1,413	14.13	21.08	20.64

DNA Fragmentierung

Sample ID: Leukos1 D

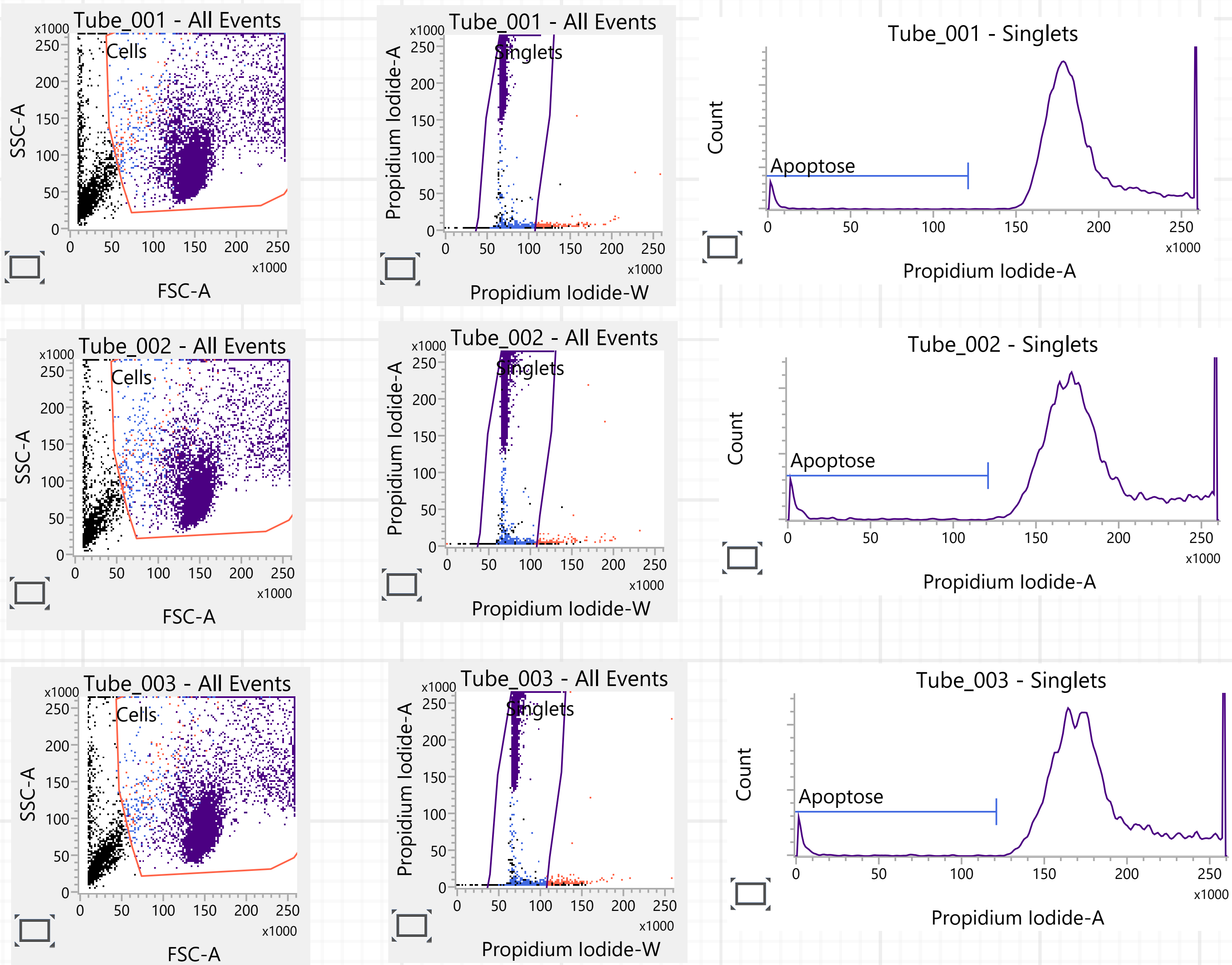

Statistics

Prozent apoptotische Zellen: Prozent apoptotische Zellen 2.2 % von Cells

Name	Events	% Total	% Parent	% Grandparent
Tube_001:All Events	11,958	100.00	***	***
Tube_001:Cells	10,000	83.63	83.63	***
Tube_001:Singlets	9,890	82.71	98.90	82.71
Tube_002:All Events	10,000	100.00	***	***
Tube_002:Cells	8,492	84.92	84.92	***
Tube_002:Singlets	8,397	83.97	98.88	83.97
Tube_003:All Events	10,000	100.00	***	***
Tube_003:Cells	8,255	82.55	82.55	***
Tube_003:Singlets	8,139	81.39	98.59	81.39
Tube_002:Apoptose	263	2.63	3.13	3.10
Tube_001:Apoptose	216	1.81	2.18	2.16
Tube_003:Apoptose	210	2.10	2.58	2.54

DNA Fragmentierung

Sample ID: Leukos1 K

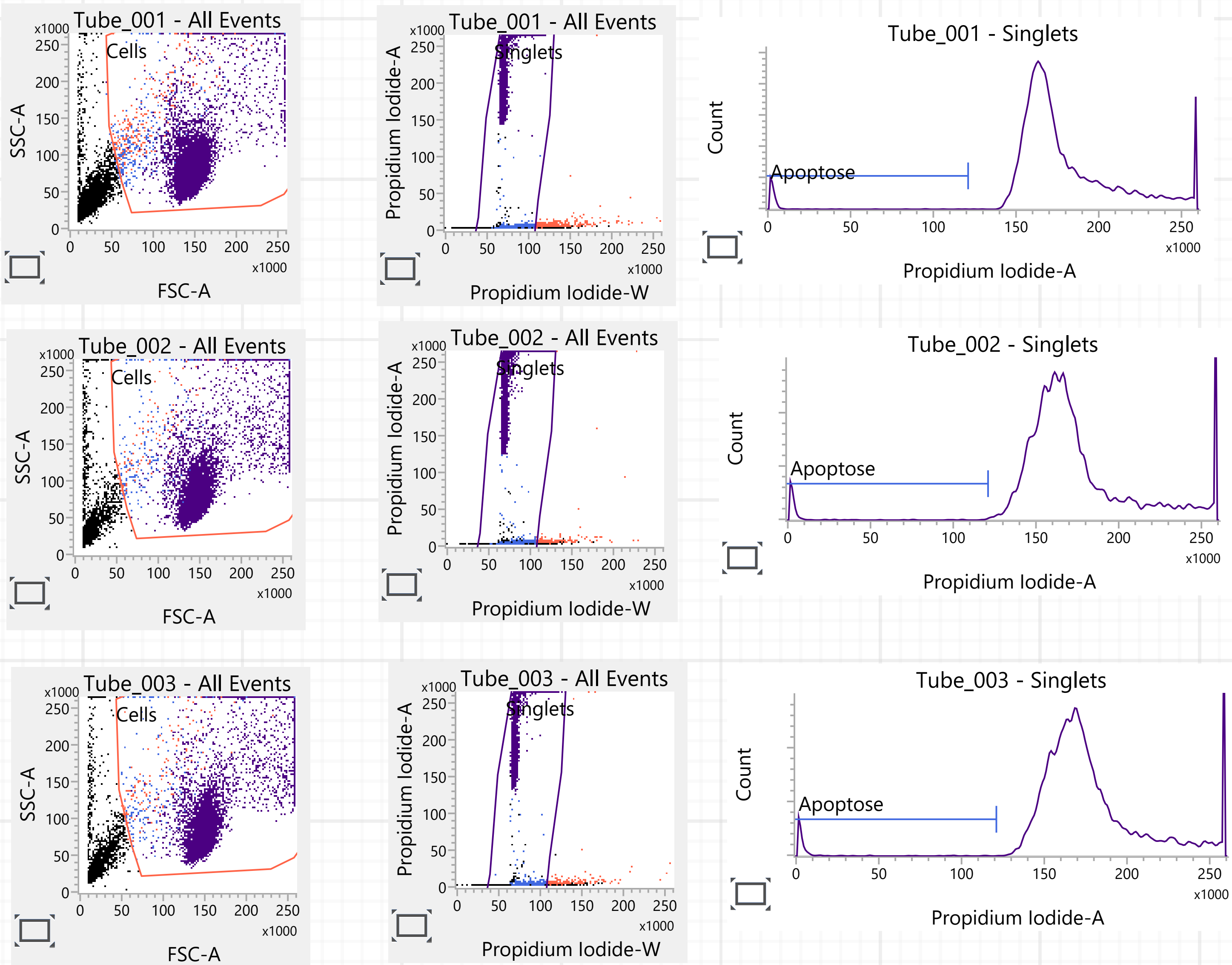

Statistics

Prozent apoptotische Zellen: Prozent apoptotische Zellen 2.5 % von Cells

Name	Events	% Total	% Parent	% Grandparent
Tube_001:All Events	13,326	100.00	***	***
Tube_001:Apoptose	245	1.84	2.53	2.45
Tube_001:Cells	10,000	75.04	75.04	***
Tube_001:Singlets	9,691	72.72	96.91	72.72
Tube_002:All Events	10,000	100.00	***	***
Tube_002:Cells	8,280	82.80	82.80	***
Tube_002:Singlets	8,157	81.57	98.51	81.57
Tube_002:Apoptose	193	1.93	2.37	2.33
Tube_003:All Events	10,000	100.00	***	***
Tube_003:Cells	8,305	83.05	83.05	***
Tube_003:Singlets	8,144	81.44	98.06	81.44
Tube_003:Apoptose	184	1.84	2.26	2.22

DNA Fragmentierung

Sample ID: Leukos2 K

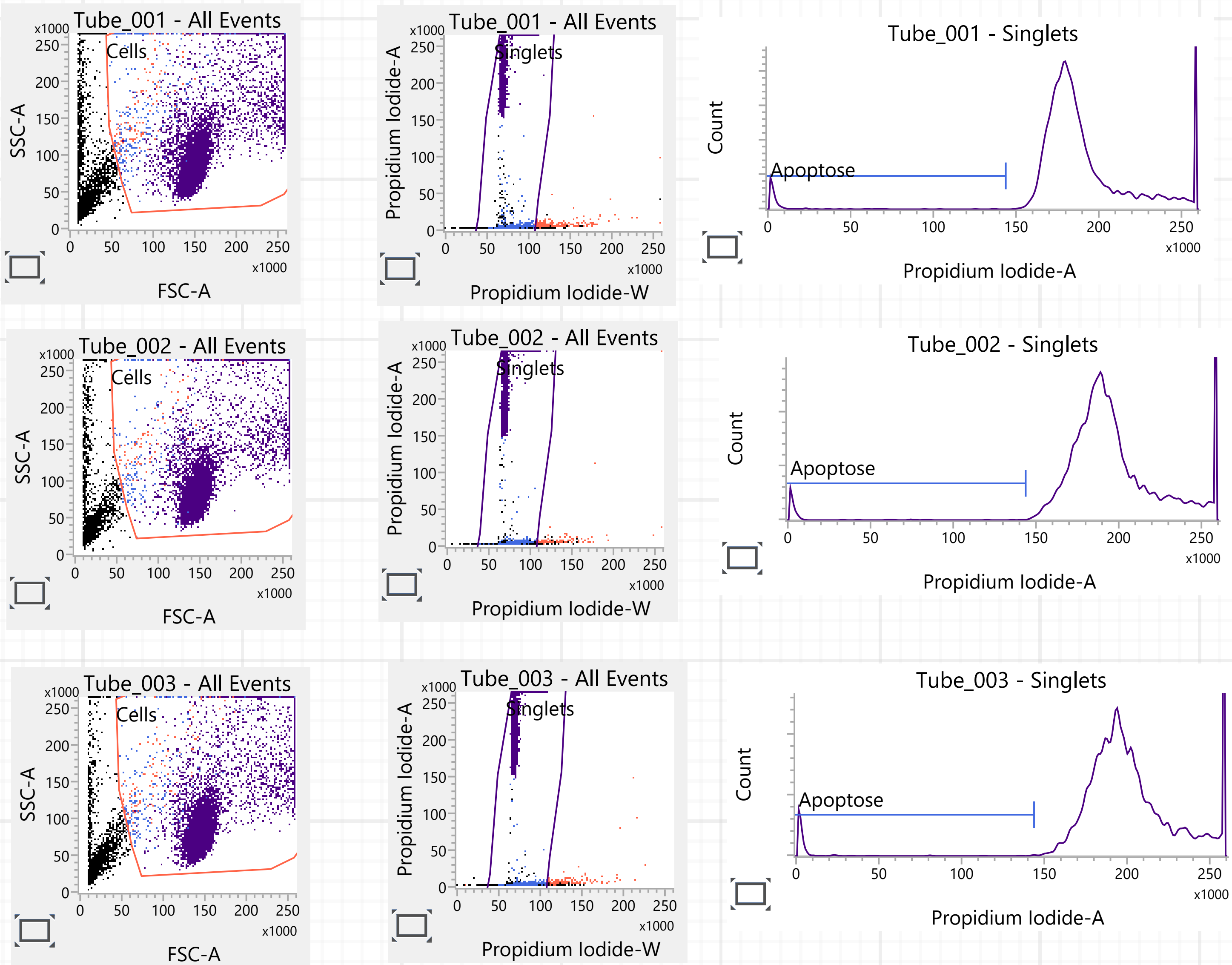

Statistics

Prozent apoptotische Zellen: Prozent apoptotische Zellen 2.8 % von Cells

Name	Events	% Total	% Parent	% Grandparent
Tube_001:All Events	12,434	100.00	***	***
Tube_001:Apoptose	277	2.23	2.82	2.77
Tube_001:Cells	10,000	80.42	80.42	***
Tube_001:Singlets	9,829	79.05	98.29	79.05
Tube_002:All Events	10,000	100.00	***	***
Tube_002:Cells	8,306	83.06	83.06	***
Tube_002:Singlets	8,207	82.07	98.81	82.07
Tube_002:Apoptose	171	1.71	2.08	2.06
Tube_003:All Events	10,000	100.00	***	***
Tube_003:Cells	8,249	82.49	82.49	***
Tube_003:Singlets	8,124	81.24	98.48	81.24
Tube_003:Apoptose	198	1.98	2.44	2.40

DNA Fragmentierung

Sample ID: N6 0,1

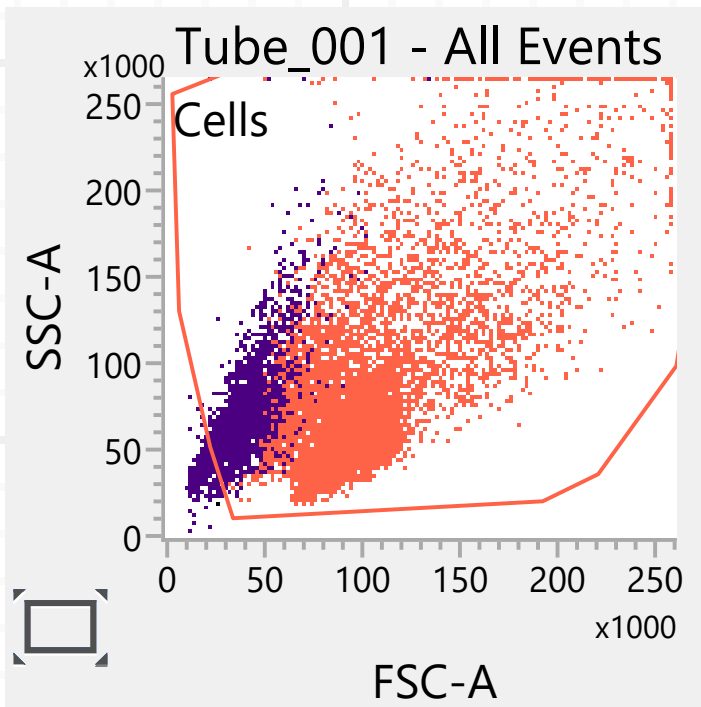

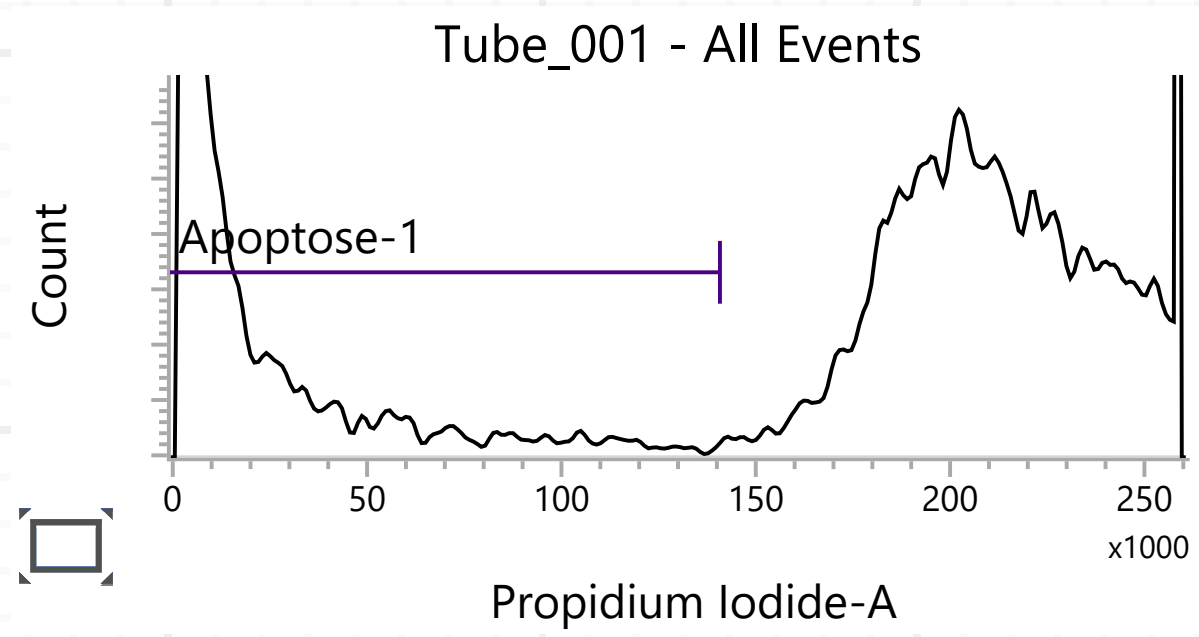

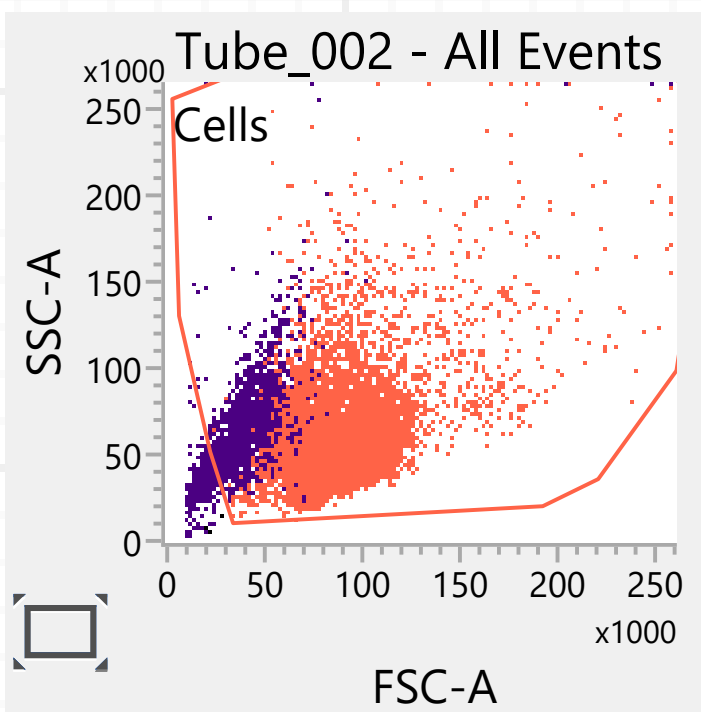

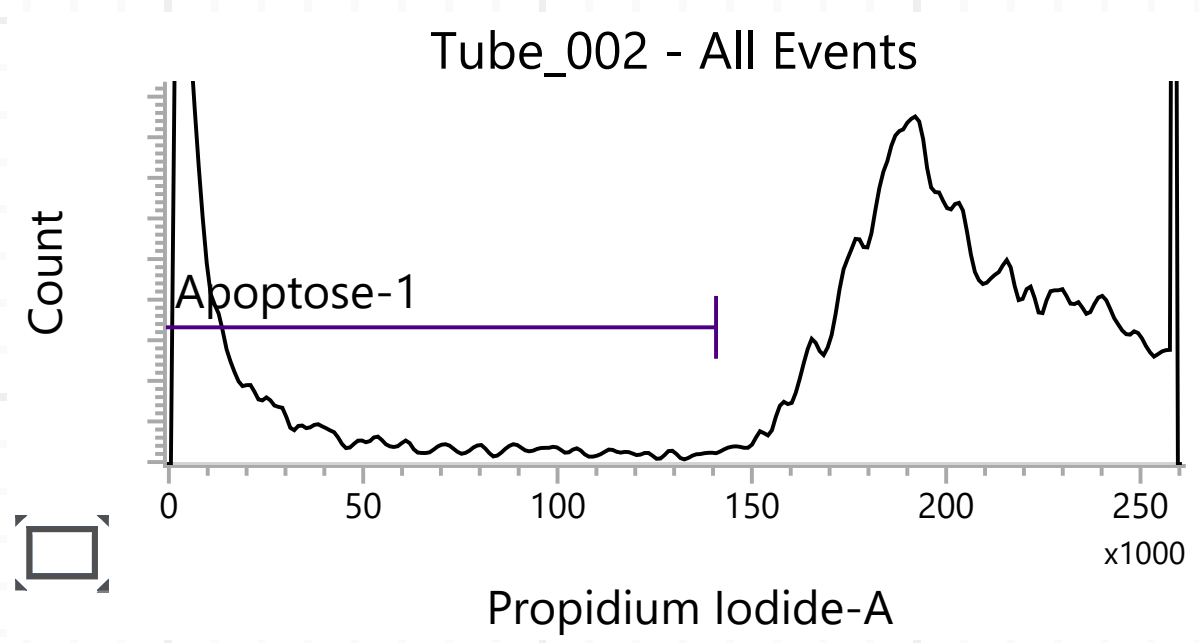

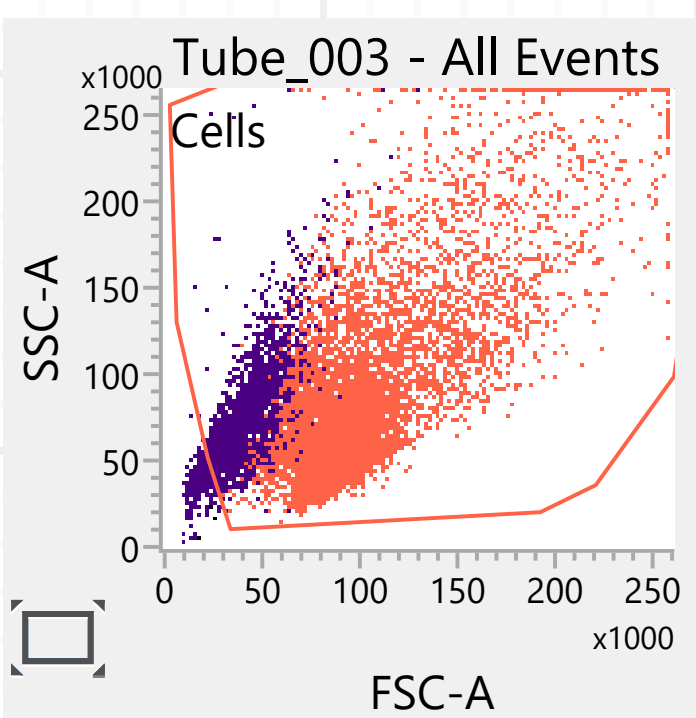

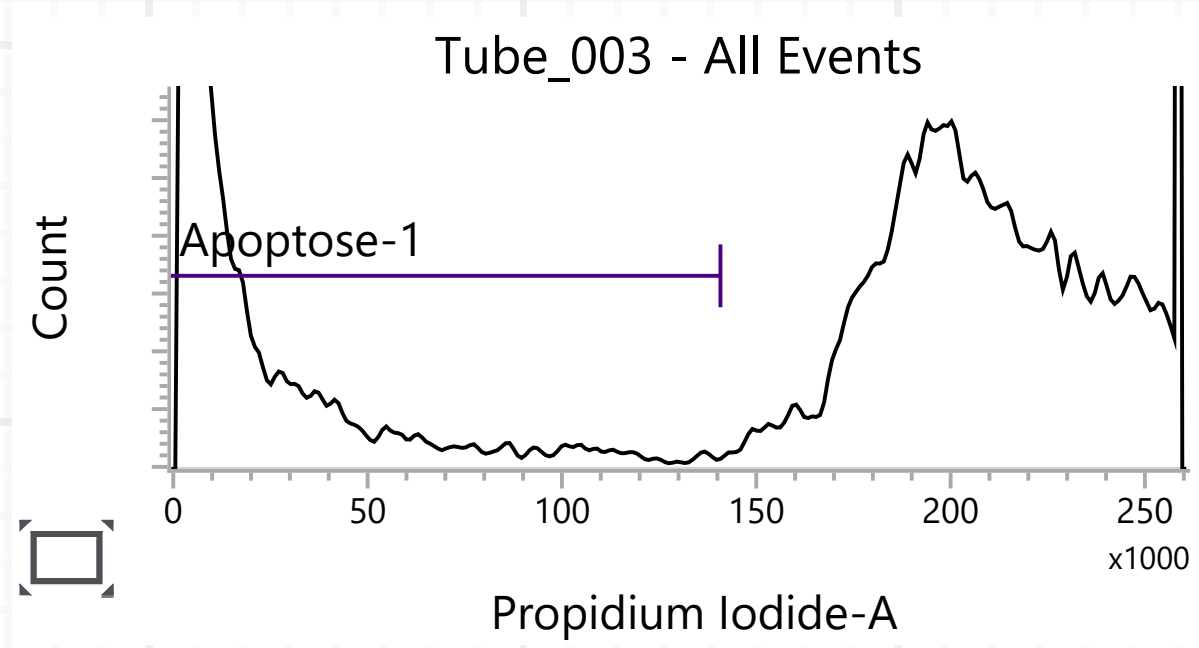

Statistics

Prozent apoptotische Zellen: Prozent apoptotische Zellen No Value % von Cells

Name	Events	% Total	% Parent	% Grandparent	
Tube_001:All Events	10,249	100.00	***	***	
Tube_001:Cells	10,000	97.57	97.57	***	
Tube_002:All Events	10,000	100.00	***	***	
Tube_002:Cells	9,641	96.41	96.41	***	
Tube_003:All Events	10,000	100.00	***	***	
Tube_003:Cells	9,750	97.50	97.50	***	
Tube_001:Apoptose-1	1,946	18.99	18.99	***	
Tube_002:Apoptose-1	1,849	18.49	18.49	***	
Tube_003:Apoptose-1	2,040	20.40	20.40	***	

DNA Fragmentierung

Sample ID: N6 0,5

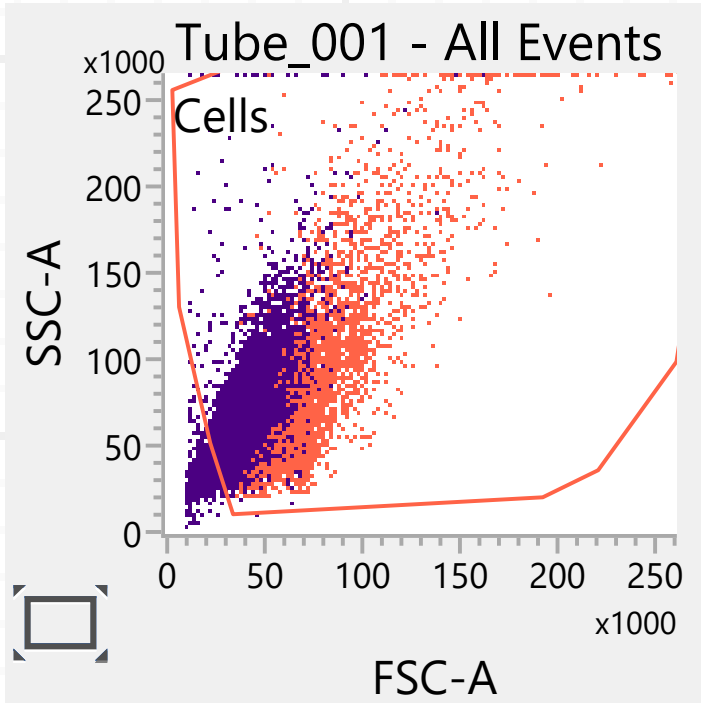

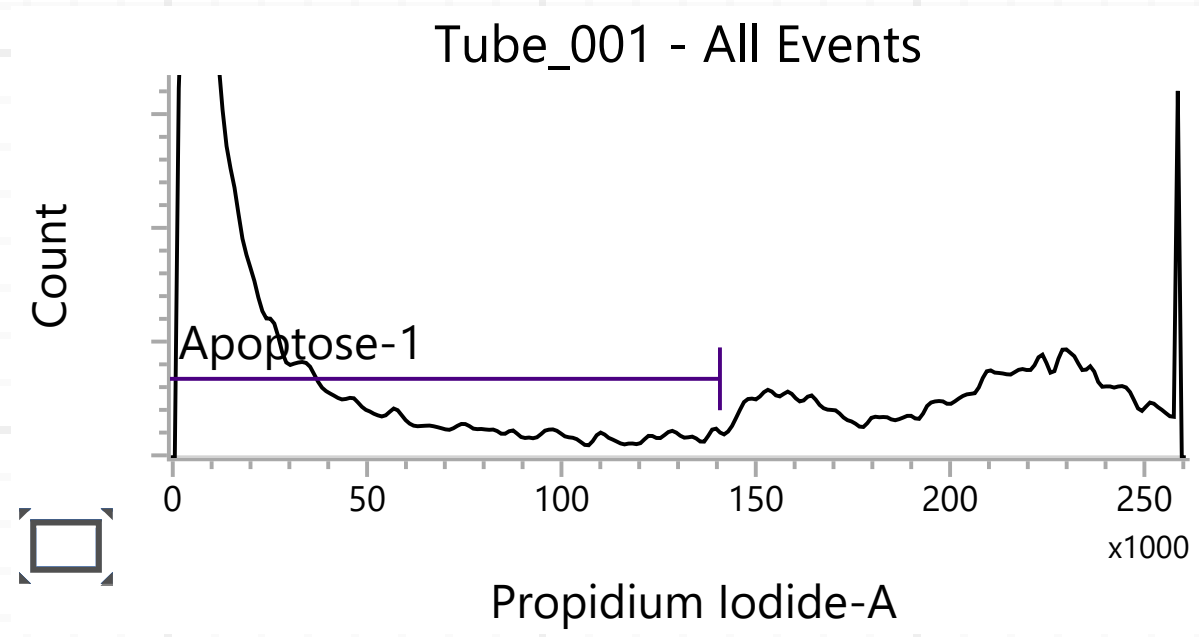

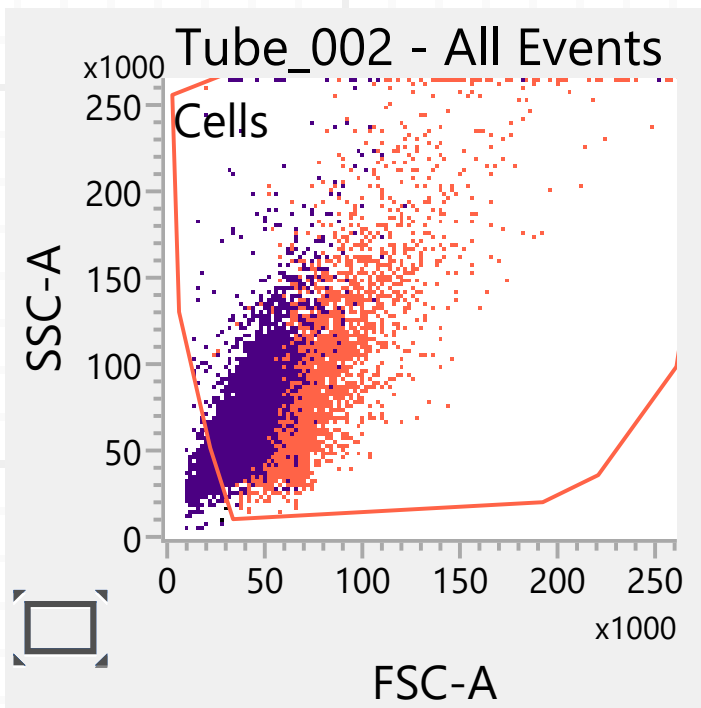

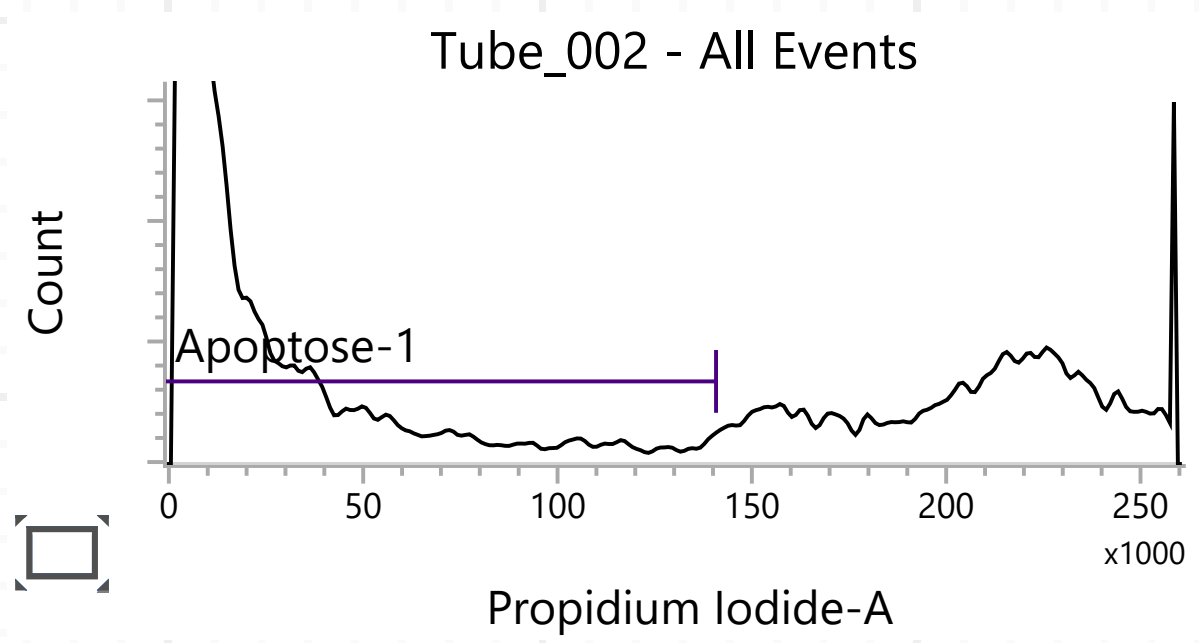

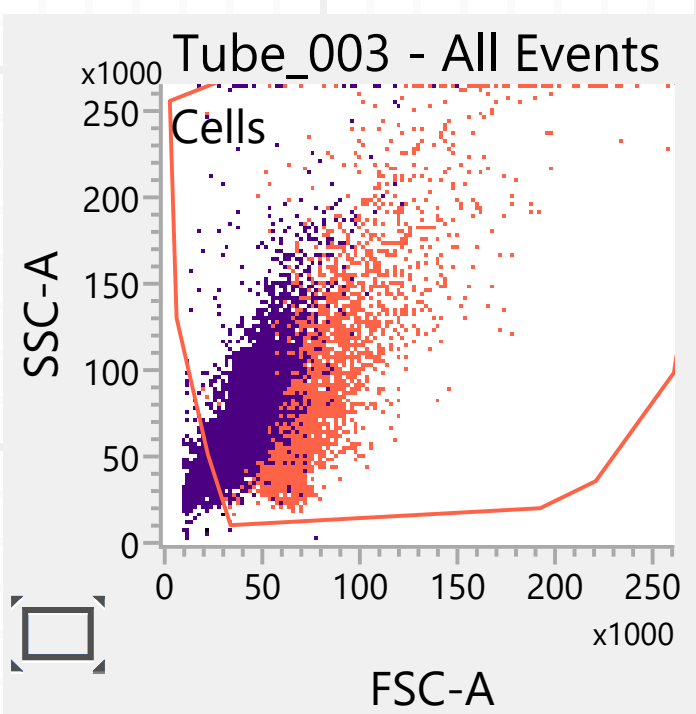

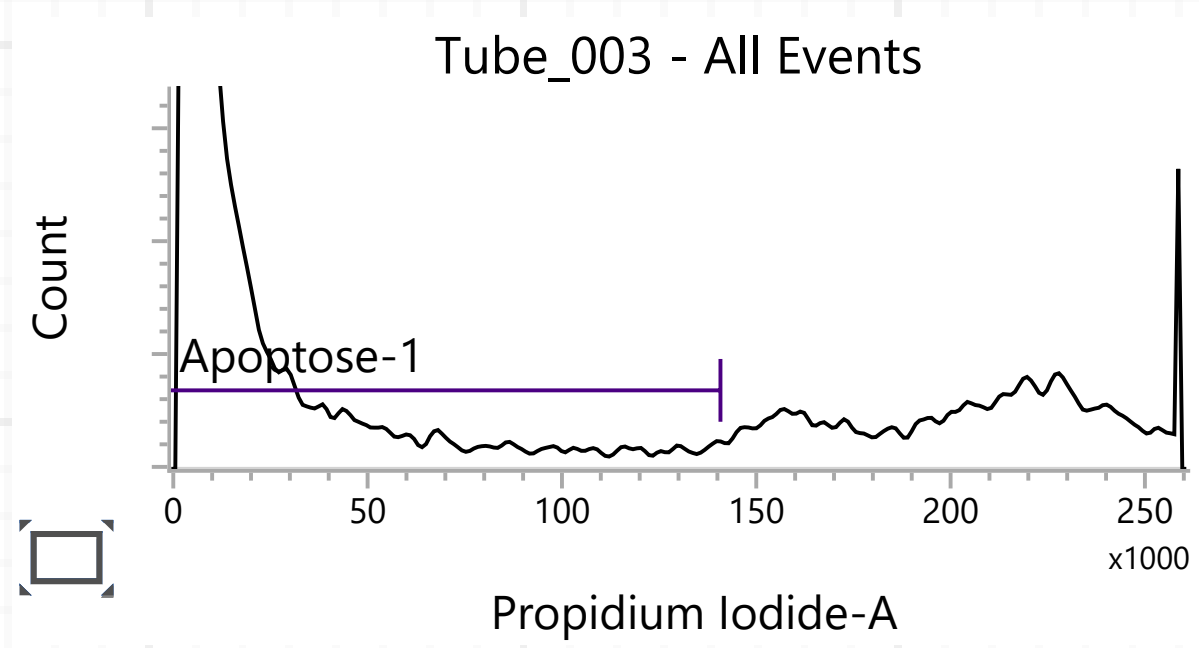

Statistics

Prozent apoptotische Zellen: Prozent apoptotische Zellen No Value % von Cells

Name	Events	% Total	% Parent	% Grandparent
Tube_001:All Events	10,619	100.00	***	***
Tube_001:Cells	10,000	94.17	94.17	***
Tube_002:All Events	10,000	100.00	***	***
Tube_002:Cells	9,433	94.33	94.33	***
Tube_003:All Events	10,000	100.00	***	***
Tube_003:Cells	9,303	93.03	93.03	***
Tube_001:Apoptose-1	5,747	54.12	54.12	***
Tube_002:Apoptose-1	5,298	52.98	52.98	***
Tube_003:Apoptose-1	5,810	58.10	58.10	***

DNA Fragmentierung

Sample ID: N6 0,25

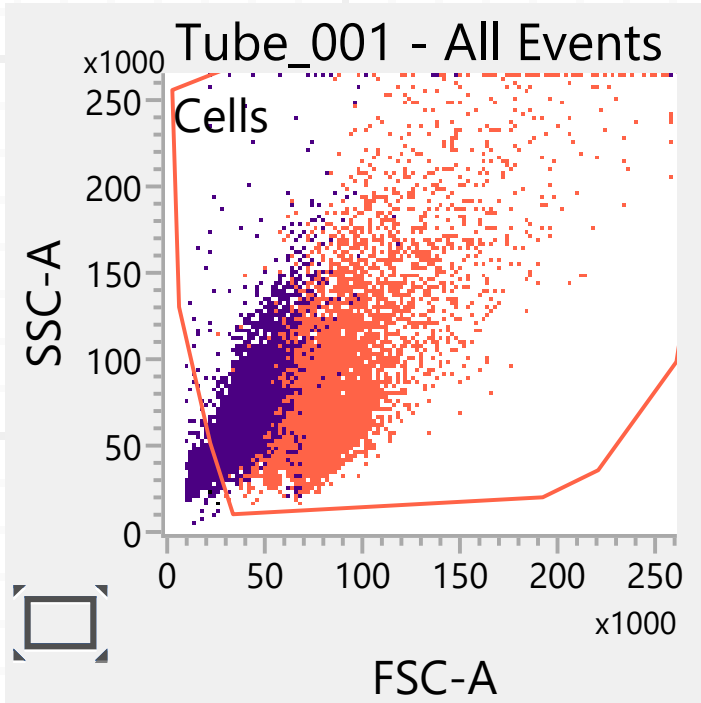

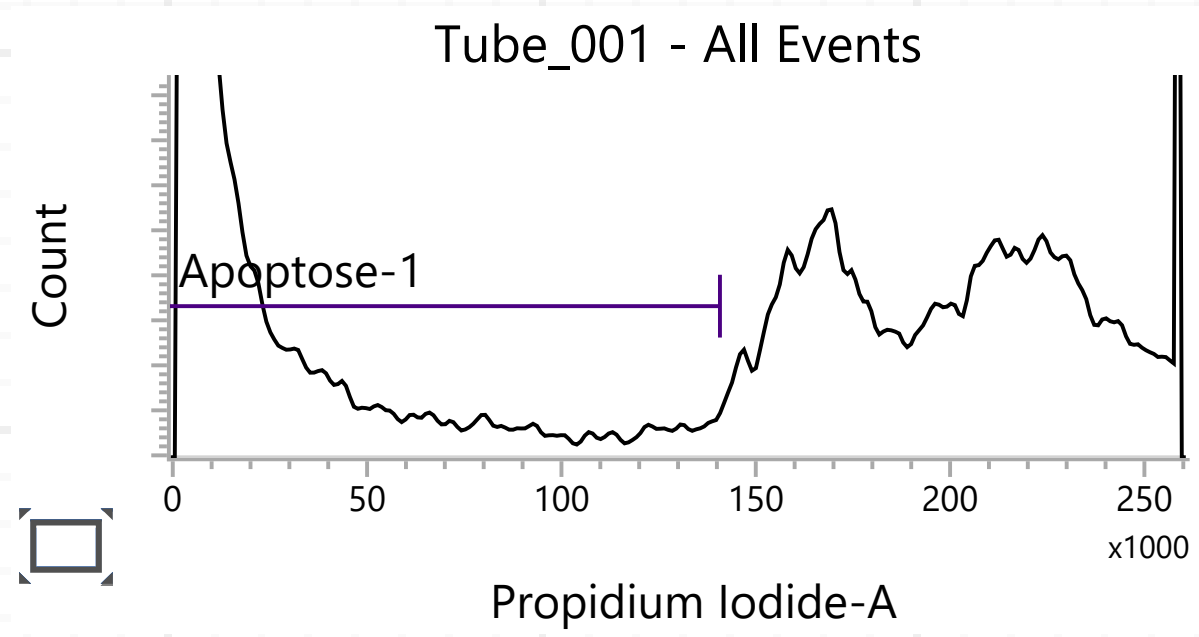

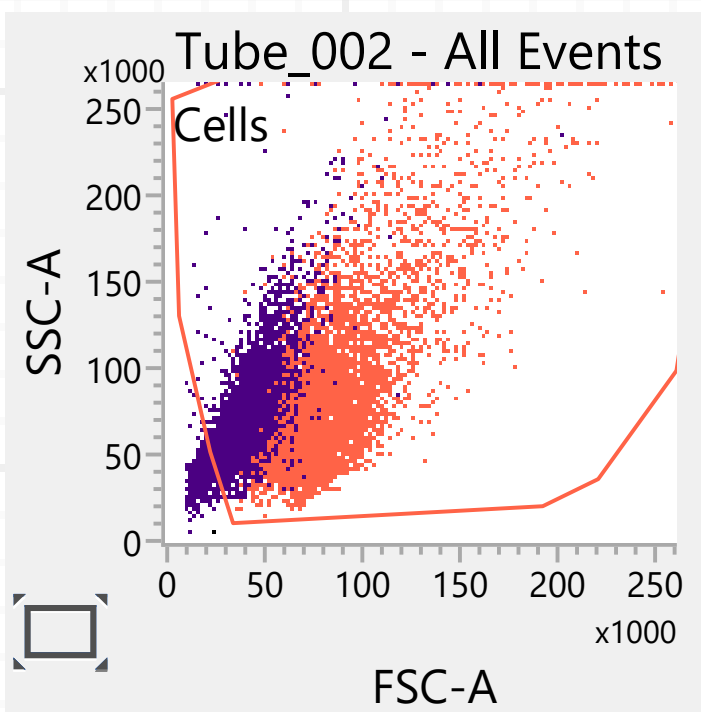

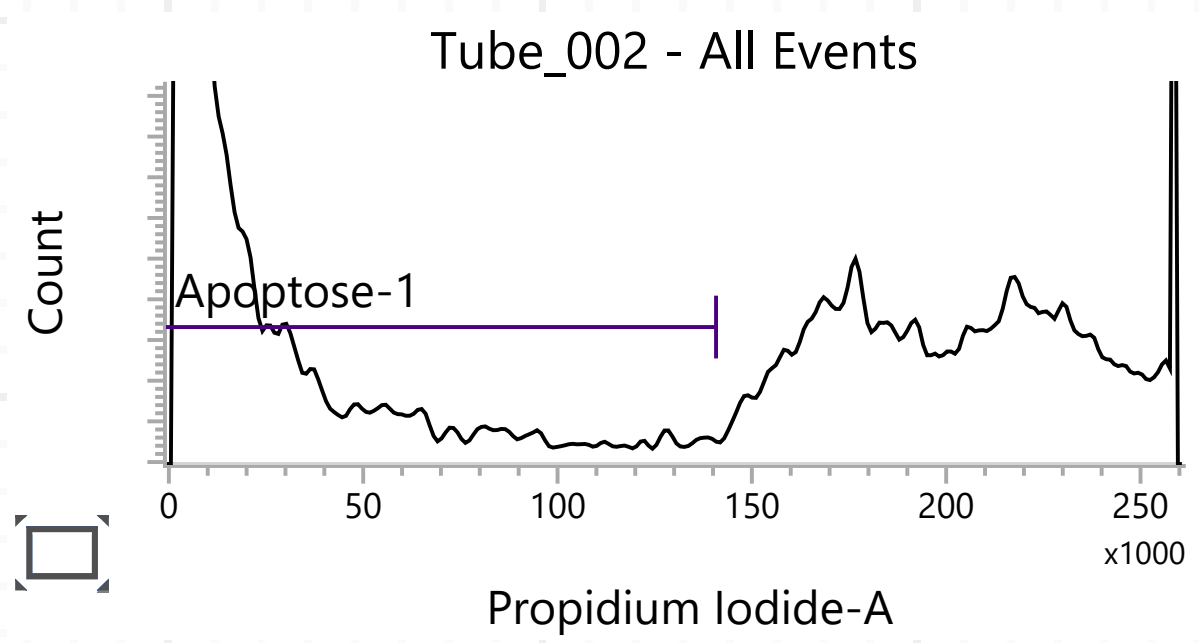

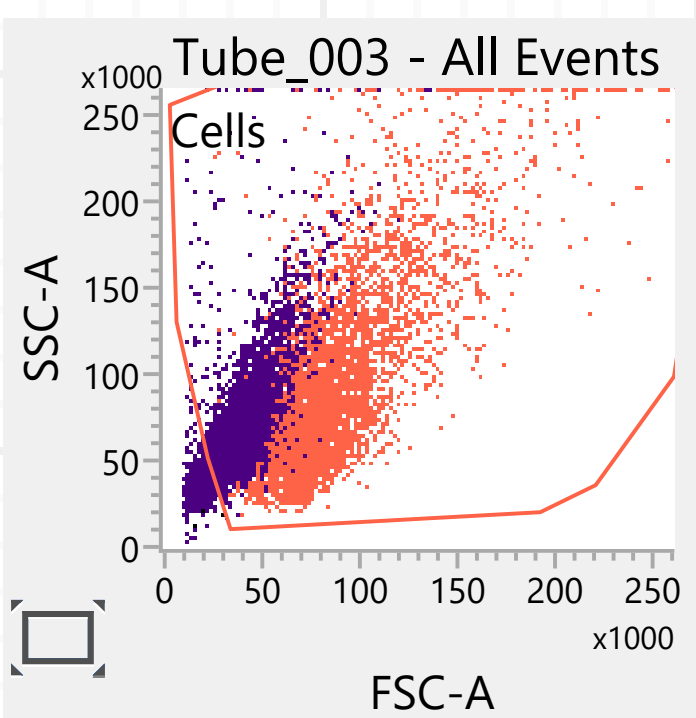

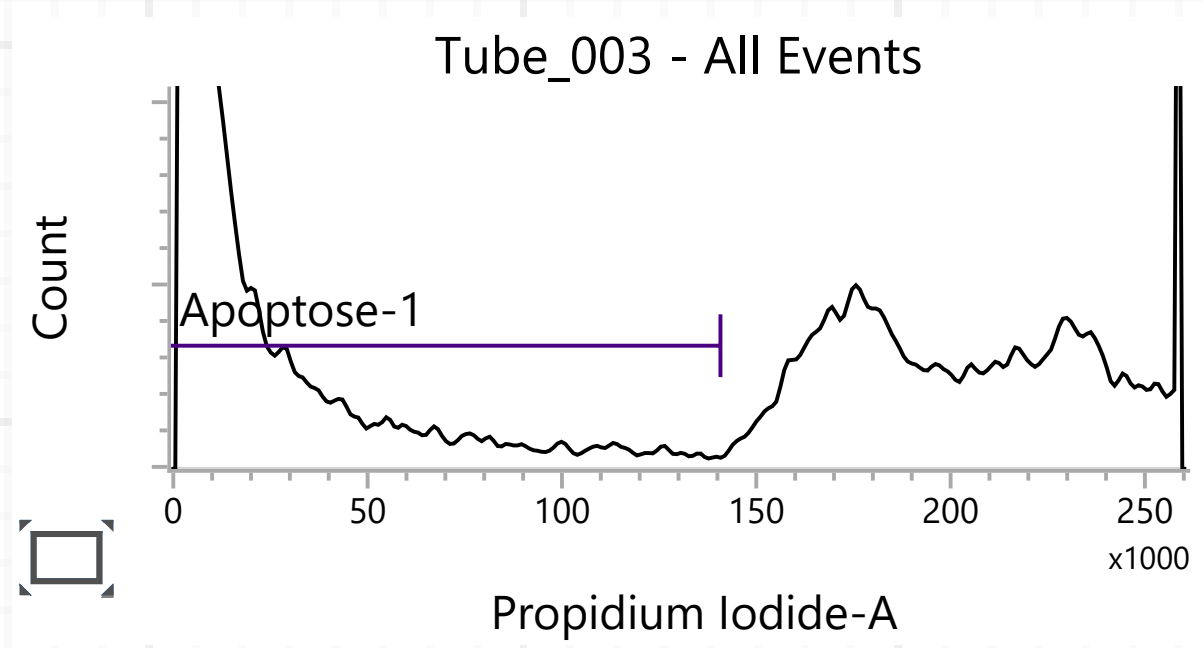

Statistics

Prozent apoptotische Zellen: Prozent apoptotische Zellen No Value % von Cells

Name	Events	% Total	% Parent	% Grandparent
Tube_001:All Events	10,474	100.00	***	***
Tube_001:Cells	10,000	95.47	95.47	***
Tube_002:All Events	10,000	100.00	***	***
Tube_002:Cells	9,490	94.90	94.90	***
Tube_003:All Events	10,000	100.00	***	***
Tube_003:Cells	9,450	94.50	94.50	***
Tube_001:Apoptose-1	3,556	33.95	33.95	***
Tube_002:Apoptose-1	3,993	39.93	39.93	***
Tube_003:Apoptose-1	4,073	40.73	40.73	***

DNA Fragmentierung

Sample ID: N6 1

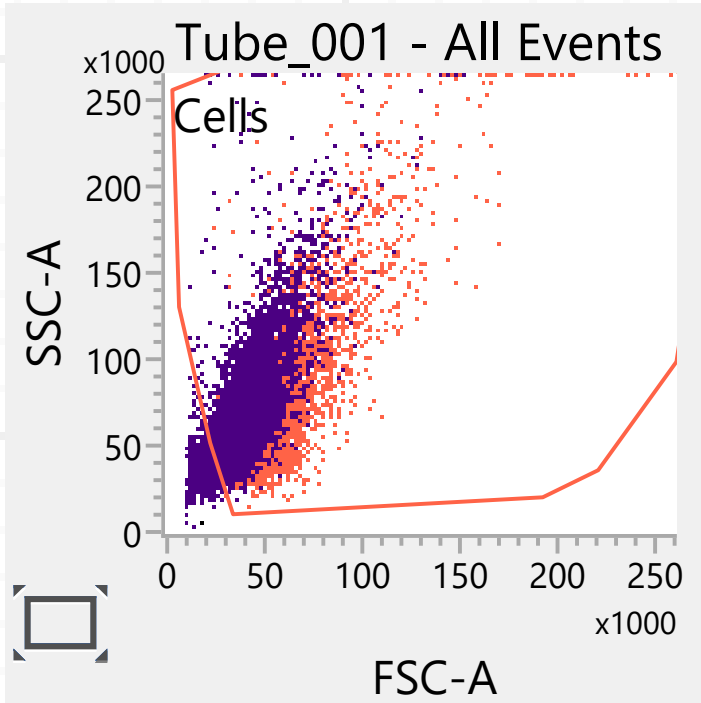

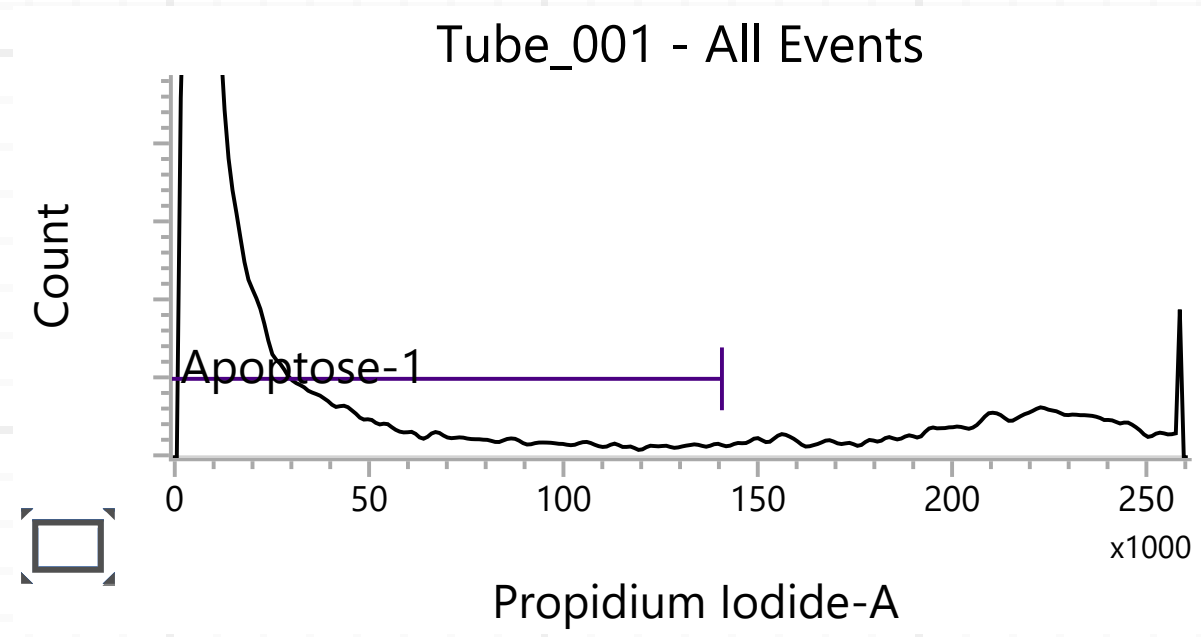

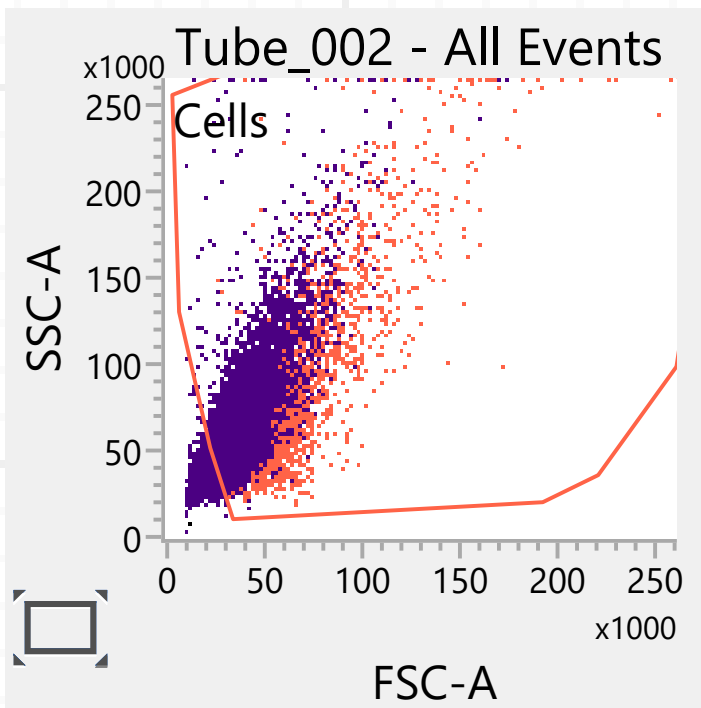

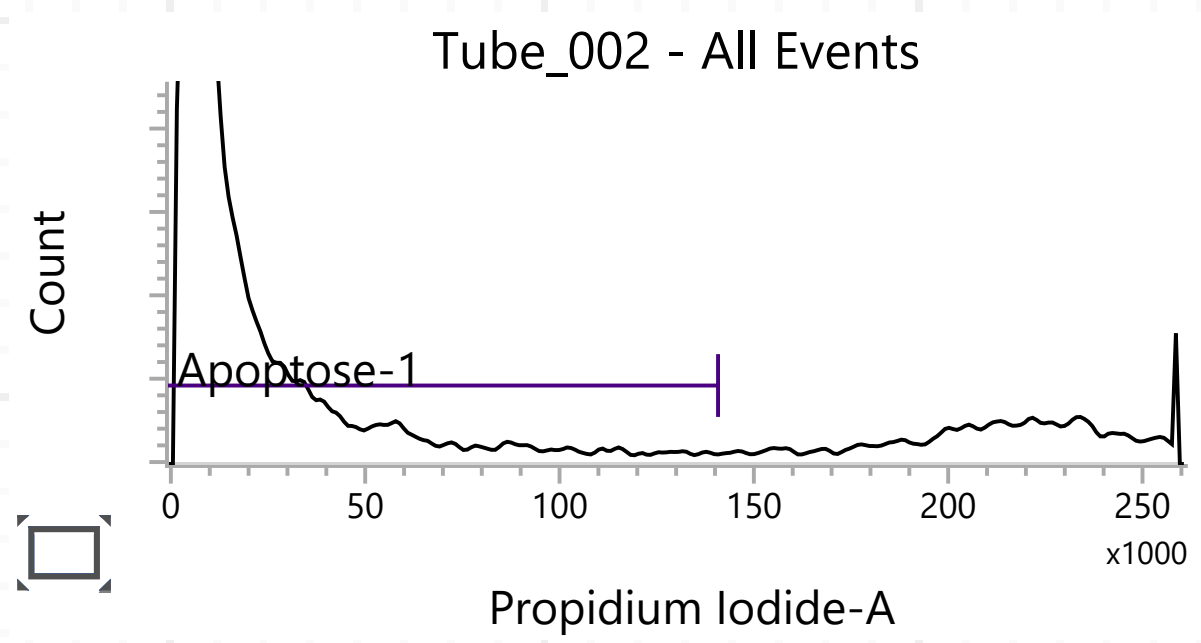

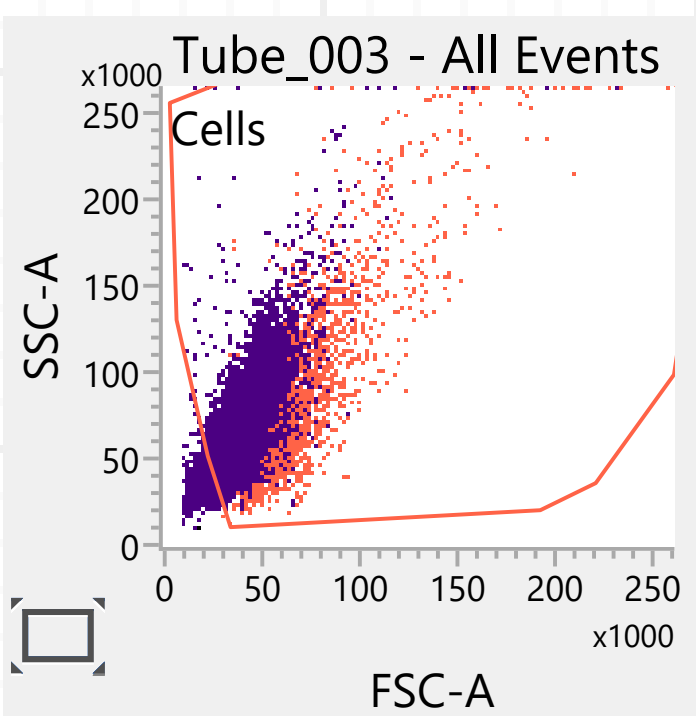

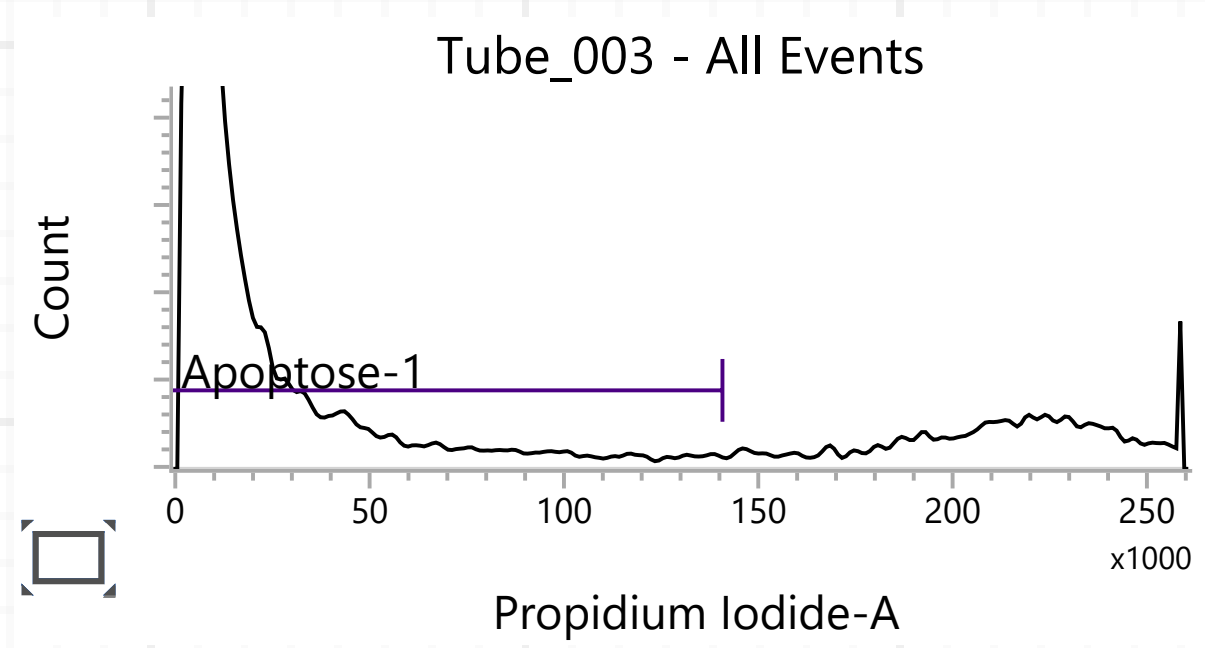

Statistics

Prozent apoptotische Zellen: Prozent apoptotische Zellen No Value % von Cells

Name	Events	% Total	% Parent	% Grandparent
Tube_001:All Events	10,927	100.00	***	***
Tube_001:Cells	10,000	91.52	91.52	***
Tube_002:All Events	10,000	100.00	***	***
Tube_002:Cells	9,132	91.32	91.32	***
Tube_003:All Events	10,000	100.00	***	***
Tube_003:Cells	9,147	91.47	91.47	***
Tube_001:Apoptose-1	7,922	72.50	72.50	***
Tube_002:Apoptose-1	7,371	73.71	73.71	***
Tube_003:Apoptose-1	7,120	71.20	71.20	***

DNA Fragmentierung

Sample ID: N6 2,5

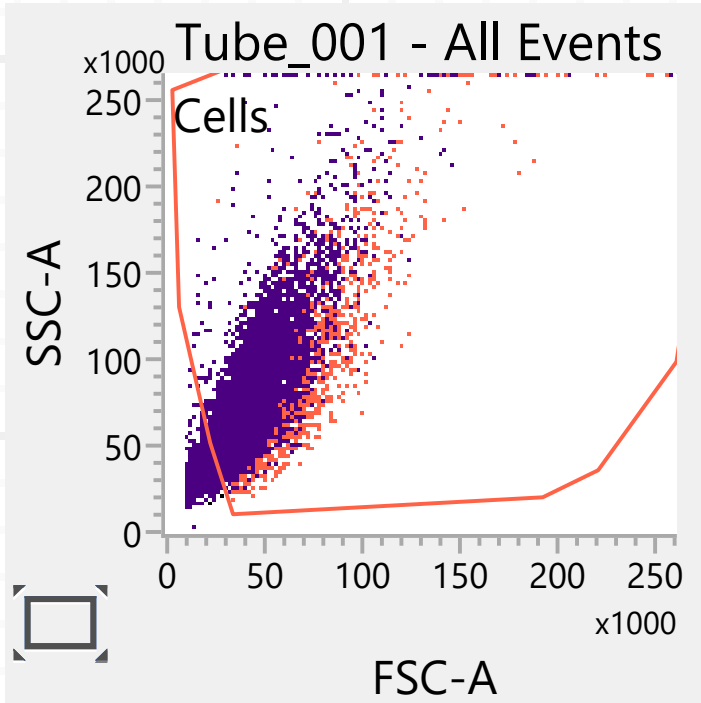

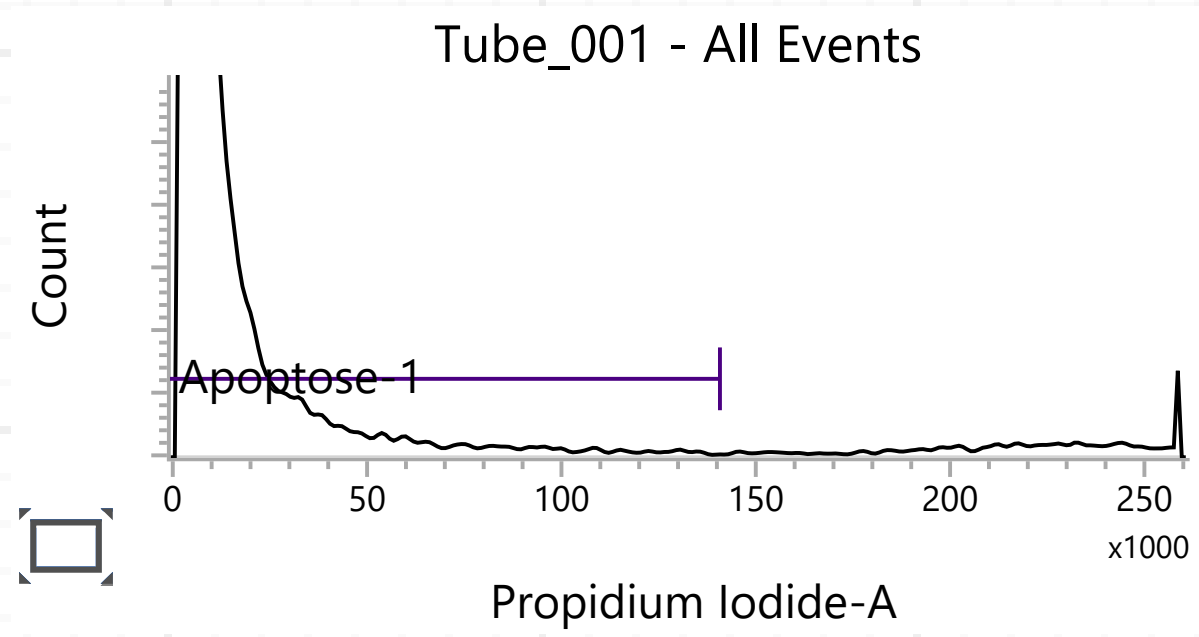

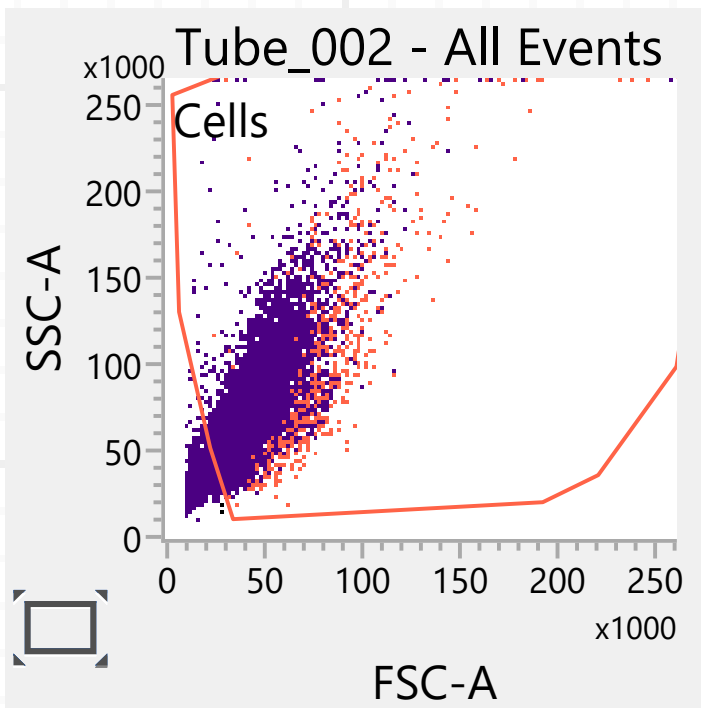

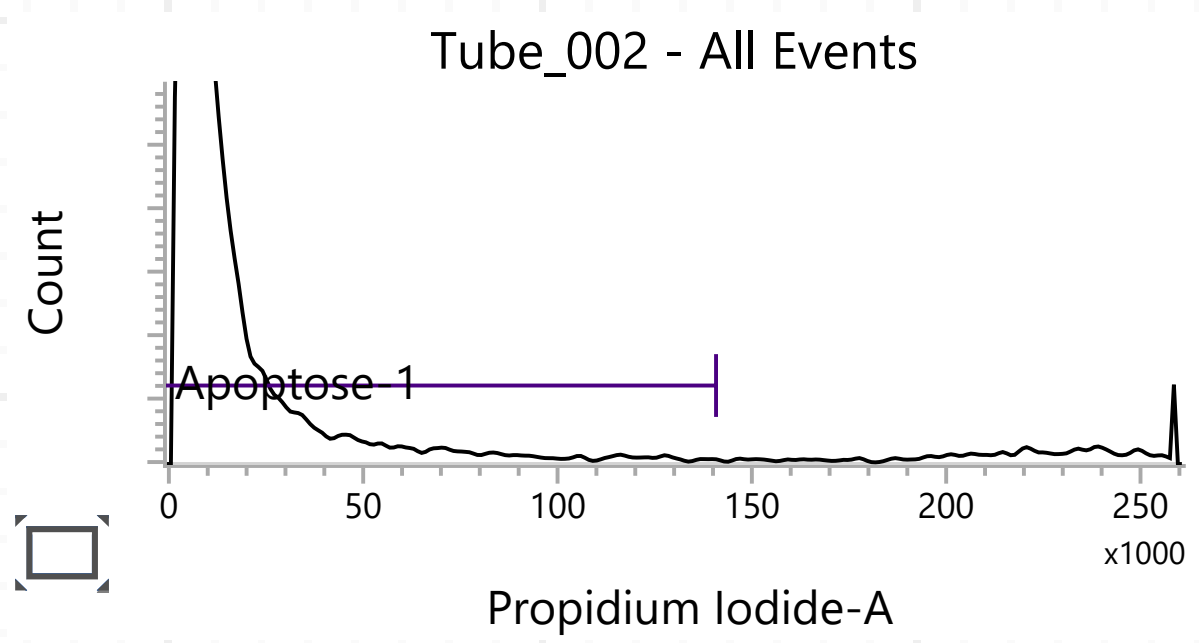

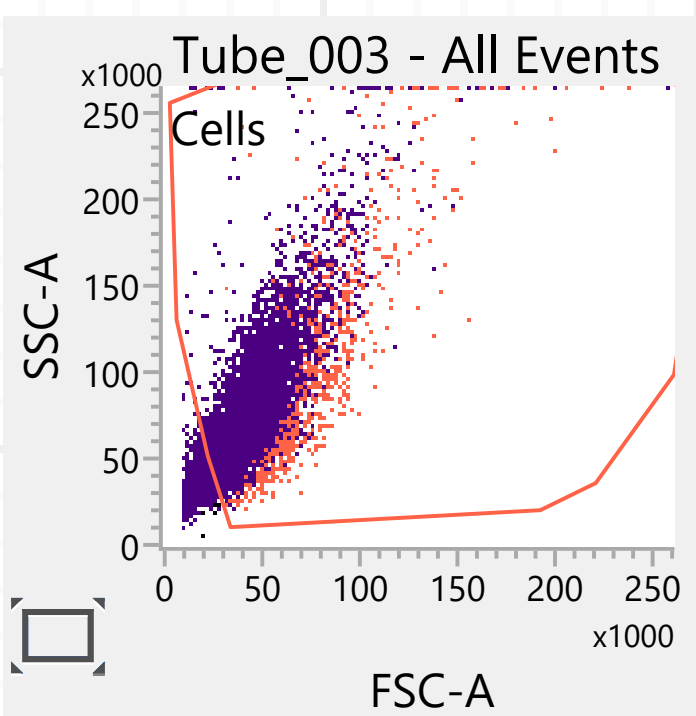

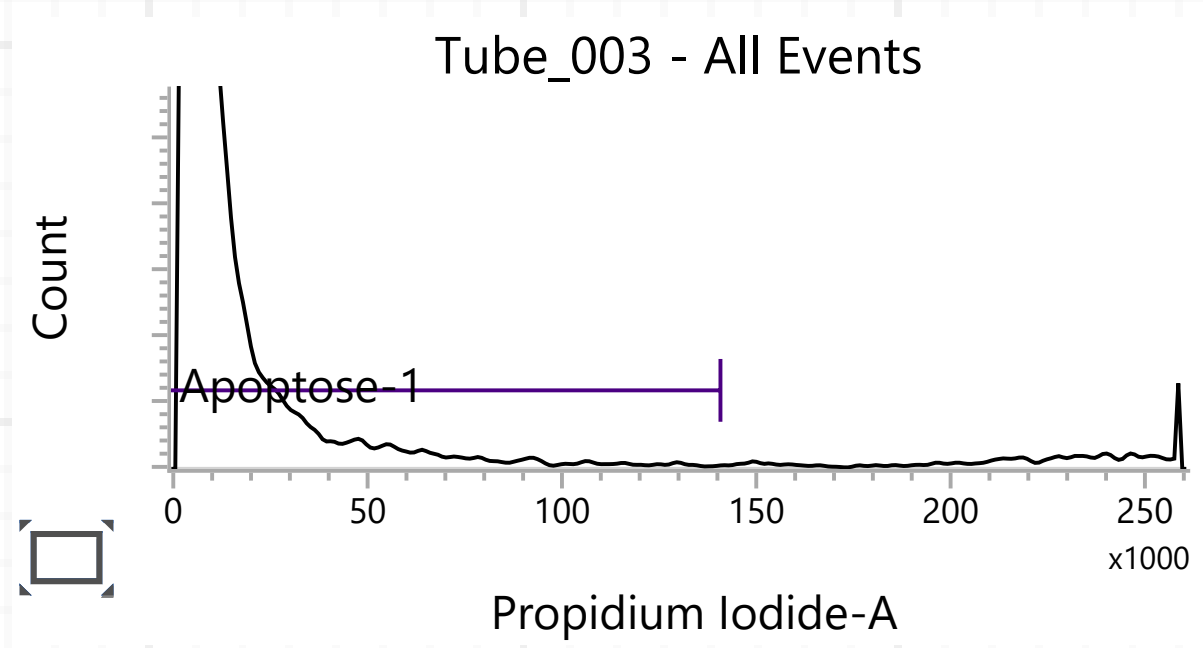

Statistics

Prozent apoptotische Zellen: Prozent apoptotische Zellen No Value % von Cells

Name	Events	% Total	% Parent	% Grandparent
Tube_001:All Events	11,460	100.00	***	***
Tube_001:Cells	10,000	87.26	87.26	***
Tube_002:All Events	10,000	100.00	***	***
Tube_002:Cells	8,812	88.12	88.12	***
Tube_003:All Events	10,000	100.00	***	***
Tube_003:Cells	8,724	87.24	87.24	***
Tube_001:Apoptose-1	9,945	86.78	86.78	***
Tube_002:Apoptose-1	8,584	85.84	85.84	***
Tube_003:Apoptose-1	8,687	86.87	86.87	***

DNA Fragmentierung

Sample ID: N6 5

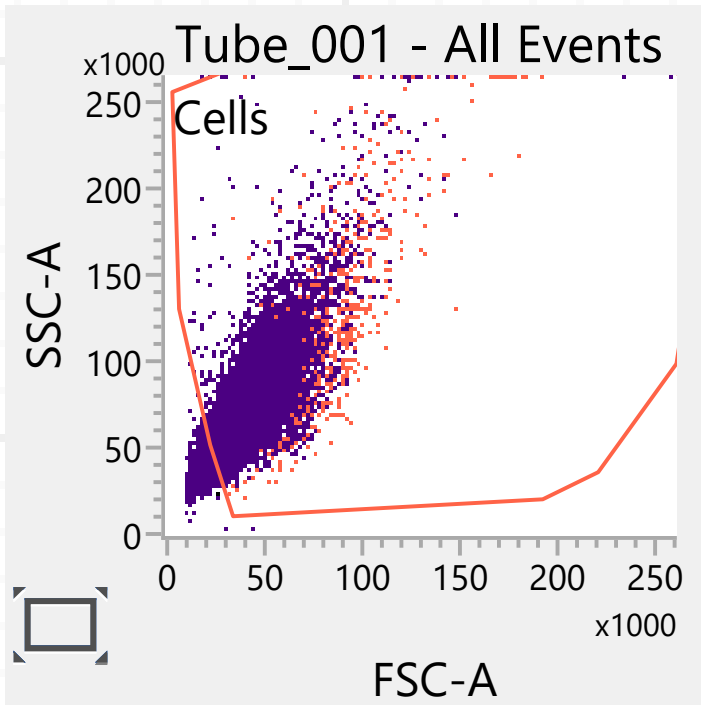

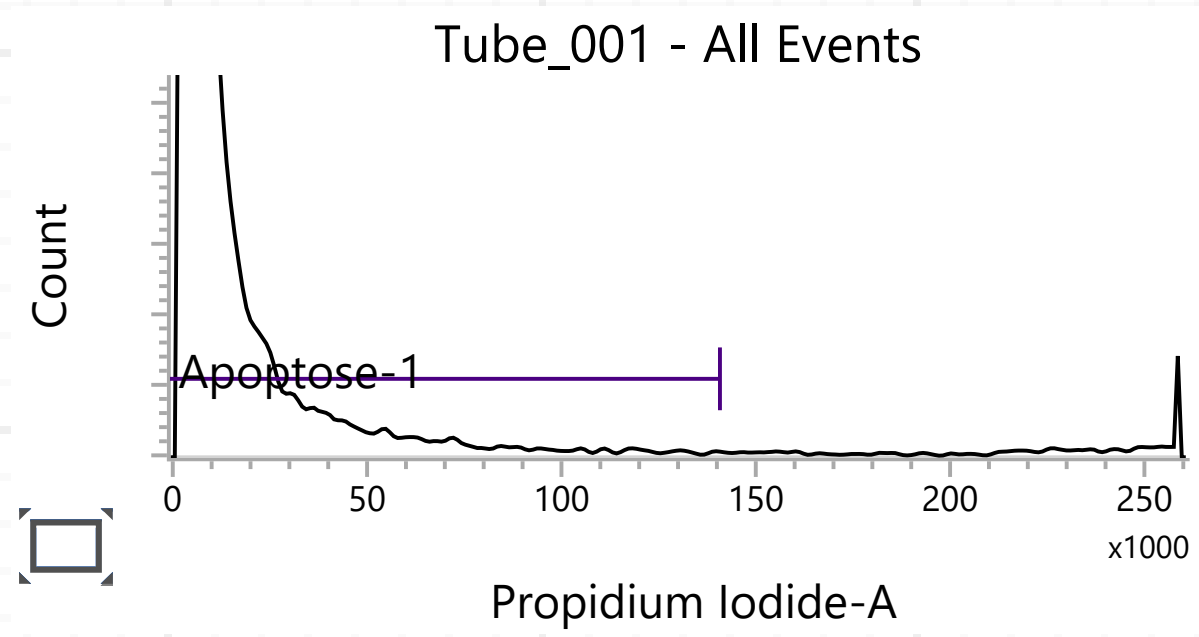

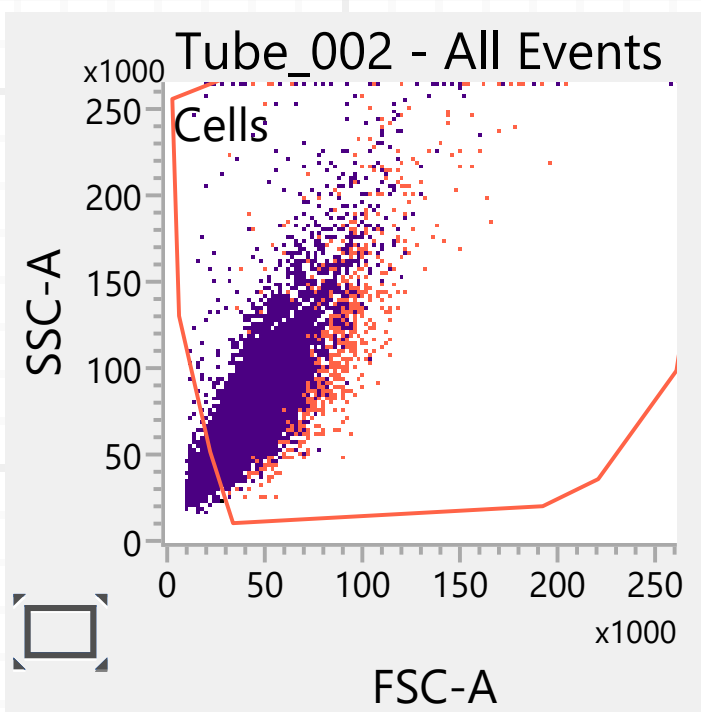

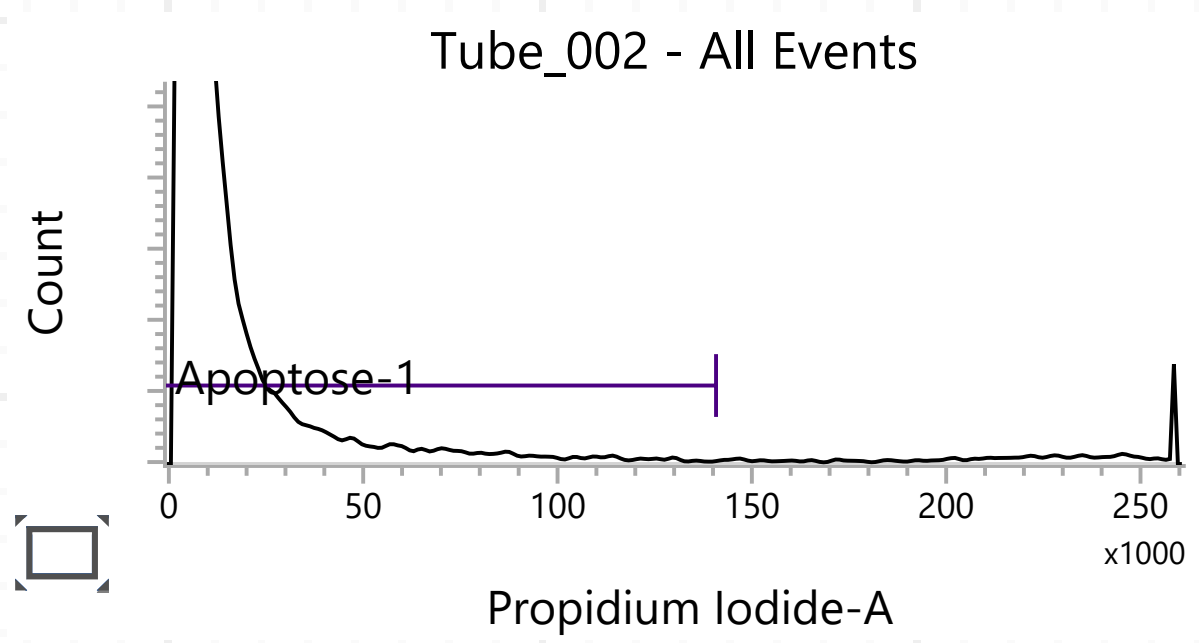

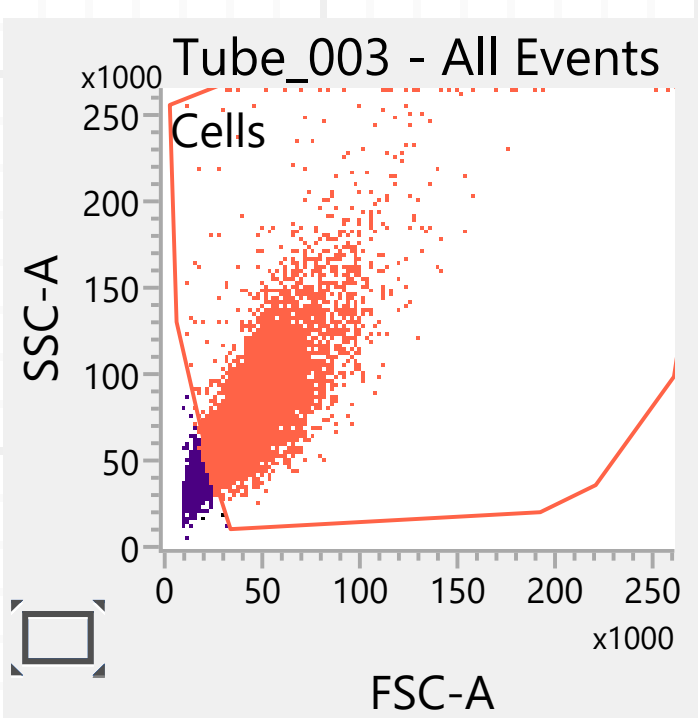

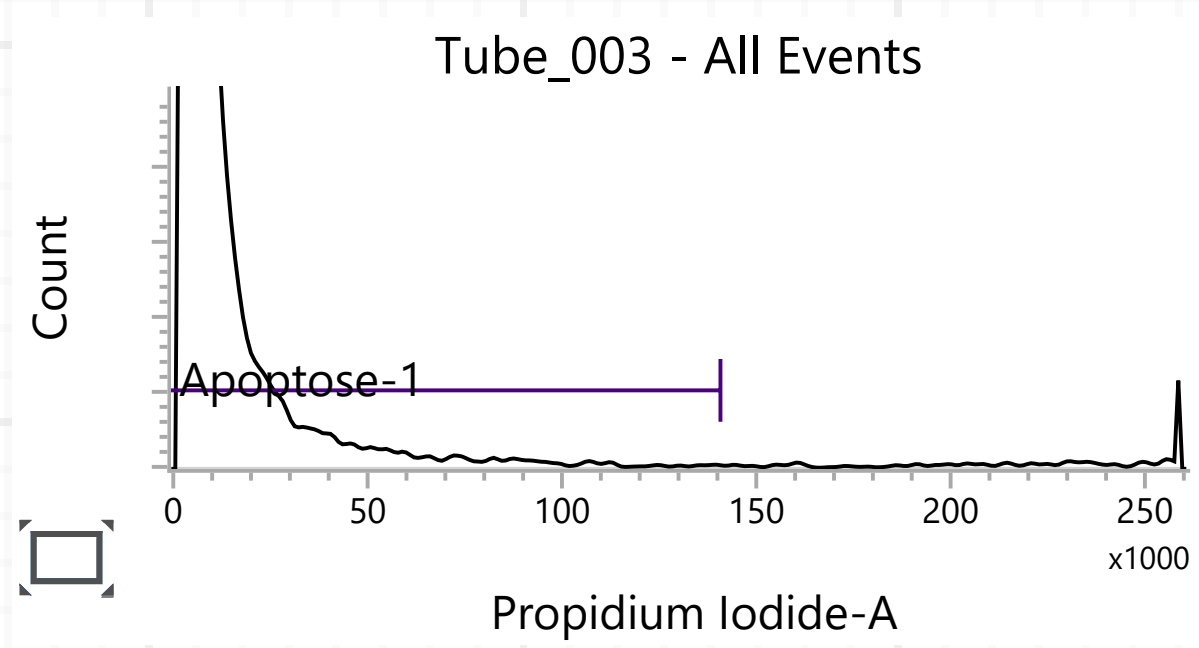

Statistics

Prozent apoptotische Zellen: Prozent apoptotische Zellen No Value % von Cells

Name	Events	% Total	% Parent	% Grandparent
Tube_001:All Events	11,179	100.00	***	***
Tube_001:Cells	10,000	89.45	89.45	***
Tube_002:All Events	10,000	100.00	***	***
Tube_002:Cells	9,154	91.54	91.54	***
Tube_003:All Events	10,000	100.00	***	***
Tube_003:Cells	8,725	87.25	87.25	***
Tube_001:Apoptose-1	9,960	89.10	89.10	***
Tube_002:Apoptose-1	8,805	88.05	88.05	***
Tube_003:Apoptose-1	9,031	90.31	90.31	***

DNA Fragmentierung

Sample ID: N6 D

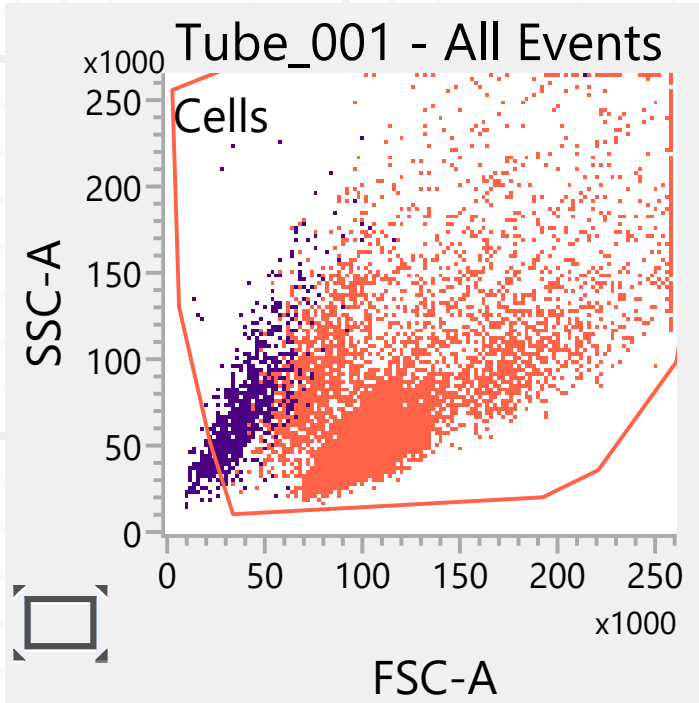

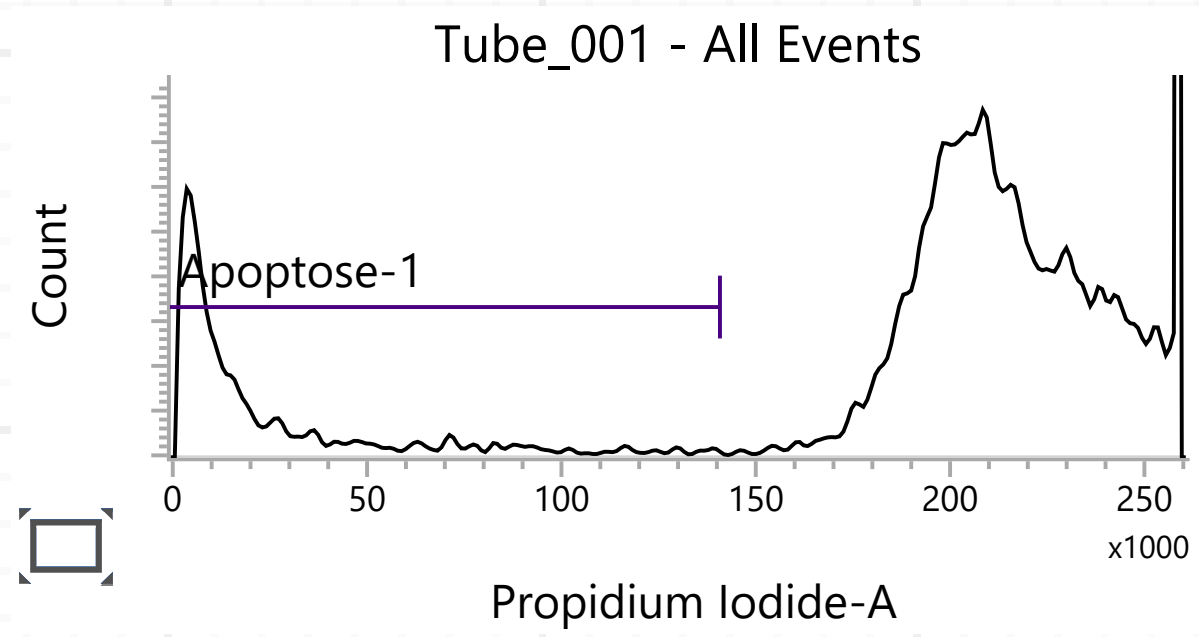

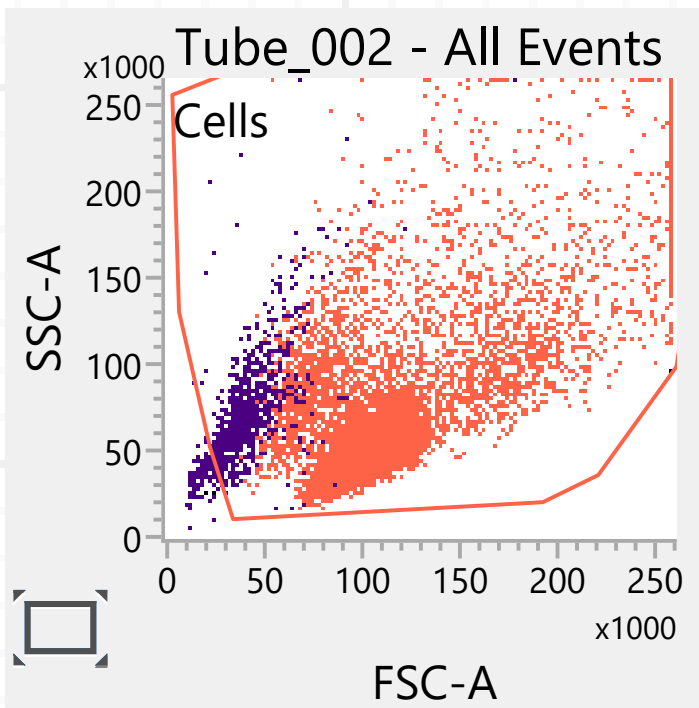

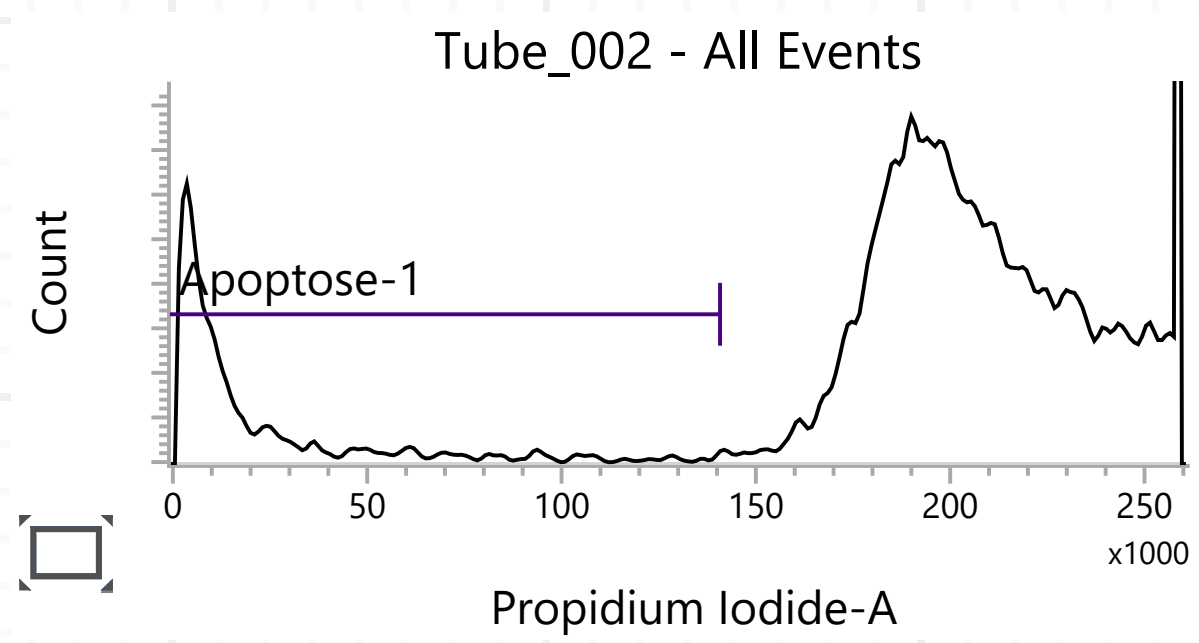

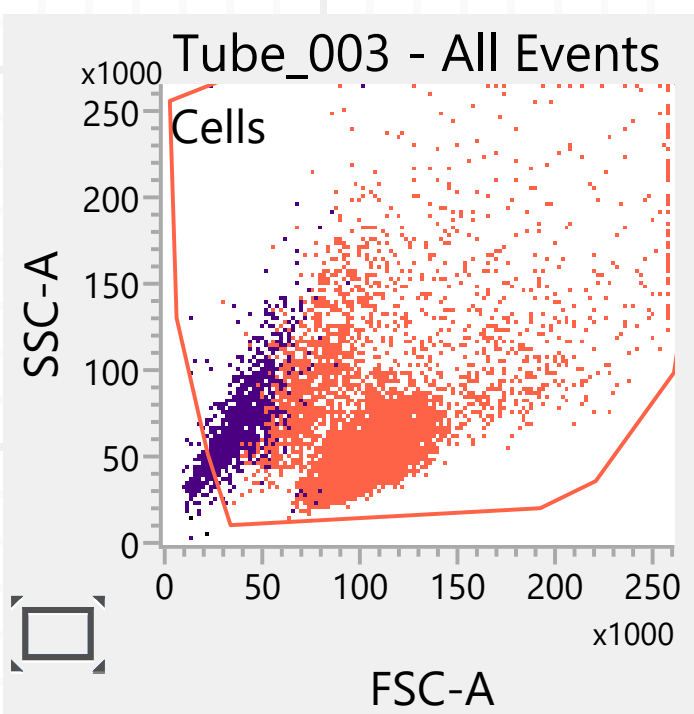

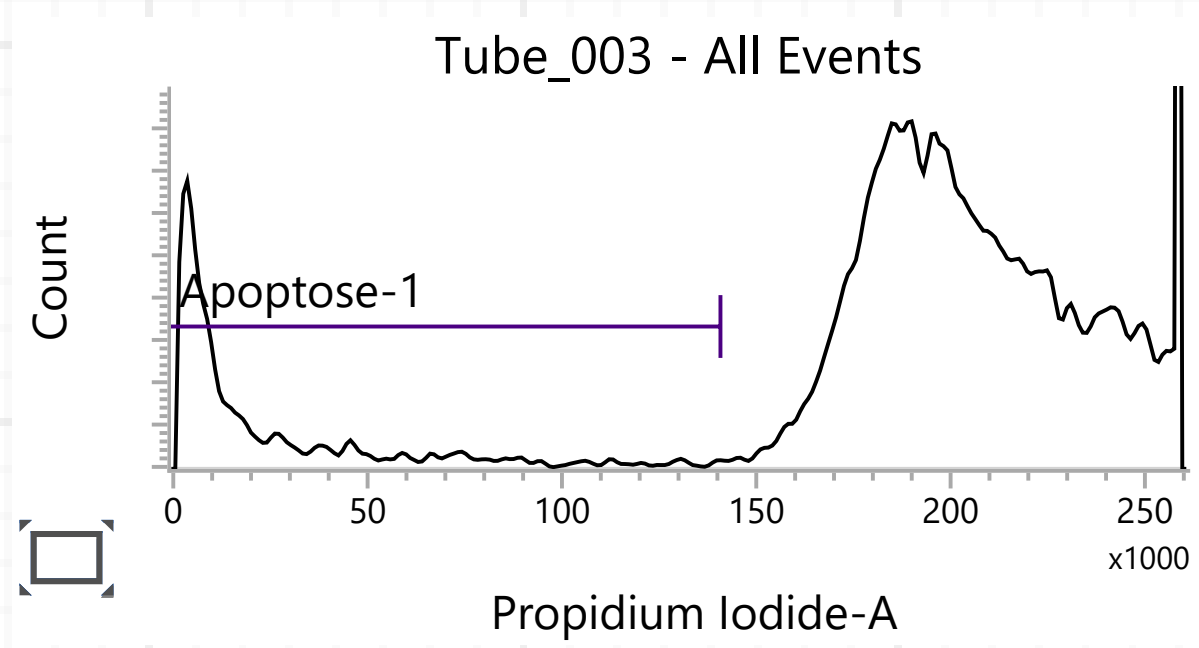

Statistics

Prozent apoptotische Zellen: Prozent apoptotische Zellen No Value % von Cells

Name	Events	% Total	% Parent	% Grandparent
Tube_001:All Events	10,120	100.00	***	***
Tube_001:Cells	10,000	98.81	98.81	***
Tube_002:All Events	10,000	100.00	***	***
Tube_002:Cells	9,896	98.96	98.96	***
Tube_003:All Events	10,000	100.00	***	***
Tube_003:Cells	9,876	98.76	98.76	***
Tube_001:Apoptose-1	935	9.24	9.24	***
Tube_002:Apoptose-1	886	8.86	8.86	***
Tube_003:Apoptose-1	961	9.61	9.61	***

DNA Fragmentierung

Sample ID: N6 K

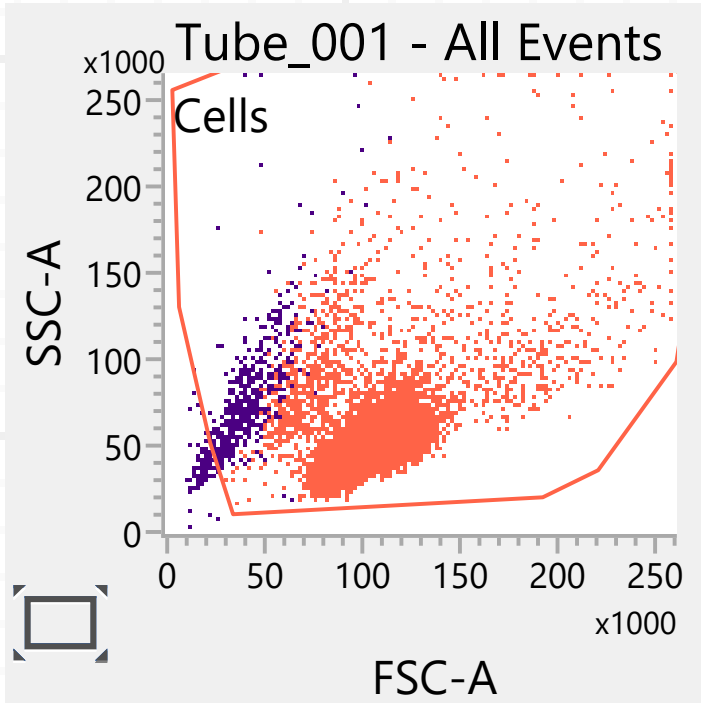

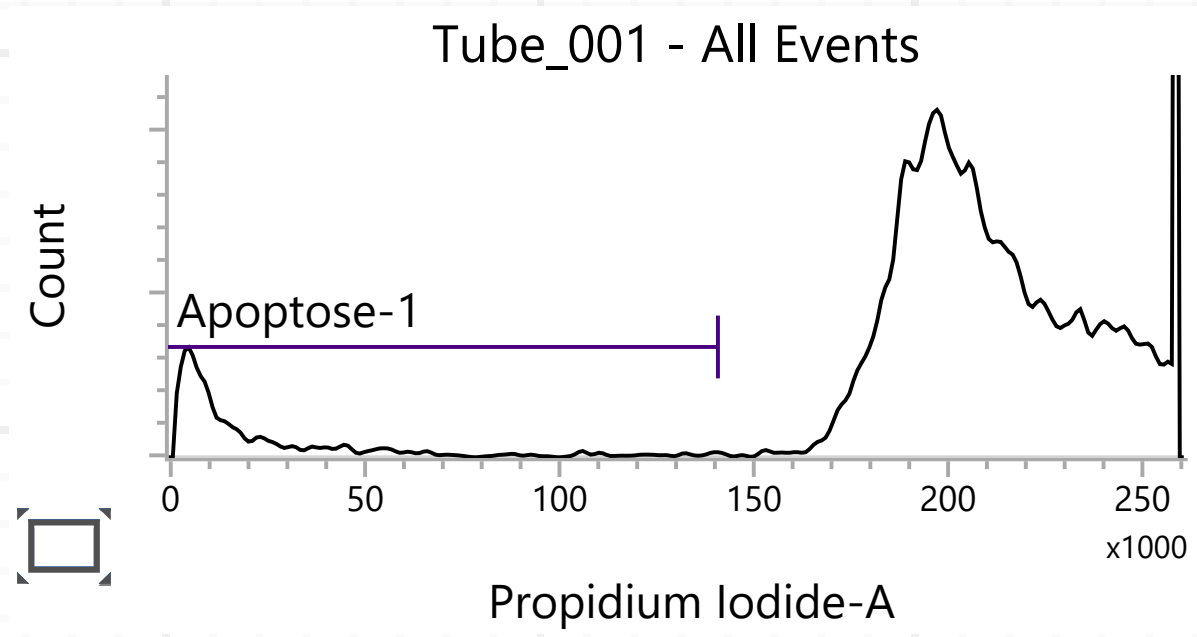

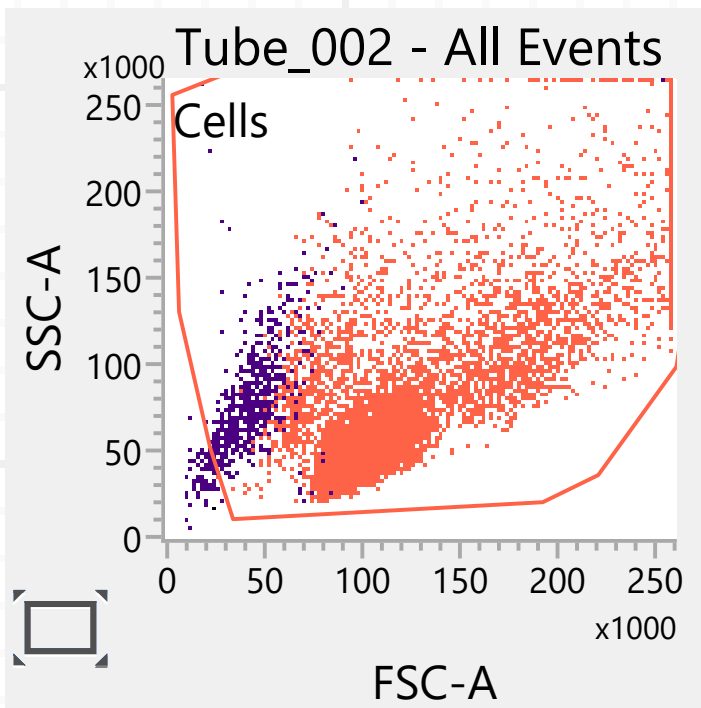

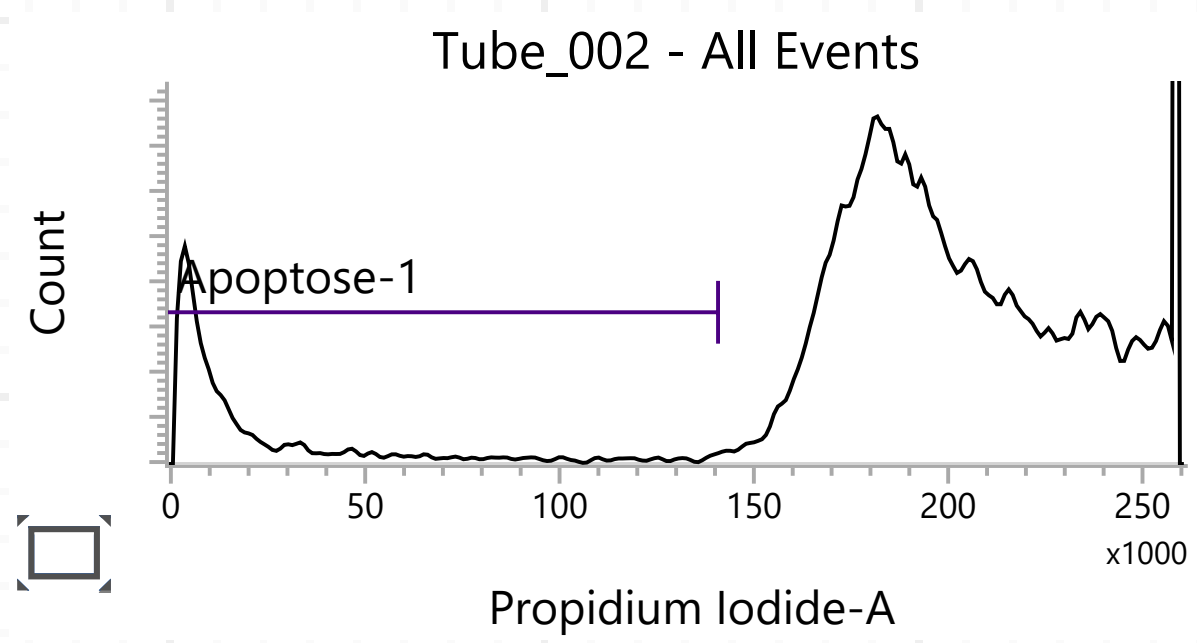

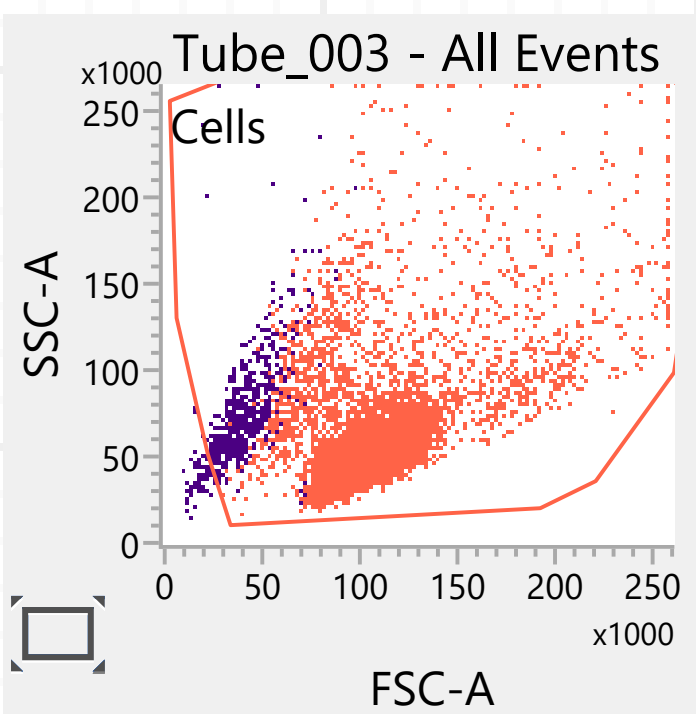

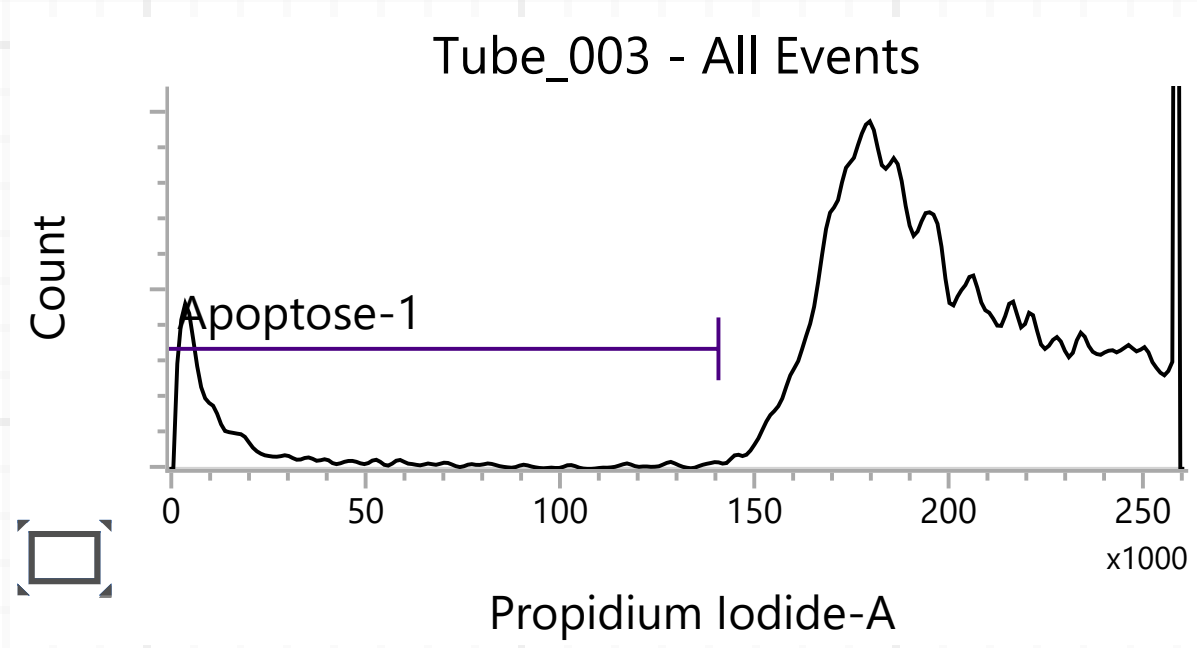

Statistics

Prozent apoptotische Zellen: Prozent apoptotische Zellen No Value % von Cells

Name	Events	% Total	% Parent	% Grandparent
Tube_001:All Events	10,075	100.00	***	***
Tube_001:Cells	10,000	99.26	99.26	***
Tube_002:All Events	10,000	100.00	***	***
Tube_002:Cells	9,926	99.26	99.26	***
Tube_003:All Events	10,000	100.00	***	***
Tube_003:Cells	9,942	99.42	99.42	***
Tube_001:Apoptose-1	539	5.35	5.35	***
Tube_002:Apoptose-1	667	6.67	6.67	***
Tube_003:Apoptose-1	600	6.00	6.00	***

DNA Fragmentierung

Sample ID: ZZ 72h N6 ATM_0,5

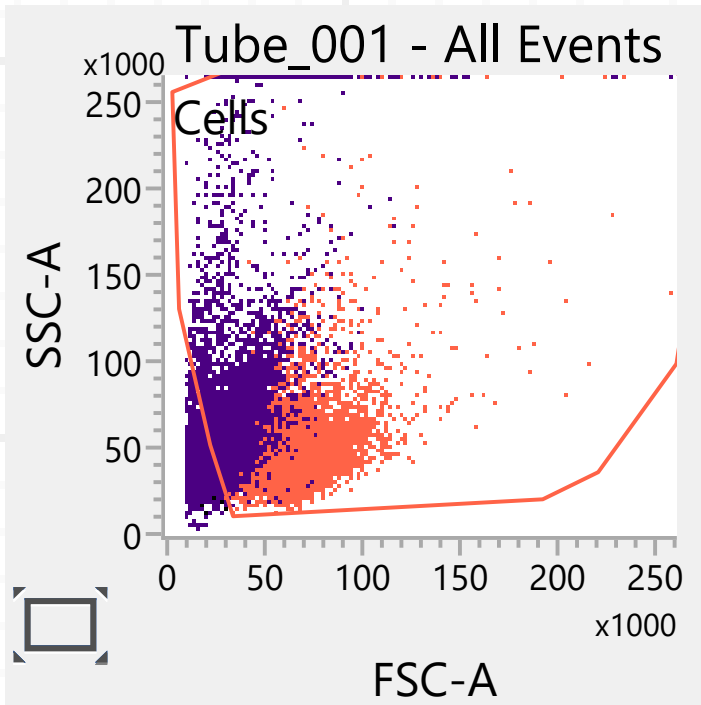

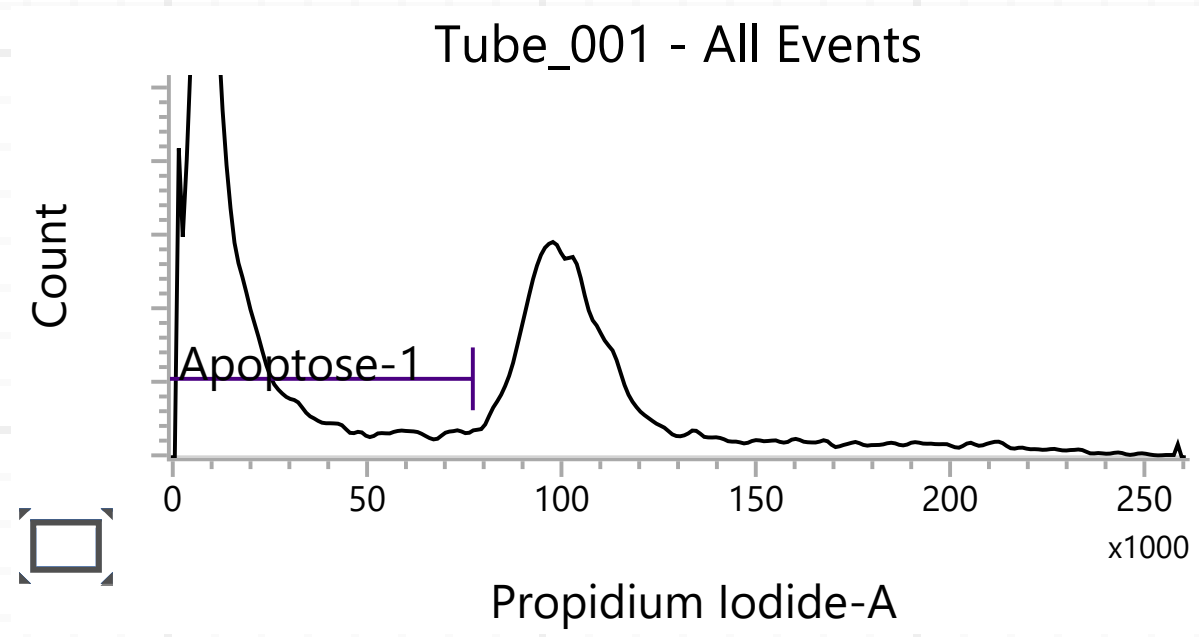

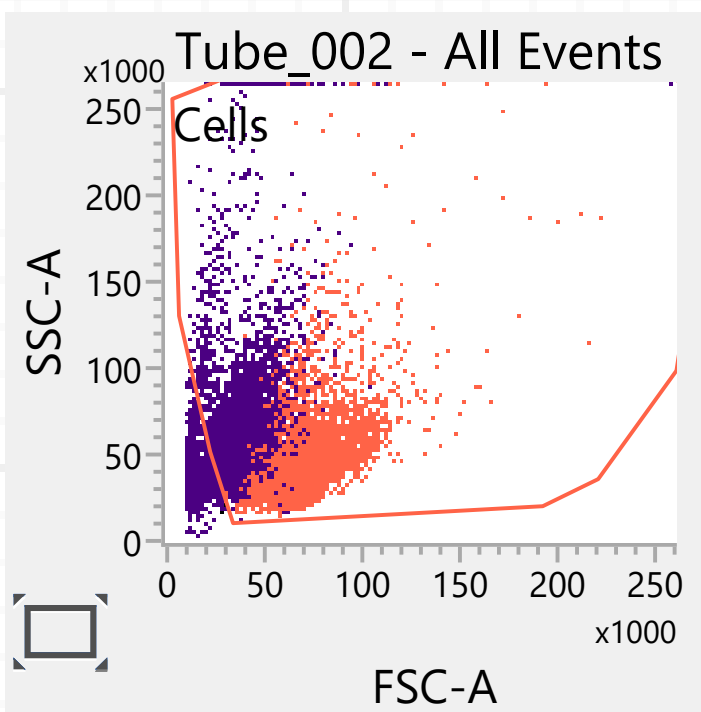

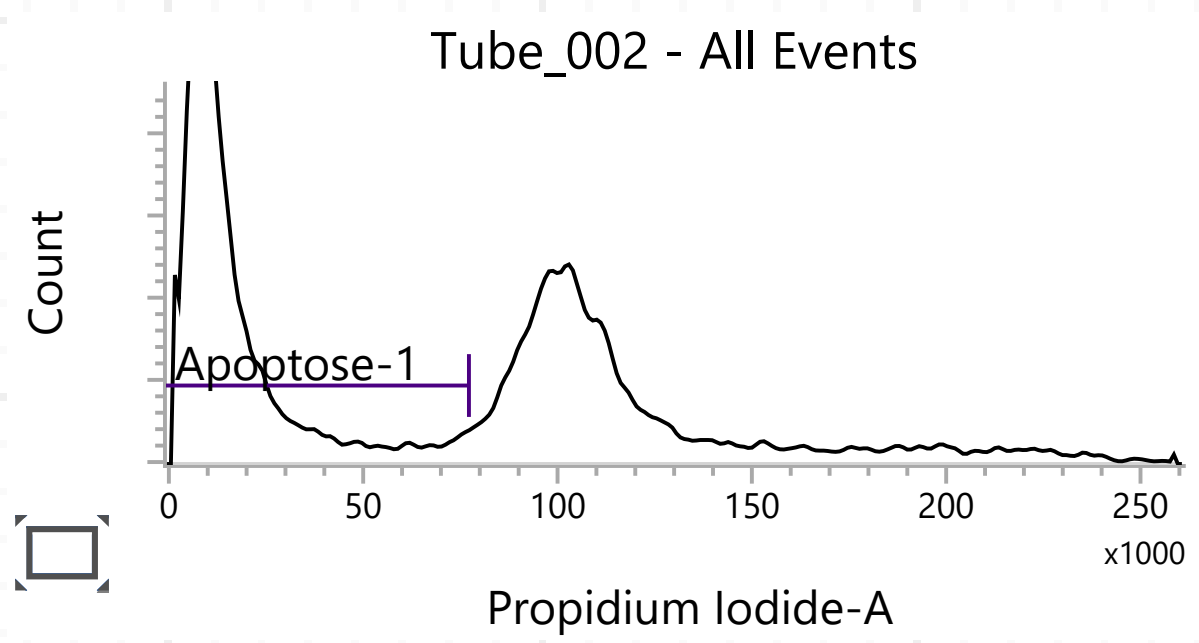

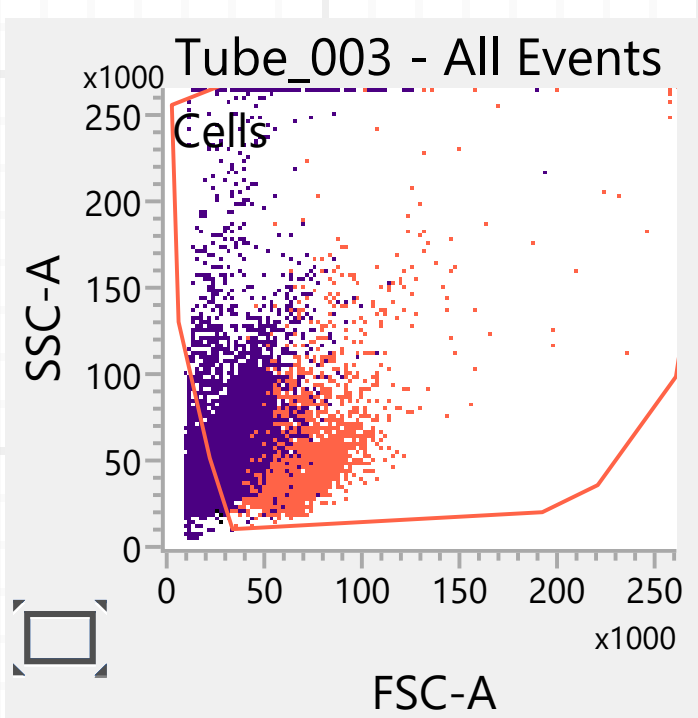

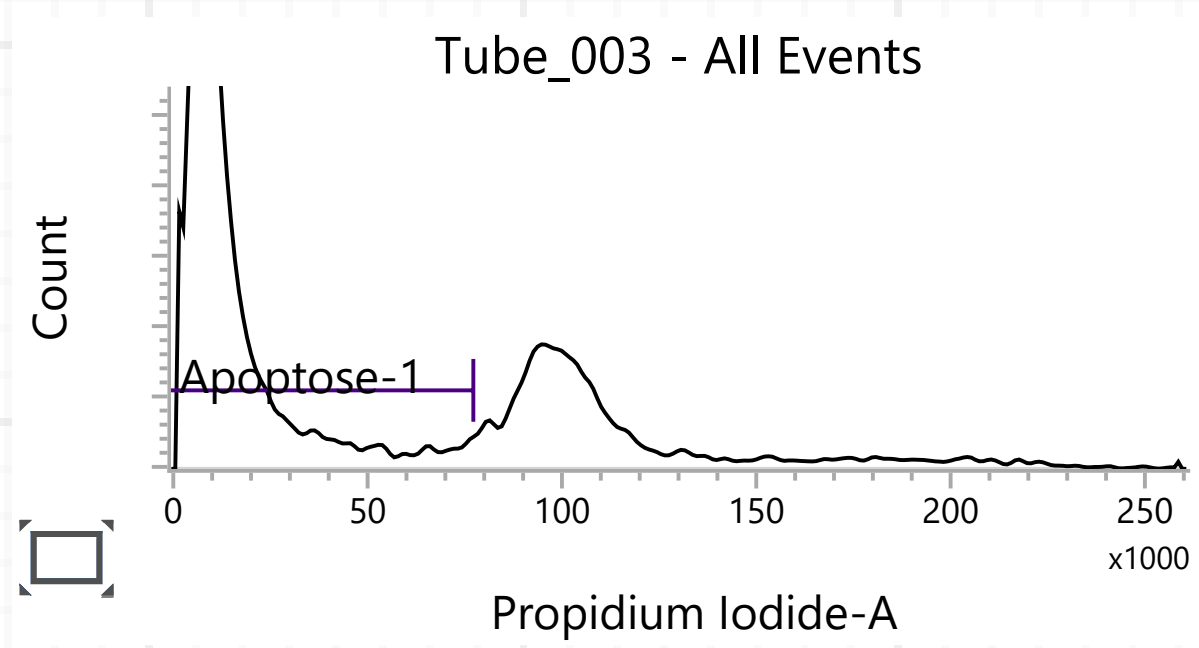

Statistics

Prozent apoptotische Zellen: Prozent apoptotische Zellen No Value % von Cells

Name	Events	% Total	% Parent	% Grandparent
Tube_001:All Events	12,131	100.00	***	***
Tube_001:Cells	10,000	82.43	82.43	***
Tube_002:All Events	10,000	100.00	***	***
Tube_002:Cells	8,542	85.42	85.42	***
Tube_003:All Events	10,000	100.00	***	***
Tube_003:Cells	8,026	80.26	80.26	***
Tube_001:Apoptose-1	7,221	59.53	59.53	***
Tube_002:Apoptose-1	5,424	54.24	54.24	***
Tube_003:Apoptose-1	6,887	68.87	68.87	***

DNA Fragmentierung

Sample ID: ZZ 72h N6 ATM_0,25

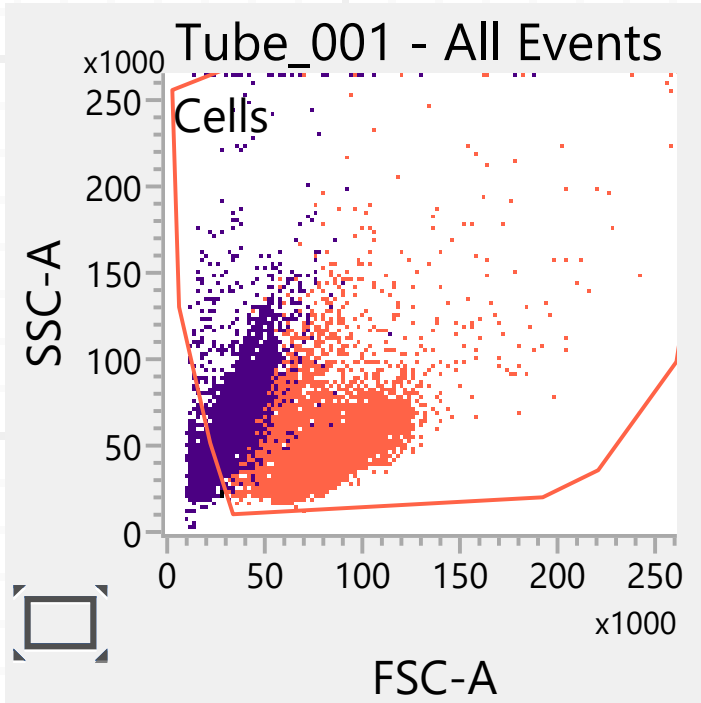

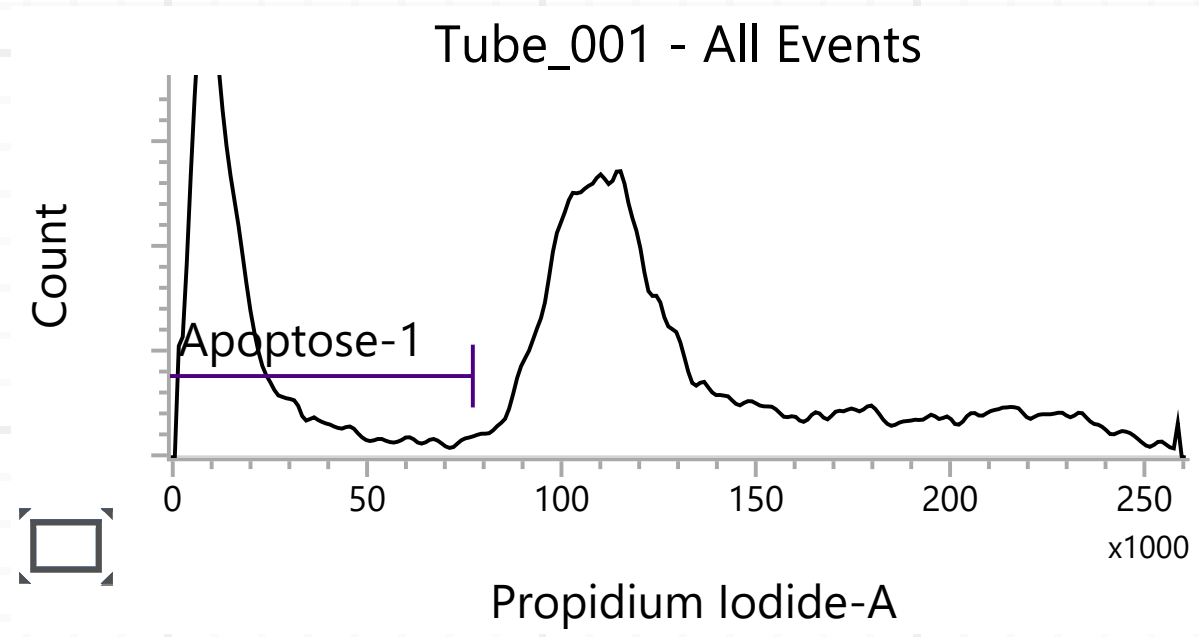

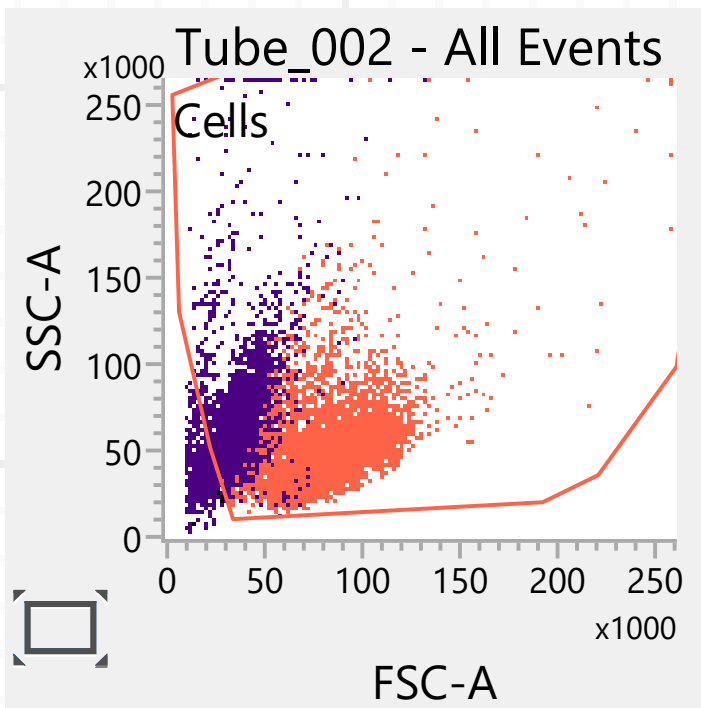

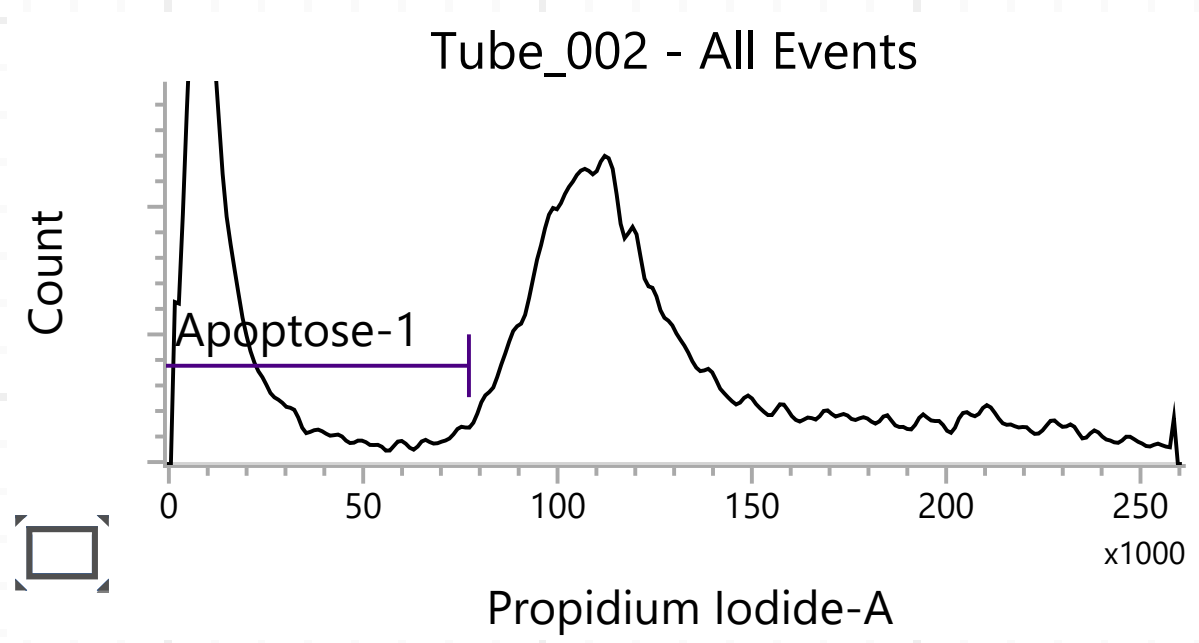

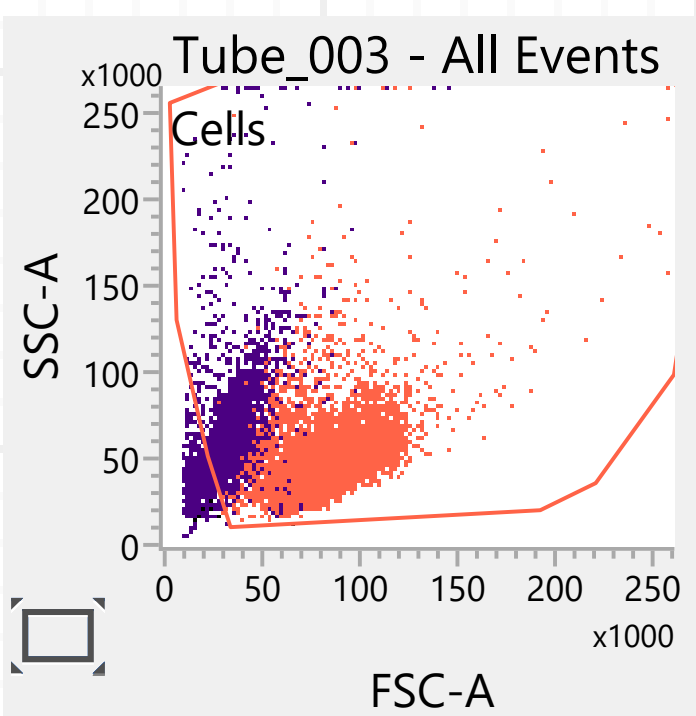

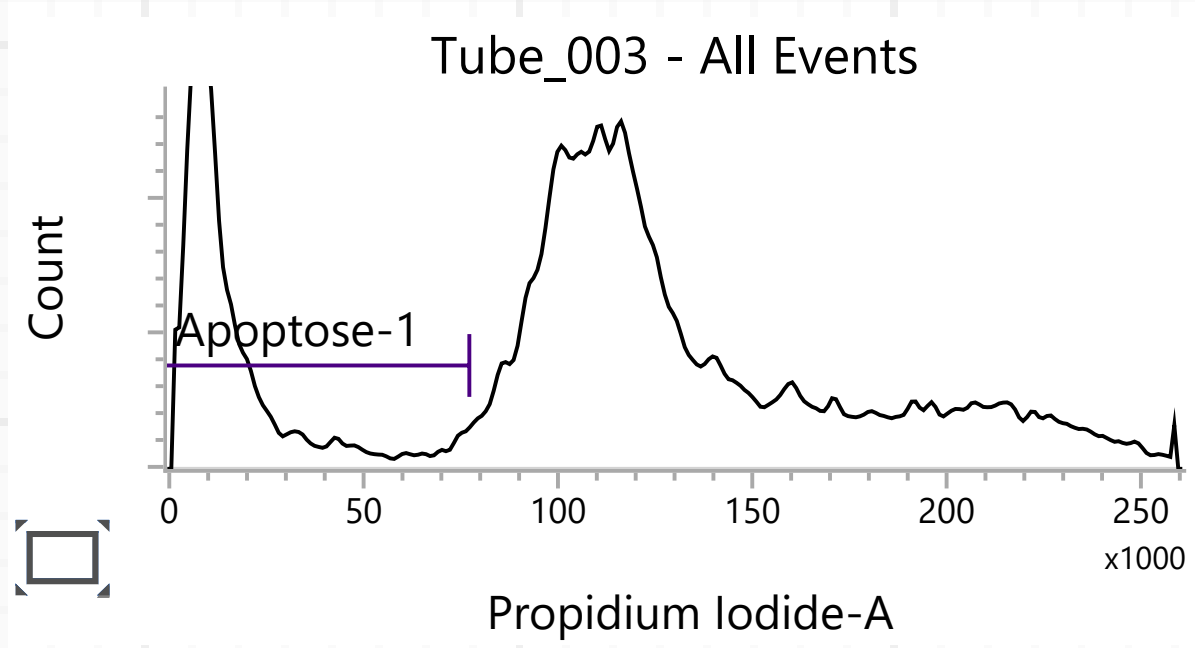

Statistics

Prozent apoptotische Zellen: Prozent apoptotische Zellen No Value % von Cells

Name	Events	% Total	% Parent	% Grandparent
Tube_001:All Events	10,851	100.00	***	***
Tube_001:Cells	10,000	92.16	92.16	***
Tube_002:All Events	10,000	100.00	***	***
Tube_002:Cells	9,167	91.67	91.67	***
Tube_003:All Events	10,000	100.00	***	***
Tube_003:Cells	9,331	93.31	93.31	***
Tube_001:Apoptose-1	3,885	35.80	35.80	***
Tube_002:Apoptose-1	3,474	34.74	34.74	***
Tube_003:Apoptose-1	2,669	26.69	26.69	***

DNA Fragmentierung

Sample ID: ZZ 72h N6 ATM_1

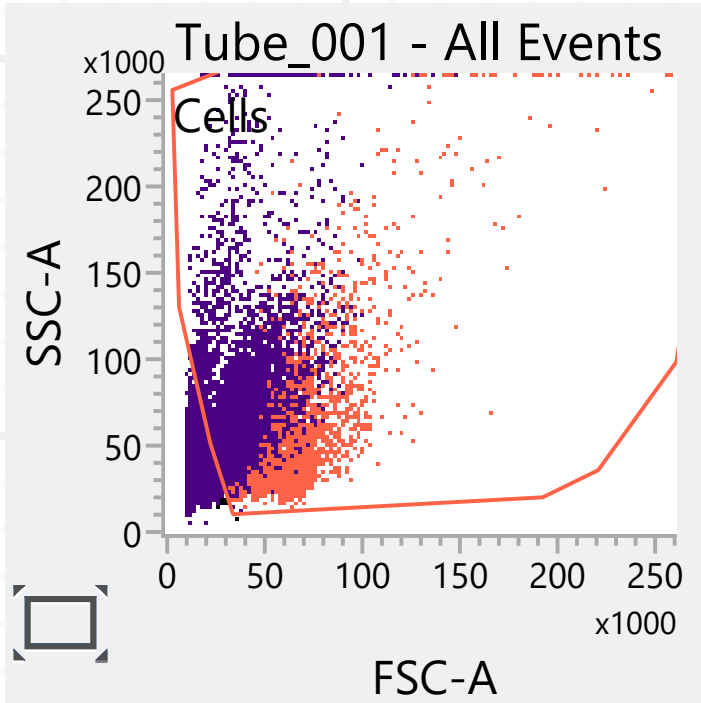

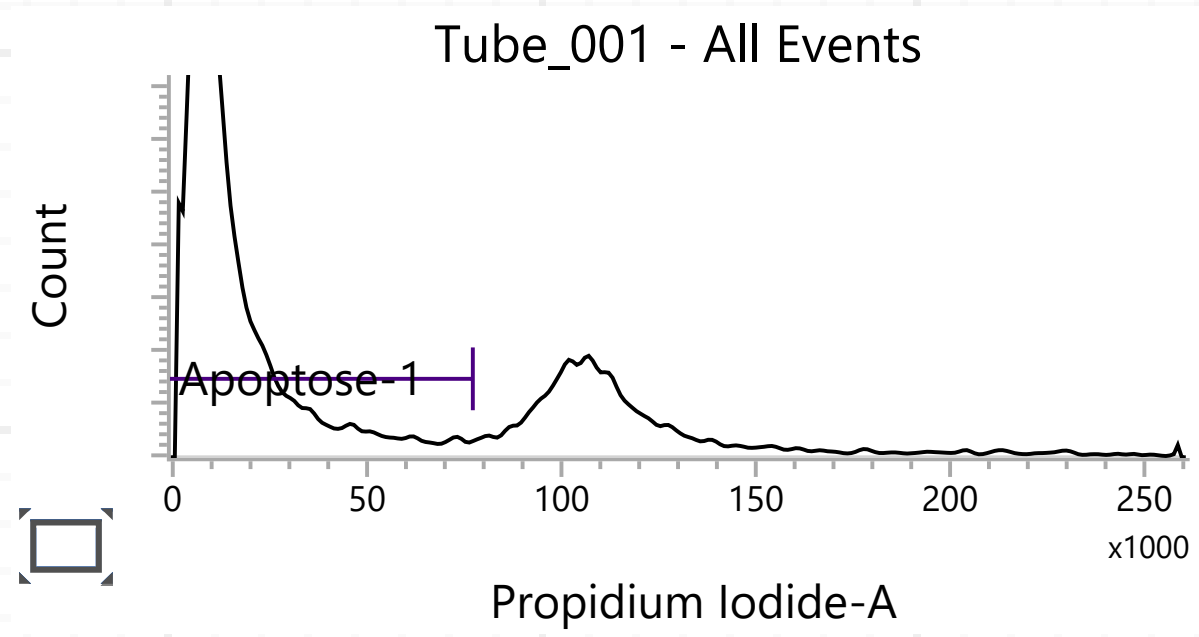

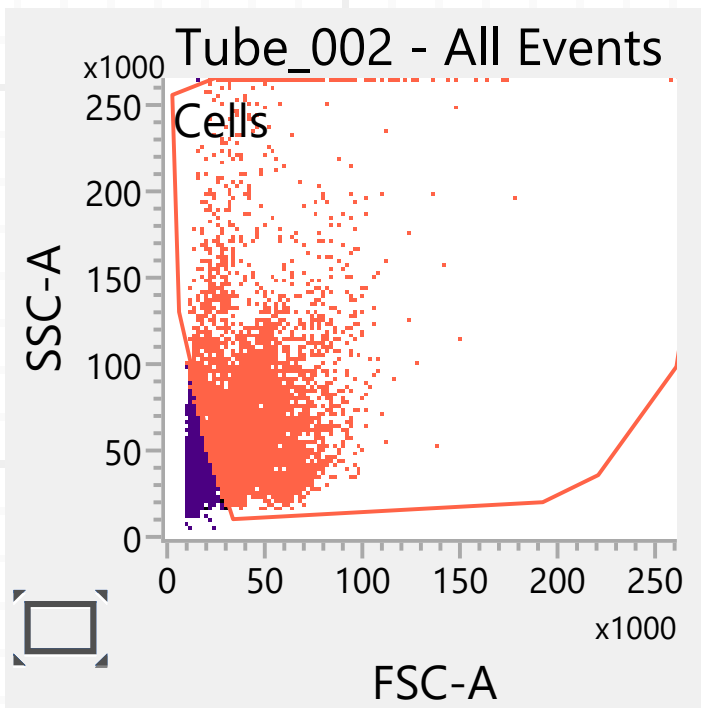

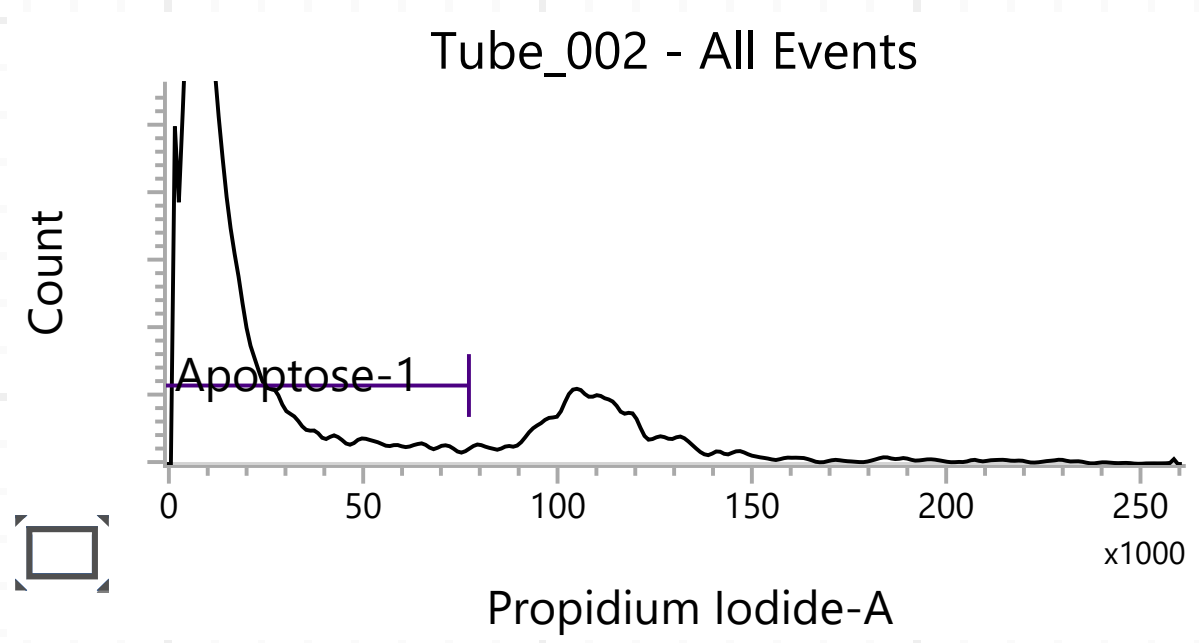

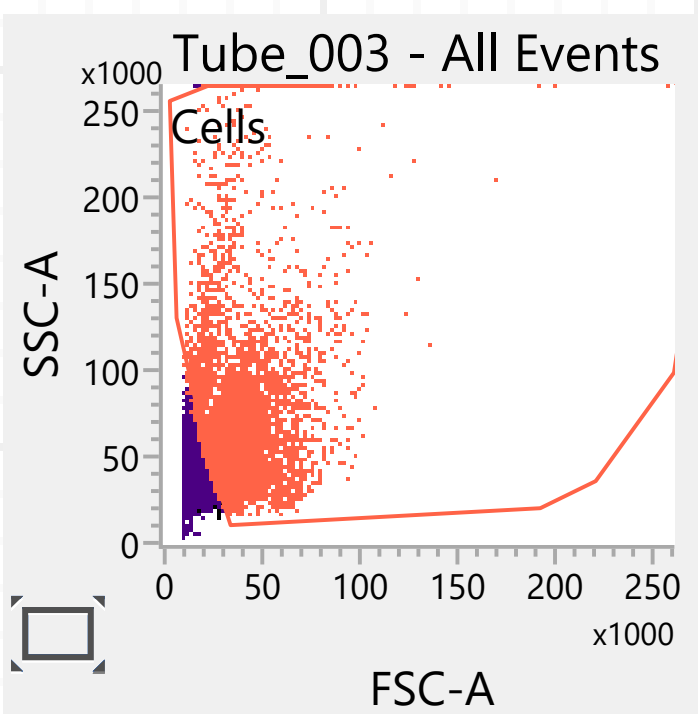

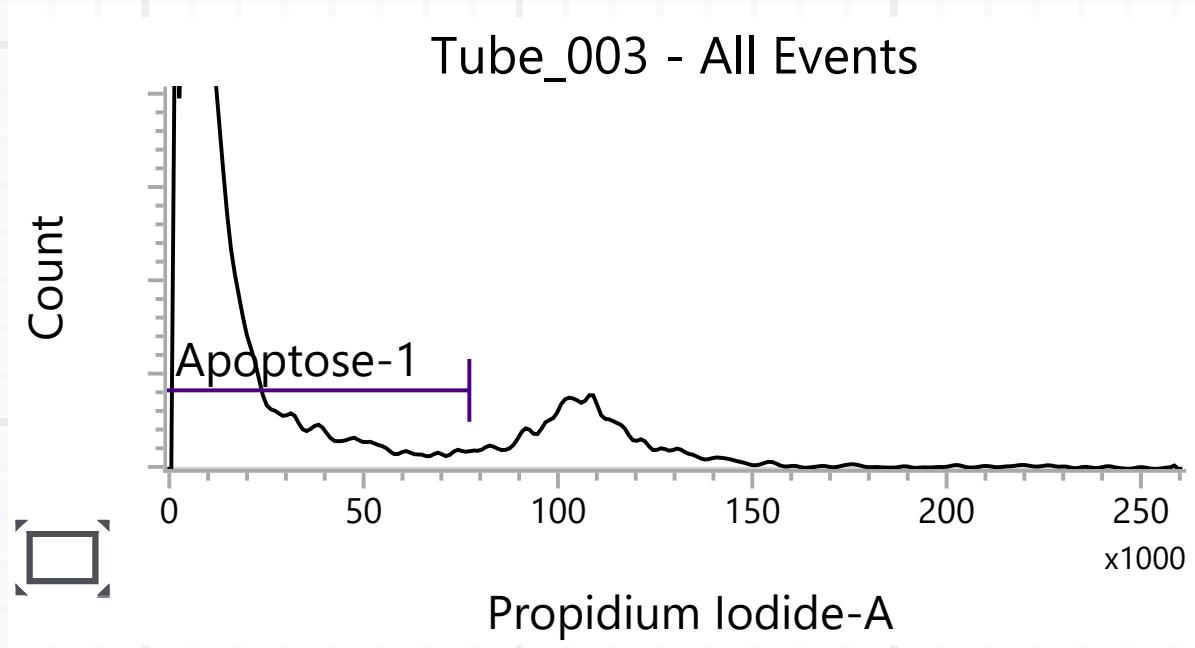

Statistics

Prozent apoptotische Zellen: Prozent apoptotische Zellen No Value % von Cells

Name	Events	% Total	% Parent	% Grandparent
Tube_001:All Events	12,839	100.00	***	***
Tube_001:Cells	10,000	77.89	77.89	***
Tube_002:All Events	10,000	100.00	***	***
Tube_002:Cells	7,524	75.24	75.24	***
Tube_003:All Events	7,827	100.00	***	***
Tube_003:Cells	5,652	72.21	72.21	***
Tube_001:Apoptose-1	9,251	72.05	72.05	***
Tube_002:Apoptose-1	7,924	79.24	79.24	***
Tube_003:Apoptose-1	6,475	82.73	82.73	***

DNA Fragmentierung

Sample ID: ZZ 72h N6 ATM_2,5

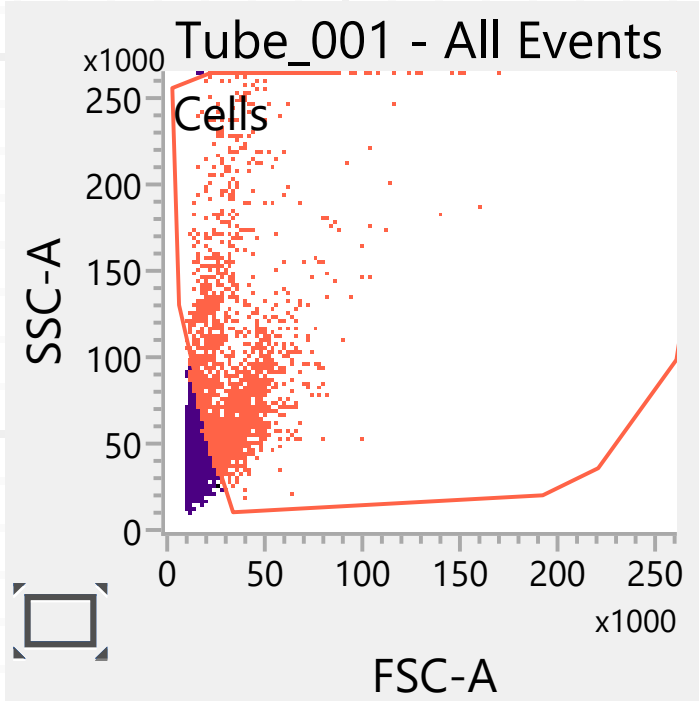

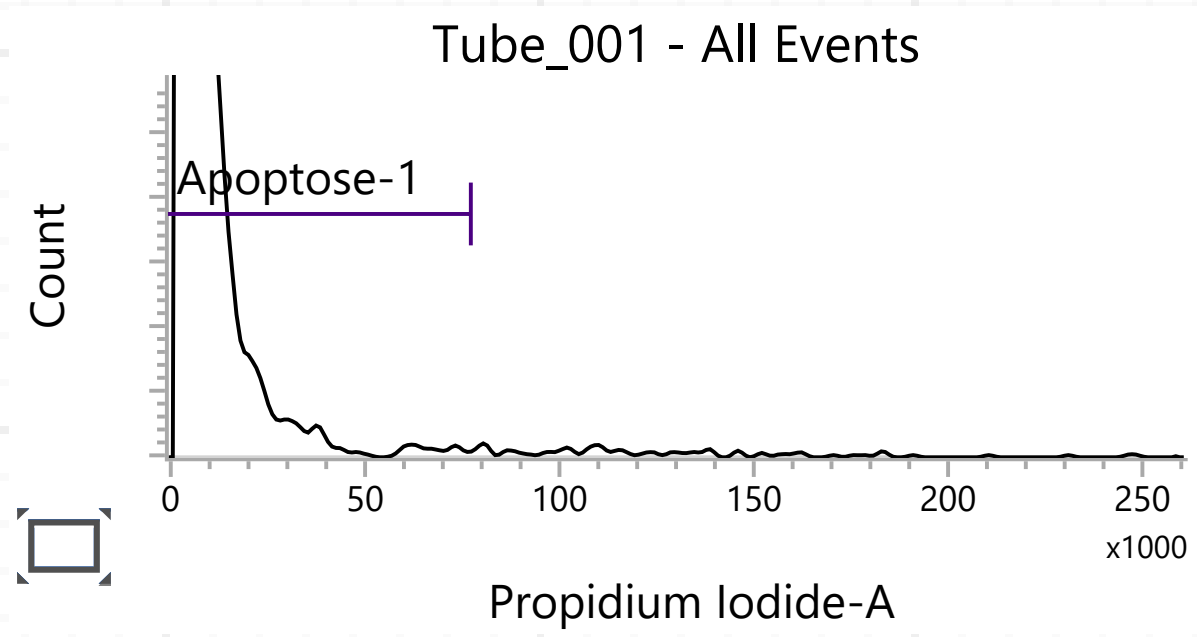

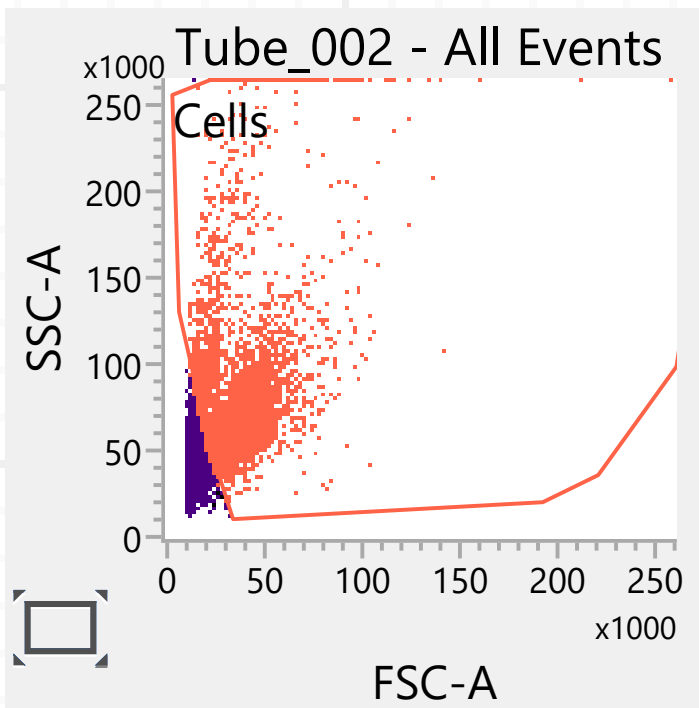

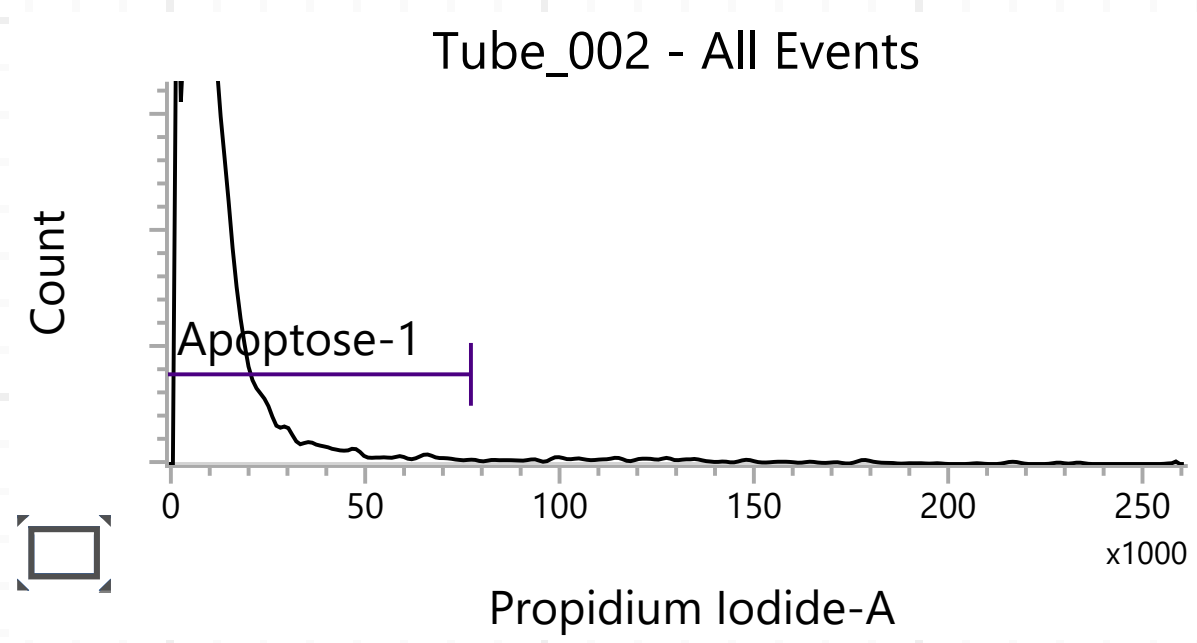

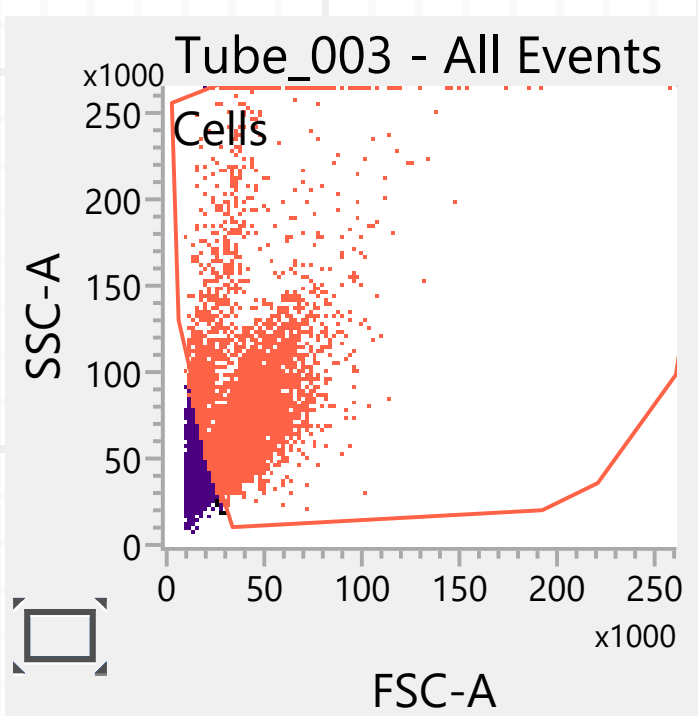

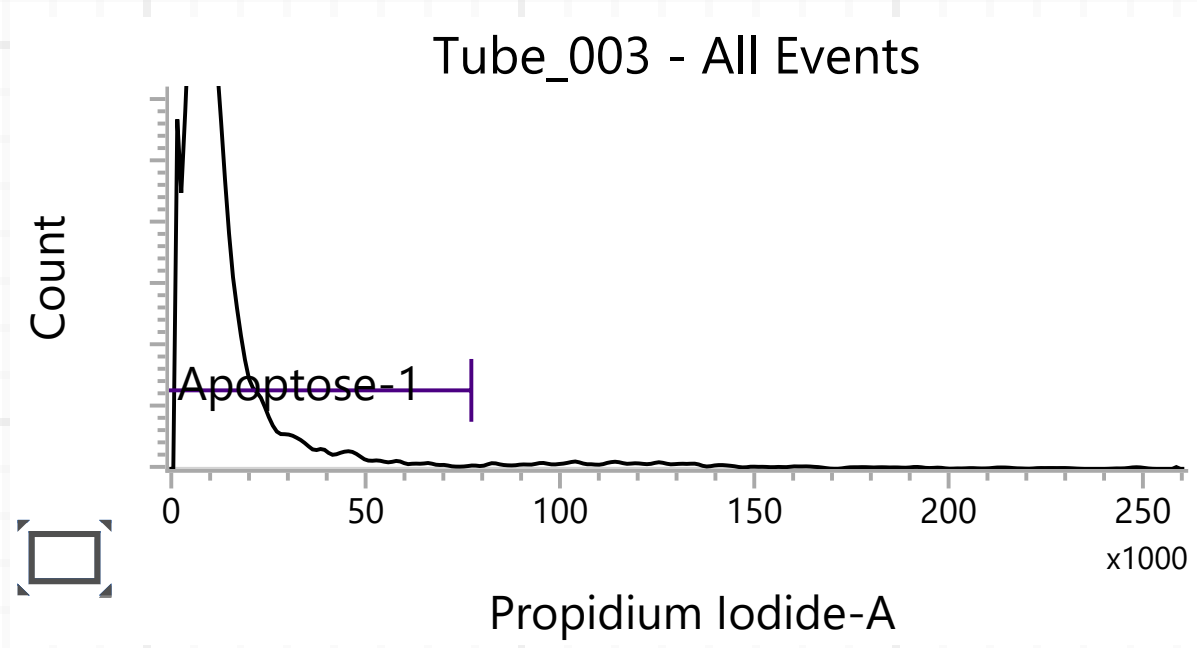

Statistics

Prozent apoptotische Zellen: Prozent apoptotische Zellen No Value % von Cells

Name	Events	% Total	% Parent	% Grandparent
Tube_001:All Events	3,031	100.00	***	***
Tube_001:Cells	2,085	68.79	68.79	***
Tube_002:All Events	5,289	100.00	***	***
Tube_002:Cells	3,956	74.80	74.80	***
Tube_003:All Events	8,180	100.00	***	***
Tube_003:Cells	6,630	81.05	81.05	***
Tube_001:Apoptose-1	2,951	97.36	97.36	***
Tube_002:Apoptose-1	5,108	96.58	96.58	***
Tube_003:Apoptose-1	7,808	95.45	95.45	***

DNA Fragmentierung

Sample ID: ZZ 72h N6 ATM_D

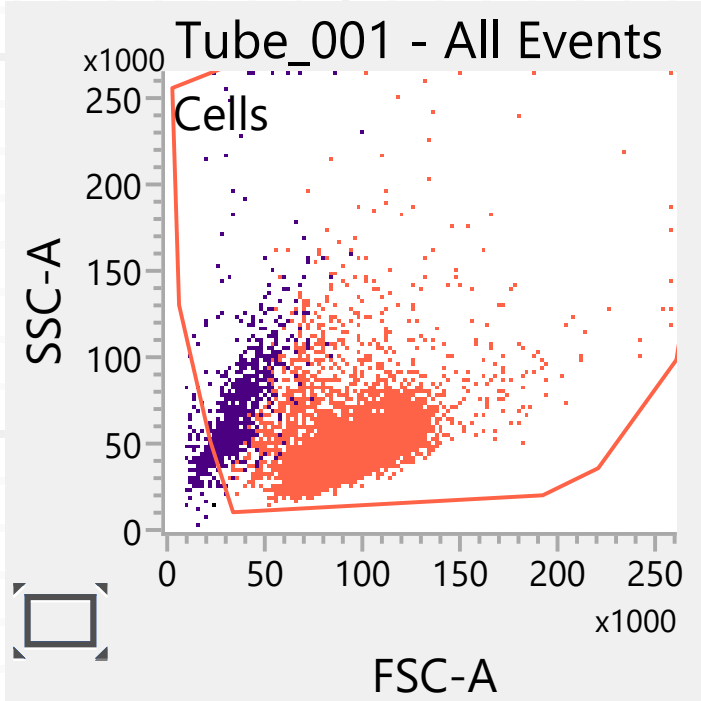

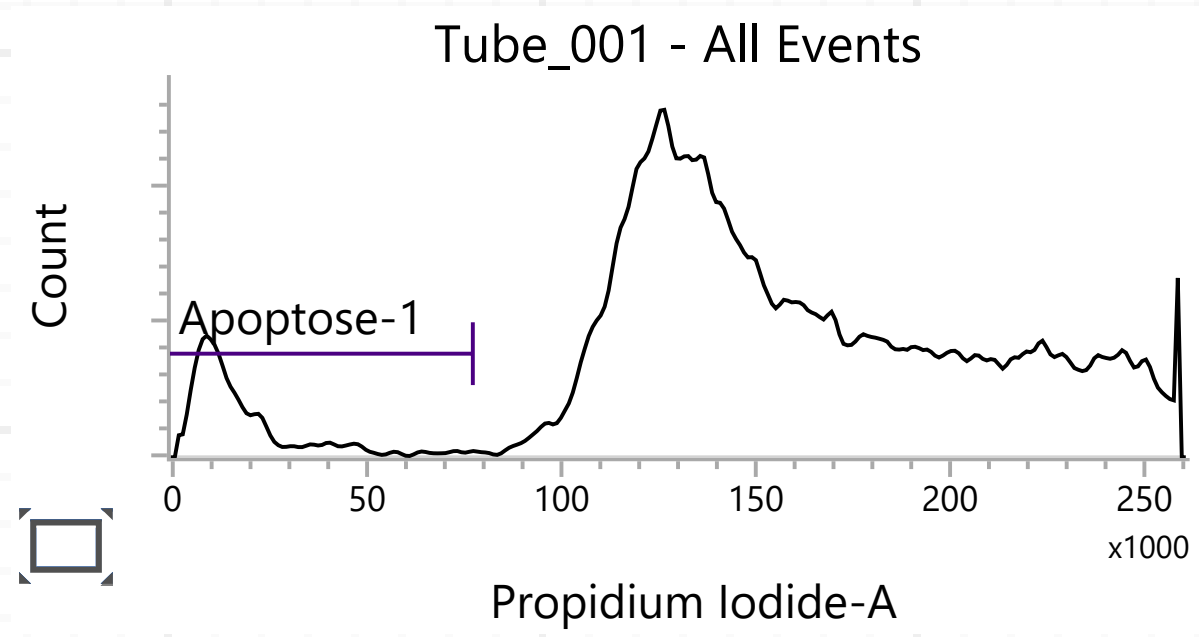

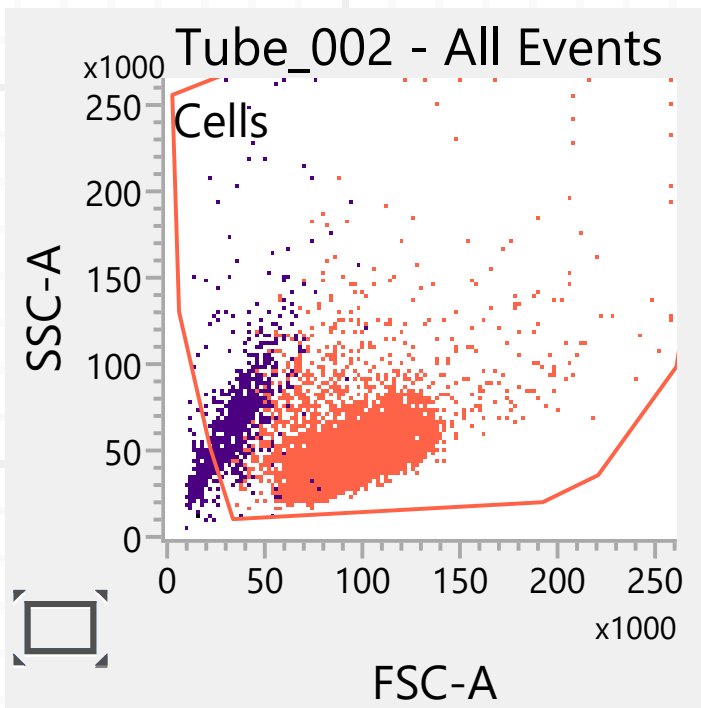

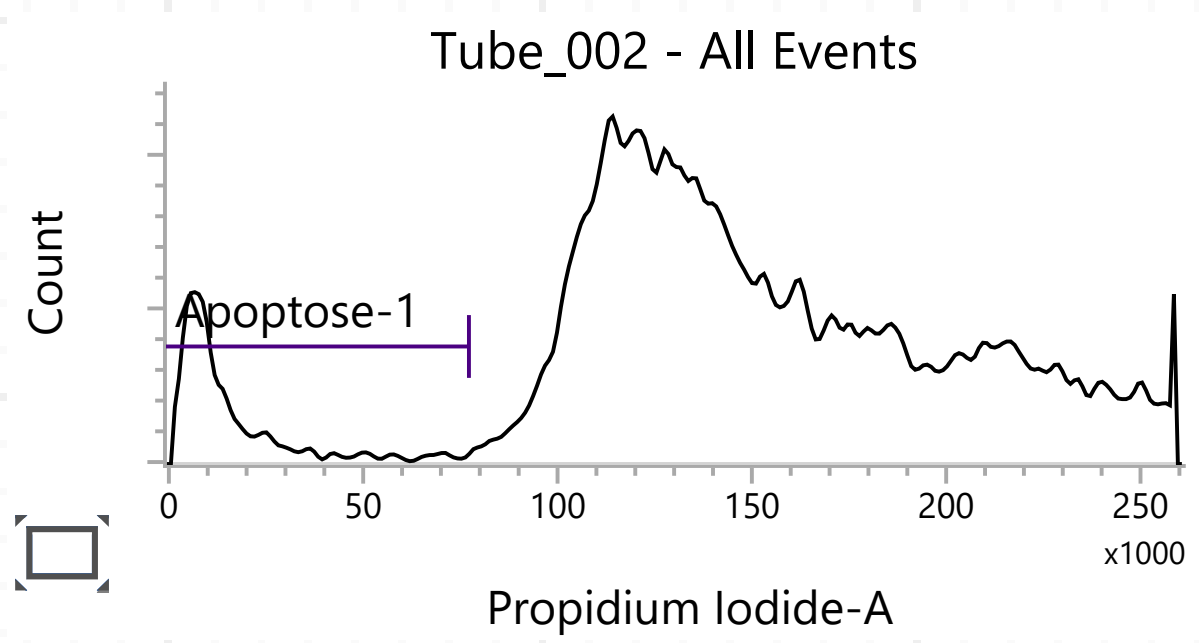

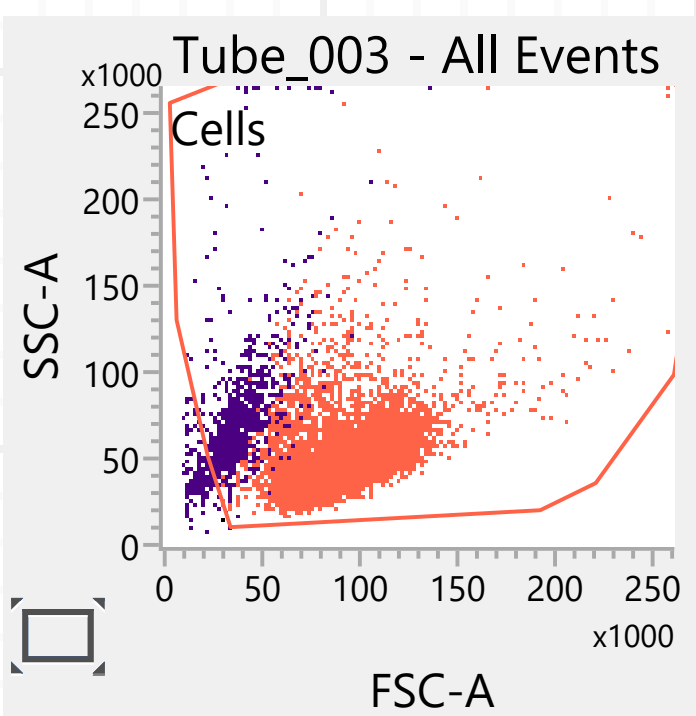

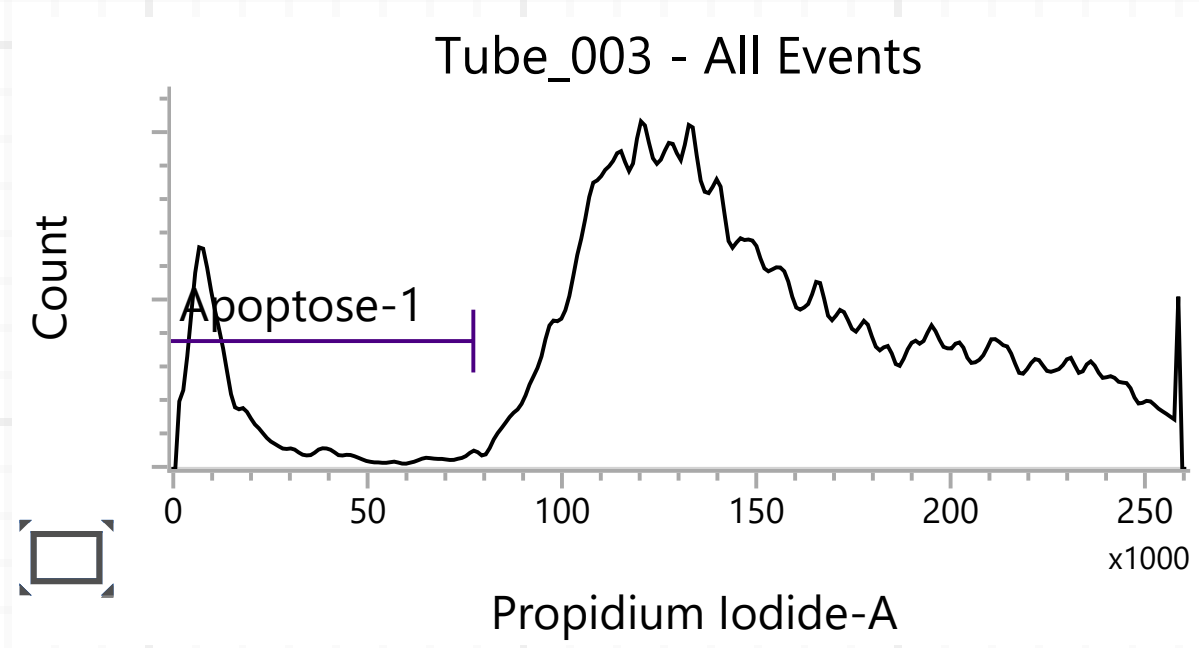

Statistics

Prozent apoptotische Zellen: Prozent apoptotische Zellen No Value % von Cells

Name	Events	% Total	% Parent	% Grandparent
Tube_001:All Events	10,123	100.00	***	***
Tube_001:Cells	10,000	98.78	98.78	***
Tube_002:All Events	10,000	100.00	***	***
Tube_002:Cells	9,822	98.22	98.22	***
Tube_003:All Events	10,000	100.00	***	***
Tube_003:Cells	9,836	98.36	98.36	***
Tube_001:Apoptose-1	793	7.83	7.83	***
Tube_002:Apoptose-1	868	8.68	8.68	***
Tube_003:Apoptose-1	1,003	10.03	10.03	***

DNA Fragmentierung

Sample ID: ZZ 72h N6 ATM_K

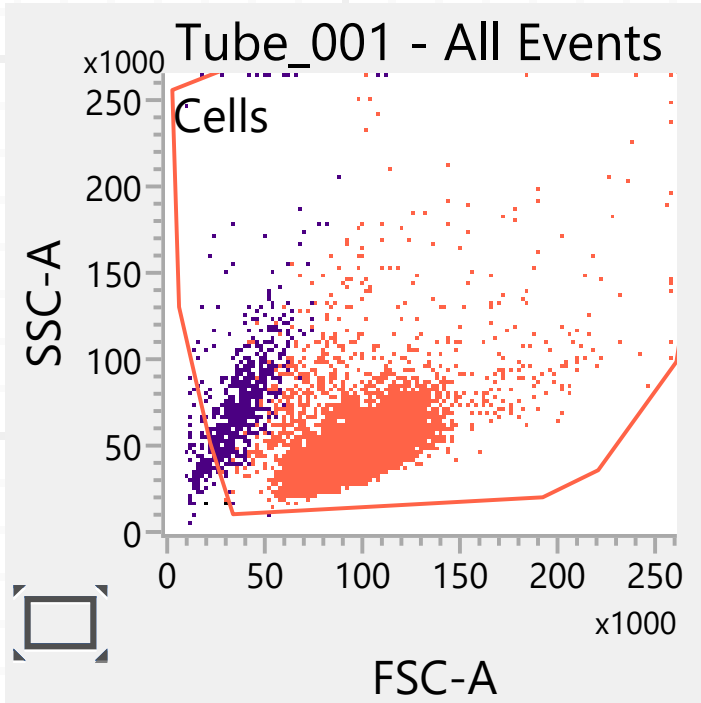

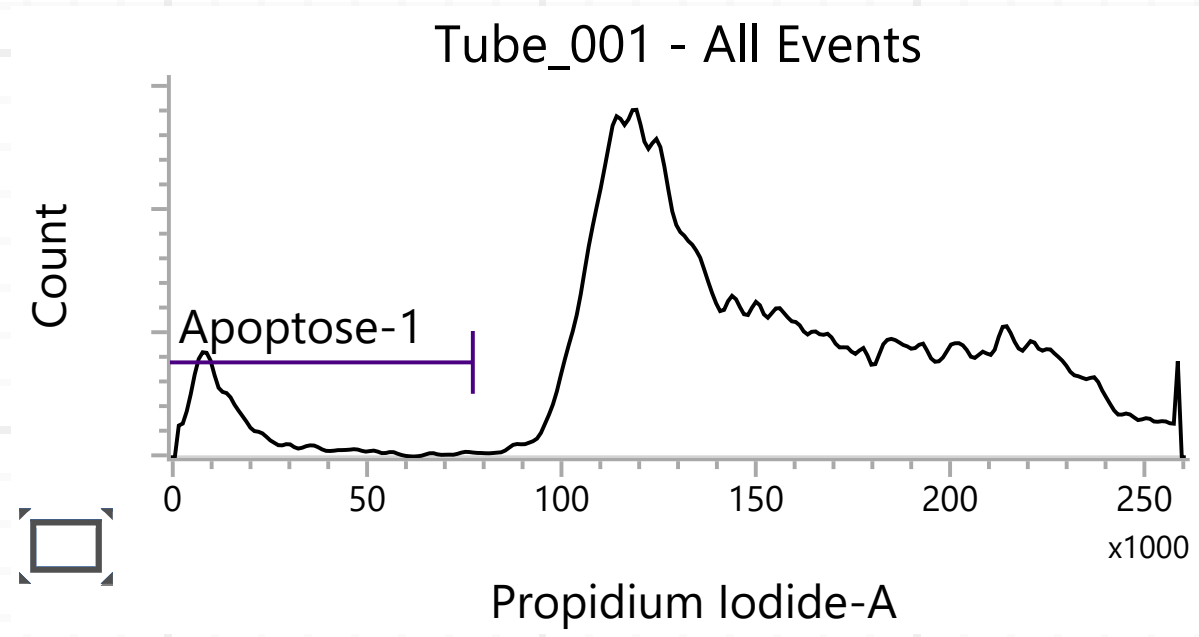

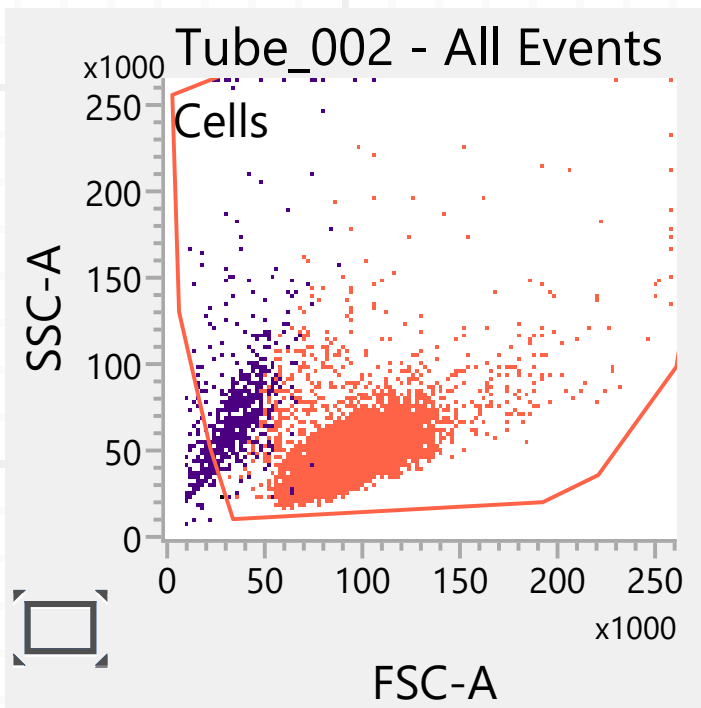

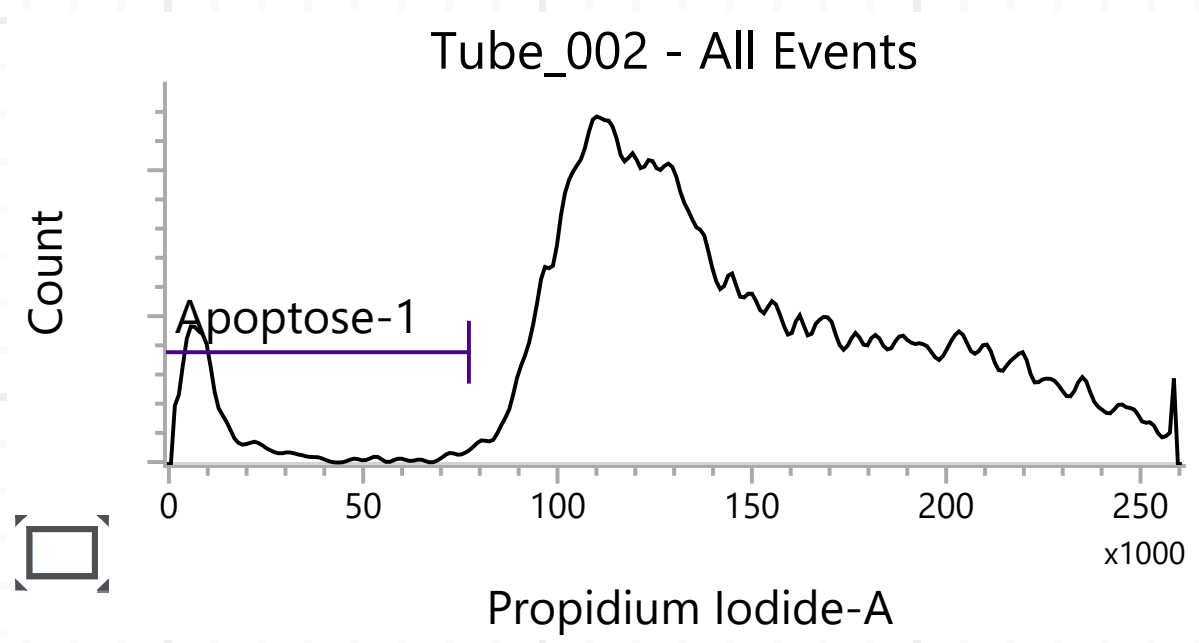

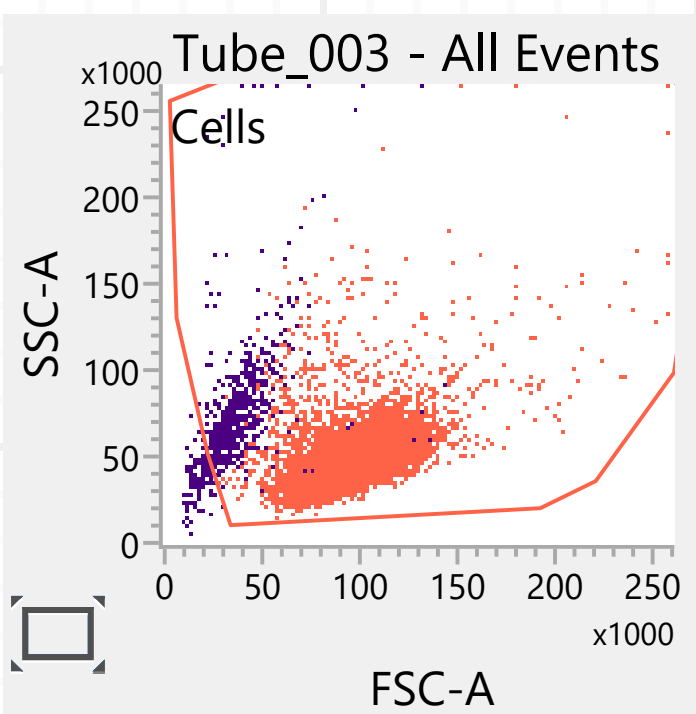

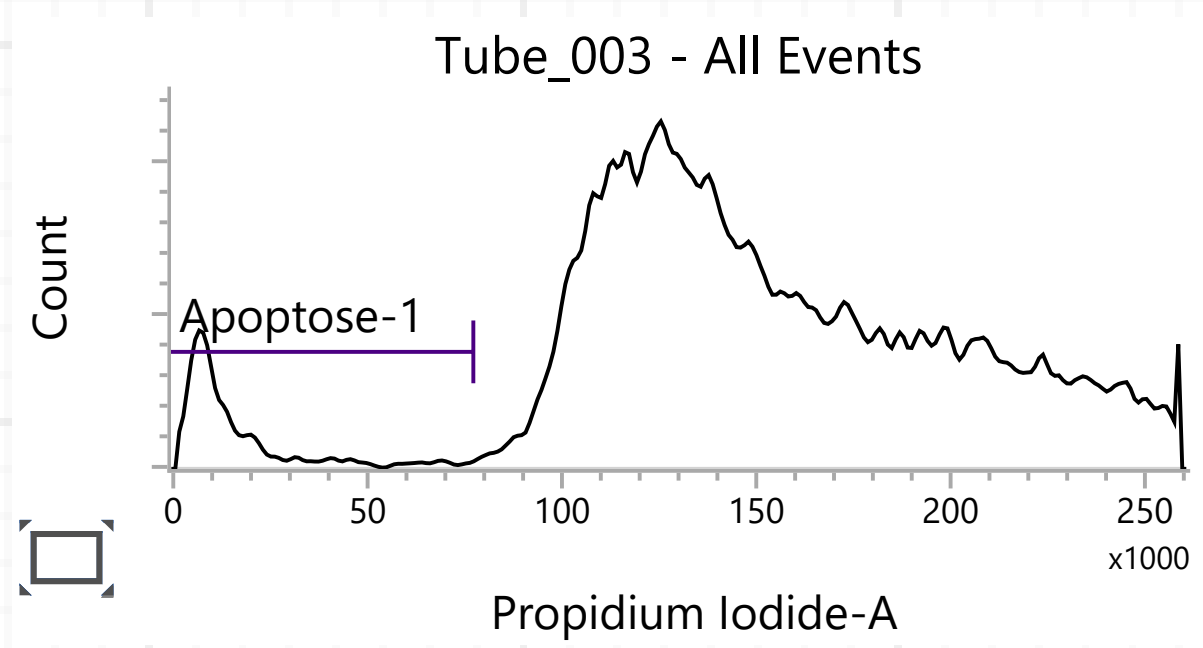

Statistics

Prozent apoptotische Zellen: Prozent apoptotische Zellen No Value % von Cells

Name	Events	% Total	% Parent	% Grandparent
Tube_001:All Events	10,107	100.00	***	***
Tube_001:Cells	10,000	98.94	98.94	***
Tube_002:All Events	10,000	100.00	***	***
Tube_002:Cells	9,874	98.74	98.74	***
Tube_003:All Events	10,000	100.00	***	***
Tube_003:Cells	9,902	99.02	99.02	***
Tube_001:Apoptose-1	720	7.12	7.12	***
Tube_002:Apoptose-1	704	7.04	7.04	***
Tube_003:Apoptose-1	644	6.44	6.44	***
